# Supplementary material for: Perceptions and practices in urban Burkina Faso: a qualitative study on gestational age estimation among health workers
Source: Int J Qual Stud Health Well-being. 2025 Jul 4;20(1):2508421. doi: 10.1080/17482631.2025.2508421 (PMC12231294; doi:10.1080/17482631.2025.2508421)
Supplement: Supplementary_file_1_transcribed_audios.docx [file ZQHW_A_2508421_SM4867.docx]

**Supplementary file** **1**: report containing the transcribed audios, then translated from French to English

**Note:**

First text box: the original transcription

Second text box: improved punctuation

Third text box: the translation from French to English

Table of Contents

[1. Informed consent and presentation of participants 3](#_Toc184378286)

[Focus group of Accart-ville urban medical center 3](#_Toc184378287)

[Manager of Accart-ville urban medical center 4](#_Toc184378288)

[Maternity manager of Accart-ville urban medical center 5](#_Toc184378289)

[Focus group of Do medical center with surgical branch 7](#_Toc184378290)

[Gynecologist 1 8](#_Toc184378291)

[Gynecologist 2 9](#_Toc184378292)

[Chief physician of Do health district 11](#_Toc184378293)

[Manager for maternity care unit of Do medical center with surgical branch 12](#_Toc184378294)

[Focus group of Colma1 health and social promotion center 14](#_Toc184378295)

[Major of Colma1 health and social promotion center 15](#_Toc184378296)

[Maternity manager of Colma1 health and social promotion center 16](#_Toc184378297)

[Focus group of Farakan health and social promotion center 18](#_Toc184378298)

[Major of Farakan health and social promotion center 20](#_Toc184378299)

[Maternity manager of Farakan health and social promotion center 22](#_Toc184378300)

[2. Definition of prematurity 23](#_Toc184378301)

[Focus group of Accart-ville urban medical center 23](#_Toc184378302)

[Manager of Accart-ville urban medical center 23](#_Toc184378303)

[Maternity manager of Accart-ville urban medical center 24](#_Toc184378304)

[Focus group of Do medical center with surgical branch 24](#_Toc184378305)

[Gynecologist 1 25](#_Toc184378306)

[Gynecologist 2 25](#_Toc184378307)

[Chief physician of Do health district 26](#_Toc184378308)

[Manager for maternity care unit of Do medical center with surgical branch 26](#_Toc184378309)

[Focus group of Colma1 health and social promotion center 27](#_Toc184378310)

[Major of Colma1 health and social promotion center 27](#_Toc184378311)

[Maternity manager of Colma1 health and social promotion center 28](#_Toc184378312)

[Focus group of Farakan health and social promotion center 28](#_Toc184378313)

[Major of Farakan health and social promotion center 29](#_Toc184378314)

[Maternity manager of Farakan health and social promotion center 30](#_Toc184378315)

[3. Measurement of preterm birth 31](#_Toc184378316)

[Focus group of Accart-ville urban medical center 31](#_Toc184378317)

[Manager of Accart-ville urban medical center 32](#_Toc184378318)

[Maternity manager of Accart-ville urban medical center 32](#_Toc184378319)

[Focus group of Do medical center with surgical branch 33](#_Toc184378320)

[Gynecologist 1 34](#_Toc184378321)

[Gynecologist 2 35](#_Toc184378322)

[Chief physician of Do health district 35](#_Toc184378323)

[Manager for maternity care unit of Do medical center with surgical branch 36](#_Toc184378324)

[Focus group of Colma1 health and social promotion center 36](#_Toc184378325)

[Major of Colma1 health and social promotion center 38](#_Toc184378326)

[Maternity manager of Colma1 health and social promotion center 38](#_Toc184378327)

[Focus group of Farakan health and social promotion center 39](#_Toc184378328)

[Major of Farakan health and social promotion center 41](#_Toc184378329)

[Maternity manager of Farakan health and social promotion center 41](#_Toc184378330)

[3.1 Control of the date of the last period 42](#_Toc184378331)

[Focus group of Accart-ville urban medical center 42](#_Toc184378332)

[Manager of Accart-ville urban medical center 44](#_Toc184378333)

[Maternity manager of Accart-ville urban medical center 45](#_Toc184378334)

[Focus group of Do medical center with surgical branch 46](#_Toc184378335)

[Gynecologist 1 48](#_Toc184378336)

[Gynecologist 2 49](#_Toc184378337)

[Chief physician of Do health district 50](#_Toc184378338)

[Manager for maternity care unit of Do medical center with surgical branch 52](#_Toc184378339)

[Focus group of Colma1 health and social promotion center 54](#_Toc184378340)

[Major of Colma1 health and social promotion center 56](#_Toc184378341)

[Maternity manager of Colma1 health and social promotion center 57](#_Toc184378342)

[Focus group of Farakan health and social promotion center 59](#_Toc184378343)

[Major of Farakan health and social promotion center 61](#_Toc184378344)

[Maternity manager of Farakan health and social promotion center 62](#_Toc184378345)

[3.2 Control of the date of the last period by women who present late for their antenatal care 63](#_Toc184378346)

[Focus group of Accart-ville urban medical center 63](#_Toc184378347)

[Manager of Accart-ville urban medical center 63](#_Toc184378348)

[Maternity manager of Accart-ville urban medical center 64](#_Toc184378349)

[Focus group of Do medical center with surgical branch 64](#_Toc184378350)

[Gynecologist 1 64](#_Toc184378351)

[Gynecologist 2 65](#_Toc184378352)

[Chief physician of Do health district 65](#_Toc184378353)

[Manager for maternity care unit of Do medical center with surgical branch 66](#_Toc184378354)

[Focus group of Colma1 health and social promotion center 66](#_Toc184378355)

[Major of Colma1 health and social promotion center 67](#_Toc184378356)

[Maternity manager of Colma1 health and social promotion center 68](#_Toc184378357)

[Focus group of Farakan health and social promotion center 68](#_Toc184378358)

[Major of Farakan health and social promotion center 69](#_Toc184378359)

[Maternity manager of Farakan health and social promotion center 70](#_Toc184378360)

[3.3 Ways health workers help women remember the date of their last period 70](#_Toc184378361)

[Focus group of Accart-ville urban medical center 70](#_Toc184378362)

[Manager of Accart-ville urban medical center 71](#_Toc184378363)

[Maternity manager of Accart-ville urban medical center 71](#_Toc184378364)

[Focus group of Do medical center with surgical branch 72](#_Toc184378365)

[Gynecologist 1 72](#_Toc184378366)

[Gynecologist 2 73](#_Toc184378367)

[Chief physician of Do health district 73](#_Toc184378368)

[Manager for maternity care unit of Do medical center with surgical branch 74](#_Toc184378369)

[Focus group of Colma1 health and social promotion center 75](#_Toc184378370)

[Major of Colma1 health and social promotion center 75](#_Toc184378371)

[Maternity manager of Colma1 health and social promotion center 76](#_Toc184378372)

[Focus group of Farakan health and social promotion center 76](#_Toc184378373)

[Major of Farakan health and social promotion center 77](#_Toc184378374)

[Maternity manager of Farakan health and social promotion center 78](#_Toc184378375)

[3.4 Method of calculating gestational age from fundal height 78](#_Toc184378376)

[Focus group of Accart-ville urban medical center 78](#_Toc184378377)

[Manager of Accart-ville urban medical center 79](#_Toc184378378)

[Maternity manager of Accart-ville urban medical center 80](#_Toc184378379)

[Focus group of Do medical center with surgical branch 81](#_Toc184378380)

[Gynecologist 1 81](#_Toc184378381)

[Gynecologist 2 82](#_Toc184378382)

[Chief physician of Do health district 83](#_Toc184378383)

[Manager for maternity care unit of Do medical center with surgical branch 84](#_Toc184378384)

[Focus group of Colma1 health and social promotion center 85](#_Toc184378385)

[Major of Colma1 health and social promotion center 85](#_Toc184378386)

[Maternity manager of Colma1 health and social promotion center 86](#_Toc184378387)

[Focus group of Farakan health and social promotion center 87](#_Toc184378388)

[Major of Farakan health and social promotion center 87](#_Toc184378389)

[Maternity manager of Farakan health and social promotion center 88](#_Toc184378390)

[3.5 Materials needed for calculating gestational age 89](#_Toc184378391)

[Focus group of Accart-ville urban medical center 89](#_Toc184378392)

[Manager of Accart-ville urban medical center 89](#_Toc184378393)

[Maternity manager of Accart-ville urban medical center 90](#_Toc184378394)

[Focus group of Do medical center with surgical branch 90](#_Toc184378395)

[Gynecologist 1 91](#_Toc184378396)

[Gynecologist 2 91](#_Toc184378397)

[Chief physician of Do health district 92](#_Toc184378398)

[Manager for maternity care unit of Do medical center with surgical branch 92](#_Toc184378399)

[Focus group of Colma1 health and social promotion center 93](#_Toc184378400)

[Major of Colma1 health and social promotion center 93](#_Toc184378401)

[Maternity manager of Colma1 health and social promotion center 94](#_Toc184378402)

[Focus group of Farakan health and social promotion center 95](#_Toc184378403)

[Major of Farakan health and social promotion center 95](#_Toc184378404)

[Maternity manager of Farakan health and social promotion center 95](#_Toc184378405)

[3.6 Priority between different methods of measuring gestational age 96](#_Toc184378406)

[Focus group of Accart-ville urban medical center 96](#_Toc184378407)

[Manager of Accart-ville urban medical center 96](#_Toc184378408)

[Maternity manager of Accart-ville urban medical center 97](#_Toc184378409)

[Focus group of Do medical center with surgical branch 97](#_Toc184378410)

[Gynecologist 1 98](#_Toc184378411)

[Gynecologist 2 99](#_Toc184378412)

[Chief physician of Do health district 99](#_Toc184378413)

[Manager for maternity care unit of Do medical center with surgical branch 100](#_Toc184378414)

[Focus group of Colma1 health and social promotion center 101](#_Toc184378415)

[Major of Colma1 health and social promotion center 101](#_Toc184378416)

[Maternity manager of Colma1 health and social promotion center 102](#_Toc184378417)

[Focus group of Farakan health and social promotion center 102](#_Toc184378418)

[Major of Farakan health and social promotion center 103](#_Toc184378419)

[Maternity manager of Farakan health and social promotion center 104](#_Toc184378420)

[3.7 Appointment for prenatal consultations with ultrasound measurement carried out 104](#_Toc184378421)

[Focus group of Accart-ville urban medical center 104](#_Toc184378422)

[Manager of Accart-ville urban medical center 106](#_Toc184378423)

[Maternity manager of Accart-ville urban medical center 107](#_Toc184378424)

[Focus group of Do medical center with surgical branch 108](#_Toc184378425)

[Gynecologist 1 109](#_Toc184378426)

[Gynecologist 2 109](#_Toc184378427)

[Chief physician of Do health district 110](#_Toc184378428)

[Manager for maternity care unit of Do medical center with surgical branch 111](#_Toc184378429)

[Focus group of Colma1 health and social promotion center 112](#_Toc184378430)

[Major of Colma1 health and social promotion center 113](#_Toc184378431)

[Maternity manager of Colma1 health and social promotion center 113](#_Toc184378432)

[Focus group of Farakan health and social promotion center 114](#_Toc184378433)

[Major of Farakan health and social promotion center 115](#_Toc184378434)

[Maternity manager of Farakan health and social promotion center 116](#_Toc184378435)

[3.8 Priority method if the date of the last period, fundal height and ultrasound are discordant 117](#_Toc184378436)

[Focus group of Accart-ville urban medical center 117](#_Toc184378437)

[Manager of Accart-ville urban medical center 118](#_Toc184378438)

[Maternity manager of Accart-ville urban medical center 118](#_Toc184378439)

[Focus group of Do medical center with surgical branch 119](#_Toc184378440)

[Gynecologist 1 120](#_Toc184378441)

[Gynecologist 2 120](#_Toc184378442)

[Chief physician of Do health district 121](#_Toc184378443)

[Manager for maternity care unit of Do medical center with surgical branch 122](#_Toc184378444)

[Focus group of Colma1 health and social promotion center 122](#_Toc184378445)

[Major of Colma1 health and social promotion center 123](#_Toc184378446)

[Maternity manager of Colma1 health and social promotion center 124](#_Toc184378447)

[Focus group of Farakan health and social promotion center 124](#_Toc184378448)

[Major of Farakan health and social promotion center 126](#_Toc184378449)

[Maternity manager of Farakan health and social promotion center 126](#_Toc184378450)

[4. Registration of preterm births 128](#_Toc184378451)

[Focus group of Accart-ville urban medical center 128](#_Toc184378452)

[Manager of Accart-ville urban medical center 128](#_Toc184378453)

[Maternity manager of Accart-ville urban medical center 129](#_Toc184378454)

[Focus group of Do medical center with surgical branch 130](#_Toc184378455)

[Gynecologist 1 130](#_Toc184378456)

[Gynecologist 2 131](#_Toc184378457)

[Chief physician of Do health district 132](#_Toc184378458)

[Manager for maternity care unit of Do medical center with surgical branch 133](#_Toc184378459)

[Focus group of Colma1 health and social promotion center 134](#_Toc184378460)

[Major of Colma1 health and social promotion center 135](#_Toc184378461)

[Maternity manager of Colma1 health and social promotion center 135](#_Toc184378462)

[Focus group of Farakan health and social promotion center 136](#_Toc184378463)

[Major of Farakan health and social promotion center 137](#_Toc184378464)

[Maternity manager of Farakan health and social promotion center 137](#_Toc184378465)

[5. Number of preterm births per month 138](#_Toc184378466)

[Focus group of Accart-ville urban medical center 138](#_Toc184378467)

[Manager of Accart-ville urban medical center 139](#_Toc184378468)

[Maternity manager of Accart-ville urban medical center 140](#_Toc184378469)

[Focus group of Do medical center with surgical branch 141](#_Toc184378470)

[Gynecologist 1 141](#_Toc184378471)

[Gynecologist 2 142](#_Toc184378472)

[Chief physician of Do health district 143](#_Toc184378473)

[Manager for maternity care unit of Do medical center with surgical branch 143](#_Toc184378474)

[Focus group of Colma1 health and social promotion center 144](#_Toc184378475)

[Major of Colma1 health and social promotion center 144](#_Toc184378476)

[Maternity manager of Colma1 health and social promotion center 145](#_Toc184378477)

[Focus group of Farakan health and social promotion center 146](#_Toc184378478)

[Major of Farakan health and social promotion center 147](#_Toc184378479)

[Maternity manager of Farakan health and social promotion center 147](#_Toc184378480)

[6. Types of challenges faced during preterm births 148](#_Toc184378481)

[Focus group of Accart-ville urban medical center 148](#_Toc184378482)

[Manager of Accart-ville urban medical center 149](#_Toc184378483)

[Maternity manager of Accart-ville urban medical center 151](#_Toc184378484)

[Focus group of Do medical center with surgical branch 153](#_Toc184378485)

[Gynecologist 1 155](#_Toc184378486)

[Gynecologist 2 156](#_Toc184378487)

[Chief physician of Do health district 159](#_Toc184378488)

[Manager for maternity care unit of Do medical center with surgical branch 159](#_Toc184378489)

[Focus group of Colma1 health and social promotion center 160](#_Toc184378490)

[Major of Colma1 health and social promotion center 162](#_Toc184378491)

[Maternity manager of Colma1 health and social promotion center 163](#_Toc184378492)

[Focus group of Farakan health and social promotion center 165](#_Toc184378493)

[Major of Farakan health and social promotion center 166](#_Toc184378494)

[Maternity manager of Farakan health and social promotion center 167](#_Toc184378495)

[7. Staff skills and existence of equipment for better care 169](#_Toc184378496)

[Focus group of Accart-ville urban medical center 169](#_Toc184378497)

[Manager of Accart-ville urban medical center 170](#_Toc184378498)

[Maternity manager of Accart-ville urban medical center 171](#_Toc184378499)

[Focus group of Do medical center with surgical branch 172](#_Toc184378500)

[Gynecologist 1 173](#_Toc184378501)

[Gynecologist 2 174](#_Toc184378502)

[Chief physician of Do health district 174](#_Toc184378503)

[Manager for maternity care unit of Do medical center with surgical branch 175](#_Toc184378504)

[Focus group of Colma1 health and social promotion center 176](#_Toc184378505)

[Major of Colma1 health and social promotion center 177](#_Toc184378506)

[Maternity manager of Colma1 health and social promotion center 177](#_Toc184378507)

[Focus group of Farakan health and social promotion center 178](#_Toc184378508)

[Major of Farakan health and social promotion center 179](#_Toc184378509)

[Maternity manager of Farakan health and social promotion center 179](#_Toc184378510)

[8. Services and care provided to premature babies and their mothers 181](#_Toc184378511)

[Focus group of Accart-ville urban medical center 181](#_Toc184378512)

[Manager of Accart-ville urban medical center 181](#_Toc184378513)

[Maternity manager of Accart-ville urban medical center 182](#_Toc184378514)

[Focus group of Do medical center with surgical branch 182](#_Toc184378515)

[Gynecologist 1 183](#_Toc184378516)

[Gynecologist 2 183](#_Toc184378517)

[Chief physician of Do health district 185](#_Toc184378518)

[Manager for maternity care unit of Do medical center with surgical branch 185](#_Toc184378519)

[Focus group of Colma1 health and social promotion center 186](#_Toc184378520)

[Major of Colma1 health and social promotion center 187](#_Toc184378521)

[Maternity manager of Colma1 health and social promotion center 188](#_Toc184378522)

[Focus group of Farakan health and social promotion center 190](#_Toc184378523)

[Major of Farakan health and social promotion center 191](#_Toc184378524)

[Maternity manager of Farakan health and social promotion center 192](#_Toc184378525)

[9. Health risks in premature infants 193](#_Toc184378526)

[Focus group of Accart-ville urban medical center 193](#_Toc184378527)

[Manager of Accart-ville urban medical center 196](#_Toc184378528)

[Maternity manager of Accart-ville urban medical center 198](#_Toc184378529)

[Focus group of Do medical center with surgical branch 199](#_Toc184378530)

[Gynecologist 1 200](#_Toc184378531)

[Gynecologist 2 200](#_Toc184378532)

[Chief physician of Do health district 202](#_Toc184378533)

[Manager for maternity care unit of Do medical center with surgical branch 203](#_Toc184378534)

[Focus group of Colma1 health and social promotion center 204](#_Toc184378535)

[Major of Colma1 health and social promotion center 205](#_Toc184378536)

[Maternity manager of Colma1 health and social promotion center 205](#_Toc184378537)

[Focus group of Farakan health and social promotion center 206](#_Toc184378538)

[Major of Farakan health and social promotion center 208](#_Toc184378539)

[Maternity manager of Farakan health and social promotion center 209](#_Toc184378540)

[10. Specificities of mothers of premature children 209](#_Toc184378541)

[Focus group of Accart-ville urban medical center 209](#_Toc184378542)

[Manager of Accart-ville urban medical center 211](#_Toc184378543)

[Maternity manager of Accart-ville urban medical center 212](#_Toc184378544)

[Focus group of Do medical center with surgical branch 214](#_Toc184378545)

[Gynecologist 1 214](#_Toc184378546)

[Gynecologist 2 215](#_Toc184378547)

[Chief physician of Do health district 216](#_Toc184378548)

[Manager for maternity care unit of Do medical center with surgical branch 218](#_Toc184378549)

[Focus group of Colma1 health and social promotion center 219](#_Toc184378550)

[Major of Colma1 health and social promotion center 220](#_Toc184378551)

[Maternity manager of Colma1 health and social promotion center 221](#_Toc184378552)

[Focus group of Farakan health and social promotion center 221](#_Toc184378553)

[Major of Farakan health and social promotion center 223](#_Toc184378554)

[Maternity manager of Farakan health and social promotion center 223](#_Toc184378555)

[11. Improvement in care of preterm birth 224](#_Toc184378556)

[Focus group of Accart-ville urban medical center 224](#_Toc184378557)

[Manager of Accart-ville urban medical center 227](#_Toc184378558)

[Maternity manager of Accart-ville urban medical center 229](#_Toc184378559)

[Focus group of Do medical center with surgical branch 231](#_Toc184378560)

[Gynecologist 1 234](#_Toc184378561)

[Gynecologist 2 236](#_Toc184378562)

[Chief physician of Do health district 239](#_Toc184378563)

[Manager for maternity care unit of Do medical center with surgical branch 241](#_Toc184378564)

[Focus group of Colma1 health and social promotion center 245](#_Toc184378565)

[Major of Colma1 health and social promotion center 250](#_Toc184378566)

[Maternity manager of Colma1 health and social promotion center 253](#_Toc184378567)

[Focus group of Farakan health and social promotion center 257](#_Toc184378568)

[Major of Farakan health and social promotion center 260](#_Toc184378569)

[Maternity manager of Farakan health and social promotion center 262](#_Toc184378570)

[12. Others 265](#_Toc184378571)

[Focus group of Accart-ville urban medical center 265](#_Toc184378572)

[Manager of Accart-ville urban medical center 265](#_Toc184378573)

[Maternity manager of Accart-ville urban medical center 267](#_Toc184378574)

[Focus group of Do medical center with surgical branch 267](#_Toc184378575)

[Gynecologist 1 270](#_Toc184378576)

[Gynecologist 2 270](#_Toc184378577)

[Chief physician of Do health district 271](#_Toc184378578)

[Manager for maternity care unit of Do medical center with surgical branch 272](#_Toc184378579)

[Focus group of Colma1 health and social promotion center 273](#_Toc184378580)

[Major of Colma1 health and social promotion center 274](#_Toc184378581)

[Maternity manager of Colma1 health and social promotion center 274](#_Toc184378582)

[Focus group of Farakan health and social promotion center 275](#_Toc184378583)

[Major of Farakan health and social promotion center 275](#_Toc184378584)

[Maternity manager of Farakan health and social promotion center 276](#_Toc184378585)

# Informed consent and presentation of participants

### Focus group of Accart-ville urban medical center

| [00:00:02-1](http://localhost:2300/file=C:/Users/USER/Desktop/ENTRETIENS%20PREMATURITE/CSPS-ACCART-VILLE/FOCUS%20GROUPE%20ACCART-VILLE-BON.mp3time=2100) Interviewer: CMU d'accart-ville, discussion de groupe, aujourd'hui c'est le 10/08/2024 je suis avec l'equipe de la maternité pour le focus groupe composé de maîeuticien d'état, d'accoucheuses auxiliaires et de sages femmes [00:00:19-9](http://localhost:2300/file=C:/User)  [00:00:19-9](http://localhost:2300/file=C:/Users/USER/Desktop/ENTRETIENS%20PREMATURITE/CSPS-ACCART-VILLE/FOCUS%20GROUPE%20ACCART-VILLE-BON.mp3time=19900) consentement éclairé: Bonjour, merci beaucoup d'avance pour votre disponibilité et le temps que vous nous accordez. Nous sommes une équipe de recherche de l’Université de Gand, de l'IRSS et de AFRICSanté. Nous menons une recherche qualitative sur la naissance prématurée dans les milieux urbains du Burkina Faso. Notre objectif est de mieux comprendre ce phénomène pour améliorer les services et les soins de santé destinés aux mères et aux nourrissons prématurés. Votre participation est essentielle pour nous aider à comprendre les défis spécifiques auxquels vous êtes confronté dans votre centre de santé et pour identifier les opportunités d’amélioration. L’entretien d’aujourd’hui sera confidentiel et les informations recueillies seront utilisées uniquement à des fins de recherche. Vous avez la liberté de ne pas répondre à toute question qui vous met mal à l'aise et vous pouvez arrêter l’entretien à tout moment. Avez-vous des questions avant que nous commencions ? [00:01:11-6](http://localhost:2300/file=C:/Users/USER/Desktop/ENTRETIENS)  [00:01:11-6](http://localhost:2300/file=C:/Users/USER/Desktop/ENTRETIENS%20PREMATURITE/CSPS-ACCART-VILLE/FOCUS%20GROUPE%20ACCART-VILLE-BON.mp3time=71600) Person 1: non nous n'avons pas de questions [00:01:13-7](http://localhost:2300/file=C:/Users/USER/Desktop/ENTRETIENS%20PREMATURITE/CSPS-ACCART-VILLE/FOCUS%20GROUPE%20ACCART-VILLE-BON.mp3time=73700)  [00:01:13-7](http://localhost:2300/file=C:/Users/USER/Desktop/ENTRETIENS) Interviewer: avec votre permission, pouvons-nous commencer l'entretien? [00:01:16-6](http://localhost:2300/file=C:/Users/USER/Desktop/ENTRETIENS)  [00:01:16-6](http://localhost:2300/file=C:/Users/USER/Desktop/ENTRETIENS%20PREMATURITE/CSPS-ACCART-VILLE/FOCUS%20GROUPE%20ACCART-VILLE-BON.mp3time=76600) Person 1: oui [00:01:17-0](http://localhost:23)  [00:01:17-0](http://localhost:2300/file=C:/Users/USER/Desktop/ENTRETIENS) Interviewer: est-ce que vous êtes d'accord pour participer à l'entretien? [00:01:20-1](http://localhost:2300/file=C:/Users/USER/Desktop/ENTRETIENS%20PREMATURITE/CSPS-ACCART-VILLE/FOCUS%20GROUPE%20ACCART-VILLE-BON.mp3time=80100)  [00:01:20-1](http://localhost:2300/file=C:/Users/USER/Desktop/ENTRETIENS%20PREMATURITE/CSPS-ACCART-VILLE/FOCUS%20GROUPE%20ACCART-VILLE-BON.mp3time=80100) Person 5: oui nous sommes d'accord [00:01:23-8](http://localhost:2300/file=C:/User)  [00:01:23-8](http://localhost:2300/file=C:/Users/USER/Desktop/ENTRETIENS) Interviewer: d'accord, je vais vous demandez de vous présenter, votre fonction, votre rôle et votre durée au sein de la formation sanitaire [00:01:34-2](http://localhost:2300/file=C:/Users/USER/Desktop/ENTRETIENS)  [00:01:34-4](http://localhost:2300/file=C:/Users/USER/Desktop/ENTRETIENS%20PREMATURITE/CSPS-ACCART-VILLE/FOCUS%20GROUPE%20ACCART-VILLE-BON.mp3time=94400) Person 1: sage-femme d'etat, cela fait une année que je suis à accart-ville nouvellement affectée [00:01:43-8](http://localhost:2300/file=C:/Users/USER/Desktop/ENTRETIENS%20PREMATURITE/CSPS-ACCART-VILLE/FOCUS%20GROUPE%20ACCART-VILLE-BON.mp3time=103800) |
| --- |

| 00:00:02-1 Interviewer: CMU d'Accart-Ville, discussion de groupe, aujourd'hui c'est le 10/08/2024. Je suis avec l'équipe de la maternité pour le focus groupe composé de maïeuticiens d'État, d'accoucheuses auxiliaires, et de sages-femmes. 00:00:19-9  00:00:19-9 Consentement éclairé: Bonjour, merci beaucoup d'avance pour votre disponibilité et le temps que vous nous accordez. Nous sommes une équipe de recherche de l’Université de Gand, de l'IRSS et de AFRICSanté. Nous menons une recherche qualitative sur la naissance prématurée dans les milieux urbains du Burkina Faso. Notre objectif est de mieux comprendre ce phénomène pour améliorer les services et les soins de santé destinés aux mères et aux nourrissons prématurés. Votre participation est essentielle pour nous aider à comprendre les défis spécifiques auxquels vous êtes confrontés dans votre centre de santé et pour identifier les opportunités d’amélioration. L’entretien d’aujourd’hui sera confidentiel et les informations recueillies seront utilisées uniquement à des fins de recherche. Vous avez la liberté de ne pas répondre à toute question qui vous met mal à l'aise et vous pouvez arrêter l’entretien à tout moment. Avez-vous des questions avant que nous commencions? 00:01:11-6  00:01:11-6 Person 1: Non, nous n'avons pas de questions. 00:01:13-7  00:01:13-7 Interviewer: Avec votre permission, pouvons-nous commencer l'entretien? 00:01:16-6  00:01:16-6 Person 1: Oui. 00:01:17-0  00:01:17-0 Interviewer: Est-ce que vous êtes d'accord pour participer à l'entretien? 00:01:20-1  00:01:20-1 Person 5: Oui, nous sommes d'accord. 00:01:23-8  00:01:23-8 Interviewer: D'accord, je vais vous demander de vous présenter, votre fonction, votre rôle et votre durée au sein de la formation sanitaire. 00:01:34-2  00:01:34-4 Person 1: Sage-femme d'État, cela fait une année que je suis à Accart-Ville, nouvellement affectée. 00:01:43-8 |
| --- |

| Interviewer (00:00:02-1): "Today is August 10, 2024. We're here at CMU d'Accart-Ville for a group discussion with the maternity team, which includes state midwives, auxiliary midwives, and midwives."  Interviewer (00:00:19-9): "Hello and thank you very much for your time and availability. We are a research team from Ghent University, IRSS, and AFRICSanté, conducting qualitative research on premature birth in urban areas of Burkina Faso. Our aim is to gain a deeper understanding of this issue in order to enhance services and care for mothers and premature infants. Your insights are crucial for us to comprehend the unique challenges you encounter at your health center and to identify areas for improvement. This interview is confidential and the information collected will be used solely for research purposes. You're free not to answer any questions you're uncomfortable with and can stop the interview at any point. Do you have any questions before we start?"  Person 1 (00:01:11-6): "No, we don't have any questions."  Interviewer (00:01:13-7): "With your permission, shall we begin the interview?"  Person 1 (00:01:16-6): "Yes."  Interviewer (00:01:17-0): "Do you consent to participate in this interview?"  Person 5 (00:01:20-1): "Yes, we agree."  Interviewer (00:01:23-8): "Great. Could you please introduce yourself, describe your role and function, and tell us how long you've been working at this health facility?"  Person 1 (00:01:34-4): "I'm a state midwife and have been assigned to Accart-Ville for a year, newly appointed. |
| --- |

### Manager of Accart-ville urban medical center

| [00:00:00-0](http://localhost:2300/file=C:/Users/USER/Desktop/ENTRETIENS%20PREMATURITE/CSPS-ACCART-VILLE/RESPONSABLE%20CMU%20ACCART-VILLE.MP3time=0)Interviewer: C'est le CMU D’accart-ville . Nous sommes le 10/08/2024. Nous sommes avec le médecin, Responsable de la formation sanitaire D’accart-Ville. Bonjour, Merci beaucoup d'avance pour votre disponibilité et le temps que vous nous accordez. Merci à l'avance. Nous sommes une équipe de recherche de l'Université de Gand de l'IRIS d'Afrique Santé. Nous menons des recherches qualitatives sur la naissance prématurée dans les milieux urbains du Burkina Faso. Notre objectif est de mieux comprendre ces phénomènes pour améliorer les services et les soins de santé destinés aux mères et aux nourrissons prématurés. Votre participation est essentielle pour nous aider à comprendre les défis spécifiques auxquels vous êtes confrontés dans votre centre de santé et pour identifier les opportunités d'amélioration. Donc, l'entretien de Violet sera confidentiel et les informations recueillies seront utilisées uniquement à des fins de recherche. Donc, vous avez la liberté de ne pas répondre à toute question qui vous met mal à l'aise et vous pouvez arrêter l'entretien à tout moment. Est-ce que vous avez des questions avant que nous nous commencions ? [00:01:07-6](http://localhost:2300/file=C:/Users/USER/Desktop/ENTRETIENS%20PREMATURITE/CSPS-ACCART-VILLE/RESPONSABLE%20CMU%20ACCART-VILLE.MP3time=67600)  [00:01:07-6](http://localhost:2300/file=C:/Users/USER/Desktop/ENTRETIENS%20PREMATURITE/CSPS-ACCART-VILLE/RESPONSABLE%20CMU%20ACCART-VILLE.MP3time=67600)Person 1: Non, c'est bon. [00:01:09-7](http://localhost:2300/file=C:/Users/USER/Desktop/ENTRETIENS%20PREMATURITE/CSPS-ACCART-VILLE/RESPONSABLE%20CMU%20ACCART-VILLE.MP3time=69700)  [00:01:09-7](http://localhost:2300/file=C:/Users/USER/Desktop/ENTRETIENS%20PREMATURITE/CSPS-ACCART-VILLE/RESPONSABLE%20CMU%20ACCART-VILLE.MP3time=69700)Interviewer: Ok, donc avec votre permission, nous pouvons commencer l'entretien. La première question pourriez-vous commencer par vous présenter ? Pas de nom ? Votre titre, votre fonction et votre rôle, s'il vous plaît ? Et depuis combien de temps occupez-vous ce poste au niveau de la formation sanitaire ? [00:01:29-3](http://localhost:2300/file=C:/Users/USER/Desktop/ENTRETIENS%20PREMATURITE/CSPS-ACCART-VILLE/RESPONSABLE%20CMU%20ACCART-VILLE.MP3time=89300)  [00:01:29-3](http://localhost:2300/file=C:/Users/USER/Desktop/ENTRETIENS%20PREMATURITE/CSPS-ACCART-VILLE/RESPONSABLE%20CMU%20ACCART-VILLE.MP3time=89300) Person 1: Je suis médecin généraliste je suis le responsables du centre médical urbain d’accart-Ville. je suis au niveau du centre depuis décembre 2021. C'est la deuxième année comme ça . Donc moi je m'occupe des questions administratives, et puis j'assure aussi les consultations médicales. [00:02:03-9](http://localhost:2300/file=C:/Users/USER/Desktop/ENTRETIENS%20PREMATURITE/CSPS-ACCART-VILLE/RESPONSABLE%20CMU%20ACCART-VILLE.MP3time=123900) |
| --- |

| 00:00:00-0 Interviewer: C'est le CMU d'Accart-Ville. Nous sommes le 10/08/2024. Nous sommes avec le médecin, responsable de la formation sanitaire d'Accart-Ville. Bonjour, merci beaucoup d'avance pour votre disponibilité et le temps que vous nous accordez. Merci à l'avance. Nous sommes une équipe de recherche de l'Université de Gand de l'IRIS d'Afrique Santé. Nous menons des recherches qualitatives sur la naissance prématurée dans les milieux urbains du Burkina Faso. Notre objectif est de mieux comprendre ces phénomènes pour améliorer les services et les soins de santé destinés aux mères et aux nourrissons prématurés. Votre participation est essentielle pour nous aider à comprendre les défis spécifiques auxquels vous êtes confrontés dans votre centre de santé et pour identifier les opportunités d'amélioration. Donc, l'entretien de Violet sera confidentiel et les informations recueillies seront utilisées uniquement à des fins de recherche. Donc, vous avez la liberté de ne pas répondre à toute question qui vous met mal à l'aise et vous pouvez arrêter l'entretien à tout moment. Est-ce que vous avez des questions avant que nous commencions ?  00:01:07-6 Person 1: Non, c'est bon.  00:01:09-7 Interviewer: Ok, donc avec votre permission, nous pouvons commencer l'entretien. La première question : pourriez-vous commencer par vous présenter ? Pas de nom ? Votre titre, votre fonction et votre rôle, s'il vous plaît ? Et depuis combien de temps occupez-vous ce poste au niveau de la formation sanitaire ?  00:01:29-3 Person 1: Je suis médecin généraliste. Je suis le responsable du centre médical urbain d'Accart-Ville. Je suis au niveau du centre depuis décembre 2021. C'est la deuxième année comme ça. Donc, moi je m'occupe des questions administratives, et puis j'assure aussi les consultations médicales. |
| --- |

| **[00:00:00-0] Interviewer**: Good day. We're here at the Accart-Ville CMU on the 10th of August, 2024. I have the pleasure of speaking with the doctor in charge of the Accart-Ville health facility. Thank you very much for your time and willingness to participate in our interview. We are a research team from Ghent University, part of IRIS d'Afrique Santé, conducting qualitative research on premature birth in urban Burkina Faso. Our aim is to gain a deeper understanding of this issue to enhance services and healthcare for mothers and premature infants. Your insights are crucial in helping us identify the unique challenges faced at your health center and potential areas for improvement. Please know that this interview is confidential, and all gathered information will be used solely for research. You are free to decline answering any question or to end the interview whenever you wish. Do you have any questions before we start?  **[00:01:07-6] Person 1**: No, I’m fine with proceeding.  **[00:01:09-7] Interviewer**: Excellent, with your consent, we'll begin. For our first question: Could you please introduce yourself, focusing on your title, role, and how long you've been in your current position? No need to mention your name.  **[00:01:29-3] Person 1**: I am a general practitioner and the manager of the Accart-Ville urban medical center. I've been with the center since December 2021, making this my second year. My responsibilities include administrative duties and providing medical consultations. |
| --- |

### Maternity manager of Accart-ville urban medical center

| [00:00:01-8](http://localhost:2300/file=C:/Users/USER/Desktop/ENTRETIENS%20PREMATURITE/CSPS-ACCART-VILLE/RESPONSABLE%20MATERNITE%20CMU%20ACCART-VILLE.MP3time=1800) Interviewer: cmu d'accart-ville aujourd'hui c'est le 10/08/2024 je suis avec le responsable de la maternité du csps d'accart-ville donc le consentement éclairé  Bonjour, merci beaucoup d'avance pour votre disponibilité et le temps que vous nous accordez. Nous sommes une équipe de recherche de l'Université de Gand, de l'IRSS et de AFRICSanté. Nous menons une recherche qualitative sur la naissance prématurée dans les milieux urbains du Burkina Faso. Notre objectif est de mieux comprendre ce phénomène pour améliorer les services et les soins de santé destinés aux mères et aux nourrissons prématurés. Votre participation est essentielle pour nous aider à comprendre les défis spécifiques auxquels vous êtes confronté dans votre centre de santé et pour identifier les opportunités d'amélioration. L'entretien d'aujourd'hui sera confidentiel et les informations recueillies seront utilisées uniquement à des fins de recherche. Vous avez la liberté de ne pas répondre à toute question qui vous met mal à l'aise et vous pouvez arrêter l'entretien à tout moment. Avez-vous des questions avant que nous commencions ? [00:01:04-9](http://localhost:2300/file=C:/Users/USER/Desktop/ENTRETIENS%20PREMATURITE/CSPS-ACCART-VILLE/RESPONSABLE%20MATERNITE%20CMU%20ACCART-VILLE.MP3time=64900)  [00:01:04-9](http://localhost:2300/file=C:/Users/USER/Desktop/ENTRETIENS%20PREMATURITE/CSPS-ACCART-VILLE/RESPONSABLE%20MATERNITE%20CMU%20ACCART-VILLE.MP3time=64900) Person 1: NON [00:01:05-6](http://localhost:2300/file=C:/Users/USER/Desktop/ENTRETIENS%20PREMATURITE/CSPS-ACCART-VILLE/RESPONSABLE%20MATERNITE%20CMU%20ACCART-VILLE.MP3time=65600)  [00:01:05-6](http://localhost:2300/file=C:/Users/USER/Desktop/ENTRETIENS%20PREMATURITE/CSPS-ACCART-VILLE/RESPONSABLE%20MATERNITE%20CMU%20ACCART-VILLE.MP3time=65600) Interviewer: OK avec votre permission, pouvons nous commencer l'entretien? [00:01:08-9](http://localhost:2300/file=C:/Users/USER/Desktop/ENTRETIENS%20PREMATURITE/CSPS-ACCART-VILLE/RESPONSABLE%20MATERNITE%20CMU%20ACCART-VILLE.MP3time=68900)  [00:01:08-9](http://localhost:2300/file=C:/Users/USER/Desktop/ENTRETIENS%20PREMATURITE/CSPS-ACCART-VILLE/RESPONSABLE%20MATERNITE%20CMU%20ACCART-VILLE.MP3time=68900) Person 1: oui nous pouvons commencer [00:01:10-3](http://localhost:2300/file=C:/Users/USER/Desktop/ENTRETIENS%20PREMATURITE/CSPS-ACCART-VILLE/RESPONSABLE%20MATERNITE%20CMU%20ACCART-VILLE.MP3time=70300)  [00:01:10-3](http://localhost:2300/file=C:/Users/USER/Desktop/ENTRETIENS%20PREMATURITE/CSPS-ACCART-VILLE/RESPONSABLE%20MATERNITE%20CMU%20ACCART-VILLE.MP3time=70300) Interviewer: ok donc la première question que je vais vous poser c'est de vous presenter sans le nom, titre fonction, rôle et la durée d'occupation du poste au sein du centre de santé. [00:01:25-8](http://localhost:2300/file=C:/Users/USER/Desktop/ENTRETIENS%20PREMATURITE/CSPS-ACCART-VILLE/RESPONSABLE%20MATERNITE%20CMU%20ACCART-VILLE.MP3time=85800)  [00:01:25-8](http://localhost:2300/file=C:/Users/USER/Desktop/ENTRETIENS%20PREMATURITE/CSPS-ACCART-VILLE/RESPONSABLE%20MATERNITE%20CMU%20ACCART-VILLE.MP3time=85800) Person 1: moi je suis un attaché de santé en soin obstetricaux et gynecologiques, j'ai une année d'ancienneté au cmu accart-ville en tant que responsble maternité [00:01:38-1](http://localhost:2300/file=C:/Users/USER/Desktop/ENTRETIENS%20PREMATURITE/CSPS-ACCART-VILLE/RESPONSABLE%20MATERNITE%20CMU%20ACCART-VILLE.MP3time=98100) |
| --- |

| 00:00:01-8 Interviewer: CMU d'Accart-Ville, aujourd'hui c'est le 10/08/2024. Je suis avec le responsable de la maternité du CSPS d'Accart-Ville, donc le consentement éclairé.  Bonjour, merci beaucoup d'avance pour votre disponibilité et le temps que vous nous accordez. Nous sommes une équipe de recherche de l'Université de Gand, de l'IRSS et de AFRICSanté. Nous menons une recherche qualitative sur la naissance prématurée dans les milieux urbains du Burkina Faso. Notre objectif est de mieux comprendre ce phénomène pour améliorer les services et les soins de santé destinés aux mères et aux nourrissons prématurés. Votre participation est essentielle pour nous aider à comprendre les défis spécifiques auxquels vous êtes confronté dans votre centre de santé et pour identifier les opportunités d'amélioration. L'entretien d'aujourd'hui sera confidentiel et les informations recueillies seront utilisées uniquement à des fins de recherche. Vous avez la liberté de ne pas répondre à toute question qui vous met mal à l'aise et vous pouvez arrêter l'entretien à tout moment. Avez-vous des questions avant que nous commencions? 00:01:04-9  00:01:04-9 Person 1: Non. 00:01:05-6  00:01:05-6 Interviewer: OK, avec votre permission, pouvons-nous commencer l'entretien? 00:01:08-9  00:01:08-9 Person 1: Oui, nous pouvons commencer. 00:01:10-3  00:01:10-3 Interviewer: OK, donc la première question que je vais vous poser c'est de vous présenter sans le nom, titre, fonction, rôle et la durée d'occupation du poste au sein du centre de santé. 00:01:25-8  00:01:25-8 Person 1: Moi, je suis un attaché de santé en soins obstétricaux et gynécologiques. J'ai une année d'ancienneté au CMU Accart-Ville en tant que responsable maternité. 00:01:38-1 |
| --- |

| **[00:00:01-8] Interviewer**: Welcome to CMU of Accart-Ville. Today's date is August 10, 2024. We're here with the maternity manager of CSPS of Accart-Ville to discuss informed consent. Thank you so much for your time and willingness to participate in our interview. Our research team from Ghent University, along with IRSS and AFRICSanté, is conducting a qualitative study on premature births in urban Burkina Faso. Our objective is to gain a deeper understanding of these occurrences to enhance healthcare services for mothers and premature infants. Your insights will be invaluable in identifying the unique challenges your health center faces and in pinpointing areas for potential improvement. Please be assured that this interview is confidential, and the information collected will be exclusively used for research purposes. You are free to decline answering any questions that may cause discomfort, and you can choose to end the interview at any moment. Are there any questions or concerns you have before we start?  **[00:01:04-9] Person 1**: No.  **[00:01:05-6] Interviewer**: Alright, with your consent, shall we start the interview?  **[00:01:08-9] Person 1**: Yes, we can begin.  **[00:01:10-3] Interviewer**: Okay, for the first question, please introduce yourself. Include your title, function, role, and how long you have held your position at the health center, but omit your name.  **[00:01:25-8] Person 1**: I am a health officer specializing in obstetric and gynecological care. I've been at CMU Accart-Ville for one year, serving as the head of maternity. |
| --- |

### Focus group of Do medical center with surgical branch

| [00:00:01-7](http://localhost:2300/file=C:/Users/USER/Desktop/ENTRETIENS%20PREMATURITE/CMA-DO/FOCUS%20GROUPE%20CMA-DO.mp3time=1700) Interviewer: Aujourd'hui c'est le 22/ 08/ 2024. Discussion de groupe avec l'équipe de sages-femmes et de maîeuticiens du CMA de DÔ. Ok, donc je vais vous lire le consentement éclairé. Bonjour, merci beaucoup d'avance pour votre disponibilité et le temps que vous nous accordez. Nous sommes une équipe de recherche de l’Université de Gand, de l'IRSS et de AFRICSanté. Nous menons une recherche qualitative sur la naissance prématurée dans les milieux urbains du Burkina Faso. Notre objectif est de mieux comprendre ce phénomène pour améliorer les services et les soins de santé destinés aux mères et aux nourrissons prématurés. Votre participation est essentielle pour nous aider à comprendre les défis spécifiques auxquels vous êtes confronté dans votre centre de santé et pour identifier les opportunités d’amélioration.  L’entretien d’aujourd’hui sera confidentiel et les informations recueillies seront utilisées uniquement à des fins de recherche. Vous avez la liberté de ne pas répondre à toute question qui vous met mal à l'aise et vous pouvez arrêter l’entretien à tout moment. Avez-vous des questions avant que nous commencions ? . Est-ce que vous avez des questions avant que nous ne commencions? Ok donc je vais déjà vous demander de vous présenter sans le nom, le titre, la fonction, votre rôle et depuis combien de temps vous occupez donc ce poste? [00:01:14-8](http://localhost:2300/file=C:/Users/USER/Desktop/ENTRETIENS%20PREMATURITE/CMA-DO/FOCUS%20GROUPE%20CMA-DO.mp3time=74800)  [00:01:14-8](http://localhost:2300/file=C:/Users/USER/Desktop/ENTRETIENS%20PREMATURITE/CMA-DO/FOCUS%20GROUPE%20CMA-DO.mp3time=74800) Person 3: Comment est ce qu'on doit se presenter? [00:01:18-1](http://localhost:2300/file=C:/Users/USER/Desktop/ENTRETIENS%20PREMATURITE/CMA-DO/FOCUS%20GROUPE%20CMA-DO.mp3time=78100)  [00:01:18-1](http://localhost:2300/file=C:/Users/USER/Desktop/ENTRETIENS%20PREMATURITE/CMA-DO/FOCUS%20GROUPE%20CMA-DO.mp3time=78100) Interviewer: C'est sans le nom, la fonction, le titre, le rôle et le nombre d'années que vous occupez du poste pour le centre de santé. [00:01:33-1](http://localhost:2300/file=C:/Users/USER/Desktop/ENTRETIENS%20PREMATURITE/CMA-DO/FOCUS%20GROUPE%20CMA-DO.mp3time=93100)  [00:01:33-1](http://localhost:2300/file=C:/Users/USER/Desktop/ENTRETIENS%20PREMATURITE/CMA-DO/FOCUS%20GROUPE%20CMA-DO.mp3time=93100) Person 1: Sage-femme au CMA de DÔ, il y a six ans de service ici au CMA de DÔ. [00:01:47-4](http://localhost:2300/file=C:/Users/USER/Desktop/ENTRETIENS%20PREMATURITE/CMA-DO/FOCUS%20GROUPE%20CMA-DO.mp3time=107400)  [00:01:47-4](http://localhost:2300/file=C:/Users/USER/Desktop/ENTRETIENS%20PREMATURITE/CMA-DO/FOCUS%20GROUPE%20CMA-DO.mp3time=107400) Person 2: Sage-femme au CMA de DÔ, ça fait douze ans que je suis au CMA . [00:01:58-4](http://localhost:2300/file=C:/Users/USER/Desktop/ENTRETIENS%20PREMATURITE/CMA-DO/FOCUS%20GROUPE%20CMA-DO.mp3time=118400)  [00:01:58-4](http://localhost:2300/file=C:/Users/USER/Desktop/ENTRETIENS%20PREMATURITE/CMA-DO/FOCUS%20GROUPE%20CMA-DO.mp3time=118400) Person 3: Sage-femme au CMA de DÔ, ça fait quatre ans que je suis responsable de la salle d'accouchement. [00:02:07-7](http://localhost:2300/file=C:/Users/USER/Desktop/ENTRETIENS%20PREMATURITE/CMA-DO/FOCUS%20GROUPE%20CMA-DO.mp3time=127700)  [00:02:07-7](http://localhost:2300/file=C:/Users/USER/Desktop/ENTRETIENS%20PREMATURITE/CMA-DO/FOCUS%20GROUPE%20CMA-DO.mp3time=127700) Person 4: Maîeuticien d'état au CMA de DÔ, je suis en salle d'accouchement. [00:02:14-2](http://localhost:2300/file=C:/Users/USER/Desktop/ENTRETIENS%20PREMATURITE/CMA-DO/FOCUS%20GROUPE%20CMA-DO.mp3time=134200)  [00:02:14-2](http://localhost:2300/file=C:/Users/USER/Desktop/ENTRETIENS%20PREMATURITE/CMA-DO/FOCUS%20GROUPE%20CMA-DO.mp3time=134200) Person 5: Sage-femme d'état, six ans d'ancienneté et je suis en salle d'accouchement. [00:02:23-2](http://localhost:2300/file=C:/Users/USER/Desktop/ENTRETIENS%20PREMATURITE/CMA-DO/FOCUS%20GROUPE%20CMA-DO.mp3time=143200)  [00:02:23-2](http://localhost:2300/file=C:/Users/USER/Desktop/ENTRETIENS%20PREMATURITE/CMA-DO/FOCUS%20GROUPE%20CMA-DO.mp3time=143200) Person 6: Maîeuticien d'état, 13 ans d'ancienneté, je suis en suite de couche, responsable. [00:02:31-8](http://localhost:2300/file=C:/Users/USER/Desktop/ENTRETIENS%20PREMATURITE/CMA-DO/FOCUS%20GROUPE%20CMA-DO.mp3time=151800)  [00:02:31-8](http://localhost:2300/file=C:/Users/USER/Desktop/ENTRETIENS%20PREMATURITE/CMA-DO/FOCUS%20GROUPE%20CMA-DO.mp3time=151800) Person 7: Sage-femme, cinq ans au CMA de DÔ, je suis en post-nat. [00:02:38-6](http://localhost:2300/file=C:/Users/USER/Desktop/ENTRETIENS%20PREMATURITE/CMA-DO/FOCUS%20GROUPE%20CMA-DO.mp3time=158600)  [00:02:38-6](http://localhost:2300/file=C:/Users/USER/Desktop/ENTRETIENS%20PREMATURITE/CMA-DO/FOCUS%20GROUPE%20CMA-DO.mp3time=158600) Person 8: Sage-femme, dix ans au CMA de DÔ. [00:02:51-8](http://localhost:2300/file=C:/Users/USER/Desktop/ENTRETIENS%20PREMATURITE/CMA-DO/FOCUS%20GROUPE%20CMA-DO.mp3time=171800)  [00:02:51-8](http://localhost:2300/file=C:/Users/USER/Desktop/ENTRETIENS%20PREMATURITE/CMA-DO/FOCUS%20GROUPE%20CMA-DO.mp3time=171800) Person 9: Je suis sage-femme, onze ans de service au CMA de DÔ. [00:03:06-7](http://localhost:2300/file=C:/Users/USER/Desktop/ENTRETIENS%20PREMATURITE/CMA-DO/FOCUS%20GROUPE%20CMA-DO.mp3time=186700) |
| --- |

| 00:00:01-7 Interviewer: Aujourd'hui, c'est le 22/08/2024. Discussion de groupe avec l'équipe de sage-femmes et de maïeuticiens du CMA de Dô. Ok, donc je vais vous lire le consentement éclairé. Bonjour, merci beaucoup d'avance pour votre disponibilité et le temps que vous nous accordez. Nous sommes une équipe de recherche de l'Université de Gand, de l'IRSS et d'AFRICSanté. Nous menons une recherche qualitative sur la naissance prématurée dans les milieux urbains du Burkina Faso. Notre objectif est de mieux comprendre ce phénomène pour améliorer les services et les soins de santé destinés aux mères et aux nourrissons prématurés. Votre participation est essentielle pour nous aider à comprendre les défis spécifiques auxquels vous êtes confronté dans votre centre de santé et pour identifier les opportunités d’amélioration. L’entretien d’aujourd’hui sera confidentiel et les informations recueillies seront utilisées uniquement à des fins de recherche. Vous avez la liberté de ne pas répondre à toute question qui vous met mal à l'aise et vous pouvez arrêter l’entretien à tout moment. Avez-vous des questions avant que nous commencions ? Est-ce que vous avez des questions avant que nous ne commencions ? Ok, donc je vais déjà vous demander de vous présenter sans le nom, le titre, la fonction, votre rôle et depuis combien de temps vous occupez donc ce poste ?  00:01:14-8 Person 3: Comment est-ce qu'on doit se présenter ?  00:01:18-1 Interviewer: C'est sans le nom, la fonction, le titre, le rôle et le nombre d'années que vous occupez du poste pour le centre de santé.  00:01:33-1 Person 1: Sage-femme au CMA de Dô, il y a six ans de service ici au CMA de Dô.  00:01:47-4 Person 2: Sage-femme au CMA de Dô, ça fait douze ans que je suis au CMA.  00:01:58-4 Person 3: Sage-femme au CMA de Dô, ça fait quatre ans que je suis responsable de la salle d'accouchement.  00:02:07-7 Person 4: Maïeuticien d'état au CMA de Dô, je suis en salle d'accouchement.  00:02:14-2 Person 5: Sage-femme d'état, six ans d'ancienneté et je suis en salle d'accouchement.  00:02:23-2 Person 6: Maïeuticien d'état, 13 ans d'ancienneté, je suis en suite de couche, responsable.  00:02:31-8 Person 7: Sage-femme, cinq ans au CMA de Dô, je suis en post-nat.  00:02:38-6 Person 8: Sage-femme, dix ans au CMA de Dô.  00:02:51-8 Person 9: Je suis sage-femme, onze ans de service au CMA de Dô |
| --- |

| **[00:00:01-7] Interviewer**: Today's date is August 22, 2024. We are here for a group discussion with the team of midwives from CMA de Dô. First, I will read the informed consent. Thank you all for your time and willingness to participate. We represent a research team from Ghent University, IRSS, and AFRICSanté, conducting a study on premature births in urban Burkina Faso. Our aim is to understand this issue better to enhance services and care for mothers and premature infants. Your experiences are crucial for us to identify challenges and opportunities for improvement at your health center. This interview is confidential, and all information will be used solely for research. You may choose not to answer any uncomfortable questions and can stop the interview at any time. Before we start, do you have any questions? Now, I'd like you to introduce yourselves, stating your title, role, and how long you've been in your current position at the health center, but please omit your names.  **[00:01:14-8] Person 3**: How exactly should we introduce ourselves?  **[00:01:18-1] Interviewer**: Please introduce yourselves without your name, but include your position, title, role, and the duration you've held the position in the health center.  **[00:01:33-1] Person 1**: I am a midwife at the CMA de Dô, with six years of service here.  **[00:01:47-4] Person 2**: I've been a midwife at CMA in Dô for twelve years.  **[00:01:58-4] Person 3**: As a midwife at CMA de Dô, I've been in charge of the delivery room for four years.  **[00:02:07-7] Person 4**: I am a state midwife at CMA in Dô, working in the delivery room.  **[00:02:14-2] Person 5**: State midwife with six years of tenure, also in the delivery room.  **[00:02:23-2] Person 6**: State midwife for 13 years, currently responsible for the maternity ward.  **[00:02:31-8] Person 7**: Midwife for five years at CMA de Dô, working in post-natal care.  **[00:02:38-6] Person 8**: I've been a midwife for ten years at CMA de Dô.  **[00:02:51-8] Person 9**: I am a midwife with eleven years of service at CMA de Dô. |
| --- |

### Gynecologist 1

| [00:00:01-7](http://localhost:2300/file=C:/Users/USER/Desktop/ENTRETIENS%20PREMATURITE/CMA-DO/GENYCOLOGUE%2001.mp3time=1700) Interviewer: Aujourd'hui c'est 17/08/2024, entretien avec le gynécologue du CMA de Dô,Donc je vais vous lire le consentement éclairé. Bonjour, merci beaucoup d'avance pour votre disponibilité et le temps que vous nous accordez. Nous sommes une équipe de recherche de l’Université de Gand, de l'IRSS et de AFRICSanté. Nous menons une recherche qualitative sur la naissance prématurée dans les milieux urbains du Burkina Faso. Notre objectif est de mieux comprendre ce phénomène pour améliorer les services et les soins de santé destinés aux mères et aux nourrissons prématurés. Votre participation est essentielle pour nous aider à comprendre les défis spécifiques auxquels vous êtes confronté dans votre centre de santé et pour identifier les opportunités d’amélioration.  L’entretien d’aujourd’hui sera confidentiel et les informations recueillies seront utilisées uniquement à des fins de recherche. Vous avez la liberté de ne pas répondre à toute question qui vous met mal à l'aise et vous pouvez arrêter l’entretien à tout moment. Je vais vous demandez déjà si vous donnez votre accord par rapport à la lecture de ce consentement éclairé, avant que nous ne poursuivons. [00:01:14-0](http://localhost:2300/file=C:/Users/USER/Desktop/ENTRETIENS%20PREMATURITE/CMA-DO/GENYCOLOGUE%2001.mp3time=74000)  [00:01:14-0](http://localhost:2300/file=C:/Users/USER/Desktop/ENTRETIENS%20PREMATURITE/CMA-DO/GENYCOLOGUE%2001.mp3time=74000) Person 1: Ok, donc je marque mon accord, mon consentement pour la dite enquête. Voilà, il n'y a pas de problème. Je suis le médecin gynécologue, responsable du service de maternité au CMA de DO. [00:01:34-1](http://localhost:2300/file=C:/Users/USER/Desktop/ENTRETIENS%20PREMATURITE/CMA-DO/GENYCOLOGUE%2001.mp3time=94100)  [00:01:34-1](http://localhost:2300/file=C:/Users/USER/Desktop/ENTRETIENS%20PREMATURITE/CMA-DO/GENYCOLOGUE%2001.mp3time=94100) Interviewer: Depuis quand vous occupez ce poste ? [00:01:37-1](http://localhost:2300/file=C:/Users/USER/Desktop/ENTRETIENS%20PREMATURITE/CMA-DO/GENYCOLOGUE%2001.mp3time=97100)  [00:01:37-1](http://localhost:2300/file=C:/Users/USER/Desktop/ENTRETIENS%20PREMATURITE/CMA-DO/GENYCOLOGUE%2001.mp3time=97100) Person 1: J'occupe ce poste depuis 13 ans. [00:01:41-9](http://localhost:2300/file=C:/Users/USER/Desktop/ENTRETIENS%20PREMATURITE/CMA-DO/GENYCOLOGUE%2001.mp3time=101900) |
| --- |

| 00:00:01-7 Interviewer: Aujourd'hui c'est le 17/08/2024, entretien avec le gynécologue du CMA de Dô. Donc je vais vous lire le consentement éclairé. Bonjour, merci beaucoup d'avance pour votre disponibilité et le temps que vous nous accordez. Nous sommes une équipe de recherche de l’Université de Gand, de l'IRSS et d'AFRICSanté. Nous menons une recherche qualitative sur la naissance prématurée dans les milieux urbains du Burkina Faso. Notre objectif est de mieux comprendre ce phénomène pour améliorer les services et les soins de santé destinés aux mères et aux nourrissons prématurés. Votre participation est essentielle pour nous aider à comprendre les défis spécifiques auxquels vous êtes confronté dans votre centre de santé et pour identifier les opportunités d’amélioration. L’entretien d’aujourd’hui sera confidentiel et les informations recueillies seront utilisées uniquement à des fins de recherche. Vous avez la liberté de ne pas répondre à toute question qui vous met mal à l'aise et vous pouvez arrêter l’entretien à tout moment. Je vais vous demandez déjà si vous donnez votre accord par rapport à la lecture de ce consentement éclairé, avant que nous ne poursuivions.  00:01:14-0 Person 1: Ok, donc je marque mon accord, mon consentement pour la dite enquête. Voilà, il n'y a pas de problème. Je suis le médecin gynécologue, responsable du service de maternité au CMA de Dô.  00:01:34-1 Interviewer: Depuis quand vous occupez ce poste ?  00:01:37-1 Person 1: J'occupe ce poste depuis 13 ans. |
| --- |

| **[00:00:01-7] Interviewer**: Today is August 17, 2024. We're conducting an interview with the gynecologist from the CMA de Dô. Before we start, I'll read the informed consent. Thank you for your willingness to participate and for the time you're providing us. We're a team from Ghent University, IRSS, and AFRICSanté, conducting qualitative research on premature birth in urban areas of Burkina Faso. Our aim is to deepen our understanding of this issue to enhance healthcare services for mothers and premature infants. Your input is vital in identifying the specific challenges at your health center and exploring improvement opportunities. Please note that this interview is confidential, and all collected information will be used strictly for research purposes. You're free to not answer any uncomfortable questions and can stop the interview at any time. May I have your consent to proceed with reading this informed consent before we continue?  **[00:01:14-0] Person 1**: Okay, I agree to participate in this research. There's no problem. I am the gynecologist in charge of the maternity department at the CMA de Dô.  **[00:01:34-1] Interviewer**: How long have you held this position?  **[00:01:37-1] Person 1**: I've been in this position for 13 years. |
| --- |

### Gynecologist 2

| [00:00:00-0](http://localhost:2300/file=C:/Users/USER/Desktop/ENTRETIENS%20PREMATURITE/CMA-DO/GENYCOLOGUE%2002.mp3time=0) Interviewer: Aujourd'hui c'est le 17/08/2024. Je suis avec le gynécologue du CMA de DO. Donc je vais vous lire le consentement éclairé. Bonjour, merci beaucoup d'avance pour votre disponibilité et le temps que vous nous accordez. Nous sommes une équipe de recherche de l’Université de Gand, de l'IRSS et de AFRICSanté. Nous menons une recherche qualitative sur la naissance prématurée dans les milieux urbains du Burkina Faso. Notre objectif est de mieux comprendre ce phénomène pour améliorer les services et les soins de santé destinés aux mères et aux nourrissons prématurés. Votre participation est essentielle pour nous aider à comprendre les défis spécifiques auxquels vous êtes confronté dans votre centre de santé et pour identifier les opportunités d’amélioration.  L’entretien d’aujourd’hui sera confidentiel et les informations recueillies seront utilisées uniquement à des fins de recherche. Vous avez la liberté de ne pas répondre à toute question qui vous met mal à l'aise et vous pouvez arrêter l’entretien à tout moment. Je vais vous demander déjà de voir si vous allez nous donner votre accord par rapport à la lecture de ce consentement éclairé avant que nous ne poursuivions. [00:01:14-1](http://localhost:2300/file=C:/Users/USER/Desktop/ENTRETIENS%20PREMATURITE/CMA-DO/GENYCOLOGUE%2002.mp3time=74100)  [00:01:14-1](http://localhost:2300/file=C:/Users/USER/Desktop/ENTRETIENS%20PREMATURITE/CMA-DO/GENYCOLOGUE%2002.mp3time=74100) Person 1: Je marque mon accord pour participer à cette étude. Je suis médecin gynécologue. Ça fait 4 ans que je suis ici. [00:01:27-5](http://localhost:2300/file=C:/Users/USER/Desktop/ENTRETIENS%20PREMATURITE/CMA-DO/GENYCOLOGUE%2002.mp3time=87500)  [00:01:27-5](http://localhost:2300/file=C:/Users/USER/Desktop/ENTRETIENS%20PREMATURITE/CMA-DO/GENYCOLOGUE%2002.mp3time=87500) Interviewer: Quel est votre rôle au sein du CMA ici? [00:01:31-0](http://localhost:2300/file=C:/Users/USER/Desktop/ENTRETIENS%20PREMATURITE/CMA-DO/GENYCOLOGUE%2002.mp3time=91000)  [00:01:31-0](http://localhost:2300/file=C:/Users/USER/Desktop/ENTRETIENS%20PREMATURITE/CMA-DO/GENYCOLOGUE%2002.mp3time=91000) Person 1: Au niveau du poste que j'occupe, il n'y a pas un rôle spécifique parce qu'il y a un responsable de service et une surveillance d'unité de soins. Je suis le deuxième gynécologue. En tant que gynécologue, je participe aux soins, à la consultation et à la prise en charge des urgences. [00:01:56-9](http://localhost:2300/file=C:/Users/USER/Desktop/ENTRETIENS%20PREMATURITE/CMA-DO/GENYCOLOGUE%2002.mp3time=116900) |
| --- |

| 00:00:00-0 Interviewer: Aujourd'hui c'est le 17/08/2024. Je suis avec le gynécologue du CMA de Dô. Donc je vais vous lire le consentement éclairé. Bonjour, merci beaucoup d'avance pour votre disponibilité et le temps que vous nous accordez. Nous sommes une équipe de recherche de l’Université de Gand, de l'IRSS et d'AFRICSanté. Nous menons une recherche qualitative sur la naissance prématurée dans les milieux urbains du Burkina Faso. Notre objectif est de mieux comprendre ce phénomène pour améliorer les services et les soins de santé destinés aux mères et aux nourrissons prématurés. Votre participation est essentielle pour nous aider à comprendre les défis spécifiques auxquels vous êtes confronté dans votre centre de santé et pour identifier les opportunités d’amélioration. L’entretien d’aujourd’hui sera confidentiel et les informations recueillies seront utilisées uniquement à des fins de recherche. Vous avez la liberté de ne pas répondre à toute question qui vous met mal à l'aise et vous pouvez arrêter l’entretien à tout moment. Je vais vous demander déjà de voir si vous allez nous donner votre accord par rapport à la lecture de ce consentement éclairé avant que nous ne poursuivions.  00:01:14-1 Person 1: Je marque mon accord pour participer à cette étude. Je suis médecin gynécologue. Ça fait 4 ans que je suis ici.  00:01:27-5 Interviewer: Quel est votre rôle au sein du CMA ici ?  00:01:31-0 Person 1: Au niveau du poste que j'occupe, il n'y a pas un rôle spécifique parce qu'il y a un responsable de service et une surveillance d'unité de soins. Je suis le deuxième gynécologue. En tant que gynécologue, je participe aux soins, à la consultation et à la prise en charge des urgences. |
| --- |

| **[00:00:00-0] Interviewer**: Today's date is August 17, 2024. We are here with the gynecologist from CMA de Dô. I'm about to read the informed consent. Thank you in advance for your time and willingness to participate in this interview. We're a research team from Ghent University, IRSS, and AFRICSanté, focusing on qualitative research about premature birth in urban areas of Burkina Faso. Our objective is to gain a deeper understanding of this issue to improve healthcare services for mothers and premature infants. Your insights are crucial to comprehend the challenges faced at your health center and identify potential improvements. This interview will remain confidential, and the information collected is solely for research purposes. Feel free to not answer any questions that make you uncomfortable, and you can terminate the interview whenever you wish. May I have your consent to read this informed consent before we proceed?  **[00:01:14-1] Person 1**: I consent to participate in this study. I am a gynecologist and have been working here for 4 years.  **[00:01:27-5] Interviewer**: Could you describe your role within the CMA?  **[00:01:31-0] Person 1**: My role isn't strictly defined as there's a department manager and care unit supervision already. As the second gynecologist here, my duties involve participating in patient care, consultations, and managing emergencies. |
| --- |

### Chief physician of Do health district

| [00:00:01-4](http://localhost:2300/file=C:/Users/USER/Desktop/ENTRETIENS%20PREMATURITE/CMA-DO/MCD-DO.mp3time=1400) Interviewer: Aujourd'hui, nous sommes le 24 08 2024, entretien avec le médecin-chef du district sanitaire de Do.Consentement éclairé: Bonjour, merci beaucoup d'avance pour votre disponibilité et le temps que vous nous accordez. Nous sommes une équipe de recherche de l’Université de Gand, de l'IRSS et de AFRICSanté. Nous menons une recherche qualitative sur la naissance prématurée dans les milieux urbains du Burkina Faso. Notre objectif est de mieux comprendre ce phénomène pour améliorer les services et les soins de santé destinés aux mères et aux nourrissons prématurés. Votre participation est essentielle pour nous aider à comprendre les défis spécifiques auxquels vous êtes confronté dans votre centre de santé et pour identifier les opportunités d’amélioration.  L’entretien d’aujourd’hui sera confidentiel et les informations recueillies seront utilisées uniquement à des fins de recherche. Vous avez la liberté de ne pas répondre à toute question qui vous met mal à l'aise et vous pouvez arrêter l’entretien à tout moment. Avez-vous des questions avant que nous commencions ? [00:00:56-4](http://localhost:2300/file=C:/Users/USER/Desktop/ENTRETIENS%20PREMATURITE/CMA-DO/MCD-DO.mp3time=56400)  [00:00:56-4](http://localhost:2300/file=C:/Users/USER/Desktop/ENTRETIENS%20PREMATURITE/CMA-DO/MCD-DO.mp3time=56400) Person 1: Je vais commencer parce que c'est mieux qu'on évacue, comme je l'ai dit à 10H30MN. [00:00:58-7](http://localhost:2300/file=C:/Users/USER/Desktop/ENTRETIENS%20PREMATURITE/CMA-DO/MCD-DO.mp3time=58700)  [00:00:58-7](http://localhost:2300/file=C:/Users/USER/Desktop/ENTRETIENS%20PREMATURITE/CMA-DO/MCD-DO.mp3time=58700) Interviewer: D'accord. Alors pour la première question, je vais déjà vous demander de vous présenter sans le nom, votre titre , votre fonction, votre rôle et la durées que vous occupez ce poste au sein du district sanitaire du Dô. [00:01:10-9](http://localhost:2300/file=C:/Users/USER/Desktop/ENTRETIENS%20PREMATURITE/CMA-DO/MCD-DO.mp3time=70900)  [00:01:10-9](http://localhost:2300/file=C:/Users/USER/Desktop/ENTRETIENS%20PREMATURITE/CMA-DO/MCD-DO.mp3time=70900) Person 1: Donc sans le nom et le prénom? #h12-0#  [00:01:12-0](http://localhost:2300/file=C:/Users/USER/Desktop/ENTRETIENS%20PREMATURITE/CMA-DO/MCD-DO.mp3time=72000) Interviewer: Oui. [00:01:13-2](http://localhost:2300/file=C:/Users/USER/Desktop/ENTRETIENS%20PREMATURITE/CMA-DO/MCD-DO.mp3time=73200)  [00:01:13-2](http://localhost:2300/file=C:/Users/USER/Desktop/ENTRETIENS%20PREMATURITE/CMA-DO/MCD-DO.mp3time=73200) Person 1: Je suis le medecin chef du district sanitaire du Dô. J'ai pris le service le 29 mai 2021, donc ça fait 2 ans 2 mois que je suis au district sanitaire du Dô. [00:01:34-7](http://localhost:2300/file=C:/Users/USER/Desktop/ENTRETIENS%20PREMATURITE/CMA-DO/MCD-DO.mp3time=94700)  [00:01:34-7](http://localhost:2300/file=C:/Users/USER/Desktop/ENTRETIENS%20PREMATURITE/CMA-DO/MCD-DO.mp3time=94700) Interviewer: Quel est votre rôle au sein du district sanitaire du Dô? [00:01:38-3](http://localhost:2300/file=C:/Users/USER/Desktop/ENTRETIENS%20PREMATURITE/CMA-DO/MCD-DO.mp3time=98300)  [00:01:38-3](http://localhost:2300/file=C:/Users/USER/Desktop/ENTRETIENS%20PREMATURITE/CMA-DO/MCD-DO.mp3time=98300) Person 1: Le medecin chef par déclinaison est censé piloter l'équipe cadre du district, qui est l'instance dirigeante du district sanitaire au niveau du Burkina Faso. En se sens il s'assure que toutes les activités prévues au niveau du district se déroulent à merveille et que la population de responsabilité ai accès aux soins pour améliorer l'état de santé de sa population. [00:02:11-1](http://localhost:2300/file=C:/Users/USER/Desktop/ENTRETIENS%20PREMATURITE/CMA-DO/MCD-DO.mp3time=131100) |
| --- |

| 00:00:01-4 Interviewer: Aujourd'hui, nous sommes le 24/08/2024, entretien avec le médecin-chef du district sanitaire de Do. Consentement éclairé : Bonjour, merci beaucoup d'avance pour votre disponibilité et le temps que vous nous accordez. Nous sommes une équipe de recherche de l’Université de Gand, de l'IRSS et d'AFRICSanté. Nous menons une recherche qualitative sur la naissance prématurée dans les milieux urbains du Burkina Faso. Notre objectif est de mieux comprendre ce phénomène pour améliorer les services et les soins de santé destinés aux mères et aux nourrissons prématurés. Votre participation est essentielle pour nous aider à comprendre les défis spécifiques auxquels vous êtes confronté dans votre centre de santé et pour identifier les opportunités d’amélioration. L’entretien d’aujourd’hui sera confidentiel et les informations recueillies seront utilisées uniquement à des fins de recherche. Vous avez la liberté de ne pas répondre à toute question qui vous met mal à l'aise et vous pouvez arrêter l’entretien à tout moment. Avez-vous des questions avant que nous commencions ?  00:00:56-4 Person 1: Je vais commencer parce que c'est mieux qu'on évacue, comme je l'ai dit à 10H30MN.  00:00:58-7 Interviewer: D'accord. Alors pour la première question, je vais déjà vous demander de vous présenter sans le nom, votre titre, votre fonction, votre rôle et la durée que vous occupez ce poste au sein du district sanitaire du Dô.  00:01:10-9 Person 1: Donc sans le nom et le prénom ?  00:01:12-0 Interviewer: Oui.  00:01:13-2 Person 1: Je suis le médecin chef du district sanitaire du Dô. J'ai pris le service le 29 mai 2021, donc ça fait 2 ans 2 mois que je suis au district sanitaire du Dô.  00:01:34-7 Interviewer: Quel est votre rôle au sein du district sanitaire du Dô ?  00:01:38-3 Person 1: Le médecin chef par déclinaison est censé piloter l'équipe cadre du district, qui est l'instance dirigeante du district sanitaire au niveau du Burkina Faso. En ce sens, il s'assure que toutes les activités prévues au niveau du district se déroulent à merveille et que la population de responsabilité ait accès aux soins pour améliorer l'état de santé de sa population. |
| --- |

| **[00:00:01-4] Interviewer**: Today is August 24, 2024. We're conducting an interview with the chief physician of the Dô Health District. Before we begin, I'd like to extend our gratitude for your time and participation. We are a research team from Ghent University, IRSS, and AFRICSanté, undertaking qualitative research on premature birth in urban areas of Burkina Faso. Our aim is to better understand this phenomenon to enhance healthcare services for mothers and premature infants. Your insights are vital in identifying challenges and opportunities for improvement at your health center. Please be informed that this interview is confidential, and all collected information will be exclusively used for research. You have the liberty to refrain from answering any uncomfortable questions and may terminate the interview at any time. Do you have any questions before we start?  **[00:00:56-4] Person 1**: Let's start, as I need to leave by 10:30 AM.  **[00:00:58-7] Interviewer**: Okay, then. For our first question, please introduce yourself, mentioning your title, role, and how long you've been in this position at the Dô Health District, but omit your name.  **[00:01:10-9] Person 1**: Without my first and last name, right?  **[00:01:12-0] Interviewer**: Yes, that's correct.  **[00:01:13-2] Person 1**: I am the chief doctor of the Dô Health District. I started my service here on May 29, 2021, so it's been 2 years and 2 months.  **[00:01:34-7] Interviewer**: Can you elaborate on your role within the Dô Health District?  **[00:01:38-3] Person 1**: As the head doctor, my primary responsibility is to lead the district management team, which governs the health district in Burkina Faso. I ensure that all planned activities at the district level are executed effectively, and I work to guarantee that our responsible population has access to healthcare to improve their overall health status. |
| --- |

### Manager for maternity care unit of Do medical center with surgical branch

| [00:00:02-2](http://localhost:2300/file=C:/Users/USER/Desktop/ENTRETIENS%20PREMATURITE/CMA-DO/RESPONSABLE%20UNITE%20DE%20SOIN%20CMA-DO.mp3time=2200) Interviewer: Entretien avec la responsable de l'unité des soins de la maternité, donc du CMA de DÔ. Aujourd'hui le 17/08/2024. OK, donc je vais vous lire le consentement éclairé.Bonjour, merci beaucoup d'avance pour votre disponibilité et le temps que vous nous accordez. Nous sommes une équipe de recherche de l’Université de Gand, de l'IRSS et de AFRICSanté. Nous menons une recherche qualitative sur la naissance prématurée dans les milieux urbains du Burkina Faso. Notre objectif est de mieux comprendre ce phénomène pour améliorer les services et les soins de santé destinés aux mères et aux nourrissons prématurés. Votre participation est essentielle pour nous aider à comprendre les défis spécifiques auxquels vous êtes confronté dans votre centre de santé et pour identifier les opportunités d’amélioration.  L’entretien d’aujourd’hui sera confidentiel et les informations recueillies seront utilisées uniquement à des fins de recherche. Vous avez la liberté de ne pas répondre à toute question qui vous met mal à l'aise et vous pouvez arrêter l’entretien à tout moment. Avez-vous des questions avant que nous commencions ?  Avec votre permission, pouvons-nous commencer l’entretien ? [00:01:09-7](http://localhost:2300/file=C:/Users/USER/Desktop/ENTRETIENS%20PREMATURITE/CMA-DO/RESPONSABLE%20UNITE%20DE%20SOIN%20CMA-DO.mp3time=69700)  [00:01:09-7](http://localhost:2300/file=C:/Users/USER/Desktop/ENTRETIENS%20PREMATURITE/CMA-DO/RESPONSABLE%20UNITE%20DE%20SOIN%20CMA-DO.mp3time=69700) Person 1: Oui. [00:01:09-0](http://localhost:2300/file=C:/Users/USER/Desktop/ENTRETIENS%20PREMATURITE/CMA-DO/RESPONSABLE%20UNITE%20DE%20SOIN%20CMA-DO.mp3time=69000)  [00:01:09-0](http://localhost:2300/file=C:/Users/USER/Desktop/ENTRETIENS%20PREMATURITE/CMA-DO/RESPONSABLE%20UNITE%20DE%20SOIN%20CMA-DO.mp3time=69000) Interviewer: D'accord. Donc je vais vous demander de vous présenter sans le nom c'est seulement le titre, la fonction, votre rôle et depuis combien de temps vous occupez ce poste au sein de la maternité. Est-ce que vous êtes même d'accord déjà pour participer à cet entretien ? [00:01:32-2](http://localhost:2300/file=C:/Users/USER/Desktop/ENTRETIENS%20PREMATURITE/CMA-DO/RESPONSABLE%20UNITE%20DE%20SOIN%20CMA-DO.mp3time=92200)  [00:01:32-2](http://localhost:2300/file=C:/Users/USER/Desktop/ENTRETIENS%20PREMATURITE/CMA-DO/RESPONSABLE%20UNITE%20DE%20SOIN%20CMA-DO.mp3time=92200) Person 1: Oui, oui. Pas de problème. [00:01:36-1](http://localhost:2300/file=C:/Users/USER/Desktop/ENTRETIENS%20PREMATURITE/CMA-DO/RESPONSABLE%20UNITE%20DE%20SOIN%20CMA-DO.mp3time=96100)  [00:01:36-1](http://localhost:2300/file=C:/Users/USER/Desktop/ENTRETIENS%20PREMATURITE/CMA-DO/RESPONSABLE%20UNITE%20DE%20SOIN%20CMA-DO.mp3time=96100) Interviewer: Est-ce que vous pouvez vous présenter ? [00:01:38-4](http://localhost:2300/file=C:/Users/USER/Desktop/ENTRETIENS%20PREMATURITE/CMA-DO/RESPONSABLE%20UNITE%20DE%20SOIN%20CMA-DO.mp3time=98400)  [00:01:38-4](http://localhost:2300/file=C:/Users/USER/Desktop/ENTRETIENS%20PREMATURITE/CMA-DO/RESPONSABLE%20UNITE%20DE%20SOIN%20CMA-DO.mp3time=98400) Person 1: D'accord. Je suis attachée de senté en soins obstétricaux et gynécologiques,donc également la surveillance d'unite de soin de la maternité du CMA de DÔ. [00:01:49-0](http://localhost:2300/file=C:/Users/USER/Desktop/ENTRETIENS%20PREMATURITE/CMA-DO/RESPONSABLE%20UNITE%20DE%20SOIN%20CMA-DO.mp3time=109000)  [00:01:49-0](http://localhost:2300/file=C:/Users/USER/Desktop/ENTRETIENS%20PREMATURITE/CMA-DO/RESPONSABLE%20UNITE%20DE%20SOIN%20CMA-DO.mp3time=109000) Interviewer: Depuis combien d'années est-ce que vous occupez cette fonction au sein de la formation sanitaire? [00:01:55-5](http://localhost:2300/file=C:/Users/USER/Desktop/ENTRETIENS%20PREMATURITE/CMA-DO/RESPONSABLE%20UNITE%20DE%20SOIN%20CMA-DO.mp3time=115500)  [00:01:55-5](http://localhost:2300/file=C:/Users/USER/Desktop/ENTRETIENS%20PREMATURITE/CMA-DO/RESPONSABLE%20UNITE%20DE%20SOIN%20CMA-DO.mp3time=115500) Person 1: Eh.. 2019 à maintenant. Ça fait combien? [00:01:56-4](http://localhost:2300/file=C:/Users/USER/Desktop/ENTRETIENS%20PREMATURITE/CMA-DO/RESPONSABLE%20UNITE%20DE%20SOIN%20CMA-DO.mp3time=116400)  [00:01:56-4](http://localhost:2300/file=C:/Users/USER/Desktop/ENTRETIENS%20PREMATURITE/CMA-DO/RESPONSABLE%20UNITE%20DE%20SOIN%20CMA-DO.mp3time=116400) Interviewer: 4 ans. Vous êtes là depuis 2019? [00:02:00-6](http://localhost:2300/file=C:/Users/USER/Desktop/ENTRETIENS%20PREMATURITE/CMA-DO/RESPONSABLE%20UNITE%20DE%20SOIN%20CMA-DO.mp3time=120600)  [00:02:00-6](http://localhost:2300/file=C:/Users/USER/Desktop/ENTRETIENS%20PREMATURITE/CMA-DO/RESPONSABLE%20UNITE%20DE%20SOIN%20CMA-DO.mp3time=120600) Person 1: Non, je suis là avant, donc depuis 2015. Mais le poste de surveillant de l'Unité de soin, c'est depuis 2019. Ah, depuis 2019. [00:02:12-2](http://localhost:2300/file=C:/Users/USER/Desktop/ENTRETIENS%20PREMATURITE/CMA-DO/RESPONSABLE%20UNITE%20DE%20SOIN%20CMA-DO.mp3time=132200) |
| --- |

| 00:00:02-2 Interviewer: Entretien avec la responsable de l'unité des soins de la maternité, donc du CMA de Dô. Aujourd'hui le 17/08/2024. OK, donc je vais vous lire le consentement éclairé. Bonjour, merci beaucoup d'avance pour votre disponibilité et le temps que vous nous accordez. Nous sommes une équipe de recherche de l’Université de Gand, de l'IRSS et d'AFRICSanté. Nous menons une recherche qualitative sur la naissance prématurée dans les milieux urbains du Burkina Faso. Notre objectif est de mieux comprendre ce phénomène pour améliorer les services et les soins de santé destinés aux mères et aux nourrissons prématurés. Votre participation est essentielle pour nous aider à comprendre les défis spécifiques auxquels vous êtes confronté dans votre centre de santé et pour identifier les opportunités d’amélioration. L’entretien d’aujourd’hui sera confidentiel et les informations recueillies seront utilisées uniquement à des fins de recherche. Vous avez la liberté de ne pas répondre à toute question qui vous met mal à l'aise et vous pouvez arrêter l’entretien à tout moment. Avez-vous des questions avant que nous commencions ? Avec votre permission, pouvons-nous commencer l’entretien ?  00:01:09-7 Person 1: Oui.  00:01:09-0 Interviewer: D'accord. Donc je vais vous demander de vous présenter sans le nom, c'est seulement le titre, la fonction, votre rôle et depuis combien de temps vous occupez ce poste au sein de la maternité. Est-ce que vous êtes même d'accord déjà pour participer à cet entretien ?  00:01:32-2 Person 1: Oui, oui. Pas de problème.  00:01:36-1 Interviewer: Est-ce que vous pouvez vous présenter ?  00:01:38-4 Person 1: D'accord. Je suis attachée de santé en soins obstétricaux et gynécologiques, donc également la surveillance d'unité de soin de la maternité du CMA de Dô.  00:01:49-0 Interviewer: Depuis combien d'années est-ce que vous occupez cette fonction au sein de la formation sanitaire ?  00:01:55-5 Person 1: Eh... 2019 à maintenant. Ça fait combien ?  00:01:56-4 Interviewer: 4 ans. Vous êtes là depuis 2019 ?  00:02:00-6 Person 1: Non, je suis là avant, donc depuis 2015. Mais le poste de surveillant de l'Unité de soin, c'est depuis 2019. Ah, depuis 2019. |
| --- |

| **[00:00:02-2] Interviewer**: Today's date is August 17, 2024, and we're here with the head of the maternity care unit at CMA de Dô. Before we begin, I want to thank you for your time and participation. Our team from Ghent University, IRSS, and AFRICSanté is conducting qualitative research on premature births in urban areas of Burkina Faso. Our aim is to enhance our understanding of this issue to improve healthcare services for mothers and premature infants. Your insights will help us identify challenges and improvement opportunities at your health center. This interview is confidential, and all information will be used solely for research purposes. You're free to not answer any uncomfortable questions and can stop the interview at any time. Do you have any questions before we start? May we begin the interview with your permission?  **[00:01:09-7] Person 1**: Yes.  **[00:01:09-0] Interviewer**: Great. Could you introduce yourself, omitting your name but including your title, role, and how long you've been in this position within the maternity ward? Are you willing to participate in this interview?  **[00:01:32-2] Person 1**: Yes, no problem.  **[00:01:36-1] Interviewer**: Please go ahead and introduce yourself.  **[00:01:38-4] Person 1**: Okay. I am the health officer in obstetric and gynecological care, and I also oversee the maternity care unit of the CMA de Dô.  **[00:01:49-0] Interviewer**: How long have you been in this position at the health facility?  **[00:01:55-5] Person 1**: Eh... since 2019. So, how many years is that?  **[00:01:56-4] Interviewer**: That's 4 years. Have you been at this facility since 2019?  **[00:02:00-6] Person 1**: No, I've been here since 2015. But I've been in the position of care unit supervisor since 2019. Ah, yes, since 2019. |
| --- |

### Focus group of Colma1 health and social promotion center

| [00:00:00-0](http://localhost:2300/file=C:/Users/USER/Desktop/ENTRETIENS%20PREMATURITE/CSPS-COLMA1/FOCUS%20GROUPE%20COLMA1.mp3time=0)Interviewer: Aujourd'hui, nous sommes le 16/08/ 2024, discussion de groupe avec les sages-femmes et les accoucheuses du CSPS de Colma1, donc je vais vous lire le consentement éclairé.Bonjour, merci beaucoup d'avance pour votre disponibilité et le temps que vous nous accordez. Nous sommes une équipe de recherche de l'Université de Gand, de l'IRSS et de AFRICSanté. Nous menons une recherche qualitative sur la naissance prématurée dans les milieux urbains du Burkina Faso. Notre objectif est de mieux comprendre ce phénomène pour améliorer les services et les soins de santé destinés aux mères et aux nourrissons prématurés. Votre participation est essentielle pour nous aider à comprendre les défis spécifiques auxquels vous êtes confronté dans votre centre de santé et pour identifier les opportunités d'amélioration. L'entretien d'aujourd'hui sera confidentiel et les informations recueillies seront utilisées uniquement à des fins de recherche. Vous avez la liberté de ne pas répondre à toute question qui vous met mal à l'aise et vous pouvez arrêter l'entretien à tout moment. Avez-vous des questions avant que nous commencions ? d'accord avec votre permission, pouvons nous commencer l'entretien? [00:01:01-6](http://localhost:2300/file=C:/Users/USER/Desktop/ENTRETIENS%20PREMATURITE/CSPS-COLMA1/FOCUS%20GROUPE%20COLMA1.mp3time=61600)  [00:01:01-6](http://localhost:2300/file=C:/Users/USER/Desktop/ENTRETIENS%20PREMATURITE/CSPS-COLMA1/FOCUS%20GROUPE%20COLMA1.mp3time=61600) Person 1: oui oui [00:01:01-4](http://localhost:2300/file=C:/Users/USER/Desktop/ENTRETIENS%20PREMATURITE/CSPS-COLMA1/FOCUS%20GROUPE%20COLMA1.mp3time=61400) |
| --- |

| 00:00:00-0 Interviewer: Aujourd'hui, nous sommes le 16/08/2024, discussion de groupe avec les sage-femmes et les accoucheuses du CSPS de Colma1, donc je vais vous lire le consentement éclairé. Bonjour, merci beaucoup d'avance pour votre disponibilité et le temps que vous nous accordez. Nous sommes une équipe de recherche de l'Université de Gand, de l'IRSS et d'AFRICSanté. Nous menons une recherche qualitative sur la naissance prématurée dans les milieux urbains du Burkina Faso. Notre objectif est de mieux comprendre ce phénomène pour améliorer les services et les soins de santé destinés aux mères et aux nourrissons prématurés. Votre participation est essentielle pour nous aider à comprendre les défis spécifiques auxquels vous êtes confrontés dans votre centre de santé et pour identifier les opportunités d'amélioration. L'entretien d'aujourd'hui sera confidentiel et les informations recueillies seront utilisées uniquement à des fins de recherche. Vous avez la liberté de ne pas répondre à toute question qui vous met mal à l'aise et vous pouvez arrêter l'entretien à tout moment. Avez-vous des questions avant que nous commencions ? D'accord, avec votre permission, pouvons-nous commencer l'entretien ?  00:01:01-6 Person 1: Oui, oui. |
| --- |

| **[00:00:00-0] Interviewer**: Today's date is August 16, 2024. We're here for a group discussion with the midwives from CSPS of Colma1. First, I'll read the informed consent. Thank you for your time and willingness to participate in this session. We are a research team from Ghent University, IRSS, and AFRICSanté, focusing on qualitative research regarding premature birth in urban areas of Burkina Faso. Our aim is to deepen our understanding of this issue to enhance healthcare services for mothers and premature infants. Your insights are invaluable in identifying the specific challenges at your health center and exploring opportunities for improvement. Be assured that today's interview is confidential, and the information collected will be used solely for research. You're free to refrain from answering any questions that may cause discomfort, and you can terminate the interview at any time. Do you have any questions before we start? May we proceed with your permission?  **[00:01:01-6] Person 1**: Yes, yes. |
| --- |

### Major of Colma1 health and social promotion center

| [00:00:00-8](http://localhost:2300/file=C:/Users/USER/Desktop/ENTRETIENS%20PREMATURITE/CSPS-COLMA1/MAJOR%20CSPS%20COLMA1.MP3time=800) Interviewer: Aujourd'hui c'est le CSPS de COLMA1 le 10/08/2024 je suis avec le major de la formation sanitaire.le consentement éclairé:Bonjour, merci beaucoup d'avance pour votre disponibilité et le temps que vous nous accordez. Nous sommes une équipe de recherche de l’Université de Gand, de l'IRSS et de AFRICSanté. Nous menons une recherche qualitative sur la naissance prématurée dans les milieux urbains du Burkina Faso. Notre objectif est de mieux comprendre ce phénomène pour améliorer les services et les soins de santé destinés aux mères et aux nourrissons prématurés. Votre participation est essentielle pour nous aider à comprendre les défis spécifiques auxquels vous êtes confronté dans votre centre de santé et pour identifier les opportunités d’amélioration.  L’entretien d’aujourd’hui sera confidentiel et les informations recueillies seront utilisées uniquement à des fins de recherche. Vous avez la liberté de ne pas répondre à toute question qui vous met mal à l'aise et vous pouvez arrêter l’entretien à tout moment. Avez-vous des questions avant que nous commencions ? [00:01:05-7](http://localhost:2300/file=C:/Users/USER/Desktop/ENTRETIENS%20PREMATURITE/CSPS-COLMA1/MAJOR%20CSPS%20COLMA1.MP3time=65700)  [00:01:05-7](http://localhost:2300/file=C:/Users/USER/Desktop/ENTRETIENS%20PREMATURITE/CSPS-COLMA1/MAJOR%20CSPS%20COLMA1.MP3time=65700) Person 1: NON , il n y a pas de questions.[00:01:06-7](http://localhost:2300/file=C:/Users/USER/Desktop/ENTRETIENS%20PREMATURITE/CSPS-COLMA1/MAJOR%20CSPS%20COLMA1.MP3time=66700)  [00:01:06-7](http://localhost:2300/file=C:/Users/USER/Desktop/ENTRETIENS%20PREMATURITE/CSPS-COLMA1/MAJOR%20CSPS%20COLMA1.MP3time=66700) D'accord.Avec votre permission, pouvons-nous commencer l’entretien ? [00:01:09-4](http://localhost:2300/file=C:/Users/USER/Desktop/ENTRETIENS%20PREMATURITE/CSPS-COLMA1/MAJOR%20CSPS%20COLMA1.MP3time=69400)  [00:01:09-4](http://localhost:2300/file=C:/Users/USER/Desktop/ENTRETIENS%20PREMATURITE/CSPS-COLMA1/MAJOR%20CSPS%20COLMA1.MP3time=69400) Person 1: oui oui vous pouvez commencer [00:01:11-1](http://localhost:2300/file=C:/Users/USER/Desktop/ENTRETIENS%20PREMATURITE/CSPS-COLMA1/MAJOR%20CSPS%20COLMA1.MP3time=71100)  [00:01:11-1](http://localhost:2300/file=C:/Users/USER/Desktop/ENTRETIENS%20PREMATURITE/CSPS-COLMA1/MAJOR%20CSPS%20COLMA1.MP3time=71100) Interviewer: Ok, maintenant je vais vous demander de vous présenter pas de nom, votre titre, votre fonction, votre rôle et depuis combien de temps vous occupiez ce poste.? [00:01:20-9](http://localhost:2300/file=C:/Users/USER/Desktop/ENTRETIENS%20PREMATURITE/CSPS-COLMA1/MAJOR%20CSPS%20COLMA1.MP3time=80900)  [00:01:20-9](http://localhost:2300/file=C:/Users/USER/Desktop/ENTRETIENS%20PREMATURITE/CSPS-COLMA1/MAJOR%20CSPS%20COLMA1.MP3time=80900) Person 1: je suis infirmier chef de poste, je suis infirmier diplômé d'État,et je suis à Colma1, ça fait déjà 3 ans.Voilà comme responsable. [00:01:31-5](http://localhost:2300/file=C:/Users/USER/Desktop/ENTRETIENS%20PREMATURITE/CSPS-COLMA1/MAJOR%20CSPS%20COLMA1.MP3time=91500)  [00:01:31-5](http://localhost:2300/file=C:/Users/USER/Desktop/ENTRETIENS%20PREMATURITE/CSPS-COLMA1/MAJOR%20CSPS%20COLMA1.MP3time=91500) Interviewer: et quel est votre rôle au sein de cette formation sanitaire? [00:01:35-9](http://localhost:2300/file=C:/Users/USER/Desktop/ENTRETIENS%20PREMATURITE/CSPS-COLMA1/MAJOR%20CSPS%20COLMA1.MP3time=95900)  [00:01:35-9](http://localhost:2300/file=C:/Users/USER/Desktop/ENTRETIENS%20PREMATURITE/CSPS-COLMA1/MAJOR%20CSPS%20COLMA1.MP3time=95900) Person 1: je disais tantot que je suis infirmier chef de poste, c'est le rôle principalement, c'est la coordination des activités, la supervision et la programmation, donc l'intégration des différentes activités, donc du CSPS afin de que tout le monde puisse converger donc vers l'objectif commun qui est d'offrir des prestations aux populations. [00:01:51-2](http://localhost:2300/file=C:/Users/USER/Desktop/ENTRETIENS%20PREMATURITE/CSPS-COLMA1/MAJOR%20CSPS%20COLMA1.MP3time=111200) |
| --- |

| 00:00:00-8 Interviewer: Aujourd'hui c'est le CSPS de Colma1, le 10/08/2024. Je suis avec le major de la formation sanitaire. Le consentement éclairé : Bonjour, merci beaucoup d'avance pour votre disponibilité et le temps que vous nous accordez. Nous sommes une équipe de recherche de l’Université de Gand, de l'IRSS et d'AFRICSanté. Nous menons une recherche qualitative sur la naissance prématurée dans les milieux urbains du Burkina Faso. Notre objectif est de mieux comprendre ce phénomène pour améliorer les services et les soins de santé destinés aux mères et aux nourrissons prématurés. Votre participation est essentielle pour nous aider à comprendre les défis spécifiques auxquels vous êtes confrontés dans votre centre de santé et pour identifier les opportunités d’amélioration. L’entretien d’aujourd’hui sera confidentiel et les informations recueillies seront utilisées uniquement à des fins de recherche. Vous avez la liberté de ne pas répondre à toute question qui vous met mal à l'aise et vous pouvez arrêter l’entretien à tout moment. Avez-vous des questions avant que nous commencions ?  00:01:05-7 Person 1: Non, il n'y a pas de questions.  00:01:06-7 Interviewer: D'accord. Avec votre permission, pouvons-nous commencer l’entretien ?  00:01:09-4 Person 1: Oui, oui, vous pouvez commencer.  00:01:20-9 Person 1: Je suis infirmier chef de poste, je suis infirmier diplômé d'État, et je suis à Colma1, ça fait déjà 3 ans. Voilà comme responsable. 00:01:31-5  00:01:31-5 Interviewer: et quel est votre rôle au sein de cette formation sanitaire ? 00:01:35-9  00:01:35-9 Person 1: Je disais tantôt que je suis infirmier chef de poste, c'est le rôle principalement, c'est la coordination des activités, la supervision et la programmation, donc l'intégration des différentes activités, donc du CSPS afin que tout le monde puisse converger donc vers l'objectif commun qui est d'offrir des prestations aux populations. 00:01:51-2 |
| --- |

| **[00:00:00-8] Interviewer**: Today's date is August 10, 2024, and we're at the CSPS of Colma1, meeting with the health training major. I'd like to start by reading the informed consent. Thank you for your time and willingness to participate in this interview. Our team from Ghent University, IRSS, and AFRICSanté is conducting qualitative research on premature birth in urban areas of Burkina Faso. The purpose of this study is to gain a better understanding of this phenomenon to improve healthcare services for mothers and premature infants. Your participation is crucial for us to identify the specific challenges at your health center and discover potential areas for improvement. Be assured that this interview is confidential, and all information gathered will be used exclusively for research purposes. You have the right not to answer any questions that make you uncomfortable, and you may end the interview at any point. Do you have any questions before we begin?  **[00:01:05-7] Person 1**: No, I don't have any questions.  **[00:01:06-7] Interviewer**: Great. With your permission, shall we start the interview?  **[00:01:09-4] Person 1**: Yes, please go ahead.  **[00:01:11-1] Interviewer**: Okay, now I'd like you to introduce yourself, including your name, title, position, role, and the duration you've held this position.  **[00:01:20-9] Person 1**: I am the head nurse, a state-certified nurse. I've been working in Colma1 for 3 years, fulfilling responsibilities as a nurse manager.  **[00:01:31-5] Interviewer**: And what is your specific role within this health facility?  **[00:01:35-9] Person 1**: As I mentioned earlier, my primary role as a nurse manager involves coordinating activities, supervising, and planning. I integrate various activities within the CSPS to ensure that everyone works towards our common goal, which is to provide services to the population. |
| --- |

### Maternity manager of Colma1 health and social promotion center

| [00:00:01-6](http://localhost:2300/file=C:/Users/USER/Desktop/ENTRETIENS%20PREMATURITE/CSPS-COLMA1/RESPONSABLE%20MATERNITE-COLMA1.mp3time=1600) Interviewer: aujourd'hui c'est 16/08/2024, entretien avec la responsable de la maternité du CSPS de COLMA 1.ok le consentement éclairé.Bonjour, merci beaucoup d'avance pour votre disponibilité et le temps que vous nous accordez. Nous sommes une équipe de recherche de l’Université de Gand, de l'IRSS et de AFRICSanté. Nous menons une recherche qualitative sur la naissance prématurée dans les milieux urbains du Burkina Faso. Notre objectif est de mieux comprendre ce phénomène pour améliorer les services et les soins de santé destinés aux mères et aux nourrissons prématurés. Votre participation est essentielle pour nous aider à comprendre les défis spécifiques auxquels vous êtes confronté dans votre centre de santé et pour identifier les opportunités d’amélioration.  L’entretien d’aujourd’hui sera confidentiel et les informations recueillies seront utilisées uniquement à des fins de recherche. Vous avez la liberté de ne pas répondre à toute question qui vous met mal à l'aise et vous pouvez arrêter l’entretien à tout moment. Avez-vous des questions avant que nous commencions ? D'accord.Avec votre permission, pouvons-nous commencer l’entretien ? [00:01:01-5](http://localhost:2300/file=C:/Users/USER/Desktop/ENTRETIENS%20PREMATURITE/CSPS-COLMA1/RESPONSABLE%20MATERNITE-COLMA1.mp3time=61500)  [00:01:01-5](http://localhost:2300/file=C:/Users/USER/Desktop/ENTRETIENS%20PREMATURITE/CSPS-COLMA1/RESPONSABLE%20MATERNITE-COLMA1.mp3time=61500) Person 1: Notre CSPS. La dernière fois, Madame Sawadogo nous a informés et la dernière fois aussi, vous étiez là par rapport à cet entretien. Je pense que les rendez- vous, c'était aujourd'hui à 11h30. On a essayé d'honnorer ce qu'on a proposé. Je pense qu'il n'y a pas de problème par rapport à l'interview. Si on nous pose des questions, c'est pour nous aider vraiment à rafraîchir la mémoire et tout ça, c'est au compte des femmes et nous- mêmes en tant que professionnelles. Lorsque tu fais un travail qui s'achève très bien, bien vrai que c'est la famille qui bénéficie, mais toi d'abord, en tant que praticien, tu as un renommé à quelque part. Donc, ce n'est pas un problème. On veut toujours apprendre. [00:01:58-3](http://localhost:2300/file=C:/Users/USER/Desktop/ENTRETIENS%20PREMATURITE/CSPS-COLMA1/RESPONSABLE%20MATERNITE-COLMA1.mp3time=118300)  [00:01:58-3](http://localhost:2300/file=C:/Users/USER/Desktop/ENTRETIENS%20PREMATURITE/CSPS-COLMA1/RESPONSABLE%20MATERNITE-COLMA1.mp3time=118300) Interviewer: Merci pour votre disponibilité, c'est touchant. J'espère que nous ne serons pas ennuyant aussi? [00:02:02-9](http://localhost:2300/file=C:/Users/USER/Desktop/ENTRETIENS%20PREMATURITE/CSPS-COLMA1/RESPONSABLE%20MATERNITE-COLMA1.mp3time=122900)  [00:02:02-9](http://localhost:2300/file=C:/Users/USER/Desktop/ENTRETIENS%20PREMATURITE/CSPS-COLMA1/RESPONSABLE%20MATERNITE-COLMA1.mp3time=122900) Person 1: Non [00:02:03-2](http://localhost:2300/file=C:/Users/USER/Desktop/ENTRETIENS%20PREMATURITE/CSPS-COLMA1/RESPONSABLE%20MATERNITE-COLMA1.mp3time=123200)  [00:02:03-2](http://localhost:2300/file=C:/Users/USER/Desktop/ENTRETIENS%20PREMATURITE/CSPS-COLMA1/RESPONSABLE%20MATERNITE-COLMA1.mp3time=123200) Interviewer: . Maintenant, pour commencer, est-ce que vous pouvez vous présenter sans le nom ? La fonction, le titre, le rôle et le nombre d'années que vous occupiez ce poste? [00:02:12-0](http://localhost:2300/file=C:/Users/USER/Desktop/ENTRETIENS%20PREMATURITE/CSPS-COLMA1/RESPONSABLE%20MATERNITE-COLMA1.mp3time=132000)  [00:02:12-0](http://localhost:2300/file=C:/Users/USER/Desktop/ENTRETIENS%20PREMATURITE/CSPS-COLMA1/RESPONSABLE%20MATERNITE-COLMA1.mp3time=132000) Person 1: d'accord [00:02:13-2](http://localhost:2300/file=C:/Users/USER/Desktop/ENTRETIENS%20PREMATURITE/CSPS-COLMA1/RESPONSABLE%20MATERNITE-COLMA1.mp3time=133200)  [00:02:13-2](http://localhost:2300/file=C:/Users/USER/Desktop/ENTRETIENS%20PREMATURITE/CSPS-COLMA1/RESPONSABLE%20MATERNITE-COLMA1.mp3time=133200) Person 1: Ok, je suis Sage femme. Voilà au CSPS de colma 1. Responsable maternité, cela dure deux ans, on peut dire. Voilà, je suis dans la fonction publique il y a 16 ans, mais en tant que sage- femme, ça fait cinq ans. [00:02:38-2](http://localhost:2300/file=C:/Users/USER/Desktop/ENTRETIENS%20PREMATURITE/CSPS-COLMA1/RESPONSABLE%20MATERNITE-COLMA1.mp3time=158200) |
| --- |

| 00:00:01-6 Interviewer: Aujourd'hui c'est le 16/08/2024, entretien avec la responsable de la maternité du CSPS de Colma 1. Ok, le consentement éclairé. Bonjour, merci beaucoup d'avance pour votre disponibilité et le temps que vous nous accordez. Nous sommes une équipe de recherche de l’Université de Gand, de l'IRSS et d'AFRICSanté. Nous menons une recherche qualitative sur la naissance prématurée dans les milieux urbains du Burkina Faso. Notre objectif est de mieux comprendre ce phénomène pour améliorer les services et les soins de santé destinés aux mères et aux nourrissons prématurés. Votre participation est essentielle pour nous aider à comprendre les défis spécifiques auxquels vous êtes confrontée dans votre centre de santé et pour identifier les opportunités d’amélioration. L’entretien d’aujourd’hui sera confidentiel et les informations recueillies seront utilisées uniquement à des fins de recherche. Vous avez la liberté de ne pas répondre à toute question qui vous met mal à l'aise et vous pouvez arrêter l’entretien à tout moment. Avez-vous des questions avant que nous commencions ? D'accord. Avec votre permission, pouvons-nous commencer l’entretien ?  00:01:01-5 Person 1: Notre CSPS. La dernière fois, Madame Sawadogo nous a informés et la dernière fois aussi, vous étiez là par rapport à cet entretien. Je pense que les rendez-vous, c'était aujourd'hui à 11h30. On a essayé d'honorer ce qu'on a proposé. Je pense qu'il n'y a pas de problème par rapport à l'interview. Si on nous pose des questions, c'est pour nous aider vraiment à rafraîchir la mémoire et tout ça, c'est au compte des femmes et nous-mêmes en tant que professionnelles. Lorsque tu fais un travail qui s'achève très bien, bien vrai que c'est la famille qui bénéficie, mais toi d'abord, en tant que praticien, tu as un renom à quelque part. Donc, ce n'est pas un problème. On veut toujours apprendre.  00:01:58-3 Interviewer: Merci pour votre disponibilité, c'est touchant. J'espère que nous ne serons pas ennuyants aussi ?  00:02:02-9 Person 1: Non.  00:02:03-2 Interviewer: Maintenant, pour commencer, est-ce que vous pouvez vous présenter sans le nom ? La fonction, le titre, le rôle et le nombre d'années que vous occupiez ce poste ? 00:02:12-0  00:02:12-0 Person 1: D'accord. 00:02:13-2  00:02:13-2 Person 1: Ok, je suis sage-femme. Voilà au CSPS de Colma 1. Responsable maternité, cela dure deux ans, on peut dire. Voilà, je suis dans la fonction publique il y a 16 ans, mais en tant que sage-femme, ça fait cinq ans. 00:02:38-2 |
| --- |

| **[00:00:01-6] Interviewer**: Today is August 16, 2024. We're here for an interview with the head of the maternity ward at CSPS of Colma 1. First, I want to read the informed consent. Thank you for making time for this interview. We're a research team from Ghent University, IRSS, and AFRICSanté, conducting a study on premature birth in urban Burkina Faso. Our aim is to better understand this issue to improve healthcare services for mothers and premature infants. Your insights will help us address specific challenges at your health center and identify areas for improvement. This interview is confidential, and all information gathered is strictly for research. You have the right to refrain from answering any uncomfortable questions and can terminate the interview at any time. Do you have any questions before we start? With your permission, shall we begin?  **[00:01:01-5] Person 1**: Regarding our CSPS, Madam Sawadogo previously informed us about this interview, and you were here last time for the same reason. I believe the appointment was set for today at 11:30. We've made an effort to meet the proposed schedule. I see no issues with proceeding with the interview. If questions are asked, they will aid in refreshing our memories and benefit us professionally, as well as the women we serve. Successful outcomes in our work not only benefit the families but also enhance our reputation as practitioners. So, there's no problem; we are always eager to learn.  **[00:01:58-3] Interviewer**: Thank you for your cooperation; it's greatly appreciated. I hope the interview won't be too tedious?  **[00:02:02-9] Person 1**: No, not at all.  **[00:02:03-2] Interviewer**: For our first question, could you please introduce yourself without mentioning your name? Include your function, title, role, and the number of years you have held this position.  **[00:02:12-0] Person 1**: Okay.  **[00:02:13-2] Person 1**: I'm a midwife at the CSPS in Colma 1. I've been the maternity manager for about two years now. Overall, I've been in public service for 16 years, but specifically as a midwife, it's been five years. |
| --- |

### Focus group of Farakan health and social promotion center

| [00:00:02-0](http://localhost:2300/file=C:/Users/USER/Desktop/ENTRETIENS%20PREMATURITE/CSPS_FARAKAN/FOCUS_GROUPE-CSPS_FARAKAN.MP3time=2000) Interviewer: CSPS de farfan. Le 09/08/2024,discussions de groupe avec accoucheuses et sage Femmes.Consentement éclairé, Bonjour, merci beaucoup d'avance pour votre disponibilité et le temps que vous nous accordez. Nous sommes une équipe de recherche de l’Université de Gand, de l'IRSS et de AFRICSanté. Nous menons une recherche qualitative sur la naissance prématurée dans les milieux urbains du Burkina Faso. Notre objectif est de mieux comprendre ce phénomène pour améliorer les services et les soins de santé destinés aux mères et aux nourrissons prématurés. Votre participation est essentielle pour nous aider à comprendre les défis spécifiques auxquels vous êtes confronté dans votre centre de santé et pour identifier les opportunités d’amélioration.  L’entretien d’aujourd’hui sera confidentiel et les informations recueillies seront utilisées uniquement à des fins de recherche. Vous avez la liberté de ne pas répondre à toute question qui vous met mal à l'aise et vous pouvez arrêter l’entretien à tout moment. Avez-vous des questions avant que nous commencions ? [00:01:06-1](http://localhost:2300/file=C:/Users/USER/Desktop/ENTRETIENS%20PREMATURITE/CSPS_FARAKAN/FOCUS_GROUPE-CSPS_FARAKAN.MP3time=66100)  [00:01:06-1](http://localhost:2300/file=C:/Users/USER/Desktop/ENTRETIENS%20PREMATURITE/CSPS_FARAKAN/FOCUS_GROUPE-CSPS_FARAKAN.MP3time=66100) Person 1: Nom [00:01:08-1](http://localhost:2300/file=C:/Users/USER/Desktop/ENTRETIENS%20PREMATURITE/CSPS_FARAKAN/FOCUS_GROUPE-CSPS_FARAKAN.MP3time=68100)  [00:01:08-1](http://localhost:2300/file=C:/Users/USER/Desktop/ENTRETIENS%20PREMATURITE/CSPS_FARAKAN/FOCUS_GROUPE-CSPS_FARAKAN.MP3time=68100) Interviewer: Avec votre permission, pouvons-nous commencer l’entretien ? Est-ce que vous êtes d'accord pour participer à l'entretien ? [00:01:14-8](http://localhost:2300/file=C:/Users/USER/Desktop/ENTRETIENS%20PREMATURITE/CSPS_FARAKAN/FOCUS_GROUPE-CSPS_FARAKAN.MP3time=74800)  [00:01:14-8](http://localhost:2300/file=C:/Users/USER/Desktop/ENTRETIENS%20PREMATURITE/CSPS_FARAKAN/FOCUS_GROUPE-CSPS_FARAKAN.MP3time=74800) Person 2: Oui, je suis d'accord. [00:01:16-5](http://localhost:2300/file=C:/Users/USER/Desktop/ENTRETIENS%20PREMATURITE/CSPS_FARAKAN/FOCUS_GROUPE-CSPS_FARAKAN.MP3time=76500)  [00:01:16-5](http://localhost:2300/file=C:/Users/USER/Desktop/ENTRETIENS%20PREMATURITE/CSPS_FARAKAN/FOCUS_GROUPE-CSPS_FARAKAN.MP3time=76500) Person 3:Oui, je suis. [00:01:17-7](http://localhost:2300/file=C:/Users/USER/Desktop/ENTRETIENS%20PREMATURITE/CSPS_FARAKAN/FOCUS_GROUPE-CSPS_FARAKAN.MP3time=77700)  [00:01:17-7](http://localhost:2300/file=C:/Users/USER/Desktop/ENTRETIENS%20PREMATURITE/CSPS_FARAKAN/FOCUS_GROUPE-CSPS_FARAKAN.MP3time=77700) Person 4: D'accord, oui, je suis d'accord. [00:01:21-6](http://localhost:2300/file=C:/Users/USER/Desktop/ENTRETIENS%20PREMATURITE/CSPS_FARAKAN/FOCUS_GROUPE-CSPS_FARAKAN.MP3time=81600)  [00:01:21-6](http://localhost:2300/file=C:/Users/USER/Desktop/ENTRETIENS%20PREMATURITE/CSPS_FARAKAN/FOCUS_GROUPE-CSPS_FARAKAN.MP3time=81600) Person 6: Oui. [00:01:23-3](http://localhost:2300/file=C:/Users/USER/Desktop/ENTRETIENS%20PREMATURITE/CSPS_FARAKAN/FOCUS_GROUPE-CSPS_FARAKAN.MP3time=83300)  [00:01:23-3](http://localhost:2300/file=C:/Users/USER/Desktop/ENTRETIENS%20PREMATURITE/CSPS_FARAKAN/FOCUS_GROUPE-CSPS_FARAKAN.MP3time=83300) Person 7: Oui. [00:01:27-1](http://localhost:2300/file=C:/Users/USER/Desktop/ENTRETIENS%20PREMATURITE/CSPS_FARAKAN/FOCUS_GROUPE-CSPS_FARAKAN.MP3time=87100)  [00:01:27-1](http://localhost:2300/file=C:/Users/USER/Desktop/ENTRETIENS%20PREMATURITE/CSPS_FARAKAN/FOCUS_GROUPE-CSPS_FARAKAN.MP3time=87100) Interviewer: Ok.D'accord, donc nous allons commencer la première question, pouvez-vous commencer par vous présenter votre titre, votre fonction et votre rôle s'il vous plaît ? [00:01:41-8](http://localhost:2300/file=C:/Users/USER/Desktop/ENTRETIENS%20PREMATURITE/CSPS_FARAKAN/FOCUS_GROUPE-CSPS_FARAKAN.MP3time=101800)  [00:01:41-8](http://localhost:2300/file=C:/Users/USER/Desktop/ENTRETIENS%20PREMATURITE/CSPS_FARAKAN/FOCUS_GROUPE-CSPS_FARAKAN.MP3time=101800) Person 1: De me présenter? Je suis accoucheuse auxiliare, 3 ans au csps de farakan. [00:01:47-3](http://localhost:2300/file=C:/Users/USER/Desktop/ENTRETIENS%20PREMATURITE/CSPS_FARAKAN/FOCUS_GROUPE-CSPS_FARAKAN.MP3time=107300)  [00:01:47-3](http://localhost:2300/file=C:/Users/USER/Desktop/ENTRETIENS%20PREMATURITE/CSPS_FARAKAN/FOCUS_GROUPE-CSPS_FARAKAN.MP3time=107300) Person 2: Je suis accoucheuse auxiliare, je suis au ici cela fait 3 ans. [00:01:52-0](http://localhost:2300/file=C:/Users/USER/Desktop/ENTRETIENS%20PREMATURITE/CSPS_FARAKAN/FOCUS_GROUPE-CSPS_FARAKAN.MP3time=112000)  [00:01:52-0](http://localhost:2300/file=C:/Users/USER/Desktop/ENTRETIENS%20PREMATURITE/CSPS_FARAKAN/FOCUS_GROUPE-CSPS_FARAKAN.MP3time=112000) Person 3: je suis accoucheuse auxiliare, 3 ans au csps. [00:01:57-4](http://localhost:2300/file=C:/Users/USER/Desktop/ENTRETIENS%20PREMATURITE/CSPS_FARAKAN/FOCUS_GROUPE-CSPS_FARAKAN.MP3time=117400)  [00:01:57-4](http://localhost:2300/file=C:/Users/USER/Desktop/ENTRETIENS%20PREMATURITE/CSPS_FARAKAN/FOCUS_GROUPE-CSPS_FARAKAN.MP3time=117400) Person 4: Je suis sage femme et je suis au csps de farakan cela fait 8 ans. [00:02:03-5](http://localhost:2300/file=C:/Users/USER/Desktop/ENTRETIENS%20PREMATURITE/CSPS_FARAKAN/FOCUS_GROUPE-CSPS_FARAKAN.MP3time=123500)  [00:02:03-5](http://localhost:2300/file=C:/Users/USER/Desktop/ENTRETIENS%20PREMATURITE/CSPS_FARAKAN/FOCUS_GROUPE-CSPS_FARAKAN.MP3time=123500) Person 5: je suis accoucheuse auxiliare, je suis au csps de farakan cela fait 7 ans. [00:02:07-8](http://localhost:2300/file=C:/Users/USER/Desktop/ENTRETIENS%20PREMATURITE/CSPS_FARAKAN/FOCUS_GROUPE-CSPS_FARAKAN.MP3time=127800)  [00:02:07-8](http://localhost:2300/file=C:/Users/USER/Desktop/ENTRETIENS%20PREMATURITE/CSPS_FARAKAN/FOCUS_GROUPE-CSPS_FARAKAN.MP3time=127800) Person 6: je suis accoucheuse auxiliare au csps de farakan. la durée c'est 4 ans. [00:02:12-9](http://localhost:2300/file=C:/Users/USER/Desktop/ENTRETIENS%20PREMATURITE/CSPS_FARAKAN/FOCUS_GROUPE-CSPS_FARAKAN.MP3time=132900)  [00:02:12-9](http://localhost:2300/file=C:/Users/USER/Desktop/ENTRETIENS%20PREMATURITE/CSPS_FARAKAN/FOCUS_GROUPE-CSPS_FARAKAN.MP3time=132900) Person 7: je suis accoucheuse auxiliare 10 ans au csps. [00:02:44-8](http://localhost:2300/file=C:/Users/USER/Desktop/ENTRETIENS%20PREMATURITE/CSPS_FARAKAN/FOCUS_GROUPE-CSPS_FARAKAN.MP3time=164800)  [00:02:44-8](http://localhost:2300/file=C:/Users/USER/Desktop/ENTRETIENS%20PREMATURITE/CSPS_FARAKAN/FOCUS_GROUPE-CSPS_FARAKAN.MP3time=164800) Person 8: Je suis accoucheuse auxiliare 10 ans au csps. [00:02:52-6](http://localhost:2300/file=C:/Users/USER/Desktop/ENTRETIENS%20PREMATURITE/CSPS_FARAKAN/FOCUS_GROUPE-CSPS_FARAKAN.MP3time=172600) |
| --- |

| 00:00:02-0 Interviewer: CSPS de Farakan. Le 09/08/2024, discussions de groupe avec accoucheuses et sage-femmes. Consentement éclairé, Bonjour, merci beaucoup d'avance pour votre disponibilité et le temps que vous nous accordez. Nous sommes une équipe de recherche de l’Université de Gand, de l'IRSS et d'AFRICSanté. Nous menons une recherche qualitative sur la naissance prématurée dans les milieux urbains du Burkina Faso. Notre objectif est de mieux comprendre ce phénomène pour améliorer les services et les soins de santé destinés aux mères et aux nourrissons prématurés. Votre participation est essentielle pour nous aider à comprendre les défis spécifiques auxquels vous êtes confrontées dans votre centre de santé et pour identifier les opportunités d’amélioration. L’entretien d’aujourd’hui sera confidentiel et les informations recueillies seront utilisées uniquement à des fins de recherche. Vous avez la liberté de ne pas répondre à toute question qui vous met mal à l'aise et vous pouvez arrêter l’entretien à tout moment. Avez-vous des questions avant que nous commencions ?  00:01:06-1 Person 1: Nom.  00:01:08-1 Interviewer: Avec votre permission, pouvons-nous commencer l’entretien ? Est-ce que vous êtes d'accord pour participer à l'entretien ?  00:01:14-8 Person 2: Oui, je suis d'accord.  00:01:16-5 Person 3: Oui, je suis.  00:01:17-7 Person 4: D'accord, oui, je suis d'accord.  00:01:21-6 Person 6: Oui.  00:01:23-3 Person 7: Oui.  00:01:27-1 Interviewer: Ok. D'accord, donc nous allons commencer la première question, pouvez-vous commencer par vous présenter, votre titre, votre fonction et votre rôle s'il vous plaît ? 00:01:41-8  00:01:41-8 Person 1: De me présenter ? Je suis accoucheuse auxiliaire, 3 ans au CSPS de Farakan. 00:01:47-3  00:01:47-3 Person 2: Je suis accoucheuse auxiliaire, je suis ici cela fait 3 ans. 00:01:52-0  00:01:52-0 Person 3: Je suis accoucheuse auxiliaire, 3 ans au CSPS. 00:01:57-4  00:01:57-4 Person 4: Je suis sage-femme et je suis au CSPS de Farakan cela fait 8 ans. 00:02:03-5  00:02:03-5 Person 5: Je suis accoucheuse auxiliaire, je suis au CSPS de Farakan cela fait 7 ans. 00:02:07-8  00:02:07-8 Person 6: Je suis accoucheuse auxiliaire au CSPS de Farakan. La durée c'est 4 ans. 00:02:12-9  00:02:12-9 Person 7: Je suis accoucheuse auxiliaire 10 ans au CSPS. 00:02:44-8  00:02:44-8 Person 8: Je suis accoucheuse auxiliaire 10 ans au CSPS. 00:02:52-6 |
| --- |

| **[00:00:02-0] Interviewer**: We're at Farakan CSPS, and today's date is August 9, 2024. This session involves group discussions with birth attendants and midwives. Before we start, I want to express our gratitude for your time and willingness to participate. We are from Ghent University, IRSS, and AFRICSanté, conducting qualitative research on premature birth in urban Burkina Faso. The purpose of this study is to gain a better understanding of this issue to enhance healthcare services for mothers and premature infants. Your insights will help us tackle specific challenges at your health center and explore improvement opportunities. Please note that this interview is confidential, and all information gathered will be used solely for research purposes. You are free to refrain from answering any questions that you find uncomfortable and can terminate the interview at any point. Do you have any questions before we begin?  **[00:01:06-1] Person 1**: [Response appears to be missing or unclear]  **[00:01:08-1] Interviewer**: With your permission, shall we start the interview? Do you consent to participate in the interview?  **[00:01:14-8] Person 2**: Yes, I agree.  **[00:01:16-5] Person 3**: Yes, I am willing.  **[00:01:17-7] Person 4**: Okay, yes, I agree.  **[00:01:21-6] Person 6**: Yes.  **[00:01:23-3] Person 7**: Yes.  **[00:01:27-1] Interviewer**: Alright. Let's begin with the first question. Can you start by introducing yourself, including your title and role, please?  **[00:01:41-8] Person 1**: To introduce myself? I am an auxiliary midwife, and I have been at the Farakan CSPS for 3 years.  **[00:01:47-3] Person 2**: I am an auxiliary birth attendant and have been here for 3 years.  **[00:01:52-0] Person 3**: I'm also an auxiliary midwife, with 3 years at the CSPS.  **[00:01:57-4] Person 4**: I am a midwife and have been working at the Farakan CSPS for 8 years.  **[00:02:03-5] Person 5**: I am an auxiliary midwife, at Farakan CSPS for 7 years.  **[00:02:07-8] Person 6**: I am an auxiliary midwife at Farakan CSPS, with a tenure of 4 years.  **[00:02:12-9] Person 7**: I've been an auxiliary midwife for 10 years at the CSPS.  **[00:02:44-8] Person 8**: Similarly, I have been an auxiliary midwife for 10 years at the CSPS. |
| --- |

### Major of Farakan health and social promotion center

| [00:00:01-4](http://localhost:2300/file=C:/Users/USER/Desktop/ENTRETIENS%20PREMATURITE/CSPS_FARAKAN/MAJOR%20FARAKAN.MP3time=1400) Interviewer: Entretien avec la responsable du CSPS de farakan. Le major du CSPS de Farakhan. Aujourd'hui, c'est le 11/08/2024, il est 10h34mn. Bonjour madame, nous sommes ici dans le cadre d'un entretien sur la prématurité en milieu urbain.Nous avons quelques questions à vous poser mais comme c'est un entretien qui doit être enregistré, il nous faut d'abord votre consentement, consentement que je vais lire à haute voix à travers lequel vous aurez les objectifs de cet entretien. [00:00:45-2](http://localhost:2300/file=C:/Users/USER/Desktop/ENTRETIENS%20PREMATURITE/CSPS_FARAKAN/MAJOR%20FARAKAN.MP3time=45200)  [00:00:45-2](http://localhost:2300/file=C:/Users/USER/Desktop/ENTRETIENS%20PREMATURITE/CSPS_FARAKAN/MAJOR%20FARAKAN.MP3time=45200) Person 1: D'accord. [00:00:45-7](http://localhost:2300/file=C:/Users/USER/Desktop/ENTRETIENS%20PREMATURITE/CSPS_FARAKAN/MAJOR%20FARAKAN.MP3time=45700)  [00:00:45-7](http://localhost:2300/file=C:/Users/USER/Desktop/ENTRETIENS%20PREMATURITE/CSPS_FARAKAN/MAJOR%20FARAKAN.MP3time=45700) Interviewer: Consentement éclairé: Bonjour, merci beaucoup d'avance pour votre disponibilité et le temps que vous nous accordez. Nous sommes une équipe de recherche de l’Université de Gand, de l'IRSS et de AFRICSanté. Nous menons une recherche qualitative sur la naissance prématurée dans les milieux urbains du Burkina Faso. Notre objectif est de mieux comprendre ce phénomène pour améliorer les services et les soins de santé destinés aux mères et aux nourrissons prématurés. Votre participation est essentielle pour nous aider à comprendre les défis spécifiques auxquels vous êtes confronté dans votre centre de santé et pour identifier les opportunités d’amélioration.  L’entretien d’aujourd’hui sera confidentiel et les informations recueillies seront utilisées uniquement à des fins de recherche. Vous avez la liberté de ne pas répondre à toute question qui vous met mal à l'aise et vous pouvez arrêter l’entretien à tout moment. Avez-vous des questions avant que nous commencions ? [00:01:38-1](http://localhost:2300/file=C:/Users/USER/Desktop/ENTRETIENS%20PREMATURITE/CSPS_FARAKAN/MAJOR%20FARAKAN.MP3time=98100)  [00:01:38-1](http://localhost:2300/file=C:/Users/USER/Desktop/ENTRETIENS%20PREMATURITE/CSPS_FARAKAN/MAJOR%20FARAKAN.MP3time=98100) Person 1: ça peut aller. [00:01:39-6](http://localhost:2300/file=C:/Users/USER/Desktop/ENTRETIENS%20PREMATURITE/CSPS_FARAKAN/MAJOR%20FARAKAN.MP3time=99600)  [00:01:39-6](http://localhost:2300/file=C:/Users/USER/Desktop/ENTRETIENS%20PREMATURITE/CSPS_FARAKAN/MAJOR%20FARAKAN.MP3time=99600) Interviewer: Ok.Avec votre permission, nous allons commencer l’entretien. Est ce que vous pouvez déjà commencer par vous presentez,titre ,fonction, durée au niveau de cette formation sanitaire sans le nom. [00:01:52-2](http://localhost:2300/file=C:/Users/USER/Desktop/ENTRETIENS%20PREMATURITE/CSPS_FARAKAN/MAJOR%20FARAKAN.MP3time=112200)  [00:01:52-2](http://localhost:2300/file=C:/Users/USER/Desktop/ENTRETIENS%20PREMATURITE/CSPS_FARAKAN/MAJOR%20FARAKAN.MP3time=112200) Person 1: Sans le nom (rire). Je suis la coordinatrice sortante parce que je suis affectée, je suis IDE. [00:02:05-1](http://localhost:2300/file=C:/Users/USER/Desktop/ENTRETIENS%20PREMATURITE/CSPS_FARAKAN/MAJOR%20FARAKAN.MP3time=125100) |
| --- |

| 00:00:01-4 Interviewer: Entretien avec la responsable du CSPS de Farakan. Le major du CSPS de Farakan. Aujourd'hui, c'est le 11/08/2024, il est 10h34mn. Bonjour Madame, nous sommes ici dans le cadre d'un entretien sur la prématurité en milieu urbain. Nous avons quelques questions à vous poser mais comme c'est un entretien qui doit être enregistré, il nous faut d'abord votre consentement, consentement que je vais lire à haute voix à travers lequel vous aurez les objectifs de cet entretien.  00:00:45-2 Person 1: D'accord.  00:00:45-7 Interviewer: Consentement éclairé: Bonjour, merci beaucoup d'avance pour votre disponibilité et le temps que vous nous accordez. Nous sommes une équipe de recherche de l’Université de Gand, de l'IRSS et d'AFRICSanté. Nous menons une recherche qualitative sur la naissance prématurée dans les milieux urbains du Burkina Faso. Notre objectif est de mieux comprendre ce phénomène pour améliorer les services et les soins de santé destinés aux mères et aux nourrissons prématurés. Votre participation est essentielle pour nous aider à comprendre les défis spécifiques auxquels vous êtes confronté dans votre centre de santé et pour identifier les opportunités d’amélioration. L’entretien d’aujourd’hui sera confidentiel et les informations recueillies seront utilisées uniquement à des fins de recherche. Vous avez la liberté de ne pas répondre à toute question qui vous met mal à l'aise et vous pouvez arrêter l’entretien à tout moment. Avez-vous des questions avant que nous commencions ?  00:01:38-1 Person 1: Ça peut aller.  00:01:39-6 Interviewer: Ok. Avec votre permission, nous allons commencer l’entretien. Est-ce que vous pouvez déjà commencer par vous présenter, titre, fonction, durée au niveau de cette formation sanitaire sans le nom ? 00:01:52-2  00:01:52-2 Person 1: Sans le nom (rire). Je suis la coordinatrice sortante parce que je suis affectée, je suis IDE. 00:02:05-1 |
| --- |

| **[00:00:01-4] Interviewer**: We're here for an interview with the head of Farakan CSPS, the CSPS major from Farakan. Today's date is August 11, 2024, and the time is 10:34 a.m. Good morning, Madam. We are conducting an interview regarding prematurity in urban areas. We have several questions for you, but before we proceed, we need your consent to record this interview. I will read the informed consent aloud for you, which includes the objectives of our interview.  **[00:00:45-2] Person 1**: Okay.  **[00:00:45-7] Interviewer**: Informed consent: Thank you in advance for your time and availability. We are a research team from Ghent University, IRSS, and AFRICSanté, conducting qualitative research on premature birth in urban areas of Burkina Faso. Our aim is to gain a deeper understanding of this issue to improve healthcare services for mothers and premature infants. Your participation is vital in helping us identify the specific challenges at your health center and discover opportunities for improvement. Please be assured that this interview is confidential, and all collected information will be used solely for research purposes. You have the freedom to not answer any questions that make you uncomfortable, and you can end the interview at any time. Do you have any questions before we begin?  **[00:01:38-1] Person 1**: It's okay.  **[00:01:39-6] Interviewer**: Okay. With your permission, let's start the interview. Could you please introduce yourself, including your title, function, and how long you've been at this health facility, but without mentioning your name?  **[00:01:52-2] Person 1**: Without the name? (laughter) I am the outgoing coordinator, as I have been reassigned. I'm an IDE (Infirmier Diplômé d'État, or State Registered Nurse). |
| --- |

### Maternity manager of Farakan health and social promotion center

| [00:00:05-1](http://localhost:2300/file=C:/Users/USER/Desktop/ENTRETIENS%20PREMATURITE/CSPS_FARAKAN/RESPONSABLE%20MATERNITE%20FARAKAN.MP3time=5100) Interviewer: CSPS de FARAKAN, [00:00:08-9](http://localhost:2300/file=C:/Users/USER/Desktop/ENTRETIENS%20PREMATURITE/CSPS_FARAKAN/RESPONSABLE%20MATERNITE%20FARAKAN.MP3time=8900)  [00:00:08-9](http://localhost:2300/file=C:/Users/USER/Desktop/ENTRETIENS%20PREMATURITE/CSPS_FARAKAN/RESPONSABLE%20MATERNITE%20FARAKAN.MP3time=8900) Person 1: c'est un CMU, centre medical urbain. [00:00:11-9](http://localhost:2300/file=C:/Users/USER/Desktop/ENTRETIENS%20PREMATURITE/CSPS_FARAKAN/RESPONSABLE%20MATERNITE%20FARAKAN.MP3time=11900)  [00:00:11-9](http://localhost:2300/file=C:/Users/USER/Desktop/ENTRETIENS%20PREMATURITE/CSPS_FARAKAN/RESPONSABLE%20MATERNITE%20FARAKAN.MP3time=11900) Interviewer: Ok CMU de FARAKAN, le 09/08/2024 il est 14h26 donc entretien avec la responsable de la maternité du CMU de farakan. Maintenant madame, je vais lire le consentement éclairé à haute voix.Bonjour, merci beaucoup d'avance pour votre disponibilité et le temps que vous nous accordez. Nous sommes une équipe de recherche de l’Université de Gand, de l'IRSS et de AFRICSanté. Nous menons une recherche qualitative sur la naissance prématurée dans les milieux urbains du Burkina Faso. Notre objectif est de mieux comprendre ce phénomène pour améliorer les services et les soins de santé destinés aux mères et aux nourrissons prématurés. Votre participation est essentielle pour nous aider à comprendre les défis spécifiques auxquels vous êtes confronté dans votre centre de santé et pour identifier les opportunités d’amélioration.  L’entretien d’aujourd’hui sera confidentiel et les informations recueillies seront utilisées uniquement à des fins de recherche. Vous avez la liberté de ne pas répondre à toute question qui vous met mal à l'aise et vous pouvez arrêter l’entretien à tout moment. Avez-vous des questions avant que nous commencions ? [00:01:26-3](http://localhost:2300/file=C:/Users/USER/Desktop/ENTRETIENS%20PREMATURITE/CSPS_FARAKAN/RESPONSABLE%20MATERNITE%20FARAKAN.MP3time=86300)  [00:01:26-3](http://localhost:2300/file=C:/Users/USER/Desktop/ENTRETIENS%20PREMATURITE/CSPS_FARAKAN/RESPONSABLE%20MATERNITE%20FARAKAN.MP3time=86300) Person 1: Je pense que ça comme ça concerne la prématurité, il n' y a pas de question. [00:01:29-4](http://localhost:2300/file=C:/Users/USER/Desktop/ENTRETIENS%20PREMATURITE/CSPS_FARAKAN/RESPONSABLE%20MATERNITE%20FARAKAN.MP3time=89400)  [00:01:29-4](http://localhost:2300/file=C:/Users/USER/Desktop/ENTRETIENS%20PREMATURITE/CSPS_FARAKAN/RESPONSABLE%20MATERNITE%20FARAKAN.MP3time=89400) Interviewer: Ok, donc d'accord donc avec votre permission, nous allons commencer l'entretien. [00:01:34-5](http://localhost:2300/file=C:/Users/USER/Desktop/ENTRETIENS%20PREMATURITE/CSPS_FARAKAN/RESPONSABLE%20MATERNITE%20FARAKAN.MP3time=94500)  [00:01:34-5](http://localhost:2300/file=C:/Users/USER/Desktop/ENTRETIENS%20PREMATURITE/CSPS_FARAKAN/RESPONSABLE%20MATERNITE%20FARAKAN.MP3time=94500) Person 1: Oui [00:01:34-8](http://localhost:2300/file=C:/Users/USER/Desktop/ENTRETIENS%20PREMATURITE/CSPS_FARAKAN/RESPONSABLE%20MATERNITE%20FARAKAN.MP3time=94800)  [00:01:34-8](http://localhost:2300/file=C:/Users/USER/Desktop/ENTRETIENS%20PREMATURITE/CSPS_FARAKAN/RESPONSABLE%20MATERNITE%20FARAKAN.MP3time=94800) Interviewer: Pourriez-vous commencer par vous présenter ? Pas de nom, votre titre, votre fonction et votre rôle s'il vous plaît? [00:01:41-6](http://localhost:2300/file=C:/Users/USER/Desktop/ENTRETIENS%20PREMATURITE/CSPS_FARAKAN/RESPONSABLE%20MATERNITE%20FARAKAN.MP3time=101600)  [00:01:41-6](http://localhost:2300/file=C:/Users/USER/Desktop/ENTRETIENS%20PREMATURITE/CSPS_FARAKAN/RESPONSABLE%20MATERNITE%20FARAKAN.MP3time=101600) Person 1: Moi je suis responsable de la maternité, je suis sage femme,je suis au csps de farakan depuis 2012. [00:02:00-6](http://localhost:2300/file=C:/Users/USER/Desktop/ENTRETIENS%20PREMATURITE/CSPS_FARAKAN/RESPONSABLE%20MATERNITE%20FARAKAN.MP3time=120600) |
| --- |

| 00:00:05-1 Interviewer: CSPS de Farakan.  00:00:08-9 Person 1: C'est un CMU, centre médical urbain.  00:00:11-9 Interviewer: Ok, CMU de Farakan, le 09/08/2024, il est 14h26 donc entretien avec la responsable de la maternité du CMU de Farakan. Maintenant Madame, je vais lire le consentement éclairé à haute voix. Bonjour, merci beaucoup d'avance pour votre disponibilité et le temps que vous nous accordez. Nous sommes une équipe de recherche de l’Université de Gand, de l'IRSS et d'AFRICSanté. Nous menons une recherche qualitative sur la naissance prématurée dans les milieux urbains du Burkina Faso. Notre objectif est de mieux comprendre ce phénomène pour améliorer les services et les soins de santé destinés aux mères et aux nourrissons prématurés. Votre participation est essentielle pour nous aider à comprendre les défis spécifiques auxquels vous êtes confrontée dans votre centre de santé et pour identifier les opportunités d’amélioration. L’entretien d’aujourd’hui sera confidentiel et les informations recueillies seront utilisées uniquement à des fins de recherche. Vous avez la liberté de ne pas répondre à toute question qui vous met mal à l'aise et vous pouvez arrêter l’entretien à tout moment. Avez-vous des questions avant que nous commencions ?  00:01:26-3 Person 1: Je pense que ça comme ça concerne la prématurité, il n'y a pas de question.  00:01:29-4 Interviewer: Ok, donc d'accord donc avec votre permission, nous allons commencer l'entretien.  00:01:34-5 Person 1: Oui.  00:01:34-8 Interviewer: Pourriez-vous commencer par vous présenter ? Pas de nom, votre titre, votre fonction et votre rôle s'il vous plaît ? 00:01:41-6  00:01:41-6 Person 1: Moi, je suis responsable de la maternité, je suis sage-femme, je suis au CSPS de Farakan depuis 2012. 00:02:00-6 |
| --- |

| **[00:00:05-1] Interviewer**: We're at Farakan CSPS for today's interview.  **[00:00:08-9] Person 1**: Actually, it's a CMU, an urban medical center.  **[00:00:11-9] Interviewer**: Okay, CMU of Farakan, the date is August 9, 2024, and the time is 2:26 p.m. We're here to interview the head of the maternity ward at CMU of Farakan. Now, Madam, I will read the informed consent aloud. Thank you in advance for your time and willingness to participate in this interview. We are a research team from Ghent University, IRSS, and AFRICSanté, conducting qualitative research on premature birth in urban areas of Burkina Faso. Our aim is to better understand this issue to enhance healthcare services for mothers and premature infants. Your insights are crucial to help us identify specific challenges at your health center and to discover improvement opportunities. This interview will remain confidential, and all information gathered will be used exclusively for research purposes. You are free to refrain from answering any questions that may cause discomfort, and you can end the interview at any time. Do you have any questions before we begin?  **[00:01:26-3] Person 1**: Regarding prematurity, no, I don't have any questions.  **[00:01:29-4] Interviewer**: Alright, with your permission, let's start the interview.  **[00:01:34-5] Person 1**: Yes, you can proceed.  **[00:01:34-8] Interviewer**: Could you begin by introducing yourself? Please include your title, function, and role, but omit your name.  **[00:01:41-6] Person 1**: I am responsible for the maternity ward. I'm a midwife and have been working at the Farakan CSPS since 2012. |
| --- |

# Definition of prematurity

### Focus group of Accart-ville urban medical center

| [00:01:43-8](http://localhost)Interviewer: maintenant comment définissez-vous la naissance prématuré dans votre centre de santé? [00:01:47-3](http://localh)  [00:01:47-3](http://localhost:2300/file=C:/Users/USER/Desktop/ENTRETIENS) Person 1: a naissance prématurée c'est toute naissance qui survient avant la trente septième semaine (avant 37 semaines) d'aménorrhée [00:01:56-0](http://localhost:2300/file=C:/Users/USER/Desktop/ENTRETIENS%20PREMATURITE/CSPS-ACCART-VILLE/FOCUS%20GROUPE%20ACCART-VILLE-BON.mp3time=116000) |
| --- |

| 00:01:43-8 Interviewer: Maintenant, comment définissez-vous la naissance prématurée dans votre centre de santé ? 00:01:47-3  00:01:47-3 Person 1: La naissance prématurée, c'est toute naissance qui survient avant la trente-septième semaine (avant 37 semaines) d'aménorrhée. 00:01:56-0 |
| --- |

| **[00:01:43-8] Interviewer**: Could you explain how you define premature birth in your health center?  **[00:01:47-3] Person 1**: Premature birth is defined as any birth that occurs before the thirty-seventh week, or before 37 weeks of amenorrhea. |
| --- |

### Manager of Accart-ville urban medical center

| [00:02:03-9](http://localhost:2300/file=C:/Users/USER/Desktop/ENTRETIENS%20PREMATURITE/CSPS-ACCART-VILLE/RESPONSABLE%20CMU%20ACCART-VILLE.MP3time=123900)Interviewer: Ok. Nous allons passer donc à la première question Comment est-ce que vous définissez la naissance prématurée dans votre centre de santé? [00:02:13-9](http://localhost:2300/file=C:/Users/USER/Desktop/ENTRETIENS%20PREMATURITE/CSPS-ACCART-VILLE/RESPONSABLE%20CMU%20ACCART-VILLE.MP3time=133900)  [00:02:13-9](http://localhost:2300/file=C:/Users/USER/Desktop/ENTRETIENS%20PREMATURITE/CSPS-ACCART-VILLE/RESPONSABLE%20CMU%20ACCART-VILLE.MP3time=133900)Person 1: la naissance prématurée dans notre centre de santé? C'est tout accouchement avant terme, c'est à dire avant 36 semaines d'aménorrhée. [00:02:26-4](http://localhost:2300/file=C:/Users/USER/Desktop/ENTRETIENS%20PREMATURITE/CSPS-ACCART-VILLE/RESPONSABLE%20CMU%20ACCART-VILLE.MP3time=146400) |
| --- |

| 00:02:03-9 Interviewer: Ok. Nous allons passer donc à la première question. Comment est-ce que vous définissez la naissance prématurée dans votre centre de santé ? 00:02:13-9  00:02:13-9 Person 1: La naissance prématurée dans notre centre de santé ? C'est tout accouchement avant terme, c'est-à-dire avant 36 semaines d'aménorrhée. 00:02:26-4 |
| --- |

| **[00:02:03-9] Interviewer**: Okay, let's move on to the first question. How do you define premature birth in your health center?  **[00:02:13-9] Person 1**: Premature birth in our health center is defined as any preterm delivery, specifically occurring before 36 weeks of amenorrhea. |
| --- |

### Maternity manager of Accart-ville urban medical center

| [00:01:38-1](http://localhost:2300/file=C:/Users/USER/Desktop/ENTRETIENS%20PREMATURITE/CSPS-ACCART-VILLE/RESPONSABLE%20MATERNITE%20CMU%20ACCART-VILLE.MP3time=98100) Interviewer: ok maintenant par rapport à la prémière question, Comment définissez-vous la naissance prématurée dans votre centre de santé ? [00:01:45-2](http://localhost:2300/file=C:/Users/USER/Desktop/ENTRETIENS%20PREMATURITE/CSPS-ACCART-VILLE/RESPONSABLE%20MATERNITE%20CMU%20ACCART-VILLE.MP3time=105200)  [00:01:45-2](http://localhost:2300/file=C:/Users/USER/Desktop/ENTRETIENS%20PREMATURITE/CSPS-ACCART-VILLE/RESPONSABLE%20MATERNITE%20CMU%20ACCART-VILLE.MP3time=105200) Person 1: la naissance prématuré c'est toute naissance qui survient entre la vingthuitième semaine et la trente sixième semaine six jours [00:01:53-5](http://localhost:2300/file=C:/Users/USER/Desktop/ENTRETIENS%20PREMATURITE/CSPS-ACCART-VILLE/RESPONSABLE%20MATERNITE%20CMU%20ACCART-VILLE.MP3time=113500) |
| --- |

| 00:01:38-1 Interviewer: Ok, maintenant par rapport à la première question, comment définissez-vous la naissance prématurée dans votre centre de santé ? 00:01:45-2  00:01:45-2 Person 1: La naissance prématurée c'est toute naissance qui survient entre la vingt-huitième semaine et la trente-sixième semaine six jours. 00:01:53-5 |
| --- |

| **[00:01:38-1] Interviewer**: Okay, moving on to the first question, how is premature birth defined in your health center?  **[00:01:45-2] Person 1**: Premature birth is defined as any birth that occurs between the twenty-eighth week and thirty-six weeks and six days of pregnancy. |
| --- |

### Focus group of Do medical center with surgical branch

| [00:03:06-7](http://localhost:2300/file=C:/Users/USER/Desktop/ENTRETIENS%20PREMATURITE/CMA-DO/FOCUS%20GROUPE%20CMA-DO.mp3time=186700) Interviewer: Ok merci pour la présentation.Donc nous allons commencer avec la première question. Comment est-ce que vous définissez la naissance prématurée au sein de votre centre de santé? [00:03:18-0](http://localhost:2300/file=C:/Users/USER/Desktop/ENTRETIENS%20PREMATURITE/CMA-DO/FOCUS%20GROUPE%20CMA-DO.mp3time=198000)  [00:03:18-0](http://localhost:2300/file=C:/Users/USER/Desktop/ENTRETIENS%20PREMATURITE/CMA-DO/FOCUS%20GROUPE%20CMA-DO.mp3time=198000) Person 1: C'est toutes les naissances avant 37 semaine. [00:03:21-0](http://localhost:2300/file=C:/Users/USER/Desktop/ENTRETIENS%20PREMATURITE/CMA-DO/FOCUS%20GROUPE%20CMA-DO.mp3time=201000)  [00:03:21-0](http://localhost:2300/file=C:/Users/USER/Desktop/ENTRETIENS%20PREMATURITE/CMA-DO/FOCUS%20GROUPE%20CMA-DO.mp3time=201000) Interviewer: Toutes naissances avant 37 semaine. Est-ce qu'il y a quelque chose à ajouter? [00:03:31-0](http://localhost:2300/file=C:/Users/USER/Desktop/ENTRETIENS%20PREMATURITE/CMA-DO/FOCUS%20GROUPE%20CMA-DO.mp3time=211000)  [00:03:31-0](http://localhost:2300/file=C:/Users/USER/Desktop/ENTRETIENS%20PREMATURITE/CMA-DO/FOCUS%20GROUPE%20CMA-DO.mp3time=211000) Person 3: Non. [00:03:31-9](http://localhost:2300/file=C:/Users/USER/Desktop/ENTRETIENS%20PREMATURITE/CMA-DO/FOCUS%20GROUPE%20CMA-DO.mp3time=211900) |
| --- |

| 00:03:06-7 Interviewer: Ok, merci pour la présentation. Donc nous allons commencer avec la première question. Comment est-ce que vous définissez la naissance prématurée au sein de votre centre de santé ? 00:03:18-0  00:03:18-0 Person 1: C'est toutes les naissances avant 37 semaines. 00:03:21-0  00:03:21-0 Interviewer: Toutes naissances avant 37 semaines. Est-ce qu'il y a quelque chose à ajouter ? 00:03:31-0  00:03:31-0 Person 3: Non. 00:03:31-9 |
| --- |

| **[00:03:06-7] Interviewer**: Thank you for the introductions. Let's start with the first question. How do you define premature birth within your health center?  **[00:03:18-0] Person 1**: It's any birth that occurs before 37 weeks.  **[00:03:21-0] Interviewer**: All births before 37 weeks. Is there anything else you would like to add?  **[00:03:31-0] Person 3**: No. |
| --- |

### Gynecologist 1

| [00:01:41-9](http://localhost:2300/file=C:/Users/USER/Desktop/ENTRETIENS%20PREMATURITE/CMA-DO/GENYCOLOGUE%2001.mp3time=101900) Interviewer: Nous allons passer à la première question. Comment est-ce que vous définissez la naissance prématurienne au sein du votre centre de santé ? [00:01:54-1](http://localhost:2300/file=C:/Users/USER/Desktop/ENTRETIENS%20PREMATURITE/CMA-DO/GENYCOLOGUE%2001.mp3time=114100)  [00:01:54-1](http://localhost:2300/file=C:/Users/USER/Desktop/ENTRETIENS%20PREMATURITE/CMA-DO/GENYCOLOGUE%2001.mp3time=114100) Person 1: La naissance prématurée, nous la définissons de deux façons. La prémière façon c'est par rapport à l'âge gestationnel, quand on est sûr de l'âge de la grossesse, une grossesse qui a moins de 36 semaines. Nous la classons parmi les prématurés. Par rapport au poids fœtal également, souvent le poids fœtal quand c'est inférieur à 2 kg,on a tendance à le classer parmi les prématurés. Bien sûr, il y a d'autres critères morphologiques qui rentrent en ligne de compte, mais c'est surtout le critère d'âge gestationnel inférieur à 36 semaines. [00:02:43-9](http://localhost:2300/file=C:/Users/USER/Desktop/ENTRETIENS%20PREMATURITE/CMA-DO/GENYCOLOGUE%2001.mp3time=163900) |
| --- |

| 00:01:41-9 Interviewer: Nous allons passer à la première question. Comment est-ce que vous définissez la naissance prématurée au sein de votre centre de santé ? 00:01:54-1  00:01:54-1 Person 1: La naissance prématurée, nous la définissons de deux façons. La première façon c'est par rapport à l'âge gestationnel, quand on est sûr de l'âge de la grossesse, une grossesse qui a moins de 36 semaines. Nous la classons parmi les prématurés. Par rapport au poids fœtal également, souvent le poids fœtal quand c'est inférieur à 2 kg, on a tendance à le classer parmi les prématurés. Bien sûr, il y a d'autres critères morphologiques qui rentrent en ligne de compte, mais c'est surtout le critère d'âge gestationnel inférieur à 36 semaines. 00:02:43-9 |
| --- |

| **[00:01:41-9] Interviewer**: Let's move on to the first question. How do you define premature birth within your health center?  **[00:01:54-1] Person 1**: In our health center, we define premature birth in two ways. Firstly, in terms of gestational age: if the age of the pregnancy is less than 36 weeks, we classify it as premature. Secondly, we consider fetal weight; often, if it is less than 2 kilograms, we tend to classify the birth as premature. Of course, there are other morphological criteria, but the primary criterion is a gestational age of less than 36 weeks. |
| --- |

### Gynecologist 2

| [00:01:56-9](http://localhost:2300/file=C:/Users/USER/Desktop/ENTRETIENS%20PREMATURITE/CMA-DO/GENYCOLOGUE%2002.mp3time=116900) Interviewer: Je pense que nous avons évolué au niveau des questions. La première question, comment définissez-vous la naissance prématurée ? [00:02:05-5](http://localhost:2300/file=C:/Users/USER/Desktop/ENTRETIENS%20PREMATURITE/CMA-DO/GENYCOLOGUE%2002.mp3time=125500)  [00:02:05-5](http://localhost:2300/file=C:/Users/USER/Desktop/ENTRETIENS%20PREMATURITE/CMA-DO/GENYCOLOGUE%2002.mp3time=125500) Person 1: Oui Ici, de façon classique, la naissance prématurée, c'est les enfants qui vont approcher entre 28 semaines et 37 semaines d'aménorrhées. Donc, de 28 à 37 semaines, l'enfant est dit prématuré. [00:02:23-4](http://localhost:2300/file=C:/Users/USER/Desktop/ENTRETIENS%20PREMATURITE/CMA-DO/GENYCOLOGUE%2002.mp3time=143400) |
| --- |

| 00:01:56-9 Interviewer: Je pense que nous avons évolué au niveau des questions. La première question, comment définissez-vous la naissance prématurée ? 00:02:05-5  00:02:05-5 Person 1: Oui, ici, de façon classique, la naissance prématurée, c'est les enfants qui vont approcher entre 28 semaines et 37 semaines d'aménorrhées. Donc, de 28 à 37 semaines, l'enfant est dit prématuré. 00:02:23-4 |
| --- |

| **[00:01:56-9] Interviewer**: I believe we're ready to delve into the questions. The first question: How do you define premature birth?  **[00:02:05-5] Person 1**: Yes, in our setting, premature birth is classically defined as births that occur between 28 weeks and 37 weeks of amenorrhea. Therefore, a child born within this range, from 28 to 37 weeks, is considered premature. |
| --- |

### Chief physician of Do health district

| [00:02:11-1](http://localhost:2300/file=C:/Users/USER/Desktop/ENTRETIENS%20PREMATURITE/CMA-DO/MCD-DO.mp3time=131100) Interviewer: Maintenant la première question, comment est-ce que vous définissez la naissance prématurée au sein du CMA-DÔ? [00:02:19-1](http://localhost:2300/file=C:/Users/USER/Desktop/ENTRETIENS%20PREMATURITE/CMA-DO/MCD-DO.mp3time=139100)  [00:02:19-1](http://localhost:2300/file=C:/Users/USER/Desktop/ENTRETIENS%20PREMATURITE/CMA-DO/MCD-DO.mp3time=139100) Person 1: Au sein du CMA? Non, est-ce qu'il y a une définition pour le CMA de DÔ? Je ne crois pas. La définition est un peu universelle pour la pratique de santé. Donc, on va dire une naissance qui n'a pas atteint les 9 mois, en global c'est ce qu'on peut dire. Toute naissance qui n'a pas atteint 37 semaines d'aménorrhée, c'est supposé comme une naissance prématurée. Mais là, il faut tenir compte de la subtilité de l'avortement. Quand les produits de conception est évacué avant les 22 semaines d'aménorrhée. Donc, il y a cette pochette là aussi il faut tenir compte. Parce que si on n'atteint pas les 22 semaines, c'est un avortement. Mais de 22 jusqu'avant 37, ça c'est une prématurité. [00:03:18-1](http://localhost:2300/file=C:/Users/USER/Desktop/ENTRETIENS%20PREMATURITE/CMA-DO/MCD-DO.mp3time=198100) |
| --- |

| 00:02:11-1 Interviewer: Maintenant, la première question, comment est-ce que vous définissez la naissance prématurée au sein du CMA-Dô ? 00:02:19-1  00:02:19-1 Person 1: Au sein du CMA ? Non, est-ce qu'il y a une définition pour le CMA de Dô ? Je ne crois pas. La définition est un peu universelle pour la pratique de santé. Donc, on va dire une naissance qui n'a pas atteint les 9 mois, en global c'est ce qu'on peut dire. Toute naissance qui n'a pas atteint 37 semaines d'aménorrhée, c'est considéré comme une naissance prématurée. Mais là, il faut tenir compte de la subtilité de l'avortement. Quand les produits de conception sont évacués avant les 22 semaines d'aménorrhée, donc, il y a cette pochette là aussi qu'il faut tenir compte. Parce que si on n'atteint pas les 22 semaines, c'est un avortement. Mais de 22 jusqu'à avant 37, ça, c'est une prématurité. 00:03:18-1 |
| --- |

| **[00:02:11-1] Interviewer**: Now for the first question, how do you define premature birth within CMA-Dô?  **[00:02:19-1] Person 1**: Within CMA-Dô? I don't think there's a specific definition for our center. The definition of premature birth is somewhat universal in health practice. Generally, it's any birth that occurs before 9 months, or more specifically, before 37 weeks of amenorrhea is considered premature. However, we also need to consider the aspect of abortion. When the products of conception are expelled before 22 weeks of amenorrhea, it's classified as an abortion. So, there's this distinction: before 22 weeks, it's an abortion, but any birth from 22 weeks up until before 37 weeks is considered premature. |
| --- |

### Manager for maternity care unit of Do medical center with surgical branch

| [00:02:12-2](http://localhost:2300/file=C:/Users/USER/Desktop/ENTRETIENS%20PREMATURITE/CMA-DO/RESPONSABLE%20UNITE%20DE%20SOIN%20CMA-DO.mp3time=132200) Interviewer: Ok, merci. Maintenant, nous allons passer directement aux questions.La première question, comment est-ce que vous définissez la naissance prématurée? [00:02:23-0](http://localhost:2300/file=C:/Users/USER/Desktop/ENTRETIENS%20PREMATURITE/CMA-DO/RESPONSABLE%20UNITE%20DE%20SOIN%20CMA-DO.mp3time=143000)  [00:02:23-0](http://localhost:2300/file=C:/Users/USER/Desktop/ENTRETIENS%20PREMATURITE/CMA-DO/RESPONSABLE%20UNITE%20DE%20SOIN%20CMA-DO.mp3time=143000) Person 1: La naissance prématurée,un accouchement prématuré, c'est un accouchement qui a eu lieu avant 36 semaines de grossesse.Donc en dessous de 36 semaines, on parlera de prématurité. [00:02:49-4](http://localhost:2300/file=C:/Users/USER/Desktop/ENTRETIENS%20PREMATURITE/CMA-DO/RESPONSABLE%20UNITE%20DE%20SOIN%20CMA-DO.mp3time=169400) |
| --- |

| 00:02:12-2 Interviewer: Ok, merci. Maintenant, nous allons passer directement aux questions. La première question, comment est-ce que vous définissez la naissance prématurée ? 00:02:23-0  00:02:23-0 Person 1: La naissance prématurée, un accouchement prématuré, c'est un accouchement qui a eu lieu avant 36 semaines de grossesse. Donc, en dessous de 36 semaines, on parlera de prématurité. 00:02:49-4 |
| --- |

| **[00:02:12-2] Interviewer**: Okay, thank you. Let's go straight to the questions. The first one is, how do you define premature birth?  **[00:02:23-0] Person 1**: Premature birth is a birth that occurs before 36 weeks of pregnancy. So, any birth below 36 weeks is considered premature. |
| --- |

### Focus group of Colma1 health and social promotion center

| [00:01:01-4](http://localhost:2300/file=C:/Users/USER/Desktop/ENTRETIENS%20PREMATURITE/CSPS-COLMA1/FOCUS%20GROUPE%20COLMA1.mp3time=61400) Interviewer: D'accord.Merci beaucoup pour la présentation.Donc je pense que si tout le monde est prêt, nous pouvons commencer.Maintenant la première question, comment est-ce que vous définissez la naissance prématurée ? [00:01:34-2](http://localhost:2300/file=C:/Users/USER/Desktop/ENTRETIENS%20PREMATURITE/CSPS-COLMA1/FOCUS%20GROUPE%20COLMA1.mp3time=94200)  [00:01:34-2](http://localhost:2300/file=C:/Users/USER/Desktop/ENTRETIENS%20PREMATURITE/CSPS-COLMA1/FOCUS%20GROUPE%20COLMA1.mp3time=94200) Person 1: la naissance prématurée, c'est un enfant qui n'est pas arrivé à sa terme de 9 mois. [00:01:38-4](http://localhost:2300/file=C:/Users/USER/Desktop/ENTRETIENS%20PREMATURITE/CSPS-COLMA1/FOCUS%20GROUPE%20COLMA1.mp3time=98400)  [00:01:38-4](http://localhost:2300/file=C:/Users/USER/Desktop/ENTRETIENS%20PREMATURITE/CSPS-COLMA1/FOCUS%20GROUPE%20COLMA1.mp3time=98400) Person 2: la naissance prématurée est un enfant qui n'est pas arrivé à sa terme de 9 mois , c'est un enfant qui est né avant les 9 mois. [00:01:47-4](http://localhost:2300/file=C:/Users/USER/Desktop/ENTRETIENS%20PREMATURITE/CSPS-COLMA1/FOCUS%20GROUPE%20COLMA1.mp3time=107400)  [00:01:47-4](http://localhost:2300/file=C:/Users/USER/Desktop/ENTRETIENS%20PREMATURITE/CSPS-COLMA1/FOCUS%20GROUPE%20COLMA1.mp3time=107400) Interviewer: est ce qu'il y a quelque chose à ajouter ou bien c'est c'est bon ? [00:01:55-8](http://localhost:2300/file=C:/Users/USER/Desktop/ENTRETIENS%20PREMATURITE/CSPS-COLMA1/FOCUS%20GROUPE%20COLMA1.mp3time=115800)  [00:01:55-8](http://localhost:2300/file=C:/Users/USER/Desktop/ENTRETIENS%20PREMATURITE/CSPS-COLMA1/FOCUS%20GROUPE%20COLMA1.mp3time=115800) Person 3:Généralement un enfant qui n'a pas atteint le tèrme des 9 mois.Mais bon, sous nos tropiques, ça à partir de la 28e semaine, mais dans les pays développés, on peut prendre jusqu'à la 22e semaine je crois.Voilà, d'après ce qu'on a eu dans les Cours. [00:02:23-5](http://localhost:2300/file=C:/Users/USER/Desktop/ENTRETIENS%20PREMATURITE/CSPS-COLMA1/FOCUS%20GROUPE%20COLMA1.mp3time=143500) |
| --- |

| 00:01:01-4 Interviewer: D'accord. Merci beaucoup pour la présentation. Donc, je pense que si tout le monde est prêt, nous pouvons commencer. Maintenant, la première question, comment est-ce que vous définissez la naissance prématurée ? 00:01:34-2  00:01:34-2 Person 1: La naissance prématurée, c'est un enfant qui n'est pas arrivé à son terme de 9 mois. 00:01:38-4  00:01:38-4 Person 2: La naissance prématurée est un enfant qui n'est pas arrivé à son terme de 9 mois, c'est un enfant qui est né avant les 9 mois. 00:01:47-4  00:01:47-4 Interviewer: Est-ce qu'il y a quelque chose à ajouter ou bien c'est bon ? 00:01:55-8  00:01:55-8 Person 3: Généralement, un enfant qui n'a pas atteint le terme des 9 mois. Mais bon, sous nos tropiques, ça à partir de la 28e semaine, mais dans les pays développés, on peut prendre jusqu'à la 22e semaine, je crois. Voilà, d'après ce qu'on a eu dans les cours. 00:02:23-5 |
| --- |

| **[00:01:01-4] Interviewer**: Okay. Thank you very much for the introductions. If everyone is ready, we can begin. The first question is, how do you define premature birth?  **[00:01:34-2] Person 1**: Premature birth is when a child has not reached its full term of 9 months.  **[00:01:38-4] Person 2**: It's a child who is born before completing the full term of 9 months.  **[00:01:47-4] Interviewer**: Is there anything else to add, or is that okay?  **[00:01:55-8] Person 3**: Generally, it refers to a child who hasn't reached the end of 9 months. However, in our region, it starts from the 28th week, but in developed countries, it can be considered from as early as the 22nd week, according to what we learned in our courses. |
| --- |

### Major of Colma1 health and social promotion center

| [00:01:51-2](http://localhost:2300/file=C:/Users/USER/Desktop/ENTRETIENS%20PREMATURITE/CSPS-COLMA1/MAJOR%20CSPS%20COLMA1.MP3time=111200) Interviewer: Après votre presentation, comment est-ce que vous définissez la naissance prématurée au sein de votre centre ? [00:01:58-9](http://localhost:2300/file=C:/Users/USER/Desktop/ENTRETIENS%20PREMATURITE/CSPS-COLMA1/MAJOR%20CSPS%20COLMA1.MP3time=118900)  [00:01:58-9](http://localhost:2300/file=C:/Users/USER/Desktop/ENTRETIENS%20PREMATURITE/CSPS-COLMA1/MAJOR%20CSPS%20COLMA1.MP3time=118900) Person 1: La naissance prématurée ici,Ce n'est pas seulement au niveau de notre centre, mais je dirais que nous sommes dans un cadre global, donc c'est une définition unique au niveau du système de santé, on dirait que un enfant est prématuré , donc lorsque l'accouchement intervient à partir de 7 mois de grossesse, voilà 7 mois de grossesse en général et on va aller jusqu'à aller voir les poids également donc le poids va être en dessous en général donc de 2 kilos 500 voilà. [00:02:38-8](http://localhost:2300/file=C:/Users/USER/Desktop/ENTRETIENS%20PREMATURITE/CSPS-COLMA1/MAJOR%20CSPS%20COLMA1.MP3time=158800) |
| --- |

| 00:01:51-2 Interviewer: Après votre présentation, comment est-ce que vous définissez la naissance prématurée au sein de votre centre ? 00:01:58-9  00:01:58-9 Person 1: La naissance prématurée ici, ce n'est pas seulement au niveau de notre centre, mais je dirais que nous sommes dans un cadre global, donc c'est une définition unique au niveau du système de santé. On dirait qu'un enfant est prématuré, donc lorsque l'accouchement intervient à partir de 7 mois de grossesse, voilà 7 mois de grossesse en général, et on va aller jusqu'à aller voir les poids également donc le poids va être en dessous en général donc de 2 kilos 500, voilà. 00:02:38-8 |
| --- |

| **[00:01:51-2] Interviewer**: Following your presentation, how do you define premature birth within your center?  **[00:01:58-9] Person 1**: In our center, and I believe this applies globally within the health system, premature birth is defined in a standard way. A child is considered premature when the birth occurs from 7 months of pregnancy, so that's generally around 7 months. We also consider the weight of the child; typically, a premature baby will weigh less than 2.5 kilograms. |
| --- |

### Maternity manager of Colma1 health and social promotion center

| [00:02:38-2](http://localhost:2300/file=C:/Users/USER/Desktop/ENTRETIENS%20PREMATURITE/CSPS-COLMA1/RESPONSABLE%20MATERNITE-COLMA1.mp3time=158200) Interviewer: Merci bien. Maintenant, on va aller directement à la question. Comment est- ce que vous définissez la naissance prématurée dans votre centre de santé? [00:02:46-3](http://localhost:2300/file=C:/Users/USER/Desktop/ENTRETIENS%20PREMATURITE/CSPS-COLMA1/RESPONSABLE%20MATERNITE-COLMA1.mp3time=166300)  [00:02:46-3](http://localhost:2300/file=C:/Users/USER/Desktop/ENTRETIENS%20PREMATURITE/CSPS-COLMA1/RESPONSABLE%20MATERNITE-COLMA1.mp3time=166300) Person 1: Oui, l'accouchement prématuré, c'est tout accouchement qui a lieu avant la 37ème semaine d'aménhorré. Plus précisément, les enfants nées entre la 28ème et la 36ème semaine, plus six jours. [00:03:03-2](http://localhost:2300/file=C:/Users/USER/Desktop/ENTRETIENS%20PREMATURITE/CSPS-COLMA1/RESPONSABLE%20MATERNITE-COLMA1.mp3time=183200) |
| --- |

| 00:02:38-2 Interviewer: Merci bien. Maintenant, on va aller directement à la question. Comment est-ce que vous définissez la naissance prématurée dans votre centre de santé ? 00:02:46-3  00:02:46-3 Person 1: Oui, l'accouchement prématuré, c'est tout accouchement qui a lieu avant la 37ème semaine d'aménorrhée. Plus précisément, les enfants nés entre la 28ème et la 36ème semaine, plus six jours. 00:03:03-2 |
| --- |

| **[00:02:38-2] Interviewer**: Thank you very much. Now let's move straight to the question. How do you define premature birth in your health center?  **[00:02:46-3] Person 1**: Yes, in our health center, premature birth is defined as any birth that occurs before the 37th week of amenorrhea. Specifically, this includes children born between the 28th week and the 36th week, plus six days. |
| --- |

### Focus group of Farakan health and social promotion center

| [00:02:52-6](http://localhost:2300/file=C:/Users/USER/Desktop/ENTRETIENS%20PREMATURITE/CSPS_FARAKAN/FOCUS_GROUPE-CSPS_FARAKAN.MP3time=172600) Interviewer: Maintenant la question, comment définissez vous la naissance prématurée au sein de votre centre de santé ? , la personne qui a un élément de réponse peut lever la main et puis répondre si les autres aussi ont des informations supplémentaires, elles peuvent attendre la fin de l'intervention. Et puis ajouter.Comment est-ce que vous définissez la naissance prématurée au sein de votre CSPS? [00:03:40-0](http://localhost:2300/file=C:/Users/USER/Desktop/ENTRETIENS%20PREMATURITE/CSPS_FARAKAN/FOCUS_GROUPE-CSPS_FARAKAN.MP3time=220000)  [00:03:40-0](http://localhost:2300/file=C:/Users/USER/Desktop/ENTRETIENS%20PREMATURITE/CSPS_FARAKAN/FOCUS_GROUPE-CSPS_FARAKAN.MP3time=220000) Person 1: Une naissance prématurée, c'est une naissance dont la femme a accouché avant les 9 mois. [00:03:49-7](http://localhost:2300/file=C:/Users/USER/Desktop/ENTRETIENS%20PREMATURITE/CSPS_FARAKAN/FOCUS_GROUPE-CSPS_FARAKAN.MP3time=229700)  [00:03:49-7](http://localhost:2300/file=C:/Users/USER/Desktop/ENTRETIENS%20PREMATURITE/CSPS_FARAKAN/FOCUS_GROUPE-CSPS_FARAKAN.MP3time=229700) Interviewer: Est ce qu'il y a quelque chose d'autre à ajouter? [00:03:52-2](http://localhost:2300/file=C:/Users/USER/Desktop/ENTRETIENS%20PREMATURITE/CSPS_FARAKAN/FOCUS_GROUPE-CSPS_FARAKAN.MP3time=232200)  [00:03:52-2](http://localhost:2300/file=C:/Users/USER/Desktop/ENTRETIENS%20PREMATURITE/CSPS_FARAKAN/FOCUS_GROUPE-CSPS_FARAKAN.MP3time=232200) Person 2: Elles vont compléter.Avant 32 semaines. [00:04:01-5](http://localhost:2300/file=C:/Users/USER/Desktop/ENTRETIENS%20PREMATURITE/CSPS_FARAKAN/FOCUS_GROUPE-CSPS_FARAKAN.MP3time=241500)  [00:04:01-5](http://localhost:2300/file=C:/Users/USER/Desktop/ENTRETIENS%20PREMATURITE/CSPS_FARAKAN/FOCUS_GROUPE-CSPS_FARAKAN.MP3time=241500) Person 5: Donc, avant 32 semaines. [00:04:03-7](http://localhost:2300/file=C:/Users/USER/Desktop/ENTRETIENS%20PREMATURITE/CSPS_FARAKAN/FOCUS_GROUPE-CSPS_FARAKAN.MP3time=243700)  [00:04:03-7](http://localhost:2300/file=C:/Users/USER/Desktop/ENTRETIENS%20PREMATURITE/CSPS_FARAKAN/FOCUS_GROUPE-CSPS_FARAKAN.MP3time=243700) Person 4: c'est avant le terme. [00:04:07-6](http://localhost:2300/file=C:/Users/USER/Desktop/ENTRETIENS%20PREMATURITE/CSPS_FARAKAN/FOCUS_GROUPE-CSPS_FARAKAN.MP3time=247600)  [00:04:07-6](http://localhost:2300/file=C:/Users/USER/Desktop/ENTRETIENS%20PREMATURITE/CSPS_FARAKAN/FOCUS_GROUPE-CSPS_FARAKAN.MP3time=247600) Person 7: C'est un bébé de faible poids. [00:04:17-3](http://localhost:2300/file=C:/Users/USER/Desktop/ENTRETIENS%20PREMATURITE/CSPS_FARAKAN/FOCUS_GROUPE-CSPS_FARAKAN.MP3time=257300) |
| --- |

| 00:02:52-6 Interviewer: Maintenant la question, comment définissez-vous la naissance prématurée au sein de votre centre de santé ? La personne qui a un élément de réponse peut lever la main et puis répondre si les autres aussi ont des informations supplémentaires, elles peuvent attendre la fin de l'intervention. Et puis ajouter. Comment est-ce que vous définissez la naissance prématurée au sein de votre CSPS ? 00:03:40-0  00:03:40-0 Person 1: Une naissance prématurée, c'est une naissance dont la femme a accouché avant les 9 mois. 00:03:49-7  00:03:49-7 Interviewer: Est-ce qu'il y a quelque chose d'autre à ajouter ? 00:03:52-2  00:03:52-2 Person 2: Elles vont compléter. Avant 32 semaines. 00:04:01-5  00:04:01-5 Person 5: Donc, avant 32 semaines. 00:04:03-7  00:04:03-7 Person 4: C'est avant le terme. 00:04:07-6  00:04:07-6 Person 7: C'est un bébé de faible poids. 00:04:17-3 |
| --- |

| **[00:02:52-6] Interviewer**: Now for the question, how do you define premature birth within your CSPS? Anyone who has an answer can raise their hand and respond. Others who have additional information can wait until the end of the response and then add their input. So, how is premature birth defined within your CSPS?  **[00:03:40-0] Person 1**: A premature birth is a birth where the woman gave birth before reaching 9 months.  **[00:03:49-7] Interviewer**: Is there anything else to add?  **[00:03:52-2] Person 2**: They will complete. It's before 32 weeks.  **[00:04:01-5] Person 5**: So, before 32 weeks.  **[00:04:03-7] Person 4**: It's before the end.  **[00:04:07-6] Person 7**: And it's typically a low-weight baby. |
| --- |

### Major of Farakan health and social promotion center

| [00:02:05-1](http://localhost:2300/file=C:/Users/USER/Desktop/ENTRETIENS%20PREMATURITE/CSPS_FARAKAN/MAJOR%20FARAKAN.MP3time=125100) Interviewer: Nous commençons donc la première question. Quelle definition vous donnez à la naissance prématurée au sein de votre centre de santé? [00:02:14-5](http://localhost:2300/file=C:/Users/USER/Desktop/ENTRETIENS%20PREMATURITE/CSPS_FARAKAN/MAJOR%20FARAKAN.MP3time=134500)  [00:02:14-5](http://localhost:2300/file=C:/Users/USER/Desktop/ENTRETIENS%20PREMATURITE/CSPS_FARAKAN/MAJOR%20FARAKAN.MP3time=134500) Person 1: C'est un enfant qui est né avant terme c'est à dire avant les 9 mois, il ya le poids aussi. On dit moins de 2kg500 mais il y a des enfants aussi qui ont moins de 2kg500 qui sont nés à terme [00:02:35-4](http://localhost:2300/file=C:/Users/USER/Desktop/ENTRETIENS%20PREMATURITE/CSPS_FARAKAN/MAJOR%20FARAKAN.MP3time=155400)  [00:02:35-4](http://localhost:2300/file=C:/Users/USER/Desktop/ENTRETIENS%20PREMATURITE/CSPS_FARAKAN/MAJOR%20FARAKAN.MP3time=155400) Interviewer: Est ce que ses enfants vous les classés dans la categorie des prématurés? [00:02:37-7](http://localhost:2300/file=C:/Users/USER/Desktop/ENTRETIENS%20PREMATURITE/CSPS_FARAKAN/MAJOR%20FARAKAN.MP3time=157700)  [00:02:37-7](http://localhost:2300/file=C:/Users/USER/Desktop/ENTRETIENS%20PREMATURITE/CSPS_FARAKAN/MAJOR%20FARAKAN.MP3time=157700) Person 1: Non non. Ils peuvent avoir moins de 2kg500 mais ils sont nés à terme, dans les delais requis. [00:02:46-2](http://localhost:2300/file=C:/Users/USER/Desktop/ENTRETIENS%20PREMATURITE/CSPS_FARAKAN/MAJOR%20FARAKAN.MP3time=166200) |
| --- |

| 00:02:05-1 Interviewer: Nous commençons donc la première question. Quelle définition vous donnez à la naissance prématurée au sein de votre centre de santé ? 00:02:14-5  00:02:14-5 Person 1: C'est un enfant qui est né avant terme, c'est-à-dire avant les 9 mois, il y a le poids aussi. On dit moins de 2kg500 mais il y a des enfants aussi qui ont moins de 2kg500 qui sont nés à terme. 00:02:35-4  00:02:35-4 Interviewer: Est-ce que ces enfants vous les classez dans la catégorie des prématurés ? 00:02:37-7  00:02:37-7 Person 1: Non, non. Ils peuvent avoir moins de 2kg500 mais ils sont nés à terme, dans les délais requis. 00:02:46-2 |
| --- |

| **[00:02:05-1] Interviewer**: Let's start with the first question. What definition do you give to premature birth within your health center?  **[00:02:14-5] Person 1**: It's a child born prematurely, which means before 9 months. There's also the aspect of weight. We often say less than 2.5 kilograms, but there are also children who weigh less than 2.5 kilograms and are born at full term.  **[00:02:35-4] Interviewer**: So, do you classify these lower-weight children as premature?  **[00:02:37-7] Person 1**: No, not necessarily. They may weigh less than 2.5 kilograms, but if they were born at full term, within the required time frame, they are not considered premature. |
| --- |

### Maternity manager of Farakan health and social promotion center

| [00:02:00-6](http://localhost:2300/file=C:/Users/USER/Desktop/ENTRETIENS%20PREMATURITE/CSPS_FARAKAN/RESPONSABLE%20MATERNITE%20FARAKAN.MP3time=120600) Interviewer: ok. maintenant que vous vous êtes présenté, comment définissez vous la naissance prématurée dans votre centre de santé? [00:02:11-1](http://localhost:2300/file=C:/Users/USER/Desktop/ENTRETIENS%20PREMATURITE/CSPS_FARAKAN/RESPONSABLE%20MATERNITE%20FARAKAN.MP3time=131100)  [00:02:11-1](http://localhost:2300/file=C:/Users/USER/Desktop/ENTRETIENS%20PREMATURITE/CSPS_FARAKAN/RESPONSABLE%20MATERNITE%20FARAKAN.MP3time=131100) Person 1: En tout cas, c'est c'est tout c'est un accouchement avant terme,quand on parle de terme, la prématurité, c'est un accouchement entre 28 et 37 semaines comme ça. Moins de 37 semaines, on peut mettre ça dans la prématurité, [00:02:31-9](http://localhost:2300/file=C:/Users/USER/Desktop/ENTRETIENS%20PREMATURITE/CSPS_FARAKAN/RESPONSABLE%20MATERNITE%20FARAKAN.MP3time=151900) |
| --- |

| 00:02:00-6 Interviewer: Ok. Maintenant que vous vous êtes présenté, comment définissez-vous la naissance prématurée dans votre centre de santé ? 00:02:11-1  00:02:11-1 Person 1: En tout cas, c'est un accouchement avant terme, quand on parle de terme, la prématurité, c'est un accouchement entre 28 et 37 semaines. Moins de 37 semaines, on peut mettre ça dans la prématurité. 00:02:31-9 |
| --- |

| **[00:02:00-6] Interviewer**: Ok. Now that everyone has introduced themselves, how do you define premature birth in your health center?  **[00:02:11-1] Person 1**: Premature birth, in our understanding, refers to any preterm birth. We define prematurity as a birth that occurs between 28 and 37 weeks of pregnancy. So, any birth that happens before 37 weeks is classified as premature. |
| --- |

# Measurement of preterm birth

### Focus group of Accart-ville urban medical center

| [00:01:56-0](http://localhost:2300/file=C:/Users/USER/Desktop/ENTRETIENS) Interviewer: maintenant que vous avez défini la naissance prématurée comment est ce qu'elle est mesurée au sein de votre centre de santé? [00:02:02-5](http://localhost:2300/file=C:/Users/USER)  [00:02:02-5](http://localhost:2300/file=C:/Users/USER/Desktop/ENTRETIENS) Person 6: mesurer? [00:02:03-8](http://localhost:2300/file=C:/Users/USER/Desktop/ENTRETIENS%20PREMATURITE/CSPS-ACCART-VILLE/FOCUS%20GROUPE%20ACCART-VILLE-BON.mp3time=123800)  [00:02:03-8](http://localhost:2300/file=C:/Users/USER/Deskto) Interviewer: quels sont les paramètres qui vous permettent de reconnaitre la naissance prématurée? [00:02:06-5](http://localhost:2300/file=C:/Users/USER/Des)  [00:02:06-5](http://localhost:2300/file=C:/Users/USER/Desktop/ENTRETIENS%20PREMATURITE/CSPS-ACCART-VILLE/FOCUS%20GROUPE%20ACCART-VILLE-BON.mp3time=126500) Person 1: d'accord, les paramètres que c'est une naissance prématurée? bon, après la naissance de l'enfant ou bien au cour du travail? [00:02:20-1](http://localhost:2300/file=C:/Users/USER/Desktop/ENTRETIENS%20PREMATURITE/CSPS-ACCART-VILLE/FOCUS%20GROUPE%20ACCART-VILLE-BON.mp3time=140100)  [00:02:20-1](http://localhost:2300/file=C:/Users/USER/De) Interviewer: au cours du travail [00:02:20-5](http://localhost:2300/file=C:/Users/USER/Desktop/ENTRETIENS)  [00:02:20-5](http://localhost:2300/file=C:/Users/USER/Desktop/ENTRETIENS%20PREMATURITE/CSPS-ACCART-VILLE/FOCUS%20GROUPE%20ACCART-VILLE-BON.mp3time=140500) Person 1: au cours du travail? nous mesurons la hauteur utérine, on peut constater que la hauteur utérine ne vaut pas les normes. Nous demandons aussi la date des dernières règles de la patiente et aussi on peut demander si elle a fait une échographie, nous pouvons regarder cet échographie. [00:02:44-6](%22htt)  [00:02:44-6](http://localhost:2300/file=C:/Users/USER/Desktop/ENTRETIENS)Interviewer: lorsque vous dites que la hauteur utérine n'est pas au niveau de la norme, la norme c'est à quel niveau? [00:02:51-1](http://localhost:2300/file=C:/Users/USER/Desktop/ENTRETIENS)  [00:02:51-1](http://localhost:2300/file=C:/Users/USER/Desktop/ENTRETIENS%20PREMATURITE/CSPS-ACCART-VILLE/FOCUS%20GROUPE%20ACCART-VILLE-BON.mp3time=171100)Person 1: oui, quand la grossesse est à terme, cela part souvent en fonction de la patiente,à 32cm en allant. quand on mesure la hauteur uterine souvent on ajoute 3cm au 3ème trimestre pour avoir l'âge gestationnel à peu prêt [00:03:15-9](http://localhost:2300/file=C:/Users/U) |
| --- |

| 00:01:56-0 Interviewer: Maintenant que vous avez défini la naissance prématurée, comment est-ce qu'elle est mesurée au sein de votre centre de santé? 00:02:02-5  00:02:02-5 Person 6: Mesurer? 00:02:03-8  00:02:03-8 Interviewer: Quels sont les paramètres qui vous permettent de reconnaître la naissance prématurée? 00:02:06-5  00:02:06-5 Person 1: D'accord, les paramètres que c'est une naissance prématurée? Bon, après la naissance de l'enfant ou bien au cours du travail? 00:02:20-1  00:02:20-1 Interviewer: Au cours du travail. 00:02:20-5  00:02:20-5 Person 1: Au cours du travail? Nous mesurons la hauteur utérine, on peut constater que la hauteur utérine ne correspond pas aux normes. Nous demandons aussi la date des dernières règles de la patiente et aussi on peut demander si elle a fait une échographie, nous pouvons regarder cette échographie. 00:02:44-6  00:02:44-6 Interviewer: Lorsque vous dites que la hauteur utérine n'est pas au niveau de la norme, la norme c'est à quel niveau? 00:02:51-1  00:02:51-1 Person 1: Oui, quand la grossesse est à terme, cela varie souvent en fonction de la patiente, à 32 cm en moyenne. Quand on mesure la hauteur utérine, souvent on ajoute 3 cm au 3ème trimestre pour avoir l'âge gestationnel à peu près. 00:03:15-9 |
| --- |

| **Interviewer (00:01:56-0):** "How do you measure or recognize premature birth in your health center?"  **Person 6 (00:02:02-5):** "Measure?"  **Interviewer (00:02:03-8):** "What are the parameters used to identify premature birth?"  **Person 1 (00:02:06-5):** "Are you asking about the parameters to determine prematurity during labor or after the child's birth?"  **Interviewer (00:02:20-1):** "During labor."  **Person 1 (00:02:20-5):** "During labor, we measure the fundal height, which should match certain standards. We also consider the date of the patient's last period and review ultrasound results if available."  **Interviewer (00:02:44-6):** "What is the standard level for fundal height?"  **Person 1 (00:02:51-1):** "In a full-term pregnancy, the fundal height typically starts at around 32 cm. In the third trimester, we usually add 3 cm to the measurement to approximate the gestational age." |
| --- |

### Manager of Accart-ville urban medical center

| [00:02:26-4](http://localhost:2300/file=C:/Users/USER/Desktop/ENTRETIENS%20PREMATURITE/CSPS-ACCART-VILLE/RESPONSABLE%20CMU%20ACCART-VILLE.MP3time=146400) Interviewer: Ok, maintenant que vous avez défini la naissance prématurée. Comment est-ce que cela est mesuré concrètement au sein de votre centre de santé ? [00:02:35-8](http://localhost:2300/file=C:/Users/USER/Desktop/ENTRETIENS%20PREMATURITE/CSPS-ACCART-VILLE/RESPONSABLE%20CMU%20ACCART-VILLE.MP3time=155800)  [00:02:35-8](http://localhost:2300/file=C:/Users/USER/Desktop/ENTRETIENS%20PREMATURITE/CSPS-ACCART-VILLE/RESPONSABLE%20CMU%20ACCART-VILLE.MP3time=155800) Person 1: Généralement on se base sur les échographies de datation ou sur l'âge chronologique. Parfois, bon, généralement, il y a des enfants qui nous parviennent aussi par rapport aux signes de prématurité si on ne voit pas. puisque generalement il y a des mamans qui n'arrivent pas à nous situer par rapport à l'âge. Si on a des signes de prématurité par rapport au poids et les signes qu'on trouve physiquement, on peut définir si c'est un prématuré ou pas. [00:03:03-3](http://localhost:2300/file=C:/Users/USER/Desktop/ENTRETIENS%20PREMATURITE/CSPS-ACCART-VILLE/RESPONSABLE%20CMU%20ACCART-VILLE.MP3time=183300) |
| --- |

| 00:02:26-4 Interviewer: Ok, maintenant que vous avez défini la naissance prématurée. Comment est-ce que cela est mesuré concrètement au sein de votre centre de santé ? 00:02:35-8  00:02:35-8 Person 1: Généralement on se base sur les échographies de datation ou sur l'âge chronologique. Parfois, il y a des enfants qui nous parviennent aussi par rapport aux signes de prématurité, si on ne voit pas, puisque généralement il y a des mamans qui n'arrivent pas à nous situer par rapport à l'âge. Si on a des signes de prématurité par rapport au poids et les signes qu'on trouve physiquement, on peut définir si c'est un prématuré ou pas. 00:03:03-3 |
| --- |

| **[00:02:26-4] Interviewer**: Now that you've defined premature birth, how is this determined in practice within your health center?  **[00:02:35-8] Person 1**: Generally, we rely on dating ultrasounds or the chronological age of the pregnancy. Sometimes, we identify premature children based on signs of prematurity, especially if mothers are unable to provide accurate age information. We assess whether a birth is premature by considering the baby's weight and other physical signs of prematurity. |
| --- |

### Maternity manager of Accart-ville urban medical center

| [00:01:53-5](http://localhost:2300/file=C:/Users/USER/Desktop/ENTRETIENS%20PREMATURITE/CSPS-ACCART-VILLE/RESPONSABLE%20MATERNITE%20CMU%20ACCART-VILLE.MP3time=113500) Interviewer: ok maintenant comment est ce que vous mesurer concretement la naissance prematuré au sein de votre structure de santé? quels sont les parametres qui vous permettent de reconnaitre une naissance prematurée au sein de votre structure de santé? [00:02:06-6](http://localhost:2300/file=C:/Users/USER/Desktop/ENTRETIENS%20PREMATURITE/CSPS-ACCART-VILLE/RESPONSABLE%20MATERNITE%20CMU%20ACCART-VILLE.MP3time=126600)  [00:02:06-6](http://localhost:2300/file=C:/Users/USER/Desktop/ENTRETIENS%20PREMATURITE/CSPS-ACCART-VILLE/RESPONSABLE%20MATERNITE%20CMU%20ACCART-VILLE.MP3time=126600) Person 1: donc, on part sur la base de l'âge gestationnel, en plus de ça nous recherchons les citères physiologiques de la prematurité qui sont entre autre le cartilage au niveau des pavins de l'oreille, l'abscence des cartilages il ya aussi la peau au niveau de l'abdomen qui est fine avec la presence de veinine de petites veines donc il y a egalement au niveau des organes genitaux on reconnait egalement à travers la non decente des testicules pour les nouveaux nées de sexe masculin et l'hypertrophie du clitoris aussi chez les nouveaux nées prématurés de sexe feminins donc en plus de cela egalement donc nous pouvons parler du poids donc et de la taille qui sont inferieurs generalement au poids normal d'un nouveau née à terme. [00:03:17-2](http://localhost:2300/file=C:/Users/USER/Desktop/ENTRETIENS%20PREMATURITE/CSPS-ACCART-VILLE/RESPONSABLE%20MATERNITE%20CMU%20ACCART-VILLE.MP3time=197200) |
| --- |

| 00:01:53-5 Interviewer: Ok, maintenant comment est-ce que vous mesurez concrètement la naissance prématurée au sein de votre structure de santé? Quels sont les paramètres qui vous permettent de reconnaître une naissance prématurée au sein de votre structure de santé? 00:02:06-6  00:02:06-6 Personne 1: Donc, on part sur la base de l'âge gestationnel. En plus de ça, nous recherchons les critères physiologiques de la prématurité, qui sont, entre autres, le cartilage au niveau des pavillons de l'oreille, l'absence de cartilages. Il y a aussi la peau au niveau de l'abdomen qui est fine, avec la présence de veinules de petites veines. Donc, il y a également au niveau des organes génitaux, on reconnaît également à travers la non-descente des testicules pour les nouveaux-nés de sexe masculin, et l'hypertrophie du clitoris aussi chez les nouveaux-nés prématurés de sexe féminin. Donc en plus de cela également, nous pouvons parler du poids et de la taille, qui sont inférieurs généralement au poids normal d'un nouveau-né à terme. 00:03:17-2 |
| --- |

| **[00:01:53-5] Interviewer**: Ok, how do you measure and identify premature birth within your health facility? What are the key parameters used for recognition?  **[00:02:06-6] Person 1**: We primarily use gestational age as a starting point. Additionally, we assess various physiological criteria indicative of prematurity. These include the absence of cartilage in the pinnae of the ears, thin abdominal skin with visible small veins, and specific genital characteristics. For male newborns, this involves the non-descent of the testicles, and for female newborns, we look for clitoral hypertrophy. Besides these, we also consider the weight and height, which are typically lower than those of a full-term newborn. |
| --- |

### Focus group of Do medical center with surgical branch

| [00:03:31-9](http://localhost:2300/file=C:/Users/USER/Desktop/ENTRETIENS%20PREMATURITE/CMA-DO/FOCUS%20GROUPE%20CMA-DO.mp3time=211900) Interviewer: Nous allons continuer avec la deuxième question. Comment est-ce que la naissance prématurée est mesurée au sein du CMA de DÔ? [00:03:45-0](http://localhost:2300/file=C:/Users/USER/Desktop/ENTRETIENS%20PREMATURITE/CMA-DO/FOCUS%20GROUPE%20CMA-DO.mp3time=225000)  [00:03:45-0](http://localhost:2300/file=C:/Users/USER/Desktop/ENTRETIENS%20PREMATURITE/CMA-DO/FOCUS%20GROUPE%20CMA-DO.mp3time=225000) Person 2: Il y a le faible poids de naissance. [00:03:46-8](http://localhost:2300/file=C:/Users/USER/Desktop/ENTRETIENS%20PREMATURITE/CMA-DO/FOCUS%20GROUPE%20CMA-DO.mp3time=226800)  [00:03:46-8](http://localhost:2300/file=C:/Users/USER/Desktop/ENTRETIENS%20PREMATURITE/CMA-DO/FOCUS%20GROUPE%20CMA-DO.mp3time=226800) Interviewer: Comment est ce que la naissance prématurée est mesurée au CMA de DÔ? Quels sont les paramètres qui vous permettent de reconnaître une naissance prématurée? [00:03:58-3](http://localhost:2300/file=C:/Users/USER/Desktop/ENTRETIENS%20PREMATURITE/CMA-DO/FOCUS%20GROUPE%20CMA-DO.mp3time=238300)  [00:03:58-3](http://localhost:2300/file=C:/Users/USER/Desktop/ENTRETIENS%20PREMATURITE/CMA-DO/FOCUS%20GROUPE%20CMA-DO.mp3time=238300) Person 2: Il y a le faible poids de naissance, Compris entre moins de 2500. Il y a aussi que la coloration du bébé peut nous aider à définir. L'enfant n'est pas totalement rouge. Un peu rouge. Aussi il y a... Les petits lèvres. [00:04:36-1](http://localhost:2300/file=C:/Users/USER/Desktop/ENTRETIENS%20PREMATURITE/CMA-DO/FOCUS%20GROUPE%20CMA-DO.mp3time=276100)  [00:04:36-1](http://localhost:2300/file=C:/Users/USER/Desktop/ENTRETIENS%20PREMATURITE/CMA-DO/FOCUS%20GROUPE%20CMA-DO.mp3time=276100) Person 6: Il y a l'âge gestationnel. Il y a l'âge gestationnel pour connaître une naissance prématurée. Maintenant, à l'accouchement, on peut voire une absence de vermix de casoesa. Et puis, voilà. Le poids qui y sièd, le poids ne qualifie pas tout, Parce que quand vous êtes en face de diabetique, l'enfant peut être prématuré avec un poids de 3000. C'est tout l'âge gestationnel avec un mal-absent de vermix casoesa. Au niveau des bourses aussi. Si c'est un garçon, on voit que les bourses ne sont pas séants. Si c'est une fille aussi, les petites lèvres sont plus séances que les grands lèvres. [00:05:21-1](http://localhost:2300/file=C:/Users/USER/Desktop/ENTRETIENS%20PREMATURITE/CMA-DO/FOCUS%20GROUPE%20CMA-DO.mp3time=321100)  [00:05:21-1](http://localhost:2300/file=C:/Users/USER/Desktop/ENTRETIENS%20PREMATURITE/CMA-DO/FOCUS%20GROUPE%20CMA-DO.mp3time=321100) Interviewer: Est-ce qu'il y a quelque chose qu'on peut ajouter? [00:05:23-2](http://localhost:2300/file=C:/Users/USER/Desktop/ENTRETIENS%20PREMATURITE/CMA-DO/FOCUS%20GROUPE%20CMA-DO.mp3time=323200)  [00:05:23-2](http://localhost:2300/file=C:/Users/USER/Desktop/ENTRETIENS%20PREMATURITE/CMA-DO/FOCUS%20GROUPE%20CMA-DO.mp3time=323200) Person 8: On peut ajouter aussi le périmètre crânien, le périmètre thoracique, la taille. Les paramètres du bébé, l'aspect même du bébé quand on le regarde, on peut savoir. [00:05:37-8](http://localhost:2300/file=C:/Users/USER/Desktop/ENTRETIENS%20PREMATURITE/CMA-DO/FOCUS%20GROUPE%20CMA-DO.mp3time=337800) |
| --- |

| 00:03:31-9 Interviewer: Nous allons continuer avec la deuxième question. Comment est-ce que la naissance prématurée est mesurée au sein du CMA de Dô ? 00:03:45-0  00:03:45-0 Person 2: Il y a le faible poids de naissance. 00:03:46-8  00:03:46-8 Interviewer: Comment est-ce que la naissance prématurée est mesurée au CMA de Dô ? Quels sont les paramètres qui vous permettent de reconnaître une naissance prématurée ? 00:03:58-3  00:03:58-3 Person 2: Il y a le faible poids de naissance, compris entre moins de 2500. Il y a aussi que la coloration du bébé peut nous aider à définir. L'enfant n'est pas totalement rouge, un peu rouge. Aussi, il y a ... les petites lèvres. 00:04:36-1  00:04:36-1 Person 6: Il y a l'âge gestationnel. Il y a l'âge gestationnel pour connaître une naissance prématurée. Maintenant, à l'accouchement, on peut voir une absence de vernix caseosa. Et puis, voilà. Le poids qui y est, le poids ne qualifie pas tout, parce que quand vous êtes en face de diabétique, l'enfant peut être prématuré avec un poids de 3000. C'est tout l'âge gestationnel avec un mal-absent de vernix caseosa. Au niveau des bourses aussi. Si c'est un garçon, on voit que les bourses ne sont pas descendues. Si c'est une fille aussi, les petites lèvres sont plus saillantes que les grandes lèvres. 00:05:21-1  00:05:21-1 Interviewer: Est-ce qu'il y a quelque chose qu'on peut ajouter ? 00:05:23-2  00:05:23-2 Person 8: On peut ajouter aussi le périmètre crânien, le périmètre thoracique, la taille. Les paramètres du bébé, l'aspect même du bébé quand on le regarde, on peut savoir. 00:05:37-8 |
| --- |

| **[00:03:31-9] Interviewer**: Let's continue with the second question. How is premature birth measured within the CMA de Dô?  **[00:03:45-0] Person 2**: One indicator is low birth weight.  **[00:03:46-8] Interviewer**: How exactly is premature birth measured at the CMA de Dô? What parameters help you recognize a premature birth?  **[00:03:58-3] Person 2**: There are several indicators. Low birth weight, typically less than 2,500 grams, is a key factor. The baby's coloration can also be a clue; a premature baby often has a slightly red appearance. Additionally, the development of the genitalia, such as the labia minora in girls, can be indicative.  **[00:04:36-1] Person 6**: Gestational age is another important factor. At birth, the absence of vernix caseosa can be a sign. The baby's weight alone doesn't always give a complete picture; for instance, a diabetic mother might have a premature baby that weighs 3,000 grams. We look at the overall gestational age along with the absence of vernix caseosa. For boys, undescended scrotal sacs can be a sign, and for girls, the prominence of the labia minora over the labia majora.  **[00:05:21-1] Interviewer**: Is there anything else we can add?  **[00:05:23-2] Person 8**: We can also consider the baby's head circumference, thoracic circumference, and length. The overall appearance of the baby often gives us a clear indication of prematurity. |
| --- |

### Gynecologist 1

| [00:02:43-9](http://localhost:2300/file=C:/Users/USER/Desktop/ENTRETIENS%20PREMATURITE/CMA-DO/GENYCOLOGUE%2001.mp3time=163900) Interviewer: Maintenant que vous avez défini la naissance prématurée,concrètement comment est-ce que vous la mesurez au sein du CMA ? [00:02:52-1](http://localhost:2300/file=C:/Users/USER/Desktop/ENTRETIENS%20PREMATURITE/CMA-DO/GENYCOLOGUE%2001.mp3time=172100)  [00:02:52-1](http://localhost:2300/file=C:/Users/USER/Desktop/ENTRETIENS%20PREMATURITE/CMA-DO/GENYCOLOGUE%2001.mp3time=172100) Person 1: Bon comme je l'ai dit, à l'accouchement, D'abord quand les femmes en fonction de ce qu'elles ont, parce que souvent , elles ont des échographies qui donnent l'âge de la grossesse, dans quelques rares cas, elles connaissent la date des dernières règles, donc on a l'âge de la grossesse et en fonction de cela on peut penser que c'est un prématuré et puis le classer. L'autre citère aussi à la naissance, quand l'enfant sort, en fonction de son poids,comme je l'ai dit, généralement quand le poids est autour de 2 kg, on regarde les critères morphologiques. On regarde le mamelon, on regarde les lobules de l'oreille. Et si on pense que cet enfant qui n'a pas un poids normal est prématuré, on le classe parmi les prématurés. [00:03:35-0](http://localhost:2300/file=C:/Users/USER/Desktop/ENTRETIENS%20PREMATURITE/CMA-DO/GENYCOLOGUE%2001.mp3time=215000) |
| --- |

| 00:02:43-9 Interviewer: Maintenant que vous avez défini la naissance prématurée, concrètement comment est-ce que vous la mesurez au sein du CMA ? 00:02:52-1  00:02:52-1 Person 1: Bon, comme je l'ai dit, à l'accouchement, d'abord quand les femmes, en fonction de ce qu'elles ont, parce que souvent, elles ont des échographies qui donnent l'âge de la grossesse, dans quelques rares cas, elles connaissent la date des dernières règles, donc on a l'âge de la grossesse et en fonction de cela on peut penser que c'est un prématuré et puis le classer. L'autre critère aussi à la naissance, quand l'enfant sort, en fonction de son poids, comme je l'ai dit, généralement quand le poids est autour de 2 kg, on regarde les critères morphologiques. On regarde le mamelon, on regarde les lobules de l'oreille. Et si on pense que cet enfant qui n'a pas un poids normal est prématuré, on le classe parmi les prématurés. 00:03:35-0 |
| --- |

| **[00:02:43-9] Interviewer**: Now that you have defined premature birth, how do you concretely measure it within the CMA?  **[00:02:52-1] Person 1**: As I mentioned, at childbirth, we first consider the information available from the mother. Often, they have ultrasounds that give the gestational age. In some cases, they know the date of their last menstrual period, so we can estimate the pregnancy age from that and determine if it's premature. Another criterion at birth is the baby's weight. Generally, when the weight is around 2 kg, we also look at morphological criteria, like the nipples and ear lobules. If we find that a child with below-normal weight exhibits these premature characteristics, we classify them as premature. |
| --- |

### Gynecologist 2

| [00:02:23-4](http://localhost:2300/file=C:/Users/USER/Desktop/ENTRETIENS%20PREMATURITE/CMA-DO/GENYCOLOGUE%2002.mp3time=143400) Interviewer: D'accord. Maintenant, comment est-ce que la naissance prématurée est mesurée au sein du CMA? [00:02:28-7](http://localhost:2300/file=C:/Users/USER/Desktop/ENTRETIENS%20PREMATURITE/CMA-DO/GENYCOLOGUE%2002.mp3time=148700)  [00:02:28-7](http://localhost:2300/file=C:/Users/USER/Desktop/ENTRETIENS%20PREMATURITE/CMA-DO/GENYCOLOGUE%2002.mp3time=148700) Person 1: Si on connaît l'âge gestationnel, ça veut dire que si on connaît la date des dernières règles ou bien si on a une échographie de datation qui permet de savoir que, la grossesse est à temps de semaines, déjà on est orienté par rapport à la naissance prématurée. Maintenant, si on ne connaît rien, si on n'a aucune information, ça sera de prendre la mesure de la hauteur utérine et ensuite d'examiner l'enfant à la naissance. Ce sont ces différents éléments pris ensemble qui nous permettent de dire si l'enfant est prématuré ou non. [00:03:00-1](http://localhost:2300/file=C:/Users/USER/Desktop/ENTRETIENS%20PREMATURITE/CMA-DO/GENYCOLOGUE%2002.mp3time=180100) |
| --- |

| 00:02:23-4 Interviewer: D'accord. Maintenant, comment est-ce que la naissance prématurée est mesurée au sein du CMA ? 00:02:28-7  00:02:28-7 Person 1: Si on connaît l'âge gestationnel, ça veut dire que si on connaît la date des dernières règles ou bien si on a une échographie de datation qui permet de savoir que la grossesse est à tant de semaines, déjà on est orienté par rapport à la naissance prématurée. Maintenant, si on ne connaît rien, si on n'a aucune information, ça sera de prendre la mesure de la hauteur utérine et ensuite d'examiner l'enfant à la naissance. Ce sont ces différents éléments pris ensemble qui nous permettent de dire si l'enfant est prématuré ou non. 00:03:00-1 |
| --- |

| **[00:02:23-4] Interviewer**: Okay. How is preterm birth measured within the CMA?  **[00:02:28-7] Person 1**: If we know the gestational age, either from the date of the last menstrual period or from a dating ultrasound, we can determine if the pregnancy is of a certain number of weeks and hence consider the likelihood of premature birth. In cases where we don’t have this information, we measure the fundal height and examine the child at birth. It's the combination of these elements that helps us conclude whether the child is premature. |
| --- |

### Chief physician of Do health district

| [00:03:18-1](http://localhost:2300/file=C:/Users/USER/Desktop/ENTRETIENS%20PREMATURITE/CMA-DO/MCD-DO.mp3time=198100) Interviewer: Maintenant que vous avez défini la prématurité, comment est-ce que cela est mesuré concrètement ici chez vous? Quels sont les paramètres qui vous permettent de connaître déjà une naissance prématurée? [00:03:32-9](http://localhost:2300/file=C:/Users/USER/Desktop/ENTRETIENS%20PREMATURITE/CMA-DO/MCD-DO.mp3time=212900)  [00:03:32-9](http://localhost:2300/file=C:/Users/USER/Desktop/ENTRETIENS%20PREMATURITE/CMA-DO/MCD-DO.mp3time=212900) Person 1: C'est ce que j'avais dit de part sa définition. Toute grossesse qui intervient avant les termes est une grossesse prématurée. Avant les 37 semaines d'aménorrhée, en tout cas, on conclu que c'est un accouchement prématuré. [00:03:52-2](http://localhost:2300/file=C:/Users/USER/Desktop/ENTRETIENS%20PREMATURITE/CMA-DO/MCD-DO.mp3time=232200) |
| --- |

| 00:03:18-1 Interviewer: Maintenant que vous avez défini la prématurité, comment est-ce que cela est mesuré concrètement ici chez vous ? Quels sont les paramètres qui vous permettent de connaître déjà une naissance prématurée ? 00:03:32-9  00:03:32-9 Person 1: C'est ce que j'avais dit de par sa définition. Toute grossesse qui intervient avant les termes est une grossesse prématurée. Avant les 37 semaines d'aménorrhée, en tout cas, on conclut que c'est un accouchement prématuré. 00:03:52-2 |
| --- |

| **[00:03:18-1] Interviewer**: Now that you have defined prematurity, how is it actually measured in practice here? What parameters do you use to determine a premature birth?  **[00:03:32-9] Person 1**: As I mentioned in its definition, any pregnancy that ends before full term is considered premature. Specifically, if a birth occurs before 37 weeks of amenorrhea, we conclude that it is a premature birth. |
| --- |

### Manager for maternity care unit of Do medical center with surgical branch

| [00:02:49-4](http://localhost:2300/file=C:/Users/USER/Desktop/ENTRETIENS%20PREMATURITE/CMA-DO/RESPONSABLE%20UNITE%20DE%20SOIN%20CMA-DO.mp3time=169400) Interviewer: Maintenant, comment est-ce que la naissance prématurée est mesurée au sein du CMA de DÔ? [00:02:57-1](http://localhost:2300/file=C:/Users/USER/Desktop/ENTRETIENS%20PREMATURITE/CMA-DO/RESPONSABLE%20UNITE%20DE%20SOIN%20CMA-DO.mp3time=177100)  [00:02:57-1](http://localhost:2300/file=C:/Users/USER/Desktop/ENTRETIENS%20PREMATURITE/CMA-DO/RESPONSABLE%20UNITE%20DE%20SOIN%20CMA-DO.mp3time=177100) Person 1: A travers d'avoir un examen, donc la mesure de la hauteur utérine peut guider. Ensuite également, la date des dernières règles qu'on recueille chez les femmes. Voilà, ça nous permet également de calculer la date probable d'accouchement. [00:03:15-9](http://localhost:2300/file=C:/Users/USER/Desktop/ENTRETIENS%20PREMATURITE/CMA-DO/RESPONSABLE%20UNITE%20DE%20SOIN%20CMA-DO.mp3time=195900) |
| --- |

| 00:02:49-4 Interviewer: Maintenant, comment est-ce que la naissance prématurée est mesurée au sein du CMA de Dô ? 00:02:57-1  00:02:57-1 Person 1: À travers d'avoir un examen, donc la mesure de la hauteur utérine peut guider. Ensuite également, la date des dernières règles qu'on recueille chez les femmes. Voilà, ça nous permet également de calculer la date probable d'accouchement. 00:03:15-9 |
| --- |

| **[00:02:49-4] Interviewer**: Now, how is premature birth measured within the CMA de Dô?  **[00:02:57-1] Person 1**: We measure it by conducting an examination. Measuring the fundal height can give us some guidance. We also consider the date of the woman's last menstrual period, which helps us calculate the probable date of delivery. |
| --- |

### Focus group of Colma1 health and social promotion center

| [00:02:23-5](http://localhost:2300/file=C:/Users/USER/Desktop/ENTRETIENS%20PREMATURITE/CSPS-COLMA1/FOCUS%20GROUPE%20COLMA1.mp3time=143500) Interviewer: ok maintenant que vous avez défini la naissance prématurée , comment est-ce que cette naissance prématurée est mesurée au sein du centre de santé ici ?Quels sont les critères qui vous permettent de determiner la naissance prématurée ici ? [00:02:42-7](http://localhost:2300/file=C:/Users/USER/Desktop/ENTRETIENS%20PREMATURITE/CSPS-COLMA1/FOCUS%20GROUPE%20COLMA1.mp3time=162700)  [00:02:42-7](http://localhost:2300/file=C:/Users/USER/Desktop/ENTRETIENS%20PREMATURITE/CSPS-COLMA1/FOCUS%20GROUPE%20COLMA1.mp3time=162700) Person 3: Bon, généralement pour les dames qui ont eu à faire des échographies, on peut déterminer l'accouchement prématurée à partir de l'échographie.Maintenant cliniquement à l'accouchement, il y a certains signes qui peut montrer que l'enfant est prématuré. [00:03:06-8](http://localhost:2300/file=C:/Users/USER/Desktop/ENTRETIENS%20PREMATURITE/CSPS-COLMA1/FOCUS%20GROUPE%20COLMA1.mp3time=186800)  [00:03:06-8](http://localhost:2300/file=C:/Users/USER/Desktop/ENTRETIENS%20PREMATURITE/CSPS-COLMA1/FOCUS%20GROUPE%20COLMA1.mp3time=186800) Interviewer: vous faites allusion à quoi, lorsque vous parlez de signes? [00:03:09-5](http://localhost:2300/file=C:/Users/USER/Desktop/ENTRETIENS%20PREMATURITE/CSPS-COLMA1/FOCUS%20GROUPE%20COLMA1.mp3time=189500)  [00:03:09-5](http://localhost:2300/file=C:/Users/USER/Desktop/ENTRETIENS%20PREMATURITE/CSPS-COLMA1/FOCUS%20GROUPE%20COLMA1.mp3time=189500) Person 3: Bon, on a, on a les cas de faible poids de naissance, il ya également la présence,Comment je vais dire ça ? Aidez-moi un peu,Ce qui recouvre le corps là, on appel ça comment? [00:03:30-7](http://localhost:2300/file=C:/Users/USER/Desktop/ENTRETIENS%20PREMATURITE/CSPS-COLMA1/FOCUS%20GROUPE%20COLMA1.mp3time=210700)  [00:03:30-7](http://localhost:2300/file=C:/Users/USER/Desktop/ENTRETIENS%20PREMATURITE/CSPS-COLMA1/FOCUS%20GROUPE%20COLMA1.mp3time=210700) Person 2: le vernix [00:03:30-4](http://localhost:2300/file=C:/Users/USER/Desktop/ENTRETIENS%20PREMATURITE/CSPS-COLMA1/FOCUS%20GROUPE%20COLMA1.mp3time=210400)  [00:03:30-4](http://localhost:2300/file=C:/Users/USER/Desktop/ENTRETIENS%20PREMATURITE/CSPS-COLMA1/FOCUS%20GROUPE%20COLMA1.mp3time=210400) Person 3:Voilà, généralement il y a l'absence de ce vernix souvent sur le prématuré. Maintenant il y a certains reflexes, il ne repond pas à certains reflexes, les reflexes de succion,on a les quatre points cardinaux. Il y a beaucoup de réflexes auxquels le prématuré ne répond pas. [00:04:02-8](http://localhost:2300/file=C:/Users/USER/Desktop/ENTRETIENS%20PREMATURITE/CSPS-COLMA1/FOCUS%20GROUPE%20COLMA1.mp3time=242800)  [00:04:02-8](http://localhost:2300/file=C:/Users/USER/Desktop/ENTRETIENS%20PREMATURITE/CSPS-COLMA1/FOCUS%20GROUPE%20COLMA1.mp3time=242800) Interviewer: Est-ce qu'il y a quelque chose d'autres qu'on peut ajouter? [00:04:05-7](http://localhost:2300/file=C:/Users/USER/Desktop/ENTRETIENS%20PREMATURITE/CSPS-COLMA1/FOCUS%20GROUPE%20COLMA1.mp3time=245700)  [00:04:05-7](http://localhost:2300/file=C:/Users/USER/Desktop/ENTRETIENS%20PREMATURITE/CSPS-COLMA1/FOCUS%20GROUPE%20COLMA1.mp3time=245700) Person 4: Je voulais ajouter quand la femme entre même en travail, la HU voilà,on voit que la hauteur utérine n'est pas adaptée et cela nous previent dabord que ce n'est pas arrivé à terme, Voilà comme elle a dit, il y a certains réflexes qui manquent également et les faibles poids en général, l'enfant en dessous de 2, 500, dès fois 1kg et quelques grammes. [00:04:30-0](http://localhost:2300/file=C:/Users/USER/Desktop/ENTRETIENS%20PREMATURITE/CSPS-COLMA1/FOCUS%20GROUPE%20COLMA1.mp3time=270000)  [00:04:30-0](http://localhost:2300/file=C:/Users/USER/Desktop/ENTRETIENS%20PREMATURITE/CSPS-COLMA1/FOCUS%20GROUPE%20COLMA1.mp3time=270000) Interviewer: quand vous dites que la hauteur uterine n'est pas adapté,à quoi faites vous allusion? [00:04:34-1](http://localhost:2300/file=C:/Users/USER/Desktop/ENTRETIENS%20PREMATURITE/CSPS-COLMA1/FOCUS%20GROUPE%20COLMA1.mp3time=274100)  [00:04:34-1](http://localhost:2300/file=C:/Users/USER/Desktop/ENTRETIENS%20PREMATURITE/CSPS-COLMA1/FOCUS%20GROUPE%20COLMA1.mp3time=274100) Person 4: Normalement une HU normale doit atteindre 32cm en allant bon voilà, il y a des hauteurs utérines excessives aussi, mais 32 en allant. On sait que la grossesse est à terme ,une femme qui vient à 27 ou 28 cm, on sait que la grossesse n'est pas à terme. [00:04:54-2](http://localhost:2300/file=C:/Users/USER/Desktop/ENTRETIENS%20PREMATURITE/CSPS-COLMA1/FOCUS%20GROUPE%20COLMA1.mp3time=294200) |
| --- |

| 00:02:23-5 Interviewer: Ok, maintenant que vous avez défini la naissance prématurée, comment est-ce que cette naissance prématurée est mesurée au sein du centre de santé ici ? Quels sont les critères qui vous permettent de déterminer la naissance prématurée ici ? 00:02:42-7  00:02:42-7 Person 3: Bon, généralement pour les dames qui ont eu à faire des échographies, on peut déterminer l'accouchement prématuré à partir de l'échographie. Maintenant cliniquement à l'accouchement, il y a certains signes qui peuvent montrer que l'enfant est prématuré. 00:03:06-8  00:03:06-8 Interviewer: Vous faites allusion à quoi, lorsque vous parlez de signes ? 00:03:09-5  00:03:09-5 Person 3: Bon, on a, on a les cas de faible poids de naissance, il y a également la présence, comment je vais dire ça ? Aidez-moi un peu, ce qui recouvre le corps là, on appelle ça comment ? 00:03:30-7  00:03:30-7 Person 2: Le vernix. 00:03:30-4  00:03:30-4 Person 3: Voilà, généralement il y a l'absence de ce vernix souvent sur le prématuré. Maintenant il y a certains réflexes, il ne répond pas à certains réflexes, les réflexes de succion, on a les quatre points cardinaux. Il y a beaucoup de réflexes auxquels le prématuré ne répond pas. 00:04:02-8  00:04:02-8 Interviewer: Est-ce qu'il y a quelque chose d'autres qu'on peut ajouter ? 00:04:05-7  00:04:05-7 Person 4: Je voulais ajouter quand la femme entre même en travail, la HU voilà, on voit que la hauteur utérine n'est pas adaptée et cela nous prévient d'abord que ce n'est pas arrivé à terme. Voilà comme elle a dit, il y a certains réflexes qui manquent également et les faibles poids en général, l'enfant en dessous de 2,500, des fois 1kg et quelques grammes. 00:04:30-0  00:04:30-0 Interviewer: Quand vous dites que la hauteur utérine n'est pas adaptée, à quoi faites-vous allusion ? 00:04:34-1  00:04:34-1 Person 4: Normalement, une HU normale doit atteindre 32cm en allant bon voilà, il y a des hauteurs utérines excessives aussi, mais 32 en allant. On sait que la grossesse est à terme, une femme qui vient à 27 ou 28 cm, on sait que la grossesse n'est pas à terme. 00:04:54-2 |
| --- |

| **[00:02:23-5] Interviewer**: Okay, now that you've defined preterm birth, how is it measured at this health center? What criteria do you use to determine premature birth here?  **[00:02:42-7] Person 3**: For women who have had ultrasounds, we can determine premature birth based on the ultrasound results. Clinically, at delivery, there are certain signs that indicate the child is premature.  **[00:03:06-8] Interviewer**: What specific signs are you referring to?  **[00:03:09-5] Person 3**: We look for low birth weight and the absence of vernix caseosa on the premature baby. Premature babies often don't respond to certain reflexes, like sucking reflexes. There are multiple reflexes a premature baby may not exhibit.  **[00:04:02-8] Interviewer**: Is there anything else we can add?  **[00:04:05-7] Person 4**: During labor, the fundal height can be a warning sign. A normal fundal height should reach 32 cm when the pregnancy is full term. If a woman's fundal height is only 27 or 28 cm, it indicates that the pregnancy is not full term. Additionally, as mentioned, certain reflexes are lacking in premature babies, and generally, a low birth weight, sometimes as little as 1 kg, can be a sign.  **[00:04:30-0] Interviewer**: When you mention inappropriate fundal height, what exactly does that mean?  **[00:04:34-1] Person 4**: Normally, a healthy pregnancy's fundal height reaches 32 cm at full term. Excessive or insufficient fundal height, like 27 or 28 cm, can indicate that the pregnancy is not full term. |
| --- |

### Major of Colma1 health and social promotion center

| [00:02:38-8](http://localhost:2300/file=C:/Users/USER/Desktop/ENTRETIENS%20PREMATURITE/CSPS-COLMA1/MAJOR%20CSPS%20COLMA1.MP3time=158800) Interviewer: maintenant, au niveau du CSPS de colma , comment est ce que la naissance prématurée est mesurée? Quels sont les paramètres qui vous permettent donc de mesurer la naissance prématurée ? [00:02:50-5](http://localhost:2300/file=C:/Users/USER/Desktop/ENTRETIENS%20PREMATURITE/CSPS-COLMA1/MAJOR%20CSPS%20COLMA1.MP3time=170500)  [00:02:50-5](http://localhost:2300/file=C:/Users/USER/Desktop/ENTRETIENS%20PREMATURITE/CSPS-COLMA1/MAJOR%20CSPS%20COLMA1.MP3time=170500) Person 1: Les paramètres qui permettent de mesurer la naissance prématurée, il y a l'âge de la grossesse , qui est très très important. Voilà, je crois que c'est ça qui détermine, voilà. [00:03:00-8](http://localhost:2300/file=C:/Users/USER/Desktop/ENTRETIENS%20PREMATURITE/CSPS-COLMA1/MAJOR%20CSPS%20COLMA1.MP3time=180800) |
| --- |

| 00:02:38-8 Interviewer: Maintenant, au niveau du CSPS de Colma, comment est-ce que la naissance prématurée est mesurée ? Quels sont les paramètres qui vous permettent donc de mesurer la naissance prématurée ? 00:02:50-5  00:02:50-5 Person 1: Les paramètres qui permettent de mesurer la naissance prématurée, il y a l'âge de la grossesse, qui est très très important. Voilà, je crois que c'est ça qui détermine, voilà. 00:03:00-8 |
| --- |

| **[00:02:38-8] Interviewer**: Now, at the Colma CSPS, how is premature birth measured? What are the parameters that you use to assess premature birth?  **[00:02:50-5] Person 1**: The key parameter for measuring premature birth is the age of the pregnancy, which is very important. I believe that is the primary determinant. |
| --- |

### Maternity manager of Colma1 health and social promotion center

| [00:03:03-2](http://localhost:2300/file=C:/Users/USER/Desktop/ENTRETIENS%20PREMATURITE/CSPS-COLMA1/RESPONSABLE%20MATERNITE-COLMA1.mp3time=183200) Interviewer: D'accord. Maintenant, concrètement, au sein de votre centre de santé, comment est- ce que la naissance prématurée est mesuré? [00:03:09-3](http://localhost:2300/file=C:/Users/USER/Desktop/ENTRETIENS%20PREMATURITE/CSPS-COLMA1/RESPONSABLE%20MATERNITE-COLMA1.mp3time=189300)  [00:03:09-3](http://localhost:2300/file=C:/Users/USER/Desktop/ENTRETIENS%20PREMATURITE/CSPS-COLMA1/RESPONSABLE%20MATERNITE-COLMA1.mp3time=189300) Person 1: D'abord, par l'interrogatoire. L'interrogatoire d'abord, par rapport à la date des derniers règles. Je pense même que c'est la première chose d'abord, la date des dernières règles. En dehors de la date des derniières règles, souvent, il y a des dates des dernières règles qui sont inconnues. lorsque, par exemple, la femme se présente très tôt, j'ai fait deux semaines, j'ai fait un mois, je n'ai pas vu mes règles et que le test immunologique de grossesse est positif. Voilà, on peut connaître aussi à peu près l'âge de cette grossesse. En dehors de tout ça aussi, la mesure de la hauteur utérine peut vraiment souvent nous aider en tout cas. Ce qui va confirmer aussi, c'est l' échographie surtout au premier trimestre. Entre cinq à douze semaines d'aménhorrés et je pense que c'est l' échographie en ce moment est plus fiable par rapport à beaucoup de choses. on peut prendre aussi,la mesure du périmètre abdominal, mais qui n'est pas connu par à tous. par exemple, cliniquement à la naissance aussi, on peut reconnaître un enfant prématuré. Si l'interogatoire, nous révèle d'autres choses.Ajouté, on peut voir aussi cliniquement que cet enfant est prématuré. L'enfant prématuré est différent de l'enfant hippotrophe, bien sûr. [00:05:00-1](http://localhost:2300/file=C:/Users/USER/Desktop/ENTRETIENS%20PREMATURITE/CSPS-COLMA1/RESPONSABLE%20MATERNITE-COLMA1.mp3time=300100)  [00:05:00-1](http://localhost:2300/file=C:/Users/USER/Desktop/ENTRETIENS%20PREMATURITE/CSPS-COLMA1/RESPONSABLE%20MATERNITE-COLMA1.mp3time=300100) Interviewer: et quelle est la difference? [00:05:01-3](http://localhost:2300/file=C:/Users/USER/Desktop/ENTRETIENS%20PREMATURITE/CSPS-COLMA1/RESPONSABLE%20MATERNITE-COLMA1.mp3time=301300)  [00:05:01-3](http://localhost:2300/file=C:/Users/USER/Desktop/ENTRETIENS%20PREMATURITE/CSPS-COLMA1/RESPONSABLE%20MATERNITE-COLMA1.mp3time=301300) Person 1: La différence... Vous voyez un enfant hippotrophe, je prends terre à terre même, cet enfant peut être recouvert de vernix de casoesa , quelque chose que tout le monde peut voir. Mais un enfant prématuré, en tout cas, le vernix de casoesa peut être absent ou bien de petites quantités. La coloration de la peau surtout. Voilà, un enfant prématuré, au lieu que la peau soit rose, la peau peut se retrouver rouge. Quand on regarde au niveau des cheveux même, on va trouver un enfant qui est né à terme, Les cheveux sont poussés longs, mais un enfant prématuré peut ne pas avoir ça. Au niveau des organes génitaux, chez la petite fille, les lèvres ne recouvrent pas le clitoris, chez le garçon, souvent,les testicules ne sont pas dans les bourses. Pourtant, un enfant hippotrophe c'est un enfant, quand vous le voyez, je ne dirais pas un petit vieux mais les réflexes archaïques sont présents. L'enfant semble être éveillé. [00:06:22-6](http://localhost:2300/file=C:/Users/USER/Desktop/ENTRETIENS%20PREMATURITE/CSPS-COLMA1/RESPONSABLE%20MATERNITE-COLMA1.mp3time=382600) |
| --- |

| 00:03:03-2 Interviewer: D'accord. Maintenant, concrètement, au sein de votre centre de santé, comment est-ce que la naissance prématurée est mesurée ? 00:03:09-3  00:03:09-3 Person 1: D'abord, par l'interrogatoire. L'interrogatoire d'abord, par rapport à la date des dernières règles. Je pense même que c'est la première chose d'abord, la date des dernières règles. En dehors de la date des dernières règles, souvent, il y a des dates des dernières règles qui sont inconnues. Lorsque, par exemple, la femme se présente très tôt, j'ai fait deux semaines, j'ai fait un mois, je n'ai pas vu mes règles et que le test immunologique de grossesse est positif. Voilà, on peut connaître aussi à peu près l'âge de cette grossesse. En dehors de tout ça aussi, la mesure de la hauteur utérine peut vraiment souvent nous aider en tout cas. Ce qui va confirmer aussi, c'est l'échographie surtout au premier trimestre. Entre cinq à douze semaines d'aménorrhée et je pense que c'est l'échographie en ce moment est plus fiable par rapport à beaucoup de choses. On peut prendre aussi la mesure du périmètre abdominal, mais qui n'est pas connu par à tous. Par exemple, cliniquement à la naissance aussi, on peut reconnaître un enfant prématuré. Si l'interrogatoire nous révèle d'autres choses. Ajouté, on peut voir aussi cliniquement que cet enfant est prématuré. L'enfant prématuré est différent de l'enfant hypotrophe, bien sûr. 00:05:00-1  00:05:00-1 Interviewer: Et quelle est la différence ? 00:05:01-3  00:05:01-3 Person 1: La différence... Vous voyez un enfant hypotrophe, je prends terre à terre même, cet enfant peut être recouvert de vernix caseosa, quelque chose que tout le monde peut voir. Mais un enfant prématuré, en tout cas, le vernix caseosa peut être absent ou bien en petites quantités. La coloration de la peau surtout. Voilà, un enfant prématuré, au lieu que la peau soit rose, la peau peut se retrouver rouge. Quand on regarde au niveau des cheveux même, on va trouver un enfant qui est né à terme, les cheveux sont poussés longs, mais un enfant prématuré peut ne pas avoir ça. Au niveau des organes génitaux, chez la petite fille, les lèvres ne recouvrent pas le clitoris, chez le garçon, souvent, les testicules ne sont pas dans les bourses. Pourtant, un enfant hypotrophe c'est un enfant, quand vous le voyez, je ne dirais pas un petit vieux mais les réflexes archaïques sont présents. L'enfant semble être éveillé. 00:06:22-6 |
| --- |

| **[00:03:03-2] Interviewer**: Okay. Concretely, how is premature birth measured within your health center?  **[00:03:09-3] Person 1**: First, it's through interrogation, specifically regarding the date of the last menstrual period. That's usually the starting point. If the date of the last period is unknown, we rely on early pregnancy indicators, like a positive pregnancy test after a missed period, to estimate the gestational age. Measuring fundal height is also helpful. Ultrasound, especially in the first trimester (between five to twelve weeks of amenorrhea), is very reliable for determining pregnancy age. The abdominal perimeter measurement is another method, but it's not as widely known. Clinically, at birth, we can also recognize a premature child based on various signs.  **[00:05:00-1] Interviewer**: And what's the difference between a premature child and a hypotrophic child?  **[00:05:01-3] Person 1**: A hypotrophic child may be covered in vernix caseosa, which is easy to spot. In contrast, a premature child may have little to no vernix caseosa. Their skin coloring is often red instead of pink. Their hair might be less developed compared to a full-term child. In terms of genital development, in girls, the labia may not fully cover the clitoris, and in boys, the testicles may not be descended. Hypotrophic children usually display archaic reflexes and appear more awake. |
| --- |

### Focus group of Farakan health and social promotion center

| [00:04:17-3](http://localhost:2300/file=C:/Users/USER/Desktop/ENTRETIENS%20PREMATURITE/CSPS_FARAKAN/FOCUS_GROUPE-CSPS_FARAKAN.MP3time=257300) Interviewer: Merci beaucoup pour votre réponse, la question qui suit maintenant que vous avez défini la naissance prématurée, comment est-ce que cela est mesuré au sein de votre centre de santé? [00:04:36-8](http://localhost:2300/file=C:/Users/USER/Desktop/ENTRETIENS%20PREMATURITE/CSPS_FARAKAN/FOCUS_GROUPE-CSPS_FARAKAN.MP3time=276800)  [00:04:36-8](http://localhost:2300/file=C:/Users/USER/Desktop/ENTRETIENS%20PREMATURITE/CSPS_FARAKAN/FOCUS_GROUPE-CSPS_FARAKAN.MP3time=276800) Person 1: Au sein de notre formation sanitaire. Bon, nous on se fait le plus souvent au semaines d'aménorrhée.Par exemple, si la femme a moins de 37 semaines d'aménorrhée, nous disons que c'est prématuré. Et lorsque aussi que l'enfant est né et qu'il a un poids inférieur vraiment à 2 500 et le plus souvent, il y a des signes accompagnateurs. Bon, on essaie d'examiner le nouveau-né par rapport certains signes pour compléter. [00:05:13-8](http://localhost:2300/file=C:/Users/USER/Desktop/ENTRETIENS%20PREMATURITE/CSPS_FARAKAN/FOCUS_GROUPE-CSPS_FARAKAN.MP3time=313800)  [00:05:13-8](http://localhost:2300/file=C:/Users/USER/Desktop/ENTRETIENS%20PREMATURITE/CSPS_FARAKAN/FOCUS_GROUPE-CSPS_FARAKAN.MP3time=313800) Person 3: Un poids très faible, inferieur à 2 kilos 500. [00:05:30-6](http://localhost:2300/file=C:/Users/USER/Desktop/ENTRETIENS%20PREMATURITE/CSPS_FARAKAN/FOCUS_GROUPE-CSPS_FARAKAN.MP3time=330600)  [00:05:30-6](http://localhost:2300/file=C:/Users/USER/Desktop/ENTRETIENS%20PREMATURITE/CSPS_FARAKAN/FOCUS_GROUPE-CSPS_FARAKAN.MP3time=330600) Interviewer: Maintenant que vous avez défini la naissance prématurée, la question qui suit, comment est-ce que la naissance prématurée est mesurée au sein de votre centre de santé ? Concrètement, comment cela est mesurée ? [00:05:55-0](http://localhost:2300/file=C:/Users/USER/Desktop/ENTRETIENS%20PREMATURITE/CSPS_FARAKAN/FOCUS_GROUPE-CSPS_FARAKAN.MP3time=355000)  [00:05:55-0](http://localhost:2300/file=C:/Users/USER/Desktop/ENTRETIENS%20PREMATURITE/CSPS_FARAKAN/FOCUS_GROUPE-CSPS_FARAKAN.MP3time=355000) Person 4: Chez nous ici, effectivement ça dépendra du poids de l'enfant. Si c'est vraiment un faible poids qui mérite à être référé, on réfère, mais si c'est gerable, on leur donne des conseils comment bien prendre en charge l'enfant. Et ça dépendra aussi du climat. Voilà par exemple, si c'est le mois de décembre, un exemple comme ça la prise en charge faut bien vraiment envelopper l'enfant et la plupart on conseille aux nouvellement accouchée d'aimer garder l'enfant sur le ventre, peau à peau avec la maman. [00:06:52-8](http://localhost:2300/file=C:/Users/USER/Desktop/ENTRETIENS%20PREMATURITE/CSPS_FARAKAN/FOCUS_GROUPE-CSPS_FARAKAN.MP3time=412800)  [00:06:52-8](http://localhost:2300/file=C:/Users/USER/Desktop/ENTRETIENS%20PREMATURITE/CSPS_FARAKAN/FOCUS_GROUPE-CSPS_FARAKAN.MP3time=412800) Person 2: la prise en charge en tout cas, nous essayons d'envelopper l'enfant dans un linge chaud et sec, et on met l'enfant peau à peau avec la mère, nous l'evacuons au niveau du district au CMA de Dô ou nous avons une unité kangourou. Par exemple pour les grands prématurés, nous les envoyons directement en ambulance à l'hôpital Souro SANOU en néonatologie. [00:07:24-0](http://localhost:2300/file=C:/Users/USER/Desktop/ENTRETIENS%20PREMATURITE/CSPS_FARAKAN/FOCUS_GROUPE-CSPS_FARAKAN.MP3time=444000)  [00:07:24-0](http://localhost:2300/file=C:/Users/USER/Desktop/ENTRETIENS%20PREMATURITE/CSPS_FARAKAN/FOCUS_GROUPE-CSPS_FARAKAN.MP3time=444000) Person 6: La prise en charge des cas de prématurés ici c'est rare donc après accouchement seulement on réfère l'enfant à l'echelon superieur pour une meilleure prise en charge. [00:07:43-1](http://localhost:2300/file=C:/Users/USER/Desktop/ENTRETIENS%20PREMATURITE/CSPS_FARAKAN/FOCUS_GROUPE-CSPS_FARAKAN.MP3time=463100)  [00:07:43-1](http://localhost:2300/file=C:/Users/USER/Desktop/ENTRETIENS%20PREMATURITE/CSPS_FARAKAN/FOCUS_GROUPE-CSPS_FARAKAN.MP3time=463100)Person 4: Par faute de par faute de matériel, on préfère référer immédiatement.Parce que nous n'avons pas de materiel pour vraiment nous occuper de l'enfant ici. [00:08:00-3](http://localhost:2300/file=C:/Users/USER/Desktop/ENTRETIENS%20PREMATURITE/CSPS_FARAKAN/FOCUS_GROUPE-CSPS_FARAKAN.MP3time=480300)  [00:08:00-3](http://localhost:2300/file=C:/Users/USER/Desktop/ENTRETIENS%20PREMATURITE/CSPS_FARAKAN/FOCUS_GROUPE-CSPS_FARAKAN.MP3time=480300) Interviewer: Vous avez quelque chose à ajouter? [00:08:01-8](http://localhost:2300/file=C:/Users/USER/Desktop/ENTRETIENS%20PREMATURITE/CSPS_FARAKAN/FOCUS_GROUPE-CSPS_FARAKAN.MP3time=481800)  [00:08:01-8](http://localhost:2300/file=C:/Users/USER/Desktop/ENTRETIENS%20PREMATURITE/CSPS_FARAKAN/FOCUS_GROUPE-CSPS_FARAKAN.MP3time=481800) Person 2: Oui. Nous administrons de la vitamine K1 à nos enfants, systématiquement ici , qu'il soit prématuré ou poids normal. [00:08:10-9](http://localhost:2300/file=C:/Users/USER/Desktop/ENTRETIENS%20PREMATURITE/CSPS_FARAKAN/FOCUS_GROUPE-CSPS_FARAKAN.MP3time=490900) |
| --- |

| 00:04:17-3 Interviewer: Merci beaucoup pour votre réponse. La question qui suit maintenant que vous avez défini la naissance prématurée, comment est-ce que cela est mesuré au sein de votre centre de santé ? 00:04:36-8  00:04:36-8 Person 1: Au sein de notre formation sanitaire, nous nous fions le plus souvent aux semaines d'aménorrhée. Par exemple, si la femme a moins de 37 semaines d'aménorrhée, nous disons que c'est prématuré. Et lorsque l'enfant est né et qu'il a un poids inférieur à 2 500, et le plus souvent, il y a des signes accompagnateurs. On essaie d'examiner le nouveau-né par rapport à certains signes pour compléter. 00:05:13-8  00:05:13-8 Person 3: Un poids très faible, inférieur à 2 kilos 500. 00:05:30-6  00:05:30-6 Interviewer: Maintenant que vous avez défini la naissance prématurée, comment est-ce que la naissance prématurée est mesurée au sein de votre centre de santé ? Concrètement, comment cela est mesurée ? 00:05:55-0  00:05:55-0 Person 4: Chez nous ici, effectivement ça dépend du poids de l'enfant. Si c'est vraiment un faible poids qui mérite à être référé, on réfère, mais si c'est gérable, on leur donne des conseils comment bien prendre en charge l'enfant. Et ça dépend aussi du climat. Voilà, par exemple, si c'est le mois de décembre, un exemple comme ça, la prise en charge faut bien vraiment envelopper l'enfant, et la plupart on conseille aux nouvellement accouchées d'aimer garder l'enfant sur le ventre, peau à peau avec la maman. 00:06:52-8  00:06:52-8 Person 2: La prise en charge en tout cas, nous essayons d'envelopper l'enfant dans un linge chaud et sec, et on met l'enfant peau à peau avec la mère, nous l'évacuons au niveau du district au CMA de Dô où nous avons une unité kangourou. Par exemple pour les grands prématurés, nous les envoyons directement en ambulance à l'hôpital Souro Sanou en néonatologie. 00:07:24-0  00:07:24-0 Person 6: La prise en charge des cas de prématurés ici c'est rare donc après accouchement seulement on réfère l'enfant à l'échelon supérieur pour une meilleure prise en charge. 00:07:43-1  00:07:43-1 Person 4: Par faute de matériel, on préfère référer immédiatement. Parce que nous n'avons pas de matériel pour vraiment nous occuper de l'enfant ici. 00:08:00-3  00:08:00-3 Interviewer: Vous avez quelque chose à ajouter ? 00:08:01-8  00:08:01-8 Person 2: Oui. Nous administrons de la vitamine K1 à nos enfants, systématiquement ici, qu'il soit prématuré ou de poids normal. 00:08:10-9 |
| --- |

| **[00:04:17-3] Interviewer**: Thank you for your response. Now that you have defined premature birth, how is it measured within your health center?  **[00:04:36-8] Person 1**: In our facility, we mainly rely on the weeks of amenorrhea. For instance, if a woman has less than 37 weeks of amenorrhea, we classify the birth as premature. When a child is born weighing less than 2,500 grams, we also look for accompanying signs and examine the newborn for specific indications of prematurity.  **[00:05:13-8] Person 3**: A key factor is very low birth weight, less than 2,500 kilograms.  **[00:05:30-6] Interviewer**: How exactly is premature birth measured at your health center? Can you describe the process?  **[00:05:55-0] Person 4**: Our approach depends on the child's weight. If it's a very low weight that warrants referral, we do so. Otherwise, we offer advice on proper care. The climate also influences our care methods. For example, in colder months like December, we emphasize keeping the child warm and advising skin-to-skin contact with the mother.  **[00:06:52-8] Person 2**: We wrap the child in warm, dry cloth and facilitate skin-to-skin contact with the mother. In cases of very premature babies, we refer them to higher-level facilities like the kangaroo unit at the CMA of Dô or, for critical cases, directly to the neonatology department at Souro Sanou Hospital by ambulance.  **[00:07:24-0] Person 6**: Our facility rarely manages cases of premature babies. We usually refer them to higher-level facilities for better care after delivery.  **[00:07:43-1] Person 4**: Due to a lack of equipment, we prefer immediate referral. We don't have the necessary equipment to adequately care for premature babies here.  **[00:08:00-3] Interviewer**: Is there anything else to add?  **[00:08:01-8] Person 2**: Yes. We administer vitamin K1 to all newborns here, regardless of whether they are premature or of normal weight. |
| --- |

### Major of Farakan health and social promotion center

| [00:02:46-2](http://localhost:2300/file=C:/Users/USER/Desktop/ENTRETIENS%20PREMATURITE/CSPS_FARAKAN/MAJOR%20FARAKAN.MP3time=166200) Interviewer: Comment est que la naissance prématurée est mesurée dans votre centre de santé? à travers quels parametres vous mesurez la naissance prématurée? [00:02:55-1](http://localhost:2300/file=C:/Users/USER/Desktop/ENTRETIENS%20PREMATURITE/CSPS_FARAKAN/MAJOR%20FARAKAN.MP3time=175100)  [00:02:55-1](http://localhost:2300/file=C:/Users/USER/Desktop/ENTRETIENS%20PREMATURITE/CSPS_FARAKAN/MAJOR%20FARAKAN.MP3time=175100) Person 1: C'est la gestité et le nombre de mois, on dit inferieur à 37 semaine non pour être prématuré. [00:03:03-8](http://localhost:2300/file=C:/Users/USER/Desktop/ENTRETIENS%20PREMATURITE/CSPS_FARAKAN/MAJOR%20FARAKAN.MP3time=183800) |
| --- |

| 00:02:46-2 Interviewer: Comment est-ce que la naissance prématurée est mesurée dans votre centre de santé ? À travers quels paramètres vous mesurez la naissance prématurée ? 00:02:55-1  00:02:55-1 Person 1: C'est la gestité et le nombre de mois, on dit inférieur à 37 semaines pour être prématuré. 00:03:03-8 |
| --- |

| **[00:02:46-2] Interviewer**: How is premature birth measured in your health center? What parameters are used to measure premature birth?  **[00:02:55-1] Person 1**: The main parameters we use are the birth rate and the number of months of pregnancy. We consider a birth to be premature if it occurs before 37 weeks. |
| --- |

### Maternity manager of Farakan health and social promotion center

| [00:02:31-9](http://localhost:2300/file=C:/Users/USER/Desktop/ENTRETIENS%20PREMATURITE/CSPS_FARAKAN/RESPONSABLE%20MATERNITE%20FARAKAN.MP3time=151900) Interviewer: Maintenant que vous avez défini la prématurée concrètement, comment cela est mesuré au sein de votre centre de santé ? [00:02:42-5](http://localhost:2300/file=C:/Users/USER/Desktop/ENTRETIENS%20PREMATURITE/CSPS_FARAKAN/RESPONSABLE%20MATERNITE%20FARAKAN.MP3time=162500)  [00:02:42-5](http://localhost:2300/file=C:/Users/USER/Desktop/ENTRETIENS%20PREMATURITE/CSPS_FARAKAN/RESPONSABLE%20MATERNITE%20FARAKAN.MP3time=162500) Person 1: En tout cas on rencontre beaucoup de cas, il y en a. Si par exemple elle arrive et que on peut la réferer, on la refère, mais y a des moments aussi, elle, elle n'arrive pas tôt.Lorsqu'elle n'arrive pas tôt, elle est venue . On constate est déjà dans la phase active c'estvà dire que l'accouchement est imminent, on peut plus rien faire. Mais maintenant, quand c'est une menace, on l'envoie au CHURSS. Mais après l'accouchement aussi, la prise en charge, on envoie au niveau de l'hôpital en pédiatrie.Surtout les grands prématurés, nous ne pouvons pas les prendre en charge ici. Mais y a des enfants aussi qui naissent.on sait que c'est la prématurée , mais l'enfant est quand même bien par rapport au poids et on peut le garder s'il n'y a pas de signes de detresse et nous donnons des conseils à la maman. [00:03:45-3](http://localhost:2300/file=C:/Users/USER/Desktop/ENTRETIENS%20PREMATURITE/CSPS_FARAKAN/RESPONSABLE%20MATERNITE%20FARAKAN.MP3time=225300)  [00:03:45-3](http://localhost:2300/file=C:/Users/USER/Desktop/ENTRETIENS%20PREMATURITE/CSPS_FARAKAN/RESPONSABLE%20MATERNITE%20FARAKAN.MP3time=225300) Interviewer: Maintenant comment est ce que vous mesurez la prématurité dans votre centre de santé? [00:03:53-8](http://localhost:2300/file=C:/Users/USER/Desktop/ENTRETIENS%20PREMATURITE/CSPS_FARAKAN/RESPONSABLE%20MATERNITE%20FARAKAN.MP3time=233800)  [00:03:53-8](http://localhost:2300/file=C:/Users/USER/Desktop/ENTRETIENS%20PREMATURITE/CSPS_FARAKAN/RESPONSABLE%20MATERNITE%20FARAKAN.MP3time=233800) Person 1: Comment on mesure ça? C'est par rapport à la prise en charge ou bien. En tout cas, ça dépend, ça demande une prise en charge en tout cas particulier, ça ne sera pas comme les autres enfants. [00:04:13-2](http://localhost:2300/file=C:/Users/USER/Desktop/ENTRETIENS%20PREMATURITE/CSPS_FARAKAN/RESPONSABLE%20MATERNITE%20FARAKAN.MP3time=253200) |
| --- |

| 00:02:31-9 Interviewer: Maintenant que vous avez défini la prématurée concrètement, comment cela est mesuré au sein de votre centre de santé ? 00:02:42-5  00:02:42-5 Person 1: En tout cas, on rencontre beaucoup de cas. Si par exemple elle arrive et qu'on peut la référer, on la réfère. Mais il y a des moments aussi où elle n'arrive pas tôt. Lorsqu'elle n'arrive pas tôt et qu'elle est déjà dans la phase active, c'est-à-dire que l'accouchement est imminent, on ne peut plus rien faire. Mais maintenant, quand c'est une menace, on l'envoie au CHURSS. Mais après l'accouchement aussi, pour la prise en charge, on envoie au niveau de l'hôpital en pédiatrie. Surtout les grands prématurés, nous ne pouvons pas les prendre en charge ici. Mais il y a des enfants aussi qui naissent, on sait que c'est la prématurée, mais l'enfant est quand même bien par rapport au poids et on peut le garder s'il n'y a pas de signes de détresse et nous donnons des conseils à la maman. 00:03:45-3  00:03:45-3 Interviewer: Maintenant comment est-ce que vous mesurez la prématurité dans votre centre de santé ? 00:03:53-8  00:03:53-8 Person 1: Comment on mesure ça ? C'est par rapport à la prise en charge ou bien. En tout cas, ça demande une prise en charge en tout cas particulière, ça ne sera pas comme les autres enfants. 00:04:13-2 |
| --- |

| **[00:02:31-9] Interviewer**: Now that you have defined premature birth concretely, how is it measured within your health center?  **[00:02:42-5] Person 1**: We encounter a variety of cases. If a woman arrives early enough for a referral, we refer her. However, if she arrives late and is already in the active phase of labor, with birth imminent, there's not much we can do. When there's a threat of premature birth, we send her to CHURSS. After delivery, for specialized care, especially for very premature babies, we send them to the pediatric hospital, as we can't manage them here. But there are also babies born prematurely who are relatively healthy in terms of weight and show no signs of distress. In such cases, we can keep them here and provide advice to the mother.  **[00:03:45-3] Interviewer**: How do you specifically measure prematurity in your health center?  **[00:03:53-8] Person 1**: How do we measure it? It's based on the level of care needed. Premature babies require special care, different from what other children need. |
| --- |

## Control of the date of the last period

### Focus group of Accart-ville urban medical center

| [00:03:15-9](http://localhost:2300/file=C:/Users/USER/Desktop/ENTRETIENS%20PREMATURITE/CSPS-ACCART-VILLE/FOCUS%20GROUPE%20ACCART-VILLE-BON.mp3time=195900)Interviewer: maintenant sur 10 femmes qui se presentent à leur premier CPN, combien de fmmes connaissent la date de leur dernières règles? [00:03:23-7](http://localhost:2300/file=C:/Users/USER/Desktop/ENTRETIENS%20PREMATURITE/CSPS-ACCART-VILLE/FOCUS%20GROUPE%20ACCART-VILLE-BON.mp3time=203700)  [00:03:23-7](http://localhost:2300/file=C:/Users/USE) Person 4: en afrique içi en tout cas, ce n'est pas beaucoup. sur 10 patientes,on peut dire au moins 4 puisqu'avec l'analphetisme, elles vont te donner en fonction des saisons, même celles qui ont fréquenté. par exemple, elles vont dire quand on coupait le maîs ou bien c'etait au mois de ramadan. donc le mois de ramadan, one connait pas le jour exacte. [00:03:58-2](http://localhost:2300/file=C:/Users/U)  [00:03:58-2](http://localhost:2300/file=C:/Users/USER/Desktop/ENTRETIENS) Interviewer: maintenant est ce que vous pensez que les femmes connaissent avec precision la date de leur dernières règles à leur première CPN? [00:04:02-0](http://localhost:2300/file=C:/Users/USER/Desktop/ENTRETIENS)  [00:04:02-0](http://localhost:2300/file=C:/Users/USER/Desktop/ENTRETIENS%20PREMATURITE/CSPS-ACCART-VILLE/FOCUS%20GROUPE%20ACCART-VILLE-BON.mp3time=242000) Person 1: pas toutes les femmes [00:04:06-2](http://localhost:2300/file=C:/Users/USER/Desktop/ENTRETIENS%20PREMATURITE/CSPS-ACCART-VILLE/FOCUS%20GROUPE%20ACCART-VILLE-BON.mp3time=246200)  [00:04:06-2](http://localhost:2300/file=C:/Users/USER/Desktop/ENTRETIENS) Interviewer: d'accord mais sur une echelle de 1 à 10? [00:04:09-1](http://localhost:2300/file=C:/Users/USER/Desktop/ENTRETIENS)  [00:04:09-1](http://localhost:2300/file=C:/Users/USER/Desktop/ENTRETIENS%20PREMATURITE/CSPS-ACCART-VILLE/FOCUS%20GROUPE%20ACCART-VILLE-BON.mp3time=249100)Person 1: moi je donne toujours les quatre. oui elles ne sont pas nombreuses [00:04:14-5](http://localhost:2300/file=C:/Users/USER/Desktop/ENTRETIENS%20PREMATURITE/CSPS-ACCART-VILLE/FOCUS%20GROUPE%20ACCART-VILLE-BON.mp3time=254500)  [00:04:14-5](http://localhost:2300/file=C:/Users/USER/Desktop/ENTRETIENS) Interviewer: et géneralement à quelle moment de la grossesse, ou date de la grossesse elles viennent à leure première CPN? [00:04:18-5](http://localhost:2300/file=C:/Users/USE)  [00:04:18-5](http://localhost:2300/file=C:/Users/USER/Desktop/ENTRETIENS) Person 1: souvent trois mois puisque c'est ce qui etait dit avant, donc elles respectent toujours cette periode c'est à dire trois mois de la grossesse, sinon l'OMS à donner des recommandations que dès que la femme constate qu'elle est enceinte, elle doit venir consulter mais elles viennent toujours les trois premiers mois. souvent d'autres viennent après les trois premiers mois [00:04:36-2](http://localhost:2300/file=C:/Users/USER/Desktop/ENTRETIENS) |
| --- |

| 00:03:15-9 Interviewer: Maintenant, sur 10 femmes qui se présentent à leur premier CPN, combien de femmes connaissent la date de leurs dernières règles? 00:03:23-7  00:03:23-7 Person 4: En Afrique, ici en tout cas, ce n'est pas beaucoup. Sur 10 patientes, on peut dire au moins 4 puisqu'avec l'analphabétisme, elles vont te donner en fonction des saisons, même celles qui ont fréquenté. Par exemple, elles vont dire quand on coupait le maïs ou bien c'était au mois de Ramadan. Donc, le mois de Ramadan, on ne connaît pas le jour exact. 00:03:58-2  00:03:58-2 Interviewer: Maintenant, est-ce que vous pensez que les femmes connaissent avec précision la date de leurs dernières règles à leur première CPN? 00:04:02-0  00:04:02-0 Person 1: Pas toutes les femmes. 00:04:06-2  00:04:06-2 Interviewer: D'accord, mais sur une échelle de 1 à 10? 00:04:09-1  00:04:09-1 Person 1: Moi, je donne toujours les quatre. Oui, elles ne sont pas nombreuses. 00:04:14-5  00:04:14-5 Interviewer: Et généralement à quel moment de la grossesse, ou date de la grossesse, elles viennent à leur première CPN? 00:04:18-5  00:04:18-5 Person 1: Souvent trois mois puisque c'est ce qui était dit avant, donc elles respectent toujours cette période, c'est-à-dire trois mois de la grossesse. Sinon, l'OMS a donné des recommandations que dès que la femme constate qu'elle est enceinte, elle doit venir consulter, mais elles viennent toujours les trois premiers mois. Souvent, d'autres viennent après les trois premiers mois. 00:04:36-2 |
| --- |

| **[00:03:15-9] Interviewer**: Now, among 10 women who attend their first antenatal care (ANC) appointment, how many of them are aware of the date of their last menstrual period?  **[00:03:23-7] Person 4**: In Africa, the number is quite low. Out of 10 patients, maybe around 4 know the date. Due to illiteracy, many women relate it to events or seasons. For example, they might refer to the time of corn harvesting or the month of Ramadan. However, such references don't provide an exact day.  **[00:03:58-2] Interviewer**: Do you think that women precisely know the date of their last period when they come for their first antenatal care (ANC) appointment?  **[00:04:02-0] Person 1**: Not all women do.  **[00:04:06-2] Interviewer**: Okay, but on a scale of 1 to 10, how many would you say?  **[00:04:09-1] Person 1**: I would say about 4 out of 10. There aren't many.  **[00:04:14-5] Interviewer**: And generally, at what point in their pregnancy do they come for their first ANC?  **[00:04:18-5] Person 1**: Often, they come around three months into their pregnancy, as that's the traditional recommendation. Though the WHO advises coming as soon as pregnancy is realized, they typically adhere to the three-month mark. Some come after the first three months. |
| --- |

### Manager of Accart-ville urban medical center

| [00:03:03-3](http://localhost:2300/file=C:/Users/USER/Desktop/ENTRETIENS%20PREMATURITE/CSPS-ACCART-VILLE/RESPONSABLE%20CMU%20ACCART-VILLE.MP3time=183300)Interviewer: Maintenant, si on prend une échelle de 1 à 10, c'est à dire que sur dix femmes qui se présentent à leur première CPN, combien connaissent leur date de dernière règle? [00:03:15-5](http://localhost:2300/file=C:/Users/USER/Desktop/ENTRETIENS%20PREMATURITE/CSPS-ACCART-VILLE/RESPONSABLE%20CMU%20ACCART-VILLE.MP3time=195500)  [00:03:15-5](http://localhost:2300/file=C:/Users/USER/Desktop/ENTRETIENS%20PREMATURITE/CSPS-ACCART-VILLE/RESPONSABLE%20CMU%20ACCART-VILLE.MP3time=195500)Person 1: Bon, généralement, c'est assez rare de pouvoir definir c'est pourquoi moi je disais toujours au sage femme qui intervient au niveau de la consultation prénatale de toujours essayer de situer par rapport aux grands événements tout ou pour pouvoir situer la date de dernière règles afin de pouvoir situer l'âge de la grossesse, elles disent que parfois c'est difficile, mais bon, on essaie de faire avec. [00:03:42-7](http://localhost:2300/file=C:/Users/USER/Desktop/ENTRETIENS%20PREMATURITE/CSPS-ACCART-VILLE/RESPONSABLE%20CMU%20ACCART-VILLE.MP3time=222700)  [00:03:42-7](http://localhost:2300/file=C:/Users/USER/Desktop/ENTRETIENS%20PREMATURITE/CSPS-ACCART-VILLE/RESPONSABLE%20CMU%20ACCART-VILLE.MP3time=222700) Interviewer: mais sur une echelle de 1 à 10 combien de femmes arrivent peut être à maitriser la date de leure dernière règle? [00:03:47-6](http://localhost:2300/file=C:/Users/USER/Desktop/ENTRETIENS%20PREMATURITE/CSPS-ACCART-VILLE/RESPONSABLE%20CMU%20ACCART-VILLE.MP3time=227600)  [00:03:47-6](http://localhost:2300/file=C:/Users/USER/Desktop/ENTRETIENS%20PREMATURITE/CSPS-ACCART-VILLE/RESPONSABLE%20CMU%20ACCART-VILLE.MP3time=227600)Person 1: Ils vont pas dépasser trois (03). [00:03:51-5](http://localhost:2300/file=C:/Users/USER/Desktop/ENTRETIENS%20PREMATURITE/CSPS-ACCART-VILLE/RESPONSABLE%20CMU%20ACCART-VILLE.MP3time=231500)  [00:03:51-5](http://localhost:2300/file=C:/Users/USER/Desktop/ENTRETIENS%20PREMATURITE/CSPS-ACCART-VILLE/RESPONSABLE%20CMU%20ACCART-VILLE.MP3time=231500) Interviewer: Maintenant, à quel trimestre de grossesse ou à quel âge gestationnel, les femmes viennent à la première CPN le plus souvent? [00:03:59-2](http://localhost:2300/file=C:/Users/USER/Desktop/ENTRETIENS%20PREMATURITE/CSPS-ACCART-VILLE/RESPONSABLE%20CMU%20ACCART-VILLE.MP3time=239200)  [00:03:59-2](http://localhost:2300/file=C:/Users/USER/Desktop/ENTRETIENS%20PREMATURITE/CSPS-ACCART-VILLE/RESPONSABLE%20CMU%20ACCART-VILLE.MP3time=239200)Person 1: generalement c'est après 3 mois à 04 mois. Puisque généralement, il y a des gens qu'on peut recevoir en consultation, par exemple, qui sont là au début de la grossesse, quand tu leurs dit de faire leurs pesées, ils disent que lorsqu'ils vont commencer la pesée ils vont trop durer içi et pourtant, avec les nouvelles mesures qui sont là quand on parle de CPN recentrée on doit commencer très tôt, mais quand on les parle il y a des gens, ils n'acceptent pas, ils vont rester après, pour ne même pas dire après 20 semaines d'aménorrhée, puis dans les quatre mois, cinq mois comme ça, pour pouvoir commencer les CPN. [00:04:34-8](http://localhost:2300/file=C:/Users/USER/Desktop/ENTRETIENS%20PREMATURITE/CSPS-ACCART-VILLE/RESPONSABLE%20CMU%20ACCART-VILLE.MP3time=274800)  [00:04:34-8](http://localhost:2300/file=C:/Users/USER/Desktop/ENTRETIENS%20PREMATURITE/CSPS-ACCART-VILLE/RESPONSABLE%20CMU%20ACCART-VILLE.MP3time=274800)Interviewer: les femmes qui viennent pour leur première CPN, combien à peu près peuvent connaître la date de leur dernière règle ? [00:04:43-4](http://localhost:2300/file=C:/Users/USER/Desktop/ENTRETIENS%20PREMATURITE/CSPS-ACCART-VILLE/RESPONSABLE%20CMU%20ACCART-VILLE.MP3time=283400)  [00:04:43-4](http://localhost:2300/file=C:/Users/USER/Desktop/ENTRETIENS%20PREMATURITE/CSPS-ACCART-VILLE/RESPONSABLE%20CMU%20ACCART-VILLE.MP3time=283400)Person 1: en fait ça, c'est très, très rare même de trouver peut être sur les 10 on peut trouver que c'est une seule personne qui va se rappeler, puisque si la personne ne commence pas tôt, la personne oublie. Et pourtant, quand il commence les pesées où il avait déjà vu un agent de santé , l'agent de santé qui l'avait aider à se souvenirr ou qui avait donné un carnet ou c'est écrit dedans cela peut rester. Généralement, quand il vient vers les premières CPN qui se trouve au tours de plus de seize semaines. C'est difficile parfois de pouvoir se rappeler sur les 10 on peut dire que c'est une seule personne, même les femmes instruites parfois c'est difficile. [00:05:18-1](http://localhost:2300/file=C:/Users/USER/Desktop/ENTRETIENS%20PREMATURITE/CSPS-ACCART-VILLE/RESPONSABLE%20CMU%20ACCART-VILLE.MP3time=318100) |
| --- |

| 00:03:03-3 Interviewer: Maintenant, si on prend une échelle de 1 à 10, c'est-à-dire que sur dix femmes qui se présentent à leur première CPN, combien connaissent leur date de dernière règle? 00:03:15-5  00:03:15-5 Person 1: Bon, généralement, c'est assez rare de pouvoir définir, c'est pourquoi moi je disais toujours aux sages-femmes qui interviennent au niveau de la consultation prénatale de toujours essayer de situer par rapport aux grands événements pour pouvoir situer la date de dernière règle afin de pouvoir situer l'âge de la grossesse. Elles disent que parfois c'est difficile, mais bon, on essaie de faire avec. 00:03:42-7  00:03:42-7 Interviewer: Mais sur une échelle de 1 à 10, combien de femmes arrivent peut-être à maîtriser la date de leur dernière règle? 00:03:47-6  00:03:47-6 Person 1: Ils ne vont pas dépasser trois (03). 00:03:51-5  00:03:51-5 Interviewer: Maintenant, à quel trimestre de grossesse ou à quel âge gestationnel, les femmes viennent-elles à la première CPN le plus souvent? 00:03:59-2  00:03:59-2 Person 1: Généralement c'est après 3 à 4 mois. Puisque généralement, il y a des gens qu'on peut recevoir en consultation, par exemple, qui sont là au début de la grossesse. Quand tu leur dis de faire leurs pesées, ils disent que lorsqu'ils vont commencer la pesée, ils vont trop durer ici. Et pourtant, avec les nouvelles mesures qui sont là, quand on parle de CPN recentrée, on doit commencer très tôt, mais quand on leur parle, il y a des gens qui n'acceptent pas, ils vont rester après, pour ne même pas dire après 20 semaines d'aménorrhée, puis dans les quatre, cinq mois comme ça, pour pouvoir commencer les CPN. 00:04:34-8  00:04:34-8 Interviewer: Les femmes qui viennent pour leur première CPN, combien à peu près peuvent connaître la date de leur dernière règle? 00:04:43-4  00:04:43-4 Person 1: En fait ça, c'est très, très rare même de trouver, peut-être sur les 10, on peut trouver que c'est une seule personne qui va se rappeler, puisque si la personne ne commence pas tôt, la personne oublie. Et pourtant, quand elle commence les pesées où elle avait déjà vu un agent de santé, l'agent de santé qui l'avait aidée à se souvenir ou qui avait donné un carnet où c'est écrit dedans, cela peut rester. Généralement, quand elle vient vers les premières CPN qui se trouvent autour de plus de seize semaines, c'est difficile parfois de pouvoir se rappeler. Sur les 10, on peut dire que c'est une seule personne, même les femmes instruites parfois c'est difficile. 00:05:18-1 |
| --- |

| **[00:03:03-3] Interviewer**: On a scale of 1 to 10, out of ten women who come for their first antenatal care (ANC), how many know the date of their last menstrual period?  **[00:03:15-5] Person 1**: It's quite rare to define that precisely. I always encourage midwives in prenatal consultations to try to correlate the date of the last period with major events to estimate the pregnancy age. It's sometimes difficult, but we manage.  **[00:03:42-7] Interviewer**: But on a scale of 1 to 10, how many women can accurately recall the date of their last period?  **[00:03:47-6] Person 1**: It won't exceed three out of ten.  **[00:03:51-5] Interviewer**: At what gestational age or during which trimester do women most often come for their first ANC?  **[00:03:59-2] Person 1**: Usually, it's after 3 to 4 months. Some women, especially at the start of pregnancy, may delay ANC until after 20 weeks of amenorrhea, around four or five months.  **[00:04:34-8] Interviewer**: Among women who come for their first ANC, approximately how many remember the date of their last period?  **[00:04:43-4] Person 1**: It's very rare. Maybe only one out of ten will remember. Even educated women find it difficult to recall if they don't start ANC early. If they've been given a notebook with the date noted down by a health worker, they might remember. But generally, for the first ANC around 16 weeks or more, it's difficult to recall. |
| --- |

### Maternity manager of Accart-ville urban medical center

| [00:03:17-2](http://localhost:2300/file=C:/Users/USER/Desktop/ENTRETIENS%20PREMATURITE/CSPS-ACCART-VILLE/RESPONSABLE%20MATERNITE%20CMU%20ACCART-VILLE.MP3time=197200) Interviewer: donc nous allons toujours poursuivre, maintenant sur une echelle de 1 à 10 femmes qui se presentent à leur premières cpn, combien connaissent leurs dates des dernières règles? [00:03:28-3](http://localhost:2300/file=C:/Users/USER/Desktop/ENTRETIENS%20PREMATURITE/CSPS-ACCART-VILLE/RESPONSABLE%20MATERNITE%20CMU%20ACCART-VILLE.MP3time=208300)  [00:03:28-3](http://localhost:2300/file=C:/Users/USER/Desktop/ENTRETIENS%20PREMATURITE/CSPS-ACCART-VILLE/RESPONSABLE%20MATERNITE%20CMU%20ACCART-VILLE.MP3time=208300) Person 1: donc très peu connaissent leur date des dernières règles , tres tres peu donc si je veux donner une estimation donc je peux dire trois (3), trois même je serai un peu plus large donc en moyenne trois vont connaitre la date des dernières règles [00:03:44-5](http://localhost:2300/file=C:/Users/USER/Desktop/ENTRETIENS%20PREMATURITE/CSPS-ACCART-VILLE/RESPONSABLE%20MATERNITE%20CMU%20ACCART-VILLE.MP3time=224500)  [00:03:44-5](http://localhost:2300/file=C:/Users/USER/Desktop/ENTRETIENS%20PREMATURITE/CSPS-ACCART-VILLE/RESPONSABLE%20MATERNITE%20CMU%20ACCART-VILLE.MP3time=224500) Interviewer: ok maintenant à quel trimestre de la grossesse ou l'âge gestationnel les femmes viennent à la première cpn? [00:03:51-7](http://localhost:2300/file=C:/Users/USER/Desktop/ENTRETIENS%20PREMATURITE/CSPS-ACCART-VILLE/RESPONSABLE%20MATERNITE%20CMU%20ACCART-VILLE.MP3time=231700)  [00:03:51-7](http://localhost:2300/file=C:/Users/USER/Desktop/ENTRETIENS%20PREMATURITE/CSPS-ACCART-VILLE/RESPONSABLE%20MATERNITE%20CMU%20ACCART-VILLE.MP3time=231700) Person 1: de façon general hein les femmes viennent tardivement, elles viennent tardivement, la plupart c'est après la seizième semaine voilà au deuxième trimestre [00:04:02-1](http://localhost:2300/file=C:/Users/USER/Desktop/ENTRETIENS%20PREMATURITE/CSPS-ACCART-VILLE/RESPONSABLE%20MATERNITE%20CMU%20ACCART-VILLE.MP3time=242100)  [00:04:02-1](http://localhost:2300/file=C:/Users/USER/Desktop/ENTRETIENS%20PREMATURITE/CSPS-ACCART-VILLE/RESPONSABLE%20MATERNITE%20CMU%20ACCART-VILLE.MP3time=242100) Interviewer: est ce que vous pensez que les femmes connaissent avec precision la date de leur dernière regle lors de leur premièr cpn? [00:04:10-6](http://localhost:2300/file=C:/Users/USER/Desktop/ENTRETIENS%20PREMATURITE/CSPS-ACCART-VILLE/RESPONSABLE%20MATERNITE%20CMU%20ACCART-VILLE.MP3time=250600)  [00:04:10-6](http://localhost:2300/file=C:/Users/USER/Desktop/ENTRETIENS%20PREMATURITE/CSPS-ACCART-VILLE/RESPONSABLE%20MATERNITE%20CMU%20ACCART-VILLE.MP3time=250600) Person 1: beaucoup ne connaissent pas avec precision, beaucoup ne connaissent pas donc generalement elles donnent en fonction des evènements sociaux qui se deroulent au sein de la communauté oubien au sein de leur famille à savoir les funerailles, les baptèmes les mariages oubien les fetes coutumières elles donnent en fonction de ça et que souvent on fait cette gynastique de calculer pour avoir à peu près quoi, à peu près, d'autres reconnaissent uniquement le mois mais le jour même elles ne connaissent pas , elles peuvent dire que c'est au mois de fevrier mais quel jour de fevrier elles savent pas. mais rares sont celles qui connaissent avec exactitude la date exacte de leur dernière règle. [00:04:51-6](http://localhost:2300/file=C:/Users/USER/Desktop/ENTRETIENS%20PREMATURITE/CSPS-ACCART-VILLE/RESPONSABLE%20MATERNITE%20CMU%20ACCART-VILLE.MP3time=291600) |
| --- |

| 00:03:17-2 Interviewer: Donc nous allons toujours poursuivre, maintenant sur une échelle de 1 à 10 femmes qui se présentent à leur première CPN, combien connaissent leurs dates des dernières règles? 00:03:28-3  00:03:28-3 Person 1: Donc très peu connaissent leur date des dernières règles, très très peu, donc si je veux donner une estimation, je peux dire trois (3), trois même je serai un peu plus large, donc en moyenne trois vont connaître la date des dernières règles. 00:03:44-5  00:03:44-5 Interviewer: OK, maintenant à quel trimestre de la grossesse ou à quel âge gestationnel les femmes viennent-elles à la première CPN? 00:03:51-7  00:03:51-7 Person 1: De façon générale, hein, les femmes viennent tardivement, elles viennent tardivement, la plupart c'est après la seizième semaine, voilà, au deuxième trimestre. 00:04:02-1  00:04:02-1 Interviewer: Est-ce que vous pensez que les femmes connaissent avec précision la date de leur dernière règle lors de leur première CPN? 00:04:10-6  00:04:10-6 Person 1: Beaucoup ne connaissent pas avec précision, beaucoup ne connaissent pas donc généralement elles donnent en fonction des événements sociaux qui se déroulent au sein de la communauté ou bien au sein de leur famille, à savoir les funérailles, les baptêmes, les mariages ou bien les fêtes coutumières. Elles donnent en fonction de ça et que souvent on fait cette gymnastique de calculer pour avoir à peu près quoi, à peu près. D'autres reconnaissent uniquement le mois mais le jour même elles ne connaissent pas, elles peuvent dire que c'est au mois de février mais quel jour de février, elles ne savent pas. Mais rares sont celles qui connaissent avec exactitude la date exacte de leur dernière règle. 00:04:51-6 |
| --- |

| **[00:03:17-2] Interviewer**: Let's continue. On a scale from 1 to 10, among women who attend their first antenatal care (ANC) appointment, how many are aware of the date of their last menstrual period?  **[00:03:28-3] Person 1**: Very few actually know their last period date, very few. To give an estimate, I would say about three out of ten. Three is a generous estimate, so on average, it's around three women who know the date of their last period.  **[00:03:44-5] Interviewer**: Okay, now at what trimester of pregnancy or gestational age do women typically come for their first ANC visit?  **[00:03:51-7] Person 1**: Generally speaking, women come late for their first visit. Most of them arrive after the sixteenth week, so they're in the second trimester.  **[00:04:02-1] Interviewer**: Do you think that women accurately know the date of their last period when they have their first ANC visit?  **[00:04:10-6] Person 1**: Many don't know the exact date. They often reference it according to social events within the community or their family, like funerals, baptisms, weddings, or customary celebrations. We then do some calculations to estimate the date. Some may recall the month, like February, but not the specific day. Only a few people know the exact date of their last period. |
| --- |

### Focus group of Do medical center with surgical branch

| [00:05:37-8](http://localhost:2300/file=C:/Users/USER/Desktop/ENTRETIENS%20PREMATURITE/CMA-DO/FOCUS%20GROUPE%20CMA-DO.mp3time=337800) Interviewer: Maintenant, si on prend une échelle de 1 à 10, sur 10 femmes qui se présentent à leur prémière CPN, combien de femmes connaissent la date de leur dernière règle? L'estimation sur une échelle de 1 à 10. [00:05:57-1](http://localhost:2300/file=C:/Users/USER/Desktop/ENTRETIENS%20PREMATURITE/CMA-DO/FOCUS%20GROUPE%20CMA-DO.mp3time=357100)  [00:05:57-1](http://localhost:2300/file=C:/Users/USER/Desktop/ENTRETIENS%20PREMATURITE/CMA-DO/FOCUS%20GROUPE%20CMA-DO.mp3time=357100) Person 6: Dans nos comtextes, ça ne depasse pas 2/10.Dans nos comtextes, c'est compliqué. [00:06:07-2](http://localhost:2300/file=C:/Users/USER/Desktop/ENTRETIENS%20PREMATURITE/CMA-DO/FOCUS%20GROUPE%20CMA-DO.mp3time=367200)  [00:06:07-2](http://localhost:2300/file=C:/Users/USER/Desktop/ENTRETIENS%20PREMATURITE/CMA-DO/FOCUS%20GROUPE%20CMA-DO.mp3time=367200) Interviewer: Généralement, à quel trimestre gestationnel, les femmes viennent à leur prémière CPN? [00:06:14-2](http://localhost:2300/file=C:/Users/USER/Desktop/ENTRETIENS%20PREMATURITE/CMA-DO/FOCUS%20GROUPE%20CMA-DO.mp3time=374200)  [00:06:14-2](http://localhost:2300/file=C:/Users/USER/Desktop/ENTRETIENS%20PREMATURITE/CMA-DO/FOCUS%20GROUPE%20CMA-DO.mp3time=374200) Person 6: À l'âge gestationnel, il y a beaucoup de femmes qui viennent à 13 semaines.ça se sont les gens de la ville, tu vois, il y en a qui viennent juste pour prendre le sachet (rire). Mais la majorité vient au premier trimestre. [00:06:35-8](http://localhost:2300/file=C:/Users/USER/Desktop/ENTRETIENS%20PREMATURITE/CMA-DO/FOCUS%20GROUPE%20CMA-DO.mp3time=395800)  [00:06:35-8](http://localhost:2300/file=C:/Users/USER/Desktop/ENTRETIENS%20PREMATURITE/CMA-DO/FOCUS%20GROUPE%20CMA-DO.mp3time=395800) Interviewer: Est-ce que vous pensez que les femmes qui viennent à leur première CPN, connaissent avec précision la date des dernières règles? [00:06:47-0](http://localhost:2300/file=C:/Users/USER/Desktop/ENTRETIENS%20PREMATURITE/CMA-DO/FOCUS%20GROUPE%20CMA-DO.mp3time=407000)  [00:06:47-0](http://localhost:2300/file=C:/Users/USER/Desktop/ENTRETIENS%20PREMATURITE/CMA-DO/FOCUS%20GROUPE%20CMA-DO.mp3time=407000) Person 6: Non. [00:06:50-6](http://localhost:2300/file=C:/Users/USER/Desktop/ENTRETIENS%20PREMATURITE/CMA-DO/FOCUS%20GROUPE%20CMA-DO.mp3time=410600)  [00:06:50-6](http://localhost:2300/file=C:/Users/USER/Desktop/ENTRETIENS%20PREMATURITE/CMA-DO/FOCUS%20GROUPE%20CMA-DO.mp3time=410600)Interviewer: Est-ce qu'il y en a qui peuvent connaitre? [00:06:52-5](http://localhost:2300/file=C:/Users/USER/Desktop/ENTRETIENS%20PREMATURITE/CMA-DO/FOCUS%20GROUPE%20CMA-DO.mp3time=412500)  [00:06:52-5](http://localhost:2300/file=C:/Users/USER/Desktop/ENTRETIENS%20PREMATURITE/CMA-DO/FOCUS%20GROUPE%20CMA-DO.mp3time=412500) Person 2: Oui, il y en a qui connaissent. [00:06:55-1](http://localhost:2300/file=C:/Users/USER/Desktop/ENTRETIENS%20PREMATURITE/CMA-DO/FOCUS%20GROUPE%20CMA-DO.mp3time=415100)  [00:06:55-1](http://localhost:2300/file=C:/Users/USER/Desktop/ENTRETIENS%20PREMATURITE/CMA-DO/FOCUS%20GROUPE%20CMA-DO.mp3time=415100) Interviewer: Mais sur une échelle de 1 à 10, elles peuvent pas valoir combien? [00:07:02-8](http://localhost:2300/file=C:/Users/USER/Desktop/ENTRETIENS%20PREMATURITE/CMA-DO/FOCUS%20GROUPE%20CMA-DO.mp3time=422800)  [00:07:02-8](http://localhost:2300/file=C:/Users/USER/Desktop/ENTRETIENS%20PREMATURITE/CMA-DO/FOCUS%20GROUPE%20CMA-DO.mp3time=422800) Person 6: Parce que les femmes qui connaissent, la majorité, ce sont les femmes qui sont en desir de grossesse. Celles qui sont en desir de grossesse, Quand enfin il y a des phases seulement, elles calculent, puisqu'elles calculent déjà l'ovulation. Donc la majorité, ce sont ses femmes qui peuvent connaitre. Mais les multipares, eux c'est un difficile. Sinon on ne peut pas depasser les 2 là, les 2/10. [00:07:31-6](http://localhost:2300/file=C:/Users/USER/Desktop/ENTRETIENS%20PREMATURITE/CMA-DO/FOCUS%20GROUPE%20CMA-DO.mp3time=451600) |
| --- |

| 00:05:37-8 Interviewer: Maintenant, si on prend une échelle de 1 à 10, sur 10 femmes qui se présentent à leur première CPN, combien de femmes connaissent la date de leur dernière règle ? L'estimation sur une échelle de 1 à 10. 00:05:57-1  00:05:57-1 Person 6: Dans nos contextes, ça ne dépasse pas 2/10. Dans nos contextes, c'est compliqué. 00:06:07-2  00:06:07-2 Interviewer: Généralement, à quel trimestre gestationnel, les femmes viennent à leur première CPN ? 00:06:14-2  00:06:14-2 Person 6: À l'âge gestationnel, il y a beaucoup de femmes qui viennent à 13 semaines. Ça, ce sont les gens de la ville, tu vois, il y en a qui viennent juste pour prendre le sachet (rire). Mais la majorité vient au premier trimestre. 00:06:35-8  00:06:35-8 Interviewer: Est-ce que vous pensez que les femmes qui viennent à leur première CPN, connaissent avec précision la date des dernières règles ? 00:06:47-0  00:06:47-0 Person 6: Non. 00:06:50-6  00:06:50-6 Interviewer: Est-ce qu'il y en a qui peuvent connaître ? 00:06:52-5  00:06:52-5 Person 2: Oui, il y en a qui connaissent. 00:06:55-1  00:06:55-1 Interviewer: Mais sur une échelle de 1 à 10, elles peuvent pas valoir combien ? 00:07:02-8  00:07:02-8 Person 6: Parce que les femmes qui connaissent, la majorité, ce sont les femmes qui sont en désir de grossesse. Celles qui sont en désir de grossesse, quand enfin il y a des phases seulement, elles calculent, puisqu'elles calculent déjà l'ovulation. Donc la majorité, ce sont ses femmes qui peuvent connaître. Mais les multipares, eux c'est difficile. Sinon on ne peut pas dépasser les 2 là, les 2/10. 00:07:31-6 |
| --- |

| **[00:05:37-8] Interviewer**: On a scale from 1 to 10, among 10 women who attend their first antenatal care (ANC) appointment, how many know the date of their last menstrual period?  **[00:05:57-1] Person 6**: In our context, it's no more than 2 out of 10. It's quite complicated here.  **[00:06:07-2] Interviewer**: Generally, in which gestational trimester do women come for their first ANC?  **[00:06:14-2] Person 6**: Many women come at 13 weeks, especially those in urban areas. Some come just for the maternity bag (laughing). But the majority do come in the first trimester.  **[00:06:35-8] Interviewer**: Do you think women who come for their first ANC accurately know the date of their last period?  **[00:06:47-0] Person 6**: No.  **[00:06:50-6] Interviewer**: Are there any who do know?  **[00:06:52-5] Person 2**: Yes, some do know.  **[00:06:55-1] Interviewer**: But on a scale of 1 to 10, how many would that be?  **[00:07:02-8] Person 6**: Those who typically know are women who are actively trying to conceive. They monitor their ovulation, so they're more likely to know. But for multiparous women, it's less common. We can't really exceed 2 out of 10. |
| --- |

### Gynecologist 1

| [00:03:35-0](http://localhost:2300/file=C:/Users/USER/Desktop/ENTRETIENS%20PREMATURITE/CMA-DO/GENYCOLOGUE%2001.mp3time=215000) Interviewer: Maintenant si on prend sur une échelle de 1 à 10, sur 10 femmes qui viennent à leure prémière CPN, combien connaissent la date de leur dernière règle ? [00:03:47-1](http://localhost:2300/file=C:/Users/USER/Desktop/ENTRETIENS%20PREMATURITE/CMA-DO/GENYCOLOGUE%2001.mp3time=227100)  [00:03:47-1](http://localhost:2300/file=C:/Users/USER/Desktop/ENTRETIENS%20PREMATURITE/CMA-DO/GENYCOLOGUE%2001.mp3time=227100) Person 1: Sur 10 femmes qui viennent à la CPN, c'est rare, extrêmement rare, peut être une ou deux femmes qui connaissent la date de leur dernière règle. [00:03:55-6](http://localhost:2300/file=C:/Users/USER/Desktop/ENTRETIENS%20PREMATURITE/CMA-DO/GENYCOLOGUE%2001.mp3time=235600)  [00:03:55-6](http://localhost:2300/file=C:/Users/USER/Desktop/ENTRETIENS%20PREMATURITE/CMA-DO/GENYCOLOGUE%2001.mp3time=235600) Interviewer: Généralement à quel trimestre de la grossesse elles viennent pour la plupart à la première CPN ? [00:04:02-5](http://localhost:2300/file=C:/Users/USER/Desktop/ENTRETIENS%20PREMATURITE/CMA-DO/GENYCOLOGUE%2001.mp3time=242500)  [00:04:02-5](http://localhost:2300/file=C:/Users/USER/Desktop/ENTRETIENS%20PREMATURITE/CMA-DO/GENYCOLOGUE%2001.mp3time=242500) Person 1: C'est vrai qu'ici, nous ne faisons pas beaucoup de CPN, mais les quelques cas que nous faisons, géneralement les femmes viennent autour de 5 mois, au deuxième trimestre .Sauf si elles ont des problèmes, sinon c'est très rare de voir au premier trimestre. [00:04:19-5](http://localhost:2300/file=C:/Users/USER/Desktop/ENTRETIENS%20PREMATURITE/CMA-DO/GENYCOLOGUE%2001.mp3time=259500)  [00:04:19-5](http://localhost:2300/file=C:/Users/USER/Desktop/ENTRETIENS%20PREMATURITE/CMA-DO/GENYCOLOGUE%2001.mp3time=259500) Interviewer: Est-ce que vous pensez que les femmes connaissent avec précision la date de leur dernière règle ? [00:04:24-2](http://localhost:2300/file=C:/Users/USER/Desktop/ENTRETIENS%20PREMATURITE/CMA-DO/GENYCOLOGUE%2001.mp3time=264200)  [00:04:24-2](http://localhost:2300/file=C:/Users/USER/Desktop/ENTRETIENS%20PREMATURITE/CMA-DO/GENYCOLOGUE%2001.mp3time=264200) Person 1: Oui, il y en a certaines qui connaissent. Vous voyez, actuellement, il y a les téléphones portables avec des applications,certaines , surtout les jeunes filles,elles notent régulièrement quand elles voient les règles. Donc quand elles ont un retard avec cette application, elles arrivent à nous donner précisément la date de leur dernière règle. [00:04:42-6](http://localhost:2300/file=C:/Users/USER/Desktop/ENTRETIENS%20PREMATURITE/CMA-DO/GENYCOLOGUE%2001.mp3time=282600) |
| --- |

| 00:03:35-0 Interviewer: Maintenant si on prend sur une échelle de 1 à 10, sur 10 femmes qui viennent à leur première CPN, combien connaissent la date de leur dernière règle ? 00:03:47-1  00:03:47-1 Person 1: Sur 10 femmes qui viennent à la CPN, c'est rare, extrêmement rare, peut-être une ou deux femmes qui connaissent la date de leur dernière règle. 00:03:55-6  00:03:55-6 Interviewer: Généralement à quel trimestre de la grossesse elles viennent pour la plupart à la première CPN ? 00:04:02-5  00:04:02-5 Person 1: C'est vrai qu'ici, nous ne faisons pas beaucoup de CPN, mais les quelques cas que nous faisons, généralement les femmes viennent autour de 5 mois, au deuxième trimestre. Sauf si elles ont des problèmes, sinon c'est très rare de voir au premier trimestre. 00:04:19-5  00:04:19-5 Interviewer: Est-ce que vous pensez que les femmes connaissent avec précision la date de leur dernière règle ? 00:04:24-2  00:04:24-2 Person 1: Oui, il y en a certaines qui connaissent. Vous voyez, actuellement, il y a les téléphones portables avec des applications, certaines, surtout les jeunes filles, elles notent régulièrement quand elles voient les règles. Donc quand elles ont un retard avec cette application, elles arrivent à nous donner précisément la date de leur dernière règle. 00:04:42-6 |
| --- |

| **[00:03:35-0] Interviewer**: On a scale of 1 to 10, among 10 women who come for their first antenatal care (ANC) appointment, how many know the date of their last menstrual period?  **[00:03:47-1] Person 1**: It's quite rare. Out of 10 women who come for ANC, maybe only one or two know the date of their last period.  **[00:03:55-6] Interviewer**: Generally, in what trimester of pregnancy do most women come for their first ANC?  **[00:04:02-5] Person 1**: Although we don’t have a large number of ANC visits, the women who do come usually arrive around 5 months, in the second trimester. It's very rare to see them in the first trimester unless they are experiencing problems.  **[00:04:19-5] Interviewer**: Do you think women accurately know the date of their last period?  **[00:04:24-2] Person 1**: Yes, some do, especially the younger girls. Nowadays, with cell phones and apps, they regularly track their periods. So, when they're late, they can precisely provide the date of their last period using the app. |
| --- |

### Gynecologist 2

| [00:03:00-1](http://localhost:2300/file=C:/Users/USER/Desktop/ENTRETIENS%20PREMATURITE/CMA-DO/GENYCOLOGUE%2002.mp3time=180100) Interviewer: Maintenant, si on prend sur une échelle de 1 à 10, par exemple sur 10 femmes qui se présentent à leur première CPN, combien connaissent la date des dernières règles? [00:03:10-7](http://localhost:2300/file=C:/Users/USER/Desktop/ENTRETIENS%20PREMATURITE/CMA-DO/GENYCOLOGUE%2002.mp3time=190700)  [00:03:10-7](http://localhost:2300/file=C:/Users/USER/Desktop/ENTRETIENS%20PREMATURITE/CMA-DO/GENYCOLOGUE%2002.mp3time=190700) Person 1: C'est difficile à dire, mais très peu connaissent la date des dernières règles. De façon classique, on voit les femmes, bon, parce qu'elles ont des systèmes de calculs qui sont un peu différents de notre façon de calculer. Ils vont dire que ça remonte peut-être à la semaine dernière, quand on va dire que bon, en termes de semaine déjà c'est encore précis. Ils vont rattacher ça à un événement donné. Donc souvent, c'est difficile. On dit par exemple que ça remonte au mois de Ramadan ou bien ça remonte à, c'était le lendemain de la tabassi ou bien c'etait le lendemain de tel fête donc ça, c'est un peu souvent difficile à calculer quand on n'a pas tous les calendriers qu'il faut. [00:03:49-5](http://localhost:2300/file=C:/Users/USER/Desktop/ENTRETIENS%20PREMATURITE/CMA-DO/GENYCOLOGUE%2002.mp3time=229500)  [00:03:49-5](http://localhost:2300/file=C:/Users/USER/Desktop/ENTRETIENS%20PREMATURITE/CMA-DO/GENYCOLOGUE%2002.mp3time=229500) Interviewer: Sur une échelle de 1 à 10, combien à peu près connaissent la date de dernières règles? [00:03:54-3](http://localhost:2300/file=C:/Users/USER/Desktop/ENTRETIENS%20PREMATURITE/CMA-DO/GENYCOLOGUE%2002.mp3time=234300)  [00:03:54-3](http://localhost:2300/file=C:/Users/USER/Desktop/ENTRETIENS%20PREMATURITE/CMA-DO/GENYCOLOGUE%2002.mp3time=234300) Person 1: Je dirais 3 ou 4. Ce n'est pas beaucoup. [00:04:03-2](http://localhost:2300/file=C:/Users/USER/Desktop/ENTRETIENS%20PREMATURITE/CMA-DO/GENYCOLOGUE%2002.mp3time=243200)  [00:04:03-2](http://localhost:2300/file=C:/Users/USER/Desktop/ENTRETIENS%20PREMATURITE/CMA-DO/GENYCOLOGUE%2002.mp3time=243200) Interviewer: A quel âge de la grossesse, elles viennent généralement à leurs premières CPN? [00:04:08-3](http://localhost:2300/file=C:/Users/USER/Desktop/ENTRETIENS%20PREMATURITE/CMA-DO/GENYCOLOGUE%2002.mp3time=248300)  [00:04:08-3](http://localhost:2300/file=C:/Users/USER/Desktop/ENTRETIENS%20PREMATURITE/CMA-DO/GENYCOLOGUE%2002.mp3time=248300) Person 1: Ça c'est variable, mais en moyenne les femmes viennent dans les 3 premiers mois. [00:04:17-7](http://localhost:2300/file=C:/Users/USER/Desktop/ENTRETIENS%20PREMATURITE/CMA-DO/GENYCOLOGUE%2002.mp3time=257700) |
| --- |

| 00:03:00-1 Interviewer: Maintenant, si on prend sur une échelle de 1 à 10, par exemple sur 10 femmes qui se présentent à leur première CPN, combien connaissent la date des dernières règles? 00:03:10-7  00:03:10-7 Person 1: C'est difficile à dire, mais très peu connaissent la date des dernières règles. De façon classique, on voit les femmes, bon, parce qu'elles ont des systèmes de calculs qui sont un peu différents de notre façon de calculer. Elles vont dire que ça remonte peut-être à la semaine dernière, quand on va dire que bon, en termes de semaine déjà c'est encore précis. Elles vont rattacher ça à un événement donné. Donc souvent, c'est difficile. On dit par exemple que ça remonte au mois de Ramadan ou bien ça remonte à, c'était le lendemain de la Tabaski ou bien c'était le lendemain de telle fête donc ça, c'est un peu souvent difficile à calculer quand on n'a pas tous les calendriers qu'il faut. 00:03:49-5  00:03:49-5 Interviewer: Sur une échelle de 1 à 10, combien à peu près connaissent la date des dernières règles? 00:03:54-3  00:03:54-3 Person 1: Je dirais 3 ou 4. Ce n'est pas beaucoup. 00:04:03-2  00:04:03-2 Interviewer: À quel âge de la grossesse, elles viennent généralement à leur première CPN? 00:04:08-3  00:04:08-3 Person 1: Ça c'est variable, mais en moyenne les femmes viennent dans les 3 premiers mois. 00:04:17-7 |
| --- |

| **[00:03:00-1] Interviewer**: On a scale from 1 to 10, out of 10 women who come for their first antenatal care (ANC), how many know the date of their last menstrual period?  **[00:03:10-7] Person 1**: It's hard to give an exact number, but very few are aware of the exact date of their last period. The women often have different systems of calculation compared to ours. They might reference it to a recent week, which is still relatively vague. They often link it to specific events, saying it was around the month of Ramadan or the day after a particular celebration. This makes it challenging to calculate accurately without all the necessary calendars.  **[00:03:49-5] Interviewer**: On a scale of 1 to 10, approximately how many women know the date of their last period?  **[00:03:54-3] Person 1**: I would estimate about 3 or 4 out of 10. It's not a large number.  **[00:04:03-2] Interviewer**: At what gestational age do women typically come for their first antenatal care (ANC) visit?  **[00:04:08-3] Person 1**: It varies, but on average, women tend to come within the first 3 months of their pregnancy. |
| --- |

### Chief physician of Do health district

| [00:03:52-2](http://localhost:2300/file=C:/Users/USER/Desktop/ENTRETIENS%20PREMATURITE/CMA-DO/MCD-DO.mp3time=232200) Interviewer: Nous allons partir sur une échelle de 1 à 10. Sur 10 femmes qui se présentent à leur première CPN, combien connaissent la date des dernières règles? [00:04:02-6](http://localhost:2300/file=C:/Users/USER/Desktop/ENTRETIENS%20PREMATURITE/CMA-DO/MCD-DO.mp3time=242600)  00:04:02-6 Person 1: Je risque de mentir. Je ne connais pas cette information, donc c'est difficile. Mais ce qu'on sait, c'est que si on part du principe que la plupart de la population, une grande partie de la population est alphabète, donc cette tranche de la population aussi probablement n'aura pas les outils pour noter la date des dernières règles. Ce n'est pas évident de garder dans sa tête pour une longue période. Moi je peux dire que par extrapolation, la moitié peut ne pas connaître. Mais je n'ai pas d'outils fiables pour dire ça. On aurait pu regarder dans les registres. A travers une collecte ça peut nous dire clairement. [00:04:52-0](http://localhost:2300/file=C:/Users/USER/Desktop/ENTRETIENS%20PREMATURITE/CMA-DO/MCD-DO.mp3time=292000)  [00:04:52-0](http://localhost:2300/file=C:/Users/USER/Desktop/ENTRETIENS%20PREMATURITE/CMA-DO/MCD-DO.mp3time=292000) Interviewer: Généralement, à quel âge gestationnel est-ce que les femmes viennent à leur première CPN? [00:04:59-6](http://localhost:2300/file=C:/Users/USER/Desktop/ENTRETIENS%20PREMATURITE/CMA-DO/MCD-DO.mp3time=299600)  [00:04:59-6](http://localhost:2300/file=C:/Users/USER/Desktop/ENTRETIENS%20PREMATURITE/CMA-DO/MCD-DO.mp3time=299600) Person 1: Si je prends les données de cette année pour l'ensemble du district, vous voyez que la proportion de femmes vues au premier trimestre pour la CPN1 là. C'est 48,8%. Donc en gros, la moitié sont là dans le premier trimestre. C'est ce qui est souhaité. Donc la moitié des femmes viennent au premier trimestre pour qu'on puisse regarder. Parce qu'au délà de cela, c'est vrai que c'est une CPN, une première consultation, mais elle est tardive pour nous. [00:05:40-7](http://localhost:2300/file=C:/Users/USER/Desktop/ENTRETIENS%20PREMATURITE/CMA-DO/MCD-DO.mp3time=340700)  [00:05:40-7](http://localhost:2300/file=C:/Users/USER/Desktop/ENTRETIENS%20PREMATURITE/CMA-DO/MCD-DO.mp3time=340700) Interviewer: Maintenant, par rapport à celles qui viennent à leur première CPN, est-ce que vous pensez qu'elles connaissent avec précision la date de leur dernière règle? [00:05:48-7](http://localhost:2300/file=C:/Users/USER/Desktop/ENTRETIENS%20PREMATURITE/CMA-DO/MCD-DO.mp3time=348700)  [00:05:48-7](http://localhost:2300/file=C:/Users/USER/Desktop/ENTRETIENS%20PREMATURITE/CMA-DO/MCD-DO.mp3time=348700) Person 1: Oui, celles qui connaissent avec précision connaissent. Puisqu'elle vont te dire, c'est telle date, elles notent. C'est ce que je disais, il y a des femmes qui notent. Elles ont des carnets,pas forcement un carnet de santé, elles ont des trucs, quand elle est en règle, elle note. Mais aujourd'hui, ça devient de plus en plus facile parce qu'il y a des applications. Elles mettent dedans, aux prochaines règles tu valident. Pour chaque règle tu peut valider, ça permet de te suivre et de dire que la période possible de fécondité. Il y a des applications qui sont là. Mais encore, il faut savoir lire et écrire. C'est pour ça que je disais que les lettrées, on trouve beaucoup plus la chance qu'elles retiennent parce qu'elles savent même la date. Pour quelqu'un qui ne sait ni lire et ni écrire, ce n'est pas évident de se rémémorer pour trouver la date. Donc, c'est ça la difficulté. [00:06:48-4](http://localhost:2300/file=C:/Users/USER/Desktop/ENTRETIENS%20PREMATURITE/CMA-DO/MCD-DO.mp3time=408400)  [00:06:48-4](http://localhost:2300/file=C:/Users/USER/Desktop/ENTRETIENS%20PREMATURITE/CMA-DO/MCD-DO.mp3time=408400) Interviewer: Maintenant, quand vous parlez d'applications, vous êtes allusion à quel genre d'application? [00:06:52-4](http://localhost:2300/file=C:/Users/USER/Desktop/ENTRETIENS%20PREMATURITE/CMA-DO/MCD-DO.mp3time=412400)  [00:06:52-4](http://localhost:2300/file=C:/Users/USER/Desktop/ENTRETIENS%20PREMATURITE/CMA-DO/MCD-DO.mp3time=412400) Person 1: Non, je dis qu'il y a des applications qui sont là actuellement. Ce sont des outils pratiques qui sont là, qui aident la personne sans même venir forcément dans un centre de santé. Voilà, ça c'est pour les règles. Pour connaitre la date des règles. Les applications là peuvent même dire, en fonction, si on a un cycle stable, l'application peut même dire que les prochaines règles sont telles dates. Ça permet à la femme, quand elle s'approche de cette période, de prendre les trucs en main, son coton, les trucs là, de se préparer, vraiment à accueillir les règles. Ce sont des applications qui sont là. C'est usuelle. [00:07:28-8](http://localhost:2300/file=C:/Users/USER/Desktop/ENTRETIENS%20PREMATURITE/CMA-DO/MCD-DO.mp3time=448800)  [00:07:28-8](http://localhost:2300/file=C:/Users/USER/Desktop/ENTRETIENS%20PREMATURITE/CMA-DO/MCD-DO.mp3time=448800) Interviewer: Vous connaissez le nom d'une application? [00:07:32-1](http://localhost:2300/file=C:/Users/USER/Desktop/ENTRETIENS%20PREMATURITE/CMA-DO/MCD-DO.mp3time=452100)  [00:07:32-1](http://localhost:2300/file=C:/Users/USER/Desktop/ENTRETIENS%20PREMATURITE/CMA-DO/MCD-DO.mp3time=452100) Person 1: Oui, je vois ,quand on est connecter, on en voit trop. Mais moi, comme je ne suis pas une femme, je n'en fais pas. Sinon, je vois ça avec les femmes. [00:07:44-9](http://localhost:2300/file=C:/Users/USER/Desktop/ENTRETIENS%20PREMATURITE/CMA-DO/MCD-DO.mp3time=464900) |
| --- |

| 00:03:52-2 Interviewer: Nous allons partir sur une échelle de 1 à 10. Sur 10 femmes qui se présentent à leur première CPN, combien connaissent la date des dernières règles? 00:04:02-6  00:04:02-6 Person 1: Je risque de mentir. Je ne connais pas cette information, donc c'est difficile. Mais ce qu'on sait, c'est que si on part du principe que la plupart de la population, une grande partie de la population est alphabète, donc cette tranche de la population aussi probablement n'aura pas les outils pour noter la date des dernières règles. Ce n'est pas évident de garder dans sa tête pour une longue période. Moi, je peux dire que par extrapolation, la moitié peut ne pas connaître. Mais je n'ai pas d'outils fiables pour dire ça. On aurait pu regarder dans les registres. À travers une collecte, ça peut nous dire clairement. 00:04:52-0  00:04:52-0 Interviewer: Généralement, à quel âge gestationnel est-ce que les femmes viennent à leur première CPN? 00:04:59-6  00:04:59-6 Person 1: Si je prends les données de cette année pour l'ensemble du district, vous voyez que la proportion de femmes vues au premier trimestre pour la CPN1 là. C'est 48,8%. Donc en gros, la moitié sont là dans le premier trimestre. C'est ce qui est souhaité. Donc la moitié des femmes viennent au premier trimestre pour qu'on puisse regarder. Parce qu'au-delà de cela, c'est vrai que c'est une CPN, une première consultation, mais elle est tardive pour nous. 00:05:40-7  00:05:40-7 Interviewer: Maintenant, par rapport à celles qui viennent à leur première CPN, est-ce que vous pensez qu'elles connaissent avec précision la date de leur dernière règle? 00:05:48-7  00:05:48-7 Person 1: Oui, celles qui connaissent avec précision, connaissent. Puisqu'elles vont te dire, c'est telle date, elles notent. C'est ce que je disais, il y a des femmes qui notent. Elles ont des carnets, pas forcément un carnet de santé, elles ont des trucs, quand elle est en règle, elle note. Mais aujourd'hui, ça devient de plus en plus facile parce qu'il y a des applications. Elles mettent dedans, aux prochaines règles tu valides. Pour chaque règle, tu peux valider, ça permet de te suivre et de dire que la période possible de fécondité. Il y a des applications qui sont là. Mais encore, il faut savoir lire et écrire. C'est pour ça que je disais que les lettrées, on trouve beaucoup plus la chance qu'elles retiennent parce qu'elles savent même la date. Pour quelqu'un qui ne sait ni lire ni écrire, ce n'est pas évident de se rémémorer pour trouver la date. Donc, c'est ça la difficulté. 00:06:48-4  00:06:48-4 Interviewer: Maintenant, quand vous parlez d'applications, vous faites allusion à quel genre d'application? 00:06:52-4  00:06:52-4 Person 1: Non, je dis qu'il y a des applications qui sont là actuellement. Ce sont des outils pratiques qui sont là, qui aident la personne sans même venir forcément dans un centre de santé. 00:07:02-7  00:07:02-7 Person 1: Voilà, ça c'est pour les règles. Pour connaître la date des règles. Les applications là peuvent même dire, en fonction, si on a un cycle stable, l'application peut même dire que les prochaines règles sont telles dates. Ça permet à la femme, quand elle s'approche de cette période, de prendre les trucs en main, son coton, les trucs là, de se préparer, vraiment à accueillir les règles. Ce sont des applications qui sont là. C'est usuel. 00:07:28-8  00:07:28-8 Interviewer: Vous connaissez le nom d'une application? 00:07:32-1  00:07:32-1 Person 1: Oui, je vois, quand on est connecté, on en voit trop. Mais moi, comme je ne suis pas une femme, je n'en fais pas. Sinon, je vois ça avec les femmes. 00:07:44-9 |
| --- |

| **[00:03:35-0] Interviewer**: On a scale of 1 to 10, among 10 women who attend their first antenatal care (ANC) appointment, how many know the date of their last menstrual period?  **[00:04:02-6] Person 1**: It's hard to give a precise number. If we assume a large portion of the population is literate, they may not have the tools to record the date of their last periods. It's not easy to remember for a long time. By extrapolation, maybe half may not know, but I don't have solid data to confirm that. We could refer to the registers for more accurate information.  **[00:04:52-0] Interviewer**: At what gestational age do women typically come for their first ANC?  **[00:04:59-6] Person 1**: According to this year's data for our district, about 48.8% of women are seen in the first trimester for their first ANC. Ideally, we prefer to see them in the first trimester for early monitoring.  **[00:05:40-7] Interviewer**: Among those who attend their first ANC, do you think they precisely know the date of their last period?  **[00:05:48-7] Person 1**: Yes, those who are precise do know. They keep track, sometimes in notebooks or with mobile applications. These apps help them track their menstrual cycles and predict fertility periods. However, literacy is key, as educated women are more likely to remember because they can read and write.  **[00:06:48-4] Interviewer**: Can you elaborate on what kind of apps you're referring to?  **[00:06:52-4] Person 1**: There are practical mobile applications available now that assist in tracking menstrual cycles. They can predict the next period based on stable cycles, helping women prepare for their menstruation. These tools are becoming quite common.  **[00:07:28-8] Interviewer**: Do you know the name of any such app?  **[00:07:32-1] Person 1**: I see many online, but as I'm not a woman, I don’t use them. I am aware of them through interactions with women. |
| --- |

### Manager for maternity care unit of Do medical center with surgical branch

| [00:03:15-9](http://localhost:2300/file=C:/Users/USER/Desktop/ENTRETIENS%20PREMATURITE/CMA-DO/RESPONSABLE%20UNITE%20DE%20SOIN%20CMA-DO.mp3time=195900) Interviewer: D'accord. Maintenant, nous allons partir sur une échelle de 1 à 10. C'est-à-dire que sur 10 femmes qui se présentent à leur premiere CPN, combien connaissent la date de leur dernière règle? [00:03:28-3](http://localhost:2300/file=C:/Users/USER/Desktop/ENTRETIENS%20PREMATURITE/CMA-DO/RESPONSABLE%20UNITE%20DE%20SOIN%20CMA-DO.mp3time=208300)  [00:03:28-3](http://localhost:2300/file=C:/Users/USER/Desktop/ENTRETIENS%20PREMATURITE/CMA-DO/RESPONSABLE%20UNITE%20DE%20SOIN%20CMA-DO.mp3time=208300) Person 1: Une estimation, parce que c'est très faible. La plupart des femmes, quand on pose la question, elles vont vous dire qu'elles ne connaissent pas. Mais bon, il arrive parfois quand on essaie de titiller un peu la femme, elle va au moins nous situer, peut-être nous donner une période comme ça.Voilà, soit généralement elles aiment dire mois de carême, mois de telles fêtes, ainsi de suite. Sinon, vraiment, le nombre est vraiment très très faible. Mais peut-être que c'est nous aussi les prestataires, on n'arrive pas à aller un peu loin. Il arrive parfois, dès qu'on demande, quand elles nous disent qu'elles ne connaissent pas.Nous, on passe déjà sur la question. Peut-être ici, ça allait peut-être être mieux faite,mais si on insistait. On dit, essaye de faire le lien avec une période où peut-être tu as vu les règles et après tu n'as pas vu les règles.Essaye de titiller un peu la femme. Je pense qu'on va arriver. Mais la plupart du temps, la question que nous posons ne permet pas vraiment aux femmes de pouvoir nous répondre. Et faisons qu'on n'a pas un grand nombre. L'échelle, vous avez dit, c'est de combien à combien? [00:04:35-5](http://localhost:2300/file=C:/Users/USER/Desktop/ENTRETIENS%20PREMATURITE/CMA-DO/RESPONSABLE%20UNITE%20DE%20SOIN%20CMA-DO.mp3time=275500)  [00:04:35-5](http://localhost:2300/file=C:/Users/USER/Desktop/ENTRETIENS%20PREMATURITE/CMA-DO/RESPONSABLE%20UNITE%20DE%20SOIN%20CMA-DO.mp3time=275500) Interviewer: De 1 à 10. [00:04:36-3](http://localhost:2300/file=C:/Users/USER/Desktop/ENTRETIENS%20PREMATURITE/CMA-DO/RESPONSABLE%20UNITE%20DE%20SOIN%20CMA-DO.mp3time=276300)  [00:04:36-3](http://localhost:2300/file=C:/Users/USER/Desktop/ENTRETIENS%20PREMATURITE/CMA-DO/RESPONSABLE%20UNITE%20DE%20SOIN%20CMA-DO.mp3time=276300) Person 1: De 1 à 10. Bon, moi j'allais me retrouver à 4.Voilà, parce que je ne pourrai même pas donner la moitié des femmes. [00:04:45-8](http://localhost:2300/file=C:/Users/USER/Desktop/ENTRETIENS%20PREMATURITE/CMA-DO/RESPONSABLE%20UNITE%20DE%20SOIN%20CMA-DO.mp3time=285800)  [00:04:45-8](http://localhost:2300/file=C:/Users/USER/Desktop/ENTRETIENS%20PREMATURITE/CMA-DO/RESPONSABLE%20UNITE%20DE%20SOIN%20CMA-DO.mp3time=285800) Interviewer: Maintenant, généralement, à quel trimestre de la grossesse, elles viennent à leure première CPN? [00:04:51-7](http://localhost:2300/file=C:/Users/USER/Desktop/ENTRETIENS%20PREMATURITE/CMA-DO/RESPONSABLE%20UNITE%20DE%20SOIN%20CMA-DO.mp3time=291700)  [00:04:51-7](http://localhost:2300/file=C:/Users/USER/Desktop/ENTRETIENS%20PREMATURITE/CMA-DO/RESPONSABLE%20UNITE%20DE%20SOIN%20CMA-DO.mp3time=291700) Person 1: Bon, en fait, ça, ça dépend. Il y a des femmes qui vont venir plus tôt.Voilà, donc ça veut dire, dès qu'elles ont constaté par exemple qu'il y a un retard de règles, voilà, elles vont venir. Par contre, il y a d'autres aussi, il y a le retard de règles qui est là, elles vont rester. Peut-être nous revenir à 12 semaines, 14 semaines, sinon même plus. Voilà, mais pour la CPN, parce que la CPN se fait dès qu'on est en contact avec la femme. Ça veut dire, dès qu'elle vient, on a posé le diagnostic de grossesse, on peut être amené à commencer en même temps cette consultation-là. Voilà, mais bon, elles nous reviennent des fois un peu, en tout cas, après, généralement, la plupart du temps, à 12 semaines, sinon au-delà. [00:05:38-2](http://localhost:2300/file=C:/Users/USER/Desktop/ENTRETIENS%20PREMATURITE/CMA-DO/RESPONSABLE%20UNITE%20DE%20SOIN%20CMA-DO.mp3time=338200)  [00:05:38-2](http://localhost:2300/file=C:/Users/USER/Desktop/ENTRETIENS%20PREMATURITE/CMA-DO/RESPONSABLE%20UNITE%20DE%20SOIN%20CMA-DO.mp3time=338200) Interviewer: Quelle est la proportion sur une echelle de 1 à 10? [00:05:42-2](http://localhost:2300/file=C:/Users/USER/Desktop/ENTRETIENS%20PREMATURITE/CMA-DO/RESPONSABLE%20UNITE%20DE%20SOIN%20CMA-DO.mp3time=342200)  [00:05:42-2](http://localhost:2300/file=C:/Users/USER/Desktop/ENTRETIENS%20PREMATURITE/CMA-DO/RESPONSABLE%20UNITE%20DE%20SOIN%20CMA-DO.mp3time=342200) Person 1: Euh, on va prendre 5. Voilà, 5. [00:05:48-9](http://localhost:2300/file=C:/Users/USER/Desktop/ENTRETIENS%20PREMATURITE/CMA-DO/RESPONSABLE%20UNITE%20DE%20SOIN%20CMA-DO.mp3time=348900)  [00:05:48-9](http://localhost:2300/file=C:/Users/USER/Desktop/ENTRETIENS%20PREMATURITE/CMA-DO/RESPONSABLE%20UNITE%20DE%20SOIN%20CMA-DO.mp3time=348900) Interviewer: Est-ce que vous pensez qu'elles connaissent avec précision quand elles viennent à la première CPN, la date de leure dernière règle? [00:05:55-7](http://localhost:2300/file=C:/Users/USER/Desktop/ENTRETIENS%20PREMATURITE/CMA-DO/RESPONSABLE%20UNITE%20DE%20SOIN%20CMA-DO.mp3time=355700)  [00:05:55-7](http://localhost:2300/file=C:/Users/USER/Desktop/ENTRETIENS%20PREMATURITE/CMA-DO/RESPONSABLE%20UNITE%20DE%20SOIN%20CMA-DO.mp3time=355700) Person 1: Justement, c'est ça. Avec précision, la plupart du temps, comme elles vont te dire même qu'elles ne connaissent pas. Maintenant, celles qui arrivent à connaître, comme j'avais dit tantôt sur l'échelle, j'avais dit 4, ça veut dire que celles qui arrivent à donner là, donnent vraiment avec précision.Voilà. [00:06:13-9](http://localhost:2300/file=C:/Users/USER/Desktop/ENTRETIENS%20PREMATURITE/CMA-DO/RESPONSABLE%20UNITE%20DE%20SOIN%20CMA-DO.mp3time=373900)  [00:06:13-9](http://localhost:2300/file=C:/Users/USER/Desktop/ENTRETIENS%20PREMATURITE/CMA-DO/RESPONSABLE%20UNITE%20DE%20SOIN%20CMA-DO.mp3time=373900) Interviewer: Les pourcentages de celles qui arrivent à donner sur une échelle de 1 à 10. Lorsqu'ellesviennent pour leure première CPN? [00:06:19-3](http://localhost:2300/file=C:/Users/USER/Desktop/ENTRETIENS%20PREMATURITE/CMA-DO/RESPONSABLE%20UNITE%20DE%20SOIN%20CMA-DO.mp3time=379300)  [00:06:19-3](http://localhost:2300/file=C:/Users/USER/Desktop/ENTRETIENS%20PREMATURITE/CMA-DO/RESPONSABLE%20UNITE%20DE%20SOIN%20CMA-DO.mp3time=379300) Person 1: C'est les 4 là. Voilà. [00:06:23-6](http://localhost:2300/file=C:/Users/USER/Desktop/ENTRETIENS%20PREMATURITE/CMA-DO/RESPONSABLE%20UNITE%20DE%20SOIN%20CMA-DO.mp3time=383600) |
| --- |

| 00:03:15-9 Interviewer: D'accord. Maintenant, nous allons partir sur une échelle de 1 à 10. C'est-à-dire que sur 10 femmes qui se présentent à leur première CPN, combien connaissent la date de leur dernière règle ? 00:03:28-3  00:03:28-3 Person 1: Une estimation, parce que c'est très faible. La plupart des femmes, quand on pose la question, elles vont vous dire qu'elles ne connaissent pas. Mais bon, il arrive parfois quand on essaie de titiller un peu la femme, elle va au moins nous situer, peut-être nous donner une période comme ça. Voilà, soit généralement elles aiment dire mois de carême, mois de telles fêtes, ainsi de suite. Sinon, vraiment, le nombre est vraiment très très faible. Mais peut-être que c'est nous aussi les prestataires, on n'arrive pas à aller un peu loin. Il arrive parfois, dès qu'on demande, quand elles nous disent qu'elles ne connaissent pas, nous, on passe déjà sur la question. Peut-être ici, ça allait peut-être être mieux faite, mais si on insistait. On dit, essaye de faire le lien avec une période où peut-être tu as vu les règles et après tu n'as pas vu les règles. Essaye de titiller un peu la femme. Je pense qu'on va arriver. Mais la plupart du temps, la question que nous posons ne permet pas vraiment aux femmes de pouvoir nous répondre. Et faisons qu'on n'a pas un grand nombre. L'échelle, vous avez dit, c'est de combien à combien ? 00:04:35-5  00:04:35-5 Interviewer: De 1 à 10. 00:04:36-3  00:04:36-3 Person 1: De 1 à 10. Bon, moi j'allais me retrouver à 4. Voilà, parce que je ne pourrais même pas donner la moitié des femmes. 00:04:45-8  00:04:45-8 Interviewer: Maintenant, généralement, à quel trimestre de la grossesse, elles viennent à leur première CPN ? 00:04:51-7  00:04:51-7 Person 1: Bon, en fait, ça, ça dépend. Il y a des femmes qui vont venir plus tôt. Voilà, donc ça veut dire, dès qu'elles ont constaté par exemple qu'il y a un retard de règles, voilà, elles vont venir. Par contre, il y a d'autres aussi, il y a le retard de règles qui est là, elles vont rester. Peut-être nous revenir à 12 semaines, 14 semaines, sinon même plus. Voilà, mais pour la CPN, parce que la CPN se fait dès qu'on est en contact avec la femme. Ça veut dire, dès qu'elle vient, on a posé le diagnostic de grossesse, on peut être amené à commencer en même temps cette consultation-là. Voilà, mais bon, elles nous reviennent des fois un peu, en tout cas, après, généralement, la plupart du temps, à 12 semaines, sinon au-delà. 00:05:38-2  00:05:38-2 Interviewer: Quelle est la proportion sur une échelle de 1 à 10 ? 00:05:42-2  00:05:42-2 Person 1: Euh, on va prendre 5. Voilà, 5. 00:05:48-9  00:05:48-9 Interviewer: Est-ce que vous pensez qu'elles connaissent avec précision quand elles viennent à la première CPN, la date de leur dernière règle ? 00:05:55-7  00:05:55-7 Person 1: Justement, c'est ça. Avec précision, la plupart du temps, comme elles vont te dire même qu'elles ne connaissent pas. Maintenant, celles qui arrivent à connaître, comme j'avais dit tantôt sur l'échelle, j'avais dit 4, ça veut dire que celles qui arrivent à donner là, donnent vraiment avec précision. Voilà. 00:06:13-9 |
| --- |

| **[00:03:15-9] Interviewer**: On a scale of 1 to 10, out of 10 women who attend their first antenatal care (ANC) appointment, how many know the date of their last menstrual period?  **[00:03:28-3] Person 1**: It's hard to say exactly, as most women don't know the precise date. They often relate it to events like the month of Lent or other feasts. It's challenging to calculate accurately based on such references. Our questioning doesn't always enable them to provide an exact date, which results in a very low number. On a scale of 1 to 10, I would estimate about 4.  **[00:04:45-8] Interviewer**: Typically, in what trimester of pregnancy do they come for their first ANC?  **[00:04:51-7] Person 1**: It varies. Some women come early, as soon as they notice a missed period. Others wait until around 12 or 14 weeks, or even longer. We start the ANC as soon as we make a pregnancy diagnosis, which is often around 12 weeks.  **[00:05:38-2] Interviewer**: What proportion would you estimate on a scale of 1 to 10?  **[00:05:42-2] Person 1**: I'd say about 5.  **[00:05:48-9] Interviewer**: Do you think they know precisely the date of their last period when they come for their first ANC?  **[00:05:55-7] Person 1**: Most of the time, they don’t know precisely. Those who do manage to provide a date, as I mentioned earlier, are about 4 out of 10. |
| --- |

### Focus group of Colma1 health and social promotion center

| [00:04:54-2](http://localhost:2300/file=C:/Users/USER/Desktop/ENTRETIENS%20PREMATURITE/CSPS-COLMA1/FOCUS%20GROUPE%20COLMA1.mp3time=294200) Interviewer: Est-ce qu'il y a quelque chose d'autre à ajouterl ? On peut continuer.D'accord, maintenant si on part sur une échelle de un à 10, sur 10 femmes qui viennent à leur première CPN, combien connaissent la date de leure dernière règle ? Une estimation. [00:05:19-5](http://localhost:2300/file=C:/Users/USER/Desktop/ENTRETIENS%20PREMATURITE/CSPS-COLMA1/FOCUS%20GROUPE%20COLMA1.mp3time=319500)  [00:05:19-5](http://localhost:2300/file=C:/Users/USER/Desktop/ENTRETIENS%20PREMATURITE/CSPS-COLMA1/FOCUS%20GROUPE%20COLMA1.mp3time=319500) Person 5: Selon moi, ça dépassera pas 2; donc c'est 2 sur 10.qui connaissent leurs date des dernières règles. [00:05:29-1](http://localhost:2300/file=C:/Users/USER/Desktop/ENTRETIENS%20PREMATURITE/CSPS-COLMA1/FOCUS%20GROUPE%20COLMA1.mp3time=329100)  [00:05:29-1](http://localhost:2300/file=C:/Users/USER/Desktop/ENTRETIENS%20PREMATURITE/CSPS-COLMA1/FOCUS%20GROUPE%20COLMA1.mp3time=329100)Person 3: la date des dernières, elles ne peuvent pas donner une date précise comme ça. Voilà peut être elles peuvent parler de mois, < soit j'ai j'ai un mois de retard ou 2 mois de retard>. Généralement c'est comme ça, les femmes s'expriment voilà. [00:05:46-4](http://localhost:2300/file=C:/Users/USER/Desktop/ENTRETIENS%20PREMATURITE/CSPS-COLMA1/FOCUS%20GROUPE%20COLMA1.mp3time=346400)  [00:05:46-4](http://localhost:2300/file=C:/Users/USER/Desktop/ENTRETIENS%20PREMATURITE/CSPS-COLMA1/FOCUS%20GROUPE%20COLMA1.mp3time=346400) Interviewer: géneralement à quel âge gestationnel les femmes viennent à leure prémière CPN? [00:05:51-4](http://localhost:2300/file=C:/Users/USER/Desktop/ENTRETIENS%20PREMATURITE/CSPS-COLMA1/FOCUS%20GROUPE%20COLMA1.mp3time=351400)  [00:05:51-4](http://localhost:2300/file=C:/Users/USER/Desktop/ENTRETIENS%20PREMATURITE/CSPS-COLMA1/FOCUS%20GROUPE%20COLMA1.mp3time=351400) Person 3: bon on va laisser les dames de la CPN repondre. [00:05:55-6](http://localhost:2300/file=C:/Users/USER/Desktop/ENTRETIENS%20PREMATURITE/CSPS-COLMA1/FOCUS%20GROUPE%20COLMA1.mp3time=355600)  [00:05:55-6](http://localhost:2300/file=C:/Users/USER/Desktop/ENTRETIENS%20PREMATURITE/CSPS-COLMA1/FOCUS%20GROUPE%20COLMA1.mp3time=355600) Person 5: La CPN, que vos dames là viennent à combien de mois la plupart ? elles commencent à combien de mois ? [00:06:05-1](http://localhost:2300/file=C:/Users/USER/Desktop/ENTRETIENS%20PREMATURITE/CSPS-COLMA1/FOCUS%20GROUPE%20COLMA1.mp3time=365100)  [00:06:05-1](http://localhost:2300/file=C:/Users/USER/Desktop/ENTRETIENS%20PREMATURITE/CSPS-COLMA1/FOCUS%20GROUPE%20COLMA1.mp3time=365100) Person 6: La plupart du temps, ça commence au 2ème et 3ème semaine, elles ne viennent pas tôt vraiment elles viennent pas tôt. [00:06:14-9](http://localhost:2300/file=C:/Users/USER/Desktop/ENTRETIENS%20PREMATURITE/CSPS-COLMA1/FOCUS%20GROUPE%20COLMA1.mp3time=374900)  [00:06:14-9](http://localhost:2300/file=C:/Users/USER/Desktop/ENTRETIENS%20PREMATURITE/CSPS-COLMA1/FOCUS%20GROUPE%20COLMA1.mp3time=374900) Interviewer: Et lorsqu'elles viennent à leure première CPN,est-ce qu'elle connaissent avec précision la date des dernières règles ? [00:06:20-9](http://localhost:2300/file=C:/Users/USER/Desktop/ENTRETIENS%20PREMATURITE/CSPS-COLMA1/FOCUS%20GROUPE%20COLMA1.mp3time=380900)  [00:06:20-9](http://localhost:2300/file=C:/Users/USER/Desktop/ENTRETIENS%20PREMATURITE/CSPS-COLMA1/FOCUS%20GROUPE%20COLMA1.mp3time=380900) Person 6: Non elles ne connaissent pas avec precision, c'est le mois qu'elles retiennent voilà. Maintenant on essaie de calculer à peu près. [00:06:35-2](http://localhost:2300/file=C:/Users/USER/Desktop/ENTRETIENS%20PREMATURITE/CSPS-COLMA1/FOCUS%20GROUPE%20COLMA1.mp3time=395200)  [00:06:35-2](http://localhost:2300/file=C:/Users/USER/Desktop/ENTRETIENS%20PREMATURITE/CSPS-COLMA1/FOCUS%20GROUPE%20COLMA1.mp3time=395200) Interviewer: sur une echelle de 1 à 10 combien arrivent à donner cette date? [00:06:49-5](http://localhost:2300/file=C:/Users/USER/Desktop/ENTRETIENS%20PREMATURITE/CSPS-COLMA1/FOCUS%20GROUPE%20COLMA1.mp3time=409500)  [00:06:49-5](http://localhost:2300/file=C:/Users/USER/Desktop/ENTRETIENS%20PREMATURITE/CSPS-COLMA1/FOCUS%20GROUPE%20COLMA1.mp3time=409500) Person 6: 3 pas plus (rire) , 3 sur 10. [00:06:55-0](http://localhost:2300/file=C:/Users/USER/Desktop/ENTRETIENS%20PREMATURITE/CSPS-COLMA1/FOCUS%20GROUPE%20COLMA1.mp3time=415000)  [00:06:55-0](http://localhost:2300/file=C:/Users/USER/Desktop/ENTRETIENS%20PREMATURITE/CSPS-COLMA1/FOCUS%20GROUPE%20COLMA1.mp3time=415000) Person 2: La date exacte? [00:06:56-7](http://localhost:2300/file=C:/Users/USER/Desktop/ENTRETIENS%20PREMATURITE/CSPS-COLMA1/FOCUS%20GROUPE%20COLMA1.mp3time=416700)  [00:06:56-7](http://localhost:2300/file=C:/Users/USER/Desktop/ENTRETIENS%20PREMATURITE/CSPS-COLMA1/FOCUS%20GROUPE%20COLMA1.mp3time=416700) Person 1: on dit la date exacte, la DDR (date des dernières règles) exacte. [00:07:05-2](http://localhost:2300/file=C:/Users/USER/Desktop/ENTRETIENS%20PREMATURITE/CSPS-COLMA1/FOCUS%20GROUPE%20COLMA1.mp3time=425200)  [00:07:05-2](http://localhost:2300/file=C:/Users/USER/Desktop/ENTRETIENS%20PREMATURITE/CSPS-COLMA1/FOCUS%20GROUPE%20COLMA1.mp3time=425200) Person 3: Bon selon moi en tout cas, sur 10 là ça ne dépasse même pas 2 voilà une femme qui va venir te donner et la date exacte, là peut-être c'est une dame qui est instruite. Par exemple les travailleurs, voilà, c'est elle seule qui peuvent dire que j'ai vu mes dernières est tel jour sinon les toutes les autres te diront non c'est le mois passé un seul mois je l'ai pas vu mes règles, ça fait 2 mois je l'ai pas vu mes règles , voilà donc sur 10 en tout cas je pense que ces 2 personnes environ. [00:07:32-8](http://localhost:2300/file=C:/Users/USER/Desktop/ENTRETIENS%20PREMATURITE/CSPS-COLMA1/FOCUS%20GROUPE%20COLMA1.mp3time=452800)  [00:07:32-8](http://localhost:2300/file=C:/Users/USER/Desktop/ENTRETIENS%20PREMATURITE/CSPS-COLMA1/FOCUS%20GROUPE%20COLMA1.mp3time=452800) Interviewer: ok donc la precision c'est de l'ordre du mois? [00:07:35-6](http://localhost:2300/file=C:/Users/USER/Desktop/ENTRETIENS%20PREMATURITE/CSPS-COLMA1/FOCUS%20GROUPE%20COLMA1.mp3time=455600)  [00:07:35-6](http://localhost:2300/file=C:/Users/USER/Desktop/ENTRETIENS%20PREMATURITE/CSPS-COLMA1/FOCUS%20GROUPE%20COLMA1.mp3time=455600) Person 3: Voilà, C'est ça surtout. [00:07:38-2](http://localhost:2300/file=C:/Users/USER/Desktop/ENTRETIENS%20PREMATURITE/CSPS-COLMA1/FOCUS%20GROUPE%20COLMA1.mp3time=458200) |
| --- |

| 00:04:54-2 Interviewer: Est-ce qu'il y a quelque chose d'autre à ajouter ? On peut continuer. D'accord, maintenant si on part sur une échelle de un à 10, sur 10 femmes qui viennent à leur première CPN, combien connaissent la date de leur dernière règle ? Une estimation. 00:05:19-5  00:05:19-5 Person 5: Selon moi, ça ne dépassera pas 2; donc c'est 2 sur 10 qui connaissent leur date des dernières règles. 00:05:29-1  00:05:29-1 Person 3: La date des dernières règles, elles ne peuvent pas donner une date précise comme ça. Voilà, peut-être elles peuvent parler de mois, < soit j'ai un mois de retard ou 2 mois de retard>. Généralement c'est comme ça que les femmes s'expriment. Voilà. 00:05:46-4  00:05:46-4 Interviewer: Généralement à quel âge gestationnel les femmes viennent à leur première CPN ? 00:05:51-4  00:05:51-4 Person 3: Bon, on va laisser les dames de la CPN répondre. 00:05:55-6  00:05:55-6 Person 5: La CPN, que vos dames là viennent à combien de mois la plupart ? Elles commencent à combien de mois ? 00:06:05-1  00:06:05-1 Person 6: La plupart du temps, ça commence au 2ème et 3ème trimestre, elles ne viennent pas tôt vraiment. 00:06:14-9  00:06:14-9 Interviewer: Et lorsqu'elles viennent à leur première CPN, est-ce qu'elles connaissent avec précision la date des dernières règles ? 00:06:20-9  00:06:20-9 Person 6: Non, elles ne connaissent pas avec précision, c'est le mois qu'elles retiennent. Voilà. Maintenant, on essaie de calculer à peu près. 00:06:35-2  00:06:35-2 Interviewer: Sur une échelle de 1 à 10, combien arrivent à donner cette date ? 00:06:49-5  00:06:49-5 Person 6: 3, pas plus (rire), 3 sur 10. 00:06:55-0  00:06:55-0 Person 2: La date exacte ? 00:06:56-7  00:06:56-7 Person 1: On dit la date exacte, la DDR (date des dernières règles) exacte. 00:07:05-2  00:07:05-2 Person 3: Bon, selon moi, en tout cas, sur 10, ça ne dépasse même pas 2. Voilà, une femme qui va venir te donner la date exacte, là, peut-être c'est une dame qui est instruite. Par exemple, les travailleuses, voilà, c'est elle seule qui peuvent dire que j'ai vu mes dernières règles tel jour sinon toutes les autres te diront non c'est le mois passé un seul mois je ne l'ai pas vu mes règles, ça fait 2 mois je ne l'ai pas vu mes règles, voilà donc sur 10, en tout cas, je pense que ce sont 2 personnes environ. 00:07:32-8  00:07:32-8 Interviewer: Ok, donc la précision, c'est de l'ordre du mois ? 00:07:35-6  00:07:35-6 Person 3: Voilà, c'est ça surtout. 00:07:38-2 |
| --- |

| **[00:04:54-2] Interviewer**: Is there anything else to add? We can continue. On a scale of 1 to 10, out of 10 women who attend their first antenatal care (ANC), how many know the date of their last period? An estimation, please.  **[00:05:19-5] Person 5**: In my opinion, it won't exceed 2 out of 10 who know their last period date.  **[00:05:29-1] Person 3**: They usually can't give a precise date. They might talk about being a month or two late. That's a common way women express it.  **[00:05:46-4] Interviewer**: At what gestational age do women typically come for their first ANC?  **[00:05:51-4] Person 3**: Let's let the ANC staff answer.  **[00:05:55-6] Person 5**: When do your ladies usually start coming for ANC? How many months into their pregnancy?  **[00:06:05-1] Person 6**: Most start in the 2nd and 3rd trimester. They don't come early.  **[00:06:14-9] Interviewer**: And when they come for their first ANC, do they know precisely the date of the last period?  **[00:06:20-9] Person 6**: No, they usually remember the month. We try to calculate roughly from that.  **[00:06:35-2] Interviewer**: On a scale of 1 to 10, how many can give this date?  **[00:06:49-5] Person 6**: About 3 out of 10, no more (laughing).  **[00:06:55-0] Person 2**: The exact date?  **[00:06:56-7] Person 1**: We're talking about the exact last period date.  **[00:07:05-2] Person 3**: In my opinion, out of 10, it doesn't even exceed 2. Educated women, like workers, may provide the exact date, but others will just mention it was in the past month or two.  **[00:07:32-8] Interviewer**: So, the precision is typically about the month?  **[00:07:35-6] Person 3**: Yes, exactly. |
| --- |

### Major of Colma1 health and social promotion center

| [00:03:00-8](http://localhost:2300/file=C:/Users/USER/Desktop/ENTRETIENS%20PREMATURITE/CSPS-COLMA1/MAJOR%20CSPS%20COLMA1.MP3time=180800) Interviewer: nous allons essayer de partir sur une échelle de un à 10.Sur 10 femmes qui se présentent à leur première CPN, combien connaissent leur date des dernières règles ? [00:03:14-5](http://localhost:2300/file=C:/Users/USER/Desktop/ENTRETIENS%20PREMATURITE/CSPS-COLMA1/MAJOR%20CSPS%20COLMA1.MP3time=194500)  [00:03:14-5](http://localhost:2300/file=C:/Users/USER/Desktop/ENTRETIENS%20PREMATURITE/CSPS-COLMA1/MAJOR%20CSPS%20COLMA1.MP3time=194500) Person 1: Sur 10 femmes, [00:03:19-6](http://localhost:2300/file=C:/Users/USER/Desktop/ENTRETIENS%20PREMATURITE/CSPS-COLMA1/MAJOR%20CSPS%20COLMA1.MP3time=199600)  [00:03:19-6](http://localhost:2300/file=C:/Users/USER/Desktop/ENTRETIENS%20PREMATURITE/CSPS-COLMA1/MAJOR%20CSPS%20COLMA1.MP3time=199600) Interviewer: c'est à dire sur une echelle de 1 à 10, combien de femmes peuvent connaître la date des dernières règles, une estimation ? [00:03:26-3](http://localhost:2300/file=C:/Users/USER/Desktop/ENTRETIENS%20PREMATURITE/CSPS-COLMA1/MAJOR%20CSPS%20COLMA1.MP3time=206300)  [00:03:26-3](http://localhost:2300/file=C:/Users/USER/Desktop/ENTRETIENS%20PREMATURITE/CSPS-COLMA1/MAJOR%20CSPS%20COLMA1.MP3time=206300) Person 1: Une estimation, je dirai 3 à 4. [00:03:30-9](http://localhost:2300/file=C:/Users/USER/Desktop/ENTRETIENS%20PREMATURITE/CSPS-COLMA1/MAJOR%20CSPS%20COLMA1.MP3time=210900)  [00:03:30-9](http://localhost:2300/file=C:/Users/USER/Desktop/ENTRETIENS%20PREMATURITE/CSPS-COLMA1/MAJOR%20CSPS%20COLMA1.MP3time=210900) Interviewer: Generalement à quel trimestre de grossesse ou quel âge gestationnel ? Les femmes viennent à la première CPN le plus souvent? [00:03:38-6](http://localhost:2300/file=C:/Users/USER/Desktop/ENTRETIENS%20PREMATURITE/CSPS-COLMA1/MAJOR%20CSPS%20COLMA1.MP3time=218600)  [00:03:38-6](http://localhost:2300/file=C:/Users/USER/Desktop/ENTRETIENS%20PREMATURITE/CSPS-COLMA1/MAJOR%20CSPS%20COLMA1.MP3time=218600) Person 1: Euh, généralement, c'est le premier trimestre, le premier trimestre. [00:03:43-9](http://localhost:2300/file=C:/Users/USER/Desktop/ENTRETIENS%20PREMATURITE/CSPS-COLMA1/MAJOR%20CSPS%20COLMA1.MP3time=223900)  [00:03:43-9](http://localhost:2300/file=C:/Users/USER/Desktop/ENTRETIENS%20PREMATURITE/CSPS-COLMA1/MAJOR%20CSPS%20COLMA1.MP3time=223900) Interviewer: Mais est-ce que vous pensez que les femmes connaissent avec précision la date de leur dernière règle ? [00:03:48-3](http://localhost:2300/file=C:/Users/USER/Desktop/ENTRETIENS%20PREMATURITE/CSPS-COLMA1/MAJOR%20CSPS%20COLMA1.MP3time=228300)  [00:03:48-3](http://localhost:2300/file=C:/Users/USER/Desktop/ENTRETIENS%20PREMATURITE/CSPS-COLMA1/MAJOR%20CSPS%20COLMA1.MP3time=228300) Person 1: non justement, C'est pourquoi j'ai dit tantôt, j'ai dit elles sont rares, sur 10 femmes, 3 par exemple peuvent connaître donc leure date des dernières règles. Par contre les 7 en général n'ont pas une idée. Voilà. [00:04:01-5](http://localhost:2300/file=C:/Users/USER/Desktop/ENTRETIENS%20PREMATURITE/CSPS-COLMA1/MAJOR%20CSPS%20COLMA1.MP3time=241500) |
| --- |

| 00:03:00-8 Interviewer: Nous allons essayer de partir sur une échelle de un à 10. Sur 10 femmes qui se présentent à leur première CPN, combien connaissent leur date des dernières règles ? 00:03:14-5  00:03:14-5 Person 1: Sur 10 femmes, 00:03:19-6  00:03:19-6 Interviewer: C'est-à-dire sur une échelle de 1 à 10, combien de femmes peuvent connaître la date des dernières règles, une estimation ? 00:03:26-3  00:03:26-3 Person 1: Une estimation, je dirais 3 à 4. 00:03:30-9  00:03:30-9 Interviewer: Généralement, à quel trimestre de grossesse ou quel âge gestationnel les femmes viennent-elles à la première CPN le plus souvent? 00:03:38-6  00:03:38-6 Person 1: Euh, généralement, c'est le premier trimestre, le premier trimestre. 00:03:43-9  00:03:43-9 Interviewer: Mais est-ce que vous pensez que les femmes connaissent avec précision la date de leur dernière règle ? 00:03:48-3  00:03:48-3 Person 1: Non justement, c'est pourquoi j'ai dit tantôt, j'ai dit qu'elles sont rares, sur 10 femmes, 3 par exemple peuvent connaître donc leur date des dernières règles. Par contre, les 7 en général n'ont pas une idée. Voilà. 00:04:01-5 |
| --- |

| **[00:03:00-8] Interviewer**: Let's start with a scale of 1 to 10. Out of 10 women who come for their first antenatal care (ANC), how many know the date of their last menstrual period?  **[00:03:14-5] Person 1**: Out of 10 women...  **[00:03:19-6] Interviewer**: On a scale of 1 to 10, how many women can estimate the date of their last period?  **[00:03:26-3] Person 1**: As an estimate, I would say about 3 to 4 out of 10.  **[00:03:30-9] Interviewer**: Typically, in what trimester of pregnancy or at what gestational age do women most often come for their first prenatal consultation (CPN)?  **[00:03:38-6] Person 1**: Usually, it's in the first trimester.  **[00:03:43-9] Interviewer**: But do you think women precisely know the date of their last period?  **[00:03:48-3] Person 1**: Not precisely, which is why I said earlier that they are rare. Out of 10 women, maybe 3 can know the date of their last period. The other 7 generally don't have an idea. |
| --- |

### Maternity manager of Colma1 health and social promotion center

| [00:06:22-6](http://localhost:2300/file=C:/Users/USER/Desktop/ENTRETIENS%20PREMATURITE/CSPS-COLMA1/RESPONSABLE%20MATERNITE-COLMA1.mp3time=382600) Interviewer: Maintenant, si on prend une échelle de 1 à 10,C'est-à-dire que sur dix femmes qui viennent à leure première CPN. Combien connaissent la date de leure dernière règle ? [00:06:36-6](http://localhost:2300/file=C:/Users/USER/Desktop/ENTRETIENS%20PREMATURITE/CSPS-COLMA1/RESPONSABLE%20MATERNITE-COLMA1.mp3time=396600)  [00:06:36-6](http://localhost:2300/file=C:/Users/USER/Desktop/ENTRETIENS%20PREMATURITE/CSPS-COLMA1/RESPONSABLE%20MATERNITE-COLMA1.mp3time=396600) Person 1: Sur dix femmes, voilà, nous sommes... Moi, je prends la communauté du CSPS de Colma 1. Je les considère comme si on était dans la zone rurale. Parce que quand j'ai quitté mon dernier poste, quand il est arrivé ici, avec le comportement, je sais que là-bas, en brousse (milieu rural), mais les femmes arrivaient avec les sensibilisations et comme elles ne sont pas aussi nombreuses, tu arrivais vraiment à les cadrer et à les inculquer vraiment des connaissances. Mais la communauté de Colma, je pense que sur 10 femmes, je vais revenir dans votre question, dans dix femmes, celles qui connaissent la date des dernieres règles, je vais prendre quatre (04). Mais lorsque nous approfondisons, en tout cas, notre interrogatoire, on peut ajouter peut- être trois à quatre. Je ne sais pas si vous me comprenez. Parce qu'il ne suffit pas de demander, même des gens qui sont des fonctionnaires. Je pense que j'ai beaucoup à faire même aux enseignants, aux agents de santé ou d'autres fonctionnaires qui vont venir, mais vont te dire « Je ne me rappelle pas bien de ma date de dernière règles. » Même si elles viennent pour desirs de grossesse. Mais lorsque tu approfondis, tu dis par exemple de lier la date des dernières règles à d'autres évènements qui se sont passés,Il y a d'autres qui viendraient vous dire qu'au moment où Aicha Tremblée était sur Facebook ou quoi que ce soit, c'est au moment où mes règles sont venues, je n'ai plus vu. Et directement, on va savoir que c'est le mois passé. Ou bien d'autres diront que c'est pendant le mois de Ramadan que je n'ai plus vu. Le deuxième jour, quand on va ajouter ces gens-là, je pense, mais il faut que l'interrogatoire soit plus approfondi. On peut avoir un moyen de six à sept femmes. [00:08:47-1](http://localhost:2300/file=C:/Users/USER/Desktop/ENTRETIENS%20PREMATURITE/CSPS-COLMA1/RESPONSABLE%20MATERNITE-COLMA1.mp3time=527100)  [00:08:47-1](http://localhost:2300/file=C:/Users/USER/Desktop/ENTRETIENS%20PREMATURITE/CSPS-COLMA1/RESPONSABLE%20MATERNITE-COLMA1.mp3time=527100) Interviewer: est ce que la précision de cette estimation est plus mensuelle? [00:08:50-5](http://localhost:2300/file=C:/Users/USER/Desktop/ENTRETIENS%20PREMATURITE/CSPS-COLMA1/RESPONSABLE%20MATERNITE-COLMA1.mp3time=530500)  [00:08:50-5](http://localhost:2300/file=C:/Users/USER/Desktop/ENTRETIENS%20PREMATURITE/CSPS-COLMA1/RESPONSABLE%20MATERNITE-COLMA1.mp3time=530500) Person 1: Oui. La précision est plus mensuelle. Mais pour quelques personnes, sur dix femmes, quatre personnes peuvent dire- La date exacte. mais généralement, les femmes aiment dire la date de la fin. Mais là, quand ils demandent encore, on te dira ça a commencé, ça fait quatre jours. C'est toi qui va investiguer. Par exemple, aujourd'hui, nous sommes aux 14. elles vont dire que non, C'est fini les 14. Et tu vas demander, mais tes règles font combien de jours, elle va te dire quatre jours. Donc tu sais que c'est le 10 [00:09:26-4](http://localhost:2300/file=C:/Users/USER/Desktop/ENTRETIENS%20PREMATURITE/CSPS-COLMA1/RESPONSABLE%20MATERNITE-COLMA1.mp3time=566400)  [00:09:26-4](http://localhost:2300/file=C:/Users/USER/Desktop/ENTRETIENS%20PREMATURITE/CSPS-COLMA1/RESPONSABLE%20MATERNITE-COLMA1.mp3time=566400) Interviewer: Et le plus souvent, les femmes viennent à leure première CPN à partir de quel âge gestationnel? [00:09:32-9](http://localhost:2300/file=C:/Users/USER/Desktop/ENTRETIENS%20PREMATURITE/CSPS-COLMA1/RESPONSABLE%20MATERNITE-COLMA1.mp3time=572900)  [00:09:32-9](http://localhost:2300/file=C:/Users/USER/Desktop/ENTRETIENS%20PREMATURITE/CSPS-COLMA1/RESPONSABLE%20MATERNITE-COLMA1.mp3time=572900) Person 1: Bon, OK. Bon, avec les sensibilisations que nous menons actuellement, les femmes viennent de plus en plus un peu tôt. Sinon, il y a des femmes qui pensent que la grossesse doit être commencé à peser à partir de cinq mois. Sinon, trois mois,les femmes qui viennent ne sont pas beaucoup . Les femmes viennent au début du deuxième trimestre, sauf celles qui ont bénéficié vraiment de nos conseils. Ou bien celles... En tout cas, généralement, c'est ça. Il y a des gens même qui sont au courant. On va leur dire, par exemple, elles sont venues avec les signes sympathiques de grossesse. Elles sont venues pour se soigner. Tu vas leur dire, il faut grouiller venir, le lundi, par exemple pour faire la CPN,elle peut aller tourner , revenir avec le même bulletin que tu avais donné, disant qu'elle était enceinte pour d'autres consultations. C'est un problème vraiment toujours, on lutte, mais ça se corrige pas comme ça. [00:10:34-6](http://localhost:2300/file=C:/Users/USER/Desktop/ENTRETIENS%20PREMATURITE/CSPS-COLMA1/RESPONSABLE%20MATERNITE-COLMA1.mp3time=634600) |
| --- |

| 00:06:22-6 Interviewer: Maintenant, si on prend une échelle de 1 à 10, c'est-à-dire que sur dix femmes qui viennent à leur première CPN, combien connaissent la date de leur dernière règle? 00:06:36-6  00:06:36-6 Person 1: Sur dix femmes, voilà, nous sommes... Moi, je prends la communauté du CSPS de Colma 1. Je les considère comme si on était dans la zone rurale. Parce que quand j'ai quitté mon dernier poste, quand je suis arrivé ici, avec le comportement, je sais que là-bas, en brousse (milieu rural), les femmes arrivaient avec les sensibilisations et comme elles ne sont pas aussi nombreuses, tu arrivais vraiment à les cadrer et à les inculquer vraiment des connaissances. Mais la communauté de Colma, je pense que sur 10 femmes, je vais revenir dans votre question, dans dix femmes, celles qui connaissent la date des dernières règles, je vais prendre quatre (04). Mais lorsque nous approfondissons, en tout cas, notre interrogatoire, on peut ajouter peut-être trois à quatre. Je ne sais pas si vous me comprenez. Parce qu'il ne suffit pas de demander, même des gens qui sont des fonctionnaires. Je pense que j'ai beaucoup à faire même aux enseignants, aux agents de santé ou d'autres fonctionnaires qui vont venir, mais vont te dire, « Je ne me rappelle pas bien de ma date de dernière règles. » Même si elles viennent pour désirs de grossesse. Mais lorsque tu approfondis, tu dis par exemple de lier la date des dernières règles à d'autres évènements qui se sont passés, il y a d'autres qui viendraient vous dire qu'au moment où Aicha tremblait était sur Facebook ou quoi que ce soit, c'est au moment où mes règles sont venues, je n'ai plus vu. Et directement, on va savoir que c'est le mois passé. Ou bien d'autres diront que c'est pendant le mois de Ramadan que je n'ai plus vu. Le deuxième jour, quand on va ajouter ces gens-là, je pense, mais il faut que l'interrogatoire soit plus approfondi. On peut avoir un moyen de six à sept femmes. 00:08:47-1  00:08:47-1 Interviewer: Est-ce que la précision de cette estimation est plus mensuelle? 00:08:50-5  00:08:50-5 Person 1: Oui. La précision est plus mensuelle. Mais pour quelques personnes, sur dix femmes, quatre personnes peuvent dire la date exacte. Mais généralement, les femmes aiment dire la date de la fin. Mais là, quand elles demandent encore, on te dira ça a commencé, ça fait quatre jours. C'est toi qui va investiguer. Par exemple, aujourd'hui, nous sommes le 14. Elles vont dire que non, c'est fini le 14. Et tu vas demander, mais tes règles font combien de jours, elle va te dire quatre jours. Donc tu sais que c'est le 10. 00:09:26-4  00:09:26-4 Interviewer: Et le plus souvent, les femmes viennent à leur première CPN à partir de quel âge gestationnel? 00:09:32-9  00:09:32-9 Person 1: Bon, OK. Bon, avec les sensibilisations que nous menons actuellement, les femmes viennent de plus en plus un peu tôt. Sinon, il y a des femmes qui pensent que la grossesse doit être commencée à peser à partir de cinq mois. Sinon, trois mois, les femmes qui viennent ne sont pas beaucoup. Les femmes viennent au début du deuxième trimestre, sauf celles qui ont bénéficié vraiment de nos conseils. Ou bien celles... En tout cas, généralement, c'est ça. Il y a des gens même qui sont au courant. On va leur dire, par exemple, elles sont venues avec les signes sympathiques de grossesse. Elles sont venues pour se soigner. Tu vas leur dire, il faut grouiller venir, le lundi, par exemple, pour faire la CPN, elle peut aller tourner, revenir avec le même bulletin que tu avais donné, disant qu'elle était enceinte pour d'autres consultations. C'est un problème vraiment toujours, on lutte, mais ça ne se corrige pas comme ça. 00:10:34-6 |
| --- |

| **[00:06:22-6] Interviewer**: On a scale of 1 to 10, how many women out of ten who come to their first antenatal care (ANC) know the date of their last menstrual period?  **[00:06:36-6] Person 1**: Considering the community at the CSPS of Colma 1, which is similar to a rural area, I would say about four out of ten women know the date of their last period. With more in-depth questioning, we might be able to add another three to four women to that number. It's not just about asking the question directly. For example, even among civil servants, including teachers and healthcare workers, many initially say they don't remember the date of their last period. But if we link the date to specific events, like something they saw on Facebook or during the month of Ramadan, more women can recall approximately when it was. With this approach, we might get six to seven out of ten women providing an estimate.  **[00:08:47-1] Interviewer**: Is the precision of this estimate more about the month?  **[00:08:50-5] Person 1**: Yes, it's more about the month. A few, about four out of ten, can give the exact date. Most women tend to provide the ending date of their period. Then, with further investigation, such as asking about the duration of their period, we can deduce the starting date. For instance, if today is the 14th and they say their period ended on the 14th and lasted four days, we know it started on the 10th.  **[00:09:26-4] Interviewer**: At what gestational age do women most often come to their first antenatal care (ANC) appointment?  **[00:09:32-9] Person 1**: With the current awareness campaigns, we're seeing women come a bit earlier than before. However, there's a common belief that prenatal weighing should start from five months. Few women come at three months; most arrive at the beginning of the second trimester. This is despite our advice. Sometimes, women who initially visit for pregnancy-related symptoms delay their ANC even after being advised to start early. They may return with the same consultation slip, indicating pregnancy, for other issues. It's a challenge, and changing this pattern isn't easy. |
| --- |

### Focus group of Farakan health and social promotion center

| [00:08:10-9](http://localhost:2300/file=C:/Users/USER/Desktop/ENTRETIENS%20PREMATURITE/CSPS_FARAKAN/FOCUS_GROUPE-CSPS_FARAKAN.MP3time=490900) Interviewer: Ok.Nous allons poursuivre avec les questions.Sur 10 femmes qui se présentent à leur première CPN. Combien connaissent leur date des dernières règles ? [00:08:48-5](http://localhost:2300/file=C:/Users/USER/Desktop/ENTRETIENS%20PREMATURITE/CSPS_FARAKAN/FOCUS_GROUPE-CSPS_FARAKAN.MP3time=528500)  [00:08:48-5](http://localhost:2300/file=C:/Users/USER/Desktop/ENTRETIENS%20PREMATURITE/CSPS_FARAKAN/FOCUS_GROUPE-CSPS_FARAKAN.MP3time=528500) Person 3: Ah, si on doit donner un pourcentage c'est 1 sur 10.Oui. [00:08:57-2](http://localhost:2300/file=C:/Users/USER/Desktop/ENTRETIENS%20PREMATURITE/CSPS_FARAKAN/FOCUS_GROUPE-CSPS_FARAKAN.MP3time=537200)  [00:08:57-2](http://localhost:2300/file=C:/Users/USER/Desktop/ENTRETIENS%20PREMATURITE/CSPS_FARAKAN/FOCUS_GROUPE-CSPS_FARAKAN.MP3time=537200) Person 5: 1 sur 10 (1/10) là même c'est rare. [00:09:02-0](http://localhost:2300/file=C:/Users/USER/Desktop/ENTRETIENS%20PREMATURITE/CSPS_FARAKAN/FOCUS_GROUPE-CSPS_FARAKAN.MP3time=542000)  [00:09:02-0](http://localhost:2300/file=C:/Users/USER/Desktop/ENTRETIENS%20PREMATURITE/CSPS_FARAKAN/FOCUS_GROUPE-CSPS_FARAKAN.MP3time=542000) Person 6: Pour ne pas dire 0 sur 10, vraiment 1 sur 10 (1/10). [00:09:09-1](http://localhost:2300/file=C:/Users/USER/Desktop/ENTRETIENS%20PREMATURITE/CSPS_FARAKAN/FOCUS_GROUPE-CSPS_FARAKAN.MP3time=549100)  [00:09:09-1](http://localhost:2300/file=C:/Users/USER/Desktop/ENTRETIENS%20PREMATURITE/CSPS_FARAKAN/FOCUS_GROUPE-CSPS_FARAKAN.MP3time=549100) Person 7: je peut dire 1 sur 10 (1/10). [00:09:13-6](http://localhost:2300/file=C:/Users/USER/Desktop/ENTRETIENS%20PREMATURITE/CSPS_FARAKAN/FOCUS_GROUPE-CSPS_FARAKAN.MP3time=553600)  [00:09:13-6](http://localhost:2300/file=C:/Users/USER/Desktop/ENTRETIENS%20PREMATURITE/CSPS_FARAKAN/FOCUS_GROUPE-CSPS_FARAKAN.MP3time=553600) Person 8: Vraiment nous avons à faire à des gens qui ne sont pas allés à l'école comme ça, donc pour vraiment déterminer la dernière date des règles, c'est dur.Le pourcentage c'est 1 vraiment pour ne rien dire. [00:09:35-7](http://localhost:2300/file=C:/Users/USER/Desktop/ENTRETIENS%20PREMATURITE/CSPS_FARAKAN/FOCUS_GROUPE-CSPS_FARAKAN.MP3time=575700)  [00:09:35-7](http://localhost:2300/file=C:/Users/USER/Desktop/ENTRETIENS%20PREMATURITE/CSPS_FARAKAN/FOCUS_GROUPE-CSPS_FARAKAN.MP3time=575700) Person 4: Sinon en CPN on leurs demande mais elles repondent ne pas connaitre.Nous posons la questions aux femmes mais disent qu'elles ne connaissent pas.Le pourcentage c'est 1 sur 10 (1/10). [00:09:49-7](http://localhost:2300/file=C:/Users/USER/Desktop/ENTRETIENS%20PREMATURITE/CSPS_FARAKAN/FOCUS_GROUPE-CSPS_FARAKAN.MP3time=589700)  [00:09:49-7](http://localhost:2300/file=C:/Users/USER/Desktop/ENTRETIENS%20PREMATURITE/CSPS_FARAKAN/FOCUS_GROUPE-CSPS_FARAKAN.MP3time=589700) Interviewer: Nous allons continuer, à quel trimestre de grossesse ou à quel âge gestationnel ? Les femmes viennent à leur première à CPN le plus souvent? [00:10:17-1](http://localhost:2300/file=C:/Users/USER/Desktop/ENTRETIENS%20PREMATURITE/CSPS_FARAKAN/FOCUS_GROUPE-CSPS_FARAKAN.MP3time=617100)  [00:10:17-1](http://localhost:2300/file=C:/Users/USER/Desktop/ENTRETIENS%20PREMATURITE/CSPS_FARAKAN/FOCUS_GROUPE-CSPS_FARAKAN.MP3time=617100) Person 7: C'est à partir de la 6ème semaine, d'autres viennent commencer [00:10:26-7](http://localhost:2300/file=C:/Users/USER/Desktop/ENTRETIENS%20PREMATURITE/CSPS_FARAKAN/FOCUS_GROUPE-CSPS_FARAKAN.MP3time=626700)  [00:10:26-7](http://localhost:2300/file=C:/Users/USER/Desktop/ENTRETIENS%20PREMATURITE/CSPS_FARAKAN/FOCUS_GROUPE-CSPS_FARAKAN.MP3time=626700) Person 4: Je pense que c'est à 4 semaines, juste un mois d'autres viennent [00:10:33-0](http://localhost:2300/file=C:/Users/USER/Desktop/ENTRETIENS%20PREMATURITE/CSPS_FARAKAN/FOCUS_GROUPE-CSPS_FARAKAN.MP3time=633000)  [00:10:33-0](http://localhost:2300/file=C:/Users/USER/Desktop/ENTRETIENS%20PREMATURITE/CSPS_FARAKAN/FOCUS_GROUPE-CSPS_FARAKAN.MP3time=633000) Person 5: Non, pour ça il y a des gens qui viennent au premier trimestre à 3 mois et pour d'autres ça dépassent le premier trimestre.C'est vraiment pas un moment exact cela varie beaucoup. [00:10:47-3](http://localhost:2300/file=C:/Users/USER/Desktop/ENTRETIENS%20PREMATURITE/CSPS_FARAKAN/FOCUS_GROUPE-CSPS_FARAKAN.MP3time=647300)  [00:10:47-3](http://localhost:2300/file=C:/Users/USER/Desktop/ENTRETIENS%20PREMATURITE/CSPS_FARAKAN/FOCUS_GROUPE-CSPS_FARAKAN.MP3time=647300) Person 3: Il y a d'autres qui viennent à 12 semaines, d'autres viennent à partir de 4 semaines. Y a d'autres mêmes qui viennent à partir de 6 mois pour la première CPN. [00:10:59-2](http://localhost:2300/file=C:/Users/USER/Desktop/ENTRETIENS%20PREMATURITE/CSPS_FARAKAN/FOCUS_GROUPE-CSPS_FARAKAN.MP3time=659200)  [00:10:59-2](http://localhost:2300/file=C:/Users/USER/Desktop/ENTRETIENS%20PREMATURITE/CSPS_FARAKAN/FOCUS_GROUPE-CSPS_FARAKAN.MP3time=659200) Person 4: Bon, avec les sensibilisations la majorité des femmes a compris et puis maintenant au premier trimestre, dans les 3 premiers mois, on reçoit le maximum de femmes en tout cas pour la première CPN. [00:11:14-7](http://localhost:2300/file=C:/Users/USER/Desktop/ENTRETIENS%20PREMATURITE/CSPS_FARAKAN/FOCUS_GROUPE-CSPS_FARAKAN.MP3time=674700)  [00:11:14-7](http://localhost:2300/file=C:/Users/USER/Desktop/ENTRETIENS%20PREMATURITE/CSPS_FARAKAN/FOCUS_GROUPE-CSPS_FARAKAN.MP3time=674700) Person 6: La plupart, c'est au premier trimestre et c'est après ça aussi que d'autres viennent à 4 mois en allant. [00:11:24-2](http://localhost:2300/file=C:/Users/USER/Desktop/ENTRETIENS%20PREMATURITE/CSPS_FARAKAN/FOCUS_GROUPE-CSPS_FARAKAN.MP3time=684200) |
| --- |

| 00:08:10-9 Interviewer: Ok. Nous allons poursuivre avec les questions. Sur 10 femmes qui se présentent à leur première CPN, combien connaissent leur date des dernières règles ? 00:08:48-5  00:08:48-5 Person 3: Ah, si on doit donner un pourcentage, c'est 1 sur 10. Oui. 00:08:57-2  00:08:57-2 Person 5: 1 sur 10 (1/10) là même, c'est rare. 00:09:02-0  00:09:02-0 Person 6: Pour ne pas dire 0 sur 10, vraiment 1 sur 10 (1/10). 00:09:09-1  00:09:09-1 Person 7: Je peux dire 1 sur 10 (1/10). 00:09:13-6  00:09:13-6 Person 8: Vraiment, nous avons à faire à des gens qui ne sont pas allés à l'école comme ça, donc pour vraiment déterminer la dernière date des règles, c'est dur. Le pourcentage, c'est 1 vraiment pour ne rien dire. 00:09:35-7  00:09:35-7 Person 4: Sinon en CPN, on leur demande mais elles répondent ne pas connaître. Nous posons la question aux femmes mais elles disent qu'elles ne connaissent pas. Le pourcentage, c'est 1 sur 10 (1/10). 00:09:49-7  00:09:49-7 Interviewer: Nous allons continuer, à quel trimestre de grossesse ou à quel âge gestationnel les femmes viennent-elles à leur première CPN le plus souvent? 00:10:17-1  00:10:17-1 Person 7: C'est à partir de la 6ème semaine, d'autres viennent commencer. 00:10:26-7  00:10:26-7 Person 4: Je pense que c'est à 4 semaines, juste un mois, d'autres viennent. 00:10:33-0  00:10:33-0 Person 5: Non, pour ça, il y a des gens qui viennent au premier trimestre à 3 mois, et pour d'autres, ça dépasse le premier trimestre. C'est vraiment pas un moment exact, cela varie beaucoup. 00:10:47-3  00:10:47-3 Person 3: Il y a d'autres qui viennent à 12 semaines, d'autres viennent à partir de 4 semaines. Il y a d'autres mêmes qui viennent à partir de 6 mois pour la première CPN. 00:10:59-2  00:10:59-2 Person 4: Bon, avec les sensibilisations, la majorité des femmes a compris et puis maintenant, au premier trimestre, dans les 3 premiers mois, on reçoit le maximum de femmes, en tout cas pour la première CPN. 00:11:14-7  00:11:14-7 Person 6: La plupart, c'est au premier trimestre et c'est après ça aussi que d'autres viennent à 4 mois en allant. 00:11:24-2 |
| --- |

| **[00:08:10-9] Interviewer**: Let's continue with the questions. Out of 10 women who come for their first prenatal consultation (CPN), how many know the date of their last menstrual period?  **[00:08:48-5] Person 3**: If we had to give a percentage, it would be about 1 in 10.  **[00:08:57-2] Person 5**: Only 1 out of 10, and even that's rare.  **[00:09:02-0] Person 6**: I would say practically 0 out of 10, but realistically, it's about 1 out of 10.  **[00:09:09-1] Person 7**: I also estimate it at about 1 out of 10.  **[00:09:13-6] Person 8**: Considering we're dealing with a population that often lacks formal education, determining the exact date of the last menstrual period is challenging. So, the percentage is really close to 1, if not zero.  **[00:09:35-7] Person 4**: In CPN, we do ask them, but they usually say they don't know. The percentage is around 1 in 10.  **[00:09:49-7] Interviewer**: Let's continue. At what trimester of pregnancy or gestational age do women most often come for their first antenatal care (ANC) appointment?  **[00:10:17-1] Person 7**: Some come as early as the 6th week, while others come earlier.  **[00:10:26-7] Person 4**: I think it's around 4 weeks, just a month, we see more women coming in.  **[00:10:33-0] Person 5**: There are women who come in the first trimester at 3 months, but for others, it extends beyond the first trimester. It really varies.  **[00:10:47-3] Person 3**: Some come at 12 weeks, others from 4 weeks, and there are those who come for their first ANC as late as 6 months.  **[00:10:59-2] Person 4**: With increased awareness, most women now understand the importance and come in the first trimester, within the first 3 months.  **[00:11:14-7] Person 6**: The majority are in the first trimester, and others come around the 4th month. |
| --- |

### Major of Farakan health and social promotion center

| [00:03:03-8](http://localhost:2300/file=C:/Users/USER/Desktop/ENTRETIENS%20PREMATURITE/CSPS_FARAKAN/MAJOR%20FARAKAN.MP3time=183800) Interviewer: Maintenant, si on prend sur une échelle de 1 à 10, sur 10 femmes qui viennent à leur première CPN, combien connaissent leurs dates des dernières règles ? [00:03:15-0](http://localhost:2300/file=C:/Users/USER/Desktop/ENTRETIENS%20PREMATURITE/CSPS_FARAKAN/MAJOR%20FARAKAN.MP3time=195000)  [00:03:15-0](http://localhost:2300/file=C:/Users/USER/Desktop/ENTRETIENS%20PREMATURITE/CSPS_FARAKAN/MAJOR%20FARAKAN.MP3time=195000) Person 1: Souvent c'est difficile. [00:03:15-8](http://localhost:2300/file=C:/Users/USER/Desktop/ENTRETIENS%20PREMATURITE/CSPS_FARAKAN/MAJOR%20FARAKAN.MP3time=195800)  [00:03:15-8](http://localhost:2300/file=C:/Users/USER/Desktop/ENTRETIENS%20PREMATURITE/CSPS_FARAKAN/MAJOR%20FARAKAN.MP3time=195800) Interviewer: Une estimation. [00:03:16-8](http://localhost:2300/file=C:/Users/USER/Desktop/ENTRETIENS%20PREMATURITE/CSPS_FARAKAN/MAJOR%20FARAKAN.MP3time=196800)  [00:03:16-8](http://localhost:2300/file=C:/Users/USER/Desktop/ENTRETIENS%20PREMATURITE/CSPS_FARAKAN/MAJOR%20FARAKAN.MP3time=196800) Person 1: Estimation en tout cas, ceux qui connaissent leur date des dernières règles, la moitié ne connaît pas. [00:03:28-9](http://localhost:2300/file=C:/Users/USER/Desktop/ENTRETIENS%20PREMATURITE/CSPS_FARAKAN/MAJOR%20FARAKAN.MP3time=208900)  [00:03:28-9](http://localhost:2300/file=C:/Users/USER/Desktop/ENTRETIENS%20PREMATURITE/CSPS_FARAKAN/MAJOR%20FARAKAN.MP3time=208900) Interviewer: Géneralement à quel âge gestationnel les femmes viennent à leur première CPN? [00:03:35-6](http://localhost:2300/file=C:/Users/USER/Desktop/ENTRETIENS%20PREMATURITE/CSPS_FARAKAN/MAJOR%20FARAKAN.MP3time=215600)  [00:03:35-6](http://localhost:2300/file=C:/Users/USER/Desktop/ENTRETIENS%20PREMATURITE/CSPS_FARAKAN/MAJOR%20FARAKAN.MP3time=215600) Person 1: En général, il y a beaucoup qui viennent, même après le premier trimestre mais avec les sensibilisations cela s'est un peu amélioré.Quand on prend les rapports, vous allez voir que, en tout cas, la majorité viennent après le premier trimestre. [00:03:53-6](http://localhost:2300/file=C:/Users/USER/Desktop/ENTRETIENS%20PREMATURITE/CSPS_FARAKAN/MAJOR%20FARAKAN.MP3time=233600)  [00:03:53-6](http://localhost:2300/file=C:/Users/USER/Desktop/ENTRETIENS%20PREMATURITE/CSPS_FARAKAN/MAJOR%20FARAKAN.MP3time=233600) Interviewer: Est ce que les femmes qui viennent à leur première à CPN connaissent avec exactitude la date de leur dernière règle ? [00:04:02-7](http://localhost:2300/file=C:/Users/USER/Desktop/ENTRETIENS%20PREMATURITE/CSPS_FARAKAN/MAJOR%20FARAKAN.MP3time=242700)  [00:04:02-7](http://localhost:2300/file=C:/Users/USER/Desktop/ENTRETIENS%20PREMATURITE/CSPS_FARAKAN/MAJOR%20FARAKAN.MP3time=242700) Person 1: Elles peuvent connaître le mois, quelques semaines près, mais le jour exacte même souvent c'est difficile, surtout celles qui ne sont pas trop alphabétisés, c'est compliqué pour elles. [00:04:17-7](http://localhost:2300/file=C:/Users/USER/Desktop/ENTRETIENS%20PREMATURITE/CSPS_FARAKAN/MAJOR%20FARAKAN.MP3time=257700) |
| --- |

| 00:03:03-8 Interviewer: Maintenant, si on prend sur une échelle de 1 à 10, sur 10 femmes qui viennent à leur première CPN, combien connaissent leurs dates des dernières règles ? 00:03:15-0  00:03:15-0 Person 1: Souvent c'est difficile. 00:03:15-8  00:03:15-8 Interviewer: Une estimation. 00:03:16-8  00:03:16-8 Person 1: En estimation, en tout cas, ceux qui connaissent leur date des dernières règles, la moitié ne connaît pas. 00:03:28-9  00:03:28-9 Interviewer: Généralement, à quel âge gestationnel les femmes viennent-elles à leur première CPN ? 00:03:35-6  00:03:35-6 Person 1: En général, il y a beaucoup qui viennent, même après le premier trimestre, mais avec les sensibilisations, cela s'est un peu amélioré. Quand on prend les rapports, vous allez voir que, en tout cas, la majorité viennent après le premier trimestre. 00:03:53-6  00:03:53-6 Interviewer: Est-ce que les femmes qui viennent à leur première CPN connaissent avec exactitude la date de leur dernière règle ? 00:04:02-7  00:04:02-7 Person 1: Elles peuvent connaître le mois, quelques semaines près, mais le jour exact, même souvent, c'est difficile, surtout celles qui ne sont pas trop alphabétisées, c'est compliqué pour elles. 00:04:17-7 |
| --- |

| **[00:03:03-8] Interviewer**: On a scale of 1 to 10, out of 10 women who come for their first antenatal care (ANC) appointment, how many know the dates of their last menstrual period?  **[00:03:15-0] Person 1**: It's often difficult...  **[00:03:15-8] Interviewer**: Could you provide an estimate?  **[00:03:16-8] Person 1**: As an estimate, I would say about half of them do not know the date of their last period.  **[00:03:28-9] Interviewer**: Typically, at what gestational age do women come for their first antenatal care (ANC) appointment?  **[00:03:35-6] Person 1**: Generally, many come even after the first trimester. Awareness campaigns have improved this a bit. According to our reports, the majority still come after the first trimester.  **[00:03:53-6] Interviewer**: Do women who come to their first ANC know exactly the date of their last period?  **[00:04:02-7] Person 1**: They often know the month, give or take a few weeks. But recalling the exact day is usually difficult, especially for those who are not very literate. |
| --- |

### Maternity manager of Farakan health and social promotion center

| [00:04:13-2](http://localhost:2300/file=C:/Users/USER/Desktop/ENTRETIENS%20PREMATURITE/CSPS_FARAKAN/RESPONSABLE%20MATERNITE%20FARAKAN.MP3time=253200) Interviewer: nous allons poursuivre sur 10 femmes qui se présentent à leur première CPN, combien connaisse leur date de dernière règle? [00:04:25-1](http://localhost:2300/file=C:/Users/USER/Desktop/ENTRETIENS%20PREMATURITE/CSPS_FARAKAN/RESPONSABLE%20MATERNITE%20FARAKAN.MP3time=265100)  [00:04:25-1](http://localhost:2300/file=C:/Users/USER/Desktop/ENTRETIENS%20PREMATURITE/CSPS_FARAKAN/RESPONSABLE%20MATERNITE%20FARAKAN.MP3time=265100) Person 1: est ce qu'à ce niveau je peux donner un nombre? (rire). Souvent, la date des dernières règles, généralement c'est difficile.Elle donne généralement le mois pour expédier ces 3 mois, c'est 2 mois, c'est 4 mois, c'est 5 mois, mais la date là en tout cas c'est difficile peut être sur 10, on peut dire 3, qui peuvent connaître la date des derniers règles. [00:04:48-7](http://localhost:2300/file=C:/Users/USER/Desktop/ENTRETIENS%20PREMATURITE/CSPS_FARAKAN/RESPONSABLE%20MATERNITE%20FARAKAN.MP3time=288700)  [00:04:48-7](http://localhost:2300/file=C:/Users/USER/Desktop/ENTRETIENS%20PREMATURITE/CSPS_FARAKAN/RESPONSABLE%20MATERNITE%20FARAKAN.MP3time=288700) Interviewer: Généralement, c'est à quelle date, à quelle trimestre de grossesse ou quel âge gestationnel, les femmes viennent pour la première CPN? [00:04:57-6](http://localhost:2300/file=C:/Users/USER/Desktop/ENTRETIENS%20PREMATURITE/CSPS_FARAKAN/RESPONSABLE%20MATERNITE%20FARAKAN.MP3time=297600)  [00:04:57-6](http://localhost:2300/file=C:/Users/USER/Desktop/ENTRETIENS%20PREMATURITE/CSPS_FARAKAN/RESPONSABLE%20MATERNITE%20FARAKAN.MP3time=297600) Person 1: Dans les normes, c'est le le premier trimestre cela veut dire, normalement même une femme lorsqu'elle constate qu'elle est enceinte, elle doit venir commencer la CPN mais on a remarqué qu'elles ne viennent pas tôt.parce que souvent il y en a qui vont venir à 4 mois, 5 mois,peut être que dans l'ancien temps on leurs disait qu'il faut attendre puisqu'avant, c'était 4 CPN, les gens ne venaient pas tôt, mais maintenant, comme nous sommes jusqu'à 8 contacts donc ça veut dire que les rendez-vous c'est chaque mois.Les rendez-vous c'est chaque mois, donc elle doit commencer la CPN, mais généralement, en tout cas, la plupart des femmes, il y en a qui viennent tôt quand même mais il y en a qui ne viennent pas tôt. [00:05:44-0](http://localhost:2300/file=C:/Users/USER/Desktop/ENTRETIENS%20PREMATURITE/CSPS_FARAKAN/RESPONSABLE%20MATERNITE%20FARAKAN.MP3time=344000) |
| --- |

| 00:04:13-2 Interviewer: Nous allons poursuivre, sur 10 femmes qui se présentent à leur première CPN, combien connaissent leur date de dernière règle ? 00:04:25-1  00:04:25-1 Person 1: Est-ce qu'à ce niveau je peux donner un nombre ? (rire). Souvent, la date des dernières règles, généralement, c'est difficile. Elle donne généralement le mois pour expédier, c'est 2 mois, c'est 4 mois, c'est 5 mois, mais la date là, en tout cas, c'est difficile, peut-être sur 10, on peut dire 3, qui peuvent connaître la date des dernières règles. 00:04:48-7  00:04:48-7 Interviewer: Généralement, c'est à quelle date, à quel trimestre de grossesse ou quel âge gestationnel, les femmes viennent pour la première CPN ? 00:04:57-6  00:04:57-6 Person 1: Dans les normes, c'est le premier trimestre, cela veut dire, normalement même une femme, lorsqu'elle constate qu'elle est enceinte, elle doit venir commencer la CPN, mais on a remarqué qu'elles ne viennent pas tôt. Parce que souvent il y en a qui vont venir à 4 mois, 5 mois, peut-être que dans l'ancien temps on leur disait qu'il faut attendre puisqu'avant, c'était 4 CPN, les gens ne venaient pas tôt, mais maintenant, comme nous sommes jusqu'à 8 contacts donc ça veut dire que les rendez-vous c'est chaque mois. Les rendez-vous, c'est chaque mois, donc elle doit commencer la CPN, mais généralement, en tout cas, la plupart des femmes, il y en a qui viennent tôt quand même mais il y en a qui ne viennent pas tôt. 00:05:44-0 |
| --- |

| **[00:04:13-2] Interviewer**: Let's continue. Out of 10 women who come for their first antenatal care (ANC) appointment, how many know the date of their last menstrual period?  **[00:04:25-1] Person 1**: Can I really give a number? (Laughs) It's usually difficult for women to recall the exact date of their last period. They generally provide the month, like it's been 2, 3, 4, or 5 months, but knowing the specific date is hard. Maybe out of 10, about 3 can recall the exact date of their last period.  **[00:04:48-7] Interviewer**: Generally, at what trimester of pregnancy or gestational age do women come for their first antenatal care (ANC)?  **[00:04:57-6] Person 1**: Ideally, it should be in the first trimester. As soon as a woman realizes she's pregnant, she should start ANC. However, we've noticed that they often don't come early. Many arrive at 4 or 5 months. In the past, with only 4 scheduled ANC visits, women tended not to come early. But now, with up to 8 contacts and monthly appointments, they should start ANC earlier. While some do come early, many still don't. |
| --- |

## Control of the date of the last period by women who present late for their antenatal care

### Focus group of Accart-ville urban medical center

| [00:04:36-2](http://localhost:2300/file=C:/Users/USER/Desktop/ENTRETIENS%20PREMATURITE/CSPS-ACCART-VILLE/FOCUS%20GROUPE%20ACCART-VILLE-BON.mp3time=276200)Interviewer: maintenant est ce que les femmes qui viennent à leures premières CPN, plus tard dans la grossesse se souviennent toujours de la date de leure dernière regles? [00:04:44-1](http://localhost:2300/file=C:/Users/USER/Desktop/ENTRETIENS)  [00:04:44-1](http://lo)Person 1: bon, celles qui ont pu nous donner la date de leure dernière règle au premier trimestre peuvent donner toujours. oui [00:04:51-8](http://localhost:2300/file=C:/Users/USER/Desktop/ENTRETIENS%20PREMATURITE/CSPS-ACCART-VILLE/FOCUS%20GROUPE%20ACCART-VILLE-BON.mp3time=291800) |
| --- |

| 00:04:36-2 Interviewer: Maintenant, est-ce que les femmes qui viennent à leurs premières CPN, plus tard dans la grossesse, se souviennent toujours de la date de leur dernière règles ? 00:04:44-1  00:04:44-1 Person 1: Bon, celles qui ont pu nous donner la date de leur dernière règle au premier trimestre peuvent donner toujours. Oui. 00:04:51-8 |
| --- |

| **[00:04:36-2] Interviewer**: Now, do women who come for their first ANC later in pregnancy still remember the date of their last period?  **[00:04:44-1] Person 1**: Those who were able to provide the date of their last period in the first trimester usually can still recall it later on. Yes. |
| --- |

### Manager of Accart-ville urban medical center

| [00:05:18-1](http://localhost:2300/file=C:/Users/USER/Desktop/ENTRETIENS%20PREMATURITE/CSPS-ACCART-VILLE/RESPONSABLE%20CMU%20ACCART-VILLE.MP3time=318100)Interviewer: maintenant par rapport à celle qui vienne toujours à leur première CPN,est ce qu'au cours de la grossesse, elle peut toujours se rappeler de.la Date des dèrnieres règles? [00:05:28-5](http://localhost:2300/file=C:/Users/USER/Desktop/ENTRETIENS%20PREMATURITE/CSPS-ACCART-VILLE/RESPONSABLE%20CMU%20ACCART-VILLE.MP3time=328500)  [00:05:28-5](http://localhost:2300/file=C:/Users/USER/Desktop/ENTRETIENS%20PREMATURITE/CSPS-ACCART-VILLE/RESPONSABLE%20CMU%20ACCART-VILLE.MP3time=328500)Person 1: bon, je dis ça en fait, c'est rare de trouver celle qui va pouvoir situer cette date là, alors c'est vraiment très rare. C'est pourquoi généralement. Bon, j'ai dit comme c'est un peu difficile de se baser sur l'äge échographique. C'est vrai, on dit que c'est la première échographie qui peut bien nous situer, mais bon j''ai toujours dis pour leur premier contact de demanderune echographie pour essayer de dater d'abord. [00:05:58-5](http://localhost:2300/file=C:/Users/USER/Desktop/ENTRETIENS%20PREMATURITE/CSPS-ACCART-VILLE/RESPONSABLE%20CMU%20ACCART-VILLE.MP3time=358500) |
| --- |

| 00:05:18-1 Interviewer: Maintenant, par rapport à celles qui viennent toujours à leur première CPN, est-ce qu'au cours de la grossesse, elle peut toujours se rappeler de la date des dernières règles ? 00:05:28-5  00:05:28-5 Person 1: Bon, je dis ça, en fait, c'est rare de trouver celle qui va pouvoir situer cette date là, alors c'est vraiment très rare. C'est pourquoi généralement. Bon, j'ai dit comme c'est un peu difficile de se baser sur l'âge échographique. C'est vrai, on dit que c'est la première échographie qui peut bien nous situer, mais bon j'ai toujours dit pour leur premier contact de demander une échographie pour essayer de dater d'abord. 00:05:58-5 |
| --- |

| **[00:05:18-1] Interviewer**: For those who attend their first ANC later during pregnancy, can they still remember the date of their last period?  **[00:05:28-5] Person 1**: It's rare to find someone who can recall this date; it's very unusual. That's why we often rely on the first ultrasound for dating, but I always recommend getting an ultrasound at the first contact to try to accurately date the pregnancy. |
| --- |

### Maternity manager of Accart-ville urban medical center

| [00:04:51-6](http://localhost:2300/file=C:/Users/USER/Desktop/ENTRETIENS%20PREMATURITE/CSPS-ACCART-VILLE/RESPONSABLE%20MATERNITE%20CMU%20ACCART-VILLE.MP3time=291600) Interviewer: maintenant pour les femmes qui viennent à leure première cpn, est ce que plutard au cours de leur grossesse, elles se souviennent toujours de la date de leure dernière règle? [00:05:00-1](http://localhost:2300/file=C:/Users/USER/Desktop/ENTRETIENS%20PREMATURITE/CSPS-ACCART-VILLE/RESPONSABLE%20MATERNITE%20CMU%20ACCART-VILLE.MP3time=300100)  [00:05:00-1](http://localhost:2300/file=C:/Users/USER/Desktop/ENTRETIENS%20PREMATURITE/CSPS-ACCART-VILLE/RESPONSABLE%20MATERNITE%20CMU%20ACCART-VILLE.MP3time=300100) Person 1: non, la plupart elles ne souviennent pas de la date de leure dernière règle [00:05:07-5](http://localhost:2300/file=C:/Users/USER/Desktop/ENTRETIENS%20PREMATURITE/CSPS-ACCART-VILLE/RESPONSABLE%20MATERNITE%20CMU%20ACCART-VILLE.MP3time=307500) |
| --- |

| 00:04:51-6 Interviewer: Maintenant pour les femmes qui viennent à leur première CPN, est-ce que plus tard au cours de leur grossesse, elles se souviennent toujours de la date de leur dernière règle ? 00:05:00-1  00:05:00-1 Person 1: Non, la plupart elles ne se souviennent pas de la date de leur dernière règle. 00:05:07-5 |
| --- |

| **[00:04:51-6] Interviewer**: For women who come to their first ANC, do they remember the date of their last period later during their pregnancy?  **[00:05:00-1] Person 1**: No, most of them don't remember the date of their last period. |
| --- |

### Focus group of Do medical center with surgical branch

| [00:07:31-6](http://localhost:2300/file=C:/Users/USER/Desktop/ENTRETIENS%20PREMATURITE/CMA-DO/FOCUS%20GROUPE%20CMA-DO.mp3time=451600) Interviewer: Est ce que c'elles qui se presentent à leur première CPN, plus tard, au cours de la grossesse, elles arrivent toujours à se souvenir de la date de leur dernière règle? [00:07:41-2](http://localhost:2300/file=C:/Users/USER/Desktop/ENTRETIENS%20PREMATURITE/CMA-DO/FOCUS%20GROUPE%20CMA-DO.mp3time=461200)  [00:07:41-2](http://localhost:2300/file=C:/Users/USER/Desktop/ENTRETIENS%20PREMATURITE/CMA-DO/FOCUS%20GROUPE%20CMA-DO.mp3time=461200) Person 6: Non. Si elle n'a pas pu trouver la date des dernières règles le jour de la première CPN, c'est pas sûr qu'elle pourra retrouver la date encore. Puiqu'elle n'a pas pu trouver lors de la première CPN. [00:07:59-8](http://localhost:2300/file=C:/Users/USER/Desktop/ENTRETIENS%20PREMATURITE/CMA-DO/FOCUS%20GROUPE%20CMA-DO.mp3time=479800) |
| --- |

| 00:07:31-6 Interviewer: Est-ce que celles qui se présentent à leur première CPN, plus tard, au cours de la grossesse, elles arrivent toujours à se souvenir de la date de leur dernière règle ? 00:07:41-2  00:07:41-2 Person 6: Non. Si elle n'a pas pu trouver la date des dernières règles le jour de la première CPN, ce n'est pas sûr qu'elle pourra retrouver la date encore. Puisqu'elle n'a pas pu trouver lors de la première CPN. 00:07:59-8 |
| --- |

| **[00:07:31-6] Interviewer**: For those who attend their first ANC, do they manage to remember the date of their last period later during the pregnancy?  **[00:07:41-2] Person 6**: No. If she couldn’t recall the date at the first ANC, it's unlikely she'll remember it later. Since she couldn't find it during the first visit. |
| --- |

### Gynecologist 1

| [00:04:42-6](http://localhost:2300/file=C:/Users/USER/Desktop/ENTRETIENS%20PREMATURITE/CMA-DO/GENYCOLOGUE%2001.mp3time=282600) Interviewer: Est-ce que vous pensez que les femmes enceintes qui se présentent à leur première CPN,plus tard dans la grossesse, elles arrivent toujours à se rappeler de la date de leur dernière règle ? [00:04:53-2](http://localhost:2300/file=C:/Users/USER/Desktop/ENTRETIENS%20PREMATURITE/CMA-DO/GENYCOLOGUE%2001.mp3time=293200)  [00:04:53-2](http://localhost:2300/file=C:/Users/USER/Desktop/ENTRETIENS%20PREMATURITE/CMA-DO/GENYCOLOGUE%2001.mp3time=293200) Person 1: Non, si vraiment elles n'ont pas noté, en dehors de cette application, généralement quand elles viennent 5 ou 6 mois, elles ne se rappellent plus. [00:05:05-1](http://localhost:2300/file=C:/Users/USER/Desktop/ENTRETIENS%20PREMATURITE/CMA-DO/GENYCOLOGUE%2001.mp3time=305100) |
| --- |

| 00:04:42-6 Interviewer: Est-ce que vous pensez que les femmes enceintes qui se présentent à leur première CPN, plus tard dans la grossesse, elles arrivent toujours à se rappeler de la date de leur dernière règle ? 00:04:53-2  00:04:53-2 Person 1: Non, si vraiment elles n'ont pas noté, en dehors de cette application, généralement quand elles viennent 5 ou 6 mois, elles ne se rappellent plus. 00:05:05-1 |
| --- |

| **[00:04:42-6] Interviewer**: Do you think that pregnant women who attend their first ANC later in the pregnancy still remember the date of their last period?  **[00:04:53-2] Person 1**: No, generally, if they haven't noted it down, especially outside of the app, by the time they are 5 or 6 months pregnant, they often don't remember. |
| --- |

### Gynecologist 2

| [00:04:17-7](http://localhost:2300/file=C:/Users/USER/Desktop/ENTRETIENS%20PREMATURITE/CMA-DO/GENYCOLOGUE%2002.mp3time=257700) Interviewer: Maintenant, est-ce que les femmes qui se présentent à leur première CPN, plus tard dans la grossesse, elles arrivent toujours à se rappeler de la date de dernières règles? [00:04:25-1](http://localhost:2300/file=C:/Users/USER/Desktop/ENTRETIENS%20PREMATURITE/CMA-DO/GENYCOLOGUE%2002.mp3time=265100)  [00:04:25-1](http://localhost:2300/file=C:/Users/USER/Desktop/ENTRETIENS%20PREMATURITE/CMA-DO/GENYCOLOGUE%2002.mp3time=265100) Person 1: Non, elles ne se rappellent pas. Voilà pourquoi on saisit l'occasion de la première consultation, si elle est faite précocement. C'est intéressant, ça permet de déjà situer approximativement l'âge du debut de la grossesse, la date des dernières règles. Mais plus on avance dans les suivis de la grossesse elles oublient, mais lorsqu'on a diagnostiquer ça dès le debut, on écrit sur le carnet et ça permet à tout le monde de se référer. [00:04:51-0](http://localhost:2300/file=C:/Users/USER/Desktop/ENTRETIENS%20PREMATURITE/CMA-DO/GENYCOLOGUE%2002.mp3time=291000) |
| --- |

| 00:04:17-7 Interviewer: Maintenant, est-ce que les femmes qui se présentent à leur première CPN, plus tard dans la grossesse, elles arrivent toujours à se rappeler de la date des dernières règles ? 00:04:25-1  00:04:25-1 Person 1: Non, elles ne se rappellent pas. Voilà pourquoi on saisit l'occasion de la première consultation, si elle est faite précocement. C'est intéressant, ça permet de déjà situer approximativement l'âge du début de la grossesse, la date des dernières règles. Mais plus on avance dans les suivis de la grossesse, elles oublient, mais lorsqu'on a diagnostiqué ça dès le début, on écrit sur le carnet et ça permet à tout le monde de se référer. 00:04:51-0 |
| --- |

| **[00:04:17-7] Interviewer**: Now, do women who go to their first ANC later in their pregnancy still manage to remember the date of their last period?  **[00:04:25-1] Person 1**: No, they often don't remember. This is why having the first consultation early is important. It helps us to approximately determine the start of the pregnancy and the date of the last menstrual period. As the pregnancy progresses, they tend to forget. However, when we diagnose pregnancy early on, we document it in their health notebook for future reference. |
| --- |

### Chief physician of Do health district

| [00:07:44-9](http://localhost:2300/file=C:/Users/USER/Desktop/ENTRETIENS%20PREMATURITE/CMA-DO/MCD-DO.mp3time=464900) Interviewer: Est-ce que celle qui vient au premier CPN, plus tard dans la grossesse, elle se rappelle toujours de la date des dernières règles? [00:07:53-6](http://localhost:2300/file=C:/Users/USER/Desktop/ENTRETIENS%20PREMATURITE/CMA-DO/MCD-DO.mp3time=473600)  [00:07:53-6](http://localhost:2300/file=C:/Users/USER/Desktop/ENTRETIENS%20PREMATURITE/CMA-DO/MCD-DO.mp3time=473600) Person 1: Ça peut ne pas être évident, mais c'est que lors de la CPN, une fois que la personne se rappelle, le plus tôt on écrit. Puisque le carnet est prévu, on demande la date des dernières règles, on demande un certain nombre d'informations jusqu'à, les grossesses antérieures, s'il y a eu des fausses couches ou bien si c'est la première fois. On recueil un certain nombre d'informations que nous appelons antécedant pour faciliter le suivi de la grossesse. [00:08:19-5](http://localhost:2300/file=C:/Users/USER/Desktop/ENTRETIENS%20PREMATURITE/CMA-DO/MCD-DO.mp3time=499500) |
| --- |

| 00:07:44-9 Interviewer: Est-ce que celle qui vient au premier CPN, plus tard dans la grossesse, elle se rappelle toujours de la date des dernières règles ? 00:07:53-6  00:07:53-6 Person 1: Ça peut ne pas être évident, mais c'est que lors de la CPN, une fois que la personne se rappelle, le plus tôt on écrit. Puisque le carnet est prévu, on demande la date des dernières règles, on demande un certain nombre d'informations jusqu'à, les grossesses antérieures, s'il y a eu des fausses couches ou bien si c'est la première fois. On recueille un certain nombre d'informations que nous appelons antécédent pour faciliter le suivi de la grossesse. 00:08:19-5 |
| --- |

| **[00:07:44-9] Interviewer**: Does a woman who attends the first ANC later still remember the date of her last period?  **[00:07:53-6] Person 1**: It's not always obvious, but we document it during ANC as soon as she recalls. The ANC record includes various details like the date of the last period, previous pregnancies, and miscarriage history to facilitate pregnancy monitoring. |
| --- |

### Manager for maternity care unit of Do medical center with surgical branch

| [00:06:23-6](http://localhost:2300/file=C:/Users/USER/Desktop/ENTRETIENS%20PREMATURITE/CMA-DO/RESPONSABLE%20UNITE%20DE%20SOIN%20CMA-DO.mp3time=383600) Interviewer: Mais est-ce que celles qui se présentent à la première CPN, est-ce que plus tard dans la grossesse, elles arrivent à se rappeler de la date des dernières règles? [00:06:33-2](http://localhost:2300/file=C:/Users/USER/Desktop/ENTRETIENS%20PREMATURITE/CMA-DO/RESPONSABLE%20UNITE%20DE%20SOIN%20CMA-DO.mp3time=393200)  [00:06:33-2](http://localhost:2300/file=C:/Users/USER/Desktop/ENTRETIENS%20PREMATURITE/CMA-DO/RESPONSABLE%20UNITE%20DE%20SOIN%20CMA-DO.mp3time=393200) Person 1: Bon, en fait, dès le premier contact, comme on demande, et on notifie. Maintenant, peut-être, comme on ne revient pas tout le temps sur ça, voilà. Donc, par rapport à ça, je ne pourrais pas dire, oui, elles peuvent se rappeler. Voilà.On ne revient pas, en fait, sur ça. Voilà. Dès qu'on a déjà notifié, nous, on sait que c'est dans le carnet. Voilà. On continue la CPN tout en se référant toujours à cette date qu'elle nous a donnée. On ne revient plus pour lui demander.est-ce que encore tu te rappelles de ta date de dernière règle? Parce qu'on se dit que c'est un acquis déjà, c'est dans le carnet. Voilà. Mais je pense que, peut-être, si on demandait, elles allaient se rappeler. Parce que, surtout celles qui savent lire, puisque c'est déjà dans le carnet, c'est noté. Voilà. [00:07:19-0](http://localhost:2300/file=C:/Users/USER/Desktop/ENTRETIENS%20PREMATURITE/CMA-DO/RESPONSABLE%20UNITE%20DE%20SOIN%20CMA-DO.mp3time=439000) |
| --- |

| 00:06:23-6 Interviewer: Mais est-ce que celles qui se présentent à la première CPN, est-ce que plus tard dans la grossesse, elles arrivent à se rappeler de la date des dernières règles ? 00:06:33-2  00:06:33-2 Person 1: Bon, en fait, dès le premier contact, comme on demande, et on notifie. Maintenant, peut-être, comme on ne revient pas tout le temps sur ça, voilà. Donc, par rapport à ça, je ne pourrais pas dire, oui, elles peuvent se rappeler. Voilà. On ne revient pas, en fait, sur ça. Voilà. Dès qu'on a déjà notifié, nous, on sait que c'est dans le carnet. Voilà. On continue la CPN tout en se référant toujours à cette date qu'elle nous a donnée. On ne revient plus pour lui demander. Est-ce que tu te rappelles encore de ta date de dernière règle ? Parce qu'on se dit que c'est un acquis déjà, c'est dans le carnet. Voilà. Mais je pense que, peut-être, si on demandait, elles allaient se rappeler. Parce que, surtout celles qui savent lire, puisque c'est déjà dans le carnet, c'est noté. Voilà. 00:07:19-0 |
| --- |

| **Interviewer (00:06:23-6):** When women come in for their first antenatal care (ANC) visit later in their pregnancy, are they usually able to remember the date of their last menstrual period?  **[00:06:33-2] Person 1**: Once we ask and record the date, we don’t usually revisit the question. We continue the ANC referring to the initially given date. It’s possible they might remember, especially if they can read and have noted it in their notebook, but we don’t typically ask them to recall it again since it's already documented. |
| --- |

### Focus group of Colma1 health and social promotion center

| [00:07:38-2](http://localhost:2300/file=C:/Users/USER/Desktop/ENTRETIENS%20PREMATURITE/CSPS-COLMA1/FOCUS%20GROUPE%20COLMA1.mp3time=458200) Interviewer: Maintenant, est-ce que celle qui viennent à leur première CPN, plus tard dans la grossesse,elles se souviennent toujours de la date de leure dernière règle ? [00:07:47-1](http://localhost:2300/file=C:/Users/USER/Desktop/ENTRETIENS%20PREMATURITE/CSPS-COLMA1/FOCUS%20GROUPE%20COLMA1.mp3time=467100)  [00:07:47-1](http://localhost:2300/file=C:/Users/USER/Desktop/ENTRETIENS%20PREMATURITE/CSPS-COLMA1/FOCUS%20GROUPE%20COLMA1.mp3time=467100) Person 3: Elle se? [00:07:47-6](http://localhost:2300/file=C:/Users/USER/Desktop/ENTRETIENS%20PREMATURITE/CSPS-COLMA1/FOCUS%20GROUPE%20COLMA1.mp3time=467600)  [00:07:47-6](http://localhost:2300/file=C:/Users/USER/Desktop/ENTRETIENS%20PREMATURITE/CSPS-COLMA1/FOCUS%20GROUPE%20COLMA1.mp3time=467600) Interviewer: Est ce qu'elles se souviennent toujours de leur date des dernières règles [00:07:50-3](http://localhost:2300/file=C:/Users/USER/Desktop/ENTRETIENS%20PREMATURITE/CSPS-COLMA1/FOCUS%20GROUPE%20COLMA1.mp3time=470300)  [00:07:50-3](http://localhost:2300/file=C:/Users/USER/Desktop/ENTRETIENS%20PREMATURITE/CSPS-COLMA1/FOCUS%20GROUPE%20COLMA1.mp3time=470300) Person 3: Elles ne se souviennent jamais à ma connaissance en tout cas. Voilà dès le début, dès qu'elle dise qu'elles ne connaissent pas, <ça fait un mois là> c'est comme ça et cela continue.Souvent même elle vous demande ça fait combien de mois ?Elle-même, parce qu'elles ne savent pas, elles vont te demander que mais< tu m'as regardé là sa fait combien de mois ?> Voilà. nous avons l'habitude de regarder dans le carnet c'est écrit, selon eux bon,c'est pour la forme. [00:08:17-1](http://localhost:2300/file=C:/Users/USER/Desktop/ENTRETIENS%20PREMATURITE/CSPS-COLMA1/FOCUS%20GROUPE%20COLMA1.mp3time=497100) |
| --- |

| 00:07:38-2 Interviewer: Maintenant, est-ce que celles qui viennent à leur première CPN, plus tard dans la grossesse, se souviennent toujours de la date de leur dernière règle ? 00:07:47-1  00:07:47-1 Person 3: Elles se ? 00:07:47-6  00:07:47-6 Interviewer: Est-ce qu'elles se souviennent toujours de leur date des dernières règles ? 00:07:50-3  00:07:50-3 Person 3: Elles ne se souviennent jamais, à ma connaissance en tout cas. Voilà, dès le début, dès qu'elles disent qu'elles ne connaissent pas, <ça fait un mois là>, c'est comme ça et cela continue. Souvent même elle vous demande ça fait combien de mois ? Elle-même, parce qu'elles ne savent pas, elles vont te demander que mais <tu m'as regardé là, ça fait combien de mois ?> Voilà. Nous avons l'habitude de regarder dans le carnet c'est écrit, selon eux bon, c'est pour la forme. 00:08:17-1 |
| --- |

| **[00:07:38-2] Interviewer**: Do those who come for their first ANC later in their pregnancy still remember the date of their last period?  **[00:07:47-1] Person 3**: Remember? They usually never remember. From the start, they might say it's been a month, and it continues like that. They often even ask us how many months it has been. We usually refer to the notebook, but it's more a formality. |
| --- |

### Major of Colma1 health and social promotion center

| [00:04:01-5](http://localhost:2300/file=C:/Users/USER/Desktop/ENTRETIENS%20PREMATURITE/CSPS-COLMA1/MAJOR%20CSPS%20COLMA1.MP3time=241500) Interviewer: est ce que les femmes qui se présentent à leur première CPN plus tard dans la grossesse, se souviennent toujours de la date des dernières règles? [00:04:08-2](http://localhost:2300/file=C:/Users/USER/Desktop/ENTRETIENS%20PREMATURITE/CSPS-COLMA1/MAJOR%20CSPS%20COLMA1.MP3time=248200)  [00:04:08-2](http://localhost:2300/file=C:/Users/USER/Desktop/ENTRETIENS%20PREMATURITE/CSPS-COLMA1/MAJOR%20CSPS%20COLMA1.MP3time=248200) Person 1: Les femmes qui viennent? [00:04:10-7](http://localhost:2300/file=C:/Users/USER/Desktop/ENTRETIENS%20PREMATURITE/CSPS-COLMA1/MAJOR%20CSPS%20COLMA1.MP3time=250700)  [00:04:10-7](http://localhost:2300/file=C:/Users/USER/Desktop/ENTRETIENS%20PREMATURITE/CSPS-COLMA1/MAJOR%20CSPS%20COLMA1.MP3time=250700) Interviewer: À la CPN est-ce que plus tard dans la grossesse,elle se souvient toujours de la date des dernières règles ? [00:04:15-3](http://localhost:2300/file=C:/Users/USER/Desktop/ENTRETIENS%20PREMATURITE/CSPS-COLMA1/MAJOR%20CSPS%20COLMA1.MP3time=255300)  [00:04:15-3](http://localhost:2300/file=C:/Users/USER/Desktop/ENTRETIENS%20PREMATURITE/CSPS-COLMA1/MAJOR%20CSPS%20COLMA1.MP3time=255300) Person 1: Pas toujours. parce que plus le temps est entrain de passer ? Non, elle est revient plus en arrière, mais elle se dit, c'est comme ce qui était au tout début. Certains vont dire que au début, au premier trimestre,quand elle vient pour la première CPN. Si les 3 là certaines vont dire en tout cas, <je sait que c'est peut-être le mois passé que j'ai vu mes dernières règles. Voilà, après ça, je n'ai plus vu, c'est le moi sur le passé, j'ai vu mes dernières, après je n'ai plus vu mes règles > mais, plus, à partir du moment ou,on a commencer la CPN et on est entrain d'avancer elle va dire que maintenant ma grossesse fait tant de mois c'est ce qu'elles vont dire, mais elles ne vont plus se rappeler la date exacte. [00:04:54-7](http://localhost:2300/file=C:/Users/USER/Desktop/ENTRETIENS%20PREMATURITE/CSPS-COLMA1/MAJOR%20CSPS%20COLMA1.MP3time=294700) |
| --- |

| 00:04:01-5 Interviewer: Est-ce que les femmes qui se présentent à leur première CPN plus tard dans la grossesse, se souviennent toujours de la date des dernières règles ? 00:04:08-2  00:04:08-2 Person 1: Les femmes qui viennent ? 00:04:10-7  00:04:10-7 Interviewer: À la CPN est-ce que plus tard dans la grossesse, elle se souvient toujours de la date des dernières règles ? 00:04:15-3  00:04:15-3 Person 1: Pas toujours. Parce que plus le temps est en train de passer ? Non, elle revient plus en arrière, mais elle se dit, c'est comme ce qui était au tout début. Certains vont dire qu'au début, au premier trimestre, quand elle vient pour la première CPN. Si les 3 là certaines vont dire en tout cas, <je sais que c'est peut-être le mois passé que j'ai vu mes dernières règles. Voilà, après ça, je n'ai plus vu, c'est le mois sur le passé, j'ai vu mes dernières, après je n'ai plus vu mes règles > mais, plus, à partir du moment où on a commencé la CPN et on est en train d'avancer elle va dire que maintenant ma grossesse fait tant de mois c'est ce qu'elles vont dire, mais elles ne vont plus se rappeler la date exacte. 00:04:54-7 |
| --- |

| **[00:04:01-5] Interviewer**: Do women who attend their first ANC later in pregnancy still remember the date of their last period?  **[00:04:08-2] Person 1**: Not always. As time passes, they start to forget. At the beginning, in the first trimester, some might recall it was about a month ago when they last saw their period. But as the pregnancy progresses and they start prenatal care, they tend to focus more on how many months pregnant they are, rather than remembering the exact date of their last period. |
| --- |

### Maternity manager of Colma1 health and social promotion center

| [00:10:34-6](http://localhost:2300/file=C:/Users/USER/Desktop/ENTRETIENS%20PREMATURITE/CSPS-COLMA1/RESPONSABLE%20MATERNITE-COLMA1.mp3time=634600) Interviewer: D'accord.Maintenant, est- ce que celle qui vient à la première CPN, plus tard dans la grossesse, est- ce qu'elle se souvient toujours de la date des dernières règles ? [00:10:46-6](http://localhost:2300/file=C:/Users/USER/Desktop/ENTRETIENS%20PREMATURITE/CSPS-COLMA1/RESPONSABLE%20MATERNITE-COLMA1.mp3time=646600)  [00:10:46-6](http://localhost:2300/file=C:/Users/USER/Desktop/ENTRETIENS%20PREMATURITE/CSPS-COLMA1/RESPONSABLE%20MATERNITE-COLMA1.mp3time=646600) Person 1: Au premier trimestre. La plupart, lorsque l'interrogatoire est bien ménée, elle arrive à vous dire c'est quel mois. La date exacte, non, mais à peu près oui. [00:11:04-2](http://localhost:2300/file=C:/Users/USER/Desktop/ENTRETIENS%20PREMATURITE/CSPS-COLMA1/RESPONSABLE%20MATERNITE-COLMA1.mp3time=664200) |
| --- |

| 00:10:34-6 Interviewer: D'accord. Maintenant, est-ce que celles qui viennent à leur première CPN, plus tard dans la grossesse, se souviennent toujours de la date des dernières règles ? 00:10:46-6  00:10:46-6 Person 1: Au premier trimestre. La plupart, lorsque l'interrogatoire est bien mené, elle arrive à vous dire c'est quel mois. La date exacte, non, mais à peu près oui. 00:11:04-2 |
| --- |

| **[00:10:34-6] Interviewer**: Do those who come for their first ANC later in the pregnancy still remember the date of their last period?  **[00:10:46-6] Person 1**: In the first trimester, if questioned properly, most women can recall the month of their last period. The exact date is often not remembered, but they can usually provide a close approximation. |
| --- |

### Focus group of Farakan health and social promotion center

| [00:11:24-2](http://localhost:2300/file=C:/Users/USER/Desktop/ENTRETIENS%20PREMATURITE/CSPS_FARAKAN/FOCUS_GROUPE-CSPS_FARAKAN.MP3time=684200) Interviewer: Ok, maintenant Pensez-vous que les femmes connaissent avec précision la date de leur dernière règle lors de leur première CPN ? [00:11:37-0](http://localhost:2300/file=C:/Users/USER/Desktop/ENTRETIENS%20PREMATURITE/CSPS_FARAKAN/FOCUS_GROUPE-CSPS_FARAKAN.MP3time=697000)  [00:11:37-0](http://localhost:2300/file=C:/Users/USER/Desktop/ENTRETIENS%20PREMATURITE/CSPS_FARAKAN/FOCUS_GROUPE-CSPS_FARAKAN.MP3time=697000) Person 3: Je ne pense pas, la preuve en est que quand elles vont donner des dates, pourtant nous allons pronostiquer des dépassements. Mais en réalité, après investigation, on se rend compte qu' il n'y a pas de dépassement. [00:11:51-6](http://localhost:2300/file=C:/Users/USER/Desktop/ENTRETIENS%20PREMATURITE/CSPS_FARAKAN/FOCUS_GROUPE-CSPS_FARAKAN.MP3time=711600)  [00:11:51-6](http://localhost:2300/file=C:/Users/USER/Desktop/ENTRETIENS%20PREMATURITE/CSPS_FARAKAN/FOCUS_GROUPE-CSPS_FARAKAN.MP3time=711600) Person 5: En tout cas, beaucoup de femmes ne connaissent pas la date exacte de leur dernière règle. [00:12:01-6](http://localhost:2300/file=C:/Users/USER/Desktop/ENTRETIENS%20PREMATURITE/CSPS_FARAKAN/FOCUS_GROUPE-CSPS_FARAKAN.MP3time=721600)  [00:12:01-6](http://localhost:2300/file=C:/Users/USER/Desktop/ENTRETIENS%20PREMATURITE/CSPS_FARAKAN/FOCUS_GROUPE-CSPS_FARAKAN.MP3time=721600) Person 7: La plupart des femmes, du faite que d'autres aussi sont sous les méthodes contraceptive, après peut-être elles sont sous dépôt, avec absence de règles après elles reviennent dire qu'elles sont surprises qu'il a une grossesse donc souvent c'est difficile de connaître l'âge exact ? Leur DDR (date des dernières règles), elle ne maîtrise pas.Il y en a qui maîtrise en tout cas mais c'est quelques unes seulement. [00:12:34-1](http://localhost:2300/file=C:/Users/USER/Desktop/ENTRETIENS%20PREMATURITE/CSPS_FARAKAN/FOCUS_GROUPE-CSPS_FARAKAN.MP3time=754100)  [00:12:34-1](http://localhost:2300/file=C:/Users/USER/Desktop/ENTRETIENS%20PREMATURITE/CSPS_FARAKAN/FOCUS_GROUPE-CSPS_FARAKAN.MP3time=754100) Person 4: Elles ne connaissent pas. [00:12:38-2](http://localhost:2300/file=C:/Users/USER/Desktop/ENTRETIENS%20PREMATURITE/CSPS_FARAKAN/FOCUS_GROUPE-CSPS_FARAKAN.MP3time=758200)  [00:12:38-2](http://localhost:2300/file=C:/Users/USER/Desktop/ENTRETIENS%20PREMATURITE/CSPS_FARAKAN/FOCUS_GROUPE-CSPS_FARAKAN.MP3time=758200) Person 8: Quelquefois elles te disent que la Tabaski passée, ou bien lors de ramadan, elles n'ont plus vue leur règle, donc tu les aides à compter à partir de cette date. Sinon vraiment elle détermine pas cela bien. [00:12:57-0](http://localhost:2300/file=C:/Users/USER/Desktop/ENTRETIENS%20PREMATURITE/CSPS_FARAKAN/FOCUS_GROUPE-CSPS_FARAKAN.MP3time=777000)  [00:12:57-0](http://localhost:2300/file=C:/Users/USER/Desktop/ENTRETIENS%20PREMATURITE/CSPS_FARAKAN/FOCUS_GROUPE-CSPS_FARAKAN.MP3time=777000) Interviewer: Est-ce que les informations par rapport à cette précision sont de l'ordre des semaines ou des mois ? [00:13:02-6](http://localhost:2300/file=C:/Users/USER/Desktop/ENTRETIENS%20PREMATURITE/CSPS_FARAKAN/FOCUS_GROUPE-CSPS_FARAKAN.MP3time=782600)  [00:13:02-6](http://localhost:2300/file=C:/Users/USER/Desktop/ENTRETIENS%20PREMATURITE/CSPS_FARAKAN/FOCUS_GROUPE-CSPS_FARAKAN.MP3time=782600) Person 2: L'ordre des mois. [00:13:19-6](http://localhost:2300/file=C:/Users/USER/Desktop/ENTRETIENS%20PREMATURITE/CSPS_FARAKAN/FOCUS_GROUPE-CSPS_FARAKAN.MP3time=799600) |
| --- |

| 00:11:24-2 Interviewer: Ok, maintenant pensez-vous que les femmes connaissent avec précision la date de leur dernière règle lors de leur première CPN ? 00:11:37-0  00:11:37-0 Person 3: Je ne pense pas, la preuve en est que quand elles vont donner des dates, pourtant nous allons pronostiquer des dépassements. Mais en réalité, après investigation, on se rend compte qu'il n'y a pas de dépassement. 00:11:51-6  00:11:51-6 Person 5: En tout cas, beaucoup de femmes ne connaissent pas la date exacte de leur dernière règle. 00:12:01-6  00:12:01-6 Person 7: La plupart des femmes, du fait que d'autres aussi sont sous les méthodes contraceptives, après peut-être elles sont sous dépôt, avec absence de règles, après elles reviennent dire qu'elles sont surprises qu'il y ait une grossesse. Donc souvent c'est difficile de connaître l'âge exact de leur DDR (date des dernières règles), elles ne maîtrisent pas. Il y en a qui maîtrisent en tout cas, mais ce sont quelques-unes seulement. 00:12:34-1  00:12:34-1 Person 4: Elles ne connaissent pas. 00:12:38-2  00:12:38-2 Person 8: Quelquefois elles te disent que la Tabaski est passée, ou bien lors du Ramadan, elles n'ont plus vu leurs règles, donc tu les aides à compter à partir de cette date. Sinon vraiment elles ne déterminent pas cela bien. 00:12:57-0  00:12:57-0 Interviewer: Est-ce que les informations par rapport à cette précision sont de l'ordre des semaines ou des mois ? 00:13:02-6  00:13:02-6 Person 2: L'ordre des mois. 00:13:19-6 |
| --- |

| **[00:11:24-2] Interviewer**: Do you think women precisely know the date of their last period when they first come for ANC?  **[00:11:37-0] Person 3**: I don't think so. Often, when they give dates, we predict excesses, but after investigation, we find there is no excess.  **[00:11:51-6] Person 5**: Many women do not know the exact date of their last period.  **[00:12:01-6] Person 7**: It's challenging because some are on contraceptive methods which affect their menstruation. They might be surprised by a pregnancy and struggle to know the exact date of their last period. Some are aware, but they are few.  **[00:12:34-1] Person 4**: They generally don't know.  **[00:12:38-2] Person 8**: Sometimes they relate it to events, like not seeing their period since Tabaski or Ramadan, so we help them count from there. They don't determine it precisely.  **[00:12:57-0] Interviewer**: Is this estimation usually done in terms of weeks or months?  **[00:13:02-6] Person 2**: It's in terms of months. |
| --- |

### Major of Farakan health and social promotion center

| [00:04:17-7](http://localhost:2300/file=C:/Users/USER/Desktop/ENTRETIENS%20PREMATURITE/CSPS_FARAKAN/MAJOR%20FARAKAN.MP3time=257700) Interviewer: Maintenant, est-ce que les femmes qui se présentent à leur première, plus tard dans la grossesse ? Est-ce qu'elles arrivent à se souvenir toujours de la date de la dernière règle ? [00:04:28-5](http://localhost:2300/file=C:/Users/USER/Desktop/ENTRETIENS%20PREMATURITE/CSPS_FARAKAN/MAJOR%20FARAKAN.MP3time=268500)  [00:04:28-5](http://localhost:2300/file=C:/Users/USER/Desktop/ENTRETIENS%20PREMATURITE/CSPS_FARAKAN/MAJOR%20FARAKAN.MP3time=268500) Person 1: C'est surtout les lettrés là, c'est à dire les fonctionnaires, ceux qui en tout cas ceux qui connaissent lire et bien écrire.celles qui connaissent bien leur organisme, elles se rappellent, mais les analphabètes là (rire), il faut les aider avec les calendriers et autres. [00:04:45-5](http://localhost:2300/file=C:/Users/USER/Desktop/ENTRETIENS%20PREMATURITE/CSPS_FARAKAN/MAJOR%20FARAKAN.MP3time=285500) |
| --- |

| 00:04:17-7 Interviewer: Maintenant, est-ce que les femmes qui se présentent à leur première CPN plus tard dans la grossesse ? Est-ce qu'elles arrivent à se souvenir toujours de la date de la dernière règle ? 00:04:28-5  00:04:28-5 Person 1: C'est surtout les lettrés là, c'est-à-dire les fonctionnaires, ceux qui, en tout cas, connaissent lire et bien écrire. Celles qui connaissent bien leur organisme, elles se rappellent, mais les analphabètes là (rire), il faut les aider avec les calendriers et autres. 00:04:45-5 |
| --- |

| **[00:04:17-7] Interviewer**: For women who present for their first ANC later in their pregnancy, can they always remember the date of the last period?  **[00:04:28-5] Person 1**: It's mainly the literate people, like civil servants, who know how to read and write, that remember. For illiterate ones, we often have to assist them with calendars and such. |
| --- |

### Maternity manager of Farakan health and social promotion center

| [00:05:44-0](http://localhost:2300/file=C:/Users/USER/Desktop/ENTRETIENS%20PREMATURITE/CSPS_FARAKAN/RESPONSABLE%20MATERNITE%20FARAKAN.MP3time=344000) Interviewer: Est-ce que vous pensez que les femmes connaissent avec précision la date de la dernière règle lors de leur première CPN? [00:05:53-3](http://localhost:2300/file=C:/Users/USER/Desktop/ENTRETIENS%20PREMATURITE/CSPS_FARAKAN/RESPONSABLE%20MATERNITE%20FARAKAN.MP3time=353300)  [00:05:53-3](http://localhost:2300/file=C:/Users/USER/Desktop/ENTRETIENS%20PREMATURITE/CSPS_FARAKAN/RESPONSABLE%20MATERNITE%20FARAKAN.MP3time=353300) Person 1: Oui, Il y en a qui connaissent avec précision. Il y en a souvent, elles vont peut-être donner par rapport aux fêtes , ça peut être une fête musulmane pendant le ramadan ou bien pendant le mois de carême que j'ai vu, mais souvent, mais la date exacte là c'est compliqué. Mais souvent quand on nous donne le mois par rapport au mois on peut déjà calculer. [00:06:17-0](http://localhost:2300/file=C:/Users/USER/Desktop/ENTRETIENS%20PREMATURITE/CSPS_FARAKAN/RESPONSABLE%20MATERNITE%20FARAKAN.MP3time=377000)  [00:06:17-0](http://localhost:2300/file=C:/Users/USER/Desktop/ENTRETIENS%20PREMATURITE/CSPS_FARAKAN/RESPONSABLE%20MATERNITE%20FARAKAN.MP3time=377000) Interviewer: Donc la precision c'est de l'ordre du mois, ce n'est pas en semaines? [00:06:23-4](http://localhost:2300/file=C:/Users/USER/Desktop/ENTRETIENS%20PREMATURITE/CSPS_FARAKAN/RESPONSABLE%20MATERNITE%20FARAKAN.MP3time=383400)  [00:06:23-4](http://localhost:2300/file=C:/Users/USER/Desktop/ENTRETIENS%20PREMATURITE/CSPS_FARAKAN/RESPONSABLE%20MATERNITE%20FARAKAN.MP3time=383400) Person 1: Oui c'est en terme de mois, ce n'est pas le nombre de semaines d'aménorrhées [00:06:33-3](http://localhost:2300/file=C:/Users/USER/Desktop/ENTRETIENS%20PREMATURITE/CSPS_FARAKAN/RESPONSABLE%20MATERNITE%20FARAKAN.MP3time=393300) |
| --- |

| 00:05:44-0 Interviewer: Est-ce que vous pensez que les femmes connaissent avec précision la date de la dernière règle lors de leur première CPN ? 00:05:53-3  00:05:53-3 Person 1: Oui, il y en a qui connaissent avec précision. Il y en a souvent, elles vont peut-être donner par rapport aux fêtes, ça peut être une fête musulmane pendant le ramadan ou bien pendant le mois de carême que j'ai vu, mais souvent, mais la date exacte là c'est compliqué. Mais souvent quand on nous donne le mois par rapport au mois on peut déjà calculer. 00:06:17-0  00:06:17-0 Interviewer: Donc la précision c'est de l'ordre du mois, ce n'est pas en semaines ? 00:06:23-4  00:06:23-4 Person 1: Oui, c'est en terme de mois, ce n'est pas le nombre de semaines d'aménorrhée. 00:06:33-3 |
| --- |

| **[00:05:44-0] Interviewer**: Do you think women precisely know the date of their last period when they have their first ANC?  **[00:05:53-3] Person 1**: Yes, some do know precisely. They often relate it to holidays, like during Ramadan or Lent, to estimate the month, but the exact date is more complicated. We can usually calculate based on the month they provide.  **[00:06:17-0] Interviewer**: So, the precision is more in terms of months rather than weeks?  **[00:06:23-4] Person 1**: Yes, it's usually in terms of months, not the number of weeks of amenorrhea. |
| --- |

## Ways health workers help women remember the date of their last period

### Focus group of Accart-ville urban medical center

| [00:04:51-8](http://localhost:2300/file=C:/Users/USER/Desktop/ENTRETIENS) Interviewer: maintenant s'elles ne souviennent pas du tout, comment est ce que vous procedez pour determiner l'äge gestationnel? [00:04:58-0](http://localhost:2300/file=C:/Users/US)  [00:04:58-0](http://localhost:2300/file=C:/Users/USER/Desktop/ENTRETIENS) Person 1: si, elles ne se souviennent pas,nous pouvons mesurer par la mensturation pour voire à peu près la hauteur uterine. souvent aussi on peut utiliser l'echographie [00:05:14-5](http://localhost:2300/file=C:/Users/USER/Desktop/ENTRETIENS%20PREMATURITE/CSPS-ACCART-VILLE/FOCUS%20GROUPE%20ACCART-VILLE-BON.mp3time=314500) |
| --- |

| 00:04:51-8 Interviewer: Maintenant, si elles ne se souviennent pas du tout, comment est-ce que vous procédez pour déterminer l'âge gestationnel ? 00:04:58-0  00:04:58-0 Person 1: Si elles ne se souviennent pas, nous pouvons mesurer par la menstruation pour voir à peu près la hauteur utérine. Souvent aussi, on peut utiliser l'échographie. 00:05:14-5 |
| --- |

| **[00:04:51-8] Interviewer**: If women don't remember the date of their last period at all, how do you determine gestational age?  **[00:04:58-0] Person 1**: If they can't recall, we use alternative methods. One approach is to measure the fundal height, which can give us an approximation of gestational age. We also frequently use ultrasound as it can provide a more precise measurement. |
| --- |

### Manager of Accart-ville urban medical center

| [00:05:58-5](http://localhost:2300/file=C:/Users/USER/Desktop/ENTRETIENS%20PREMATURITE/CSPS-ACCART-VILLE/RESPONSABLE%20CMU%20ACCART-VILLE.MP3time=358500)Interviewer: maintenant au cas ou les femmes ne se souviennent même pas du tout comment est ce que vous vous prenez pour déterminer l'âge gestationnel? [00:06:05-8](http://localhost:2300/file=C:/Users/USER/Desktop/ENTRETIENS%20PREMATURITE/CSPS-ACCART-VILLE/RESPONSABLE%20CMU%20ACCART-VILLE.MP3time=365800)  [00:06:05-8](http://localhost:2300/file=C:/Users/USER/Desktop/ENTRETIENS%20PREMATURITE/CSPS-ACCART-VILLE/RESPONSABLE%20CMU%20ACCART-VILLE.MP3time=365800)Person 1: l'äge gestationnel. generalement on se base sur l'âge échographique s'elles n'arrivent pas à donner la date des dernières règles, puisqu'il y a des dames aussi qui ne réalisent pas de d'échographie. en se moment on sera obligé de partir avec le nombre de mois qu'elles vont nous donner on essaie d'estimer ou par rapport à l'examen pour voir la hauteur utérine. [00:06:29-7](http://localhost:2300/file=C:/Users/USER/Desktop/ENTRETIENS%20PREMATURITE/CSPS-ACCART-VILLE/RESPONSABLE%20CMU%20ACCART-VILLE.MP3time=389700) |
| --- |

| 00:05:58-5 Interviewer: Maintenant, au cas où les femmes ne se souviennent même pas du tout, comment est-ce que vous vous prenez pour déterminer l'âge gestationnel ? 00:06:05-8  00:06:05-8 Person 1: L'âge gestationnel. Généralement, on se base sur l'âge échographique si elles n'arrivent pas à donner la date des dernières règles. Puisqu'il y a des dames aussi qui ne réalisent pas d'échographie. En ce moment, on sera obligé de partir avec le nombre de mois qu'elles vont nous donner, on essaie d'estimer ou par rapport à l'examen pour voir la hauteur utérine. 00:06:29-7 |
| --- |

| **[00:05:58-5] Interviewer**: In cases where women can't remember their last period at all, how do you determine gestational age?  **[00:06:05-8] Person 1**: When determining gestational age and the woman is unable to recall the date of her last period, we primarily rely on ultrasound measurements. However, there are cases where women haven't undergone an ultrasound. In such situations, we start by considering the number of months they think they might be pregnant. We also estimate based on a physical examination, particularly by assessing the uterine height. |
| --- |

### Maternity manager of Accart-ville urban medical center

| [00:05:07-5](http://localhost:2300/file=C:/Users/USER/Desktop/ENTRETIENS%20PREMATURITE/CSPS-ACCART-VILLE/RESPONSABLE%20MATERNITE%20CMU%20ACCART-VILLE.MP3time=307500) Interviewer: maintenant si la femme ne souvient pas du tout de ces dates comment est ce que la sage femme ou comment est ce que l'âge gestationnel est elle determiné? [00:05:18-1](http://localhost:2300/file=C:/Users/USER/Desktop/ENTRETIENS%20PREMATURITE/CSPS-ACCART-VILLE/RESPONSABLE%20MATERNITE%20CMU%20ACCART-VILLE.MP3time=318100)  [00:05:18-1](http://localhost:2300/file=C:/Users/USER/Desktop/ENTRETIENS%20PREMATURITE/CSPS-ACCART-VILLE/RESPONSABLE%20MATERNITE%20CMU%20ACCART-VILLE.MP3time=318100) Person 1: donc on peut aller sur la base, sur le plan clinique hein donc à defaut de l'âge chronologique qu'on determine en fonction de la date des dernières règles si la femme ne se rappel pas, on part sur le plan clinique. sur le plan clinique voila donc si ça aussi on a des difficultés on peut demander l'échographie si toutefois ya pas un problème de moyens financiers de la femme comme le centre ne dispose pas d'appareil échographe on la fait faire ça en externe voila pourquoi ça, ça vient en dernier recours, l'âge clinique en fonction de la hauteur uterine qu'on determine. [00:05:59-1](http://localhost:2300/file=C:/Users/USER/Desktop/ENTRETIENS%20PREMATURITE/CSPS-ACCART-VILLE/RESPONSABLE%20MATERNITE%20CMU%20ACCART-VILLE.MP3time=359100) |
| --- |

| 00:05:07-5 Interviewer: Maintenant, si la femme ne souvient pas du tout de ces dates, comment est-ce que la sage-femme ou comment est-ce que l'âge gestationnel est-elle déterminé ? 00:05:18-1  00:05:18-1 Person 1: Donc on peut aller sur la base, sur le plan clinique hein. Donc à défaut de l'âge chronologique qu'on détermine en fonction de la date des dernières règles si la femme ne se rappelle pas, on part sur le plan clinique. Sur le plan clinique voilà donc si ça aussi on a des difficultés on peut demander l'échographie si toutefois il n'y a pas de problème de moyens financiers de la femme, comme le centre ne dispose pas d'appareil échographe on la fait faire ça en externe. Voilà pourquoi ça, ça vient en dernier recours, l'âge clinique en fonction de la hauteur utérine qu'on détermine. 00:05:59-1 |
| --- |

| **[00:05:07-5] Interviewer**: If a woman doesn't remember the dates at all, how does the midwife determine the gestational age?  **[00:05:18-1] Person 1**: In such cases, we rely on clinical assessments. When the chronological age, which is usually determined based on the date of the last period, is unknown, we start with a clinical approach. If we face difficulties on the clinical level, and if the woman's financial situation allows, we might request an ultrasound. However, as our center doesn't have an ultrasound machine, this would need to be done externally. Therefore, it's a last resort. We generally determine the clinical age based on the measurement of the uterine height. |
| --- |

### Focus group of Do medical center with surgical branch

| [00:07:59-8](http://localhost:2300/file=C:/Users/USER/Desktop/ENTRETIENS%20PREMATURITE/CMA-DO/FOCUS%20GROUPE%20CMA-DO.mp3time=479800) Interviewer: Maintenant, si les femmes ne se souviennent pas de la date de leur dernière règle, comment est-ce que vous procédez ici pour determiner l'âge gestationnel? [00:07:46-7](http://localhost:2300/file=C:/Users/USER/Desktop/ENTRETIENS%20PREMATURITE/CMA-DO/FOCUS%20GROUPE%20CMA-DO.mp3time=466700) [00:08:08-3](http://localhost:2300/file=C:/Users/USER/Desktop/ENTRETIENS%20PREMATURITE/CMA-DO/FOCUS%20GROUPE%20CMA-DO.mp3time=488300)  [00:08:08-3](http://localhost:2300/file=C:/Users/USER/Desktop/ENTRETIENS%20PREMATURITE/CMA-DO/FOCUS%20GROUPE%20CMA-DO.mp3time=488300) Person 6: Bon, il y a des événements. La femme peut dire que peut-être c'est "Sounkalo", ou mois de carène, ou bien un tabaski, qu'elle n'a pas eu. Le mois de tabaski, le mois de carène, par exemple. Si le moi de tabaski était en juin, en calcul, bon,en juin, elle a eu ses derniers règles. On suppose que c'est en Mars qu'elle doit accoucher. [00:08:32-1](http://localhost:2300/file=C:/Users/USER/Desktop/ENTRETIENS%20PREMATURITE/CMA-DO/FOCUS%20GROUPE%20CMA-DO.mp3time=512100) |
| --- |

| 00:07:59-8 Interviewer: Maintenant, si les femmes ne se souviennent pas de la date de leur dernière règle, comment est-ce que vous procédez ici pour déterminer l'âge gestationnel ? 00:07:46-7 00:08:08-3  00:08:08-3 Person 6: Bon, il y a des événements. La femme peut dire que peut-être c'est "Sounkalo", ou le mois de carême, ou bien un tabaski, qu'elle n'a pas eu. Le mois de tabaski, le mois de carême, par exemple. Si le mois de tabaski était en juin, en calcul, bon, en juin, elle a eu ses dernières règles. On suppose que c'est en mars qu'elle doit accoucher. 00:08:32-1 |
| --- |

| **[00:07:59-8] Interviewer**: If women don't remember the date of their last period, how do you determine gestational age here?  **[00:08:08-3] Person 6**: In such cases, we refer to significant events that the woman can recall. She might mention that she didn't have her period during a specific time like 'Sounkalo', the month of Lent, or Tabaski. For instance, if she recalls not having her period during the month of Tabaski and if Tabaski was in June, we calculate from that point. Based on this, if her last period was in June, we would estimate that her due date is around March. |
| --- |

### Gynecologist 1

| [00:05:05-1](http://localhost:2300/file=C:/Users/USER/Desktop/ENTRETIENS%20PREMATURITE/CMA-DO/GENYCOLOGUE%2001.mp3time=305100) Maintenant au cas où la femme enceinte ne se souvient pas du tout,comment est-ce que vous procédez pour établir l'âge de la grossesse ? [00:05:13-1](http://localhost:2300/file=C:/Users/USER/Desktop/ENTRETIENS%20PREMATURITE/CMA-DO/GENYCOLOGUE%2001.mp3time=313100)  [00:05:13-1](http://localhost:2300/file=C:/Users/USER/Desktop/ENTRETIENS%20PREMATURITE/CMA-DO/GENYCOLOGUE%2001.mp3time=313100) Person 1: Si elle ne se souvient pas du tout, ici on a deux options. Soit on se base sur la hauteur utérine, parce qu'il y a une corrélation entre la hauteur utérine et l'âge de la grossesse. Voilà.Et ça, ça vous permet d'avoir l'âge de la grossesse à peu près en mois. C'est pas précis à chose près, mais ça vous donne une idée. L'autre chose aussi, on a l'avantage d'avoir l'échographie. L'échographie peut nous donner un âge gestationnel. Mais bien sûr, on sait que plus on s'éloigne du début de la grossesse, plus la marge d'erreur est grande par rapport à l'échographie. Donc se sont les deux, la hauteur utérine et puis l'échographie. [00:05:51-1](http://localhost:2300/file=C:/Users/USER/Desktop/ENTRETIENS%20PREMATURITE/CMA-DO/GENYCOLOGUE%2001.mp3time=351100) |
| --- |

| 00:05:05-1 Maintenant, au cas où la femme enceinte ne se souvient pas du tout, comment est-ce que vous procédez pour établir l'âge de la grossesse ? 00:05:13-1  00:05:13-1 Person 1: Si elle ne se souvient pas du tout, ici on a deux options. Soit on se base sur la hauteur utérine, parce qu'il y a une corrélation entre la hauteur utérine et l'âge de la grossesse. Voilà. Et ça, ça vous permet d'avoir l'âge de la grossesse à peu près en mois. Ce n'est pas précis à chose près, mais ça vous donne une idée. L'autre chose aussi, on a l'avantage d'avoir l'échographie. L'échographie peut nous donner un âge gestationnel. Mais bien sûr, on sait que plus on s'éloigne du début de la grossesse, plus la marge d'erreur est grande par rapport à l'échographie. Donc ce sont les deux, la hauteur utérine et puis l'échographie. 00:05:51-1 |
| --- |

| **[00:05:05-1] Interviewer**: In cases where the pregnant woman doesn't remember her last period, how do you establish the age of the pregnancy?  **[00:05:13-1] Person 1**: If she can't recall at all, we use two main methods. One option is to base it on the fundal height. There's a correlation between the fundal height and the gestational age, which gives us an approximate age in months. It's not exact but offers a general idea. The other method is using ultrasound, which can provide us with a gestational age. However, it's important to note that the further along in the pregnancy, the larger the margin of error with ultrasound measurements. So, we rely on both fundal height and ultrasound to determine the gestational age. |
| --- |

### Gynecologist 2

| [00:04:51-0](http://localhost:2300/file=C:/Users/USER/Desktop/ENTRETIENS%20PREMATURITE/CMA-DO/GENYCOLOGUE%2002.mp3time=291000) Interviewer: Maintenant, au cas où elles ne se rappellent pas, comment est-ce que vous procédez pour déterminer l'âge de la grossesse? [00:04:55-6](http://localhost:2300/file=C:/Users/USER/Desktop/ENTRETIENS%20PREMATURITE/CMA-DO/GENYCOLOGUE%2002.mp3time=295600)  [00:04:55-6](http://localhost:2300/file=C:/Users/USER/Desktop/ENTRETIENS%20PREMATURITE/CMA-DO/GENYCOLOGUE%2002.mp3time=295600) Person 1: Si une femme se présente et qu'elle ne connait pas la date de ses dernières règles, ça ne facilite pas le calcul. On va se baser sur la mesure de la hauteur utérieure. Ça dépend du niveaux où on se situe ou sur l'échographie. Si on a un échographe à notre disposition, on fait l'estimation et ça permet de calculer le terme de la grossesse. Mais si on n'a pas d'échographie, on va se pencher sur la hauteur utérine. [00:05:28-1](http://localhost:2300/file=C:/Users/USER/Desktop/ENTRETIENS%20PREMATURITE/CMA-DO/GENYCOLOGUE%2002.mp3time=328100) |
| --- |

| 00:04:51-0 Interviewer: Maintenant, au cas où elles ne se rappellent pas, comment est-ce que vous procédez pour déterminer l'âge de la grossesse ? 00:04:55-6  00:04:55-6 Person 1: Si une femme se présente et qu'elle ne connaît pas la date de ses dernières règles, ça ne facilite pas le calcul. On va se baser sur la mesure de la hauteur utérine. Ça dépend du niveau où on se situe ou sur l'échographie. Si on a un échographe à notre disposition, on fait l'estimation et ça permet de calculer le terme de la grossesse. Mais si on n'a pas d'échographie, on va se pencher sur la hauteur utérine. 00:05:28-1 |
| --- |

| **[00:04:51-0] Interviewer**: In cases where they don't remember, how do you determine the age of the pregnancy?  **[00:04:55-6] Person 1**: When a woman comes in and doesn't know the date of her last period, it complicates the calculation of gestational age. In such situations, we base our estimate on the measurement of the fundal height, which varies depending on the stage of pregnancy. If we have access to an ultrasound machine, we use it to estimate and calculate the term of the pregnancy. However, if an ultrasound isn't available, we rely on assessing the fundal height to determine the gestational age. |
| --- |

### Chief physician of Do health district

| [00:08:19-5](http://localhost:2300/file=C:/Users/USER/Desktop/ENTRETIENS%20PREMATURITE/CMA-DO/MCD-DO.mp3time=499500) Interviewer: Au cas où les femmes ne se souviennent pas du tout, comment est-ce que vous procédez ici pour déterminer l'âge gestationnel? [00:08:26-8](http://localhost:2300/file=C:/Users/USER/Desktop/ENTRETIENS%20PREMATURITE/CMA-DO/MCD-DO.mp3time=506800)  [00:08:26-8](http://localhost:2300/file=C:/Users/USER/Desktop/ENTRETIENS%20PREMATURITE/CMA-DO/MCD-DO.mp3time=506800) Person 1: Si la femme ne se souvient pas de la date des dernières règles, en général, il y a de l'approximation qui est faite par deux méthodes, soit c'est par méthode échographique. Sur la taille du foetus, l'embryon, on peut penser déjà à un âge. Ou bien il y a la hauteur uterine aussi, qui nous permet par des méthodes de calcul de s'approcher de l'âge de la grossesse. Ceci étant dit, c'est opérateur, dépendant et technique dépendante. Quand je prends la hauteur uterine, pour quelqu'un qui a une forte corpulence, on peut prendre de la peau avec la graisse, qui peut majoriser l'âge. Au-delà de cela, ça nous permet un meilleur suivi. Ce qu'on ne peut pas avoir l'échographie partout, qui est encore plus sensible par rapport à la hauteur uterine. Mais ailleurs aussi, ce n'est pas évident que les femmes se rémemorent les dates de leures dernières règles. De façon globale, en milieu urbain, ça va parce que les gens sont plus lettrés.Mais en milieu rural, c'est vraiment la hauteur uterine qui est notre outil d'estimation. [00:10:04-7](http://localhost:2300/file=C:/Users/USER/Desktop/ENTRETIENS%20PREMATURITE/CMA-DO/MCD-DO.mp3time=604700) |
| --- |

| 00:08:19-5 Interviewer: Au cas où les femmes ne se souviennent pas du tout, comment est-ce que vous procédez ici pour déterminer l'âge gestationnel ? 00:08:26-8  00:08:26-8 Person 1: Si la femme ne se souvient pas de la date des dernières règles, en général, il y a de l'approximation qui est faite par deux méthodes, soit c'est par méthode échographique. Sur la taille du fœtus, l'embryon, on peut penser déjà à un âge. Ou bien il y a la hauteur utérine aussi, qui nous permet par des méthodes de calcul de s'approcher de l'âge de la grossesse. Ceci étant dit, c'est opérateur, dépendant et technique dépendante. Quand je prends la hauteur utérine, pour quelqu'un qui a une forte corpulence, on peut prendre de la peau avec la graisse, qui peut majorer l'âge. Au-delà de cela, ça nous permet un meilleur suivi. Ce qu'on ne peut pas avoir l'échographie partout, qui est encore plus sensible par rapport à la hauteur utérine. Mais ailleurs aussi, ce n'est pas évident que les femmes se remémorent les dates de leurs dernières règles. De façon globale, en milieu urbain, ça va parce que les gens sont plus lettrés. Mais en milieu rural, c'est vraiment la hauteur utérine qui est notre outil d'estimation. 00:10:04-7 |
| --- |

| **[00:08:19-5] Interviewer**: In cases where women don't remember the date of their last period at all, how do you determine gestational age here?  **[00:08:26-8] Person 1**: If a woman can't recall the date of her last period, we generally use two methods for approximation. One method is ultrasound, where we estimate the age of the pregnancy based on the size of the fetus or embryo. The other method involves measuring the fundal height, which allows us to approximate the pregnancy age through certain calculation techniques. However, this approach can vary depending on the operator and technical factors. For example, in individuals with a larger build, skin and fat might be included in the measurement, potentially leading to an overestimation of the gestational age. Despite these limitations, fundal height measurement is useful for monitoring. While ultrasound is more sensitive and widely used in urban areas where literacy levels are higher, in rural areas, we primarily rely on fundal height as our estimation tool, as ultrasounds are not always readily available. |
| --- |

### Manager for maternity care unit of Do medical center with surgical branch

| [00:07:19-0](http://localhost:2300/file=C:/Users/USER/Desktop/ENTRETIENS%20PREMATURITE/CMA-DO/RESPONSABLE%20UNITE%20DE%20SOIN%20CMA-DO.mp3time=439000) Interviewer: Au cas où la femme enceinte ne se souvient pas du tout, comment est-ce que vous procédez pour déterminer l'âge gestationnel? [00:07:25-9](http://localhost:2300/file=C:/Users/USER/Desktop/ENTRETIENS%20PREMATURITE/CMA-DO/RESPONSABLE%20UNITE%20DE%20SOIN%20CMA-DO.mp3time=445900)  [00:07:25-9](http://localhost:2300/file=C:/Users/USER/Desktop/ENTRETIENS%20PREMATURITE/CMA-DO/RESPONSABLE%20UNITE%20DE%20SOIN%20CMA-DO.mp3time=445900) Person 1: Voilà. Au cas où elle n'arrive pas à connaître, c'est ça aussi. Bon, à travers l'examen, la mesure de la hauteur utérine, ça peut nous guider un peu vers l'âge. Également, il y a l'échographie aussi, où on est amèné à demander. [00:07:42-5](http://localhost:2300/file=C:/Users/USER/Desktop/ENTRETIENS%20PREMATURITE/CMA-DO/RESPONSABLE%20UNITE%20DE%20SOIN%20CMA-DO.mp3time=462500) |
| --- |

| 00:07:19-0 Interviewer: Au cas où la femme enceinte ne se souvient pas du tout, comment est-ce que vous procédez pour déterminer l'âge gestationnel? 00:07:25-9  00:07:25-9 Personne 1: Voilà. Au cas où elle n'arrive pas à connaître, c'est ça aussi. Bon, à travers l'examen, la mesure de la hauteur utérine, ça peut nous guider un peu vers l'âge. Également, il y a l'échographie aussi, où on est amené à demander. 00:07:42-5 |
| --- |

| **[00:07:19-0] Interviewer**: In cases where the pregnant woman doesn't remember her last period at all, how do you determine the gestational age?  **[00:07:25-9] Person 1**: If she can't recall the date of her last period, we use a couple of methods. First, we perform a physical examination, including measuring the fundal height. This measurement can give us some guidance regarding the gestational age. Additionally, we also rely on ultrasounds. If necessary, we request an ultrasound to help establish a more accurate gestational age. |
| --- |

### Focus group of Colma1 health and social promotion center

| [00:08:17-1](http://localhost:2300/file=C:/Users/USER/Desktop/ENTRETIENS%20PREMATURITE/CSPS-COLMA1/FOCUS%20GROUPE%20COLMA1.mp3time=497100) Interviewer: Maintenant, au cas ou la femme enceinte ne se souvient pas du tout, comment est ce que vous procedez pour determiner l'äge gestationnel? [00:08:25-3](http://localhost:2300/file=C:/Users/USER/Desktop/ENTRETIENS%20PREMATURITE/CSPS-COLMA1/FOCUS%20GROUPE%20COLMA1.mp3time=505300)  [00:08:25-3](http://localhost:2300/file=C:/Users/USER/Desktop/ENTRETIENS%20PREMATURITE/CSPS-COLMA1/FOCUS%20GROUPE%20COLMA1.mp3time=505300) Person 3: Bon, généralement avec la HU quand nous prenons la HU, on essaie d'estimer puique en fonction de la HU on peut donner approximativement une date .C'est ça non ? [00:08:34-2](http://localhost:2300/file=C:/Users/USER/Desktop/ENTRETIENS%20PREMATURITE/CSPS-COLMA1/FOCUS%20GROUPE%20COLMA1.mp3time=514200)  [00:08:34-2](http://localhost:2300/file=C:/Users/USER/Desktop/ENTRETIENS%20PREMATURITE/CSPS-COLMA1/FOCUS%20GROUPE%20COLMA1.mp3time=514200) Person 1: oui [00:08:36-7](http://localhost:2300/file=C:/Users/USER/Desktop/ENTRETIENS%20PREMATURITE/CSPS-COLMA1/FOCUS%20GROUPE%20COLMA1.mp3time=516700)  [00:08:36-7](http://localhost:2300/file=C:/Users/USER/Desktop/ENTRETIENS%20PREMATURITE/CSPS-COLMA1/FOCUS%20GROUPE%20COLMA1.mp3time=516700) Person 3: Voilà donc c'est en fonction de ça. [00:08:39-6](http://localhost:2300/file=C:/Users/USER/Desktop/ENTRETIENS%20PREMATURITE/CSPS-COLMA1/FOCUS%20GROUPE%20COLMA1.mp3time=519600) |
| --- |

| 00:07:19-0 Interviewer: Au cas où la femme enceinte ne se souvient pas du tout, comment est-ce que vous procédez pour déterminer l'âge gestationnel ? 00:07:25-9  00:07:25-9 Person 1: Voilà. Au cas où elle n'arrive pas à connaître, c'est ça aussi. Bon, à travers l'examen, la mesure de la hauteur utérine, ça peut nous guider un peu vers l'âge. Également, il y a l'échographie aussi, où on est amené à demander. 00:07:42-5 |
| --- |

| **[00:08:17-1] Interviewer**: If a pregnant woman doesn't remember her last period at all, how do you determine the gestational age?  **[00:08:25-3] Person 3**: In such cases, we primarily use the measurement of the fundal height (HU). By measuring the HU, we can estimate the gestational age. The height of the uterus provides an approximate date of how far along the pregnancy is.  **[00:08:34-2] Person 1**: Yes.  **[00:08:36-7] Person 3**: So, our estimation largely depends on that measurement. |
| --- |

### Major of Colma1 health and social promotion center

| [00:04:54-7](http://localhost:2300/file=C:/Users/USER/Desktop/ENTRETIENS%20PREMATURITE/CSPS-COLMA1/MAJOR%20CSPS%20COLMA1.MP3time=294700) Interviewer: Maintenant, si la femme enceinte ne se souvient pas du tout, comment est ce que vous procedez pour déterminer l'âge gestationnel ? [00:05:03-0](http://localhost:2300/file=C:/Users/USER/Desktop/ENTRETIENS%20PREMATURITE/CSPS-COLMA1/MAJOR%20CSPS%20COLMA1.MP3time=303000)  [00:05:03-0](http://localhost:2300/file=C:/Users/USER/Desktop/ENTRETIENS%20PREMATURITE/CSPS-COLMA1/MAJOR%20CSPS%20COLMA1.MP3time=303000) Person 1: Euh.. en général,il y a la hauteur utérine sur laquelle les sages femmes se basent. Voilà, il y a également l'échographie aussi, que les sages femmes demandent généralement, voilà, ça permet de déterminer l'âge de la grossesse et de rechercher bien d'autres choses. Mais comme nous sommes dans un milieu et qu'on appelle ça milieu urbain, c'est vrai, l'échographie est accessible. Bon, je veux dire, c'est en termes de géographie, mais souvent le coup fait que souvent certains n'arrivent pas à aller, donc ce l'offrir donc reviennent donc à la CPN suivante, souvent sans avoir fait donc l'échographie. [00:05:39-2](http://localhost:2300/file=C:/Users/USER/Desktop/ENTRETIENS%20PREMATURITE/CSPS-COLMA1/MAJOR%20CSPS%20COLMA1.MP3time=339200) |
| --- |

| 00:04:54-7 Interviewer: Maintenant, si la femme enceinte ne se souvient pas du tout, comment est-ce que vous procédez pour déterminer l'âge gestationnel ? 00:05:03-0  00:05:03-0 Person 1: Euh... en général, il y a la hauteur utérine sur laquelle les sage-femmes se basent. Voilà, il y a également l'échographie aussi, que les sage-femmes demandent généralement, voilà, ça permet de déterminer l'âge de la grossesse et de rechercher bien d'autres choses. Mais comme nous sommes dans un milieu et qu'on appelle ça milieu urbain, c'est vrai, l'échographie est accessible. Bon, je veux dire, c'est en termes de géographie, mais souvent le coût fait que souvent certains n'arrivent pas à aller, donc cela leur coûte cher donc reviennent donc à la CPN suivante, souvent sans avoir fait donc l'échographie. 00:05:39-2 |
| --- |

| **[00:04:54-7] Interviewer**: If a pregnant woman doesn't remember her last period at all, how do you determine the gestational age?  **[00:05:03-0] Person 1**: Generally, midwives use the measurement of the fundal height as a basis for estimating gestational age. Additionally, ultrasounds are often requested, as they not only help determine the pregnancy age but also provide insight into many other aspects. In our urban environment, ultrasounds are geographically accessible. However, the cost can be a barrier for some people. Consequently, they might not undergo the ultrasound and return for the next prenatal consultation (CPN) without having had it, due to the high expense. |
| --- |

### Maternity manager of Colma1 health and social promotion center

| [00:11:04-2](http://localhost:2300/file=C:/Users/USER/Desktop/ENTRETIENS%20PREMATURITE/CSPS-COLMA1/RESPONSABLE%20MATERNITE-COLMA1.mp3time=664200) Interviewer: Maintenant, si la femme ne se souvient pas du tout,Comment est- ce que vous procédez pour déterminer l'âge gestationnel ? [00:11:12-0](http://localhost:2300/file=C:/Users/USER/Desktop/ENTRETIENS%20PREMATURITE/CSPS-COLMA1/RESPONSABLE%20MATERNITE-COLMA1.mp3time=672000)  [00:11:12-0](http://localhost:2300/file=C:/Users/USER/Desktop/ENTRETIENS%20PREMATURITE/CSPS-COLMA1/RESPONSABLE%20MATERNITE-COLMA1.mp3time=672000) Person 1: Si elle ne se souvient pas avec la HU (hauteur uterine), souvent, on arrive à déterminer. Mais pas chez toutes les femmes, parce qu'il y a des femmes, elles sont là... Par exemple, on peut mesurer la HU, tu vas trouver 16 centimètres, plus P (Paroi). Il y a des gens qui mettent comme ça plus Paroi, donc ça serait un peu difficile. Mais c'est l'échographie réellement qui peut déterminer l'âge exacte de la grossesse . Sinon, avec la HU, nous pouvons vraiment connaître à peu près l'âge de la grossesse, mais ce n'est pas... Ce n'est pas avec une précision. Voilà, ce n'est pas avec une précision. [00:11:54-8](http://localhost:2300/file=C:/Users/USER/Desktop/ENTRETIENS%20PREMATURITE/CSPS-COLMA1/RESPONSABLE%20MATERNITE-COLMA1.mp3time=714800) |
| --- |

| 00:11:04-2 Interviewer: Maintenant, si la femme ne se souvient pas du tout, comment est-ce que vous procédez pour déterminer l'âge gestationnel ? 00:11:12-0  00:11:12-0 Person 1: Si elle ne se souvient pas avec la HU (hauteur utérine), souvent, on arrive à déterminer. Mais pas chez toutes les femmes, parce qu'il y a des femmes, elles sont là... Par exemple, on peut mesurer la HU, tu vas trouver 16 centimètres, plus P (Paroi). Il y a des gens qui mettent comme ça plus Paroi, donc ça serait un peu difficile. Mais c'est l'échographie réellement qui peut déterminer l'âge exact de la grossesse. Sinon, avec la HU, nous pouvons vraiment connaître à peu près l'âge de la grossesse, mais ce n'est pas... Ce n'est pas avec une précision. Voilà, ce n'est pas avec une précision. 00:11:54-8 |
| --- |

| **[00:11:04-2] Interviewer**: If a woman doesn't remember the date of her last period at all, how do you go about determining the gestational age?  **[00:11:12-0] Person 1**: When she can't recall, we often use the fundal height (HU) measurement to estimate. However, this method isn't always reliable for every woman. For instance, you might measure a fundal height of 16 centimeters, but there are variables like the abdominal wall thickness that can affect the accuracy. In cases like these, it becomes a bit challenging. Ultrasound is the tool that can truly determine the exact age of the pregnancy. While fundal height can give us a rough idea of gestational age, it lacks precise accuracy. |
| --- |

### Focus group of Farakan health and social promotion center

[00:13:19-6](http://localhost:2300/file=C:/Users/USER/Desktop/ENTRETIENS%20PREMATURITE/CSPS_FARAKAN/FOCUS_GROUPE-CSPS_FARAKAN.MP3time=799600) Interviewer: Maintenant, nous allons continuer si la femme enceinte ne se souvient pas du tout, comment est ce que la sage femme détermine-t- elle l'âge gestationnel ? [00:13:30-9](http://localhost:2300/file=C:/Users/USER/Desktop/ENTRETIENS%20PREMATURITE/CSPS_FARAKAN/FOCUS_GROUPE-CSPS_FARAKAN.MP3time=810900)

[00:13:30-9](http://localhost:2300/file=C:/Users/USER/Desktop/ENTRETIENS%20PREMATURITE/CSPS_FARAKAN/FOCUS_GROUPE-CSPS_FARAKAN.MP3time=810900) Person 7: Souvent on demande une échographie. [00:13:40-3](http://localhost:2300/file=C:/Users/USER/Desktop/ENTRETIENS%20PREMATURITE/CSPS_FARAKAN/FOCUS_GROUPE-CSPS_FARAKAN.MP3time=820300)

[00:13:40-3](http://localhost:2300/file=C:/Users/USER/Desktop/ENTRETIENS%20PREMATURITE/CSPS_FARAKAN/FOCUS_GROUPE-CSPS_FARAKAN.MP3time=820300) Person 3: C'etait la même reponse que je voulais dire, nous demandons une échographie pour pouvoir determiner. [00:13:46-6](http://localhost:2300/file=C:/Users/USER/Desktop/ENTRETIENS%20PREMATURITE/CSPS_FARAKAN/FOCUS_GROUPE-CSPS_FARAKAN.MP3time=826600)

[00:13:46-6](http://localhost:2300/file=C:/Users/USER/Desktop/ENTRETIENS%20PREMATURITE/CSPS_FARAKAN/FOCUS_GROUPE-CSPS_FARAKAN.MP3time=826600) Person 8: Nous leur demandons une échographie et là ils vont bien déterminer cela pour nous. [00:13:55-0](http://localhost:2300/file=C:/Users/USER/Desktop/ENTRETIENS%20PREMATURITE/CSPS_FARAKAN/FOCUS_GROUPE-CSPS_FARAKAN.MP3time=835000)

[00:13:55-0](http://localhost:2300/file=C:/Users/USER/Desktop/ENTRETIENS%20PREMATURITE/CSPS_FARAKAN/FOCUS_GROUPE-CSPS_FARAKAN.MP3time=835000) Person 5: Bon souvent, on demande l'échographie, mais souvent aussi on se base aussi sur la hauteur utérine pour donner l'âge de la grossesse. [00:14:06-6](http://localhost:2300/file=C:/Users/USER/Desktop/ENTRETIENS%20PREMATURITE/CSPS_FARAKAN/FOCUS_GROUPE-CSPS_FARAKAN.MP3time=846600)

[00:14:06-6](http://localhost:2300/file=C:/Users/USER/Desktop/ENTRETIENS%20PREMATURITE/CSPS_FARAKAN/FOCUS_GROUPE-CSPS_FARAKAN.MP3time=846600) Person 3: Notre élément principal, c'est la HU (hauteur uterine). [00:14:13-7](http://localhost:2300/file=C:/Users/USER/Desktop/ENTRETIENS%20PREMATURITE/CSPS_FARAKAN/FOCUS_GROUPE-CSPS_FARAKAN.MP3time=853700)

[00:14:13-7](http://localhost:2300/file=C:/Users/USER/Desktop/ENTRETIENS%20PREMATURITE/CSPS_FARAKAN/FOCUS_GROUPE-CSPS_FARAKAN.MP3time=853700) Person 4: La majorité, ça a été dite, sinon c'est sur la HU surtout la plupart du temps qu'on se base. [00:14:19-3](http://localhost:2300/file=C:/Users/USER/Desktop/ENTRETIENS%20PREMATURITE/CSPS_FARAKAN/FOCUS_GROUPE-CSPS_FARAKAN.MP3time=859300)

[00:14:19-3](http://localhost:2300/file=C:/Users/USER/Desktop/ENTRETIENS%20PREMATURITE/CSPS_FARAKAN/FOCUS_GROUPE-CSPS_FARAKAN.MP3time=859300) Interviewer: Quand vous dites HU, vous faites allusion à quoi ? [00:14:22-5](http://localhost:2300/file=C:/Users/USER/Desktop/ENTRETIENS%20PREMATURITE/CSPS_FARAKAN/FOCUS_GROUPE-CSPS_FARAKAN.MP3time=862500)

[00:14:22-5](http://localhost:2300/file=C:/Users/USER/Desktop/ENTRETIENS%20PREMATURITE/CSPS_FARAKAN/FOCUS_GROUPE-CSPS_FARAKAN.MP3time=862500) Person 2: C'est la hauteur uterine. [00:14:24-8](http://localhost:2300/file=C:/Users/USER/Desktop/ENTRETIENS%20PREMATURITE/CSPS_FARAKAN/FOCUS_GROUPE-CSPS_FARAKAN.MP3time=864800)

00:13:19-6 Interviewer: Maintenant, nous allons continuer si la femme enceinte ne se souvient pas du tout, comment est-ce que la sage-femme détermine-t-elle l'âge gestationnel ? 00:13:30-9

00:13:30-9 Person 7: Souvent on demande une échographie. 00:13:40-3

00:13:40-3 Person 3: C'était la même réponse que je voulais dire, nous demandons une échographie pour pouvoir déterminer. 00:13:46-6

00:13:46-6 Person 8: Nous leur demandons une échographie et là ils vont bien déterminer cela pour nous. 00:13:55-0

00:13:55-0 Person 5: Bon souvent, on demande l'échographie, mais souvent aussi on se base aussi sur la hauteur utérine pour donner l'âge de la grossesse. 00:14:06-6

00:14:06-6 Person 3: Notre élément principal, c'est la HU (hauteur utérine). 00:14:13-7

00:14:13-7 Person 4: La majorité, ça a été dite, sinon c'est sur la HU surtout la plupart du temps qu'on se base. 00:14:19-3

00:14:19-3 Interviewer: Quand vous dites HU, vous faites allusion à quoi ? 00:14:22-5

00:14:22-5 Person 2: C'est la hauteur utérine. 00:14:24-8

**[00:13:19-6] Interviewer**: If a pregnant woman doesn't remember her last period at all, how does the midwife determine the gestational age?

**[00:13:30-9] Person 7**: We often request an ultrasound.

**[00:13:40-3] Person 3**: I agree. Requesting an ultrasound is our usual approach to determine gestational age in such cases.

**[00:13:46-6] Person 8**: We ask them to undergo an ultrasound, which then helps us in determining the age of the pregnancy.

**[00:13:55-0] Person 5**: While we often ask for an ultrasound, we also rely on measuring the fundal height as a way to estimate the pregnancy age.

**[00:14:06-6] Person 3**: Our main tool for estimation is the fundal height (HU).

**[00:14:13-7] Person 4**: The majority of the time, we base our estimates on the fundal height measurement.

**[00:14:19-3] Interviewer**: When you mention HU, what exactly are you referring to?

**[00:14:22-5] Person 2**: HU stands for fundal height.

**[00:14:24-8] Person 2**: This is the measurement of the height of the uterus.

### Major of Farakan health and social promotion center

[00:04:45-5](http://localhost:2300/file=C:/Users/USER/Desktop/ENTRETIENS%20PREMATURITE/CSPS_FARAKAN/MAJOR%20FARAKAN.MP3time=285500) Interviewer: Maintenant , si les les femmes ne se souviennent pas du tout de la date des dernières règles, Comment est-ce que vous vous y prenez pour determiner l'âge gestationnel ? [00:04:53-2](http://localhost:2300/file=C:/Users/USER/Desktop/ENTRETIENS%20PREMATURITE/CSPS_FARAKAN/MAJOR%20FARAKAN.MP3time=293200)

[00:04:53-2](http://localhost:2300/file=C:/Users/USER/Desktop/ENTRETIENS%20PREMATURITE/CSPS_FARAKAN/MAJOR%20FARAKAN.MP3time=293200) Person 1: Généralement, si on arrive à demander l'échographie, ça peut nous situer, ou bien on part à partir de la hauteur utérine. Ça aussi à partir de ça, on peut à peu près savoir le nombre de mois. [00:05:08-5](http://localhost:2300/file=C:/Users/USER/Desktop/ENTRETIENS%20PREMATURITE/CSPS_FARAKAN/MAJOR%20FARAKAN.MP3time=308500)

00:04:45-5 Interviewer: Maintenant , si les femmes ne se souviennent pas du tout de la date des dernières règles, Comment est-ce que vous vous y prenez pour déterminer l'âge gestationnel ? 00:04:53-2

00:04:53-2 Person 1: Généralement, si on arrive à demander l'échographie, ça peut nous situer, ou bien on part à partir de la hauteur utérine. Ça aussi à partir de ça, on peut à peu près savoir le nombre de mois. 00:05:08-5

**[00:04:45-5] Interviewer**: If women can't recall the date of their last period at all, how do you determine gestational age?

**[00:04:53-2] Person 1**: In general, if we're able to request an ultrasound, it can help us determine the gestational age. Alternatively, we start by measuring the fundal height. From this measurement, we can estimate the pregnancy's duration in months, although it's a rough estimate.

### Maternity manager of Farakan health and social promotion center

[00:06:33-3](http://localhost:2300/file=C:/Users/USER/Desktop/ENTRETIENS%20PREMATURITE/CSPS_FARAKAN/RESPONSABLE%20MATERNITE%20FARAKAN.MP3time=393300) Interviewer: Maintenant, au cas ou la femme ne se souvient pas du tout de la date des dernières règles, comment est ce que la sage femme procede pour determiner l'âge gestationnel? [00:06:40-6](http://localhost:2300/file=C:/Users/USER/Desktop/ENTRETIENS%20PREMATURITE/CSPS_FARAKAN/RESPONSABLE%20MATERNITE%20FARAKAN.MP3time=400600)

[00:06:40-6](http://localhost:2300/file=C:/Users/USER/Desktop/ENTRETIENS%20PREMATURITE/CSPS_FARAKAN/RESPONSABLE%20MATERNITE%20FARAKAN.MP3time=400600) Person 1: C'est par rapport à la H U, à la hauteur uterine , et puis maintenant demander aussi une échographie pour voire. [00:06:53-6](http://localhost:2300/file=C:/Users/USER/Desktop/ENTRETIENS%20PREMATURITE/CSPS_FARAKAN/RESPONSABLE%20MATERNITE%20FARAKAN.MP3time=413600)

00:06:33-3 Interviewer: Maintenant, au cas où la femme ne se souvient pas du tout de la date des dernières règles, comment est-ce que la sage-femme procède pour déterminer l'âge gestationnel ? 00:06:40-6

00:06:40-6 Person 1: C'est par rapport à la HU, à la hauteur utérine, et puis maintenant demander aussi une échographie pour voir. 00:06:53-6

**[00:06:33-3] Interviewer**: If a woman doesn't remember the date of her last period at all, how does the midwife determine the gestational age?

**[00:06:40-6] Person 1**: The determination is based on the fundal height (HU). We measure the fundal height as an initial assessment. Additionally, we also request an ultrasound, which provides further clarification and helps us ascertain the gestational age more accurately.

## 3.4 Method of calculating gestational age from fundal height

### Focus group of Accart-ville urban medical center

[00:05:14-5](http://localhost:2300/file=C:/Users/USER/Desktop/ENTRETIENS) Interviewer: comment es ce que l'äge gestationnel est calculé àpartir de la hauteur uterine? [00:05:19-2](http://localhost:2300/file=C:/Users/USER/Desktop/ENTRETIENS)

[00:05:19-2](http://localhost:2300/file=C:/Users/USER/Desktop/ENTRETIENS%20PREMATURITE/CSPS-ACCART-VILLE/FOCUS%20GROUPE%20ACCART-VILLE-BON.mp3time=319200) Person 1: a partir de la hauteur uterine,nous demandons souvent à la femme si l'enfant commence à bouger pour savoir que nous sommes au deuxième trimestre, puisqu'au premier trimestre l'enfant ne bouge pas.l'enfant commence à bouger lorsqu'on rentre au deuxième trimestre c'est à dire les quatre mois en allant. donc nous demandons souvent l'echographie ou bien il arrive que nous mesurons. lorsque nous mesurons nous ajoutons (+3) trois pour avoir approximativement la date ou la semaine d'amenhorré de la grossesse. [00:05:52-6](http://local)

[00:05:52-6](http://localhost:2300/file=C:/Users/USER/Desktop/ENTRETIENS) Interviewer: est ce que vous servez de gestogramme ou un tableau? [00:05:55-4](http://localhost:2300/file=C:/Users/USER/Desktop/ENTRETIENS%20PREMATURITE/CSPS-ACCART-VILLE/FOCUS%20GROUPE%20ACCART-VILLE-BON.mp3time=355400)

[00:05:55-4](http://localhost:2300/file=C:/Users/USER/Desktop/ENTRETIENS%20PREMATURITE/CSPS-ACCART-VILLE/FOCUS%20GROUPE%20ACCART-VILLE-BON.mp3time=355400) Person 1: nous n'avons pas de gestogramme içi, depuis que je suis arrivée je n'ai pas rencontré de gestogramme, les collègues disent que se sont les délégués qui donnaient cet appareil avant mais maintenant nous ne savons pas ce qui se passe. avec le gestogramme s'il arrive que la femme ne connait pa la date de ses dernières règles, nous allons faire comment pour mesurer? nous ne pouvons pas connaitre, tout depend du fait que la femme connaisse la date de ses dernières règles. [00:06:16-8](http://localhost:2300/file=C:/Users/USER/Desktop/ENTRETIENS)

00:05:14-5 Interviewer: Comment est-ce que l'âge gestationnel est calculé à partir de la hauteur utérine ? 00:05:19-2

00:05:19-2 Person 1: À partir de la hauteur utérine, nous demandons souvent à la femme si l'enfant commence à bouger pour savoir que nous sommes au deuxième trimestre, puisqu'au premier trimestre l'enfant ne bouge pas. L'enfant commence à bouger lorsqu'on rentre au deuxième trimestre, c'est-à-dire les quatre mois en allant. Donc, nous demandons souvent l'échographie ou bien il arrive que nous mesurions. Lorsque nous mesurons, nous ajoutons (+3) trois pour avoir approximativement la date ou la semaine d'aménorrhée de la grossesse. 00:05:52-6

00:05:52-6 Interviewer: Est-ce que vous servez de gestogramme ou un tableau ? 00:05:55-4

00:05:55-4 Person 1: Nous n'avons pas de gestogramme ici, depuis que je suis arrivée je n'ai pas rencontré de gestogramme, les collègues disent que ce sont les délégués qui donnaient cet appareil avant mais maintenant nous ne savons pas ce qui se passe. Avec le gestogramme, s'il arrive que la femme ne connaisse pas la date de ses dernières règles, nous allons faire comment pour mesurer ? Nous ne pouvons pas connaître, tout dépend du fait que la femme connaisse la date de ses dernières règles. 00:06:16-8

**Interviewer (00:05:14-5):** How do you calculate gestational age using fundal height?

**Person 1 (00:05:19-2):** To estimate gestational age from fundal height, we first determine if the pregnancy is in the second trimester. This is done by asking the woman if she has started feeling fetal movements, as such movements are not typically present in the first trimester. Fetal movements usually begin in the second trimester, which is after the first four months. We might also use an ultrasound for more accuracy. For a rough estimate, when measuring fundal height, we add three (+3) to the measurement to approximate the week of pregnancy in terms of amenorrhea.

**Interviewer (00:05:52-6):** Do you use a gestogram or any kind of chart for measurements?

**Person 1 (00:05:55-4):** We don’t have a gestogram here. Since I’ve been here, I haven’t seen one. My colleagues mentioned that it used to be provided by the delegates, but now its availability is uncertain. The effectiveness of a gestogram largely depends on the woman knowing the date of her last menstrual period. Without that information, it’s challenging to use the gestogram effectively for measurements.

### Manager of Accart-ville urban medical center

[00:06:29-7](http://localhost:2300/file=C:/Users/USER/Desktop/ENTRETIENS%20PREMATURITE/CSPS-ACCART-VILLE/RESPONSABLE%20CMU%20ACCART-VILLE.MP3time=389700)Interviewer: maintenant par rapport à la hauteur uterine, comment est ce que le calcul de l'âge gestationnel est fait? [00:06:33-3](http://localhost:2300/file=C:/Users/USER/Desktop/ENTRETIENS%20PREMATURITE/CSPS-ACCART-VILLE/RESPONSABLE%20CMU%20ACCART-VILLE.MP3time=393300)

[00:06:33-3](http://localhost:2300/file=C:/Users/USER/Desktop/ENTRETIENS%20PREMATURITE/CSPS-ACCART-VILLE/RESPONSABLE%20CMU%20ACCART-VILLE.MP3time=393300) Person 1: bon il faut dire que j'ai pas ça en tête, alors ça vous sera difficile de répondre quand vous n'avez pas en tête par rapport à la hauteur uterine. Puisque c'est un tableau que je regarde? [00:06:46-9](http://localhost:2300/file=C:/Users/USER/Desktop/ENTRETIENS%20PREMATURITE/CSPS-ACCART-VILLE/RESPONSABLE%20CMU%20ACCART-VILLE.MP3time=406900)

[00:06:46-9](http://localhost:2300/file=C:/Users/USER/Desktop/ENTRETIENS%20PREMATURITE/CSPS-ACCART-VILLE/RESPONSABLE%20CMU%20ACCART-VILLE.MP3time=406900) Interviewer: vous disposez d'un tableau pour regarder et puis faire des évaluations? [00:06:49-6](http://localhost:2300/file=C:/Users/USER/Desktop/ENTRETIENS%20PREMATURITE/CSPS-ACCART-VILLE/RESPONSABLE%20CMU%20ACCART-VILLE.MP3time=409600)

[00:06:49-6](http://localhost:2300/file=C:/Users/USER/Desktop/ENTRETIENS%20PREMATURITE/CSPS-ACCART-VILLE/RESPONSABLE%20CMU%20ACCART-VILLE.MP3time=409600)Person 1: Oui, oui. [00:06:51-9](http://localhost:2300/file=C:/Users/USER/Desktop/ENTRETIENS%20PREMATURITE/CSPS-ACCART-VILLE/RESPONSABLE%20CMU%20ACCART-VILLE.MP3time=411900)

00:06:29-7 Interviewer: Maintenant par rapport à la hauteur utérine, comment est-ce que le calcul de l'âge gestationnel est fait ? 00:06:33-3

00:06:33-3 Person 1: Bon, il faut dire que je n'ai pas ça en tête, alors ça vous sera difficile de répondre quand vous n'avez pas en tête par rapport à la hauteur utérine. Puisque c'est un tableau que je regarde ? 00:06:46-9

00:06:46-9 Interviewer: Vous disposez d'un tableau pour regarder et puis faire des évaluations ? 00:06:49-6

00:06:49-6 Person 1: Oui, oui. 00:06:51-9

**[00:06:29-7] Interviewer**: Now, in relation to fundal height, how is the calculation of gestational age done?

**[00:06:33-3] Person 1**: Honestly, I don’t have the specifics memorized, so it’s challenging to answer without having the fundal height chart on hand. It’s something I usually refer to.

**[00:06:46-9] Interviewer**: Do you use a chart for reference when making these evaluations?

**[00:06:49-6] Person 1**: Yes, that's correct. I use a chart for guidance.

### Maternity manager of Accart-ville urban medical center

[00:05:59-1](http://localhost:2300/file=C:/Users/USER/Desktop/ENTRETIENS%20PREMATURITE/CSPS-ACCART-VILLE/RESPONSABLE%20MATERNITE%20CMU%20ACCART-VILLE.MP3time=359100) Interviewer: maintenant comment est ce que le calcul se fait par rapport à la hauteur uterine? [00:06:06-3](http://localhost:2300/file=C:/Users/USER/Desktop/ENTRETIENS%20PREMATURITE/CSPS-ACCART-VILLE/RESPONSABLE%20MATERNITE%20CMU%20ACCART-VILLE.MP3time=366300)

[00:06:06-3](http://localhost:2300/file=C:/Users/USER/Desktop/ENTRETIENS%20PREMATURITE/CSPS-ACCART-VILLE/RESPONSABLE%20MATERNITE%20CMU%20ACCART-VILLE.MP3time=366300) Person 1: on part sur la base de truc, on prend la hauteur uterine et que on ajoute 4cm jusqu'au 7ème mois, après le 7ème mois on ajoute 3cm, après le 8ème mois on ajoute 2cmet en fonction pour determiner. par exemple la HU à 20 on ajoute 4 ça donne 24 semaines d'amenhoré. [00:06:33-6](http://localhost:2300/file=C:/Users/USER/Desktop/ENTRETIENS%20PREMATURITE/CSPS-ACCART-VILLE/RESPONSABLE%20MATERNITE%20CMU%20ACCART-VILLE.MP3time=393600)

[00:06:33-6](http://localhost:2300/file=C:/Users/USER/Desktop/ENTRETIENS%20PREMATURITE/CSPS-ACCART-VILLE/RESPONSABLE%20MATERNITE%20CMU%20ACCART-VILLE.MP3time=393600) Interviewer: est ce que ce calcul se fait par un gestogramme ou à travers un tableau? [00:06:39-3](http://localhost:2300/file=C:/Users/USER/Desktop/ENTRETIENS%20PREMATURITE/CSPS-ACCART-VILLE/RESPONSABLE%20MATERNITE%20CMU%20ACCART-VILLE.MP3time=399300)

[00:06:39-3](http://localhost:2300/file=C:/Users/USER/Desktop/ENTRETIENS%20PREMATURITE/CSPS-ACCART-VILLE/RESPONSABLE%20MATERNITE%20CMU%20ACCART-VILLE.MP3time=399300) Person 1: donc oui on fait des calculs mais c'est un calcul mental et il arrive souvent qu'on utilise des gestogrammes aussi. il arrive qu'on utilise des gestogrammes. on utilise les gestogrammes quand la femme connait la date de ses dernières règles si elle ne connait pas, le gestogramme que nous avons, on ne peut pas utiliser [00:07:07-2](http://localhost:2300/file=C:/Users/USER/Desktop/ENTRETIENS%20PREMATURITE/CSPS-ACCART-VILLE/RESPONSABLE%20MATERNITE%20CMU%20ACCART-VILLE.MP3time=427200)

[00:07:07-2](http://localhost:2300/file=C:/Users/USER/Desktop/ENTRETIENS%20PREMATURITE/CSPS-ACCART-VILLE/RESPONSABLE%20MATERNITE%20CMU%20ACCART-VILLE.MP3time=427200) Interviewer: est ce que c'est le gestogramme que vous utilisez? [00:07:10-5](http://localhost:2300/file=C:/Users/USER/Desktop/ENTRETIENS%20PREMATURITE/CSPS-ACCART-VILLE/RESPONSABLE%20MATERNITE%20CMU%20ACCART-VILLE.MP3time=430500)

[00:07:10-5](http://localhost:2300/file=C:/Users/USER/Desktop/ENTRETIENS%20PREMATURITE/CSPS-ACCART-VILLE/RESPONSABLE%20MATERNITE%20CMU%20ACCART-VILLE.MP3time=430500) Person 1: on utilise assez la date des dernières règles connues. si c'est pas connue on part sur le plan clinique [00:07:18-3](http://localhost:2300/file=C:/Users/USER/Desktop/ENTRETIENS%20PREMATURITE/CSPS-ACCART-VILLE/RESPONSABLE%20MATERNITE%20CMU%20ACCART-VILLE.MP3time=438300)

00:05:59-1 Interviewer: Maintenant comment est-ce que le calcul se fait par rapport à la hauteur utérine ? 00:06:06-3

00:06:06-3 Person 1: On part sur la base de truc, on prend la hauteur utérine et que on ajoute 4 cm jusqu'au 7ème mois, après le 7ème mois on ajoute 3 cm, après le 8ème mois on ajoute 2 cm et en fonction pour déterminer. Par exemple, la HU à 20, on ajoute 4, ça donne 24 semaines d'aménorrhée. 00:06:33-6

00:06:33-6 Interviewer: Est-ce que ce calcul se fait par un gestogramme ou à travers un tableau ? 00:06:39-3

00:06:39-3 Person 1: Donc oui, on fait des calculs mais c'est un calcul mental et il arrive souvent qu'on utilise des gestogrammes aussi. Il arrive qu'on utilise des gestogrammes. On utilise les gestogrammes quand la femme connaît la date de ses dernières règles, si elle ne connaît pas, le gestogramme que nous avons, on ne peut pas utiliser. 00:07:07-2

00:07:07-2 Interviewer: Est-ce que c'est le gestogramme que vous utilisez ? 00:07:10-5

00:07:10-5 Person 1: On utilise assez la date des dernières règles connues. Si ce n'est pas connu, on part sur le plan clinique. 00:07:18-3

**[00:05:59-1] Interviewer**: How is the calculation of gestational age done in relation to fundal height?

**[00:06:06-3] Person 1**: We base our calculations on fundal height measurements. Specifically, we add 4 cm to the measurement until the 7th month of pregnancy. After the 7th month, we add 3 cm, and after the 8th month, we add 2 cm. For example, a fundal height (HU) of 20 cm, plus 4 cm, gives an estimated 24 weeks of amenorrhea.

**[00:06:33-6] Interviewer**: Do you use a gestogram or a table for this calculation?

**[00:06:39-3] Person 1**: Yes, we do mental calculations, but we also sometimes use gestograms. A gestogram is particularly useful when the woman knows the date of her last period. If she doesn’t, the gestogram we have can't be used effectively.

**[00:07:07-2] Interviewer**: Is this the gestogram you typically use?

**[00:07:10-5] Person 1**: We often rely on the date of the last known period for calculations. If this date is unknown, we start from a clinical perspective.

### Focus group of Do medical center with surgical branch

[00:08:32-1](http://localhost:2300/file=C:/Users/USER/Desktop/ENTRETIENS%20PREMATURITE/CMA-DO/FOCUS%20GROUPE%20CMA-DO.mp3time=512100) Interviewer: Vous avez parlé du calcul par la hauteur uterine. Comment est-ce que le calcul se fait pour determiner l'âge gestationnel par la hauteur uterine? [00:08:46-2](http://localhost:2300/file=C:/Users/USER/Desktop/ENTRETIENS%20PREMATURITE/CMA-DO/FOCUS%20GROUPE%20CMA-DO.mp3time=526200)

[00:08:46-2](http://localhost:2300/file=C:/Users/USER/Desktop/ENTRETIENS%20PREMATURITE/CMA-DO/FOCUS%20GROUPE%20CMA-DO.mp3time=526200) Person 2: À partir du 4ème ou 7ème mois, on ajoute quatre. On ajoute 4 à chaque fois. [00:08:57-5](http://localhost:2300/file=C:/Users/USER/Desktop/ENTRETIENS%20PREMATURITE/CMA-DO/FOCUS%20GROUPE%20CMA-DO.mp3time=537500)

[00:08:57-5](http://localhost:2300/file=C:/Users/USER/Desktop/ENTRETIENS%20PREMATURITE/CMA-DO/FOCUS%20GROUPE%20CMA-DO.mp3time=537500) Interviewer: Est-ce que vous utilisez le gestogramme ou bien c'est à partir d'un tableau? [00:09:02-4](http://localhost:2300/file=C:/Users/USER/Desktop/ENTRETIENS%20PREMATURITE/CMA-DO/FOCUS%20GROUPE%20CMA-DO.mp3time=542400)

[00:09:02-4](http://localhost:2300/file=C:/Users/USER/Desktop/ENTRETIENS%20PREMATURITE/CMA-DO/FOCUS%20GROUPE%20CMA-DO.mp3time=542400) Person 2: Nous avons le gestogramme. [00:09:07-4](http://localhost:2300/file=C:/Users/USER/Desktop/ENTRETIENS%20PREMATURITE/CMA-DO/FOCUS%20GROUPE%20CMA-DO.mp3time=547400)

00:08:32-1 Interviewer: Vous avez parlé du calcul par la hauteur utérine. Comment est-ce que le calcul se fait pour déterminer l'âge gestationnel par la hauteur utérine ? 00:08:46-2

00:08:46-2 Person 2: À partir du 4ème ou 7ème mois, on ajoute quatre. On ajoute 4 à chaque fois. 00:08:57-5

00:08:57-5 Interviewer: Est-ce que vous utilisez le gestogramme ou bien c'est à partir d'un tableau ? 00:09:02-4

00:09:02-4 Person 2: Nous avons le gestogramme. 00:09:07-4

**[00:08:32-1] Interviewer**: You mentioned calculating gestational age by fundal height. How is this calculation performed?

**[00:08:46-2] Person 2**: From the 4th or 7th month onwards, we add four centimeters to the fundal height measurement each time.

**[00:08:57-5] Interviewer**: Do you use a gestogram for this, or is it based on a table?

**[00:09:02-4] Person 2**: We utilize a gestogram for these calculations.

### Gynecologist 1

[00:05:51-1](http://localhost:2300/file=C:/Users/USER/Desktop/ENTRETIENS%20PREMATURITE/CMA-DO/GENYCOLOGUE%2001.mp3time=351100) Interviewer: Maintenant, comment est-ce que l'âge de la grossesse est calculé à partir de la hauteur utérine ? [00:05:55-3](http://localhost:2300/file=C:/Users/USER/Desktop/ENTRETIENS%20PREMATURITE/CMA-DO/GENYCOLOGUE%2001.mp3time=355300)

[00:05:55-3](http://localhost:2300/file=C:/Users/USER/Desktop/ENTRETIENS%20PREMATURITE/CMA-DO/GENYCOLOGUE%2001.mp3time=355300) Person 1: L'âge de la grossesse, généralement, on prend la hauteur utérine et puis on divise par 4. Voilà. [00:06:02-9](http://localhost:2300/file=C:/Users/USER/Desktop/ENTRETIENS%20PREMATURITE/CMA-DO/GENYCOLOGUE%2001.mp3time=362900)

[00:06:02-9](http://localhost:2300/file=C:/Users/USER/Desktop/ENTRETIENS%20PREMATURITE/CMA-DO/GENYCOLOGUE%2001.mp3time=362900) Interviewer: Est-ce que pour cela, vous utilisez des gestogrammes ou des tableaux ? [00:06:07-3](http://localhost:2300/file=C:/Users/USER/Desktop/ENTRETIENS%20PREMATURITE/CMA-DO/GENYCOLOGUE%2001.mp3time=367300)

[00:06:07-3](http://localhost:2300/file=C:/Users/USER/Desktop/ENTRETIENS%20PREMATURITE/CMA-DO/GENYCOLOGUE%2001.mp3time=367300) Person 1: On a des tableaux pour ça. Parce que le gestogramme, en fait, on l'utilise si la femme connaît la date de ses dernières règles.Voilà. Mais si c'est sur la base de la hauteur utérine, il y a des tableaux en fonction de la hauteur utérine qui vous donne l'âge de la grossesse. [00:06:22-8](http://localhost:2300/file=C:/Users/USER/Desktop/ENTRETIENS%20PREMATURITE/CMA-DO/GENYCOLOGUE%2001.mp3time=382800)

00:05:51-1 Interviewer: Maintenant, comment est-ce que l'âge de la grossesse est calculé à partir de la hauteur utérine ? 00:05:55-3

00:05:55-3 Person 1: L'âge de la grossesse, généralement, on prend la hauteur utérine et puis on divise par 4. Voilà. 00:06:02-9

00:06:02-9 Interviewer: Est-ce que pour cela, vous utilisez des gestogrammes ou des tableaux ? 00:06:07-3

00:06:07-3 Person 1: On a des tableaux pour ça. Parce que le gestogramme, en fait, on l'utilise si la femme connaît la date de ses dernières règles. Voilà. Mais si c'est sur la base de la hauteur utérine, il y a des tableaux en fonction de la hauteur utérine qui vous donne l'âge de la grossesse. 00:06:22-8

**[00:05:51-1] Interviewer**: Can you explain how gestational age is calculated from fundal height?

**[00:05:55-3] Person 1**: To determine the pregnancy age, we measure the fundal height and then divide that number by 4.

**[00:06:02-9] Interviewer**: Do you use gestograms or tables for these calculations?

**[00:06:07-3] Person 1**: We rely on tables specifically designed for this purpose. The gestogram is used only when the woman knows the date of her last menstrual period. However, when using fundal height, we refer to tables that correlate this measurement with the gestational age.

### Gynecologist 2

[00:05:28-1](http://localhost:2300/file=C:/Users/USER/Desktop/ENTRETIENS%20PREMATURITE/CMA-DO/GENYCOLOGUE%2002.mp3time=328100) Interviewer: Comment le calcul de l'âge gestationnel se fait à partir de la hauteur utérine? [00:05:33-4](http://localhost:2300/file=C:/Users/USER/Desktop/ENTRETIENS%20PREMATURITE/CMA-DO/GENYCOLOGUE%2002.mp3time=333400)

[00:05:33-4](http://localhost:2300/file=C:/Users/USER/Desktop/ENTRETIENS%20PREMATURITE/CMA-DO/GENYCOLOGUE%2002.mp3time=333400) Person 1: À partir de la hauteur utérine, en fonction de l'évolution de la hauteur utérine.C'est-à-dire que, c'est en termes de centimètres en fait, on mesure la hauteur utérine en centimètres. Par exemple, au premier trimestre, la hauteur utérine va se situer entre l'ombilic et les pubis. Quand on est au premier trimestre, on va voir les femmes et on va dire que la hauteur utérine est de 12 ou 13 centimètres. Si on est autour de 12 ou 15 centimètres, en moyenne, mais géneralement c'est à partir de 16 centimètres ou on peut bien mesurer là, c'est 4 mois à peu près. On va dire que la grossesse est àpeu près 4 mois. Si c'est un peu moins, à 12 centimètres c'est à 3 mois. Maintenant, 20 centimètres, ça fait 5 mois en moyenne. Je n'ai pas tous les données en tête, mais à partir de 20 centimètres, on est autour de 5 mois.

24 centimètres, je pense que ça fait 6 mois, mais à la fin de la grossesse, la hauteur utérine va se limiter entre 34 et 36 centimètres. Donc là, on est au 9e mois. Entre les 8e et les 9e mois. Mais généralement, on est au 9e mois. Parce qu'au dernier mois, ça va diminuer légèrement. [00:07:06-5](http://localhost:2300/file=C:/Users/USER/Desktop/ENTRETIENS%20PREMATURITE/CMA-DO/GENYCOLOGUE%2002.mp3time=426500)

[00:07:06-5](http://localhost:2300/file=C:/Users/USER/Desktop/ENTRETIENS%20PREMATURITE/CMA-DO/GENYCOLOGUE%2002.mp3time=426500) Interviewer: Est-ce que pour ça, vous vous servez d'un gestogramme ou vous avez un tableau ? [00:07:11-0](http://localhost:2300/file=C:/Users/USER/Desktop/ENTRETIENS%20PREMATURITE/CMA-DO/GENYCOLOGUE%2002.mp3time=431000)

[00:07:11-0](http://localhost:2300/file=C:/Users/USER/Desktop/ENTRETIENS%20PREMATURITE/CMA-DO/GENYCOLOGUE%2002.mp3time=431000) Person 1: Les gestogrammes, c'est si on connaît la date des dernières règles. Si on a la date de dernières règles, on utilise le gestogramme, mais ça nous permet de calculer immédiatement l'âge actuel et projeter en même temps le terme de la grossesse. Et si on n'a pas la date des dernières règles, en ce moment, le gestogramme ne nous sert plus à grand-chose. [00:07:32-3](http://localhost:2300/file=C:/Users/USER/Desktop/ENTRETIENS%20PREMATURITE/CMA-DO/GENYCOLOGUE%2002.mp3time=452300)

00:05:28-1 Interviewer: Comment le calcul de l'âge gestationnel se fait à partir de la hauteur utérine ? 00:05:33-4

00:05:33-4 Person 1: À partir de la hauteur utérine, en fonction de l'évolution de la hauteur utérine. C'est-à-dire que, c'est en termes de centimètres en fait, on mesure la hauteur utérine en centimètres. Par exemple, au premier trimestre, la hauteur utérine va se situer entre l'ombilic et les pubis. Quand on est au premier trimestre, on va voir les femmes et on va dire que la hauteur utérine est de 12 ou 13 centimètres. Si on est autour de 12 ou 15 centimètres, en moyenne, mais généralement c'est à partir de 16 centimètres ou on peut bien mesurer là, c'est 4 mois à peu près. On va dire que la grossesse est à peu près 4 mois. Si c'est un peu moins, à 12 centimètres c'est à 3 mois. Maintenant, 20 centimètres, ça fait 5 mois en moyenne. Je n'ai pas toutes les données en tête, mais à partir de 20 centimètres, on est autour de 5 mois. 24 centimètres, je pense que ça fait 6 mois, mais à la fin de la grossesse, la hauteur utérine va se limiter entre 34 et 36 centimètres. Donc là, on est au 9e mois. Entre les 8e et les 9e mois. Mais généralement, on est au 9e mois. Parce qu'au dernier mois, ça va diminuer légèrement. 00:07:06-5

00:07:06-5 Interviewer: Est-ce que pour ça, vous vous servez d'un gestogramme ou vous avez un tableau ? 00:07:11-0

00:07:11-0 Person 1: Les gestogrammes, c'est si on connaît la date des dernières règles. Si on a la date de dernières règles, on utilise le gestogramme, mais ça nous permet de calculer immédiatement l'âge actuel et projeter en même temps le terme de la grossesse. Et si on n'a pas la date des dernières règles, en ce moment, le gestogramme ne nous sert plus à grand-chose. 00:07:32-3

**[00:05:28-1] Interviewer**: How do you calculate gestational age from fundal height?

**[00:05:33-4] Person 1**: We measure the fundal height in centimeters. In the first trimester, it's typically between 12 to 15 centimeters, indicating about 3 to 4 months of pregnancy. Around 20 centimeters correlates with approximately 5 months, and 24 centimeters with around 6 months. Towards the end of pregnancy, the fundal height reaches between 34 and 36 centimeters, indicating the 9th month. The height may decrease slightly in the last month.

**[00:07:06-5] Interviewer**: Do you use a gestogram for these measurements?

**[00:07:11-0] Person 1**: Gestograms are useful when we know the date of the last menstrual period. It helps us calculate the current gestational age and predict the due date. If the date of the last period is unknown, the gestogram isn't as useful.

### Chief physician of Do health district

[00:10:04-7](http://localhost:2300/file=C:/Users/USER/Desktop/ENTRETIENS%20PREMATURITE/CMA-DO/MCD-DO.mp3time=604700) Interviewer: D'accord. Maintenant, comment le calcul se fait par la hauteur uterine? [00:10:09-2](http://localhost:2300/file=C:/Users/USER/Desktop/ENTRETIENS%20PREMATURITE/CMA-DO/MCD-DO.mp3time=609200)

[00:10:09-2](http://localhost:2300/file=C:/Users/USER/Desktop/ENTRETIENS%20PREMATURITE/CMA-DO/MCD-DO.mp3time=609200) Person 1: Comment les calculs se font par la hauteur uterine? [00:10:12-9](http://localhost:2300/file=C:/Users/USER/Desktop/ENTRETIENS%20PREMATURITE/CMA-DO/MCD-DO.mp3time=612900)

[00:10:12-9](http://localhost:2300/file=C:/Users/USER/Desktop/ENTRETIENS%20PREMATURITE/CMA-DO/MCD-DO.mp3time=612900) Interviewer: Oui, oui. [00:10:21-7](http://localhost:2300/file=C:/Users/USER/Desktop/ENTRETIENS%20PREMATURITE/CMA-DO/MCD-DO.mp3time=621700)

[00:10:21-7](http://localhost:2300/file=C:/Users/USER/Desktop/ENTRETIENS%20PREMATURITE/CMA-DO/MCD-DO.mp3time=621700) Person 1: Géneralement, c'est après le premier trimestre,quand on regarde, on prend le metre ruban, on part à partir de la ceinture vers le fond uterin, de la symphyse vers le fond uterin, donc il faut chercher le fond uterin à l'aide du metre ruban, et sur la verticale, maintenant on mesure cette distance. C"est cette distance qui est la hauteur uterine. C'est le même mètre urbain que les couturiers utilisent. Ça veut dire que c'est accessible. Voilà, une fois qu'il est... puisque comme c'est flexible, ça suit la courbure. Voilà, donc c'est deux fois plus intéressant à utiliser. Maintenant, en fonction de la longueur, généralement, c'est à partir du 4ème mois jusqu'à la fin, qu'on peut avoir des mesures. Parce qu'avant, ça n'était pas évident de toucher même le fond uterin en bas de la symphyse pubienne, on va voir ce fond uterine. Donc, le nombre de mois, on recherche comme ça. Donc, la hauteur, si tu divises par 4, ça te fait autour du nombre de mois. [00:11:49-3](http://localhost:2300/file=C:/Users/USER/Desktop/ENTRETIENS%20PREMATURITE/CMA-DO/MCD-DO.mp3time=709300)

00:10:04-7 Interviewer: D'accord. Maintenant, comment le calcul se fait par la hauteur utérine ? 00:10:09-2

00:10:09-2 Person 1: Comment les calculs se font par la hauteur utérine ? 00:10:12-9

00:10:12-9 Interviewer: Oui, oui. 00:10:21-7

00:10:21-7 Person 1: Généralement, c'est après le premier trimestre, quand on regarde, on prend le mètre ruban, on part à partir de la ceinture vers le fond utérin, de la symphyse vers le fond utérin, donc il faut chercher le fond utérin à l'aide du mètre ruban, et sur la verticale, maintenant on mesure cette distance. C'est cette distance qui est la hauteur utérine. C'est le même mètre urbain que les couturiers utilisent. Ça veut dire que c'est accessible. Voilà, une fois qu'il est... puisque comme c'est flexible, ça suit la courbure. Voilà, donc c'est deux fois plus intéressant à utiliser. Maintenant, en fonction de la longueur, généralement, c'est à partir du 4ème mois jusqu'à la fin, qu'on peut avoir des mesures. Parce qu'avant, ça n'était pas évident de toucher même le fond utérin en bas de la symphyse pubienne, on va voir ce fond utérin. Donc, le nombre de mois, on recherche comme ça. Donc, la hauteur, si tu divises par 4, ça te fait autour du nombre de mois. 00:11:49-3

**[00:10:04-7] Interviewer**: How is gestational age calculated from fundal height?

**[00:10:21-7] Person 1**: After the first trimester, we use a tape measure to determine fundal height. We measure from the symphysis pubis to the fundus of the uterus vertically. The tape measure is flexible, which helps in following the curvature of the abdomen. This method is typically used from the fourth month of pregnancy until the end. The height measured in centimeters, when divided by 4, gives an approximate number of months of pregnancy.

**[00:11:54-9] Person 1**: We use a standard tape measure for this purpose. Measuring fundal height is straightforward. After measuring, I divide the height by 4 to estimate the gestational age in months.

### Manager for maternity care unit of Do medical center with surgical branch

[00:07:42-5](http://localhost:2300/file=C:/Users/USER/Desktop/ENTRETIENS%20PREMATURITE/CMA-DO/RESPONSABLE%20UNITE%20DE%20SOIN%20CMA-DO.mp3time=462500) Interviewer: Maintenant, comment vous calculez l'âge gestionnel à partir de la hauteur utérine? [00:07:47-1](http://localhost:2300/file=C:/Users/USER/Desktop/ENTRETIENS%20PREMATURITE/CMA-DO/RESPONSABLE%20UNITE%20DE%20SOIN%20CMA-DO.mp3time=467100)

[00:07:47-1](http://localhost:2300/file=C:/Users/USER/Desktop/ENTRETIENS%20PREMATURITE/CMA-DO/RESPONSABLE%20UNITE%20DE%20SOIN%20CMA-DO.mp3time=467100) Person 1: Euh... à partir de la hauteur utérine... Dès que nous mesurons la hauteur utérine, parce qu'il y a une échelle qui est là. Dès que j'ai une hauteur utérine à, utérus globuleux, je vais aller d'abord par ça, parce qu'on ne mesure pas, la taille, orange, citron, orange, pamplemousse, ainsi de suite. Dès que j'ai cette taille, ça me permet par exemple... taille orange ou autre, ça me permet de me dire... Ah, je dois être en face d'une grossesse qui est environ 12 semaines.

Voilà. Ainsi de suite. Et dès que j'ai une hauteur utérine également qui est à... 16 centimètres, 20 centimètres, j'essaie de faire le lien pour dire... Ah, c'est une grossesse de 16 semaines, ou c'est une grossesse de 20 semaines. Voilà. D'aménorrhée. Voilà. Également, ça va être confirmé avec l'échographie, parce qu'on peut se retrouver avec une grossesse gémellaire, où je vais prendre la hauteur utérine qui va être grande, mais ça ne correspond pas avec l'âge. Voilà. Ou également, en fonction de l'évolution de la grossesse, avec une hauteur utérine qui est grande, mais c'est un foetus qui est gros.C'est ça? Voilà. Mais on essaie approximativement, en tout cas, de faire le lien et de confirmer toujours aussi avec l'échographie. Voilà. [00:09:11-6](http://localhost:2300/file=C:/Users/USER/Desktop/ENTRETIENS%20PREMATURITE/CMA-DO/RESPONSABLE%20UNITE%20DE%20SOIN%20CMA-DO.mp3time=551600)

[00:09:11-6](http://localhost:2300/file=C:/Users/USER/Desktop/ENTRETIENS%20PREMATURITE/CMA-DO/RESPONSABLE%20UNITE%20DE%20SOIN%20CMA-DO.mp3time=551600) Interviewer: Est-ce que vous vous servez d'un gestogramme ou c'est à travers un tableau? [00:09:15-4](http://localhost:2300/file=C:/Users/USER/Desktop/ENTRETIENS%20PREMATURITE/CMA-DO/RESPONSABLE%20UNITE%20DE%20SOIN%20CMA-DO.mp3time=555400)

[00:09:15-4](http://localhost:2300/file=C:/Users/USER/Desktop/ENTRETIENS%20PREMATURITE/CMA-DO/RESPONSABLE%20UNITE%20DE%20SOIN%20CMA-DO.mp3time=555400) Person 1: Il y a un gestogramme. Si on connaît la date de dernière règle, on utilise le gestogramme pour donner la date probable d'accouchement.Ça nous donne même une idée sur la période de conception et tout. Voilà. [00:09:27-1](http://localhost:2300/file=C:/Users/USER/Desktop/ENTRETIENS%20PREMATURITE/CMA-DO/RESPONSABLE%20UNITE%20DE%20SOIN%20CMA-DO.mp3time=567100)

00:07:42-5 Interviewer: Maintenant, comment calculez-vous l'âge gestationnel à partir de la hauteur utérine ? 00:07:47-1

00:07:47-1 Person 1: Euh... À partir de la hauteur utérine... Dès que nous mesurons la hauteur utérine, parce qu'il y a une échelle qui est là. Dès que j'ai une hauteur utérine à utérus globuleux, je vais aller d'abord par ça, parce qu'on ne mesure pas la taille, orange, citron, orange, pamplemousse, ainsi de suite. Dès que j'ai cette taille, ça me permet par exemple... taille orange ou autre, ça me permet de me dire... Ah, je dois être en face d'une grossesse qui est environ 12 semaines.

Voilà. Ainsi de suite. Et dès que j'ai une hauteur utérine également qui est à... 16 centimètres, 20 centimètres, j'essaie de faire le lien pour dire... Ah, c'est une grossesse de 16 semaines, ou c'est une grossesse de 20 semaines d'aménorrhée. Voilà. Également, ça va être confirmé avec l'échographie, parce qu'on peut se retrouver avec une grossesse gémellaire, où je vais prendre la hauteur utérine qui va être grande, mais ça ne correspond pas avec l'âge. Voilà. Ou également, en fonction de l'évolution de la grossesse, avec une hauteur utérine qui est grande, mais c'est un fœtus qui est gros. C'est ça ? Voilà. Mais on essaie approximativement, en tout cas, de faire le lien et de confirmer toujours aussi avec l'échographie. Voilà. 00:09:11-6

00:09:11-6 Interviewer: Est-ce que vous vous servez d'un gestogramme ou c'est à travers un tableau ? 00:09:15-4

00:09:15-4 Person 1: Il y a un gestogramme. Si on connaît la date de dernière règle, on utilise le gestogramme pour donner la date probable d'accouchement. Ça nous donne même une idée sur la période de conception et tout. Voilà. 00:09:27-1

**Interviewer (00:07:42-5):** "Can you explain how gestational age is calculated based on fundal height?"

**Person 1 (00:07:47-1):** "Well, regarding fundal height... When we measure the fundal height, we use a specific scale. For instance, if I observe a globular uterus, I start there. We don't measure it in terms of size like an orange, lemon, or grapefruit. When I determine a certain size, like that of an orange, it suggests to me that I might be dealing with a pregnancy around 12 weeks. Similarly, if I measure a uterine height of 16 or 20 centimeters, I correlate it to a pregnancy of 16 or 20 weeks, respectively. However, we always confirm these assessments with an ultrasound, since factors like twin pregnancies or the fetus's size can affect the uterine height. The ultrasound helps to confirm the pregnancy's progression and gestational age."

**Interviewer (00:09:11-6):** "Do you use a gestogram, or is it based on a chart?"

**Person 1 (00:09:15-4):** "Yes, there's a gestogram. If we know the date of the last menstrual period, we use the gestogram to determine the probable delivery date. It even helps us estimate the conception period."

### Focus group of Colma1 health and social promotion center

[00:08:39-6](http://localhost:2300/file=C:/Users/USER/Desktop/ENTRETIENS%20PREMATURITE/CSPS-COLMA1/FOCUS%20GROUPE%20COLMA1.mp3time=519600) Interviewer: Maintenant comment est ce que le calcul se fait à partir de la hauteur uterine? [00:08:42-4](http://localhost:2300/file=C:/Users/USER/Desktop/ENTRETIENS%20PREMATURITE/CSPS-COLMA1/FOCUS%20GROUPE%20COLMA1.mp3time=522400)

[00:08:42-4](http://localhost:2300/file=C:/Users/USER/Desktop/ENTRETIENS%20PREMATURITE/CSPS-COLMA1/FOCUS%20GROUPE%20COLMA1.mp3time=522400) Person 2: bon la HU, géneralement quand on prend à partir de 4ème mois, on estime que la dame doit atteindre les 16 cm; 5ème mois, 20 cm; 6ème mois, 24; ensuite 7ème mois, 28; Voilà 8ème mois, on a autour de 30.maintenant au 9ème on va aller au 30 à 32 au-delà. C'est comme ça, on estime géneralement. [00:09:18-3](http://localhost:2300/file=C:/Users/USER/Desktop/ENTRETIENS%20PREMATURITE/CSPS-COLMA1/FOCUS%20GROUPE%20COLMA1.mp3time=558300)

00:08:39-6 Interviewer: Maintenant comment est-ce que le calcul se fait à partir de la hauteur utérine ? 00:08:42-4

00:08:42-4 Person 2: Bon la HU, généralement quand on prend à partir du 4ème mois, on estime que la dame doit atteindre les 16 cm; 5ème mois, 20 cm; 6ème mois, 24; ensuite 7ème mois, 28; Voilà 8ème mois, on a autour de 30. Maintenant, au 9ème mois, on va aller au 30 à 32 au-delà. C'est comme ça, on estime généralement. 00:09:18-3

[00:08:39-6] Interviewer: How is the calculation done from fundal height?

[00:08:42-4] Person 2: The fundal height (HU) measurements are estimated as follows: 4th month: 16 cm ; 5th month: 20 cm ; 6th month: 24 cm ; 7th month: 28 cm ; 8th month: Around 30 cm ; 9th month: 30 to 32 cm or more. These measurements are used to estimate gestational age in months based on the fundal height.

### Major of Colma1 health and social promotion center

[00:05:39-2](http://localhost:2300/file=C:/Users/USER/Desktop/ENTRETIENS%20PREMATURITE/CSPS-COLMA1/MAJOR%20CSPS%20COLMA1.MP3time=339200) Interviewer: Maintenant, comment est ce que vous calculez l'âge gestationnel à partir de la hauteur utérine ? [00:05:43-5](http://localhost:2300/file=C:/Users/USER/Desktop/ENTRETIENS%20PREMATURITE/CSPS-COLMA1/MAJOR%20CSPS%20COLMA1.MP3time=343500)

[00:05:43-5](http://localhost:2300/file=C:/Users/USER/Desktop/ENTRETIENS%20PREMATURITE/CSPS-COLMA1/MAJOR%20CSPS%20COLMA1.MP3time=343500) Person 1: À partir de la hauteur utérine? Voilà. Euh. Je sais qu'il y a différentes mesures lorsque par exemple la HU est inférieure, donc à 12 cm on dirait que c'est le premier trimestre, ça ne dépassé par 3 mois. à partir de 16 cm, on dit qu'on a dépassé le premier trimestre. Nous sommes au 4e mois, voilà 20 cm, voilà c'est le 5e mois, 24 cm, 6e mois, 27 ou 28 equivaut au 7e mois.Voilà , c'est un peu comme ça qu'on évalue. [00:06:22-3](http://localhost:2300/file=C:/Users/USER/Desktop/ENTRETIENS%20PREMATURITE/CSPS-COLMA1/MAJOR%20CSPS%20COLMA1.MP3time=382300)

[00:06:22-3](http://localhost:2300/file=C:/Users/USER/Desktop/ENTRETIENS%20PREMATURITE/CSPS-COLMA1/MAJOR%20CSPS%20COLMA1.MP3time=382300) Interviewer: est ce à dire que vous disposez d'un gestogramme ou d'un tableau? [00:06:26-7](http://localhost:2300/file=C:/Users/USER/Desktop/ENTRETIENS%20PREMATURITE/CSPS-COLMA1/MAJOR%20CSPS%20COLMA1.MP3time=386700)

[00:06:26-7](http://localhost:2300/file=C:/Users/USER/Desktop/ENTRETIENS%20PREMATURITE/CSPS-COLMA1/MAJOR%20CSPS%20COLMA1.MP3time=386700) Person 1: Oui, c'est un tableau, c'est un tableau qui est disponible en salle de CPN que les sages-femmes, les accoucheuses suivent. c'est afficher. [00:06:35-4](http://localhost:2300/file=C:/Users/USER/Desktop/ENTRETIENS%20PREMATURITE/CSPS-COLMA1/MAJOR%20CSPS%20COLMA1.MP3time=395400)

00:05:39-2 Interviewer: Maintenant, comment est-ce que vous calculez l'âge gestationnel à partir de la hauteur utérine ?

00:05:43-5 Person 1: À partir de la hauteur utérine ? Voilà. Euh. Je sais qu'il y a différentes mesures lorsque par exemple la HU est inférieure, donc à 12 cm on dirait que c'est le premier trimestre, ça ne dépasse pas 3 mois. À partir de 16 cm, on dit qu'on a dépassé le premier trimestre. Nous sommes au 4e mois, voilà 20 cm, voilà c'est le 5e mois, 24 cm, 6e mois, 27 ou 28 équivaut au 7e mois. Voilà, c'est un peu comme ça qu'on évalue.

00:06:22-3 Interviewer: Est-ce à dire que vous disposez d'un gestogramme ou d'un tableau ?
00:06:26-7 Person 1: Oui, c'est un tableau, c'est un tableau qui est disponible en salle de CPN que les sage-femmes, les accoucheuses suivent. C'est affiché.

[00:05:39-2] Interviewer: How is gestational age calculated from fundal height?

[00:05:43-5] Person 1: Fundal height measurements vary with gestational age. For instance: 12 cm indicates the first trimester (up to 3 months); 16 cm suggests the fourth month; 20 cm corresponds to the fifth month; 24 cm is typical of the sixth month ; 27 or 28 cm indicates the seventh month.

[00:06:22-3] Interviewer: Do you have a gestogram or chart for this?

[00:06:26-7] Person 1: Yes, there's a chart in the CPN (Prenatal Care) room that midwives and birth attendants use as a guide. It's displayed for reference.

### Maternity manager of Colma1 health and social promotion center

[00:11:54-8](http://localhost:2300/file=C:/Users/USER/Desktop/ENTRETIENS%20PREMATURITE/CSPS-COLMA1/RESPONSABLE%20MATERNITE-COLMA1.mp3time=714800) Interviewer: maintenant , comment est- ce que le calcul se fait par la Hauteur Uterine ? [00:11:58-9](http://localhost:2300/file=C:/Users/USER/Desktop/ENTRETIENS%20PREMATURITE/CSPS-COLMA1/RESPONSABLE%20MATERNITE-COLMA1.mp3time=718900)

[00:11:58-9](http://localhost:2300/file=C:/Users/USER/Desktop/ENTRETIENS%20PREMATURITE/CSPS-COLMA1/RESPONSABLE%20MATERNITE-COLMA1.mp3time=718900) Person 1: Par HU, Tout simplement, on prend la H. U, par exemple, elle vient, elle a 16 centimètres. Voilà, c'est 16 centimètres. Puisqu'on a déjà définii. Il y a un tableau qu'on a déjà écrit. Par exemple, H U, 16 centimètres, ça équival à combien de mois ? . Mais à partir de sept mois de grossesse, la règle diminue. Sinon, on multiplie l'âge fois quatre, par exemple, mais lorsqu'on dépasse sept mois, on enlève deux. Quand tu calcules, tu multiplies, tu enlèves moins deux. Si, par exemple, elle te dit c'est janvier, j'ai vu mes règles. Janvier, directement, nous savons c'est quel mois elle doit accoucher. À peu près, c'est comme ça qu'on fait. Mais avec le projet d'Embalo, actuellement, nos femmes bénéficient en tout cas de l' échographie. Avant, c'était des femmes qu'on ciblait, mais actuellement, les femmes qui suivent correctement ont la chance de bénéficier de l’échographie qui nous aide beaucoup à connaître l'âge de la grossesse. [00:13:16-1](http://localhost:2300/file=C:/Users/USER/Desktop/ENTRETIENS%20PREMATURITE/CSPS-COLMA1/RESPONSABLE%20MATERNITE-COLMA1.mp3time=796100)

00:11:54-8 Interviewer: Maintenant, comment est-ce que le calcul se fait par la Hauteur Utérine ?
00:11:58-9 Person 1: Par HU, tout simplement, on prend la H.U., par exemple, elle vient, elle a 16 centimètres. Voilà, c'est 16 centimètres. Puisqu'on a déjà défini. Il y a un tableau qu'on a déjà écrit. Par exemple, H.U., 16 centimètres, ça équivaut à combien de mois ? Mais à partir de sept mois de grossesse, la règle diminue. Sinon, on multiplie l'âge fois quatre, par exemple, mais lorsqu'on dépasse sept mois, on enlève deux. Quand tu calcules, tu multiplies, tu enlèves moins deux. Si, par exemple, elle te dit c'est janvier, j'ai vu mes règles. Janvier, directement, nous savons c'est quel mois elle doit accoucher. À peu près, c'est comme ça qu'on fait. Mais avec le projet DenBalo, actuellement, nos femmes bénéficient en tout cas de l'échographie. Avant, c'était des femmes qu'on ciblait, mais actuellement, les femmes qui suivent correctement ont la chance de bénéficier de l’échographie qui nous aide beaucoup à connaître l'âge de la grossesse.

**[00:11:54-8] Interviewer**: How do you calculate gestational age using Fundal Height?

**[00:11:58-9] Person 1**: The calculation is based on fundal height (HU). For example, if a woman has a fundal height of 16 centimeters, we refer to a pre-established table to equate this to the number of months of pregnancy. Generally, the age is multiplied by four, but after seven months of pregnancy, the calculation changes: we subtract two from the result. If a woman says her last period was in January, we can estimate her due month accordingly. Currently, under the DenBalo project, many women benefit from ultrasound services, which significantly aid in determining pregnancy age, especially compared to when only targeted women had access to this facility.

### Focus group of Farakan health and social promotion center

[00:14:24-8](http://localhost:2300/file=C:/Users/USER/Desktop/ENTRETIENS%20PREMATURITE/CSPS_FARAKAN/FOCUS_GROUPE-CSPS_FARAKAN.MP3time=864800) Interviewer: Comment calculez vous l'âge gestationnel à partir de la hauteur uterine? [00:14:38-0](http://localhost:2300/file=C:/Users/USER/Desktop/ENTRETIENS%20PREMATURITE/CSPS_FARAKAN/FOCUS_GROUPE-CSPS_FARAKAN.MP3time=878000)

[00:14:38-0](http://localhost:2300/file=C:/Users/USER/Desktop/ENTRETIENS%20PREMATURITE/CSPS_FARAKAN/FOCUS_GROUPE-CSPS_FARAKAN.MP3time=878000) Person 4: Selon la HU, par exemple, si on a 12 cm, on sait, on imagine que ça ne dépasse pas les 3 mois. Voilà et si c'est 16 cm, la plupart ça ne dépasse pas 4 mois. On s'imagine selon les HU là comme ça. [00:14:55-4](http://localhost:2300/file=C:/Users/USER/Desktop/ENTRETIENS%20PREMATURITE/CSPS_FARAKAN/FOCUS_GROUPE-CSPS_FARAKAN.MP3time=895400)

[00:14:55-4](http://localhost:2300/file=C:/Users/USER/Desktop/ENTRETIENS%20PREMATURITE/CSPS_FARAKAN/FOCUS_GROUPE-CSPS_FARAKAN.MP3time=895400) Person 3: Au premier trimestre,nous mesurons la HU, nous calculons l'âge de la grossesse en ajoutant 4, 4. Par exemple pour chaque mois, nous ajoutant +4 cm à partir du 2e trimestre, nous faisons +5.Voilà et au 3e trimestre, +4+3. [00:15:21-7](http://localhost:2300/file=C:/Users/USER/Desktop/ENTRETIENS%20PREMATURITE/CSPS_FARAKAN/FOCUS_GROUPE-CSPS_FARAKAN.MP3time=921700)

00:14:24-8 Interviewer: Comment calculez-vous l'âge gestationnel à partir de la hauteur utérine ?

00:14:38-0 Person 4: Selon la HU, par exemple, si on a 12 cm, on sait, on imagine que ça ne dépasse pas les 3 mois. Voilà et si c'est 16 cm, la plupart ça ne dépasse pas 4 mois. On s'imagine selon les HU là comme ça.

00:14:55-4 Person 3: Au premier trimestre, nous mesurons la HU, nous calculons l'âge de la grossesse en ajoutant 4, 4. Par exemple pour chaque mois, nous ajoutons +4 cm à partir du 2e trimestre, nous faisons +5. Voilà et au 3e trimestre, +4+3.

**[00:14:24-8] Interviewer**: How is gestational age calculated from fundal height?

**[00:14:38-0] Person 4**: The calculation is based on fundal height (HU). For example, if the fundal height is 12 cm, it suggests that the pregnancy is not more than 3 months. Similarly, a fundal height of 16 cm usually indicates that the pregnancy is no more than 4 months.

**[00:14:55-4] Person 3**: In the first trimester, we measure the fundal height and calculate the pregnancy age by adding 4 cm for each month. From the second trimester, we add 5 cm per month, and in the third trimester, the increment changes to 4 cm and then 3 cm.

### Major of Farakan health and social promotion center

[00:05:08-5](http://localhost:2300/file=C:/Users/USER/Desktop/ENTRETIENS%20PREMATURITE/CSPS_FARAKAN/MAJOR%20FARAKAN.MP3time=308500) Interviewer: Maintenant,comment est-ce que vous calculez l'âge gestationnel à partir de la hauteur uterine ? [00:05:14-5](http://localhost:2300/file=C:/Users/USER/Desktop/ENTRETIENS%20PREMATURITE/CSPS_FARAKAN/MAJOR%20FARAKAN.MP3time=314500)

[00:05:14-5](http://localhost:2300/file=C:/Users/USER/Desktop/ENTRETIENS%20PREMATURITE/CSPS_FARAKAN/MAJOR%20FARAKAN.MP3time=314500) Person 1: Puisque selon le nombre de centimètres, il y a un certain nombre d'âges qu'on donne.Mais ça aussi les paramètres, là souvent c'est biaisé. Il y a des femmes qui sont grosses, qui ont beaucoup de parois, ça peut fausser. Donc c'est approximative. [00:05:34-1](http://localhost:2300/file=C:/Users/USER/Desktop/ENTRETIENS%20PREMATURITE/CSPS_FARAKAN/MAJOR%20FARAKAN.MP3time=334100)

[00:05:34-1](http://localhost:2300/file=C:/Users/USER/Desktop/ENTRETIENS%20PREMATURITE/CSPS_FARAKAN/MAJOR%20FARAKAN.MP3time=334100) Interviewer: Est ce que vous avez un gestogramme? [00:05:35-9](http://localhost:2300/file=C:/Users/USER/Desktop/ENTRETIENS%20PREMATURITE/CSPS_FARAKAN/MAJOR%20FARAKAN.MP3time=335900)

[00:05:35-9](http://localhost:2300/file=C:/Users/USER/Desktop/ENTRETIENS%20PREMATURITE/CSPS_FARAKAN/MAJOR%20FARAKAN.MP3time=335900) Person 1: Oui il y a des gestogrammes aussi. A partir de ça aussi on peut calculer [00:05:41-9](http://localhost:2300/file=C:/Users/USER/Desktop/ENTRETIENS%20PREMATURITE/CSPS_FARAKAN/MAJOR%20FARAKAN.MP3time=341900)

00:05:08-5 Interviewer: Maintenant, comment est-ce que vous calculez l'âge gestationnel à partir de la hauteur utérine ?

00:05:14-5 Person 1: Puisque selon le nombre de centimètres, il y a un certain nombre d'âges qu'on donne. Mais ça aussi les paramètres, là souvent c'est biaisé. Il y a des femmes qui sont grosses, qui ont beaucoup de parois, ça peut fausser. Donc c'est approximatif.

00:05:34-1 Interviewer: Est-ce que vous avez un gestogramme ?

00:05:35-9 Person 1: Oui, il y a des gestogrammes aussi. À partir de ça aussi on peut calculer.

**[00:05:08-5] Interviewer**: How is gestational age calculated from fundal height?

**[00:05:14-5] Person 1**: Gestational age is estimated based on the number of centimeters of fundal height, with a specific age assigned to each measurement. However, these parameters can be biased. For instance, larger women with more abdominal wall can have distorted measurements, making the estimation approximate.

**[00:05:34-1] Interviewer**: Do you have a gestogram?

**[00:05:35-9] Person 1**: Yes, gestograms are also available. They are used to assist in calculating gestational age.

### Maternity manager of Farakan health and social promotion center

[00:06:53-6](http://localhost:2300/file=C:/Users/USER/Desktop/ENTRETIENS%20PREMATURITE/CSPS_FARAKAN/RESPONSABLE%20MATERNITE%20FARAKAN.MP3time=413600) Interviewer: Comment est-ce que vous calculer l'âge gestationnel à partir de la hauteur uterine ? [00:07:00-4](http://localhost:2300/file=C:/Users/USER/Desktop/ENTRETIENS%20PREMATURITE/CSPS_FARAKAN/RESPONSABLE%20MATERNITE%20FARAKAN.MP3time=420400)

[00:07:00-4](http://localhost:2300/file=C:/Users/USER/Desktop/ENTRETIENS%20PREMATURITE/CSPS_FARAKAN/RESPONSABLE%20MATERNITE%20FARAKAN.MP3time=420400) Person 1: À partir de la hauteur uterine,déjà si par exemple, elle a 12 cm comme ça, on peut lui donner 3 mois.Donc, c'est le premier trimestre. [00:07:11-5](http://localhost:2300/file=C:/Users/USER/Desktop/ENTRETIENS%20PREMATURITE/CSPS_FARAKAN/RESPONSABLE%20MATERNITE%20FARAKAN.MP3time=431500)

[00:07:11-5](http://localhost:2300/file=C:/Users/USER/Desktop/ENTRETIENS%20PREMATURITE/CSPS_FARAKAN/RESPONSABLE%20MATERNITE%20FARAKAN.MP3time=431500) Interviewer: Mais comment est ce que le calcul se fait? comment est ce que vous determinez l'âge gestationnel? [00:07:19-1](http://localhost:2300/file=C:/Users/USER/Desktop/ENTRETIENS%20PREMATURITE/CSPS_FARAKAN/RESPONSABLE%20MATERNITE%20FARAKAN.MP3time=439100)

[00:07:19-1](http://localhost:2300/file=C:/Users/USER/Desktop/ENTRETIENS%20PREMATURITE/CSPS_FARAKAN/RESPONSABLE%20MATERNITE%20FARAKAN.MP3time=439100) Person 1: l'âge gestationnel avec la hauteur uterine? en général, c'est par rapport à la date des dernières règles, les mois, si par exemple on connaissait la la date des dernières règles, avec un gestogramme aussi on peut calculer. Mais maintenant comme nous ne connaissons pas , ça sera approximatif.On dit, voilà 12 cm, ça correspond à tant de mois, 16cm correspond à tant de mois ainsi de suite. [00:07:53-5](http://localhost:2300/file=C:/Users/USER/Desktop/ENTRETIENS%20PREMATURITE/CSPS_FARAKAN/RESPONSABLE%20MATERNITE%20FARAKAN.MP3time=473500)

00:06:53-6 Interviewer: Comment est-ce que vous calculez l'âge gestationnel à partir de la hauteur utérine ?

00:07:00-4 Person 1: À partir de la hauteur utérine, déjà si par exemple, elle a 12 cm comme ça, on peut lui donner 3 mois. Donc, c'est le premier trimestre.

00:07:11-5 Interviewer: Mais comment est-ce que le calcul se fait ? Comment est-ce que vous déterminez l'âge gestationnel ?

00:07:19-1 Person 1: L'âge gestationnel avec la hauteur utérine ? En général, c'est par rapport à la date des dernières règles, les mois. Si par exemple on connaissait la date des dernières règles, avec un gestogramme aussi on peut calculer. Mais maintenant comme nous ne connaissons pas, ça sera approximatif. On dit, voilà 12 cm, ça correspond à tant de mois, 16 cm correspond à tant de mois, ainsi de suite.

**[00:06:53-6] Interviewer**: How is gestational age calculated from fundal height?

**[00:07:00-4] Person 1**: The gestational age is approximated based on fundal height measurements. For instance, a measurement of 12 cm is typically associated with a pregnancy of 3 months, indicating the first trimester.

**[00:07:11-5] Interviewer**: How exactly is this calculation made? How do you determine gestational age?

**[00:07:19-1] Person 1**: The gestational age calculation using fundal height is generally based on the date of the last period. If the date is known, a gestogram can be used for more accurate calculations. Without this date, the calculation is more approximate, with specific centimeter measurements (like 12 cm, 16 cm, etc.) being equated to certain months of pregnancy.

## 3.5 Materials needed for calculating gestational age

### Focus group of Accart-ville urban medical center

[00:06:16-8](http://localhost:2300/file=C:/Users/USER/Desktop/ENTRETIENS%20PREMATURITE/CSPS-ACCART-VILLE/FOCUS%20GROUPE%20ACCART-VILLE-BON.mp3time=376800) Interviewer: est ce que vous avez le materiel necessaire pour effectuer le calcul de l'äge gestationnel à partir de la hauteur uterine? [00:06:20-9](http://localhost:2300/file=C:/Users/USER/Desktop/ENTRETIENS%20PREMATURITE/CSPS-ACCART-VILLE/FOCUS%20GROUPE%20ACCART-VILLE-BON.mp3time=380900)

[00:06:20-9](http://localhost:2300/file=C:/Users/USER/Desktop/ENTRETIENS%20PREMATURITE/CSPS-ACCART-VILLE/FOCUS%20GROUPE%20ACCART-VILLE-BON.mp3time=380900) Person 1: le materiel necessaire? bon nous avons le mettre ruban en tout cas, à part ça c'est l'échographie nous demandons. [00:06:28-8](http://localhost:2300/file=C:/Users/USER/Desktop/ENTRETIENS%20PREMATURITE/CSPS-ACCART-VILLE/FOCUS%20GROUPE%20ACCART-VILLE-BON.mp3time=388800)

00:06:16-8 Interviewer: Est-ce que vous avez le matériel nécessaire pour effectuer le calcul de l'âge gestationnel à partir de la hauteur utérine ?
00:06:20-9 Person 1: Le matériel nécessaire ? Bon, nous avons le mètre ruban en tout cas, à part ça c'est l'échographie nous demandons.

**00:06:16-8] Interviewer**: Do you have the necessary equipment to calculate gestational age from fundal height?

**[00:06:20-9] Person 1**: Necessary equipment? Yes, we do have a tape measure, which we use for fundal height measurement. Apart from that, we rely on ultrasound for more detailed information.

### Manager of Accart-ville urban medical center

[00:06:51-9](http://localhost:2300/file=C:/Users/USER/Desktop/ENTRETIENS%20PREMATURITE/CSPS-ACCART-VILLE/RESPONSABLE%20CMU%20ACCART-VILLE.MP3time=411900)Interviewer: Est ce que vous avez le matériel nécessaire pour effectuer le calcul de l'âge gestationnel au sein de votre centre de santé? [00:07:03-2](http://localhost:2300/file=C:/Users/USER/Desktop/ENTRETIENS%20PREMATURITE/CSPS-ACCART-VILLE/RESPONSABLE%20CMU%20ACCART-VILLE.MP3time=423200)

[00:07:03-2](http://localhost:2300/file=C:/Users/USER/Desktop/ENTRETIENS%20PREMATURITE/CSPS-ACCART-VILLE/RESPONSABLE%20CMU%20ACCART-VILLE.MP3time=423200)Person 1: effectivement ce sont des applications qu'on met dans les téléphones pour pouvoir calculer.personnelement moi je me base sur une application que j'ai dans mon téléphone. [00:07:11-2](http://localhost:2300/file=C:/Users/USER/Desktop/ENTRETIENS%20PREMATURITE/CSPS-ACCART-VILLE/RESPONSABLE%20CMU%20ACCART-VILLE.MP3time=431200)

[00:07:11-2](http://localhost:2300/file=C:/Users/USER/Desktop/ENTRETIENS%20PREMATURITE/CSPS-ACCART-VILLE/RESPONSABLE%20CMU%20ACCART-VILLE.MP3time=431200)Interviewer: C'est que application? [00:07:11-9](http://localhost:2300/file=C:/Users/USER/Desktop/ENTRETIENS%20PREMATURITE/CSPS-ACCART-VILLE/RESPONSABLE%20CMU%20ACCART-VILLE.MP3time=431900)

[00:07:11-9](http://localhost:2300/file=C:/Users/USER/Desktop/ENTRETIENS%20PREMATURITE/CSPS-ACCART-VILLE/RESPONSABLE%20CMU%20ACCART-VILLE.MP3time=431900) Person 1: Cest medicalcud que moi j'utilise le plus. [00:07:16-8](http://localhost:2300/file=C:/Users/USER/Desktop/ENTRETIENS%20PREMATURITE/CSPS-ACCART-VILLE/RESPONSABLE%20CMU%20ACCART-VILLE.MP3time=436800)

00:06:51-9 Interviewer: Est-ce que vous avez le matériel nécessaire pour effectuer le calcul de l'âge gestationnel au sein de votre centre de santé ?
00:07:03-2 Person 1: Effectivement, ce sont des applications qu'on met dans les téléphones pour pouvoir calculer. Personnellement, moi je me base sur une application que j'ai dans mon téléphone.

00:07:11-2 Interviewer: C'est quelle application ?

00:07:11-9 Person 1: C'est Medicalcul que moi j'utilise le plus.

**[00:06:51-9] Interviewer**: Do you have the necessary equipment to calculate gestational age in your health center?

**[00:07:03-2] Person 1**: Yes, we use applications on our phones for this purpose. I personally rely on an app that I have on my phone to calculate gestational age.

**[00:07:11-2] Interviewer**: Which app is that?

**[00:07:11-9] Person 1**: I mostly use an app called Medicalcul.

### Maternity manager of Accart-ville urban medical center

[00:07:18-3](http://localhost:2300/file=C:/Users/USER/Desktop/ENTRETIENS%20PREMATURITE/CSPS-ACCART-VILLE/RESPONSABLE%20MATERNITE%20CMU%20ACCART-VILLE.MP3time=438300) Interviewer: maintenant est ce que vous avez le materiel necessaire pour effectuer le calcul de l'âge gestationnel? [00:07:22-6](http://localhost:2300/file=C:/Users/USER/Desktop/ENTRETIENS%20PREMATURITE/CSPS-ACCART-VILLE/RESPONSABLE%20MATERNITE%20CMU%20ACCART-VILLE.MP3time=442600)

[00:07:22-6](http://localhost:2300/file=C:/Users/USER/Desktop/ENTRETIENS%20PREMATURITE/CSPS-ACCART-VILLE/RESPONSABLE%20MATERNITE%20CMU%20ACCART-VILLE.MP3time=442600) Person 1: comme je l'ai dit là, à part le gestogramme qu'on a, et le mettre ruban, on en dispose pas d'autres moyens. c'est les deux,le gestogramme et le mettre ruban que nous utilisons [00:07:37-9](http://localhost:2300/file=C:/Users/USER/Desktop/ENTRETIENS%20PREMATURITE/CSPS-ACCART-VILLE/RESPONSABLE%20MATERNITE%20CMU%20ACCART-VILLE.MP3time=457900)

00:07:18-3 Interviewer: Maintenant, est-ce que vous avez le matériel nécessaire pour effectuer le calcul de l'âge gestationnel ?
00:07:22-6 Person 1: Comme je l'ai dit là, à part le gestogramme qu'on a, et le mètre ruban, on ne dispose pas d'autres moyens. C'est les deux, le gestogramme et le mètre ruban que nous utilisons.

**[00:07:18-3] Interviewer**: Do you have the necessary equipment to calculate the gestational age?

**[00:07:22-6] Person 1**: As I mentioned earlier, our primary tools for calculating gestational age are the gestogram and the tape measure. These are the only means we have at our disposal. We rely on both the gestogram and the tape measure for this purpose.

### Focus group of Do medical center with surgical branch

[00:09:07-4](http://localhost:2300/file=C:/Users/USER/Desktop/ENTRETIENS%20PREMATURITE/CMA-DO/FOCUS%20GROUPE%20CMA-DO.mp3time=547400) Interviewer: Est c Est-ce que vous avez le matériel nécessaire pour le calcul de l'âge de gestationnel? [00:09:19-3](http://localhost:2300/file=C:/Users/USER/Desktop/ENTRETIENS%20PREMATURITE/CMA-DO/FOCUS%20GROUPE%20CMA-DO.mp3time=559300)

[00:09:19-3](http://localhost:2300/file=C:/Users/USER/Desktop/ENTRETIENS%20PREMATURITE/CMA-DO/FOCUS%20GROUPE%20CMA-DO.mp3time=559300) Person 6: Ah, puisqu' il y a une échographie ici.C'est le matériel nécessaire. [00:09:26-0](http://localhost:2300/file=C:/Users/USER/Desktop/ENTRETIENS%20PREMATURITE/CMA-DO/FOCUS%20GROUPE%20CMA-DO.mp3time=566000)

[00:09:26-0](http://localhost:2300/file=C:/Users/USER/Desktop/ENTRETIENS%20PREMATURITE/CMA-DO/FOCUS%20GROUPE%20CMA-DO.mp3time=566000) Person 4: Ça ne fonctionne pas pour nous.Ce ne sont pas les sages femmes ou maîeuticiens qui utilsent ça, ce n'est pas pour nous. Tout ce qu'on est entrain de dire, ce n'est pas systématique. Par exemple, si on va utiliser l'échographie, ce n'est pas systématique.C'est face à une situation qu'on amène chez le gynecologue qui va déterminer l'âge de gestationnel. Mais les femmes que nous croisons de façon courante là. C'est avec les différentes méthodes qu'on vient de citer là. Si elles connaissent la date des dernières règles, si elles ne connaissent la date des dernières règles on peut lier à un evènement. Et là, c'est approximatif.Parce qu'elle peut te dire que j'ai vu les règles une semaine après avoir fêté la Tabaski par exemple. Là, on peut se référer à la date de la Tabaski , donc en ce moment. Et là aussi, on est pas toujours sûr qu'on l'aura. [00:10:25-9](http://localhost:2300/file=C:/Users/USER/Desktop/ENTRETIENS%20PREMATURITE/CMA-DO/FOCUS%20GROUPE%20CMA-DO.mp3time=625900)

00:09:07-4 Interviewer: Est-ce que vous avez le matériel nécessaire pour le calcul de l'âge gestationnel ?

00:09:19-3 Person 6: Ah, puisqu'il y a une échographie ici. C'est le matériel nécessaire.

00:09:26-0 Person 4: Ça ne fonctionne pas pour nous. Ce ne sont pas les sage-femmes ou maïeuticiens qui utilisent ça, ce n'est pas pour nous. Tout ce qu'on est en train de dire, ce n'est pas systématique. Par exemple, si on va utiliser l'échographie, ce n'est pas systématique. C'est face à une situation qu'on amène chez le gynécologue qui va déterminer l'âge gestationnel. Mais les femmes que nous croisons de façon courante là, c'est avec les différentes méthodes qu'on vient de citer là. Si elles connaissent la date des dernières règles, si elles ne connaissent pas la date des dernières règles, on peut lier à un événement. Et là, c'est approximatif. Parce qu'elle peut te dire que j'ai vu les règles une semaine après avoir fêté la Tabaski par exemple. Là, on peut se référer à la date de la Tabaski, donc en ce moment. Et là aussi, on n'est pas toujours sûr qu'on l'aura.

**[00:09:07-4] Interviewer**: Do you have the necessary equipment for calculating gestational age?

**[00:09:19-3] Person 6**: Yes, we have an ultrasound machine here, which is the necessary equipment for that purpose.

**[00:09:26-0] Person 4**: However, it's not commonly used by us, the midwives. We don't use ultrasound systematically. It's typically employed in specific situations where we need to consult with a gynecologist to determine the gestational age. In our regular encounters with women, we use different methods. If they know the date of their last period, we use that. If not, we try to link it to an event, like Tabaski, for an approximate date. But even then, we're not always certain of the accuracy.

### Gynecologist 1

[00:06:22-8](http://localhost:2300/file=C:/Users/USER/Desktop/ENTRETIENS%20PREMATURITE/CMA-DO/GENYCOLOGUE%2001.mp3time=382800) Interviewer: Est-ce qu'au sein de votre service de santé, vous avez le matériel nécessaire pour effectuer le calcul de l'âge gestationnel ? [00:06:30-4](http://localhost:2300/file=C:/Users/USER/Desktop/ENTRETIENS%20PREMATURITE/CMA-DO/GENYCOLOGUE%2001.mp3time=390400)

[00:06:30-4](http://localhost:2300/file=C:/Users/USER/Desktop/ENTRETIENS%20PREMATURITE/CMA-DO/GENYCOLOGUE%2001.mp3time=390400) Person 1: Oui, le matériel nécessaire. [00:06:33-6](http://localhost:2300/file=C:/Users/USER/Desktop/ENTRETIENS%20PREMATURITE/CMA-DO/GENYCOLOGUE%2001.mp3time=393600)

[00:06:33-6](http://localhost:2300/file=C:/Users/USER/Desktop/ENTRETIENS%20PREMATURITE/CMA-DO/GENYCOLOGUE%2001.mp3time=393600) Interviewer: Quel matériel est-ce que vous utilisez pour travaillez ? [00:06:34-8](http://localhost:2300/file=C:/Users/USER/Desktop/ENTRETIENS%20PREMATURITE/CMA-DO/GENYCOLOGUE%2001.mp3time=394800)

[00:06:34-8](http://localhost:2300/file=C:/Users/USER/Desktop/ENTRETIENS%20PREMATURITE/CMA-DO/GENYCOLOGUE%2001.mp3time=394800) Person 1: Bon, puisqu'il n'y a pas besoin de grand-chose à voir, on a un échographe qui peut nous donner l'âge gestationnel. Également, comme je vous ai dit, on a la table en fonction de la hauteur utérine qu'il nous donne.Mais ça, bon, le risque d'erreur est plus grand parce qu'il suffit qu'il n'y ait pas suffisamment de liquide amniotique ou bien il suffit qu'il y ait un problème chez l'enfant qui fait qu'il est plus petit que la normale. Ça peut biaiser les choses. Mais généralement, on arrive à s'en sortir. [00:07:05-1](http://localhost:2300/file=C:/Users/USER/Desktop/ENTRETIENS%20PREMATURITE/CMA-DO/GENYCOLOGUE%2001.mp3time=425100)

00:06:22-8 Interviewer: Est-ce qu'au sein de votre service de santé, vous avez le matériel nécessaire pour effectuer le calcul de l'âge gestationnel ?
00:06:30-4 Person 1: Oui, le matériel nécessaire.
00:06:33-6 Interviewer: Quel matériel est-ce que vous utilisez pour travailler ?
00:06:34-8 Person 1: Bon, puisqu'il n'y a pas besoin de grand-chose à voir, on a un échographe qui peut nous donner l'âge gestationnel. Également, comme je vous ai dit, on a la table en fonction de la hauteur utérine qu'il nous donne. Mais ça, bon, le risque d'erreur est plus grand parce qu'il suffit qu'il n'y ait pas suffisamment de liquide amniotique ou bien il suffit qu'il y ait un problème chez l'enfant qui fait qu'il est plus petit que la normale. Ça peut biaiser les choses. Mais généralement, on arrive à s'en sortir.

**Interviewer [00:06:30-4]**: Do you have the necessary equipment in your health service to calculate gestational age?

**Person 1 [00:06:30-4]**: Yes, we have the necessary materials.

**Interviewer [00:06:33-6]**: What equipment do you use in your work?

**Person 1 [00:06:34-8]**: We don't need much for this purpose. We have an ultrasound machine that can provide us with the gestational age. Additionally, as I mentioned earlier, we use a table based on uterine height for estimation. However, there's a higher risk of error with this method. Factors like insufficient amniotic fluid or developmental issues in the fetus that result in smaller size can lead to inaccuracies. But generally, we manage with the tools we have.

### Gynecologist 2

[00:07:32-3](http://localhost:2300/file=C:/Users/USER/Desktop/ENTRETIENS%20PREMATURITE/CMA-DO/GENYCOLOGUE%2002.mp3time=452300) Interviewer: Est-ce que vous avez le matériel nécessaire pour effectuer le calcul de l'âge gestationnel ? [00:07:38-0](http://localhost:2300/file=C:/Users/USER/Desktop/ENTRETIENS%20PREMATURITE/CMA-DO/GENYCOLOGUE%2002.mp3time=458000)

[00:07:38-0](http://localhost:2300/file=C:/Users/USER/Desktop/ENTRETIENS%20PREMATURITE/CMA-DO/GENYCOLOGUE%2002.mp3time=458000) Person 1: Oui, on a juste besoin d'un mètre ruban pour calculer la hauteur utérine. Mesurer la hauteur utérine et à partir maintenant de la hauteur utérine, on fait le calcul dont j'ai parlé tout à l'heure, pour voir à peu près, ça fait combien de mois. Maintenant, il y a des tableaux aussi. Voilà pourquoi je disais que je n'avais pas tous les éléments en tête. Il y a un tableau, des éléments qu'on n'a pas forcément besoin de retenir. Il y a un tableau qui convertit, c'est-à-dire qui fait la corrélation entre la hauteur utérine et l'âge gestationnel. Donc on peut se référer directement à ce tableau. [00:08:14-4](http://localhost:2300/file=C:/Users/USER/Desktop/ENTRETIENS%20PREMATURITE/CMA-DO/GENYCOLOGUE%2002.mp3time=494400)

00:07:32-3 Interviewer: Est-ce que vous avez le matériel nécessaire pour effectuer le calcul de l'âge gestationnel ?

00:07:38-0 Person 1: Oui, on a juste besoin d'un mètre ruban pour calculer la hauteur utérine. Mesurer la hauteur utérine et à partir de là, on fait le calcul dont j'ai parlé tout à l'heure, pour voir à peu près, ça fait combien de mois. Maintenant, il y a des tableaux aussi. Voilà pourquoi je disais que je n'avais pas tous les éléments en tête. Il y a un tableau, des éléments qu'on n'a pas forcément besoin de retenir. Il y a un tableau qui convertit, c'est-à-dire qui fait la corrélation entre la hauteur utérine et l'âge gestationnel. Donc on peut se référer directement à ce tableau.

**Interviewer [00:07:32-3]**: Do you have the necessary equipment to calculate gestational age?

**Person 1 [00:07:38-0]**: Yes, the primary tool we need is a tape measure for calculating the fundal height. We measure the fundal height and then use it to estimate roughly how many months the pregnancy has progressed. Additionally, we have access to tables that correlate fundal height with gestational age. These tables allow us to convert the fundal height measurement directly into an estimated gestational age, which is quite helpful as we don’t need to remember all the conversion details.

### Chief physician of Do health district

[00:11:49-3](http://localhost:2300/file=C:/Users/USER/Desktop/ENTRETIENS%20PREMATURITE/CMA-DO/MCD-DO.mp3time=709300) Interviewer: Est-ce qu'au sein de votre centre de santé, vous avez le matériel nécessaire pour effectuer le calcul de l'âge gestationnel? [00:11:54-9](http://localhost:2300/file=C:/Users/USER/Desktop/ENTRETIENS%20PREMATURITE/CMA-DO/MCD-DO.mp3time=714900)

[00:11:54-9](http://localhost:2300/file=C:/Users/USER/Desktop/ENTRETIENS%20PREMATURITE/CMA-DO/MCD-DO.mp3time=714900) Person 1: Le matériel, là, c'est le mètre urbain. C'est ce que je disais. Pour la hauteur uterine, c'est pas compliqué. C'est le mètre ruban. Une fois que je mesure directement, je sais le diviser et puis je vois à combien ça me fait autour. [00:12:09-3](http://localhost:2300/file=C:/Users/USER/Desktop/ENTRETIENS%20PREMATURITE/CMA-DO/MCD-DO.mp3time=729300)

00:11:49-3 Interviewer: Est-ce qu'au sein de votre centre de santé, vous avez le matériel nécessaire pour effectuer le calcul de l'âge gestationnel ? 00:11:54-9

00:11:54-9 Person 1: Le matériel, là, c'est le mètre ruban. C'est ce que je disais. Pour la hauteur utérine, ce n'est pas compliqué. C'est le mètre ruban. Une fois que je mesure directement, je sais le diviser et puis je vois à combien ça me fait autour. 00:12:09-3

**Interviewer [00:11:49-3]:** Do you have all the necessary equipment at your health center for calculating gestational age?

**[00:11:54-9] Person 1**: The tool we use here is a tailor’s tape measure. For fundal height, it's simple: once I measure, I know how to divide it and then I can estimate the pregnancy duration.

### Manager for maternity care unit of Do medical center with surgical branch

[00:09:27-1](http://localhost:2300/file=C:/Users/USER/Desktop/ENTRETIENS%20PREMATURITE/CMA-DO/RESPONSABLE%20UNITE%20DE%20SOIN%20CMA-DO.mp3time=567100) Interviewer: Est-ce qu'au sein de votre centre de santé, vous avez le matériel nécessaire pour effectuer le calcul de l'âge gestationnel? [00:09:34-6](http://localhost:2300/file=C:/Users/USER/Desktop/ENTRETIENS%20PREMATURITE/CMA-DO/RESPONSABLE%20UNITE%20DE%20SOIN%20CMA-DO.mp3time=574600)

[00:09:34-6](http://localhost:2300/file=C:/Users/USER/Desktop/ENTRETIENS%20PREMATURITE/CMA-DO/RESPONSABLE%20UNITE%20DE%20SOIN%20CMA-DO.mp3time=574600) Person 1: Bon, le gestogramme, oui, il y en a. Voilà, il y a le gestogramme.Pour faire les mensurations aussi, c'est à travers un mètre ruban, pour prendre la hauteur utérine, oui. Maintenant, également, bon, il y a l'échographie aussi qui est là. C'est vrai que ce n'est pas en salle, mais bon, en consultation gynecologique on peut être amené aussi à confirmer. Voilà. [00:09:57-6](http://localhost:2300/file=C:/Users/USER/Desktop/ENTRETIENS%20PREMATURITE/CMA-DO/RESPONSABLE%20UNITE%20DE%20SOIN%20CMA-DO.mp3time=597600)

00:09:27-1 Interviewer: Est-ce qu'au sein de votre centre de santé, vous avez le matériel nécessaire pour effectuer le calcul de l'âge gestationnel ?

00:09:34-6 Person 1: Bon, le gestogramme, oui, il y en a. Voilà, il y a le gestogramme. Pour faire les mensurations aussi, c'est à travers un mètre ruban, pour prendre la hauteur utérine, oui. Maintenant, également, bon, il y a l'échographie aussi qui est là. C'est vrai que ce n'est pas en salle, mais bon, en consultation gynécologique on peut être amené aussi à confirmer. Voilà

**[00:09:27-1] Interviewer**: Do you have the necessary equipment at your health center to calculate gestational age?

**[00:09:34-6] Person 1**: Yes, we have a gestogram available. That's one tool we use. Additionally, we utilize a tape measure to take uterine height measurements. We also have access to ultrasound, although it's not located within the same room. It's typically used in gynecological consultations for confirmation purposes.

### Focus group of Colma1 health and social promotion center

[00:09:18-3](http://localhost:2300/file=C:/Users/USER/Desktop/ENTRETIENS%20PREMATURITE/CSPS-COLMA1/FOCUS%20GROUPE%20COLMA1.mp3time=558300) Interviewer: Est ce que vous avez le materiel necessaire pour effectuer le calcul de l'âge gestationnel? [00:09:24-0](http://localhost:2300/file=C:/Users/USER/Desktop/ENTRETIENS%20PREMATURITE/CSPS-COLMA1/FOCUS%20GROUPE%20COLMA1.mp3time=564000)

[00:09:24-0](http://localhost:2300/file=C:/Users/USER/Desktop/ENTRETIENS%20PREMATURITE/CSPS-COLMA1/FOCUS%20GROUPE%20COLMA1.mp3time=564000) Person 2: on en a pas,on a le metre ruban, mais je ne pense pas qu'on a un gestogramme, mais il faut que la femme puisse donner un mois et c'est en fonction de ce mois que nous pouvons utiliser le gestogramme, mais la plupart du temps, elle ne connaît même pas le mois. [00:09:59-9](http://localhost:2300/file=C:/Users/USER/Desktop/ENTRETIENS%20PREMATURITE/CSPS-COLMA1/FOCUS%20GROUPE%20COLMA1.mp3time=599900)

[00:09:59-9](http://localhost:2300/file=C:/Users/USER/Desktop/ENTRETIENS%20PREMATURITE/CSPS-COLMA1/FOCUS%20GROUPE%20COLMA1.mp3time=599900) Interviewer: En ce moment comment est ce que vous procédez pour dater les grossesses? [00:10:02-5](http://localhost:2300/file=C:/Users/USER/Desktop/ENTRETIENS%20PREMATURITE/CSPS-COLMA1/FOCUS%20GROUPE%20COLMA1.mp3time=602500)

[00:10:02-5](http://localhost:2300/file=C:/Users/USER/Desktop/ENTRETIENS%20PREMATURITE/CSPS-COLMA1/FOCUS%20GROUPE%20COLMA1.mp3time=602500) Person 1: Comme il l'a dit, c'est seulement à travers la HU. [00:10:04-5](http://localhost:2300/file=C:/Users/USER/Desktop/ENTRETIENS%20PREMATURITE/CSPS-COLMA1/FOCUS%20GROUPE%20COLMA1.mp3time=604500)

[00:10:04-5](http://localhost:2300/file=C:/Users/USER/Desktop/ENTRETIENS%20PREMATURITE/CSPS-COLMA1/FOCUS%20GROUPE%20COLMA1.mp3time=604500) Person 3: Géneralement c'est en fonction de la HU. [00:10:09-4](http://localhost:2300/file=C:/Users/USER/Desktop/ENTRETIENS%20PREMATURITE/CSPS-COLMA1/FOCUS%20GROUPE%20COLMA1.mp3time=609400)

00:09:18-3 Interviewer: Est-ce que vous avez le matériel nécessaire pour effectuer le calcul de l'âge gestationnel ?

00:09:24-0 Person 2: On en a pas, on a le mètre ruban, mais je ne pense pas qu'on a un gestogramme, mais il faut que la femme puisse donner un mois et c'est en fonction de ce mois que nous pouvons utiliser le gestogramme, mais la plupart du temps, elle ne connaît même pas le mois.

00:09:59-9 Interviewer: En ce moment comment est-ce que vous procédez pour dater les grossesses ?

00:10:02-5 Person 1: Comme il l'a dit, c'est seulement à travers la HU.

00:10:04-5 Person 3: Généralement c'est en fonction de la HU.

**[00:09:18-3] Interviewer**: Do you have the necessary equipment to calculate gestational age?

**[00:09:24-0] Person 2**: We don't have a gestogram. We use a tape measure, but the effectiveness of this method depends on the woman being able to provide at least a month of her last period. Most of the time, however, she doesn’t even know the month.

**[00:09:59-9] Interviewer**: How do you currently date pregnancies?

**[00:10:02-5] Person 1**: As he mentioned, we rely on measuring the fundal height (HU).

**[00:10:04-5] Person 3**: Yes, generally it depends on the HU measurement.

### Major of Colma1 health and social promotion center

[00:06:35-4](http://localhost:2300/file=C:/Users/USER/Desktop/ENTRETIENS%20PREMATURITE/CSPS-COLMA1/MAJOR%20CSPS%20COLMA1.MP3time=395400) Interviewer: Est-ce que vous avez le matériel nécessaire pour effectuer donc le calcul de l'âge gestationnel? [00:06:40-8](http://localhost:2300/file=C:/Users/USER/Desktop/ENTRETIENS%20PREMATURITE/CSPS-COLMA1/MAJOR%20CSPS%20COLMA1.MP3time=400800)

[00:06:40-8](http://localhost:2300/file=C:/Users/USER/Desktop/ENTRETIENS%20PREMATURITE/CSPS-COLMA1/MAJOR%20CSPS%20COLMA1.MP3time=400800) Person 1: Materiel nécessaire,Bon, en dehors de l'affiche que nous avons fait, comme ce que je viens de décrire ici en dehors de l'affiche, là, euh, gestogramme, on en a pas. Voilà, c'est ça. [00:06:55-1](http://localhost:2300/file=C:/Users/USER/Desktop/ENTRETIENS%20PREMATURITE/CSPS-COLMA1/MAJOR%20CSPS%20COLMA1.MP3time=415100)

00:06:35-4 Interviewer: Est-ce que vous avez le matériel nécessaire pour effectuer donc le calcul de l'âge gestationnel ?

00:06:40-8 Person 1: Matériel nécessaire, bon, en dehors de l'affiche que nous avons fait, comme ce que je viens de décrire ici en dehors de l'affiche, là, euh, gestogramme, on en a pas. Voilà, c'est ça.

**[00:06:35-4] Interviewer**: Do you have the necessary equipment to calculate gestational age?

**[00:06:40-8] Person 1**: Regarding the necessary materials, aside from the poster we've created that outlines the procedure I just described, we don't have a gestogram. That's the extent of our resources for calculating gestational age.

### Maternity manager of Colma1 health and social promotion center

[00:13:16-1](http://localhost:2300/file=C:/Users/USER/Desktop/ENTRETIENS%20PREMATURITE/CSPS-COLMA1/RESPONSABLE%20MATERNITE-COLMA1.mp3time=796100) Interviewer: d'accord, mais maintenant, Est-ce que vous avez le matériel nécessaires pour effectuer le calcul de l'âge gestationnel ? [00:13:21-8](http://localhost:2300/file=C:/Users/USER/Desktop/ENTRETIENS%20PREMATURITE/CSPS-COLMA1/RESPONSABLE%20MATERNITE-COLMA1.mp3time=801800)

[00:13:21-8](http://localhost:2300/file=C:/Users/USER/Desktop/ENTRETIENS%20PREMATURITE/CSPS-COLMA1/RESPONSABLE%20MATERNITE-COLMA1.mp3time=801800) Person 1: Lorsque la date des derniers règles est connue, il y a des calculs que nous avons tous appris à l'école. Par exemple, une femme qui dit que j'ai vu mes règles le 14 août et qu'est- ce que nous faisons ? à 14 nous ajoutons 10 jours et puis au mois d'août,nous ajoutons trois mois et on attend l'année. Et maintenant, on fait le calcul pour donner la date probable d'accouchement et on ajoute qu'elle peut accoucher deux semaines. Comme on a ajouter 10 jours, 10 jours avant ou dix jours après, nous pouvons le dire comme ça, on fait le calcul. Mais lorsque la femme ne connait pas, elle peut dire non. C'est en février, j'ai vu mes règles, on a déjà pré établi. Février, normalement, c'est en novembre, elle va accoucher. Donc, on essaie de regarder. Mais si réellement on a l'opportunité de donner une échographie à cette femme, ou bien si le projet nous aide en tout cas, parce qu'il y a des gens, on donne l'échographie, on peut donner même pour confirmation de la grossesse, ou bien peut- être, on demande l'échographie parce qu'on doute vraiment de la morphologie même de l'enfant. Il y a des femmes qui reviennent sans pouvoir faire l'échographie jusqu'au dernier jour.et souvent, on se retrouve avec des cas bizarres. [00:14:45-9](http://localhost:2300/file=C:/Users/USER/Desktop/ENTRETIENS%20PREMATURITE/CSPS-COLMA1/RESPONSABLE%20MATERNITE-COLMA1.mp3time=885900)

00:13:16-1 Interviewer: D'accord, mais maintenant, est-ce que vous avez le matériel nécessaire pour effectuer le calcul de l'âge gestationnel ?

00:13:21-8 Person 1: Lorsque la date des dernières règles est connue, il y a des calculs que nous avons tous appris à l'école. Par exemple, une femme qui dit que j'ai vu mes règles le 14 août et qu'est-ce que nous faisons ? À 14 nous ajoutons 10 jours et puis au mois d'août, nous ajoutons trois mois et on attend l'année. Et maintenant, on fait le calcul pour donner la date probable d'accouchement et on ajoute qu'elle peut accoucher deux semaines. Comme on a ajouté 10 jours, 10 jours avant ou dix jours après, nous pouvons le dire comme ça, on fait le calcul. Mais lorsque la femme ne connaît pas, elle peut dire non. C'est en février, j'ai vu mes règles, on a déjà pré établi. Février, normalement, c'est en novembre, elle va accoucher. Donc, on essaie de regarder. Mais si réellement on a l'opportunité de donner une échographie à cette femme, ou bien si le projet nous aide en tout cas, parce qu'il y a des gens, on donne l'échographie, on peut donner même pour confirmation de la grossesse, ou bien peut-être, on demande l'échographie parce qu'on doute vraiment de la morphologie même de l'enfant. Il y a des femmes qui reviennent sans pouvoir faire l'échographie jusqu'au dernier jour et souvent, on se retrouve avec des cas bizarres.

**[00:13:16-1] Interviewer**: Okay, but now, do you have the necessary equipment to calculate gestational age?

**[00:13:21-8] Person 1**: When the date of the last menstrual period is known, we use calculation methods we all learned in school. For instance, if a woman says she had her period on August 14th, we add 10 days to the 14th and then add three months to August to estimate the year of delivery. This calculation gives us the probable date of delivery, plus or minus two weeks, considering the 10-day variation. However, when the woman doesn't know the exact date and can only specify the month, like February, we estimate the delivery around November. We try our best to determine the date, but the precision depends on the information provided. If we can, we recommend an ultrasound for more accurate confirmation of pregnancy or to assess the fetus's development. Unfortunately, some women are unable to get an ultrasound until very late in their pregnancy, leading to unexpected cases at times.

### Focus group of Farakan health and social promotion center

[00:15:21-7](http://localhost:2300/file=C:/Users/USER/Desktop/ENTRETIENS%20PREMATURITE/CSPS_FARAKAN/FOCUS_GROUPE-CSPS_FARAKAN.MP3time=921700) Interviewer: Avez-vous le matériel nécessaire pour effectuer le calcul de l'âge gestationnel ? [00:15:42-7](http://localhost:2300/file=C:/Users/USER/Desktop/ENTRETIENS%20PREMATURITE/CSPS_FARAKAN/FOCUS_GROUPE-CSPS_FARAKAN.MP3time=942700)

[00:15:42-7](http://localhost:2300/file=C:/Users/USER/Desktop/ENTRETIENS%20PREMATURITE/CSPS_FARAKAN/FOCUS_GROUPE-CSPS_FARAKAN.MP3time=942700) Person 4: Oui, nous avons un gestogramme, que beaucoup n'utilise pas sinon nous en avons dans nos sacs, et un autre élément, nous nous basons sur la HU surtout comme nous sommes en ville, nous utilisons beaucoup les échographies, quand bien même on ne le repproche, mais en abusons souvent parce qu' au premier trimestre nous en demandons, au deuxieme trimestre, au troisième trimestre [00:16:15-4](http://localhost:2300/file=C:/Users/USER/Desktop/ENTRETIENS%20PREMATURITE/CSPS_FARAKAN/FOCUS_GROUPE-CSPS_FARAKAN.MP3time=975400)

00:15:21-7 Interviewer: Avez-vous le matériel nécessaire pour effectuer le calcul de l'âge gestationnel ?

00:15:42-7 Person 4: Oui, nous avons un gestogramme, que beaucoup n'utilisent pas sinon nous en avons dans nos sacs, et un autre élément, nous nous basons sur la HU surtout comme nous sommes en ville, nous utilisons beaucoup les échographies, quand bien même on ne le reproche, mais en abusons souvent parce qu'au premier trimestre nous en demandons, au deuxième trimestre, au troisième trimestre.

**[00:15:21-7] Interviewer**: Do you have the necessary equipment to calculate gestational age?

**[00:15:42-7] Person 4**: Yes, we have a gestogram, though it's often underused. Many of us carry one in our bags. Additionally, we rely heavily on measuring fundal height (HU), especially in urban settings where ultrasounds are frequently used. We tend to overuse them, requesting them in the first, second, and third trimesters.

### Major of Farakan health and social promotion center

[00:05:41-9](http://localhost:2300/file=C:/Users/USER/Desktop/ENTRETIENS%20PREMATURITE/CSPS_FARAKAN/MAJOR%20FARAKAN.MP3time=341900) Interviewer: Est ce que vous avez le materiel necessaire pour calculer l'âge gestationnel? [00:05:47-3](http://localhost:2300/file=C:/Users/USER/Desktop/ENTRETIENS%20PREMATURITE/CSPS_FARAKAN/MAJOR%20FARAKAN.MP3time=347300)

[00:05:47-3](http://localhost:2300/file=C:/Users/USER/Desktop/ENTRETIENS%20PREMATURITE/CSPS_FARAKAN/MAJOR%20FARAKAN.MP3time=347300) Person 1: C'est le gestogramme [00:05:51-3](http://localhost:2300/file=C:/Users/USER/Desktop/ENTRETIENS%20PREMATURITE/CSPS_FARAKAN/MAJOR%20FARAKAN.MP3time=351300)

00:05:41-9 Interviewer: Est-ce que vous avez le matériel nécessaire pour calculer l'âge gestationnel ?

00:05:47-3 Person 1: C'est le gestogramme.

**[00:05:41-9] Interviewer**: Do you have the necessary equipment to calculate gestational age?

**[00:05:47-3] Person 1**: Yes, we use the gestogram for this purpose.

### Maternity manager of Farakan health and social promotion center

[00:07:53-5](http://localhost:2300/file=C:/Users/USER/Desktop/ENTRETIENS%20PREMATURITE/CSPS_FARAKAN/RESPONSABLE%20MATERNITE%20FARAKAN.MP3time=473500) Interviewer: Avez-vous le matériel nécessaire pour effectuer les calculs de l'âge gestationnel ? [00:07:58-6](http://localhost:2300/file=C:/Users/USER/Desktop/ENTRETIENS%20PREMATURITE/CSPS_FARAKAN/RESPONSABLE%20MATERNITE%20FARAKAN.MP3time=478600)

[00:07:58-6](http://localhost:2300/file=C:/Users/USER/Desktop/ENTRETIENS%20PREMATURITE/CSPS_FARAKAN/RESPONSABLE%20MATERNITE%20FARAKAN.MP3time=478600) Person 1: Le matériel nécessaire.Moi en tout cas dans mon bureau j'ai un gestogramme. Mais maintenant, c'est en fonction de ce qu'elle va me dire. Par exemple, s'elle me dit, voilà, c'est le 12, le 12 décembre qu'elle a vu ses règles, s'elle me dit que c'est le 12, donc je recule de 3 mois et je peux j'ajoute 10 jours [00:08:30-9](http://localhost:2300/file=C:/Users/USER/Desktop/ENTRETIENS%20PREMATURITE/CSPS_FARAKAN/RESPONSABLE%20MATERNITE%20FARAKAN.MP3time=510900)

00:07:53-5 Interviewer: Avez-vous le matériel nécessaire pour effectuer les calculs de l'âge gestationnel ?

00:07:58-6 Person 1: Le matériel nécessaire. Moi en tout cas dans mon bureau j'ai un gestogramme. Mais maintenant, c'est en fonction de ce qu'elle va me dire. Par exemple, si elle me dit, voilà, c'est le 12, le 12 décembre qu'elle a vu ses règles, si elle me dit que c'est le 12, donc je recule de 3 mois et j'ajoute 10 jours.

**[00:07:53-5] Interviewer**: Do you have the necessary equipment to perform gestational age calculations?

**[00:07:58-6] Person 1**: Yes, I have the essential tools. Specifically, I have a gestogram in my office. However, the calculations depend on the information provided by the patient. For instance, if a woman informs me that she last had her period on December 12th, my approach would be to subtract three months from this date and then add ten days to estimate the gestational age.

## 3.6 Priority between different methods of measuring gestational age

### Focus group of Accart-ville urban medical center

[00:06:28-8](http://localhost:2300/file=C:/Users/USER/Desktop/ENTR) Interviewer: maintenant au sein de votre de santé, quelle estimation a la priorité par rapport à toutes ses methodes d'evaluations de l'âge gestationnel? [00:06:37-4](http://localhost:2300/file=C:/Users/USER/Desktop/ENTRETIENS%20PREMATURITE/CSPS-ACCART-VILLE/FOCUS%20GROUPE%20ACCART-VILLE-BON.mp3time=397400)

[00:06:37-4](http://localhost:2300/file=C:/Users/USER/Desktop/ENTRETIENS)Person 1: en terme de priorité c'est la mensuration bien sûr,nous mesurons et on ajoute ce qu'on doit ajouter et approximativement on peut evaluer [00:06:49-8](http://localhost:2300/file=C:/Users/U)

00:06:28-8 Interviewer: Maintenant, au sein de votre centre de santé, quelle estimation a la priorité par rapport à toutes ces méthodes d'évaluations de l'âge gestationnel ? 00:06:37-4

00:06:37-4 Person 1: En termes de priorité, c'est la mensuration, bien sûr. Nous mesurons et on ajoute ce qu'on doit ajouter, et approximativement on peut évaluer. 00:06:49-8

**Interviewer (00:06:28-8):** Within your health center, which method is given priority for assessing gestational age?

**Person 1 (00:06:37-4):** In terms of priority, we focus on physical measurements. We measure the relevant parameters and add any necessary adjustments to approximate the gestational age.

### Manager of Accart-ville urban medical center

[00:07:16-8](http://localhost:2300/file=C:/Users/USER/Desktop/ENTRETIENS%20PREMATURITE/CSPS-ACCART-VILLE/RESPONSABLE%20CMU%20ACCART-VILLE.MP3time=436800) Interviewer: maintenant pour le calcul de l'âge gestationnel quelle estimation a la priorité au sein de votre centre de santé, Lequel des l'estimation à la priorité? [00:07:29-3](http://localhost:2300/file=C:/Users/USER/Desktop/ENTRETIENS%20PREMATURITE/CSPS-ACCART-VILLE/RESPONSABLE%20CMU%20ACCART-VILLE.MP3time=449300)

[00:07:29-3](http://localhost:2300/file=C:/Users/USER/Desktop/ENTRETIENS%20PREMATURITE/CSPS-ACCART-VILLE/RESPONSABLE%20CMU%20ACCART-VILLE.MP3time=449300) Person 1: Généralement, la priorité,c'est l'âge échographique qu'on utilise le plus. comme l'âge chronologique est un peu difficile à trouver.donc on se base plus sur la femme qui a une échographie avec l'âge echographique. [00:07:48-4](http://localhost:2300/file=C:/Users/USER/Desktop/ENTRETIENS%20PREMATURITE/CSPS-ACCART-VILLE/RESPONSABLE%20CMU%20ACCART-VILLE.MP3time=468400)

00:07:16-8 Interviewer: Maintenant, pour le calcul de l'âge gestationnel, quelle estimation a la priorité au sein de votre centre de santé ? Lequel des estimations a la priorité ?

00:07:29-3 Person 1: Généralement, la priorité, c'est l'âge échographique qu'on utilise le plus, comme l'âge chronologique est un peu difficile à trouver. Donc, on se base plus sur la femme qui a une échographie avec l'âge échographique.

**Interviewer (00:07:16-8):** In your health center, which method for calculating gestational age is given priority?

**Person 1 (00:07:29-3):** Generally, we prioritize the age determined by ultrasound, as it's often challenging to ascertain the exact chronological age. So, we rely more on the ultrasound age when a woman has one.

### Maternity manager of Accart-ville urban medical center

[00:07:37-9](http://localhost:2300/file=C:/Users/USER/Desktop/ENTRETIENS%20PREMATURITE/CSPS-ACCART-VILLE/RESPONSABLE%20MATERNITE%20CMU%20ACCART-VILLE.MP3time=457900) Interviewer: maintenant pour le calcul de l'âge gestationnel, quelle est l'estimation qui a la priorité au sein de votre centre de santé? [00:07:42-7](http://localhost:2300/file=C:/Users/USER/Desktop/ENTRETIENS%20PREMATURITE/CSPS-ACCART-VILLE/RESPONSABLE%20MATERNITE%20CMU%20ACCART-VILLE.MP3time=462700)

[00:07:42-7](http://localhost:2300/file=C:/Users/USER/Desktop/ENTRETIENS%20PREMATURITE/CSPS-ACCART-VILLE/RESPONSABLE%20MATERNITE%20CMU%20ACCART-VILLE.MP3time=462700) Person 1: la priorité c'est les DDR la date des dernières règles [00:07:47-2](http://localhost:2300/file=C:/Users/USER/Desktop/ENTRETIENS%20PREMATURITE/CSPS-ACCART-VILLE/RESPONSABLE%20MATERNITE%20CMU%20ACCART-VILLE.MP3time=467200)

[00:07:47-2](http://localhost:2300/file=C:/Users/USER/Desktop/ENTRETIENS%20PREMATURITE/CSPS-ACCART-VILLE/RESPONSABLE%20MATERNITE%20CMU%20ACCART-VILLE.MP3time=467200) Interviewer: la date des dernières règles? [00:07:47-2](http://localhost:2300/file=C:/Users/USER/Desktop/ENTRETIENS%20PREMATURITE/CSPS-ACCART-VILLE/RESPONSABLE%20MATERNITE%20CMU%20ACCART-VILLE.MP3time=467200)

[00:07:47-2](http://localhost:2300/file=C:/Users/USER/Desktop/ENTRETIENS%20PREMATURITE/CSPS-ACCART-VILLE/RESPONSABLE%20MATERNITE%20CMU%20ACCART-VILLE.MP3time=467200) Person 1: oui [00:07:48-3](http://localhost:2300/file=C:/Users/USER/Desktop/ENTRETIENS%20PREMATURITE/CSPS-ACCART-VILLE/RESPONSABLE%20MATERNITE%20CMU%20ACCART-VILLE.MP3time=468300)

[00:07:48-3](http://localhost:2300/file=C:/Users/USER/Desktop/ENTRETIENS%20PREMATURITE/CSPS-ACCART-VILLE/RESPONSABLE%20MATERNITE%20CMU%20ACCART-VILLE.MP3time=468300) Interviewer: pourquoi? [00:07:50-1](http://localhost:2300/file=C:/Users/USER/Desktop/ENTRETIENS%20PREMATURITE/CSPS-ACCART-VILLE/RESPONSABLE%20MATERNITE%20CMU%20ACCART-VILLE.MP3time=470100)

[00:07:50-1](http://localhost:2300/file=C:/Users/USER/Desktop/ENTRETIENS%20PREMATURITE/CSPS-ACCART-VILLE/RESPONSABLE%20MATERNITE%20CMU%20ACCART-VILLE.MP3time=470100) Person 1: parce que c'est l'âge chronologique compte tenu des marges d'erreurs des autres moyens de datation de la grossesse. par exemple avec la HU (hauteur uterine) souvent cela peut être biaisé en fonction de la corpulance de la femme, souvent même pour retrouver l'uterus c'est compliqué si la femme est obèse. il y a également les autres moyens, si l'échographe n'est pas fait de façon précoce avant la quizième semaine c'est difficile de determiner avec exactitude la date des dernières règles. donc c'est l'âge chronologique qui est priorisé à notre niveau. [00:08:33-5](http://localhost:2300/file=C:/Users/USER/Desktop/ENTRETIENS%20PREMATURITE/CSPS-ACCART-VILLE/RESPONSABLE%20MATERNITE%20CMU%20ACCART-VILLE.MP3time=513500)

00:07:37-9 Interviewer: Maintenant, pour le calcul de l'âge gestationnel, quelle est l'estimation qui a la priorité au sein de votre centre de santé ? 00:07:42-7

00:07:42-7 Person 1: La priorité, c'est les DDR, la date des dernières règles. 00:07:47-2

00:07:47-2 Interviewer: La date des dernières règles ? 00:07:47-2

00:07:47-2 Person 1: Oui. 00:07:48-3

00:07:48-3 Interviewer: Pourquoi ? 00:07:50-1

00:07:50-1 Person 1: Parce que c'est l'âge chronologique compte tenu des marges d'erreurs des autres moyens de datation de la grossesse. Par exemple, avec la HU (hauteur utérine), souvent cela peut être biaisé en fonction de la corpulence de la femme. Souvent, même pour retrouver l'utérus, c'est compliqué si la femme est obèse. Il y a également les autres moyens, si l'échographie n'est pas faite de façon précoce avant la quinzième semaine, c'est difficile de déterminer avec exactitude la date des dernières règles. Donc, c'est l'âge chronologique qui est priorisé à notre niveau. 00:08:33-5

**Interviewer (00:07:37-9):** In your health center, which method is prioritized for calculating gestational age?

**Person 1 (00:07:42-7):** We prioritize the date of the last menstrual period (DDR).

**Interviewer (00:07:47-2):** The last period date?

**Person 1 (00:07:47-2):** Yes.

**Interviewer (00:07:48-3):** Why is that?

**Person 1 (00:07:50-1):** We rely on the chronological age, considering the margins of error in other dating methods. For instance, fundal height (HU) measurements can be influenced by the woman's body size. In obese women, even locating the uterus can be challenging. Additionally, if an ultrasound isn't performed early, before the fifteenth week, it becomes difficult to accurately determine the last period date. Therefore, we prioritize the chronological age based on the last menstrual period in our assessments.

### Focus group of Do medical center with surgical branch

[00:10:25-9](http://localhost:2300/file=C:/Users/USER/Desktop/ENTRETIENS%20PREMATURITE/CMA-DO/FOCUS%20GROUPE%20CMA-DO.mp3time=625900) Interviewer: Vous avez parlé tantôt de plusieurs méthodes pour dater une grossesse. Quelle est la méthode qui a la priorité ici chez vous? [00:10:36-1](http://localhost:2300/file=C:/Users/USER/Desktop/ENTRETIENS%20PREMATURITE/CMA-DO/FOCUS%20GROUPE%20CMA-DO.mp3time=636100)

[00:10:36-1](http://localhost:2300/file=C:/Users/USER/Desktop/ENTRETIENS%20PREMATURITE/CMA-DO/FOCUS%20GROUPE%20CMA-DO.mp3time=636100) Person 7: Le gestogramme. [00:10:39-3](http://localhost:2300/file=C:/Users/USER/Desktop/ENTRETIENS%20PREMATURITE/CMA-DO/FOCUS%20GROUPE%20CMA-DO.mp3time=639300)

[00:10:39-3](http://localhost:2300/file=C:/Users/USER/Desktop/ENTRETIENS%20PREMATURITE/CMA-DO/FOCUS%20GROUPE%20CMA-DO.mp3time=639300) Interviewer: Pourquoi? [00:10:42-3](http://localhost:2300/file=C:/Users/USER/Desktop/ENTRETIENS%20PREMATURITE/CMA-DO/FOCUS%20GROUPE%20CMA-DO.mp3time=642300)

[00:10:42-3](http://localhost:2300/file=C:/Users/USER/Desktop/ENTRETIENS%20PREMATURITE/CMA-DO/FOCUS%20GROUPE%20CMA-DO.mp3time=642300) Person 7: Parce que nous l'avons à portée de main. [00:10:48-5](http://localhost:2300/file=C:/Users/USER/Desktop/ENTRETIENS%20PREMATURITE/CMA-DO/FOCUS%20GROUPE%20CMA-DO.mp3time=648500)

00:10:25-9 Interviewer: Vous avez parlé tantôt de plusieurs méthodes pour dater une grossesse. Quelle est la méthode qui a la priorité ici chez vous ? 00:10:36-1

00:10:36-1 Person 7: Le gestogramme. 00:10:39-3

00:10:39-3 Interviewer: Pourquoi ? 00:10:42-3

00:10:42-3 Person 7: Parce que nous l'avons à portée de main. 00:10:48-5

**Interviewer (00:10:25-9):** Earlier, you mentioned several methods for dating a pregnancy. Which one is given priority in your facility?

**Person 7 (00:10:36-1):** The gestogram.

**Interviewer (00:10:39-3):** Why is that?

**Person 7 (00:10:42-3):** Because it's readily accessible to us. We have it at our fingertips.

### Gynecologist 1

[00:07:05-1](http://localhost:2300/file=C:/Users/USER/Desktop/ENTRETIENS%20PREMATURITE/CMA-DO/GENYCOLOGUE%2001.mp3time=425100) Interviewer: Maintenant, au sein de votre service de santé, pour la datation de la grossesse, quelle estimation a la priorité chez vous ? [00:07:12-2](http://localhost:2300/file=C:/Users/USER/Desktop/ENTRETIENS%20PREMATURITE/CMA-DO/GENYCOLOGUE%2001.mp3time=432200)

[00:07:12-2](http://localhost:2300/file=C:/Users/USER/Desktop/ENTRETIENS%20PREMATURITE/CMA-DO/GENYCOLOGUE%2001.mp3time=432200) Person 1: C'est l'échographie. Oui, c'est l'échographie. [00:07:15-7](http://localhost:2300/file=C:/Users/USER/Desktop/ENTRETIENS%20PREMATURITE/CMA-DO/GENYCOLOGUE%2001.mp3time=435700)

[00:07:15-7](http://localhost:2300/file=C:/Users/USER/Desktop/ENTRETIENS%20PREMATURITE/CMA-DO/GENYCOLOGUE%2001.mp3time=435700) Interviewer: Pourquoi ? [00:07:16-6](http://localhost:2300/file=C:/Users/USER/Desktop/ENTRETIENS%20PREMATURITE/CMA-DO/GENYCOLOGUE%2001.mp3time=436600)

[00:07:16-6](http://localhost:2300/file=C:/Users/USER/Desktop/ENTRETIENS%20PREMATURITE/CMA-DO/GENYCOLOGUE%2001.mp3time=436600) Person 1: Parce qu'avec l'échographie, avec les différentes mensurations, on a un âge gestationnel qui est un peu plus précis que la hauteur utérine.Parce que, comme je vous ai dit, la hauteur utérine, ça va dépendre de la quantité de liquide amniotique, ça va dépendre de... s'il n'y a pas de problème chez l'enfant, il y a beaucoup de facteurs qui peuvent influencer. Alors que, bon, avec l'échographie, avec les mensurations, on peut avoir un âge gestationnel avec une marge d'erreur de peut-être une semaine à dix jours. [00:07:48-3](http://localhost:2300/file=C:/Users/USER/Desktop/ENTRETIENS%20PREMATURITE/CMA-DO/GENYCOLOGUE%2001.mp3time=468300)

00:07:05-1 Interviewer: Maintenant, au sein de votre service de santé, pour la datation de la grossesse, quelle estimation a la priorité chez vous ?

00:07:12-2 Person 1: C'est l'échographie. Oui, c'est l'échographie.

00:07:15-7 Interviewer: Pourquoi ?

00:07:16-6 Person 1: Parce qu'avec l'échographie, avec les différentes mensurations, on a un âge gestationnel qui est un peu plus précis que la hauteur utérine. Parce que, comme je vous ai dit, la hauteur utérine, ça va dépendre de la quantité de liquide amniotique, ça va dépendre de... s'il n'y a pas de problème chez l'enfant, il y a beaucoup de facteurs qui peuvent influencer. Alors que, bon, avec l'échographie, avec les mensurations, on peut avoir un âge gestationnel avec une marge d'erreur de peut-être une semaine à dix jours.

**Interviewer (00:07:05-1):** Within your health service, when dating a pregnancy, which estimate do you prioritize?

**Person 1 (00:07:12-2):** It's the ultrasound. Definitely, the ultrasound.

**Interviewer (00:07:15-7):** Why is that?

**Person 1 (00:07:16-6):** The ultrasound offers a more precise gestational age due to its various measurements. Fundal height can be influenced by many factors, like the amount of amniotic fluid or potential issues with the fetus. In contrast, an ultrasound provides a gestational age with a relatively small margin of error, maybe one week to ten days.

### Gynecologist 2

[00:08:14-4](http://localhost:2300/file=C:/Users/USER/Desktop/ENTRETIENS%20PREMATURITE/CMA-DO/GENYCOLOGUE%2002.mp3time=494400) Interviewer: Maintenant, pour le calcul de l'âge gestationnel, quelle est l'estimation qui est la priorité pour déterminer l'âge gestationnel ? [00:08:26-7](http://localhost:2300/file=C:/Users/USER/Desktop/ENTRETIENS%20PREMATURITE/CMA-DO/GENYCOLOGUE%2002.mp3time=506700)

[00:08:26-7](http://localhost:2300/file=C:/Users/USER/Desktop/ENTRETIENS%20PREMATURITE/CMA-DO/GENYCOLOGUE%2002.mp3time=506700) Person 1: C'est-à-dire qui donne plus d'informations valables ? [00:08:29-1](http://localhost:2300/file=C:/Users/USER/Desktop/ENTRETIENS%20PREMATURITE/CMA-DO/GENYCOLOGUE%2002.mp3time=509100)

[00:08:29-1](http://localhost:2300/file=C:/Users/USER/Desktop/ENTRETIENS%20PREMATURITE/CMA-DO/GENYCOLOGUE%2002.mp3time=509100) Interviewer: La méthode que vous utilisez le plus pour dater la grossesse. [00:08:31-8](http://localhost:2300/file=C:/Users/USER/Desktop/ENTRETIENS%20PREMATURITE/CMA-DO/GENYCOLOGUE%2002.mp3time=511800)

[00:08:31-8](http://localhost:2300/file=C:/Users/USER/Desktop/ENTRETIENS%20PREMATURITE/CMA-DO/GENYCOLOGUE%2002.mp3time=511800) Person 1: La méthode qu'on utilise le plus ici, en fait, on fait une combinaison de tout. Parce qu'on utilise la hauteur utérine ? Mais très souvent, comme on est en ville ici, on demande l'échographie. Mais quand une femme se présente au premier trimestre, on n'a pas la date des dernières règles, elle ne se souvient pas de grand chose, on demande l'échographie des datations. Donc ça, c'est la méthode la plus utilisée ici. [00:08:56-1](http://localhost:2300/file=C:/Users/USER/Desktop/ENTRETIENS%20PREMATURITE/CMA-DO/GENYCOLOGUE%2002.mp3time=536100)

00:08:14-4 Interviewer: Maintenant, pour le calcul de l'âge gestationnel, quelle est l'estimation qui est la priorité pour déterminer l'âge gestationnel ?

00:08:26-7 Person 1: C'est-à-dire, qui donne plus d'informations valables ?

00:08:29-1 Interviewer: La méthode que vous utilisez le plus pour dater la grossesse.

00:08:31-8 Person 1: La méthode qu'on utilise le plus ici, en fait, on fait une combinaison de tout. Parce qu'on utilise la hauteur utérine, mais très souvent, comme on est en ville ici, on demande l'échographie. Mais quand une femme se présente au premier trimestre, on n'a pas la date des dernières règles, elle ne se souvient pas de grand-chose, on demande l'échographie de datation. Donc ça, c'est la méthode la plus utilisée ici.

**Interviewer (00:08:14-4):** When calculating gestational age, which method do you prioritize?

**Person 1 (00:08:26-7):** You mean, which method provides the most accurate information?

**Interviewer (00:08:29-1):** Yes, what's your primary method for dating pregnancies?

**Person 1 (00:08:31-8):** In our practice, we typically use a combination of methods. We measure fundal height, but often, especially in urban areas, we recommend an ultrasound. If a woman in her first trimester doesn’t remember the date of her last period, we specifically request a dating ultrasound. That’s our most commonly used approach.

### Chief physician of Do health district

[00:12:09-3](http://localhost:2300/file=C:/Users/USER/Desktop/ENTRETIENS%20PREMATURITE/CMA-DO/MCD-DO.mp3time=729300) Interviewer: D'accord. Maintenant, pour la datation de la grossesse, quelle est la méthode qui a la priorité chez vous ici? [00:12:14-4](http://localhost:2300/file=C:/Users/USER/Desktop/ENTRETIENS%20PREMATURITE/CMA-DO/MCD-DO.mp3time=734400)

[00:12:14-4](http://localhost:2300/file=C:/Users/USER/Desktop/ENTRETIENS%20PREMATURITE/CMA-DO/MCD-DO.mp3time=734400) Person 1: Bon, quand on arrive au CMA, on va dire que c'est toujours... Non, la question, c'est d'abord... Est-ce que tu te souviens de la date dernière? Parce que si tu connais, ça c'est plus simple. On prend le gestogramme, on regarde, ça nous génère ça en semaines d'aménorrhée. C'est vrai que la semaine d'aménorrhée, il peut y avoir une différence. Parce qu'il y a deux semaines qu'on ajoute dans la semaine d'aménorrhée. Parce que la phase de maturation, c'est en gros deux semaines. Donc on ajoute les deux semaines, voilà, sur la période pour calculer. Si on ne connait pas, si tu ne connais pas la date, donc quand on a ça, on a un gestogramme qui, quand tu mets la date de dernière règle et puis la date d'aujourd'hui là, ça te permet de te dire concrètement, tu es à combien de semaines d'aménorrhée directement.Ça c'est un outil gestogramme, on retrouve, c'est usuel. C'est dans les maternités. Maintenant si on ne connait pas la date des dernières règles, on va utiliser notre metre ruban pour la hauteur uterine. En fonction du nombre de centimètres, on essaie de faire le rapprochement pour avoir. Mais si on a encore l'échographie, on peut utiliser l'échographie. Ce qui est le plus utilisé c'est les deux, le gestogramme et la hauteur uterine. Mais si on prend l'ensemble du district, ça va être la hauteur uterine. Parce qu'en milieu rural, on n'a pas d'échographie. [00:13:53-7](http://localhost:2300/file=C:/Users/USER/Desktop/ENTRETIENS%20PREMATURITE/CMA-DO/MCD-DO.mp3time=833700)

00:12:09-3 Interviewer: D'accord. Maintenant, pour la datation de la grossesse, quelle est la méthode qui a la priorité chez vous ici ?

00:12:14-4 Person 1: Bon, quand on arrive au CMA, on va dire que c'est toujours... Non, la question, c'est d'abord... Est-ce que tu te souviens de la date dernière ? Parce que si tu connais, ça c'est plus simple. On prend le gestogramme, on regarde, ça nous génère ça en semaines d'aménorrhée. C'est vrai que la semaine d'aménorrhée, il peut y avoir une différence. Parce qu'il y a deux semaines qu'on ajoute dans la semaine d'aménorrhée. Parce que la phase de maturation, c'est en gros deux semaines. Donc on ajoute les deux semaines, voilà, sur la période pour calculer. Si on ne connait pas, si tu ne connais pas la date, donc quand on a ça, on a un gestogramme qui, quand tu mets la date de dernière règle et puis la date d'aujourd'hui là, ça te permet de te dire concrètement, tu es à combien de semaines d'aménorrhée directement. Ça, c'est un outil gestogramme, on retrouve, c'est usuel. C'est dans les maternités. Maintenant si on ne connait pas la date des dernières règles, on va utiliser notre mètre ruban pour la hauteur utérine. En fonction du nombre de centimètres, on essaie de faire le rapprochement pour avoir. Mais si on a encore l'échographie, on peut utiliser l'échographie. Ce qui est le plus utilisé c'est les deux, le gestogramme et la hauteur utérine. Mais si on prend l'ensemble du district, ça va être la hauteur utérine. Parce qu'en milieu rural, on n'a pas d'échographie.

**Interviewer (00:12:09-3):** For pregnancy dating, which method is prioritized in your facility?

**Person 1 (00:12:14-4):** At the CMA, we first ask if the woman remembers her last menstrual period. Knowing this date simplifies things. We use the gestogram, which calculates the weeks of amenorrhea, adding two weeks for the maturation phase. If the last period date is unknown, we then measure fundal height. The number of centimeters helps us estimate the gestational age. Ultrasounds are also used, but the most common methods here are the gestogram and fundal height measurement. In rural areas, we mainly rely on fundal height due to the lack of ultrasound facilities.

### Manager for maternity care unit of Do medical center with surgical branch

[00:09:57-6](http://localhost:2300/file=C:/Users/USER/Desktop/ENTRETIENS%20PREMATURITE/CMA-DO/RESPONSABLE%20UNITE%20DE%20SOIN%20CMA-DO.mp3time=597600) Interviewer: Maintenant, pour le calcul de l'âge gestationnel, quelle estimation a la priorité ici chez vous? Quelle méthode d'estimation a la priorité ici chez vous? [00:10:08-1](http://localhost:2300/file=C:/Users/USER/Desktop/ENTRETIENS%20PREMATURITE/CMA-DO/RESPONSABLE%20UNITE%20DE%20SOIN%20CMA-DO.mp3time=608100)

[00:10:08-1](http://localhost:2300/file=C:/Users/USER/Desktop/ENTRETIENS%20PREMATURITE/CMA-DO/RESPONSABLE%20UNITE%20DE%20SOIN%20CMA-DO.mp3time=608100) Person 1: La méthode d'estimation, bon, moi, en tout cas, c'est avec le gestogramme.Voilà. Dès que j'ai une idée, parce que je fais tout pour amener la femme à pouvoir me donner la date des dernières règles. Voilà. Moi, c'est à travers le gestogramme que j'arrive à le faire. [00:10:25-8](http://localhost:2300/file=C:/Users/USER/Desktop/ENTRETIENS%20PREMATURITE/CMA-DO/RESPONSABLE%20UNITE%20DE%20SOIN%20CMA-DO.mp3time=625800)

[00:10:25-8](http://localhost:2300/file=C:/Users/USER/Desktop/ENTRETIENS%20PREMATURITE/CMA-DO/RESPONSABLE%20UNITE%20DE%20SOIN%20CMA-DO.mp3time=625800) Interviewer: Mais pourquoi? [00:10:25-5](http://localhost:2300/file=C:/Users/USER/Desktop/ENTRETIENS%20PREMATURITE/CMA-DO/RESPONSABLE%20UNITE%20DE%20SOIN%20CMA-DO.mp3time=625500)

[00:10:25-5](http://localhost:2300/file=C:/Users/USER/Desktop/ENTRETIENS%20PREMATURITE/CMA-DO/RESPONSABLE%20UNITE%20DE%20SOIN%20CMA-DO.mp3time=625500) Bon, comme j'ai déjà la date des dernières règles, en fait, c'est ça.C'est le recueil de cette date-là qui pose problème. Mais comme les gens ne sont pas trop patients pour amener les femmes à pouvoir donner cette période-là, on part rapidement. Dès qu'on demande et s'elle dit qu'elle ne connait pas , on dit inconnus, et on continue. Alors que si tu essaies d'insister un peu, tu vas l'amener à situer cette période-là et à te donner.Voilà. Bon, ici, de façon générale, les gens passent par l'échographie la plupart du temps. [00:10:55-6](http://localhost:2300/file=C:/Users/USER/Desktop/ENTRETIENS%20PREMATURITE/CMA-DO/RESPONSABLE%20UNITE%20DE%20SOIN%20CMA-DO.mp3time=655600)

00:09:57-6 Interviewer: Maintenant, pour le calcul de l'âge gestationnel, quelle estimation a la priorité ici chez vous ? Quelle méthode d'estimation a la priorité ici chez vous ? 00:10:08-1

00:10:08-1 Person 1: La méthode d'estimation, bon, moi, en tout cas, c'est avec le gestogramme. Voilà. Dès que j'ai une idée, parce que je fais tout pour amener la femme à pouvoir me donner la date des dernières règles. Voilà. Moi, c'est à travers le gestogramme que j'arrive à le faire. 00:10:25-8

00:10:25-8 Interviewer: Mais pourquoi ? 00:10:25-5

00:10:25-5 Person 1: Bon, comme j'ai déjà la date des dernières règles, en fait, c'est ça. C'est le recueil de cette date-là qui pose problème. Mais comme les gens ne sont pas trop patients pour amener les femmes à pouvoir donner cette période-là, on part rapidement. Dès qu'on demande et si elle dit qu'elle ne connaît pas, on dit inconnus, et on continue. Alors que si tu essaies d'insister un peu, tu vas l'amener à situer cette période-là et à te donner. Voilà. Bon, ici, de façon générale, les gens passent par l'échographie la plupart du temps. 00:10:55-6

**Interviewer (00:09:57-6):** When calculating gestational age, which estimation method is given priority in your practice?

**Person 1 (00:10:08-1):** For me, the primary method is using the gestogram. I make every effort to have the woman provide the date of her last menstrual period. With that information, I can effectively use the gestogram to estimate gestational age.

**Interviewer (00:10:25-8):** But why do you prefer this method?

**Person 1 (00:10:25-5):** It's because I already have the date of the last period, which is crucial. The challenge is in gathering this date. Often, people don't take the time to encourage women to recall and provide this period. If a woman says she doesn't know, many quickly move on to labeling it as unknown. But if you persist a bit, she can usually provide the date, which is very helpful. However, generally, people here rely more on ultrasound for estimation.

### Focus group of Colma1 health and social promotion center

[00:10:09-4](http://localhost:2300/file=C:/Users/USER/Desktop/ENTRETIENS%20PREMATURITE/CSPS-COLMA1/FOCUS%20GROUPE%20COLMA1.mp3time=609400) Interviewer: Maintenant au sein de votre centre de santé, quelle estimation a la priorité? [00:10:22-2](http://localhost:2300/file=C:/Users/USER/Desktop/ENTRETIENS%20PREMATURITE/CSPS-COLMA1/FOCUS%20GROUPE%20COLMA1.mp3time=622200)

[00:10:22-2](http://localhost:2300/file=C:/Users/USER/Desktop/ENTRETIENS%20PREMATURITE/CSPS-COLMA1/FOCUS%20GROUPE%20COLMA1.mp3time=622200) Person 1: De façon prioritaire on se base sur la date des dernières règles, si la femme n'arrive pas à donner la date des dernières règles, on prend la HU. [00:10:40-4](http://localhost:2300/file=C:/Users/USER/Desktop/ENTRETIENS%20PREMATURITE/CSPS-COLMA1/FOCUS%20GROUPE%20COLMA1.mp3time=640400)

00:10:09-4 Interviewer: Maintenant, au sein de votre centre de santé, quelle estimation a la priorité ? 00:10:22-2

00:10:22-2 Person 1: De façon prioritaire, on se base sur la date des dernières règles, si la femme n'arrive pas à donner la date des dernières règles, on prend la HU. 00:10:40-4

**Interviewer (00:10:09-4):** Within your health center, which method is prioritized for estimating gestational age?

**Person 1 (00:10:22-2):** Our priority is to base the estimate on the date of the last menstrual period. If the woman cannot provide the date of her last period, we then turn to using the fundal height (HU) measurement.

### Major of Colma1 health and social promotion center

[00:06:55-1](http://localhost:2300/file=C:/Users/USER/Desktop/ENTRETIENS%20PREMATURITE/CSPS-COLMA1/MAJOR%20CSPS%20COLMA1.MP3time=415100) Interviewer: Maintenant au niveau de votre centre de santé, pour le calcul de l'âge gestationnel, quelle estimation a la priorité par rapport à vos methodes de datation de la grossesse? [00:07:13-8](http://localhost:2300/file=C:/Users/USER/Desktop/ENTRETIENS%20PREMATURITE/CSPS-COLMA1/MAJOR%20CSPS%20COLMA1.MP3time=433800)

[00:07:13-8](http://localhost:2300/file=C:/Users/USER/Desktop/ENTRETIENS%20PREMATURITE/CSPS-COLMA1/MAJOR%20CSPS%20COLMA1.MP3time=433800) Person 1: pour etre le plus exact possible c'est l'échographie qui permet de donner avec precision l'âge exacte de la grossesse. mais d'après ce que j'ai dis , certaines femmes vont aller le faire par contre d'autres n'iront pas faire, sinon c'est ce qui est recommandé.c'est vrai qu'en se basant sur les methodes standards,en regardant l'evolution normal, c'est à dire ce qui est affiché sur le tableau dans les salles, en se referant à cela on peut se tromper. [00:07:41-6](http://localhost:2300/file=C:/Users/USER/Desktop/ENTRETIENS%20PREMATURITE/CSPS-COLMA1/MAJOR%20CSPS%20COLMA1.MP3time=461600)

00:06:55-1 Interviewer: Maintenant, au niveau de votre centre de santé, pour le calcul de l'âge gestationnel, quelle estimation a la priorité par rapport à vos méthodes de datation de la grossesse ? 00:07:13-8

00:07:13-8 Person 1: Pour être le plus exact possible, c'est l'échographie qui permet de donner avec précision l'âge exact de la grossesse. Mais, d'après ce que j'ai dit, certaines femmes vont aller le faire, par contre, d'autres n'iront pas faire, sinon c'est ce qui est recommandé. C'est vrai qu'en se basant sur les méthodes standards, en regardant l'évolution normale, c'est-à-dire ce qui est affiché sur le tableau dans les salles, en se référant à cela on peut se tromper. 00:07:41-6

**Interviewer (00:06:55-1):** At your health center, which estimate is prioritized for calculating gestational age?

**Person 1 (00:07:13-8):** For the most accurate determination of pregnancy age, we rely on ultrasound. It's the recommended method. While some women get it done, others don't. Relying solely on standard methods or charts displayed in the rooms can sometimes lead to errors.

### Maternity manager of Colma1 health and social promotion center

[00:14:45-9](http://localhost:2300/file=C:/Users/USER/Desktop/ENTRETIENS%20PREMATURITE/CSPS-COLMA1/RESPONSABLE%20MATERNITE-COLMA1.mp3time=885900) Interviewer: Maintenant, au niveau de la détermination de l'âge gestionnel, quelle est la méthode qui a la priorité dans votre centre de santé? [00:14:54-7](http://localhost:2300/file=C:/Users/USER/Desktop/ENTRETIENS%20PREMATURITE/CSPS-COLMA1/RESPONSABLE%20MATERNITE-COLMA1.mp3time=894700)

[00:14:54-7](http://localhost:2300/file=C:/Users/USER/Desktop/ENTRETIENS%20PREMATURITE/CSPS-COLMA1/RESPONSABLE%20MATERNITE-COLMA1.mp3time=894700) Person 1: La methode? lorsque la femme connait la date des dernières règles, c'est l'idéal pour nous.La femme connait et la methode de calcul est là. Même si on prend 14 jours, on prend 10 jours, on prend 7 jours, on s'est réellement que c'est ce qui est l'idéal pour vraiment connaître l'âge de la grossesse de la femme, c'est la date des dernières règles. [00:15:20-0](http://localhost:2300/file=C:/Users/USER/Desktop/ENTRETIENS%20PREMATURITE/CSPS-COLMA1/RESPONSABLE%20MATERNITE-COLMA1.mp3time=920000)

00:14:45-9 Interviewer: Maintenant, au niveau de la détermination de l'âge gestationnel, quelle est la méthode qui a la priorité dans votre centre de santé ? 00:14:54-7

00:14:54-7 Person 1: La méthode ? Lorsque la femme connaît la date des dernières règles, c'est l'idéal pour nous. La femme connaît et la méthode de calcul est là. Même si on prend 14 jours, on prend 10 jours, on prend 7 jours, on sait réellement que c'est ce qui est l'idéal pour vraiment connaître l'âge de la grossesse de la femme, c'est la date des dernières règles. 00:15:20-0

**Interviewer (00:14:45-9):** In your health center, which method is prioritized for determining gestational age?

**Person 1 (00:14:54-7):** The preferred method? Ideally, it's when the woman knows the date of her last menstrual period. That's the best scenario for us. Once we have that date, our calculation method, whether we add 14 days, 10 days, or 7 days, becomes very effective. Knowing the date of the last menstrual period is crucial for accurately determining the gestational age of the pregnancy.

### Focus group of Farakan health and social promotion center

[00:16:15-4](http://localhost:2300/file=C:/Users/USER/Desktop/ENTRETIENS%20PREMATURITE/CSPS_FARAKAN/FOCUS_GROUPE-CSPS_FARAKAN.MP3time=975400) Interviewer: S'il n y a plus rien à ajouter nous allons continuer.Maintenant, pour le calcul de l'âge gestationnel quelle estimation a la priorité, celle de la hauteur utérine ou celle de la date des dernières règles ? [00:16:30-8](http://localhost:2300/file=C:/Users/USER/Desktop/ENTRETIENS%20PREMATURITE/CSPS_FARAKAN/FOCUS_GROUPE-CSPS_FARAKAN.MP3time=990800)

[00:16:30-8](http://localhost:2300/file=C:/Users/USER/Desktop/ENTRETIENS%20PREMATURITE/CSPS_FARAKAN/FOCUS_GROUPE-CSPS_FARAKAN.MP3time=990800) Person 7: Madame Santé Ramata, la date des dernières DDR on se fie plus à ça si la femme connaît ou bien souvent, l'échographie souvent c'est plus ou moins 2 semaines, souvent plus ou moins 2 semaines. [00:16:51-9](http://localhost:2300/file=C:/Users/USER/Desktop/ENTRETIENS%20PREMATURITE/CSPS_FARAKAN/FOCUS_GROUPE-CSPS_FARAKAN.MP3time=1011900)

[00:16:51-9](http://localhost:2300/file=C:/Users/USER/Desktop/ENTRETIENS%20PREMATURITE/CSPS_FARAKAN/FOCUS_GROUPE-CSPS_FARAKAN.MP3time=1011900) Person 4: Le matériel qu'on préfère, comme en appel, le gestogramme là c'est mieux si vraiment on se concentre bien sur ça, même si la femme ne connaît pas, toi tu peux l'aider. Moi j'ai confiance à aux gestogramme. [00:17:12-6](http://localhost:2300/file=C:/Users/USER/Desktop/ENTRETIENS%20PREMATURITE/CSPS_FARAKAN/FOCUS_GROUPE-CSPS_FARAKAN.MP3time=1032600)

[00:17:12-6](http://localhost:2300/file=C:/Users/USER/Desktop/ENTRETIENS%20PREMATURITE/CSPS_FARAKAN/FOCUS_GROUPE-CSPS_FARAKAN.MP3time=1032600) Person 3: L'élément phare, en tout cas, reste la date des dernières règles, mais vu que le plus souvent cet élément n'est pas receuilli , nous nous basons surtout sur la HU la hauteur utérine. [00:17:31-0](http://localhost:2300/file=C:/Users/USER/Desktop/ENTRETIENS%20PREMATURITE/CSPS_FARAKAN/FOCUS_GROUPE-CSPS_FARAKAN.MP3time=1051000)

[00:17:31-0](http://localhost:2300/file=C:/Users/USER/Desktop/ENTRETIENS%20PREMATURITE/CSPS_FARAKAN/FOCUS_GROUPE-CSPS_FARAKAN.MP3time=1051000) Person 5: (rire).Comme la sage femme l'a dit en tout cas, l'élément phare, c'est la date des dernières règles,mais maintenant, comme les femmes ne connaissent pas la date des dernières règles, on est obligé de se baser sur la hauteur uterine pour calculer l'âge de la grossesse. [00:17:49-5](http://localhost:2300/file=C:/Users/USER/Desktop/ENTRETIENS%20PREMATURITE/CSPS_FARAKAN/FOCUS_GROUPE-CSPS_FARAKAN.MP3time=1069500)

[00:17:49-5](http://localhost:2300/file=C:/Users/USER/Desktop/ENTRETIENS%20PREMATURITE/CSPS_FARAKAN/FOCUS_GROUPE-CSPS_FARAKAN.MP3time=1069500) Person 6: Ok, comme les autres ont dit, en dehors de la date des dernières règles c'est avec la hauteur utérine qu'on se base pour se rattrapper. [00:18:01-2](http://localhost:2300/file=C:/Users/USER/Desktop/ENTRETIENS%20PREMATURITE/CSPS_FARAKAN/FOCUS_GROUPE-CSPS_FARAKAN.MP3time=1081200)

00:16:15-4 Interviewer: S'il n'y a plus rien à ajouter, nous allons continuer. Maintenant, pour le calcul de l'âge gestationnel, quelle estimation a la priorité, celle de la hauteur utérine ou celle de la date des dernières règles ?

00:16:30-8 Person 7: Madame Santé Ramata, la date des dernières DDR, on se fie plus à ça si la femme connaît ou bien souvent, l'échographie souvent c'est plus ou moins 2 semaines, souvent plus ou moins 2 semaines.

00:16:51-9 Person 4: Le matériel qu'on préfère, comme en appel, le gestogramme là c'est mieux si vraiment on se concentre bien sur ça, même si la femme ne connaît pas, toi tu peux l'aider. Moi j'ai confiance aux gestogrammes.

00:17:12-6 Person 3: L'élément phare, en tout cas, reste la date des dernières règles, mais vu que le plus souvent cet élément n'est pas recueilli, nous nous basons surtout sur la HU, la hauteur utérine.

00:17:31-0 Person 5: (rire) Comme la sage-femme l'a dit en tout cas, l'élément phare, c'est la date des dernières règles, mais maintenant, comme les femmes ne connaissent pas la date des dernières règles, on est obligé de se baser sur la hauteur utérine pour calculer l'âge de la grossesse.

00:17:49-5 Person 6: Ok, comme les autres ont dit, en dehors de la date des dernières règles, c'est avec la hauteur utérine qu'on se base pour se rattraper.

**Interviewer (00:16:15-4):** If there's nothing else to add, let's proceed. When calculating gestational age, which is given priority: fundal height measurement or the date of the last period?

**Person 7 (00:16:30-8):** Usually, we rely more on the date of the last menstrual period (LMP), if the woman remembers it. However, ultrasound estimates can vary by about two weeks.

**Person 4 (00:16:51-9):** I prefer using the gestogram, even if the woman doesn't know her LMP date. I find it helpful.

**Person 3 (00:17:12-6):** The LMP date is key, but since it's often unknown, we mainly use fundal height (HU) measurements.

**Person 5 (00:17:31-0):** (laughing) As mentioned, the LMP date is important, but when unknown, we rely on fundal height to estimate pregnancy age.

**Person 6 (00:17:49-5):** Agreeing with others, besides the LMP date, we depend on fundal height measurements.

### Major of Farakan health and social promotion center

[00:05:51-3](http://localhost:2300/file=C:/Users/USER/Desktop/ENTRETIENS%20PREMATURITE/CSPS_FARAKAN/MAJOR%20FARAKAN.MP3time=351300) Interviewer: Au sein de votre formation sanitaire, quelle estimation a la priorité? [00:05:59-6](http://localhost:2300/file=C:/Users/USER/Desktop/ENTRETIENS%20PREMATURITE/CSPS_FARAKAN/MAJOR%20FARAKAN.MP3time=359600)

[00:05:59-6](http://localhost:2300/file=C:/Users/USER/Desktop/ENTRETIENS%20PREMATURITE/CSPS_FARAKAN/MAJOR%20FARAKAN.MP3time=359600) Person 1: Ici là souvent c'est la hauteur uterine qu'on utilise beaucoup. [00:06:05-0](http://localhost:2300/file=C:/Users/USER/Desktop/ENTRETIENS%20PREMATURITE/CSPS_FARAKAN/MAJOR%20FARAKAN.MP3time=365000)

[00:06:05-0](http://localhost:2300/file=C:/Users/USER/Desktop/ENTRETIENS%20PREMATURITE/CSPS_FARAKAN/MAJOR%20FARAKAN.MP3time=365000) Interviewer: Pourquoi? [00:06:06-3](http://localhost:2300/file=C:/Users/USER/Desktop/ENTRETIENS%20PREMATURITE/CSPS_FARAKAN/MAJOR%20FARAKAN.MP3time=366300)

[00:06:06-3](http://localhost:2300/file=C:/Users/USER/Desktop/ENTRETIENS%20PREMATURITE/CSPS_FARAKAN/MAJOR%20FARAKAN.MP3time=366300) Person 1: Il y a le gestogramme aussi. Comme nous sommes en ville, nous demandons souvent l'échographie et celles qui ont les moyens géneralement font l'echographie.Parce que l'echographie est plus exacte. [00:06:24-3](http://localhost:2300/file=C:/Users/USER/Desktop/ENTRETIENS%20PREMATURITE/CSPS_FARAKAN/MAJOR%20FARAKAN.MP3time=384300)

00:05:51-3 Interviewer: Au sein de votre formation sanitaire, quelle estimation a la priorité ?

00:05:59-6 Person 1: Ici, là souvent c'est la hauteur utérine qu'on utilise beaucoup.

00:06:05-0 Interviewer: Pourquoi ?

00:06:06-3 Person 1: Il y a le gestogramme aussi. Comme nous sommes en ville, nous demandons souvent l'échographie et celles qui ont les moyens généralement font l'échographie. Parce que l'échographie est plus exacte.

**Interviewer (00:05:51-3):** In your health facility, which method do you prioritize for estimating gestational age?

**Person 1 (00:05:59-6):** Here, we frequently use fundal height measurements.

**Interviewer (00:06:05-0):** Why is that?

**Person 1 (00:06:06-3):** We also use gestograms, but being in an urban area, we often recommend ultrasounds for those who can afford them. Ultrasounds are preferred because they are more accurate.

### Maternity manager of Farakan health and social promotion center

[00:08:30-9](http://localhost:2300/file=C:/Users/USER/Desktop/ENTRETIENS%20PREMATURITE/CSPS_FARAKAN/RESPONSABLE%20MATERNITE%20FARAKAN.MP3time=510900) Interviewer: Maintenant, Pour le calcul de l'âge gestationnel, quelle est l'estimation qu'il a priorité ? [00:08:39-5](http://localhost:2300/file=C:/Users/USER/Desktop/ENTRETIENS%20PREMATURITE/CSPS_FARAKAN/RESPONSABLE%20MATERNITE%20FARAKAN.MP3time=519500)

[00:08:39-5](http://localhost:2300/file=C:/Users/USER/Desktop/ENTRETIENS%20PREMATURITE/CSPS_FARAKAN/RESPONSABLE%20MATERNITE%20FARAKAN.MP3time=519500) Person 1: L'estimation qui a la priorité, c'est l'échographie mais il y a la date des dernières règles aussi. En fait il y a les deux, mais maintenant s'elle n'a pas la date des dernières règles, on sera obligé d'aller sur la base de l'échographie, c'est à dire les resultats de l'échographie.Puisque même s'elle connaît la date des dernières règles,on peut calculer la date probable d'accouchement. il y a aussi les resultats de l'echographie aussi. En tout cas les deux sont toujours demandées. [00:09:16-6](http://localhost:2300/file=C:/Users/USER/Desktop/ENTRETIENS%20PREMATURITE/CSPS_FARAKAN/RESPONSABLE%20MATERNITE%20FARAKAN.MP3time=556600)

00:08:30-9 Interviewer: Maintenant, pour le calcul de l'âge gestationnel, quelle est l'estimation qui a priorité ? 00:08:39-5

00:08:39-5 Person 1: L'estimation qui a la priorité, c'est l'échographie, mais il y a la date des dernières règles aussi. En fait, il y a les deux, mais maintenant, si elle n'a pas la date des dernières règles, on sera obligé d'aller sur la base de l'échographie, c'est-à-dire les résultats de l'échographie. Puisque même si elle connaît la date des dernières règles, on peut calculer la date probable d'accouchement. Il y a aussi les résultats de l'échographie aussi. En tout cas, les deux sont toujours demandées. 00:09:16-6

**Interviewer (00:08:30-9):** When calculating gestational age, which estimate is given precedence?

**Person 1 (00:08:39-5):** The primary estimate we rely on is the ultrasound, but the date of the last menstrual period is also important. If the date of the last period is unknown, we have to depend on the ultrasound results. However, if she knows the date of her last period, we can calculate the probable date of delivery using both the last period date and ultrasound results. Both methods are frequently used.

## 3.7 Appointment for prenatal consultations with ultrasound measurement carried out

### Focus group of Accart-ville urban medical center

[00:06:49-8](http://localhost:2300/file=C:/Users/USER/Desktop/ENTRETIENS)Interviewer: est ce que les femmes viennent souvent avec une estimation par echographie? [00:06:54-1](http://localhost:2300/file=C:/Users/USER/Desktop/ENTRETIENS%20PREMATURITE/CSPS-ACCART-VILLE/FOCUS%20GROUPE%20ACCART-VILLE-BON.mp3time=414100)

[00:06:54-1](http://localhost:2300/file=C:/Users/USER/Desktop/ENTRETIENS%20PREMATURITE/CSPS-ACCART-VILLE/FOCUS%20GROUPE%20ACCART-VILLE-BON.mp3time=414100)Person 1: bon, celles qui ont eu des soucis avant de commencer leurs CPN,des maux de ventre donc elles viennent consulter non pas pour la CPN mais pour causes de maladies. a ses femmes nous demandons l'echographie et par la suite quand elles viennent à leurs premières CPN elles viennent avec l'echographie. [00:07:08-5](http://localhost)

[00:07:08-5](http://localhost:2300/file=C:/Users/USER/Desktop/ENTRETIENS)Interviewer: dans le cas ou à leurs première CPN elles apportent l'echographie, quelle estimation aura la priorité? [00:07:13-0](http://localhost:2300/file=C:/Users/USER/Desktop/ENTRETIENS%20PREMATURITE/CSPS-ACCART-VILLE/FOCUS%20GROUPE%20ACCART-VILLE-BON.mp3time=433000)

[00:07:13-0](http://localhost:2300/file=C:/Users/USER/Desktop/ENTRETIENS%20PREMATURITE/CSPS-ACCART-VILLE/FOCUS%20GROUPE%20ACCART-VILLE-BON.mp3time=433000) Person 1: si elle vient avec l'echographie, nous regardons la date de l'echographie,et si nous nous constatons que c'est une echographie recente, nous considerons en ce moment l'echographie et nous comparons avec ce que nous avons trouvé par la mesure. dans le cas ou elle a fait l'echographie il y a longtemps avant de venir pour la CPN, on mesure avec notre methode et lorsqu'il y a des doutes nous l'envoyons faire une autre echographie. [00:07:39-2](http://localhost:2300/file=C:/Users/USER/Desktop/ENTRETIENS%20PREMATURITE/CSPS-ACCART-VILLE/FOCUS%20GROUPE%20ACCART-VILLE-BON.mp3time=459200)

[00:07:39-2](http://localhost:2300/file=C:/Users/USER/Desktop/ENTRETIENS%20PREMATURITE/CSPS-ACCART-VILLE/FOCUS%20GROUPE%20ACCART-VILLE-BON.mp3time=459200) Interviewer: est ce qu'il y a d'autres contributions par rapport à la question? [00:07:43-3](http://lo)

[00:07:43-3](http://localhost:2300/file=C:/Users/USER/Desktop/ENTRETIENS) Person 2: premièrement nous partons sur le plan clinique, si le plan clinique c'est difficile nous passons maintenant à l'echogrphie c'est de façon prioritaire la manière dont nous procedons içi. [00:08:19-7](%22h)

[00:08:19-7](http://localhost:2300/file=C:/Users/USER/Desktop/ENTRETIENS) Person 5: oui quelques unes viennent avec l'echographie mais pas elles toutes c'est quelques unes seulement elles ne sont pas nombreuses. [00:08:30-5](http://localhost:2300/file=C:/Users/USER/Desktop/EN)

[00:08:30-5](http://localhost:2300/file=C:/Users/USER/Desktop/ENTRETIENS%20PREMATURITE/CSPS-ACCART-VILLE/FOCUS%20GROUPE%20ACCART-VILLE-BON.mp3time=510500)Person 6: quelques unes viennent, surtout celles qui sont d'abord venues en salle pour des soucis de santé,par exemple pour des douleurs qui persistent ou bien des saignements à qui nous avons demander l'echographie ,elles le plus souvent elles viennent avec lors de leurs CPN. [00:08:52-4](http://localhost:2300/file=C:/Users/USER/Desktop/ENTRETIENS%20PREMATURITE/CSPS-ACCART-VILLE/FOCUS%20GROUPE%20ACCART-VILLE-BON.mp3time=532400)

[00:08:52-4](http://localhost:2300/file=C:/Users/USER/Desktop/ENTRET) Interviewer: ok. dans ce cas, s'elles viennent avec une estimation par echographie, quelle est l'estimation qui aura la priorité en ce moment? [00:09:15-0](http://l)

[00:09:15-0](http://localhost:2300/file=C:/Users/USER/Desktop/ENTRETIENS) Person 1: cela depend du temps qu'elle a fait l'echographie avant de venir commencer sa première CPN [00:09:26-4](http://localhost:2300/file=C:/Users/USER/Desktop/ENTRETIENS)

[00:09:26-4](http://localhost:2300/file=C:/Users/USER/Desktop/ENTRETIENS%20PREMATURITE/CSPS-ACCART-VILLE/FOCUS%20GROUPE%20ACCART-VILLE-BON.mp3time=566400) Person 2: il arrive aussi que nous fassions des comparaisons s'elle amène une echographie, nous demandons aussi la date des dernières règles pour confronter puis, l'echographie aussi a une marge d'erreurs. c'est vraiment l'âge chronologique qui est beaucoup plus fiable que les autres moyens de datation de la grossesse. si elle vient avec son echographie et que sur le plan clinique nous constatons qu'il y a une discordance, on peut demander la date des dernières règles pour confronter afin de trouver lâge exate de la grossesse. [00:10:09-4](http://l)

00:06:49-8 Interviewer: Est-ce que les femmes viennent souvent avec une estimation par échographie ?

00:06:54-1 Person 1: Bon, celles qui ont eu des soucis avant de commencer leurs CPN, des maux de ventre donc elles viennent consulter non pas pour la CPN mais pour causes de maladies. À ces femmes, nous demandons l'échographie et par la suite quand elles viennent à leurs premières CPN, elles viennent avec l'échographie.

00:07:08-5 Interviewer: Dans le cas où, à leurs premières CPN, elles apportent l'échographie, quelle estimation aura la priorité ?

00:07:13-0 Person 1: Si elle vient avec l'échographie, nous regardons la date de l'échographie, et si nous constatons que c'est une échographie récente, nous considérons en ce moment l'échographie et nous comparons avec ce que nous avons trouvé par la mesure. Dans le cas où elle a fait l'échographie il y a longtemps avant de venir pour la CPN, on mesure avec notre méthode et lorsqu'il y a des doutes, nous l'envoyons faire une autre échographie.

00:07:39-2 Person 2: Premièrement, nous partons sur le plan clinique, si le plan clinique, c'est difficile, nous passons maintenant à l'échographie, c'est de façon prioritaire la manière dont nous procédons ici.

00:08:19-7 Person 5: Oui, quelques-unes viennent avec l'échographie mais pas elles toutes, c'est quelques-unes seulement, elles ne sont pas nombreuses.

00:08:30-5 Person 6: Quelques-unes viennent, surtout celles qui sont d'abord venues en salle pour des soucis de santé, par exemple pour des douleurs qui persistent ou bien des saignements à qui nous avons demandé l'échographie, elles le plus souvent elles viennent avec lors de leurs CPN.

00:08:52-4 Interviewer: Ok. Dans ce cas, s'elles viennent avec une estimation par échographie, quelle est l'estimation qui aura la priorité en ce moment ?

00:09:15-0 Person 1: Cela dépend du temps qu'elle a fait l'échographie avant de venir commencer sa première CPN.

00:09:26-4 Person 2: Il arrive aussi que nous fassions des comparaisons si elle amène une échographie, nous demandons aussi la date des dernières règles pour confronter puis, l'échographie aussi a une marge d'erreurs. C'est vraiment l'âge chronologique qui est beaucoup plus fiable que les autres moyens de datation de la grossesse. Si elle vient avec son échographie et que sur le plan clinique nous constatons qu'il y a une discordance, on peut demander la date.

**Interviewer (00:06:49-8):** Do women often come to your facility with an ultrasound estimate?

**Person 1 (00:06:54-1):** Women who experience issues like stomach pains before starting their antenatal care (ANC) often consult us not for ANC but due to these ailments. We request an ultrasound for them. Then, when they attend their first prenatal visit (CPN), they typically bring this ultrasound with them.

**Interviewer (00:07:08-5):** If they bring an ultrasound to their first ANC visit, which estimate do you prioritize?

**Person 1 (00:07:13-0):** If the ultrasound is recent, we prioritize it and compare its findings with our measurements. If the ultrasound was done a while ago, we use our measurement methods and, if there are doubts, we may send her for another ultrasound.

**Person 2 (00:07:39-2):** Initially, we start with clinical assessments. If these are inconclusive, we then prioritize ultrasound.

**Person 5 (00:08:19-7):** Some women come with an ultrasound, but they are a minority.

**Person 6 (00:08:30-5):** Women who first visited us for health issues like persistent pain or bleeding, and were asked to get an ultrasound, often bring it to their prenatal visit.

**Interviewer (00:08:52-4):** In such cases, when they arrive with an ultrasound estimate, which one do you prioritize?

**Person 1 (00:09:15-0):** It depends on the age of the ultrasound at the time of her first prenatal visit.

**Person 2 (00:09:26-4):** We also make comparisons. If necessary, we request the date of the last menstrual period for additional comparison. Ultrasounds have a margin of error, so the chronological age from the last period can be more reliable. If there's a discrepancy between the clinical observation and the ultrasound, we may ask for the LMP date.

### Manager of Accart-ville urban medical center

[00:07:48-4](http://localhost:2300/file=C:/Users/USER/Desktop/ENTRETIENS%20PREMATURITE/CSPS-ACCART-VILLE/RESPONSABLE%20CMU%20ACCART-VILLE.MP3time=468400)Interviewer: cela veut dire que les femmes viennent souvent au sein de votre structure de santé avec des estimations par échographie. [00:07:55-1](http://localhost:2300/file=C:/Users/USER/Desktop/ENTRETIENS%20PREMATURITE/CSPS-ACCART-VILLE/RESPONSABLE%20CMU%20ACCART-VILLE.MP3time=475100)

[00:07:55-1](http://localhost:2300/file=C:/Users/USER/Desktop/ENTRETIENS%20PREMATURITE/CSPS-ACCART-VILLE/RESPONSABLE%20CMU%20ACCART-VILLE.MP3time=475100) Person 1: Oui [00:07:56-6](http://localhost:2300/file=C:/Users/USER/Desktop/ENTRETIENS%20PREMATURITE/CSPS-ACCART-VILLE/RESPONSABLE%20CMU%20ACCART-VILLE.MP3time=476600)

[00:07:56-6](http://localhost:2300/file=C:/Users/USER/Desktop/ENTRETIENS%20PREMATURITE/CSPS-ACCART-VILLE/RESPONSABLE%20CMU%20ACCART-VILLE.MP3time=476600)Interviewer: maintenant s'elles viennent avec une estimation par échographie, est ce sur cette dernière que vous allez vous baser pour estimer l'âge gestationnel [00:08:02-4](http://localhost:2300/file=C:/Users/USER/Desktop/ENTRETIENS%20PREMATURITE/CSPS-ACCART-VILLE/RESPONSABLE%20CMU%20ACCART-VILLE.MP3time=482400)

[00:08:02-4](http://localhost:2300/file=C:/Users/USER/Desktop/ENTRETIENS%20PREMATURITE/CSPS-ACCART-VILLE/RESPONSABLE%20CMU%20ACCART-VILLE.MP3time=482400)Person 1: Oui, c'est comme cela. [00:08:09-1](http://localhost:2300/file=C:/Users/USER/Desktop/ENTRETIENS%20PREMATURITE/CSPS-ACCART-VILLE/RESPONSABLE%20CMU%20ACCART-VILLE.MP3time=489100)

00:07:48-4 Interviewer: Cela veut dire que les femmes viennent souvent au sein de votre structure de santé avec des estimations par échographie.

00:07:55-1 Person 1: Oui.

00:07:56-6 Interviewer: Maintenant, s'elles viennent avec une estimation par échographie, est-ce sur cette dernière que vous allez vous baser pour estimer l'âge gestationnel ?

00:08:02-4 Person 1: Oui, c'est comme cela.

**Interviewer (00:07:48-4):** Does this mean women frequently come to your facility with ultrasound estimates?

**Person 1 (00:07:55-1):** Yes, they do.

**Interviewer (00:07:56-6):** If they arrive with an ultrasound estimate, is that the primary method you use to determine gestational age?

**Person 1 (00:08:02-4):** Yes, that's our standard practice.

### Maternity manager of Accart-ville urban medical center

[00:08:33-5](http://localhost:2300/file=C:/Users/USER/Desktop/ENTRETIENS%20PREMATURITE/CSPS-ACCART-VILLE/RESPONSABLE%20MATERNITE%20CMU%20ACCART-VILLE.MP3time=513500) Interviewer: maintenant est ce que les femmes viennent souvent avec une estimation par echographie? [00:08:37-1](http://localhost:2300/file=C:/Users/USER/Desktop/ENTRETIENS%20PREMATURITE/CSPS-ACCART-VILLE/RESPONSABLE%20MATERNITE%20CMU%20ACCART-VILLE.MP3time=517100)

[00:08:37-1](http://localhost:2300/file=C:/Users/USER/Desktop/ENTRETIENS%20PREMATURITE/CSPS-ACCART-VILLE/RESPONSABLE%20MATERNITE%20CMU%20ACCART-VILLE.MP3time=517100) Person 1: oui rares sont ces femmes qui viennent avec une echographie.c'est quand la femme a eu un problème de santé en debut de grossesse. par exemple des douleurs pelviennes oubien des saignements qu'elle peut consulter et on peut lui demander une echographie, et quand elle va commencer ses pésées elle vient avec l'echographie. mais se celles qui ont eu des problèmes de santé avant le debut de leur CPN ou bien de leurs soins prénataux. celles qui n'ont pas eu de problème la plupart c'est au centre içi qu'on leur demande l'echographie. [00:09:13-6](http://localhost:2300/file=C:/Users/USER/Desktop/ENTRETIENS%20PREMATURITE/CSPS-ACCART-VILLE/RESPONSABLE%20MATERNITE%20CMU%20ACCART-VILLE.MP3time=553600)

[00:09:13-6](http://localhost:2300/file=C:/Users/USER/Desktop/ENTRETIENS%20PREMATURITE/CSPS-ACCART-VILLE/RESPONSABLE%20MATERNITE%20CMU%20ACCART-VILLE.MP3time=553600) Interviewer: au cas ou les femmes viennent avec une échographie, qu'elle est la methode d'estimation de l'âge gestationnel de la grossesse qui sera priorisé? [00:09:21-7](http://localhost:2300/file=C:/Users/USER/Desktop/ENTRETIENS%20PREMATURITE/CSPS-ACCART-VILLE/RESPONSABLE%20MATERNITE%20CMU%20ACCART-VILLE.MP3time=561700)

[00:09:21-7](http://localhost:2300/file=C:/Users/USER/Desktop/ENTRETIENS%20PREMATURITE/CSPS-ACCART-VILLE/RESPONSABLE%20MATERNITE%20CMU%20ACCART-VILLE.MP3time=561700) Person 1: si elle connait la date de ses dernières règles, c'est sur cette base qu'on part.on a l'habitude de dire que le coran c'est pas la bible. c'est la clinique qui est encore mieux l'interrogatoire et la clinique qui sont plus fiables si on est convaincu on priorise cette methode par rapport à l'échographie. l'echographie c'est lorsque les autres moyens ne nous permettent pas de dater la grossesse que nous avons recours à l'échographie. [00:09:51-1](http://localhost:2300/file=C:/Users/USER/Desktop/ENTRETIENS%20PREMATURITE/CSPS-ACCART-VILLE/RESPONSABLE%20MATERNITE%20CMU%20ACCART-VILLE.MP3time=591100)

00:08:33-5 Interviewer: Maintenant, est-ce que les femmes viennent souvent avec une estimation par échographie ?

00:08:37-1 Person 1: Oui, rares sont ces femmes qui viennent avec une échographie. C'est quand la femme a eu un problème de santé en début de grossesse, par exemple des douleurs pelviennes ou bien des saignements qu'elle peut consulter et on peut lui demander une échographie, et quand elle va commencer ses pesées, elle vient avec l'échographie. Mais ce sont celles qui ont eu des problèmes de santé avant le début de leur CPN ou bien de leurs soins prénataux. Celles qui n'ont pas eu de problème, la plupart c'est au centre ici qu'on leur demande l'échographie.

00:09:13-6 Interviewer: Au cas où les femmes viennent avec une échographie, quelle est la méthode d'estimation de l'âge gestationnel de la grossesse qui sera priorisée ? 00:09:21-7

00:09:21-7 Person 1: Si elle connaît la date de ses dernières règles, c'est sur cette base qu'on part. On a l'habitude de dire que le coran, ce n'est pas la bible. C'est la clinique qui est encore mieux, l'interrogatoire et la clinique qui sont plus fiables. Si on est convaincu, on priorise cette méthode par rapport à l'échographie. L'échographie, c'est lorsque les autres moyens ne nous permettent pas de dater la grossesse que nous avons recours à l'échographie. 00:09:51-1

**Interviewer (00:08:33-5):** Do women often come to your facility with an ultrasound estimate?

**Person 1 (00:08:37-1):** It's actually rare for women to come in with an ultrasound already done. Usually, it's only those who have encountered health issues early in pregnancy, like pelvic pain or bleeding. These women might consult for these issues, and we ask them to get an ultrasound. When they begin their antenatal care (ANC) or prenatal visits, they bring this ultrasound with them. However, for those who haven’t had early pregnancy problems, we typically request an ultrasound here at our center.

**Interviewer (00:09:13-6):** When women come in with an ultrasound, which method of estimating gestational age do you prioritize?

**Person 1 (00:09:21-7):** We start with the date of the last menstrual period, if she knows it. We usually believe that clinical evaluation, including patient interviews and physical examinations, are more reliable than just relying on ultrasound. If we are convinced of the accuracy based on these clinical methods, we prioritize them over ultrasound. Ultrasound is used primarily when other means don’t provide a clear dating of the pregnancy.

### Focus group of Do medical center with surgical branch

[00:10:48-5](http://localhost:2300/file=C:/Users/USER/Desktop/ENTRETIENS%20PREMATURITE/CMA-DO/FOCUS%20GROUPE%20CMA-DO.mp3time=648500) Interviewer: Est ce qu'il arrive que les femmes viennent avec une estimation par échographie? [00:10:54-8](http://localhost:2300/file=C:/Users/USER/Desktop/ENTRETIENS%20PREMATURITE/CMA-DO/FOCUS%20GROUPE%20CMA-DO.mp3time=654800)

[00:10:54-8](http://localhost:2300/file=C:/Users/USER/Desktop/ENTRETIENS%20PREMATURITE/CMA-DO/FOCUS%20GROUPE%20CMA-DO.mp3time=654800) Person 8: Oui. [00:10:55-0](http://localhost:2300/file=C:/Users/USER/Desktop/ENTRETIENS%20PREMATURITE/CMA-DO/FOCUS%20GROUPE%20CMA-DO.mp3time=655000)

[00:10:55-0](http://localhost:2300/file=C:/Users/USER/Desktop/ENTRETIENS%20PREMATURITE/CMA-DO/FOCUS%20GROUPE%20CMA-DO.mp3time=655000) Interviewer: Dans ce cas, si elles amènent une estimation par échographie, quelle est l'estimation qui aura la priorité? [00:11:02-5](http://localhost:2300/file=C:/Users/USER/Desktop/ENTRETIENS%20PREMATURITE/CMA-DO/FOCUS%20GROUPE%20CMA-DO.mp3time=662500)

[00:11:02-5](http://localhost:2300/file=C:/Users/USER/Desktop/ENTRETIENS%20PREMATURITE/CMA-DO/FOCUS%20GROUPE%20CMA-DO.mp3time=662500) Person 3: Ça dépend aussi de quand elle va réaliser l'échographie. Si c'est une échographie précoce, on se fie à ça, c'est la meilleure datation. Mais si c'est une épopraphie tardive, je pense que c'est approximatif. [00:11:13-8](http://localhost:2300/file=C:/Users/USER/Desktop/ENTRETIENS%20PREMATURITE/CMA-DO/FOCUS%20GROUPE%20CMA-DO.mp3time=673800)

[00:11:13-8](http://localhost:2300/file=C:/Users/USER/Desktop/ENTRETIENS%20PREMATURITE/CMA-DO/FOCUS%20GROUPE%20CMA-DO.mp3time=673800) Interviewer: Une échographie tardive, à peu près à quelle periode de la grossesse? [00:11:16-2](http://localhost:2300/file=C:/Users/USER/Desktop/ENTRETIENS%20PREMATURITE/CMA-DO/FOCUS%20GROUPE%20CMA-DO.mp3time=676200)

[00:11:16-2](http://localhost:2300/file=C:/Users/USER/Desktop/ENTRETIENS%20PREMATURITE/CMA-DO/FOCUS%20GROUPE%20CMA-DO.mp3time=676200) Person 3: ... Par exemple s'elle fait l'échographie au troisième trimestre , ça donne une datation qui n'est pas juste. Mais s'elle fait l'échographie au premier trimestre, géneralement ça donne la meilleure datation. [00:11:29-3](http://localhost:2300/file=C:/Users/USER/Desktop/ENTRETIENS%20PREMATURITE/CMA-DO/FOCUS%20GROUPE%20CMA-DO.mp3time=689300)

00:10:48-5 Interviewer: Est-ce qu'il arrive que les femmes viennent avec une estimation par échographie ?

00:10:54-8 Person 8: Oui.

00:10:55-0 Interviewer: Dans ce cas, si elles amènent une estimation par échographie, quelle est l'estimation qui aura la priorité ?

00:11:02-5 Person 3: Ça dépend aussi de quand elle va réaliser l'échographie. Si c'est une échographie précoce, on se fie à ça, c'est la meilleure datation. Mais si c'est une échographie tardive, je pense que c'est approximatif.

00:11:13-8 Interviewer: Une échographie tardive, à peu près à quelle période de la grossesse ?

00:11:16-2 Person 3: Par exemple, si elle fait l'échographie au troisième trimestre, ça donne une datation qui n'est pas juste. Mais si elle fait l'échographie au premier trimestre, généralement ça donne la meilleure datation.

**Interviewer (00:10:48-5):** Do women sometimes arrive with an ultrasound estimate?

**Person 8 (00:10:54-8):** Yes.

**Interviewer (00:10:55-0):** If they bring an ultrasound estimate, which one do you prioritize?

**Person 3 (00:11:02-5):** It depends on the timing of the ultrasound. An early ultrasound is more reliable and provides the best dating. However, if it's a late ultrasound, it's less precise.

**Interviewer (00:11:13-8):** What do you consider a late ultrasound in terms of pregnancy timing?

**Person 3 (00:11:16-2):** An ultrasound done in the third trimester is less accurate. But one done in the first trimester generally offers the best dating.

### Gynecologist 1

[00:07:48-3](http://localhost:2300/file=C:/Users/USER/Desktop/ENTRETIENS%20PREMATURITE/CMA-DO/GENYCOLOGUE%2001.mp3time=468300) Interviewer: Est-ce qu'il arrive souvent que les femmes viennent avec une estimation par échographie ? [00:07:52-8](http://localhost:2300/file=C:/Users/USER/Desktop/ENTRETIENS%20PREMATURITE/CMA-DO/GENYCOLOGUE%2001.mp3time=472800)

[00:07:52-8](http://localhost:2300/file=C:/Users/USER/Desktop/ENTRETIENS%20PREMATURITE/CMA-DO/GENYCOLOGUE%2001.mp3time=472800) Person 1: Oui. [00:07:54-0](http://localhost:2300/file=C:/Users/USER/Desktop/ENTRETIENS%20PREMATURITE/CMA-DO/GENYCOLOGUE%2001.mp3time=474000)

[00:07:54-0](http://localhost:2300/file=C:/Users/USER/Desktop/ENTRETIENS%20PREMATURITE/CMA-DO/GENYCOLOGUE%2001.mp3time=474000) Interviewer: Dans ce cas, quelle estimation a la priorité ? [00:07:56-5](http://localhost:2300/file=C:/Users/USER/Desktop/ENTRETIENS%20PREMATURITE/CMA-DO/GENYCOLOGUE%2001.mp3time=476500)

[00:07:56-5](http://localhost:2300/file=C:/Users/USER/Desktop/ENTRETIENS%20PREMATURITE/CMA-DO/GENYCOLOGUE%2001.mp3time=476500) Person 1: Non, c'est l'échographie, toujours. [00:08:00-0](http://localhost:2300/file=C:/Users/USER/Desktop/ENTRETIENS%20PREMATURITE/CMA-DO/GENYCOLOGUE%2001.mp3time=480000)

00:07:48-3 Interviewer: Est-ce qu'il arrive souvent que les femmes viennent avec une estimation par échographie ?

00:07:52-8 Person 1: Oui.

00:07:54-0 Interviewer: Dans ce cas, quelle estimation a la priorité ?

00:07:56-5 Person 1: Non, c'est l'échographie, toujours.

**Interviewer (00:07:48-3):** Do women often come to you with an ultrasound estimate?

**Person 1 (00:07:52-8):** Yes, they do.

**Interviewer (00:07:54-0):** In that case, which estimate do you prioritize?

**Person 1 (00:07:56-5):** We always prioritize the ultrasound.

### Gynecologist 2

[00:08:56-1](http://localhost:2300/file=C:/Users/USER/Desktop/ENTRETIENS%20PREMATURITE/CMA-DO/GENYCOLOGUE%2002.mp3time=536100) Interviewer: Est-ce que les femmes viennent souvent en consultation avec l'estimation par l'échographie ? [00:09:00-3](http://localhost:2300/file=C:/Users/USER/Desktop/ENTRETIENS%20PREMATURITE/CMA-DO/GENYCOLOGUE%2002.mp3time=540300)

[00:09:00-3](http://localhost:2300/file=C:/Users/USER/Desktop/ENTRETIENS%20PREMATURITE/CMA-DO/GENYCOLOGUE%2002.mp3time=540300) Person 1: Oui, il y en a qui font déjà l'échographie avant de venir en consultation. Elles sont peut-être allées dans un CSPS, beaucoup même se lèvent d'elles-mêmes,

[09:07 - 09:23]

elles font l'échographie et puis elles viennent pour présenter l'échographie. Donc on se base tout simplement sur ça et on fait les extrapolations. [00:09:17-0](http://localhost:2300/file=C:/Users/USER/Desktop/ENTRETIENS%20PREMATURITE/CMA-DO/GENYCOLOGUE%2002.mp3time=557000)

[00:09:17-0](http://localhost:2300/file=C:/Users/USER/Desktop/ENTRETIENS%20PREMATURITE/CMA-DO/GENYCOLOGUE%2002.mp3time=557000) Interviewer: Maintenant, dans les cas où elles viennent avec l'estimation par l'échographie, c'est l'estimation échographique qui sera la priorité ? [00:09:22-7](http://localhost:2300/file=C:/Users/USER/Desktop/ENTRETIENS%20PREMATURITE/CMA-DO/GENYCOLOGUE%2002.mp3time=562700)

[00:09:22-7](http://localhost:2300/file=C:/Users/USER/Desktop/ENTRETIENS%20PREMATURITE/CMA-DO/GENYCOLOGUE%2002.mp3time=562700) Person 1: C'est ça qui sera la priorité puisqu'elle ne connait pas la date des dernières règles. Donc au moins ça, c'est un élément matériel sur lequel on peut se baser pour évoluer. [00:09:32-1](http://localhost:2300/file=C:/Users/USER/Desktop/ENTRETIENS%20PREMATURITE/CMA-DO/GENYCOLOGUE%2002.mp3time=572100)

00:08:56-1 Interviewer: Est-ce que les femmes viennent souvent en consultation avec l'estimation par l'échographie ?

00:09:00-3 Person 1: Oui, il y en a qui font déjà l'échographie avant de venir en consultation. Elles sont peut-être allées dans un CSPS, beaucoup même se lèvent d'elles-mêmes, elles font l'échographie et puis elles viennent pour présenter l'échographie. Donc on se base tout simplement sur ça et on fait les extrapolations.

00:09:17-0 Interviewer: Maintenant, dans les cas où elles viennent avec l'estimation par l'échographie, c'est l'estimation échographique qui sera la priorité ?

00:09:22-7 Person 1: C'est ça qui sera la priorité puisqu'elle ne connait pas la date des dernières règles. Donc au moins ça, c'est un élément matériel sur lequel on peut se baser pour évoluer.

**Interviewer (00:08:56-1):** Do women frequently come for consultations with an ultrasound already done?

**Person 1 (00:09:00-3):** Yes, many women have an ultrasound before consulting with us. They might visit a local health center or even get an ultrasound independently, then bring the results to us. We then base our assessment on the ultrasound findings.

**Interviewer (00:09:17-0):** So in cases where they come with an ultrasound, is that usually the primary method you rely on?

**Person 1 (00:09:22-7):** Yes, it becomes the priority, especially if the woman is uncertain about her last menstrual period. The ultrasound provides a tangible reference for us to use.

### Chief physician of Do health district

[00:13:53-7](http://localhost:2300/file=C:/Users/USER/Desktop/ENTRETIENS%20PREMATURITE/CMA-DO/MCD-DO.mp3time=833700) Interviewer: Est-ce qu'il y a des femmes qui viennent avec une estimation par échographie? [00:13:57-7](http://localhost:2300/file=C:/Users/USER/Desktop/ENTRETIENS%20PREMATURITE/CMA-DO/MCD-DO.mp3time=837700)

[00:13:57-7](http://localhost:2300/file=C:/Users/USER/Desktop/ENTRETIENS%20PREMATURITE/CMA-DO/MCD-DO.mp3time=837700) Person 1: Ça peut arriver. La chose qui paraît de plus en plus intéressante, on a des sages femmes dans toutes les formations sanitaires périphériques. Surtout à Bobo et en milieu rural, on en a dans certains coins. Donc elles-mêmes demandent par moment l'échographie dans le cas du suivi. La femme peut prendre ça et elle se retrouve ici. Elle se retrouve avec l'échographie qui nous dit à quelle période on était à telle semaine d'aménorrhée. Sinon, on a un appareil d'échographie au niveau du CMA ici. Normalement, les CPN du CMA doivent être des CPN de référence. Ce n'est pas une activité première d'un CMA. Le CMA vient en complément des formations sanitaires périphériques. Ce sont les acteurs d'ici qui devraient demander. Mais on n'en fait pas rigueur. Si on sait que c'est un continium. Si l'autre demande, on regarde, ça ne pose pas de problème. #44 15:07-9#

[00:15:07-9](http://localhost:2300/file=C:/Users/USER/Desktop/ENTRETIENS%20PREMATURITE/CMA-DO/MCD-DO.mp3time=907900) Interviewer: Maintenant, dans le cas où les femmes amènent l'estimation par échographie, quelle est la méthode qui sera priorisée? [00:15:17-6](http://localhost:2300/file=C:/Users/USER/Desktop/ENTRETIENS%20PREMATURITE/CMA-DO/MCD-DO.mp3time=917600)

[00:15:17-6](http://localhost:2300/file=C:/Users/USER/Desktop/ENTRETIENS%20PREMATURITE/CMA-DO/MCD-DO.mp3time=917600) Person 1: Que tu aies fait l'échographie ou pas, on va mesurer notre H.U. Que tu connais ta date de dernière règle ou pas, on va mesurer notre H.U. Parce que ça nous permet d'apprécier l'évolution aussi de la grossesse. On peut connaitre la date de dernière règle. Des fois, on sent que les choses ne vont pas ensemble. L'enfant peut être un macrosome ou un hypotrophe. La date de dernière règles va dire que non, au regard de ça, normalement on est à 8 mois de grossesse. Toi tu vas faire ta H.U. et tu vois que tu es à 7 mois ou 6 mois. Là, tu sais que l'enfant soit est un hypotrophe ou un enfant qui souffre. Tu peux demander l'échographie même complémentaire pour voir. Comme l'échographie permet d'avoir des visuels, on va regarder. On peut faire la H.U. et on voit que c'est gros alors qu'en réalité, c'est du liquide même qui est dedans qui a rendu ce resultat. Donc, quel qu'en soit, la H.U. est mesurée parce que c'est dans la consultation classique. [00:16:27-1](http://localhost:2300/file=C:/Users/USER/Desktop/ENTRETIENS%20PREMATURITE/CMA-DO/MCD-DO.mp3time=987100)

00:13:53-7 Interviewer: Est-ce qu'il y a des femmes qui viennent avec une estimation par échographie ?

00:13:57-7 Person 1: Ça peut arriver. La chose qui paraît de plus en plus intéressante, on a des sage-femmes dans toutes les formations sanitaires périphériques. Surtout à Bobo et en milieu rural, on en a dans certains coins. Donc elles-mêmes demandent par moment l'échographie dans le cas du suivi. La femme peut prendre ça et elle se retrouve ici. Elle se retrouve avec l'échographie qui nous dit à quelle période on était à telle semaine d'aménorrhée. Sinon, on a un appareil d'échographie au niveau du CMA ici. Normalement, les CPN du CMA doivent être des CPN de référence. Ce n'est pas une activité première d'un CMA. Le CMA vient en complément des formations sanitaires périphériques. Ce sont les acteurs d'ici qui devraient demander. Mais on n'en fait pas rigueur. Si on sait que c'est un continuum. Si l'autre demande, on regarde, ça ne pose pas de problème.

00:15:07-9 Interviewer: Maintenant, dans le cas où les femmes amènent l'estimation par échographie, quelle est la méthode qui sera priorisée ?

**00:15:17-6 Person 1:** Que tu aies fait l'échographie ou pas, on va mesurer notre HU. Que tu connais ta date de dernière règle ou pas, on va mesurer notre HU. Parce que ça nous permet d'apprécier l'évolution aussi de la grossesse. On peut connaître la date de dernière règle. Parfois, on sent que les choses ne vont pas ensemble. L'enfant peut être un macrosome ou un hypotrophe. La date de dernière règle va dire que non, au regard de ça, normalement on est à 8 mois de grossesse. Toi tu vas faire ta HU et tu vois que tu es à 7 mois ou 6 mois. Là, tu sais que l'enfant soit est un hypotrophe ou un enfant qui souffre. Tu peux demander l'échographie même complémentaire pour voir. Comme l'échographie permet d'avoir des visuels, on va regarder. On peut faire la H.U. et on voit que c'est gros alors qu'en réalité, c'est du liquide même qui est dedans qui a rendu ce résultat. Donc, quel qu'en soit, la H.U. est mesurée parce que c'est dans la consultation classique. [00:16:27-1](http://localhost:2300/file=C:/Users/USER/Desktop/ENTRETIENS%20PREMATURITE/CMA-DO/MCD-DO.mp3time=987100)

**Interviewer (00:13:53-7):** Do some women come with an ultrasound estimate?

**Person 1 (00:13:57-7):** Occasionally. Midwives in peripheral health facilities, especially in areas like Bobo and rural districts, sometimes request ultrasounds. Women may bring these ultrasounds to the CMA. We also have an ultrasound machine here. The CMA CPNs are reference centers, complementing peripheral health facilities. If a woman brings an ultrasound, we review it without issue.

**Interviewer (00:15:07-9):** When women bring an ultrasound estimate, which method do you prioritize?

**Person 1 (00:15:17-6):** Regardless of whether a woman has had an ultrasound or knows her last period date, we measure fundal height (HU). This measurement helps us assess the pregnancy's progress. Sometimes, there are discrepancies between the last period date and fundal height measurements, indicating potential fetal growth issues. In such cases, we might request an additional ultrasound for a clearer understanding. Fundal height is always measured as part of a standard consultation.

### Manager for maternity care unit of Do medical center with surgical branch

[00:10:55-6](http://localhost:2300/file=C:/Users/USER/Desktop/ENTRETIENS%20PREMATURITE/CMA-DO/RESPONSABLE%20UNITE%20DE%20SOIN%20CMA-DO.mp3time=655600) Interviewer: Est-ce qu'il arrive que des femmes viennent avec une estimation par échographie? [00:10:58-8](http://localhost:2300/file=C:/Users/USER/Desktop/ENTRETIENS%20PREMATURITE/CMA-DO/RESPONSABLE%20UNITE%20DE%20SOIN%20CMA-DO.mp3time=658800)

[00:10:58-8](http://localhost:2300/file=C:/Users/USER/Desktop/ENTRETIENS%20PREMATURITE/CMA-DO/RESPONSABLE%20UNITE%20DE%20SOIN%20CMA-DO.mp3time=658800) Person 1: Oui. [00:10:59-9](http://localhost:2300/file=C:/Users/USER/Desktop/ENTRETIENS%20PREMATURITE/CMA-DO/RESPONSABLE%20UNITE%20DE%20SOIN%20CMA-DO.mp3time=659900)

[00:10:59-9](http://localhost:2300/file=C:/Users/USER/Desktop/ENTRETIENS%20PREMATURITE/CMA-DO/RESPONSABLE%20UNITE%20DE%20SOIN%20CMA-DO.mp3time=659900) Interviewer: Et dans ce cas, quelle est la méthode qui sera priorisée? [00:11:03-3](http://localhost:2300/file=C:/Users/USER/Desktop/ENTRETIENS%20PREMATURITE/CMA-DO/RESPONSABLE%20UNITE%20DE%20SOIN%20CMA-DO.mp3time=663300)

[00:11:03-3](http://localhost:2300/file=C:/Users/USER/Desktop/ENTRETIENS%20PREMATURITE/CMA-DO/RESPONSABLE%20UNITE%20DE%20SOIN%20CMA-DO.mp3time=663300) Person 1: Priorisée, c'est-à-dire? [00:11:04-7](http://localhost:2300/file=C:/Users/USER/Desktop/ENTRETIENS%20PREMATURITE/CMA-DO/RESPONSABLE%20UNITE%20DE%20SOIN%20CMA-DO.mp3time=664700)

[00:11:04-7](http://localhost:2300/file=C:/Users/USER/Desktop/ENTRETIENS%20PREMATURITE/CMA-DO/RESPONSABLE%20UNITE%20DE%20SOIN%20CMA-DO.mp3time=664700) Interviewer: Vous avez parlé tantôt du gestogramme. [00:11:08-0](http://localhost:2300/file=C:/Users/USER/Desktop/ENTRETIENS%20PREMATURITE/CMA-DO/RESPONSABLE%20UNITE%20DE%20SOIN%20CMA-DO.mp3time=668000)

[00:11:08-0](http://localhost:2300/file=C:/Users/USER/Desktop/ENTRETIENS%20PREMATURITE/CMA-DO/RESPONSABLE%20UNITE%20DE%20SOIN%20CMA-DO.mp3time=668000) Person 1: Parce que l'échographie à un coup. Comme l'échographie n'est pas gratuit. C'est ça, non? C'est pas gratuit. Donc, ça veut dire c'est de moins. Mais dans les recommandations, il est recommandé qu'une femme enceinte, en tout cas, puisse bénéficier au moins d'une échographie.C'est ça, surtout l'échographie précoce, voilà, qui nous aide d'abord à savoir que c'est une grossesse, une grossesse intra uterine, et ainsi de suite. Donc, je me dis, dans tous les cas, on sera amené toujours à faire une échographie. Voilà, une échographie. Mais maintenant, ça, c'est une question de moyens.Si la femme a les moyens, parce que tu vas donner le bulletin, si elle n'a pas l'argent, pour pouvoir aller faire. Si c'était gratuit, oui. Ça allait être peut-être le moyen le plus utilisé. Sinon, moi, particulièrement, c'est à travers la date des dernières règles et je fais le calcul avec le gestogramme. [00:11:53-4](http://localhost:2300/file=C:/Users/USER/Desktop/ENTRETIENS%20PREMATURITE/CMA-DO/RESPONSABLE%20UNITE%20DE%20SOIN%20CMA-DO.mp3time=713400)

00:10:55-6 Interviewer: Est-ce qu'il arrive que des femmes viennent avec une estimation par échographie ? 00:10:58-8

00:10:58-8 Person 1: Oui. 00:10:59-9

00:10:59-9 Interviewer: Et dans ce cas, quelle est la méthode qui sera priorisée ? 00:11:03-3

00:11:03-3 Person 1: Priorisée, c'est-à-dire ? 00:11:04-7

00:11:04-7 Interviewer: Vous avez parlé tantôt du gestogramme. 00:11:08-0

00:11:08-0 Person 1: Parce que l'échographie a un coût. Comme l'échographie n'est pas gratuite. C'est ça, non ? Ce n'est pas gratuit. Donc, ça veut dire c'est de moins. Mais dans les recommandations, il est recommandé qu'une femme enceinte, en tout cas, puisse bénéficier au moins d'une échographie. C'est ça, surtout l'échographie précoce, voilà, qui nous aide d'abord à savoir que c'est une grossesse, une grossesse intra-utérine, et ainsi de suite. Donc, je me dis, dans tous les cas, on sera amené toujours à faire une échographie. Voilà, une échographie. Mais maintenant, ça, c'est une question de moyens. Si la femme a les moyens, parce que tu vas donner le bulletin, si elle n'a pas l'argent, pour pouvoir aller faire. Si c'était gratuit, oui. Ça aurait été peut-être le moyen le plus utilisé. Sinon, moi, particulièrement, c'est à travers la date des dernières règles et je fais le calcul avec le gestogramme. 00:11:53-4

**Interviewer (00:10:55-6):** Do women ever come to your facility with an ultrasound estimate?

**Person 1 (00:10:58-8):** Yes, they do.

**Interviewer (00:10:59-9):** In such cases, which method do you prioritize for pregnancy dating?

**Person 1 (00:11:03-3):** Prioritize in what sense?

**Interviewer (00:11:04-7):** Earlier, you mentioned the gestogram.

**Person 1 (00:11:08-0):** Ultrasound is not always used as the primary method due to its cost. While it is recommended that every pregnant woman should have at least one ultrasound, especially early in pregnancy, it's not always feasible. An early ultrasound is helpful for confirming intrauterine pregnancy. However, if the woman can't afford it, we can't rely solely on this method. In such cases, I personally use the date of the last menstrual period and calculate with the gestogram.

### Focus group of Colma1 health and social promotion center

[00:10:40-4](http://localhost:2300/file=C:/Users/USER/Desktop/ENTRETIENS%20PREMATURITE/CSPS-COLMA1/FOCUS%20GROUPE%20COLMA1.mp3time=640400) Interviewer: Est ce qu'il arrive souvent que les femmes viennent avec une estimation par échographie? [00:10:48-8](http://localhost:2300/file=C:/Users/USER/Desktop/ENTRETIENS%20PREMATURITE/CSPS-COLMA1/FOCUS%20GROUPE%20COLMA1.mp3time=648800)

[00:10:48-8](http://localhost:2300/file=C:/Users/USER/Desktop/ENTRETIENS%20PREMATURITE/CSPS-COLMA1/FOCUS%20GROUPE%20COLMA1.mp3time=648800) certainement c'est une échographie que nous avons demandé sinon elles ne partent pas d'elles même pour faire de l'échographie [00:10:55-5](http://localhost:2300/file=C:/Users/USER/Desktop/ENTRETIENS%20PREMATURITE/CSPS-COLMA1/FOCUS%20GROUPE%20COLMA1.mp3time=655500)

[00:10:55-5](http://localhost:2300/file=C:/Users/USER/Desktop/ENTRETIENS%20PREMATURITE/CSPS-COLMA1/FOCUS%20GROUPE%20COLMA1.mp3time=655500) Interviewer: maintenant au cas ou elle vient avec une échographie, quelle estimation aura la priorité? [00:11:02-1](http://localhost:2300/file=C:/Users/USER/Desktop/ENTRETIENS%20PREMATURITE/CSPS-COLMA1/FOCUS%20GROUPE%20COLMA1.mp3time=662100)

[00:11:02-1](http://localhost:2300/file=C:/Users/USER/Desktop/ENTRETIENS%20PREMATURITE/CSPS-COLMA1/FOCUS%20GROUPE%20COLMA1.mp3time=662100) Person 3: bon, géneralement on priorise l'échographie,on se base plus sur l'échographie. [00:11:06-7](http://localhost:2300/file=C:/Users/USER/Desktop/ENTRETIENS%20PREMATURITE/CSPS-COLMA1/FOCUS%20GROUPE%20COLMA1.mp3time=666700)

[00:11:06-7](http://localhost:2300/file=C:/Users/USER/Desktop/ENTRETIENS%20PREMATURITE/CSPS-COLMA1/FOCUS%20GROUPE%20COLMA1.mp3time=666700) Interviewer: Pourquoi? [00:11:08-5](http://localhost:2300/file=C:/Users/USER/Desktop/ENTRETIENS%20PREMATURITE/CSPS-COLMA1/FOCUS%20GROUPE%20COLMA1.mp3time=668500)

[00:11:08-5](http://localhost:2300/file=C:/Users/USER/Desktop/ENTRETIENS%20PREMATURITE/CSPS-COLMA1/FOCUS%20GROUPE%20COLMA1.mp3time=668500) Person 3: (rires) parce qu'au niveau de l'échographie, on se base sur plusieurs parametres afin de calculer, je crois qu'ils des calculs en fonction du foetus, de la mensuration, le fémur etc donc on se dit que c'est plus fiable que la HU. Puisque la HU peut être un cas de grossesse gémelaire aussi voilà pourquoi nous trouvons que c'est plus fiable avec l'échographie. [00:11:44-8](http://localhost:2300/file=C:/Users/USER/Desktop/ENTRETIENS%20PREMATURITE/CSPS-COLMA1/FOCUS%20GROUPE%20COLMA1.mp3time=704800)

00:10:40-4 Interviewer: Est-ce qu'il arrive souvent que les femmes viennent avec une estimation par échographie ? 00:10:48-8

00:10:48-8 Certainement c'est une échographie que nous avons demandée sinon elles ne partent pas d'elles-mêmes pour faire de l'échographie 00:10:55-5

00:10:55-5 Interviewer: Maintenant au cas où elle vient avec une échographie, quelle estimation aura la priorité ? 00:11:02-1

00:11:02-1 Person 3: Bon, généralement on priorise l'échographie, on se base plus sur l'échographie. 00:11:06-7

00:11:06-7 Interviewer: Pourquoi ? 00:11:08-5

00:11:08-5 Person 3: (rires) Parce qu'au niveau de l'échographie, on se base sur plusieurs paramètres afin de calculer, je crois qu'ils font des calculs en fonction du fœtus, de la mensuration, le fémur etc. donc on se dit que c'est plus fiable que la HU. Puisque la HU peut être un cas de grossesse gémellaire aussi voilà pourquoi nous trouvons que c'est plus fiable avec l'échographie. 00:11:44-8

**Interviewer (00:10:40-4):** Do women often come to your facility with an ultrasound estimate?

**Person 1 (00:10:48-8):** Usually, the ultrasounds are ones we've requested. It's rare for women to get them on their own initiative.

**Interviewer (00:10:55-5):** If a woman arrives with an ultrasound, which estimate do you prioritize for dating the pregnancy?

**Person 3 (00:11:02-1):** We generally prioritize the ultrasound. We rely more on it for accuracy.

**Interviewer (00:11:06-7):** Why is that?

**Person 3 (00:11:08-5):** (laughs) With ultrasounds, we use various parameters to calculate gestational age, including measurements of the fetus and the femur. We find it more reliable than fundal height measurement (HU), especially because HU can be misleading in cases like twin pregnancies.

### Major of Colma1 health and social promotion center

[00:07:41-6](http://localhost:2300/file=C:/Users/USER/Desktop/ENTRETIENS%20PREMATURITE/CSPS-COLMA1/MAJOR%20CSPS%20COLMA1.MP3time=461600) Interviewer: est ce que les femmes viennent souvent avec une estimation par echographie? [00:07:45-8](http://localhost:2300/file=C:/Users/USER/Desktop/ENTRETIENS%20PREMATURITE/CSPS-COLMA1/MAJOR%20CSPS%20COLMA1.MP3time=465800)

[00:07:45-8](http://localhost:2300/file=C:/Users/USER/Desktop/ENTRETIENS%20PREMATURITE/CSPS-COLMA1/MAJOR%20CSPS%20COLMA1.MP3time=465800) Person 1: oui, celle qui arrive à faire directement elle amène et l'âge est mis ainsi que le sexe, s'il y a des malformations, la quantité du liquide amiotique, tout ceci ressort generalement sur l'échographie. [00:08:01-8](http://localhost:2300/file=C:/Users/USER/Desktop/ENTRETIENS%20PREMATURITE/CSPS-COLMA1/MAJOR%20CSPS%20COLMA1.MP3time=481800)

[00:08:01-8](http://localhost:2300/file=C:/Users/USER/Desktop/ENTRETIENS%20PREMATURITE/CSPS-COLMA1/MAJOR%20CSPS%20COLMA1.MP3time=481800) Interviewer: est ce à dire que dans ce cas c'est l'estimation par l'échographie qui sera priorisée? [00:08:05-2](http://localhost:2300/file=C:/Users/USER/Desktop/ENTRETIENS%20PREMATURITE/CSPS-COLMA1/MAJOR%20CSPS%20COLMA1.MP3time=485200)

[00:08:05-2](http://localhost:2300/file=C:/Users/USER/Desktop/ENTRETIENS%20PREMATURITE/CSPS-COLMA1/MAJOR%20CSPS%20COLMA1.MP3time=485200) Person 1: Oui justement [00:08:06-3](http://localhost:2300/file=C:/Users/USER/Desktop/ENTRETIENS%20PREMATURITE/CSPS-COLMA1/MAJOR%20CSPS%20COLMA1.MP3time=486300)

00:07:41-6 Interviewer: Est-ce que les femmes viennent souvent avec une estimation par échographie ? 00:07:45-8

00:07:45-8 Person 1: Oui, celle qui arrive à faire directement elle amène, et l'âge est mis ainsi que le sexe, s'il y a des malformations, la quantité du liquide amniotique, tout ceci ressort généralement sur l'échographie. 00:08:01-8

00:08:01-8 Interviewer: Est-ce à dire que dans ce cas c'est l'estimation par l'échographie qui sera priorisée ? 00:08:05-2

00:08:05-2 Person 1: Oui, justement. 00:08:06-3

**Interviewer (00:07:41-6):** Do women often come to your facility with an ultrasound estimate?

**Person 1 (00:07:45-8):** Yes, those who manage to get an ultrasound bring it along. It provides details like the pregnancy age, sex of the baby, any malformations, and the amount of amniotic fluid.

**Interviewer (00:08:01-8):** So, in such cases, do you prioritize the ultrasound estimation?

**Person 1 (00:08:05-2):** Yes, exactly.

### Maternity manager of Colma1 health and social promotion center

[00:15:20-0](http://localhost:2300/file=C:/Users/USER/Desktop/ENTRETIENS%20PREMATURITE/CSPS-COLMA1/RESPONSABLE%20MATERNITE-COLMA1.mp3time=920000) Interviewer: Maintenant, est- ce qu'il arrive souvent que les femmes viennent avec une estimation par échographie ? [00:15:24-8](http://localhost:2300/file=C:/Users/USER/Desktop/ENTRETIENS%20PREMATURITE/CSPS-COLMA1/RESPONSABLE%20MATERNITE-COLMA1.mp3time=924800)

[00:15:24-8](http://localhost:2300/file=C:/Users/USER/Desktop/ENTRETIENS%20PREMATURITE/CSPS-COLMA1/RESPONSABLE%20MATERNITE-COLMA1.mp3time=924800) Person 1: Oui, beaucoup. [00:15:28-0](http://localhost:2300/file=C:/Users/USER/Desktop/ENTRETIENS%20PREMATURITE/CSPS-COLMA1/RESPONSABLE%20MATERNITE-COLMA1.mp3time=928000)

[00:15:28-0](http://localhost:2300/file=C:/Users/USER/Desktop/ENTRETIENS%20PREMATURITE/CSPS-COLMA1/RESPONSABLE%20MATERNITE-COLMA1.mp3time=928000) Interviewer: Dans ce cas, quelle estimation aura la priorité ? [00:15:31-0](http://localhost:2300/file=C:/Users/USER/Desktop/ENTRETIENS%20PREMATURITE/CSPS-COLMA1/RESPONSABLE%20MATERNITE-COLMA1.mp3time=931000)

[00:15:31-0](http://localhost:2300/file=C:/Users/USER/Desktop/ENTRETIENS%20PREMATURITE/CSPS-COLMA1/RESPONSABLE%20MATERNITE-COLMA1.mp3time=931000) Person 1: Lorsque c'est une échographie, c'est ça que je vous ai dit, si l'échographie est faite au premier trimestre,chez nous içi au nous mettons l'accent sur l'échographie sii c'est au premier trimestre, une fois que nous dépassons le premier trimestre, le reste là, parce que je ne fais pas d' échographie, mais le petit cours que j'ai eu à l'école, parce que les mesures qui se font, c'est par rapport aux perimetre cranien,fémur, ça dépend. Quelqu'un peut ne pas avoir mon âge, mais qui est plus en forme que moi. Donc, lorsqu'il s'est fait au premier trimestre, on met l'accent vraiment sur l'échographie au premier trimestre, parce que même les femmes instruites peuvent penser que c'est peut- être la grossesse à cinq mois, la date de ces dernieres règles c'etait en juillet, elle va venir vous dire août et vous allez voir. [00:16:30-9](http://localhost:2300/file=C:/Users/USER/Desktop/ENTRETIENS%20PREMATURITE/CSPS-COLMA1/RESPONSABLE%20MATERNITE-COLMA1.mp3time=990900)

00:15:20-0 Interviewer: Maintenant, est-ce qu'il arrive souvent que les femmes viennent avec une estimation par échographie ? 00:15:24-8

00:15:24-8 Person 1: Oui, beaucoup. 00:15:28-0

00:15:28-0 Interviewer: Dans ce cas, quelle estimation aura la priorité ? 00:15:31-0

00:15:31-0 Person 1: Lorsque c'est une échographie, c'est ça que je vous ai dit, si l'échographie est faite au premier trimestre, chez nous ici, nous mettons l'accent sur l'échographie si c'est au premier trimestre. Une fois que nous dépassons le premier trimestre, le reste là, parce que je ne fais pas d'échographie, mais le petit cours que j'ai eu à l'école, parce que les mesures qui se font, c'est par rapport aux périmètres crânien, fémur, ça dépend. Quelqu'un peut ne pas avoir mon âge, mais qui est plus en forme que moi. Donc, lorsque c'est fait au premier trimestre, on met l'accent vraiment sur l'échographie au premier trimestre, parce que même les femmes instruites peuvent penser que c'est peut-être la grossesse à cinq mois, la date de ces dernières règles c'était en juillet, elle va venir vous dire août et vous allez voir. 00:16:30-9

**Interviewer (00:15:20-0):** How often do women come to your facility with an ultrasound estimate?

**Person 1 (00:15:24-8):** Yes, quite a lot.

**Interviewer (00:15:28-0):** In such cases, which estimate do you prioritize?

**Person 1 (00:15:31-0):** We prioritize the ultrasound, especially if it's done in the first trimester. After the first trimester, it becomes a bit more complex. From what I learned, ultrasound measurements are based on the cranial and femoral perimeters, which can vary. For example, a younger person may have different measurements compared to someone older. So, a strong focus is placed on the first trimester ultrasound, as it's crucial for accurate dating.

### Focus group of Farakan health and social promotion center

[00:18:01-2](http://localhost:2300/file=C:/Users/USER/Desktop/ENTRETIENS%20PREMATURITE/CSPS_FARAKAN/FOCUS_GROUPE-CSPS_FARAKAN.MP3time=1081200) Interviewer: D'accord, est ce que les femmes enceintes viennent souvent avec une estimation par échographie ? Les femmes viennent souvent avec une estimation par échographie? [00:18:19-6](http://localhost:2300/file=C:/Users/USER/Desktop/ENTRETIENS%20PREMATURITE/CSPS_FARAKAN/FOCUS_GROUPE-CSPS_FARAKAN.MP3time=1099600)

[00:18:19-6](http://localhost:2300/file=C:/Users/USER/Desktop/ENTRETIENS%20PREMATURITE/CSPS_FARAKAN/FOCUS_GROUPE-CSPS_FARAKAN.MP3time=1099600) Person 2: Ah ça c'est rare. Mais la plupart elles viennent d'abord nous voir expliquer que ses règles sont coupées et là maintenant nous voyons s'il y a necessité de demander une échographie, nous leur demandons l'échographie. [00:18:40-7](http://localhost:2300/file=C:/Users/USER/Desktop/ENTRETIENS%20PREMATURITE/CSPS_FARAKAN/FOCUS_GROUPE-CSPS_FARAKAN.MP3time=1120700)

[00:18:40-7](http://localhost:2300/file=C:/Users/USER/Desktop/ENTRETIENS%20PREMATURITE/CSPS_FARAKAN/FOCUS_GROUPE-CSPS_FARAKAN.MP3time=1120700) Person 4: Il y a des femmes aussi qui viennent d'ailleurs et en venant elles amènent leur échographie en main , on prend ,on interprète et nous, on fait notre travail. [00:18:53-3](http://localhost:2300/file=C:/Users/USER/Desktop/ENTRETIENS%20PREMATURITE/CSPS_FARAKAN/FOCUS_GROUPE-CSPS_FARAKAN.MP3time=1133300)

[00:18:53-3](http://localhost:2300/file=C:/Users/USER/Desktop/ENTRETIENS%20PREMATURITE/CSPS_FARAKAN/FOCUS_GROUPE-CSPS_FARAKAN.MP3time=1133300) Person 8: La plupart des échographies, c'est nous qui les proposons. Sinon c'est rare qu'une femme arrive avec son échographie et dire que je ne connais pas la date de la grossesse donc je vais aller faire une échographie, c'est nous, qui les proposons chaque fois l'échographie, parce qu'elles ne connaissent pas leur date des dernieres règles. [00:19:10-5](http://localhost:2300/file=C:/Users/USER/Desktop/ENTRETIENS%20PREMATURITE/CSPS_FARAKAN/FOCUS_GROUPE-CSPS_FARAKAN.MP3time=1150500)

[00:19:10-5](http://localhost:2300/file=C:/Users/USER/Desktop/ENTRETIENS%20PREMATURITE/CSPS_FARAKAN/FOCUS_GROUPE-CSPS_FARAKAN.MP3time=1150500) Person 3: Le plus souvent c'est nous,nous demandons des Échographies mais en en ville Ici la plupart,les femmes mêmes sont exigeantes se sont des convenances personnelles. Elles viennent nous voir," Je veux faire échographie".Donc, sans discuter, nous aussi nous délivrons le bulletin. [00:19:36-2](http://localhost:2300/file=C:/Users/USER/Desktop/ENTRETIENS%20PREMATURITE/CSPS_FARAKAN/FOCUS_GROUPE-CSPS_FARAKAN.MP3time=1176200)

[00:19:36-2](http://localhost:2300/file=C:/Users/USER/Desktop/ENTRETIENS%20PREMATURITE/CSPS_FARAKAN/FOCUS_GROUPE-CSPS_FARAKAN.MP3time=1176200) Interviewer: Maintenant au cas où elle viendrait avec une échographie. Quelle est l'estimation qui aura la priorité à votre niveau ? [00:19:49-9](http://localhost:2300/file=C:/Users/USER/Desktop/ENTRETIENS%20PREMATURITE/CSPS_FARAKAN/FOCUS_GROUPE-CSPS_FARAKAN.MP3time=1189900)

[00:19:49-9](http://localhost:2300/file=C:/Users/USER/Desktop/ENTRETIENS%20PREMATURITE/CSPS_FARAKAN/FOCUS_GROUPE-CSPS_FARAKAN.MP3time=1189900) Person 7: Bon la catégorie des femmes qui le font, se sont les femmes instruites, le plus souvent, elles peuvent aller en consultation dans des cliniques et s'est à l'issu de ses consultations qu'elles font ses échographies, qu'elles reviennent par la suite pour se faire suivre ici.Nous n'avons pas le choix, nous encaissons ça comme ça. [00:20:16-6](http://localhost:2300/file=C:/Users/USER/Desktop/ENTRETIENS%20PREMATURITE/CSPS_FARAKAN/FOCUS_GROUPE-CSPS_FARAKAN.MP3time=1216600)

00:18:01-2 Interviewer: D'accord, est-ce que les femmes enceintes viennent souvent avec une estimation par échographie ? Les femmes viennent souvent avec une estimation par échographie ?

00:18:19-6 Person 2: Ah ça, c'est rare. Mais la plupart elles viennent d'abord nous voir expliquer que ses règles sont coupées et là maintenant nous voyons s'il y a nécessité de demander une échographie, nous leur demandons l'échographie.

00:18:40-7 Person 4: Il y a des femmes aussi qui viennent d'ailleurs et en venant elles amènent leur échographie en main, on prend, on interprète et nous, on fait notre travail.

00:18:53-3 Person 8: La plupart des échographies, c'est nous qui les proposons. Sinon c'est rare qu'une femme arrive avec son échographie et dire que je ne connais pas la date de la grossesse donc je vais aller faire une échographie, c'est nous, qui les proposons chaque fois l'échographie, parce qu'elles ne connaissent pas leur date des dernières règles.

00:19:10-5 Person 3: Le plus souvent c'est nous, nous demandons des échographies mais en ville ici la plupart, les femmes mêmes sont exigeantes, c'est des convenances personnelles. Elles viennent nous voir, "Je veux faire échographie". Donc, sans discuter, nous aussi nous délivrons le bulletin.

00:19:36-2 Interviewer: Maintenant, au cas où elle viendrait avec une échographie. Quelle est l'estimation qui aura la priorité à votre niveau ?

00:19:49-9 Person 7: Bon, la catégorie des femmes qui le font, ce sont les femmes instruites, le plus souvent, elles peuvent aller en consultation dans des cliniques et c'est à l'issue de ces consultations qu'elles font ces échographies, qu'elles reviennent par la suite pour se faire suivre ici. Nous n'avons pas le choix, nous encaissons ça comme ça.

**Interviewer (00:18:01-2):** Do pregnant women often come with an ultrasound estimate?

**Person 2 (00:18:19-6):** It's rare. They usually come to us first when their periods stop, and then we might request an ultrasound.

**Person 4 (00:18:40-7):** Some women come from other places with their ultrasound, which we then interpret and use.

**Person 8 (00:18:53-3):** We usually provide most of the ultrasounds. It's uncommon for women to come with their own ultrasound unless they don't know their LMP date.

**Person 3 (00:19:10-5):** Most often, we request ultrasounds. However, in urban areas, many women request them for personal convenience.

**Interviewer (00:19:36-2):** If a woman comes with an ultrasound, which estimate takes priority?

**Person 7 (00:19:49-9):** Educated women often get ultrasounds after clinic consultations and bring them to us. We accept and use these results.

### Major of Farakan health and social promotion center

[00:06:24-3](http://localhost:2300/file=C:/Users/USER/Desktop/ENTRETIENS%20PREMATURITE/CSPS_FARAKAN/MAJOR%20FARAKAN.MP3time=384300) Interviewer: Est ce que les femmes viennent parfois avec une estimation par échographie? [00:06:29-0](http://localhost:2300/file=C:/Users/USER/Desktop/ENTRETIENS%20PREMATURITE/CSPS_FARAKAN/MAJOR%20FARAKAN.MP3time=389000)

[00:06:29-0](http://localhost:2300/file=C:/Users/USER/Desktop/ENTRETIENS%20PREMATURITE/CSPS_FARAKAN/MAJOR%20FARAKAN.MP3time=389000) Person 1: Puisque l'échographie ce sont les agents de santé qui demandent, elles ne se lèvent pas d'elles même pour aller faire. C'est à partir de la CPN, on propose à la femme s'elle accepte, nous faisons le bulletin pour l'échographie. [00:06:54-1](http://localhost:2300/file=C:/Users/USER/Desktop/ENTRETIENS%20PREMATURITE/CSPS_FARAKAN/MAJOR%20FARAKAN.MP3time=414100)

[00:06:54-1](http://localhost:2300/file=C:/Users/USER/Desktop/ENTRETIENS%20PREMATURITE/CSPS_FARAKAN/MAJOR%20FARAKAN.MP3time=414100) Interviewer: Lorsqu'elle apporte l'échographie, quelle estimation aura la priorité par rapport à la datation de la grossesse? [00:07:19-1](http://localhost:2300/file=C:/Users/USER/Desktop/ENTRETIENS%20PREMATURITE/CSPS_FARAKAN/MAJOR%20FARAKAN.MP3time=439100)

[00:07:19-1](http://localhost:2300/file=C:/Users/USER/Desktop/ENTRETIENS%20PREMATURITE/CSPS_FARAKAN/MAJOR%20FARAKAN.MP3time=439100) Person 1: Bon, nous prenons l'échographie car c'est plus fiable. [00:07:25-6](http://localhost:2300/file=C:/Users/USER/Desktop/ENTRETIENS%20PREMATURITE/CSPS_FARAKAN/MAJOR%20FARAKAN.MP3time=445600)

00:06:24-3 Interviewer: Est-ce que les femmes viennent parfois avec une estimation par échographie ? 00:06:29-0

00:06:29-0 Person 1: Puisque l'échographie ce sont les agents de santé qui demandent, elles ne se lèvent pas d'elles-mêmes pour aller faire. C'est à partir de la CPN, on propose à la femme si elle accepte, nous faisons le bulletin pour l'échographie. 00:06:54-1

00:06:54-1 Interviewer: Lorsqu'elle apporte l'échographie, quelle estimation aura la priorité par rapport à la datation de la grossesse ? 00:07:19-1

00:07:19-1 Person 1: Bon, nous prenons l'échographie car c'est plus fiable. 00:07:25-6

**Interviewer (00:06:24-3):** Do women often come to your facility with an ultrasound estimate?

**Person 1 (00:06:29-0):** Usually, it's the health workers who suggest an ultrasound, not the women themselves. During prenatal visits (CPNs), we propose an ultrasound to the woman, and if she agrees, we arrange it for her.

**Interviewer (00:06:54-1):** When a woman brings in an ultrasound, which estimate do you prioritize for dating the pregnancy?

**Person 1 (00:07:19-1):** We prioritize the ultrasound because it's more reliable.

### Maternity manager of Farakan health and social promotion center

[00:09:16-6](http://localhost:2300/file=C:/Users/USER/Desktop/ENTRETIENS%20PREMATURITE/CSPS_FARAKAN/RESPONSABLE%20MATERNITE%20FARAKAN.MP3time=556600) Interviewer: Est-ce que, au sein de votre centre de santé, les femmes viennent souvent avec une estimation par échographie? [00:09:23-3](http://localhost:2300/file=C:/Users/USER/Desktop/ENTRETIENS%20PREMATURITE/CSPS_FARAKAN/RESPONSABLE%20MATERNITE%20FARAKAN.MP3time=563300)

[00:09:23-3](http://localhost:2300/file=C:/Users/USER/Desktop/ENTRETIENS%20PREMATURITE/CSPS_FARAKAN/RESPONSABLE%20MATERNITE%20FARAKAN.MP3time=563300) Person 1: Oui elles viennent, mais en fait le problème qui est là aussi, il faut que les résultats de l'échographie soient tôt, en tout cas, c'est ce que généralement les gynécologues disent.Parce que, une échographie du premier trimestre pour la date probable de l'accouchement par rapport à une échographie, qui par exemple la grossesse part de 5e mois, 6e mois, on va donner une date de naissance qui ne sera pas une date exacte. [00:09:51-7](http://localhost:2300/file=C:/Users/USER/Desktop/ENTRETIENS%20PREMATURITE/CSPS_FARAKAN/RESPONSABLE%20MATERNITE%20FARAKAN.MP3time=591700)

[00:09:51-7](http://localhost:2300/file=C:/Users/USER/Desktop/ENTRETIENS%20PREMATURITE/CSPS_FARAKAN/RESPONSABLE%20MATERNITE%20FARAKAN.MP3time=591700) Interviewer: Donc en ce moment, même s'elles qui viennent avec une estimation par échographie, l'échographie n'est pas tellement priorisée? [00:10:01-7](http://localhost:2300/file=C:/Users/USER/Desktop/ENTRETIENS%20PREMATURITE/CSPS_FARAKAN/RESPONSABLE%20MATERNITE%20FARAKAN.MP3time=601700)

[00:10:01-7](http://localhost:2300/file=C:/Users/USER/Desktop/ENTRETIENS%20PREMATURITE/CSPS_FARAKAN/RESPONSABLE%20MATERNITE%20FARAKAN.MP3time=601700) Person 1: Non, en tout cas pas trop comme ça, mais si c'est au tout début là, on va prioriser. Maintenant, je ne sais pas s'il y a des changements.C'est selon en tout cas ce que les gynécologues disent, ceux qui font les échographies. Ils disent que l'échographie au premier trimestre donne une date probable d'accouchement plus fiable , par rapport à une qui vient faire une échographie pour la première fois à 5 mois, 6 mois de grossesse. [00:10:30-2](http://localhost:2300/file=C:/Users/USER/Desktop/ENTRETIENS%20PREMATURITE/CSPS_FARAKAN/RESPONSABLE%20MATERNITE%20FARAKAN.MP3time=630200)

00:09:16-6 Interviewer: Est-ce que, au sein de votre centre de santé, les femmes viennent souvent avec une estimation par échographie ? 00:09:23-3

00:09:23-3 Person 1: Oui, elles viennent, mais en fait le problème qui est là aussi, il faut que les résultats de l'échographie soient tôt, en tout cas, c'est ce que généralement les gynécologues disent. Parce que, une échographie du premier trimestre pour la date probable de l'accouchement par rapport à une échographie, qui, par exemple, la grossesse part de 5e mois, 6e mois, on va donner une date de naissance qui ne sera pas une date exacte. 00:09:51-7

00:09:51-7 Interviewer: Donc en ce moment, même si elles viennent avec une estimation par échographie, l'échographie n'est pas tellement priorisée ? 00:10:01-7

00:10:01-7 Person 1: Non, en tout cas pas trop comme ça, mais si c'est au tout début là, on va prioriser. Maintenant, je ne sais pas s'il y a des changements. C'est selon en tout cas ce que les gynécologues disent, ceux qui font les échographies. Ils disent que l'échographie au premier trimestre donne une date probable d'accouchement plus fiable, par rapport à une qui vient faire une échographie pour la première fois à 5 mois, 6 mois de grossesse. 00:10:30-2

**Interviewer (00:09:16-6):** Do women often come to your health center with an ultrasound estimate?

**Person 1 (00:09:23-3):** Yes, they do come with ultrasound results. However, the timing of the ultrasound is crucial. Gynecologists generally recommend an early, first trimester ultrasound for a more accurate probable date of delivery. Ultrasounds performed later, like in the 5th or 6th month of pregnancy, provide less exact birth dates.

**Interviewer (00:09:51-7):** So, even if they come with an ultrasound estimate, is it not always prioritized?

**Person 1 (00:10:01-7):** If the ultrasound is from the very beginning of the pregnancy, we prioritize it. Gynecologists say that an ultrasound in the first trimester gives a more reliable probable date of delivery compared to one done for the first time at 5 or 6 months of pregnancy.

## 3.8 Priority method if the date of the last period, fundal height and ultrasound are discordant

### Focus group of Accart-ville urban medical center

[00:10:09-4](http://localhost:2300/file=C:/Users/USER/Desktop/ENTRETIENS) Interviewer: maintenant dans le cas où les estimations d'âge gestationnel par la date des dernières règles, la hauteur utérine et l'échographie sont différentes, quelle estimation décidez-vous d'utiliser et pourquoi ? s'il n' y a pas de concordance entre toutes ses methodes de datation de la grossesse? [00:10:35-4](http://localhost:2300/file=C:/Users/USER/Desktop/ENTRETIENS%20PREMATURITE/CSPS-ACCART-VILLE/FOCUS%20GROUPE%20ACCART-VILLE-BON.mp3time=635400)

[00:10:35-4](http://localhost:2300/file=C:/Users/USER/Desktop/ENTRETIENS%20PREMATURITE/CSPS-ACCART-VILLE/FOCUS%20GROUPE%20ACCART-VILLE-BON.mp3time=635400)Person 2: il faut parler [00:10:43-3](http://localhost:2300/file=C:/Users/USER/Desktop/ENTRETIE)

[00:10:43-3](http://localhost:2300/file=C:/Users/USER/Desktop/ENTRETIENS%20PREMATURITE/CSPS-ACCART-VILLE/FOCUS%20GROUPE%20ACCART-VILLE-BON.mp3time=643300) Person 1: quand il n'y a pas de concordance dans les deux examens, nous referons à l'echelon superieur(rire) on envoie vers l'ASOG pour qu'il puisse aussi investiguer à son niveau. [00:11:02-9](http://localhost:2300/file=C:/Users/USER/Desktop/ENTRETIENS)

[00:11:02-9](http://localhost:2300/file=C:/Users/USER/Desktop/ENTRETIENS%20PREMATURITE/CSPS-ACCART-VILLE/FOCUS%20GROUPE%20ACCART-VILLE-BON.mp3time=662900) Interviewer: quand vous parler d'echelon, vous faites allusion à quoi? [00:11:03-5](http://localhost:2300/file=C:/Users/USER/Desktop/ENTRETIENS%20PREMATURITE/CSPS-ACCART-VILLE/FOCUS%20GROUPE%20ACCART-VILLE-BON.mp3time=663500)

[00:11:03-5](http://localhost:23)Person 1: à un agent plus superieur que nous donc la sage femme doit referer à l'ASOG. [00:11:26-8](http://localhost:2300/file=C)

[00:11:26-8](http://localhost:2300/file=C:/Users/USER/Desktop/ENTRETIENS)Person 6: quand vous dites que les deux examens ne concordent pas, l'echographie et l'examen clinique ne concordent pas. cela arrive lorsque l'echographie n'est pas recente et dans ce cas nous redandons encore une echographie pour voire s'il y a un soucis [00:11:45-6](http://localhost:2300/file=C:/Users/USER/Desktop/ENTRETIENS%20PREMATURITE/CSPS-ACCART-VILLE/FOCUS%20GROUPE%20ACCART-VILLE-BON.mp3time=705600)

[00:11:45-6](http://localhost:2300/file=C:/Users/USER/Desktop/ENTRETIENS%20PREMATURITE/CSPS-ACCART-VILLE/FOCUS%20GROUPE%20ACCART-VILLE-BON.mp3time=705600) Interviewer: dans ce cas qu'elle estimation aura la priorité? [00:12:00-5](http://localhost:2300/f)

[00:12:00-5](http://localhost:2300/file=C:/Users/USER/Desktop/ENTRETIENS)Person 2: la plus part du temps quand cela arrive c'est dû au fait que c'est une ancienne echographie donc nous redamandons une echographie pour voire [00:12:18-9](http://localhost:2300/file=C:/Users/USER/Desktop/ENTRETIENS)

00:10:09-4 Interviewer: Maintenant, dans le cas où les estimations d'âge gestationnel par la date des dernières règles, la hauteur utérine et l'échographie sont différentes, quelle estimation décidez-vous d'utiliser et pourquoi ? S'il n'y a pas de concordance entre toutes ces méthodes de datation de la grossesse ?

00:10:35-4 Person 2: Il faut parler.

00:10:43-3 Person 1: Quand il n'y a pas de concordance dans les deux examens, nous référons à l'échelon supérieur (rire), on envoie vers l'ASOG pour qu'il puisse aussi investiguer à son niveau.

00:11:02-9 Interviewer: Quand vous parlez d'échelon, vous faites allusion à quoi ?

00:11:03-5 Person 1: À un agent plus supérieur que nous, donc la sage-femme doit référer à l'ASSOG.

00:11:26-8 Person 6: Quand vous dites que les deux examens ne concordent pas, l'échographie et l'examen clinique ne concordent pas. Cela arrive lorsque l'échographie n'est pas récente et dans ce cas, nous redemandons encore une échographie pour voir s'il y a un souci.

00:11:45-6 Interviewer: Dans ce cas, quelle estimation aura la priorité ?

00:12:00-5 Person 2: La plupart du temps quand cela arrive, c'est dû au fait que c'est une ancienne échographie, donc nous redemandons une échographie pour voir.

**Interviewer (00:10:09-4):** What do you do when there's no agreement between gestational age estimates from the last menstrual date, fundal height, and ultrasound? How do you decide which one to use?

**Person 2 (00:10:35-4):** We need to discuss this further.

**Person 1 (00:10:43-3):** If the two examinations don't match, we escalate the case to a higher level (laughs). We refer it to the ASOG for further investigation.

**Interviewer (00:11:02-9):** When you mention 'scale,' what does that mean?

**Person 1 (00:11:03-5):** We mean consulting someone more senior than us. For example, a midwife would refer the case to ASOG.

**Person 6 (00:11:26-8):** When the ultrasound and clinical examination don't align, often it's because the ultrasound is outdated. In such cases, we request a new ultrasound to check for any issues.

**Interviewer (00:11:45-6):** In these situations, which estimate is given priority?

**Person 2 (00:12:00-5):** Usually, when there's a discrepancy, it's due to the ultrasound being old. Therefore, we order a new ultrasound to determine the next steps.

### Manager of Accart-ville urban medical center

[00:08:09-1](http://localhost:2300/file=C:/Users/USER/Desktop/ENTRETIENS%20PREMATURITE/CSPS-ACCART-VILLE/RESPONSABLE%20CMU%20ACCART-VILLE.MP3time=489100)Interviewer: maintenant au cas où il y a une discordance entre les estimations échographiques, la hauteur utérine et les autres méthodes de datation. Quelle est la méthode qui est priorisée? [00:08:23-9](http://localhost:2300/file=C:/Users/USER/Desktop/ENTRETIENS%20PREMATURITE/CSPS-ACCART-VILLE/RESPONSABLE%20CMU%20ACCART-VILLE.MP3time=503900)

[00:08:23-9](http://localhost:2300/file=C:/Users/USER/Desktop/ENTRETIENS%20PREMATURITE/CSPS-ACCART-VILLE/RESPONSABLE%20CMU%20ACCART-VILLE.MP3time=503900)Person 1: puisqu'à ma connaissance on dit d'utiliser une échographie précoce,c'est ce qui est la meilleure estimation. Généralement quand on fait les staffs, c'est ce que je leurs dit. Si on a uneéchographie inferieure à douze semaines c'est la meilleure. Mais si on a pas ça, on peut maintenant se baser sur l'âge chronologique. A partir de là maintenant, si on n'a pas cela, on peut aller avec l'examen clinique. [00:08:51-2](http://localhost:2300/file=C:/Users/USER/Desktop/ENTRETIENS%20PREMATURITE/CSPS-ACCART-VILLE/RESPONSABLE%20CMU%20ACCART-VILLE.MP3time=531200)

00:08:09-1 Interviewer: Maintenant, au cas où il y a une discordance entre les estimations échographiques, la hauteur utérine et les autres méthodes de datation, quelle est la méthode qui est priorisée ?

00:08:23-9 Person 1: Puisqu'à ma connaissance, on dit d'utiliser une échographie précoce, c'est ce qui est la meilleure estimation. Généralement, quand on fait les staffs, c'est ce que je leur dis. Si on a une échographie inférieure à douze semaines, c'est la meilleure. Mais si on n'a pas ça, on peut maintenant se baser sur l'âge chronologique. À partir de là maintenant, si on n'a pas cela, on peut aller avec l'examen clinique.

**Interviewer (00:08:09-1):** In cases where there's a discrepancy between ultrasound estimates, fundal height, and other dating methods, which method do you prioritize?

**Person 1 (00:08:23-9):** We consider an early ultrasound, performed before twelve weeks, to be the most accurate. That's the guideline I usually give to our staff. If we have an ultrasound from within the first twelve weeks, we prioritize it. If that's not available, we then consider the chronological age. Lacking both, we resort to clinical examination.

### Maternity manager of Accart-ville urban medical center

[00:09:51-1](http://localhost:2300/file=C:/Users/USER/Desktop/ENTRETIENS%20PREMATURITE/CSPS-ACCART-VILLE/RESPONSABLE%20MATERNITE%20CMU%20ACCART-VILLE.MP3time=591100) Interviewer: maintenant dans le cas où les estimations d'âge gestationnel par la date des dernières règles, la hauteur utérine et l'échographie sont différentes, quelle estimation décidez-vous d'utiliser et pourquoi? [00:10:04-7](http://localhost:2300/file=C:/Users/USER/Desktop/ENTRETIENS%20PREMATURITE/CSPS-ACCART-VILLE/RESPONSABLE%20MATERNITE%20CMU%20ACCART-VILLE.MP3time=604700)

[00:10:04-7](http://localhost:2300/file=C:/Users/USER/Desktop/ENTRETIENS%20PREMATURITE/CSPS-ACCART-VILLE/RESPONSABLE%20MATERNITE%20CMU%20ACCART-VILLE.MP3time=604700) Person 1: effectivement il arrive qu'on ait des discordances cela fait partie du suivi , la discordance peut nous permettre de poser des diagnostiques. par exemple les grossesses gemellaires oubien les hydrmnios. maintenant s'il y a une discordance on part sur la base de la date des dernières règles si cela est connue [00:10:27-4](http://localhost:2300/file=C:/Users/USER/Desktop/ENTRETIENS%20PREMATURITE/CSPS-ACCART-VILLE/RESPONSABLE%20MATERNITE%20CMU%20ACCART-VILLE.MP3time=627400)

[00:10:27-4](http://localhost:2300/file=C:/Users/USER/Desktop/ENTRETIENS%20PREMATURITE/CSPS-ACCART-VILLE/RESPONSABLE%20MATERNITE%20CMU%20ACCART-VILLE.MP3time=627400) Interviewer: Pourquoi? [00:10:28-4](http://localhost:2300/file=C:/Users/USER/Desktop/ENTRETIENS%20PREMATURITE/CSPS-ACCART-VILLE/RESPONSABLE%20MATERNITE%20CMU%20ACCART-VILLE.MP3time=628400)

[00:10:28-4](http://localhost:2300/file=C:/Users/USER/Desktop/ENTRETIENS%20PREMATURITE/CSPS-ACCART-VILLE/RESPONSABLE%20MATERNITE%20CMU%20ACCART-VILLE.MP3time=628400) Person 1: parce que c'est le moyen le plus efficace, comme les autres se sont des mesures donc ces mesures peuvent varier d'une personne à une autre, on peut ne avoir les mêmes mesures ça depend aussi du physique de la personne si la personne est obèse , si la personne a une parroi abdominale epaisse il est difficile aussi de mesurer, même l'échographie aussi varie d'un échographe à un autre voilà pourquoi on priorise la date des dernières règles. [00:11:04-3](http://localhost:2300/file=C:/Users/USER/Desktop/ENTRETIENS%20PREMATURITE/CSPS-ACCART-VILLE/RESPONSABLE%20MATERNITE%20CMU%20ACCART-VILLE.MP3time=664300)

00:09:51-1 Interviewer: Maintenant, dans le cas où les estimations d'âge gestationnel par la date des dernières règles, la hauteur utérine et l'échographie sont différentes, quelle estimation décidez-vous d'utiliser et pourquoi ? 00:10:04-7

00:10:04-7 Person 1: Effectivement, il arrive qu'on ait des discordances, cela fait partie du suivi, la discordance peut nous permettre de poser des diagnostics, par exemple les grossesses gémellaires ou bien les hydramnios. Maintenant, s'il y a une discordance, on part sur la base de la date des dernières règles si cela est connu. 00:10:27-4

00:10:27-4 Interviewer: Pourquoi ? 00:10:28-4

00:10:28-4 Person 1: Parce que c'est le moyen le plus efficace, comme les autres sont des mesures, donc ces mesures peuvent varier d'une personne à une autre, on peut ne pas avoir les mêmes mesures, ça dépend aussi du physique de la personne si la personne est obèse, si la personne a une paroi abdominale épaisse, il est difficile aussi de mesurer, même l'échographie aussi varie d'un échographe à un autre, voilà pourquoi on priorise la date des dernières règles. 00:11:04-3

**Interviewer (00:09:51-1):** If there’s a difference in gestational age estimates based on the last menstrual date, fundal height, and ultrasound, which do you choose and why?

**Person 1 (00:10:04-7):** Discrepancies can occur and can be helpful in diagnosing conditions like twin pregnancies or polyhydramnios. In such cases, we start with the last menstrual period if it's known.

**Interviewer (00:10:27-4):** Why is that?

**Person 1 (00:10:28-4):** The last menstrual period is the most effective way because other methods involve measurements, which can vary. For example, measurements may differ based on an individual’s physique. In obese individuals or those with a thick abdominal wall, it's difficult to measure accurately. Even ultrasounds can vary depending on the machine used. That's why we prioritize the date of the last menstrual period.

### Focus group of Do medical center with surgical branch

[00:11:29-3](http://localhost:2300/file=C:/Users/USER/Desktop/ENTRETIENS%20PREMATURITE/CMA-DO/FOCUS%20GROUPE%20CMA-DO.mp3time=689300) Interviewer: Dans le cas ou les differentes methodes de datation de la grossesse sont incohérentes, par exemple la hauteur uterine, la datation par échographie, comment est ce que vous procedez et pourquoi? S'il n' y a pas de concordance entre les differentes methodes de datation, comment est qu'à votre niveau ici, vous procedez? [00:11:55-0](http://localhost:2300/file=C:/Users/USER/Desktop/ENTRETIENS%20PREMATURITE/CMA-DO/FOCUS%20GROUPE%20CMA-DO.mp3time=715000)

[00:11:55-0](http://localhost:2300/file=C:/Users/USER/Desktop/ENTRETIENS%20PREMATURITE/CMA-DO/FOCUS%20GROUPE%20CMA-DO.mp3time=715000) Person 4: C'est à dire, ce que la femme dit, et ce que l'échographie dit et puis ce que le gestogramme dit et ce que l'examen clinique donne, et lorsqu'il y a une discordance entre toutes ses methodes. Bon, on va se referer à ce que la femme dit parce que c'est elle qui porte la grossesse et je pense que, même s'elle s'est trompée, bon, au moins on se dit que ça vient d'elle et ce qui ressort de l'examen clinique, ce sont ses deux élemnts que nous priorisons. [00:12:44-6](http://localhost:2300/file=C:/Users/USER/Desktop/ENTRETIENS%20PREMATURITE/CMA-DO/FOCUS%20GROUPE%20CMA-DO.mp3time=764600)

00:11:29-3 Interviewer: Dans le cas où les différentes méthodes de datation de la grossesse sont incohérentes, par exemple la hauteur utérine, la datation par échographie, comment est-ce que vous procédez et pourquoi ? S'il n'y a pas de concordance entre les différentes méthodes de datation, comment est-ce qu'à votre niveau ici, vous procédez ?

00:11:55-0 Person 4: C'est à dire, ce que la femme dit, et ce que l'échographie dit et puis ce que le gestogramme dit et ce que l'examen clinique donne, et lorsqu'il y a une discordance entre toutes ces méthodes. Bon, on va se référer à ce que la femme dit parce que c'est elle qui porte la grossesse et je pense que, même si elle s'est trompée, bon, au moins on se dit que ça vient d'elle et ce qui ressort de l'examen clinique, ce sont ces deux éléments que nous priorisons.

**Interviewer (00:11:29-3):** When different pregnancy dating methods, like fundal height and ultrasound, are inconsistent, how do you handle it? If there's no agreement between these methods, what's your approach?

**Person 4 (00:11:55-0):** In cases of discrepancy between what the woman reports, the ultrasound, the gestogram, and the clinical examination, we prioritize the woman's account. We believe it's important to trust the woman carrying the pregnancy. Even if there's a chance she might be mistaken, we value her input along with the results of the clinical examination. These are the two elements we focus on the most.

### Gynecologist 1

[00:08:00-0](http://localhost:2300/file=C:/Users/USER/Desktop/ENTRETIENS%20PREMATURITE/CMA-DO/GENYCOLOGUE%2001.mp3time=480000) Interviewer: Dans le cas où il y a un décalage entre les différentes méthodes d'estimation, avec l'échographie, la hauteur utérine, quelle est l'estimation qui aura la priorité ? [00:08:10-8](http://localhost:2300/file=C:/Users/USER/Desktop/ENTRETIENS%20PREMATURITE/CMA-DO/GENYCOLOGUE%2001.mp3time=490800)

[00:08:10-8](http://localhost:2300/file=C:/Users/USER/Desktop/ENTRETIENS%20PREMATURITE/CMA-DO/GENYCOLOGUE%2001.mp3time=490800) Person 1: Non, l'échographie. Nous gardons l'échographie. Parce qu'il y a plus de fiabilité. Oui, il y a plus de fiabilité. [00:08:19-9](http://localhost:2300/file=C:/Users/USER/Desktop/ENTRETIENS%20PREMATURITE/CMA-DO/GENYCOLOGUE%2001.mp3time=499900)

00:08:00-0 Interviewer: Dans le cas où il y a un décalage entre les différentes méthodes d'estimation, avec l'échographie, la hauteur utérine, quelle est l'estimation qui aura la priorité ?

00:08:10-8 Person 1: Non, l'échographie. Nous gardons l'échographie. Parce qu'il y a plus de fiabilité. Oui, il y a plus de fiabilité.

**Interviewer (00:08:00-0):** If there's a discrepancy between different estimation methods, like ultrasound and fundal height, which one do you prioritize?

**Person 1 (00:08:10-8):** We stick with the ultrasound. It’s more reliable.

### Gynecologist 2

[00:09:32-1](http://localhost:2300/file=C:/Users/USER/Desktop/ENTRETIENS%20PREMATURITE/CMA-DO/GENYCOLOGUE%2002.mp3time=572100) Interviewer: Maintenant, dans les cas où les différentes méthodes d'estimation diffèrent, il n y a pas de concordances , comment est-ce que vous procédez ? Sur quelle méthode maintenant vous allez vous basez pour procéder à l'estimation de l'âge gestationnel ? [00:09:43-0](http://localhost:2300/file=C:/Users/USER/Desktop/ENTRETIENS%20PREMATURITE/CMA-DO/GENYCOLOGUE%2002.mp3time=583000)

[00:09:43-0](http://localhost:2300/file=C:/Users/USER/Desktop/ENTRETIENS%20PREMATURITE/CMA-DO/GENYCOLOGUE%2002.mp3time=583000) Person 1: C'est à dire si, on a mesuré la hauteur utérine parce qu'avec la hauteur utérine, il y a déjà beaucoup de biais. Parce qu'en fonction de la corpulence de la patiente, les choses peuvent changer. Donc si on a une femme qui a une forte corpulence, souvent il y a une difficulté pour mesurer correctement la hauteur utérine, ça peut être surestimé. Donc quand vous avez une femme qui est maigre, ça peut être sous-estimé. Donc ça fait que ce n'est pas très fiable à 100%. L'échographie, quand elle est faite dans les trois premiers mois, dans les premiers trimestres, elle est beaucoup plus fiable. En dehors de ces différents éléments-ci, c'est une échographie qui est faite tardivement. Quand la femme arrive, elle est au sixième mois et fait l'échographie, en ce moment l'adaptation n'est pas très précise. C'est une extrapolation. Et à partir de là aussi, la mésure de la hauteur utérine ne permet pas de façon précise de connaître l'âge gestationnel. Donc en fait, on se retrouve dans un flou, donc on est obligé d'utiliser les deux paramètres pour évoluer. [00:10:42-8](http://localhost:2300/file=C:/Users/USER/Desktop/ENTRETIENS%20PREMATURITE/CMA-DO/GENYCOLOGUE%2002.mp3time=642800)

[00:10:42-8](http://localhost:2300/file=C:/Users/USER/Desktop/ENTRETIENS%20PREMATURITE/CMA-DO/GENYCOLOGUE%2002.mp3time=642800) Interviewer: Donc en moment en cas de discordance, vous êtes obligés d'utiliser les deux méthodes pour faire une synthèse. [00:10:48-0](http://localhost:2300/file=C:/Users/USER/Desktop/ENTRETIENS%20PREMATURITE/CMA-DO/GENYCOLOGUE%2002.mp3time=648000)

[00:10:48-0](http://localhost:2300/file=C:/Users/USER/Desktop/ENTRETIENS%20PREMATURITE/CMA-DO/GENYCOLOGUE%2002.mp3time=648000) Person 1: Nous sommes obligés d'utiliser les deux methodes pour faire une synthèse. Ça c'est pour la recherche de plus de fiabilité. [00:10:58-2](http://localhost:2300/file=C:/Users/USER/Desktop/ENTRETIENS%20PREMATURITE/CMA-DO/GENYCOLOGUE%2002.mp3time=658200)

00:09:32-1 Interviewer: Maintenant, dans les cas où les différentes méthodes d'estimation diffèrent, il n'y a pas de concordances, comment est-ce que vous procédez ? Sur quelle méthode maintenant vous allez vous baser pour procéder à l'estimation de l'âge gestationnel ?

00:09:43-0 Person 1: C'est à dire si, on a mesuré la hauteur utérine parce qu'avec la hauteur utérine, il y a déjà beaucoup de biais. Parce qu'en fonction de la corpulence de la patiente, les choses peuvent changer. Donc si on a une femme qui a une forte corpulence, souvent il y a une difficulté pour mesurer correctement la hauteur utérine, ça peut être surestimé. Donc quand vous avez une femme qui est maigre, ça peut être sous-estimé. Donc ça fait que ce n'est pas très fiable à 100%. L'échographie, quand elle est faite dans les trois premiers mois, dans les premiers trimestres, elle est beaucoup plus fiable. En dehors de ces différents éléments-ci, c'est une échographie qui est faite tardivement. Quand la femme arrive, elle est au sixième mois et fait l'échographie, en ce moment l'adaptation n'est pas très précise. C'est une extrapolation. Et à partir de là aussi, la mesure de la hauteur utérine ne permet pas de façon précise de connaître l'âge gestationnel. Donc en fait, on se retrouve dans un flou, donc on est obligé d'utiliser les deux paramètres pour évoluer.

00:10:42-8 Interviewer: Donc en moment en cas de discordance, vous êtes obligés d'utiliser les deux méthodes pour faire une synthèse.

00:10:48-0 Person 1: Nous sommes obligés d'utiliser les deux méthodes pour faire une synthèse. Ça, c'est pour la recherche de plus de fiabilité.

**Interviewer (00:09:32-1):** What about situations where there’s a discrepancy between different estimation methods? How do you resolve that?

**Person 1 (00:09:43-0):** If there’s a mismatch, say, between fundal height and ultrasound, we have to consider several factors. Fundal height can be biased, influenced by a woman’s body size. It might be overestimated in larger-bodied women and underestimated in slimmer women, so it’s not always reliable. An ultrasound in the first trimester is more accurate. However, if an ultrasound is done later, like in the sixth month, it’s less precise for dating. In such cases, neither method alone gives a clear answer, so we combine both to get a more reliable estimate.

**Interviewer (00:10:42-8):** So in case of conflicting results, you synthesize information from both methods?

**Person 1 (00:10:48-0):** Exactly, we use both to synthesize a more reliable estimate.

### Chief physician of Do health district

[00:16:27-1](http://localhost:2300/file=C:/Users/USER/Desktop/ENTRETIENS%20PREMATURITE/CMA-DO/MCD-DO.mp3time=987100) Interviewer: C'est à dire qu'en cas de discordance, vous privilégiez toujours la mesure de la hauteur utérine? [00:16:33-5](http://localhost:2300/file=C:/Users/USER/Desktop/ENTRETIENS%20PREMATURITE/CMA-DO/MCD-DO.mp3time=993500)

[00:16:33-5](http://localhost:2300/file=C:/Users/USER/Desktop/ENTRETIENS%20PREMATURITE/CMA-DO/MCD-DO.mp3time=993500) Person 1: On utilise toujours la hauteur utérine parce que ce n'est pas évident qu'on utilise la H.U. Donc, la hauteur utérine. Maintenant, on essaie de regarder s'il y a des discordances, qu'est-ce qui peut expliquer. Mais maintenant, si on a l'échographie à côté, comme au CMA, une fois qu'il y a c'est comme cela, on peut passer l'échographie dessus pour voir qu'est-ce qui peut expliquer ça. Peut-être que c'est deux enfants même qui sont dedans et puis on est entrain de mal mesurer. Voilà, ça arrive, les grossesses gemellaires. Les pôles peuvent ne pas être bien perçu par quelqu'un. Parce qu'en plus de la hauteur, on touche le ventre, on regarde où il est l'enfant. Donc, il peut y avoir deux enfants, mais celui même qui le touche, il peut passer à côté et penser que c'est un enfant. [00:17:25-2](http://localhost:2300/file=C:/Users/USER/Desktop/ENTRETIENS%20PREMATURITE/CMA-DO/MCD-DO.mp3time=1045200)

00:16:27-1 Interviewer: C'est à dire qu'en cas de discordance, vous privilégiez toujours la mesure de la hauteur utérine ?

00:16:33-5 Person 1: On utilise toujours la hauteur utérine parce que ce n'est pas évident qu'on utilise la H.U. Donc, la hauteur utérine. Maintenant, on essaie de regarder s'il y a des discordances, qu'est-ce qui peut expliquer. Mais maintenant, si on a l'échographie à côté, comme au CMA, une fois qu'il y a c'est comme cela, on peut passer l'échographie dessus pour voir qu'est-ce qui peut expliquer ça. Peut-être que c'est deux enfants même qui sont dedans et puis on est en train de mal mesurer. Voilà, ça arrive, les grossesses gémellaires. Les pôles peuvent ne pas être bien perçus par quelqu'un. Parce qu'en plus de la hauteur, on touche le ventre, on regarde où est l'enfant. Donc, il peut y avoir deux enfants, mais celui même qui le touche, il peut passer à côté et penser que c'est un enfant.

**Interviewer (00:16:27-1):** So, in cases of discrepancy, do you always prioritize fundal height measurements?

**Person 1 (00:16:33-5):** Yes, we typically rely on fundal height as the primary measure. It's not always straightforward, though. When there are discrepancies, we try to understand their causes. For instance, at the CMA, if we suspect a discrepancy, we can use ultrasound to investigate further. Sometimes the discrepancy might be due to a twin pregnancy, which can lead to measurement errors. It's possible that during a physical examination, only one fetus is detected when there are actually two. This is why we not only measure fundal height but also palpate the abdomen to assess the position and number of fetuses.

### Manager for maternity care unit of Do medical center with surgical branch

[00:11:53-4](http://localhost:2300/file=C:/Users/USER/Desktop/ENTRETIENS%20PREMATURITE/CMA-DO/RESPONSABLE%20UNITE%20DE%20SOIN%20CMA-DO.mp3time=713400) Interviewer: Maintenant, au cas où il y a un décalage entre les différentes méthodes de datation, l'échographie, la date des dernières règles, la hauteur uterine, etc. Quelles estimations maintenant? Comment est-ce que vous procédez? [00:12:06-1](http://localhost:2300/file=C:/Users/USER/Desktop/ENTRETIENS%20PREMATURITE/CMA-DO/RESPONSABLE%20UNITE%20DE%20SOIN%20CMA-DO.mp3time=726100)

[00:12:06-1](http://localhost:2300/file=C:/Users/USER/Desktop/ENTRETIENS%20PREMATURITE/CMA-DO/RESPONSABLE%20UNITE%20DE%20SOIN%20CMA-DO.mp3time=726100) Person 1: Moi, on se base plus sur la date des dernières règles. C'est plus fiable. Voilà. Et généralement, vous allez voir si vous avez une idée vraiment sur la date des dernières règles et que vous arrivez à suivre. Bon, la différence n'est pas trop grande avec l'échographie. Surtout l'échographie, quand l'échographie est précoce aussi, on a une estimation vraiment parfaite. Voilà, l'échographie précoce, donc, avant les 24 semaines. [00:12:31-3](http://localhost:2300/file=C:/Users/USER/Desktop/ENTRETIENS%20PREMATURITE/CMA-DO/RESPONSABLE%20UNITE%20DE%20SOIN%20CMA-DO.mp3time=751300)

00:11:53-4 Interviewer: Maintenant, au cas où il y a un décalage entre les différentes méthodes de datation, l'échographie, la date des dernières règles, la hauteur utérine, etc. Quelles estimations maintenant ? Comment est-ce que vous procédez ? 00:12:06-1

00:12:06-1 Person 1: Moi, on se base plus sur la date des dernières règles. C'est plus fiable. Voilà. Et généralement, vous allez voir si vous avez une idée vraiment sur la date des dernières règles et que vous arrivez à suivre. Bon, la différence n'est pas trop grande avec l'échographie. Surtout l'échographie, quand l'échographie est précoce aussi, on a une estimation vraiment parfaite. Voilà, l'échographie précoce, donc, avant les 24 semaines. 00:12:31-3

**Interviewer (00:11:53-4):** If there is a discrepancy between different dating methods, like ultrasound, last period date, and fundal height, how do you proceed?

**Person 1 (00:12:06-1):** I tend to rely more on the last menstrual period date, as it's generally reliable. You'll find that if you have a clear idea of this date and track it, the difference isn’t too significant compared to an early ultrasound. An early ultrasound, especially before 24 weeks, gives a very accurate estimate.

### Focus group of Colma1 health and social promotion center

[00:11:44-8](http://localhost:2300/file=C:/Users/USER/Desktop/ENTRETIENS%20PREMATURITE/CSPS-COLMA1/FOCUS%20GROUPE%20COLMA1.mp3time=704800) Interviewer: Maintenant au cas ou il y a une difference entre les differentes methodes d'estimations de l'âge gestationnel, comment est ce que vous procedez? [00:12:12-1](http://localhost:2300/file=C:/Users/USER/Desktop/ENTRETIENS%20PREMATURITE/CSPS-COLMA1/FOCUS%20GROUPE%20COLMA1.mp3time=732100)

[00:12:12-1](http://localhost:2300/file=C:/Users/USER/Desktop/ENTRETIENS%20PREMATURITE/CSPS-COLMA1/FOCUS%20GROUPE%20COLMA1.mp3time=732100) Person 1: Franchement, on fait plus confiance à l'échographie, parce que d'abord ce que la femme va vous dire est ce vraiment juste? est ce que la date que la dame va donner est vraiment juste? et aussi la HU qui sera prise, comme le predecesseur l'a dit, cela peut être une autre pathologie gémelaire ou hydramnios ou bien oligoamnios encore donc vous n'allez pas trouver la HU exacte. Mais avec l'échographie,on se dit comme c'est avec la machine, ils ont plus accès à l'interieur, au foetus qu'à nous. parce que nous vraiment c'est sur la peau nous partons. donc je me dis qu'avec l'échographie, ils ont plus accès au foetus donc ils ont plus de details. [00:12:54-7](http://localhost:2300/file=C:/Users/USER/Desktop/ENTRETIENS%20PREMATURITE/CSPS-COLMA1/FOCUS%20GROUPE%20COLMA1.mp3time=774700)

00:11:44-8 Interviewer: Maintenant au cas où il y a une différence entre les différentes méthodes d'estimations de l'âge gestationnel, comment est-ce que vous procédez ? 00:12:12-1

00:12:12-1 Person 1: Franchement, on fait plus confiance à l'échographie, parce que d'abord ce que la femme va vous dire est-ce vraiment juste ? Est-ce que la date que la dame va donner est vraiment juste ? Et aussi la HU qui sera prise, comme le prédécesseur l'a dit, cela peut être une autre pathologie gémellaire ou hydramnios ou bien oligoamnios encore donc vous n'allez pas trouver la HU exacte. Mais avec l'échographie, on se dit comme c'est avec la machine, ils ont plus accès à l'intérieur, au fœtus qu'à nous. Parce que nous vraiment c'est sur la peau nous partons. Donc je me dis qu'avec l'échographie, ils ont plus accès au fœtus donc ils ont plus de détails. 00:12:54-7

**Interviewer (00:11:44-8):** How do you proceed if there's a discrepancy between different methods of estimating gestational age?

**Person 1 (00:12:12-1):** We place more trust in the ultrasound. The accuracy of the information provided by the woman, such as her last menstrual period, can be uncertain. Similarly, fundal height can be affected by factors like twin pregnancies or amniotic fluid variations, leading to inaccurate measurements. Ultrasounds give us a closer look at the fetus, providing more detailed information than external measurements.

### Major of Colma1 health and social promotion center

[00:08:06-3](http://localhost:2300/file=C:/Users/USER/Desktop/ENTRETIENS%20PREMATURITE/CSPS-COLMA1/MAJOR%20CSPS%20COLMA1.MP3time=486300) Interviewer: maintenant dans le cas ou il y a une difference ou un decallage entre les differentes methodes d'estimation de l'âge gestationnel, comment est ce que vous procedez? [00:08:17-1](http://localhost:2300/file=C:/Users/USER/Desktop/ENTRETIENS%20PREMATURITE/CSPS-COLMA1/MAJOR%20CSPS%20COLMA1.MP3time=497100)

[00:08:17-1](http://localhost:2300/file=C:/Users/USER/Desktop/ENTRETIENS%20PREMATURITE/CSPS-COLMA1/MAJOR%20CSPS%20COLMA1.MP3time=497100) Person 1: lorsqu'il y a un decallage entre les differentes methodes, c'est l'échographie qui sera priorisée [00:08:53-5](http://localhost:2300/file=C:/Users/USER/Desktop/ENTRETIENS%20PREMATURITE/CSPS-COLMA1/MAJOR%20CSPS%20COLMA1.MP3time=533500)

[00:08:53-5](http://localhost:2300/file=C:/Users/USER/Desktop/ENTRETIENS%20PREMATURITE/CSPS-COLMA1/MAJOR%20CSPS%20COLMA1.MP3time=533500) Interviewer: et pourquoi est que c'est l'échographie qui sera priorisée? [00:08:55-1](http://localhost:2300/file=C:/Users/USER/Desktop/ENTRETIENS%20PREMATURITE/CSPS-COLMA1/MAJOR%20CSPS%20COLMA1.MP3time=535100)

[00:08:55-1](http://localhost:2300/file=C:/Users/USER/Desktop/ENTRETIENS%20PREMATURITE/CSPS-COLMA1/MAJOR%20CSPS%20COLMA1.MP3time=535100) Person 1: parce qu' au moins làbas c'est un peu plus exacte. je ne dis pas qu'il n y a pas d'erreurs làbas aussi puisque ce sont des hommes qui travaillent , mais comme c'est le materiel et l'outil scientifique qu'on utilise, nous avons beaucoup plus confiance à l'échographie.effectivement,en regardant par la methode standard que nous utilisons c'est à dire les affiches que nous avons,oû on regarde l'âge de la grossesse à travers la Hauteur Uterine,il y a beaucoup de facteurs à ce niveau qui peut biaiser par exemple la paroi uterine avec la couche graisseuse et autres cela peut biaiser.nous savons que cela peut arriver.mais l'échographie c'est beaucoup plus fiable. [00:09:41-2](http://localhost:2300/file=C:/Users/USER/Desktop/ENTRETIENS%20PREMATURITE/CSPS-COLMA1/MAJOR%20CSPS%20COLMA1.MP3time=581200)

00:08:06-3 Interviewer: Maintenant, dans le cas où il y a une différence ou un décalage entre les différentes méthodes d'estimation de l'âge gestationnel, comment est-ce que vous procédez ? 00:08:17-1

00:08:17-1 Person 1: Lorsqu'il y a un décalage entre les différentes méthodes, c'est l'échographie qui sera priorisée. 00:08:53-5

00:08:53-5 Interviewer: Et pourquoi est-ce que c'est l'échographie qui sera priorisée ? 00:08:55-1

00:08:55-1 Person 1: Parce qu'au moins là-bas c'est un peu plus exact. Je ne dis pas qu'il n'y a pas d'erreurs là-bas aussi puisque ce sont des hommes qui travaillent, mais comme c'est le matériel et l'outil scientifique qu'on utilise, nous avons beaucoup plus confiance à l'échographie. Effectivement, en regardant par la méthode standard que nous utilisons c'est-à-dire les affiches que nous avons, où on regarde l'âge de la grossesse à travers la Hauteur Utérine, il y a beaucoup de facteurs à ce niveau qui peut biaiser, par exemple la paroi utérine avec la couche graisseuse et autres, cela peut biaiser. Nous savons que cela peut arriver. Mais l'échographie, c'est beaucoup plus fiable. 00:09:41-2

**Interviewer (00:08:06-3):** How do you handle discrepancies between different methods of estimating gestational age?

**Person 1 (00:08:17-1):** When there's a discrepancy, we prioritize the ultrasound.

**Interviewer (00:08:53-5):** Why is ultrasound given priority?

**Person 1 (00:08:55-1):** Because it's generally more accurate. Although there can be errors, since it involves human interpretation, we have more confidence in it as a scientific tool. Standard methods, like estimating based on uterine height using posters, can be influenced by various factors, such as the thickness of the uterine wall. Ultrasound is considered much more reliable.

### Maternity manager of Colma1 health and social promotion center

[00:16:30-9](http://localhost:2300/file=C:/Users/USER/Desktop/ENTRETIENS%20PREMATURITE/CSPS-COLMA1/RESPONSABLE%20MATERNITE-COLMA1.mp3time=990900) Interviewer: maintenant, au cas où il y a un décalage ou un déséquilibre entre les différents méthodes d'estimation, comment est- ce que vous procédez ? S'il n y a pas de concordance entre l'estimation par la date des dernières règles, l'estimation par la hauteur uterine et l' échographie. Si toutes ces estimations sont différentes, Comment est- ce que vous procédez ?[00:16:43-8](http://localhost:2300/file=C:/Users/USER/Desktop/ENTRETIENS%20PREMATURITE/CSPS-COLMA1/RESPONSABLE%20MATERNITE-COLMA1.mp3time=1003800)

[00:16:43-8](http://localhost:2300/file=C:/Users/USER/Desktop/ENTRETIENS%20PREMATURITE/CSPS-COLMA1/RESPONSABLE%20MATERNITE-COLMA1.mp3time=1003800) Person 1: Person 1: Par exemple, la femme vient au premier trimestre. Toi, tu as vu la femme très tôt. Tu as pensé que non, la grossesse est à quatre mois. L' échographie vous dit que la grossesse à deux mois. Moi, je prends l' échographie. Pourquoi ? Je m'explique. Parce que, par exemple, quelqu'un peut venir au premier trimestre, le ventre n'a pas grossi. Tu as mis que c'est le premier trimestre et tu donnes un mois seulement, tu vas remarquer peut- être que non, l'uterus a subitement augmenté de volume. Dans le cas de grossesse gemellaire. Dans ces cas. Et pourtant, l'échographie va aller regarder tout ça dedans. Je préfère l' échographier en cet temps. [00:17:51-9](http://localhost:2300/file=C:/Users/USER/Desktop/ENTRETIENS%20PREMATURITE/CSPS-COLMA1/RESPONSABLE%20MATERNITE-COLMA1.mp3time=1071900)

00:16:30-9 Interviewer: Maintenant, au cas où il y a un décalage ou un déséquilibre entre les différentes méthodes d'estimation, comment est-ce que vous procédez ? S'il n'y a pas de concordance entre l'estimation par la date des dernières règles, l'estimation par la hauteur utérine et l'échographie. Si toutes ces estimations sont différentes, comment est-ce que vous procédez ? 00:16:43-8

00:16:43-8 Person 1: Par exemple, la femme vient au premier trimestre. Toi, tu as vu la femme très tôt. Tu as pensé que non, la grossesse est à quatre mois. L'échographie vous dit que la grossesse à deux mois. Moi, je prends l'échographie. Pourquoi ? Je m'explique. Parce que, par exemple, quelqu'un peut venir au premier trimestre, le ventre n'a pas grossi. Tu as mis que c'est le premier trimestre et tu donnes un mois seulement, tu vas remarquer peut-être que non, l'utérus a subitement augmenté de volume. Dans le cas de grossesse gémellaire. Dans ces cas. Et pourtant, l'échographie va aller regarder tout ça dedans. Je préfère l'échographier en cet temps. 00:17:51-9

**Interviewer (00:16:30-9):** If there is a discrepancy between different estimation methods, like the last menstrual period, fundal height, and ultrasound, how do you handle it?

**Person 1 (00:16:43-8):** Let's say a woman comes in the first trimester, and we initially think the pregnancy is four months along, but the ultrasound indicates two months. In such cases, I would rely on the ultrasound. For instance, if a woman arrives early in her pregnancy and the physical examination suggests a month of pregnancy, but the ultrasound shows an advanced gestational age, like in twin pregnancies, the ultrasound provides a more comprehensive view. In these scenarios, I prefer to base our assessment on the ultrasound findings.

### Focus group of Farakan health and social promotion center

[00:20:16-6](http://localhost:2300/file=C:/Users/USER/Desktop/ENTRETIENS%20PREMATURITE/CSPS_FARAKAN/FOCUS_GROUPE-CSPS_FARAKAN.MP3time=1216600) Interviewer: Maintenant, au cas où toutes ces méthodes d'estimation, que ce soit l'échographie, que ce soit est la hauteur utérine ou que ce soit à la date des dernières règles, au cas où toutes ces estimations diffèrent.Quelle estimation décidez vous d'utiliser ? S'il n'y a pas de concordance entre toutes ces estimations que vous venez de presenter, S'il n'y a pas de concordance, quelle est l'estimation que vous vous allez utiliser ? [00:20:47-0](http://localhost:2300/file=C:/Users/USER/Desktop/ENTRETIENS%20PREMATURITE/CSPS_FARAKAN/FOCUS_GROUPE-CSPS_FARAKAN.MP3time=1247000)

[00:20:47-0](http://localhost:2300/file=C:/Users/USER/Desktop/ENTRETIENS%20PREMATURITE/CSPS_FARAKAN/FOCUS_GROUPE-CSPS_FARAKAN.MP3time=1247000) Person 7: Nous attendons maintenant à la naissance nous-mêmes, nous examinons le nouveaux nés et à l'issu de ce qui se presente, il y a certains signes que nous pouvons détecter pour ou conclure à la prématurité, ça, c'est l'examen, le poids et de l'aspect physique, même la tour de cité. [00:21:09-9](http://localhost:2300/file=C:/Users/USER/Desktop/ENTRETIENS%20PREMATURITE/CSPS_FARAKAN/FOCUS_GROUPE-CSPS_FARAKAN.MP3time=1269900)

[00:21:09-9](http://localhost:2300/file=C:/Users/USER/Desktop/ENTRETIENS%20PREMATURITE/CSPS_FARAKAN/FOCUS_GROUPE-CSPS_FARAKAN.MP3time=1269900) Interviewer: La question c'est par rapport à l'âge gestationnel,s'il n' y a pas de concordance entre toutes les methodes d'estimation de l'âge de la grossesse, quelle methode utilisez vous et pourquoi? [00:21:21-6](http://localhost:2300/file=C:/Users/USER/Desktop/ENTRETIENS%20PREMATURITE/CSPS_FARAKAN/FOCUS_GROUPE-CSPS_FARAKAN.MP3time=1281600)

[00:21:21-6](http://localhost:2300/file=C:/Users/USER/Desktop/ENTRETIENS%20PREMATURITE/CSPS_FARAKAN/FOCUS_GROUPE-CSPS_FARAKAN.MP3time=1281600) Person 3: Bon, à défaut de tout ça, nous faisons confiance aux dires de la femme. [00:21:31-7](http://localhost:2300/file=C:/Users/USER/Desktop/ENTRETIENS%20PREMATURITE/CSPS_FARAKAN/FOCUS_GROUPE-CSPS_FARAKAN.MP3time=1291700)

[00:21:31-7](http://localhost:2300/file=C:/Users/USER/Desktop/ENTRETIENS%20PREMATURITE/CSPS_FARAKAN/FOCUS_GROUPE-CSPS_FARAKAN.MP3time=1291700) Person 5: Bon, même si la femme ne connaît pas sa date des dernières règles, la plupart il y à d'autres au moins, le mois elles connaissent donc ça nous guide aussi. [00:21:48-3](http://localhost:2300/file=C:/Users/USER/Desktop/ENTRETIENS%20PREMATURITE/CSPS_FARAKAN/FOCUS_GROUPE-CSPS_FARAKAN.MP3time=1308300)

[00:21:48-3](http://localhost:2300/file=C:/Users/USER/Desktop/ENTRETIENS%20PREMATURITE/CSPS_FARAKAN/FOCUS_GROUPE-CSPS_FARAKAN.MP3time=1308300) Person 7: On attend la naissance pour maintenant évaluer avec le poids de l'enfant. [00:22:04-1](http://localhost:2300/file=C:/Users/USER/Desktop/ENTRETIENS%20PREMATURITE/CSPS_FARAKAN/FOCUS_GROUPE-CSPS_FARAKAN.MP3time=1324100)

[00:22:04-1](http://localhost:2300/file=C:/Users/USER/Desktop/ENTRETIENS%20PREMATURITE/CSPS_FARAKAN/FOCUS_GROUPE-CSPS_FARAKAN.MP3time=1324100) Person 8: Après tous ces examens, c'est rare de ne pas déterminer l'âge de la grossesse. [00:22:25-9](http://localhost:2300/file=C:/Users/USER/Desktop/ENTRETIENS%20PREMATURITE/CSPS_FARAKAN/FOCUS_GROUPE-CSPS_FARAKAN.MP3time=1345900)

[00:22:25-9](http://localhost:2300/file=C:/Users/USER/Desktop/ENTRETIENS%20PREMATURITE/CSPS_FARAKAN/FOCUS_GROUPE-CSPS_FARAKAN.MP3time=1345900) Interviewer: Je ne sais pas si vous avez eu bien compris la question? [00:22:27-9](http://localhost:2300/file=C:/Users/USER/Desktop/ENTRETIENS%20PREMATURITE/CSPS_FARAKAN/FOCUS_GROUPE-CSPS_FARAKAN.MP3time=1347900)

[00:22:27-9](http://localhost:2300/file=C:/Users/USER/Desktop/ENTRETIENS%20PREMATURITE/CSPS_FARAKAN/FOCUS_GROUPE-CSPS_FARAKAN.MP3time=1347900) Person 3: Ok, ce que j'aimerais ajouter, si la dame ne connaît pas sa date des dernières règles,on se base sur la hauteur uterine. Avec la hauteur utérine on peut s'en sortir puisque par exemple, c'est ce que j'avais dit au début si on prend la Hu et que c'est 12 ou 16 ou 20, ça nous guide par exemple, 12 ce n'est que 3 mois, et 16 cm c'est 4 mois et 20cm c'est 5 mois, la plupart, ça ne peut pas depasser ça. [00:23:05-1](http://localhost:2300/file=C:/Users/USER/Desktop/ENTRETIENS%20PREMATURITE/CSPS_FARAKAN/FOCUS_GROUPE-CSPS_FARAKAN.MP3time=1385100)

00:20:16-6 Interviewer: Maintenant, au cas où toutes ces méthodes d'estimation, que ce soit l'échographie, que ce soit la hauteur utérine ou que ce soit à la date des dernières règles, au cas où toutes ces estimations diffèrent, quelle estimation décidez-vous d'utiliser ? S'il n'y a pas de concordance entre toutes ces estimations que vous venez de présenter, s'il n'y a pas de concordance, quelle est l'estimation que vous allez utiliser ?

00:20:47-0 Person 7: Nous attendons maintenant à la naissance nous-mêmes, nous examinons le nouveau-né et à l'issue de ce qui se présente, il y a certains signes que nous pouvons détecter pour ou conclure à la prématurité, ça, c'est l'examen, le poids et de l'aspect physique, même la tour de cité.

00:21:09-9 Interviewer: La question c'est par rapport à l'âge gestationnel, s'il n'y a pas de concordance entre toutes les méthodes d'estimation de l'âge de la grossesse, quelle méthode utilisez-vous et pourquoi ?

00:21:21-6 Person 3: Bon, à défaut de tout ça, nous faisons confiance aux dires de la femme.

00:21:31-7 Person 5: Bon, même si la femme ne connaît pas sa date des dernières règles, la plupart il y a d'autres au moins, le mois elles connaissent donc ça nous guide aussi.

00:21:48-3 Person 7: On attend la naissance pour maintenant évaluer avec le poids de l'enfant.
00:22:04-1 Person 8: Après tous ces examens, c'est rare de ne pas déterminer l'âge de la grossesse.
00:22:25-9 Interviewer: Je ne sais pas si vous avez bien compris la question ?

00:22:27-9 Person 3: Ok, ce que j'aimerais ajouter, si la dame ne connaît pas sa date des dernières règles, on se base sur la hauteur utérine. Avec la hauteur utérine, on peut s'en sortir puisque par exemple, c'est ce que j'avais dit au début si on prend la Hu et que c'est 12 ou 16 ou 20, ça nous guide par exemple, 12 ce n'est que 3 mois, et 16 cm c'est 4 mois et 20 cm c'est 5 mois, la plupart, ça ne peut pas dépasser ça.

**Interviewer (00:20:16-6):** If there's a discrepancy between ultrasound, fundal height, and LMP date estimates, which do you choose?

**Person 7 (00:20:47-0):** In such cases, we await the birth and examine the newborn for signs of prematurity, like weight and physical appearance.

**Interviewer (00:21:09-9):** Specifically, if there's no agreement among these gestational age estimates, which do you prioritize?

**Person 3 (00:21:21-6):** We trust the woman's words in such cases.

**Person 5 (00:21:31-7):** Even if they don't know the exact LMP date, most know the month, which guides us.

**Person 7 (00:21:48-3):** We wait for the birth to evaluate based on the baby's weight.

**Person 8 (00:22:04-1):** After these exams, it's rare not to determine the pregnancy age.

**Interviewer (00:22:25-9):** Are you sure you understood the question?

**Person 3 (00:22:27-9):** If the woman doesn't know her LMP date, we base it on fundal height. For example, a measurement of 12, 16, or 20 cm corresponds to 3, 4, or 5 months of pregnancy, respectively.

### Major of Farakan health and social promotion center

[00:07:25-6](http://localhost:2300/file=C:/Users/USER/Desktop/ENTRETIENS%20PREMATURITE/CSPS_FARAKAN/MAJOR%20FARAKAN.MP3time=445600) Interviewer: Maintenant au cas ou il ya une differnce entre les diferentes methodes d'estimation de l'âge de la grossesse, quelle est la methode qui sera utilisée et pourquoi? [00:07:48-2](http://localhost:2300/file=C:/Users/USER/Desktop/ENTRETIENS%20PREMATURITE/CSPS_FARAKAN/MAJOR%20FARAKAN.MP3time=468200)

[00:07:48-2](http://localhost:2300/file=C:/Users/USER/Desktop/ENTRETIENS%20PREMATURITE/CSPS_FARAKAN/MAJOR%20FARAKAN.MP3time=468200) Person 1: Ahh, moi je trouve que l'échographie est plus fiable. Prace que si on connait vraiment la date exacte des dernières règles, on peut utiliser nos propre methode de calcul, mais si la date des dernières règles ne sont pas connue, c'est difficile c'est pourquoi on va se fier à l'échographie. [00:08:10-2](http://localhost:2300/file=C:/Users/USER/Desktop/ENTRETIENS%20PREMATURITE/CSPS_FARAKAN/MAJOR%20FARAKAN.MP3time=490200)

00:07:25-6 Interviewer: Maintenant, au cas où il y a une différence entre les différentes méthodes d'estimation de l'âge de la grossesse, quelle est la méthode qui sera utilisée et pourquoi ? 00:07:48-2

00:07:48-2 Person 1: Ahh, moi je trouve que l'échographie est plus fiable. Parce que si on connaît vraiment la date exacte des dernières règles, on peut utiliser nos propres méthodes de calcul, mais si la date des dernières règles n'est pas connue, c'est difficile, c'est pourquoi on va se fier à l'échographie. 00:08:10-2

**Interviewer (00:07:25-6):** If there's a discrepancy between different methods of estimating pregnancy age, which method do you rely on and why?

**Person 1 (00:07:48-2):** I find the ultrasound to be more reliable. If we know the exact date of the last menstrual period, we can use our own calculation methods. However, if that date is unknown, it becomes difficult. That's why we rely on the ultrasound.

### Maternity manager of Farakan health and social promotion center

[00:10:30-2](http://localhost:2300/file=C:/Users/USER/Desktop/ENTRETIENS%20PREMATURITE/CSPS_FARAKAN/RESPONSABLE%20MATERNITE%20FARAKAN.MP3time=630200) Interviewer: Maintenant au cas où les estimations d'âge gestationnel par la date des dernières règles, la hauteur utérine et l'échgraphie sont différentes. Quelle estimation décidez vous d'utiliser ? [00:10:56-9](http://localhost:2300/file=C:/Users/USER/Desktop/ENTRETIENS%20PREMATURITE/CSPS_FARAKAN/RESPONSABLE%20MATERNITE%20FARAKAN.MP3time=656900)

[00:10:56-9](http://localhost:2300/file=C:/Users/USER/Desktop/ENTRETIENS%20PREMATURITE/CSPS_FARAKAN/RESPONSABLE%20MATERNITE%20FARAKAN.MP3time=656900) Person 1: Ce qui est sûr par rapport à la date probable d'accouchement, quand l'échographie fait, c'est soit 2 semaines en avance ou bien 2 semaines après la date donnée, on peut toujours corriger cela. Mais maintenant, si on voit que ce que l'échographie a donnée que ça dépasse par exemple les 2 semaines,on peut dire que ça a une grossesse prolongée, mais généralement en tout cas on demande une échographie pour voire.Mais maintenant, après le 3e trimestre, ce serait la biométrie, c'est la biométrie, surtout le poids de l'enfant.Maintenant , ils vont nous donner certains éléments par rapport au placenta.Puisque souvent aussi, tu peux prendre la hauteur utérine, tu vas voir que en tout cas ça ne concorde pas aussi avec l'âge de la grossesse, ce que la femme a dit donc ça aussi souvent ça pose problème parce que ça peut être une grossesse gemelaire, il y a des femmes aussi qui obèses donc il y a la paroi qui là ,ce sont tous ses facteurs qui peuvent faire fausser la datation de la grossesse.Mais maintenant quand elle te dit que ça fait 3 mois, tu prends,et tu vois comme si c'est une grossesse de 5 ou 6 mois c'est qu'il y a quelque chose qui ne va pas. cela peut arriver et en ce moment on demande une échographe pour pouvoir nous situer. [00:12:33-4](http://localhost:2300/file=C:/Users/USER/Desktop/ENTRETIENS%20PREMATURITE/CSPS_FARAKAN/RESPONSABLE%20MATERNITE%20FARAKAN.MP3time=753400)

00:10:30-2 Interviewer: Maintenant au cas où les estimations d'âge gestationnel par la date des dernières règles, la hauteur utérine et l'échographie sont différentes. Quelle estimation décidez-vous d'utiliser ? 00:10:56-9

00:10:56-9 Person 1: Ce qui est sûr par rapport à la date probable d'accouchement, quand l'échographie fait, c'est soit 2 semaines en avance ou bien 2 semaines après la date donnée, on peut toujours corriger cela. Mais maintenant, si on voit que ce que l'échographie a donné dépasse par exemple les 2 semaines, on peut dire que c'est une grossesse prolongée, mais généralement en tout cas on demande une échographie pour voir. Mais maintenant, après le 3e trimestre, ce serait la biométrie, c'est la biométrie, surtout le poids de l'enfant. Maintenant, ils vont nous donner certains éléments par rapport au placenta. Puisque souvent aussi, tu peux prendre la hauteur utérine, tu vas voir que ça ne concorde pas aussi avec l'âge de la grossesse, ce que la femme a dit donc ça aussi souvent ça pose problème parce que ça peut être une grossesse gémellaire, il y a des femmes aussi qui sont obèses donc il y a la paroi qui là, ce sont tous ces facteurs qui peuvent faire fausser la datation de la grossesse. Mais maintenant quand elle te dit que ça fait 3 mois, tu prends, et tu vois comme si c'est une grossesse de 5 ou 6 mois c'est qu'il y a quelque chose qui ne va pas. Cela peut arriver et en ce moment on demande une échographie pour pouvoir nous situer. 00:12:33-4

**Interviewer (00:10:30-2):** If there’s a difference between gestational age estimates from the last period, fundal height, and ultrasound, which do you choose?

**Person 1 (00:10:56-9):** For the probable date of delivery, an ultrasound can vary by about two weeks from the actual date, and we can adjust for this. If the ultrasound suggests a prolonged pregnancy, we generally request another ultrasound. After the third trimester, biometrics, especially the child's weight, and placental factors become important. Sometimes, fundal height doesn't align with the pregnancy age either, which could indicate twin pregnancy or be affected by maternal obesity. If a woman says she's been pregnant for 3 months but physical examination suggests 5 or 6 months, there's a discrepancy, and we request an ultrasound for clarity.

# 4. Registration of preterm births

### Focus group of Accart-ville urban medical center

[00:12:18-9](http://localhost:2300/file=C:/Users/USER/Desktop/ENTRETIENS%20PREMATURITE/CSPS-ACCART-VILLE/FOCUS%20GROUPE%20ACCART-VILLE-BON.mp3time=738900)Interviewer: ok nous allons poursuivre.Est-ce que les informations sur les naissances prématurées sont enregistrées dans le registre d'accouchement ? [00:12:32-2](http://localhost:2300/file=C:/Users/USER/Desktop/ENTRETIENS)

[00:12:32-2](http://localhost:2300/file=C:/Users/USER/Desktop/ENTRETIENS%20PREMATURITE/CSPS-ACCART-VILLE/FOCUS%20GROUPE%20ACCART-VILLE-BON.mp3time=752200) Person 7: oui (rire) c'est enregistré [00:12:37-5](http://localhost:2300/file=C:/Users/USER/Desktop/ENTRETIENS%20PREMATURITE/CSPS-ACCART-VILLE/FOCUS%20GROUPE%20ACCART-VILLE-BON.mp3time=757500)

[00:12:37-5](http://localhost:2300/file=C:/Users/USER/Desktop/ENTRETIENS)Interviewer: Est-ce que cela ressort de votre rapport mensuel d’activités ? [00:12:43-1](http://localhost:2300/file=C:/Users/USER/Desktop/ENTRETIENS)

[00:12:43-1](http://localhost:2300/file=C:/Users/USER/Desktop/ENTRETIENS%20PREMATURITE/CSPS-ACCART-VILLE/FOCUS%20GROUPE%20ACCART-VILLE-BON.mp3time=763100) Person 7: oui tous les accouchements sont enregistrés et dans le rapport aussi il y a tous les paramètres. il y a le paramètre de la prematurité également [00:12:59-4](http://localhost:2300/file=C:/Users/USER/Desktop/ENTRETIENS%20PREMATURITE/CSPS-ACCART-VILLE/FOCUS%20GROUPE%20ACCART-VILLE-BON.mp3time=779400)

00:12:18-9 Interviewer: Ok, nous allons poursuivre. Est-ce que les informations sur les naissances prématurées sont enregistrées dans le registre d’accouchement ? 00:12:32-2

00:12:32-2 Personne 7: Oui (rire), c'est enregistré. 00:12:37-5

00:12:37-5 Interviewer: Est-ce que cela ressort de votre rapport mensuel d’activités ? 00:12:43-1

00:12:43-1 Personne 7: Oui, tous les accouchements sont enregistrés et dans le rapport aussi il y a tous les paramètres. Il y a le paramètre de la prématurité également. 00:12:59-4

**Interviewer (00:12:18-9):** Okay, let's move on. Do you record information about premature births in the birth register?

**Person 7 (00:12:32-2):** Yes (laughs), it's recorded.

**Interviewer (00:12:37-5):** Is this information also included in your monthly activity reports?

**Person 7 (00:12:43-1):** Yes, all deliveries, including premature ones, are recorded. In our monthly reports, we document all relevant parameters, which includes data on prematurity.

### Manager of Accart-ville urban medical center

[00:08:51-2](http://localhost:2300/file=C:/Users/USER/Desktop/ENTRETIENS%20PREMATURITE/CSPS-ACCART-VILLE/RESPONSABLE%20CMU%20ACCART-VILLE.MP3time=531200)Interviewer: maintenant est ce que les informations sur les naissances prématurés sont enregistrées dans le registre d'accouchement? [00:08:57-3](http://localhost:2300/file=C:/Users/USER/Desktop/ENTRETIENS%20PREMATURITE/CSPS-ACCART-VILLE/RESPONSABLE%20CMU%20ACCART-VILLE.MP3time=537300)

[00:08:57-3](http://localhost:2300/file=C:/Users/USER/Desktop/ENTRETIENS%20PREMATURITE/CSPS-ACCART-VILLE/RESPONSABLE%20CMU%20ACCART-VILLE.MP3time=537300) Person 1: Oui mais bon, je pense qu'il y a une sous notification qui est là. puisqu' on essaye de mettre en place un contrôle de qualité ces deux derniers temps, on a eu une rencontre, on va essayer de voir comment pouvoir rattraper tout ça. [00:09:13-4](http://localhost:2300/file=C:/Users/USER/Desktop/ENTRETIENS%20PREMATURITE/CSPS-ACCART-VILLE/RESPONSABLE%20CMU%20ACCART-VILLE.MP3time=553400)

[00:09:13-4](http://localhost:2300/file=C:/Users/USER/Desktop/ENTRETIENS%20PREMATURITE/CSPS-ACCART-VILLE/RESPONSABLE%20CMU%20ACCART-VILLE.MP3time=553400)Interviewer: est ce que ses informations ressortent dans le rapport d'activité mensuel? [00:09:21-9](http://localhost:2300/file=C:/Users/USER/Desktop/ENTRETIENS%20PREMATURITE/CSPS-ACCART-VILLE/RESPONSABLE%20CMU%20ACCART-VILLE.MP3time=561900)

[00:09:21-9](http://localhost:2300/file=C:/Users/USER/Desktop/ENTRETIENS%20PREMATURITE/CSPS-ACCART-VILLE/RESPONSABLE%20CMU%20ACCART-VILLE.MP3time=561900)Person 1: puisque généralement c'est par rapport à notre rapport d'activité mensuel, qu'on a constaté que y a pas cela.on ne fait pas cas en fait,le RMA quand on en voit ou rarement,il n'y en a pas, on ne précise pas ça, c'est le nombre d'accouchements on précise, c'est à nous de voir maintenant comment on va pouvoir procéder pour pouvoir estimer. [00:09:47-6](http://localhost:2300/file=C:/Users/USER/Desktop/ENTRETIENS%20PREMATURITE/CSPS-ACCART-VILLE/RESPONSABLE%20CMU%20ACCART-VILLE.MP3time=587600)

00:08:51-2 Interviewer: Maintenant, est-ce que les informations sur les naissances prématurées sont enregistrées dans le registre d'accouchement ? 00:08:57-3

00:08:57-3 Personne 1: Oui, mais bon, je pense qu'il y a une sous-notification qui est là. Puisqu'on essaye de mettre en place un contrôle de qualité ces deux derniers temps, on a eu une rencontre, on va essayer de voir comment pouvoir rattraper tout ça. 00:09:13-4

00:09:13-4 Interviewer: Est-ce que ces informations ressortent dans le rapport d'activité mensuel ? 00:09:21-9

00:09:21-9 Personne 1: Puisque généralement c'est par rapport à notre rapport d'activité mensuel, qu'on a constaté qu'il n'y a pas cela. On ne fait pas cas en fait, le RMA quand on en voit ou rarement, il n'y en a pas, on ne précise pas ça, c'est le nombre d'accouchements on précise, c'est à nous de voir maintenant comment on va pouvoir procéder pour pouvoir estimer. 00:09:47-6

**Interviewer (00:08:51-2):** Is information on premature births recorded in the birth register?

**Person 1 (00:08:57-3):** Yes, but I believe there's an issue with under-reporting. We've recently been focusing on quality control and had a meeting to discuss how we can improve and catch up on this.

**Interviewer (00:09:13-4):** Does this information appear in your monthly activity reports?

**Person 1 (00:09:21-9):** In our monthly reports, we've noticed that premature births aren’t always detailed. We usually just count the number of deliveries without specifying if they were premature. We're now considering how to modify our reporting procedures to better estimate and document this information.

### Maternity manager of Accart-ville urban medical center

[00:11:04-3](http://localhost:2300/file=C:/Users/USER/Desktop/ENTRETIENS%20PREMATURITE/CSPS-ACCART-VILLE/RESPONSABLE%20MATERNITE%20CMU%20ACCART-VILLE.MP3time=664300) Interviewer: est ce que les informations sur les naissances prématurées sont enregistrées dans le registre d'accouchement? [00:11:08-5](http://localhost:2300/file=C:/Users/USER/Desktop/ENTRETIENS%20PREMATURITE/CSPS-ACCART-VILLE/RESPONSABLE%20MATERNITE%20CMU%20ACCART-VILLE.MP3time=668500)

[00:11:08-5](http://localhost:2300/file=C:/Users/USER/Desktop/ENTRETIENS%20PREMATURITE/CSPS-ACCART-VILLE/RESPONSABLE%20MATERNITE%20CMU%20ACCART-VILLE.MP3time=668500) Person 1: A part le registre d'accochement nous n'avons pas un autre registre, en temps normal on devrait avoir un autre registre pour la prématurité. je viens de finir une supervision sur la prise en charge des nouveaux nées, les soins essentiels aux nouveaux nées et la prise en charge aussi des nouveaux nées prématurés. maintenant ce qui est recommandé il faut qu'il y ait un registre à part entière pour l'enregistrement des nouveaux nées prématurés. nous n'avons pas d'autres registres à part le registre d'accouchement qui comporte aussi des parametres de primaturités. c'est dire si c'est une naissance prématurée ou pas, il ya aussi l'âge gestationnel qui y figure, on nous demande aussi de noter le nombre d'enfants de faible poids et de preciser si c'est prematurés ou pas.nous avons ses paramettres dans le registre d'accouchement [00:12:05-9](http://localhost:2300/file=C:/Users/USER/Desktop/ENTRETIENS%20PREMATURITE/CSPS-ACCART-VILLE/RESPONSABLE%20MATERNITE%20CMU%20ACCART-VILLE.MP3time=725900)

[00:12:05-9](http://localhost:2300/file=C:/Users/USER/Desktop/ENTRETIENS%20PREMATURITE/CSPS-ACCART-VILLE/RESPONSABLE%20MATERNITE%20CMU%20ACCART-VILLE.MP3time=725900) Interviewer: est ce que ses informations ressortent dans le rapport mensuel dactivité? [00:12:08-6](http://localhost:2300/file=C:/Users/USER/Desktop/ENTRETIENS%20PREMATURITE/CSPS-ACCART-VILLE/RESPONSABLE%20MATERNITE%20CMU%20ACCART-VILLE.MP3time=728600)

[00:12:08-6](http://localhost:2300/file=C:/Users/USER/Desktop/ENTRETIENS%20PREMATURITE/CSPS-ACCART-VILLE/RESPONSABLE%20MATERNITE%20CMU%20ACCART-VILLE.MP3time=728600) Person 1: effectivement cela ressort dans le rapport mensuel d'activité. il y a des item à ce niveau aussi pour la prématurité [00:12:17-9](http://localhost:2300/file=C:/Users/USER/Desktop/ENTRETIENS%20PREMATURITE/CSPS-ACCART-VILLE/RESPONSABLE%20MATERNITE%20CMU%20ACCART-VILLE.MP3time=737900)

00:11:04-3 Interviewer: Est-ce que les informations sur les naissances prématurées sont enregistrées dans le registre d'accouchement ? 00:11:08-5

00:11:08-5 Personne 1: À part le registre d'accouchement, nous n'avons pas un autre registre, en temps normal on devrait avoir un autre registre pour la prématurité. Je viens de finir une supervision sur la prise en charge des nouveau-nés, les soins essentiels aux nouveau-nés et la prise en charge aussi des nouveau-nés prématurés. Maintenant ce qui est recommandé, il faut qu'il y ait un registre à part entière pour l'enregistrement des nouveau-nés prématurés. Nous n'avons pas d'autres registres à part le registre d'accouchement qui comporte aussi des paramètres de prématurités. C'est dire si c'est une naissance prématurée ou pas, il y a aussi l'âge gestationnel qui y figure, on nous demande aussi de noter le nombre d'enfants de faible poids et de préciser si c'est prématuré ou pas. Nous avons ses paramètres dans le registre d'accouchement. 00:12:05-9

00:12:05-9 Interviewer: Est-ce que ces informations ressortent dans le rapport mensuel d'activité ? 00:12:08-6

00:12:08-6 Personne 1: Effectivement, cela ressort dans le rapport mensuel d'activité. Il y a des items à ce niveau aussi pour la prématurité. 00:12:17-9

**Interviewer (00:11:04-3):** Is information on premature births recorded in the birth register?

**Person 1 (00:11:08-5):** Currently, we only have the birth register, which also includes parameters for prematurity. Ideally, we should have a separate register specifically for premature births. I've recently completed supervision on newborn care, including the care of premature newborns. The recommendation is to maintain a distinct registry for premature newborns. In our birth register, we record whether a birth is premature or not, along with the gestational age. We also note the number of low birth weight children and specify if they are premature. All these details are included in our delivery register.

**Interviewer (00:12:05-9):** Is this information reflected in your monthly activity report?

**Person 1 (00:12:08-6):** Yes, this information is included in our monthly activity report. We have specific sections dedicated to recording data on prematurity.

### Focus group of Do medical center with surgical branch

[00:12:44-6](http://localhost:2300/file=C:/Users/USER/Desktop/ENTRETIENS%20PREMATURITE/CMA-DO/FOCUS%20GROUPE%20CMA-DO.mp3time=764600) Interviewer: Est ce que les informations sur les naissances prématurées sont enregistrées dans le registre d'accouchement? [00:12:54-7](http://localhost:2300/file=C:/Users/USER/Desktop/ENTRETIENS%20PREMATURITE/CMA-DO/FOCUS%20GROUPE%20CMA-DO.mp3time=774700)

[00:12:54-7](http://localhost:2300/file=C:/Users/USER/Desktop/ENTRETIENS%20PREMATURITE/CMA-DO/FOCUS%20GROUPE%20CMA-DO.mp3time=774700) Person 9: Oui oui c'est enregistrée puisqu'il y a une partie ou on met l'âge gestationnel. [00:13:06-0](http://localhost:2300/file=C:/Users/USER/Desktop/ENTRETIENS%20PREMATURITE/CMA-DO/FOCUS%20GROUPE%20CMA-DO.mp3time=786000)

[00:13:06-0](http://localhost:2300/file=C:/Users/USER/Desktop/ENTRETIENS%20PREMATURITE/CMA-DO/FOCUS%20GROUPE%20CMA-DO.mp3time=786000) Interviewer: Est-ce que ces informations ressortent dans le rapport mensuel d'activité ? [00:13:10-4](http://localhost:2300/file=C:/Users/USER/Desktop/ENTRETIENS%20PREMATURITE/CMA-DO/FOCUS%20GROUPE%20CMA-DO.mp3time=790400)

[00:13:10-4](http://localhost:2300/file=C:/Users/USER/Desktop/ENTRETIENS%20PREMATURITE/CMA-DO/FOCUS%20GROUPE%20CMA-DO.mp3time=790400) Person 6: Oui, oui. La prématurité ressort. [00:13:15-9](http://localhost:2300/file=C:/Users/USER/Desktop/ENTRETIENS%20PREMATURITE/CMA-DO/FOCUS%20GROUPE%20CMA-DO.mp3time=795900)

00:12:44-6 Interviewer: Est ce que les informations sur les naissances prématurées sont enregistrées dans le registre d'accouchement ? 00:12:54-7

00:12:54-7 Person 9: Oui oui. C'est enregistré puisqu'il y a une partie ou on met l'âge gestationnel. 00:13:06-0

00:13:06-0 Interviewer: Est-ce que ces informations ressortent dans le rapport mensuel d'activité ? 00:13:10-4

00:13:10-4 Personne 6: Oui, oui. La prématurité ressort. 00:13:15-9

**Interviewer (00:12:44-6):** Is information on premature births recorded in the birth register?

**Person 9 (00:12:54-7):** Yes, it is. We record it, as there is a specific section in the register for noting the gestational age of each birth.

**Interviewer (00:13:06-0):** Is this information also included in your monthly activity reports?

**Person 6 (00:13:10-4):** Yes, it is. Prematurity details are included in the monthly activity reports.

### Gynecologist 1

[00:08:19-9](http://localhost:2300/file=C:/Users/USER/Desktop/ENTRETIENS%20PREMATURITE/CMA-DO/GENYCOLOGUE%2001.mp3time=499900) Interviewer: Est-ce que les informations sur les naissances prématurées sont enregistrées dans les registres ? [00:08:24-5](http://localhost:2300/file=C:/Users/USER/Desktop/ENTRETIENS%20PREMATURITE/CMA-DO/GENYCOLOGUE%2001.mp3time=504500)

[00:08:24-5](http://localhost:2300/file=C:/Users/USER/Desktop/ENTRETIENS%20PREMATURITE/CMA-DO/GENYCOLOGUE%2001.mp3time=504500) Person 1: Oui. [00:08:26-7](http://localhost:2300/file=C:/Users/USER/Desktop/ENTRETIENS%20PREMATURITE/CMA-DO/GENYCOLOGUE%2001.mp3time=506700)

[00:08:26-7](http://localhost:2300/file=C:/Users/USER/Desktop/ENTRETIENS%20PREMATURITE/CMA-DO/GENYCOLOGUE%2001.mp3time=506700) Interviewer: Est-ce que cela ressort dans les rapports mensuels ? [00:08:28-9](http://localhost:2300/file=C:/Users/USER/Desktop/ENTRETIENS%20PREMATURITE/CMA-DO/GENYCOLOGUE%2001.mp3time=508900)

[00:08:28-9](http://localhost:2300/file=C:/Users/USER/Desktop/ENTRETIENS%20PREMATURITE/CMA-DO/GENYCOLOGUE%2001.mp3time=508900) Person 1: Oui, ça ressort dans les rapports mensuels. [00:08:31-3](http://localhost:2300/file=C:/Users/USER/Desktop/ENTRETIENS%20PREMATURITE/CMA-DO/GENYCOLOGUE%2001.mp3time=511300)

00:08:19-9 Interviewer: Est-ce que les informations sur les naissances prématurées sont enregistrées dans les registres ? 00:08:24-5

00:08:24-5 Personne 1: Oui. 00:08:26-7

00:08:26-7 Interviewer: Est-ce que cela ressort dans les rapports mensuels ? 00:08:28-9

00:08:28-9 Personne 1: Oui, ça ressort dans les rapports mensuels. 00:08:31-3

**Interviewer (00:08:19-9):** Is information on premature births recorded in your registers?

**Person 1 (00:08:24-5):** Yes, it is.

**Interviewer (00:08:26-7):** Does this information also appear in your monthly reports?

**Person 1 (00:08:28-9):** Yes, details on premature births are included in the monthly reports.

### Gynecologist 2

[00:10:58-2](http://localhost:2300/file=C:/Users/USER/Desktop/ENTRETIENS%20PREMATURITE/CMA-DO/GENYCOLOGUE%2002.mp3time=658200) Interviewer: Maintenant, est-ce que les informations sur les naissances prématurées sont enregistrées dans un régistre d'accouchement ? [00:11:03-9](http://localhost:2300/file=C:/Users/USER/Desktop/ENTRETIENS%20PREMATURITE/CMA-DO/GENYCOLOGUE%2002.mp3time=663900)

[00:11:03-9](http://localhost:2300/file=C:/Users/USER/Desktop/ENTRETIENS%20PREMATURITE/CMA-DO/GENYCOLOGUE%2002.mp3time=663900) Person 1: Oui, dans le régistre d'accouchement, comme les autres naissances. C'est-à-dire qu'on a un régistre de naissance, quand l'enfant naît, on enrégistre les différents paramètres de naissance dans le régistre. Donc à partir des paramètres de naissance et de l'âge gestationnel qu'on a trouvé au début de la grossesse, puisqu'il y a déjà le dossier qui signifie, que si on a les informations à l'avance, ça signifie qu'il s'agit d'un accouchement prématuré. Donc sur les dossiers, on trouve les traces. Maintenant dans le régistre d'accouchement, on trouve également les paramètres de l'enfant qui montrent que l'enfant est prématuré. Mais il n'y a pas un régistre spécial pour les accouchements prématurés. [00:11:42-2](http://localhost:2300/file=C:/Users/USER/Desktop/ENTRETIENS%20PREMATURITE/CMA-DO/GENYCOLOGUE%2002.mp3time=702200)

[00:11:42-2](http://localhost:2300/file=C:/Users/USER/Desktop/ENTRETIENS%20PREMATURITE/CMA-DO/GENYCOLOGUE%2002.mp3time=702200) Interviewer: Est-ce que ces informations sont dans le rapport mensuel d'activité ? [00:11:46-6](http://localhost:2300/file=C:/Users/USER/Desktop/ENTRETIENS%20PREMATURITE/CMA-DO/GENYCOLOGUE%2002.mp3time=706600)

[00:11:46-6](http://localhost:2300/file=C:/Users/USER/Desktop/ENTRETIENS%20PREMATURITE/CMA-DO/GENYCOLOGUE%2002.mp3time=706600) Person 1: Je ne sais pas vraiment. Je ne pense pas, parce que ça doit être mis ensemble avec les accouchements. Mais là, je pense que la responsable de l'unité de soin de la maternité doit être un peu plus disposée à donner ce genre d'informations, parce que je ne sais pas si on fait la part des choses entre les accouchements prématurés et les enfants nés à terme. [00:12:09-9](http://localhost:2300/file=C:/Users/USER/Desktop/ENTRETIENS%20PREMATURITE/CMA-DO/GENYCOLOGUE%2002.mp3time=729900)

[00:12:09-9](http://localhost:2300/file=C:/Users/USER/Desktop/ENTRETIENS%20PREMATURITE/CMA-DO/GENYCOLOGUE%2002.mp3time=729900) Interviewer: Donc cela veut dire qu'il va falloir que je rentre en contacte avec elle ? [00:12:13-8](http://localhost:2300/file=C:/Users/USER/Desktop/ENTRETIENS%20PREMATURITE/CMA-DO/GENYCOLOGUE%2002.mp3time=733800)

[00:12:13-8](http://localhost:2300/file=C:/Users/USER/Desktop/ENTRETIENS%20PREMATURITE/CMA-DO/GENYCOLOGUE%2002.mp3time=733800) Person 1: Elle peut voir si on fait la part des choses entre les accouchements prématurés et les accouchements à terme. Ce que je sais, c'est que tous les accouchements sont répertoriés dans le régistre d'accouchement. [00:12:25-2](http://localhost:2300/file=C:/Users/USER/Desktop/ENTRETIENS%20PREMATURITE/CMA-DO/GENYCOLOGUE%2002.mp3time=745200)

00:10:58-2 Interviewer: Maintenant, est-ce que les informations sur les naissances prématurées sont enregistrées dans un registre d'accouchement ? 00:11:03-9

00:11:03-9 Personne 1: Oui, dans le registre d'accouchement, comme les autres naissances. C'est-à-dire qu'on a un registre de naissance, quand l'enfant naît, on enregistre les différents paramètres de naissance dans le registre. Donc à partir des paramètres de naissance et de l'âge gestationnel qu'on a trouvé au début de la grossesse, puisqu'il y a déjà le dossier qui signifie, que si on a les informations à l'avance, ça signifie qu'il s'agit d'un accouchement prématuré. Donc sur les dossiers, on trouve les traces. Maintenant dans le registre d'accouchement, on trouve également les paramètres de l'enfant qui montrent que l'enfant est prématuré. Mais il n'y a pas un registre spécial pour les accouchements prématurés. 00:11:42-2

00:11:42-2 Interviewer: Est-ce que ces informations sont dans le rapport mensuel d'activité ? 00:11:46-6

00:11:46-6 Personne 1: Je ne sais pas vraiment. Je ne pense pas, parce que ça doit être mis ensemble avec les accouchements. Mais là, je pense que la responsable de l'unité de soin de la maternité doit être un peu plus disposée à donner ce genre d'informations, parce que je ne sais pas si on fait la part des choses entre les accouchements prématurés et les enfants nés à terme. 00:12:09-9

00:12:09-9 Interviewer: Donc cela veut dire qu'il va falloir que je rentre en contact avec elle ? 00:12:13-8

00:12:13-8 Personne 1: Elle peut voir si on fait la part des choses entre les accouchements prématurés et les accouchements à terme. Ce que je sais, c'est que tous les accouchements sont répertoriés dans le registre d'accouchement. 00:12:25-2

**Interviewer (00:10:58-2):** Is information on premature births recorded in a birth register?

**Person 1 (00:11:03-9):** Yes, premature births are recorded in the birth register, just like other births. We have a register where we note various birth parameters when a child is born. These parameters, along with the gestational age determined at the start of the pregnancy, help us identify if the birth is premature. So, the evidence of prematurity is found in the patient files. In the delivery register, we record the newborn's parameters that indicate prematurity. However, we do not have a separate register specifically for premature births.

**Interviewer (00:11:42-2):** Is this information included in the monthly activity report?

**Person 1 (00:11:46-6):** I'm not entirely sure. I don't think so, as it should be consolidated with other childbirth data. I believe the head of the maternity care unit might have more detailed information. I'mMCD not certain if there's a distinction between premature and full-term births in the report. All I know is that all births, regardless of their nature, are recorded in the birth register.

**Interviewer (00:12:09-9):** So, should I contact the head of the maternity unit for more information?

**Person 1 (00:12:13-8):** Yes, she can clarify whether there's a differentiation between premature and full-term births in our reporting. But as far as I know, all births are listed in the birth register.

### Chief physician of Do health district

[00:17:25-2](http://localhost:2300/file=C:/Users/USER/Desktop/ENTRETIENS%20PREMATURITE/CMA-DO/MCD-DO.mp3time=1045200) Interviewer: D'accord. Maintenant, est-ce que les informations sur les naissances prématurées sont enregistrées dans les registres d'accouchements? [00:17:33-9](http://localhost:2300/file=C:/Users/USER/Desktop/ENTRETIENS%20PREMATURITE/CMA-DO/MCD-DO.mp3time=1053900)

[00:17:33-9](http://localhost:2300/file=C:/Users/USER/Desktop/ENTRETIENS%20PREMATURITE/CMA-DO/MCD-DO.mp3time=1053900) Person 1: Oui, ici, c'est écrit, mais ce n'est pas collecté. Voilà, ce n'est pas collecté, mais on va dire que c'est subtilement écrit. Si nous, on dit que l'enfant est né à 7 mois, c'est une prématurité. Même si on n'a pas dit prématurité, parce que ce n'est pas une information qu'on collecte comme ça. [00:17:55-9](http://localhost:2300/file=C:/Users/USER/Desktop/ENTRETIENS%20PREMATURITE/CMA-DO/MCD-DO.mp3time=1075900)

[00:17:55-9](http://localhost:2300/file=C:/Users/USER/Desktop/ENTRETIENS%20PREMATURITE/CMA-DO/MCD-DO.mp3time=1075900) Interviewer: Donc, ça veut dire que ça ne ressort pas dans les rapports mensuels d'activités? [00:17:58-9](http://localhost:2300/file=C:/Users/USER/Desktop/ENTRETIENS%20PREMATURITE/CMA-DO/MCD-DO.mp3time=1078900)

[00:17:58-9](http://localhost:2300/file=C:/Users/USER/Desktop/ENTRETIENS%20PREMATURITE/CMA-DO/MCD-DO.mp3time=1078900) Person 1: Non, non, non, non, on ne le collecte pas. La collecte primaire ne tient pas compte de ça. [00:18:04-4](http://localhost:2300/file=C:/Users/USER/Desktop/ENTRETIENS%20PREMATURITE/CMA-DO/MCD-DO.mp3time=1084400)

00:17:25-2 Interviewer: D'accord. Maintenant, est-ce que les informations sur les naissances prématurées sont enregistrées dans les registres d’accouchements ? 00:17:33-9

00:17:33-9 Personne 1: Oui, ici, c'est écrit, mais ce n'est pas collecté. Voilà, ce n'est pas collecté, mais on va dire que c'est subtilement écrit. Si nous, on dit que l'enfant est né à 7 mois, c'est une prématurité. Même si on n'a pas dit prématurité, parce que ce n'est pas une information qu'on collecte comme ça. 00:17:55-9

00:17:55-9 Interviewer: Donc, ça veut dire que ça ne ressort pas dans les rapports mensuels d'activités ? 00:17:58-9

00:17:58-9 Personne 1: Non, non, non, non, on ne le collecte pas. La collecte primaire ne tient pas compte de ça. 00:18:04-4

**Interviewer (00:17:25-2):** Okay. Is information on premature births recorded in birth registers at your facility?

**Person 1 (00:17:33-9):** Yes, we do note it in the registers, but it's not systematically collected. For example, if we record that a child was born at 7 months, we implicitly indicate it's a premature birth. However, we don't explicitly collect 'prematurity' as a separate data point.

**Interviewer (00:17:55-9):** So, does that mean this information isn't included in your monthly activity reports?

**Person 1 (00:17:58-9):** No, we don't include it. Our primary data collection process doesn't specifically account for prematurity details.

### Manager for maternity care unit of Do medical center with surgical branch

[00:12:31-3](http://localhost:2300/file=C:/Users/USER/Desktop/ENTRETIENS%20PREMATURITE/CMA-DO/RESPONSABLE%20UNITE%20DE%20SOIN%20CMA-DO.mp3time=751300) Interviewer: Est-ce que les informations sur les naissance prématurées sont enregistrées dans le registre d'accouchement? [00:12:39-5](http://localhost:2300/file=C:/Users/USER/Desktop/ENTRETIENS%20PREMATURITE/CMA-DO/RESPONSABLE%20UNITE%20DE%20SOIN%20CMA-DO.mp3time=759500)

[00:12:39-5](http://localhost:2300/file=C:/Users/USER/Desktop/ENTRETIENS%20PREMATURITE/CMA-DO/RESPONSABLE%20UNITE%20DE%20SOIN%20CMA-DO.mp3time=759500) Person 1: Les informations, pas trop. Pas trop. On met le poids, oui. Mais ce n'est pas le poids seulement. Donc, il y a d'autres éléments qui devraient suivre. Mais la plupart du temps, ce n'est pas noté. Parce qu'on ne peut pas aussi partir du poids pour dire que c'est un prématuré.Il y a les hypotrophes aussi. Voilà. Les faibles poids de naissance. Donc, on n'a pas souvent assez d'informations pour nous permettre de dire que c'est un accouchement, un prématuré. [00:13:09-0](http://localhost:2300/file=C:/Users/USER/Desktop/ENTRETIENS%20PREMATURITE/CMA-DO/RESPONSABLE%20UNITE%20DE%20SOIN%20CMA-DO.mp3time=789000)

[00:13:09-0](http://localhost:2300/file=C:/Users/USER/Desktop/ENTRETIENS%20PREMATURITE/CMA-DO/RESPONSABLE%20UNITE%20DE%20SOIN%20CMA-DO.mp3time=789000) Interviewer: Est-ce que les informations, quand même, qui sont relevées dans le registre apparaissent dans les rapports mensuels d'activités? [00:13:16-4](http://localhost:2300/file=C:/Users/USER/Desktop/ENTRETIENS%20PREMATURITE/CMA-DO/RESPONSABLE%20UNITE%20DE%20SOIN%20CMA-DO.mp3time=796400)

[00:13:16-4](http://localhost:2300/file=C:/Users/USER/Desktop/ENTRETIENS%20PREMATURITE/CMA-DO/RESPONSABLE%20UNITE%20DE%20SOIN%20CMA-DO.mp3time=796400) Person 1: Bon, parce que le prématuré, là, quand je prends le rapport mensuel, là, c'est difficile en faite de pouvoir le retracer, parce que ce n'est pas noté dans le registre. Parce que c'est à travers le registre qu'on fait la collecte. Voilà. On arrive à savoir, en tout cas, quand on a des faibles poids de naissance.Poids inférieur à 2500. Mais on n'a pas assez d'éléments qui nous permettent de dire que c'est un prématuré ou que c'est un hypotrophe. [00:13:43-7](http://localhost:2300/file=C:/Users/USER/Desktop/ENTRETIENS%20PREMATURITE/CMA-DO/RESPONSABLE%20UNITE%20DE%20SOIN%20CMA-DO.mp3time=823700)

[00:13:43-7](http://localhost:2300/file=C:/Users/USER/Desktop/ENTRETIENS%20PREMATURITE/CMA-DO/RESPONSABLE%20UNITE%20DE%20SOIN%20CMA-DO.mp3time=823700) Interviewer: Vous avez dit qu'au sein du centre, il n'y a pas un registre spécifique pour le prématuré? [00:13:48-1](http://localhost:2300/file=C:/Users/USER/Desktop/ENTRETIENS%20PREMATURITE/CMA-DO/RESPONSABLE%20UNITE%20DE%20SOIN%20CMA-DO.mp3time=828100)

[00:13:48-1](http://localhost:2300/file=C:/Users/USER/Desktop/ENTRETIENS%20PREMATURITE/CMA-DO/RESPONSABLE%20UNITE%20DE%20SOIN%20CMA-DO.mp3time=828100) Person 1: Non, il n'y a pas un registre spécifique. Normalement, ça devrait ressortir dans le registre d'accouchement. [00:13:54-7](http://localhost:2300/file=C:/Users/USER/Desktop/ENTRETIENS%20PREMATURITE/CMA-DO/RESPONSABLE%20UNITE%20DE%20SOIN%20CMA-DO.mp3time=834700)

00:12:31-3 Interviewer: Est-ce que les informations sur les naissances prématurées sont enregistrées dans le registre d'accouchement ? 00:12:39-5

00:12:39-5 Personne 1: Les informations, pas trop. Pas trop. On met le poids, oui. Mais ce n'est pas le poids seulement. Donc, il y a d'autres éléments qui devraient suivre. Mais la plupart du temps, ce n'est pas noté. Parce qu'on ne peut pas aussi partir du poids pour dire que c'est un prématuré. Il y a les hypotrophes aussi. Voilà. Les faibles poids de naissance. Donc, on n'a pas souvent assez d'informations pour nous permettre de dire que c'est un accouchement, un prématuré. 00:13:09-0

00:13:09-0 Interviewer: Est-ce que les informations, quand même, qui sont relevées dans le registre apparaissent dans les rapports mensuels d'activités ? 00:13:16-4

00:13:16-4 Personne 1: Bon, parce que le prématuré, là, quand je prends le rapport mensuel, là, c'est difficile en fait de pouvoir le retracer, parce que ce n'est pas noté dans le registre. Parce que c'est à travers le registre qu'on fait la collecte. Voilà. On arrive à savoir, en tout cas, quand on a des faibles poids de naissance. Poids inférieur à 2500. Mais on n'a pas assez d'éléments qui nous permettent de dire que c'est un prématuré ou que c'est un hypotrophe. 00:13:43-7

00:13:43-7 Interviewer: Vous avez dit qu'au sein du centre, il n'y a pas un registre spécifique pour le prématuré ? 00:13:48-1

00:13:48-1 Personne 1: Non, il n'y a pas un registre spécifique. Normalement, ça devrait ressortir dans le registre d'accouchement. 00:13:54-7

**Interviewer (00:12:31-3):** Is information on premature births recorded in the birth register?

**Person 1 (00:12:39-5):** Not really. We do record the birth weight, but that alone isn't enough. There are other factors that should be considered, but most of the time, they aren't noted. We can't determine prematurity based solely on weight, as there are also cases of low birth weight due to other reasons, like hypotrophy. So, we often lack sufficient information to classify a birth as premature.

**Interviewer (00:13:09-0):** Does the information that is recorded in the register appear in the monthly activity reports?

**Person 1 (00:13:16-4):** When preparing the monthly report, it's hard to trace prematurity because it's not specifically noted in the register. We use the register for data collection. We can identify low birth weight, like weights less than 2500 grams, but we don’t have enough information to distinguish between premature births and hypotrophic cases.

**Interviewer (00:13:43-7):** So, within your center, there isn't a specific register for premature babies?

**Person 1 (00:13:48-1):** No, there isn’t a specific register for that. Ideally, such details should be included in the birth register.

### Focus group of Colma1 health and social promotion center

[00:12:54-7](http://localhost:2300/file=C:/Users/USER/Desktop/ENTRETIENS%20PREMATURITE/CSPS-COLMA1/FOCUS%20GROUPE%20COLMA1.mp3time=774700) Interviewer: Est ce que les informations sur les naissances prématurées sont enregistrées dans un registre d'accouchement? [00:13:02-1](http://localhost:2300/file=C:/Users/USER/Desktop/ENTRETIENS%20PREMATURITE/CSPS-COLMA1/FOCUS%20GROUPE%20COLMA1.mp3time=782100)

[00:13:02-1](http://localhost:2300/file=C:/Users/USER/Desktop/ENTRETIENS%20PREMATURITE/CSPS-COLMA1/FOCUS%20GROUPE%20COLMA1.mp3time=782100) Person 5: Dans un registre? [00:13:03-1](http://localhost:2300/file=C:/Users/USER/Desktop/ENTRETIENS%20PREMATURITE/CSPS-COLMA1/FOCUS%20GROUPE%20COLMA1.mp3time=783100)

[00:13:03-1](http://localhost:2300/file=C:/Users/USER/Desktop/ENTRETIENS%20PREMATURITE/CSPS-COLMA1/FOCUS%20GROUPE%20COLMA1.mp3time=783100) Interviewer: Oui [00:13:03-3](http://localhost:2300/file=C:/Users/USER/Desktop/ENTRETIENS%20PREMATURITE/CSPS-COLMA1/FOCUS%20GROUPE%20COLMA1.mp3time=783300)

[00:13:03-3](http://localhost:2300/file=C:/Users/USER/Desktop/ENTRETIENS%20PREMATURITE/CSPS-COLMA1/FOCUS%20GROUPE%20COLMA1.mp3time=783300) Person 6: Bien sûr cela ressort dans le registre on fait même le rapport mensuel,ça ressort. [00:13:07-4](http://localhost:2300/file=C:/Users/USER/Desktop/ENTRETIENS%20PREMATURITE/CSPS-COLMA1/FOCUS%20GROUPE%20COLMA1.mp3time=787400)

[00:13:07-4](http://localhost:2300/file=C:/Users/USER/Desktop/ENTRETIENS%20PREMATURITE/CSPS-COLMA1/FOCUS%20GROUPE%20COLMA1.mp3time=787400) Person 2: Dans le registre d'accouchement,cela ressort puisqu'il y a une partie ou on demande si l'enfant est à terme ou bien s'il est prématuré? oui oui ça ressort. [00:13:29-2](http://localhost:2300/file=C:/Users/USER/Desktop/ENTRETIENS%20PREMATURITE/CSPS-COLMA1/FOCUS%20GROUPE%20COLMA1.mp3time=809200)

[00:13:29-2](http://localhost:2300/file=C:/Users/USER/Desktop/ENTRETIENS%20PREMATURITE/CSPS-COLMA1/FOCUS%20GROUPE%20COLMA1.mp3time=809200) Interviewer: Nous aurons besoins des informations sur les naissances prématurées des sept derniers mois. [00:13:34-0](http://localhost:2300/file=C:/Users/USER/Desktop/ENTRETIENS%20PREMATURITE/CSPS-COLMA1/FOCUS%20GROUPE%20COLMA1.mp3time=814000)

[00:13:34-0](http://localhost:2300/file=C:/Users/USER/Desktop/ENTRETIENS%20PREMATURITE/CSPS-COLMA1/FOCUS%20GROUPE%20COLMA1.mp3time=814000) Person 2: Des sept derniers mois aujourd'hui? [00:13:34-9](http://localhost:2300/file=C:/Users/USER/Desktop/ENTRETIENS%20PREMATURITE/CSPS-COLMA1/FOCUS%20GROUPE%20COLMA1.mp3time=814900)

[00:13:34-9](http://localhost:2300/file=C:/Users/USER/Desktop/ENTRETIENS%20PREMATURITE/CSPS-COLMA1/FOCUS%20GROUPE%20COLMA1.mp3time=814900) Interviewer: plutard [00:13:36-1](http://localhost:2300/file=C:/Users/USER/Desktop/ENTRETIENS%20PREMATURITE/CSPS-COLMA1/FOCUS%20GROUPE%20COLMA1.mp3time=816100)

[00:13:36-1](http://localhost:2300/file=C:/Users/USER/Desktop/ENTRETIENS%20PREMATURITE/CSPS-COLMA1/FOCUS%20GROUPE%20COLMA1.mp3time=816100) Person 2: ok, parce que le major est abcent. [00:13:40-5](http://localhost:2300/file=C:/Users/USER/Desktop/ENTRETIENS%20PREMATURITE/CSPS-COLMA1/FOCUS%20GROUPE%20COLMA1.mp3time=820500)

00:12:54-7 Interviewer: Est-ce que les informations sur les naissances prématurées sont enregistrées dans un registre d'accouchement ? 00:13:02-1

00:13:02-1 Personne 5: Dans un registre ? 00:13:03-1

00:13:03-1 Interviewer: Oui. 00:13:03-3

00:13:03-3 Personne 6: Bien sûr, cela ressort dans le registre, on fait même le rapport mensuel, ça ressort. 00:13:07-4

00:13:07-4 Personne 2: Dans le registre d'accouchement, cela ressort puisqu'il y a une partie où on demande si l'enfant est à terme ou bien s'il est prématuré ? Oui, oui ça ressort. 00:13:29-2

00:13:29-2 Interviewer: Nous aurons besoin des informations sur les naissances prématurées des sept derniers mois. 00:13:34-0

00:13:34-0 Personne 2: Des sept derniers mois aujourd'hui ? 00:13:34-9

00:13:34-9 Interviewer: Plus tard. 00:13:36-1

00:13:36-1 Personne 2: Ok, parce que le major est absent. 00:13:40-5

**Interviewer (00:12:54-7):** Is information on premature births recorded in a birth register?

**Person 5 (00:13:02-1):** In a register?

**Interviewer (00:13:03-1):** Yes.

**Person 6 (00:13:03-3):** Of course, premature births are recorded in the register. We even include this information in our monthly reports.

**Person 2 (00:13:07-4):** Yes, in the birth register, we specifically note whether a child is full-term or premature. It's clearly indicated.

**Interviewer (00:13:29-2):** We will need information on premature births from the last seven months.

**Person 2 (00:13:34-0):** Do you mean starting from today, going back seven months?

**Interviewer (00:13:34-9):** Yes, later on.

**Person 2 (00:13:36-1):** Okay, I understand. However, our senior staff member who usually handles this is currently away.

### Major of Colma1 health and social promotion center

[00:09:41-2](http://localhost:2300/file=C:/Users/USER/Desktop/ENTRETIENS%20PREMATURITE/CSPS-COLMA1/MAJOR%20CSPS%20COLMA1.MP3time=581200) Interviewer: est ce que les informations sur les naissances prématurées sont enregistrées dans le registre d'accouchement? [00:09:46-8](http://localhost:2300/file=C:/Users/USER/Desktop/ENTRETIENS%20PREMATURITE/CSPS-COLMA1/MAJOR%20CSPS%20COLMA1.MP3time=586800)

[00:09:46-8](http://localhost:2300/file=C:/Users/USER/Desktop/ENTRETIENS%20PREMATURITE/CSPS-COLMA1/MAJOR%20CSPS%20COLMA1.MP3time=586800) Person 1: oui c'est enregistrée [00:09:48-8](http://localhost:2300/file=C:/Users/USER/Desktop/ENTRETIENS%20PREMATURITE/CSPS-COLMA1/MAJOR%20CSPS%20COLMA1.MP3time=588800)

[00:09:48-8](http://localhost:2300/file=C:/Users/USER/Desktop/ENTRETIENS%20PREMATURITE/CSPS-COLMA1/MAJOR%20CSPS%20COLMA1.MP3time=588800) Interviewer: est ce que ses informations ressortent dans le rapport mensuel d'activités? [00:09:50-7](http://localhost:2300/file=C:/Users/USER/Desktop/ENTRETIENS%20PREMATURITE/CSPS-COLMA1/MAJOR%20CSPS%20COLMA1.MP3time=590700)

[00:09:50-7](http://localhost:2300/file=C:/Users/USER/Desktop/ENTRETIENS%20PREMATURITE/CSPS-COLMA1/MAJOR%20CSPS%20COLMA1.MP3time=590700) Person 1: oui cela ressort. [00:09:53-4](http://localhost:2300/file=C:/Users/USER/Desktop/ENTRETIENS%20PREMATURITE/CSPS-COLMA1/MAJOR%20CSPS%20COLMA1.MP3time=593400)

00:09:41-2 Interviewer: Est-ce que les informations sur les naissances prématurées sont enregistrées dans le registre d'accouchement ? 00:09:46-8

00:09:46-8 Personne 1: Oui, c'est enregistré. 00:09:48-8

00:09:48-8 Interviewer: Est-ce que ces informations ressortent dans le rapport mensuel d'activités ? 00:09:50-7

00:09:50-7 Personne 1: Oui, cela ressort. 00:09:53-4

**Interviewer (00:09:41-2):** Is information on premature births recorded in the birth register?

**Person 1 (00:09:46-8):** Yes, it's recorded.

**Interviewer (00:09:48-8):** Does this information also appear in the monthly activity report?

**Person 1 (00:09:50-7):** Yes, information about premature births is included in the monthly report.

### Maternity manager of Colma1 health and social promotion center

[00:17:51-9](http://localhost:2300/file=C:/Users/USER/Desktop/ENTRETIENS%20PREMATURITE/CSPS-COLMA1/RESPONSABLE%20MATERNITE-COLMA1.mp3time=1071900) Interviewer: Maintenant, est-ce que les informations sur les naissances prématurées sont enregistrées dans le registre d'accouchement ? [00:17:58-0](http://localhost:2300/file=C:/Users/USER/Desktop/ENTRETIENS%20PREMATURITE/CSPS-COLMA1/RESPONSABLE%20MATERNITE-COLMA1.mp3time=1078000)

[00:17:58-0](http://localhost:2300/file=C:/Users/USER/Desktop/ENTRETIENS%20PREMATURITE/CSPS-COLMA1/RESPONSABLE%20MATERNITE-COLMA1.mp3time=1078000) Person 1: Oui, dans le registre d'accouchement. Parce qu'il y a une partie même qui nous dit enfant à terme, enfant prématuré. [00:18:06-0](http://localhost:2300/file=C:/Users/USER/Desktop/ENTRETIENS%20PREMATURITE/CSPS-COLMA1/RESPONSABLE%20MATERNITE-COLMA1.mp3time=1086000)

[00:18:06-0](http://localhost:2300/file=C:/Users/USER/Desktop/ENTRETIENS%20PREMATURITE/CSPS-COLMA1/RESPONSABLE%20MATERNITE-COLMA1.mp3time=1086000) Interviewer: Est-ce que cela ressort dans le rapport mensuel d'activité (RMA)? [00:18:09-7](http://localhost:2300/file=C:/Users/USER/Desktop/ENTRETIENS%20PREMATURITE/CSPS-COLMA1/RESPONSABLE%20MATERNITE-COLMA1.mp3time=1089700)

[00:18:09-7](http://localhost:2300/file=C:/Users/USER/Desktop/ENTRETIENS%20PREMATURITE/CSPS-COLMA1/RESPONSABLE%20MATERNITE-COLMA1.mp3time=1089700) Person 1: Oui, bien sûr, parce qu'on nous demande. Ça ressort. [00:18:16-5](http://localhost:2300/file=C:/Users/USER/Desktop/ENTRETIENS%20PREMATURITE/CSPS-COLMA1/RESPONSABLE%20MATERNITE-COLMA1.mp3time=1096500)

[00:18:16-5](http://localhost:2300/file=C:/Users/USER/Desktop/ENTRETIENS%20PREMATURITE/CSPS-COLMA1/RESPONSABLE%20MATERNITE-COLMA1.mp3time=1096500) Interviewer: J'aurais besoin des estimations de sept derniers mois, si ça ne vous dérange pas. [00:18:21-1](http://localhost:2300/file=C:/Users/USER/Desktop/ENTRETIENS%20PREMATURITE/CSPS-COLMA1/RESPONSABLE%20MATERNITE-COLMA1.mp3time=1101100)

[00:18:21-1](http://localhost:2300/file=C:/Users/USER/Desktop/ENTRETIENS%20PREMATURITE/CSPS-COLMA1/RESPONSABLE%20MATERNITE-COLMA1.mp3time=1101100) Interviewer: Il n'y a pas de problème. [00:18:22-6](http://localhost:2300/file=C:/Users/USER/Desktop/ENTRETIENS%20PREMATURITE/CSPS-COLMA1/RESPONSABLE%20MATERNITE-COLMA1.mp3time=1102600)

00:17:51-9 Interviewer: Maintenant, est-ce que les informations sur les naissances prématurées sont enregistrées dans le registre d'accouchement ? 00:17:58-0

00:17:58-0 Personne 1: Oui, dans le registre d'accouchement. Parce qu'il y a une partie même qui nous dit enfant à terme, enfant prématuré. 00:18:06-0

00:18:06-0 Interviewer: Est-ce que cela ressort dans le rapport mensuel d'activité (RMA) ? 00:18:09-7

00:18:09-7 Personne 1: Oui, bien sûr, parce qu'on nous demande. Ça ressort. 00:18:16-5

00:18:16-5 Interviewer: J'aurais besoin des estimations des sept derniers mois, si ça ne vous dérange pas. 00:18:21-1

00:18:21-1 Interviewer: Il n'y a pas de problème. 00:18:22-6

**Interviewer (00:17:51-9):** Is information on premature births recorded in the birth register?

**Person 1 (00:17:58-0):** Yes, it's included in the birth register. There's even a specific section that indicates whether a child is full-term or premature.

**Interviewer (00:18:06-0):** Is this information also reflected in the monthly activity report (MAR)?

**Person 1 (00:18:09-7):** Yes, of course. We are required to include it. Premature births are clearly marked in the report.

**Interviewer (00:18:16-5):** I'll need the estimates for the last seven months, if that's possible.

**Person 1 (00:18:21-1):** There is no problem with that. We can provide those estimates.

### Focus group of Farakan health and social promotion center

[00:23:05-1](http://localhost:2300/file=C:/Users/USER/Desktop/ENTRETIENS%20PREMATURITE/CSPS_FARAKAN/FOCUS_GROUPE-CSPS_FARAKAN.MP3time=1385100) Interviewer: Donc nous allons poursuivre, est-ce que les informations sur la naissance prématurée sont enregistrées dans le registre d'accouchement ? [00:23:16-1](http://localhost:2300/file=C:/Users/USER/Desktop/ENTRETIENS%20PREMATURITE/CSPS_FARAKAN/FOCUS_GROUPE-CSPS_FARAKAN.MP3time=1396100)

[00:23:16-1](http://localhost:2300/file=C:/Users/USER/Desktop/ENTRETIENS%20PREMATURITE/CSPS_FARAKAN/FOCUS_GROUPE-CSPS_FARAKAN.MP3time=1396100) Person 2: oui, les accouchements prématurés sont enregistrés dans les registres d'accouchements. [00:23:22-3](http://localhost:2300/file=C:/Users/USER/Desktop/ENTRETIENS%20PREMATURITE/CSPS_FARAKAN/FOCUS_GROUPE-CSPS_FARAKAN.MP3time=1402300)

[00:23:22-3](http://localhost:2300/file=C:/Users/USER/Desktop/ENTRETIENS%20PREMATURITE/CSPS_FARAKAN/FOCUS_GROUPE-CSPS_FARAKAN.MP3time=1402300) Interviewer: Est ce que cela ressort dans les rapports mensuels d'activités? [00:23:24-2](http://localhost:2300/file=C:/Users/USER/Desktop/ENTRETIENS%20PREMATURITE/CSPS_FARAKAN/FOCUS_GROUPE-CSPS_FARAKAN.MP3time=1404200)

[00:23:24-2](http://localhost:2300/file=C:/Users/USER/Desktop/ENTRETIENS%20PREMATURITE/CSPS_FARAKAN/FOCUS_GROUPE-CSPS_FARAKAN.MP3time=1404200) Person 2: Oui,ça ressort dans les rapports mensuels d'activité aussi. [00:23:28-4](http://localhost:2300/file=C:/Users/USER/Desktop/ENTRETIENS%20PREMATURITE/CSPS_FARAKAN/FOCUS_GROUPE-CSPS_FARAKAN.MP3time=1408400)

[00:23:28-4](http://localhost:2300/file=C:/Users/USER/Desktop/ENTRETIENS%20PREMATURITE/CSPS_FARAKAN/FOCUS_GROUPE-CSPS_FARAKAN.MP3time=1408400) Person 3: Effectivement, dans nos registres, il y a la partie de prématurés ça ressort et dans les rapports aussi, ça ressort. [00:23:39-5](http://localhost:2300/file=C:/Users/USER/Desktop/ENTRETIENS%20PREMATURITE/CSPS_FARAKAN/FOCUS_GROUPE-CSPS_FARAKAN.MP3time=1419500)

[00:23:39-5](http://localhost:2300/file=C:/Users/USER/Desktop/ENTRETIENS%20PREMATURITE/CSPS_FARAKAN/FOCUS_GROUPE-CSPS_FARAKAN.MP3time=1419500) Person 7: Oui dans les registres ça ressort, on écrit là-bas dans le registre d'accouchement. Si on a eu à référer l'enfant aussi dans le registre de référence, tout est mentionné. [00:23:56-6](http://localhost:2300/file=C:/Users/USER/Desktop/ENTRETIENS%20PREMATURITE/CSPS_FARAKAN/FOCUS_GROUPE-CSPS_FARAKAN.MP3time=1436600)

00:23:05-1 Interviewer: Donc nous allons poursuivre, est-ce que les informations sur la naissance prématurée sont enregistrées dans le registre d'accouchement ? 00:23:16-1

00:23:16-1 Personne 2: Oui, les accouchements prématurés sont enregistrés dans les registres d'accouchements. 00:23:22-3

00:23:22-3 Interviewer: Est-ce que cela ressort dans les rapports mensuels d'activités ? 00:23:24-2

00:23:24-2 Personne 2: Oui, ça ressort dans les rapports mensuels d'activité aussi. 00:23:28-4

00:23:28-4 Personne 3: Effectivement, dans nos registres, il y a la partie de prématurés ça ressort et dans les rapports aussi, ça ressort. 00:23:39-5

00:23:39-5 Personne 7: Oui dans les registres ça ressort, on écrit là-bas dans le registre d'accouchement. Si on a eu à référer l'enfant aussi dans le registre de référence, tout est mentionné. 00:23:56-6

**Interviewer (00:23:05-1):** Moving on, is information about premature birth recorded in the birth register?

**Person 2 (00:23:16-1):** Yes, premature births are recorded in the birth registers.

**Interviewer (00:23:22-3):** Does this information appear in the monthly activity reports?

**Person 2 (00:23:24-2):** Yes, information on premature births is included in the monthly reports as well.

**Person 3 (00:23:28-4):** Indeed, our registers have a specific section for premature babies, and this is also highlighted in our reports.

**Person 7 (00:23:39-5):** In the registers, premature births are clearly noted. We document it in the birth register, and if there is a need for referral, that information is also included.

### Major of Farakan health and social promotion center

[00:08:10-2](http://localhost:2300/file=C:/Users/USER/Desktop/ENTRETIENS%20PREMATURITE/CSPS_FARAKAN/MAJOR%20FARAKAN.MP3time=490200) Interviewer: Est ce que les informations sur la naissance prématurée sont enregistrées dans le registre d'accouchement? [00:08:15-6](http://localhost:2300/file=C:/Users/USER/Desktop/ENTRETIENS%20PREMATURITE/CSPS_FARAKAN/MAJOR%20FARAKAN.MP3time=495600)

[00:08:15-6](http://localhost:2300/file=C:/Users/USER/Desktop/ENTRETIENS%20PREMATURITE/CSPS_FARAKAN/MAJOR%20FARAKAN.MP3time=495600) Person 1: Oui [00:08:17-9](http://localhost:2300/file=C:/Users/USER/Desktop/ENTRETIENS%20PREMATURITE/CSPS_FARAKAN/MAJOR%20FARAKAN.MP3time=497900)

[00:08:17-9](http://localhost:2300/file=C:/Users/USER/Desktop/ENTRETIENS%20PREMATURITE/CSPS_FARAKAN/MAJOR%20FARAKAN.MP3time=497900) Interviewer: Est ce que ses informations ressortent dans le rapport mensuel d'activité? [00:08:22-8](http://localhost:2300/file=C:/Users/USER/Desktop/ENTRETIENS%20PREMATURITE/CSPS_FARAKAN/MAJOR%20FARAKAN.MP3time=502800)

[00:08:22-8](http://localhost:2300/file=C:/Users/USER/Desktop/ENTRETIENS%20PREMATURITE/CSPS_FARAKAN/MAJOR%20FARAKAN.MP3time=502800) Person 1: Le model de rapport que nous utilisons actuellement, la partie prématurité ne ressort pas.Mais dans les registres d'accouchements, il y en a. Puisque dans le rapport on dit " nombre de nouveaux nés à terme", donc cela ne ressort pas. [00:08:49-5](http://localhost:2300/file=C:/Users/USER/Desktop/ENTRETIENS%20PREMATURITE/CSPS_FARAKAN/MAJOR%20FARAKAN.MP3time=529500)

00:08:10-2 Interviewer: Est-ce que les informations sur la naissance prématurée sont enregistrées dans le registre d'accouchement ? 00:08:15-6

00:08:15-6 Personne 1: Oui. 00:08:17-9

00:08:17-9 Interviewer: Est-ce que ces informations ressortent dans le rapport mensuel d'activité ? 00:08:22-8

00:08:22-8 Personne 1: Le modèle de rapport que nous utilisons actuellement, la partie prématurité ne ressort pas. Mais dans les registres d'accouchements, il y en a. Puisque dans le rapport on dit 'nombre de nouveaux nés à terme', donc cela ne ressort pas. 00:08:49-5

**Interviewer (00:08:10-2):** Is information on premature birth recorded in the birth register?

**Person 1 (00:08:15-6):** Yes, it is.

**Interviewer (00:08:17-9):** Does this information appear in the monthly activity report?

**Person 1 (00:08:22-8):** In our current reporting template, details about prematurity are not highlighted. However, in the birth registers, we do record information on premature births. The monthly report primarily mentions the 'number of full-term newborns,' so prematurity data isn't explicitly noted there.

### Maternity manager of Farakan health and social promotion center

[00:12:33-4](http://localhost:2300/file=C:/Users/USER/Desktop/ENTRETIENS%20PREMATURITE/CSPS_FARAKAN/RESPONSABLE%20MATERNITE%20FARAKAN.MP3time=753400) Interviewer: Est-ce que les informations sur les naissances prématurées sont enregistrées dans le registre d'accouchement? [00:12:38-7](http://localhost:2300/file=C:/Users/USER/Desktop/ENTRETIENS%20PREMATURITE/CSPS_FARAKAN/RESPONSABLE%20MATERNITE%20FARAKAN.MP3time=758700)

[00:12:38-7](http://localhost:2300/file=C:/Users/USER/Desktop/ENTRETIENS%20PREMATURITE/CSPS_FARAKAN/RESPONSABLE%20MATERNITE%20FARAKAN.MP3time=758700) Person 1: Oui.Il y a la partie prématurée,si c'est un cas de prématuré on mentionne, il y a l'âge gestationnel même dans les registres d'accouchement en semaine. [00:12:49-1](http://localhost:2300/file=C:/Users/USER/Desktop/ENTRETIENS%20PREMATURITE/CSPS_FARAKAN/RESPONSABLE%20MATERNITE%20FARAKAN.MP3time=769100)

[00:12:49-1](http://localhost:2300/file=C:/Users/USER/Desktop/ENTRETIENS%20PREMATURITE/CSPS_FARAKAN/RESPONSABLE%20MATERNITE%20FARAKAN.MP3time=769100) Interviewer: Est ce que cela ressort dans le rapport d'activité mensuel? [00:12:53-0](http://localhost:2300/file=C:/Users/USER/Desktop/ENTRETIENS%20PREMATURITE/CSPS_FARAKAN/RESPONSABLE%20MATERNITE%20FARAKAN.MP3time=773000)

[00:12:53-0](http://localhost:2300/file=C:/Users/USER/Desktop/ENTRETIENS%20PREMATURITE/CSPS_FARAKAN/RESPONSABLE%20MATERNITE%20FARAKAN.MP3time=773000) Person 1: Dans les rapports mensuels? Oui,puisque c'est ce qui est dans les rapports qui sont dans le registre. [00:13:02-7](http://localhost:2300/file=C:/Users/USER/Desktop/ENTRETIENS%20PREMATURITE/CSPS_FARAKAN/RESPONSABLE%20MATERNITE%20FARAKAN.MP3time=782700)

00:12:33-4 Interviewer: Est-ce que les informations sur les naissances prématurées sont enregistrées dans le registre d'accouchement ? 00:12:38-7

00:12:38-7 Personne 1: Oui. Il y a la partie prématurée, si c'est un cas de prématuré on mentionne, il y a l'âge gestationnel même dans les registres d'accouchement en semaine. 00:12:49-1

00:12:49-1 Interviewer: Est-ce que cela ressort dans le rapport d'activité mensuel ? 00:12:53-0

00:12:53-0 Personne 1: Dans les rapports mensuels ? Oui, puisque c'est ce qui est dans les rapports qui sont dans le registre. 00:13:02-7

**Interviewer (00:12:33-4):** Is information on premature births recorded in the birth register?

**Person 1 (00:12:38-7):** Yes, it is. We specifically mention if a birth is premature in the birth register, including the gestational age.

**Interviewer (00:12:49-1):** Is this information also included in the monthly activity report?

**Person 1 (00:12:53-0):** In the monthly reports? Yes, it is. The data in our monthly reports come from what is recorded in the register.

# 5. Number of preterm births per month

### Focus group of Accart-ville urban medical center

[00:12:59-4](http://localhost:2300/file=C:/Users/USER/Desktop/ENTRETIENS%20PREMATURITE/CSPS-ACCART-VILLE/FOCUS%20GROUPE%20ACCART-VILLE-BON.mp3time=779400) Interviewer: Combien de bébés prématurés naissent chaque mois dans votre centre de santé ?

[00:13:05-6](http://localhost:2300/file=C:/Users/USER/Desktop/ENTRETIENS%20PREMATURITE/CSPS-ACCART-VILLE/FOCUS%20GROUPE%20ACCART-VILLE-BON.mp3time=785600) Person 2: nous pouvons nous referer au RMA(rapport mensuel d'activité) sinon c'est assez difficile à dire

[00:13:11-2](http://localhost:2300/file=C:/Users/USER/Desktop/ENTRETIENS%20PREMATURITE/CSPS-ACCART-VILLE/FOCUS%20GROUPE%20ACCART-VILLE-BON.mp3time=791200) Interviewer: mais une estimation? [00:13:13-9](http://localhost:2300/file=C:/Us)

[00:13:13-9](http://localhost:2300/file=C:/Users/USER/Desktop/ENTRETIENS%20PREMATURITE/CSPS-ACCART-VILLE/FOCUS%20GROUPE%20ACCART-VILLE-BON.mp3time=793900) Person 1: ce n'est pas beaucoup en tout cas 01 ou 02 accouchements prematurés par mois, ça ne depasse pas cela [00:13:19-3](http://localhost:2300/file=C:/Users/USER/Desktop/ENTRETIENS%20PREMATURITE/CSPS-ACCART-VILLE/FOCUS%20GROUPE%20ACCART-VILLE-BON.mp3time=799300)

[00:13:19-3](http://localhost:2300/file=C:/Users/USER/Desktop/ENTRETIENS) Person 3: moi je pense que ça depasse puisque je prends souvent le rapport pour examiner le niveau, je pense que cela vaut 10 à 15% de prematurités par mois,c'est un peu beaucoup puisque j'ai le rapport des neuf derniers mois à ma possession [00:13:47-5](http://localhost:2300/file=C:/Users/USER/Desktop/ENTRETIENS%20PREMATURITE/CSPS-ACCART-VILLE/FOCUS%20GROUPE%20ACCART-VILLE-BON.mp3time=827500)

[00:13:47-5](http://localho) Interviewer: d'accord nous aurons besoin par la suite, des données des sept derniers mois.nous pourrons repasser aprèspour prendre ses informations avec vous. [00:13:57-1](http://localhost:2300/file=C:/Users/USER/Desktop/ENTRETIENS%20PREMATURITE/CSPS-ACCART-VILLE/FOCUS%20GROUPE%20ACCART-VILLE-BON.mp3time=837100)

[00:13:57-1](http://localhost:2300/file=C:/Users/USER/Des) Person 3: d'accord. [00:13:59-3](http://localhost:2300/file=C:/Users/USER/Desktop/ENTRETIENS)

00:12:59-4 Interviewer: Combien de bébés prématurés naissent chaque mois dans votre centre de santé ?

00:13:05-6 Personne 2: Nous pouvons nous référer au RMA (rapport mensuel d'activité), sinon c'est assez difficile à dire.

00:13:11-2 Interviewer: Mais une estimation ? 00:13:13-9

00:13:13-9 Personne 1: Ce n'est pas beaucoup en tout cas, 01 ou 02 accouchements prématurés par mois, ça ne dépasse pas cela. 00:13:19-3

00:13:19-3 Personne 3: Moi, je pense que ça dépasse, puisque je prends souvent le rapport pour examiner le niveau, je pense que cela vaut 10 à 15% de prématurités par mois, c'est un peu beaucoup puisque j'ai le rapport des neuf derniers mois à ma possession. 00:13:47-5

00:13:47-5 Interviewer: D'accord, nous aurons besoin par la suite, des données des sept derniers mois. Nous pourrons repasser après pour prendre ces informations avec vous. 00:13:57-1

00:13:57-1 Personne 3: D'accord. 00:13:59-3

**Interviewer (00:12:59-4):** How many premature babies are born each month at your health center?

**Person 2 (00:13:05-6):** We would need to refer to the monthly activity report (RMA) for exact numbers. It's hard to say without checking.

**Interviewer (00:13:11-2):** Could you provide an estimate?

**Person 1 (00:13:13-9):** It's not a high number. On average, there are about one or two premature births per month. It doesn't usually exceed that.

**Person 3 (00:13:19-3):** I believe it's higher than that. Based on the reports I've reviewed, prematurity rates are about 10 to 15% per month. I have the reports for the last nine months.

**Interviewer (00:13:47-5):** Okay, we will need data from the last seven months. We'll return later to collect this information from you.

**Person 3 (00:13:57-1):** Okay.

### Manager of Accart-ville urban medical center

[00:09:47-6](http://localhost:2300/file=C:/Users/USER/Desktop/ENTRETIENS%20PREMATURITE/CSPS-ACCART-VILLE/RESPONSABLE%20CMU%20ACCART-VILLE.MP3time=587600) Interviewer: Maintenant combien de bébés prématurée naissent chaque mois au sein de votre centre de santé? [00:09:52-2](http://localhost:2300/file=C:/Users/USER/Desktop/ENTRETIENS%20PREMATURITE/CSPS-ACCART-VILLE/RESPONSABLE%20CMU%20ACCART-VILLE.MP3time=592200)

[00:09:52-2](http://localhost:2300/file=C:/Users/USER/Desktop/ENTRETIENS%20PREMATURITE/CSPS-ACCART-VILLE/RESPONSABLE%20CMU%20ACCART-VILLE.MP3time=592200)Person 1: C'est un peu difficile de dire puisque comme c'est si on doit prendre le registre, essayez de voir l'âge de certains, puisque quand on fait le rapport final, il n'y en a pas pour vous dire y a combien de prématurés on a,puisque généralement, quand on regarde de façon globale, c'est ça. Et pourtant, il faut prendre le registre, regarder, c'est à dire l'âge gestationnel personne par personne, pour essayer de déterminer s'il y avait une partie dans les rapports mensuels d'activité qui nous permettait de dire il y a tant de prématurés les gens allaient faire plus attention, mais maintenant les gens ne plus attention. Voilà. [00:10:27-8](http://localhost:2300/file=C:/Users/USER/Desktop/ENTRETIENS%20PREMATURITE/CSPS-ACCART-VILLE/RESPONSABLE%20CMU%20ACCART-VILLE.MP3time=627800)

[00:10:27-8](http://localhost:2300/file=C:/Users/USER/Desktop/ENTRETIENS%20PREMATURITE/CSPS-ACCART-VILLE/RESPONSABLE%20CMU%20ACCART-VILLE.MP3time=627800) Interviewer: j'aurai besoin des informations sur les naissances prématurés des sept derniers mois. Peut être que je pourrai voir avec le responsable de la maternité. [00:10:33-8](http://localhost:2300/file=C:/Users/USER/Desktop/ENTRETIENS%20PREMATURITE/CSPS-ACCART-VILLE/RESPONSABLE%20CMU%20ACCART-VILLE.MP3time=633800)

[00:10:33-8](http://localhost:2300/file=C:/Users/USER/Desktop/ENTRETIENS%20PREMATURITE/CSPS-ACCART-VILLE/RESPONSABLE%20CMU%20ACCART-VILLE.MP3time=633800) Person 1: Maternité. Même lui, Je ne pense pas, ça serait un peu difficile puisque comme je l'ai dit, en fait rapport mensuel nous dit on a eu combien d'accouchement dans le mois? et nous dit pas on a eu combien d'accouchement prématuré, pour avoir le nombre d'accouchement prématuré il faut qu'on prenne les données mêmes de l'accouchement de chaque personne et essayer de voir les paramètres . Est ce que cette dame a accouché à terme? Ou n'a pas accouché à terme. Quel était le poids? Quels étaient les autres paramètres? L'examen physique a montrer quoi? Donc il faut prendre femme par femme pour essayer d'examiner pour pouvoir le savoir. [00:11:14-4](http://localhost:2300/file=C:/Users/USER/Desktop/ENTRETIENS%20PREMATURITE/CSPS-ACCART-VILLE/RESPONSABLE%20CMU%20ACCART-VILLE.MP3time=674400)

00:11:14-4Person 1: De manière claire,dans le rapport final, il y en a pas en fait. [00:11:19-9](http://localhost:2300/file=C:/Users/USER/Desktop/ENTRETIENS%20PREMATURITE/CSPS-ACCART-VILLE/RESPONSABLE%20CMU%20ACCART-VILLE.MP3time=679900)

00:09:47-6 Interviewer: Maintenant, combien de bébés prématurés naissent chaque mois au sein de votre centre de santé ? 00:09:52-2

00:09:52-2 Personne 1: C'est un peu difficile de dire puisque, comme c'est, si on doit prendre le registre, essayer de voir l'âge de certains, puisque quand on fait le rapport final, il n'y en a pas pour vous dire combien de prématurés on a, puisque généralement, quand on regarde de façon globale, c'est ça. Et pourtant, il faut prendre le registre, regarder, c'est-à-dire l'âge gestationnel personne par personne, pour essayer de déterminer s'il y avait une partie dans les rapports mensuels d'activité qui nous permettait de dire il y a tant de prématurés, les gens allaient faire plus attention, mais maintenant les gens ne font plus attention. Voilà. 00:10:27-8

00:10:27-8 Interviewer: J'aurai besoin des informations sur les naissances prématurées des sept derniers mois. Peut-être que je pourrai voir avec le responsable de la maternité. 00:10:33-8

00:10:33-8 Personne 1: Maternité. Même lui, je ne pense pas, ça serait un peu difficile puisque comme je l'ai dit, en fait, le rapport mensuel nous dit on a eu combien d'accouchements dans le mois ? et ne nous dit pas on a eu combien d'accouchements prématurés, pour avoir le nombre d'accouchements prématurés il faut qu'on prenne les données mêmes de l'accouchement de chaque personne et essayer de voir les paramètres. Est-ce que cette dame a accouché à terme ? Ou n'a pas accouché à terme. Quel était le poids ? Quels étaient les autres paramètres ? L'examen physique a montré quoi ? Donc il faut prendre femme par femme pour essayer d'examiner pour pouvoir le savoir. 00:11:14-4

00:11:14-4 Personne 1: De manière claire, dans le rapport final, il n'y en a pas en fait. 00:11:19-9

**Interviewer (00:09:47-6):** How many premature babies are born each month at your health center?

**Person 1 (00:09:52-2):** It's difficult to give an exact number. Our current system doesn't specifically track the number of premature births in the final report. We would need to individually review the gestational age recorded in the birth register for each birth to determine this. If our monthly activity reports specifically asked for the number of premature births, it would encourage more careful tracking. As it stands, however, this level of detail is not routinely monitored.

**Interviewer (00:10:27-8):** I need information on premature births for the past seven months. Perhaps I can get this from the maternity manager.

**Person 1 (00:10:33-8):** Even for the maternity manager, it might be challenging. As I mentioned, our monthly reports indicate the total number of births, but not the number of premature births. To determine that, we'd need to analyze the birth data for each individual, checking gestational age, birth weight, and other parameters from the physical examination. This analysis would have to be done case by case to gather the information.

**Person 1 (00:11:14-4):** In summary, our final reports currently don't include specific data on premature births.

### Maternity manager of Accart-ville urban medical center

[00:12:17-9](http://localhost:2300/file=C:/Users/USER/Desktop/ENTRETIENS%20PREMATURITE/CSPS-ACCART-VILLE/RESPONSABLE%20MATERNITE%20CMU%20ACCART-VILLE.MP3time=737900) Interviewer: maintenant combien de bébés prématurés naissent chaque mois dans votre centre de santé ? [00:12:24-1](http://localhost:2300/file=C:/Users/USER/Desktop/ENTRETIENS%20PREMATURITE/CSPS-ACCART-VILLE/RESPONSABLE%20MATERNITE%20CMU%20ACCART-VILLE.MP3time=744100)

[00:12:24-1](http://localhost:2300/file=C:/Users/USER/Desktop/ENTRETIENS%20PREMATURITE/CSPS-ACCART-VILLE/RESPONSABLE%20MATERNITE%20CMU%20ACCART-VILLE.MP3time=744100) Person 1: on peut dire que beaucoup n'arrive pas à distinguer les faibles poids de naissance , les hypotrophes et la prematurité ce qui fait que c'est sous estimé mais en realité on a beaucoup de cas de prematurés. je peux dire que ça part de l'ordre de 5 à 10% par mois de nos accouchements. [00:12:53-6](http://localhost:2300/file=C:/Users/USER/Desktop/ENTRETIENS%20PREMATURITE/CSPS-ACCART-VILLE/RESPONSABLE%20MATERNITE%20CMU%20ACCART-VILLE.MP3time=773600)

[00:12:53-6](http://localhost:2300/file=C:/Users/USER/Desktop/ENTRETIENS%20PREMATURITE/CSPS-ACCART-VILLE/RESPONSABLE%20MATERNITE%20CMU%20ACCART-VILLE.MP3time=773600) Interviewer: j'aurai besoin des estimations des sept derniers mois au cas ou vous voudriez bien consulter le registre [00:13:03-2](http://localhost:2300/file=C:/Users/USER/Desktop/ENTRETIENS%20PREMATURITE/CSPS-ACCART-VILLE/RESPONSABLE%20MATERNITE%20CMU%20ACCART-VILLE.MP3time=783200)

[00:13:03-2](http://localhost:2300/file=C:/Users/USER/Desktop/ENTRETIENS%20PREMATURITE/CSPS-ACCART-VILLE/RESPONSABLE%20MATERNITE%20CMU%20ACCART-VILLE.MP3time=783200)Person 1: ok il n' y a pas de problème nous avons les registres et les rapports mensuels d'activités le (RMA) des neuf derniers mois à ma possession. comme je viens de faire une activité je les ai exploité pour preparer mes diapositives [00:13:20-5](http://localhost:2300/file=C:/Users/USER/Desktop/ENTRETIENS%20PREMATURITE/CSPS-ACCART-VILLE/RESPONSABLE%20MATERNITE%20CMU%20ACCART-VILLE.MP3time=800500)

00:12:17-9 Interviewer: Maintenant, combien de bébés prématurés naissent chaque mois dans votre centre de santé ? 00:12:24-1

00:12:24-1 Personne 1: On peut dire que beaucoup n'arrivent pas à distinguer les faibles poids de naissance, les hypotrophes et la prématurité, ce qui fait que c'est sous-estimé, mais en réalité on a beaucoup de cas de prématurés. Je peux dire que ça part de l'ordre de 5 à 10% par mois de nos accouchements. 00:12:53-6

00:12:53-6 Interviewer: J'aurai besoin des estimations des sept derniers mois au cas où vous voudriez bien consulter le registre. 00:13:03-2

00:13:03-2 Personne 1: Ok, il n'y a pas de problème, nous avons les registres et les rapports mensuels d'activités, le (RMA) des neuf derniers mois à ma possession. Comme je viens de faire une activité, je les ai exploités pour préparer mes diapositives. 00:13:20-5

**Interviewer (00:12:17-9):** Can you tell me how many premature babies are born each month at your health center?

**Person 1 (00:12:24-1):** It's challenging to give an exact figure because there's often confusion between low birth weight, hypotrophy, and prematurity, leading to underestimation. However, we do see a significant number of premature births. I would estimate it to be around 5 to 10% of our monthly deliveries.

**Interviewer (00:12:53-6):** I need the estimates for the past seven months. Could you check the register for this information?

**Person 1 (00:13:03-2):** Sure, there's no problem with that. We have both the logs and the monthly activity reports (RMA) for the last nine months. I recently used them to prepare my slides for an activity.

### Focus group of Do medical center with surgical branch

[00:13:15-9](http://localhost:2300/file=C:/Users/USER/Desktop/ENTRETIENS%20PREMATURITE/CMA-DO/FOCUS%20GROUPE%20CMA-DO.mp3time=795900) Interviewer: Ok. Combien de bébés prématurés naissent chaque mois au sein de votre centre de santé ? [00:13:23-9](http://localhost:2300/file=C:/Users/USER/Desktop/ENTRETIENS%20PREMATURITE/CMA-DO/FOCUS%20GROUPE%20CMA-DO.mp3time=803900)

[00:13:23-9](http://localhost:2300/file=C:/Users/USER/Desktop/ENTRETIENS%20PREMATURITE/CMA-DO/FOCUS%20GROUPE%20CMA-DO.mp3time=803900) Person 6: Les accouchements prématurés, ça dépasse pas 30 à 40. Ça dépasse pas 30 à 40. [00:13:53-0](http://localhost:2300/file=C:/Users/USER/Desktop/ENTRETIENS%20PREMATURITE/CMA-DO/FOCUS%20GROUPE%20CMA-DO.mp3time=833000)

[00:13:53-0](http://localhost:2300/file=C:/Users/USER/Desktop/ENTRETIENS%20PREMATURITE/CMA-DO/FOCUS%20GROUPE%20CMA-DO.mp3time=833000) Person 1: Ça dépend des mois. Il ya des mois ça depasse et il y a des mois ça ne depasse pas. C'est la moyenne [00:14:05-8](http://localhost:2300/file=C:/Users/USER/Desktop/ENTRETIENS%20PREMATURITE/CMA-DO/FOCUS%20GROUPE%20CMA-DO.mp3time=845800)

[00:14:05-8](http://localhost:2300/file=C:/Users/USER/Desktop/ENTRETIENS%20PREMATURITE/CMA-DO/FOCUS%20GROUPE%20CMA-DO.mp3time=845800) Interviewer: Est ce que nous pouvons avoir les informations sur les naissances prématurées des sept derniers mois? [00:14:09-2](http://localhost:2300/file=C:/Users/USER/Desktop/ENTRETIENS%20PREMATURITE/CMA-DO/FOCUS%20GROUPE%20CMA-DO.mp3time=849200)

[00:14:09-2](http://localhost:2300/file=C:/Users/USER/Desktop/ENTRETIENS%20PREMATURITE/CMA-DO/FOCUS%20GROUPE%20CMA-DO.mp3time=849200) Person 6: C'est dans le rapport mensuel d'activité, avec la responsable SUS (Surveillance Unité de Soins). [00:14:24-7](http://localhost:2300/file=C:/Users/USER/Desktop/ENTRETIENS%20PREMATURITE/CMA-DO/FOCUS%20GROUPE%20CMA-DO.mp3time=864700)

00:13:15-9 Interviewer: Ok. Combien de bébés prématurés naissent chaque mois au sein de votre centre de santé ? 00:13:23-9

00:13:23-9 Personne 6: Les accouchements prématurés, ça dépasse pas 30 à 40. Ça ne dépasse pas 30 à 40. 00:13:53-0

00:13:53-0 Personne 1: Ça dépend des mois. Il y a des mois ça dépasse et il y a des mois ça ne dépasse pas. C'est la moyenne. 00:14:05-8

00:14:05-8 Interviewer: Est-ce que nous pouvons avoir les informations sur les naissances prématurées des sept derniers mois ? 00:14:09-2

00:14:09-2 Personne 6: C'est dans le rapport mensuel d'activité, avec la responsable SUS (Surveillance Unité de Soins). 00:14:24-7

**Interviewer (00:13:15-9):** How many premature babies are born each month at your health center?

**Person 6 (00:13:23-9):** The number of premature births usually doesn't exceed 30 to 40 per month.

**Person 1 (00:13:53-0):** It varies from month to month. Some months we might exceed that number, while in others we don't. That's just an average.

**Interviewer (00:14:05-8):** Can we access information on premature births for the last seven months?

**Person 6 (00:14:09-2):** Yes, that information can be found in the monthly activity reports, which are managed by the SUS (Care Unit Monitoring) manager.

### Gynecologist 1

[00:08:31-3](http://localhost:2300/file=C:/Users/USER/Desktop/ENTRETIENS%20PREMATURITE/CMA-DO/GENYCOLOGUE%2001.mp3time=511300) Interviewer: A peu près combien de bébés naissent chaque mois au sein de votre centre de santé ? [00:08:37-8](http://localhost:2300/file=C:/Users/USER/Desktop/ENTRETIENS%20PREMATURITE/CMA-DO/GENYCOLOGUE%2001.mp3time=517800)

[00:08:37-8](http://localhost:2300/file=C:/Users/USER/Desktop/ENTRETIENS%20PREMATURITE/CMA-DO/GENYCOLOGUE%2001.mp3time=517800) Person 1: L'estimation, si je veux vous donner un chiffre exact, je risque de me tromper.Peut-être si on voit les statistiques, mais à ma connaissance, si je prends le mois passé, peut-être qu'on a eu deux ou trois prématurés. [00:08:56-3](http://localhost:2300/file=C:/Users/USER/Desktop/ENTRETIENS%20PREMATURITE/CMA-DO/GENYCOLOGUE%2001.mp3time=536300)

[00:08:56-3](http://localhost:2300/file=C:/Users/USER/Desktop/ENTRETIENS%20PREMATURITE/CMA-DO/GENYCOLOGUE%2001.mp3time=536300) Est-ce que c'est possible d'avoir les informations sur les naissances prématurés des sept derniers mois ? [00:09:01-7](http://localhost:2300/file=C:/Users/USER/Desktop/ENTRETIENS%20PREMATURITE/CMA-DO/GENYCOLOGUE%2001.mp3time=541700)

[00:09:01-7](http://localhost:2300/file=C:/Users/USER/Desktop/ENTRETIENS%20PREMATURITE/CMA-DO/GENYCOLOGUE%2001.mp3time=541700) Person 1: Oui, les naissances prématurées, c'est possible. On va collecter l'information. [00:09:07-6](http://localhost:2300/file=C:/Users/USER/Desktop/ENTRETIENS%20PREMATURITE/CMA-DO/GENYCOLOGUE%2001.mp3time=547600)

[00:09:07-6](http://localhost:2300/file=C:/Users/USER/Desktop/ENTRETIENS%20PREMATURITE/CMA-DO/GENYCOLOGUE%2001.mp3time=547600) Interviewer: Est-ce que dans les registres, il y a une mention spécifique pour les naissances prématurées ? [00:09:12-7](http://localhost:2300/file=C:/Users/USER/Desktop/ENTRETIENS%20PREMATURITE/CMA-DO/GENYCOLOGUE%2001.mp3time=552700)

00:09:12-7 Personne 1: Oui.

00:08:31-3 Interviewer: À peu près combien de bébés naissent chaque mois au sein de votre centre de santé ? 00:08:37-8

00:08:37-8 Personne 1: L'estimation, si je veux vous donner un chiffre exact, je risque de me tromper. Peut-être si on voit les statistiques, mais à ma connaissance, si je prends le mois passé, peut-être qu'on a eu deux ou trois prématurés. 00:08:56-3

00:08:56-3 Interviewer: Est-ce que c'est possible d'avoir les informations sur les naissances prématurées des sept derniers mois ? 00:09:01-7

00:09:01-7 Personne 1: Oui, les naissances prématurées, c'est possible. On va collecter l'information. 00:09:07-6

00:09:07-6 Interviewer: Est-ce que dans les registres, il y a une mention spécifique pour les naissances prématurées ? 00:09:12-7

00:09:12-7 Personne 1: Oui.

**Interviewer (00:08:31-3):** Approximately how many babies are born each month at your health center?

**Person 1 (00:08:37-8):** To give you an exact figure would be difficult without consulting the statistics. But from what I recall, last month, we had about two or three premature babies.

**Interviewer (00:08:56-3):** Is it possible to get information on premature births over the last seven months?

**Person 1 (00:09:01-7):** Yes, we can collect that information on premature births.

**Interviewer (00:09:07-6):** Is there a specific section in the registers for recording premature births?

**Person 1 (00:09:12-7):** Yes, there is.

### Gynecologist 2

[00:12:25-2](http://localhost:2300/file=C:/Users/USER/Desktop/ENTRETIENS%20PREMATURITE/CMA-DO/GENYCOLOGUE%2002.mp3time=745200) Interviewer: En estimation, combien de bébés prématurés naissent chaque mois dans votre centre de santé? [00:12:33-7](http://localhost:2300/file=C:/Users/USER/Desktop/ENTRETIENS%20PREMATURITE/CMA-DO/GENYCOLOGUE%2002.mp3time=753700)

[00:12:33-7](http://localhost:2300/file=C:/Users/USER/Desktop/ENTRETIENS%20PREMATURITE/CMA-DO/GENYCOLOGUE%2002.mp3time=753700) Person 1: On a en moyenne une centaine de naissances par mois. Non, par semaine. [00:12:43-0](http://localhost:2300/file=C:/Users/USER/Desktop/ENTRETIENS%20PREMATURITE/CMA-DO/GENYCOLOGUE%2002.mp3time=763000)

[00:12:43-0](http://localhost:2300/file=C:/Users/USER/Desktop/ENTRETIENS%20PREMATURITE/CMA-DO/GENYCOLOGUE%2002.mp3time=763000) Interviewer: Des bébés prématurés ? [00:12:43-8](http://localhost:2300/file=C:/Users/USER/Desktop/ENTRETIENS%20PREMATURITE/CMA-DO/GENYCOLOGUE%2002.mp3time=763800)

[00:12:43-8](http://localhost:2300/file=C:/Users/USER/Desktop/ENTRETIENS%20PREMATURITE/CMA-DO/GENYCOLOGUE%2002.mp3time=763800) Person 1: Non, non, non. Confondus. De façon globale ? De façon globale. Maintenant, à l'intérieur, on n'a pas fait des études statistiques pour voir quelle est la part des naissances prématurées. C'est difficile à dire. Parce qu'on ne s'est pas assis pour faire un calcul, et voir quel est le pourcentage des accouchements prématurés. Ça, c'est un peu difficile à dire. Mais peut-être si on se base sur les régistres d'accouchements, de façon rétrospective, on peut retrouver les éléments qu'on recherche. [00:13:22-7](http://localhost:2300/file=C:/Users/USER/Desktop/ENTRETIENS%20PREMATURITE/CMA-DO/GENYCOLOGUE%2002.mp3time=802700)

00:12:25-2 Interviewer: En estimation, combien de bébés prématurés naissent chaque mois dans votre centre de santé ? 00:12:33-7

00:12:33-7 Personne 1: On a en moyenne une centaine de naissances par mois. Non, par semaine. 00:12:43-0

00:12:43-0 Interviewer: Des bébés prématurés ? 00:12:43-8

00:12:43-8 Personne 1: Non, non, non. Confondus. De façon globale ? De façon globale. Maintenant, à l'intérieur, on n'a pas fait des études statistiques pour voir quelle est la part des naissances prématurées. C'est difficile à dire. Parce qu'on ne s'est pas assis pour faire un calcul, et voir quel est le pourcentage des accouchements prématurés. Ça, c'est un peu difficile à dire. Mais peut-être si on se base sur les registres d'accouchements, de façon rétrospective, on peut retrouver les éléments qu'on recherche. 00:13:22-7

**Interviewer (00:12:25-2):** Can you give an estimate of how many premature babies are born each month at your health center?

**Person 1 (00:12:33-7):** We average around a hundred births per week.

**Interviewer (00:12:43-0):** And out of these, how many are premature babies?

**Person 1 (00:12:43-8):** No, not a hundred premature births, I got confused. That's the overall number of births. As for premature births, we haven't conducted statistical studies to determine their proportion. It's difficult to provide an exact figure because we haven't done the calculations yet. However, if we were to examine the birth registers retrospectively, we might be able to find the information we need.

### Chief physician of Do health district

[00:18:04-4](http://localhost:2300/file=C:/Users/USER/Desktop/ENTRETIENS%20PREMATURITE/CMA-DO/MCD-DO.mp3time=1084400) Interviewer: Maintenant, approximativement, combien de bébés naissent chaque mois, des bébés prématurés naissent chaque mois? [00:18:12-9](http://localhost:2300/file=C:/Users/USER/Desktop/ENTRETIENS%20PREMATURITE/CMA-DO/MCD-DO.mp3time=1092900)

[00:18:12-9](http://localhost:2300/file=C:/Users/USER/Desktop/ENTRETIENS%20PREMATURITE/CMA-DO/MCD-DO.mp3time=1092900) Person 1: On ne le collecte pas. On ne le collecte pas. C'est ce que j'ai dit, j'ai demandé aussi, il y a des gens qui nous ont demandé ça. Peut-être, s'ils viennent, je pourrais fournir ça dans le cadre de leur étude. Ça peut permettre de faire une extrapolation, sinon ce n'est pas un truc qui est collecté classiquement. [00:18:33-4](http://localhost:2300/file=C:/Users/USER/Desktop/ENTRETIENS%20PREMATURITE/CMA-DO/MCD-DO.mp3time=1113400)

00:18:04-4 Interviewer: Maintenant, approximativement, combien de bébés naissent chaque mois, des bébés prématurés naissent chaque mois ? 00:18:12-9

00:18:12-9 Personne 1: On ne le collecte pas. On ne le collecte pas. C'est ce que j'ai dit, j'ai demandé aussi, il y a des gens qui nous ont demandé ça. Peut-être, s'ils viennent, je pourrais fournir ça dans le cadre de leur étude. Ça peut permettre de faire une extrapolation, sinon ce n'est pas un truc qui est collecté classiquement. 00:18:33-4

**Interviewer (00:18:04-4):** Approximately, how many premature babies are born each month at your health center?

**Person 1 (00:18:12-9):** We don’t specifically collect that data. It's not part of our traditional data collection. I've had similar inquiries before, and I've mentioned that we don't track this information routinely. However, if someone needed this information for a study, I could potentially help them gather it, allowing for an extrapolation. But as it stands, we don’t have this data readily available.

### Manager for maternity care unit of Do medical center with surgical branch

[00:13:54-7](http://localhost:2300/file=C:/Users/USER/Desktop/ENTRETIENS%20PREMATURITE/CMA-DO/RESPONSABLE%20UNITE%20DE%20SOIN%20CMA-DO.mp3time=834700) Interviewer: D'accord. Maintenant, Combien de bébés prématurés naissent chaque mois dans votre centre de santé ici? Une estimation? [00:14:02-6](http://localhost:2300/file=C:/Users/USER/Desktop/ENTRETIENS%20PREMATURITE/CMA-DO/RESPONSABLE%20UNITE%20DE%20SOIN%20CMA-DO.mp3time=842600)

[00:14:02-6](http://localhost:2300/file=C:/Users/USER/Desktop/ENTRETIENS%20PREMATURITE/CMA-DO/RESPONSABLE%20UNITE%20DE%20SOIN%20CMA-DO.mp3time=842600) Person 1: Une estimation. Environ, on peut dire, bon... On peut avoir 10. Je vais aller avec 10. Voilà.On peut avoir 10. [00:14:16-0](http://localhost:2300/file=C:/Users/USER/Desktop/ENTRETIENS%20PREMATURITE/CMA-DO/RESPONSABLE%20UNITE%20DE%20SOIN%20CMA-DO.mp3time=856000)

00:13:54-7 Interviewer: D'accord. Maintenant, combien de bébés prématurés naissent chaque mois dans votre centre de santé ici ? Une estimation ? 00:14:02-6

00:14:02-6 Personne 1: Une estimation. Environ, on peut dire, bon... On peut avoir 10. Je vais aller avec 10. Voilà. On peut avoir 10. 00:14:16-0

**Interviewer (00:13:54-7):** Okay. Can you estimate how many premature babies are born each month at your health center?

**Person 1 (00:14:02-6):** As an estimate, I would say we have about 10 premature births per month. I'll go with 10 as the approximate number.

### Focus group of Colma1 health and social promotion center

[00:13:40-5](http://localhost:2300/file=C:/Users/USER/Desktop/ENTRETIENS%20PREMATURITE/CSPS-COLMA1/FOCUS%20GROUPE%20COLMA1.mp3time=820500) Interviewer: Maintenant, combien de bébés prématurés naissent chaque mois dans votre centre de santé? [00:13:55-7](http://localhost:2300/file=C:/Users/USER/Desktop/ENTRETIENS%20PREMATURITE/CSPS-COLMA1/FOCUS%20GROUPE%20COLMA1.mp3time=835700)

[00:13:55-7](http://localhost:2300/file=C:/Users/USER/Desktop/ENTRETIENS%20PREMATURITE/CSPS-COLMA1/FOCUS%20GROUPE%20COLMA1.mp3time=835700) Person 2: Une estimation en terme de naissances prématurées, il y a des mois ou on peut avoir deux, dès fois il n y en a même pas. [00:14:01-5](http://localhost:2300/file=C:/Users/USER/Desktop/ENTRETIENS%20PREMATURITE/CSPS-COLMA1/FOCUS%20GROUPE%20COLMA1.mp3time=841500)

[00:14:01-5](http://localhost:2300/file=C:/Users/USER/Desktop/ENTRETIENS%20PREMATURITE/CSPS-COLMA1/FOCUS%20GROUPE%20COLMA1.mp3time=841500) Person 3: Nous notre dernier garde nous avons eu un cas de prématuré [00:14:13-9](http://localhost:2300/file=C:/Users/USER/Desktop/ENTRETIENS%20PREMATURITE/CSPS-COLMA1/FOCUS%20GROUPE%20COLMA1.mp3time=853900)

00:13:40-5 Interviewer: Maintenant, combien de bébés prématurés naissent chaque mois dans votre centre de santé ? 00:13:55-7

00:13:55-7 Personne 2: Une estimation en termes de naissances prématurées, il y a des mois où on peut avoir deux, des fois il n'y en a même pas. 00:14:01-5

00:14:01-5 Personne 3: Nous, notre dernier garde, nous avons eu un cas de prématuré. 00:14:13-9

**Interviewer (00:13:40-5):** Can you tell me how many premature babies are typically born each month at your health center?

**Person 2 (00:13:55-7):** As an estimate, the number of premature births varies. Some months, we might have two cases, and other times, there might not be any.

**Person 3 (00:14:01-5):** During our last shift, we had one case of a premature birth.

### Major of Colma1 health and social promotion center

[00:09:53-4](http://localhost:2300/file=C:/Users/USER/Desktop/ENTRETIENS%20PREMATURITE/CSPS-COLMA1/MAJOR%20CSPS%20COLMA1.MP3time=593400) Interviewer: combien de bébés prématurés naissent chaque mois dans votre centre de santé? [00:09:58-6](http://localhost:2300/file=C:/Users/USER/Desktop/ENTRETIENS%20PREMATURITE/CSPS-COLMA1/MAJOR%20CSPS%20COLMA1.MP3time=598600)

[00:09:58-6](http://localhost:2300/file=C:/Users/USER/Desktop/ENTRETIENS%20PREMATURITE/CSPS-COLMA1/MAJOR%20CSPS%20COLMA1.MP3time=598600) Person 1: combien? [00:09:59-1](http://localhost:2300/file=C:/Users/USER/Desktop/ENTRETIENS%20PREMATURITE/CSPS-COLMA1/MAJOR%20CSPS%20COLMA1.MP3time=599100)

[00:09:59-1](http://localhost:2300/file=C:/Users/USER/Desktop/ENTRETIENS%20PREMATURITE/CSPS-COLMA1/MAJOR%20CSPS%20COLMA1.MP3time=599100) Interviewer: oui même en terme d'estimation [00:10:00-6](http://localhost:2300/file=C:/Users/USER/Desktop/ENTRETIENS%20PREMATURITE/CSPS-COLMA1/MAJOR%20CSPS%20COLMA1.MP3time=600600)

[00:10:00-6](http://localhost:2300/file=C:/Users/USER/Desktop/ENTRETIENS%20PREMATURITE/CSPS-COLMA1/MAJOR%20CSPS%20COLMA1.MP3time=600600) Person 1: bon, euh d'accord.le rapport mensuel ne relève pas de façon manifeste , c'est à dire que l'Item ne mantionne pas prématurés à part en tant que tel, par exemple on relève Nombre de nouveaux nés à terme moins de 2kg500, on estime que la grossesse est à terme et les enfants sont nés de faible poids. Par contre prématurés directement, il n y a pas une ligne specifique dans le rapport qui repertorie ses naissances là.donc nous n'allons pas voire prématuré dans le rapport,mais c'est dans le registre d'accouchement qu'il faut aller compterpour pouvoir avoir une idée. [00:11:20-0](http://localhost:2300/file=C:/Users/USER/Desktop/ENTRETIENS%20PREMATURITE/CSPS-COLMA1/MAJOR%20CSPS%20COLMA1.MP3time=680000)

[00:11:20-0](http://localhost:2300/file=C:/Users/USER/Desktop/ENTRETIENS%20PREMATURITE/CSPS-COLMA1/MAJOR%20CSPS%20COLMA1.MP3time=680000) Interviewer: ok donc je pourrai avoir ses informations un peu plus tard. [00:11:20-8](http://localhost:2300/file=C:/Users/USER/Desktop/ENTRETIENS%20PREMATURITE/CSPS-COLMA1/MAJOR%20CSPS%20COLMA1.MP3time=680800)

[00:11:20-8](http://localhost:2300/file=C:/Users/USER/Desktop/ENTRETIENS%20PREMATURITE/CSPS-COLMA1/MAJOR%20CSPS%20COLMA1.MP3time=680800) Person 1: oui oui les registres sont disponibles. [00:11:22-1](http://localhost:2300/file=C:/Users/USER/Desktop/ENTRETIENS%20PREMATURITE/CSPS-COLMA1/MAJOR%20CSPS%20COLMA1.MP3time=682100)

00:09:53-4 Interviewer: Combien de bébés prématurés naissent chaque mois dans votre centre de santé ? 00:09:58-6

00:09:58-6 Personne 1: Combien ? 00:09:59-1

00:09:59-1 Interviewer: Oui, même en termes d'estimation. 00:10:00-6

00:10:00-6 Personne 1: Bon, euh d'accord. Le rapport mensuel ne relève pas de façon manifeste, c'est-à-dire que l'item ne mentionne pas prématurés à part en tant que tel, par exemple on relève nombre de nouveau-nés à terme moins de 2kg500, on estime que la grossesse est à terme et les enfants sont nés de faible poids. Par contre, les prématurés directement, il n'y a pas une ligne spécifique dans le rapport qui répertorie ces naissances là. Donc nous n'allons pas voir prématuré dans le rapport, mais c'est dans le registre d'accouchement qu'il faut aller compter pour pouvoir avoir une idée. 00:11:20-0

00:11:20-0 Interviewer: Ok donc je pourrai avoir ces informations un peu plus tard. 00:11:20-8

00:11:20-8 Personne 1: Oui, oui, les registres sont disponibles. 00:11:22-1

**Interviewer (00:09:53-4):** Can you estimate how many premature babies are born each month at your health center?

**Person 1 (00:09:58-6):** How many?

**Interviewer (00:09:59-1):** Yes, even just an estimation.

**Person 1 (00:10:00-6):** Well, the monthly report doesn’t clearly specify premature births. For instance, we note the number of full-term newborns weighing less than 2.5kg, implying that these are full-term but low-weight births. However, there isn't a specific line in the report that lists premature births separately. We don't see a separate count for premature births in the report, but we can find this information in the birth register.

**Interviewer (00:11:20-0):** Okay, so I can gather this information later?

**Person 1 (00:11:20-8):** Yes, the registers with this information are available.

### Maternity manager of Colma1 health and social promotion center

[00:18:22-6](http://localhost:2300/file=C:/Users/USER/Desktop/ENTRETIENS%20PREMATURITE/CSPS-COLMA1/RESPONSABLE%20MATERNITE-COLMA1.mp3time=1102600) Interviewer: Combien de bébés prématurés naissent chaque mois au sein de votre centre de santé ? [00:18:27-3](http://localhost:2300/file=C:/Users/USER/Desktop/ENTRETIENS%20PREMATURITE/CSPS-COLMA1/RESPONSABLE%20MATERNITE-COLMA1.mp3time=1107300)

[00:18:27-3](http://localhost:2300/file=C:/Users/USER/Desktop/ENTRETIENS%20PREMATURITE/CSPS-COLMA1/RESPONSABLE%20MATERNITE-COLMA1.mp3time=1107300) Person 1: Chaque moi ? [00:18:28-6](http://localhost:2300/file=C:/Users/USER/Desktop/ENTRETIENS%20PREMATURITE/CSPS-COLMA1/RESPONSABLE%20MATERNITE-COLMA1.mp3time=1108600)

[00:18:28-6](http://localhost:2300/file=C:/Users/USER/Desktop/ENTRETIENS%20PREMATURITE/CSPS-COLMA1/RESPONSABLE%20MATERNITE-COLMA1.mp3time=1108600) Interviewer: une estimation. [00:18:29-5](http://localhost:2300/file=C:/Users/USER/Desktop/ENTRETIENS%20PREMATURITE/CSPS-COLMA1/RESPONSABLE%20MATERNITE-COLMA1.mp3time=1109500)

[00:18:29-5](http://localhost:2300/file=C:/Users/USER/Desktop/ENTRETIENS%20PREMATURITE/CSPS-COLMA1/RESPONSABLE%20MATERNITE-COLMA1.mp3time=1109500) Person 1: Une estimation. Je sais que sur 100 femmes qui accouchent, il y a cinq à dix accouchements prématurés. Ça s'est d'emblée, c'est comme ça, cinq à dix. Mais ici, c'est que je ne pourrais pas parler autrement que dans le registre. Souvent, on peut avoir deux dans le mois. Souvent, l'estimation, comme vous l'avez dit, il y a des gens qui vont pour la... Par exemple, quand l'enfant sort avec un bon score d'Avgard et que l'enfant n'a pas de problème de tété, les gens peuvent mettre ça dans les hypothrophes, dans les faits pour faible poids de naissance, par exemple. Mais ce qu'il y a dans le registre, je pense que ça ne va pas dépasser par mois deux si c'est beaucoup trois. Mais à quel moment, par exemple, on a beaucoup d'accouchements prématurés ? C'est pendant l'hivernage. Parce que les accès PALUS font vraiment des ravages par rapport aux avortements, aux accouchements prématurés. Même s'il y a d'autres infections qui sortent dedans aussi. [00:19:41-7](http://localhost:2300/file=C:/Users/USER/Desktop/ENTRETIENS%20PREMATURITE/CSPS-COLMA1/RESPONSABLE%20MATERNITE-COLMA1.mp3time=1181700)

00:18:22-6 Interviewer: Combien de bébés prématurés naissent chaque mois au sein de votre centre de santé ? 00:18:27-3

00:18:27-3 Personne 1: Chaque mois ? 00:18:28-6

00:18:28-6 Interviewer: Une estimation. 00:18:29-5

00:18:29-5 Personne 1: Une estimation. Je sais que sur 100 femmes qui accouchent, il y a cinq à dix accouchements prématurés. Ça, c'est d'emblée, c'est comme ça, cinq à dix. Mais ici, c'est que je ne pourrais pas parler autrement que dans le registre. Souvent, on peut avoir deux dans le mois. Souvent, l'estimation, comme vous l'avez dit, il y a des gens qui vont pour la... Par exemple, quand l'enfant sort avec un bon score d'Apgar et que l'enfant n'a pas de problème de téter, les gens peuvent mettre ça dans les hypotrophes, dans les faits pour faible poids de naissance, par exemple. Mais ce qu'il y a dans le registre, je pense que ça ne va pas dépasser par mois deux, si c'est beaucoup trois. Mais à quel moment, par exemple, on a beaucoup d'accouchements prématurés ? C'est pendant l'hivernage. Parce que les accès PALU font vraiment des ravages par rapport aux avortements, aux accouchements prématurés. Même s'il y a d'autres infections qui sortent dedans aussi. 00:19:41-7

**Interviewer (00:18:22-6):** Can you estimate how many premature babies are born each month at your health center?

**Person 1 (00:18:27-3):** Every month?

**Interviewer (00:18:28-6):** Yes, just an estimate.

**Person 1 (00:18:29-5):** As an estimate, out of 100 women who give birth, around five to ten are premature births. That’s a general figure. In terms of monthly numbers, it’s often around two, but it varies. In our register, I don't think the number of premature births exceeds two or three a month. However, during the winter, the number of premature births tends to increase due to complications from conditions like malaria and other infections. These can lead to an uptick in premature births as well as abortions.

### Focus group of Farakan health and social promotion center

[00:23:56-6](http://localhost:2300/file=C:/Users/USER/Desktop/ENTRETIENS%20PREMATURITE/CSPS_FARAKAN/FOCUS_GROUPE-CSPS_FARAKAN.MP3time=1436600) Interviewer: Ok, nous allons poursuivre.Combien de bébés prématurés naissent chaque mois dans votre centre de santé ? [00:24:10-8](http://localhost:2300/file=C:/Users/USER/Desktop/ENTRETIENS%20PREMATURITE/CSPS_FARAKAN/FOCUS_GROUPE-CSPS_FARAKAN.MP3time=1450800)

[00:24:10-8](http://localhost:2300/file=C:/Users/USER/Desktop/ENTRETIENS%20PREMATURITE/CSPS_FARAKAN/FOCUS_GROUPE-CSPS_FARAKAN.MP3time=1450800) Person 6: En tout cas dans notre CSPS ici, il y a des mois même qu'on peut ne pas avoir de la prématurité.Et si on veut bien voir dans 6 mois comme ça si on a trop eu ces 3 ou 2. [00:24:35-6](http://localhost:2300/file=C:/Users/USER/Desktop/ENTRETIENS%20PREMATURITE/CSPS_FARAKAN/FOCUS_GROUPE-CSPS_FARAKAN.MP3time=1475600)

[00:24:35-6](http://localhost:2300/file=C:/Users/USER/Desktop/ENTRETIENS%20PREMATURITE/CSPS_FARAKAN/FOCUS_GROUPE-CSPS_FARAKAN.MP3time=1475600) Person 8: Les accouchements prématurés, vraiment, sont très rares.Ça existe, mais c'est très rare. [00:24:46-9](http://localhost:2300/file=C:/Users/USER/Desktop/ENTRETIENS%20PREMATURITE/CSPS_FARAKAN/FOCUS_GROUPE-CSPS_FARAKAN.MP3time=1486900)

[00:24:46-9](http://localhost:2300/file=C:/Users/USER/Desktop/ENTRETIENS%20PREMATURITE/CSPS_FARAKAN/FOCUS_GROUPE-CSPS_FARAKAN.MP3time=1486900) Person 4: Depuis lors de la CPN on Conseille bien aux femmes de biens manger pour éviter la prématurité des enfants.Voilà on les conseille très bien, donc ce qui fait qu'on est moins de bébé prématuré chez nous aussi. [00:25:09-0](http://localhost:2300/file=C:/Users/USER/Desktop/ENTRETIENS%20PREMATURITE/CSPS_FARAKAN/FOCUS_GROUPE-CSPS_FARAKAN.MP3time=1509000)

00:23:56-6 Interviewer: Ok, nous allons poursuivre. Combien de bébés prématurés naissent chaque mois dans votre centre de santé ? 00:24:10-8

00:24:10-8 Personne 6: En tout cas dans notre CSPS ici, il y a des mois même qu'on peut ne pas avoir de la prématurité. Et si on veut bien voir dans 6 mois comme ça, si on a trop eu, ce sont 3 ou 2. 00:24:35-6

00:24:35-6 Personne 8: Les accouchements prématurés, vraiment, sont très rares. Ça existe, mais c'est très rare. 00:24:46-9

00:24:46-9 Personne 4: Depuis lors de la CPN, on conseille bien aux femmes de bien manger pour éviter la prématurité des enfants. Voilà, on les conseille très bien, donc ce qui fait qu'on a moins de bébés prématurés chez nous aussi. 00:25:09-0

**Interviewer (00:23:56-6):** Okay, let's move on. Can you tell me how many premature babies are born each month at your health center?

**Person 6 (00:24:10-8):** At our health center, there are some months when we don't have any cases of prematurity. Looking back over the last six months, the highest number we've seen in a single month would be around two or three premature births.

**Person 8 (00:24:35-6):** Premature births are actually quite rare here. They do occur, but it’s not a common situation.

**Person 4 (00:24:46-9):** Since we provide antenatal care (ANC), we advise women on proper nutrition to help prevent premature births. We emphasize good dietary habits, which has led to a decrease in the number of premature babies at our center.

### Major of Farakan health and social promotion center

[00:08:49-5](http://localhost:2300/file=C:/Users/USER/Desktop/ENTRETIENS%20PREMATURITE/CSPS_FARAKAN/MAJOR%20FARAKAN.MP3time=529500) Interviewer: Combien de bébés prématurés naissent chaque mois au sein de votre formation sanitaire? [00:08:54-4](http://localhost:2300/file=C:/Users/USER/Desktop/ENTRETIENS%20PREMATURITE/CSPS_FARAKAN/MAJOR%20FARAKAN.MP3time=534400)

[00:08:54-4](http://localhost:2300/file=C:/Users/USER/Desktop/ENTRETIENS%20PREMATURITE/CSPS_FARAKAN/MAJOR%20FARAKAN.MP3time=534400) Person 1: Prématurés là ici c'est rare. Dans le mois on ne va pas depasser 1 ou 2 par exemple, c'est très rare. [00:09:10-0](http://localhost:2300/file=C:/Users/USER/Desktop/ENTRETIENS%20PREMATURITE/CSPS_FARAKAN/MAJOR%20FARAKAN.MP3time=550000)

[00:09:10-0](http://localhost:2300/file=C:/Users/USER/Desktop/ENTRETIENS%20PREMATURITE/CSPS_FARAKAN/MAJOR%20FARAKAN.MP3time=550000) Interviewer: J'aurai besoin des informations sur les naissances prématurées des sept derniers mois. [00:09:18-7](http://localhost:2300/file=C:/Users/USER/Desktop/ENTRETIENS%20PREMATURITE/CSPS_FARAKAN/MAJOR%20FARAKAN.MP3time=558700)

[00:09:18-7](http://localhost:2300/file=C:/Users/USER/Desktop/ENTRETIENS%20PREMATURITE/CSPS_FARAKAN/MAJOR%20FARAKAN.MP3time=558700) Person 1: Dans le RMA même cela ne ressort pas par contre dans l'ancien RMA cela ressortait. Dans l'actuel RMA on dit "Bébés nés à terme" de moins de 2kg500.ça c'est pas une prématuré. [00:09:38-0](http://localhost:2300/file=C:/Users/USER/Desktop/ENTRETIENS%20PREMATURITE/CSPS_FARAKAN/MAJOR%20FARAKAN.MP3time=578000)

00:08:49-5 Interviewer: Combien de bébés prématurés naissent chaque mois au sein de votre formation sanitaire ? 00:08:54-4

00:08:54-4 Personne 1: Prématurés là, ici c'est rare. Dans le mois, on ne va pas dépasser 1 ou 2 par exemple, c'est très rare. 00:09:10-0

00:09:10-0 Interviewer: J'aurai besoin des informations sur les naissances prématurées des sept derniers mois. 00:09:18-7

00:09:18-7 Personne 1: Dans le RMA, même cela ne ressort pas, par contre dans l'ancien RMA, cela ressortait. Dans l'actuel RMA, on dit 'Bébés nés à terme' de moins de 2kg500. Ça, ce n’est pas une prématurité. 00:09:38-0

**Interviewer (00:08:49-5):** How many premature babies are born each month at your health facility?

**Person 1 (00:08:54-4):** Premature births are quite rare here. We usually don't have more than one or two per month.

**Interviewer (00:09:10-0):** I will need information on premature births for the last seven months.

**Person 1 (00:09:18-7):** In the current monthly activity report (RMA), premature births aren’t specifically highlighted. In the old RMA format, they were more apparent. Now, the RMA mentions 'Full term babies' weighing less than 2.5kg, but that's not the same as tracking premature births.

### Maternity manager of Farakan health and social promotion center

[00:13:02-7](http://localhost:2300/file=C:/Users/USER/Desktop/ENTRETIENS%20PREMATURITE/CSPS_FARAKAN/RESPONSABLE%20MATERNITE%20FARAKAN.MP3time=782700) Interviewer: Combien de bébés prématurés naissent chaque mois dans votre centre de santé ? [00:13:09-0](http://localhost:2300/file=C:/Users/USER/Desktop/ENTRETIENS%20PREMATURITE/CSPS_FARAKAN/RESPONSABLE%20MATERNITE%20FARAKAN.MP3time=789000)

[00:13:09-0](http://localhost:2300/file=C:/Users/USER/Desktop/ENTRETIENS%20PREMATURITE/CSPS_FARAKAN/RESPONSABLE%20MATERNITE%20FARAKAN.MP3time=789000) Person 1: Vraiment, je n'ai pas une idée comme ça. [00:13:14-6](http://localhost:2300/file=C:/Users/USER/Desktop/ENTRETIENS%20PREMATURITE/CSPS_FARAKAN/RESPONSABLE%20MATERNITE%20FARAKAN.MP3time=794600)

[00:13:14-6](http://localhost:2300/file=C:/Users/USER/Desktop/ENTRETIENS%20PREMATURITE/CSPS_FARAKAN/RESPONSABLE%20MATERNITE%20FARAKAN.MP3time=794600) Interviewer: Mais approximativement [00:13:17-4](http://localhost:2300/file=C:/Users/USER/Desktop/ENTRETIENS%20PREMATURITE/CSPS_FARAKAN/RESPONSABLE%20MATERNITE%20FARAKAN.MP3time=797400)

[00:13:17-4](http://localhost:2300/file=C:/Users/USER/Desktop/ENTRETIENS%20PREMATURITE/CSPS_FARAKAN/RESPONSABLE%20MATERNITE%20FARAKAN.MP3time=797400) Person 1: Bon, peut être 4 à 5 dans le mois. Souvent aussi nous avons des accouchements gemelaires [00:13:36-6](http://localhost:2300/file=C:/Users/USER/Desktop/ENTRETIENS%20PREMATURITE/CSPS_FARAKAN/RESPONSABLE%20MATERNITE%20FARAKAN.MP3time=816600)

[00:13:36-6](http://localhost:2300/file=C:/Users/USER/Desktop/ENTRETIENS%20PREMATURITE/CSPS_FARAKAN/RESPONSABLE%20MATERNITE%20FARAKAN.MP3time=816600) Interviewer: Est-ce qu'on peut avoir une idée sur les 7 derniers mois par rapport aux naissances prématurées ou bien après il faut qu'on vienne vous voir pour ses informations ? [00:13:45-3](http://localhost:2300/file=C:/Users/USER/Desktop/ENTRETIENS%20PREMATURITE/CSPS_FARAKAN/RESPONSABLE%20MATERNITE%20FARAKAN.MP3time=825300)

[00:13:45-3](http://localhost:2300/file=C:/Users/USER/Desktop/ENTRETIENS%20PREMATURITE/CSPS_FARAKAN/RESPONSABLE%20MATERNITE%20FARAKAN.MP3time=825300) Person 1: Oui il faut que je consulte les rapports pour vous donner. j'avais le rapport mais je l'ai remis. [00:13:54-1](http://localhost:2300/file=C:/Users/USER/Desktop/ENTRETIENS%20PREMATURITE/CSPS_FARAKAN/RESPONSABLE%20MATERNITE%20FARAKAN.MP3time=834100)

00:13:02-7 Interviewer: Combien de bébés prématurés naissent chaque mois dans votre centre de santé ? 00:13:09-0

00:13:09-0 Personne 1: Vraiment, je n'ai pas une idée comme ça. 00:13:14-6

00:13:14-6 Interviewer: Mais approximativement. 00:13:17-4

00:13:17-4 Personne 1: Bon, peut-être 4 à 5 dans le mois. Souvent aussi nous avons des accouchements gémellaires. 00:13:36-6

00:13:36-6 Interviewer: Est-ce qu'on peut avoir une idée sur les 7 derniers mois par rapport aux naissances prématurées ou bien après il faut qu'on vienne vous voir pour ces informations ? 00:13:45-3

00:13:45-3 Personne 1: Oui, il faut que je consulte les rapports pour vous donner. J'avais le rapport mais je l'ai remis. 00:13:54-1

**Interviewer (00:13:02-7):** Can you tell me how many premature babies are born each month at your health center?

**Person 1 (00:13:09-0):** I don't have an exact figure offhand.

**Interviewer (00:13:14-6):** But can you provide an approximate number?

**Person 1 (00:13:17-4):** Well, it could be around 4 to 5 premature births per month. We also frequently have twin deliveries.

**Interviewer (00:13:36-6):** Would it be possible to get data on premature births for the last seven months, or should we come back to you for this information later?

**Person 1 (00:13:45-3):** Yes, I would need to check the reports to provide accurate information. I had the report earlier, but I need to retrieve it again.

# 6. Types of challenges faced during preterm births

### Focus group of Accart-ville urban medical center

[00:13:59-3](http://localhost:2300/file=C:/Users/USER/Desktop/ENTRETIENS%20PREMATURITE/CSPS-ACCART-VILLE/FOCUS%20GROUPE%20ACCART-VILLE-BON.mp3time=839300) Interviewer: nous allons poursuivre, la naissance prématurée est-elle un défi de santé publique important auquel vous êtes confronté dans votre centre de santé ? [00:14:10-6](http://localhost:2300/file=C:/Users/USER/Desktop/ENTRETIENS%20PREMATURITE/CSPS-ACCART-VILLE/FOCUS%20GROUPE%20ACCART-VILLE-BON.mp3time=850600)

[00:14:10-6](http://localhost:2300/file=C:/Users/USER/Desktop/ENTRETIENS%20PREMATURITE/CSPS-ACCART-VILLE/FOCUS%20GROUPE%20ACCART-VILLE-BON.mp3time=850600) Person 2: nous pouvons dire que c''est une priorité, cest un problème de santé vue les consequences dela prematurité nouspouvons dire qu'est une priorité.nous sommes tous les jours confrontés aux cas de prematurités et nous evacuons à l'echelon superieur pour unemeilleure pise en charge. [00:14:39-9](http://localhost:2300/file=C:/Users/USER/Desktop/ENT)

[00:14:39-9](http://localhost:2300/file=C:/Users/USER/Desktop/ENTRETIENS%20PREMATURITE/CSPS-ACCART-VILLE/FOCUS%20GROUPE%20ACCART-VILLE-BON.mp3time=879900)Interviewer: géneralement à quels types de defis vous faites face au sein de votre centre de santé? [00:14:43-7](http://localhost:2300/file=C:/Users/USER/Desktop/ENTRETIENS%20PREMATURITE/CSPS-ACCART-VILLE/FOCUS%20GROUPE%20ACCART-VILLE-BON.mp3time=883700)

[00:14:43-7](http://localhost:2300/file=C:/Users/USER/Desktop/ENTRETIENS%20PREMATURITE/CSPS-ACCART-VILLE/FOCUS%20GROUPE%20ACCART-VILLE-BON.mp3time=883700) Person 2: notamment c'est la prise en charge qui fait defaut parce que nos n'avons pas les moyens necessaires, nous n'avons pas une unité kangourou qui permet la prise en charge, la lutte contre l"hypothermie,nous n'avons pas de couveuses egalement. ce sont ces genres de defis auxquels nous faisons face. lorsque c'est une prematurité et que tous les reflexes ne sont pas presents on evacue à l'echelon superieur [00:15:11-1](http://localhost:2300/file=C:/Users/USER/Desktop/ENTRETIENS%20PREMATURITE/CSPS-ACCART-VILLE/FOCUS%20GROUPE%20ACCART-VILLE-BON.mp3time=911100)

[00:15:11-1](http://lo)Interviewer: quand vous parlez d'unité kangourou, vous faites allusion à quoi? [00:15:14-1](http://localhost:2300/file)

[00:15:14-1](http://localhost:2300/file=C:/Users/USER/Desktop/ENTRETIENS) Person 1: elle lutte contre l'hypothermie qui est facteur de decès important néo-natal. [00:15:25-4](http://localhost:2300/file=C:/Users/USER/Desktop/ENTRETIENS)

[00:15:25-4](http://localhost:2300/file=C:/Users/USER/Desktop/ENTRETIENS%20PREMATURITE/CSPS-ACCART-VILLE/FOCUS%20GROUPE%20ACCART-VILLE-BON.mp3time=925400)Interviewer: est ce qu'il ya d'autre informations complementaires que quelqu'un d'autre veut ajouter? [00:15:33-1](http://localhost:2300/file=C:/Users/USER/Desktop/ENTRETIENS%20PREMATURITE/CSPS-ACCART-VILLE/FOCUS%20GROUPE%20ACCART-VILLE-BON.mp3time=933100)

[00:15:33-1](http://localhost:2300/file=C:/Users/USER/Desktop/ENTRETIENS%20PREMATURITE/CSPS-ACCART-VILLE/FOCUS%20GROUPE%20ACCART-VILLE-BON.mp3time=933100) Interviewer: en fait quelles sont vos préocupations par rapport aux naissances prematurés? quand il y a des prématurés quelles sont vos inquiétudes? [00:15:49-6](http://localhost:2300/file=C:/Users/USER/Desktop/ENTRETIENS%20PREMATURITE/CSPS-ACCART-VILLE/FOCUS%20GROUPE%20ACCART-VILLE-BON.mp3time=949600)

[00:15:49-6](http://localhost:2300/file=C:) Person 1: comme l4ASOG l'a dit c'est l'hypothermie et nous n'avons pas les moyens pour nous occuper des prematurés içi , donc nous les envoyons au CHUSS pour qu'on puisse les mettre sous les lampes et aussi lutter contre les infections.nous ne prenons pas en charge les prematurés içi nous les evacuons à l'echelon superieur. [00:16:23-6](http://localhost:2300/file=C:/Users/USER/Desktop/ENTRET)

00:13:59-3 Interviewer: Nous allons poursuivre, la naissance prématurée est-elle un défi de santé publique important auquel vous êtes confronté dans votre centre de santé ? 00:14:10-6

00:14:10-6 Personne 2: Nous pouvons dire que c'est une priorité, c'est un problème de santé vue les conséquences de la prématurité, nous pouvons dire que c'est une priorité. Nous sommes tous les jours confrontés aux cas de prématurités et nous évacuons à l'échelon supérieur pour une meilleure prise en charge. 00:14:39-9

00:14:39-9 Interviewer: Généralement à quels types de défis vous faites face au sein de votre centre de santé ? 00:14:43-7

00:14:43-7 Personne 2: Notamment c'est la prise en charge qui fait défaut parce que nous n'avons pas les moyens nécessaires, nous n'avons pas une unité kangourou qui permet la prise en charge, la lutte contre l'hypothermie, nous n'avons pas de couveuses également. Ce sont ces genres de défis auxquels nous faisons face. Lorsque c'est une prématurité et que tous les réflexes ne sont pas présents on évacue à l'échelon supérieur. 00:15:11-1

00:15:11-1 Interviewer: Quand vous parlez d'unité kangourou, vous faites allusion à quoi ? 00:15:14-1

00:15:14-1 Personne 1: Elle lutte contre l'hypothermie qui est facteur de décès important néo-natal. 00:15:25-4

00:15:25-4 Interviewer: Est-ce qu'il y a d'autres informations complémentaires que quelqu'un d'autre veut ajouter ? 00:15:33-1

00:15:33-1 Interviewer: En fait, quelles sont vos préoccupations par rapport aux naissances prématurées ? Quand il y a des prématurés, quelles sont vos inquiétudes ? 00:15:49-6

00:15:49-6 Personne 1: Comme l'ASSOG l'a dit, c'est l'hypothermie, et nous n'avons pas les moyens pour nous occuper des prématurés ici, donc nous les envoyons au CHUSS pour qu'on puisse les mettre sous les lampes et aussi lutter contre les infections. Nous ne prenons pas en charge les prématurés ici, nous les évacuons à l'échelon supérieur. 00:16:23-6

**Interviewer (00:13:59-3):** We'll continue, is premature birth a significant public health challenge that you face in your health center?

**Person 2 (00:14:10-6):** Premature birth is indeed a priority and a health problem due to its consequences. We deal with prematurity cases daily and often need to transfer them to higher-level facilities for better care.

**Interviewer (00:14:39-9):** What types of challenges do you generally face in your health center regarding premature births?

**Person 2 (00:14:43-7):** Our main challenge is the lack of adequate care resources. We don't have a kangaroo care unit or incubators, which are essential for managing hypothermia and other issues in premature babies. When a baby's reflexes are not fully developed, we have to evacuate them to a facility with more resources.

**Interviewer (00:15:11-1):** Could you explain what you mean by a kangaroo unit?

**Person 1 (00:15:14-1):** A kangaroo unit is crucial for combating hypothermia, a leading cause of neonatal death.

**Interviewer (00:15:25-4):** Does anyone else want to add anything about the challenges of premature births?

**Person 1 (00:15:49-6):** As previously mentioned, hypothermia is a major concern. We lack the facilities to adequately care for premature babies, so they are usually transferred to the CHUSS for specialized care under lamps and to prevent infections. We don't have the capacity to treat premature babies on-site and must rely on higher-level facilities.

### Manager of Accart-ville urban medical center

[00:11:19-9](http://localhost:2300/file=C:/Users/USER/Desktop/ENTRETIENS%20PREMATURITE/CSPS-ACCART-VILLE/RESPONSABLE%20CMU%20ACCART-VILLE.MP3time=679900)Interviewer: Est ce que la naissance prématurée estt un défi de santé publique important auquel vous êtes confronté dans votrecentre de santé? [00:11:27-3](http://localhost:2300/file=C:/Users/USER/Desktop/ENTRETIENS%20PREMATURITE/CSPS-ACCART-VILLE/RESPONSABLE%20CMU%20ACCART-VILLE.MP3time=687300)

[00:11:27-3](http://localhost:2300/file=C:/Users/USER/Desktop/ENTRETIENS%20PREMATURITE/CSPS-ACCART-VILLE/RESPONSABLE%20CMU%20ACCART-VILLE.MP3time=687300)Person 1: bon,géneralement, comme nous, on a pas de moyens disponibles en fait pour mettre en place en fait le moins disponible en fait pour pouvoir à prendre en charge. En fait, on envoit presque tout à l'hôpital Souro SANOU. Je discutais même aujourd'hui avec mon responsable. On allait essayer de mettre une fiche de liaison entre nous et puis la néonatologie, en fait, je pense que ça, ça va nous permettre même de voir, c'est à dire les statistiques réelles même de la prématurité puisque chaque nouveau né qu'on va envoyer, on aura un oeil dessus, on a un cahier de référence qui est disponible pour nous permettre de faire un compte après, savoir nos performances. l'équipe va disponibiliser le document,peut être dans la semaine. [00:12:21-8](http://localhost:2300/file=C:/Users/USER/Desktop/ENTRETIENS%20PREMATURITE/CSPS-ACCART-VILLE/RESPONSABLE%20CMU%20ACCART-VILLE.MP3time=741800)

[00:12:21-8](http://localhost:2300/file=C:/Users/USER/Desktop/ENTRETIENS%20PREMATURITE/CSPS-ACCART-VILLE/RESPONSABLE%20CMU%20ACCART-VILLE.MP3time=741800)Interviewer: maintenant est ce que, concernant la grossesse, l'accouchement et la prise en charge, vous êtes confrontés à des defis au sein de votre centre de santé? [00:12:32-7](http://localhost:2300/file=C:/Users/USER/Desktop/ENTRETIENS%20PREMATURITE/CSPS-ACCART-VILLE/RESPONSABLE%20CMU%20ACCART-VILLE.MP3time=752700)

[00:12:32-7](http://localhost:2300/file=C:/Users/USER/Desktop/ENTRETIENS%20PREMATURITE/CSPS-ACCART-VILLE/RESPONSABLE%20CMU%20ACCART-VILLE.MP3time=752700) Person 1: oui il faut dire qu'il y a beaucoup de défis à rélèver.C'est vrai, c'était un niveau CSPS on dit on est redirigé en CM, mais bon, en a fait. Cela dit, les infrastructurs n'ont pas suivi en fait. Donc on est CM maintenant et notre demande est plus, mais parfois on arrive pas en fait à satisfaire tout.Iil y a des fois on peut avoir en une nuit, on peut avoir dix accouchements mais le personnel à l'occasion ne sont que deux ou trois déjà, on ne peut pas satisfaire tout le monde, ça devient un problème. Le nombre des lits n'est pas aussi satisfaisant, puisqu'on a des dames qui se couchent à terre. Il n'y a même pas de place. Donc tout cela nous pose des problèmes. Et puis les pathologies, même liées à la grossesse parfois en fait, vu que le personnel est insuffisant, en fait, c'est difficile d'assurer les accouchements et d'assurer les malades, les grossesses pathologiques en même temps,autrefois il n y avait pas cela puisque chaque maladie liée à la grossesse était directement renvoyer au niveau superieur c'est à dire au CMA ou à l'hôpital Souro SANOU,mais actuellement vu que maintenant c'est un CM, on peut hospitaliser à la vue que nous sommes deux médecins et puis associés à un attaché en soins obstetricall, je pense qu'on peut hospitaliser.mais l'hospitalisation avec le personnel en fait parfois, ça ne suit pas, on essaie en tout cas de faire ce qu'on peut. [00:14:08-5](http://localhost:2300/file=C:/Users/USER/Desktop/ENTRETIENS%20PREMATURITE/CSPS-ACCART-VILLE/RESPONSABLE%20CMU%20ACCART-VILLE.MP3time=848500)

00:11:19-9 Interviewer: Est-ce que la naissance prématurée est un défi de santé publique important auquel vous êtes confronté dans votre centre de santé ? 00:11:27-3

00:11:27-3 Person 1: Bon, généralement, comme nous, on n'a pas de moyens disponibles en fait pour mettre en place en fait le moins disponible en fait pour pouvoir à prendre en charge. En fait, on envoie presque tout à l'hôpital Souro Sanou. Je discutais même aujourd'hui avec mon responsable. On allait essayer de mettre une fiche de liaison entre nous et puis la néonatologie, en fait, je pense que ça, ça va nous permettre même de voir, c'est-à-dire les statistiques réelles même de la prématurité puisque chaque nouveau-né qu'on va envoyer, on aura un œil dessus, on a un cahier de référence qui est disponible pour nous permettre de faire un compte après, savoir nos performances. L'équipe va disponibiliser le document, peut-être dans la semaine. 00:12:21-8

00:12:21-8 Interviewer: Maintenant est-ce que, concernant la grossesse, l'accouchement et la prise en charge, vous êtes confrontés à des défis au sein de votre centre de santé ? 00:12:32-7

00:12:32-7 Person 1: Oui, il faut dire qu'il y a beaucoup de défis à relever. C'est vrai, c'était un niveau CSPS on dit on est redirigé en CM, mais bon, en a fait. Cela dit, les infrastructures n'ont pas suivi en fait. Donc on est CM maintenant et notre demande est plus, mais parfois on n’arrive pas en fait à satisfaire tout. Il y a des fois où on peut avoir en une nuit, on peut avoir dix accouchements mais le personnel à l'occasion ne sont que deux ou trois déjà, on ne peut pas satisfaire tout le monde, ça devient un problème. Le nombre de lits n'est pas aussi satisfaisant, puisqu'on a des dames qui se couchent à terre. Il n'y a même pas de place. Donc tout cela nous pose des problèmes. Et puis les pathologies, même liées à la grossesse parfois en fait, vu que le personnel est insuffisant, en fait, c'est difficile d'assurer les accouchements et d'assurer les malades, les grossesses pathologiques en même temps, autrefois il n'y avait pas cela puisque chaque maladie liée à la grossesse était directement renvoyée au niveau supérieur c'est-à-dire au CMA ou à l'hôpital Souro Sanou, mais actuellement vu que maintenant c'est un CM, on peut hospitaliser à la vue que nous sommes deux médecins et puis associés à un attaché en soins obstétricaux, je pense qu'on peut hospitaliser. Mais l'hospitalisation avec le personnel en fait parfois, ça ne suit pas, on essaie en tout cas de faire ce qu'on peut. 00:14:08-5

**Interviewer (00:11:19-9):** Is premature birth a significant public health challenge at your health center?

**Person 1 (00:11:27-3):** Yes, we face challenges with premature births. Our facility lacks the resources to adequately care for these cases, leading us to transfer almost all cases to Souro Sanou hospital. We're planning to establish a link with neonatology to better track statistics on prematurity. We'll have a reference book to monitor and evaluate our performance, which should be ready within the week.

**Interviewer (00:12:32-7):** What other challenges do you face regarding pregnancy, childbirth, and care?

**Person 1 (00:12:32-7):** We face several challenges. Despite being upgraded to a higher-level health center, our infrastructure hasn't kept up. We often experience high patient volume, with up to ten deliveries in one night, but with only two or three staff members available, it's difficult to provide adequate care to everyone. We also have a shortage of beds, leading to patients lying on the floor. Additionally, handling pathologies linked to pregnancy is challenging due to staff limitations. Previously, we would refer any pregnancy-related illness to higher-level facilities, but now, being a higher-level center ourselves, we're expected to manage these cases with limited resources and staff.

### Maternity manager of Accart-ville urban medical center

[00:13:20-5](http://localhost:2300/file=C:/Users/USER/Desktop/ENTRETIENS%20PREMATURITE/CSPS-ACCART-VILLE/RESPONSABLE%20MATERNITE%20CMU%20ACCART-VILLE.MP3time=800500)Interviewer: nous allons toujours continuer.Est-ce que la naissance prématurée est un défi de santé publique important auquel vous êtes confronté dans votre centre de santé ? [00:13:31-5](http://localhost:2300/file=C:/Users/USER/Desktop/ENTRETIENS%20PREMATURITE/CSPS-ACCART-VILLE/RESPONSABLE%20MATERNITE%20CMU%20ACCART-VILLE.MP3time=811500)

[00:13:31-5](http://localhost:2300/file=C:/Users/USER/Desktop/ENTRETIENS%20PREMATURITE/CSPS-ACCART-VILLE/RESPONSABLE%20MATERNITE%20CMU%20ACCART-VILLE.MP3time=811500) Person 1: c'est un problème de santé dont on est confronté parce que la prise en charge pose problème et les parents refusent l'evacuation souvent. quand on evacue, l'hopital a aussi des problèmes, l'enfant est prématuré, par exemple 1800g on evacue mais ça revient pour manque de place à l'hopital, donc les parents reviennent avec une ordonnance voie orale et nous donnons des conseils. c'est vraiment un problème parce que nous avons des difficultés à ce niveau dun fait que c'est le seul centre qui a des couveuses (le centre hospitalier universitaire souro sanou) dans toute la ville de bobo et dans toute la region même si je peux le dire.ce qui fait quand on evacue les enfants nous reviennent malgré leur prematurité par manque de places. [00:14:29-7](http://localhost:2300/file=C:/Users/USER/Desktop/ENTRETIENS%20PREMATURITE/CSPS-ACCART-VILLE/RESPONSABLE%20MATERNITE%20CMU%20ACCART-VILLE.MP3time=869700)

[00:14:29-7](http://localhost:2300/file=C:/Users/USER/Desktop/ENTRETIENS%20PREMATURITE/CSPS-ACCART-VILLE/RESPONSABLE%20MATERNITE%20CMU%20ACCART-VILLE.MP3time=869700) Interviewer: vous dites que parfois les parents même refusent l'evacuation des enfants prematurés au niveau de l'échelon superieur pour la prsie en charge. Pourqui? [00:14:37-9](http://localhost:2300/file=C:/Users/USER/Desktop/ENTRETIENS%20PREMATURITE/CSPS-ACCART-VILLE/RESPONSABLE%20MATERNITE%20CMU%20ACCART-VILLE.MP3time=877900)

[00:14:37-9](http://localhost:2300/file=C:/Users/USER/Desktop/ENTRETIENS%20PREMATURITE/CSPS-ACCART-VILLE/RESPONSABLE%20MATERNITE%20CMU%20ACCART-VILLE.MP3time=877900) Person 1: effectivement les parents refusent souvent d'aller malgré les explications que nous donnons pour le bien de l'enfant prématuré. les parents refusent malgré la gratuité de l'evacuation et de la prise en charge [00:14:52-9](http://localhost:2300/file=C:/Users/USER/Desktop/ENTRETIENS%20PREMATURITE/CSPS-ACCART-VILLE/RESPONSABLE%20MATERNITE%20CMU%20ACCART-VILLE.MP3time=892900)

[00:14:52-9](http://localhost:2300/file=C:/Users/USER/Desktop/ENTRETIENS%20PREMATURITE/CSPS-ACCART-VILLE/RESPONSABLE%20MATERNITE%20CMU%20ACCART-VILLE.MP3time=892900) Interviewer: Pourquoi? [00:14:54-2](http://localhost:2300/file=C:/Users/USER/Desktop/ENTRETIENS%20PREMATURITE/CSPS-ACCART-VILLE/RESPONSABLE%20MATERNITE%20CMU%20ACCART-VILLE.MP3time=894200)

[00:14:54-2](http://localhost:2300/file=C:/Users/USER/Desktop/ENTRETIENS%20PREMATURITE/CSPS-ACCART-VILLE/RESPONSABLE%20MATERNITE%20CMU%20ACCART-VILLE.MP3time=894200) Person 1: souvent cela est dû à la perception qu'ils ont pour le nouveau née prematuré , le doute sur sa viabilité , les gens trouvent qu'ils vont manquer du temps cela va les occuper, ils pourront pas mener à bien leurs activités. ils se disent que l'enfant ne va pas survivre voilà pourquoi ils ne vont pas se fatiguer.ce sont ces raisons qui ressortent souvent. [00:15:26-6](http://localhost:2300/file=C:/Users/USER/Desktop/ENTRETIENS%20PREMATURITE/CSPS-ACCART-VILLE/RESPONSABLE%20MATERNITE%20CMU%20ACCART-VILLE.MP3time=926600)

[00:15:26-6](http://localhost:2300/file=C:/Users/USER/Desktop/ENTRETIENS%20PREMATURITE/CSPS-ACCART-VILLE/RESPONSABLE%20MATERNITE%20CMU%20ACCART-VILLE.MP3time=926600) Interviewer: maintenant au sein de votre centre de santé, est ce que vous etes confrontés à des defis lors dela grossesse, de l'accouchement des nouveaux-nées prematurés? [00:15:38-6](http://localhost:2300/file=C:/Users/USER/Desktop/ENTRETIENS%20PREMATURITE/CSPS-ACCART-VILLE/RESPONSABLE%20MATERNITE%20CMU%20ACCART-VILLE.MP3time=938600)

[00:15:38-6](http://localhost:2300/file=C:/Users/USER/Desktop/ENTRETIENS%20PREMATURITE/CSPS-ACCART-VILLE/RESPONSABLE%20MATERNITE%20CMU%20ACCART-VILLE.MP3time=938600) Person 1: nous sommes confrontés souvent à des problèmes d'infections qui peuvent etre la cause de la prematurité.quand on fait les prescriptions, au niveau de la gratuité il n'y a pas souvent les produits indiqués pour prendre en charge la menace des accouchements prematurés . on n'a pas ces produits et lorsque tu prescrits pour que la femme aille chercher le produit en officine privé elle revients dire qu'elle n'a pas les moyens. donc on est vraiment confronté à ce problème dans la prise en charge des menaces.une autre difficulté, une fois l'enfant née, nous ne disposons pas de materiels necessaire à la prise en charge du nouveau-née prématuré. [00:16:26-6](http://localhost:2300/file=C:/Users/USER/Desktop/ENTRETIENS%20PREMATURITE/CSPS-ACCART-VILLE/RESPONSABLE%20MATERNITE%20CMU%20ACCART-VILLE.MP3time=986600)

[00:16:26-6](http://localhost:2300/file=C:/Users/USER/Desktop/ENTRETIENS%20PREMATURITE/CSPS-ACCART-VILLE/RESPONSABLE%20MATERNITE%20CMU%20ACCART-VILLE.MP3time=986600) Interviewer: lorsque vous parlez de materiels necessaires pour la prise en charge du nouveau-née, vous faites allusion à quels types de materiels? [00:16:31-1](http://localhost:2300/file=C:/Users/USER/Desktop/ENTRETIENS%20PREMATURITE/CSPS-ACCART-VILLE/RESPONSABLE%20MATERNITE%20CMU%20ACCART-VILLE.MP3time=991100)

[00:16:31-1](http://localhost:2300/file=C:/Users/USER/Desktop/ENTRETIENS%20PREMATURITE/CSPS-ACCART-VILLE/RESPONSABLE%20MATERNITE%20CMU%20ACCART-VILLE.MP3time=991100) Person 1: je fais allusion vraiment aux lampes chauffantes pour lutter contre l'hypothermie, je fais allusion aussi à la prevention de l'infection parce qu'on a pas de chambres individuelles, on a pas de chambres dediées aux prematurés, au nouveau-née même. nous avons une seule salle ou on hospitalise et les malades, les femmes en travaille et les femmes en post-partum egalement. c'est la même salle avec 10 lits qui sont tout le temps occupés et il arrive que certaines femmes etallent des pagnes à même le sol pour se coucher dessus avec un nouveau-née prematuré ce qui n'est pas interessant. nous avons une grande salle d'une capacité de 10 lits [00:17:22-9](http://localhost:2300/file=C:/Users/USER/Desktop/ENTRETIENS%20PREMATURITE/CSPS-ACCART-VILLE/RESPONSABLE%20MATERNITE%20CMU%20ACCART-VILLE.MP3time=1042900)

00:13:20-5 Interviewer: Nous allons toujours continuer. Est-ce que la naissance prématurée est un défi de santé publique important auquel vous êtes confronté dans votre centre de santé ? 00:13:31-5

00:13:31-5 Person 1: C'est un problème de santé dont on est confronté parce que la prise en charge pose problème et les parents refusent l'évacuation souvent. Quand on évacue, l'hôpital a aussi des problèmes, l'enfant est prématuré, par exemple 1800g on évacue mais ça revient pour manque de place à l'hôpital, donc les parents reviennent avec une ordonnance voie orale et nous donnons des conseils. C'est vraiment un problème parce que nous avons des difficultés à ce niveau du fait que c'est le seul centre qui a des couveuses (le centre hospitalier universitaire Souro Sanou) dans toute la ville de Bobo et dans toute la région même si je peux le dire. Ce qui fait quand on évacue les enfants nous reviennent malgré leur prématurité par manque de places. 00:14:29-7

00:14:29-7 Interviewer: Vous dites que parfois les parents même refusent l'évacuation des enfants prématurés au niveau de l'échelon supérieur pour la prise en charge. Pourquoi ? 00:14:37-9

00:14:37-9 Person 1: Effectivement, les parents refusent souvent d'aller malgré les explications que nous donnons pour le bien de l'enfant prématuré. Les parents refusent malgré la gratuité de l'évacuation et de la prise en charge 00:14:52-9

00:14:52-9 Interviewer: Pourquoi ? 00:14:54-2

00:14:54-2 Person 1: Souvent cela est dû à la perception qu'ils ont pour le nouveau-né prématuré, le doute sur sa viabilité, les gens trouvent qu'ils vont manquer du temps cela va les occuper, ils pourront pas mener à bien leurs activités. Ils se disent que l'enfant ne va pas survivre voilà pourquoi ils ne vont pas se fatiguer. Ce sont ces raisons qui ressortent souvent. 00:15:26-6

00:15:26-6 Interviewer: Maintenant au sein de votre centre de santé, est-ce que vous êtes confrontés à des défis lors de la grossesse, de l'accouchement des nouveau-nés prématurés ? 00:15:38-6

00:15:38-6 Person 1: Nous sommes confrontés souvent à des problèmes d'infections qui peuvent être la cause de la prématurité. Quand on fait les prescriptions, au niveau de la gratuité il n'y a pas souvent les produits indiqués pour prendre en charge la menace des accouchements prématurés. On n'a pas ces produits et lorsque tu prescris pour que la femme aille chercher le produit en officine privée elle revient dire qu'elle n'a pas les moyens. Donc on est vraiment confronté à ce problème dans la prise en charge des menaces. Une autre difficulté, une fois l'enfant né, nous ne disposons pas de matériels nécessaires à la prise en charge du nouveau-né prématuré. 00:16:26-6

00:16:26-6 Interviewer: Lorsque vous parlez de matériels nécessaires pour la prise en charge du nouveau-né, vous faites allusion à quels types de matériels ? 00:16:31-1

00:16:31-1 Person 1: Je fais allusion vraiment aux lampes chauffantes pour lutter contre l'hypothermie, je fais allusion aussi à la prévention de l'infection parce qu'on n'a pas de chambres individuelles, on n'a pas de chambres dédiées aux prématurés, au nouveau-né même. Nous avons une seule salle où on hospitalise les malades, les femmes en travail et les femmes en post-partum également. C'est la même salle avec 10 lits qui sont tout le temps occupés et il arrive que certaines femmes étalent des pagnes à même le sol pour se coucher dessus avec un nouveau-né prématuré, ce qui n'est pas intéressant. Nous avons une grande salle d'une capacité de 10 lits. 00:17:22-9

**Interviewer (00:13:20-5):** Is premature birth a significant public health challenge at your health center?

**Person 1 (00:13:31-5):** Yes, premature birth poses a major health challenge. We often face difficulties in providing care, and parents frequently refuse to transfer their premature infants to higher-level facilities for further treatment. Even when we do evacuate, hospitals like Souro Sanou, which are equipped with incubators, often lack space, leading to infants being sent back to us with only oral prescriptions. This situation is problematic due to the limited resources and space for premature infants in the entire region.

**Interviewer (00:14:37-9):** Why do parents often refuse to transfer their premature infants for further care?

**Person 1 (00:14:54-2):** Parents' refusal is often rooted in their perceptions about the viability of premature newborns. Many believe that the child may not survive, so they feel it's not worth the time and effort. They worry about the time commitment and the impact on their daily activities.

**Interviewer (00:15:38-6):** What challenges do you face during pregnancy and the delivery of premature newborns in your health center?

**Person 1 (00:15:38-6):** Infections are a common problem that can lead to prematurity. We often lack the necessary medications to manage the threat of premature births effectively. When prescriptions are given for medication from private pharmacies, many families cannot afford them. Additionally, once a premature child is born, we lack the essential equipment and resources to provide adequate care.

**Interviewer (00:16:26-6):** "Can you specify what types of materials are needed for newborn care in your facility?"

**Person 1 (00:16:31-1):** "Primarily, we need heat lamps to prevent hypothermia in newborns, especially premature ones. Infection prevention is another concern, as we don't have individual rooms or dedicated spaces for premature babies or even newborns. Our facility only has one large room that serves multiple purposes. It accommodates sick individuals, women in labor, and postpartum women, all sharing 10 beds. Frequently, these beds are fully occupied, leading some women to spread loincloths on the floor for themselves and their premature newborns, which is far from ideal. This shared room is designed for 10 beds."

### Focus group of Do medical center with surgical branch

[00:14:24-7](http://localhost:2300/file=C:/Users/USER/Desktop/ENTRETIENS%20PREMATURITE/CMA-DO/FOCUS%20GROUPE%20CMA-DO.mp3time=864700) Interviewer: Maintenant, la question suivante, est-ce que la naissance prématurée est un défi de santé publique auquel vous êtes confrontés ici? [00:14:34-3](http://localhost:2300/file=C:/Users/USER/Desktop/ENTRETIENS%20PREMATURITE/CMA-DO/FOCUS%20GROUPE%20CMA-DO.mp3time=874300)

[00:14:34-3](http://localhost:2300/file=C:/Users/USER/Desktop/ENTRETIENS%20PREMATURITE/CMA-DO/FOCUS%20GROUPE%20CMA-DO.mp3time=874300) Person 6: Ah oui, parce que la naissance prématurée, c'est une urgence obstétricale, c'est clair. Donc, ça fait partie d'un défi dans nos contextes. C'est parce que nous, on n'est pas dans un coin là où c'est. Sinon, la naissance prématurée a besoin d'un pédiatre à côté. L'obstétricien est là, le pédiatre est là, peut être plus ou moins même un réanimateur. Mais dans nos contextes, c'est vrai, même s'il n'y a pas ça chez nous, c'est une exigence obstétricale. [00:14:59-4](http://localhost:2300/file=C:/Users/USER/Desktop/ENTRETIENS%20PREMATURITE/CMA-DO/FOCUS%20GROUPE%20CMA-DO.mp3time=899400)

[00:14:59-4](http://localhost:2300/file=C:/Users/USER/Desktop/ENTRETIENS%20PREMATURITE/CMA-DO/FOCUS%20GROUPE%20CMA-DO.mp3time=899400) Interviewer: Est-ce qu'il y a quelque chose d'autre à ajouter? [00:15:02-9](http://localhost:2300/file=C:/Users/USER/Desktop/ENTRETIENS%20PREMATURITE/CMA-DO/FOCUS%20GROUPE%20CMA-DO.mp3time=902900)

[00:15:02-9](http://localhost:2300/file=C:/Users/USER/Desktop/ENTRETIENS%20PREMATURITE/CMA-DO/FOCUS%20GROUPE%20CMA-DO.mp3time=902900) Person 7: On a besoin d'équipement pour prendre en charge les naissances prématurées. [00:15:10-7](http://localhost:2300/file=C:/Users/USER/Desktop/ENTRETIENS%20PREMATURITE/CMA-DO/FOCUS%20GROUPE%20CMA-DO.mp3time=910700)

[00:15:10-7](http://localhost:2300/file=C:/Users/USER/Desktop/ENTRETIENS%20PREMATURITE/CMA-DO/FOCUS%20GROUPE%20CMA-DO.mp3time=910700) Interviewer: En quoi est ce que c'est un défi? [00:15:14-1](http://localhost:2300/file=C:/Users/USER/Desktop/ENTRETIENS%20PREMATURITE/CMA-DO/FOCUS%20GROUPE%20CMA-DO.mp3time=914100)

[00:15:14-1](http://localhost:2300/file=C:/Users/USER/Desktop/ENTRETIENS%20PREMATURITE/CMA-DO/FOCUS%20GROUPE%20CMA-DO.mp3time=914100) Person 6: A cause de la mortalité néonatale [00:15:28-1](http://localhost:2300/file=C:/Users/USER/Desktop/ENTRETIENS%20PREMATURITE/CMA-DO/FOCUS%20GROUPE%20CMA-DO.mp3time=928100)

[00:15:28-1](http://localhost:2300/file=C:/Users/USER/Desktop/ENTRETIENS%20PREMATURITE/CMA-DO/FOCUS%20GROUPE%20CMA-DO.mp3time=928100) Interviewer: Est ce que vous avez souvent des cas de grand prématurés ici? [00:15:31-8](http://localhost:2300/file=C:/Users/USER/Desktop/ENTRETIENS%20PREMATURITE/CMA-DO/FOCUS%20GROUPE%20CMA-DO.mp3time=931800)

[00:15:31-8](http://localhost:2300/file=C:/Users/USER/Desktop/ENTRETIENS%20PREMATURITE/CMA-DO/FOCUS%20GROUPE%20CMA-DO.mp3time=931800) Person 6: Oui il y a des grands prématurés. [00:15:35-6](http://localhost:2300/file=C:/Users/USER/Desktop/ENTRETIENS%20PREMATURITE/CMA-DO/FOCUS%20GROUPE%20CMA-DO.mp3time=935600)

[00:15:35-6](http://localhost:2300/file=C:/Users/USER/Desktop/ENTRETIENS%20PREMATURITE/CMA-DO/FOCUS%20GROUPE%20CMA-DO.mp3time=935600) Person 3: Si il y a des grands prématurés et là c'est compliqué, dans notre contexte même est ce que c'est viable? [00:16:12-0](http://localhost:2300/file=C:/Users/USER/Desktop/ENTRETIENS%20PREMATURITE/CMA-DO/FOCUS%20GROUPE%20CMA-DO.mp3time=972000)

[00:16:12-0](http://localhost:2300/file=C:/Users/USER/Desktop/ENTRETIENS%20PREMATURITE/CMA-DO/FOCUS%20GROUPE%20CMA-DO.mp3time=972000) Person 6: Bon se sont des bébés qu'on envoie au SUS dans la majorité des cas. Parce que, ici c'est soins mères kangourou et ils ne recoivent que de faibles poids de naissances.Mais les grands prématurés sont évacués au CHURSS. [00:16:32-7](http://localhost:2300/file=C:/Users/USER/Desktop/ENTRETIENS%20PREMATURITE/CMA-DO/FOCUS%20GROUPE%20CMA-DO.mp3time=992700)

[00:16:32-7](http://localhost:2300/file=C:/Users/USER/Desktop/ENTRETIENS%20PREMATURITE/CMA-DO/FOCUS%20GROUPE%20CMA-DO.mp3time=992700) Interviewer: Est ce que vous êtes souvent confrontés à des défi lors de l'accouchement des enfants prématurés? [00:16:39-7](http://localhost:2300/file=C:/Users/USER/Desktop/ENTRETIENS%20PREMATURITE/CMA-DO/FOCUS%20GROUPE%20CMA-DO.mp3time=999700)

[00:16:39-7](http://localhost:2300/file=C:/Users/USER/Desktop/ENTRETIENS%20PREMATURITE/CMA-DO/FOCUS%20GROUPE%20CMA-DO.mp3time=999700) Person 6: Ah oui c'est à beaucoup de défi. Parce que vous êtes en face d'une grande plus maturité, on évite les manœuvres. En ce cas, pour faciliter le passage du fœtus, des fois, tu peut être amené à faire une épisiotomie . Faciliter le passage. Surtout si c'est un siège, on ne doit pas faire de manœuvres. C'est ça qui amène des fois à faire la césarienne sur les prématurés [00:17:06-7](http://localhost:2300/file=C:/Users/USER/Desktop/ENTRETIENS%20PREMATURITE/CMA-DO/FOCUS%20GROUPE%20CMA-DO.mp3time=1026700)

[00:17:06-7](http://localhost:2300/file=C:/Users/USER/Desktop/ENTRETIENS%20PREMATURITE/CMA-DO/FOCUS%20GROUPE%20CMA-DO.mp3time=1026700) Person 5: Il y a aussi la réanimation. Parce que facilement, il y a une detresse qui est là. Donc il faut prévoir aussi la réanimation. Y compris tout ce qui rentre dans le cas de la réanimation. Le chauffage. Les obstructions, l'oxygénation. C'est vraiment une prise en charge qu'il faut rapidement operer pour sauver la femme. [00:17:43-9](http://localhost:2300/file=C:/Users/USER/Desktop/ENTRETIENS%20PREMATURITE/CMA-DO/FOCUS%20GROUPE%20CMA-DO.mp3time=1063900)

[00:17:43-9](http://localhost:2300/file=C:/Users/USER/Desktop/ENTRETIENS%20PREMATURITE/CMA-DO/FOCUS%20GROUPE%20CMA-DO.mp3time=1063900) Interviewer: Et lors de la grossesse,est-ce qu'il y a des défis egalement à ce niveau ? [00:17:51-1](http://localhost:2300/file=C:/Users/USER/Desktop/ENTRETIENS%20PREMATURITE/CMA-DO/FOCUS%20GROUPE%20CMA-DO.mp3time=1071100)

[00:17:51-1](http://localhost:2300/file=C:/Users/USER/Desktop/ENTRETIENS%20PREMATURITE/CMA-DO/FOCUS%20GROUPE%20CMA-DO.mp3time=1071100) Person 9: Puisqu'on ne peut pas savoir d'emblé s'elle va faire un accouchement prématuré ou pas . S'il n y a pas de menaces ou s'elle n'a pas d'antécedant. [00:18:09-7](http://localhost:2300/file=C:/Users/USER/Desktop/ENTRETIENS%20PREMATURITE/CMA-DO/FOCUS%20GROUPE%20CMA-DO.mp3time=1089700)

00:14:24-7 Interviewer: Maintenant, la question suivante, est-ce que la naissance prématurée est un défi de santé publique auquel vous êtes confrontés ici ? 00:14:34-3

00:14:34-3 Person 6: Ah oui, parce que la naissance prématurée, c'est une urgence obstétricale, c'est clair. Donc, ça fait partie d'un défi dans nos contextes. C'est parce que nous, on n'est pas dans un coin là où c'est. Sinon, la naissance prématurée a besoin d'un pédiatre à côté. L'obstétricien est là, le pédiatre est là, peut être plus ou moins même un réanimateur. Mais dans nos contextes, c'est vrai, même s'il n'y a pas ça chez nous, c'est une exigence obstétricale. 00:14:59-4

00:14:59-4 Interviewer: Est-ce qu'il y a quelque chose d'autre à ajouter ? 00:15:02-9

00:15:02-9 Person 7: On a besoin d'équipement pour prendre en charge les naissances prématurées. 00:15:10-7

00:15:10-7 Interviewer: En quoi est-ce que c'est un défi ? 00:15:14-1

00:15:14-1 Person 6: À cause de la mortalité néonatale. 00:15:28-1

00:15:28-1 Interviewer: Est-ce que vous avez souvent des cas de grand prématurés ici ? 00:15:31-8

00:15:31-8 Person 6: Oui, il y a des grands prématurés. 00:15:35-6

00:15:35-6 Person 3: S'il y a des grands prématurés et là c'est compliqué, dans notre contexte même est-ce que c'est viable ? 00:16:12-0

00:16:12-0 Person 6: Bon, ce sont des bébés qu'on envoie au SUS dans la majorité des cas. Parce que, ici c'est soins mères kangourou et ils ne reçoivent que de faibles poids de naissances. Mais les grands prématurés sont évacués au CHURSS. 00:16:32-7

00:16:32-7 Interviewer: Est-ce que vous êtes souvent confrontés à des défis lors de l'accouchement des enfants prématurés ? 00:16:39-7

00:16:39-7 Person 6: Ah oui, c'est un grand défi. Parce que vous êtes en face d'une grande prématurité, on évite les manœuvres. En ce cas, pour faciliter le passage du fœtus, des fois, tu peux être amené à faire une épisiotomie. Faciliter le passage. Surtout si c'est un siège, on ne doit pas faire de manœuvres. C'est ça qui amène des fois à faire la césarienne sur les prématurés. 00:17:06-7

00:17:06-7 Person 5: Il y a aussi la réanimation. Parce que facilement, il y a une détresse qui est là. Donc il faut prévoir aussi la réanimation. Y compris tout ce qui rentre dans le cas de la réanimation. Le chauffage. Les obstructions, l'oxygénation. C'est vraiment une prise en charge qu'il faut rapidement opérer pour sauver la femme. 00:17:43-9

00:17:43-9 Interviewer: Et lors de la grossesse, est-ce qu'il y a des défis également à ce niveau ? 00:17:51-1

00:17:51-1 Person 9: Puisqu'on ne peut pas savoir d'emblée si elle va faire un accouchement prématuré ou pas. S'il n'y a pas de menaces ou si elle n'a pas d'antécédents. 00:18:09-7

**Interviewer (00:14:24-7):** Is premature birth a significant public health challenge here?

**Person 6 (00:14:34-3):** Yes, premature birth is definitely an obstetric emergency and a major challenge, especially in our context where we lack specialists like pediatricians and intensivists.

**Person 7 (00:15:02-9):** The main challenge is the lack of equipment needed to care for premature births.

**Person 6 (00:15:14-1):** This leads to a high rate of neonatal mortality.

**Interviewer (00:15:28-1):** Are cases of very premature births common here?

**Person 6 (00:15:31-8):** Yes, and these cases are usually sent to the SUS or CHURSS, as our facility primarily provides kangaroo mother care and manages only low birth weights.

**Interviewer (00:16:39-7):** What challenges do you face during the delivery of premature children?

**Person 6 (00:16:39-7):** Delivering extremely premature babies is a big challenge. We have to be careful with maneuvers, sometimes requiring episiotomies or cesarean sections, especially in breech presentations.

**Person 5 (00:17:43-9):** Resuscitation is also a critical aspect, including heating, clearing obstructions, and ensuring oxygenation.

**Interviewer (00:17:51-1):** Are there challenges during pregnancy as well?

**Person 9 (00:17:51-1):** Yes, especially since it's hard to predict premature births unless there are obvious threats or a patient's history indicating a risk.

### Gynecologist 1

[00:09:12-7](http://localhost:2300/file=C:/Users/USER/Desktop/ENTRETIENS%20PREMATURITE/CMA-DO/GENYCOLOGUE%2001.mp3time=552700) Person 1: Oui. Est-ce que pour vous, la naissance prématurée est un défi de santé publique ? [00:09:20-7](http://localhost:2300/file=C:/Users/USER/Desktop/ENTRETIENS%20PREMATURITE/CMA-DO/GENYCOLOGUE%2001.mp3time=560700)

[00:09:20-7](http://localhost:2300/file=C:/Users/USER/Desktop/ENTRETIENS%20PREMATURITE/CMA-DO/GENYCOLOGUE%2001.mp3time=560700) Person 1: Oui, parce que les prématurés nous posent beaucoup de problèmes. Surtout la prise en charge, l'élévage des prématurés, ça nous pose beaucoup de problèmes. [00:09:32-6](http://localhost:2300/file=C:/Users/USER/Desktop/ENTRETIENS%20PREMATURITE/CMA-DO/GENYCOLOGUE%2001.mp3time=572600)

[00:09:32-6](http://localhost:2300/file=C:/Users/USER/Desktop/ENTRETIENS%20PREMATURITE/CMA-DO/GENYCOLOGUE%2001.mp3time=572600) Interviewer: Quel genre de problèmes vous avez avec les prématurés ? [00:09:34-9](http://localhost:2300/file=C:/Users/USER/Desktop/ENTRETIENS%20PREMATURITE/CMA-DO/GENYCOLOGUE%2001.mp3time=574900)

[00:09:34-9](http://localhost:2300/file=C:/Users/USER/Desktop/ENTRETIENS%20PREMATURITE/CMA-DO/GENYCOLOGUE%2001.mp3time=574900) Person 1: D'abord, ce sont des enfants qui sont fragiles, qui se retrouvent facilement avec des détresses respiratoires, avec des problèmes de régulation de la température, avec des problèmes d'infection. Ce sont vraiment des enfants qu'il faut surveiller, qu'il faut prendre avec munici. [00:09:56-3](http://localhost:2300/file=C:/Users/USER/Desktop/ENTRETIENS%20PREMATURITE/CMA-DO/GENYCOLOGUE%2001.mp3time=596300)

[00:09:56-3](http://localhost:2300/file=C:/Users/USER/Desktop/ENTRETIENS%20PREMATURITE/CMA-DO/GENYCOLOGUE%2001.mp3time=596300) Interviewer: Quel est le défi au niveau de la grossesse ? [00:09:59-1](http://localhost:2300/file=C:/Users/USER/Desktop/ENTRETIENS%20PREMATURITE/CMA-DO/GENYCOLOGUE%2001.mp3time=599100)

[00:09:59-1](http://localhost:2300/file=C:/Users/USER/Desktop/ENTRETIENS%20PREMATURITE/CMA-DO/GENYCOLOGUE%2001.mp3time=599100) Person 1: Le défi, au niveau de la grossesse, peut-être pas. Parce que la prématurité, comme souvent, il y a des causes, ça, ça peut être également un défi. Parce que la plupart des prématurés qu'on a ici, c'est souvent après la rupture de la poche des os, après la préeclampsie sévère, qu'on a les cas de prématurés. On est obligé de sortir les enfants avant terme. Voilà, je pense que ça aussi, ça pose un défi pendant la grossesse. [00:10:28-2](http://localhost:2300/file=C:/Users/USER/Desktop/ENTRETIENS%20PREMATURITE/CMA-DO/GENYCOLOGUE%2001.mp3time=628200)

00:09:12-7 Person 1: Oui. Est-ce que pour vous, la naissance prématurée est un défi de santé publique ? 00:09:20-7

00:09:20-7 Person 1: Oui, parce que les prématurés nous posent beaucoup de problèmes. Surtout la prise en charge, l'élevage des prématurés, ça nous pose beaucoup de problèmes. 00:09:32-6

00:09:32-6 Interviewer: Quel genre de problèmes avez-vous avec les prématurés ? 00:09:34-9

00:09:34-9 Person 1: D'abord, ce sont des enfants qui sont fragiles, qui se retrouvent facilement avec des détresses respiratoires, avec des problèmes de régulation de la température, avec des problèmes d'infection. Ce sont vraiment des enfants qu'il faut surveiller, qu'il faut prendre avec minutie. 00:09:56-3

00:09:56-3 Interviewer: Quel est le défi au niveau de la grossesse ? 00:09:59-1

00:09:59-1 Person 1: Le défi, au niveau de la grossesse, peut-être pas. Parce que la prématurité, comme souvent, il y a des causes, ça, ça peut être également un défi. Parce que la plupart des prématurés qu'on a ici, c'est souvent après la rupture de la poche des eaux, après la prééclampsie sévère, qu'on a les cas de prématurés. On est obligé de sortir les enfants avant terme. Voilà, je pense que ça aussi, ça pose un défi pendant la grossesse. 00:10:28-2

**Person 1 (00:09:12-7):** Do you consider premature birth a significant public health challenge?

**Person 1 (00:09:20-7):** Yes, absolutely. Premature babies present numerous challenges for us, especially in terms of their care and rearing. These aspects pose significant difficulties.

**Interviewer (00:09:32-6):** What kind of problems do you have with premature babies?

**Person 1 (00:09:34-9):** Premature babies are very fragile and often experience respiratory distress, difficulties in regulating temperature, and are prone to infections. They require close monitoring and careful treatment.

**Interviewer (00:09:56-3):** What is the challenge with pregnancy?

**Person 1 (00:09:59-1):** In terms of pregnancy, the challenge is often related to the causes leading to prematurity. Many premature births here occur due to reasons like the breaking of water or severe preeclampsia, necessitating early delivery of the babies. This situation itself poses a significant challenge during pregnancy.

### Gynecologist 2

[00:13:22-7](http://localhost:2300/file=C:/Users/USER/Desktop/ENTRETIENS%20PREMATURITE/CMA-DO/GENYCOLOGUE%2002.mp3time=802700) Interviewer: Maintenant, est-ce que selon vous, la naissance prématurée est un défi de santé publique auquel vous êtes confronté ? [00:13:32-3](http://localhost:2300/file=C:/Users/USER/Desktop/ENTRETIENS%20PREMATURITE/CMA-DO/GENYCOLOGUE%2002.mp3time=812300)

[00:13:32-3](http://localhost:2300/file=C:/Users/USER/Desktop/ENTRETIENS%20PREMATURITE/CMA-DO/GENYCOLOGUE%2002.mp3time=812300) Person 1: C'est un défi pour nous, mais je pense que ça l'est pour les autres centres aussi. Parce que la majorité de nos hospitalisations sont des femmes enceintes hospitalisées. Ce sont les femmes enceintes qui ne sont pas à terme. Soit pour une pathologie donnée, c'est une femme qui n'est pas à terme, mais elle se retrouve être hospitalisée. Le défi maintenant, pour nous, est de pouvoir conduire cette grossesse à terme ou bien de conduire cette grossesse jusqu'à ce que l'enfant ait un terme de maturité suffisant pour un accouchement ou une césarienne. C'est vraiment un problème que nous vivons tous les jours. On a des femmes hospitalisées en grossesse dont la grossesse n'est pas à terme. Et souvent, les pathologies font telle qu'on est obligé de faire un accouchement prématuré. [00:14:18-7](http://localhost:2300/file=C:/Users/USER/Desktop/ENTRETIENS%20PREMATURITE/CMA-DO/GENYCOLOGUE%2002.mp3time=858700)

[00:14:18-7](http://localhost:2300/file=C:/Users/USER/Desktop/ENTRETIENS%20PREMATURITE/CMA-DO/GENYCOLOGUE%2002.mp3time=858700) Interviewer: Est-ce que la naissance prématurée est un défi pour vous? [00:14:21-0](http://localhost:2300/file=C:/Users/USER/Desktop/ENTRETIENS%20PREMATURITE/CMA-DO/GENYCOLOGUE%2002.mp3time=861000)

[00:14:21-0](http://localhost:2300/file=C:/Users/USER/Desktop/ENTRETIENS%20PREMATURITE/CMA-DO/GENYCOLOGUE%2002.mp3time=861000) Person 1: Si si. Si l'enfant naît avant le terme, alors ça expose un certain nombre de problèmes. Et donc, dans le service, on prend des mesures, on essaie de prendre des mesures, on n'a pas tout ce qu'il faut. On essaie de prendre des mesures pour permettre de mettre l'enfant dans des situations acceptables pour sa survie. [00:14:40-3](http://localhost:2300/file=C:/Users/USER/Desktop/ENTRETIENS%20PREMATURITE/CMA-DO/GENYCOLOGUE%2002.mp3time=880300)

[00:14:40-3](http://localhost:2300/file=C:/Users/USER/Desktop/ENTRETIENS%20PREMATURITE/CMA-DO/GENYCOLOGUE%2002.mp3time=880300) Interviewer: Quand vous dites que vous prenez des mesures, vous faites à des gens quel type de mesures? [00:14:43-8](http://localhost:2300/file=C:/Users/USER/Desktop/ENTRETIENS%20PREMATURITE/CMA-DO/GENYCOLOGUE%2002.mp3time=883800)

[00:14:43-8](http://localhost:2300/file=C:/Users/USER/Desktop/ENTRETIENS%20PREMATURITE/CMA-DO/GENYCOLOGUE%2002.mp3time=883800) Person 1: Dès la naissance, puisqu'on a une unité kangourou,ce n'est pas une unité de pediatrie, mais c'est une unité où les agents ont été formés pour prendre en charge les enfants prématurés qui ne sont pas malades. Donc si l'enfant est prématuré, dès la naissance, l'enfant est confié à cette unité. Mais à l'intérieur de l'équipe des sages femmes qui font les accouchements, beaucoup ont déjà été initiés au soin lié à la prématurité. Donc déjà, c'est de mettre l'enfant dans une atmosphère chaude, c'est à dire qu'on l'enveloppe assez convenablement pour ne pas le mettre en contact avec le courant d'air extérieur. Si par exemple, il y a les ventilos, on les arrête. Mais ça, c'est valable pour tous les accouchements. Et si l'enfant a des bons réflexes, c'est-à-dire s'il s'adapte correctement, on le confie à l'unité kangourou. Eux (l'unité kangourou) ils vont maintenant éduquer la maman et les autres membres de la famille sur comment alimenter cet enfant, comment garder cet enfant au chaud jusqu'à ce qu'il ait un poids acceptable pour pouvoir se comporter comme les autres bébés. [00:15:57-9](http://localhost:2300/file=C:/Users/USER/Desktop/ENTRETIENS%20PREMATURITE/CMA-DO/GENYCOLOGUE%2002.mp3time=957900)

[00:15:57-9](http://localhost:2300/file=C:/Users/USER/Desktop/ENTRETIENS%20PREMATURITE/CMA-DO/GENYCOLOGUE%2002.mp3time=957900) Interviewer: Un poids acceptable? [00:15:58-9](http://localhost:2300/file=C:/Users/USER/Desktop/ENTRETIENS%20PREMATURITE/CMA-DO/GENYCOLOGUE%2002.mp3time=958900)

[00:15:58-9](http://localhost:2300/file=C:/Users/USER/Desktop/ENTRETIENS%20PREMATURITE/CMA-DO/GENYCOLOGUE%2002.mp3time=958900) Person 1: Oui, c'est-à-dire chez nous, le poids minimum acceptable, c'est 2,5 kg. Donc si l'enfant est par exemple à 2 kg, généralement il est prématuré, mais ce n'est pas toujours qu'il est à 2 kg qu'il est prématuré, parce qu'il y a des hypotrophes. Il y a des enfants qui sont nés à terme, mais qui ont un poids de moins de 2,5 kg. Donc si il est prématuré, la plupart du temps le poids est inférieur à 2,5 kg. Donc on le met au chaud, ça c'est s'il a des bons réflexes, s'il peut sucer le sein, s'il peut tenir le sein, ou alors s'il peut boire le lait qu'on va lui donner à la tasse. Donc on le met au chaud et on le confie à l'unité kangourou qui va continuer la suite. Là on dit qu'il est acceptable. Maintenant il se trouve que souvent il y a des enfants prématurés dont le poids est inférieur à 2,5 kg, mais qui ne présentent pas de bons réflexes, ils ne peuvent pas tenir le sein, ils ne peuvent pas sucer correctement, on est obligé de les envoyer à la pédiatrie, à Souro SANOU. [00:17:07-3](http://localhost:2300/file=C:/Users/USER/Desktop/ENTRETIENS%20PREMATURITE/CMA-DO/GENYCOLOGUE%2002.mp3time=1027300)

[00:17:07-3](http://localhost:2300/file=C:/Users/USER/Desktop/ENTRETIENS%20PREMATURITE/CMA-DO/GENYCOLOGUE%2002.mp3time=1027300) Interviewer: Est-ce que vous avez souvent des naissances de grands prématurés ici? [00:17:12-0](http://localhost:2300/file=C:/Users/USER/Desktop/ENTRETIENS%20PREMATURITE/CMA-DO/GENYCOLOGUE%2002.mp3time=1032000)

[00:17:12-0](http://localhost:2300/file=C:/Users/USER/Desktop/ENTRETIENS%20PREMATURITE/CMA-DO/GENYCOLOGUE%2002.mp3time=1032000) Person 1: Parce que l'intervalle que j'avais donné c'est de 28 semaines à 37 semaines. Donc 36 semaines et 6 jours l'enfant est toujours prématuré. A partir de 37 semaines l'enfant est à terme. Donc vous avez des prématurés de 36 semaines, eux ils se comporteront pratiquement comme les enfants nés à terme. C'est vrai qu'il est prématuré mais après tous les réflexes sont développés, il peut attraper le sein correctement, il peut s'adapter plus facilement. Maintenant vous avez des grands prématurés qui sont autour de 28 semaines. Donc souvent le poids c'est 1,2 kg, 1,3 kg, souvent au maximum 1,5 kg. C'est le gros défi, c'est à leur niveau. Puisque, eux, très souvent on ne peut pas les alimenter correctement ici, et ils sont la plupart du temps évacués à l'hôpital. [00:18:04-2](http://localhost:2300/file=C:/Users/USER/Desktop/ENTRETIENS%20PREMATURITE/CMA-DO/GENYCOLOGUE%2002.mp3time=1084200)

00:13:22-7 Interviewer: Maintenant, est-ce que selon vous, la naissance prématurée est un défi de santé publique auquel vous êtes confronté ? 00:13:32-3

00:13:32-3 Person 1: C'est un défi pour nous, mais je pense que ça l'est pour les autres centres aussi. Parce que la majorité de nos hospitalisations sont des femmes enceintes hospitalisées. Ce sont les femmes enceintes qui ne sont pas à terme. Soit pour une pathologie donnée, c'est une femme qui n'est pas à terme, mais elle se retrouve être hospitalisée. Le défi maintenant, pour nous, est de pouvoir conduire cette grossesse à terme ou bien de conduire cette grossesse jusqu'à ce que l'enfant ait un terme de maturité suffisant pour un accouchement ou une césarienne. C'est vraiment un problème que nous vivons tous les jours. On a des femmes hospitalisées en grossesse dont la grossesse n'est pas à terme. Et souvent, les pathologies font telle qu'on est obligé de faire un accouchement prématuré. 00:14:18-7

00:14:18-7 Interviewer: Est-ce que la naissance prématurée est un défi pour vous ? 00:14:21-0

00:14:21-0 Person 1: Si, si. Si l'enfant naît avant le terme, alors ça expose un certain nombre de problèmes. Et donc, dans le service, on prend des mesures, on essaie de prendre des mesures, on n'a pas tout ce qu'il faut. On essaie de prendre des mesures pour permettre de mettre l'enfant dans des situations acceptables pour sa survie. 00:14:40-3

00:14:40-3 Interviewer: Quand vous dites que vous prenez des mesures, vous faites référence à quel type de mesures ? 00:14:43-8

00:14:43-8 Person 1: Dès la naissance, puisqu'on a une unité kangourou, ce n'est pas une unité de pédiatrie, mais c'est une unité où les agents ont été formés pour prendre en charge les enfants prématurés qui ne sont pas malades. Donc si l'enfant est prématuré, dès la naissance, l'enfant est confié à cette unité. Mais à l'intérieur de l'équipe des sage-femmes qui font les accouchements, beaucoup ont déjà été initiés au soin lié à la prématurité. Donc déjà, c'est de mettre l'enfant dans une atmosphère chaude, c'est-à-dire qu'on l'enveloppe assez convenablement pour ne pas le mettre en contact avec le courant d'air extérieur. Si par exemple, il y a les ventilateurs, on les arrête. Mais ça, c'est valable pour tous les accouchements. Et si l'enfant a des bons réflexes, c'est-à-dire s'il s'adapte correctement, on le confie à l'unité kangourou. Eux (l'unité kangourou), ils vont maintenant éduquer la maman et les autres membres de la famille sur comment alimenter cet enfant, comment garder cet enfant au chaud jusqu'à ce qu'il ait un poids acceptable pour pouvoir se comporter comme les autres bébés. 00:15:57-9

00:15:57-9 Interviewer: Un poids acceptable ? 00:15:58-9

00:15:58-9 Person 1: Oui, c'est-à-dire chez nous, le poids minimum acceptable, c'est 2,5 kg. Donc si l'enfant est par exemple à 2 kg, généralement il est prématuré, mais ce n'est pas toujours qu'il est à 2 kg qu'il est prématuré, parce qu'il y a des hypotrophes. Il y a des enfants qui sont nés à terme, mais qui ont un poids de moins de 2,5 kg. Donc s'il est prématuré, la plupart du temps le poids est inférieur à 2,5 kg. Donc on le met au chaud, ça c'est s'il a des bons réflexes, s'il peut sucer le sein, s'il peut tenir le sein, ou alors s'il peut boire le lait qu'on va lui donner à la tasse. Donc on le met au chaud et on le confie à l'unité kangourou qui va continuer la suite. Là on dit qu'il est acceptable. Maintenant il se trouve que souvent il y a des enfants prématurés dont le poids est inférieur à 2,5 kg, mais qui ne présentent pas de bons réflexes, ils ne peuvent pas tenir le sein, ils ne peuvent pas sucer correctement, on est obligé de les envoyer à la pédiatrie, à Souro SANOU. 00:17:07-3

00:17:07-3 Interviewer: Est-ce que vous avez souvent des naissances de grands prématurés ici ? 00:17:12-0

00:17:12-0 Person 1: Parce que l'intervalle que j'avais donné c'est de 28 semaines à 37 semaines. Donc 36 semaines et 6 jours l'enfant est toujours prématuré. À partir de 37 semaines l'enfant est à terme. Donc vous avez des prématurés de 36 semaines, eux ils se comporteront pratiquement comme les enfants nés à terme. C'est vrai qu'il est prématuré mais après tous les réflexes sont développés, il peut attraper le sein correctement, il peut s'adapter plus facilement. Maintenant vous avez des grands prématurés qui sont autour de 28 semaines. Donc souvent le poids c'est 1,2 kg, 1,3 kg, souvent au maximum 1,5 kg. C'est le gros défi, c'est à leur niveau. Puisque, eux, très souvent on ne peut pas les alimenter correctement ici, et ils sont la plupart du temps évacués à l'hôpital. 00:18:04-2

**Interviewer (00:13:22-7):** Do you think premature birth is a public health challenge that you face?

**Person 1 (00:13:32-3):** Yes, it's definitely a challenge, not just for us but for other centers too. A majority of our hospitalizations involve pregnant women who are not full term. The challenge is to manage these pregnancies, either to carry them to term or until the child is mature enough for delivery or cesarean section. We frequently encounter pregnant women hospitalized with pregnancies that aren't full term, often leading to premature births.

**Interviewer (00:14:18-7):** Is premature birth a challenge for you?

**Person 1 (00:14:21-0):** Yes, premature birth presents numerous problems. We take measures, though we lack some resources, to ensure that the premature baby is in a viable condition for survival.

**Interviewer (00:14:40-3):** What type of measures are you referring to?

**Person 1 (00:14:43-8):** From birth, we utilize our kangaroo unit. It's not a pediatric unit but is staffed with trained personnel for caring for non-sick premature babies. We focus on keeping the child warm, avoiding contact with external air, and if the child shows good reflexes and adapts well, we entrust them to the kangaroo unit. This unit then educates the mother and family on how to feed and keep the child warm until they reach an acceptable weight to behave like other babies.

**Interviewer (00:15:57-9):** What is considered an acceptable weight?

**Person 1 (00:15:58-9):** In our facility, the minimum acceptable weight is 2.5 kg. Premature babies usually weigh less than this. If the child has good reflexes and can feed properly, we provide care until they reach this weight. However, if they can't feed or suck properly and are below 2.5 kg, they are usually sent to pediatrics in Souro Sanou.

**Interviewer (00:17:07-3):** Do you often have very premature births here?

**Person 1 (00:17:12-0):** Yes, we see a range of prematurity. Babies born between 28 and 37 weeks are considered premature. Those around 36 weeks can behave like full-term babies, but very premature babies, around 28 weeks and often weighing between 1.2 to 1.5 kg, are the biggest challenge. They can't be fed properly here and are usually evacuated to the hospital.

### Chief physician of Do health district

[00:18:33-4](http://localhost:2300/file=C:/Users/USER/Desktop/ENTRETIENS%20PREMATURITE/CMA-DO/MCD-DO.mp3time=1113400) Interviewer: Maintenant, est-ce que la naissance prématurée est un défi de santé publique auquel vous êtes confrontés? [00:18:42-7](http://localhost:2300/file=C:/Users/USER/Desktop/ENTRETIENS%20PREMATURITE/CMA-DO/MCD-DO.mp3time=1122700)

[00:18:42-7](http://localhost:2300/file=C:/Users/USER/Desktop/ENTRETIENS%20PREMATURITE/CMA-DO/MCD-DO.mp3time=1122700) Person 1: Oui, on a quelques cas de prématurité qui sont un peu emmerdants quand même. Parce que, en fait, ça dépend du stade de prématurité. Parce que les grandes prématurités sont difficiles à gérer. Dans ce sens que nous, nous n'avons pas, à un certain degré, tout le dispositif pour pouvoir gérer. Donc, on est obligé dans ces cas d'envoyer vers l'hôpital. Sinon ceux qui sont en tout cas à un poids raisonnable, on a un service de soins bébés kangourous qui nous permet quand même d'assister la maman et son bébé jusqu'à ce que les poids soient suffisamment intéressants pour faciliter vraiment la croissance. Donc, ce dispositif nous manque, pour répondre. Et aussi, à un certain niveau, la formation qui va avec pour répondre suffisamment. Parce que ce sont des situations qui ne sont pas simples pour l'agent de santé pour répondre à tout moment. Donc, oui, c'est un autre défi pour nous. Donc, on espère qu'à l'avenir, on va être plus opérationnel pour répondre à ça. [00:20:14-2](http://localhost:2300/file=C:/Users/USER/Desktop/ENTRETIENS%20PREMATURITE/CMA-DO/MCD-DO.mp3time=1214200)

00:18:33-4 Interviewer: Maintenant, est-ce que la naissance prématurée est un défi de santé publique auquel vous êtes confrontés ? 00:18:42-7

00:18:42-7 Person 1: Oui, on a quelques cas de prématurité qui sont un peu embêtants quand même. Parce que, en fait, ça dépend du stade de prématurité. Parce que les grandes prématurités sont difficiles à gérer. Dans ce sens que nous, nous n'avons pas, à un certain degré, tout le dispositif pour pouvoir gérer. Donc, on est obligé dans ces cas d'envoyer vers l'hôpital. Sinon ceux qui sont en tout cas à un poids raisonnable, on a un service de soins bébés kangourous qui nous permet quand même d'assister la maman et son bébé jusqu'à ce que les poids soient suffisamment intéressants pour faciliter vraiment la croissance. Donc, ce dispositif nous manque, pour répondre. Et aussi, à un certain niveau, la formation qui va avec pour répondre suffisamment. Parce que ce sont des situations qui ne sont pas simples pour l'agent de santé pour répondre à tout moment. Donc, oui, c'est un autre défi pour nous. Donc, on espère qu'à l'avenir, on va être plus opérationnel pour répondre à ça. 00:20:14-2

**Interviewer (00:18:33-4):** Is premature birth a public health challenge that you face?

**Person 1 (00:18:42-7):** Yes, prematurity presents significant challenges. The difficulty lies in the degree of prematurity; extreme cases are particularly hard to manage due to limited resources. In such instances, we are compelled to send the babies to the hospital. However, for those with reasonable birth weights, we have a kangaroo baby care service that supports the mother and her baby until the baby reaches a weight conducive to growth. Our current limitations include a lack of equipment and training necessary to effectively handle these situations. They are challenging for health workers to manage effectively at any time. Hence, prematurity is indeed a significant challenge for us, and we are hopeful for improvements in our operational capacity to better address these challenges in the future.

### Manager for maternity care unit of Do medical center with surgical branch

[00:14:16-0](http://localhost:2300/file=C:/Users/USER/Desktop/ENTRETIENS%20PREMATURITE/CMA-DO/RESPONSABLE%20UNITE%20DE%20SOIN%20CMA-DO.mp3time=856000) Interviewer: Est-ce que, pour vous, la naissance prématurée est un défi de santé publique? [00:14:23-8](http://localhost:2300/file=C:/Users/USER/Desktop/ENTRETIENS%20PREMATURITE/CMA-DO/RESPONSABLE%20UNITE%20DE%20SOIN%20CMA-DO.mp3time=863800)

[00:14:23-8](http://localhost:2300/file=C:/Users/USER/Desktop/ENTRETIENS%20PREMATURITE/CMA-DO/RESPONSABLE%20UNITE%20DE%20SOIN%20CMA-DO.mp3time=863800) Person 1: Bon. Un défi...Oui et puis non. Parce que, bon, la prise en charge, en fait, des fois... D'abord, le diagnostic, il arrive de fois, c'est pas bien posé. Parce que le contexte d'accouchement aussi doit suivre.Parce que si on arrive à savoir que je suis en face d'un accouchement prématuré, il y a des précautions à prendre pour faire l'accouchement. Voilà. Et également, vous allez voir peut-être dans les précautions là, si c'est un accouchement prématuré qui se présente en siège.Vous savez que si cet accouchement doit avoir, par exemple, par voie basse, c'est des complications. On pourra même sortir un mort-né. Voilà. Donc, on va aller pour la césariènne. Alors que si le diagnostic n'est pas bien posé, ça veut dire que l'accouchement aussi, la technique d'accouchement, l'accouchement aussi va prendre un coup. Donc, on va se retrouver avec un bébé prématuré. Peut-être qu'on a réanimé. Et également, le système de référence aussi pour pouvoir prendre en charge. Parce qu'au niveau CMA ici, nous, on les réfère, les prématurés. Voilà. On les réfère au niveau CHU.Donc, là-bas également, la prise en charge aussi n'est pas vraiment simple. Et même du côté aussi des familles aussi. Parce que la prise en charge du prématuré est vraiment, voilà, spécifique surtout dans notre contexte où on a tendance rapidement à vouloir laver le nouveau-né, à vouloir faire si...Alors qu'avec le prématuré, il y a vraiment, vraiment, il faut un suivi. Et il faut vraiment des precautions pour ne pas vraiment avoir, soit des répercussions sur cet enfant. Voilà. Donc, la prise en charge, vraiment, je pense que c'est pas à notre niveau. Mais sur toute la chaîne, on a vraiment des difficultés pour pouvoir le faire. [00:16:16-0](http://localhost:2300/file=C:/Users/USER/Desktop/ENTRETIENS%20PREMATURITE/CMA-DO/RESPONSABLE%20UNITE%20DE%20SOIN%20CMA-DO.mp3time=976000)

00:14:16-0 Interviewer: Est-ce que, pour vous, la naissance prématurée est un défi de santé publique ? 00:14:23-8

00:14:23-8 Person 1: Bon. Un défi... Oui et puis non. Parce que, bon, la prise en charge, en fait, des fois ... D'abord, le diagnostic, il arrive de fois, ce n'est pas bien posé. Parce que le contexte d'accouchement aussi doit suivre. Parce que si on arrive à savoir que je suis en face d'un accouchement prématuré, il y a des précautions à prendre pour faire l'accouchement. Voilà. Et également, vous allez voir peut-être dans les précautions là, si c'est un accouchement prématuré qui se présente en siège. Vous savez que si cet accouchement doit avoir, par exemple, par voie basse, c'est des complications. On pourra même sortir un mort-né. Voilà. Donc, on va aller pour la césarienne. Alors que si le diagnostic n'est pas bien posé, ça veut dire que l'accouchement aussi, la technique d'accouchement, l'accouchement aussi va prendre un coup. Donc, on va se retrouver avec un bébé prématuré. Peut-être qu'on a réanimé. Et également, le système de référence aussi pour pouvoir prendre en charge. Parce qu'au niveau CMA ici, nous, on les réfère, les prématurés. Voilà. On les réfère au niveau CHU. Donc, là-bas également, la prise en charge aussi n'est pas vraiment simple. Et même du côté aussi des familles aussi. Parce que la prise en charge du prématuré est vraiment, voilà, spécifique surtout dans notre contexte où on a tendance rapidement à vouloir laver le nouveau-né, à vouloir faire si... Alors qu'avec le prématuré, il y a vraiment, vraiment, il faut un suivi. Et il faut vraiment des précautions pour ne pas vraiment avoir, soit des répercussions sur cet enfant. Voilà. Donc, la prise en charge, vraiment, je pense que ce n'est pas à notre niveau. Mais sur toute la chaîne, on a vraiment des difficultés pour pouvoir le faire. 00:16:16-0

**Interviewer (00:14:16-0):** Is premature birth a public health challenge for you?

**Person 1 (00:14:23-8):** It's both a challenge and not. Firstly, the diagnosis of prematurity is sometimes inaccurately made, impacting the management of childbirth. If a premature birth, especially in a breech position, is not correctly diagnosed, the chosen method of delivery, like vaginal birth, could lead to complications, including stillbirth. Therefore, a cesarean section might be preferred. If the diagnosis is inaccurate, it affects the entire delivery process and the outcome for the premature baby. After birth, these babies are usually referred to the CHU (Central Hospital Unit) due to the specific and complex care they require, which is challenging at the CMA level. Additionally, family involvement and understanding are crucial in caring for premature babies. Our context often leans towards immediate newborn care practices like bathing, which might not be suitable for premature babies. They need careful monitoring and specific care to avoid any negative impacts. Thus, effectively supporting premature births is a challenge not just at our level but throughout the entire healthcare chain.

### Focus group of Colma1 health and social promotion center

[00:14:13-9](http://localhost:2300/file=C:/Users/USER/Desktop/ENTRETIENS%20PREMATURITE/CSPS-COLMA1/FOCUS%20GROUPE%20COLMA1.mp3time=853900) Interviewer: Est ce que la naissance prématurée represente un defi de santé publique important auquel votre centre de santé est confronté? [00:14:22-9](http://localhost:2300/file=C:/Users/USER/Desktop/ENTRETIENS%20PREMATURITE/CSPS-COLMA1/FOCUS%20GROUPE%20COLMA1.mp3time=862900)

[00:14:22-9](http://localhost:2300/file=C:/Users/USER/Desktop/ENTRETIENS%20PREMATURITE/CSPS-COLMA1/FOCUS%20GROUPE%20COLMA1.mp3time=862900) Person 3: Oui, bien sûr. [00:14:24-3](http://localhost:2300/file=C:/Users/USER/Desktop/ENTRETIENS%20PREMATURITE/CSPS-COLMA1/FOCUS%20GROUPE%20COLMA1.mp3time=864300)

[00:14:24-3](http://localhost:2300/file=C:/Users/USER/Desktop/ENTRETIENS%20PREMATURITE/CSPS-COLMA1/FOCUS%20GROUPE%20COLMA1.mp3time=864300) Interviewer: Quels sont les defis auxquels vous etes confrontés? [00:14:28-3](http://localhost:2300/file=C:/Users/USER/Desktop/ENTRETIENS%20PREMATURITE/CSPS-COLMA1/FOCUS%20GROUPE%20COLMA1.mp3time=868300)

[00:14:28-3](http://localhost:2300/file=C:/Users/USER/Desktop/ENTRETIENS%20PREMATURITE/CSPS-COLMA1/FOCUS%20GROUPE%20COLMA1.mp3time=868300) Person 2: C'est un défi parce que, ça nécessite une prise en charge spécifique, il faut avoir les moyens, par exemple, l'enfant doit être pris en charge dans une couveuse. nous au niveau du CSPS on en a pas, même au CMA,il n y en a pas, il faut forcément receuillir les enfants au CHURSS.donc ça necessite une prise en charge specifique. [00:14:51-2](http://localhost:2300/file=C:/Users/USER/Desktop/ENTRETIENS%20PREMATURITE/CSPS-COLMA1/FOCUS%20GROUPE%20COLMA1.mp3time=891200)

[00:14:51-2](http://localhost:2300/file=C:/Users/USER/Desktop/ENTRETIENS%20PREMATURITE/CSPS-COLMA1/FOCUS%20GROUPE%20COLMA1.mp3time=891200) Interviewer: Donc le defi se trouve au niveau de l'équipement? [00:14:52-5](http://localhost:2300/file=C:/Users/USER/Desktop/ENTRETIENS%20PREMATURITE/CSPS-COLMA1/FOCUS%20GROUPE%20COLMA1.mp3time=892500)

[00:14:52-5](http://localhost:2300/file=C:/Users/USER/Desktop/ENTRETIENS%20PREMATURITE/CSPS-COLMA1/FOCUS%20GROUPE%20COLMA1.mp3time=892500) Person 2: oui au niveau de l'équipement. [00:14:54-6](http://localhost:2300/file=C:/Users/USER/Desktop/ENTRETIENS%20PREMATURITE/CSPS-COLMA1/FOCUS%20GROUPE%20COLMA1.mp3time=894600)

[00:14:54-6](http://localhost:2300/file=C:/Users/USER/Desktop/ENTRETIENS%20PREMATURITE/CSPS-COLMA1/FOCUS%20GROUPE%20COLMA1.mp3time=894600) Interviewer: Est ce qu'au niveau de l'accouchement, il y a un defi par rapport aux naissances prématurées? [00:15:04-7](http://localhost:2300/file=C:/Users/USER/Desktop/ENTRETIENS%20PREMATURITE/CSPS-COLMA1/FOCUS%20GROUPE%20COLMA1.mp3time=904700)

[00:15:04-7](http://localhost:2300/file=C:/Users/USER/Desktop/ENTRETIENS%20PREMATURITE/CSPS-COLMA1/FOCUS%20GROUPE%20COLMA1.mp3time=904700) Person 2: Bien sur que ça represente un defi. [00:15:07-6](http://localhost:2300/file=C:/Users/USER/Desktop/ENTRETIENS%20PREMATURITE/CSPS-COLMA1/FOCUS%20GROUPE%20COLMA1.mp3time=907600)

[00:15:07-6](http://localhost:2300/file=C:/Users/USER/Desktop/ENTRETIENS%20PREMATURITE/CSPS-COLMA1/FOCUS%20GROUPE%20COLMA1.mp3time=907600) Person 5: c'est un defi puisque la vitalité du foetus. quand il sort, s'il n'a pas tous les reflexes comme precedemment dit,cela peut jouer sur sa vie. [00:15:16-8](http://localhost:2300/file=C:/Users/USER/Desktop/ENTRETIENS%20PREMATURITE/CSPS-COLMA1/FOCUS%20GROUPE%20COLMA1.mp3time=916800)

[00:15:16-8](http://localhost:2300/file=C:/Users/USER/Desktop/ENTRETIENS%20PREMATURITE/CSPS-COLMA1/FOCUS%20GROUPE%20COLMA1.mp3time=916800) Person 3: Se sont des enfants fragiles, il faut eviter de les exposer à l'hypothermie,parce que, en cas d'hypothermie tu peut facilement le perdre [00:15:29-9](http://localhost:2300/file=C:/Users/USER/Desktop/ENTRETIENS%20PREMATURITE/CSPS-COLMA1/FOCUS%20GROUPE%20COLMA1.mp3time=929900)

[00:15:29-9](http://localhost:2300/file=C:/Users/USER/Desktop/ENTRETIENS%20PREMATURITE/CSPS-COLMA1/FOCUS%20GROUPE%20COLMA1.mp3time=929900) Person 5: Dès fois quand ils sortent, ils ont des detresses respiratoires, il faut les envoyer au CHURSS pour qu'on les assiste.on donne souvent des conseils aux mamans des enfants prématurés [00:15:45-3](http://localhost:2300/file=C:/Users/USER/Desktop/ENTRETIENS%20PREMATURITE/CSPS-COLMA1/FOCUS%20GROUPE%20COLMA1.mp3time=945300)

[00:15:45-3](http://localhost:2300/file=C:/Users/USER/Desktop/ENTRETIENS%20PREMATURITE/CSPS-COLMA1/FOCUS%20GROUPE%20COLMA1.mp3time=945300) Interviewer: quel genre de conseil vous donnez aux mères des enfants prématurés? [00:15:50-6](http://localhost:2300/file=C:/Users/USER/Desktop/ENTRETIENS%20PREMATURITE/CSPS-COLMA1/FOCUS%20GROUPE%20COLMA1.mp3time=950600)

[00:15:50-6](http://localhost:2300/file=C:/Users/USER/Desktop/ENTRETIENS%20PREMATURITE/CSPS-COLMA1/FOCUS%20GROUPE%20COLMA1.mp3time=950600) Person 7: l'allaitement, comment entretenir l'enfant,de ne pas tout le temps le laver, d'eviter de le donner aux visiteurs à tout moment, c'est surtout ses conseils que nous donnons aux mères et aussi bien protèger l'enfant. donc la maman doit vraiment bien s'occuper de l'enfant [00:16:13-0](http://localhost:2300/file=C:/Users/USER/Desktop/ENTRETIENS%20PREMATURITE/CSPS-COLMA1/FOCUS%20GROUPE%20COLMA1.mp3time=973000)

00:14:13-9 Interviewer: Est-ce que la naissance prématurée représente un défi de santé publique important auquel votre centre de santé est confronté ? 00:14:22-9

00:14:22-9 Person 3: Oui, bien sûr. 00:14:24-3

00:14:24-3 Interviewer: Quels sont les défis auxquels vous êtes confrontés ? 00:14:28-3

00:14:28-3 Person 2: C'est un défi parce que, ça nécessite une prise en charge spécifique, il faut avoir les moyens, par exemple, l'enfant doit être pris en charge dans une couveuse. Nous, au niveau du CSPS, on n'en a pas, même au CMA, il n'y en a pas, il faut forcément recueillir les enfants au CHURSS. Donc ça nécessite une prise en charge spécifique. 00:14:51-2

00:14:51-2 Interviewer: Donc le défi se trouve au niveau de l'équipement ? 00:14:52-5

00:14:52-5 Person 2: Oui, au niveau de l'équipement. 00:14:54-6

00:14:54-6 Interviewer: Est-ce qu'au niveau de l'accouchement, il y a un défi par rapport aux naissances prématurées ? 00:15:04-7

00:15:04-7 Person 2: Bien sûr que ça représente un défi. 00:15:07-6

00:15:07-6 Person 5: C'est un défi puisque la vitalité du fœtus. Quand il sort, s'il n'a pas tous les réflexes comme précédemment dit, cela peut jouer sur sa vie. 00:15:16-8

00:15:16-8 Person 3: Ce sont des enfants fragiles, il faut éviter de les exposer à l'hypothermie, parce que, en cas d'hypothermie tu peux facilement le perdre. 00:15:29-9

00:15:29-9 Person 5: Des fois quand ils sortent, ils ont des détresses respiratoires, il faut les envoyer au CHURSS pour qu'on les assiste. On donne souvent des conseils aux mamans des enfants prématurés. 00:15:45-3

00:15:45-3 Interviewer: Quel genre de conseil donnez-vous aux mères des enfants prématurés ? 00:15:50-6

00:15:50-6 Person 7: L'allaitement, comment entretenir l'enfant, de ne pas tout le temps le laver, d'éviter de le donner aux visiteurs à tout moment, c'est surtout ces conseils que nous donnons aux mères et aussi bien protéger l'enfant. Donc la maman doit vraiment bien s'occuper de l'enfant. 00:16:13-0

**Interviewer (00:14:13-9):** Is premature birth a significant public health challenge facing your health center?

**Person 3 (00:14:22-9):** Yes, it certainly is.

**Interviewer (00:14:24-3):** What are the challenges you face?

**Person 2 (00:14:28-3):** Premature birth presents significant challenges, especially in terms of the specialized care required. A primary issue is the lack of necessary equipment, like incubators, at our CSPS and even at the CMA level. We often have to transfer premature infants to CHURSS for adequate care. This lack of equipment makes providing appropriate support for these infants challenging.

**Interviewer (00:14:52-5):** So, the challenge is mainly about equipment?

**Person 2 (00:14:54-6):** Yes, it's largely a matter of having the right equipment.

**Interviewer (00:15:04-7):** In terms of childbirth, is there a challenge in relation to premature births?

**Person 2 (00:15:07-6):** Definitely, it's a challenge.

**Person 5 (00:15:16-8):** The challenge is also about the vitality of the fetus. Premature babies may lack certain reflexes at birth, which could endanger their lives.

**Person 3 (00:15:29-9):** These infants are extremely fragile and must be protected from hypothermia, as it can quickly lead to life-threatening conditions.

**Person 5 (00:15:45-3):** Respiratory distress is another common issue in premature births. When this occurs, we have to send them to CHURSS for specialized assistance. We also offer guidance to mothers of premature infants to help manage these challenges.

**Interviewer (00:15:45-3):** "What advice do you typically offer to mothers with premature babies?"

**Person 7 (00:15:50-6):** "We emphasize several key points: breastfeeding, proper child care, and minimizing washing the baby too frequently. We also advise against allowing visitors to handle the baby indiscriminately. Overall, these tips focus on protecting the child, so it's crucial for the mother to take good care of her baby."

### Major of Colma1 health and social promotion center

[00:11:22-1](http://localhost:2300/file=C:/Users/USER/Desktop/ENTRETIENS%20PREMATURITE/CSPS-COLMA1/MAJOR%20CSPS%20COLMA1.MP3time=682100) Interviewer: d'accord.maintenant est ce que la naissance prematuré est un defis de santé publique important auquel vous etes confrontés dans votre centre de santé? [00:11:32-9](http://localhost:2300/file=C:/Users/USER/Desktop/ENTRETIENS%20PREMATURITE/CSPS-COLMA1/MAJOR%20CSPS%20COLMA1.MP3time=692900)

[00:11:32-9](http://localhost:2300/file=C:/Users/USER/Desktop/ENTRETIENS%20PREMATURITE/CSPS-COLMA1/MAJOR%20CSPS%20COLMA1.MP3time=692900) Person 1: Naissance prématurée? [00:11:35-3](http://localhost:2300/file=C:/Users/USER/Desktop/ENTRETIENS%20PREMATURITE/CSPS-COLMA1/MAJOR%20CSPS%20COLMA1.MP3time=695300)

[00:11:35-3](http://localhost:2300/file=C:/Users/USER/Desktop/ENTRETIENS%20PREMATURITE/CSPS-COLMA1/MAJOR%20CSPS%20COLMA1.MP3time=695300) Interviewer: est ce que cela represente un defi de santé publique au sein de votre centre de santé? [00:11:39-3](http://localhost:2300/file=C:/Users/USER/Desktop/ENTRETIENS%20PREMATURITE/CSPS-COLMA1/MAJOR%20CSPS%20COLMA1.MP3time=699300)

[00:11:39-3](http://localhost:2300/file=C:/Users/USER/Desktop/ENTRETIENS%20PREMATURITE/CSPS-COLMA1/MAJOR%20CSPS%20COLMA1.MP3time=699300) Person 1: Bon au regard, donc de la quantité,Parce que ça arrive, mais c'est pas beaucoup chez nous. Prématuré, il y en a, mais ce n'est pas beaucoup, mais ce n'est pas pour autant qu'il faut dire que ça n'a pas une ampleur parce que c'est une vie. Ce sont des vies qui sont en danger. Voilà, ce sont des vies qui sont en danger, c'est à prendre en compte. On aurait souhaité que les enfants naissent tous à terme. Voilà par ce n'est pas sans conséquence pour un bébé qui naît avant terme voilà donc c'est c'est un défi, vraiment voilà, tout est à prendre en compte, même si les chiffres ne sont pas très très élevés, ça c'est à ma connaissance, peut-être que moi je n'ai pas les chiffres rapporter ce qui fait peut-être que on dirait que je minimise un peu la chose, mais peut-être si on parcourt les registres, il se pourrait que ce soit un peu plus élevé. [00:12:31-7](http://localhost:2300/file=C:/Users/USER/Desktop/ENTRETIENS%20PREMATURITE/CSPS-COLMA1/MAJOR%20CSPS%20COLMA1.MP3time=751700)

[00:12:31-7](http://localhost:2300/file=C:/Users/USER/Desktop/ENTRETIENS%20PREMATURITE/CSPS-COLMA1/MAJOR%20CSPS%20COLMA1.MP3time=751700) Interviewer: maintenant est-ce que le défi peut se présenter au niveau de la grossesse, de l'accouchement ou de la prise en charge à votre niveau ici ? [00:12:40-8](http://localhost:2300/file=C:/Users/USER/Desktop/ENTRETIENS%20PREMATURITE/CSPS-COLMA1/MAJOR%20CSPS%20COLMA1.MP3time=760800)

[00:12:40-8](http://localhost:2300/file=C:/Users/USER/Desktop/ENTRETIENS%20PREMATURITE/CSPS-COLMA1/MAJOR%20CSPS%20COLMA1.MP3time=760800) Interviewer: euh.. parce que ici,au niveau de la grossesse.D'abord, je dirais que, au niveau de la grossesse, on peut pas savoir que c'est c'est cette femme va accoucher d'un prématuré. Voilà donc on peut pas savoir ça maintenant au moment de l'accouchement lorsque ça intervient immediatement Nous n'avons pas les moyens nécessaire ici pour s'occuper donc des prématurés. Voilà on est obligé de les orienter vers le CMA de dô et peut être d'autres même partent se retrouver au CHURSS. la prise en charge c'est , on n'a pas la capacité pour les prendre en charge et on peut voir ça aussi comme un problème. mais quand on se fie donc aux objectifs qu'on assigne à la structure ici, parce que ces cas nous ne pouvons pas les prendre en charge. On dirait que bon en fait les défis se posent pas à ce niveau quoi. voilà ça c'est en d'autres termes. [00:13:37-6](http://localhost:2300/file=C:/Users/USER/Desktop/ENTRETIENS%20PREMATURITE/CSPS-COLMA1/MAJOR%20CSPS%20COLMA1.MP3time=817600)

00:11:22-1 Interviewer: D'accord. Maintenant est-ce que la naissance prématurée est un défi de santé publique important auquel vous êtes confrontés dans votre centre de santé ? 00:11:32-9

00:11:32-9 Person 1: Naissance prématurée ? 00:11:35-3

00:11:35-3 Interviewer: Est-ce que cela représente un défi de santé publique au sein de votre centre de santé ? 00:11:39-3

00:11:39-3 Person 1: Bon, au regard, donc de la quantité, parce que ça arrive, mais c'est pas beaucoup chez nous. Prématuré, il y en a, mais ce n'est pas beaucoup, mais ce n'est pas pour autant qu'il faut dire que ça n'a pas une ampleur parce que c'est une vie. Ce sont des vies qui sont en danger. Voilà, ce sont des vies qui sont en danger, c'est à prendre en compte. On aurait souhaité que les enfants naissent tous à terme. Voilà parce ce n'est pas sans conséquence pour un bébé qui naît avant terme voilà donc c'est, c'est un défi, vraiment voilà, tout est à prendre en compte, même si les chiffres ne sont pas très, très élevés, ça c'est à ma connaissance, peut-être que moi je n'ai pas les chiffres rapporter ce qui fait peut-être que on dirait que je minimise un peu la chose, mais peut-être si on parcourt les registres, il se pourrait que ce soit un peu plus élevé. 00:12:31-7

00:12:31-7 Interviewer: Maintenant est-ce que le défi peut se présenter au niveau de la grossesse, de l'accouchement ou de la prise en charge à votre niveau ici ? 00:12:40-8

00:12:40-8 Person 1: Euh... parce que ici, au niveau de la grossesse. D'abord, je dirais que, au niveau de la grossesse, on ne peut pas savoir que c'est cette femme qui va accoucher d'un prématuré. Voilà donc on ne peut pas savoir ça maintenant au moment de l'accouchement lorsque ça intervient immédiatement. Nous n'avons pas les moyens nécessaires ici pour s'occuper donc des prématurés. Voilà on est obligé de les orienter vers le CMA de Dô et peut-être d'autres même partent se retrouver au CHURSS. La prise en charge, c'est que, on n'a pas la capacité pour les prendre en charge et on peut voir ça aussi comme un problème. Mais quand on se fie donc aux objectifs qu'on assigne à la structure ici, parce que ces cas nous ne pouvons pas les prendre en charge. On dirait que bon en fait les défis ne se posent pas à ce niveau. Voilà ça c'est en d'autres termes. 00:13:37-6

**Interviewer (00:11:22-1):** Is premature birth a significant public health challenge that you face in your health center?

**Person 1 (00:11:32-9):** Premature birth, yes. Although the numbers might not be very high, it's still a critical issue. Each case of prematurity is significant as it involves a life at risk. Even if the statistics don't show a high frequency of such births, the impact and risks associated with each case make it a substantial challenge. It's important to recognize the gravity of each instance, as premature births have serious consequences for the infants.

**Interviewer (00:11:39-3):** Does this represent a public health challenge within your health center?

**Person 1 (00:12:31-7):** Yes, it does. In terms of pregnancy, we cannot predict which woman will have a premature birth. When premature births occur, our facility lacks the necessary resources to provide adequate care for these infants. We have to refer them to larger healthcare facilities like the CMA of Dô or even CHURSS for specialized care. Our limitations in handling these cases mean that the challenges mostly arise due to our inability to provide necessary care within our facility.

### Maternity manager of Colma1 health and social promotion center

[00:19:41-7](http://localhost:2300/file=C:/Users/USER/Desktop/ENTRETIENS%20PREMATURITE/CSPS-COLMA1/RESPONSABLE%20MATERNITE-COLMA1.mp3time=1181700) Interviewer: ok.est ce que la naissance prématurée est un defi de santé publique important auxquels vous êtes confrontés dans votre centre de santé? [00:19:50-9](http://localhost:2300/file=C:/Users/USER/Desktop/ENTRETIENS%20PREMATURITE/CSPS-COLMA1/RESPONSABLE%20MATERNITE-COLMA1.mp3time=1190900)

[00:19:50-9](http://localhost:2300/file=C:/Users/USER/Desktop/ENTRETIENS%20PREMATURITE/CSPS-COLMA1/RESPONSABLE%20MATERNITE-COLMA1.mp3time=1190900) Person 1: Oui. La prématurité, c'est un problème de santé publique parce que je pense que c'est une cause principale, en tout cas, de morbidité et de mortalité périnatale. Vu que le plateau technique n'est pas vraiment accessible partout, quand on prend, par exemple, la prise en charge de la prématurité au Burkina ou à Bobo-Dioulasso, seul le CHURSS, peut vraiment faire un peu. Mais lorsqu' on part là- bas pour un petit regard, vous allez voir que ce n'est pas la joie. Parce que les conditions même de prise en charge, ne sont pas aussi adequat je veux dire (rires). [00:20:46-8](http://localhost:2300/file=C:/Users/USER/Desktop/ENTRETIENS%20PREMATURITE/CSPS-COLMA1/RESPONSABLE%20MATERNITE-COLMA1.mp3time=1246800)

[00:20:46-8](http://localhost:2300/file=C:/Users/USER/Desktop/ENTRETIENS%20PREMATURITE/CSPS-COLMA1/RESPONSABLE%20MATERNITE-COLMA1.mp3time=1246800) Interviewer: Généralement, au sein de votre centre de santé ici, à quel type de défis vous êtes confrontée par rapport aux naissances prématurés ? [00:20:53-9](http://localhost:2300/file=C:/Users/USER/Desktop/ENTRETIENS%20PREMATURITE/CSPS-COLMA1/RESPONSABLE%20MATERNITE-COLMA1.mp3time=1253900)

[00:20:53-9](http://localhost:2300/file=C:/Users/USER/Desktop/ENTRETIENS%20PREMATURITE/CSPS-COLMA1/RESPONSABLE%20MATERNITE-COLMA1.mp3time=1253900) Person 1: vous même vous voyez, je pense que j'ai déjà même dit. Donc, vous voyez, la prise en charge n'est pas vraiment adéquate. Et généralement, même lorsque s'est déjà connu que c'est un accouchement prématuré et la femme vient à petite dilatation, on la réfère. Mais lorsqu'elle vient à sept (7cm) centimètres, c'est le cas de... Le maïtissien, je pense que la dernière fois, avant-hier, ils ont fait un accouchement prématuré de 1 500 grammes. 1 500 grammes, on l'a référé. Et quand on m'a dit, Madame Sawadogo même était là et c'est elle même qui m'a informée. je dis 1 500 grammes, et quelle condition ? L'enfant est parti dans quelle condition ? Oui. Parce qu'il faut vraiment tellement envelopper cet enfant- là. Vraiment, est- ce que c'est évident ? Est- ce que c'est évident ? Est- ce que en voulant vraiment l'envelopper on ne va pas l'etouffer ? Est- ce que, par exemple, le temps d'arriver là- bas, l'hypoglycémie ne va pas partir avec cet enfant ? L'infection, parce que la peau n'est pas mature. Oui, on va dire quoi ? Les poumons ne sont pas pas bien developpés il y a beaucoup. [00:22:17-0](http://localhost:2300/file=C:/Users/USER/Desktop/ENTRETIENS%20PREMATURITE/CSPS-COLMA1/RESPONSABLE%20MATERNITE-COLMA1.mp3time=1337000)

00:19:41-7 Interviewer: Ok. Est-ce que la naissance prématurée est un défi de santé publique important auxquels vous êtes confrontés dans votre centre de santé ? 00:19:50-9

00:19:50-9 Person 1: Oui. La prématurité, c'est un problème de santé publique parce que je pense que c'est une cause principale, en tout cas, de morbidité et de mortalité périnatale. Vu que le plateau technique n'est pas vraiment accessible partout, quand on prend, par exemple, la prise en charge de la prématurité au Burkina ou à Bobo-Dioulasso, seul le CHURSS, peut vraiment faire un peu. Mais lorsqu'on part là-bas pour un petit regard, vous allez voir que ce n'est pas la joie. Parce que les conditions même de prise en charge, ne sont pas aussi adéquates je veux dire (rires). 00:20:46-8

00:20:46-8 Interviewer: Généralement, au sein de votre centre de santé ici, à quel type de défis vous êtes confrontée par rapport aux naissances prématurées ? 00:20:53-9

00:20:53-9 Person 1: Vous même vous voyez, je pense que j'ai déjà même dit. Donc, vous voyez, la prise en charge n'est pas vraiment adéquate. Et généralement, même lorsque c'est déjà connu que c'est un accouchement prématuré et la femme vient à petite dilatation, on la réfère. Mais lorsqu'elle vient à sept (7 cm) centimètres, c'est le cas de... Le maïeuticien, je pense que la dernière fois, avant-hier, ils ont fait un accouchement prématuré de 1 500 grammes. 1 500 grammes, on l'a référé. Et quand on m'a dit, Madame Sawadogo même était là et c'est elle-même qui m'a informée. Je dis 1 500 grammes, et quelle condition ? L'enfant est parti dans quelle condition ? Oui. Parce qu'il faut vraiment tellement envelopper cet enfant-là. Vraiment, est-ce que c'est évident ? Est-ce que c'est évident ? Est-ce qu'en voulant vraiment l'envelopper on ne va pas l'étouffer ? Est-ce que, par exemple, le temps d'arriver là-bas, l'hypoglycémie ne va pas partir avec cet enfant ? L'infection, parce que la peau n'est pas mature. Oui, on va dire quoi ? Les poumons ne sont pas bien développés il y a beaucoup. 00:22:17-0

**Interviewer (00:19:41-7):** Is premature birth a significant public health challenge that you face in your health center?

**Person 1 (00:19:50-9):** Yes, premature birth is indeed a major public health issue. It's a leading cause of perinatal morbidity and mortality. The technical resources required for the proper care of premature babies are not widely accessible, particularly in Burkina or Bobo-Dioulasso. The CHURSS is one of the few facilities that can somewhat manage these cases, but even there, the conditions and resources are not fully adequate.

**Interviewer (00:20:53-9):** Generally, within your health center, what challenges do you encounter with premature births?

**Person 1 (00:20:53-9):** The main challenge is the inadequacy of support for premature births. Often, when a woman is in premature labor, she is referred to another facility, especially if the dilation reaches about 7 cm. For instance, a recent case involved a premature baby weighing 1,500 grams. In such situations, we face dilemmas in ensuring the baby's safe transport to a better-equipped facility. There's a risk of suffocating the baby while trying to keep it warm, the possibility of hypoglycemia developing during transport, and the concern of infection due to the baby's immature skin. Additionally, premature babies often have underdeveloped lungs, which complicates their care even further.

### Focus group of Farakan health and social promotion center

[00:25:09-0](http://localhost:2300/file=C:/Users/USER/Desktop/ENTRETIENS%20PREMATURITE/CSPS_FARAKAN/FOCUS_GROUPE-CSPS_FARAKAN.MP3time=1509000) Interviewer: C'est bon. Ok. Je pense que nous allons poursuivre.La question qui suit maintenant, est-ce que la naissance prématurée est un défi de santé publique important auquel vous êtes confronté dans votre salle de santé ?Est-ce que la naissance prématurée au sein de votre centre de santé est un défi de santé publique ?Est ce que vous êtes confronté à des défis.De la grossesse à l'accouchement et jusqu'aux différents soins. [00:25:42-6](http://localhost:2300/file=C:/Users/USER/Desktop/ENTRETIENS%20PREMATURITE/CSPS_FARAKAN/FOCUS_GROUPE-CSPS_FARAKAN.MP3time=1542600)

[00:25:42-6](http://localhost:2300/file=C:/Users/USER/Desktop/ENTRETIENS%20PREMATURITE/CSPS_FARAKAN/FOCUS_GROUPE-CSPS_FARAKAN.MP3time=1542600) Person 8: C'est pas un défi parce que l'accouchement prématuré même ne montre pas que c'est la faute de l'agent de santé ou bien de la faute de nous qui les suivons, ça peut arriver. Donc c'est pas une defi si ça arrive on le fait, mais on grouille vraiment pour que l'enfant soit dans des bonnes conditions pour pouvoir récupérer.[00:26:17-6](http://localhost:2300/file=C:/Users/USER/Desktop/ENTRETIENS%20PREMATURITE/CSPS_FARAKAN/FOCUS_GROUPE-CSPS_FARAKAN.MP3time=1577600)

[00:26:17-6](http://localhost:2300/file=C:/Users/USER/Desktop/ENTRETIENS%20PREMATURITE/CSPS_FARAKAN/FOCUS_GROUPE-CSPS_FARAKAN.MP3time=1577600) Interviewer: Je réprends la question, est-ce que la naissance prématurée au sein de votre CSPS est un défini de santé publique ? [00:26:32-2](http://localhost:2300/file=C:/Users/USER/Desktop/ENTRETIENS%20PREMATURITE/CSPS_FARAKAN/FOCUS_GROUPE-CSPS_FARAKAN.MP3time=1592200)

[00:26:32-2](http://localhost:2300/file=C:/Users/USER/Desktop/ENTRETIENS%20PREMATURITE/CSPS_FARAKAN/FOCUS_GROUPE-CSPS_FARAKAN.MP3time=1592200) Person 5: Non, c'est pas un défi. Pourquoi ? Parce qu'Il y a certaines femmes de nature même, elles ne font que faire des prématurer, c'est inévitable chez eux voilà, mais il y a d'autres c'est malgré eux.Elles n'ont pas voulu, donc nous les agents de santé aussi, personne ne souhaite, mais quand ça vient, pourvu que ça se passe dans des bonnes conditions et que nous puissions sauver l'innocent bébé qui viens d'arriver, qu'il puisse être dans les bons termes que la suite aussi continue, que l'évolution soi bien fait pour lui, voilà. [00:27:14-8](http://localhost:2300/file=C:/Users/USER/Desktop/ENTRETIENS%20PREMATURITE/CSPS_FARAKAN/FOCUS_GROUPE-CSPS_FARAKAN.MP3time=1634800)

[00:27:14-8](http://localhost:2300/file=C:/Users/USER/Desktop/ENTRETIENS%20PREMATURITE/CSPS_FARAKAN/FOCUS_GROUPE-CSPS_FARAKAN.MP3time=1634800) Person 3: C'est un défi pour nous parce que on a même créé une unité kangourou dans notre district qui fonctionne très bien, elle est même l'un des meilleurs dans la ville ici donc, il y a un protocole, quand nous recevons par exemple un accouchement prématuré il nous démande même depuis le SMK (soins mères kangourou) que nous pouvons faire la maturation pulmonaire, si possible évacuer la femme pour qu'elle accouche déjà au sein du CMA là-bas pour que la prise en charge soit plus facile. Dans les cas extrêmes, dès que nous faisons l'accouchement l'enfant est acheminé automatiquement, et concernant aussi la prévention, bon, ces derniers temps et malgré le manque de sulphadoxine, en tout cas, on grouille vraiment pour prévenir les accouchements prématurés avec la gratuité, franchement c'est une urgence. [00:28:09-9](http://localhost:2300/file=C:/Users/USER/Desktop/ENTRETIENS%20PREMATURITE/CSPS_FARAKAN/FOCUS_GROUPE-CSPS_FARAKAN.MP3time=1689900)

[00:28:09-9](http://localhost:2300/file=C:/Users/USER/Desktop/ENTRETIENS%20PREMATURITE/CSPS_FARAKAN/FOCUS_GROUPE-CSPS_FARAKAN.MP3time=1689900) Interviewer: Quand vous parlez d'unité kangourou, qu'est ce que cela signifie? [00:28:13-9](http://localhost:2300/file=C:/Users/USER/Desktop/ENTRETIENS%20PREMATURITE/CSPS_FARAKAN/FOCUS_GROUPE-CSPS_FARAKAN.MP3time=1693900)

[00:28:13-9](http://localhost:2300/file=C:/Users/USER/Desktop/ENTRETIENS%20PREMATURITE/CSPS_FARAKAN/FOCUS_GROUPE-CSPS_FARAKAN.MP3time=1693900) Person 3: L'unité kangourou est destinée aux prématurés.Ça n'y est pas ici, mais c'est au sein de notre district. [00:28:29-8](http://localhost:2300/file=C:/Users/USER/Desktop/ENTRETIENS%20PREMATURITE/CSPS_FARAKAN/FOCUS_GROUPE-CSPS_FARAKAN.MP3time=1709800)

00:25:09-0 Interviewer: C'est bon. Ok. Je pense que nous allons poursuivre. La question qui suit maintenant : Est-ce que la naissance prématurée est un défi de santé publique important auquel vous êtes confronté dans votre salle de santé ? Est-ce que la naissance prématurée au sein de votre centre de santé est un défi de santé publique ? Est-ce que vous êtes confronté à des défis, de la grossesse à l'accouchement et jusqu'aux différents soins ? 00:25:42-6

00:25:42-6 Person 8: Ce n'est pas un défi, car l'accouchement prématuré même ne montre pas que c'est la faute de l'agent de santé ou bien de la faute de nous qui les suivons, ça peut arriver. Donc ce n'est pas un défi, si ça arrive, on le fait, mais on se hâte vraiment pour que l'enfant soit dans de bonnes conditions pour pouvoir récupérer. 00:26:17-6

00:26:17-6 Interviewer: Je reprends la question, est-ce que la naissance prématurée au sein de votre CSPS est un défi de santé publique ? 00:26:32-2

00:26:32-2 Person 5: Non, ce n'est pas un défi. Pourquoi ? Parce qu'il y a certaines femmes de nature même, elles ne font que faire des prématurés, c'est inévitable chez eux voilà, mais il y en a d'autres, c'est malgré elles. Elles n'ont pas voulu, donc nous, les agents de santé aussi, personne ne souhaite, mais quand ça vient, pourvu que ça se passe dans de bonnes conditions et que nous puissions sauver l'innocent bébé qui vient d'arriver, qu'il puisse être dans les bonnes conditions pour que la suite aussi continue, que l'évolution soit bien faite pour lui, voilà. 00:27:14-8

00:27:14-8 Person 3: C'est un défi pour nous, car on a même créé une unité kangourou dans notre district qui fonctionne très bien. Elle est même l'une des meilleures dans la ville ici donc, il y a un protocole, quand nous recevons par exemple un accouchement prématuré, il nous demande même depuis le SMK (soins mères kangourou) que nous pouvons faire la maturation pulmonaire, si possible évacuer la femme pour qu'elle accouche déjà au sein du CMA là-bas pour que la prise en charge soit plus facile. Dans les cas extrêmes, dès que nous faisons l'accouchement, l'enfant est acheminé automatiquement, et concernant aussi la prévention, bon, ces derniers temps et malgré le manque de sulfadoxine, en tout cas, on se hâte vraiment pour prévenir les accouchements prématurés avec la gratuité, franchement c'est une urgence. 00:28:09-9

00:28:09-9 Interviewer: Quand vous parlez d'unité kangourou, qu'est-ce que cela signifie ? 00:28:13-9

00:28:13-9 Person 3: L'unité kangourou est destinée aux prématurés. Ça n'y est pas ici, mais c'est au sein de notre district. 00:28:29-8

**Interviewer (00:25:09-0):** Is preterm birth a significant public health challenge in your healthcare setting? Are there challenges you face, from pregnancy to childbirth and even various treatments?

**Person 8 (00:25:42-6):** Premature birth is not seen as a challenge per se because it's not necessarily the fault of the health worker or the healthcare system. It's a situation that can occur naturally. When it does happen, we make sure to act swiftly and ensure the child is in good condition for recovery.

**Interviewer (00:26:32-2):** Is premature birth within your CSPS a public health challenge?

**Person 5 (00:26:32-2):** It's not a challenge in the conventional sense. Some women naturally have premature births, and while it's not desired, it's inevitable in some cases. Our role as health workers is to ensure the well-being of the baby when such situations arise, ensuring proper care and monitoring for optimal recovery and development.

**Person 3 (00:27:14-8):** It is a challenge for us. We've even established a kangaroo unit in our district, which is performing well and is one of the best in the city. We have protocols in place for premature deliveries, including lung maturation and possible evacuation of the mother for delivery in better-equipped facilities. In severe cases, we ensure immediate care of the newborn. We are also proactive in preventing premature births, despite some resource limitations. The kangaroo unit specifically caters to premature babies, providing specialized care.

**Interviewer (00:28:13-9):** What is a kangaroo unit?

**Person 3 (00:28:13-9):** The kangaroo unit is a specialized facility for the care of premature babies. It's not located here but is part of our district healthcare services.

### Major of Farakan health and social promotion center

[00:09:38-0](http://localhost:2300/file=C:/Users/USER/Desktop/ENTRETIENS%20PREMATURITE/CSPS_FARAKAN/MAJOR%20FARAKAN.MP3time=578000) Interviewer: Est ce la naissance prématurée est un defi de santé publique important auxquelles vous êtes confronté au sein de votre centre de santé? [00:09:47-7](http://localhost:2300/file=C:/Users/USER/Desktop/ENTRETIENS%20PREMATURITE/CSPS_FARAKAN/MAJOR%20FARAKAN.MP3time=587700)

[00:09:47-7](http://localhost:2300/file=C:/Users/USER/Desktop/ENTRETIENS%20PREMATURITE/CSPS_FARAKAN/MAJOR%20FARAKAN.MP3time=587700) Person 1: En tout cas c'est un problème, parce qu'un bébé qui est né prématuré a besoin de beaucoup de soins et d'attentions pour en tout cas pouvoir survivre.Cela veut dire que toute vie humaine c'est une priorité.Nous essayons de les prendre en charge pour leur survie. [00:10:11-6](http://localhost:2300/file=C:/Users/USER/Desktop/ENTRETIENS%20PREMATURITE/CSPS_FARAKAN/MAJOR%20FARAKAN.MP3time=611600)

[00:10:11-6](http://localhost:2300/file=C:/Users/USER/Desktop/ENTRETIENS%20PREMATURITE/CSPS_FARAKAN/MAJOR%20FARAKAN.MP3time=611600) Interviewer: A quel niveau se situe le defi? Pendant la grossesse, pendant l'accouchement ou pendant la prise en charge? [00:10:17-2](http://localhost:2300/file=C:/Users/USER/Desktop/ENTRETIENS%20PREMATURITE/CSPS_FARAKAN/MAJOR%20FARAKAN.MP3time=617200)

[00:10:17-2](http://localhost:2300/file=C:/Users/USER/Desktop/ENTRETIENS%20PREMATURITE/CSPS_FARAKAN/MAJOR%20FARAKAN.MP3time=617200) Person 1: C'est partout.Depuis la grossesse, parce qu'il faut bien suivre les grossesses pour éviter la prématurité, au niveau de l'accouchement aussi, il faut que ça soit bien conduit pour que le bébé sorte en bonne santé et surtout après la naissance là,il faut bien entretenir pour qu'il puisse survivre. [00:10:36-6](http://localhost:2300/file=C:/Users/USER/Desktop/ENTRETIENS%20PREMATURITE/CSPS_FARAKAN/MAJOR%20FARAKAN.MP3time=636600)

00:09:38-0 Interviewer: Est-ce que la naissance prématurée est un défi de santé publique important auquel vous êtes confronté au sein de votre centre de santé ? 00:09:47-7

00:09:47-7 Person 1: En tout cas, c'est un problème, parce qu'un bébé qui est né prématuré a besoin de beaucoup de soins et d'attentions pour en tout cas pouvoir survivre. Cela veut dire que toute vie humaine c'est une priorité. Nous essayons de les prendre en charge pour leur survie. 00:10:11-6

00:10:11-6 Interviewer: A quel niveau se situe le défi ? Pendant la grossesse, pendant l'accouchement ou pendant la prise en charge ? 00:10:17-2

00:10:17-2 Person 1: C'est partout. Depuis la grossesse, parce qu'il faut bien suivre les grossesses pour éviter la prématurité, au niveau de l'accouchement aussi, il faut que ça soit bien conduit pour que le bébé sorte en bonne santé et surtout après la naissance là, il faut bien entretenir pour qu'il puisse survivre. 00:10:36-6

**Interviewer (00:09:38-0):** Is premature birth a significant public health challenge at your health center?

**Person 1 (00:09:47-7):** Definitely, it's a problem. Premature babies require extensive care and attention to survive. Every human life is a priority, so we focus on caring for these babies to ensure their survival.

**Interviewer (00:10:11-6):** At which stage do you find the challenge most significant? Is it during pregnancy, childbirth, or the treatment phase?

**Person 1 (00:10:17-2):** The challenge is present at all stages. During pregnancy, it's crucial to monitor women closely to prevent premature births. Childbirth needs to be well-managed to ensure the health of the baby. And after birth, the newborn requires proper maintenance and care to survive. Each phase is critical in its own right.

### Maternity manager of Farakan health and social promotion center

[00:13:54-1](http://localhost:2300/file=C:/Users/USER/Desktop/ENTRETIENS%20PREMATURITE/CSPS_FARAKAN/RESPONSABLE%20MATERNITE%20FARAKAN.MP3time=834100) Interviewer: D'accord,maintenant la question suivante.Est ce que la naissance est un defi de santé publique importante auquel vous êtes confronter dans votre centre de santé ? [00:14:05-9](http://localhost:2300/file=C:/Users/USER/Desktop/ENTRETIENS%20PREMATURITE/CSPS_FARAKAN/RESPONSABLE%20MATERNITE%20FARAKAN.MP3time=845900)

[00:14:05-9](http://localhost:2300/file=C:/Users/USER/Desktop/ENTRETIENS%20PREMATURITE/CSPS_FARAKAN/RESPONSABLE%20MATERNITE%20FARAKAN.MP3time=845900) Person 1: En tout cas c'est un defi important. Je sais qu'entre temps il y avait un projet qui était venu pour s'occuper des bébés de faible poids de naissance. [00:14:24-6](http://localhost:2300/file=C:/Users/USER/Desktop/ENTRETIENS%20PREMATURITE/CSPS_FARAKAN/RESPONSABLE%20MATERNITE%20FARAKAN.MP3time=864600)

[00:14:24-6](http://localhost:2300/file=C:/Users/USER/Desktop/ENTRETIENS%20PREMATURITE/CSPS_FARAKAN/RESPONSABLE%20MATERNITE%20FARAKAN.MP3time=864600) Interviewer: Généralement,à quel type de défi vous faites face par rapport aux naissances prématurées ? [00:14:34-3](http://localhost:2300/file=C:/Users/USER/Desktop/ENTRETIENS%20PREMATURITE/CSPS_FARAKAN/RESPONSABLE%20MATERNITE%20FARAKAN.MP3time=874300)

[00:14:34-3](http://localhost:2300/file=C:/Users/USER/Desktop/ENTRETIENS%20PREMATURITE/CSPS_FARAKAN/RESPONSABLE%20MATERNITE%20FARAKAN.MP3time=874300) Person 1: c'est de prévenir.Prévenir l'accouchement et tout ce qui va avec, cela va passe par l'interrogatoire, ça va passer par surtout l'interrogatoire.On sait que souvent les approches, les naissances prématurées, les femmes, souvent avec les infections, en tout cas la plupart de nos jours-là c'est surtout les infections et puis aussi les accouchements gémelaires. [00:15:06-4](http://localhost:2300/file=C:/Users/USER/Desktop/ENTRETIENS%20PREMATURITE/CSPS_FARAKAN/RESPONSABLE%20MATERNITE%20FARAKAN.MP3time=906400)

[00:15:06-4](http://localhost:2300/file=C:/Users/USER/Desktop/ENTRETIENS%20PREMATURITE/CSPS_FARAKAN/RESPONSABLE%20MATERNITE%20FARAKAN.MP3time=906400) Interviewer: lorsque vous parlez d'infections, vous faites allusion àquel type d'infections? [00:15:09-2](http://localhost:2300/file=C:/Users/USER/Desktop/ENTRETIENS%20PREMATURITE/CSPS_FARAKAN/RESPONSABLE%20MATERNITE%20FARAKAN.MP3time=909200)

[00:15:09-2](http://localhost:2300/file=C:/Users/USER/Desktop/ENTRETIENS%20PREMATURITE/CSPS_FARAKAN/RESPONSABLE%20MATERNITE%20FARAKAN.MP3time=909200) Person 1: Surtout les infections urinaires. [00:15:17-1](http://localhost:2300/file=C:/Users/USER/Desktop/ENTRETIENS%20PREMATURITE/CSPS_FARAKAN/RESPONSABLE%20MATERNITE%20FARAKAN.MP3time=917100)

[00:15:17-1](http://localhost:2300/file=C:/Users/USER/Desktop/ENTRETIENS%20PREMATURITE/CSPS_FARAKAN/RESPONSABLE%20MATERNITE%20FARAKAN.MP3time=917100) Interviewer: Est ce que les accouchements prématurés répresentent un defi pour vous au sein de votre centre de santé? [00:15:31-6](http://localhost:2300/file=C:/Users/USER/Desktop/ENTRETIENS%20PREMATURITE/CSPS_FARAKAN/RESPONSABLE%20MATERNITE%20FARAKAN.MP3time=931600)

[00:15:31-6](http://localhost:2300/file=C:/Users/USER/Desktop/ENTRETIENS%20PREMATURITE/CSPS_FARAKAN/RESPONSABLE%20MATERNITE%20FARAKAN.MP3time=931600) Person 1: Je ne sais pas comment il faut expliquer (rire) [00:15:35-3](http://localhost:2300/file=C:/Users/USER/Desktop/ENTRETIENS%20PREMATURITE/CSPS_FARAKAN/RESPONSABLE%20MATERNITE%20FARAKAN.MP3time=935300)

[00:15:35-3](http://localhost:2300/file=C:/Users/USER/Desktop/ENTRETIENS%20PREMATURITE/CSPS_FARAKAN/RESPONSABLE%20MATERNITE%20FARAKAN.MP3time=935300) Interviewer: Est ce que par rapport à la naissance prématurée,est ce que l'accouchement relève d'un defi pour vous? [00:15:44-7](http://localhost:2300/file=C:/Users/USER/Desktop/ENTRETIENS%20PREMATURITE/CSPS_FARAKAN/RESPONSABLE%20MATERNITE%20FARAKAN.MP3time=944700)

[00:15:44-7](http://localhost:2300/file=C:/Users/USER/Desktop/ENTRETIENS%20PREMATURITE/CSPS_FARAKAN/RESPONSABLE%20MATERNITE%20FARAKAN.MP3time=944700) Person 1: En tout cas personne ne souhaite avoir un accouchement prématuré parce qu'on sait qu'on sait que la prématurité n'est pas simple pour les parents ce n'est pas simple. Un bébé prématuré c'est difficile surtout les grands prematurés. Souvent si le poids varie entre 2kg200 ou 2kg100 cela peut aller, mais quand c'est autour de 1kg800, 1kg500, la prise en charge est difficile. Maintenant s'il est possible de prevenir je pense qu'il faut prevenir pour ne pas avoir ce cas de naissance prematurée. Souvent il y a les maladies qui peuvent provoquer la naissance prématurée, comme le paludisme. [00:16:33-6](http://localhost:2300/file=C:/Users/USER/Desktop/ENTRETIENS%20PREMATURITE/CSPS_FARAKAN/RESPONSABLE%20MATERNITE%20FARAKAN.MP3time=993600)

00:13:54-1 Interviewer: D'accord, maintenant la question suivante. Est-ce que la naissance est un défi de santé publique important auquel vous êtes confronté dans votre centre de santé ? 00:14:05-9

00:14:05-9 Person 1: En tout cas, c'est un défi important. Je sais qu'entre temps il y avait un projet qui était venu pour s'occuper des bébés de faible poids de naissance. 00:14:24-6

00:14:24-6 Interviewer: Généralement, à quel type de défi vous faites face par rapport aux naissances prématurées ? 00:14:34-3

00:14:34-3 Person 1: C'est de prévenir. Prévenir l'accouchement et tout ce qui va avec, cela va passer par l'interrogatoire, ça va passer par surtout l'interrogatoire. On sait que souvent les approches, les naissances prématurées, les femmes, souvent avec les infections, en tout cas la plupart de nos jours-là, ce sont surtout les infections et puis aussi les accouchements gémellaires. 00:15:06-4

00:15:06-4 Interviewer: Lorsque vous parlez d'infections, vous faites allusion à quel type d'infections ? 00:15:09-2

00:15:09-2 Person 1: Surtout les infections urinaires. 00:15:17-1

00:15:17-1 Interviewer: Est-ce que les accouchements prématurés représentent un défi pour vous au sein de votre centre de santé ? 00:15:31-6

00:15:31-6 Person 1: Je ne sais pas comment il faut expliquer (rire) 00:15:35-3

00:15:35-3 Interviewer: Est-ce que par rapport à la naissance prématurée, est-ce que l'accouchement relève d'un défi pour vous ? 00:15:44-7

00:15:44-7 Person 1: En tout cas, personne ne souhaite avoir un accouchement prématuré parce qu'on sait que la prématurité n'est pas simple pour les parents, ce n'est pas simple. Un bébé prématuré c'est difficile surtout les grands prématurés. Souvent si le poids varie entre 2kg200 ou 2kg100 cela peut aller, mais quand c'est autour de 1kg800, 1kg500, la prise en charge est difficile. Maintenant s'il est possible de prévenir, je pense qu'il faut prévenir pour ne pas avoir ce cas de naissance prématurée. Souvent il y a les maladies qui peuvent provoquer la naissance prématurée, comme le paludisme. 00:16:33-6

**Interviewer (00:14:05-9):** Is childbirth a significant public health challenge in your health center?

**Person 1 (00:14:05-9):** Yes, childbirth is an important challenge. There was a project focused on caring for babies with low birth weight, which indicates the significance of this issue.

**Interviewer (00:14:24-6):** What specific challenges do you face with premature births?

**Person 1 (00:14:34-3):** The primary challenge is prevention. To prevent premature births, interrogation, or thorough questioning, is vital. Many premature births are associated with infections and twin deliveries. Recently, infections have been the major cause.

**Interviewer (00:15:06-4):** What types of infections are commonly involved?

**Person 1 (00:15:09-2):** Urinary infections are particularly common.

**Interviewer (00:15:17-1):** Do premature births present a challenge at your health center?

**Person 1 (00:15:31-6):** It's challenging to explain. Nobody wants a premature birth because it's tough for the parents and managing a premature baby, especially extremely premature ones, is difficult. Babies weighing around 2.1kg to 2.2kg can be managed more easily, but those around 1.5kg to 1.8kg are more challenging. Prevention is key to avoid such situations. Diseases like malaria can also lead to premature births.

# 7. Staff skills and existence of equipment for better care

### Focus group of Accart-ville urban medical center

[00:16:23-6](http://localhost:2300/file=C:/Users/USER/Desktop/ENTRETIENS%20PREMATURITE/CSPS-ACCART-VILLE/FOCUS%20GROUPE%20ACCART-VILLE-BON.mp3time=983600) Interviewer: d'accord, nous allons poursuivre toujours.Est-ce que vous avez le plateau technique nécessaire, c'est-à-dire le personnel formé, les médicaments et les équipements pour prendre en charge les cas de naissance prématurée ? avez vous le plateau technique necessaire [00:16:52-2](http://localhost:2300/file=C:/Users/USER/Desktop/ENTRETIENS%20PREMATURITE/CSPS-ACCART-VILLE/FOCUS%20GROUPE%20ACCART-VILLE-BON.mp3time=1012200)

[00:16:52-2](http://localhost:2300/file=C:/Users/USER/Desktop/ENTRETIENS) Person 1: bon içi même c'est lorsque les femmes arrivent à terme que nous les laissons accoucher donc dès que nous nous rendons compte c'est un accouchement prématuré qui est en cours,c'est mieux de les envoyer au CMA pour pouvoir les prendre en charge parce que nous n'avons pas le materiel içi. [00:17:19-8](http://localhost:2300/file=C:/Users/USER/Desktop/ENTRETIENS)

[00:17:19-8](http://localhost:2300/file=C:/Users/USER/Desktop/ENTRETIENS%20PREMATURITE/CSPS-ACCART-VILLE/FOCUS%20GROUPE%20ACCART-VILLE-BON.mp3time=1039800) Person 3: ce que je pourrai ajouter c'est par rapport au type de prématurité, lorsqu'il s'agit d'une grande prematurité et que la femme se presente avant l'accouchement c'est à dire avant le travail, on l'evacue au CMA pour qu'on puisse faire le transfert utero. lorsqu'il s'agit d'une prematurité moyenne nous essayons de gerer içi et l'envoyer après dans une unité de néonatologie.par rapport aux produits (medicaments), nous ne disposons de tout même ce matin nous en avons discuté au staff,nous manquons de vitamine K1, nous manquons aussi de éosine acqueuse pour les soins du cordon, nous manquons egalement de couveuses et même de salles parce que nous devons les separer des adultes, les prematurés doivent être separés des adultes nous manquons de salles pour ses prises en charge. donc le materiel nous n'en disponsons pas et les differents moyens également de façon génerale. c'est ce qu'on retenir. [00:18:29-7](http://localhost:2300/file=C:/Users/USER/Desktop/ENTRETIENS%20PREMATURITE/CSPS-ACCART-VILLE/FOCUS%20GROUPE%20ACCART-VILLE-BON.mp3time=1109700)

[00:18:29-7](http://localhost:2300/file=C:/Users/USER/Desktop/ENTRETIENS%20PREMATURITE/CSPS-ACCART-VILLE/FOCUS%20GROUPE%20ACCART-VILLE-BON.mp3time=1109700)Interviewer: quand vous parlez de grand prematurité vous faites allusion à quel type de prematurité? [00:18:33-1](http://localhost:2300/file=C:/Users/USER/Desktop/ENTRETIENS%20PREMATURITE/CSPS-ACCART-VILLE/FOCUS%20GROUPE%20ACCART-VILLE-BON.mp3time=1113100)

[00:18:33-1](http://localhost:2300/file=C:/Users/USER/Desktop/ENTRETIENS) Person 2: la grande prematurité ce sont les naissances qui surviennent entre la 28ème semaine et la 32ème semaine, la prematurité moyenne ce sont les naissances qui surviennent entre la 33ème semaine et la 36ème semaine 06 jours. [00:19:01-8](http://localhost:2300/file=C:/Users/USER/Desktop/ENTRETIENS%20PREMATURITE/CSPS-ACCART-VILLE/FOCUS%20GROUPE%20ACCART-VILLE-BON.mp3time=1141800)

00:16:23-6 Interviewer: D'accord, nous allons poursuivre toujours. Est-ce que vous avez le plateau technique nécessaire, c'est-à-dire le personnel formé, les médicaments et les équipements pour prendre en charge les cas de naissance prématurée ? Avez-vous le plateau technique nécessaire ? 00:16:52-2

00:16:52-2 Person 1: Bon, ici même c'est lorsque les femmes arrivent à terme que nous les laissons accoucher donc dès que nous nous rendons compte que c'est un accouchement prématuré qui est en cours, c'est mieux de les envoyer au CMA pour pouvoir les prendre en charge parce que nous n'avons pas le matériel ici. 00:17:19-8

00:17:19-8 Person 3: Ce que je pourrais ajouter, c'est par rapport au type de prématurité. Lorsqu'il s'agit d'une grande prématurité et que la femme se présente avant l'accouchement, c'est-à-dire avant le travail, on l'évacue au CMA pour qu'on puisse faire le transfert utéro. Lorsqu'il s'agit d'une prématurité moyenne, nous essayons de gérer ici et de l'envoyer après dans une unité de néonatologie. Par rapport aux produits (médicaments), nous ne disposons de tout. Même ce matin, nous en avons discuté au staff, nous manquons de vitamine K1, nous manquons aussi d'éosine aqueuse pour les soins du cordon, nous manquons également de couveuses et même de salles parce que nous devons les séparer des adultes. Les prématurés doivent être séparés des adultes. Nous manquons de salles pour ces prises en charge. Donc, le matériel, nous n'en disposons pas et les différents moyens également de façon générale. C'est ce qu'on peut retenir. 00:18:29-7

00:18:29-7 Interviewer: Quand vous parlez de grande prématurité, vous faites allusion à quel type de prématurité ? 00:18:33-1

00:18:33-1 Person 2: La grande prématurité ce sont les naissances qui surviennent entre la 28ème semaine et la 32ème semaine, la prématurité moyenne ce sont les naissances qui surviennent entre la 33ème semaine et la 36ème semaine 06 jours. 00:19:01-8

**Interviewer (00:16:23-6):** Do you have the necessary technical support, including trained personnel, medications, and equipment, to manage cases of premature birth?

**Person 1 (00:16:52-2):** At our facility, we usually manage full-term births. When a premature birth is imminent, we prefer transferring the patient to the CMA (Central Medical Association) due to our lack of equipment for such cases.

**Person 3 (00:17:19-8):** Regarding different types of prematurity, we evacuate cases of extreme prematurity to the CMA before labor begins for better care. For average prematurity, we try to manage it here before transferring to a neonatal unit. We face shortages in medications, such as vitamin K1 and aqueous eosin for cord care. Our facility also lacks incubators and separate rooms for premature babies, which is essential as they should be isolated from adults. Overall, we are lacking in both equipment and resources.

**Interviewer (00:18:29-7):** Can you define extreme prematurity?

**Person 2 (00:18:33-1):** Extreme prematurity refers to births occurring between the 28th and 32nd weeks of pregnancy, while medium prematurity is classified as births occurring between the 33rd and 36th weeks and 6 days.

### Manager of Accart-ville urban medical center

[00:14:08-5](http://localhost:2300/file=C:/Users/USER/Desktop/ENTRETIENS%20PREMATURITE/CSPS-ACCART-VILLE/RESPONSABLE%20CMU%20ACCART-VILLE.MP3time=848500)Interviewer: Maintenant? Est ce que vous avez. Le plateau technique nécessaire, le personnel formé, les médicaments, les équipements pour prendre en charge le cas des prématurés? [00:14:20-9](http://localhost:2300/file=C:/Users/USER/Desktop/ENTRETIENS%20PREMATURITE/CSPS-ACCART-VILLE/RESPONSABLE%20CMU%20ACCART-VILLE.MP3time=860900)

[00:14:20-9](http://localhost:2300/file=C:/Users/USER/Desktop/ENTRETIENS%20PREMATURITE/CSPS-ACCART-VILLE/RESPONSABLE%20CMU%20ACCART-VILLE.MP3time=860900) Person 1: Non, on en a pas. [00:14:24-6](http://localhost:2300/file=C:/Users/USER/Desktop/ENTRETIENS%20PREMATURITE/CSPS-ACCART-VILLE/RESPONSABLE%20CMU%20ACCART-VILLE.MP3time=864600)

[00:14:24-6](http://localhost:2300/file=C:/Users/USER/Desktop/ENTRETIENS%20PREMATURITE/CSPS-ACCART-VILLE/RESPONSABLE%20CMU%20ACCART-VILLE.MP3time=864600)Interviewer: Le cas échéant comment est ce que cela se fait? [00:14:28-3](http://localhost:2300/file=C:/Users/USER/Desktop/ENTRETIENS%20PREMATURITE/CSPS-ACCART-VILLE/RESPONSABLE%20CMU%20ACCART-VILLE.MP3time=868300)

[00:14:28-3](http://localhost:2300/file=C:/Users/USER/Desktop/ENTRETIENS%20PREMATURITE/CSPS-ACCART-VILLE/RESPONSABLE%20CMU%20ACCART-VILLE.MP3time=868300) Person 1: C'est ce que j'ai di c'est la référence en fait, même le matériel de première réanimation en fait, on n'en a pas. Voilà, on est très, très, très limité. Ça veut dire, et c'est ce que j'essayais d'expliquer, c'est à dire le changement n'a pas suivi, en fait, quoi? On a été érigé en centre médical, mais rien n'a été suivi. On essaie de faire avec. [00:14:56-0](http://localhost:2300/file=C:/Users/USER/Desktop/ENTRETIENS%20PREMATURITE/CSPS-ACCART-VILLE/RESPONSABLE%20CMU%20ACCART-VILLE.MP3time=896000)

00:14:08-5 Interviewer: Maintenant, est-ce que vous avez le plateau technique nécessaire, le personnel formé, les médicaments, les équipements pour prendre en charge le cas des prématurés ? 00:14:20-9

00:14:20-9 Person 1: Non, on n'en a pas. 00:14:24-6

00:14:24-6 Interviewer: Le cas échéant, comment est-ce que cela se fait ? 00:14:28-3

00:14:28-3 Person 1: C'est ce que j'ai dit, c'est la référence en fait, même le matériel de première réanimation en fait, on n'en a pas. Voilà, on est très, très, très limité. Ça veut dire, et c'est ce que j'essayais d'expliquer, c'est-à-dire le changement n'a pas suivi, en fait, quoi ? On a été érigé en centre médical, mais rien n'a été suivi. On essaie de faire avec. 00:14:56-0

**Interviewer (00:14:08-5):** Do you have the necessary technical support, trained personnel, medications, and equipment to care for premature babies?

**Person 1 (00:14:20-9):** No, we don't have any of those resources.

**Interviewer (00:14:24-6):** How do you manage in such situations?

**Person 1 (00:14:28-3):** As I mentioned earlier, we lack even the basic resuscitation equipment. We're extremely limited in our capabilities. Although we've been designated as a medical center, the necessary upgrades and resources haven't followed. We're doing the best we can with what we have.

### Maternity manager of Accart-ville urban medical center

[00:17:22-9](http://localhost:2300/file=C:/Users/USER/Desktop/ENTRETIENS%20PREMATURITE/CSPS-ACCART-VILLE/RESPONSABLE%20MATERNITE%20CMU%20ACCART-VILLE.MP3time=1042900) Interviewer: est ce qu'au sein de votre centre de santé vous avez le plateau technique necessaire? c'est à dire le personnel formé, les medicaments, les equipements pour prendre en charge les cas de naissances prematurés?

[00:17:33-8](http://localhost:2300/file=C:/Users/USER/Desktop/ENTRETIENS%20PREMATURITE/CSPS-ACCART-VILLE/RESPONSABLE%20MATERNITE%20CMU%20ACCART-VILLE.MP3time=1053800) Person 1: le personnel formé, je peux dire oui puisque nous avons des sages femmes qui sont au nombre de huit (08) , nous avons des accoucheuses auxiliares qui sont au nombre de cinq (05) nous avons un ASSOG(attaché de santé en soin obstetricaux et gynecologiques), nous avons deux medecins. maintenant si nous avons des difficultés nous appelons les medecins qui vont nous aider.les sages femmes aussi de part leurs formations sont habilitées à prendre en charge les nouveaux nées. mais comme les conditions ne sont pas reunis ,nous ne disposons pas de salles specifiques donc la prise en charge est très limité et aussi si la pratique est quotidienne cela peut aamener les praticiens à avoir la main dans les prises en charges des naissances prématurés. il faut ajouter que les experiences particulières des unes et des autres dans la prise en charge des naissances prématurés est à noter. la qualité des prises en charge depend de l'experience des sages femmes.

[00:18:23-9](http://localhost:2300/file=C:/Users/USER/Desktop/ENTRETIENS%20PREMATURITE/CSPS-ACCART-VILLE/RESPONSABLE%20MATERNITE%20CMU%20ACCART-VILLE.MP3time=1103900) Interviewer: d'accord

[00:18:25-8](http://localhost:2300/file=C:/Users/USER/Desktop/ENTRETIENS%20PREMATURITE/CSPS-ACCART-VILLE/RESPONSABLE%20MATERNITE%20CMU%20ACCART-VILLE.MP3time=1105800) Person 1: d'autres ont fait des centres de santé comme les hopitaux , ou il y a le materiel ce qui fait que ces dernières ont des notions dans la prise en charge des nouveaux nées prématurés, d'autres n'n ont pas fait elles ont evolué dans les CSPS, donc ce qui fait qu'au niveau peripherique beaucoup n'ont pas de notions sur les prises en charges.

[00:18:43-4](http://localhost:2300/file=C:/Users/USER/Desktop/ENTRETIENS%20PREMATURITE/CSPS-ACCART-VILLE/RESPONSABLE%20MATERNITE%20CMU%20ACCART-VILLE.MP3time=1123400) Interviewer: maintenant, est ce que votre centre peut prendre en charge les grands prématurés?

[00:18:48-6](http://localhost:2300/file=C:/Users/USER/Desktop/ENTRETIENS%20PREMATURITE/CSPS-ACCART-VILLE/RESPONSABLE%20MATERNITE%20CMU%20ACCART-VILLE.MP3time=1128600) Person 1: non, tout ce qui est prématuré on evacue

00:17:22-9 Interviewer: Est-ce qu'au sein de votre centre de santé vous avez le plateau technique nécessaire ? C'est-à-dire le personnel formé, les médicaments, les équipements pour prendre en charge les cas de naissances prématurés ?

00:17:33-8 Person 1: Le personnel formé, je peux dire oui, puisque nous avons des sage-femmes qui sont au nombre de huit (08), nous avons des accoucheuses auxiliaires qui sont au nombre de cinq (05), nous avons un ASSOG (attaché de santé en soin obstétrical et gynécologique), nous avons deux médecins. Maintenant, si nous avons des difficultés, nous appelons les médecins qui vont nous aider. Les sage-femmes aussi, de par leurs formations, sont habilitées à prendre en charge les nouveau-nés. Mais comme les conditions ne sont pas réunies, nous ne disposons pas de salles spécifiques, donc la prise en charge est très limitée. Et aussi, si la pratique est quotidienne, cela peut amener les praticiens à avoir la main dans les prises en charge des naissances prématurées. Il faut ajouter que les expériences particulières des unes et des autres dans la prise en charge des naissances prématurées sont à noter. La qualité des prises en charge dépend de l'expérience des sage-femmes.

00:18:23-9 Interviewer: D'accord.

00:18:25-8 Person 1: D'autres ont fait des centres de santé comme les hôpitaux, où il y a le matériel, ce qui fait que ces dernières ont des notions dans la prise en charge des nouveau-nés prématurés, d'autres n'en ont pas fait, elles ont évolué dans les CSPS, donc ce qui fait qu'au niveau périphérique, beaucoup n'ont pas de notions sur les prises en charges.

00:18:43-4 Interviewer: Maintenant, est-ce que votre centre peut prendre en charge les grands prématurés ?

00:18:48-6 Person 1: Non, tout ce qui est prématuré, on évacue.

**Interviewer (00:17:22-9):** Do you have the necessary technical support in your health center? Specifically, trained staff, medications, equipment for managing premature births?

**Person 1 (00:17:33-8):** We do have trained staff, including eight midwives, five auxiliary birth attendants, an obstetric and gynecological health officer, and two doctors. When difficulties arise, we can call on the doctors for assistance. Our midwives are also trained in newborn care. However, our support capabilities are limited due to a lack of specific facilities and rooms for premature infants. Daily practice in managing premature births can enhance the practitioners' skills, and individual experiences in handling such cases contribute to the quality of care. The level of expertise varies; some have worked in well-equipped hospitals and are knowledgeable in premature newborn care, while others, mainly those who have worked at the peripheral level, may lack this experience.

Person 1 (00:18:25-8): Others have established health centers like hospitals, where there is equipment, which means that they have knowledge of the care of premature newborns, others have not done so, they have evolved in the CSPS, which means that at the peripheral level many have no idea about support.

**Interviewer (00:18:43-4):** Can your center take care of very premature babies?

**Person 1 (00:18:48-6):** No, we typically evacuate cases of prematurity.

### Focus group of Do medical center with surgical branch

[00:18:09-7](http://localhost:2300/file=C:/Users/USER/Desktop/ENTRETIENS%20PREMATURITE/CMA-DO/FOCUS%20GROUPE%20CMA-DO.mp3time=1089700) Interviewer: Est ce que vous avez le plateau technique necessaire ici pour pouvoir prendre en charge les naissances prématurées? [00:18:15-1](http://localhost:2300/file=C:/Users/USER/Desktop/ENTRETIENS%20PREMATURITE/CMA-DO/FOCUS%20GROUPE%20CMA-DO.mp3time=1095100)

[00:18:15-1](http://localhost:2300/file=C:/Users/USER/Desktop/ENTRETIENS%20PREMATURITE/CMA-DO/FOCUS%20GROUPE%20CMA-DO.mp3time=1095100) Person 6: Nous n'en avons pas. on a même pas de plateau technique, on jongle seulement sinon, on n'a pas de plateau technique pour prendre en charge les naissances prématurées. [00:18:25-5](http://localhost:2300/file=C:/Users/USER/Desktop/ENTRETIENS%20PREMATURITE/CMA-DO/FOCUS%20GROUPE%20CMA-DO.mp3time=1105500)

[00:18:25-5](http://localhost:2300/file=C:/Users/USER/Desktop/ENTRETIENS%20PREMATURITE/CMA-DO/FOCUS%20GROUPE%20CMA-DO.mp3time=1105500) Person 9: Si se sont les prématuritées saintes , il ya les soins mères kangourou, mais quand c'est pathologique, là on est obligé d'évacuer à l'hôpital [00:18:41-0](http://localhost:2300/file=C:/Users/USER/Desktop/ENTRETIENS%20PREMATURITE/CMA-DO/FOCUS%20GROUPE%20CMA-DO.mp3time=1121000)

[00:18:41-0](http://localhost:2300/file=C:/Users/USER/Desktop/ENTRETIENS%20PREMATURITE/CMA-DO/FOCUS%20GROUPE%20CMA-DO.mp3time=1121000) Interviewer: Est ce que vous avez les medicaments, le personnel qualifié, le cas écheant? [00:18:50-4](http://localhost:2300/file=C:/Users/USER/Desktop/ENTRETIENS%20PREMATURITE/CMA-DO/FOCUS%20GROUPE%20CMA-DO.mp3time=1130400)

[00:18:50-4](http://localhost:2300/file=C:/Users/USER/Desktop/ENTRETIENS%20PREMATURITE/CMA-DO/FOCUS%20GROUPE%20CMA-DO.mp3time=1130400) Person 5: Oui l'unité devrait faire qu' il y est le personnel qualifié au moins la pediatre est là , il y a aussi les sages femmes [00:19:07-5](http://localhost:2300/file=C:/Users/USER/Desktop/ENTRETIENS%20PREMATURITE/CMA-DO/FOCUS%20GROUPE%20CMA-DO.mp3time=1147500)

[00:19:07-5](http://localhost:2300/file=C:/Users/USER/Desktop/ENTRETIENS%20PREMATURITE/CMA-DO/FOCUS%20GROUPE%20CMA-DO.mp3time=1147500) Interviewer: Lorsque vous constatez que vous ne pouvez pas prendre en charge les cas de naissances prématurées, comment est ce que vous procedez? [00:19:15-0](http://localhost:2300/file=C:/Users/USER/Desktop/ENTRETIENS%20PREMATURITE/CMA-DO/FOCUS%20GROUPE%20CMA-DO.mp3time=1155000)

[00:19:15-0](http://localhost:2300/file=C:/Users/USER/Desktop/ENTRETIENS%20PREMATURITE/CMA-DO/FOCUS%20GROUPE%20CMA-DO.mp3time=1155000) Person 7: On évacue au CHURSS en néonatologie [00:19:23-3](http://localhost:2300/file=C:/Users/USER/Desktop/ENTRETIENS%20PREMATURITE/CMA-DO/FOCUS%20GROUPE%20CMA-DO.mp3time=1163300)

00:18:09-7 Interviewer: Est-ce que vous avez le plateau technique nécessaire ici pour pouvoir prendre en charge les naissances prématurées ? 00:18:15-1

00:18:15-1 Person 6: Nous n'en avons pas. On n’a même pas de plateau technique, on jongle seulement sinon, on n'a pas de plateau technique pour prendre en charge les naissances prématurées. 00:18:25-5

00:18:25-5 Person 9: Si ce sont les prématurités saines, il y a les soins mères kangourou, mais quand c'est pathologique, là on est obligé d'évacuer à l'hôpital. 00:18:41-0

00:18:41-0 Interviewer: Est-ce que vous avez les médicaments, le personnel qualifié, le cas échéant ? 00:18:50-4

00:18:50-4 Person 5: Oui, l'unité devrait faire qu'il y ait le personnel qualifié, au moins la pédiatre est là, il y a aussi les sage-femmes. 00:19:07-5

00:19:07-5 Interviewer: Lorsque vous constatez que vous ne pouvez pas prendre en charge les cas de naissances prématurées, comment est-ce que vous procédez ? 00:19:15-0

00:19:15-0 Person 7: On évacue au CHURSS en néonatologie. 00:19:23-3

**Interviewer (00:18:15-1):** Do you have the necessary technical support for managing premature births?

**Person 6 (00:18:25-5):** Unfortunately, we lack a technical platform for managing premature births. We make do with what we have.

**Person 9 (00:18:41-0):** For healthy prematurity cases, we provide kangaroo mother care. However, pathological cases require hospitalization.

**Interviewer (00:18:50-4):** Do you have the required medicines and qualified personnel?

**Person 5 (00:19:07-5):** Our unit ensures the availability of qualified personnel, including pediatricians and midwives.

**Interviewer (00:19:15-0):** How do you handle cases that are beyond your capability?

**Person 7 (00:19:23-3):** In such scenarios, we evacuate the cases to CHURSS's neonatology department for specialized care.

### Gynecologist 1

[00:10:28-2](http://localhost:2300/file=C:/Users/USER/Desktop/ENTRETIENS%20PREMATURITE/CMA-DO/GENYCOLOGUE%2001.mp3time=628200) Interviewer: Est-ce qu'au sein de votre centre de santé, vous avez le plateau technique nécessaire? [00:10:32-7](http://localhost:2300/file=C:/Users/USER/Desktop/ENTRETIENS%20PREMATURITE/CMA-DO/GENYCOLOGUE%2001.mp3time=632700)

[00:10:32-7](http://localhost:2300/file=C:/Users/USER/Desktop/ENTRETIENS%20PREMATURITE/CMA-DO/GENYCOLOGUE%2001.mp3time=632700) Person 1: Pour la prise en charge du prématuré, non. C'est ça qui pose problème. Parce qu'on a une unité ici, une unité de soins mère kangourou, qui se débrouille comme elle peut. Mais là, on ne peut prendre que des prématurés sains.

Dès qu'il y a une pathologie, on ne peut plus, il faut les envoyer à l'hôpital. [00:10:58-1](http://localhost:2300/file=C:/Users/USER/Desktop/ENTRETIENS%20PREMATURITE/CMA-DO/GENYCOLOGUE%2001.mp3time=658100)

[00:10:58-1](http://localhost:2300/file=C:/Users/USER/Desktop/ENTRETIENS%20PREMATURITE/CMA-DO/GENYCOLOGUE%2001.mp3time=658100) Interviewer: Qu'est-ce que vous appelez prématurés sains ? [00:10:59-9](http://localhost:2300/file=C:/Users/USER/Desktop/ENTRETIENS%20PREMATURITE/CMA-DO/GENYCOLOGUE%2001.mp3time=659900)

[00:10:59-9](http://localhost:2300/file=C:/Users/USER/Desktop/ENTRETIENS%20PREMATURITE/CMA-DO/GENYCOLOGUE%2001.mp3time=659900) Person 1: Un prématuré, juste un problème de poids de naissance. Il n'a pas encore atteint les 2,5 kg. Mais il a bien crié, il a bien réagi, il est bien rose, il n'a pas d'infection,il n'a pas de détresse respiratoire. Là, on peut le prendre en charge. [00:11:18-1](http://localhost:2300/file=C:/Users/USER/Desktop/ENTRETIENS%20PREMATURITE/CMA-DO/GENYCOLOGUE%2001.mp3time=678100)

[00:11:18-1](http://localhost:2300/file=C:/Users/USER/Desktop/ENTRETIENS%20PREMATURITE/CMA-DO/GENYCOLOGUE%2001.mp3time=678100) Interviewer: Est-ce que vous avez aussi souvent des grands prématurés ? [00:11:21-1](http://localhost:2300/file=C:/Users/USER/Desktop/ENTRETIENS%20PREMATURITE/CMA-DO/GENYCOLOGUE%2001.mp3time=681100)

[00:11:21-1](http://localhost:2300/file=C:/Users/USER/Desktop/ENTRETIENS%20PREMATURITE/CMA-DO/GENYCOLOGUE%2001.mp3time=681100) Person 1: Oui, il nous arrive d'avoir des grands prématurés. [00:11:24-4](http://localhost:2300/file=C:/Users/USER/Desktop/ENTRETIENS%20PREMATURITE/CMA-DO/GENYCOLOGUE%2001.mp3time=684400)

[00:11:24-4](http://localhost:2300/file=C:/Users/USER/Desktop/ENTRETIENS%20PREMATURITE/CMA-DO/GENYCOLOGUE%2001.mp3time=684400) Interviewer: Et le cas écheant, comment est-ce que cela est gerer ? [00:11:27-5](http://localhost:2300/file=C:/Users/USER/Desktop/ENTRETIENS%20PREMATURITE/CMA-DO/GENYCOLOGUE%2001.mp3time=687500)

[00:11:27-5](http://localhost:2300/file=C:/Users/USER/Desktop/ENTRETIENS%20PREMATURITE/CMA-DO/GENYCOLOGUE%2001.mp3time=687500) Person 1: Comme je vous l'ai dit, souvent les enfants de 1,2 kg, 1,3 kg,avec l'unité soins mères kangourou, s'il n'y a pas de problème associé, on arrive à les prendre en charge ici. [00:11:45-3](http://localhost:2300/file=C:/Users/USER/Desktop/ENTRETIENS%20PREMATURITE/CMA-DO/GENYCOLOGUE%2001.mp3time=705300)

[00:11:45-3](http://localhost:2300/file=C:/Users/USER/Desktop/ENTRETIENS%20PREMATURITE/CMA-DO/GENYCOLOGUE%2001.mp3time=705300) Interviewer: Au cas où vous constatez que vous n'arrivez pas à les prendre en charge,quelles sont les dispositions qui sont prises ? [00:11:51-6](http://localhost:2300/file=C:/Users/USER/Desktop/ENTRETIENS%20PREMATURITE/CMA-DO/GENYCOLOGUE%2001.mp3time=711600)

[00:11:51-6](http://localhost:2300/file=C:/Users/USER/Desktop/ENTRETIENS%20PREMATURITE/CMA-DO/GENYCOLOGUE%2001.mp3time=711600) Person 1: On les évacue à l'hôpital, au CHU, en unité de néonatologie. [00:12:01-9](http://localhost:2300/file=C:/Users/USER/Desktop/ENTRETIENS%20PREMATURITE/CMA-DO/GENYCOLOGUE%2001.mp3time=721900)

00:10:28-2 Interviewer: Est-ce qu'au sein de votre centre de santé, vous avez le plateau technique nécessaire ? 00:10:32-7

00:10:32-7 Person 1: Pour la prise en charge du prématuré, non. C'est ça qui pose problème. Parce qu'on a une unité ici, une unité de soins mère kangourou, qui se débrouille comme elle peut. Mais là, on ne peut prendre que des prématurés sains.

Dès qu'il y a une pathologie, on ne peut plus, il faut les envoyer à l'hôpital. 00:10:58-1

00:10:58-1 Interviewer: Qu'est-ce que vous appelez prématurés sains ? 00:10:59-9

00:10:59-9 Person 1: Un prématuré, juste un problème de poids de naissance. Il n'a pas encore atteint les 2,5 kg. Mais il a bien crié, il a bien réagi, il est bien rose, il n'a pas d'infection, il n'a pas de détresse respiratoire. Là, on peut le prendre en charge. 00:11:18-1

00:11:18-1 Interviewer: Est-ce que vous avez aussi souvent des grands prématurés ? 00:11:21-1

00:11:21-1 Person 1: Oui, il nous arrive d'avoir des grands prématurés. 00:11:24-4

00:11:24-4 Interviewer: Et le cas échéant, comment est-ce que cela est géré ? 00:11:27-5

00:11:27-5 Person 1: Comme je vous l'ai dit, souvent les enfants de 1,2 kg, 1,3 kg, avec l'unité soins mères kangourou, s'il n'y a pas de problème associé, on arrive à les prendre en charge ici. 00:11:45-3

00:11:45-3 Interviewer: Au cas où vous constatez que vous n'arrivez pas à les prendre en charge, quelles sont les dispositions qui sont prises ? 00:11:51-6

00:11:51-6 Person 1: On les évacue à l'hôpital, au CHU, en unité de néonatologie.

**Interviewer (00:10:28-2):** Do you have the necessary technical support in your health center for caring for premature babies?

**Person 1 (00:10:32-7):** For premature babies, unfortunately, no. We have a kangaroo mother care unit that does its best, but it's limited to healthy premature babies. Any baby with additional pathologies must be sent to the hospital.

**Interviewer (00:10:59-9):** What defines a healthy premature baby?

**Person 1 (00:11:18-1):** A healthy premature baby typically only has a birth weight issue, not yet reaching 2.5 kg. However, if they cry well, show good reactions, appear pink without infections or respiratory distress, we can care for them here.

**Interviewer (00:11:21-1):** Do you also encounter very premature babies?

**Person 1 (00:11:24-4):** Yes, we do sometimes have very premature babies.

**Interviewer (00:11:27-5):** How are these cases managed?

**Person 1 (00:11:45-3):** If the very premature babies, like those weighing around 1.2 or 1.3 kg, don't have associated problems, we can manage them here with the kangaroo mother care unit.

**Interviewer (00:11:51-6):** What happens if you find you cannot take care of them?

**Person 1:** We evacuate such cases to the hospital, specifically to the neonatology unit at the university hospital.

### Gynecologist 2

**No response elements**

### Chief physician of Do health district

[00:20:14-2](http://localhost:2300/file=C:/Users/USER/Desktop/ENTRETIENS%20PREMATURITE/CMA-DO/MCD-DO.mp3time=1214200) Interviewer: D'accord. Est-ce que vous avez les plateaux techniques nécessaires? [00:20:17-7](http://localhost:2300/file=C:/Users/USER/Desktop/ENTRETIENS%20PREMATURITE/CMA-DO/MCD-DO.mp3time=1217700)

[00:20:17-7](http://localhost:2300/file=C:/Users/USER/Desktop/ENTRETIENS%20PREMATURITE/CMA-DO/MCD-DO.mp3time=1217700) Person 1: Ça se retrouvait dans ce que je disais. Moi, j'ai vu dans les plateaux techniques, c'est ce que je disais. Les plateaux techniques, ça va être l'agent de santé suffisamment compétent pour ça. Les équipements médicaux qui vont répondre à ça. Dans l'équipement médical, on n'a pas de couveuses. Donc, d'abord, ça va commencer par... On n'a pas une unité même. Il n'y a pas un service adapté pour ça ici au CMA. On n'a pas un service de néonatologie. Donc, le service dont je parlais, SMK, Soins Maternels bébés kangourous, là.

Au fait, ça nous permet, c'est ceux qui sont vraiment... Ce ne sont pas les très grands prématurés, là. C'est ceux qui ne sont pas loin de la date de l'accouchement normale. Et qu'on récupère avec les faibles poids de naissance. Ça nous permet de les gérer. Mais à une certaine phase, là, on ne peut pas.On ne peut pas gérer ça. Ils sont obligés donc d'envoyer. Donc, je veux dire qu'on n'a pas le dispositif nécessaire pour gérer les grands prématurés. Il faut les envoyer à l'hôpital. [00:21:21-1](http://localhost:2300/file=C:/Users/USER/Desktop/ENTRETIENS%20PREMATURITE/CMA-DO/MCD-DO.mp3time=1281100)

[00:21:21-1](http://localhost:2300/file=C:/Users/USER/Desktop/ENTRETIENS%20PREMATURITE/CMA-DO/MCD-DO.mp3time=1281100)Interviewer: Quand vous parlez des grands prématurés, vous faites allusion à quel type de prématurés? [00:21:25-4](http://localhost:2300/file=C:/Users/USER/Desktop/ENTRETIENS%20PREMATURITE/CMA-DO/MCD-DO.mp3time=1285400)

[00:21:25-4](http://localhost:2300/file=C:/Users/USER/Desktop/ENTRETIENS%20PREMATURITE/CMA-DO/MCD-DO.mp3time=1285400) Person 1: Je parle des prématurés qui, puisqu'à partir d'un certain nombre de poids, nous, nous pouvons les gérer. Et quand on ne peut pas les gérer, il faut envoyer à l'hôpital. [00:21:37-7](http://localhost:2300/file=C:/Users/USER/Desktop/ENTRETIENS%20PREMATURITE/CMA-DO/MCD-DO.mp3time=1297700)

00:20:14-2 Interviewer: D'accord. Est-ce que vous avez les plateaux techniques nécessaires ? 00:20:17-7

00:20:17-7 Person 1: Ça se retrouvait dans ce que je disais. Moi, j'ai vu dans les plateaux techniques, c'est ce que je disais. Les plateaux techniques, ça va être l'agent de santé suffisamment compétent pour ça. Les équipements médicaux qui vont répondre à ça. Dans l'équipement médical, on n'a pas de couveuses. Donc, d'abord, ça va commencer par... On n'a pas une unité même. Il n'y a pas un service adapté pour ça ici au CMA. On n'a pas un service de néonatologie. Donc, le service dont je parlais, SMK, Soins Maternels bébés kangourous, là. Au fait, ça nous permet, c'est ceux qui sont vraiment... Ce ne sont pas les très grands prématurés, là. C'est ceux qui ne sont pas loin de la date de l'accouchement normale. Et qu'on récupère avec les faibles poids de naissance. Ça nous permet de les gérer. Mais à une certaine phase, là, on ne peut pas. On ne peut pas gérer ça. Ils sont obligés donc d'envoyer. Donc, je veux dire qu'on n'a pas le dispositif nécessaire pour gérer les grands prématurés. Il faut les envoyer à l'hôpital. 00:21:21-1

00:21:21-1 Interviewer: Quand vous parlez des grands prématurés, vous faites allusion à quel type de prématurés ? 00:21:25-4

00:21:25-4 Person 1: Je parle des prématurés qui, puisqu'à partir d'un certain nombre de poids, nous, nous pouvons les gérer. Et quand on ne peut pas les gérer, il faut envoyer à l'hôpital. 00:21:37-7

**Interviewer (00:20:14-2):** Do you have the necessary technical platforms?

**Person 1 (00:20:17-7):** Our technical platforms are limited. We lack essential medical equipment, such as incubators, and we don't have a specialized neonatology department. Our Kangaroo Mother Care (SMK) service can manage babies who are near their due date and have low birth weights, but not the very premature ones. When babies reach a certain stage of prematurity, beyond our capacity, we must send them to the hospital. We're not equipped to handle very premature babies here at the CMA.

**Interviewer (00:21:25-4):** What kind of premature babies are you referring to?

**Person 1 (00:21:25-4):** I'm referring to those premature babies who, when they reach a certain weight, we can manage. If they are too premature or under a specific weight threshold, we cannot handle them and must refer them to the hospital for specialized care.

### Manager for maternity care unit of Do medical center with surgical branch

[00:16:16-0](http://localhost:2300/file=C:/Users/USER/Desktop/ENTRETIENS%20PREMATURITE/CMA-DO/RESPONSABLE%20UNITE%20DE%20SOIN%20CMA-DO.mp3time=976000) Interviewer: Est-ce que vous avez le plateau technique nécessaire ici... Ça veut dire que le personnel formé, les équipements, les médicaments, pour les prises en charge des cas de naissances prématurées? [00:16:28-0](http://localhost:2300/file=C:/Users/USER/Desktop/ENTRETIENS%20PREMATURITE/CMA-DO/RESPONSABLE%20UNITE%20DE%20SOIN%20CMA-DO.mp3time=988000)

[00:16:28-0](http://localhost:2300/file=C:/Users/USER/Desktop/ENTRETIENS%20PREMATURITE/CMA-DO/RESPONSABLE%20UNITE%20DE%20SOIN%20CMA-DO.mp3time=988000) Person 1: Non, je dirais non. Le plateau technique n'est pas, en tout cas, suffisamment fourni pour pouvoir le faire. Parce que même quand nous, on va vouloir les référer là, il n'y a pas trop de conditions, on va dire, bon, c'est de bien envelopper l'enfant, le nouveau, ce prématuré là, et voilà.Et l'envoyer directement. Donc, ça fait que ça peut jouer sur le temps de transfert et tout ça. Voilà. Donc, on n'a pas vraiment les équipements nécessaires pour le faire correctement. [00:16:56-6](http://localhost:2300/file=C:/Users/USER/Desktop/ENTRETIENS%20PREMATURITE/CMA-DO/RESPONSABLE%20UNITE%20DE%20SOIN%20CMA-DO.mp3time=1016600)

00:16:16-0 Interviewer: Est-ce que vous avez le plateau technique nécessaire ici... Ça veut dire que le personnel formé, les équipements, les médicaments, pour les prises en charge des cas de naissances prématurées ? 00:16:28-0

00:16:28-0 Person 1: Non, je dirais non. Le plateau technique n'est pas, en tout cas, suffisamment fourni pour pouvoir le faire. Parce que même quand nous, on va vouloir les référer là, il n'y a pas trop de conditions, on va dire, bon, c'est de bien envelopper l'enfant, le nouveau, ce prématuré là, et voilà. Et l'envoyer directement. Donc, ça fait que ça peut jouer sur le temps de transfert et tout ça. Voilà. Donc, on n'a pas vraiment les équipements nécessaires pour le faire correctement. 00:16:56-6

**Interviewer (00:16:16-0):** Do you have the necessary technical platform here... That means trained personnel, equipment, medications, for the management of cases of premature births?

**Person 1 (00:16:28-0):** No, I would say no. The technical platform is not, in any case, sufficiently provided to be able to do this. Because even when we want to refer them there, there aren't too many conditions, we'll say, well, it's to wrap the child well, the new one, this premature baby, and that's it. And send it directly. So, that means that it can affect the transfer time and all that. So. So, we don't really have the necessary equipment to do it properly.

### Focus group of Colma1 health and social promotion center

[00:16:13-0](http://localhost:2300/file=C:/Users/USER/Desktop/ENTRETIENS%20PREMATURITE/CSPS-COLMA1/FOCUS%20GROUPE%20COLMA1.mp3time=973000) Interviewer: Est ce que vous avez lze plateau technique necessaire au sein du CSPS , les medicaments, le personnel formé, les équipements? [00:16:28-0](http://localhost:2300/file=C:/Users/USER/Desktop/ENTRETIENS%20PREMATURITE/CSPS-COLMA1/FOCUS%20GROUPE%20COLMA1.mp3time=988000)

[00:16:28-0](http://localhost:2300/file=C:/Users/USER/Desktop/ENTRETIENS%20PREMATURITE/CSPS-COLMA1/FOCUS%20GROUPE%20COLMA1.mp3time=988000) Person 6: (rires) nous n'en avons pas. franchement on en a pas, on fait avec les moyens de bord. quand il sort on essaie vraiment de le proteger de le mettre au chaud et comme, elle l'a dit de conseiller sa maman. En tout cas si les reflexes sont bons ce sont les conseils seulement sinon nous n'avons rien d'autre. Maintenant s'il y a un autre problème, on le refère c'est tout. [00:16:49-8](http://localhost:2300/file=C:/Users/USER/Desktop/ENTRETIENS%20PREMATURITE/CSPS-COLMA1/FOCUS%20GROUPE%20COLMA1.mp3time=1009800)

[00:16:49-8](http://localhost:2300/file=C:/Users/USER/Desktop/ENTRETIENS%20PREMATURITE/CSPS-COLMA1/FOCUS%20GROUPE%20COLMA1.mp3time=1009800) Interviewer: Est ce que vous avez eu à faire à des cas de grand prematurité? [00:16:55-3](http://localhost:2300/file=C:/Users/USER/Desktop/ENTRETIENS%20PREMATURITE/CSPS-COLMA1/FOCUS%20GROUPE%20COLMA1.mp3time=1015300)

[00:16:55-3](http://localhost:2300/file=C:/Users/USER/Desktop/ENTRETIENS%20PREMATURITE/CSPS-COLMA1/FOCUS%20GROUPE%20COLMA1.mp3time=1015300) Person 5: Oui, on a eu des grands prématurés içi. dans ce cas on les refère [00:17:02-0](http://localhost:2300/file=C:/Users/USER/Desktop/ENTRETIENS%20PREMATURITE/CSPS-COLMA1/FOCUS%20GROUPE%20COLMA1.mp3time=1022000)

[00:17:02-0](http://localhost:2300/file=C:/Users/USER/Desktop/ENTRETIENS%20PREMATURITE/CSPS-COLMA1/FOCUS%20GROUPE%20COLMA1.mp3time=1022000) Interviewer: Comment est ce que ces cas sont gerés içi au sein du CSPS? [00:17:04-4](http://localhost:2300/file=C:/Users/USER/Desktop/ENTRETIENS%20PREMATURITE/CSPS-COLMA1/FOCUS%20GROUPE%20COLMA1.mp3time=1024400)

[00:17:04-4](http://localhost:2300/file=C:/Users/USER/Desktop/ENTRETIENS%20PREMATURITE/CSPS-COLMA1/FOCUS%20GROUPE%20COLMA1.mp3time=1024400) Person 1: (rires) comme on vous l'a dit les moyens sont limités ici. c'est de le protèger, le mettre au chaud et appeler l'ambulance pour une evacuation. géneralement une heure après son accouchement il est déjà parti au niveau superieur, nous ne le gardons pas ici. [00:17:19-2](http://localhost:2300/file=C:/Users/USER/Desktop/ENTRETIENS%20PREMATURITE/CSPS-COLMA1/FOCUS%20GROUPE%20COLMA1.mp3time=1039200)

00:16:13-0 Interviewer: Est-ce que vous avez le plateau technique nécessaire au sein du CSPS, les médicaments, le personnel formé, les équipements ? 00:16:28-0

00:16:28-0 Personne 6: (rires) Nous n'en avons pas. Franchement, on en a pas, on fait avec les moyens de bord. Quand il sort, on essaie vraiment de le protéger, de le mettre au chaud et comme elle l'a dit, de conseiller sa maman. En tout cas, si les réflexes sont bons, ce sont les conseils seulement, sinon nous n'avons rien d'autre. Maintenant, s'il y a un autre problème, on le réfère, c'est tout. 00:16:49-8

00:16:49-8 Interviewer: Est-ce que vous avez eu à faire à des cas de grande prématurité ? 00:16:55-3

00:16:55-3 Personne 5: Oui, on a eu des grands prématurés ici. Dans ce cas, on les réfère. 00:17:02-0

00:17:02-0 Interviewer: Comment est-ce que ces cas sont gérés ici au sein du CSPS ? 00:17:04-4

00:17:04-4 Personne 1: (rires) Comme on vous l'a dit, les moyens sont limités ici. C'est de le protéger, le mettre au chaud et appeler l'ambulance pour une évacuation. Généralement, une heure après son accouchement, il est déjà parti au niveau supérieur, nous ne le gardons pas ici. 00:17:19-2

**Interviewer (00:16:13-0):** Do you have the necessary technical platform within the CSPS, the medicines, the trained personnel, the equipment?

**Person 6 (00:16:28-0):** (laughs) We don't have any. Frankly, we don't have any; we make do with the means at hand. When he goes out we really try to protect him, keep him warm, and like, she said, advise his mom. In any case, if the reflexes are good, it is only the advice; otherwise, we have nothing else. Now if there is another problem, we refer it, that's all.

**Interviewer (00:16:49-8):** Have you had to deal with cases of extreme prematurity?

**Person 5 (00:16:55-3):** Yes, we had very premature babies here. In this case, we refer them.

**Interviewer (00:17:02-0):** How are these cases managed here within the CSPS?

**Person 1 (00:17:04-4):** (laughs) As we told you, resources are limited here. It's to protect him, keep him warm, and call the ambulance for an evacuation. Usually, an hour after giving birth, he has already gone to the next level; we do not keep him here.

### Major of Colma1 health and social promotion center

[00:13:37-6](http://localhost:2300/file=C:/Users/USER/Desktop/ENTRETIENS%20PREMATURITE/CSPS-COLMA1/MAJOR%20CSPS%20COLMA1.MP3time=817600) Interviewer: Maintenant, est-ce que vous avez le plateau technique nécessaire dans votre centre de santé ? [00:13:41-4](http://localhost:2300/file=C:/Users/USER/Desktop/ENTRETIENS%20PREMATURITE/CSPS-COLMA1/MAJOR%20CSPS%20COLMA1.MP3time=821400)

[00:13:41-4](http://localhost:2300/file=C:/Users/USER/Desktop/ENTRETIENS%20PREMATURITE/CSPS-COLMA1/MAJOR%20CSPS%20COLMA1.MP3time=821400) Person 1: voilà justement donc nous, on n'a pas le plateau technique justement, nous repondons à un CSPS,et un CSPS ne peut pas prendre en charge donc les prématurés, voilà c'est ça. [00:13:50-7](http://localhost:2300/file=C:/Users/USER/Desktop/ENTRETIENS%20PREMATURITE/CSPS-COLMA1/MAJOR%20CSPS%20COLMA1.MP3time=830700)

[00:13:50-7](http://localhost:2300/file=C:/Users/USER/Desktop/ENTRETIENS%20PREMATURITE/CSPS-COLMA1/MAJOR%20CSPS%20COLMA1.MP3time=830700) Interviewer: Maintenant, Vous dites que vous les référez lorsque vous vous rendez compte que vous ne pouvez pas les prendre en charge ou bien au cas échéant vous les transférez ? [00:13:59-7](http://localhost:2300/file=C:/Users/USER/Desktop/ENTRETIENS%20PREMATURITE/CSPS-COLMA1/MAJOR%20CSPS%20COLMA1.MP3time=839700)

[00:13:59-7](http://localhost:2300/file=C:/Users/USER/Desktop/ENTRETIENS%20PREMATURITE/CSPS-COLMA1/MAJOR%20CSPS%20COLMA1.MP3time=839700) Person 1: Oui. [00:14:00-9](http://localhost:2300/file=C:/Users/USER/Desktop/ENTRETIENS%20PREMATURITE/CSPS-COLMA1/MAJOR%20CSPS%20COLMA1.MP3time=840900)

00:13:37-6 Interviewer: Maintenant, est-ce que vous avez le plateau technique nécessaire dans votre centre de santé ? 00:13:41-4

00:13:41-4 Person 1: Voilà justement donc nous, on n'a pas le plateau technique justement, nous répondons à un CSPS, et un CSPS ne peut pas prendre en charge donc les prématurés, voilà c'est ça. 00:13:50-7

00:13:50-7 Interviewer: Maintenant, Vous dites que vous les référez lorsque vous vous rendez compte que vous ne pouvez pas les prendre en charge ou bien au cas échéant vous les transférez ? 00:13:59-7

00:13:59-7 Person 1: Oui. 00:14:00-9

**Interviewer (00:13:37-6):** Do you have the necessary technical support in your health center?

**Person 1 (00:13:41-4):** No, we don't have the technical platform needed. Our facility operates as a CSPS (Community Health Service Provider), and CSPS facilities are not equipped to handle premature babies.

**Interviewer (00:13:59-7):** So, you refer them to another facility when you realize you can't take care of them?

**Person 1 (00:14:00-9):** Yes, that's correct. When we encounter cases of premature births that we can't manage due to our limited resources, we refer or transfer them to facilities that have the necessary capabilities and equipment for their care.

### Maternity manager of Colma1 health and social promotion center

[00:22:17-0](http://localhost:2300/file=C:/Users/USER/Desktop/ENTRETIENS%20PREMATURITE/CSPS-COLMA1/RESPONSABLE%20MATERNITE-COLMA1.mp3time=1337000) Interviewer: Est ce que vous avez le plateau technique necessaire ici ? [00:22:19-4](http://localhost:2300/file=C:/Users/USER/Desktop/ENTRETIENS%20PREMATURITE/CSPS-COLMA1/RESPONSABLE%20MATERNITE-COLMA1.mp3time=1339400)

[00:22:19-4](http://localhost:2300/file=C:/Users/USER/Desktop/ENTRETIENS%20PREMATURITE/CSPS-COLMA1/RESPONSABLE%20MATERNITE-COLMA1.mp3time=1339400) Person 1: Je vous ai dit, on n'a pas le plateau technique. Les plateaux techniques, il n'y en a pas. [00:22:26-3](http://localhost:2300/file=C:/Users/USER/Desktop/ENTRETIENS%20PREMATURITE/CSPS-COLMA1/RESPONSABLE%20MATERNITE-COLMA1.mp3time=1346300)

[00:22:26-3](http://localhost:2300/file=C:/Users/USER/Desktop/ENTRETIENS%20PREMATURITE/CSPS-COLMA1/RESPONSABLE%20MATERNITE-COLMA1.mp3time=1346300) Est ce qu'il vous arrive de faire face à des cas de grand prématurés dans votre centre de santé? [00:22:31-4](http://localhost:2300/file=C:/Users/USER/Desktop/ENTRETIENS%20PREMATURITE/CSPS-COLMA1/RESPONSABLE%20MATERNITE-COLMA1.mp3time=1351400)

[00:22:31-4](http://localhost:2300/file=C:/Users/USER/Desktop/ENTRETIENS%20PREMATURITE/CSPS-COLMA1/RESPONSABLE%20MATERNITE-COLMA1.mp3time=1351400) Person 1: grande prématurité ? Grande prématurité ? Oui, entre 28 à 34 semaines d'aménorrhée.Oui [00:22:40-9](http://localhost:2300/file=C:/Users/USER/Desktop/ENTRETIENS%20PREMATURITE/CSPS-COLMA1/RESPONSABLE%20MATERNITE-COLMA1.mp3time=1360900)

00:22:17-0 Interviewer: Est-ce que vous avez le plateau technique nécessaire ici ? 00:22:19-4

00:22:19-4 Personne 1: Je vous ai dit, on n'a pas le plateau technique. Les plateaux techniques, il n'y en a pas. 00:22:26-3

00:22:26-3 Est-ce qu'il vous arrive de faire face à des cas de grands prématurés dans votre centre de santé ? 00:22:31-4

00:22:31-4 Personne 1: Grande prématurité ? Grande prématurité ? Oui, entre 28 à 34 semaines d'aménorrhée. Oui 00:22:40-9

**Interviewer (00:22:17-0):** Do you have the necessary technical support here?

**Person 1 (00:22:19-4):** I told you, we don't have the technical platform. There are no technical platforms.

**Interviewer (00:22:26-3):** Do you ever encounter cases of very premature babies in your health center?

**Person 1 (00:22:31-4):** Extreme prematurity? Great prematurity? Yes, between 28 to 34 weeks of amenorrhea. Yes.

### Focus group of Farakan health and social promotion center

[00:28:29-8](http://localhost:2300/file=C:/Users/USER/Desktop/ENTRETIENS%20PREMATURITE/CSPS_FARAKAN/FOCUS_GROUPE-CSPS_FARAKAN.MP3time=1709800) Interviewer: Est-ce que vous avez de plateaux techniques nécessaires ? C'est-à-dire les personnels formés, les médicaments, les équipements pour prendre en charge les cas de naissance prématurée? [00:28:42-6](http://localhost:2300/file=C:/Users/USER/Desktop/ENTRETIENS%20PREMATURITE/CSPS_FARAKAN/FOCUS_GROUPE-CSPS_FARAKAN.MP3time=1722600)

[00:28:42-6](http://localhost:2300/file=C:/Users/USER/Desktop/ENTRETIENS%20PREMATURITE/CSPS_FARAKAN/FOCUS_GROUPE-CSPS_FARAKAN.MP3time=1722600) Person 3: Non, nous n'avons pas d'équipements nécessaires. [00:28:50-1](http://localhost:2300/file=C:/Users/USER/Desktop/ENTRETIENS%20PREMATURITE/CSPS_FARAKAN/FOCUS_GROUPE-CSPS_FARAKAN.MP3time=1730100)

[00:28:50-1](http://localhost:2300/file=C:/Users/USER/Desktop/ENTRETIENS%20PREMATURITE/CSPS_FARAKAN/FOCUS_GROUPE-CSPS_FARAKAN.MP3time=1730100) Person 1: Non pas d'équipement nécessaire. C'est pour ça nous les évacuations à temps, mais comme il y a toujours des forces majeures quand ça se produit aussi nous l'acheminons immédiatement. [00:29:13-4](http://localhost:2300/file=C:/Users/USER/Desktop/ENTRETIENS%20PREMATURITE/CSPS_FARAKAN/FOCUS_GROUPE-CSPS_FARAKAN.MP3time=1753400)

[00:29:13-4](http://localhost:2300/file=C:/Users/USER/Desktop/ENTRETIENS%20PREMATURITE/CSPS_FARAKAN/FOCUS_GROUPE-CSPS_FARAKAN.MP3time=1753400) Interviewer: Est qu'au niveau de la naissance prématurée au sein de votre centre de santé, vous avez eu à faire à des cas de grands prématurés? [00:29:24-9](http://localhost:2300/file=C:/Users/USER/Desktop/ENTRETIENS%20PREMATURITE/CSPS_FARAKAN/FOCUS_GROUPE-CSPS_FARAKAN.MP3time=1764900)

[00:29:24-9](http://localhost:2300/file=C:/Users/USER/Desktop/ENTRETIENS%20PREMATURITE/CSPS_FARAKAN/FOCUS_GROUPE-CSPS_FARAKAN.MP3time=1764900) Person 7: Il y en a parce qu'avec le problème d'insécurité, il y en a qui arrivent. Peut-être même accoucher à domicile en nous achemine ici ou bien souvent la femme arrive à dilatation complète on ne peut plus évacuer.Nous effectuons l'accouchement et ensuite maintenant voilà, sinon il y en a eu beaucoup. [00:29:52-6](http://localhost:2300/file=C:/Users/USER/Desktop/ENTRETIENS%20PREMATURITE/CSPS_FARAKAN/FOCUS_GROUPE-CSPS_FARAKAN.MP3time=1792600)

00:28:29-8 Interviewer: Est-ce que vous avez de plateaux techniques nécessaires ? C'est-à-dire les personnels formés, les médicaments, les équipements pour prendre en charge les cas de naissance prématurée ? 00:28:42-6

00:28:42-6 Person 3: Non, nous n'avons pas d'équipements nécessaires. 00:28:50-1

00:28:50-1 Person 1: Non pas d'équipement nécessaire. C'est pour ça nous les évacuations à temps, mais comme il y a toujours des forces majeures quand ça se produit aussi nous l'acheminons immédiatement. 00:29:13-4

00:29:13-4 Interviewer: Est-ce qu'au niveau de la naissance prématurée au sein de votre centre de santé, vous avez eu à faire à des cas de grands prématurés? 00:29:24-9

00:29:24-9 Personne 7: Il y en a, parce qu'avec le problème d'insécurité, il y en a qui arrivent. Peut-être même accoucher à domicile et nous acheminent ici, ou bien souvent la femme arrive à dilatation complète et on ne peut plus évacuer. Nous effectuons l'accouchement et ensuite, maintenant, voilà, sinon il y en a eu beaucoup. 00:29:52-6

**Interviewer (00:28:29-8):** Do you have the necessary technical platforms? That is to say trained personnel, medications, equipment to manage cases of premature birth?

**Person 3 (00:28:42-6):** No, we don't have any of the necessary equipment.

**Person 1 (00:28:50-1):** No, we lack the needed equipment. That's why we evacuate cases of premature births as soon as possible. However, in cases of force majeure, when immediate evacuation is required, we transport them right away.

**Interviewer (00:29:13-4):** "Regarding premature births at your health center, have you encountered cases involving very premature babies?"

**Person 7 (00:29:24-9):** "Yes, we have had such cases. Often due to security issues, some women arrive late. Some might even give birth at home before being sent here. At times, a woman might reach full dilation before she can be evacuated, so we have to conduct the delivery here. We've indeed encountered many such instances."

### Major of Farakan health and social promotion center

[00:10:36-6](http://localhost:2300/file=C:/Users/USER/Desktop/ENTRETIENS%20PREMATURITE/CSPS_FARAKAN/MAJOR%20FARAKAN.MP3time=636600) Interviewer: (Après une pause).Je pense que nous etons au niveau des defis par rapport à la prématurité au sein de votre centre de santé. Est ce que vous avez le plateau technique necessaire, c'est à dire le personnel formé, les medicaments, et les equipements pour prendre en charge les cas de naissances prématurés? [00:11:10-5](http://localhost:2300/file=C:/Users/USER/Desktop/ENTRETIENS%20PREMATURITE/CSPS_FARAKAN/MAJOR%20FARAKAN.MP3time=670500)

[00:11:10-5](http://localhost:2300/file=C:/Users/USER/Desktop/ENTRETIENS%20PREMATURITE/CSPS_FARAKAN/MAJOR%20FARAKAN.MP3time=670500) Person 1: Lorsqu'il y a des prématurés, on réfère à l'echelon superieur, parce que nous n'avons pas le plateau technique necessaire. [00:11:21-6](http://localhost:2300/file=C:/Users/USER/Desktop/ENTRETIENS%20PREMATURITE/CSPS_FARAKAN/MAJOR%20FARAKAN.MP3time=681600)

00:10:36-6 Interviewer: (Après une pause). Je pense que nous en sommes au niveau des défis par rapport à la prématurité au sein de votre centre de santé. Est-ce que vous avez le plateau technique nécessaire, c'est-à-dire le personnel formé, les médicaments et les équipements pour prendre en charge les cas de naissances prématurées ? 00:11:10-5

00:11:10-5 Person 1: Lorsqu'il y a des prématurés, on réfère à l'échelon supérieur, parce que nous n'avons pas le plateau technique nécessaire. 00:11:21-6

**Interviewer (00:10:36-6):** (After a pause). I think we are discussing the challenges related to prematurity within your health center. Do you have the necessary technical support, that is to say trained personnel, medications, and equipment to handle cases of premature births?

**Person 1 (00:11:10-5):** When we encounter cases of premature births, we refer them to a higher-level facility because we do not have the necessary technical support here.

### Maternity manager of Farakan health and social promotion center

[00:16:33-6](http://localhost:2300/file=C:/Users/USER/Desktop/ENTRETIENS%20PREMATURITE/CSPS_FARAKAN/RESPONSABLE%20MATERNITE%20FARAKAN.MP3time=993600) Interviewer: Est ce que vous avez le plateau technique necessaire au sein de votre centre de santé pour prendre en charge un nouveau né prématuré? [00:16:39-2](http://localhost:2300/file=C:/Users/USER/Desktop/ENTRETIENS%20PREMATURITE/CSPS_FARAKAN/RESPONSABLE%20MATERNITE%20FARAKAN.MP3time=999200)

[00:16:39-2](http://localhost:2300/file=C:/Users/USER/Desktop/ENTRETIENS%20PREMATURITE/CSPS_FARAKAN/RESPONSABLE%20MATERNITE%20FARAKAN.MP3time=999200) Person 1: Pour prendre en charge un nouveau né prématuré, non.Les nouveaux nés prématurés s'il n y a pas de problème, ils sont pris en charge au niveau du CMA qui a une unité kangourou. [00:16:59-3](http://localhost:2300/file=C:/Users/USER/Desktop/ENTRETIENS%20PREMATURITE/CSPS_FARAKAN/RESPONSABLE%20MATERNITE%20FARAKAN.MP3time=1019300)

[00:16:59-3](http://localhost:2300/file=C:/Users/USER/Desktop/ENTRETIENS%20PREMATURITE/CSPS_FARAKAN/RESPONSABLE%20MATERNITE%20FARAKAN.MP3time=1019300) Interviewer: Qu'est ce que vous faites au cas écheant et si vous constater que vous ne pouvez pas prendre en charge une naissance prématurée? [00:17:05-6](http://localhost:2300/file=C:/Users/USER/Desktop/ENTRETIENS%20PREMATURITE/CSPS_FARAKAN/RESPONSABLE%20MATERNITE%20FARAKAN.MP3time=1025600)

[00:17:05-6](http://localhost:2300/file=C:/Users/USER/Desktop/ENTRETIENS%20PREMATURITE/CSPS_FARAKAN/RESPONSABLE%20MATERNITE%20FARAKAN.MP3time=1025600) Person 1: Nous referons [00:17:09-3](http://localhost:2300/file=C:/Users/USER/Desktop/ENTRETIENS%20PREMATURITE/CSPS_FARAKAN/RESPONSABLE%20MATERNITE%20FARAKAN.MP3time=1029300)

[00:17:09-3](http://localhost:2300/file=C:/Users/USER/Desktop/ENTRETIENS%20PREMATURITE/CSPS_FARAKAN/RESPONSABLE%20MATERNITE%20FARAKAN.MP3time=1029300) Interviewer: Vous referez ou? [00:17:11-0](http://localhost:2300/file=C:/Users/USER/Desktop/ENTRETIENS%20PREMATURITE/CSPS_FARAKAN/RESPONSABLE%20MATERNITE%20FARAKAN.MP3time=1031000)

[00:17:11-0](http://localhost:2300/file=C:/Users/USER/Desktop/ENTRETIENS%20PREMATURITE/CSPS_FARAKAN/RESPONSABLE%20MATERNITE%20FARAKAN.MP3time=1031000) Person 1: Nous les referons à l'hôpital SOURO SANOU. Les grands prématurés sont évacués à l'hôpital, l'unité kangourou au niveau du CMA ne peut pas en charge les prematurés avec les detresses respiratoires. Même les cas d'infections sont evacués vers le CHURSS. Géneralement, l'unité kangourou se sont les bébés saints, il n' y a pas d'infection et aussi le poids compte , je pense que c'est à partir de 2kg. [00:17:50-0](http://localhost:2300/file=C:/Users/USER/Desktop/ENTRETIENS%20PREMATURITE/CSPS_FARAKAN/RESPONSABLE%20MATERNITE%20FARAKAN.MP3time=1070000)

[00:17:50-0](http://localhost:2300/file=C:/Users/USER/Desktop/ENTRETIENS%20PREMATURITE/CSPS_FARAKAN/RESPONSABLE%20MATERNITE%20FARAKAN.MP3time=1070000) Interviewer: L'unité kangourou s'occupe de la prise en charge des nouveaux nés prématurés [00:17:53-8](http://localhost:2300/file=C:/Users/USER/Desktop/ENTRETIENS%20PREMATURITE/CSPS_FARAKAN/RESPONSABLE%20MATERNITE%20FARAKAN.MP3time=1073800)

[00:17:53-8](http://localhost:2300/file=C:/Users/USER/Desktop/ENTRETIENS%20PREMATURITE/CSPS_FARAKAN/RESPONSABLE%20MATERNITE%20FARAKAN.MP3time=1073800) Interviewer: Oui [00:17:56-3](http://localhost:2300/file=C:/Users/USER/Desktop/ENTRETIENS%20PREMATURITE/CSPS_FARAKAN/RESPONSABLE%20MATERNITE%20FARAKAN.MP3time=1076300)

[00:17:56-3](http://localhost:2300/file=C:/Users/USER/Desktop/ENTRETIENS%20PREMATURITE/CSPS_FARAKAN/RESPONSABLE%20MATERNITE%20FARAKAN.MP3time=1076300) Interviewer: Est ce que la mère aussi est inclue dans la prise en charge? [00:17:58-0](http://localhost:2300/file=C:/Users/USER/Desktop/ENTRETIENS%20PREMATURITE/CSPS_FARAKAN/RESPONSABLE%20MATERNITE%20FARAKAN.MP3time=1078000)

[00:17:58-0](http://localhost:2300/file=C:/Users/USER/Desktop/ENTRETIENS%20PREMATURITE/CSPS_FARAKAN/RESPONSABLE%20MATERNITE%20FARAKAN.MP3time=1078000) Person 1: Oui il y a la mère qui est inclue puisqu'on va lui montrer comment il faire. [00:18:04-9](http://localhost:2300/file=C:/Users/USER/Desktop/ENTRETIENS%20PREMATURITE/CSPS_FARAKAN/RESPONSABLE%20MATERNITE%20FARAKAN.MP3time=1084900)

00:16:33-6 Interviewer: Est-ce que vous avez le plateau technique nécessaire au sein de votre centre de santé pour prendre en charge un nouveau-né prématuré ? 00:16:39-2

00:16:39-2 Person 1: Pour prendre en charge un nouveau-né prématuré, non. Les nouveau-nés prématurés s'il n'y a pas de problème, ils sont pris en charge au niveau du CMA qui a une unité kangourou. 00:16:59-3

00:16:59-3 Interviewer: Qu'est-ce que vous faites au cas échéant et si vous constatez que vous ne pouvez pas prendre en charge une naissance prématurée ? 00:17:05-6

00:17:05-6 Person 1: Nous référons. 00:17:09-3

00:17:09-3 Interviewer: Vous référerez où ? 00:17:11-0

00:17:11-0 Person 1: Nous les référons à l'hôpital SOURO SANOU. Les grands prématurés sont évacués à l'hôpital, l'unité kangourou au niveau du CMA ne peut pas prendre en charge les prématurés avec des détresses respiratoires. Même les cas d'infections sont évacués vers le CHURSS. Généralement, l'unité kangourou s'occupe des bébés sains, il n'y a pas d'infection, et aussi le poids compte, je pense que c'est à partir de 2 kg. 00:17:50-0

00:17:50-0 Interviewer: L'unité kangourou s'occupe de la prise en charge des nouveau-nés prématurés ? 00:17:53-8

00:17:53-8 Interviewer: Oui. 00:17:56-3

00:17:56-3 Interviewer: Est-ce que la mère aussi est incluse dans la prise en charge ? 00:17:58-0

00:17:58-0 Person 1: Oui, il y a la mère qui est incluse puisqu'on va lui montrer comment faire. 00:18:04-9

**Interviewer (00:16:33-6):** Do you have the necessary technical support within your health center to care for a premature newborn?

**Person 1 (00:16:39-2):** To take care of a premature newborn, no. Premature newborns, if there are no problems, are taken care of at the CMA level which has a kangaroo unit.

**Interviewer (00:16:59-3):** What do you do if you find that you cannot handle a premature birth?

**Person 1 (00:17:05-6):** We refer.

**Interviewer (00:17:09-3):** Where will you refer?

**Person 1 (00:17:11-0):** We refer them to SOURO SANOU hospital. Extremely premature babies are evacuated to the hospital; the kangaroo unit at the CMA cannot care for premature babies with respiratory distress. Even cases of infections are evacuated to the CHURSS. Generally, the kangaroo unit takes care of healthy babies, there is no infection, and also weight matters, I think it's from 2 kg.

**Interviewer (00:17:50-0):** The kangaroo unit takes care of premature newborns?

**Interviewer (00:17:53-8):** Yes.

**Interviewer (00:17:56-3):** Is the mother also included in the care?

**Person 1 (00:17:58-0):** Yes, there is the mother who is included since we will show her how to do it.

# 8. Services and care provided to premature babies and their mothers

### Focus group of Accart-ville urban medical center

[00:19:01-8](http://localhost:2300/file=C:/Users) Interviewer: nous allons toujours poursuivre,maintenant quel types de services sont fournis aux nourrissons prématurés et à leur mère le cas échéant dans votre centre de santé? lorsque la prématurité survient au sein de votre centre de santé, quels sont les types de services qui sont fournis aux nourrissons prematurés et à leurs mères? [00:19:35-1](http://localhost:2300/file=C:/Users/USER/)

[00:19:35-1](http://localhost:2300/file=C:/Users/USER/Desktop/ENTRETIENS%20PREMATURITE/CSPS-ACCART-VILLE/FOCUS%20GROUPE%20ACCART-VILLE-BON.mp3time=1175100) Person 6: lorsqu'un prematuré naît içi, nous donnons à la maman les mêmes soins que nous donnons aux autres femmes mais le bébé on le protège bien contre l'hypothermie et comme nous l'avons dit par manque de moyens nous les evacuons à l'echelon superieur [00:20:00-5](http://localhost:2300/file=C:/Users/USER/Desktop/ENTRETIENS)

00:19:01-8 Interviewer: Nous allons toujours poursuivre, maintenant, quels types de services sont fournis aux nourrissons prématurés et à leur mère, le cas échéant, dans votre centre de santé ? Lorsque la prématurité survient au sein de votre centre de santé, quels sont les types de services qui sont fournis aux nourrissons prématurés et à leurs mères ? 00:19:35-1

00:19:35-1 Personne 6: Lorsqu'un prématuré naît ici, nous donnons à la maman les mêmes soins que nous donnons aux autres femmes, mais le bébé, on le protège bien contre l'hypothermie et, comme nous l'avons dit, par manque de moyens, nous les évacuons à l'échelon supérieur. 00:20:00-5

**Interviewer (00:19:01-8):** "Let's move on. Could you describe the types of services your health center provides to premature infants and their mothers? Specifically, what care is available when prematurity occurs?"

**Person 6 (00:19:35-1):** "When we have a premature birth, the mother receives the same care as other women. However, we take extra precautions to protect the baby from hypothermia. As we've mentioned earlier, due to our limited resources, we often need to transfer these cases to a higher-level facility for more specialized care.

### Manager of Accart-ville urban medical center

[00:14:56-0](http://localhost:2300/file=C:/Users/USER/Desktop/ENTRETIENS%20PREMATURITE/CSPS-ACCART-VILLE/RESPONSABLE%20CMU%20ACCART-VILLE.MP3time=896000)Interviewer: Mais maintenant, le cas échéant, lorsque vous avez affaire à un cas de prématuré au sein de votre santé, quels sont les soins et les services qui sont rapportés au nourrisson et à la mère avant le transfert? [00:15:09-9](http://localhost:2300/file=C:/Users/USER/Desktop/ENTRETIENS%20PREMATURITE/CSPS-ACCART-VILLE/RESPONSABLE%20CMU%20ACCART-VILLE.MP3time=909900)

[00:15:09-9](http://localhost:2300/file=C:/Users/USER/Desktop/ENTRETIENS%20PREMATURITE/CSPS-ACCART-VILLE/RESPONSABLE%20CMU%20ACCART-VILLE.MP3time=909900)Person 1: Bon, en fait c'est de faire les prémières réanimations qu'on peut d'abord, c'est de désobstruer de liberer les voies , voir s'il n y a pas de détresse surtout, on peut mesurer puisqu'on a un saturometre que moi même j'avais disponibilité pour essayer de voir mais bon en fait on ne peut pas aller très loin. En fait, on est très très limité. par exemple je pense que chez les prématurés, il faut une voie ombilical. Donc je pense que chez moi personne n'a été formé pour ça, même personnellement moi même je ne suis pas formé pour ça. [00:15:52-2](http://localhost:2300/file=C:/Users/USER/Desktop/ENTRETIENS%20PREMATURITE/CSPS-ACCART-VILLE/RESPONSABLE%20CMU%20ACCART-VILLE.MP3time=952200)

00:14:56-0 Interviewer: Mais maintenant, le cas échéant, lorsque vous avez affaire à un cas de prématuré au sein de votre santé, quels sont les soins et les services qui sont rapportés au nourrisson et à la mère avant le transfert ? 00:15:09-9

00:15:09-9 Person 1: Bon, en fait, c'est de faire les premières réanimations qu'on peut d'abord, c'est de désobstruer, de libérer les voies, voir s'il n'y a pas de détresse surtout, on peut mesurer puisqu'on a un saturomètre que moi-même j'avais disponible pour essayer de voir, mais bon en fait on ne peut pas aller très loin. En fait, on est très, très limité. Par exemple, je pense que chez les prématurés, il faut une voie ombilicale. Donc je pense que chez moi, personne n'a été formé pour ça, même personnellement moi-même je ne suis pas formé pour ça. 00:15:52-2

**Interviewer (00:14:56-0):** What care and services do you provide to the infant and mother before transferring them in case of a premature birth?

**Person 1 (00:15:09-9):** Our initial response includes basic resuscitation efforts, like clearing airways and checking for any signs of distress. We use a saturometer to monitor oxygen levels, but our capabilities are quite restricted. For instance, setting up an umbilical route is critical for premature babies, but no one here, including myself, is trained for that procedure.

### Maternity manager of Accart-ville urban medical center

[00:18:53-7](http://localhost:2300/file=C:/Users/USER/Desktop/ENTRETIENS%20PREMATURITE/CSPS-ACCART-VILLE/RESPONSABLE%20MATERNITE%20CMU%20ACCART-VILLE.MP3time=1133700) Interviewer: et au cas échéant?

[00:18:54-6](http://localhost:2300/file=C:/Users/USER/Desktop/ENTRETIENS%20PREMATURITE/CSPS-ACCART-VILLE/RESPONSABLE%20MATERNITE%20CMU%20ACCART-VILLE.MP3time=1134600) Person 1: au cas écheant , on peut garder s'il n 'y a pas de place, si la personne revient, le temps de surveiller au moins 72 heures avant de liberer tout en donnant des conseils sur la lutte contre l'hypothermie et surtout l'hygiène alimentaire et la propreté des mères, sensibiliser les mères du fait que ces enfants sont fragiles. nous faisons de notre mieux

00:18:53-7 Interviewer: Et au cas échéant ?

00:18:54-6 Person 1: Au cas échéant, on peut garder s'il n'y a pas de place, si la personne revient, le temps de surveiller au moins 72 heures avant de libérer, tout en donnant des conseils sur la lutte contre l'hypothermie et surtout l'hygiène alimentaire et la propreté des mères, sensibiliser les mères du fait que ces enfants sont fragiles. Nous faisons de notre mieux.

**Interviewer (00:18:53-7):** And in cases where evacuation isn't possible?

**Person 1 (00:18:54-6):** If necessary, and if there's no space available for transfer, we keep the baby for at least 72 hours for monitoring. During this time, we focus on combating hypothermia and maintaining food hygiene and cleanliness. We also educate mothers about the fragility of these children and do our best under the circumstances.

### Focus group of Do medical center with surgical branch

[00:19:23-3](http://localhost:2300/file=C:/Users/USER/Desktop/ENTRETIENS%20PREMATURITE/CMA-DO/FOCUS%20GROUPE%20CMA-DO.mp3time=1163300) Interviewer: Quels types de services sont fournisaux nourrissons prématurés et à leurs mères le cas échéant au sein du CMA de DÔ? [00:19:33-7](http://localhost:2300/file=C:/Users/USER/Desktop/ENTRETIENS%20PREMATURITE/CMA-DO/FOCUS%20GROUPE%20CMA-DO.mp3time=1173700)

[00:19:33-7](http://localhost:2300/file=C:/Users/USER/Desktop/ENTRETIENS%20PREMATURITE/CMA-DO/FOCUS%20GROUPE%20CMA-DO.mp3time=1173700) Person 6: A la mère et à l'enfant. La mère ce sont les mêmes soins que les accouchements normaux, mais à l'enfant se sont les soins du nouveau-né, il y a le chauffage, il faut tout ce qui rentre dans les soins des prématurés c'est à dire , il faut eviter le froid, c'est à dire que l'enfant soit refroidi, si l'enfant a une detresse respiratoire,il faut l'intuber et puis oxygener [00:20:11-2](http://localhost:2300/file=C:/Users/USER/Desktop/ENTRETIENS%20PREMATURITE/CMA-DO/FOCUS%20GROUPE%20CMA-DO.mp3time=1211200)

[00:20:11-2](http://localhost:2300/file=C:/Users/USER/Desktop/ENTRETIENS%20PREMATURITE/CMA-DO/FOCUS%20GROUPE%20CMA-DO.mp3time=1211200) Interviewer: Est ce que vous avez tout ce materiel? [00:20:13-2](http://localhost:2300/file=C:/Users/USER/Desktop/ENTRETIENS%20PREMATURITE/CMA-DO/FOCUS%20GROUPE%20CMA-DO.mp3time=1213200)

[00:20:13-2](http://localhost:2300/file=C:/Users/USER/Desktop/ENTRETIENS%20PREMATURITE/CMA-DO/FOCUS%20GROUPE%20CMA-DO.mp3time=1213200) Person 9: Il y en a, ce n'est pas assez mais il y en a. [00:20:16-7](http://localhost:2300/file=C:/Users/USER/Desktop/ENTRETIENS%20PREMATURITE/CMA-DO/FOCUS%20GROUPE%20CMA-DO.mp3time=1216700)

[00:20:16-7](http://localhost:2300/file=C:/Users/USER/Desktop/ENTRETIENS%20PREMATURITE/CMA-DO/FOCUS%20GROUPE%20CMA-DO.mp3time=1216700) Person 6: Il semble qu'il y en a. [00:20:20-8](http://localhost:2300/file=C:/Users/USER/Desktop/ENTRETIENS%20PREMATURITE/CMA-DO/FOCUS%20GROUPE%20CMA-DO.mp3time=1220800)

[00:20:20-8](http://localhost:2300/file=C:/Users/USER/Desktop/ENTRETIENS%20PREMATURITE/CMA-DO/FOCUS%20GROUPE%20CMA-DO.mp3time=1220800) Person 9: Oui en salle nous avons des chauffages, à l'unité kangourou, on a le materiel de réanimation, mais ce n'est pas assez si nous avons 2 à 3 bébés. [00:20:33-6](http://localhost:2300/file=C:/Users/USER/Desktop/ENTRETIENS%20PREMATURITE/CMA-DO/FOCUS%20GROUPE%20CMA-DO.mp3time=1233600)

00:19:23-3 Interviewer: Quels types de services sont fournis aux nourrissons prématurés et à leurs mères le cas échéant au sein du CMA de Dô ? 00:19:33-7

00:19:33-7 Personne 6: À la mère et à l'enfant. La mère, ce sont les mêmes soins que les accouchements normaux, mais pour l'enfant, ce sont les soins du nouveau-né. Il y a le chauffage, il faut tout ce qui rentre dans les soins des prématurés, c'est-à-dire, il faut éviter le froid, c'est-à-dire que l'enfant ne soit pas refroidi. Si l'enfant a une détresse respiratoire, il faut l'intuber et puis oxygéner. 00:20:11-2

00:20:11-2 Interviewer: Est-ce que vous avez tout ce matériel ? 00:20:13-2

00:20:13-2 Personne 9: Il y en a, ce n'est pas assez mais il y en a. 00:20:16-7

00:20:16-7 Personne 6: Il semble qu'il y en a. 00:20:20-8

00:20:20-8 Personne 9: Oui, en salle nous avons des chauffages, à l'unité kangourou, on a le matériel de réanimation, mais ce n'est pas assez si nous avons 2 à 3 bébés. 00:20:33-6

**Interviewer (00:19:23-3):** "Can you describe the types of services provided to premature infants and their mothers at the CMA de Dô?"

**Person 6 (00:19:33-7):** "For mothers, we offer the same care as for normal deliveries. The care for premature infants, however, includes specific newborn care. This involves heating and other measures to prevent the baby from getting cold. In cases where the child experiences respiratory distress, intubation and oxygenation are necessary."

**Interviewer (00:20:11-2):** "Do you have all the necessary equipment for this?"

**Person 9 (00:20:13-2):** "We have some equipment, but it's not sufficient. There are heaters in the room and resuscitation equipment in the kangaroo unit, but it's inadequate for handling more than two or three babies."

**Person 6 (00:20:16-7):** "It seems that we have some equipment."

**Person 9 (00:20:20-8):** "Yes, we have the essential items like heaters and resuscitation equipment, but it's not enough for multiple cases simultaneously.

### Gynecologist 1

[00:12:01-9](http://localhost:2300/file=C:/Users/USER/Desktop/ENTRETIENS%20PREMATURITE/CMA-DO/GENYCOLOGUE%2001.mp3time=721900) Interviewer: Quel type de soins sont fournis aux nourrissons prématurés et à leurs mères? [00:12:09-3](http://localhost:2300/file=C:/Users/USER/Desktop/ENTRETIENS%20PREMATURITE/CMA-DO/GENYCOLOGUE%2001.mp3time=729300)

[00:12:09-3](http://localhost:2300/file=C:/Users/USER/Desktop/ENTRETIENS%20PREMATURITE/CMA-DO/GENYCOLOGUE%2001.mp3time=729300) Person 1: Pour la mère, il n'y a pas de soins particuliers, ce sont les mêmes soins du postpartum que nous dispensons. Chez l'enfant, les soins qu'on peut faire ici, il y a la protection thermique pour que l'enfant ne se refroidisse pas, il y a l'alimentation,

il y a certains qui ont déjà le reflet de succion, qui arrivent à sucer, d'autres n'ont pas encore le reflet de succion, donc au niveau de l'unité, on arrive à mettre des sondes pour faire un gavage, et comme ça on surveille la prise pondérale, la température des enfants. [00:12:44-5](http://localhost:2300/file=C:/Users/USER/Desktop/ENTRETIENS%20PREMATURITE/CMA-DO/GENYCOLOGUE%2001.mp3time=764500)

00:12:01-9 Interviewer: Quel type de soins sont fournis aux nourrissons prématurés et à leurs mères ? 00:12:09-3

00:12:09-3 Personne 1: Pour la mère, il n'y a pas de soins particuliers, ce sont les mêmes soins du post-partum que nous dispensons. Chez l'enfant, les soins qu'on peut faire ici, il y a la protection thermique pour que l'enfant ne se refroidisse pas, il y a l'alimentation. Il y a certains qui ont déjà le réflexe de succion, qui arrivent à sucer, d'autres n'ont pas encore le réflexe de succion, donc au niveau de l'unité, on arrive à mettre des sondes pour faire un gavage, et comme ça on surveille la prise pondérale, la température des enfants. 00:12:44-5

**Interviewer (00:12:01-9):** "Could you describe the type of care provided to premature infants and their mothers at your facility?"

**Person 1 (00:12:09-3):** "For mothers, the care is standard postpartum care, no different from what we offer to all mothers. However, for premature infants, the focus is on thermal protection to prevent them from getting cold. Nutrition is also crucial. Some premature babies have a developed sucking reflex and can feed on their own, while others don't. In such cases, we use feeding tubes for force-feeding. We closely monitor these infants for weight gain and temperature regulation.

### Gynecologist 2

[00:18:04-2](http://localhost:2300/file=C:/Users/USER/Desktop/ENTRETIENS%20PREMATURITE/CMA-DO/GENYCOLOGUE%2002.mp3time=1084200) Interviewer: Maintenant de façon globale. Quels sont les services qui sont fournis aux nourrissons prématurés et à leurs mères ? [00:18:14-7](http://localhost:2300/file=C:/Users/USER/Desktop/ENTRETIENS%20PREMATURITE/CMA-DO/GENYCOLOGUE%2002.mp3time=1094700)

[00:18:14-7](http://localhost:2300/file=C:/Users/USER/Desktop/ENTRETIENS%20PREMATURITE/CMA-DO/GENYCOLOGUE%2002.mp3time=1094700) Person 1: L'essentiel ce sont les conseils. Comme je l'ai dit au début, on met l'enfant dans une situation confortable, l'envelopper est assez chaudement, les vêtements chauds et de façon permanente. On va enseigner ça à la maman, on va enseigner ça au papa si il est à côté, on va même enseigner aux autres accompagnants, c'est à dire les grands-parents ou les tantes qui accompagnent l'enfant et sa maman. On leur enseigne que l'enfant doit être gardé au chaud de façon permanente. Et là l'unité kangourou est assez développée pour faire ça. Il y a même la façon de porter le bébé qui compte, généralement ils portent le bébé sur la poitrine toujours couvert, et on le met peau à peau avec sa maman ou alors l'individu qui le porte. Souvent, il peut s'agir des cas de césariennes où la maman n'est pas en mesure de porter l'enfant. Donc si c'est la grand-mère ou la tante ou un autre membre de la famille qui porte l'enfant, on met l'enfant en kangourou, c'est à dire que l'enfant, la tête couverte, les pieds couverts, on le met peau à peau contre celui qui le porte et on l'enveloppe d'un tissu de façon permanente. Et c'est seulement pendant la période de tétés, s'il ne s'agit pas de la mère, qu'on va dérouler l'enfant et le remettre à la maman pour la période de tétés. Et s'il s'agit de la mère qui porte l'enfant, ça veut dire que l'enfant reste dans sa position et il est alimenté en permanence. Donc ça permet de réchauffer l'enfant en continu avec la température corporelle, ça permet d'alimenter l'enfant de façon continuelle et en même temps il est gardé au chaud. Et ça, ça améliore nettement la survie. [00:19:52-4](http://localhost:2300/file=C:/Users/USER/Desktop/ENTRETIENS%20PREMATURITE/CMA-DO/GENYCOLOGUE%2002.mp3time=1192400)

[00:19:52-4](http://localhost:2300/file=C:/Users/USER/Desktop/ENTRETIENS%20PREMATURITE/CMA-DO/GENYCOLOGUE%2002.mp3time=1192400) Interviewer: D'accord. Et par rapport aux mères des enfants prématurés ? [00:19:54-4](http://localhost:2300/file=C:/Users/USER/Desktop/ENTRETIENS%20PREMATURITE/CMA-DO/GENYCOLOGUE%2002.mp3time=1194400)

[00:19:54-4](http://localhost:2300/file=C:/Users/USER/Desktop/ENTRETIENS%20PREMATURITE/CMA-DO/GENYCOLOGUE%2002.mp3time=1194400) Person 1: Donc la mère, ce sont les conseils liés à ce comportement, garder l'enfant au chaud, les conseils liés à l'hygiène corporelle et vestimentaire. Puisque pour faire ça, il faut que soi-même vous soyez propre, que vous ayez de la linge propre pour pouvoir couvrir l'enfant et que vous vous alimentez de façon convenable pour une production quantitative de lait maternel. [00:20:21-7](http://localhost:2300/file=C:/Users/USER/Desktop/ENTRETIENS%20PREMATURITE/CMA-DO/GENYCOLOGUE%2002.mp3time=1221700)

00:18:04-2 Interviewer: Maintenant, de façon globale, quels sont les services qui sont fournis aux nourrissons prématurés et à leurs mères ? 00:18:14-7

00:18:14-7 Personne 1: L'essentiel, ce sont les conseils. Comme je l'ai dit au début, on met l'enfant dans une situation confortable, on l'enveloppe assez chaudement, avec des vêtements chauds et de façon permanente. On va enseigner ça à la maman, on va enseigner ça au papa s'il est à côté, on va même enseigner aux autres accompagnants, c'est-à-dire les grands-parents ou les tantes qui accompagnent l'enfant et sa maman. On leur enseigne que l'enfant doit être gardé au chaud de façon permanente. Et là, l'unité kangourou est assez développée pour faire ça. Il y a même la façon de porter le bébé qui compte, généralement ils portent le bébé sur la poitrine, toujours couvert, et on le met peau à peau avec sa maman ou alors l'individu qui le porte. Souvent, il peut s'agir des cas de césariennes où la maman n'est pas en mesure de porter l'enfant. Donc si c'est la grand-mère ou la tante ou un autre membre de la famille qui porte l'enfant, on met l'enfant en kangourou, c'est-à-dire que l'enfant, la tête couverte, les pieds couverts, on le met peau à peau contre celui qui le porte et on l'enveloppe d'un tissu de façon permanente. Et c'est seulement pendant la période de tétées, s'il ne s'agit pas de la mère, qu'on va dérouler l'enfant et le remettre à la maman pour la période de tétées. Et s'il s'agit de la mère qui porte l'enfant, ça veut dire que l'enfant reste dans sa position et il est alimenté en permanence. Donc ça permet de réchauffer l'enfant en continu avec la température corporelle, ça permet d'alimenter l'enfant de façon continue et en même temps il est gardé au chaud. Et ça, ça améliore nettement la survie. 00:19:52-4

00:19:52-4 Interviewer: D'accord. Et par rapport aux mères des enfants prématurés ? 00:19:54-4

00:19:54-4 Personne 1: Donc pour la mère, ce sont les conseils liés à ce comportement, garder l'enfant au chaud, les conseils liés à l'hygiène corporelle et vestimentaire. Puisque pour faire ça, il faut que soi-même vous soyez propre, que vous ayez du linge propre pour pouvoir couvrir l'enfant et que vous vous alimentez de façon convenable pour une production quantitative de lait maternel. 00:20:21-7

**Interviewer (00:18:04-2):** "Could you tell us about the overall services provided to premature infants and their mothers at your facility?"

**Person 1 (00:18:14-7):** "The primary service we offer is guidance. Initially, we ensure the child is in a comfortable and warm environment, using warm clothes consistently. We teach these practices to the mother, and if present, the father, as well as other family members like grandparents or aunts accompanying the child. It's important that the child is kept warm at all times. Our kangaroo care unit is well-equipped for this. The method of carrying the baby, usually skin-to-skin on the chest and covered, is crucial. In cases like cesarean sections, where the mother can't carry the baby, another family member steps in. The baby, with a covered head and feet, is held skin-to-skin and wrapped in cloth. During feeding, if someone other than the mother is carrying the baby, we unwrap it for feeding by the mother. If the mother is carrying, the baby remains in position for continuous feeding. This approach ensures constant body warmth, continuous feeding, and warmth for the baby, significantly improving survival."

**Interviewer (00:19:52-4):** "And what about the services for the mothers of premature children?"

**Person 1 (00:19:54-4):** "For mothers, we focus on advice related to these practices, especially keeping the child warm and maintaining personal and clothing hygiene. The mother must be clean, with clean linen for covering the child, and she should eat well to ensure sufficient breast milk production.

### Chief physician of Do health district

[00:21:37-7](http://localhost:2300/file=C:/Users/USER/Desktop/ENTRETIENS%20PREMATURITE/CMA-DO/MCD-DO.mp3time=1297700) Interviewer: Vous avez parlé de gérer à partir d'un certain nombre de poids. Quel type de services et de soins, sont offerts à ces bébés que vous arrivez à gérer et à leur mère? Le cas échéant où vous avez à faire à ces bébés de faible poids? [00:21:50-6](http://localhost:2300/file=C:/Users/USER/Desktop/ENTRETIENS%20PREMATURITE/CMA-DO/MCD-DO.mp3time=1310600)

[00:21:50-6](http://localhost:2300/file=C:/Users/USER/Desktop/ENTRETIENS%20PREMATURITE/CMA-DO/MCD-DO.mp3time=1310600) Person 1: Oui, les services. Ce sont les conseils sur l'alimentation qu'on va offrir à l'enfant. Ce sont les conseils aussi à donner à la maman, elle-même, son alimentation pour le bébé. Ce sont les conseils en rapport avec l'hygiène de la maman et de l'enfant parce que l'enfant est fragile. La lingérie en passant partout. Donc, c'est ces apports qu'on fait. Et il y a un service qui est là avec du personnel qui est un peu aguéri. Une attachée en pédiatre avec des sages-femmes qui offrent ces soins-là. [00:22:33-9](http://localhost:2300/file=C:/Users/USER/Desktop/ENTRETIENS%20PREMATURITE/CMA-DO/MCD-DO.mp3time=1353900)

00:21:37-7 Interviewer: Vous avez parlé de gérer à partir d'un certain nombre de poids. Quel type de services et de soins sont offerts à ces bébés que vous arrivez à gérer et à leur mère, le cas échéant, où vous avez affaire à ces bébés de faible poids ? 00:21:50-6

00:21:50-6 Personne 1: Oui, les services. Ce sont les conseils sur l'alimentation qu'on va offrir à l'enfant. Ce sont aussi les conseils à donner à la maman, elle-même, sur son alimentation pour le bébé. Ce sont les conseils en rapport avec l'hygiène de la maman et de l'enfant, parce que l'enfant est fragile. La lingère en passant partout. Donc, c'est ces apports qu'on fait. Et il y a un service qui est là avec du personnel qui est un peu aguerri. Une attachée en pédiatrie avec des sages-femmes qui offrent ces soins-là. 00:22:33-9

**Interviewer (00:21:37-7):** You mentioned managing care based on certain weight criteria. Can you describe the types of services and care provided to low birth weight babies and their mothers in your facility?

**Person 1 (00:21:50-6):** Yes, the services primarily involve dietary advice for the child. We also offer guidance to the mother regarding her baby's nutrition. Additionally, we emphasize the importance of hygiene for both the mother and child, given the baby's fragility. We contribute by ensuring these aspects are taken care of. Our team includes experienced staff, including a pediatrician and midwives, who are dedicated to offering this specialized care.

### Manager for maternity care unit of Do medical center with surgical branch

[00:16:56-6](http://localhost:2300/file=C:/Users/USER/Desktop/ENTRETIENS%20PREMATURITE/CMA-DO/RESPONSABLE%20UNITE%20DE%20SOIN%20CMA-DO.mp3time=1016600) Interviewer: Mais le cas échéant, lorsque vous avez affaire à des prématurés au sein de votre centre de santé,quel type de soins sont fournis, donc, aux mères et aux enfants prématurés? [00:17:06-1](http://localhost:2300/file=C:/Users/USER/Desktop/ENTRETIENS%20PREMATURITE/CMA-DO/RESPONSABLE%20UNITE%20DE%20SOIN%20CMA-DO.mp3time=1026100)

[00:17:06-1](http://localhost:2300/file=C:/Users/USER/Desktop/ENTRETIENS%20PREMATURITE/CMA-DO/RESPONSABLE%20UNITE%20DE%20SOIN%20CMA-DO.mp3time=1026100) Person 1: Aux mères, bon, ce sont les mêmes soins qu'on offre aux autres femmes. Maintenant, au nouveau-né prématuré, c'est vrai qu'on a l'unité SMK qui est là, il arrive des fois, ce bébé transite par l'unité kangourou, qui offre peut-être les premiers soins rapidement, et on transfère. Voilà. Donc, je ne veux pas dire, il n'y a pas de soins spécifiques qu'on offre au niveau de la salle d'accouchement. C'est peut-être bien couvrir cet enfant-là. Donner les conseils à la mère ou aux accompagnantes, comment tenir ce nouveau-né-là. Donc, éviter surtout qu'il prenne froid et tout ça. [00:17:43-5](http://localhost:2300/file=C:/Users/USER/Desktop/ENTRETIENS%20PREMATURITE/CMA-DO/RESPONSABLE%20UNITE%20DE%20SOIN%20CMA-DO.mp3time=1063500)

00:16:56-6 Interviewer: Mais le cas échéant, lorsque vous avez affaire à des prématurés au sein de votre centre de santé, quel type de soins sont fournis, donc, aux mères et aux enfants prématurés ?

00:17:06-1 Person 1: Aux mères, bon, ce sont les mêmes soins qu'on offre aux autres femmes. Maintenant, au nouveau-né prématuré, c'est vrai qu'on a l'unité SMK qui est là, il arrive des fois, ce bébé transite par l'unité kangourou, qui offre peut-être les premiers soins rapidement, et on transfère. Voilà. Donc, je ne veux pas dire, il n'y a pas de soins spécifiques qu'on offre au niveau de la salle d'accouchement. C'est peut-être bien couvrir cet enfant-là. Donner les conseils à la mère ou aux accompagnantes, comment tenir ce nouveau-né-là. Donc, éviter surtout qu'il prenne froid et tout ça.

**Interviewer (00:16:56-6):** But if at all, when you're dealing with premature babies within your health center, what type of care is provided, then, to mothers and premature babies?

**Person 1 (00:17:06-1):** To mothers, well, it's the same care that we offer to other women. Now, for premature newborns, it's true that we have the SMK unit which is there, sometimes this baby passes through the kangaroo unit, which perhaps offers first aid quickly, and we transferred. So. So, I don't want to say, there is no specific care that we offer in the delivery room. Maybe it's a good cover for this child. Give advice to the mother or caregivers on how to hold this newborn. So, above all, avoid him getting cold and all that.

### Focus group of Colma1 health and social promotion center

[00:17:19-2](http://localhost:2300/file=C:/Users/USER/Desktop/ENTRETIENS%20PREMATURITE/CSPS-COLMA1/FOCUS%20GROUPE%20COLMA1.mp3time=1039200) Interviewer: Maintenant la question va porter aux services apportés aux nourrissons prématurés et à leurs mères,quels sont les types de services que vous apportez à ces derniers le cas écheant? [00:17:31-5](http://localhost:2300/file=C:/Users/USER/Desktop/ENTRETIENS%20PREMATURITE/CSPS-COLMA1/FOCUS%20GROUPE%20COLMA1.mp3time=1051500)

[00:17:31-5](http://localhost:2300/file=C:/Users/USER/Desktop/ENTRETIENS%20PREMATURITE/CSPS-COLMA1/FOCUS%20GROUPE%20COLMA1.mp3time=1051500) Person 5: Heu.. la maternité on est beaucoup hein..(rires) [00:17:38-3](http://localhost:2300/file=C:/Users/USER/Desktop/ENTRETIENS%20PREMATURITE/CSPS-COLMA1/FOCUS%20GROUPE%20COLMA1.mp3time=1058300)

[00:17:38-3](http://localhost:2300/file=C:/Users/USER/Desktop/ENTRETIENS%20PREMATURITE/CSPS-COLMA1/FOCUS%20GROUPE%20COLMA1.mp3time=1058300) Person 3: nous n'avons pas de soins adaptés à ça. Après l'accouchement nous protegeons correctement l'enfant, nous ne trainons même pas le temps avec l'enfant parce que la vie de l'enfant est en jeu. donc une fois que le bébé sort, on le pèse pour estimer son poids et on evacue rapidement parce que nous n'avons pas des medicaments adaptés, nous n'avons pas été formé, le plateau technique voilà donc on ne traine même pas avec l'enfant. [00:18:11-2](http://localhost:2300/file=C:/Users/USER/Desktop/ENTRETIENS%20PREMATURITE/CSPS-COLMA1/FOCUS%20GROUPE%20COLMA1.mp3time=1091200)

[00:18:11-2](http://localhost:2300/file=C:/Users/USER/Desktop/ENTRETIENS%20PREMATURITE/CSPS-COLMA1/FOCUS%20GROUPE%20COLMA1.mp3time=1091200) Interviewer: Ou est ce que vous evacuez l'enfant? [00:18:13-1](http://localhost:2300/file=C:/Users/USER/Desktop/ENTRETIENS%20PREMATURITE/CSPS-COLMA1/FOCUS%20GROUPE%20COLMA1.mp3time=1093100)

[00:18:13-1](http://localhost:2300/file=C:/Users/USER/Desktop/ENTRETIENS%20PREMATURITE/CSPS-COLMA1/FOCUS%20GROUPE%20COLMA1.mp3time=1093100) Person 3: on l'evacue au CHURSS. [00:18:18-3](http://localhost:2300/file=C:/Users/USER/Desktop/ENTRETIENS%20PREMATURITE/CSPS-COLMA1/FOCUS%20GROUPE%20COLMA1.mp3time=1098300)

00:17:19-2 Interviewer: Maintenant, la question va porter sur les services apportés aux nourrissons prématurés et à leurs mères. Quels sont les types de services que vous apportez à ces derniers le cas échéant ? 00:17:31-5

00:17:31-5 Personne 5: Heu... la maternité, on est beaucoup, hein... (rires) 00:17:38-3

00:17:38-3 Personne 3: Nous n'avons pas de soins adaptés à ça. Après l'accouchement, nous protégeons correctement l'enfant, nous ne traînons même pas le temps avec l'enfant parce que la vie de l'enfant est en jeu. Donc, une fois que le bébé sort, on le pèse pour estimer son poids et on évacue rapidement parce que nous n'avons pas des médicaments adaptés, nous n'avons pas été formés, le plateau technique, voilà, donc on ne traîne même pas avec l'enfant. 00:18:11-2

00:18:11-2 Interviewer: Où est-ce que vous évacuez l'enfant ? 00:18:13-1

00:18:13-1 Personne 3: On l'évacue au CHURSS. 00:18:18-3

**Interviewer (00:17:19-2):** I'd like to focus now on the services provided to premature infants and their mothers at your center. Could you tell us what types of services are offered?

**Person 5 (00:17:31-5):** [Hesitantly] Well, in terms of maternity care, we have quite a few... [laughs]

**Person 3 (00:17:38-3):** Actually, we don't have specialized care for premature infants. After delivery, our priority is to protect the child. We don't delay because their life is critical. We start by weighing the baby to estimate its weight and then quickly evacuate it. We lack suitable medications, training, and technical equipment, so we don't keep the child here for long.

**Interviewer (00:18:11-2):** Where do you evacuate the child to?

**Person 3 (00:18:13-1):** We evacuate them to CHURSS.

### Major of Colma1 health and social promotion center

[00:14:00-9](http://localhost:2300/file=C:/Users/USER/Desktop/ENTRETIENS%20PREMATURITE/CSPS-COLMA1/MAJOR%20CSPS%20COLMA1.MP3time=840900) Interviewer: quel type de services sont fournis au nourrisson prématuré et à leur mère le cas échéant? [00:14:05-3](http://localhost:2300/file=C:/Users/USER/Desktop/ENTRETIENS%20PREMATURITE/CSPS-COLMA1/MAJOR%20CSPS%20COLMA1.MP3time=845300)

[00:14:05-3](http://localhost:2300/file=C:/Users/USER/Desktop/ENTRETIENS%20PREMATURITE/CSPS-COLMA1/MAJOR%20CSPS%20COLMA1.MP3time=845300) Ahh le prématuré? [00:14:08-3](http://localhost:2300/file=C:/Users/USER/Desktop/ENTRETIENS%20PREMATURITE/CSPS-COLMA1/MAJOR%20CSPS%20COLMA1.MP3time=848300)

[00:14:08-3](http://localhost:2300/file=C:/Users/USER/Desktop/ENTRETIENS%20PREMATURITE/CSPS-COLMA1/MAJOR%20CSPS%20COLMA1.MP3time=848300) Interviewer: et la mère le cas échéant. [00:14:10-4](http://localhost:2300/file=C:/Users/USER/Desktop/ENTRETIENS%20PREMATURITE/CSPS-COLMA1/MAJOR%20CSPS%20COLMA1.MP3time=850400)

[00:14:10-4](http://localhost:2300/file=C:/Users/USER/Desktop/ENTRETIENS%20PREMATURITE/CSPS-COLMA1/MAJOR%20CSPS%20COLMA1.MP3time=850400) Person 1: d'accord, mais c'est ce qui veut dire comme nous sommes en milieu urbain ici déjà dans la prise en charge en tout cas, on va les orienter vers le niveau supérieur. Mais avant ça, il y a ce qu'on fait, c'est-à-dire la protection a chaud et pour envelopper l'enfant, il faut le mettre corps à corps avec la maman ou bien en tout cas pour que l'enfant ne soit pas exposé au refroidissement.Donc en général c'est vraiment de le couvrir à chaud et les metre peau à peau avec la maman, c'est ce que nous pouvons faire ici. Voilà, c'est ce que nous pouvons faire ici.En général, souvent vous allez voir qu' il y a certains prématuré qui n'ont pas ce qu'on appelle la succion correcte, donc ça fait qu'ils ne pas prendre le sien d'abord, donc c'est quelques glucosés qu'on met au niveau de la bouche, pour qu'ils sucent, afin donc d'éviter l'hypoglycémie. Voilà c'est ce qu'on fait ici en général, c'est ce qu'on peut faire, mais dans tous les cas, l'enfant est referré vers les structures supérieures. [00:15:13-0](http://localhost:2300/file=C:/Users/USER/Desktop/ENTRETIENS%20PREMATURITE/CSPS-COLMA1/MAJOR%20CSPS%20COLMA1.MP3time=913000)

00:14:00-9 Interviewer: Quel type de services sont fournis au nourrisson prématuré et à leur mère le cas échéant ? 00:14:05-3

00:14:05-3 Ah, le prématuré ? 00:14:08-3

00:14:08-3 Interviewer: Et la mère le cas échéant. 00:14:10-4

00:14:10-4 Personne 1: D'accord, mais c'est ce qui veut dire comme nous sommes en milieu urbain ici, déjà dans la prise en charge, en tout cas, on va les orienter vers le niveau supérieur. Mais avant ça, il y a ce qu'on fait, c'est-à-dire la protection à chaud et pour envelopper l'enfant, il faut le mettre corps à corps avec la maman, ou bien en tout cas pour que l'enfant ne soit pas exposé au refroidissement. Donc en général, c'est vraiment de le couvrir à chaud et les mettre peau à peau avec la maman, c'est ce que nous pouvons faire ici. Voilà, c'est ce que nous pouvons faire ici. En général, souvent vous allez voir qu'il y a certains prématurés qui n'ont pas ce qu'on appelle la succion correcte, donc ça fait qu'ils ne peuvent pas prendre le sein d'abord, donc c'est quelques glucosés qu'on met au niveau de la bouche, pour qu'ils sucent, afin donc d'éviter l'hypoglycémie. Voilà, c'est ce qu'on fait ici en général, c'est ce qu'on peut faire, mais dans tous les cas, l'enfant est référé vers les structures supérieures. 00:15:13-0

**Interviewer (00:14:00-9):** "Could you describe the type of services provided to premature infants and their mothers at your facility?"

**Person 1 (00:14:05-3):** "The premature one?"

**Interviewer (00:14:08-3):** "Yes, and the mother, if applicable."

**Person 1 (00:14:10-4):** "Right. Since we're in an urban environment, our primary approach is to refer them to a higher level of care. However, before that, we focus on essential immediate care. This includes thermal protection – we ensure the child is wrapped warmly and placed skin-to-skin with the mother to prevent exposure to cold. That's our general practice. Often, some premature infants struggle with proper sucking reflex, so they can't breastfeed immediately. In such cases, we give them a little glucose orally to stimulate sucking and prevent hypoglycemia. These are the primary services we provide here, but ultimately, we refer the child to more specialized facilities.

### Maternity manager of Colma1 health and social promotion center

[00:22:40-9](http://localhost:2300/file=C:/Users/USER/Desktop/ENTRETIENS%20PREMATURITE/CSPS-COLMA1/RESPONSABLE%20MATERNITE-COLMA1.mp3time=1360900) Interviewer: le cas échéant comment est ce que vous vous y prenez pour la gestion de ces cas? [00:22:47-7](http://localhost:2300/file=C:/Users/USER/Desktop/ENTRETIENS%20PREMATURITE/CSPS-COLMA1/RESPONSABLE%20MATERNITE-COLMA1.mp3time=1367700)

[00:22:47-7](http://localhost:2300/file=C:/Users/USER/Desktop/ENTRETIENS%20PREMATURITE/CSPS-COLMA1/RESPONSABLE%20MATERNITE-COLMA1.mp3time=1367700) Person 1: Pour la gestion. D'abord, généralement même à petite dilatation, nous, nous la réferons. Mais lorsque ça arrive et qu'on ne peut plus, normalement,c'est celestène qu'on devrait faire même à la mère. Je pense que c'est 12 milligrammes, non ? Et 12 heures de temps après, on doit faire pour vraiment les poumons,mais souvent, les femmes qui viennent dans ces cas-là, viennent, par exemple, à dilatation complète.Une fois seulement que c'est une grande prématurité, on sait bien que la prise en charge même au CHURSS sera assez difficile, mais on va mettre cet enfant dans les conditions avec sa mère pour vraiment évacuer d'urgence. Mais lorsque c'est une petite prématurité, peut- être à 36 semaines, quelques jours et que vraiment les réflexes sont bons, surtout le réflexe de succion, peut- être on va vraiment observer cet enfant. On va peut- être essayer d'aider la mère pour l'alimentation, parce qu'il faut que l'enfant prenne quelque chose, prenne le lait surtout et puis éviter l'hypoglycemie, vraiment l'envelopper. Donner les conseils nécessaires, vraiment. Mais si on voit qu'il y a un petit problème seulement, cet enfant sera évacué.Pas référé, mais évacué. Et on va demander de ne pas laver cet enfant. Le bain même des enfants, en général, que ça soit un enfant à terme, c'est jusqu'à 24 heures après l'accouchement. Cet enfant, on va tout faire pour ne pas le laver. C'est peut- être nettoyer les selles, en tout cas, l'envelopper, surtout les chaussons et les bonnets, c'est au premier moment. Dès qu'on a une femme en salle, c'est ce qu'on demande. Comme on est à côté du marché, si ce n'est pas la nuit, ils sortent pour payer. [00:25:04-7](http://localhost:2300/file=C:/Users/USER/Desktop/ENTRETIENS%20PREMATURITE/CSPS-COLMA1/RESPONSABLE%20MATERNITE-COLMA1.mp3time=1504700)

[00:25:04-7](http://localhost:2300/file=C:/Users/USER/Desktop/ENTRETIENS%20PREMATURITE/CSPS-COLMA1/RESPONSABLE%20MATERNITE-COLMA1.mp3time=1504700) Interviewer: Quand vous dites que lorsque vous sentez qu'il y a un petit problème au niveau l'enfant vous l'évacueri, vous faites allusion à quel type de problème ? [00:25:12-2](http://localhost:2300/file=C:/Users/USER/Desktop/ENTRETIENS%20PREMATURITE/CSPS-COLMA1/RESPONSABLE%20MATERNITE-COLMA1.mp3time=1512200)

[00:25:12-2](http://localhost:2300/file=C:/Users/USER/Desktop/ENTRETIENS%20PREMATURITE/CSPS-COLMA1/RESPONSABLE%20MATERNITE-COLMA1.mp3time=1512200) Person 1: Par exemple, les réflexes, ça veut dire que l'enfant ne peut pas parler. Si les réflexes archaiques ne sont pas là, surtout si la succion n'est pas là, est- ce qu'on peut faire quelque chose ? Et l'enfant n'a pas crié. Comme je parle de parler des scores d'Avgar qui est bon, les scores d'Avgar est bon, les réflexes sont là, l'enfant arrive à têter, pour un petit prématuré, ça veut dire l'enfant même, en tout cas, c'est quelques jours, deux jours peut- être qui manquaient pour que l'enfant soit vraiment à terme, 37 semaines là. Cet enfant-là, sûrement, peut- être, c'est si ça va, on peut garder. Le reste, on envoie. [00:25:50-0](http://localhost:2300/file=C:/Users/USER/Desktop/ENTRETIENS%20PREMATURITE/CSPS-COLMA1/RESPONSABLE%20MATERNITE-COLMA1.mp3time=1550000)

[00:25:50-0](http://localhost:2300/file=C:/Users/USER/Desktop/ENTRETIENS%20PREMATURITE/CSPS-COLMA1/RESPONSABLE%20MATERNITE-COLMA1.mp3time=1550000) Interviewer: Et par rapport à la mère ? [00:25:52-3](http://localhost:2300/file=C:/Users/USER/Desktop/ENTRETIENS%20PREMATURITE/CSPS-COLMA1/RESPONSABLE%20MATERNITE-COLMA1.mp3time=1552300)

[00:25:52-3](http://localhost:2300/file=C:/Users/USER/Desktop/ENTRETIENS%20PREMATURITE/CSPS-COLMA1/RESPONSABLE%20MATERNITE-COLMA1.mp3time=1552300) Person 1: À la mère, oui. Puisque lorsqu'il y a accouchement prématuré, c'est qu'il y a un problème. Il faut décèler le problème et prendre en charge. Beaucoup de choses peuvent faire que la femme fasse un accouchement prématuré. D'abord, les problèmes sociaux, culturels sont là. Je commence par là. Peut- être une grossesse avant, 18 ans ou bien supérieure à 35 ans. Une femme célibataire. Voilà. Et grossesse illégitime. Voilà. Et grossesse non désirée. Vous voyez tout ce qui est là. [00:26:38-7](http://localhost:2300/file=C:/Users/USER/Desktop/ENTRETIENS%20PREMATURITE/CSPS-COLMA1/RESPONSABLE%20MATERNITE-COLMA1.mp3time=1598700)

[00:26:38-7](http://localhost:2300/file=C:/Users/USER/Desktop/ENTRETIENS%20PREMATURITE/CSPS-COLMA1/RESPONSABLE%20MATERNITE-COLMA1.mp3time=1598700) Interviewer: Grossesse illégitime, pourquoi ? [00:26:41-5](http://localhost:2300/file=C:/Users/USER/Desktop/ENTRETIENS%20PREMATURITE/CSPS-COLMA1/RESPONSABLE%20MATERNITE-COLMA1.mp3time=1601500)

[00:26:41-5](http://localhost:2300/file=C:/Users/USER/Desktop/ENTRETIENS%20PREMATURITE/CSPS-COLMA1/RESPONSABLE%20MATERNITE-COLMA1.mp3time=1601500) Person 1: Bon, je vais dire quoi ? Peut- être, c'est quelqu'une qui est chez sa tante et le mari de la tante sort avec elle. La seste ? Çela peut ne pas être direct. Peut- être tu as pris une fille au village pour t'aider. Ok. Dans la grande famille lointaine. Et la fille vient chez toi, ça veut dire, ce n'est pas normal qu'elle tombe enceinte de ton mari. Ok. C'est truc, elle est là, ça veut dire, je vais dire quoi ? Et interruption volontaire de la grossesse selon la loi n'est pas appliquée. Et voilà, cet enfant. Tout ça, ça fait amener quelqu'un à accoucher prématurément. Ça, c'est ainsi. On prend côté maternel toujours. Et les causes locales, ça peut être beaucoup de choses. Voilà, ça peut être malformation utérine. Voilà, ça peut être quoi encore ? Fibrome. Un fibrome peut amener une femme à accoucher prématurément. Il y a beaucoup de choses dedans. Et encore, les infections, quand on prend de manière générale les infections, surtout les infections urinaires , l'accès palustre, l'hystériose, il y a beaucoup de maladies qui peuvent amener, le VIH même, diabète.Je veux dire quoi ? Beaucoup de pathologies peuvent amener la femme à faire un accouchement. Donc, il faut vraiment faire l'interrogatoire, se focaliser vraiment sur cette femme- là, connaître le problème et l'aider vraiment à corriger. Ça peut amener même les béances. [00:28:30-8](http://localhost:2300/file=C:/Users/USER/Desktop/ENTRETIENS%20PREMATURITE/CSPS-COLMA1/RESPONSABLE%20MATERNITE-COLMA1.mp3time=1710800)

00:22:40-9 Interviewer: Le cas échéant, comment est-ce que vous vous y prenez pour la gestion de ces cas ?

00:22:47-7 Person 1: Pour la gestion. D'abord, généralement même à petite dilatation, nous, nous la référons. Mais lorsque ça arrive et qu'on ne peut plus, normalement, c'est célestène qu'on devrait faire même à la mère. Je pense que c'est 12 milligrammes, non ? Et 12 heures de temps après, on doit faire pour vraiment les poumons, mais souvent, les femmes qui viennent dans ces cas-là, viennent, par exemple, à dilatation complète. Une fois seulement que c'est une grande prématurité, on sait bien que la prise en charge même au CHURSS sera assez difficile, mais on va mettre cet enfant dans les conditions avec sa mère pour vraiment évacuer d'urgence. Mais lorsque c'est une petite prématurité, peut-être à 36 semaines, quelques jours et que vraiment les réflexes sont bons, surtout le réflexe de succion, peut-être on va vraiment observer cet enfant. On va peut-être essayer d'aider la mère pour l'alimentation, parce qu'il faut que l'enfant prenne quelque chose, prenne le lait surtout et puis éviter l'hypoglycémie, vraiment l'envelopper. Donner les conseils nécessaires, vraiment. Mais si on voit qu'il y a un petit problème seulement, cet enfant sera évacué. Pas référé, mais évacué. Et on va demander de ne pas laver cet enfant. Le bain même des enfants, en général, que ça soit un enfant à terme, c'est jusqu'à 24 heures après l'accouchement. Cet enfant, on va tout faire pour ne pas le laver. C'est peut-être nettoyer les selles, en tout cas, l'envelopper, surtout les chaussons et les bonnets, c'est au premier moment. Dès qu'on a une femme en salle, c'est ce qu'on demande. Comme on est à côté du marché, si ce n'est pas la nuit, ils sortent pour payer.

00:25:04-7 Interviewer: Quand vous dites que lorsque vous sentez qu'il y a un petit problème au niveau de l'enfant vous l'évacuez, vous faites allusion à quel type de problème ?

00:25:12-2 Person 1: Par exemple, les réflexes, ça veut dire que l'enfant ne peut pas parler. Si les réflexes archaïques ne sont pas là, surtout si la succion n'est pas là, est-ce qu'on peut faire quelque chose ? Et l'enfant n'a pas crié. Comme je parle des scores d'Apgar qui est bon, les scores d'Apgar sont bons, les réflexes sont là, l'enfant arrive à téter, pour un petit prématuré, ça veut dire l'enfant même, en tout cas, c'est quelques jours, deux jours peut-être qui manquaient pour que l'enfant soit vraiment à terme, 37 semaines là. Cet enfant-là, sûrement, peut-être, c'est si ça va, on peut garder. Le reste, on envoie.

00:25:50-0 Interviewer: Et par rapport à la mère ?

00:25:52-3 Person 1: À la mère, oui. Puisque lorsqu'il y a accouchement prématuré, c'est qu'il y a un problème. Il faut déceler le problème et prendre en charge. Beaucoup de choses peuvent faire que la femme fasse un accouchement prématuré. D'abord, les problèmes sociaux, culturels sont là. Je commence par là. Peut-être une grossesse avant, 18 ans ou bien supérieure à 35 ans. Une femme célibataire. Voilà. Et grossesse illégitime. Voilà. Et grossesse non désirée. Vous voyez tout ce qui est là.

00:26:38-7 Interviewer: Grossesse illégitime, pourquoi ?

00:26:41-5 Person 1: Bon, je vais dire quoi ? Peut-être, c'est quelqu'une qui est chez sa tante et le mari de la tante sort avec elle. La sieste ? Cela peut ne pas être direct. Peut-être tu as pris une fille au village pour t'aider. Ok. Dans la grande famille lointaine. Et la fille vient chez toi, ça veut dire, ce n'est pas normal qu'elle tombe enceinte de ton mari. Ok. C'est truc, elle est là, ça veut dire, je vais dire quoi ? Et interruption volontaire de la grossesse selon la loi n'est pas appliquée. Et voilà, cet enfant. Tout ça, ça fait amener quelqu'un à accoucher prématurément. Ça, c'est ainsi. On prend côté maternel toujours. Et les causes locales, ça peut être beaucoup de choses. Voilà, ça peut être malformation utérine. Voilà, ça peut être quoi encore ? Fibrome. Un fibrome peut amener une femme à accoucher prématurément. Il y a beaucoup de choses dedans. Et encore, les infections, quand on prend de manière générale les infections, surtout les infections urinaires, l'accès palustre, l'hystériose, il y a beaucoup de maladies qui peuvent amener, le VIH même, diabète. Je veux dire quoi ? Beaucoup de pathologies peuvent amener la femme à faire un accouchement. Donc, il faut vraiment faire l'interrogatoire, se focaliser vraiment sur cette femme-là, connaître le problème et l'aider vraiment à corriger. Ça peut amener même les béances.

**Interviewer (00:22:40-9):** If so, how do you go about handling these cases?

**Person 1 (00:22:47-7):** For management. First, generally even at small dilation, we refer to it. But when that happens and we can't do it anymore, normally, it's Célestène that we should do even to the mother. I think it's 12 milligrams, right? And 12 hours later, we have to really do the lungs, but often, women who come in these cases, come, for example, to full dilation. Once it is extremely prematurity, we know that the care even at the CHURSS will be quite difficult, but we will put this child in the conditions with his mother to really evacuate urgently. But when it is a little prematurity, perhaps at 36 weeks, a few days and the reflexes are really good, especially the sucking reflex, perhaps we will really observe this child. We will perhaps try to help the mother with feeding, because the child needs to take something, especially take milk and then avoid hypoglycemia, really wrap him up. Give the necessary advice, really. But if we see that there is only a small problem, this child will be evacuated. Not referred, but evacuated. And we are going to ask not to bathe this child. The very bathing of children, in general, whether it is a full-term child, is up to 24 hours after delivery. This child, we will do everything not to wash him. Maybe it's cleaning the stools, in any case, wrapping it, especially the slippers and hats, it's at the first moment. As soon as we have a woman in the room, that's what we ask for. As we are next to the market, if it is not night, they come out to pay.

**Interviewer (00:25:04-7):** When you say that when you feel that there is a small problem with the child you evacuate it, what type of problem are you referring to?

**Person 1 (00:25:12-2):** For example, reflexes mean that the child cannot speak. If the archaic reflexes are not there, especially if the suction is not there, can we do anything? And the child didn't cry. As I'm talking about the Apgar scores which are good, the Apgar scores are good, the reflexes are there, the child is able to breastfeed, for a small premature baby, that means the child himself, in any case , it's a few days, maybe two days that were missing for the child to really be full term, 37 weeks there. This child, surely, perhaps, if it goes well, we can keep it. The rest we send.

**Interviewer (00:25:50-0):** And in relation to the mother?

**Person 1 (00:25:52-3):** To the mother, yes. Because when there is premature birth, there is a problem. The problem must be identified and taken care of. Many things can cause a woman to have a premature birth. First, the social and cultural problems are there. I start there. Perhaps a pregnancy before, 18 years old or well over 35 years old. A single woman. So. And illegitimate pregnancy. So. And unwanted pregnancy. You see everything that is there.

**Interviewer (00:26:38-7):** Illegitimate pregnancy, why?

**Person 1 (00:26:41-5):** Okay, what am I going to say? Maybe it's someone who is at her aunt's house and the aunt's husband is going out with her. Nap ? It may not be direct. Maybe you took a girl from the village to help you. Ok. In the big distant family. And the girl comes to your house, that means, it's not normal for her to get pregnant by your husband. Ok. That's it, she's here, that means, what am I going to say? And voluntary termination of pregnancy according to the law is not applied. And there you have it, this child. All of this causes someone to give birth prematurely. That's how it is. We always take the maternal side. And local causes can be many things. There you go, it could be a uterine malformation. There you go, what else could it be? Fibroma. A fibroid can cause a woman to give birth prematurely. There's a lot in there. And again, infections, when we take infections in general, especially urinary infections, malaria, hysteriosis, there are many diseases that can lead to, even HIV, diabetes. What do I mean ? Many pathologies can lead a woman to give birth. So, you really have to do the questioning, really focus on this woman, know the problem and really help her to correct it. It can even lead to gaping.

### Focus group of Farakan health and social promotion center

[00:29:52-6](http://localhost:2300/file=C:/Users/USER/Desktop/ENTRETIENS%20PREMATURITE/CSPS_FARAKAN/FOCUS_GROUPE-CSPS_FARAKAN.MP3time=1792600) Interviewer: Quand ces accouchements prématurés arrivent au sein de votre CSPS, Quels sont les types de services qui sont fournis aux nourrissons prématurés et à leur mère ? [00:30:07-3](http://localhost:2300/file=C:/Users/USER/Desktop/ENTRETIENS%20PREMATURITE/CSPS_FARAKAN/FOCUS_GROUPE-CSPS_FARAKAN.MP3time=1807300)

[00:30:07-3](http://localhost:2300/file=C:/Users/USER/Desktop/ENTRETIENS%20PREMATURITE/CSPS_FARAKAN/FOCUS_GROUPE-CSPS_FARAKAN.MP3time=1807300) Person 3: C'est l'envol et l'enveloppement chaud et sec.La vitamine K1.Voilà, c'est tout ce que nous faisons au niveau du nouveau-né, pour la maman, ce sont les autres soins comme aux femmes ordinaires.C'est le contact peau à peau que nous priorisons surtout même lors du transport pour l'évacuation. [00:30:45-0](http://localhost:2300/file=C:/Users/USER/Desktop/ENTRETIENS%20PREMATURITE/CSPS_FARAKAN/FOCUS_GROUPE-CSPS_FARAKAN.MP3time=1845000)

00:29:52-6 Interviewer: Quand ces accouchements prématurés arrivent au sein de votre CSPS, quels sont les types de services qui sont fournis aux nourrissons prématurés et à leur mère ? 00:30:07-3

00:30:07-3 Personne 3: C'est l'envol et l'enveloppement chaud et sec. La vitamine K1. Voilà, c'est tout ce que nous faisons au niveau du nouveau-né, pour la maman, ce sont les autres soins comme aux femmes ordinaires. C'est le contact peau à peau que nous priorisons surtout, même lors du transport pour l'évacuation. 00:30:45-0

**Interviewer (00:29:52-6):** "When you encounter premature births at your CSPS, what specific types of services do you provide for the premature infants and their mothers?"

**Person 3 (00:30:07-3):** "For the infants, our primary actions include immediate assessment and a warm, dry wrap. We also administer Vitamin K1. That's essentially all we do for the newborn. As for the mothers, they receive the standard care given to all women in our facility. We place a strong emphasis on skin-to-skin contact, which is especially important during transport for evacuation."

### Major of Farakan health and social promotion center

[00:11:21-6](http://localhost:2300/file=C:/Users/USER/Desktop/ENTRETIENS%20PREMATURITE/CSPS_FARAKAN/MAJOR%20FARAKAN.MP3time=681600) Interviewer: Quels types de services sont fournis aux nourrissons prématurés et à leurs mères le cas écheant? [00:11:28-5](http://localhost:2300/file=C:/Users/USER/Desktop/ENTRETIENS%20PREMATURITE/CSPS_FARAKAN/MAJOR%20FARAKAN.MP3time=688500)

[00:11:28-5](http://localhost:2300/file=C:/Users/USER/Desktop/ENTRETIENS%20PREMATURITE/CSPS_FARAKAN/MAJOR%20FARAKAN.MP3time=688500) Person 1: Ici, après les premiers soins, generalement, on réfère le bébé.La maman reste ici mais on refère le bébé en attendant que notre surveillance prenne fin. [00:11:41-8](http://localhost:2300/file=C:/Users/USER/Desktop/ENTRETIENS%20PREMATURITE/CSPS_FARAKAN/MAJOR%20FARAKAN.MP3time=701800)

[00:11:41-8](http://localhost:2300/file=C:/Users/USER/Desktop/ENTRETIENS%20PREMATURITE/CSPS_FARAKAN/MAJOR%20FARAKAN.MP3time=701800) Interviewer: Et lors de la surveillance qu'est ce qui est fait? [00:11:44-4](http://localhost:2300/file=C:/Users/USER/Desktop/ENTRETIENS%20PREMATURITE/CSPS_FARAKAN/MAJOR%20FARAKAN.MP3time=704400)

[00:11:44-4](http://localhost:2300/file=C:/Users/USER/Desktop/ENTRETIENS%20PREMATURITE/CSPS_FARAKAN/MAJOR%20FARAKAN.MP3time=704400) Person 1: Le nouveau né, on le nettoie , on le met à chaud.S'il peut têter on commence la mise au sein, en attendant de l'acheminer à l'echelon superieur. [00:11:58-6](http://localhost:2300/file=C:/Users/USER/Desktop/ENTRETIENS%20PREMATURITE/CSPS_FARAKAN/MAJOR%20FARAKAN.MP3time=718600)

00:11:21-6 Interviewer: Quels types de services sont fournis aux nourrissons prématurés et à leurs mères le cas échéant ? 00:11:28-5

00:11:28-5 Personne 1: Ici, après les premiers soins, généralement, on réfère le bébé. La maman reste ici mais on réfère le bébé en attendant que notre surveillance prenne fin. 00:11:41-8

00:11:41-8 Interviewer: Et lors de la surveillance, qu'est-ce qui est fait ? 00:11:44-4

00:11:44-4 Personne 1: Le nouveau-né, on le nettoie, on le met à chaud. S'il peut téter, on commence la mise au sein, en attendant de l'acheminer à l'échelon supérieur. 00:11:58-6

**Interviewer (00:11:21-6):** "Could you tell us about the types of services provided to premature infants and their mothers at your facility?"

**Person 1 (00:11:28-5):** "In our facility, after providing first aid, we generally refer the premature baby to a higher level of care. The mother stays here under our surveillance until it's appropriate for her to leave."

**Interviewer (00:11:41-8):** "What procedures are followed during this period of surveillance?"

**Person 1 (00:11:44-4):** "For the newborn, we start by cleaning and warming them up. If the baby is capable of breastfeeding, we initiate that process. Meanwhile, we prepare for the baby's transfer to a facility with more specialized care

### Maternity manager of Farakan health and social promotion center

[00:18:04-9](http://localhost:2300/file=C:/Users/USER/Desktop/ENTRETIENS%20PREMATURITE/CSPS_FARAKAN/RESPONSABLE%20MATERNITE%20FARAKAN.MP3time=1084900) Interviewer: Le cas échéant, s'il y a au sein de votre centre de santé une naissance prématurée, quels type de service sont sont fournis au nourrissons ? [00:18:23-2](http://localhost:2300/file=C:/Users/USER/Desktop/ENTRETIENS%20PREMATURITE/CSPS_FARAKAN/RESPONSABLE%20MATERNITE%20FARAKAN.MP3time=1103200)

[00:18:23-2](http://localhost:2300/file=C:/Users/USER/Desktop/ENTRETIENS%20PREMATURITE/CSPS_FARAKAN/RESPONSABLE%20MATERNITE%20FARAKAN.MP3time=1103200) Person 1: Après la naissance, en tout cas géneralement nous ne prenons pas en charge.Si c'est un cas de grande prematurité nous evacuons parce que chez nous ici nous ne pouvons pas prendre en charge. par contre il y a des prématurés de moins de 2kg500 (2kg200 ou 2kg100) quand on voit, ça va. se sont des enfants avec les reflexes et ses enfants nous pouvons les garder et conseiller la maman sur son entretien, ne pas l'exposer au vent, il faut que cet enfant soit bien protègé.Maintenant en dessous de 2 kg, avec une detresse respiratoire, nous ne pouvons pas gerer ses cas ici. [00:19:34-8](http://localhost:2300/file=C:/Users/USER/Desktop/ENTRETIENS%20PREMATURITE/CSPS_FARAKAN/RESPONSABLE%20MATERNITE%20FARAKAN.MP3time=1174800)

[00:19:34-8](http://localhost:2300/file=C:/Users/USER/Desktop/ENTRETIENS%20PREMATURITE/CSPS_FARAKAN/RESPONSABLE%20MATERNITE%20FARAKAN.MP3time=1174800) Interviewer: Lorsque vous parlez de grand prématuré, vous faites allusion à quel type de prématuré? [00:19:41-1](http://localhost:2300/file=C:/Users/USER/Desktop/ENTRETIENS%20PREMATURITE/CSPS_FARAKAN/RESPONSABLE%20MATERNITE%20FARAKAN.MP3time=1181100)

[00:19:41-1](http://localhost:2300/file=C:/Users/USER/Desktop/ENTRETIENS%20PREMATURITE/CSPS_FARAKAN/RESPONSABLE%20MATERNITE%20FARAKAN.MP3time=1181100) Person 1: En tout cas les moins de 1kg800, meme 1kg 800 déjà c'est un grand prematuré.il y a même des prématurés de 1kg. Il nous ait arrivé d'avoir des jumeaux de 800g et 900g et nous les avons referés à l'hôpital et cela n'a pas été facile je pense qu'il y a un qui est décedé par la suite, c'elle qui est restée, jusqu'à 2 ans elle ne pouvait pas s'assoire donc c'etait vraiment une charge pour les parents. [00:20:26-5](http://localhost:2300/file=C:/Users/USER/Desktop/ENTRETIENS%20PREMATURITE/CSPS_FARAKAN/RESPONSABLE%20MATERNITE%20FARAKAN.MP3time=1226500)

00:18:04-9 Interviewer: Le cas échéant, s'il y a au sein de votre centre de santé une naissance prématurée, quels types de services sont fournis au nourrisson ? 00:18:23-2

00:18:23-2 Person 1: Après la naissance, en tout cas généralement, nous ne prenons pas en charge. Si c'est un cas de grande prématurité, nous évacuons parce que chez nous ici nous ne pouvons pas prendre en charge. Par contre, il y a des prématurés de moins de 2 kg 500 (2 kg 200 ou 2 kg 100) quand on voit, ça va. Ce sont des enfants avec les réflexes, et ces enfants nous pouvons les garder et conseiller la maman sur son entretien, ne pas l'exposer au vent, il faut que cet enfant soit bien protégé. Maintenant en dessous de 2 kg, avec une détresse respiratoire, nous ne pouvons pas gérer ces cas ici. 00:19:34-8

00:19:34-8 Interviewer: Lorsque vous parlez de grand prématuré, vous faites allusion à quel type de prématuré ? 00:19:41-1

00:19:41-1 Person 1: En tout cas, les moins de 1 kg 800, même 1 kg 800 déjà c'est un grand prématuré. Il y a même des prématurés de 1 kg. Il nous est arrivé d'avoir des jumeaux de 800 g et 900 g et nous les avons référés à l'hôpital, et cela n'a pas été facile. Je pense qu'il y en a un qui est décédé par la suite, celle qui est restée, jusqu'à 2 ans, elle ne pouvait pas s'asseoir, donc c'était vraiment une charge pour les parents. 00:20:26-5

**Interviewer (00:18:04-9):** If applicable, if there is a premature birth in your health center, what types of services are provided to the infant?

**Person 1 (00:18:23-2):** After birth, generally speaking, we don't take care of it. If it is a case of extreme prematurity, we evacuate because at home here we cannot provide care. On the other hand, there are premature babies weighing less than 2 kg 500 (2 kg 200 or 2 kg 100) when we see, they are fine. These are children with reflexes, and these children we can keep them and advise the mother on their care, not expose them to the wind, this child must be well protected. Now below 2 kg, with respiratory distress, we cannot manage these cases here.

**Interviewer (00:19:34-8):** When you talk about very premature, what type of premature are you referring to?

**Person 1 (00:19:41-1):** In any case, those under 1 kg 800, even 1 kg 800 are already very premature. There are even premature babies weighing 1 kg. We happened to have twins weighing 800g and 900g and we referred them to the hospital, and it wasn't easy. I think there was one who died afterwards, the one who stayed, until she was 2 years old, she couldn't sit up, so it was really a burden for the parents.

# 9. Health risks in premature infants

### Focus group of Accart-ville urban medical center

[00:20:00-5](http://localhost:2300/file=C:/Users/USER/Desktop/ENTRETIENS%20PREMATURITE/CSPS-ACCART-VILLE/FOCUS%20GROUPE%20ACCART-VILLE-BON.mp3time=1200500)Interviewer: nous pouvons poursuivre, maintenant, quels sont les risques sanitaires particuliers auxquels les nourrissons prématurés sont confrontés à milieu urbain au Burkina Faso? [00:20:40-5](http://localhost:2300/file=C:/Users/USER/Desktop/ENTRETIENS%20PREMATURITE/CSPS-ACCART-VILLE/FOCUS%20GROUPE%20ACCART-VILLE-BON.mp3time=1240500)

[00:20:40-5](http://localhost:2300/file=C:/Users/USER/Desktop/ENTRETIENS%20PREMATURITE/CSPS-ACCART-VILLE/FOCUS%20GROUPE%20ACCART-VILLE-BON.mp3time=1240500) Interviewer: nous allons reformuler la question, de façon géneral, quels sont les risques sanitaires particuliers auxquels les nourrissons prématurés sont confrontés en milieu urbain au Burkina Faso? [00:20:57-6](http://localhost:2300/file=C:/Users/USER/Desktop/ENTRETIENS%20PREMATURITE/CSPS-ACCART-VILLE/FOCUS%20GROUPE%20ACCART-VILLE-BON.mp3time=1257600)

[00:20:57-6](http://localhost:2300/file=C:/Users/USER/Desktop/ENTRETIENS%20PREMATURITE/CSPS-ACCART-VILLE/FOCUS%20GROUPE%20ACCART-VILLE-BON.mp3time=1257600)Person 7: moi je me dit que ce sont les infections parce que c'est ce qui joue sur beaucoup de prematurés. si les prématurés ne sont pas bien entretenus cela ouvre les portes aux maladies et géneralement les enfants ne survivent pas [00:21:21-6](http://localhost:2300/file=C:/Users/USER/Desktop/ENTRETIENS)

[00:21:21-6](http://localhost:2300/file=C:/Users/USER/Desktop/ENTRETIENS%20PREMATURITE/CSPS-ACCART-VILLE/FOCUS%20GROUPE%20ACCART-VILLE-BON.mp3time=1281600)Interviewer: lorsque vous dites maladies vous faites allusion à quel genre de maladie? [00:21:21-8](http://localhost:2300/file=C:/Users/USER/Desktop/ENTRETIENS%20PREMATURITE/CSPS-ACCART-VILLE/FOCUS%20GROUPE%20ACCART-VILLE-BON.mp3time=1281800)

[00:21:21-8](http://localhost:2300/file=C:/U) Person 4: les prematurés peuvent être considerés comme des immunodeprimés puisque leurs organismes ne sont pas comme pour les enfants qui sont nées à terme en plus il y en a d'autres qui ne peuvent même pas bien tirer le lait maternel donc ils ne beneficient pas des anticorps du lait maternel cela les fragilise et ils ne sont pas immunisés. donc cela ouvre les portes aux maladies opportunistes. [00:22:05-6](http://localhost:2300/file=C:/Users/USER/Desktop/ENTRETIENS%20PREMATURITE/CSPS-ACCART-VILLE/FOCUS%20GROUPE%20ACCART-VILLE-BON.mp3time=1325600)

[00:22:05-6](http://localhost:2300/file=C:/Users/USER/Desktop/ENTRETIENS) Person 3: ce qu'on pourrait ajouter également ce sont les deficits qui sont liés à la prématurité, il peut avoir un deficit statuquo ponderal, il peut avoir aussi un deficit qui peut atteindre les organes de sens, on peut avoir également la stigmatisation parce que ces enfants peuvent être stigmatisés et ils (enfants prématurés) ont une appelation même en fonction des communautés pour montrer qu'il n'est pas venu à terme. il y a aussi les perceptions par rapport à leur viabilité, on dit géneralement dans nos communautés que ce ne sont pas des enfants qui sont viables et que l'enfant née au 8ème mois ne pourra pas vivre. tous ses aspects peuvent être des facteurs negatifs à la prise en charge des nourrissons prématurés. [00:23:18-9](http://localhost:2300/file=C:/Users/USER/Desktop/ENTRETIENS%20PREMATURITE/CSPS-ACCART-VILLE/FOCUS%20GROUPE%20ACCART-VILLE-BON.mp3time=1398900)

[00:23:18-9](http://localhost:2300/file=C:/Users/USER/Desk)Interviewer: quelles appelations ont ses enfants prématurés dans la communauté? [00:23:28-7](http://localhost:2300/file=C:/Users/USER/Desktop/ENTRETIENS)

[00:23:28-7](http://localhost:2300/file=C:/Users/USER/Desktop/ENTRETIENS%20PREMATURITE/CSPS-ACCART-VILLE/FOCUS%20GROUPE%20ACCART-VILLE-BON.mp3time=1408700)Person 2: les appelations varient selon les communautés, à bobo içi je ne connait pas mais dans le plateau mossi ils ont un nom dont je ne rappelle pas mais je sais qu'il y a des noms qu'on les attribue en fonction de leur terme de naissance, peut être qu'on pourrait revenir la dessus après. [00:24:02-6](http://localhost:2300/file=C:/Users/USER/Desktop/ENTRETIENS%20PREMATURITE/CSPS-ACCART-VILLE/FOCUS%20GROUPE%20ACCART-VILLE-BON.mp3time=1442600)

[00:24:02-6](http://localhost:2300/file=C:/Users/USER/Deskt) Person 1: c'est pour revenir sur les infections, on peut aussi ajouter les infections respiratoires est en plus remarquée chez les prematurés puisse que les bronchites ne sont pas bien developpés donc les prematurés ont tendances à avoir des problèmes respiratoires

[00:24:20-7](http://localhost:2300/file=C:/Users/USER/Desktop/ENTRETIENS) Interviewer: qu'est ce que vous avez remarqués comme comportement dans l'entourage immediat de l'enfant quand c'est un enfant prematuré? comment l'entourage se comporte avec la maman et l'enfant prematuré?

[00:24:56-0](http://localhost)Person 2: le problème qui peut se poser comme precendemment dit c'est la viabilité par ce que beaucoup pense que l'enfant ne pourra pas survivre, il y a aussi le fait que l'entourage peut avoir une conduite non convenable avec la maman. il y a des aspects positifs aussi qu'on peut relever,nous constatons que la maman est beaucoup plus entourée pour une assistance par rapport à la prise en charge de l'enfant, c'est à dire qu'on aide la maman par rapport à l'entretien de l'enfant, on lui dit d'eviter le courant d'air en couvrant bien l'enfant. il y a des aspects positifs comme des aspects negatifs. les aspects negatifs sont liés au fait que la maman n'est pas bien consideré parce qu'elle a mis un enfant au monde qui n'est pas à terme, souvent cela peut même être un objet d'accusion d'infidelité au niveau de la communauté.mais de façon géneral nous constatons qu'il y a un acharnement sur la maman en communauté dans le suivi.

[00:26:49-5](http://localhost:2300/file=C:/Users/USER/Desktop/ENTRETIENS%20PREMATURITE/CSPS-ACCART-VILLE/FOCUS%20GROUPE%20ACCART-VILLE-BON.mp3time=1609500)Interviewer: j'aimerais faire le lien entre ses pesenteurs socioculturels de l'environnement immediat de la maman et la prise en charge du nourrisson et sa maman dans les structures de santé.est ce que cela vous affecte beaucoup dans le suivi de ses enfants prématurés et de leurs mères? est ce que cela affecte la santé de la maman et de son bébé?

[00:27:36-8](http://localhost:2300/file=C:/Users/USER/Desktop/ENTRETIENS%20PREMATURITE/CSPS-ACCART-VILLE/FOCUS%20GROUPE%20ACCART-VILLE-BON.mp3time=1656800) Person 2: bien sûr ces pesenteurs affectent la santé de l'enfant parce que lorsqu'on leurs dit d'ammener l'enfant à l'hôpital à l'echelon superieur , ils refusent puisqu'ils pensent que l'enfant ne va pas vivre et ils vont aller gaspiller l'argent. aussi ceux qui sont à cotés vont raconter ailleus et lorsque la maman rentre en famille avec l'enfant, les gens par curiosité veulent venir voire l'enfant. donc nous disons aux parents de garder l'enfant dans la chambre et de ne pas laisser l'enfant à la portée de tout le monde c'est à dire les gens qui viennent pour voire puique c'est juste pour venir prendre des nouvelles et aller raconter. donc pour cette raison nous conseillons aux parents de laisser l'enfant dans la chambre et de ne pas le faire sortir. ensuite nous donnons rendez-vous à la maman pour qu'elle nous ramène l'enfant chaque deux semaine pour la pesée et administrer le vaccin parce que le poids n'est pas à la norme lorsqu'il ne s'agit pas d'un grand prematuré. donc nous donnons rendez-vous à la maman frequemment pour voire l'evolution de l'enfant.

00:20:00-5 Interviewer: Nous pouvons poursuivre, maintenant, quels sont les risques sanitaires particuliers auxquels les nourrissons prématurés sont confrontés en milieu urbain au Burkina Faso ?

00:20:40-5 Interviewer: Nous allons reformuler la question, de façon générale, quels sont les risques sanitaires particuliers auxquels les nourrissons prématurés sont confrontés en milieu urbain au Burkina Faso ?

00:20:57-6 Person 7: Moi je me dis que ce sont les infections parce que c'est ce qui joue sur beaucoup de prématurés. Si les prématurés ne sont pas bien entretenus, cela ouvre les portes aux maladies et généralement les enfants ne survivent pas.

00:21:21-6 Interviewer: Lorsque vous dites maladies, vous faites allusion à quel genre de maladie ?

00:21:21-8 Person 4: Les prématurés peuvent être considérés comme des immunodéprimés puisque leurs organismes ne sont pas comme pour les enfants qui sont nés à terme. En plus, il y en a d'autres qui ne peuvent même pas bien tirer le lait maternel, donc ils ne bénéficient pas des anticorps du lait maternel, cela les fragilise et ils ne sont pas immunisés. Donc cela ouvre les portes aux maladies opportunistes.

00:22:05-6 Person 3: Ce qu'on pourrait ajouter également ce sont les déficits qui sont liés à la prématurité, il peut avoir un déficit staturo-pondéral, il peut avoir aussi un déficit qui peut atteindre les organes de sens, on peut avoir également la stigmatisation parce que ces enfants peuvent être stigmatisés et ils (enfants prématurés) ont une appellation même en fonction des communautés pour montrer qu'il n'est pas venu à terme. Il y a aussi les perceptions par rapport à leur viabilité, on dit généralement dans nos communautés que ce ne sont pas des enfants qui sont viables et que l'enfant né au 8ème mois ne pourra pas vivre. Tous ces aspects peuvent être des facteurs négatifs à la prise en charge des nourrissons prématurés.

00:23:18-9 Interviewer: Quelles appellations ont ces enfants prématurés dans la communauté ? 00:23:28-7

00:23:28-7 Personne 2: Les appellations varient selon les communautés. À Bobo ici, je ne connais pas, mais dans le plateau Mossi, ils ont un nom dont je ne me rappelle pas, mais je sais qu'il y a des noms qu'on leur attribue en fonction de leur terme de naissance. Peut-être qu'on pourrait revenir là-dessus après. 00:24:02-6

00:24:02-6 Personne 1: C'est pour revenir sur les infections, on peut aussi ajouter que les infections respiratoires sont plus remarquées chez les prématurés puisque les bronches ne sont pas bien développées, donc les prématurés ont tendance à avoir des problèmes respiratoires.

00:24:20-7 Interviewer: Qu'est-ce que vous avez remarqué comme comportement dans l'entourage immédiat de l'enfant quand c'est un enfant prématuré ? Comment l'entourage se comporte avec la maman et l'enfant prématuré ?

00:24:56-0 Personne 2: Le problème qui peut se poser, comme précédemment dit, c'est la viabilité parce que beaucoup pensent que l'enfant ne pourra pas survivre. Il y a aussi le fait que l'entourage peut avoir une conduite non convenable avec la maman. Il y a des aspects positifs aussi qu'on peut relever. Nous constatons que la maman est beaucoup plus entourée pour une assistance par rapport à la prise en charge de l'enfant, c'est-à-dire qu'on aide la maman par rapport à l'entretien de l'enfant, on lui dit d'éviter le courant d'air en couvrant bien l'enfant. Il y a des aspects positifs comme des aspects négatifs. Les aspects négatifs sont liés au fait que la maman n'est pas bien considérée parce qu'elle a mis au monde un enfant qui n'est pas à terme, souvent cela peut même être un objet d'accusation d'infidélité au niveau de la communauté. Mais de façon générale, nous constatons qu'il y a un acharnement sur la maman en communauté dans le suivi.

00:26:49-5 Interviewer: J'aimerais faire le lien entre ces pesanteurs socioculturelles de l'environnement immédiat de la maman et la prise en charge du nourrisson et sa maman dans les structures de santé. Est-ce que cela vous affecte beaucoup dans le suivi de ces enfants prématurés et de leurs mères ? Est-ce que cela affecte la santé de la maman et de son bébé ?

00:27:36-8 Personne 2: Bien sûr, ces pesanteurs affectent la santé de l'enfant parce que lorsqu'on leur dit d'amener l'enfant à l'hôpital à l'échelon supérieur, ils refusent puisqu'ils pensent que l'enfant ne va pas vivre et ils vont aller gaspiller l'argent. Aussi ceux qui sont à côté vont raconter ailleurs, et lorsque la maman rentre en famille avec l'enfant, les gens par curiosité veulent venir voir l'enfant. Donc nous disons aux parents de garder l'enfant dans la chambre et de ne pas laisser l'enfant à la portée de tout le monde, c'est-à-dire les gens qui viennent pour voir puisque c'est juste pour venir prendre des nouvelles et aller raconter. Donc pour cette raison, nous conseillons aux parents de laisser l'enfant dans la chambre et de ne pas le faire sortir. Ensuite, nous donnons rendez-vous à la maman pour qu'elle nous ramène l'enfant chaque deux semaines pour la pesée et administrer le vaccin parce que le poids n'est pas à la norme lorsqu'il ne s'agit pas d'un grand prématuré. Donc nous donnons rendez-vous à la maman fréquemment pour voir l'évolution de l'enfant.

**00:20:00-5 Interviewer:** Let's continue. What specific health risks do premature infants face in urban areas of Burkina Faso?

**00:20:40-5 Interviewer:** Let me rephrase the question for clarity. In general, what particular health risks do premature infants face in urban areas of Burkina Faso?

**00:20:57-6 Person 7:** I believe that infections are a major risk for many premature babies. Without proper care, they are highly vulnerable to diseases, and unfortunately, many do not survive.

**00:21:21-6 Interviewer:** When you mention illnesses, could you specify what kinds you are referring to?

**00:21:21-8 Person 4:** Premature babies can be considered immunocompromised as their bodies aren't as developed as those of full-term children. Additionally, some are unable to effectively breastfeed, missing out on the antibodies in breast milk. This further weakens their immune system, making them susceptible to opportunistic diseases.

**00:22:05-6 Person 3:** Another aspect to consider are the deficits associated with prematurity, like weight and possibly deficits affecting the sense organs. There's also the issue of stigmatization. Premature children are often given specific names in different communities, signifying their premature birth. This stigmatization, along with a general perception of their lack of viability, can negatively impact their care.

**00:23:18-9 Interviewer:** What are these premature children called within the community?

**00:23:28-7 Person 2:** The names vary by community. In Bobo, the name might be different, but on the Mossi plateau, they have a specific name, which I can't recall right now. These names are based on their term of birth.

**00:24:02-6 Person 1:** Returning to the topic of infections, respiratory infections are also common in premature babies. Their bronchi are underdeveloped, making them prone to respiratory problems.

**Interviewer (00:20:40-5):** We are going to rephrase the question, in general terms, what are the particular health risks that premature infants face in urban areas in Burkina Faso?

**Person 7 (00:20:57-6):** I tell myself that it's the infections because that's what affects many premature babies. If premature babies are not well cared for, it opens the doors to diseases and usually the children do not survive.

**Interviewer (00:21:21-6):** When you say illnesses, what kind of illness are you referring to?

**Person 4 (00:21:21-8):** Premature babies can be considered immunocompromised since their bodies are not like those of children who are born full term. In addition, there are others who cannot even express breast milk well, so they do not benefit from the antibodies in breast milk, this weakens them and they are not immune. So this opens the doors to opportunistic diseases.

**Person 3 (00:22:05-6):** What we could also add are the deficits which are linked to prematurity, he can have a height and weight deficit, he can also have a deficit which can affect the sense organs, we can also have stigmatization because these children can be stigmatized and they (premature children) have a name even depending on the community to show that they have not come to term. There are also perceptions regarding their viability, we generally say in our communities that these are not children who are viable and that the child born in the 8th month will not be able to live. All of these aspects can be negative factors in the care of premature infants.

**Interviewer (00:23:18-9):** What are these premature children called in the community?

**Person 2 (00:23:28-7):** The names vary depending on the community, in Bobo here I don't know, but in the Mossi plateau they have a name that I don't remember, but I know that there are names that we attribute to them based on their term of birth, perhaps we could come back to that later.

**Person 1 (00:24:02-6):** This is to come back to infections, we can also add respiratory infections, which are also noticed in premature babies since the bronchi are not well developed, so premature babies tend to have breathing problems.

**Interviewer (00:26:49-5):** I would like to make the link between these sociocultural constraints of the mother's immediate environment and the care of the infant and his mother in health structures. Does this affect you a lot in monitoring these premature children and their mothers? Does this affect the health of the mother and her baby?

**Person 2 (00:27:36-8):** Of course, these burdens affect the health of the child because when they are told to take the child to the hospital at the higher level, they refuse since they think that the child is not going to live and they are going to waste the money. Also those who are nearby will tell the story elsewhere, and when the mother returns to the family with the child, people out of curiosity want to come and see the child. So, we tell the parents to keep the child in the room and not to leave the child within reach of everyone, that is to say people who come to see since it is just to come get news and go tell. So for this reason, we advise parents to leave the child in the room and not take them out. Then, we make an appointment with the mother so that she can bring the child back to us every two weeks for weighing and administering the vaccine, because the weight is not normal when it is not a question of 'a very premature baby. So, we make appointments with the mother frequently to see how the child is progressing.

### Manager of Accart-ville urban medical center

[00:15:52-2](http://localhost:2300/file=C:/Users/USER/Desktop/ENTRETIENS%20PREMATURITE/CSPS-ACCART-VILLE/RESPONSABLE%20CMU%20ACCART-VILLE.MP3time=952200) Interviewer: Maintenant, quels sont les risques sanitaires particuliers auxquels les nourrissons prématurés sont confrontés en milieu urbain au Burkina Faso? [00:16:01-5](http://localhost:2300/file=C:/Users/USER/Desktop/ENTRETIENS%20PREMATURITE/CSPS-ACCART-VILLE/RESPONSABLE%20CMU%20ACCART-VILLE.MP3time=961500)

[00:16:01-5](http://localhost:2300/file=C:/Users/USER/Desktop/ENTRETIENS%20PREMATURITE/CSPS-ACCART-VILLE/RESPONSABLE%20CMU%20ACCART-VILLE.MP3time=961500) Person 1: les problèmes sanitaires qui sont liés à la prématurité? generalement la prématurité entraine beaucoup d'infections puisqu'ils sont des êtres déjà fragiles. Voilà, ils sont soumis à des infections, des infections néonatal qui sont là. Les problèmes nutritionnels.ils Sont soumis à ça. Et puis aussi les problèmes respiratoires puisque leurs poumons ne sont pas encore assez développé pour cela , ça pose un vrai problème. quand on a un enfant prématuré, si on doit l'envoyer à l'hôpital Souro SANOU cela pose un problème économique puisque les parents sont obligés de cesser leur travail pour pouvoir être à côté de leur nouveau né. En fait, il y a tous ces facteurs là qui rentrent en compte. [00:16:56-5](http://localhost:2300/file=C:/Users/USER/Desktop/ENTRETIENS%20PREMATURITE/CSPS-ACCART-VILLE/RESPONSABLE%20CMU%20ACCART-VILLE.MP3time=1016500)

[00:16:56-5](http://localhost:2300/file=C:/Users/USER/Desktop/ENTRETIENS%20PREMATURITE/CSPS-ACCART-VILLE/RESPONSABLE%20CMU%20ACCART-VILLE.MP3time=1016500)Interviewer: Mais quand vous parlez d'infection, vous faites allusion à quel type d'infection en particulier? [00:17:01-3](http://localhost:2300/file=C:/Users/USER/Desktop/ENTRETIENS%20PREMATURITE/CSPS-ACCART-VILLE/RESPONSABLE%20CMU%20ACCART-VILLE.MP3time=1021300)

[00:17:01-3](http://localhost:2300/file=C:/Users/USER/Desktop/ENTRETIENS%20PREMATURITE/CSPS-ACCART-VILLE/RESPONSABLE%20CMU%20ACCART-VILLE.MP3time=1021300)Person 1: Bon, ce sont les infections néonatales. Il y a les infections pulmonaires, peuvent être là. Quoi qu'on dise, c'est de façon globale, peut être même s'est une infection chez la maman qui a causé la prématurité. Madame avait une infection génitale qui était presente, mais ça a fait que la grossesse n'a pas pu tenir. donc toutes ces infections généralement, ça rentre.ce sont les bacteriémies,les bactéries qui rentent chez l'enfant en même temps par la filière de la maman qui va entrainer que l'accouchement soit un accouchement prématuré.Donc l'enfant va subir ça aussi en fait, et en étant des êtres fragiles, s'il sort aussi il peut prendre une infection circulante au niveau de la société si on ne le protège pas bien. [00:17:55-2](http://localhost:2300/file=C:/Users/USER/Desktop/ENTRETIENS%20PREMATURITE/CSPS-ACCART-VILLE/RESPONSABLE%20CMU%20ACCART-VILLE.MP3time=1075200)

[00:17:55-2](http://localhost:2300/file=C:/Users/USER/Desktop/ENTRETIENS%20PREMATURITE/CSPS-ACCART-VILLE/RESPONSABLE%20CMU%20ACCART-VILLE.MP3time=1075200)Interviewer: Vous avez parlé tantôt de l'impact de la prématurité sur la mère. Est ce que vous pensez que ça peut avoir un impact sur la mortalité? [00:18:03-1](http://localhost:2300/file=C:/Users/USER/Desktop/ENTRETIENS%20PREMATURITE/CSPS-ACCART-VILLE/RESPONSABLE%20CMU%20ACCART-VILLE.MP3time=1083100)

[00:18:03-1](http://localhost:2300/file=C:/Users/USER/Desktop/ENTRETIENS%20PREMATURITE/CSPS-ACCART-VILLE/RESPONSABLE%20CMU%20ACCART-VILLE.MP3time=1083100)Person 1: Oui,c'est un facteur de mortalité en fait. Alors oui, chez les nouveaux nés d'abord. C'est un facteur psychologique aussi chez la maman. Le fait de pouvoir supporter une grossesse pendant un bon bout de temps et puis perdre l'enfant à l'accouchement en fait, en fait, c'est quelque chose qui n'est pas facile. ça peut jouer sur le mental de la maman. [00:18:28-1](http://localhost:2300/file=C:/Users/USER/Desktop/ENTRETIENS%20PREMATURITE/CSPS-ACCART-VILLE/RESPONSABLE%20CMU%20ACCART-VILLE.MP3time=1108100)

[00:18:28-1](http://localhost:2300/file=C:/Users/USER/Desktop/ENTRETIENS%20PREMATURITE/CSPS-ACCART-VILLE/RESPONSABLE%20CMU%20ACCART-VILLE.MP3time=1108100)Interviewer: cela peut jouer sur son mental comment? [00:18:29-5](http://localhost:2300/file=C:/Users/USER/Desktop/ENTRETIENS%20PREMATURITE/CSPS-ACCART-VILLE/RESPONSABLE%20CMU%20ACCART-VILLE.MP3time=1109500)

[00:18:29-5](http://localhost:2300/file=C:/Users/USER/Desktop/ENTRETIENS%20PREMATURITE/CSPS-ACCART-VILLE/RESPONSABLE%20CMU%20ACCART-VILLE.MP3time=1109500)Person 1: géneralement il y a des gens qui vont faire ce qu'on appelle des maladies psychologiques liées à l'accouchement, anciennement, on appelait des psychoses puerpérale c'est à dire après l'accouchement, il y a des dames dont le mental n'est plus comme avant. Alors c'est comme s'elles sont entrain de delirer.Finalement, tout ces facteurs peuventavoir un effet sur son affect, c'est à dire ue cela peut jouer sur l'affectif. [00:18:59-2](http://localhost:2300/file=C:/Users/USER/Desktop/ENTRETIENS%20PREMATURITE/CSPS-ACCART-VILLE/RESPONSABLE%20CMU%20ACCART-VILLE.MP3time=1139200)

00:15:52-2 Interviewer: Maintenant, quels sont les risques sanitaires particuliers auxquels les nourrissons prématurés sont confrontés en milieu urbain au Burkina Faso ?

00:16:01-5 Person 1: Les problèmes sanitaires qui sont liés à la prématurité ? Généralement la prématurité entraîne beaucoup d'infections puisqu'ils sont des êtres déjà fragiles. Voilà, ils sont soumis à des infections, des infections néonatales qui sont là. Les problèmes nutritionnels. Ils sont soumis à ça. Et puis aussi les problèmes respiratoires puisque leurs poumons ne sont pas encore assez développés pour cela, ça pose un vrai problème. Quand on a un enfant prématuré, si on doit l'envoyer à l'hôpital Souro SANOU cela pose un problème économique puisque les parents sont obligés de cesser leur travail pour pouvoir être à côté de leur nouveau-né. En fait, il y a tous ces facteurs-là qui rentrent en compte.

00:16:56-5 Interviewer: Mais quand vous parlez d'infection, vous faites allusion à quel type d'infection en particulier ?

00:17:01-3 Person 1: Bon, ce sont les infections néonatales. Il y a les infections pulmonaires, peuvent être là. Quoi qu'on dise, c'est de façon globale, peut-être même, c’est une infection chez la maman qui a causé la prématurité. Madame avait une infection génitale qui était présente, mais ça a fait que la grossesse n'a pas pu tenir. Donc toutes ces infections généralement, ça rentre. Ce sont les bactériémies, les bactéries qui rentrent chez l'enfant en même temps par la filière de la maman qui va entraîner que l'accouchement soit un accouchement prématuré. Donc l'enfant va subir ça aussi en fait, et en étant des êtres fragiles, s'il sort aussi il peut prendre une infection circulante au niveau de la société si on ne le protège pas bien.

00:17:55-2 Interviewer: Vous avez parlé tantôt de l'impact de la prématurité sur la mère. Est-ce que vous pensez que ça peut avoir un impact sur la mortalité ?

00:18:03-1 Person 1: Oui, c'est un facteur de mortalité en fait. Alors oui, chez les nouveau-nés d'abord. C'est un facteur psychologique aussi chez la maman. Le fait de pouvoir supporter une grossesse pendant un bon bout de temps et puis perdre l'enfant à l'accouchement, en fait, c'est quelque chose qui n'est pas facile. Ça peut jouer sur le mental de la maman. [00:18:28-1](http://localhost:2300/file=C:/Users/USER/Desktop/ENTRETIENS%20PREMATURITE/CSPS-ACCART-VILLE/RESPONSABLE%20CMU%20ACCART-VILLE.MP3time=1108100)

00:18:28-1 Interviewer: Cela peut jouer sur son mental comment ? 00:18:29-5

00:18:29-5 Personne 1: Généralement, il y a des gens qui vont faire ce qu'on appelle des maladies psychologiques liées à l'accouchement, anciennement, on appelait ça des psychoses puerpérales, c'est-à-dire après l'accouchement, il y a des dames dont le mental n'est plus comme avant. Alors c'est comme si elles sont en train de délirer. Finalement, tous ces facteurs peuvent avoir un effet sur son affect, c'est-à-dire que cela peut jouer sur l'affectif. 00:18:59-2

**00:15:52-2 Interviewer:** Could you elaborate on the specific health risks that premature infants face in urban areas of Burkina Faso?

**00:16:01-5 Person 1:** Health issues related to prematurity are significant. Premature infants are inherently fragile and are therefore highly susceptible to various infections, particularly neonatal infections. They also face nutritional challenges due to their underdeveloped state. Respiratory issues are common as well, given that their lungs are not fully developed. Moreover, caring for a premature infant presents an economic burden. If a child needs to be treated at Souro SANOU hospital, for example, parents often have to leave work to be with their newborn, which adds to the challenges.

**00:16:56-5 Interviewer:** When you mention infections, could you specify which types of infections you're referring to?

**00:17:01-3 Person 1:** Primarily, we're talking about neonatal infections. This includes lung infections, among others. Sometimes, the prematurity itself might be caused by an infection in the mother, like a genital infection that disrupts the pregnancy. These infections can lead to bacteremia, where bacteria enter the baby through the birth canal, causing premature birth. Being fragile, the child is also at risk of acquiring circulating infections in the community if not adequately protected.

**Interviewer (00:17:55-2):** You spoke earlier about the impact of prematurity on the mother. Do you think this could have an impact on mortality?

**Person 1 (00:18:03-1):** Yes, it is actually a mortality factor. So yes, in newborns first. It is also a psychological factor for the mother. Being able to endure a pregnancy for a long time and then losing the child during childbirth is actually something that is not easy. It can affect the mother's mind.

**00:18:28-1 Interviewer:** How can childbirth affect a woman's mental health?

**00:18:29-5 Person 1:** There are cases where childbirth leads to psychological disorders, what was previously known as puerperal psychoses. This occurs after giving birth. Some women experience a significant change in their mental state. It's like they enter a state of delirium. These changes can significantly affect their emotional well-being and mental health.

### Maternity manager of Accart-ville urban medical center

[00:19:30-7](http://localhost:2300/file=C:/Users/USER/Desktop/ENTRETIENS%20PREMATURITE/CSPS-ACCART-VILLE/RESPONSABLE%20MATERNITE%20CMU%20ACCART-VILLE.MP3time=1170700) Interviewer: ok. maintenant quels sont les risques sanitaires particuliers auxquels les nourrissons prématurés sont confrontés en milieu urbain au Burkina faso?

[00:19:42-7](http://localhost:2300/file=C:/Users/USER/Desktop/ENTRETIENS%20PREMATURITE/CSPS-ACCART-VILLE/RESPONSABLE%20MATERNITE%20CMU%20ACCART-VILLE.MP3time=1182700) Person 1: les risques sanitaires, on peut dire que ce sont les infections en général liées au milieu de vie même des mères, avec la promiscuité les gens sont nombreux dans les cours donc partage de germes. il y a egalement l'hygiène alimentaire c'est à dire rares sont ceux qui ont plus de trois repas par jour donc si la maman est malnutrie il va s'en dire que l'enfant aussi aurait des problèmes de nutrition à partir du lait maternel qui ne serait pas en quantité suffisante. ce sont géneralement ses problèmes que nous rencontrons, le problème de nutrtion et le problème d'hygiène qui sont des sources d'infections. il y a aussi le fait que lorsque la femme retourne en communauté, la maison n'est pas adapté, elle n'est pas seule dans la maison donc elle ne peut pas imposer certaines règles pour lutter contre l'hypothermie c'est à dire fermer les portes et les fenêtres, nous avons très souvent des retours d'informations sur ces cas de conflits du fait que lorsque la femme veut fermer les ouvertures de la maison certains s'y opposent donc il est difficile dans ces conditions de lutter efficacement contre l'hypothermie et les infections

[00:21:02-8](http://localhost:2300/file=C:/Users/USER/Desktop/ENTRETIENS%20PREMATURITE/CSPS-ACCART-VILLE/RESPONSABLE%20MATERNITE%20CMU%20ACCART-VILLE.MP3time=1262800) Interviewer: est ce que vous pensez que ces facteurs de risque peuvent impacter sur la mortalité des nourrissons prématurés?

[00:21:06-6](http://localhost:2300/file=C:/Users/USER/Desktop/ENTRETIENS%20PREMATURITE/CSPS-ACCART-VILLE/RESPONSABLE%20MATERNITE%20CMU%20ACCART-VILLE.MP3time=1266600) Person 1: effectivement cela à une influence sur la mortalité. l'hypothermie est une cause de mortalité, l'infections aussi fait partie des cinq prémières causes de mortalités néo-natales.vue la fragilité du nouveau née, l'infection est un facteur important de decès néo-natal de façon génerale

00:19:30-7 Interviewer: OK. Maintenant, quels sont les risques sanitaires particuliers auxquels les nourrissons prématurés sont confrontés en milieu urbain au Burkina Faso ?

00:19:42-7 Person 1: Les risques sanitaires, on peut dire que ce sont les infections en général liées au milieu de vie même des mères, avec la promiscuité les gens sont nombreux dans les cours donc partage de germes. Il y a également l'hygiène alimentaire, c'est-à-dire rares sont ceux qui ont plus de trois repas par jour, donc si la maman est malnutrie il va s'en dire que l'enfant aussi aurait des problèmes de nutrition à partir du lait maternel qui ne serait pas en quantité suffisante. Ce sont généralement ses problèmes que nous rencontrons, le problème de nutrition et le problème d'hygiène qui sont des sources d'infections. Il y a aussi le fait que lorsque la femme retourne en communauté, la maison n'est pas adaptée, elle n'est pas seule dans la maison donc elle ne peut pas imposer certaines règles pour lutter contre l'hypothermie, c'est-à-dire fermer les portes et les fenêtres. Nous avons très souvent des retours d'informations sur ces cas de conflits du fait que lorsque la femme veut fermer les ouvertures de la maison certains s'y opposent donc il est difficile dans ces conditions de lutter efficacement contre l'hypothermie et les infections.

00:21:02-8 Interviewer: Est-ce que vous pensez que ces facteurs de risque peuvent impacter sur la mortalité des nourrissons prématurés ?

00:21:06-6 Person 1: Effectivement, cela a une influence sur la mortalité. L'hypothermie est une cause de mortalité, l'infection aussi fait partie des cinq premières causes de mortalités néonatales. Vue la fragilité du nouveau-né, l'infection est un facteur important de décès néonatal de façon générale.

**00:19:30-7 Interviewer:** Okay. Can you tell us about the specific health risks that premature infants face in urban areas of Burkina Faso?

**00:19:42-7 Person 1:** The primary health risks for these infants are infections, often linked to the living conditions of their mothers. In crowded living spaces, the spread of germs is common. Additionally, food hygiene is a concern. Many people have less than three meals a day. If a mother is malnourished, it's likely that her child will also face nutritional issues due to insufficient breast milk. These are the common problems we encounter: nutrition and hygiene, both of which are significant sources of infections. Another issue is the unsuitability of housing when the mother returns to the community. She often shares her living space with others and can't impose necessary measures to combat hypothermia, such as closing doors and windows. We often hear about conflicts arising when a mother tries to take these precautions against hypothermia and infection, but faces objections from others in the house. In such conditions, it becomes challenging to effectively protect against these risks.

**Interviewer (00:21:02-8):** Do you think that these risk factors can impact the mortality of premature infants?

**Person 1 (00:21:06-6):** Indeed, it has an influence on mortality. Hypothermia is a cause of mortality, and infection is also one of the top five causes of neonatal mortality. Given the fragility of the newborn, infection is an important factor in neonatal death in general.

### Focus group of Do medical center with surgical branch

[00:20:33-6](http://localhost:2300/file=C:/Users/USER/Desktop/ENTRETIENS%20PREMATURITE/CMA-DO/FOCUS%20GROUPE%20CMA-DO.mp3time=1233600) Interviewer: De façon globale, quels sont les risques sanitaires particuliers auxquels les nourrissons prématurés sont confrontés en milieu urbain au Burkina Faso? [00:20:43-5](http://localhost:2300/file=C:/Users/USER/Desktop/ENTRETIENS%20PREMATURITE/CMA-DO/FOCUS%20GROUPE%20CMA-DO.mp3time=1243500)

[00:20:43-5](http://localhost:2300/file=C:/Users/USER/Desktop/ENTRETIENS%20PREMATURITE/CMA-DO/FOCUS%20GROUPE%20CMA-DO.mp3time=1243500) Person 6: Ce sont les infections néonatales [00:20:54-7](http://localhost:2300/file=C:/Users/USER/Desktop/ENTRETIENS%20PREMATURITE/CMA-DO/FOCUS%20GROUPE%20CMA-DO.mp3time=1254700) [00:20:49-4](http://localhost:2300/file=C:/Users/USER/Desktop/ENTRETIENS%20PREMATURITE/CMA-DO/FOCUS%20GROUPE%20CMA-DO.mp3time=1249400)

[00:20:49-4](http://localhost:2300/file=C:/Users/USER/Desktop/ENTRETIENS%20PREMATURITE/CMA-DO/FOCUS%20GROUPE%20CMA-DO.mp3time=1249400) Interviewer: Est ce qu'il y a quelque chose d'autre à ajouter? [00:20:54-5](http://localhost:2300/file=C:/Users/USER/Desktop/ENTRETIENS%20PREMATURITE/CMA-DO/FOCUS%20GROUPE%20CMA-DO.mp3time=1254500)

[00:20:54-5](http://localhost:2300/file=C:/Users/USER/Desktop/ENTRETIENS%20PREMATURITE/CMA-DO/FOCUS%20GROUPE%20CMA-DO.mp3time=1254500) Person 9: On avait parlé tantôt de la mortalité, la mortalité néonatale. [00:21:04-7](http://localhost:2300/file=C:/Users/USER/Desktop/ENTRETIENS%20PREMATURITE/CMA-DO/FOCUS%20GROUPE%20CMA-DO.mp3time=1264700)

[00:21:04-7](http://localhost:2300/file=C:/Users/USER/Desktop/ENTRETIENS%20PREMATURITE/CMA-DO/FOCUS%20GROUPE%20CMA-DO.mp3time=1264700) Person 5: La protection thermique parce que l'habillement souvent n'est pas adapté, le chapeau et les chaussettes doivent être systématiques, mais comme ce n'est pas dans la gratuité, c'est payant donc c'est pas toujours évident. [00:21:33-8](http://localhost:2300/file=C:/Users/USER/Desktop/ENTRETIENS%20PREMATURITE/CMA-DO/FOCUS%20GROUPE%20CMA-DO.mp3time=1293800)

00:20:33-6 Interviewer: De façon globale, quels sont les risques sanitaires particuliers auxquels les nourrissons prématurés sont confrontés en milieu urbain au Burkina Faso ? 00:20:43-5

00:20:43-5 Person 6: Ce sont les infections néonatales 00:20:54-7 00:20:49-4

00:20:49-4 Interviewer: Est-ce qu'il y a quelque chose d'autre à ajouter ? 00:20:54-5

00:20:54-5 Person 9: On avait parlé tantôt de la mortalité, la mortalité néonatale. 00:21:04-7

00:21:04-7 Person 5: La protection thermique parce que l'habillement souvent n'est pas adapté, le chapeau et les chaussettes doivent être systématiques, mais comme ce n'est pas dans la gratuité, c'est payant donc c'est pas toujours évident. 00:21:33-8

**00:20:33-6 Interviewer:** In general, what specific health risks do premature infants face in urban areas of Burkina Faso?

**00:20:43-5 Person 6:** The primary risk is neonatal infections.

**00:20:49-4 Interviewer:** Is there anything else you would like to add?

**00:20:54-5 Person 9:** We previously discussed neonatal mortality, which is a significant concern.

**00:21:04-7 Person 5:** Thermal protection is also crucial. The clothing for these infants, including hats and socks, is essential but not always affordable. Since these items aren't free, the cost can be a barrier, making it challenging to adequately protect these babies from the cold.

### Gynecologist 1

[00:12:44-5](http://localhost:2300/file=C:/Users/USER/Desktop/ENTRETIENS%20PREMATURITE/CMA-DO/GENYCOLOGUE%2001.mp3time=764500) Interviewer: Quels sont les risques sanitaires particuliers auxquels les nourrissons prématurés sont confrontés en milieu urbain au burkina faso ? [00:12:52-8](http://localhost:2300/file=C:/Users/USER/Desktop/ENTRETIENS%20PREMATURITE/CMA-DO/GENYCOLOGUE%2001.mp3time=772800)

[00:12:52-8](http://localhost:2300/file=C:/Users/USER/Desktop/ENTRETIENS%20PREMATURITE/CMA-DO/GENYCOLOGUE%2001.mp3time=772800) Person 1: Généralement c'est le risque infectieux. [00:12:59-3](http://localhost:2300/file=C:/Users/USER/Desktop/ENTRETIENS%20PREMATURITE/CMA-DO/GENYCOLOGUE%2001.mp3time=779300)

[00:12:59-3](http://localhost:2300/file=C:/Users/USER/Desktop/ENTRETIENS%20PREMATURITE/CMA-DO/GENYCOLOGUE%2001.mp3time=779300) Interviewer: Est-ce que cela a une incidence sur la mortalité ? [00:13:02-7](http://localhost:2300/file=C:/Users/USER/Desktop/ENTRETIENS%20PREMATURITE/CMA-DO/GENYCOLOGUE%2001.mp3time=782700)

[00:13:02-7](http://localhost:2300/file=C:/Users/USER/Desktop/ENTRETIENS%20PREMATURITE/CMA-DO/GENYCOLOGUE%2001.mp3time=782700) Person 1: Oui. Il y a une grande influence. [00:13:09-7](http://localhost:2300/file=C:/Users/USER/Desktop/ENTRETIENS%20PREMATURITE/CMA-DO/GENYCOLOGUE%2001.mp3time=789700)

00:12:44-5 Interviewer: Quels sont les risques sanitaires particuliers auxquels les nourrissons prématurés sont confrontés en milieu urbain au Burkina Faso ? 00:12:52-8

00:12:52-8 Person 1: Généralement c'est le risque infectieux. 00:12:59-3

00:12:59-3 Interviewer: Est-ce que cela a une incidence sur la mortalité ? 00:13:02-7

00:13:02-7 Person 1: Oui. Il y a une grande influence. 00:13:09-7

**00:12:44-5 Interviewer:** Could you describe the specific health risks that premature infants face in urban areas of Burkina Faso?

**00:12:52-8 Person 1:** The main risk for these infants is infection.

**00:12:59-3 Interviewer:** And does this risk of infection significantly affect mortality rates?

**00:13:02-7 Person 1:** Yes, it does. The risk of infection has a considerable impact on mortality.

### Gynecologist 2

[00:20:21-7](http://localhost:2300/file=C:/Users/USER/Desktop/ENTRETIENS%20PREMATURITE/CMA-DO/GENYCOLOGUE%2002.mp3time=1221700) Interviewer: D'accord. Maintenant de façon globale, quels sont les risques de sanitaires auxquels les enfants prématurés sont confrontés en milieu urbain au Burkina Faso ? [00:20:29-9](http://localhost:2300/file=C:/Users/USER/Desktop/ENTRETIENS%20PREMATURITE/CMA-DO/GENYCOLOGUE%2002.mp3time=1229900)

[00:20:29-9](http://localhost:2300/file=C:/Users/USER/Desktop/ENTRETIENS%20PREMATURITE/CMA-DO/GENYCOLOGUE%2002.mp3time=1229900) Person 1: Pour ce qui concerne l'enfant, le plus gros risque c'est l'hypothermie. C'est-à-dire que l'enfant soit exposé à un environnement où il prend froid et là c'est souvent fatal. Le plus gros risque c'est l'hypothermie, ensuite c'est les infections. Nous sommes dans un système hospitalier où souvent l'hygiène n'est pas forcément au rendez-vous donc l'enfant peut être infecté, que ce soit par le milieu extérieur ou bien le milieu même, le cadre hospitalier, l'enfant peut être infecté d'une manière ou d'une autre. Voilà pourquoi on met l'accent sur les conseils liés à l'hygiène. Parce que si l'enfant prématuré est infecté par un germe alimentaire par exemple et qu'il doit se retrouver dans la diarrhée, ça aggrave encore son état de santé. Donc pour la mère, les conseils vont s'orienter dans ce sens également. Dans le sens d'apporter de l'hygiène suffisamment à l'enfant, de le nourrir de façon convenable et de l'habiller chaudement. Pour ce qui concerne sa propre santé (la maman), puisque, géneralement, la prématurité peut être liée à une cause, une cause de maladie ou bien une cause sociale. Si par exemple c'est une maladie qui a fait que la maman a couché de façon prématurée, ça veut dire qu'elle a besoin de soin. Les soins vont s'orienter en fonction de la cause.Mais il arrive des situations où on n'a pas trouvé de cause, seulement les conseils qu'on donne à la maman pour bien se tenir par rapport à l'enfant. [00:22:04-9](http://localhost:2300/file=C:/Users/USER/Desktop/ENTRETIENS%20PREMATURITE/CMA-DO/GENYCOLOGUE%2002.mp3time=1324900)

[00:22:04-9](http://localhost:2300/file=C:/Users/USER/Desktop/ENTRETIENS%20PREMATURITE/CMA-DO/GENYCOLOGUE%2002.mp3time=1324900) Interviewer: Quand vous parlez d'infection, vous faites allusion à quelle type d'infection ? [00:22:09-2](http://localhost:2300/file=C:/Users/USER/Desktop/ENTRETIENS%20PREMATURITE/CMA-DO/GENYCOLOGUE%2002.mp3time=1329200)

[00:22:09-2](http://localhost:2300/file=C:/Users/USER/Desktop/ENTRETIENS%20PREMATURITE/CMA-DO/GENYCOLOGUE%2002.mp3time=1329200) Person 1: Ce sont les infections bactériennes. Parce que les prématurés qu'on voit ici, très souvent c'est lié à des ruptures prématurées des membranes. Ça veut dire que la grossesse évoluait et entre temps la poche des os s'est rompue, et quand la poche des os se rompt, très souvent c'est lié à des infections bactériennes au niveau génital chez la maman. La poche va se rompre et quand la maman sera hospitalisée, on fait des examens pour rechercher l'infection. La question est traitée. On essaie de maintenir la grossesse le plus longtemps possible jusqu'à ce que ça ne soit plus possible. Là on est obligé de faire sortir l'enfant, Soit par voie basse, soit par cesarienne. Généralement ce sont les infections bactériennes. [00:22:54-3](http://localhost:2300/file=C:/Users/USER/Desktop/ENTRETIENS%20PREMATURITE/CMA-DO/GENYCOLOGUE%2002.mp3time=1374300)

[00:22:54-3](http://localhost:2300/file=C:/Users/USER/Desktop/ENTRETIENS%20PREMATURITE/CMA-DO/GENYCOLOGUE%2002.mp3time=1374300) Interviewer: Est-ce que vous pensez que ces aspects influent sur la mortalité ? [00:22:58-7](http://localhost:2300/file=C:/Users/USER/Desktop/ENTRETIENS%20PREMATURITE/CMA-DO/GENYCOLOGUE%2002.mp3time=1378700)

[00:22:58-7](http://localhost:2300/file=C:/Users/USER/Desktop/ENTRETIENS%20PREMATURITE/CMA-DO/GENYCOLOGUE%2002.mp3time=1378700) Person 1: Bien sûr. Si par exemple il s'agissait d'une infection bactérienne qui a causé une rupture prématurée des membranes, la même infection peut se propager dans les liquides amniotiques et puis infecter l'enfant avant même sa naissance. Donc l'enfant va naître avec un problème infectieux qu'il va falloir prendre en charge pendant qu'il est là. Et ça peut aggraver son état de survie. [00:23:24-8](http://localhost:2300/file=C:/Users/USER/Desktop/ENTRETIENS%20PREMATURITE/CMA-DO/GENYCOLOGUE%2002.mp3time=1404800)

00:20:21-7 Interviewer: D'accord. Maintenant de façon globale, quels sont les risques sanitaires auxquels les enfants prématurés sont confrontés en milieu urbain au Burkina Faso ? 00:20:29-9

00:20:29-9 Person 1: Pour ce qui concerne l'enfant, le plus gros risque c'est l'hypothermie. C'est-à-dire que l'enfant soit exposé à un environnement où il prend froid et là c'est souvent fatal. Le plus gros risque c'est l'hypothermie, ensuite c'est les infections. Nous sommes dans un système hospitalier où souvent l'hygiène n'est pas forcément au rendez-vous donc l'enfant peut être infecté, que ce soit par le milieu extérieur ou bien le milieu même, le cadre hospitalier, l'enfant peut être infecté d'une manière ou d'une autre. Voilà pourquoi on met l'accent sur les conseils liés à l'hygiène. Parce que si l'enfant prématuré est infecté par un germe alimentaire par exemple et qu'il doit se retrouver dans la diarrhée, ça aggrave encore son état de santé. Donc pour la mère, les conseils vont s'orienter dans ce sens également. Dans le sens d'apporter de l'hygiène suffisamment à l'enfant, de le nourrir de façon convenable et de l'habiller chaudement. Pour ce qui concerne sa propre santé (la maman), puisque, généralement, la prématurité peut être liée à une cause, une cause de maladie ou bien une cause sociale. Si par exemple c'est une maladie qui a fait que la maman a accouché de façon prématurée, ça veut dire qu'elle a besoin de soin. Les soins vont s'orienter en fonction de la cause. Mais il arrive des situations où on n'a pas trouvé de cause, seulement les conseils qu'on donne à la maman pour bien se tenir par rapport à l'enfant. 00:22:04-9

00:22:04-9 Interviewer: Quand vous parlez d'infection, vous faites allusion à quel type d'infection ? 00:22:09-2

00:22:09-2 Person 1: Ce sont les infections bactériennes. Parce que les prématurés qu'on voit ici, très souvent c'est lié à des ruptures prématurées des membranes. Ça veut dire que la grossesse évoluait et entre temps la poche des eaux s'est rompue, et quand la poche des eaux se rompt, très souvent c'est lié à des infections bactériennes au niveau génital chez la maman. La poche va se rompre et quand la maman sera hospitalisée, on fait des examens pour rechercher l'infection. La question est traitée. On essaie de maintenir la grossesse le plus longtemps possible jusqu'à ce que ça ne soit plus possible. Là on est obligé de faire sortir l'enfant, soit par voie basse, soit par césarienne. Généralement ce sont les infections bactériennes. 00:22:54-3

00:22:54-3 Interviewer: Est-ce que vous pensez que ces aspects influent sur la mortalité ?

00:22:58-7 Person 1: Bien sûr. Si par exemple il s'agissait d'une infection bactérienne qui a causé une rupture prématurée des membranes, la même infection peut se propager dans les liquides amniotiques et puis infecter l'enfant avant même sa naissance. Donc l'enfant va naître avec un problème infectieux qu'il va falloir prendre en charge pendant qu'il est là. Et ça peut aggraver son état de survie.

**00:20:21-7 Interviewer:** Okay, can you tell us about the overall health risks that premature children face in urban areas of Burkina Faso?

**00:20:29-9 Person 1:** The primary risk for these children is hypothermia. They are exposed to cold environments which can often be fatal. After hypothermia, the next major risk is infection. In our hospital system, where hygiene standards may not always be optimal, children can easily become infected, either from the external environment or within the hospital setting itself. This is why we emphasize hygiene-related advice. For example, if a premature child is infected by a foodborne germ and develops diarrhea, this further deteriorates their health. We give similar advice to mothers, focusing on maintaining sufficient hygiene for the child, ensuring proper feeding, and keeping them warm. Concerning the mothers, prematurity is often linked to a health or social issue. If an illness caused premature birth, the mother will need care based on the specific cause. In cases where no cause is identified, we advise the mother on how to care for her child effectively.

**00:22:04-9 Interviewer:** When you mention infection, could you specify which type of infection you're referring to?

**00:22:09-2 Person 1:** We're mostly dealing with bacterial infections. Often in the premature babies we see, the condition is linked to premature rupture of membranes. This means the pregnancy was progressing, but then the amniotic sac broke prematurely, which is frequently associated with bacterial infections in the mother's genital area. After the sac ruptures, and the mother is hospitalized, we conduct tests to identify the infection. We try to maintain the pregnancy as long as possible until delivery is unavoidable, which might result in either a vaginal birth or a cesarean section. Typically, these are bacterial infections.

**Interviewer (00:22:54-3):** Do you think these aspects influence mortality?

**Person 1: (00:22:58-7):** Of course. If, for example, it was a bacterial infection that caused premature rupture of the membranes, the same infection can spread in the amniotic fluids and then infect the child even before birth. So the child will be born with an infectious problem that will have to be taken care of while he is there. And that can worsen his state of survival.

### Chief physician of Do health district

[00:22:33-9](http://localhost:2300/file=C:/Users/USER/Desktop/ENTRETIENS%20PREMATURITE/CMA-DO/MCD-DO.mp3time=1353900) Interviewer: De façon globale, quelles sont les risques sanitaires auxquelles les nourrissons prématurés sont confrontés ici, en milieu urbain au Burkina Faso? #4 :22:42-1#

[00:22:42-1](http://localhost:2300/file=C:/Users/USER/Desktop/ENTRETIENS%20PREMATURITE/CMA-DO/MCD-DO.mp3time=1362100) Person 1: L'enfant est né même à terme est fragile à plus forte raison qu'un enfant qui n'est pas né à terme. Donc, c'est la mortalité. C'est le risque lié aux infections. Ils peuvent en tout cas avoir. Et puis, comme ils ne sont pas suffisamment bien préparés, donc cela peut survenir si le suivi n'est pas vraiment optimal. [00:23:15-2](http://localhost:2300/file=C:/Users/USER/Desktop/ENTRETIENS%20PREMATURITE/CMA-DO/MCD-DO.mp3time=1395200)

00:22:33-9 Interviewer: De façon globale, quelles sont les risques sanitaires auxquels les nourrissons prématurés sont confrontés ici, en milieu urbain au Burkina Faso ?

00:22:42-1 Person 1: L'enfant est né même à terme est fragile, à plus forte raison qu'un enfant qui n'est pas né à terme. Donc, c'est la mortalité. C'est le risque lié aux infections. Ils peuvent en tout cas avoir. Et puis, comme ils ne sont pas suffisamment bien préparés, donc cela peut survenir si le suivi n'est pas vraiment optimal.

**Interviewer (00:22:33-9):** Overall, what are the health risks that premature infants face here, in urban areas in Burkina Faso?

**Person 1:** A child born even at full term is fragile, even more so than a child who is not born at full term. So, the risk is mortality, linked to infections. They can have these infections, and as they are not sufficiently well prepared, this can happen if the follow-up is not really optimal.

### Manager for maternity care unit of Do medical center with surgical branch

[00:17:43-5](http://localhost:2300/file=C:/Users/USER/Desktop/ENTRETIENS%20PREMATURITE/CMA-DO/RESPONSABLE%20UNITE%20DE%20SOIN%20CMA-DO.mp3time=1063500) Interviewer: De façon globale, selon vous, quels sont les risques sanitaires auxquels les enfants prématurés sont confrontés à milieu urbain ici à Burkina Faso? [00:17:52-8](http://localhost:2300/file=C:/Users/USER/Desktop/ENTRETIENS%20PREMATURITE/CMA-DO/RESPONSABLE%20UNITE%20DE%20SOIN%20CMA-DO.mp3time=1072800)

[00:17:52-8](http://localhost:2300/file=C:/Users/USER/Desktop/ENTRETIENS%20PREMATURITE/CMA-DO/RESPONSABLE%20UNITE%20DE%20SOIN%20CMA-DO.mp3time=1072800) Person 1: Les risques sanitaires...Comment? [00:17:57-1](http://localhost:2300/file=C:/Users/USER/Desktop/ENTRETIENS%20PREMATURITE/CMA-DO/RESPONSABLE%20UNITE%20DE%20SOIN%20CMA-DO.mp3time=1077100)

[00:17:57-1](http://localhost:2300/file=C:/Users/USER/Desktop/ENTRETIENS%20PREMATURITE/CMA-DO/RESPONSABLE%20UNITE%20DE%20SOIN%20CMA-DO.mp3time=1077100) Interviewer: À quel type de risque sanitaire? C'est-à-dire, est-ce qu'il y a un certain type de maladie auxquels les enfants, les nourrissons prématurées sont confrontés? [00:18:06-8](http://localhost:2300/file=C:/Users/USER/Desktop/ENTRETIENS%20PREMATURITE/CMA-DO/RESPONSABLE%20UNITE%20DE%20SOIN%20CMA-DO.mp3time=1086800)

[00:18:06-8](http://localhost:2300/file=C:/Users/USER/Desktop/ENTRETIENS%20PREMATURITE/CMA-DO/RESPONSABLE%20UNITE%20DE%20SOIN%20CMA-DO.mp3time=1086800) Person 1: Oui, c'est généralement les infections. Ce sont les infections. Ils ont un système immunitaire qui va être faible, donc facilement, ils peuvent contracter les maladies infectieuses rapidement. C'est surtout les infections. [00:18:25-4](http://localhost:2300/file=C:/Users/USER/Desktop/ENTRETIENS%20PREMATURITE/CMA-DO/RESPONSABLE%20UNITE%20DE%20SOIN%20CMA-DO.mp3time=1105400)

[00:18:25-4](http://localhost:2300/file=C:/Users/USER/Desktop/ENTRETIENS%20PREMATURITE/CMA-DO/RESPONSABLE%20UNITE%20DE%20SOIN%20CMA-DO.mp3time=1105400) Interviewer: Quel genre d'infection, par exemple? [00:18:27-3](http://localhost:2300/file=C:/Users/USER/Desktop/ENTRETIENS%20PREMATURITE/CMA-DO/RESPONSABLE%20UNITE%20DE%20SOIN%20CMA-DO.mp3time=1107300)

[00:18:27-3](http://localhost:2300/file=C:/Users/USER/Desktop/ENTRETIENS%20PREMATURITE/CMA-DO/RESPONSABLE%20UNITE%20DE%20SOIN%20CMA-DO.mp3time=1107300) Person 1: Quand je dis les infections, il y a l'infection, par exemple, les infections du néonatales.Qu'est-ce qu'on peut mettre dedans? Parce que ce n'est pas trop mon domaine. Mais bon, on peut mettre... Quand on parle d'infection, il y a le paludisme qui est dedans. Il y a le paludisme et les infections bronchopulmonaires. [00:18:56-2](http://localhost:2300/file=C:/Users/USER/Desktop/ENTRETIENS%20PREMATURITE/CMA-DO/RESPONSABLE%20UNITE%20DE%20SOIN%20CMA-DO.mp3time=1136200)

[00:18:56-2](http://localhost:2300/file=C:/Users/USER/Desktop/ENTRETIENS%20PREMATURITE/CMA-DO/RESPONSABLE%20UNITE%20DE%20SOIN%20CMA-DO.mp3time=1136200) Interviewer: Est-ce que ces infections peuvent avoir une incidence sur la mortalité? [00:19:04-6](http://localhost:2300/file=C:/Users/USER/Desktop/ENTRETIENS%20PREMATURITE/CMA-DO/RESPONSABLE%20UNITE%20DE%20SOIN%20CMA-DO.mp3time=1144600)

[00:19:04-6](http://localhost:2300/file=C:/Users/USER/Desktop/ENTRETIENS%20PREMATURITE/CMA-DO/RESPONSABLE%20UNITE%20DE%20SOIN%20CMA-DO.mp3time=1144600) Person 1: Oui. Ça peut avoir une incidence sur la mortalité. Quand je parle de pathologie, il y a l'hypoglycémie aussi que ces nouveaux-nés prématurés peuvent avoir. Alors que le hypoglycémie aussi peut être fatal pour ces nouveaux-nés.Voilà. Donc il y a tout ça. [00:19:24-9](http://localhost:2300/file=C:/Users/USER/Desktop/ENTRETIENS%20PREMATURITE/CMA-DO/RESPONSABLE%20UNITE%20DE%20SOIN%20CMA-DO.mp3time=1164900)

00:17:43-5 Interviewer: De façon globale, selon vous, quels sont les risques sanitaires auxquels les enfants prématurés sont confrontés en milieu urbain ici au Burkina Faso ? 00:17:52-8

00:17:52-8 Person 1: Les risques sanitaires... Comment ? 00:17:57-1

00:17:57-1 Interviewer: À quel type de risque sanitaire ? C'est-à-dire, est-ce qu'il y a un certain type de maladie auxquels les enfants, les nourrissons prématurés sont confrontés ? 00:18:06-8

00:18:06-8 Person 1: Oui, c'est généralement les infections. Ce sont les infections. Ils ont un système immunitaire qui va être faible, donc facilement, ils peuvent contracter les maladies infectieuses rapidement. C'est surtout les infections. 00:18:25-4

00:18:25-4 Interviewer: Quel genre d'infection, par exemple ? 00:18:27-3

00:18:27-3 Person 1: Quand je dis les infections, il y a l'infection, par exemple, les infections néonatales. Qu'est-ce qu'on peut mettre dedans ? Parce que ce n'est pas trop mon domaine. Mais bon, on peut mettre... Quand on parle d'infection, il y a le paludisme qui est dedans. Il y a le paludisme et les infections bronchopulmonaires. 00:18:56-2

00:18:56-2 Interviewer: Est-ce que ces infections peuvent avoir une incidence sur la mortalité ?

00:19:04-6 Person 1: Oui. Ça peut avoir une incidence sur la mortalité. Quand je parle de pathologie, il y a l'hypoglycémie aussi que ces nouveau-nés prématurés peuvent avoir. Alors que l'hypoglycémie aussi peut être fatal pour ces nouveau-nés. Voilà. Donc il y a tout ça.

**00:17:43-5 Interviewer:** In your opinion, what are the health risks that premature children face in urban areas here in Burkina Faso?

**00:17:52-8 Person 1:** Health risks... Could you clarify?

**00:17:57-1 Interviewer:** I mean, are there specific types of illnesses or health issues that premature infants are particularly susceptible to?

**00:18:06-8 Person 1:** Yes, the primary risk is infections. Premature infants have a weaker immune system, making them more susceptible to infectious diseases. It's primarily infections that are the concern.

**00:18:25-4 Interviewer:** Can you give an example of such infections?

**00:18:27-3 Person 1:** When we talk about infections, I mean things like neonatal infections. As for specific examples, although it's not exactly my area of expertise, we can include conditions like malaria and bronchopulmonary infections.

**00:18:56-2 Interviewer:** Can these infections impact mortality rates?

**00:19:04-6 Person 1:** Yes, they can indeed affect mortality. In discussing pathology, it's important to note that premature newborns can also experience hypoglycemia. This condition, hypoglycemia, can be life-threatening for these newborns. So, that's another critical aspect to consider.

### Focus group of Colma1 health and social promotion center

[00:18:18-3](http://localhost:2300/file=C:/Users/USER/Desktop/ENTRETIENS%20PREMATURITE/CSPS-COLMA1/FOCUS%20GROUPE%20COLMA1.mp3time=1098300) Interviewer: de manière global, quels sont les risques sanitaires auxquels les nourrissons prématurés sont confrontés en milieu urbain au burkina faso? [00:18:30-4](http://localhost:2300/file=C:/Users/USER/Desktop/ENTRETIENS%20PREMATURITE/CSPS-COLMA1/FOCUS%20GROUPE%20COLMA1.mp3time=1110400)

[00:18:30-4](http://localhost:2300/file=C:/Users/USER/Desktop/ENTRETIENS%20PREMATURITE/CSPS-COLMA1/FOCUS%20GROUPE%20COLMA1.mp3time=1110400) Person 2: les reisques sanitaires, géneralemnt ce sont les cas d'hypothermie, il ne faut pas que l'enfant prenne froid, il faut le garder au chaud. Maintenant je me dis qu'un enfant prématuré n'est pas comme un enfant né à terme parce qu'il est plus exposé donc aux infections qu'un enfant né à terme. il faut eviter que l'enfant ne s'infecte avant qu'on ne puisse l'evacuer. Qu'est ce qu'on peut ajouter encore? [00:19:16-8](http://localhost:2300/file=C:/Users/USER/Desktop/ENTRETIENS%20PREMATURITE/CSPS-COLMA1/FOCUS%20GROUPE%20COLMA1.mp3time=1156800)

[00:19:16-8](http://localhost:2300/file=C:/Users/USER/Desktop/ENTRETIENS%20PREMATURITE/CSPS-COLMA1/FOCUS%20GROUPE%20COLMA1.mp3time=1156800) Person 3: il y aura la malnutrition, si l'enfant n'est pas bien nourri, il arrive que l'enfant prématuré n'ait pas le reflexe de succion il faut le nourrir. Ensuite ils sont exposés aux problèmes respiratoires [00:19:35-2](http://localhost:2300/file=C:/Users/USER/Desktop/ENTRETIENS%20PREMATURITE/CSPS-COLMA1/FOCUS%20GROUPE%20COLMA1.mp3time=1175200)

[00:19:35-2](http://localhost:2300/file=C:/Users/USER/Desktop/ENTRETIENS%20PREMATURITE/CSPS-COLMA1/FOCUS%20GROUPE%20COLMA1.mp3time=1175200) Interviewer: Est ce que ses risquent ont une incidence sur le taux de mortalité? [00:19:39-0](http://localhost:2300/file=C:/Users/USER/Desktop/ENTRETIENS%20PREMATURITE/CSPS-COLMA1/FOCUS%20GROUPE%20COLMA1.mp3time=1179000)

[00:19:39-0](http://localhost:2300/file=C:/Users/USER/Desktop/ENTRETIENS%20PREMATURITE/CSPS-COLMA1/FOCUS%20GROUPE%20COLMA1.mp3time=1179000) Person 3: Bien sur, bien sur parce que, un prématuré malnutri ou bien qui a problème respiratoire qui n'est pas pris en charge correctement va mourir. [00:19:53-9](http://localhost:2300/file=C:/Users/USER/Desktop/ENTRETIENS%20PREMATURITE/CSPS-COLMA1/FOCUS%20GROUPE%20COLMA1.mp3time=1193900)

00:18:18-3 Interviewer: De manière globale, quels sont les risques sanitaires auxquels les nourrissons prématurés sont confrontés en milieu urbain au Burkina Faso ? 00:18:30-4

00:18:30-4 Person 2: Les risques sanitaires, généralement ce sont les cas d'hypothermie, il ne faut pas que l'enfant prenne froid, il faut le garder au chaud. Maintenant je me dis qu'un enfant prématuré n'est pas comme un enfant né à terme parce qu'il est plus exposé donc aux infections qu'un enfant né à terme. Il faut éviter que l'enfant ne s'infecte avant qu'on ne puisse l'évacuer. Qu'est-ce qu'on peut ajouter encore ? 00:19:16-8

00:19:16-8 Person 3: Il y aura la malnutrition, si l'enfant n'est pas bien nourri, il arrive que l'enfant prématuré n'ait pas le réflexe de succion il faut le nourrir. Ensuite ils sont exposés aux problèmes respiratoires. 00:19:35-2

00:19:35-2 Interviewer: Est-ce que ces risques ont une incidence sur le taux de mortalité ?

00:19:39-0 Person 3: Bien sûr, bien sûr, parce que, un prématuré malnutri ou bien qui a un problème respiratoire qui n'est pas pris en charge correctement va mourir.

**00:18:18-3 Interviewer:** What are the overall health risks that premature infants face in urban areas of Burkina Faso?

**00:18:30-4 Person 2:** The main health risks for premature infants generally include hypothermia; it's crucial to keep them warm. A premature child, being more vulnerable than a full-term infant, is significantly more exposed to infections. It's important to prevent infections in these infants before considering their evacuation for further care. Are there other risks to consider?

**00:19:16-8 Person 3:** Malnutrition is another major concern. Premature infants often lack the sucking reflex necessary for feeding and require special attention in this regard. Additionally, they are susceptible to respiratory problems.

**00:19:35-2 Interviewer:** Do these risks have an impact on the mortality rate?

**00:19:39-0 Person 3:** Absolutely, they do. A premature baby who is malnourished or has a respiratory issue that isn't adequately addressed is at a significant risk of mortality.

### Major of Colma1 health and social promotion center

[00:15:13-0](http://localhost:2300/file=C:/Users/USER/Desktop/ENTRETIENS%20PREMATURITE/CSPS-COLMA1/MAJOR%20CSPS%20COLMA1.MP3time=913000) Interviewer: De façon globale, en milieu urbain au Burkina Faso, quels sont les risques sanitaires particuliers auxquels les nourrissons prématurées sont confrontés ? [00:15:21-5](http://localhost:2300/file=C:/Users/USER/Desktop/ENTRETIENS%20PREMATURITE/CSPS-COLMA1/MAJOR%20CSPS%20COLMA1.MP3time=921500)

[00:15:21-5](http://localhost:2300/file=C:/Users/USER/Desktop/ENTRETIENS%20PREMATURITE/CSPS-COLMA1/MAJOR%20CSPS%20COLMA1.MP3time=921500) Person 1: Prématurés? [00:15:22-2](http://localhost:2300/file=C:/Users/USER/Desktop/ENTRETIENS%20PREMATURITE/CSPS-COLMA1/MAJOR%20CSPS%20COLMA1.MP3time=922200)

[00:15:22-2](http://localhost:2300/file=C:/Users/USER/Desktop/ENTRETIENS%20PREMATURITE/CSPS-COLMA1/MAJOR%20CSPS%20COLMA1.MP3time=922200) Interviewer: oui [00:15:22-8](http://localhost:2300/file=C:/Users/USER/Desktop/ENTRETIENS%20PREMATURITE/CSPS-COLMA1/MAJOR%20CSPS%20COLMA1.MP3time=922800)

[00:15:22-8](http://localhost:2300/file=C:/Users/USER/Desktop/ENTRETIENS%20PREMATURITE/CSPS-COLMA1/MAJOR%20CSPS%20COLMA1.MP3time=922800) Person 1: mais c'est ce que je viens de dire tantôt. Il y a le refroidissement parce que leur Thermorégulation n'est pas assez mature donc on a peur du refroidissement, l'hypothermie. Voilà, parce que le prématuré, il est exposé à l'hypothermie et et à l'hypoglycémie principalement.Parce que, d'autres vont sortir, n'arrivent pas à téter tout ça là donc l'hypoglycémie vient immediatement donc ce sont des risques et puis lorsqu'une prise en charge immédiate n'est pas faite à cet enfant en tout cas ça risque d'être fatal. [00:16:00-2](http://localhost:2300/file=C:/Users/USER/Desktop/ENTRETIENS%20PREMATURITE/CSPS-COLMA1/MAJOR%20CSPS%20COLMA1.MP3time=960200)

[00:16:00-2](http://localhost:2300/file=C:/Users/USER/Desktop/ENTRETIENS%20PREMATURITE/CSPS-COLMA1/MAJOR%20CSPS%20COLMA1.MP3time=960200) Interviewer: ce sont des facteurs de risque sur la mortalité? [00:16:01-8](http://localhost:2300/file=C:/Users/USER/Desktop/ENTRETIENS%20PREMATURITE/CSPS-COLMA1/MAJOR%20CSPS%20COLMA1.MP3time=961800)

[00:16:01-8](http://localhost:2300/file=C:/Users/USER/Desktop/ENTRETIENS%20PREMATURITE/CSPS-COLMA1/MAJOR%20CSPS%20COLMA1.MP3time=961800) Person 1: voilà c'est ça. [00:16:03-9](http://localhost:2300/file=C:/Users/USER/Desktop/ENTRETIENS%20PREMATURITE/CSPS-COLMA1/MAJOR%20CSPS%20COLMA1.MP3time=963900)

00:15:13-0 Interviewer: De façon globale, en milieu urbain au Burkina Faso, quels sont les risques sanitaires particuliers auxquels les nourrissons prématurés sont confrontés ? 00:15:21-5

00:15:21-5 Person 1: Prématurés ? 00:15:22-2

00:15:22-2 Interviewer: Oui. 00:15:22-8

00:15:22-8 Person 1: Mais c'est ce que je viens de dire tantôt. Il y a le refroidissement parce que leur thermorégulation n'est pas assez mature donc on a peur du refroidissement, de l'hypothermie. Voilà, parce que le prématuré, il est exposé à l'hypothermie et à l'hypoglycémie principalement. Parce que, d'autres vont sortir, n'arrivent pas à téter tout ça là donc l'hypoglycémie vient immédiatement donc ce sont des risques et puis lorsqu'une prise en charge immédiate n'est pas faite à cet enfant en tout cas ça risque d'être fatal. 00:16:00-2

00:16:00-2 Interviewer: Ce sont des facteurs de risque sur la mortalité ? 00:16:01-8

00:16:01-8 Person 1: Voilà, c'est ça. 00:16:03-9

**00:15:13-0 Interviewer:** In the context of an urban environment in Burkina Faso, what specific health risks do premature infants face?

**00:15:21-5 Person 1:** Are you referring to preemies?

**00:15:22-2 Interviewer:** Yes.

**00:15:22-8 Person 1:** Right, as I mentioned earlier, one major concern is their underdeveloped thermoregulation, leading to a risk of hypothermia. Premature babies are particularly vulnerable to both hypothermia and hypoglycemia. These conditions can arise quickly, especially if they are unable to feed properly. Without immediate care, these risks can indeed be life-threatening.

**00:16:00-2 Interviewer:** So, would you consider these as risk factors for increased mortality?

**00:16:01-8 Person 1:** Exactly, that's precisely it.

### Maternity manager of Colma1 health and social promotion center

[00:28:30-8](http://localhost:2300/file=C:/Users/USER/Desktop/ENTRETIENS%20PREMATURITE/CSPS-COLMA1/RESPONSABLE%20MATERNITE-COLMA1.mp3time=1710800) Interviewer: Vous avez répondu à deux questions à la fois, mais c'est pour confirmer. Quels sont les risques sanitaires particuliers auxquels les nourrissons prématurés sont confrontés ? [00:28:41-9](http://localhost:2300/file=C:/Users/USER/Desktop/ENTRETIENS%20PREMATURITE/CSPS-COLMA1/RESPONSABLE%20MATERNITE-COLMA1.mp3time=1721900)

[00:28:41-9](http://localhost:2300/file=C:/Users/USER/Desktop/ENTRETIENS%20PREMATURITE/CSPS-COLMA1/RESPONSABLE%20MATERNITE-COLMA1.mp3time=1721900) Person 1: Morbidité, mortalité. [00:28:44-2](http://localhost:2300/file=C:/Users/USER/Desktop/ENTRETIENS%20PREMATURITE/CSPS-COLMA1/RESPONSABLE%20MATERNITE-COLMA1.mp3time=1724200)

[00:28:44-2](http://localhost:2300/file=C:/Users/USER/Desktop/ENTRETIENS%20PREMATURITE/CSPS-COLMA1/RESPONSABLE%20MATERNITE-COLMA1.mp3time=1724200) Interviewer: Comment ? [00:28:45-5](http://localhost:2300/file=C:/Users/USER/Desktop/ENTRETIENS%20PREMATURITE/CSPS-COLMA1/RESPONSABLE%20MATERNITE-COLMA1.mp3time=1725500)

[00:28:45-5](http://localhost:2300/file=C:/Users/USER/Desktop/ENTRETIENS%20PREMATURITE/CSPS-COLMA1/RESPONSABLE%20MATERNITE-COLMA1.mp3time=1725500) Person 1: Oui, par exemple, un prématuré qui naît. Je vous dis même un enfant sain. Il y a trois choses si l'agent de santé ne met pas en place rapidement, l'enfant peut repartir. Voici quand, par exemple, on ne traite pas le nouveau né avec beaucoup de précautions, il faut être automatique. L'infection,sa peau même, n'est pas mature. L'infection est là, et l'hypoglycémie. Ce sont ses trois qui peuvent amener le prématuré. L'hypoglycémie même, je vais parler de l'hypothermie chez les nouveaux, les prématurés. Nous, tous les enfants, mais parce que ça commence par la tête, tout est fragile, rien n'est préparé pour même être... C'est un peu compliqué. [00:29:29-5](http://localhost:2300/file=C:/Users/USER/Desktop/ENTRETIENS%20PREMATURITE/CSPS-COLMA1/RESPONSABLE%20MATERNITE-COLMA1.mp3time=1769500)

[00:29:29-5](http://localhost:2300/file=C:/Users/USER/Desktop/ENTRETIENS%20PREMATURITE/CSPS-COLMA1/RESPONSABLE%20MATERNITE-COLMA1.mp3time=1769500) Interviewer: Vous avez même abordé un peu la catégorie de femmes qui sont le plus assujetties à donner naissance au prématuré. Vous avez parlé un peu des facteurs socio- culturels. Vous avez abordé aussi les femmes qui sont assujetties à certains types de maladies - [00:29:46-4](http://localhost:2300/file=C:/Users/USER/Desktop/ENTRETIENS%20PREMATURITE/CSPS-COLMA1/RESPONSABLE%20MATERNITE-COLMA1.mp3time=1786400)

[00:29:46-4](http://localhost:2300/file=C:/Users/USER/Desktop/ENTRETIENS%20PREMATURITE/CSPS-COLMA1/RESPONSABLE%20MATERNITE-COLMA1.mp3time=1786400) Person 1: C'est ça. [00:29:47-6](http://localhost:2300/file=C:/Users/USER/Desktop/ENTRETIENS%20PREMATURITE/CSPS-COLMA1/RESPONSABLE%20MATERNITE-COLMA1.mp3time=1787600)

00:28:30-8 Interviewer: Vous avez répondu à deux questions à la fois, mais c'est pour confirmer. Quels sont les risques sanitaires particuliers auxquels les nourrissons prématurés sont confrontés ? 00:28:41-9

00:28:41-9 Person 1: Morbidité, mortalité. 00:28:44-2

00:28:44-2 Interviewer: Comment ? 00:28:45-5

00:28:45-5 Person 1: Oui, par exemple, un prématuré qui naît. Je vous dis même un enfant sain. Il y a trois choses si l'agent de santé ne met pas en place rapidement, l'enfant peut repartir. Voici quand, par exemple, on ne traite pas le nouveau-né avec beaucoup de précautions, il faut être automatique. L'infection, sa peau même, n'est pas mature. L'infection est là, et l'hypoglycémie. Ce sont ses trois qui peuvent amener le prématuré. L'hypoglycémie même, je vais parler de l'hypothermie chez les nouveaux, les prématurés. Nous, tous les enfants, mais parce que ça commence par la tête, tout est fragile, rien n'est préparé pour même être... C'est un peu compliqué. 00:29:29-5

00:29:29-5 Interviewer: Vous avez même abordé un peu la catégorie de femmes qui sont le plus assujetties à donner naissance au prématuré. Vous avez parlé un peu des facteurs socio-culturels. Vous avez abordé aussi les femmes qui sont assujetties à certains types de maladies - 00:29:46-4

00:29:46-4 Person 1: C'est ça. 00:29:47-6

**00:28:30-8 Interviewer:** You've covered two topics simultaneously, but just to confirm, could you specify the particular health risks that premature infants face?

**00:28:41-9 Person 1:** The main risks are morbidity and mortality.

**00:28:44-2 Interviewer:** Can you elaborate on that?

**00:28:45-5 Person 1:** Certainly. Consider a premature baby, for instance. Even a healthy child is at risk if certain crucial steps aren't taken immediately after birth. One significant risk is infection due to their immature skin. Then there's hypoglycemia. These factors are critical in the survival of a premature infant. I should also mention hypothermia, particularly in newborns and preemies. In these babies, every system, starting from the head, is fragile and underdeveloped, making their care complex and delicate.

**00:29:29-5 Interviewer:** You also touched on the category of women most likely to have premature births, discussing socio-cultural factors and illnesses.

**00:29:46-4 Person 1:** That's correct

### Focus group of Farakan health and social promotion center

[00:30:45-0](http://localhost:2300/file=C:/Users/USER/Desktop/ENTRETIENS%20PREMATURITE/CSPS_FARAKAN/FOCUS_GROUPE-CSPS_FARAKAN.MP3time=1845000) Interviewer: Nous allons poursuivre. Quels sont les risques sanitaires particuliers auxquels les nourrissons prématurés sont confrontés en milieu urbain au Burkina Faso? [00:31:11-4](http://localhost:2300/file=C:/Users/USER/Desktop/ENTRETIENS%20PREMATURITE/CSPS_FARAKAN/FOCUS_GROUPE-CSPS_FARAKAN.MP3time=1871400)

[00:31:11-4](http://localhost:2300/file=C:/Users/USER/Desktop/ENTRETIENS%20PREMATURITE/CSPS_FARAKAN/FOCUS_GROUPE-CSPS_FARAKAN.MP3time=1871400) Person 3: Il y a la prise en charge. Parce que souvent, le problème majeur que moi je vois. Il arrive des fois qu'il soit même évacué, ce n'est même pas évacué. Il sont référés, leurs parents sont obligés de transporter l'enfant, le nouveau-né au lieu des référence parce qu'il y a manque de moyens de transport.Voilà, à défaut d'ambulance on est obligé qu'ils acheminent les nouveaux nés à moyen personnel, ça, c'est un problème. Et deuxièmement, bon, souvent il y a des femmes avec l'insecurité, elles viennent avec le linge qui n'ai même pas adapté. Voilà par exemple un tissu en nylon, ça ne peut pas vraiment envelopper l'enfant correctement.Ça, c'est ce que moi je vois surtout. [00:32:09-4](http://localhost:2300/file=C:/Users/USER/Desktop/ENTRETIENS%20PREMATURITE/CSPS_FARAKAN/FOCUS_GROUPE-CSPS_FARAKAN.MP3time=1929400)

[00:32:09-4](http://localhost:2300/file=C:/Users/USER/Desktop/ENTRETIENS%20PREMATURITE/CSPS_FARAKAN/FOCUS_GROUPE-CSPS_FARAKAN.MP3time=1929400) Interviewer: Est-ce que vous avez une idée du taux de mortalité par rapport à aux naissances prématurées ? Quelles sont les maladies auxquelles généralement les enfants prématurés sont le plus exposés? [00:32:35-7](http://localhost:2300/file=C:/Users/USER/Desktop/ENTRETIENS%20PREMATURITE/CSPS_FARAKAN/FOCUS_GROUPE-CSPS_FARAKAN.MP3time=1955700)

[00:32:35-7](http://localhost:2300/file=C:/Users/USER/Desktop/ENTRETIENS%20PREMATURITE/CSPS_FARAKAN/FOCUS_GROUPE-CSPS_FARAKAN.MP3time=1955700) Person 7: Les maladies respiratoires, Pulmonaire surtout et puis les infections néonatales. [00:32:59-3](http://localhost:2300/file=C:/Users/USER/Desktop/ENTRETIENS%20PREMATURITE/CSPS_FARAKAN/FOCUS_GROUPE-CSPS_FARAKAN.MP3time=1979300)

[00:32:59-3](http://localhost:2300/file=C:/Users/USER/Desktop/ENTRETIENS%20PREMATURITE/CSPS_FARAKAN/FOCUS_GROUPE-CSPS_FARAKAN.MP3time=1979300) Person 8: Parfois l'anemie même peut survenir sur les enfants prématurés et leur maman et c'est très grave. [00:33:17-6](http://localhost:2300/file=C:/Users/USER/Desktop/ENTRETIENS%20PREMATURITE/CSPS_FARAKAN/FOCUS_GROUPE-CSPS_FARAKAN.MP3time=1997600)

[00:33:17-6](http://localhost:2300/file=C:/Users/USER/Desktop/ENTRETIENS%20PREMATURITE/CSPS_FARAKAN/FOCUS_GROUPE-CSPS_FARAKAN.MP3time=1997600) Person 4: Il y a la malnutrition aussi, si l'enfant n'arrive pas à bien prendre le lait cela peut jouer sur sa santé. [00:33:37-1](http://localhost:2300/file=C:/Users/USER/Desktop/ENTRETIENS%20PREMATURITE/CSPS_FARAKAN/FOCUS_GROUPE-CSPS_FARAKAN.MP3time=2017100)

[00:33:37-1](http://localhost:2300/file=C:/Users/USER/Desktop/ENTRETIENS%20PREMATURITE/CSPS_FARAKAN/FOCUS_GROUPE-CSPS_FARAKAN.MP3time=2017100) Person 3: Personnellement, je n'ai pas une grande connaissance sur les maladies parce que comme nous ne sommes pas un centre de prise en charge mais bon à travers les carnets, souvent quand les femmes reviennent pour les enfants qui ont survécu, bon on dit souvent certains symptômes làbas on ne sait pas , comme par exemple, l' ictère febrile c'est surtout ça que je vois dans les carnets souvent. [00:34:24-5](http://localhost:2300/file=C:/Users/USER/Desktop/ENTRETIENS%20PREMATURITE/CSPS_FARAKAN/FOCUS_GROUPE-CSPS_FARAKAN.MP3time=2064500)

[00:34:24-5](http://localhost:2300/file=C:/Users/USER/Desktop/ENTRETIENS%20PREMATURITE/CSPS_FARAKAN/FOCUS_GROUPE-CSPS_FARAKAN.MP3time=2064500) Person 6: Selon moi, je me dis la plupart aussi, si certains prématurés c'est dû au paludisme, l'enfant peut être né avec le paludisme aussi, donc tout ça, ça peut jouer. [00:34:41-3](http://localhost:2300/file=C:/Users/USER/Desktop/ENTRETIENS%20PREMATURITE/CSPS_FARAKAN/FOCUS_GROUPE-CSPS_FARAKAN.MP3time=2081300)

00:30:45-0 Interviewer: Nous allons poursuivre. Quels sont les risques sanitaires particuliers auxquels les nourrissons prématurés sont confrontés en milieu urbain au Burkina Faso ? 00:31:11-4

00:31:11-4 Person 3: Il y a la prise en charge. Parce que souvent, le problème majeur que moi je vois. Il arrive des fois qu'il soit même évacué, ce n'est même pas évacué. Ils sont référés, leurs parents sont obligés de transporter l'enfant, le nouveau-né, au lieu des références parce qu'il y a un manque de moyens de transport. Voilà, à défaut d'ambulance, on est obligé qu'ils acheminent les nouveau-nés à moyen personnel, ça, c'est un problème. Et deuxièmement, bon, souvent il y a des femmes avec l'insécurité, elles viennent avec le linge qui n'est même pas adapté. Voilà par exemple un tissu en nylon, ça ne peut pas vraiment envelopper l'enfant correctement. Ça, c'est ce que moi je vois surtout. 00:32:09-4

00:32:09-4 Interviewer: Est-ce que vous avez une idée du taux de mortalité par rapport aux naissances prématurées ? Quelles sont les maladies auxquelles généralement les enfants prématurés sont le plus exposés ? 00:32:35-7

00:32:35-7 Person 7: Les maladies respiratoires, pulmonaires surtout, et puis les infections néonatales. 00:32:59-3

00:32:59-3 Person 8: Parfois l'anémie même peut survenir sur les enfants prématurés et leur maman, et c'est très grave. 00:33:17-6

00:33:17-6 Person 4: Il y a la malnutrition aussi, si l'enfant n'arrive pas à bien prendre le lait cela peut jouer sur sa santé. 00:33:37-1

00:33:37-1 Person 3: Personnellement, je n'ai pas une grande connaissance sur les maladies parce que comme nous ne sommes pas un centre de prise en charge, mais bon à travers les carnets, souvent quand les femmes reviennent pour les enfants qui ont survécu, bon on dit souvent certains symptômes là-bas on ne sait pas, comme par exemple, l'ictère fébrile c'est surtout ça que je vois dans les carnets souvent. 00:34:24-5

00:34:24-5 Person 6: Selon moi, je me dis que la plupart aussi, si certains prématurés c'est dû au paludisme, l'enfant peut être né avec le paludisme aussi, donc tout ça, ça peut jouer. 00:34:41-3

**00:30:45-0 Interviewer:** Let's continue. What specific health risks do premature infants face in urban areas of Burkina Faso?

**00:31:11-4 Person 3:** A major issue is the lack of adequate support and transportation. Often, premature infants need to be referred to specialized facilities, but due to the absence of ambulances, their parents must use personal means to transport them, which is a significant problem. Additionally, there's the issue of inadequate clothing for the newborns, like using nylon fabric, which isn't suitable for wrapping them properly.

**00:32:09-4 Interviewer:** Do you have any insights into the mortality rate related to premature births? What diseases are premature children most susceptible to?

**00:32:35-7 Person 7:** Premature babies are particularly prone to respiratory diseases, especially pulmonary issues, and neonatal infections.

**00:32:59-3 Person 8:** There's also a risk of anemia, both in premature children and their mothers, which can be quite serious.

**00:33:17-6 Person 4:** Malnutrition is another concern. If a child is unable to feed properly, it can severely impact their health.

**00:33:37-1 Person 3:** I don't have extensive knowledge about specific illnesses, as we're not a treatment center. However, from what I've seen in medical records, symptoms like febrile jaundice are quite common among surviving premature infants.

**00:34:24-5 Person 6:** Additionally, malaria can be a contributing factor to prematurity. If a mother has malaria, the child can be born with it, further complicating their health.

### Major of Farakan health and social promotion center

[00:11:58-6](http://localhost:2300/file=C:/Users/USER/Desktop/ENTRETIENS%20PREMATURITE/CSPS_FARAKAN/MAJOR%20FARAKAN.MP3time=718600) Interviewer: Quels sont les risques sanitaires auxquels les nourrissons prématurés sont confrontés de façon global en milieu urbain au Burkina Faso? [00:12:06-2](http://localhost:2300/file=C:/Users/USER/Desktop/ENTRETIENS%20PREMATURITE/CSPS_FARAKAN/MAJOR%20FARAKAN.MP3time=726200)

[00:12:06-2](http://localhost:2300/file=C:/Users/USER/Desktop/ENTRETIENS%20PREMATURITE/CSPS_FARAKAN/MAJOR%20FARAKAN.MP3time=726200) Person 1: Les risques sanitaires, en tout cas, un enfant prématuré c'est un enfant vulnerable, il faut en prendre bien soin pour eviter les infections, pour ne pas qu'il prenne froid,il faut bien le nourrir aussi pour qu'il puisse grandir. [00:12:35-0](http://localhost:2300/file=C:/Users/USER/Desktop/ENTRETIENS%20PREMATURITE/CSPS_FARAKAN/MAJOR%20FARAKAN.MP3time=755000)

[00:12:35-0](http://localhost:2300/file=C:/Users/USER/Desktop/ENTRETIENS%20PREMATURITE/CSPS_FARAKAN/MAJOR%20FARAKAN.MP3time=755000) Interviewer: Est ce que le risque de mortalité est élèvé chez les enfants prématurés? [00:12:39-5](http://localhost:2300/file=C:/Users/USER/Desktop/ENTRETIENS%20PREMATURITE/CSPS_FARAKAN/MAJOR%20FARAKAN.MP3time=759500)

[00:12:39-5](http://localhost:2300/file=C:/Users/USER/Desktop/ENTRETIENS%20PREMATURITE/CSPS_FARAKAN/MAJOR%20FARAKAN.MP3time=759500) Person 1: En tout cas,si on ne s'occupe pas bien de lui,il peut mourir.les infections, s'il prend froid aussi ce n'est pas bon pour lui. [00:12:53-4](http://localhost:2300/file=C:/Users/USER/Desktop/ENTRETIENS%20PREMATURITE/CSPS_FARAKAN/MAJOR%20FARAKAN.MP3time=773400)

00:11:58-6 Interviewer: Quels sont les risques sanitaires auxquels les nourrissons prématurés sont confrontés de façon globale en milieu urbain au Burkina Faso ?

00:12:06-2 Person 1: Les risques sanitaires, en tout cas, un enfant prématuré, c'est un enfant vulnérable, il faut en prendre bien soin pour éviter les infections, pour ne pas qu'il prenne froid, il faut bien le nourrir aussi pour qu'il puisse grandir.

00:12:35-0 Interviewer: Est-ce que le risque de mortalité est élevé chez les enfants prématurés ?

00:12:39-5 Person 1: En tout cas, si on ne s'occupe pas bien de lui, il peut mourir. Les infections, s'il prend froid aussi ce n'est pas bon pour lui.

**00:11:58-6 Interviewer:** Can you describe the health risks that premature infants face in urban areas of Burkina Faso?

**00:12:06-2 Person 1:** Premature infants are extremely vulnerable. They require careful attention to prevent infections and protect them from catching colds. Proper nutrition is also vital for their growth.

**00:12:35-0 Interviewer:** Is there a high risk of mortality among premature children?

**00:12:39-5 Person 1:** Yes, the risk is significant. Without proper care, their chances of survival decrease. Infections and susceptibility to colds can be particularly harmful for them.

### Maternity manager of Farakan health and social promotion center

[00:20:26-5](http://localhost:2300/file=C:/Users/USER/Desktop/ENTRETIENS%20PREMATURITE/CSPS_FARAKAN/RESPONSABLE%20MATERNITE%20FARAKAN.MP3time=1226500) Interviewer: Maintenant par rapport à une naissance prématuré.comment est le taux de mortalité et le taux de morbidité? qu'est ce que vous pouvez dire par rapport à cela? [00:20:47-5](http://localhost:2300/file=C:/Users/USER/Desktop/ENTRETIENS%20PREMATURITE/CSPS_FARAKAN/RESPONSABLE%20MATERNITE%20FARAKAN.MP3time=1247500)

[00:20:47-5](http://localhost:2300/file=C:/Users/USER/Desktop/ENTRETIENS%20PREMATURITE/CSPS_FARAKAN/RESPONSABLE%20MATERNITE%20FARAKAN.MP3time=1247500) Person 1: La mortalité est beaucoup plus élèvée. [00:20:56-8](http://localhost:2300/file=C:/Users/USER/Desktop/ENTRETIENS%20PREMATURITE/CSPS_FARAKAN/RESPONSABLE%20MATERNITE%20FARAKAN.MP3time=1256800)

[00:20:56-8](http://localhost:2300/file=C:/Users/USER/Desktop/ENTRETIENS%20PREMATURITE/CSPS_FARAKAN/RESPONSABLE%20MATERNITE%20FARAKAN.MP3time=1256800) Interviewer: Et le taux de morbidité? Est ce qu'il y a des maladies auxquelles ses enfants prématurées sont exposés? [00:21:00-9](http://localhost:2300/file=C:/Users/USER/Desktop/ENTRETIENS%20PREMATURITE/CSPS_FARAKAN/RESPONSABLE%20MATERNITE%20FARAKAN.MP3time=1260900)

[00:21:00-9](http://localhost:2300/file=C:/Users/USER/Desktop/ENTRETIENS%20PREMATURITE/CSPS_FARAKAN/RESPONSABLE%20MATERNITE%20FARAKAN.MP3time=1260900) Person 1: Oui.En tout cas ses enfants sont plus fragile par rapport à un enfant qui est né à terme.parce qu'il sera exposé aux infections [00:21:16-3](http://localhost:2300/file=C:/Users/USER/Desktop/ENTRETIENS%20PREMATURITE/CSPS_FARAKAN/RESPONSABLE%20MATERNITE%20FARAKAN.MP3time=1276300)

[00:21:16-3](http://localhost:2300/file=C:/Users/USER/Desktop/ENTRETIENS%20PREMATURITE/CSPS_FARAKAN/RESPONSABLE%20MATERNITE%20FARAKAN.MP3time=1276300) Interviewer: A quel type d'infections? [00:21:18-5](http://localhost:2300/file=C:/Users/USER/Desktop/ENTRETIENS%20PREMATURITE/CSPS_FARAKAN/RESPONSABLE%20MATERNITE%20FARAKAN.MP3time=1278500)

[00:21:18-5](http://localhost:2300/file=C:/Users/USER/Desktop/ENTRETIENS%20PREMATURITE/CSPS_FARAKAN/RESPONSABLE%20MATERNITE%20FARAKAN.MP3time=1278500) Interviewer: cela peut être des infections respiratoires. [00:21:34-3](http://localhost:2300/file=C:/Users/USER/Desktop/ENTRETIENS%20PREMATURITE/CSPS_FARAKAN/RESPONSABLE%20MATERNITE%20FARAKAN.MP3time=1294300)

00:20:26-5 Interviewer: Maintenant, par rapport à une naissance prématurée, comment est le taux de mortalité et le taux de morbidité ? Qu'est-ce que vous pouvez dire par rapport à cela ?

00:20:47-5 Person 1: La mortalité est beaucoup plus élevée.

00:20:56-8 Interviewer: Et le taux de morbidité ? Est-ce qu'il y a des maladies auxquelles ces enfants prématurés sont exposés ?

00:21:00-9 Person 1: Oui. En tout cas, ces enfants sont plus fragiles par rapport à un enfant qui est né à terme parce qu'il sera exposé aux infections.

00:21:16-3 Interviewer: À quel type d'infections ?

00:21:18-5 Interviewer: Cela peut être des infections respiratoires.

**00:20:26-5 Interviewer:** Now, in comparison to full-term births, how do the mortality and morbidity rates for premature births differ? What can you say about this?

**00:20:47-5 Person 1:** The mortality rate for premature infants is significantly higher.

**00:20:56-8 Interviewer:** And what about the morbidity rate? Are there specific diseases that these premature children are more susceptible to?

**00:21:00-9 Person 1:** Yes, premature children are more fragile compared to full-term infants, making them more prone to infections.

**00:21:16-3 Interviewer:** Can you specify the types of infections they are prone to?

**00:21:18-5 Person 1:** Respiratory infections are a common risk for these infants.

# 10. Specificities of mothers of premature children

### Focus group of Accart-ville urban medical center

[00:28:42-7](http://localhost:2300/file=C:/Users/USER/Desktop/ENTRETIENS%20PREMATURITE/CSPS-ACCART-VILLE/FOCUS%20GROUPE%20ACCART-VILLE-BON.mp3time=1722700) Interviewer: ok nous allons poursuivre, Avez-vous remarqué des particularités spécifiques concernant les mères des enfants prématures ? est ce que c'est une certaine categorie de femmes données qui donnent naissances aux enfants prématurés?

[00:29:26-1](http://loca)Person 3: : ce qu'on peut dire,la plupart c'est la pauvrété. la prematurité est constaté dans les menages dont les revenues ne sont pas bons c'est à dire financièrement posées de façon génerale.on rencontre egalement d'autres categories de femmes mais la plupart c'est là ou la pauvrété sevit le plus qu'il y a plus de prematurités.

[00:30:14-2](http://localhost:2300/file=C) Interviewer: est ce qu'il y a quelque chose d'autre à ajouter?

[00:30:21-3](http://localhost:2300/file=C:/Users/USER/Desktop/ENTRETIEN)Person 3: il y a egalement les milieux ou l'hygiène, la promiscuité, l'état de santé de la maman , la malnutrition font defauts donc on constate que les femmes qui donnent naissances aux prematurés ont un niveau socio-economique très bas.

[00:30:47-7](http://localhost:2300/file=C:/Users/USER/Desktop/ENTRETIENS%20PREMATURITE/CSPS-ACCART-VILLE/FOCUS%20GROUPE%20ACCART-VILLE-BON.mp3time=1847700) Interviewer: et par rapport à la tranche d'âge?

[00:30:53-0](http://localhost:2300/file=C:/Users/USER/Desktop/ENTRETIENS%20PREMATURITE/CSPS-ACCART-VILLE/FOCUS%20GROUPE%20ACCART-VILLE-BON.mp3time=1853000)Person 1: : la tranche d'âge oui, nous constatons aussi que les primipares font plus de prematurités par rapport aux multipares. par rapport à l'âge aussi on constate que les femmes qui ont moins de 18 ans en font beaucoup plus aussi de prématurés que les femmes qui ont plus de 18 ans.

[00:31:18-4](http://localhost:2) Interviewer: autres observations? nous savons que vous etes fatigués mais faites un effort.

00:28:42-7 Interviewer: Ok, nous allons poursuivre. Avez-vous remarqué des particularités spécifiques concernant les mères des enfants prématurés ? Est-ce qu'il y a une certaine catégorie de femmes qui donnent naissance aux enfants prématurés ?

00:29:26-1 Person 3: Ce qu'on peut dire, la plupart c'est la pauvreté. La prématurité est constatée dans les ménages dont les revenus ne sont pas bons, c'est-à-dire financièrement posés de façon générale. On rencontre également d'autres catégories de femmes mais la plupart c'est là où la pauvreté sévit le plus qu'il y a plus de prématurités.

00:30:14-2 Interviewer: Est-ce qu'il y a quelque chose d'autre à ajouter ?

00:30:21-3 Person 3: Il y a également les milieux où l'hygiène, la promiscuité, l'état de santé de la maman, la malnutrition font défauts donc on constate que les femmes qui donnent naissance aux prématurés ont un niveau socio-économique très bas.

00:30:47-7 Interviewer: Et par rapport à la tranche d'âge ?

00:30:53-0 Person 1: La tranche d'âge oui, nous constatons aussi que les primipares font plus de prématurités par rapport aux multipares. Par rapport à l'âge aussi on constate que les femmes qui ont moins de 18 ans en font beaucoup plus aussi de prématurés que les femmes qui ont plus de 18 ans.

00:31:18-4 Interviewer: Autres observations ? Nous savons que vous êtes fatigués mais faites un effort.

**00:28:42-7 Interviewer:** Okay, moving on. Have you noticed any particular characteristics among mothers of premature infants? Is there a specific category of women who tend to give birth prematurely?

**00:29:26-1 Person 3:** What we often see is a link to poverty. Prematurity is more common in households with lower incomes or financial instability. While there are exceptions, the majority of premature births occur in areas where poverty is more prevalent.

**00:30:14-2 Interviewer:** Is there anything else you would like to add?

**00:30:21-3 Person 3:** Another factor is the living environment, including issues like poor hygiene, crowded living conditions, and the mother's health status, especially malnutrition. So, we observe that women giving birth to premature babies often come from very low socio-economic backgrounds.

**00:30:47-7 Interviewer:** What about the age of these mothers?

**00:30:53-0 Person 1:** Regarding age, we notice that primiparas, or first-time mothers, tend to have more premature births compared to multiparas, or mothers who have had multiple births. Also, women under 18 years old have a higher incidence of premature births compared to those over 18.

**00:31:18-4 Interviewer:** Any other observations? We understand you're tired, but please, share what you can.

### Manager of Accart-ville urban medical center

[00:18:59-2](http://localhost:2300/file=C:/Users/USER/Desktop/ENTRETIENS%20PREMATURITE/CSPS-ACCART-VILLE/RESPONSABLE%20CMU%20ACCART-VILLE.MP3time=1139200) Interviewer: Est ce que vous avez remarqué des particularités spécifiques concernant les mères des enfants prématurés? Est ce qu'elles viennent d'un certain type de milieu? Est ce qu'elles sont jeunes? Quel est leur état de santé? Est ce que vous avez remarqué des particularités au niveau des mères des enfants prématurés? [00:19:24-0](http://localhost:2300/file=C:/Users/USER/Desktop/ENTRETIENS%20PREMATURITE/CSPS-ACCART-VILLE/RESPONSABLE%20CMU%20ACCART-VILLE.MP3time=1164000)

[00:19:24-0](http://localhost:2300/file=C:/Users/USER/Desktop/ENTRETIENS%20PREMATURITE/CSPS-ACCART-VILLE/RESPONSABLE%20CMU%20ACCART-VILLE.MP3time=1164000)Person 1: Bon au niveau de mon centre il faut dire non, pas tellement, mais dans mon expérience, je veux dire oui, puisque généralement au cours de mes stages que j'ai eu à faire, bon, j'ai constaté finalement les prématurés c'etait soient à des mamans qui faisaient le paludisme presque tout le temps qui étaient anémiés ou soit des mamans qui avaient une infection génitale qui a entrainé, c'est à dire une infection au niveau intime et génitale, qui est monté et entraîner la prématurité, soit des mamans qui avaient des maladies telles que une préeclampsie qui est entrée avec la tension on est obligée de faire l'accouchement. ce sont des facteurs que j'avais observé au moins dans mon expérience. [00:20:16-9](http://localhost:2300/file=C:/Users/USER/Desktop/ENTRETIENS%20PREMATURITE/CSPS-ACCART-VILLE/RESPONSABLE%20CMU%20ACCART-VILLE.MP3time=1216900)

[00:20:16-9](http://localhost:2300/file=C:/Users/USER/Desktop/ENTRETIENS%20PREMATURITE/CSPS-ACCART-VILLE/RESPONSABLE%20CMU%20ACCART-VILLE.MP3time=1216900)Interviewer: Maintenant, toujours concernant l'impact de la prématurité sur la vie de la mère en communauté. Est ce que vous pensez que c'est un facteur de risque également pour la prise en charge. [00:20:29-8](http://localhost:2300/file=C:/Users/USER/Desktop/ENTRETIENS%20PREMATURITE/CSPS-ACCART-VILLE/RESPONSABLE%20CMU%20ACCART-VILLE.MP3time=1229800)

[00:20:29-8](http://localhost:2300/file=C:/Users/USER/Desktop/ENTRETIENS%20PREMATURITE/CSPS-ACCART-VILLE/RESPONSABLE%20CMU%20ACCART-VILLE.MP3time=1229800)Person 1: généralement. Bon, quand tu dis les prématurité faut pour les protéger, en fait les gens ne comprennent pas. Peut être il peut être un prématuré qui est un peu bien portant, qui nécessite pas une hospitalisation, tu vas dire de le protéger, de ne pas le laver comme ça, de l'essuyer seulement, les parents en fait, avec nos traditions, ils vont dire qu'ils vont aller le laver, ils vont le gaver. Donc tous ceci en fait devient un facteur de risque pour lui même, pour l'enfant en quelque sorte. Puisqu'il est déjà fragile donc c'est ça même qui peut faire qu'il fasse des infections associées. Il y a des gens qui vont les raser. On dit de ne pas les raser, d'attendre un peu à rien. Ils vont aller les raser et pourtant il est très fragile. [00:21:18-8](http://localhost:2300/file=C:/Users/USER/Desktop/ENTRETIENS%20PREMATURITE/CSPS-ACCART-VILLE/RESPONSABLE%20CMU%20ACCART-VILLE.MP3time=1278800)

00:18:59-2 Interviewer: Est-ce que vous avez remarqué des particularités spécifiques concernant les mères des enfants prématurés ? Est-ce qu'elles viennent d'un certain type de milieu ? Est-ce qu'elles sont jeunes ? Quel est leur état de santé ? Est-ce que vous avez remarqué des particularités au niveau des mères des enfants prématurés ? 00:19:24-0

00:19:24-0 Personne 1: Bon, au niveau de mon centre, il faut dire non, pas tellement, mais dans mon expérience, je veux dire oui, puisque généralement, au cours de mes stages que j'ai eu à faire, bon, j'ai constaté finalement les prématurés c'était soit à des mamans qui faisaient le paludisme presque tout le temps, qui étaient anémiées, ou soit des mamans qui avaient une infection génitale qui a entraîné, c'est-à-dire une infection au niveau intime et génitale, qui est montée et a entraîné la prématurité, soit des mamans qui avaient des maladies telles que la prééclampsie qui est entrée avec la tension, on est obligée de faire l'accouchement. Ce sont des facteurs que j'avais observés au moins dans mon expérience. 00:20:16-9

00:20:16-9 Interviewer: Maintenant, toujours concernant l'impact de la prématurité sur la vie de la mère en communauté. Est-ce que vous pensez que c'est un facteur de risque également pour la prise en charge ? 00:20:29-8

00:20:29-8 Personne 1: Généralement. Bon, quand tu dis les prématurités, faut pour les protéger, en fait les gens ne comprennent pas. Peut-être il peut être un prématuré qui est un peu bien portant, qui ne nécessite pas une hospitalisation, tu vas dire de le protéger, de ne pas le laver comme ça, de l'essuyer seulement, les parents en fait, avec nos traditions, ils vont dire qu'ils vont aller le laver, ils vont le gaver. Donc tout ceci en fait devient un facteur de risque pour lui-même, pour l'enfant en quelque sorte. Puisqu'il est déjà fragile, donc c'est ça même qui peut faire qu'il fasse des infections associées. Il y a des gens qui vont les raser. On dit de ne pas les raser, d'attendre un peu à rien. Ils vont aller les raser, et pourtant il est très fragile. 00:21:18-8

**00:18:59-2 Interviewer:** Have you noticed any specific characteristics among mothers of premature children? Do they come from a particular background or age group? What about their health status? Any particularities you've observed?

**00:19:24-0 Person 1:** In my experience, I have noticed certain patterns. During my internships, I observed that premature births often involved mothers who had malaria, were anemic, or had genital infections that led to rising infections and ultimately prematurity. Others had conditions like preeclampsia, which necessitated early delivery. These are some of the factors I've observed.

**00:20:16-9 Interviewer:** Regarding the impact of prematurity on a mother's life in the community, do you think this affects treatment?

**00:20:29-8 Person 1:** Often, there is a lack of understanding about how to care for a premature baby. For example, if a premature baby is in relatively good health and doesn't require hospitalization, we advise protective measures like not washing them too rigorously, only wiping them. However, due to traditional practices, parents may not follow this advice. They might bathe the baby extensively or force-feed them. Such practices can become risk factors, leading to associated infections. We advise against shaving them, as their skin is very fragile, but often this advice is ignored.

### Maternity manager of Accart-ville urban medical center

[00:21:25-9](http://localhost:2300/file=C:/Users/USER/Desktop/ENTRETIENS%20PREMATURITE/CSPS-ACCART-VILLE/RESPONSABLE%20MATERNITE%20CMU%20ACCART-VILLE.MP3time=1285900) Interviewer: ok. est ce que vous avez remarqué des particularités spécifiques concernant les mères des enfants prématurés ?

[00:21:35-1](http://localhost:2300/file=C:/Users/USER/Desktop/ENTRETIENS%20PREMATURITE/CSPS-ACCART-VILLE/RESPONSABLE%20MATERNITE%20CMU%20ACCART-VILLE.MP3time=1295100) Person 1: leurs particularités c'est l'angoisse, elles sont angoissées, elles se sentent coupables d'avoir donner naissance à un enfant prématuré, certaines même vont vous posez la question pourquoi elle a accouché avant terme? elles se sentent coupables de ce qui est arrivé ,elle est angoissé et elle doute même de la viabilité de son enfant, est ce que l'enfant serait viable? est ce que l'enfant va s'en sortir? se sont ces questions qui reviennent très souvent.Nous sommes obligés de la rassurer et de lui dire de suivre les conseils qu'on va donner.

[00:22:19-9](http://localhost:2300/file=C:/Users/USER/Desktop/ENTRETIENS%20PREMATURITE/CSPS-ACCART-VILLE/RESPONSABLE%20MATERNITE%20CMU%20ACCART-VILLE.MP3time=1339900) Interviewer: est ce que ses femmes viennent d'un certain type de milieu? de quelle tranche d'âge généralement sont elles?

[00:22:30-4](http://localhost:2300/file=C:/Users/USER/Desktop/ENTRETIENS%20PREMATURITE/CSPS-ACCART-VILLE/RESPONSABLE%20MATERNITE%20CMU%20ACCART-VILLE.MP3time=1350400) Person 1: on rencontre toutes les couches socio-professionnelles mais en générale les couches les plus frequemments rencontrées sont celles ou la pauvrété sévi c'est à dire là ou le niveau de vie socio-economique est très faible on a plus de cas de prématurités.il y a aussi des cas de prematurités dans les milieux ou la femme fait beaucoup de travaux pénibles pendant sa grossesse et comme dans nos registre la profession ressort, la plupart ce sont des femmes ménagères.en ce qui concerne l'âge ce sont les primipares pour la plupart et les femmes qui ont moins de 18 ans qui font plus de prematurités.

[00:23:37-2](http://localhost:2300/file=C:/Users/USER/Desktop/ENTRETIENS%20PREMATURITE/CSPS-ACCART-VILLE/RESPONSABLE%20MATERNITE%20CMU%20ACCART-VILLE.MP3time=1417200)Interviewer: quand vous dites primipares vous faites allusions à quel type de femme?

[00:23:38-8](http://localhost:2300/file=C:/Users/USER/Desktop/ENTRETIENS%20PREMATURITE/CSPS-ACCART-VILLE/RESPONSABLE%20MATERNITE%20CMU%20ACCART-VILLE.MP3time=1418800) Person 1: des femmes qui accouchent pour la première fois leur première grossesse.

[00:23:44-1](http://localhost:2300/file=C:/Users/USER/Desktop/ENTRETIENS%20PREMATURITE/CSPS-ACCART-VILLE/RESPONSABLE%20MATERNITE%20CMU%20ACCART-VILLE.MP3time=1424100) Interviewer: est ce que ses femmes ont des conditions de santé assez particulières?

[00:23:49-7](http://localhost:2300/file=C:/Users/USER/Desktop/ENTRETIENS%20PREMATURITE/CSPS-ACCART-VILLE/RESPONSABLE%20MATERNITE%20CMU%20ACCART-VILLE.MP3time=1429700) Person 1: le plus souvent s'il y a une prematurité, en nous basant sur les facteurs favorisant la prematurité, on trouve qu'il y a les infections maternelles, c'est à dire que ces femmes vont generalement presenter des infections parce que les CPN ne sont pas de bonnes qualités vue le nombre qu'on prend par jour c'est assez difficile. nous pouvons faire plus de quarante (40) CPN par jour. la sage femme ne peut pas etre efficace pour prendre plus de 40 personnes en charge c'est à dire rechercher les pathologies qui peuvent affecter la grossesse comme les infections urinaires, les vaginoses bacteriennes et aussi echanger avec la femme pour comprendre son milieu de vie, est ce qu'elle est stréssé? est e qu'elle a des difficultés particulières en communauté? et essayer de rencontrer le mari pour echanger avec lui afin d'eviter que l'accouchement prematuré n'intervienne. ce qu'on remarque en general se sont les infections urinaires qui sont à l'origine des accouchements prematurés [00:25:09-2](http://localhost:2300/file=C:/Users/USER/Desktop/ENTRETIENS%20PREMATURITE/CSPS-ACCART-VILLE/RESPONSABLE%20MATERNITE%20CMU%20ACCART-VILLE.MP3time=1509200)

[00:25:09-2](http://localhost:2300/file=C:/Users/USER/Desktop/ENTRETIENS%20PREMATURITE/CSPS-ACCART-VILLE/RESPONSABLE%20MATERNITE%20CMU%20ACCART-VILLE.MP3time=1509200) Interviewer: c'est dire que ses infections ne sont pas apparents au contact de la sage femme lors de leur CPN à cause d'une contrainte de temps pour pousser les investigations aussi loin afin de detecter ses pathologies tôt? [00:25:24-7](http://localhost:2300/file=C:/Users/USER/Desktop/ENTRETIENS%20PREMATURITE/CSPS-ACCART-VILLE/RESPONSABLE%20MATERNITE%20CMU%20ACCART-VILLE.MP3time=1524700)

[00:25:24-7](http://localhost:2300/file=C:/Users/USER/Desktop/ENTRETIENS%20PREMATURITE/CSPS-ACCART-VILLE/RESPONSABLE%20MATERNITE%20CMU%20ACCART-VILLE.MP3time=1524700)Person 1: oui il y a ce volet mais il y a aussi le volet des examens complementaires puisque le centre ne dispose pas de laboratoire. il y a des pays ou le test des infections urinaires est systematique pourtant au Burkina nous n'avons pas cela, le test n'est pas systematique ce qui fait que la sage femme peut ne pas deceler l'infection. en plus se sont des examens qui sont payants beaucoup n'ont pas les moyens de le faire donc la sage femme ne peut pas faire un diagnostique plus efficace afin de prendre en charge pour eviter la survenue de l'accouchement prematuré [00:26:04-4](http://localhost:2300/file=C:/Users/USER/Desktop/ENTRETIENS%20PREMATURITE/CSPS-ACCART-VILLE/RESPONSABLE%20MATERNITE%20CMU%20ACCART-VILLE.MP3time=1564400)

00:21:25-9 Interviewer: Ok. Est-ce que vous avez remarqué des particularités spécifiques concernant les mères des enfants prématurés ?

00:21:35-1 Person 1: Leurs particularités c'est l'angoisse, elles sont angoissées, elles se sentent coupables d'avoir donné naissance à un enfant prématuré, certaines même vont vous poser la question pourquoi elles ont accouché avant terme ? Elles se sentent coupables de ce qui est arrivé, elle est angoissée et elle doute même de la viabilité de son enfant, est-ce que l'enfant sera viable ? Est-ce que l'enfant va s'en sortir ? Ce sont ces questions qui reviennent très souvent. Nous sommes obligés de les rassurer et de leur dire de suivre les conseils qu'on va donner.

00:22:19-9 Interviewer: Est-ce que ces femmes viennent d'un certain type de milieu ? De quelle tranche d'âge généralement sont-elles ?

00:22:30-4 Person 1: On rencontre toutes les couches socio-professionnelles mais en général les couches les plus fréquemment rencontrées sont celles où la pauvreté sévit, c'est-à-dire là où le niveau de vie socio-économique est très faible on a plus de cas de prématurités. Il y a aussi des cas de prématurités dans les milieux où la femme fait beaucoup de travaux pénibles pendant sa grossesse et comme dans nos registres la profession ressort, la plupart ce sont des femmes ménagères. En ce qui concerne l'âge ce sont les primipares pour la plupart et les femmes qui ont moins de 18 ans qui font plus de prématurités.

00:23:37-2 Interviewer: Quand vous dites primipares, vous faites allusion à quel type de femme ?

00:23:38-8 Person 1: Des femmes qui accouchent pour la première fois, leur première grossesse.

00:23:44-1 Interviewer: Est-ce que ces femmes ont des conditions de santé assez particulières ?

00:23:49-7 Person 1: Le plus souvent s'il y a une prématurité, en nous basant sur les facteurs favorisant la prématurité, on trouve qu'il y a les infections maternelles, c'est-à-dire que ces femmes vont généralement présenter des infections parce que les CPN ne sont pas de bonnes qualités vu le nombre qu'on prend par jour c'est assez difficile. Nous pouvons faire plus de quarante (40) CPN par jour. La sage-femme ne peut pas être efficace pour prendre plus de 40 personnes en charge, c'est-à-dire rechercher les pathologies qui peuvent affecter la grossesse comme les infections urinaires, les vaginoses bactériennes et aussi échanger avec la femme pour comprendre son milieu de vie, est-ce qu'elle est stressée ? Est-ce qu'elle a des difficultés particulières en communauté ? Et essayer de rencontrer le mari pour échanger avec lui afin d'éviter que l'accouchement prématuré n'intervienne. Ce qu'on remarque en général ce sont les infections urinaires qui sont à l'origine des accouchements prématurés. 00:25:09-2

00:25:09-2 Interviewer: C'est dire que ces infections ne sont pas apparentes au contact de la sage-femme lors de leur CPN à cause d'une contrainte de temps pour pousser les investigations aussi loin afin de détecter ces pathologies tôt ? 00:25:24-7

00:25:24-7 Person 1: Oui, il y a ce volet mais il y a aussi le volet des examens complémentaires puisque le centre ne dispose pas de laboratoire. Il y a des pays où le test des infections urinaires est systématique, pourtant au Burkina nous n'avons pas cela, le test n'est pas systématique, ce qui fait que la sage-femme peut ne pas déceler l'infection. En plus ce sont des examens qui sont payants, beaucoup n'ont pas les moyens de les faire, donc la sage-femme ne peut pas faire un diagnostic plus efficace afin de prendre en charge pour éviter la survenue de l'accouchement prématuré. 00:26:04-4

**00:21:25-9 Interviewer:** Ok. Have you noticed any specific characteristics among mothers of premature children?

**00:21:35-1 Person 1:** These mothers often experience anxiety and guilt, questioning why they gave birth prematurely. They worry about the viability and survival of their child. We need to reassure them and encourage them to follow our advice.

**00:22:19-9 Interviewer:** Do these women come from a particular background? What age group do they typically belong to?

**00:22:30-4 Person 1:** We see mothers from all socio-professional strata, but prematurity is most common in environments where poverty is prevalent. Many are housewives who engage in heavy physical work during pregnancy. Regarding age, prematurity is mostly seen in first-time mothers and women under 18.

**00:23:37-2 Interviewer:** When you say 'primiparous', are you referring to women who are pregnant for the first time?

**00:23:38-8 Person 1:** Yes, women experiencing their first pregnancy.

**00:23:44-1 Interviewer:** Do these women have specific health conditions?

**00:23:49-7 Person 1:** Often, maternal infections are a factor in prematurity. Due to the high number of prenatal consultations we handle daily, more than forty, it's challenging for a midwife to effectively screen for pathologies that can affect pregnancy, such as urinary infections or bacterial vaginosis. We also try to understand their living conditions and stress levels, and discuss with their partners to prevent premature delivery. Urinary infections are a common cause of premature births.

**00:25:09-2 Interviewer:** Are you saying that these infections might go undetected during prenatal consultations due to time constraints and lack of thorough investigations?

**00:25:24-7 Person 1:** Yes, there's the time constraint, but also the lack of necessary laboratory facilities. In some countries, testing for urinary infections is routine, but in Burkina, it's not. This means that midwives may not detect the infection. Additionally, these tests are not free, and many women can't afford them, limiting the midwife's ability to make an effective diagnosis and prevent premature births.

### Focus group of Do medical center with surgical branch

[00:21:33-8](http://localhost:2300/file=C:/Users/USER/Desktop/ENTRETIENS%20PREMATURITE/CMA-DO/FOCUS%20GROUPE%20CMA-DO.mp3time=1293800) Interviewer: Est ce que vous avez remarqué des particularités concernant les mères des enfants prématurés? Est ce qu'elles viennent d'un certain type de milieu socio-économique? sont elles plus jeunes ou plus âgées? Ont elles des conditions de santé particulières? Est ce qu'il y a des particularités que vous avez remarqué au niveau des femmes qui donnent naissances aux enfants prématurés? [00:22:00-4](http://localhost:2300/file=C:/Users/USER/Desktop/ENTRETIENS%20PREMATURITE/CMA-DO/FOCUS%20GROUPE%20CMA-DO.mp3time=1320400)

[00:22:00-4](http://localhost:2300/file=C:/Users/USER/Desktop/ENTRETIENS%20PREMATURITE/CMA-DO/FOCUS%20GROUPE%20CMA-DO.mp3time=1320400) Person 9:Oui la maladie ressort parce que certaines pathologies obstericales entrainent forcement un accouchement prématuré, maintenant concernant l'âge, toutes les femmes sont prédisposées. [00:22:28-1](http://localhost:2300/file=C:/Users/USER/Desktop/ENTRETIENS%20PREMATURITE/CMA-DO/FOCUS%20GROUPE%20CMA-DO.mp3time=1348100)

00:21:33-8 Interviewer: Est-ce que vous avez remarqué des particularités concernant les mères des enfants prématurés ? Est-ce qu'elles viennent d'un certain type de milieu socio-économique ? Sont-elles plus jeunes ou plus âgées ? Ont-elles des conditions de santé particulières ? Est-ce qu'il y a des particularités que vous avez remarqué au niveau des femmes qui donnent naissance aux enfants prématurés ? 00:22:00-4

00:22:00-4 Person 9: Oui, la maladie ressort parce que certaines pathologies obstétricales entraînent forcément un accouchement prématuré, maintenant concernant l'âge, toutes les femmes sont prédisposées. 00:22:28-1

**00:21:33-8 Interviewer:** Have you observed any specific characteristics among mothers of premature children? Do they come from a particular socio-economic background, or do they tend to be younger or older? Do they have any special health conditions? Are there any notable patterns among these women?

**00:22:00-4 Person 9:** Yes, certain health conditions stand out. Some obstetric pathologies almost invariably lead to premature delivery. As for age, it seems that all women, regardless of their age, are susceptible to these risks.

### Gynecologist 1

[00:13:09-7](http://localhost:2300/file=C:/Users/USER/Desktop/ENTRETIENS%20PREMATURITE/CMA-DO/GENYCOLOGUE%2001.mp3time=789700) Interviewer: Est-ce que vous avez remarqué des particularités au niveau des mères des enfants prématurés ? Sont elles plus jeunes? viennent elles d'un certain type de milieu socio-economique? est-ce qu'il y a des particularités ? [00:13:24-5](http://localhost:2300/file=C:/Users/USER/Desktop/ENTRETIENS%20PREMATURITE/CMA-DO/GENYCOLOGUE%2001.mp3time=804500)

[00:13:24-5](http://localhost:2300/file=C:/Users/USER/Desktop/ENTRETIENS%20PREMATURITE/CMA-DO/GENYCOLOGUE%2001.mp3time=804500) Person 1: Ce que nous remarquons ici, ça vient de tous les milieux et tous les âges également. [00:13:33-9](http://localhost:2300/file=C:/Users/USER/Desktop/ENTRETIENS%20PREMATURITE/CMA-DO/GENYCOLOGUE%2001.mp3time=813900)

00:13:09-7 Interviewer: Est-ce que vous avez remarqué des particularités au niveau des mères des enfants prématurés ? Sont-elles plus jeunes ? Viennent-elles d'un certain type de milieu socio-économique ? Est-ce qu'il y a des particularités ? 00:13:24-5

00:13:24-5 Person 1: Ce que nous remarquons ici, ça vient de tous les milieux et tous les âges également. 00:13:33-9

**00:13:09-7 Interviewer:** Have you observed any specific characteristics among mothers of premature infants? Are they typically younger? Do they come from a certain socio-economic background? Are there any notable patterns?

**00:13:24-5 Person 1:** From what we've noticed, prematurity affects mothers from all socio-economic backgrounds and ages equally. There doesn't seem to be a particular trend or specific demographic that is more affected than others in our observations

### Gynecologist 2

[00:23:24-8](http://localhost:2300/file=C:/Users/USER/Desktop/ENTRETIENS%20PREMATURITE/CMA-DO/GENYCOLOGUE%2002.mp3time=1404800) Interviewer: Est-ce que vous avez remarqué des particularités spécifiques au niveau des mères qui donnent naissance à des enfants prématurés ? Est-ce qu'elles viennent d'un certain type de milieu social ?Est-ce qu'elles sont plus âgées, plus jeunes ? Est-ce qu'elles sont assujeties à certaines types de maladies ? Est-ce qu'il y a des particularités ici ? [00:23:45-4](http://localhost:2300/file=C:/Users/USER/Desktop/ENTRETIENS%20PREMATURITE/CMA-DO/GENYCOLOGUE%2002.mp3time=1425400)

[00:23:45-4](http://localhost:2300/file=C:/Users/USER/Desktop/ENTRETIENS%20PREMATURITE/CMA-DO/GENYCOLOGUE%2002.mp3time=1425400) Person 1: Alors la prématurité se retrouve dans tous les milieux. La prématurité va se retrouver dans tous les milieux, aussi bien chez les classes sociales aisées que chez les classes sociales défavorisées. Mais ce qu'on remarque le plus, c'est souvent lié à certaines maladies. Par exemple dans notre système ici, c'est très souvent lié à la pré-éclampsie. Et ça c'est l'hypertension artérielle qui va se développer au cours de la grossesse. On a beaucoup de prématurité dans cette catégorie de maladies. Alors que cette patologie se retrouve aussi bien chez les classes défavorisées que chez les classes favorisées en général. Maintenant pour ce qui concerne les problèmes infectieux, là souvent on se retrouve dans les classes sociales beaucoup plus défavorisées. Donc ce sont les femmes qui n'ont pas fait de CPN, qui n'ont pas eu de traitement préventif contre le paludisme, qui n'ont pas été en consultation, qui n'ont pas eu de suppléments en nutriments, notamment les fer et les autres vitamines. Ou alors ce sont les femmes qui ont travaillé beaucoup pendant la grossesse, si bien qu'à un moment donné, peut-être la poche des os va se rompre ou bien la femme va accoucher de façon prématurée parce qu'elle-même physiquement est faible. On retrouve également la prématurité dans les grossesses multiples, c'est-à-dire la grossesse gemellaire, quand il s'agit de deux bébés ou bien plus de deux bébés. Donc lié à la tension abdominale, c'est-à-dire que la grossesse va se distendre jusqu'à... l'abdomen va se distendre jusqu'à une certaine proportion. Et puis le poids peut faire en sorte que de façon mécanique la poche se rompt et puis on assiste à un accouchement prématuré. Et ça, ça se retrouve dans tous les milieux. Si on essaie de résumer, si on fait la part des choses, les problèmes infectieux se retrouvent beaucoup plus dans les classes défavorisées, souvent qui n'ont pas les moyens de prendre en charge ou bien qui ont négligé leur état de santé à un moment donné. Mais s'il s'agit de la pré-éclampsie ou bien des grossesses multiples, ça se retrouve chez tout le monde. [00:25:55-0](http://localhost:2300/file=C:/Users/USER/Desktop/ENTRETIENS%20PREMATURITE/CMA-DO/GENYCOLOGUE%2002.mp3time=1555000)

00:23:24-8 Interviewer: Est-ce que vous avez remarqué des particularités spécifiques au niveau des mères qui donnent naissance à des enfants prématurés ? Est-ce qu'elles viennent d'un certain type de milieu social ? Est-ce qu'elles sont plus âgées, plus jeunes ? Est-ce qu'elles sont assujetties à certains types de maladies ? Est-ce qu'il y a des particularités ici ? 00:23:45-4

00:23:45-4 Person 1: Alors la prématurité se retrouve dans tous les milieux. La prématurité va se retrouver dans tous les milieux, aussi bien chez les classes sociales aisées que chez les classes sociales défavorisées. Mais ce qu'on remarque le plus, c'est souvent lié à certaines maladies. Par exemple dans notre système ici, c'est très souvent lié à la prééclampsie. Et ça, c'est l'hypertension artérielle qui va se développer au cours de la grossesse. On a beaucoup de prématurité dans cette catégorie de maladies. Alors que cette pathologie se retrouve aussi bien chez les classes défavorisées que chez les classes favorisées en général. Maintenant pour ce qui concerne les problèmes infectieux, là souvent on se retrouve dans les classes sociales beaucoup plus défavorisées. Donc ce sont les femmes qui n'ont pas fait de CPN, qui n'ont pas eu de traitement préventif contre le paludisme, qui n'ont pas été en consultation, qui n'ont pas eu de suppléments en nutriments, notamment le fer et les autres vitamines. Ou alors ce sont les femmes qui ont travaillé beaucoup pendant la grossesse, si bien qu'à un moment donné, peut-être la poche des eaux va se rompre ou bien la femme va accoucher de façon prématurée parce qu'elle-même physiquement est faible. On retrouve également la prématurité dans les grossesses multiples, c'est-à-dire la grossesse gémellaire, quand il s'agit de deux bébés ou bien plus de deux bébés. Donc lié à la tension abdominale, c'est-à-dire que la grossesse va se distendre jusqu'à... l'abdomen va se distendre jusqu'à une certaine proportion. Et puis le poids peut faire en sorte que de façon mécanique la poche se rompt et puis on assiste à un accouchement prématuré. Et ça, ça se retrouve dans tous les milieux. Si on essaie de résumer, si on fait la part des choses, les problèmes infectieux se retrouvent beaucoup plus dans les classes défavorisées, souvent qui n'ont pas les moyens de prendre en charge ou bien qui ont négligé leur état de santé à un moment donné. Mais s'il s'agit de la prééclampsie ou bien des grossesses multiples, ça se retrouve chez tout le monde. 00:25:55-0

**00:23:24-8 Interviewer:** Have you noticed any specific characteristics among mothers who give birth to premature children? Do they come from a certain social background? Are they typically older or younger? Are they prone to certain types of illnesses? Any particularities in this context?

**00:23:45-4 Person 1:** Prematurity occurs across all social strata. We see premature births in both wealthy and disadvantaged social classes. However, a common factor we often observe is linked to certain illnesses. For example, in our system, prematurity is frequently associated with preeclampsia, a condition characterized by high blood pressure developing during pregnancy. This condition leads to a lot of premature births and is found across all social classes.In terms of infectious problems, these are more commonly seen in the disadvantaged social classes. These women often miss antenatal care (ANC), lack preventive treatments against malaria, and do not receive consultations or necessary nutrient supplements like iron and other vitamins. Sometimes, they are women who have physically exerted themselves during pregnancy, leading to premature labor due to physical weakness or premature rupture of membranes.Prematurity is also common in multiple pregnancies, such as twins or more, which can lead to increased abdominal tension and cause the sac to mechanically rupture, resulting in premature birth. This can happen in any social class. To summarize, while infectious problems leading to prematurity are more prevalent in disadvantaged classes, conditions like preeclampsia and complications from multiple pregnancies are found across all social backgrounds.

### Chief physician of Do health district

[00:23:15-2](http://localhost:2300/file=C:/Users/USER/Desktop/ENTRETIENS%20PREMATURITE/CMA-DO/MCD-DO.mp3time=1395200) Interviewer: Est-ce que vous avez remarqué des particularités spécifiques aux femmes qui donnent naissance aux enfants prématurés? Est-ce qu'elles viennent d'un certain type de milieu? Est-ce qu'elles sont d'une certaine tranche d'âge? Est-ce qu'elles sont assujetties à un certain type de maladie? Est-ce qu'il y a des particularités que vous avez remarquées au niveau de ses mères? [00:23:31-1](http://localhost:2300/file=C:/Users/USER/Desktop/ENTRETIENS%20PREMATURITE/CMA-DO/MCD-DO.mp3time=1411100)

[00:23:31-1](http://localhost:2300/file=C:/Users/USER/Desktop/ENTRETIENS%20PREMATURITE/CMA-DO/MCD-DO.mp3time=1411100) Person 1: Je n'ai pas fait ce retour. Je n'ai pas fait ce retour parce que je ne l'ai pas checké. Je n'ai pas vu de particularité. [00:23:40-3](http://localhost:2300/file=C:/Users/USER/Desktop/ENTRETIENS%20PREMATURITE/CMA-DO/MCD-DO.mp3time=1420300) Interviewer: Généralement, quelles sont les femmes qui sont prédisposées à donner naissance aux prématurés? [00:23:46-3](http://localhost:2300/file=C:/Users/USER/Desktop/ENTRETIENS%20PREMATURITE/CMA-DO/MCD-DO.mp3time=1426300)

[00:23:46-3](http://localhost:2300/file=C:/Users/USER/Desktop/ENTRETIENS%20PREMATURITE/CMA-DO/MCD-DO.mp3time=1426300) Person 1: Est-ce qu'on va dire qu'il y a des femmes qui sont prédisposées à donner des enfants prématurés? Non, mais moi, je dirais que c'est beaucoup plus... Il y a des circonstances qui font des maladies qui font qu'au cours de la grossesse, qui peuvent vraiment, en tout cas, faciliter ou déclencher la prématurité. Il y a des maladies qui font que, par exemple un simple paludisme, ça provoque une grossesse, en tout cas, prématurée. C'est un exemple que je donne parmi tant d'autres maladies. Mais chez nous, il y a les paludisme, il y a d'autres pathologies qui donne. Donc, on ne va pas dire que c'est une prédisposition.Je ne pense pas que ça va être... Déjà, ça va être beaucoup plus de facteurs. [00:24:45-2](http://localhost:2300/file=C:/Users/USER/Desktop/ENTRETIENS%20PREMATURITE/CMA-DO/MCD-DO.mp3time=1485200)

[00:24:45-2](http://localhost:2300/file=C:/Users/USER/Desktop/ENTRETIENS%20PREMATURITE/CMA-DO/MCD-DO.mp3time=1485200) Interviewer: Peut-être une exposition. [00:24:47-5](http://localhost:2300/file=C:/Users/USER/Desktop/ENTRETIENS%20PREMATURITE/CMA-DO/MCD-DO.mp3time=1487500)

[00:24:47-5](http://localhost:2300/file=C:/Users/USER/Desktop/ENTRETIENS%20PREMATURITE/CMA-DO/MCD-DO.mp3time=1487500) Person 1: Voilà quand on est exposé au paludisme, on ne va pas aller loin. Si tu t'amuses, ça va te créer ça. Mais bon, à cela, les conditions socio-économiques très compliquées, avec le travail pénible, ça va être un facteur physique. Ça, c'est ça aussi, c'est connu. Je crois que les femmes qui fument, la drogue, ça, ce sont des facteurs qui peuvent vraiment générer certaines maladies chroniques qui sont sujets, diabète, mais c'est beaucoup plus des infections ça, c'est très, très rapidement ça déclenche. Bon, il y a certaines malformations même de l'utérus.L'utérus doit contenir, si l'utérus même est malformé à un moment, ça peut vraiment impacter. L'utérus même, sa partie qu'on appelle le col, c'est normalement lui, il doit serrer pour que l'enfant ne tombe pas. Ça reste dedans, ça doit tenir. Il y a certaines maladies même de l'utérus qu'on appelle les béances cervicaux du col utérin. Donc, ça fait que le col, il est ouvert. Quand il est ouvert à un certain moment avec la pression liée au poids du bébé, ça lâche. Bon, c'est un peu ça. Ça, c'est du côté de la maman. Bon, du côté de l'enfant même, c'est quand il y a beaucoup d'enfants dedans. On dit que la grossesse est multiple. Il y a beaucoup d'enfants dedans. Ça peut être... Il y a des enfants qui ont des anomalies chromosomiques. Ça peut faciliter. Donc, il y a plusieurs facteurs qui peuvent faire ça. Donc, c'est ce que je disais. Il y a plus de facteurs que de causes, qui peuvent améner vraiment à ça. [00:27:04-2](http://localhost:2300/file=C:/Users/USER/Desktop/ENTRETIENS%20PREMATURITE/CMA-DO/MCD-DO.mp3time=1624200)

00:23:15-2 Interviewer: Est-ce que vous avez remarqué des particularités spécifiques aux femmes qui donnent naissance aux enfants prématurés ? Est-ce qu'elles viennent d'un certain type de milieu ? Est-ce qu'elles sont d'une certaine tranche d'âge ? Est-ce qu'elles sont assujetties à un certain type de maladie ? Est-ce qu'il y a des particularités que vous avez remarquées au niveau de ces mères ? 00:23:31-1

00:23:31-1 Person 1: Je n'ai pas fait ce retour. Je n'ai pas fait ce retour parce que je ne l'ai pas vérifié. Je n'ai pas vu de particularité. 00:23:40-3 Interviewer: Généralement, quelles sont les femmes qui sont prédisposées à donner naissance aux prématurés ? 00:23:46-3

00:23:46-3 Person 1: Est-ce qu'on va dire qu'il y a des femmes qui sont prédisposées à donner des enfants prématurés ? Non, mais moi, je dirais que c'est beaucoup plus... Il y a des circonstances qui font des maladies qui font qu'au cours de la grossesse, qui peuvent vraiment, en tout cas, faciliter ou déclencher la prématurité. Il y a des maladies qui font que, par exemple un simple paludisme, ça provoque une grossesse, en tout cas, prématurée. C'est un exemple que je donne parmi tant d'autres maladies. Mais chez nous, il y a le paludisme, il y a d'autres pathologies qui donnent. Donc, on ne va pas dire que c'est une prédisposition. Je ne pense pas que ça va être... Déjà, ça va être beaucoup plus de facteurs. 00:24:45-2

00:24:45-2 Interviewer: Peut-être une exposition. 00:24:47-5

00:24:47-5 Person 1: Voilà, quand on est exposé au paludisme, on ne va pas aller loin. Si tu t'amuses, ça va te créer ça. Mais bon, à cela, les conditions socio-économiques très compliquées, avec le travail pénible, ça va être un facteur physique. Ça, c'est ça aussi, c'est connu. Je crois que les femmes qui fument, la drogue, ça, ce sont des facteurs qui peuvent vraiment générer certaines maladies chroniques qui sont sujets, diabète, mais c'est beaucoup plus des infections, ça, c'est très, très rapidement ça déclenche. Bon, il y a certaines malformations même de l'utérus. L'utérus doit contenir, si l'utérus même est malformé à un moment, ça peut vraiment impacter. L'utérus même, sa partie qu'on appelle le col, c'est normalement lui, il doit serrer pour que l'enfant ne tombe pas. Ça reste dedans, ça doit tenir. Il y a certaines maladies même de l'utérus qu'on appelle les béances cervicales du col utérin. Donc, ça fait que le col, il est ouvert. Quand il est ouvert à un certain moment avec la pression liée au poids du bébé, ça lâche. Bon, c'est un peu ça. Ça, c'est du côté de la maman. Bon, du côté de l'enfant même, c'est quand il y a beaucoup d'enfants dedans. On dit que la grossesse est multiple. Il y a beaucoup d'enfants dedans. Ça peut être... Il y a des enfants qui ont des anomalies chromosomiques. Ça peut faciliter. Donc, il y a plusieurs facteurs qui peuvent faire ça. Donc, c'est ce que je disais. Il y a plus de facteurs que de causes, qui peuvent amener vraiment à ça. 00:27:04-2

**00:23:15-2 Interviewer:** Have you noticed any specific characteristics among women who give birth to premature children? Do they come from a certain background, belong to a particular age group, or have specific types of illnesses?

**00:23:31-1 Person 1:** I haven't observed any specific trends or particularities in this regard. I haven't conducted a detailed analysis to identify such patterns.

**00:23:40-3 Interviewer:** Generally, are there certain women who are more predisposed to giving birth prematurely?

**00:23:46-3 Person 1:** It's not so much about predisposition as it is about certain conditions and illnesses during pregnancy that can lead to prematurity. For instance, a simple case of malaria can result in a premature birth. This is just one example among many other illnesses. In our country, aside from malaria, there are other pathologies that can cause prematurity. It's more about exposure to certain conditions rather than a predisposition.

**00:24:45-2 Interviewer:** Maybe an exposure?

**00:24:47-5 Person 1:** Exactly. Exposure to conditions like malaria can certainly contribute to prematurity. Additionally, socio-economic challenges and physically demanding work can be contributing factors. Lifestyle choices, such as smoking or drug use, can also play a role, potentially leading to chronic conditions like diabetes that can trigger prematurity. There are also certain uterine malformations, including issues with the cervix, which can impact pregnancy. In cases of multiple pregnancies, the presence of chromosomal abnormalities in the children can also be a factor. So, there are several factors, rather than specific causes, that can lead to premature birth

### Manager for maternity care unit of Do medical center with surgical branch

[00:19:24-9](http://localhost:2300/file=C:/Users/USER/Desktop/ENTRETIENS%20PREMATURITE/CMA-DO/RESPONSABLE%20UNITE%20DE%20SOIN%20CMA-DO.mp3time=1164900) Interviewer: Maintenant, est-ce que vous avez remarqué des particularités concernant les mères qui donnent naissance aux enfants prématurés? Est-ce qu'elles viennent d'un certain type de milieu socio-économique? Est-ce qu'elles sont assujetties à des maladies chroniques? Est-ce qu'elles sont plus jeunes, plus âgées? Est-ce qu'il y a des particularités pour celles qui donnent naissance a des enfants prématurés? [00:19:45-6](http://localhost:2300/file=C:/Users/USER/Desktop/ENTRETIENS%20PREMATURITE/CMA-DO/RESPONSABLE%20UNITE%20DE%20SOIN%20CMA-DO.mp3time=1185600)

[00:19:45-6](http://localhost:2300/file=C:/Users/USER/Desktop/ENTRETIENS%20PREMATURITE/CMA-DO/RESPONSABLE%20UNITE%20DE%20SOIN%20CMA-DO.mp3time=1185600) Person 1: Pas trop. C'est vrai qu'on n'a pas essayé de faire ce lien. On n'a pas essayé. Mais non, il n'y a pas de particularité.Il n'y a pas de niveau de vie. On n'a pas fait ce lien. Même également au niveau de l'âge, pour voir si c'est les jeunes, de 19 ans à 30 ans qui ont plus ça. On n'a pas essayé de faire le lien.Mais je pense que c'est de façon générale. [00:20:18-6](http://localhost:2300/file=C:/Users/USER/Desktop/ENTRETIENS%20PREMATURITE/CMA-DO/RESPONSABLE%20UNITE%20DE%20SOIN%20CMA-DO.mp3time=1218600)

00:19:24-9 Interviewer: Maintenant, est-ce que vous avez remarqué des particularités concernant les mères qui donnent naissance aux enfants prématurés ? Est-ce qu'elles viennent d'un certain type de milieu socio-économique ? Est-ce qu'elles sont assujetties à des maladies chroniques ? Est-ce qu'elles sont plus jeunes, plus âgées ? Est-ce qu'il y a des particularités pour celles qui donnent naissance à des enfants prématurés ? 00:19:45-6

00:19:45-6 Person 1: Pas trop. C'est vrai qu'on n'a pas essayé de faire ce lien. On n'a pas essayé. Mais non, il n'y a pas de particularité. Il n'y a pas de niveau de vie. On n'a pas fait ce lien. Même également au niveau de l'âge, pour voir si ce sont les jeunes, de 19 ans à 30 ans qui ont plus ça. On n'a pas essayé de faire le lien. Mais je pense que c'est de façon générale. 00:20:18-6

**00:19:24-9 Interviewer:** Have you noticed any specific characteristics among mothers who give birth to premature children? Do they come from a particular socio-economic background, or have chronic illnesses? Are they generally younger or older? Any particularities?

**00:19:45-6 Person 1:** Not really. We haven’t specifically looked for correlations between premature births and factors like socio-economic background or age. So, we don't have any particular observations in that regard. We haven’t made a connection between prematurity and specific age groups, such as those between 19 to 30 years old. Overall, it seems to be a general occurrence without distinct patterns based on our observations and approach.

### Focus group of Colma1 health and social promotion center

[00:19:53-9](http://localhost:2300/file=C:/Users/USER/Desktop/ENTRETIENS%20PREMATURITE/CSPS-COLMA1/FOCUS%20GROUPE%20COLMA1.mp3time=1193900) Interviewer: Est ce que vous avez remarqué des particularités spécifiques concernant les mères des enfants prématurés? Est ce qu'elles viennent d'un certain type de milieu? sont elles plus jeunes ou plus âgées? Ont elles des conditions de santé particulières? [00:20:18-5](http://localhost:2300/file=C:/Users/USER/Desktop/ENTRETIENS%20PREMATURITE/CSPS-COLMA1/FOCUS%20GROUPE%20COLMA1.mp3time=1218500)

[00:20:18-5](http://localhost:2300/file=C:/Users/USER/Desktop/ENTRETIENS%20PREMATURITE/CSPS-COLMA1/FOCUS%20GROUPE%20COLMA1.mp3time=1218500) Person 2: je vais prendre le cas d'une femme que nous avons reçu, qui a fait un accouchement prématuré, elle disait que cela faisait près de trois jours qu'elle venait consulter, nous avons constatés que le col était déjà ouvert et elle était déjà en travail donc nous ne pouvions plus rien faire, c'est pour dire que se sont les cas de consultations tardives qui peuvent amener generalement ses cas de naissances prématurées. Si on l'avait pris en charge plutôt, je ne pense pas qu'elle serait rentrée en travail. Souvent se sont les consultations tardives donc les cols que nous ne suivons pas regulièrement. [00:21:06-7](http://localhost:2300/file=C:/Users/USER/Desktop/ENTRETIENS%20PREMATURITE/CSPS-COLMA1/FOCUS%20GROUPE%20COLMA1.mp3time=1266700)

[00:21:06-7](http://localhost:2300/file=C:/Users/USER/Desktop/ENTRETIENS%20PREMATURITE/CSPS-COLMA1/FOCUS%20GROUPE%20COLMA1.mp3time=1266700) Interviewer: Par consultation est que vous parlez de CPN? [00:21:10-5](http://localhost:2300/file=C:/Users/USER/Desktop/ENTRETIENS%20PREMATURITE/CSPS-COLMA1/FOCUS%20GROUPE%20COLMA1.mp3time=1270500)

[00:21:10-5](http://localhost:2300/file=C:/Users/USER/Desktop/ENTRETIENS%20PREMATURITE/CSPS-COLMA1/FOCUS%20GROUPE%20COLMA1.mp3time=1270500) Person 2: non non géneralement quand elles sont malades, elles viennent tardivement en consultation et c'est parce qu'elle est venue tardivement en consultation qu'elle est rentrée en travail cela pouvait s'eviter [00:21:24-2](http://localhost:2300/file=C:/Users/USER/Desktop/ENTRETIENS%20PREMATURITE/CSPS-COLMA1/FOCUS%20GROUPE%20COLMA1.mp3time=1284200)

[00:21:24-2](http://localhost:2300/file=C:/Users/USER/Desktop/ENTRETIENS%20PREMATURITE/CSPS-COLMA1/FOCUS%20GROUPE%20COLMA1.mp3time=1284200) Interviewer: Est ce qu'il y a quelque chose d'autre à ajouter? [00:21:28-8](http://localhost:2300/file=C:/Users/USER/Desktop/ENTRETIENS%20PREMATURITE/CSPS-COLMA1/FOCUS%20GROUPE%20COLMA1.mp3time=1288800)

[00:21:28-8](http://localhost:2300/file=C:/Users/USER/Desktop/ENTRETIENS%20PREMATURITE/CSPS-COLMA1/FOCUS%20GROUPE%20COLMA1.mp3time=1288800) Person 2: Souvent aussi, il y a des femmes qui ont des difficultés au niveau social. Soit se sont des élèves ou bien la rélation de couple ne va pas bien. Géneralement se sont des femmes exposées à ses cas qui donnent naissance à des enfants prématurés. il y a d'autres aussi, au niveau social ça ne va pas, et d'autres c'est parce qu'elles prennent du temps pour venir faire la consultation. je ne sais pas s'il y a quelque chose d'autre? [00:22:05-0](http://localhost:2300/file=C:/Users/USER/Desktop/ENTRETIENS%20PREMATURITE/CSPS-COLMA1/FOCUS%20GROUPE%20COLMA1.mp3time=1325000)

[00:22:05-0](http://localhost:2300/file=C:/Users/USER/Desktop/ENTRETIENS%20PREMATURITE/CSPS-COLMA1/FOCUS%20GROUPE%20COLMA1.mp3time=1325000) Person 5: c'est pour dire qu'effectivement que se sont des femmes qui ont des problèmes, qui ont d'autres pathologies comme le paludisme, l'hypertension. [00:22:19-9](http://localhost:2300/file=C:/Users/USER/Desktop/ENTRETIENS%20PREMATURITE/CSPS-COLMA1/FOCUS%20GROUPE%20COLMA1.mp3time=1339900)

00:19:53-9 Interviewer: Est-ce que vous avez remarqué des particularités spécifiques concernant les mères des enfants prématurés ? Est-ce qu'elles viennent d'un certain type de milieu ? Sont-elles plus jeunes ou plus âgées ? Ont-elles des conditions de santé particulières ? 00:20:18-5

00:20:18-5 Person 2: Je vais prendre le cas d'une femme que nous avons reçu, qui a fait un accouchement prématuré, elle disait que cela faisait près de trois jours qu'elle venait consulter, nous avons constaté que le col était déjà ouvert et elle était déjà en travail donc nous ne pouvions plus rien faire, c'est pour dire que ce sont les cas de consultations tardives qui peuvent amener généralement ces cas de naissances prématurées. Si on l'avait prise en charge plutôt, je ne pense pas qu'elle serait entrée en travail. Souvent ce sont les consultations tardives donc les cols que nous ne suivons pas régulièrement. 00:21:06-7

00:21:06-7 Interviewer: Par consultation est-ce que vous parlez de CPN ? 00:21:10-5

00:21:10-5 Person 2: Non, non, généralement quand elles sont malades, elles viennent tardivement en consultation et c'est parce qu'elle est venue tardivement en consultation qu'elle est entrée en travail, cela pouvait s'éviter. 00:21:24-2

00:21:24-2 Interviewer: Est-ce qu'il y a quelque chose d'autre à ajouter ? 00:21:28-8

00:21:28-8 Person 2: Souvent aussi, il y a des femmes qui ont des difficultés au niveau social. Soit ce sont des élèves ou bien la relation de couple ne va pas bien. Généralement ce sont des femmes exposées à ces cas qui donnent naissance à des enfants prématurés. Il y a d'autres aussi, au niveau social ça ne va pas, et d'autres c'est parce qu'elles prennent du temps pour venir faire la consultation. Je ne sais pas s'il y a quelque chose d'autre ? 00:22:05-0

00:22:05-0 Person 5: C'est pour dire qu'effectivement ce sont des femmes qui ont des problèmes, qui ont d'autres pathologies comme le paludisme, l'hypertension. 00:22:19-9

**00:19:53-9 Interviewer:** Have you noticed any specific characteristics among mothers of premature children? Do they come from a particular background? Are they younger or older? Do they have any special health conditions?

**00:20:18-5 Person 2:** One example we had was a woman who experienced premature labor. She came for consultation when her cervix was already open, and she was already in labor, so it was too late to intervene. This suggests that late consultations are a common factor leading to premature births. If she had received earlier care, her premature labor might have been avoided. It’s often the case of delayed medical consultations and missed prenatal check-ups that contribute to these situations.

**00:21:06-7 Interviewer:** Are you referring to prenatal consultations?

**00:21:10-5 Person 2:** No, I mean general medical consultations. When they are sick, they often come late for consultation. In this case, the delay led to premature labor, which could have been avoided.

**00:21:24-2 Interviewer:** Is there anything else you’d like to add?

**00:21:28-8 Person 2:** Often, these women face social challenges. They might be students or experiencing relationship problems in their couple. It's usually women in these situations who give birth prematurely. Others may be struggling socially, and some delay seeking medical consultations.

**00:22:05-0 Person 5:** To add, these women often have other health issues as well, such as malaria or hypertension, contributing to the risk of premature birth.

### Major of Colma1 health and social promotion center

[00:16:03-9](http://localhost:2300/file=C:/Users/USER/Desktop/ENTRETIENS%20PREMATURITE/CSPS-COLMA1/MAJOR%20CSPS%20COLMA1.MP3time=963900) Interviewer: Est-ce que vous avez remarqué des particularités spécifiques concernant les mères des enfants prématurés ? Est-ce qu'elles viennent d'un certain type de milieu ? Est ce qu'elles sont plus ou moins jeunes ? Est-ce qu'elles sont soumises à certaines maladies ? Est-ce que de façon spécifique vous avez remarqué des particularités pour les mères? [00:16:22-3](http://localhost:2300/file=C:/Users/USER/Desktop/ENTRETIENS%20PREMATURITE/CSPS-COLMA1/MAJOR%20CSPS%20COLMA1.MP3time=982300)

[00:16:22-3](http://localhost:2300/file=C:/Users/USER/Desktop/ENTRETIENS%20PREMATURITE/CSPS-COLMA1/MAJOR%20CSPS%20COLMA1.MP3time=982300) Person 1: Bon, bon, bon, bon, sincèrement je n'ai jamais analysé ça, mais ce que je sais, je sais que certaines prématuritées sont dûes, beaucoup plus au paludisme chez la mère, avec la fièvre rapidement donc elle expluse pendant que la grossesse n'est pas arrivée à terme. c'est ce que je sais . Donc les infections et le paludisme sont les 2 principales causes que moi je retiens principalement de la prématurité. [00:17:01-0](http://localhost:2300/file=C:/Users/USER/Desktop/ENTRETIENS%20PREMATURITE/CSPS-COLMA1/MAJOR%20CSPS%20COLMA1.MP3time=1021000)

[00:17:01-0](http://localhost:2300/file=C:/Users/USER/Desktop/ENTRETIENS%20PREMATURITE/CSPS-COLMA1/MAJOR%20CSPS%20COLMA1.MP3time=1021000) Interviewer: C'est surtout les l'état de santé de la mère? [00:17:02-7](http://localhost:2300/file=C:/Users/USER/Desktop/ENTRETIENS%20PREMATURITE/CSPS-COLMA1/MAJOR%20CSPS%20COLMA1.MP3time=1022700)

[00:17:02-7](http://localhost:2300/file=C:/Users/USER/Desktop/ENTRETIENS%20PREMATURITE/CSPS-COLMA1/MAJOR%20CSPS%20COLMA1.MP3time=1022700) Person 1: oui c'est lié à l'état de santé de la mère, voilà, moi, c'est ce que je retiens. [00:17:07-9](http://localhost:2300/file=C:/Users/USER/Desktop/ENTRETIENS%20PREMATURITE/CSPS-COLMA1/MAJOR%20CSPS%20COLMA1.MP3time=1027900)

00:16:03-9 Interviewer: Est-ce que vous avez remarqué des particularités spécifiques concernant les mères des enfants prématurés ? Est-ce qu'elles viennent d'un certain type de milieu ? Est-ce qu'elles sont plus ou moins jeunes ? Est-ce qu'elles sont soumises à certaines maladies ? Est-ce que de façon spécifique vous avez remarqué des particularités pour les mères ? 00:16:22-3

00:16:22-3 Person 1: Bon, bon, bon, bon, sincèrement je n'ai jamais analysé ça, mais ce que je sais, je sais que certaines prématurités sont dues, beaucoup plus au paludisme chez la mère, avec la fièvre rapidement donc elle expulse pendant que la grossesse n'est pas arrivée à terme. C'est ce que je sais. Donc les infections et le paludisme sont les 2 principales causes que moi je retiens principalement de la prématurité. 00:17:01-0

00:17:01-0 Interviewer: C'est surtout l'état de santé de la mère ? 00:17:02-7

00:17:02-7 Person 1: Oui, c'est lié à l'état de santé de la mère, voilà, moi, c'est ce que je retiens. 00:17:07-9

**00:16:03-9 Interviewer:** Have you observed any specific characteristics among mothers of premature children? Do they come from a certain background, are they typically younger, or do they have certain health conditions? Have you noticed any particular patterns?

**00:16:22-3 Person 1:** Honestly, I've never conducted a detailed analysis on that. However, from what I know, many cases of prematurity are due to malaria in the mother. The fever associated with malaria can trigger premature labor before the pregnancy has reached full term. In my experience, infections and malaria are the two main health issues that I associate with prematurity.

**00:17:01-0 Interviewer:** So, it's primarily related to the mother's health condition?

**00:17:02-7 Person 1:** Yes, it's linked to the mother's health. That's what I can recall from my experiences.

### Maternity manager of Colma1 health and social promotion center

**No response elements**

### Focus group of Farakan health and social promotion center

[00:34:41-3](http://localhost:2300/file=C:/Users/USER/Desktop/ENTRETIENS%20PREMATURITE/CSPS_FARAKAN/FOCUS_GROUPE-CSPS_FARAKAN.MP3time=2081300) Interviewer: Maintenant, nous allons poursuivre, est-ce que vous avez remarqué des particularités spécifiques concernant les mères des enfants prématurés ? Sont elles plus jeunes? Est-ce qu'elles viennent d'un milieu socio-économique spécifique? Où sont-elles dans des conditions de santé assez particulière ? Qu'est-ce que vous avez remarqué au niveau des mères des enfants prématurés ? [00:35:11-9](http://localhost:2300/file=C:/Users/USER/Desktop/ENTRETIENS%20PREMATURITE/CSPS_FARAKAN/FOCUS_GROUPE-CSPS_FARAKAN.MP3time=2111900)

[00:35:11-9](http://localhost:2300/file=C:/Users/USER/Desktop/ENTRETIENS%20PREMATURITE/CSPS_FARAKAN/FOCUS_GROUPE-CSPS_FARAKAN.MP3time=2111900) Person 3: Ce que j'ai particulièrement remarqué, ce sont des femmes le plus souvent un peu démunies.La majorité des femmes sont démunies et la plupart de ses femmes présentent même dès la grossesse déjà des complications.Le plus souvent, la préclampsie, c'est l'hypertension il y a le paludisme aussi. [00:35:46-2](http://localhost:2300/file=C:/Users/USER/Desktop/ENTRETIENS%20PREMATURITE/CSPS_FARAKAN/FOCUS_GROUPE-CSPS_FARAKAN.MP3time=2146200)

[00:35:46-2](http://localhost:2300/file=C:/Users/USER/Desktop/ENTRETIENS%20PREMATURITE/CSPS_FARAKAN/FOCUS_GROUPE-CSPS_FARAKAN.MP3time=2146200) Interviewer: Est ce qu'il ya quelque chose à ajouter? [00:35:54-4](http://localhost:2300/file=C:/Users/USER/Desktop/ENTRETIENS%20PREMATURITE/CSPS_FARAKAN/FOCUS_GROUPE-CSPS_FARAKAN.MP3time=2154400)

[00:35:54-4](http://localhost:2300/file=C:/Users/USER/Desktop/ENTRETIENS%20PREMATURITE/CSPS_FARAKAN/FOCUS_GROUPE-CSPS_FARAKAN.MP3time=2154400) Person 7: Pour moi c'est la, c'est la négligence, hein, la négligence des femmes. Souvent elles viennent tu leurs donnes des conseils, elles negligent ça, donc pour moi c'est la négligence. [00:36:07-5](http://localhost:2300/file=C:/Users/USER/Desktop/ENTRETIENS%20PREMATURITE/CSPS_FARAKAN/FOCUS_GROUPE-CSPS_FARAKAN.MP3time=2167500)

[00:36:07-5](http://localhost:2300/file=C:/Users/USER/Desktop/ENTRETIENS%20PREMATURITE/CSPS_FARAKAN/FOCUS_GROUPE-CSPS_FARAKAN.MP3time=2167500) Interviewer: Et c'est c'est quel genre de conseil que vous donnez? [00:36:08-8](http://localhost:2300/file=C:/Users/USER/Desktop/ENTRETIENS%20PREMATURITE/CSPS_FARAKAN/FOCUS_GROUPE-CSPS_FARAKAN.MP3time=2168800)

[00:36:08-8](http://localhost:2300/file=C:/Users/USER/Desktop/ENTRETIENS%20PREMATURITE/CSPS_FARAKAN/FOCUS_GROUPE-CSPS_FARAKAN.MP3time=2168800) Person 7: Conseil pour éviter les accouchements prématurés, souvent les femmes ne prennent pas leurs traitements, elles ne prennent pas bien leurs traitements préventifs contre le PALU.Souvent, il y a la malnutrition, elles disent qu'elles n'arrivent pas à manger, elles ne s'efforcent pas aussi. Elles n'écoutent pas nos conseils en tout cas. [00:36:38-6](http://localhost:2300/file=C:/Users/USER/Desktop/ENTRETIENS%20PREMATURITE/CSPS_FARAKAN/FOCUS_GROUPE-CSPS_FARAKAN.MP3time=2198600)

[00:36:38-6](http://localhost:2300/file=C:/Users/USER/Desktop/ENTRETIENS%20PREMATURITE/CSPS_FARAKAN/FOCUS_GROUPE-CSPS_FARAKAN.MP3time=2198600) Person 8: La plupart, c'est la pauvreté la qui amène ça , toi tu as 5 enfants dedans et tu te demandes même si tu pourras avoir quelque chose à leurs donner, donc tout cela joue sur la grossesse que tu portes. C'est vraiment la pauvrété, le niveau social, vraiment, ça ne va pas. [00:37:04-6](http://localhost:2300/file=C:/Users/USER/Desktop/ENTRETIENS%20PREMATURITE/CSPS_FARAKAN/FOCUS_GROUPE-CSPS_FARAKAN.MP3time=2224600)

[00:37:04-6](http://localhost:2300/file=C:/Users/USER/Desktop/ENTRETIENS%20PREMATURITE/CSPS_FARAKAN/FOCUS_GROUPE-CSPS_FARAKAN.MP3time=2224600) Person 6: La plupart aussi il y a la malnutrition.En milieu rural (les travaux champêtres) et même en ville il y a aussi les travaux penibles, tous ses facteurs peuvent entrainer la prématurité. [00:37:19-6](http://localhost:2300/file=C:/Users/USER/Desktop/ENTRETIENS%20PREMATURITE/CSPS_FARAKAN/FOCUS_GROUPE-CSPS_FARAKAN.MP3time=2239600)

[00:37:19-6](http://localhost:2300/file=C:/Users/USER/Desktop/ENTRETIENS%20PREMATURITE/CSPS_FARAKAN/FOCUS_GROUPE-CSPS_FARAKAN.MP3time=2239600) Person 3: Il y a un phénomène aussi qui fait aggraver la prématurité ces derniers temps, c'est la gratuité. Les gens ont une mauvaise compréhension de la gratuité. Ça fait que les femmes viennent avec des complications lors des grossesses. Des traitements sont prescrits, mais il y a la mauvaise observance en cas de rupture, par exemple de la sulfadoxine, (80%) Quatre-vingts pour 100 des femmes ne payent plus la sulfadoxine et nous sommes en rupture depuis un an, personne ne paie. A ce titre le paludisme n'est pas prévenu. Chacun vient avec une ordonnance parallèle, on dit non, c'est gratuit. Le mari refuse de payer.Les pathologies ne sont pas correctement traités. Sinon les complications sont détectées, mais l'observance n'est pas bon. [00:38:08-2](http://localhost:2300/file=C:/Users/USER/Desktop/ENTRETIENS%20PREMATURITE/CSPS_FARAKAN/FOCUS_GROUPE-CSPS_FARAKAN.MP3time=2288200)

[00:38:08-2](http://localhost:2300/file=C:/Users/USER/Desktop/ENTRETIENS%20PREMATURITE/CSPS_FARAKAN/FOCUS_GROUPE-CSPS_FARAKAN.MP3time=2288200) Person 2: Moi je rejoins ma predecesseure qui dit qu'il y a la négligence c'est vrai il y a la négligence, il y a la pauvreté. Quand on dit négligeance, voilà tout de suite, on est face à une qui est malade, depuis elle fait son paludisme, on dit qu'on lui a donné le moustiquaire, elle a pris dans son sachet, elle l'a deposé sans l'utiliser et elle est malade depuis 2 jours ou 3 jours elle arrive même pas à se tenir. C'est pour cela que nous sommes entrain de prendre la voie. Il y a la pauvreté, il y a la négligence, tous les facteurs sont réunis chez nous ici pour qu'on fasse des accouchements prématurés. [00:38:40-1](http://localhost:2300/file=C:/Users/USER/Desktop/ENTRETIENS%20PREMATURITE/CSPS_FARAKAN/FOCUS_GROUPE-CSPS_FARAKAN.MP3time=2320100)

[00:38:40-1](http://localhost:2300/file=C:/Users/USER/Desktop/ENTRETIENS%20PREMATURITE/CSPS_FARAKAN/FOCUS_GROUPE-CSPS_FARAKAN.MP3time=2320100) Person 6: La plupart les femmes négligent la prise des produits comme le fer. Beaucoup negligent la prise du fer, et pourtant il y a une importance dedans. Voilà pourquoi aussi l'anemie se presente chez d'autres. [00:39:03-4](http://localhost:2300/file=C:/Users/USER/Desktop/ENTRETIENS%20PREMATURITE/CSPS_FARAKAN/FOCUS_GROUPE-CSPS_FARAKAN.MP3time=2343400)

00:34:41-3 Interviewer: Maintenant, nous allons poursuivre, est-ce que vous avez remarqué des particularités spécifiques concernant les mères des enfants prématurés ? Sont-elles plus jeunes ? Est-ce qu'elles viennent d'un milieu socio-économique spécifique ? Ou sont-elles dans des conditions de santé assez particulières ? Qu'est-ce que vous avez remarqué au niveau des mères des enfants prématurés ? 00:35:11-9

00:35:11-9 Person 3: Ce que j'ai particulièrement remarqué, ce sont des femmes le plus souvent un peu démunies. La majorité des femmes sont démunies et la plupart de ces femmes présentent même dès la grossesse déjà des complications. Le plus souvent, la prééclampsie, c'est l'hypertension, il y a le paludisme aussi. 00:35:46-2

00:35:46-2 Interviewer: Est-ce qu'il y a quelque chose à ajouter ? 00:35:54-4

00:35:54-4 Person 7: Pour moi c'est la négligence, hein, la négligence des femmes. Souvent elles viennent, tu leur prodigues des conseils, elles négligent ça, donc pour moi c'est la négligence. 00:36:07-5

00:36:07-5 Interviewer: Et c'est quel genre de conseil que vous donnez ? 00:36:08-8

00:36:08-8 Person 7: Conseil pour éviter les accouchements prématurés, souvent les femmes ne prennent pas leurs traitements, elles ne prennent pas bien leurs traitements préventifs contre le PALU. Souvent, il y a la malnutrition, elles disent qu'elles n'arrivent pas à manger, elles ne s'efforcent pas aussi. Elles n'écoutent pas nos conseils en tout cas. 00:36:38-6

00:36:38-6 Person 8: La plupart, c'est la pauvreté qui amène ça, toi tu as 5 enfants dedans et tu te demandes même si tu pourras avoir quelque chose à leur donner, donc tout cela joue sur la grossesse que tu portes. C'est vraiment la pauvreté, le niveau social, vraiment, ça ne va pas. 00:37:04-6

00:37:04-6 Person 6: La plupart aussi il y a la malnutrition. En milieu rural (les travaux champêtres) et même en ville il y a aussi les travaux pénibles, tous ces facteurs peuvent entraîner la prématurité. 00:37:19-6

00:37:19-6 Person 3: Il y a un phénomène aussi qui fait aggraver la prématurité ces derniers temps, c'est la gratuité. Les gens ont une mauvaise compréhension de la gratuité. Ça fait que les femmes viennent avec des complications lors des grossesses. Des traitements sont prescrits, mais il y a la mauvaise observance en cas de rupture, par exemple de la sulfadoxine, (80%) Quatre-vingts pour cent des femmes ne payent plus la sulfadoxine et nous sommes en rupture depuis un an, personne ne paie. À ce titre le paludisme n'est pas prévenu. Chacun vient avec une ordonnance parallèle, on dit non, c'est gratuit. Le mari refuse de payer. Les pathologies ne sont pas correctement traitées. Sinon les complications sont détectées, mais l'observance n'est pas bonne. 00:38:08-2

00:38:08-2 Person 2: Moi je rejoins ma prédécesseur qui dit qu'il y a la négligence c'est vrai il y a la négligence, il y a la pauvreté. Quand on dit négligence, voilà tout de suite, on est face à une qui est malade, depuis elle fait son paludisme, on dit qu'on lui a donné la moustiquaire, elle a pris dans son sachet, elle l'a déposé sans l'utiliser et elle est malade depuis 2 jours ou 3 jours elle arrive même pas à se tenir. C'est pour cela que nous sommes en train de prendre la voie. Il y a la pauvreté, il y a la négligence, tous les facteurs sont réunis chez nous ici pour qu'on fasse des accouchements prématurés. 00:38:40-1

00:38:40-1 Person 6: La plupart des femmes négligent la prise des produits comme le fer. Beaucoup négligent la prise du fer, et pourtant il y a une importance dedans. Voilà pourquoi aussi l'anémie se présente chez d'autres. 00:39:03-4

**00:34:41-3 Interviewer:** Now, let's continue. Have you noticed any specific characteristics among mothers of premature children? Are they younger, from a particular socio-economic background, or in specific health conditions?

**00:35:11-9 Person 3:** What I've particularly noticed is that these women are often from deprived backgrounds. The majority are underprivileged, and many of them experience complications during pregnancy, such as preeclampsia and malaria.

**00:35:46-2 Interviewer:** Is there anything else you'd like to add?

**00:35:54-4 Person 7:** I think a key issue is neglect. Often, these women neglect the advice given to them. For example, they don't adhere to their treatments or preventive measures against malaria, and malnutrition is common. They don’t follow our dietary advice.

**00:36:38-6 Person 8:** Poverty is a major contributing factor. When a woman already has several children and struggles to provide for them, this stress affects her pregnancy. The socio-economic challenges are significant.

**00:37:04-6 Person 6:** Malnutrition is a common issue, both in rural and urban settings, where heavy work can also lead to prematurity.

**00:37:19-6 Person 3:** Recently, the misconception about free medical services has worsened the situation. When treatments are prescribed, compliance is poor, particularly for malaria prevention. Many women don’t pay for essential medications like sulfadoxine, leading to untreated conditions.

**00:38:08-2 Person 2:** I agree about the issue of neglect and poverty. For instance, women might receive mosquito nets but don't use them, and then suffer from malaria. These factors, along with poor health practices, contribute to premature births.

**00:39:03-4 Person 6:** Many women also neglect taking supplements like iron, which is crucial. This neglect leads to conditions like anemia, further complicating their pregnancies.

### Major of Farakan health and social promotion center

[00:12:53-4](http://localhost:2300/file=C:/Users/USER/Desktop/ENTRETIENS%20PREMATURITE/CSPS_FARAKAN/MAJOR%20FARAKAN.MP3time=773400) Interviewer: Est ce que vous avez remarqué des particularités specifiques au niveau des mères des enfants prématurés? Est ce qu'elles viennent d'un certain type de milieu,Est ce qu'elles sont d'une certaine tranche d'âge, ont elles des conditions de santé particulières? [00:13:11-7](http://localhost:2300/file=C:/Users/USER/Desktop/ENTRETIENS%20PREMATURITE/CSPS_FARAKAN/MAJOR%20FARAKAN.MP3time=791700)

[00:13:11-7](http://localhost:2300/file=C:/Users/USER/Desktop/ENTRETIENS%20PREMATURITE/CSPS_FARAKAN/MAJOR%20FARAKAN.MP3time=791700) Person 1: Géneralement, ce sont les jeunes filles.Les prémières grossesses se terminent souvent par une naissance prématurées. ça concerne géneralement les jeunes femmes. [00:13:32-0](http://localhost:2300/file=C:/Users/USER/Desktop/ENTRETIENS%20PREMATURITE/CSPS_FARAKAN/MAJOR%20FARAKAN.MP3time=812000)

00:12:53-4 Interviewer: Est-ce que vous avez remarqué des particularités spécifiques au niveau des mères des enfants prématurés ? Est-ce qu'elles viennent d'un certain type de milieu, est-ce qu'elles sont d'une certaine tranche d'âge, ont-elles des conditions de santé particulières ? 00:13:11-7

00:13:11-7 Person 1: Généralement, ce sont les jeunes filles. Les premières grossesses se terminent souvent par une naissance prématurée. Ça concerne généralement les jeunes femmes. 00:13:32-0

**00:12:53-4 Interviewer:** Have you observed any particular characteristics among mothers of premature children? Do they belong to a specific background, age group, or have certain health conditions?

**00:13:11-7 Person 1:** Typically, we see premature births more frequently in young girls. It's often the case that first pregnancies result in premature birth, so it predominantly concerns younger women.

### Maternity manager of Farakan health and social promotion center

[00:21:34-3](http://localhost:2300/file=C:/Users/USER/Desktop/ENTRETIENS%20PREMATURITE/CSPS_FARAKAN/RESPONSABLE%20MATERNITE%20FARAKAN.MP3time=1294300) Interviewer: Nous allons poursuivre. Est ce que vous avez remarqué des particularités spécifiques concernant les mères des enfants prématurés? [00:21:45-0](http://localhost:2300/file=C:/Users/USER/Desktop/ENTRETIENS%20PREMATURITE/CSPS_FARAKAN/RESPONSABLE%20MATERNITE%20FARAKAN.MP3time=1305000)

[00:21:45-0](http://localhost:2300/file=C:/Users/USER/Desktop/ENTRETIENS%20PREMATURITE/CSPS_FARAKAN/RESPONSABLE%20MATERNITE%20FARAKAN.MP3time=1305000) Person 1: La mère des enfants prématurés, même la malnutrition peut entrainer la prématurité. Il y a souvent l'anemie, la tension c'est à dire l'hypertension, la prééclampsie peuvent être des facteurs qui favorisesnt les naissances prématurées. [00:22:10-3](http://localhost:2300/file=C:/Users/USER/Desktop/ENTRETIENS%20PREMATURITE/CSPS_FARAKAN/RESPONSABLE%20MATERNITE%20FARAKAN.MP3time=1330300)

[00:22:10-3](http://localhost:2300/file=C:/Users/USER/Desktop/ENTRETIENS%20PREMATURITE/CSPS_FARAKAN/RESPONSABLE%20MATERNITE%20FARAKAN.MP3time=1330300) Interviewer: Et par rapport à leur âge, tendent elles à être jeune ou plus âgées? [00:22:12-1](http://localhost:2300/file=C:/Users/USER/Desktop/ENTRETIENS%20PREMATURITE/CSPS_FARAKAN/RESPONSABLE%20MATERNITE%20FARAKAN.MP3time=1332100)

[00:22:12-1](http://localhost:2300/file=C:/Users/USER/Desktop/ENTRETIENS%20PREMATURITE/CSPS_FARAKAN/RESPONSABLE%20MATERNITE%20FARAKAN.MP3time=1332100) Person 1: Je pense que c'est à tous les niveaux [00:22:29-6](http://localhost:2300/file=C:/Users/USER/Desktop/ENTRETIENS%20PREMATURITE/CSPS_FARAKAN/RESPONSABLE%20MATERNITE%20FARAKAN.MP3time=1349600)

[00:22:29-6](http://localhost:2300/file=C:/Users/USER/Desktop/ENTRETIENS%20PREMATURITE/CSPS_FARAKAN/RESPONSABLE%20MATERNITE%20FARAKAN.MP3time=1349600) Interviewer: Est ce qu'elles viennent souvent d'un certain type de milieu socio-économique? [00:22:36-5](http://localhost:2300/file=C:/Users/USER/Desktop/ENTRETIENS%20PREMATURITE/CSPS_FARAKAN/RESPONSABLE%20MATERNITE%20FARAKAN.MP3time=1356500)

[00:22:36-5](http://localhost:2300/file=C:/Users/USER/Desktop/ENTRETIENS%20PREMATURITE/CSPS_FARAKAN/RESPONSABLE%20MATERNITE%20FARAKAN.MP3time=1356500) Person 1: En tout cas, c'est ce que j'ai dt avec la malnutrition, si la maman n'arrive même pas à bien se nourrir, ça peut arriver. il ya souvent des jeunes filles avec des grossesses non desirées, nous avons vu des cas comme ça aussi. [00:23:16-2](http://localhost:2300/file=C:/Users/USER/Desktop/ENTRETIENS%20PREMATURITE/CSPS_FARAKAN/RESPONSABLE%20MATERNITE%20FARAKAN.MP3time=1396200)

00:21:34-3 Interviewer: Nous allons poursuivre. Est-ce que vous avez remarqué des particularités spécifiques concernant les mères des enfants prématurés ? 00:21:45-0

00:21:45-0 Person 1: La mère des enfants prématurés, même la malnutrition peut entraîner la prématurité. Il y a souvent l'anémie, la tension, c'est-à-dire l'hypertension, la prééclampsie peuvent être des facteurs qui favorisent les naissances prématurées. 00:22:10-3

00:22:10-3 Interviewer: Et par rapport à leur âge, tendent-elles à être jeunes ou plus âgées ? 00:22:12-1

00:22:12-1 Person 1: Je pense que c'est à tous les niveaux. 00:22:29-6

00:22:29-6 Interviewer: Est-ce qu'elles viennent souvent d'un certain type de milieu socio-économique ? 00:22:36-5

00:22:36-5 Person 1: En tout cas, c'est ce que j'ai dit avec la malnutrition, si la maman n'arrive même pas à bien se nourrir, ça peut arriver. Il y a souvent des jeunes filles avec des grossesses non désirées, nous avons vu des cas comme ça aussi. 00:23:16-2

**00:21:34-3 Interviewer:** Let's continue. Have you noticed any specific characteristics among mothers of premature children?

**00:21:45-0 Person 1:** Yes, several factors can lead to prematurity. Malnutrition is a significant one. Additionally, anemia, high blood pressure, and preeclampsia are common factors that favor premature births.

**00:22:10-3 Interviewer:** Regarding their age, do these mothers tend to be younger or older?

**00:22:12-1 Person 1:** It varies; we see prematurity across all age groups.

**00:22:29-6 Interviewer:** Do they often come from a certain socio-economic background?

**00:22:36-5 Person 1:** Malnutrition, which is linked to socio-economic status, plays a role. If a mother can't eat well, it can lead to prematurity. We also frequently see young girls with unwanted pregnancies experiencing premature births.

# 11. Improvement in care of preterm birth

### Focus group of Accart-ville urban medical center

[00:31:34-9](http://localhost:23) Interviewer: d'accord nous allons poursuivre.comment peut on ameliorer la prise en charge des naissances prématurés dans votre centre de santé? qu'est ce qu'il faut faire selon vous pour ameliore la prise en charge des naissances prematurés au sein de votre centre de santé? [00:31:56-8](http://localhost:2300/file=C:/Users/USER/Desktop/ENTRETIENS)

[00:31:56-8](http://localhost:2300/file=C:/Users/USER/Desktop/ENTRETIENS%20PREMATURITE/CSPS-ACCART-VILLE/FOCUS%20GROUPE%20ACCART-VILLE-BON.mp3time=1916800) Person 3: bon, je pense à travers la sensibilisation surtout dans la communauté, que les femmes viennent très tôt commencer la CPN, la prise du fer et l'alimentation sont très importantes. que le suivi soit vraiment correct.avec la sensibilisation on peut eviter la prematurité [00:32:28-4](%22ht)

[00:32:28-4](http://localhost:2300/file=C:/Users/USER/Desktop/ENTRETIENS) Interviewer: est ce qu'il y a autres choses à ajouter? [00:32:31-6](http://localhost:2300/file=C:/Users/USER/Desktop/ENTRETIENS)

[00:32:31-6](http://localhost:2300/file=C:/Users/USER/Desktop/ENTRETIENS%20PREMATURITE/CSPS-ACCART-VILLE/FOCUS%20GROUPE%20ACCART-VILLE-BON.mp3time=1951600) Person 3: ce qu'on peut dire, on va situer la question à deux étapes. la première etape c'est de lutter contre la prematurité d'abord c'est à dire prendre en charge correctement la menace des accochements prematurés en faisant un suivi correct des grossesses pour eviter la prematurité et une fois que la prematurité est déjà installée, il faut prendre en charge à savoir il faut lutter contre les infections,lutter contre l'hypothermie. maintenant comme precedemment dit plus haut, à notre niveau nous n'avons pas de materiels necessaires pour prende correctement en charge les nouveaux nées prématurés. au niveau du personnel nous pouvons dire que ça va puisque nous avons deux medecins içi mais c'est au niveau du materiel medico-technique que ça pose problème en commençant par les locaux. nous n'avons pas une salle dediée uniquement aux prématurés, nous n'avons pas egalement de couveuses, d'appareil d'echographie pour pouvoir dater les grossesses correctement et situer même le type de prematurité. donc de façon general le materiel nous manque pour prendre en charge de façon efficace les nouveaux nées prématurés. [00:33:56-2](http://localhost:2300/file=C:/Users/USER/Desktop/ENTRETIENS%20PREMATURITE/CSPS-ACCART-VILLE/FOCUS%20GROUPE%20ACCART-VILLE-BON.mp3time=2036200)

[00:33:56-2](http://localhost:2300/file=C:/Users/USER/Desktop/ENTRETIE) Interviewer: est ce qu'il y a quelque chose d'autre à ajouter? est ce que par rapport à toutes ses propositions le centre de santé dispose de personnel qualifié pour prendre en charge les nouveaux nées prematurés , le cas echeant ou cela arriverait au sein de votre centre de santé? [00:34:26-6](http://localhost:2300/file=C:/Users/USER/Desktop/ENTRETIENS)

[00:34:26-6](http://localhost:2300/file=C:/Users/USER/Desktop/ENTRETIENS%20PREMATURITE/CSPS-ACCART-VILLE/FOCUS%20GROUPE%20ACCART-VILLE-BON.mp3time=2066600)Person 3: oui nous pouvons dire que nous avons le personnel qualifié mais quand on parle du personnel il faut un recyclage parce que le travail qu'on ne fait pas tous les jours on ne peut pas le faire correctement, quelqu'un disait qu'on fait bien ce qu'on fait tous les jours. a defaut c'est le recyclage parce que nous avons 2 medecins 8 sages femme, 5 accoucheuses donc je pense qu'au niveau du personnel ça va. au niveau du personnel il y a le minimum pour prendre en charge le nouveau né prematuré. [00:34:59-7](http://localhost:2300/file=C:/Users/USER/Desktop/ENTRETIENS)

[00:34:59-7](http://localhost:2300/file=C:/Users/USER/Desktop/ENTRETIENS%20PREMATURITE/CSPS-ACCART-VILLE/FOCUS%20GROUPE%20ACCART-VILLE-BON.mp3time=2099700) Interviewer: ok nous allons toujours pousser plus loin les propositions d'ameliorations, nous avons constater qu'il y a des mouvements des agents de santé pour cause d'affection ou autre, comment faire pour ne pas perdre la qualité des prises en charge au sein du centre de santé? [00:35:51-8](http://localhost:2300/file=C:/Users/USER/Desktop/ENTRETIENS%20PREMATURITE/CSPS-ACCART-VILLE/FOCUS%20GROUPE%20ACCART-VILLE-BON.mp3time=2151800)

[00:35:51-8](http://localhost:2300/file=C:/Users/USER/Desktop/ENTRETIENS%20PREMATURITE/CSPS-ACCART-VILLE/FOCUS%20GROUPE%20ACCART-VILLE-BON.mp3time=2151800)Person 1: bon moi je me dis que lorsqu'on forme un certain nombre d'agent de santé,c'est pour que ses agents partagent leurs competences avec ceux qui n'ont pas beneficier de ladite formation puisqu'on ne peut pas prendre tout le monde pour former en même temps. Même si c'est 2 ou 3 agents qu'on arrive à former, ses agent à leur retour peuvent mettre en application pour que ceux qui sont restés suivent afin d'apprendre pour mieux intervenir. la formation ce n'est pas pour garder pour soi même, je pense que c'est pour venir appliquer afin que les autres puissent apprendre afin de pour être plus efficace et même si ceux qui ont réçu la formation partent ailleurs, les autres qui ont beneficier de ce partage de competence pourront toujours assurer ce service. [00:36:44-8](%22)

[00:36:44-8](http://localhost:2300/file=C:/Users/USER/Desktop/ENTRETIENS) Person 3: ce qu'on ajouter à ce niveau, effectivement cela est un problème, les agents sont formés et avec les mouvements ceux qui ont été formé peuvent être amenés à partir ailleurs , le centre devient orphélin des agents qualifiés pour la prise en charge des nouveaux nés. par experience, au titre des propositions je pense qu'avant de former on peut donner un certain nombre d'année que la personne doit faire. ou bien identifier les gens qui sont de la localité ou identifier ceux qui ont decider par convenance personnel de rester dans le centre pendant un certain nombre d'année. aussi, la structure peut faire signer un engagement de 3 ans minimum par exemple à ceux qui vont participer à la formation de rester dans le centre. [00:38:20-6](http://localhost:2300/file=C:/Users/USER/Desktop/ENTRETIENS%20PREMATURITE/CSPS-ACCART-VILLE/FOCUS%20GROUPE%20ACCART-VILLE-BON.mp3time=2300600)

00:31:34-9 Interviewer: D'accord, nous allons poursuivre. Comment peut-on améliorer la prise en charge des naissances prématurées dans votre centre de santé ? Qu'est-ce qu'il faut faire selon vous pour améliorer la prise en charge des naissances prématurées au sein de votre centre de santé ? 00:31:56-8

00:31:56-8 Person 3: Bon, je pense à travers la sensibilisation surtout dans la communauté, que les femmes viennent très tôt commencer la CPN, la prise du fer et l'alimentation sont très importantes. Que le suivi soit vraiment correct. Avec la sensibilisation on peut éviter la prématurité. 00:32:28-4

00:32:28-4 Interviewer: Est-ce qu'il y a autres choses à ajouter ? 00:32:31-6

00:32:31-6 Person 3: Ce qu'on peut dire, on va situer la question à deux étapes. La première étape c'est de lutter contre la prématurité, d'abord c'est-à-dire prendre en charge correctement la menace des accouchements prématurés en faisant un suivi correct des grossesses pour éviter la prématurité et une fois que la prématurité est déjà installée, il faut prendre en charge, à savoir il faut lutter contre les infections, lutter contre l'hypothermie. Maintenant comme précédemment dit plus haut, à notre niveau nous n'avons pas de matériels nécessaires pour prendre correctement en charge les nouveau-nés prématurés. Au niveau du personnel, nous pouvons dire que ça va puisque nous avons deux médecins ici mais c'est au niveau du matériel médico-technique que ça pose problème en commençant par les locaux. Nous n'avons pas une salle dédiée uniquement aux prématurés, nous n'avons pas également de couveuses, d'appareil d'échographie pour pouvoir dater les grossesses correctement et situer même le type de prématurité. Donc de façon générale, le matériel nous manque pour prendre en charge de façon efficace les nouveau-nés prématurés. 00:33:56-2

00:33:56-2 Interviewer: Est-ce qu'il y a quelque chose d'autre à ajouter ? Est-ce que par rapport à toutes ces propositions le centre de santé dispose de personnel qualifié pour prendre en charge les nouveau-nés prématurés, le cas échéant où cela arriverait au sein de votre centre de santé ? 00:34:26-6

00:34:26-6 Person 3: Oui, nous pouvons dire que nous avons le personnel qualifié mais quand on parle du personnel, il faut un recyclage parce que le travail qu'on ne fait pas tous les jours on ne peut pas le faire correctement, quelqu'un disait qu'on fait bien ce qu'on fait tous les jours. À défaut, c'est le recyclage parce que nous avons 2 médecins, 8 sage-femmes, 5 accoucheuses donc je pense qu'au niveau du personnel ça va. Au niveau du personnel, il y a le minimum pour prendre en charge le nouveau-né prématuré. 00:34:59-7

00:34:59-7 Interviewer: Ok, nous allons toujours pousser plus loin les propositions d'améliorations, nous avons constaté qu'il y a des mouvements des agents de santé pour cause d'affectation ou autre, comment faire pour ne pas perdre la qualité des prises en charge au sein du centre de santé ? 00:35:51-8

00:35:51-8 Person 1: Bon, moi je me dis que lorsqu'on forme un certain nombre d'agents de santé, c'est pour que ces agents partagent leurs compétences avec ceux qui n'ont pas bénéficié de ladite formation puisqu'on ne peut pas prendre tout le monde pour former en même temps. Même si c'est 2 ou 3 agents qu'on arrive à former, ces agents à leur retour peuvent mettre en application pour que ceux qui sont restés suivent afin d'apprendre pour mieux intervenir. La formation ce n'est pas pour garder pour soi-même, je pense que c'est pour venir appliquer afin que les autres puissent apprendre afin d'être plus efficace et même si ceux qui ont reçu la formation partent ailleurs, les autres qui ont bénéficié de ce partage de compétence pourront toujours assurer ce service. 00:36:44-8

00:36:44-8 Person 3: Ce qu'on ajouter à ce niveau, effectivement cela est un problème, les agents sont formés et avec les mouvements ceux qui ont été formés peuvent être amenés à partir ailleurs, le centre devient orphelin des agents qualifiés pour la prise en charge des nouveau-nés. Par expérience, au titre des propositions, je pense qu'avant de former on peut donner un certain nombre d'années que la personne doit faire. Ou bien identifier les gens qui sont de la localité ou identifier ceux qui ont décidé par convenance personnelle de rester dans le centre pendant un certain nombre d'années. Aussi, la structure peut faire signer un engagement de 3 ans minimum par exemple à ceux qui vont participer à la formation de rester dans le centre. 00:38:20-6

**00:31:34-9 Interviewer:** Okay, let's move on. How can the management of premature births in your health center be improved? What steps do you think should be taken?

**00:31:56-8 Person 3:** Raising awareness in the community is crucial. Women should start antenatal care (ANC) early and focus on taking iron supplements and maintaining a good diet. Proper follow-up care is also key. With increased awareness, we can potentially reduce the incidence of prematurity.

**00:32:28-4 Interviewer:** Is there anything else you'd like to add?

**00:32:31-6 Person 3:** We can approach this in two stages. Firstly, we need to focus on preventing prematurity by closely monitoring pregnancies. Once prematurity occurs, managing issues like infections and hypothermia is vital. However, we currently lack the necessary medical-technical equipment, including a dedicated room for premature babies, incubators, and ultrasound machines. While we have two doctors and adequate staff, the challenge lies in having the right equipment.

**00:34:26-6 Interviewer:** In terms of staff, does the health center have qualified personnel to take care of premature newborns?

**00:34:26-6 Person 3:** Yes, we have qualified staff, but continuous training is essential since the skills needed for this care are not used daily. The idea is to train a few staff members who can then share their knowledge with others, ensuring skills are passed on and care quality is maintained even if trained personnel leave.

**00:35:51-8 Interviewer:** How can we avoid losing the quality of care within the health center due to staff movements?

**00:35:51-8 Person 1:** When we train health agents, they should share their skills with those who didn’t receive the training. This way, the knowledge is disseminated, ensuring continuous and effective care. Even if trained staff move on, the remaining staff who have learned from them can maintain the quality of care.

**00:38:20-6 Person 3:** To add, staff movement is indeed a challenge. To address this, we could have a policy where staff sign a commitment to stay for a certain number of years after receiving training or focus on training local staff who are more likely to remain in the center.

### Manager of Accart-ville urban medical center

[00:21:18-8](http://localhost:2300/file=C:/Users/USER/Desktop/ENTRETIENS%20PREMATURITE/CSPS-ACCART-VILLE/RESPONSABLE%20CMU%20ACCART-VILLE.MP3time=1278800)Interviewer: maintenant selon vous, comment est ce qu'on peut améliorer la prise en charge des naissances prématurées au sein de votre centre de santé? [00:21:28-8](http://localhost:2300/file=C:/Users/USER/Desktop/ENTRETIENS%20PREMATURITE/CSPS-ACCART-VILLE/RESPONSABLE%20CMU%20ACCART-VILLE.MP3time=1288800)

[00:21:28-8](http://localhost:2300/file=C:/Users/USER/Desktop/ENTRETIENS%20PREMATURITE/CSPS-ACCART-VILLE/RESPONSABLE%20CMU%20ACCART-VILLE.MP3time=1288800)Person 1: le premier point, c'est d'abord le plateau technique et le personnel. Si on nous affecte plus des personnels, je pense qu'on peut essayer de voir comme on peut améliorer la prise en charge des femmes afin d'éviter d'abord la prématurés chez nous d'abord . Si on gagne des prématurés, au moins que nous arrivons à faire quelque chose pour pouvoir désengorger au moins les niveaux superieurs que ce soit le CMA ou au niveau du CHURSS.Il n y a même pas de couveuses au niveau du CMA, c'est au niveau du CHURSS et cela entraine une surcharge au sein du CHURSS.si un enfant a besoin d'oxygène nous n'en avons pas alors cela pose un problème. Si le materiel et le plateau y est je pense que les competences vont suivre avec. je pense que les gens ont déjà des bases, on peut essayer d'ameliorer si le plateau technique est disponible. [00:22:36-7](http://localhost:2300/file=C:/Users/USER/Desktop/ENTRETIENS%20PREMATURITE/CSPS-ACCART-VILLE/RESPONSABLE%20CMU%20ACCART-VILLE.MP3time=1356700)

[00:22:36-7](http://localhost:2300/file=C:/Users/USER/Desktop/ENTRETIENS%20PREMATURITE/CSPS-ACCART-VILLE/RESPONSABLE%20CMU%20ACCART-VILLE.MP3time=1356700)Interviewer: Est ce que vous pensez que les mouvements des agents de santé peuvent impacter sur la qualité de service au niveau du centre de santé par rapport à ceux qui ont reçu la formation specifique sur la prématurité? [00:23:01-3](http://localhost:2300/file=C:/Users/USER/Desktop/ENTRETIENS%20PREMATURITE/CSPS-ACCART-VILLE/RESPONSABLE%20CMU%20ACCART-VILLE.MP3time=1381300)

[00:23:01-3](http://localhost:2300/file=C:/Users/USER/Desktop/ENTRETIENS%20PREMATURITE/CSPS-ACCART-VILLE/RESPONSABLE%20CMU%20ACCART-VILLE.MP3time=1381300) Person 1: le mouvement des agents. bon, c'est vrai, ça peut jouer sur le service, mais en réalité, ça ne doit pas affecter sur la continuité du service. c'est vrai si on perd des compétences, ceux qui sont déjà habitués partent ailleurs et ceux qui vont venir, il faut un temps pour qu'ils s'impregnent de la chose. Mais je pense que c'est quelque chose qui peut être gérable puisque dans leurs mouvements en fait, tout le monde ne part pas en même temps avec c'est 1 ou 2, sinon on affecte pas tout le monde en même temps. je pense que ça c'est quelque chose qui est gérable en fait. [00:23:56-8](http://localhost:2300/file=C:/Users/USER/Desktop/ENTRETIENS%20PREMATURITE/CSPS-ACCART-VILLE/RESPONSABLE%20CMU%20ACCART-VILLE.MP3time=1436800)

[00:23:56-8](http://localhost:2300/file=C:/Users/USER/Desktop/ENTRETIENS%20PREMATURITE/CSPS-ACCART-VILLE/RESPONSABLE%20CMU%20ACCART-VILLE.MP3time=1436800) Interviewer: Maintenant, comment est ce que la femme prématurée ou enfant prématuré est perçue en communauté? Est ce que vous pensez que cette perception peut influencer un peu les differentes prises en charges? [00:24:12-2](http://localhost:2300/file=C:/Users/USER/Desktop/ENTRETIENS%20PREMATURITE/CSPS-ACCART-VILLE/RESPONSABLE%20CMU%20ACCART-VILLE.MP3time=1452200)

[00:24:12-2](http://localhost:2300/file=C:/Users/USER/Desktop/ENTRETIENS%20PREMATURITE/CSPS-ACCART-VILLE/RESPONSABLE%20CMU%20ACCART-VILLE.MP3time=1452200)Person 1: bon je ne pense pas que cela puisse jouer sur la prise en charge en quelque sorte. mais dans mon constat, comme nous avons l'habitude de le dire le prématuré rattrape son retard generalement s'il devrait être né à neuf mois et qu'il soit venu à sept mois donc cela veut dire qu'à deux mois il va rattraper son retard et on ne va même pas savoir qu'il était prématuré. dans la société il y a une stigmatisation qui se pose même s'il devient grand ,on dira toujours qu'il était prématuré et cela fait mal parfois.même dans mon quartier quand j'attends dire un tel est prématuré je dis toujours qu'on ne plus le considerer comme prématuré parce qu'il a déjà rattraper son retard. si on stigmatise les gens( prématurés) cela peut jouer sur eux en fait. Parce qu'en ce moment, la stigmatisation, ça peut créer des sentiments de rejet. [00:25:12-9](http://localhost:2300/file=C:/Users/USER/Desktop/ENTRETIENS%20PREMATURITE/CSPS-ACCART-VILLE/RESPONSABLE%20CMU%20ACCART-VILLE.MP3time=1512900)

[00:25:12-9](http://localhost:2300/file=C:/Users/USER/Desktop/ENTRETIENS%20PREMATURITE/CSPS-ACCART-VILLE/RESPONSABLE%20CMU%20ACCART-VILLE.MP3time=1512900)Interviewer: Est ce que l'amour,les perceptions de la communauté par rapport à la prématurité a un impact sur la santé et affecter un peu le travail des agents de santé et alourdir un peu le travail au niveau de la sensibilisation dans les différents centres de santé? [00:25:31-9](http://localhost:2300/file=C:/Users/USER/Desktop/ENTRETIENS%20PREMATURITE/CSPS-ACCART-VILLE/RESPONSABLE%20CMU%20ACCART-VILLE.MP3time=1531900)

[00:25:31-9](http://localhost:2300/file=C:/Users/USER/Desktop/ENTRETIENS%20PREMATURITE/CSPS-ACCART-VILLE/RESPONSABLE%20CMU%20ACCART-VILLE.MP3time=1531900)Person 1: Oui mais bon par rapport à ça santé, moi je pense que ça ne pose pas problème, sauf si c'est le mental en fait, c'est au niveau du mental que généralement ça peut jouer au niveau psychologique, c'est pas sur sa santé physique en tant que tel,c'est la stigmatisation qui pose problème par exemple en 2 mois pour un prématuré qui est né à 7 mois comme je l'ai dis, en 2 mois s'il ratrappe son retard cela veut dire qu'il a déjà recuperé comme celui qui est né à terme donc cela ne plus poser de problème au niveau de son physique. c'est le ragard de la société qui va entrainer une stygmatisation qui est plus forte, sinon je ne pense pas que physiquement cela puisse poser problème. [00:26:15-6](http://localhost:2300/file=C:/Users/USER/Desktop/ENTRETIENS%20PREMATURITE/CSPS-ACCART-VILLE/RESPONSABLE%20CMU%20ACCART-VILLE.MP3time=1575600)

00:21:18-8 Interviewer: Maintenant selon vous, comment est-ce qu'on peut améliorer la prise en charge des naissances prématurées au sein de votre centre de santé ? 00:21:28-8

00:21:28-8 Person 1: Le premier point, c'est d'abord le plateau technique et le personnel. Si on nous affecte plus de personnels, je pense qu'on peut essayer de voir comment on peut améliorer la prise en charge des femmes afin d'éviter d'abord les prématurés chez nous d'abord. Si on gagne des prématurés, au moins que nous arrivons à faire quelque chose pour pouvoir désengorger au moins les niveaux supérieurs que ce soit le CMA ou au niveau du CHUSS. Il n'y a même pas de couveuses au niveau du CMA, c'est au niveau du CHUSS et cela entraîne une surcharge au sein du CHUSS. Si un enfant a besoin d'oxygène, nous n'en avons pas alors cela pose un problème. Si le matériel et le plateau y est, je pense que les compétences vont suivre avec. Je pense que les gens ont déjà des bases, on peut essayer d'améliorer si le plateau technique est disponible. 00:22:36-7

00:22:36-7 Interviewer: Est-ce que vous pensez que les mouvements des agents de santé peuvent impacter sur la qualité de service au niveau du centre de santé par rapport à ceux qui ont reçu la formation spécifique sur la prématurité ? 00:23:01-3

00:23:01-3 Person 1: Le mouvement des agents. Bon, c'est vrai, ça peut jouer sur le service, mais en réalité, ça ne doit pas affecter sur la continuité du service. C'est vrai si on perd des compétences, ceux qui sont déjà habitués partent ailleurs et ceux qui vont venir, il faut un temps pour qu'ils s'imprègnent de la chose. Mais je pense que c'est quelque chose qui peut être gérable puisque dans leurs mouvements en fait, tout le monde ne part pas en même temps avec c'est 1 ou 2, sinon on n'affecte pas tout le monde en même temps. Je pense que ça c'est quelque chose qui est gérable en fait. 00:23:56-8

00:23:56-8 Interviewer: Maintenant, comment est-ce que la femme prématurée ou l'enfant prématuré est perçu en communauté ? Est-ce que vous pensez que cette perception peut influencer un peu les différentes prises en charge ? 00:24:12-2

00:24:12-2 Personne 1: Bon, je ne pense pas que cela puisse jouer sur la prise en charge en quelque sorte. Mais dans mon constat, comme nous avons l'habitude de le dire, le prématuré rattrape son retard généralement s'il devait être né à neuf mois et qu'il est venu à sept mois, donc cela veut dire qu'à deux mois il va rattraper son retard et on ne va même pas savoir qu'il était prématuré. Dans la société, il y a une stigmatisation qui se pose, même s'il devient grand, on dira toujours qu'il était prématuré et cela fait mal parfois. Même dans mon quartier quand j'entends dire qu'un tel est prématuré, je dis toujours qu'on ne peut plus le considérer comme prématuré parce qu'il a déjà rattrapé son retard. Si on stigmatise les gens (prématurés), cela peut jouer sur eux en fait. Parce qu'en ce moment, la stigmatisation, ça peut créer des sentiments de rejet. 00:25:12-9

00:25:12-9 Interviewer: Est-ce que l'amour, les perceptions de la communauté par rapport à la prématurité, a un impact sur la santé et affecte un peu le travail des agents de santé et alourdit un peu le travail au niveau de la sensibilisation dans les différents centres de santé ? 00:25:31-9

00:25:31-9 Personne 1: Oui, mais bon par rapport à sa santé, moi je pense que ça ne pose pas de problème, sauf si c'est au niveau mental en fait, c'est au niveau du mental que généralement ça peut jouer, au niveau psychologique, ce n'est pas sur sa santé physique en tant que tel, c'est la stigmatisation qui pose problème. Par exemple, en 2 mois pour un prématuré qui est né à 7 mois, comme je l'ai dit, en 2 mois s'il rattrape son retard, cela veut dire qu'il a déjà récupéré comme celui qui est né à terme donc cela ne pose plus de problème au niveau de son physique. C'est le regard de la société qui va entraîner une stigmatisation qui est plus forte, sinon je ne pense pas que physiquement cela puisse poser problème. 00:26:15-6

**00:21:18-8 Interviewer:** In your opinion, how can the management of premature births be improved within your health center?

**00:21:28-8 Person 1:** The first step is enhancing our technical platform and staffing. More staff would allow us to improve care for women, aiming to reduce premature births. If we do have premature babies, we need to be able to manage them effectively to ease the burden on higher-level facilities like the CMA or CHUSS. Currently, we lack incubators, which are only available at the CHUSS level, causing overcrowding there. The lack of essential equipment like oxygen is a significant problem. If we had the right equipment and infrastructure, I believe the necessary skills among our staff would follow. We have the basic knowledge, but we need an improved technical platform to enhance our capabilities.

**00:22:36-7 Interviewer:** Do you think the movement of health workers impacts the quality of service at the health center, especially concerning those who have received specific training on prematurity?

**00:23:01-3 Person 1:** Staff movement can affect the service, but it shouldn't disrupt continuity. Losing skilled personnel who are familiar with prematurity care and onboarding new staff does take time. However, typically not everyone leaves at once; it's usually one or two people at a time. So, I believe it's a manageable situation. We can maintain service quality despite these movements.

**Interviewer (00:23:56-8):** "How is a premature baby or a premature birth perceived in the community? Do you think this perception can affect the care they receive?"

**Person 1 (00:24:12-2):** "In my opinion, the community's perception doesn't directly affect the support provided. However, there's a common belief that premature children eventually catch up in development. For example, a child born two months early is often expected to reach developmental milestones within two months. Despite this, there's still a stigma attached to being premature, which can be hurtful. Even when a child has caught up developmentally, they may still be labeled as 'premature' in the community. This kind of stigma can lead to feelings of rejection, which can be psychologically damaging."

**Interviewer (00:25:12-9):** "Do these community perceptions and attitudes towards prematurity impact health outcomes and the work of health professionals, especially in terms of raising awareness?"

**Person 1 (00:25:31-9):** "Yes, but I believe it doesn't pose a physical health issue, rather a mental or psychological one. For instance, if a baby born at 7 months catches up developmentally in 2 months, physically, they're no different from a full-term baby. However, societal views and stigmatization can have a profound impact. It's not the physical aspect but the social stigma that's the real problem.

### Maternity manager of Accart-ville urban medical center

[00:26:04-4](http://localhost:2300/file=C:/Users/USER/Desktop/ENTRETIENS%20PREMATURITE/CSPS-ACCART-VILLE/RESPONSABLE%20MATERNITE%20CMU%20ACCART-VILLE.MP3time=1564400) Interviewer: maintenant au vue de toutes ses contraintes et defis, comment peut on ameliorer la prise en charge des naissances prematurés dans votre centre de santé? [00:26:19-0](http://localhost:2300/file=C:/Users/USER/Desktop/ENTRETIENS%20PREMATURITE/CSPS-ACCART-VILLE/RESPONSABLE%20MATERNITE%20CMU%20ACCART-VILLE.MP3time=1579000)

[00:26:19-0](http://localhost:2300/file=C:/Users/USER/Desktop/ENTRETIENS%20PREMATURITE/CSPS-ACCART-VILLE/RESPONSABLE%20MATERNITE%20CMU%20ACCART-VILLE.MP3time=1579000) Person 1: la solution que nous pouvons proposer c'est d'avoir une salle specifique pour les nouveaux-nées prematurés, il est vrai que nous ne prenons pas en charge mais si nous pouvons avoir des couveuses cela serait bien parce que quand on evacue à l'hopital les bébés nous reviennent souvent et aussi le temps que la femme va mettre pour attendre son tour c'est à dire une couveuse de libre joue sur la survie du nouveau-née.si nous à notre niveau nous avons l'équipement pour lever l'urgence mettre l'enfant dans une couveuse avec une lampe chauffante pendant un bout de temps pour que l'enfant se ressaisisse un peu avant de l'évacuer. la femme ira en ce moment à l'hopital pour aller voire uniquement un pediatre. il serait bien également que le centre dispose d'un pediatre ou d'un attaché de santé en pediatrie cela peut nous aider à ameliorer la prise en charge des enfants en general et des nouveaux-nées en particulier. donc les besoin c'est d'avoir des couveuses, une salle specifique dediée aux nouveaux-nées, des lampes chauffantes. [00:27:40-4](http://localhost:2300/file=C:/Users/USER/Desktop/ENTRETIENS%20PREMATURITE/CSPS-ACCART-VILLE/RESPONSABLE%20MATERNITE%20CMU%20ACCART-VILLE.MP3time=1660400)

[00:27:40-4](http://localhost:2300/file=C:/Users/USER/Desktop/ENTRETIENS%20PREMATURITE/CSPS-ACCART-VILLE/RESPONSABLE%20MATERNITE%20CMU%20ACCART-VILLE.MP3time=1660400)Interviewer: les formations de qualités ou des recyclages? [00:27:44-0](http://localhost:2300/file=C:/Users/USER/Desktop/ENTRETIENS%20PREMATURITE/CSPS-ACCART-VILLE/RESPONSABLE%20MATERNITE%20CMU%20ACCART-VILLE.MP3time=1664000)

[00:27:44-0](http://localhost:2300/file=C:/Users/USER/Desktop/ENTRETIENS%20PREMATURITE/CSPS-ACCART-VILLE/RESPONSABLE%20MATERNITE%20CMU%20ACCART-VILLE.MP3time=1664000) Person 1: le recyclage aussi peut aiderparce que affecter un pediatre ou un attaché de santé en pediatrie c'est un peu compliqué mais à defaut si nous pouvons beneficier d'un recyclage dans la prise en charge du nouveau née en general et du nouveau-née prematuré en particulier cela peut nous aider à lutter contre la mortalité néo-natale et particulierement lamortalité des nouveaux-nées prématurés qui est très élèvé. [00:28:21-7](http://localhost:2300/file=C:/Users/USER/Desktop/ENTRETIENS%20PREMATURITE/CSPS-ACCART-VILLE/RESPONSABLE%20MATERNITE%20CMU%20ACCART-VILLE.MP3time=1701700)

[00:28:21-7](http://localhost:2300/file=C:/Users/USER/Desktop/ENTRETIENS%20PREMATURITE/CSPS-ACCART-VILLE/RESPONSABLE%20MATERNITE%20CMU%20ACCART-VILLE.MP3time=1701700) Interviewer: maintenant par rapport aux mouvements ou affectations des agents de santé, est ce que cela ne constitue pas un handicap au sein de la formation sanitaire du fait que ceux qui ont beneficier de la formation ou du recyclage vont ailleurs? [00:28:47-2](http://localhost:2300/file=C:/Users/USER/Desktop/ENTRETIENS%20PREMATURITE/CSPS-ACCART-VILLE/RESPONSABLE%20MATERNITE%20CMU%20ACCART-VILLE.MP3time=1727200)

[00:28:47-2](http://localhost:2300/file=C:/Users/USER/Desktop/ENTRETIENS%20PREMATURITE/CSPS-ACCART-VILLE/RESPONSABLE%20MATERNITE%20CMU%20ACCART-VILLE.MP3time=1727200) Person 1: effectivement c'est un phenomène qui existe, partout il y a ses plaintes parce que quand nous sortons pour la suppervision nous constatons qu'il n'y a pas d'agents formés et que ceux qui ont été formé sont partis. maintenant ce qu'on proposer comme solution à ce niveau c'est d'identifier les agents qui seront formés et leurs faire signer un engagement en determinant un certain nombre d'années qu'ils vont faire au sein de la formation sanitaire. une autre solution après des echanges avec des pediatres, c'est de former des gens, pas forcement du centre de santé, qui vont etre comme des mentors et travailler une semaine par trimestre en temps réel dans la formation sanitaire pour former les sages femmes à prendre en charge les nouveaux-nées. ce sont ses deux aspects que je peut proposer comme solution, la première c'est de faire signer un engagement à ceux qui vont beneficier de la formation et definir le nombre d'années qu'ils font faire obligatoirement avant de pouvoir postuler pour une affectation, à defaut recruter des personnels qualifiés dans la prise en charge des nouveaux-nées prématurés. il ezt possible aussi de former des personnels qui vont etre comme des mentors en leurs affectant un certain nombre de centre de santé, ils vont travailler au niveau de ces centre une semaine en temps réel avec les differents agents des services pour enseigner et expliquer la prise en charge du nouveau-née en general et du prématuré en particulier.ce sont ces deux solutions que je peux proposer par rapport aux mouvements permanents des agents de santés qui sont formés et qui vont se retrouver à quelque part et que cela ne profite pas au service. [00:31:07-4](http://localhost:2300/file=C:/Users/USER/Desktop/ENTRETIENS%20PREMATURITE/CSPS-ACCART-VILLE/RESPONSABLE%20MATERNITE%20CMU%20ACCART-VILLE.MP3time=1867400)

00:26:04-4 Interviewer: Maintenant, au vue de toutes ces contraintes et défis, comment peut-on améliorer la prise en charge des naissances prématurées dans votre centre de santé ? 00:26:19-0

00:26:19-0 Person 1: La solution que nous pouvons proposer c'est d'avoir une salle spécifique pour les nouveau-nés prématurés. Il est vrai que nous ne prenons pas en charge mais si nous pouvons avoir des couveuses cela serait bien parce que quand on évacue à l'hôpital, les bébés nous reviennent souvent et aussi le temps que la femme va mettre pour attendre son tour, c'est-à-dire une couveuse de libre, joue sur la survie du nouveau-né. Si nous, à notre niveau, avons l'équipement pour lever l'urgence, mettre l'enfant dans une couveuse avec une lampe chauffante pendant un bout de temps pour que l'enfant se ressaisisse un peu avant de l'évacuer. La femme ira en ce moment à l'hôpital pour aller voir uniquement un pédiatre. Il serait bien également que le centre dispose d'un pédiatre ou d'un attaché de santé en pédiatrie, cela peut nous aider à améliorer la prise en charge des enfants en général et des nouveau-nés en particulier. Donc les besoins, c'est d'avoir des couveuses, une salle spécifique dédiée aux nouveau-nés, des lampes chauffantes. 00:27:40-4

00:27:40-4 Interviewer: Les formations de qualités ou des recyclages ? 00:27:44-0

00:27:44-0 Person 1: Le recyclage aussi peut aider parce que affecter un pédiatre ou un attaché de santé en pédiatrie, c'est un peu compliqué, mais à défaut, si nous pouvons bénéficier d'un recyclage dans la prise en charge du nouveau-né en général et du nouveau-né prématuré en particulier, cela peut nous aider à lutter contre la mortalité néonatale et particulièrement la mortalité des nouveau-nés prématurés qui est très élevée. 00:28:21-7

00:28:21-7 Interviewer: Maintenant, par rapport aux mouvements ou affectations des agents de santé, est-ce que cela ne constitue pas un handicap au sein de la formation sanitaire du fait que ceux qui ont bénéficié de la formation ou du recyclage vont ailleurs ? 00:28:47-2

00:28:47-2 Person 1: Effectivement, c'est un phénomène qui existe, partout il y a ces plaintes parce que quand nous sortons pour la supervision, nous constatons qu'il n'y a pas d'agents formés et que ceux qui ont été formés sont partis. Maintenant, ce qu'on proposerait comme solution à ce niveau, c'est d'identifier les agents qui seront formés et leur faire signer un engagement en déterminant un certain nombre d'années qu'ils vont faire au sein de la formation sanitaire. Une autre solution, après des échanges avec des pédiatres, c'est de former des gens, pas forcément du centre de santé, qui vont être comme des mentors et travailler une semaine par trimestre en temps réel dans la formation sanitaire pour former les sage-femmes à prendre en charge les nouveau-nés. Ce sont ces deux aspects que je peux proposer comme solution, la première c'est de faire signer un engagement à ceux qui vont bénéficier de la formation et définir le nombre d'années qu'ils feront obligatoirement avant de pouvoir postuler pour une affectation, à défaut recruter des personnels qualifiés dans la prise en charge des nouveau-nés prématurés. Il est possible aussi de former des personnels qui vont être comme des mentors en leur affectant un certain nombre de centres de santé, ils vont travailler au niveau de ces centres une semaine en temps réel avec les différents agents des services pour enseigner et expliquer la prise en charge du nouveau-né en général et du prématuré en particulier. Ce sont ces deux solutions que je peux proposer par rapport aux mouvements permanents des agents de santé qui sont formés et qui vont se retrouver à quelque part et que cela ne profite pas au service. 00:31:07-4

**00:26:04-4 Interviewer:** Considering these constraints and challenges, how can the management of premature births be improved in your health center?

**00:26:19-0 Person 1:** One solution is to have a specific room dedicated to premature newborns. Even though we don't currently manage them, having incubators would be beneficial. When we evacuate babies to the hospital, they often return to us, and waiting for an available incubator impacts their survival. If we had emergency equipment like incubators and heat lamps, we could stabilize the child before evacuation. It would also be helpful if our center had a pediatrician or a pediatric health representative to improve overall care for children, especially newborns. So, our needs include incubators, a dedicated room for newborns, and heating lamps.

**00:27:40-4 Interviewer:** What about quality training or refresher courses?

**00:27:44-0 Person 1:** Retraining is also crucial. While having a pediatrician or pediatric health officer is ideal, retraining in newborn care, especially for premature infants, can help reduce neonatal mortality.

**00:28:21-7 Interviewer:** Regarding staff movements or assignments, does this not pose a challenge for the health facility?

**00:28:47-2 Person 1:** It is a challenge, as trained staff often move to other locations. A potential solution is to have trained staff sign a commitment to stay for a certain number of years. Another approach could be to train mentors not necessarily from our health center. These mentors could work for a week per quarter in real-time at the center, training our staff on newborn care. This way, even if trained staff leave, there is continued mentorship and knowledge transfer. These solutions could help maintain a consistent level of care despite the movement of trained health workers.

### Focus group of Do medical center with surgical branch

[00:22:28-1](http://localhost:2300/file=C:/Users/USER/Desktop/ENTRETIENS%20PREMATURITE/CMA-DO/FOCUS%20GROUPE%20CMA-DO.mp3time=1348100) Interviewer: Maintenant, qu'est ce que vous properiez pour ameliorer la prise en charge des naissances prématurées au sein de votre centre de santé? [00:22:40-0](http://localhost:2300/file=C:/Users/USER/Desktop/ENTRETIENS%20PREMATURITE/CMA-DO/FOCUS%20GROUPE%20CMA-DO.mp3time=1360000)

[00:22:40-0](http://localhost:2300/file=C:/Users/USER/Desktop/ENTRETIENS%20PREMATURITE/CMA-DO/FOCUS%20GROUPE%20CMA-DO.mp3time=1360000) Person 3: Il faut des couveuses, il faut qu'il y ait un sevice dedié à la prématurité,une néonatologie avec les équipements et le personnel qu'il faut. [00:22:58-1](http://localhost:2300/file=C:/Users/USER/Desktop/ENTRETIENS%20PREMATURITE/CMA-DO/FOCUS%20GROUPE%20CMA-DO.mp3time=1378100)

[00:22:58-1](http://localhost:2300/file=C:/Users/USER/Desktop/ENTRETIENS%20PREMATURITE/CMA-DO/FOCUS%20GROUPE%20CMA-DO.mp3time=1378100) Interviewer: Est ce qu'il y a quelque chose d'autre à ajouter? [00:23:03-0](http://localhost:2300/file=C:/Users/USER/Desktop/ENTRETIENS%20PREMATURITE/CMA-DO/FOCUS%20GROUPE%20CMA-DO.mp3time=1383000)

[00:23:03-0](http://localhost:2300/file=C:/Users/USER/Desktop/ENTRETIENS%20PREMATURITE/CMA-DO/FOCUS%20GROUPE%20CMA-DO.mp3time=1383000) Person 9: Renforcer notre materiel de réanimation, les lampes chauffantes, l'oxygenation, l'aspiration aussi, surtout les sondes naso-gastriques qui sont très rares. Souvent lorsqu'il n y en a pas, il faut envoyer payer et c'est un peu chère.Si nous en avons ici ,on doit pouvoir gerer. [00:23:34-9](http://localhost:2300/file=C:/Users/USER/Desktop/ENTRETIENS%20PREMATURITE/CMA-DO/FOCUS%20GROUPE%20CMA-DO.mp3time=1414900)

[00:23:34-9](http://localhost:2300/file=C:/Users/USER/Desktop/ENTRETIENS%20PREMATURITE/CMA-DO/FOCUS%20GROUPE%20CMA-DO.mp3time=1414900) Interviewer: Est ce que vous pensez que les pesenteurs socio-culturelles peuvent impacter la santé du nourrisson prématuré et de la mère et alourdir du même coup votre charge de travail concernant la prise en charge? [00:23:44-8](http://localhost:2300/file=C:/Users/USER/Desktop/ENTRETIENS%20PREMATURITE/CMA-DO/FOCUS%20GROUPE%20CMA-DO.mp3time=1424800)

[00:23:44-8](http://localhost:2300/file=C:/Users/USER/Desktop/ENTRETIENS%20PREMATURITE/CMA-DO/FOCUS%20GROUPE%20CMA-DO.mp3time=1424800) Person 1: Oui oui (tous) [00:23:51-8](http://localhost:2300/file=C:/Users/USER/Desktop/ENTRETIENS%20PREMATURITE/CMA-DO/FOCUS%20GROUPE%20CMA-DO.mp3time=1431800)

[00:23:51-8](http://localhost:2300/file=C:/Users/USER/Desktop/ENTRETIENS%20PREMATURITE/CMA-DO/FOCUS%20GROUPE%20CMA-DO.mp3time=1431800) Person 9: Ce sont les bains des bébés. Quand les bébés arrivent, il y a des specialistes de bain de bébé, pourtant les bébés prématurés ont besoin d'être au chaud après la sortie. il y a des vielles qui trempent ses bébés là dans l'eau, souvent c'est difficile de leurs faire comprendre que ce n'est pas necessaire, parce qu'ils sont convaincus que ce bébé, s'il n'est pas lavé, va sentir plus tard ou bien en tout cas va avoir peut être d'autres problèmes de santé. il y a aussi le rasage de la tête le septième jour, la circoncision, donc tout ceci va jouer sur la santé de l'enfant. [00:24:34-7](http://localhost:2300/file=C:/Users/USER/Desktop/ENTRETIENS%20PREMATURITE/CMA-DO/FOCUS%20GROUPE%20CMA-DO.mp3time=1474700)

[00:24:34-7](http://localhost:2300/file=C:/Users/USER/Desktop/ENTRETIENS%20PREMATURITE/CMA-DO/FOCUS%20GROUPE%20CMA-DO.mp3time=1474700) Person 6: Même la femme enceinte, il y a certain endroit ou il est dit que la femme enceinte ne doit pas manger tel ou tel type de nourriture, ce sont des restrictions alimentaires qui entrainent un deficit en oligo-élements qui peuvent sur la mère et entrainer un accouchement prématuré. Il ya certain milieu, ou la femme ne doit pas manger telle sauce etant enceinte sinon l'enfant peut devenir un voleur. Donc on peut mettre toutes pratiques comme causes socio-économiques dans les accouchements prématurés. [00:25:17-5](http://localhost:2300/file=C:/Users/USER/Desktop/ENTRETIENS%20PREMATURITE/CMA-DO/FOCUS%20GROUPE%20CMA-DO.mp3time=1517500)

[00:25:17-5](http://localhost:2300/file=C:/Users/USER/Desktop/ENTRETIENS%20PREMATURITE/CMA-DO/FOCUS%20GROUPE%20CMA-DO.mp3time=1517500) Interviewer: Quelles sont les perceptions du nourrisson prématuré et de la maman dans la communauté? Est ce que ces perceptions peuvent impacter le nourrissons prématuré? [00:25:32-9](http://localhost:2300/file=C:/Users/USER/Desktop/ENTRETIENS%20PREMATURITE/CMA-DO/FOCUS%20GROUPE%20CMA-DO.mp3time=1532900)

[00:25:32-9](http://localhost:2300/file=C:/Users/USER/Desktop/ENTRETIENS%20PREMATURITE/CMA-DO/FOCUS%20GROUPE%20CMA-DO.mp3time=1532900) Person 4: Bien sûr parce que, beaucoup sont convaincus que le prématuré n'est pas viable, il n'est pas vue comme un enfant de mariage, donc on n'en prend pas soin, on le laisse partir (mourir). Puisqu'ils pensent que l'enfant porte malheur. [00:25:50-1](http://localhost:2300/file=C:/Users/USER/Desktop/ENTRETIENS%20PREMATURITE/CMA-DO/FOCUS%20GROUPE%20CMA-DO.mp3time=1550100)

[00:25:50-1](http://localhost:2300/file=C:/Users/USER/Desktop/ENTRETIENS%20PREMATURITE/CMA-DO/FOCUS%20GROUPE%20CMA-DO.mp3time=1550100) Person 6: Puisqu'il y a des sociétés, pour eux l'enfant de 7 mois peut vivre, mais l'enfant de 8 mois c'est pas un enfant qui peuvre dans la société (rire), pourtant les enfants c'est Dieu qui les nourri. [00:26:19-1](http://localhost:2300/file=C:/Users/USER/Desktop/ENTRETIENS%20PREMATURITE/CMA-DO/FOCUS%20GROUPE%20CMA-DO.mp3time=1579100)

[00:26:19-1](http://localhost:2300/file=C:/Users/USER/Desktop/ENTRETIENS%20PREMATURITE/CMA-DO/FOCUS%20GROUPE%20CMA-DO.mp3time=1579100) Interviewer: Maintenant, il arrive souvent dans le milieu de la santé qu'il y ait un mouvement des agents de santé pour cause d'affectation ou de retraite, quelles sont les propositions que vous avez à ce nveau pour ne perdre la qualité en terme de prestation de soins par rapport à la prématurité, surtout pour ceux qui ont réçu la formation? [00:26:53-5](http://localhost:2300/file=C:/Users/USER/Desktop/ENTRETIENS%20PREMATURITE/CMA-DO/FOCUS%20GROUPE%20CMA-DO.mp3time=1613500)

[00:26:53-5](http://localhost:2300/file=C:/Users/USER/Desktop/ENTRETIENS%20PREMATURITE/CMA-DO/FOCUS%20GROUPE%20CMA-DO.mp3time=1613500) Person 1: Les affectation vont forcement impacter sur la qualité des prestations de soins [00:27:00-8](http://localhost:2300/file=C:/Users/USER/Desktop/ENTRETIENS%20PREMATURITE/CMA-DO/FOCUS%20GROUPE%20CMA-DO.mp3time=1620800)

[00:27:00-8](http://localhost:2300/file=C:/Users/USER/Desktop/ENTRETIENS%20PREMATURITE/CMA-DO/FOCUS%20GROUPE%20CMA-DO.mp3time=1620800) Interviewer: Quelles solutions proposez vous? [00:27:03-4](http://localhost:2300/file=C:/Users/USER/Desktop/ENTRETIENS%20PREMATURITE/CMA-DO/FOCUS%20GROUPE%20CMA-DO.mp3time=1623400)

[00:27:03-4](http://localhost:2300/file=C:/Users/USER/Desktop/ENTRETIENS%20PREMATURITE/CMA-DO/FOCUS%20GROUPE%20CMA-DO.mp3time=1623400) Person 1: Peut être de permettre à ceux qui sont compétents de former pour qu'il y aient des repondants au cas ou ils sont amenés à partir. [00:27:15-7](http://localhost:2300/file=C:/Users/USER/Desktop/ENTRETIENS%20PREMATURITE/CMA-DO/FOCUS%20GROUPE%20CMA-DO.mp3time=1635700)

[00:27:15-7](http://localhost:2300/file=C:/Users/USER/Desktop/ENTRETIENS%20PREMATURITE/CMA-DO/FOCUS%20GROUPE%20CMA-DO.mp3time=1635700) Person 6: En fait dans les normes, tout le monde doit être formé à la prise en charge. Donc celui qui quitte Bobo pour aller à Ouaga, il a la qualifacation pour la prise en charge des prématurés. Maintenant s'il y a des gens qui ne sont pas formés, peut être dans chaque équipe, ceux qui sont formés peuvent apprendre à ceux qui ne sont pas formés. 4est dire que tout agent de santé, là ou il se trouve doit être qualifié pour prendre en charge les nouveaux nés prématurés. [00:27:53-8](http://localhost:2300/file=C:/Users/USER/Desktop/ENTRETIENS%20PREMATURITE/CMA-DO/FOCUS%20GROUPE%20CMA-DO.mp3time=1673800)

[00:22:28-1](http://localhost:2300/file=C:/Users/USER/Desktop/ENTRETIENS%20PREMATURITE/CMA-DO/FOCUS%20GROUPE%20CMA-DO.mp3time=1348100) Interviewer: Maintenant, qu'est ce que vous prospériez pour améliorer la prise en charge des naissances prématurées au sein de votre centre de santé ? [00:22:40-0](http://localhost:2300/file=C:/Users/USER/Desktop/ENTRETIENS%20PREMATURITE/CMA-DO/FOCUS%20GROUPE%20CMA-DO.mp3time=1360000)

[00:22:40-0](http://localhost:2300/file=C:/Users/USER/Desktop/ENTRETIENS%20PREMATURITE/CMA-DO/FOCUS%20GROUPE%20CMA-DO.mp3time=1360000) Person 3: Il faut des couveuses, il faut qu'il y ait un service dédié à la prématurité, une néonatologie avec les équipements et le personnel qu'il faut. [00:22:58-1](http://localhost:2300/file=C:/Users/USER/Desktop/ENTRETIENS%20PREMATURITE/CMA-DO/FOCUS%20GROUPE%20CMA-DO.mp3time=1378100)

[00:22:58-1](http://localhost:2300/file=C:/Users/USER/Desktop/ENTRETIENS%20PREMATURITE/CMA-DO/FOCUS%20GROUPE%20CMA-DO.mp3time=1378100) Interviewer: Est ce qu'il y a quelque chose d'autre à ajouter ? [00:23:03-0](http://localhost:2300/file=C:/Users/USER/Desktop/ENTRETIENS%20PREMATURITE/CMA-DO/FOCUS%20GROUPE%20CMA-DO.mp3time=1383000)

[00:23:03-0](http://localhost:2300/file=C:/Users/USER/Desktop/ENTRETIENS%20PREMATURITE/CMA-DO/FOCUS%20GROUPE%20CMA-DO.mp3time=1383000) Person 9: Renforcer notre matériel de réanimation, les lampes chauffantes, l'oxygénation, l'aspiration aussi, surtout les sondes naso-gastriques qui sont très rares. Souvent lorsqu'il n’y en a pas, il faut envoyer payer et c'est un peu cher. Si nous en avons ici, on doit pouvoir gérer. [00:23:34-9](http://localhost:2300/file=C:/Users/USER/Desktop/ENTRETIENS%20PREMATURITE/CMA-DO/FOCUS%20GROUPE%20CMA-DO.mp3time=1414900)

00:23:34-9 Interviewer: Est-ce que vous pensez que les pesanteurs socio-culturelles peuvent impacter la santé du nourrisson prématuré et de la mère et alourdir du même coup votre charge de travail concernant la prise en charge ? 00:23:44-8

00:23:44-8 Personne 1: Oui, oui (tous). 00:23:51-8

00:23:51-8 Personne 9: Ce sont les bains des bébés. Quand les bébés arrivent, il y a des spécialistes de bain de bébé, pourtant les bébés prématurés ont besoin d'être au chaud après la sortie. Il y a des vieilles qui trempent ces bébés-là dans l'eau, souvent c'est difficile de leur faire comprendre que ce n'est pas nécessaire, parce qu'ils sont convaincus que ce bébé, s'il n'est pas lavé, va sentir plus tard ou bien en tout cas va avoir peut-être d'autres problèmes de santé. Il y a aussi le rasage de la tête le septième jour, la circoncision, donc tout ceci va jouer sur la santé de l'enfant. 00:24:34-7

00:24:34-7 Personne 6: Même la femme enceinte, il y a certain endroit où il est dit que la femme enceinte ne doit pas manger tel ou tel type de nourriture, ce sont des restrictions alimentaires qui entraînent un déficit en oligo-éléments qui peuvent jouer sur la mère et entraîner un accouchement prématuré. Il y a certain milieu où la femme ne doit pas manger telle sauce étant enceinte, sinon l'enfant peut devenir un voleur. Donc on peut mettre toutes ces pratiques comme causes socio-économiques dans les accouchements prématurés. 00:25:17-5

00:25:17-5 Interviewer: Quelles sont les perceptions du nourrisson prématuré et de la maman dans la communauté ? Est-ce que ces perceptions peuvent impacter le nourrisson prématuré ? 00:25:32-9

00:25:32-9 Personne 4: Bien sûr, parce que beaucoup sont convaincus que le prématuré n'est pas viable, il n'est pas vu comme un enfant de mariage, donc on n'en prend pas soin, on le laisse partir (mourir). Puisqu'ils pensent que l'enfant porte malheur. 00:25:50-1

00:25:50-1 Personne 6: Puisqu'il y a des sociétés, pour eux, l'enfant de 7 mois peut vivre, mais l'enfant de 8 mois, ce n'est pas un enfant qui peut vivre dans la société (rire), pourtant les enfants, c'est Dieu qui les nourrit. 00:26:19-1

00:26:19-1 Interviewer: Maintenant, il arrive souvent dans le milieu de la santé qu'il y ait un mouvement des agents de santé pour cause d'affectation ou de retraite, quelles sont les propositions que vous avez à ce niveau pour ne pas perdre la qualité en termes de prestation de soins par rapport à la prématurité, surtout pour ceux qui ont reçu la formation ? 00:26:53-5

00:26:53-5 Person 1: Les affectations vont forcément impacter sur la qualité des prestations de soins. 00:27:00-8

00:27:00-8 Interviewer: Quelles solutions proposez-vous ? 00:27:03-4

00:27:03-4 Person 1: Peut-être de permettre à ceux qui sont compétents de former pour qu'il y ait des répondants au cas où ils sont amenés à partir. 00:27:15-7

00:27:15-7 Person 6: En fait, dans les normes, tout le monde doit être formé à la prise en charge. Donc celui qui quitte Bobo pour aller à Ouaga, il a la qualification pour la prise en charge des prématurés. Maintenant, s'il y a des gens qui ne sont pas formés, peut-être dans chaque équipe, ceux qui sont formés peuvent apprendre à ceux qui ne sont pas formés. C'est dire que tout agent de santé, là où il se trouve, doit être qualifié pour prendre en charge les nouveau-nés prématurés. 00:27:53-8

**00:22:28-1 Interviewer:** What measures are being taken to improve the management of premature births within your health center?

**00:22:40-0 Person 3:** We urgently need incubators and a dedicated department for prematurity, essentially a neonatology unit equipped with the necessary tools and staff.

**00:22:58-1 Interviewer:** Is there anything else you would like to add?

**00:23:03-0 Person 9:** It's crucial to strengthen our resuscitation equipment. We need heating lamps, oxygenation facilities, and improved suction capabilities. There's also a shortage of nasogastric tubes, which are essential but rare and expensive. Having these available at our center would significantly improve our ability to manage premature births.

**00:26:19-1 Interviewer:** In the health sector, staff movements due to assignments or retirements are common. What proposals do you have to maintain quality care for premature infants, especially when trained personnel leave?

**00:26:53-5 Person 1:** Staff assignments will inevitably affect the quality of care services.

**00:27:00-8 Interviewer:** What solutions do you suggest?

**00:27:03-4 Person 1:** One approach is to ensure that competent staff members are trained so that they can pass on their knowledge. This way, there are always trained responders available, even if some have to leave.

**00:27:15-7 Person 6:** According to our standards, all health workers should be trained in neonatal care. So, a person moving from one location to another, say from Bobo to Ouaga, should already be qualified to care for premature babies. If there are untrained individuals in a team, those who are trained should educate their untrained colleagues. The goal is for every health worker, regardless of their location, to be qualified in caring for premature newborns.

### Gynecologist 1

[00:13:33-9](http://localhost:2300/file=C:/Users/USER/Desktop/ENTRETIENS%20PREMATURITE/CMA-DO/GENYCOLOGUE%2001.mp3time=813900) Interviewer: Comment peut-on améliorer la prise en charge des naissance prématurés au sein de votre centre de santé? [00:13:41-1](http://localhost:2300/file=C:/Users/USER/Desktop/ENTRETIENS%20PREMATURITE/CMA-DO/GENYCOLOGUE%2001.mp3time=821100)

[00:13:41-1](http://localhost:2300/file=C:/Users/USER/Desktop/ENTRETIENS%20PREMATURITE/CMA-DO/GENYCOLOGUE%2001.mp3time=821100) Person 1: Pour améliorer la prise en charge des prématurés, je pense qu'il faut peut-être renforcer, si je prends nos structures ici, il faut renforcer les capacités de prise en charge, qui est clairement de creer une unité de néonatologie avec un pédiatre qui pourra les suivre.Voilà, comme ça ces nouveaux-nés qu'on a, ils pourront rester ici. Parce que l'autre difficulté, à Bobo on a une seule unité de néonatologie qui est au CHU, qui est à tout moment surchargée. Et le fait qu'elle soit surchargée, les enfants sont deux deux ou trois trois et il y a risque d'infection, et je pense que ça, ça plombe encore plus la mortalité. Donc pour vraiment pouvoir améliorer, d'abord au niveau des deux CMA, il faut qu'on puisse créer des unités de néonatologie qui vont prendre en charge certains prématurés, avec des pédiatres qui pourront prendre en charge même les infections néonatales,certaines détresses, et ça va désengorger l'hôpital, et ça va vraiment donner plus de chance à ces enfants-là. Il faut vraiment renforcer les capacités des CMA, les capacités en infrastructures, en personnel et en compétences. [00:14:49-8](http://localhost:2300/file=C:/Users/USER/Desktop/ENTRETIENS%20PREMATURITE/CMA-DO/GENYCOLOGUE%2001.mp3time=889800)

[00:14:49-8](http://localhost:2300/file=C:/Users/USER/Desktop/ENTRETIENS%20PREMATURITE/CMA-DO/GENYCOLOGUE%2001.mp3time=889800) Interviewer: Nous avons souvent remarqué qu'il y a un mouvement au niveau des agents de santé par rapport aux affections. Est-ce que vous pensez que si ceux qui ont reçu une formation par rapport à la prématuritée partent ailleurs, cela peut impacter la qualité des prestations de soins par rapport à la prématurité? [00:15:10-6](http://localhost:2300/file=C:/Users/USER/Desktop/ENTRETIENS%20PREMATURITE/CMA-DO/GENYCOLOGUE%2001.mp3time=910600)

[00:15:10-6](http://localhost:2300/file=C:/Users/USER/Desktop/ENTRETIENS%20PREMATURITE/CMA-DO/GENYCOLOGUE%2001.mp3time=910600) Person 1: Oui, ça peut impacter. [00:15:13-2](http://localhost:2300/file=C:/Users/USER/Desktop/ENTRETIENS%20PREMATURITE/CMA-DO/GENYCOLOGUE%2001.mp3time=913200)

[00:15:13-2](http://localhost:2300/file=C:/Users/USER/Desktop/ENTRETIENS%20PREMATURITE/CMA-DO/GENYCOLOGUE%2001.mp3time=913200) Interviewer: Quelles solutions proposerez vous? [00:15:16-6](http://localhost:2300/file=C:/Users/USER/Desktop/ENTRETIENS%20PREMATURITE/CMA-DO/GENYCOLOGUE%2001.mp3time=916600)

[00:15:16-6](http://localhost:2300/file=C:/Users/USER/Desktop/ENTRETIENS%20PREMATURITE/CMA-DO/GENYCOLOGUE%2001.mp3time=916600) Person 1: En fait, vous voyez, si par exemple il y a une unité de néonatologie, on n'a pas besoin d'une longue formation spécifique pour la prise en charge de ces nouveaux-nés.Si quelqu'un par exemple est affecté, je me dis qu'en un ou deux mois, la personne doit pouvoir être opérationnelle. Ce qui peut lui poser des problèmes, c'est la prise des voies veineux chez le prématuré, qui est plus difficile que chez l'enfant ou l'adulte. Donc je pense que même s'il y a des mouvements, si dans un service,il ne faut pas qu'on décapite tout le service, même s'il y a des mouvements, il y a la mémoire qui reste, et cette mémoire peut servir à reformer les autres, et ainsi de suite on avance. [00:16:01-2](http://localhost:2300/file=C:/Users/USER/Desktop/ENTRETIENS%20PREMATURITE/CMA-DO/GENYCOLOGUE%2001.mp3time=961200)

[00:16:01-2](http://localhost:2300/file=C:/Users/USER/Desktop/ENTRETIENS%20PREMATURITE/CMA-DO/GENYCOLOGUE%2001.mp3time=961200) Interviewer: Est-ce que vous pensez que les pésenteurs socio-culturels peuvent aussi impacter sur la santé de la mère? [00:16:08-2](http://localhost:2300/file=C:/Users/USER/Desktop/ENTRETIENS%20PREMATURITE/CMA-DO/GENYCOLOGUE%2001.mp3time=968200)

[00:16:08-2](http://localhost:2300/file=C:/Users/USER/Desktop/ENTRETIENS%20PREMATURITE/CMA-DO/GENYCOLOGUE%2001.mp3time=968200) Person 1: Oui, sur la santé de la mère de l'enfant prématuré, oui. [00:16:15-7](http://localhost:2300/file=C:/Users/USER/Desktop/ENTRETIENS%20PREMATURITE/CMA-DO/GENYCOLOGUE%2001.mp3time=975700)

[00:16:15-7](http://localhost:2300/file=C:/Users/USER/Desktop/ENTRETIENS%20PREMATURITE/CMA-DO/GENYCOLOGUE%2001.mp3time=975700) Interviewer: Comment? [00:16:17-8](http://localhost:2300/file=C:/Users/USER/Desktop/ENTRETIENS%20PREMATURITE/CMA-DO/GENYCOLOGUE%2001.mp3time=977800)

[00:16:17-8](http://localhost:2300/file=C:/Users/USER/Desktop/ENTRETIENS%20PREMATURITE/CMA-DO/GENYCOLOGUE%2001.mp3time=977800) Person 1: Il y a un cas par exemple, on dit qu'il n'a même pas encore de nom,donc on ne va pas se sacrifier trop pour lui. Ça veut dire quoi? C'est un enfant qui n'a même pas encore reçu le baptême, il n'a pas de nom, on ne voit pas pourquoi on va investir pour sa santé. S'il doit partir, il n'a qu'à partir. C'est un exemple qu'on a vécu il n'y a pas longtemps. Donc ça fait qu'on n'a pas le même enthousiasme pour s'occuper des prématurés que pour s'occuper des autres. Et le fait que le prématuré, sa prise en charge, ça nécessite plus de temps, ça nécessite plus de patience. Alors que la maman doit s'occuper des autres frères, la maman doit s'occuper des autres choses, ça devient difficile. [00:17:00-8](http://localhost:2300/file=C:/Users/USER/Desktop/ENTRETIENS%20PREMATURITE/CMA-DO/GENYCOLOGUE%2001.mp3time=1020800)

[00:17:00-8](http://localhost:2300/file=C:/Users/USER/Desktop/ENTRETIENS%20PREMATURITE/CMA-DO/GENYCOLOGUE%2001.mp3time=1020800) Interviewer: Lorsque ces genres de situations vous arrivent, comment est-ce que, au niveau de votre centre de santé, elles sont gérés? [00:17:07-1](http://localhost:2300/file=C:/Users/USER/Desktop/ENTRETIENS%20PREMATURITE/CMA-DO/GENYCOLOGUE%2001.mp3time=1027100)

[00:17:07-1](http://localhost:2300/file=C:/Users/USER/Desktop/ENTRETIENS%20PREMATURITE/CMA-DO/GENYCOLOGUE%2001.mp3time=1027100) Person 1: On essaie de discuter avec les parents, leur expliquer le bienfait, leur expliquer les soins qu'on va faire.C'est vrai que ça va prendre du temps, mais c'est des soins qui sont en ambulatoire. Et même ces prématurés, avec le système mère kangourou, ils peuvent rester avec leur mère pendant que la mère mène des petites activités. Donc on essaie de les sensibiliser comme ça pour qu'ils acceptent.Certains acceptent, mais d'autres sont perdus de vue, ça veut dire qu'ils n'acceptent pas. [00:17:39-2](http://localhost:2300/file=C:/Users/USER/Desktop/ENTRETIENS%20PREMATURITE/CMA-DO/GENYCOLOGUE%2001.mp3time=1059200)

00:13:33-9 Interviewer: Comment peut-on améliorer la prise en charge des naissances prématurées au sein de votre centre de santé ? 00:13:41-1

00:13:41-1 Person 1: Pour améliorer la prise en charge des prématurés, je pense qu'il faut peut-être renforcer, si je prends nos structures ici, il faut renforcer les capacités de prise en charge, qui est clairement de créer une unité de néonatologie avec un pédiatre qui pourra les suivre. Voilà, comme ça ces nouveau-nés qu'on a, ils pourront rester ici. Parce que l'autre difficulté, à Bobo on a une seule unité de néonatologie qui est au CHU, qui est à tout moment surchargée. Et le fait qu'elle soit surchargée, les enfants sont deux, deux ou trois, trois et il y a risque d'infection, et je pense que ça, ça plombe encore plus la mortalité. Donc pour vraiment pouvoir améliorer, d'abord au niveau des deux CMA, il faut qu'on puisse créer des unités de néonatologie qui vont prendre en charge certains prématurés, avec des pédiatres qui pourront prendre en charge même les infections néonatales, certaines détresses, et ça va désengorger l'hôpital, et ça va vraiment donner plus de chance à ces enfants-là. Il faut vraiment renforcer les capacités des CMA, les capacités en infrastructures, en personnel et en compétences. 00:14:49-8

00:14:49-8 Interviewer: Nous avons souvent remarqué qu'il y a un mouvement au niveau des agents de santé par rapport aux affectations. Est-ce que vous pensez que si ceux qui ont reçu une formation par rapport à la prématurité partent ailleurs, cela peut impacter la qualité des prestations de soins par rapport à la prématurité ? 00:15:10-6

00:15:10-6 Person 1: Oui, ça peut impacter. 00:15:13-2

00:15:13-2 Interviewer: Quelles solutions proposeriez-vous ? 00:15:16-6

00:15:16-6 Person 1: En fait, vous voyez, si par exemple il y a une unité de néonatologie, on n'a pas besoin d'une longue formation spécifique pour la prise en charge de ces nouveau-nés. Si quelqu'un par exemple est affecté, je me dis qu'en un ou deux mois, la personne doit pouvoir être opérationnelle. Ce qui peut lui poser des problèmes, c'est la prise des voies veineux chez le prématuré, qui est plus difficile que chez l'enfant ou l'adulte. Donc je pense que même s'il y a des mouvements, si dans un service, il ne faut pas qu'on décapite tout le service, même s'il y a des mouvements, il y a la mémoire qui reste, et cette mémoire peut servir à reformer les autres, et ainsi de suite on avance. 00:16:01-2

00:16:01-2 Interviewer: Est-ce que vous pensez que les pesanteurs socio-culturelles peuvent aussi impacter sur la santé de la mère ? 00:16:08-2

00:16:08-2 Personne 1: Oui, sur la santé de la mère de l'enfant prématuré, oui. 00:16:15-7

00:16:15-7 Interviewer: Comment ? 00:16:17-8

00:16:17-8 Personne 1: Il y a un cas, par exemple, on dit qu'il n'a même pas encore de nom, donc on ne va pas se sacrifier trop pour lui. Ça veut dire quoi ? C'est un enfant qui n'a même pas encore reçu le baptême, il n'a pas de nom, on ne voit pas pourquoi on va investir pour sa santé. S'il doit partir, il n'a qu'à partir. C'est un exemple qu'on a vécu il n'y a pas longtemps. Donc ça fait qu'on n'a pas le même enthousiasme pour s'occuper des prématurés que pour s'occuper des autres. Et le fait que le prématuré, sa prise en charge, ça nécessite plus de temps, ça nécessite plus de patience. Alors que la maman doit s'occuper des autres frères, la maman doit s'occuper des autres choses, ça devient difficile. 00:17:00-8

00:17:00-8 Interviewer: Lorsque ces genres de situations vous arrivent, comment est-ce que, au niveau de votre centre de santé, elles sont gérées ? 00:17:07-1

00:17:07-1 Personne 1: On essaie de discuter avec les parents, leur expliquer le bienfait, leur expliquer les soins qu'on va faire. C'est vrai que ça va prendre du temps, mais ce sont des soins qui sont en ambulatoire. Et même ces prématurés, avec le système mère kangourou, ils peuvent rester avec leur mère pendant que la mère mène des petites activités. Donc on essaie de les sensibiliser comme ça pour qu'ils acceptent. Certains acceptent, mais d'autres sont perdus de vue, ça veut dire qu'ils n'acceptent pas. 00:17:39-2

**00:13:33-9 Interviewer:** How can the management of premature births be improved within your health center?

**00:13:41-1 Person 1:** To enhance the care of premature babies, we need to strengthen our capabilities. Specifically, we should create a neonatology unit staffed with pediatricians to provide dedicated care. This is crucial because the only neonatology unit in Bobo, at the University Hospital, is constantly overloaded. The overcrowding increases the risk of infection and contributes to higher mortality rates. Creating neonatology units at the two CMAs would not only relieve congestion at the hospital but also provide these children with better care opportunities. We need to bolster our infrastructure, personnel, and skills at the CMAs.

**00:14:49-8 Interviewer:** We’ve noticed staff movement due to assignments. Do you think this could impact the quality of care for premature births?

**00:15:10-6 Person 1:** Yes, it can have an impact.

**00:15:13-2 Interviewer:** What solutions would you propose?

**00:15:16-6 Person 1:** If we have a neonatology unit, specific training for newborn care isn't necessarily lengthy. A staff member can become operational in about one or two months. Challenges like venous line placement in premature babies, which is more difficult than in older children or adults, can be managed. Even with staff movements, the institutional memory and expertise remain. This knowledge can be passed on to train new staff, ensuring continuity and progress in care.

**Interviewer (00:16:01-2):** "Do you think socio-cultural constraints can impact the health of a mother, especially in the context of premature births?"

**Person 1 (00:16:08-2):** "Yes, they can significantly affect the health of the mother of a premature child."

**Interviewer (00:16:15-7):** "In what ways?"

**Person 1 (00:16:17-8):** "For instance, there's a cultural notion that if a baby doesn't yet have a name or hasn't been baptized, there's less inclination to invest in their health. The attitude is like, 'If the child is meant to pass away, let it be.' We encountered a case like this not long ago. This leads to less enthusiasm for caring for premature babies compared to others. Moreover, caring for a premature baby demands more time and patience, which can be challenging for a mother also responsible for other children and household duties."

**Interviewer (00:17:00-8):** "How do you manage such situations at your health center?"

**Person 1 (00:17:07-1):** "We engage in discussions with the parents, explaining the benefits of the care we provide and the process involved. We acknowledge that it takes time but emphasize that it's outpatient care. With the kangaroo mother care system, premature babies can stay close to their mothers, allowing the mothers to continue with minor activities. We strive to raise awareness and encourage acceptance of this approach. Some parents are receptive, but others, unfortunately, are lost to follow-up, indicating their non-acceptance

### Gynecologist 2

[00:25:55-0](http://localhost:2300/file=C:/Users/USER/Desktop/ENTRETIENS%20PREMATURITE/CMA-DO/GENYCOLOGUE%2002.mp3time=1555000) Interviewer: Maintenant, au-delà de tout ce que vous venez d'évoquer, qu'est-ce que vous avez comme proposition pour améliorer la prise en charge des naissances prématurés au sein de votre centre de santé ici ? [00:26:06-3](http://localhost:2300/file=C:/Users/USER/Desktop/ENTRETIENS%20PREMATURITE/CMA-DO/GENYCOLOGUE%2002.mp3time=1566300)

[00:26:06-3](http://localhost:2300/file=C:/Users/USER/Desktop/ENTRETIENS%20PREMATURITE/CMA-DO/GENYCOLOGUE%2002.mp3time=1566300) Person 1: Alors ici, comme je le disais au début, beaucoup de sages femmes ont été formés. Formés, ce n'est pas une formation formelle, mais il y en a qui ont eu un temps de passage à l'Unité Kangourou. Donc, ils ont quelques aptitudes pour pouvoir prendre en charge les prématurés. Et cette unité ne fonctionne pas à 100%. Donc, il n'y a pas suffisamment de personnel pour faire fonctionner l'unité 24h sur 24. Donc, souvent, c'est dans la journée ou bien dans l'après-midi. Mais la nuit, on n'a pas quelqu'un qui fait fonctionner l'unité. Et ensuite, il y a le problème du matériel. Donc, ça veut dire qu'on a un problème de personnel formé. Donc, on devrait avoir des agents formés qui font fonctionner l'unité de Kangourou de façon permanente 24h sur 24. Et le mieux même, c'est une unité de pédiatrie. C'est-à-dire un bâtiment avec un pédiatre et tout son personnel. Parce qu'on a environ 6.000 naissances dans l'année. Le pourcentage, comme je le disais, on n'a pas calculé pour savoir ça fait combien. Mais il y a assez de prématurés. Et si on a une unité de pédiatrie avec une néonatologie, ça améliore la prise en charge. donc il y aura certainement des couveuses pour les grands prématurés. Au lieu les envoyer à l'hôpital, ils seront prise en charge ici avec le pédiatre. Et il y a un personnel qui fait fonctionner l'unité de pédiatrie 24h sur 24. Ça veut dire que tout de suite, s'il y a une naissance prématurée, on ne va pas dire par exemple que l'unité Kangourou est fermée, on va envoyer l'enfant à l'hôpital. On va le transférer tout simplement en pédiatrie. On a besoin de matériel, on a besoin de bâtiments, on a besoin d'agents formés. [00:27:42-3](http://localhost:2300/file=C:/Users/USER/Desktop/ENTRETIENS%20PREMATURITE/CMA-DO/GENYCOLOGUE%2002.mp3time=1662300)

[00:27:42-3](http://localhost:2300/file=C:/Users/USER/Desktop/ENTRETIENS%20PREMATURITE/CMA-DO/GENYCOLOGUE%2002.mp3time=1662300) Interviewer: Maintenant, quand vous parlez de formation, une formation spécifique à la prise en charge de naissance prématurée ? [00:27:49-0](http://localhost:2300/file=C:/Users/USER/Desktop/ENTRETIENS%20PREMATURITE/CMA-DO/GENYCOLOGUE%2002.mp3time=1669000)

[00:27:49-0](http://localhost:2300/file=C:/Users/USER/Desktop/ENTRETIENS%20PREMATURITE/CMA-DO/GENYCOLOGUE%2002.mp3time=1669000) Person 1: Si on a une unité de pédiatrie, ça veut dire que... Ici, le défaut, c'est que ce sont les sages-femmes qui font les accouchements et qui prennent en charge, qui font les premiers soins du prématuré. Donc là, ils sont formés à ça. Maintenant, s'il s'agit de faire des soins approfondis, il faut carrément aller à l'unité Kangourou. Et comme elles sont à la fois en train de faire les accouchements, il y a une certaine mesure qu'elles ne pourront pas faire. Là, ça ramène à avoir une unité qui fonctionne à tout moment. S'il y a un pédiatre, il va former son personnel par rapport à la prise en charge des prématurés et des autres enfants. À la longue, c'est les gens qui auront beaucoup plus de compétences par rapport à ça. [00:28:34-9](http://localhost:2300/file=C:/Users/USER/Desktop/ENTRETIENS%20PREMATURITE/CMA-DO/GENYCOLOGUE%2002.mp3time=1714900)

[00:28:34-9](http://localhost:2300/file=C:/Users/USER/Desktop/ENTRETIENS%20PREMATURITE/CMA-DO/GENYCOLOGUE%2002.mp3time=1714900) Interviewer: Est-ce que vous pensez que les présentations socioculturelles peuvent impacter la prise en charge des nourrissons prématurés et la mère ? [00:28:46-4](http://localhost:2300/file=C:/Users/USER/Desktop/ENTRETIENS%20PREMATURITE/CMA-DO/GENYCOLOGUE%2002.mp3time=1726400)

[00:28:46-4](http://localhost:2300/file=C:/Users/USER/Desktop/ENTRETIENS%20PREMATURITE/CMA-DO/GENYCOLOGUE%2002.mp3time=1726400) Person 1: Oui. Par exemple, une femme enceinte qui n'est pas bien nourrie, qui n'a pas suffisamment de moyens pour s'occuper de son hygiène alimentaire, vestimentaire, corporelle et autres, peut donner naissance à un enfant prématuré. Et si l'enfant naît dans un contexte prématuré où le milieu est défavorable, il y a moins de chances de pouvoir le récupérer. [00:29:10-5](http://localhost:2300/file=C:/Users/USER/Desktop/ENTRETIENS%20PREMATURITE/CMA-DO/GENYCOLOGUE%2002.mp3time=1750500)

[00:29:10-5](http://localhost:2300/file=C:/Users/USER/Desktop/ENTRETIENS%20PREMATURITE/CMA-DO/GENYCOLOGUE%2002.mp3time=1750500) Interviewer: Est-ce que dans la communauté, la conception peut aussi influer ? [00:29:16-1](http://localhost:2300/file=C:/Users/USER/Desktop/ENTRETIENS%20PREMATURITE/CMA-DO/GENYCOLOGUE%2002.mp3time=1756100)

[00:29:16-1](http://localhost:2300/file=C:/Users/USER/Desktop/ENTRETIENS%20PREMATURITE/CMA-DO/GENYCOLOGUE%2002.mp3time=1756100) Person 1: Oui, mais je pense que les choses sont en train de changer parce qu'il y a un certain nombre d'interdits ou bien de pratiques dans la communauté qui peuvent être à l'origine des cas de prématurés. Par exemple, on prend le cas des mariages précoces, les mariages d'enfants. Si le mariage est très précoce et que l'enfant est une enfant qui est enceinte, qui n'a pas assez d'expérience pour suivre sa grossesse ou souvent même qui essaie de cacher la grossesse, souvent on aboutit à une naissance prématurée. Et s'il n'y a pas quelqu'un à côté pour l'aider à s'occuper de l'enfant, la prise en charge est vraiment difficile. Il y a les cas de mariages précoces et même les cas de grossesses hors mariage. Les grossesses en milieu scolaire, par exemple, souvent c'est des enfants et des adolescents qui tombent enceintes qui veulent même masquer la grossesse. Donc on peut aboutir à une naissance prématurée parce que la grossesse n'a pas été accompagnée de façon suffisante. Là on aboutit à une naissance prématurée et si déjà à l'intérieur de la cellule familiale l'enfant qui a accouché n'est pas supporté par la famille parce que souvent on considère ça comme un déshonneur, comme une honte pour la famille et l'enfant est rejeté avec son enfant. Et là ça complique vraiment la prise en charge. Et c'est la même chose dans le cas de mariages précoces. Si dans la famille la belle-mère ou les tantes ou les autres parents du monsieur n'accompagnent pas cette jeune dame, la prise en charge va être encore difficile. [00:31:02-6](http://localhost:2300/file=C:/Users/USER/Desktop/ENTRETIENS%20PREMATURITE/CMA-DO/GENYCOLOGUE%2002.mp3time=1862600)

00:25:55-0 Interviewer: Maintenant, au-delà de tout ce que vous venez d'évoquer, qu'est-ce que vous avez comme proposition pour améliorer la prise en charge des naissances prématurées au sein de votre centre de santé ici ? 00:26:06-3

00:26:06-3 Person 1: Alors ici, comme je le disais au début, beaucoup de sage-femmes ont été formées. Formées, ce n'est pas une formation formelle, mais il y en a qui ont eu un temps de passage à l'Unité Kangourou. Donc, ils ont quelques aptitudes pour pouvoir prendre en charge les prématurés. Et cette unité ne fonctionne pas à 100%. Donc, il n'y a pas suffisamment de personnel pour faire fonctionner l'unité 24h sur 24. Donc, souvent, c'est dans la journée ou bien dans l'après-midi. Mais la nuit, on n'a pas quelqu'un qui fait fonctionner l'unité. Et ensuite, il y a le problème du matériel. Donc, ça veut dire qu'on a un problème de personnel formé. Donc, on devrait avoir des agents formés qui font fonctionner l'unité de Kangourou de façon permanente 24h sur 24. Et le mieux même, c'est une unité de pédiatrie. C'est-à-dire un bâtiment avec un pédiatre et tout son personnel. Parce qu'on a environ 6.000 naissances dans l'année. Le pourcentage, comme je le disais, on n'a pas calculé pour savoir ça fait combien. Mais il y a assez de prématurés. Et si on a une unité de pédiatrie avec une néonatologie, ça améliore la prise en charge. Donc il y aura certainement des couveuses pour les grands prématurés. Au lieu de les envoyer à l'hôpital, ils seront pris en charge ici avec le pédiatre. Et il y a un personnel qui fait fonctionner l'unité de pédiatrie 24h sur 24. Ça veut dire que tout de suite, s'il y a une naissance prématurée, on ne va pas dire par exemple que l'unité Kangourou est fermée, on va envoyer l'enfant à l'hôpital. On va le transférer tout simplement en pédiatrie. On a besoin de matériel, on a besoin de bâtiments, on a besoin d'agents formés. 00:27:42-3

00:27:42-3 Interviewer: Maintenant, quand vous parlez de formation, une formation spécifique à la prise en charge de naissances prématurées ? 00:27:49-0

00:27:49-0 Person 1: Si on a une unité de pédiatrie, ça veut dire que... Ici, le défaut, c'est que ce sont les sage-femmes qui font les accouchements et qui prennent en charge, qui font les premiers soins du prématuré. Donc là, ils sont formés à ça. Maintenant, s'il s'agit de faire des soins approfondis, il faut carrément aller à l'unité Kangourou. Et comme elles sont à la fois en train de faire les accouchements, il y a une certaine mesure qu'elles ne pourront pas faire. Là, ça ramène à avoir une unité qui fonctionne à tout moment. S'il y a un pédiatre, il va former son personnel par rapport à la prise en charge des prématurés et des autres enfants. À la longue, c'est les gens qui auront beaucoup plus de compétences par rapport à ça. 00:28:34-9

00:28:34-9 Interviewer: Est-ce que vous pensez que les pesanteurs socioculturelles peuvent impacter la prise en charge des nourrissons prématurés et de la mère ? 00:28:46-4

00:28:46-4 Personne 1: Oui. Par exemple, une femme enceinte qui n'est pas bien nourrie, qui n'a pas suffisamment de moyens pour s'occuper de son hygiène alimentaire, vestimentaire, corporelle et autres, peut donner naissance à un enfant prématuré. Et si l'enfant naît dans un contexte prématuré où le milieu est défavorable, il y a moins de chances de pouvoir le récupérer. 00:29:10-5

00:29:10-5 Interviewer: Est-ce que dans la communauté, la conception peut aussi influer ? 00:29:16-1

00:29:16-1 Personne 1: Oui, mais je pense que les choses sont en train de changer parce qu'il y a un certain nombre d'interdits ou bien de pratiques dans la communauté qui peuvent être à l'origine des cas de prématurés. Par exemple, on prend le cas des mariages précoces, les mariages d'enfants. Si le mariage est très précoce et que l'enfant est une enfant qui est enceinte, qui n'a pas assez d'expérience pour suivre sa grossesse ou souvent même qui essaie de cacher la grossesse, souvent on aboutit à une naissance prématurée. Et s'il n'y a pas quelqu'un à côté pour l'aider à s'occuper de l'enfant, la prise en charge est vraiment difficile. Il y a les cas de mariages précoces et même les cas de grossesses hors mariage. Les grossesses en milieu scolaire, par exemple, souvent ce sont des enfants et des adolescents qui tombent enceintes qui veulent même masquer la grossesse. Donc on peut aboutir à une naissance prématurée parce que la grossesse n'a pas été accompagnée de façon suffisante. Là, on aboutit à une naissance prématurée et si déjà à l'intérieur de la cellule familiale, l'enfant qui a accouché n'est pas supporté par la famille parce que souvent on considère ça comme un déshonneur, comme une honte pour la famille, et l'enfant est rejeté avec son enfant. Et là, ça complique vraiment la prise en charge. Et c'est la même chose dans le cas de mariages précoces. Si dans la famille la belle-mère ou les tantes ou les autres parents du monsieur n'accompagnent pas cette jeune dame, la prise en charge va être encore difficile. 00:31:02-6

**00:25:55-0 Interviewer:** Beyond what you've already mentioned, do you have any proposals for improving the management of premature births in your health center?

**00:26:06-3 Person 1:** As I mentioned earlier, many of our midwives have been trained, although not formally. Some have spent time in the Kangaroo Unit and have acquired skills to care for premature babies. However, this unit isn't operating at full capacity; we don't have enough staff to run it 24/7. It's mostly operational during the day or afternoon, but not at night. Additionally, we're facing equipment shortages. We need trained personnel operating the Kangaroo unit around the clock. Ideally, we should establish a full pediatric unit with a pediatrician and dedicated staff. We have around 6,000 births per year, and a significant number of these are premature. Having a neonatology unit within the pediatric facility would greatly improve care, including the provision of incubators for very premature babies. Instead of sending them to the hospital, they could be cared for here. This requires additional equipment, dedicated buildings, and trained staff.

**00:27:42-3 Interviewer:** When you talk about training, are you referring to specific training in managing premature births?

**00:27:49-0 Person 1:** With a pediatric unit, the dynamic changes. Currently, midwives handle deliveries and provide initial care to premature babies, but for in-depth care, they need to be transferred to the Kangaroo unit. The midwives are limited in how much they can do while also delivering babies. Having a unit that operates full-time with a pediatrician would mean comprehensive training for staff in caring for premature babies and other children, leading to more skilled personnel in this area.

**Interviewer (00:28:34-9):** "Do you think that socio-cultural factors can impact the care of premature infants and their mothers?"

**Person 1 (00:28:46-4):** "Yes, definitely. For instance, a pregnant woman who is undernourished or lacks the means for proper food, clothing, and hygiene can be at risk of giving birth prematurely. Furthermore, if the child is born into an unfavorable environment, the chances of successfully nurturing the child are reduced."

**Interviewer (00:29:10-5):** "Does the societal framework also influence these cases?"

**Person 1 (00:29:16-1):** "Certainly, but I think there's a shift happening. Community practices and prohibitions can contribute to premature births. Early and child marriages are prime examples. A very young bride, lacking experience in managing her pregnancy or even trying to conceal it, often results in premature birth. If there's no support system to assist with the baby, care becomes exceedingly difficult. Early marriages and pregnancies out of wedlock, like those occurring in schools, frequently involve adolescents who may attempt to hide their pregnancies. This lack of support often leads to premature births. Moreover, if the young mother is not supported by her family, viewed as bringing dishonor or shame, both she and her child are left in a vulnerable position. This lack of support makes care extremely challenging, a scenario that's also common in early marriages. Without the support of the husband's family, such as the mother-in-law or aunts, care for the young mother becomes difficult.

### Chief physician of Do health district

00 : 27 :04-2 Interviewer: Au vu de tout ce que vous avez dit, qu'est-ce que vous avez comme proposition pour améliorer les prises en charge des naissances prématurées au sein même du CMA de DÔ? [00:27:17-8](http://localhost:2300/file=C:/Users/USER/Desktop/ENTRETIENS%20PREMATURITE/CMA-DO/MCD-DO.mp3time=1637800)

[00:27:17-8](http://localhost:2300/file=C:/Users/USER/Desktop/ENTRETIENS%20PREMATURITE/CMA-DO/MCD-DO.mp3time=1637800) Person 1: Bon. Comme on est... Ça commence par un meilleur suivi de la grossesse. En ce sens que nous, nous demandons toujours aux dames de venir un peu plus tôt, au premier trimestre, pour qu'on puisse leur donner les meilleurs conseils pour la grossesse. Parce que la grossesse, ce n'est pas une maladie. C'est un état. Mais bon, ce n'est pas une maladie. Donc, nous, nous donnons les conseils pour pouvoir bien assumer et achever la grossesse. Pour ce faire il y a un certain nombre de visites avec une périodicité que nous donnons à la maman. En plus de ça, nous lui donnons des signes de danger. Ces signes-là peuvent dire qu'on tend vers un accouchement prématuré. Tu t'imagines quelqu'un qui a une douleur abdominale atroce et une grossesse qui n'est pas à terme, il faut venir à temps. Ou bien, quelqu'un qui est entrain de saigner sur une grossesse, ça peut être des signes, quelqu'un qui a des céphalées atroces, des maux de tête atroces. Ce sont des signes de danger que nous donnons à la maman, et en même temps, lui donner l'alimentation qui va avec la grossesse en fonction de l'avancée de la grossesse. Tout ça pour que la maman puisse avoir une belle expérience de la grossesse. Ce qui va conduire à une facilité de l'accouchement. Parce qu'elle sera mieux préparée pour ça. Maintenant, pour l'enfant primatisé, même il y a le suivi de la grossesse, il y a la phase où le primaturé est dehors. Il est avec nous. Comment il faut le gérer ? Là, il faut vraiment un plateau qui répond. C'est ce que je disais, par le plateau, nous on n'a pas de couveuse. On n'a pas d'unité de pédiatrie à plus forte raison de néonatologie. Donc on a une unité de SMK, soins maternels kangourou. Vous savez que c'est l'hypothermie qui tue beaucoup plus. En dehors même de l'infection, l'hypothermie les tue plus. Les malnutris. C'est pour ça que je parlais de la lingerie, c'était pour les couvrir. donc, SMK, on montre comment il faut faire. Le ventre à ventre, la maman et l'enfant, il faut couvrir pour que la chaleur maternelle puisse aider. Le kangourou, c'est comme l'animal kangourou qui porte son enfant. C'est un peu ça pour accompagner. Mais bon, si on ne peut pas gérer manifestement, qu'on l'évacue. On envoie ça à l'hôpital parce qu'ils ont un dispositif encore améliorer. [00:30:11-4](http://localhost:2300/file=C:/Users/USER/Desktop/ENTRETIENS%20PREMATURITE/CMA-DO/MCD-DO.mp3time=1811400)

[00:30:11-4](http://localhost:2300/file=C:/Users/USER/Desktop/ENTRETIENS%20PREMATURITE/CMA-DO/MCD-DO.mp3time=1811400) Interviewer: Est-ce que vous pensez que les présenteurs socio-culturels peuvent impacter la prise en charge de la maman et même du nourrisson prématuré? [00:30:22-2](http://localhost:2300/file=C:/Users/USER/Desktop/ENTRETIENS%20PREMATURITE/CMA-DO/MCD-DO.mp3time=1822200)

[00:30:22-2](http://localhost:2300/file=C:/Users/USER/Desktop/ENTRETIENS%20PREMATURITE/CMA-DO/MCD-DO.mp3time=1822200) Person 1: Quels présenteurs? Je ne sais pas s'il y a des présenteurs pour la prématurité. Je ne sais pas dire grand chose. Je ne sais pas bien. Je ne sais pas si il y a des appréhensions avec la prématurité. La grossesse, il y a des fausses couches. On dit que la grossesse n'est pas arrivée en maturité. Ça, c'est connu. L'avortement aussi, la société connaît ça. Je ne sarai pas dire grand chose. [00:31:03-2](http://localhost:2300/file=C:/Users/USER/Desktop/ENTRETIENS%20PREMATURITE/CMA-DO/MCD-DO.mp3time=1863200)

00:27:04-2 Interviewer: Au vu de tout ce que vous avez dit, qu'est-ce que vous avez comme proposition pour améliorer les prises en charge des naissances prématurées au sein même du CMA de Dô ? 00:27:17-8

00:27:17-8 Personne 1: Bon. Comme on est... Ça commence par un meilleur suivi de la grossesse. En ce sens que nous, nous demandons toujours aux dames de venir un peu plus tôt, au premier trimestre, pour qu'on puisse leur donner les meilleurs conseils pour la grossesse. Parce que la grossesse, ce n'est pas une maladie. C'est un état. Mais bon, ce n'est pas une maladie. Donc, nous, nous donnons les conseils pour pouvoir bien assumer et achever la grossesse. Pour ce faire, il y a un certain nombre de visites avec une périodicité que nous donnons à la maman. En plus de ça, nous lui donnons des signes de danger. Ces signes-là peuvent dire qu'on tend vers un accouchement prématuré. Tu t'imagines quelqu'un qui a une douleur abdominale atroce et une grossesse qui n'est pas à terme, il faut venir à temps. Ou bien, quelqu'un qui est en train de saigner sur une grossesse, ça peut être des signes, quelqu'un qui a des céphalées atroces, des maux de tête atroces. Ce sont des signes de danger que nous donnons à la maman, et en même temps, lui donner l'alimentation qui va avec la grossesse en fonction de l'avancée de la grossesse. Tout ça pour que la maman puisse avoir une belle expérience de la grossesse. Ce qui va conduire à une facilité de l'accouchement. Parce qu'elle sera mieux préparée pour ça. Maintenant, pour l'enfant prématuré, même il y a le suivi de la grossesse, il y a la phase où le prématuré est dehors. Il est avec nous. Comment il faut le gérer ? Là, il faut vraiment un plateau qui répond. C'est ce que je disais, par le plateau, nous on n'a pas de couveuse. On n'a pas d'unité de pédiatrie, à plus forte raison de néonatologie. Donc, on a une unité de SMK, soins maternels kangourou. Vous savez que c'est l'hypothermie qui tue beaucoup plus. En dehors même de l'infection, l'hypothermie les tue plus. Les malnutris. C'est pour ça que je parlais de la lingerie, c'était pour les couvrir. Donc, SMK, on montre comment il faut faire. Le ventre à ventre, la maman et l'enfant, il faut couvrir pour que la chaleur maternelle puisse aider. Le kangourou, c'est comme l'animal kangourou qui porte son enfant. C'est un peu ça pour accompagner. Mais bon, si on ne peut pas gérer manifestement, qu'on l'évacue. On envoie ça à l'hôpital parce qu'ils ont un dispositif encore amélioré. 00:30:11-4

00:30:11-4 Interviewer: Est-ce que vous pensez que les pesanteurs socio-culturelles peuvent impacter la prise en charge de la maman et même du nourrisson prématuré ? 00:30:22-2

00:30:22-2 Personne 1: Quelles pesanteurs ? Je ne sais pas s'il y a des pesanteurs pour la prématurité. Je ne sais pas dire grand-chose. Je ne sais pas bien. Je ne sais pas s'il y a des appréhensions avec la prématurité. La grossesse, il y a des fausses couches. On dit que la grossesse n'est pas arrivée à maturité. Ça, c'est connu. L'avortement aussi, la société connaît ça. Je ne saurais pas dire grand-chose. 00:31:03-2

**00:27:04-2 Interviewer:** Based on everything you've discussed, what is your proposal to improve the care of premature births within the CMA de DÔ itself?

**00:27:17-8 Person 1:** Firstly, it begins with improved pregnancy monitoring. We encourage women to visit us early in the first trimester to receive optimal advice for their pregnancy. Pregnancy isn't an illness; it's a state that requires guidance on managing it effectively. During visits, we not only offer routine care but also educate mothers on danger signs that might indicate a risk of premature birth. Signs like severe abdominal pain, bleeding, or excruciating headaches in an incomplete term of pregnancy are red flags we teach them to recognize.We also emphasize proper nutrition throughout the pregnancy to ensure the mother's and baby's health. This preparation can lead to easier delivery as the mother is better informed and prepared.Regarding the care of the premature child, once the baby is born, we need a responsive platform. Currently, we lack facilities like an incubator, a pediatric unit, or a neonatology unit. We manage with a Kangaroo Mother Care (SMK) unit. Hypothermia is a major risk for premature babies, often more dangerous than infection. The SMK method involves skin-to-skin contact where the baby is held close to the mother's belly, similar to a kangaroo carrying its young, to provide warmth and prevent hypothermia. But if we're unable to manage a case effectively, we have no choice but to evacuate the child to a better-equipped facility.

**Interviewer (00:30:11-4):** "Do you believe that socio-cultural constraints can affect the care provided to the mother and the premature infant?"

**Person 1 (00:30:22-2):** "Regarding the impact of socio-cultural factors on prematurity, I'm not entirely sure. I'm not well-informed about any specific consequences related to prematurity in our society. As for pregnancy, miscarriages are a known issue, and it's understood that this means the pregnancy hasn't reached full term. Society is also aware of abortions. But when it comes to the specifics of how these issues might relate to premature births, I can't say much. I'm not fully aware of any particular apprehensions or beliefs surrounding prematurity.

### Manager for maternity care unit of Do medical center with surgical branch

[00:20:18-6](http://localhost:2300/file=C:/Users/USER/Desktop/ENTRETIENS%20PREMATURITE/CMA-DO/RESPONSABLE%20UNITE%20DE%20SOIN%20CMA-DO.mp3time=1218600) Interviewer: Comment est-ce qu'on peut améliorer la prise en charge des naissances prématurées? [00:20:34-2](http://localhost:2300/file=C:/Users/USER/Desktop/ENTRETIENS%20PREMATURITE/CMA-DO/RESPONSABLE%20UNITE%20DE%20SOIN%20CMA-DO.mp3time=1234200)

[00:20:34-2](http://localhost:2300/file=C:/Users/USER/Desktop/ENTRETIENS%20PREMATURITE/CMA-DO/RESPONSABLE%20UNITE%20DE%20SOIN%20CMA-DO.mp3time=1234200) Person 1: D'abord, pour pouvoir améliorer, je pense qu'il faut qu'on arrive à bien poser des diagnostics. À savoir qu'on est en face, par exemple, d'un accouchement prématuré. Parce que ça nous amène à prendre des dispositions pour bien le faire. D'abord, c'est ça. C'est le côté diagnostique. Que ce soit à travers la date des dènières règles ou bien un échographie qui va venir confirmer ou autre. Dès que cela est fait, je pense que si les précautions sont prises pour bien conduire cet accouchement-là, ça peut nous amener à éviter, ça veut dire que la mère même va être préparée déjà. Par exemple, la complication que j'ai soulignée à travers l'hypoglycémie. Savoir que peut-être ce bébé qui ne pourra pas prendre le lait rapidement, quelle va être la solution également pour parer à ça? Pour ne pas qu'on se retrouve avec une hypoglycémie et ainsi de suite. Et même parlant des infections aussi. Elle va être sensibilisée et même son entourage après cet accouchement. Également, peut-être prendre des dispositions nécessaires pour pouvoir assurer le transfert de ces bébés prématurés par exemple, du CMA vers le CHU. Parce que des fois, ça se fait soit par propre moyen de transport, si il n'y a pas l'ambulance. Ils seront obligés soit de transporter ce nouveau-né là, soit dans un véhicule, vraiment enveloppé c'est vrai.Mais ils n'ont pas assez d'informations des fois. Également, il faut que les agents aussi, nous les agents, il faut qu'il y ait vraiment peut-être une formation dans ce sens. Parce que c'est vrai que c'est fait à l'école de base. On a quelques notions là-dessus. Mais il arrive des fois, les gens ne maîtrisent pas trop la prise en charge. Même si ce n'est pas la prise en charge, comment transférer vraiment ce nouveau-né là du centre de naissance jusqu'au centre de prise en charge, qui est la neonatologie par exemple. Il faut qu'on ait vraiment, et que le circuit soit vraiment clairement défini. Les moyens également mis à disposition pour pouvoir le faire. Je pense que ça pourra en tout cas aider. [00:22:41-9](http://localhost:2300/file=C:/Users/USER/Desktop/ENTRETIENS%20PREMATURITE/CMA-DO/RESPONSABLE%20UNITE%20DE%20SOIN%20CMA-DO.mp3time=1361900)

[00:22:41-9](http://localhost:2300/file=C:/Users/USER/Desktop/ENTRETIENS%20PREMATURITE/CMA-DO/RESPONSABLE%20UNITE%20DE%20SOIN%20CMA-DO.mp3time=1361900) Interviewer: Par rapport à la formation dont vous venez de faire cas, est-ce que vous ne pensez pas que, à cause des mouvements souvent qui sont constatés au niveau des centres de santé, les mouvements des agents, souvent dûs à des affectations, des cas de retraites, est-ce que vous ne pensez pas que cela peut impacter sur la qualité des soins et des prises en charges au niveau du centre de santé? [00:23:03-8](http://localhost:2300/file=C:/Users/USER/Desktop/ENTRETIENS%20PREMATURITE/CMA-DO/RESPONSABLE%20UNITE%20DE%20SOIN%20CMA-DO.mp3time=1383800)

[00:23:03-8](http://localhost:2300/file=C:/Users/USER/Desktop/ENTRETIENS%20PREMATURITE/CMA-DO/RESPONSABLE%20UNITE%20DE%20SOIN%20CMA-DO.mp3time=1383800) Person 1: C'est vrai, oui, mais pas trop. Parce que je me dis, même si les gens sont formés, si la personne doit aller par affectation dans un autre poste, ça veut dire que dès qu'on est formé, on n'est pas sûr qu'on pourra former tout le monde. Le groupe peut-être qui a été formé pourra répercuter également cette compétence-là,aux autres qui sont là. Et même s'il y a un mouvement, ça veut dire que la compétence doit toujours rester. Et cette personne qui part ailleurs aussi pourra mettre en place cette compétence qu'elle a bénéficiée de l'autre côté, au profit également de cette population que nous avons la charge. Je me dis en tout cas, ça ne devrait pas vraiment impacter sur l'offre des soins. Parce que même si c'est une seule personne qui est formée, cette personne devrait pouvoir faire la restitution, également amener les autres aussi à pouvoir prendre en charge correctement ces cas-là. [00:24:00-7](http://localhost:2300/file=C:/Users/USER/Desktop/ENTRETIENS%20PREMATURITE/CMA-DO/RESPONSABLE%20UNITE%20DE%20SOIN%20CMA-DO.mp3time=1440700)

[00:24:00-7](http://localhost:2300/file=C:/Users/USER/Desktop/ENTRETIENS%20PREMATURITE/CMA-DO/RESPONSABLE%20UNITE%20DE%20SOIN%20CMA-DO.mp3time=1440700) Interviewer: Est-ce que vous pensez que les pesanteurs socio-culturelles peuvent impacter la prise en charge, peuvent impacter la mère et contribuer à biaiser un peu la prise en charge de l'enfant prématuré? [00:24:15-6](http://localhost:2300/file=C:/Users/USER/Desktop/ENTRETIENS%20PREMATURITE/CMA-DO/RESPONSABLE%20UNITE%20DE%20SOIN%20CMA-DO.mp3time=1455600)

[00:24:15-6](http://localhost:2300/file=C:/Users/USER/Desktop/ENTRETIENS%20PREMATURITE/CMA-DO/RESPONSABLE%20UNITE%20DE%20SOIN%20CMA-DO.mp3time=1455600) Person 1: Oui, je pense oui. Parce qu'ils vont se dire, déjà quand c'est un petit bébé prématuré, tout le monde va dire, aïe. Déjà, il y a le découragement qui peut se faire sentir. On va se dire, cet enfant-là ne va pas survivre. Et surtout, il arrive de voir quand c'est fille ou garçon, les gens ont une idée, en tout cas, une idée qui est là, une conception. Même dans le milieu des soins, on a tendance à dire, quand c'est les filles prématurées, les bébés-filles, elles survivent plus que les bébés-garçons. Alors qu'on n'a pas de preuves scientifiques par rapport à ça. Nous même, on le dit des fois. Quand c'est fille, on dit, si c'est fille, rassurez-vous, ça va bien se passer. Alors que quand c'est garçon, voilà, ça, on est un peu hésitant. Donc, ça fera que, effectivement, quand ils vont se retrouver avec un bébé prématuré sexe masculin, ils ne vont pas essayer aussi de mettre les moyens parce qu'ils se disent, aïe, est ce que ça va donner? Voilà. Également, parlant des pésenteurs, toujours aussi, il y a tellement des conceptions, des trucs autour de ça, où les gens vont vouloir donner d'autres informations, peut-être, dans la prise en charge de ce bébé prématuré-là, qui pourra peut-être même être la source d'infection. J'ai eu un cas, entre-temps, je sais que c'était quand j'étais élève, sage femme, c'était même une parente, elle avait accouché, mais elle avait eu, c'était un bébé prématuré, mais c'était même pas prématuré, c'était faible poids de naissance. Mais ils se sont dit en ce moment-là que c'était prématuré, tout, tout, tout. Parce que faible poids de naissance, les gens ne comprennent pas trop. Généralement, ils mettent tout dans la prématurité, dans la communauté, quoi. Ils mettent tout dans la prématurité. Donc, je pense qu'on avait dit que de faire quoi à l'enfant, au nouveau-né. Et ils avaient rasé, en fait, la tête du nouveau-né-là, et puis maintenant mettre un produit, voilà, sur la tête. Et celle qui avait fait les soins, parce que c'était une tradi-praticienne, et elle avait mis un produit dans la bouche, en tout cas, du nouveau-né.Et en voulant mettre le produit, je ne sais pas, parce que je n'ai pas trop demandé, mais bon, après, l'enfant même n'a pas survecu . Ils ont blessé, en fait, pas la langue, le palais, en haut, là. Tout était arraché, je ne sais pas. Voilà. Donc, ça fait qu'il y avait une plaie entre temps dans la bouche du nouveau-né. Il n'arrivait même plus à taîter. Et dans les consultations, les gens n'ont pas vraiment examiné pour savoir ce qui était dans la bouche, effectivement, du nouveau-né. Et c'est un jour, moi, j'ai essayé, bon, j'étais élève sage femme en 3ème année. Quand j'ai fait la bouche du nouveau-né, là, comme ça, du bébé, là, j'ai fait comme ça, et j'ai vu une masse au fond, en haut, là. Et quand j'ai vu ça, j'ai dit, ça, c'est quoi ? Elle a dit que non, non, non, que c'est quand ils sont allés voir la dame, là, le produit qu'ils ont mis en haut, là, et on a profité. Puisqu'on avait dit que "gnouna djiguira" (en dioula), je ne sais pas si vous comprenez le dioula. Alors que ce n'était pas ça. C'était un bébé prématuré qui avait autre difficulté, bon, faible poids de naissance, où on devrait vraiment prendre en charge le nouveau-né au niveau pédiatrique. Mais ils avaient séjourné pendant un temps donné et entre-temps, on les avait libérés. Ils devraient normalement continuer les soins. Et comme ils ont trouvé que cette dame, à travers ses soins, elle faisait des miracles, faisait quoi, quoi, quoi, ils sont allés là-bas. Et je pense que c'est en voulant mettre le doigt pour déposer un produit là-bas, elle a entraîné une plaie dans la bouche de l'enfant et ça s'est infecté. Ça s'est infecté. Donc je me dis, bon, il y a également cette conception là-autour, là, qui va aussi impacter un peu sur les soins qu'on va vraiment offrir à ce nouveau-né prématuré. Mais je pense que si les conseil accompagnent la prise en charge, en donnant vraiment clairement des informations, comment ils doivent tenir ce nouveau-né, voilà, est-ce qu'on doit le laver, est-ce qu'on doit le gaver. Déjà, on sait que le gavage, purge et autres là, tout est interdit chez tout nouveau-né. Voilà. Donc, mais c'est d'insister encore avec ce bébé qui est fragile. Voilà. Je pense que ça peut aider. [00:28:32-0](http://localhost:2300/file=C:/Users/USER/Desktop/ENTRETIENS%20PREMATURITE/CMA-DO/RESPONSABLE%20UNITE%20DE%20SOIN%20CMA-DO.mp3time=1712000)

00:20:18-6 Interviewer: Comment est-ce qu'on peut améliorer la prise en charge des naissances prématurées ? 00:20:34-2

00:20:34-2 Person 1: D'abord, pour pouvoir améliorer, je pense qu'il faut qu'on arrive à bien poser des diagnostics. À savoir qu'on est en face, par exemple, d'un accouchement prématuré. Parce que ça nous amène à prendre des dispositions pour bien le faire. D'abord, c'est ça. C'est le côté diagnostique. Que ce soit à travers la date des dernières règles ou bien une échographie qui va venir confirmer ou autre. Dès que cela est fait, je pense que si les précautions sont prises pour bien conduire cet accouchement-là, ça peut nous amener à éviter, ça veut dire que la mère même va être préparée déjà. Par exemple, la complication que j'ai soulignée à travers l'hypoglycémie. Savoir que peut-être ce bébé qui ne pourra pas prendre le lait rapidement, quelle va être la solution également pour parer à ça ? Pour ne pas qu'on se retrouve avec une hypoglycémie et ainsi de suite. Et même parlant des infections aussi. Elle va être sensibilisée et même son entourage après cet accouchement. Également, peut-être prendre des dispositions nécessaires pour pouvoir assurer le transfert de ces bébés prématurés par exemple, du CMA vers le CHU. Parce que des fois, ça se fait soit par propre moyen de transport, si il n'y a pas l'ambulance. Ils seront obligés soit de transporter ce nouveau-né là, soit dans un véhicule, vraiment enveloppé c'est vrai. Mais ils n'ont pas assez d'informations des fois. Également, il faut que les agents aussi, nous les agents, il faut qu'il y ait vraiment peut-être une formation dans ce sens. Parce que c'est vrai que c'est fait à l'école de base. On a quelques notions là-dessus. Mais il arrive des fois, les gens ne maîtrisent pas trop la prise en charge. Même si ce n'est pas la prise en charge, comment transférer vraiment ce nouveau-né là du centre de naissance jusqu'au centre de prise en charge, qui est la néonatologie par exemple. Il faut qu'on ait vraiment, et que le circuit soit vraiment clairement défini. Les moyens également mis à disposition pour pouvoir le faire. Je pense que ça pourra en tout cas aider. 00:22:41-9

00:22:41-9 Interviewer: Par rapport à la formation dont vous venez de faire cas, est-ce que vous ne pensez pas que, à cause des mouvements souvent qui sont constatés au niveau des centres de santé, les mouvements des agents, souvent dus à des affectations, des cas de retraites, est-ce que vous ne pensez pas que cela peut impacter sur la qualité des soins et des prises en charges au niveau du centre de santé ? 00:23:03-8

00:23:03-8 Person 1: C'est vrai, oui, mais pas trop. Parce que je me dis, même si les gens sont formés, si la personne doit aller par affectation dans un autre poste, ça veut dire que dès qu'on est formé, on n'est pas sûr qu'on pourra former tout le monde. Le groupe peut-être qui a été formé pourra répercuter également cette compétence-là, aux autres qui sont là. Et même s'il y a un mouvement, ça veut dire que la compétence doit toujours rester. Et cette personne qui part ailleurs aussi pourra mettre en place cette compétence qu'elle a bénéficiée de l'autre côté, au profit également de cette population que nous avons la charge. Je me dis en tout cas, ça ne devrait pas vraiment impacter sur l'offre des soins. Parce que même si c'est une seule personne qui est formée, cette personne devrait pouvoir faire la restitution, également amener les autres aussi à pouvoir prendre en charge correctement ces cas-là. 00:24:00-7

00:24:00-7 Interviewer: Est-ce que vous pensez que les pesanteurs socio-culturelles peuvent impacter la prise en charge, peuvent impacter la mère et contribuer à biaiser un peu la prise en charge de l'enfant prématuré ? 00:24:15-6

00:24:15-6 Personne 1: Oui, je pense oui. Parce qu'ils vont se dire, déjà quand c'est un petit bébé prématuré, tout le monde va dire, aïe. Déjà, il y a le découragement qui peut se faire sentir. On va se dire, cet enfant-là ne va pas survivre. Et surtout, il arrive de voir quand c'est fille ou garçon, les gens ont une idée, en tout cas, une conception. Même dans le milieu des soins, on a tendance à dire, quand c'est les filles prématurées, les bébés-filles, elles survivent plus que les bébés-garçons. Alors qu'on n'a pas de preuves scientifiques par rapport à ça. Nous-même, on le dit des fois. Quand c'est fille, on dit, si c'est fille, rassurez-vous, ça va bien se passer. Alors que quand c'est garçon, voilà, ça, on est un peu hésitant. Donc, ça fera que, effectivement, quand ils vont se retrouver avec un bébé prématuré de sexe masculin, ils ne vont pas essayer aussi de mettre les moyens parce qu'ils se disent, aïe, est-ce que ça va donner ? Voilà. Également, parlant des pesanteurs, toujours aussi, il y a tellement de conceptions, des trucs autour de ça, où les gens vont vouloir donner d'autres informations, peut-être, dans la prise en charge de ce bébé prématuré-là, qui pourra peut-être même être la source d'infection. J'ai eu un cas, entre-temps, je sais que c'était quand j'étais élève sage-femme, c'était même une parente, elle avait accouché, mais elle avait eu, c'était un bébé prématuré, mais c'était même pas prématuré, c'était un faible poids de naissance. Mais ils se sont dit en ce moment-là que c'était prématuré, tout, tout, tout. Parce que faible poids de naissance, les gens ne comprennent pas trop. Généralement, ils mettent tout dans la prématurité, dans la communauté, quoi. Ils mettent tout dans la prématurité. Donc, je pense qu'on avait dit que de faire quoi à l'enfant, au nouveau-né. Et ils avaient rasé, en fait, la tête du nouveau-né-là, et puis maintenant mettre un produit, voilà, sur la tête. Et celle qui avait fait les soins, parce que c'était une tradi-praticienne, et elle avait mis un produit dans la bouche, en tout cas, du nouveau-né. Et en voulant mettre le produit, je ne sais pas, parce que je n'ai pas trop demandé, mais bon, après, l'enfant même n'a pas survécu. Ils ont blessé, en fait, pas la langue, le palais, en haut, là. Tout était arraché, je ne sais pas. Voilà. Donc, ça fait qu'il y avait une plaie entre-temps dans la bouche du nouveau-né. Il n'arrivait même plus à téter. Et dans les consultations, les gens n'ont pas vraiment examiné pour savoir ce qui était dans la bouche, effectivement, du nouveau-né. Et c'est un jour, moi, j'ai essayé, bon, j'étais élève sage-femme en 3ème année. Quand j'ai fait la bouche du nouveau-né, là, comme ça, du bébé, là, j'ai fait comme ça, et j'ai vu une masse au fond, en haut, là. Et quand j'ai vu ça, j'ai dit, ça, c'est quoi ? Elle a dit que non, non, non, que c'est quand ils sont allés voir la dame, là, le produit qu'ils ont mis en haut, là, et on a profité. Puisqu'on avait dit que "gnouna djiguira" (en dioula), je ne sais pas si vous comprenez le dioula. Alors que ce n'était pas ça. C'était un bébé prématuré qui avait autre difficulté, bon, un faible poids de naissance, où on devrait vraiment prendre en charge le nouveau-né au niveau pédiatrique. Mais ils avaient séjourné pendant un temps donné et entre-temps, on les avait libérés. Ils devraient normalement continuer les soins. Et comme ils ont trouvé que cette dame, à travers ses soins, elle faisait des miracles, faisait quoi, quoi, quoi, ils sont allés là-bas. Et je pense que c'est en voulant mettre le doigt pour déposer un produit là-bas, elle a entraîné une plaie dans la bouche de l'enfant et ça s'est infecté. Ça s'est infecté. Donc je me dis, bon, il y a également cette conception là-autour, là, qui va aussi impacter un peu sur les soins qu'on va vraiment offrir à ce nouveau-né prématuré. Mais je pense que si les conseils accompagnent la prise en charge, en donnant vraiment clairement des informations, comment ils doivent tenir ce nouveau-né, voilà, est-ce qu'on doit le laver, est-ce qu'on doit le gaver. Déjà, on sait que le gavage, purge et autres là, tout est interdit chez tout nouveau-né. Voilà. Donc, mais c'est d'insister encore avec ce bébé qui est fragile. Voilà. Je pense que ça peut aider. 00:28:32-0

**00:20:18-6 Interviewer:** How can we improve the care of premature births?

**00:20:34-2 Person 1:** First, accurate diagnosis is crucial. We need to reliably identify premature births, whether through the date of the last menstrual period or an ultrasound. Once diagnosed, we can take appropriate measures to manage the birth, preparing the mother for potential complications, like hypoglycemia, and how to address them. Awareness and preparation for potential infections post-delivery are also important.Another key aspect is arranging for the safe transfer of premature babies from facilities like the CMA to the CHU, especially in cases where there is no ambulance available. We also need training for health workers, not just in care but in how to safely transfer newborns to specialized care centers like neonatology units. It's essential to have a clear protocol and the necessary resources for these transfers.

**00:22:41-9 Interviewer:** Regarding training, given the frequent staff movements in health centers, do you think this affects the quality of care and support?

**00:23:03-8 Person 1:** It can have an impact, but not significantly. Even if staff are trained and then assigned elsewhere, the knowledge they've acquired can be shared with others in the group. This way, the expertise remains within the center. Additionally, those who move to new locations can impart their skills there, benefiting a wider population. So, while staff movement is a factor, it should not drastically affect the overall quality of care. Trained individuals can play a key role in disseminating knowledge and guiding others in managing premature births effectively.

**Interviewer (00:24:00-7):** "Do you think socio-cultural constraints can influence the care provided to premature infants and their mothers, possibly leading to biased treatment?"

**Person 1 (00:24:15-6):** "Yes, I believe so. There's a tendency to feel discouraged when dealing with a premature baby. People often prematurely assume that the child won't survive. There's also a perception, even among healthcare workers, that premature girls have better survival rates than boys, though there's no scientific evidence for this. This belief can lead to hesitancy in providing care, especially for premature boys, as people might doubt the effectiveness of treatment. Moreover, there are many misconceptions and harmful practices in the community regarding the care of premature babies. For instance, I recall a case from my time as a midwifery student. A relative had a baby with low birth weight, but the community assumed it was a case of prematurity. They shaved the baby's head and applied a product to it, and a traditional practitioner put something in the baby's mouth, which caused an injury and infection. The baby couldn't breastfeed anymore, and the injury went unnoticed in medical examinations. Only when I examined the baby did I discover a mass in its mouth, a result of the harmful practice.This incident highlights the impact of socio-cultural beliefs and practices on the care of premature babies. However, I think proper guidance and clear information about how to care for these fragile babies, including advice against force-feeding and purging, can make a significant difference

### Focus group of Colma1 health and social promotion center

[00:22:19-9](http://localhost:2300/file=C:/Users/USER/Desktop/ENTRETIENS%20PREMATURITE/CSPS-COLMA1/FOCUS%20GROUPE%20COLMA1.mp3time=1339900) Interviewer: Maintenant à travers tout ce qui vient d'être dit, qu'est ce que vous proposez pour ameliorer la prise en charge des naissances prématurées au sein de votre centre de santé? [00:22:35-0](http://localhost:2300/file=C:/Users/USER/Desktop/ENTRETIENS%20PREMATURITE/CSPS-COLMA1/FOCUS%20GROUPE%20COLMA1.mp3time=1355000)

[00:22:35-0](http://localhost:2300/file=C:/Users/USER/Desktop/ENTRETIENS%20PREMATURITE/CSPS-COLMA1/FOCUS%20GROUPE%20COLMA1.mp3time=1355000) Person 2: La prise en charge après naissance ou bien avant? [00:22:39-1](http://localhost:2300/file=C:/Users/USER/Desktop/ENTRETIENS%20PREMATURITE/CSPS-COLMA1/FOCUS%20GROUPE%20COLMA1.mp3time=1359100)

[00:22:39-1](http://localhost:2300/file=C:/Users/USER/Desktop/ENTRETIENS%20PREMATURITE/CSPS-COLMA1/FOCUS%20GROUPE%20COLMA1.mp3time=1359100) Interviewer: Quel que soit avant ou après.Quelles sont les propositions que vous pouvez faire? [00:22:46-0](http://localhost:2300/file=C:/Users/USER/Desktop/ENTRETIENS%20PREMATURITE/CSPS-COLMA1/FOCUS%20GROUPE%20COLMA1.mp3time=1366000)

[00:22:46-0](http://localhost:2300/file=C:/Users/USER/Desktop/ENTRETIENS%20PREMATURITE/CSPS-COLMA1/FOCUS%20GROUPE%20COLMA1.mp3time=1366000) Person 2: Bon si c'est pendant la grossesse on peut insister pour dire aux femmes de consulter tôt, de ne pas laisser le mal s'aggraver avant de venir à la consultation, sensibiliser les femmes sur les signes de danger pendant la CPN.Maintenant, après accouchement, si au niveau CSPS nous pouvons avoir des couveuses (rires) ci-possible par exemple même le CMA n'en a pas c'est pourquoi nous sommes obligés d'aller jusqu'au CHURSS . Au moins si on pouvait doter les grandes formations sanitaires en couveuses cela contribuerait à amoindrir les risques de decès néonatal. Il y a quelque chose à ajouter? [00:23:50-1](http://localhost:2300/file=C:/Users/USER/Desktop/ENTRETIENS%20PREMATURITE/CSPS-COLMA1/FOCUS%20GROUPE%20COLMA1.mp3time=1430100)

[00:23:50-1](http://localhost:2300/file=C:/Users/USER/Desktop/ENTRETIENS%20PREMATURITE/CSPS-COLMA1/FOCUS%20GROUPE%20COLMA1.mp3time=1430100) Person 5: Moi j'allais ajouter,peut être former le personnel sur la prise en charge immediate dès la sortie. Qu'est ce qu'il faut faire au nouveau né pour le protèger en attendant qu'il soit evacuer au CHURSS? cela peut être capital parce que s'il n'a pas eu les premiers soins necessaires cela peut jouer sur la suite. donc il faut former le personnel à la prématurité pour qu'il puisse mieux agir avant l'evacuation. [00:24:15-8](http://localhost:2300/file=C:/Users/USER/Desktop/ENTRETIENS%20PREMATURITE/CSPS-COLMA1/FOCUS%20GROUPE%20COLMA1.mp3time=1455800)

[00:24:15-8](http://localhost:2300/file=C:/Users/USER/Desktop/ENTRETIENS%20PREMATURITE/CSPS-COLMA1/FOCUS%20GROUPE%20COLMA1.mp3time=1455800) Person 2: il y a les cas de lampes chauffantes aussi. si nous pouvons avoir des lampes chauffantes au niveau CSPS, parce qu'à defaut de couveuses au moins les lampes chauffantes cela pourrait aider. [00:24:29-9](http://localhost:2300/file=C:/Users/USER/Desktop/ENTRETIENS%20PREMATURITE/CSPS-COLMA1/FOCUS%20GROUPE%20COLMA1.mp3time=1469900)

[00:24:29-9](http://localhost:2300/file=C:/Users/USER/Desktop/ENTRETIENS%20PREMATURITE/CSPS-COLMA1/FOCUS%20GROUPE%20COLMA1.mp3time=1469900) Interviewer: Vous avez parler tantôt des signes de dangers, à quels signes vous faites allusion? [00:24:41-6](http://localhost:2300/file=C:/Users/USER/Desktop/ENTRETIENS%20PREMATURITE/CSPS-COLMA1/FOCUS%20GROUPE%20COLMA1.mp3time=1481600)

[00:24:41-6](http://localhost:2300/file=C:/Users/USER/Desktop/ENTRETIENS%20PREMATURITE/CSPS-COLMA1/FOCUS%20GROUPE%20COLMA1.mp3time=1481600) Person 2: Au niveau des signes, on a les douleurs pelviennes, les cas de saignement, les cas de fièvres, céphalés, vertiges voilà il y a beaucoup de signes. [00:25:05-9](http://localhost:2300/file=C:/Users/USER/Desktop/ENTRETIENS%20PREMATURITE/CSPS-COLMA1/FOCUS%20GROUPE%20COLMA1.mp3time=1505900)

[00:25:05-9](http://localhost:2300/file=C:/Users/USER/Desktop/ENTRETIENS%20PREMATURITE/CSPS-COLMA1/FOCUS%20GROUPE%20COLMA1.mp3time=1505900) Person 5: aussi il y a un aspect que toi même tu as dis, psychologique et social. Souvent les femmes ont des problèmes à la maison, ça ne va pas, elles viennent se confier à tantie(Person 7: ). Donc le problème social est là et lorsqu'elles (les femmes enceintes) se confient souvent tantie convoque le mari pour echanger avec ce dernier et beaucoup de fois cela donne des resultats concluants cela veut dire que la mediation marche bien.,il y a aussi cet aspect. [00:25:26-1](http://localhost:2300/file=C:/Users/USER/Desktop/ENTRETIENS%20PREMATURITE/CSPS-COLMA1/FOCUS%20GROUPE%20COLMA1.mp3time=1526100)

[00:25:26-1](http://localhost:2300/file=C:/Users/USER/Desktop/ENTRETIENS%20PREMATURITE/CSPS-COLMA1/FOCUS%20GROUPE%20COLMA1.mp3time=1526100) Interviewer: C'est un aspect qui allait venir en question mais nous allons evoluer. Vous avez evoquer entre temps pour ameliorer la prise en charge des naissances prématurées de la formation, mais quelles propositions pouvez vous faire en cas d'affectation des agents de santé qui ont réçu une formation specifique sur la prematurité pour que cela n'affecte pas sur la qualité au sein du CSPS en terme de prestation de services de qualités? [00:25:56-2](http://localhost:2300/file=C:/Users/USER/Desktop/ENTRETIENS%20PREMATURITE/CSPS-COLMA1/FOCUS%20GROUPE%20COLMA1.mp3time=1556200)

[00:25:56-2](http://localhost:2300/file=C:/Users/USER/Desktop/ENTRETIENS%20PREMATURITE/CSPS-COLMA1/FOCUS%20GROUPE%20COLMA1.mp3time=1556200) Person 5: bon moi je me dis que sur 10 agents formés, deux ou trois partent, mais ceux qui vont venir les remplacer vont venir trouver une habitude au sein du CSPS, c'est comme s'ils vont se former sur site, ils vont suivre l'habitude que nous faisons. L'agent qui part ailleurs va apporter sa connaissance làbas et cela sera un apport pour ce CSPS. [00:26:40-0](http://localhost:2300/file=C:/Users/USER/Desktop/ENTRETIENS%20PREMATURITE/CSPS-COLMA1/FOCUS%20GROUPE%20COLMA1.mp3time=1600000)

[00:26:40-0](http://localhost:2300/file=C:/Users/USER/Desktop/ENTRETIENS%20PREMATURITE/CSPS-COLMA1/FOCUS%20GROUPE%20COLMA1.mp3time=1600000) Interviewer: Maintenant vous avez parler des pesenteurs socio-culturels qui peuvent être des facteurs de decès pour les naissances prématurées, A quoi faites vous allusion? [00:26:52-9](http://localhost:2300/file=C:/Users/USER/Desktop/ENTRETIENS%20PREMATURITE/CSPS-COLMA1/FOCUS%20GROUPE%20COLMA1.mp3time=1612900)

[00:26:52-9](http://localhost:2300/file=C:/Users/USER/Desktop/ENTRETIENS%20PREMATURITE/CSPS-COLMA1/FOCUS%20GROUPE%20COLMA1.mp3time=1612900) Person 6: Dès fois il y a des femmes qui viennent se confier pour dire que <tantie, je suis enceinte de 6 mois et le monsieur dit que ce n'est pas pour lui>, et moi je suis obligée d'inviter le monsieur, j'ai une note d'invitation même pour cela. j'echange avec le monsieur, je le sensibilise et je conseil d'accepter la paternité de sa grossesse, en tout cas la plus part du temps cela marche bien. j'ai eu ce matin même un cas ou une femme au 7ème mois, le monsieur voulait que la dame avorte, c'est compliquer il y a des cas ou l'homme demande à la femme de quitter la maison parce qu'il n'est pas prêt pour un enfant et ceci dans le foyer, donc j'essaie toujours de sensibiliser les parties. vous voyez, lorsque ces cas arrivent, sil n y a pas de conseils,l'enfant va aller dans le cas de prematurité puisqu'il n y a pas d'entente.Se sont toutes ses situations conflictuelles qui peuvent amener la femme à donner naissance à un enfant prématuré. C'est pour cela que j'ai une note d'invitation, quand une femme vient pour la première CPN, je lui donne la note d'invitation à l'endroit de son mari pour avoir un entretien avec le couple pour qu'il y ait plus d'union entre eux parce que leur désunion joue negativement sur l'enfant. je sensibilise les hommes et beaucoup acceptent les conseils et me remercient. [00:29:54-4](http://localhost:2300/file=C:/Users/USER/Desktop/ENTRETIENS%20PREMATURITE/CSPS-COLMA1/FOCUS%20GROUPE%20COLMA1.mp3time=1794400)

[00:29:54-4](http://localhost:2300/file=C:/Users/USER/Desktop/ENTRETIENS%20PREMATURITE/CSPS-COLMA1/FOCUS%20GROUPE%20COLMA1.mp3time=1794400) Person 7: moi je voulais ajouter, souvent nous avons des cas de violence physique et même pendant les rapports il y a des hommes qui sont très violents jusqu'à la femme "se cherche même". une grossesse que nous sommes entrain de suivre parce qu'il y a un risque de décollement, on appel le monsieur pour qu'il fasse doucement mais il ne va pas accepter, il va forcer et cela peut entrainer une naissance prématurée. [00:30:32-6](http://localhost:2300/file=C:/Users/USER/Desktop/ENTRETIENS%20PREMATURITE/CSPS-COLMA1/FOCUS%20GROUPE%20COLMA1.mp3time=1832600)

[00:30:32-6](http://localhost:2300/file=C:/Users/USER/Desktop/ENTRETIENS%20PREMATURITE/CSPS-COLMA1/FOCUS%20GROUPE%20COLMA1.mp3time=1832600) Interviewer: Quelle est le regard de la société sur l'enfant prématuré ou la mère qui a donné naissance à cet enfant prématuré? Comment est ce que cette femme est perçue dans la communauté? [00:30:46-9](http://localhost:2300/file=C:/Users/USER/Desktop/ENTRETIENS%20PREMATURITE/CSPS-COLMA1/FOCUS%20GROUPE%20COLMA1.mp3time=1846900)

[00:30:46-9](http://localhost:2300/file=C:/Users/USER/Desktop/ENTRETIENS%20PREMATURITE/CSPS-COLMA1/FOCUS%20GROUPE%20COLMA1.mp3time=1846900) Person 5: Par rapport à la perception ça, je ne peut rien dire mais l'enfant, je constate que presentement les gens prennent un peu conscience de la prématurité des bébés. ils disent que cet enfant ne doit pas sortir dehors, il doit être protèger, en tout cas de bouche à oreille, ils ont un peu de connaissance sur la prématurité. il ne faut pas le laver ou bien à defaut que se soit dans la chambre. pour l'entretien du nouveau né, ils comprennent beaucoup de chose et lorsque nous les conseillons, ils acceptent, surtout s'il y a une belle-mère à qui tu donne les conseils, je sais qu'elle va les suivre à la lettre. [00:31:26-3](http://localhost:2300/file=C:/Users/USER/Desktop/ENTRETIENS%20PREMATURITE/CSPS-COLMA1/FOCUS%20GROUPE%20COLMA1.mp3time=1886300)

[00:31:26-3](http://localhost:2300/file=C:/Users/USER/Desktop/ENTRETIENS%20PREMATURITE/CSPS-COLMA1/FOCUS%20GROUPE%20COLMA1.mp3time=1886300) Person 3: moi je peux ajouter que, pour la femme, s'elle a ses problèmes comme précedemment évoqués, cela peut augmenter sa tension (hypertension) et cela joue vraiment sur la grossesse. lorsque nous ne nous rendons pas compte tôt de son hypertension,rapidement elle peut donner naissance à un enfant prématuré. [00:32:05-5](http://localhost:2300/file=C:/Users/USER/Desktop/ENTRETIENS%20PREMATURITE/CSPS-COLMA1/FOCUS%20GROUPE%20COLMA1.mp3time=1925500)

[00:32:05-5](http://localhost:2300/file=C:/Users/USER/Desktop/ENTRETIENS%20PREMATURITE/CSPS-COLMA1/FOCUS%20GROUPE%20COLMA1.mp3time=1925500) Interviewer: Est ce qu'il y a quelque chose d'autre à ajouter? [00:32:11-6](http://localhost:2300/file=C:/Users/USER/Desktop/ENTRETIENS%20PREMATURITE/CSPS-COLMA1/FOCUS%20GROUPE%20COLMA1.mp3time=1931600)

[00:32:11-6](http://localhost:2300/file=C:/Users/USER/Desktop/ENTRETIENS%20PREMATURITE/CSPS-COLMA1/FOCUS%20GROUPE%20COLMA1.mp3time=1931600) Person 6: il y avait un cas chez nous ou une femme a donner naissance à un prématuré mais cela était dû au fait que son mari soit déceder , dans la belle famille ça n'allait pas du tout, on ne l'aime pas, on ne la donne pas à manger.Cette dernière avait même commencer ses CPN, mais la deuxième fois qu'elle est revenue, sa tension n'était pas decendue et ça continuait de monter.Nous l'avons dmandé s'elle suit vraiment son traitement, et c'est en ce moment qu'elle a dit que depuis le decès de son mari, étant en belle famille ce n'est pas simple, nous avons prescrit les medicaments sur l'ordonnance qu'elle devait aller payer et au cas ou elle n'arrive pas à payer elle pouvait toujours revenir,nous n'avons même pas eu ce temps là, cette dernière n'est plus revenue, donc elle a accouché d'un mort-né et ce n'était même pas prématuré. [00:33:13-3](http://localhost:2300/file=C:/Users/USER/Desktop/ENTRETIENS%20PREMATURITE/CSPS-COLMA1/FOCUS%20GROUPE%20COLMA1.mp3time=1993300)

[00:33:13-3](http://localhost:2300/file=C:/Users/USER/Desktop/ENTRETIENS%20PREMATURITE/CSPS-COLMA1/FOCUS%20GROUPE%20COLMA1.mp3time=1993300) Interviewer: Elle était à combien de moi de grossesse? [00:33:14-8](http://localhost:2300/file=C:/Users/USER/Desktop/ENTRETIENS%20PREMATURITE/CSPS-COLMA1/FOCUS%20GROUPE%20COLMA1.mp3time=1994800)

[00:33:14-8](http://localhost:2300/file=C:/Users/USER/Desktop/ENTRETIENS%20PREMATURITE/CSPS-COLMA1/FOCUS%20GROUPE%20COLMA1.mp3time=1994800) Person 6: Ah, est que je me rappel encore, en tout cas ça ne depasse pas le deuxième trimestre, ça devrait être autour de 5 mois. le jour qu'elle a eu les medicaments, elle a fait une seule prise et c'est ce jour elle venue expulser. [00:33:50-0](http://localhost:2300/file=C:/Users/USER/Desktop/ENTRETIENS%20PREMATURITE/CSPS-COLMA1/FOCUS%20GROUPE%20COLMA1.mp3time=2030000)

[00:33:50-0](http://localhost:2300/file=C:/Users/USER/Desktop/ENTRETIENS%20PREMATURITE/CSPS-COLMA1/FOCUS%20GROUPE%20COLMA1.mp3time=2030000) Interviewer: Est ce qu'il y a quelque chose d'autre à ajouter? [00:33:55-7](http://localhost:2300/file=C:/Users/USER/Desktop/ENTRETIENS%20PREMATURITE/CSPS-COLMA1/FOCUS%20GROUPE%20COLMA1.mp3time=2035700)

[00:33:55-7](http://localhost:2300/file=C:/Users/USER/Desktop/ENTRETIENS%20PREMATURITE/CSPS-COLMA1/FOCUS%20GROUPE%20COLMA1.mp3time=2035700) Person 6: il y a aussi les cas des personnes déplacées internes, quand elles viennent ,e mari est soit déceder soit abscent, elle a eu la chance de fuir pur venir chez ses parents, il n' y a personne pour s'occuper d'elle. Moi je suis obligée d'appeler la famille pour les sensibiliser.Parce que sans le soutien la grossesse de la femme va être très difficile. je sensibilise beaucoup sur l'amour dans le foyer, le soutien etc. [00:35:13-7](http://localhost:2300/file=C:/Users/USER/Desktop/ENTRETIENS%20PREMATURITE/CSPS-COLMA1/FOCUS%20GROUPE%20COLMA1.mp3time=2113700)

00:22:19-9 Interviewer: Maintenant, à travers tout ce qui vient d'être dit, qu'est-ce que vous proposez pour améliorer la prise en charge des naissances prématurées au sein de votre centre de santé ? 00:22:35-0

00:22:35-0 Personne 2: La prise en charge après naissance ou bien avant ? 00:22:39-1

00:22:39-1 Interviewer: Quel que soit, avant ou après. Quelles sont les propositions que vous pouvez faire ? 00:22:46-0

00:22:46-0 Personne 2: Bon, si c'est pendant la grossesse, on peut insister pour dire aux femmes de consulter tôt, de ne pas laisser le mal s'aggraver avant de venir à la consultation, sensibiliser les femmes sur les signes de danger pendant la CPN. Maintenant, après accouchement, si au niveau CSPS nous pouvons avoir des couveuses (rires), si possible, par exemple, même le CMA n'en a pas, c'est pourquoi nous sommes obligés d'aller jusqu'au CHURSS. Au moins si on pouvait doter les grandes formations sanitaires en couveuses, cela contribuerait à amoindrir les risques de décès néonatal. Il y a quelque chose à ajouter ? 00:23:50-1

00:23:50-1 Personne 5: Moi, j'allais ajouter, peut-être former le personnel sur la prise en charge immédiate dès la sortie. Qu'est-ce qu'il faut faire au nouveau-né pour le protéger en attendant qu'il soit évacué au CHURSS ? Cela peut être capital, parce que s'il n'a pas eu les premiers soins nécessaires, cela peut jouer sur la suite. Donc, il faut former le personnel à la prématurité pour qu'il puisse mieux agir avant l'évacuation. 00:24:15-8

00:24:15-8 Personne 2: Il y a les cas de lampes chauffantes aussi. Si nous pouvons avoir des lampes chauffantes au niveau CSPS, parce qu'à défaut de couveuses, au moins les lampes chauffantes, cela pourrait aider. 00:24:29-9

00:24:29-9 Interviewer: Vous avez parlé tantôt des signes de dangers, à quels signes vous faites allusion ? 00:24:41-6

00:24:41-6 Personne 2: Au niveau des signes, on a les douleurs pelviennes, les cas de saignement, les cas de fièvres, céphalées, vertiges, voilà, il y a beaucoup de signes. 00:25:05-9

00:25:05-9 Personne 5: Aussi, il y a un aspect que toi-même tu as dit, psychologique et social. Souvent les femmes ont des problèmes à la maison, ça ne va pas, elles viennent se confier à tantie (Personne 7: ). Donc, le problème social est là, et lorsqu'elles (les femmes enceintes) se confient, souvent tantie convoque le mari pour échanger avec ce dernier, et beaucoup de fois, cela donne des résultats concluants, cela veut dire que la médiation marche bien. Il y a aussi cet aspect. 00:25:26-1

00:25:26-1 Interviewer: C'est un aspect qui allait venir en question, mais nous allons évoluer. Vous avez évoqué entre-temps pour améliorer la prise en charge des naissances prématurées de la formation, mais quelles propositions pouvez-vous faire en cas d'affectation des agents de santé qui ont reçu une formation spécifique sur la prématurité pour que cela n'affecte pas la qualité au sein du CSPS en terme de prestation de services de qualité ? 00:25:56-2

00:25:56-2 Personne 5: Bon, moi, je me dis que sur 10 agents formés, deux ou trois partent, mais ceux qui vont venir les remplacer vont trouver une habitude au sein du CSPS, c'est comme s'ils vont se former sur site, ils vont suivre l'habitude que nous faisons. L'agent qui part ailleurs va apporter sa connaissance là-bas et cela sera un apport pour ce CSPS. 00:26:40-0

00:26:40-0 Interviewer: Maintenant, vous avez parlé des pesanteurs socio-culturelles qui peuvent être des facteurs de décès pour les naissances prématurées. À quoi faites-vous allusion ? 00:26:52-9

00:26:52-9 Personne 6: Parfois, il y a des femmes qui viennent se confier pour dire que <tantie, je suis enceinte de 6 mois et le monsieur dit que ce n'est pas pour lui>, et moi, je suis obligée d'inviter le monsieur, j'ai une note d'invitation même pour cela. J'échange avec le monsieur, je le sensibilise et je conseille d'accepter la paternité de sa grossesse, en tout cas la plupart du temps cela marche bien. J'ai eu ce matin même un cas où une femme au 7ème mois, le monsieur voulait que la dame avorte, c'est compliqué, il y a des cas où l'homme demande à la femme de quitter la maison parce qu'il n'est pas prêt pour un enfant et ceci dans le foyer, donc j'essaie toujours de sensibiliser les parties. Vous voyez, lorsque ces cas arrivent, s'il n'y a pas de conseils, l'enfant va aller dans le cas de prématurité puisqu'il n'y a pas d'entente. Ce sont toutes ces situations conflictuelles qui peuvent amener la femme à donner naissance à un enfant prématuré. C'est pour cela que j'ai une note d'invitation, quand une femme vient pour la première CPN, je lui donne la note d'invitation à l'endroit de son mari pour avoir un entretien avec le couple pour qu'il y ait plus d'union entre eux, parce que leur désunion joue négativement sur l'enfant. Je sensibilise les hommes, et beaucoup acceptent les conseils et me remercient. 00:29:54-4

00:29:54-4 Personne 7: Moi, je voulais ajouter, souvent nous avons des cas de violence physique et même pendant les rapports, il y a des hommes qui sont très violents jusqu'à ce que la femme "se cherche même". Une grossesse que nous sommes en train de suivre parce qu'il y a un risque de décollement, on appelle le monsieur pour qu'il fasse doucement, mais il ne va pas accepter, il va forcer et cela peut entraîner une naissance prématurée. 00:30:32-6

00:30:32-6 Interviewer: Quel est le regard de la société sur l'enfant prématuré ou la mère qui a donné naissance à cet enfant prématuré ? Comment est-ce que cette femme est perçue dans la communauté ? 00:30:46-9

00:30:46-9 Personne 5: Par rapport à la perception, je ne peux rien dire, mais pour l'enfant, je constate que présentement les gens prennent un peu conscience de la prématurité des bébés. Ils disent que cet enfant ne doit pas sortir dehors, il doit être protégé, en tout cas de bouche à oreille, ils ont un peu de connaissance sur la prématurité. Il ne faut pas le laver ou bien, à défaut, que ce soit dans la chambre. Pour l'entretien du nouveau-né, ils comprennent beaucoup de choses et lorsque nous les conseillons, ils acceptent, surtout s'il y a une belle-mère à qui tu donnes les conseils, je sais qu'elle va les suivre à la lettre. 00:31:26-3

00:31:26-3 Personne 3: Moi, je peux ajouter que, pour la femme, si elle a ses problèmes comme précédemment évoqués, cela peut augmenter sa tension (hypertension) et cela joue vraiment sur la grossesse. Lorsque nous ne nous rendons pas compte tôt de son hypertension, rapidement elle peut donner naissance à un enfant prématuré. 00:32:05-5

00:32:05-5 Interviewer: Est-ce qu'il y a quelque chose d'autre à ajouter ? 00:32:11-6

00:32:11-6 Personne 6: Il y avait un cas chez nous où une femme a donné naissance à un prématuré mais cela était dû au fait que son mari soit décédé, dans la belle famille ça n'allait pas du tout, on ne l'aimait pas, on ne lui donnait pas à manger. Cette dernière avait même commencé ses CPN, mais la deuxième fois qu'elle est revenue, sa tension n'était pas descendue et ça continuait de monter. Nous lui avons demandé si elle suivait vraiment son traitement, et c'est en ce moment qu'elle a dit que depuis le décès de son mari, étant en belle famille, ce n'est pas simple. Nous avons prescrit les médicaments sur l'ordonnance qu'elle devait aller payer et au cas où elle n'arrivait pas à payer, elle pouvait toujours revenir. Nous n'avons même pas eu ce temps-là, cette dernière n'est plus revenue, donc elle a accouché d'un mort-né et ce n'était même pas prématuré. 00:33:13-3

00:33:13-3 Interviewer: Elle était à combien de mois de grossesse ? 00:33:14-8

00:33:14-8 Personne 6: Ah, est-ce que je me rappelle encore, en tout cas ça ne dépasse pas le deuxième trimestre, ça devrait être autour de 5 mois. Le jour qu'elle a eu les médicaments, elle a fait une seule prise et c'est ce jour-là qu'elle est venue expulser. 00:33:50-0

00:33:50-0 Interviewer: Est-ce qu'il y a quelque chose d'autre à ajouter ? 00:33:55-7

00:33:55-7 Personne 6: Il y a aussi les cas des personnes déplacées internes, quand elles viennent, le mari est soit décédé, soit absent. Elle a eu la chance de fuir pour venir chez ses parents, il n'y a personne pour s'occuper d'elle. Moi, je suis obligée d'appeler la famille pour les sensibiliser. Parce que sans le soutien, la grossesse de la femme va être très difficile. Je sensibilise beaucoup sur l'amour dans le foyer, le soutien, etc. 00:35:13-7

**00:22:19-9 Interviewer:** Considering everything discussed, what do you propose to improve the management of premature births at your health center?

**00:22:35-0 Person 2:** For prenatal care, we emphasize early consultation and awareness of danger signs during antenatal care (ANC). Postnatally, providing incubators in larger health facilities could significantly reduce neonatal death risks.

**00:22:46-0 Interviewer:** Before or after birth, what suggestions do you have?

**00:22:39-1 Person 2:** Regarding postnatal care, if we had incubators, even basic ones, at our health center, it would help immensely. Currently, we have to transfer cases to CHURSS for lack of facilities.

**00:23:50-1 Person 5:** Training staff on immediate postnatal care is crucial. Knowing what to do for the newborn while waiting for evacuation can save lives. Training in prematurity care is essential for our staff.

**00:24:15-8 Person 2:** Adding heat lamps at our health center could be beneficial, especially when we don't have access to incubators.

**00:24:29-9 Interviewer:** Can you elaborate on the danger signs you mentioned?

**00:24:41-6 Person 2:** The danger signs include pelvic pain, bleeding, fever, severe headaches, and dizziness.

**00:25:05-9 Person 5:** There's also the psychological and social aspect. Addressing social issues and mediating conflicts within families can have a positive impact on preventing prematurity.

**00:25:26-1 Interviewer:** Regarding the training of health workers, how can we maintain quality care when trained staff are reassigned?

**00:25:56-2 Person 5:** Continuity is key. If trained staff leave, the habits and protocols they've established can be passed on to new staff. It's like on-site training.

**00:26:40-0 Interviewer:** You mentioned socio-cultural constraints as factors in premature birth. Can you explain?

**00:26:52-9 Person 6:** Socio-cultural issues, like domestic disputes or denial of paternity, can stress the mother and lead to premature births. We use invitation notes to involve husbands in consultations, aiming to resolve conflicts and promote unity, as discord negatively impacts the pregnancy.

**00:29:54-4 Person 7:** We also encounter cases of physical violence and aggression. Counseling the couple, especially the husband, to be gentle and supportive is crucial as such stress can trigger premature births.

**00:30:32-6 Interviewer:** Thank you for your insights and suggestions on improving premature birth management at your health center.

**Person 5 (00:30:46-9):** "Regarding societal perceptions, I can't comment much. However, I've noticed an increasing awareness about the care of premature babies. People now understand that such babies shouldn’t be exposed outdoors and need to be protected. There’s growing knowledge about the special needs of premature babies, like avoiding baths or keeping them in a bedroom. When we give advice, especially to a mother-in-law, they tend to follow it meticulously."

**Person 3 (00:31:26-3):** "Adding to that, for the mother, issues like hypertension can seriously affect pregnancy. If hypertension isn’t detected early, it can lead to premature birth."

**Interviewer (00:32:05-5):** "Is there anything else you'd like to add?"

**Person 6 (00:32:11-6):** "We had a case where a woman’s premature birth was linked to her husband's death. She faced neglect and lack of care from her in-laws, leading to unmanaged hypertension. Despite starting antenatal care, her condition worsened due to the stress and lack of support. She couldn’t afford her medication and eventually delivered a stillborn, before the end of her second trimester."

**Interviewer (00:33:13-3):** "How many months pregnant was she?"

**Person 6 (00:33:14-8):** "If I recall correctly, it was around 5 months. She had only taken one dose of her medication before going into labor."

**Interviewer (00:33:50-0):** "Anything more to add?"

**Person 6 (00:33:55-7):** "We also see cases involving internally displaced women. Often, their husbands are dead or absent, and they lack family support. In these situations, I make calls to their families to raise awareness. Without support, these women's pregnancies become extremely challenging. I focus a lot on raising awareness about love and support in the family."

### Major of Colma1 health and social promotion center

[00:17:07-9](http://localhost:2300/file=C:/Users/USER/Desktop/ENTRETIENS%20PREMATURITE/CSPS-COLMA1/MAJOR%20CSPS%20COLMA1.MP3time=1027900) Interviewer: Maintenant, est-ce que par rapport à tout ce que vous avez à dire, comment est-ce que on peut améliorer la prise en charge des naissances prématurées ? [00:17:16-1](http://localhost:2300/file=C:/Users/USER/Desktop/ENTRETIENS%20PREMATURITE/CSPS-COLMA1/MAJOR%20CSPS%20COLMA1.MP3time=1036100)

[00:17:16-1](http://localhost:2300/file=C:/Users/USER/Desktop/ENTRETIENS%20PREMATURITE/CSPS-COLMA1/MAJOR%20CSPS%20COLMA1.MP3time=1036100) Person 1: Ah OK bien donc il s'agit donc d'agir sur ces quelques causes que je viens de citer ici. Voilà la prévention du PALU. Voilà, c'est vrai que déjà et au cours de la CPN, si vraiment la CPN est précoce,il y a des dispositions que nous prenons déjà donc au niveau de la CPN, on les donne des moustiquaires, on les donne donc la SP. Voilà pour la prévention du paludisme.Ensuite on les invite à venir au centre de santé devant tout problème de santé, devant tout danger, on les enseigne tout ça, mais nous restons dans un milieu encore,je ne vais pas dire analphabète ou le niveau de connaissance n'est pas encore à un niveau qu'on souhaite. Donc si bien qu'il y a des problèmes qui vont se poser en communauté et les gens ont resté avec et traîner, traîner, traîner jusqu'à la dernière minute jusqu'au moment ou on ne peut rien faire ils vont arriver ici. [00:18:03-2](http://localhost:2300/file=C:/Users/USER/Desktop/ENTRETIENS%20PREMATURITE/CSPS-COLMA1/MAJOR%20CSPS%20COLMA1.MP3time=1083200)

[00:18:03-2](http://localhost:2300/file=C:/Users/USER/Desktop/ENTRETIENS%20PREMATURITE/CSPS-COLMA1/MAJOR%20CSPS%20COLMA1.MP3time=1083200) Interviewer: Voilà maintenant au sein même de votre centre de santé puisque vous avez évoqué tantôt le manque de plateau technique, [00:18:08-9](http://localhost:2300/file=C:/Users/USER/Desktop/ENTRETIENS%20PREMATURITE/CSPS-COLMA1/MAJOR%20CSPS%20COLMA1.MP3time=1088900)

[00:18:08-9](http://localhost:2300/file=C:/Users/USER/Desktop/ENTRETIENS%20PREMATURITE/CSPS-COLMA1/MAJOR%20CSPS%20COLMA1.MP3time=1088900) Person 1: oui. [00:18:09-7](http://localhost:2300/file=C:/Users/USER/Desktop/ENTRETIENS%20PREMATURITE/CSPS-COLMA1/MAJOR%20CSPS%20COLMA1.MP3time=1089700)

[00:18:09-7](http://localhost:2300/file=C:/Users/USER/Desktop/ENTRETIENS%20PREMATURITE/CSPS-COLMA1/MAJOR%20CSPS%20COLMA1.MP3time=1089700) Interviewer: Pour déjà une première offre de soins et de services est ce que vous pensez que l'apport du plateau technique des médicaments, une formation du personnel, peut aussi vous aider déjà à ce niveau de vous ameliorer? [00:18:24-8](http://localhost:2300/file=C:/Users/USER/Desktop/ENTRETIENS%20PREMATURITE/CSPS-COLMA1/MAJOR%20CSPS%20COLMA1.MP3time=1104800)

[00:18:24-8](http://localhost:2300/file=C:/Users/USER/Desktop/ENTRETIENS%20PREMATURITE/CSPS-COLMA1/MAJOR%20CSPS%20COLMA1.MP3time=1104800) Person 1: Oui, oui, oui, oui, oui, oui, ça peut améliorer le plateau si effectivement la formation du personnel, ça peut renforcer les capacités, ça peut les permettre donc de percevoir certaines choses et qui peuvent exposer donc à la prématurité. Voilà, parce que essentiellement dans notre équipe ici le maximum là ce sont des accoucheuses auxiliaires.je ne dirai pas que le niveau n'est pas très élevé, mais quand même ces gens ont besoin donc d'être mis à niveau. c'est vrai qu'en interne ici nous faisons donc des staffs et des formations en continu, mais si un plus vient s'ajouter, je crois que ça peut renforcer encore les capacités et au-delà de ça, il y a également les moyens. Les consommables peuvent aider également.Parce qu'effectivement, toujours nous, on n'a pas, ce qu'on appelle de la SP ici, la sulfatdoxine puri étamine qu'on utilise donc pour la prévention du paludisme, ce n'est pas toujours disponible dans le système normal d'approvisionnement, il y a des moments c'est disponible mais il y a des moments, ce n'est pas disponible donc cela fait que certaines femmes peuvent partir parce queles sages femmes vont prescrire mais les gens ne vont pas payer, voilà, les gens peuvent repartir à la maison comme ça, sans prendre et ça peut exposer la femme au paludisme et exposer donc à la prématurité. Donc s'il y a un renforcement donc aussi bien au niveau des médicaments et consommables qu'en capacité donc formation du personnel, je crois que ça peut renforcer un tant soit peu ce que nous faisons. [00:19:51-4](http://localhost:2300/file=C:/Users/USER/Desktop/ENTRETIENS%20PREMATURITE/CSPS-COLMA1/MAJOR%20CSPS%20COLMA1.MP3time=1191400)

[00:19:51-4](http://localhost:2300/file=C:/Users/USER/Desktop/ENTRETIENS%20PREMATURITE/CSPS-COLMA1/MAJOR%20CSPS%20COLMA1.MP3time=1191400) Interviewer: Par rapport à la mobilité des agents de santé, que ce que vous proposez pour que le le centre de santé n'en sort pas impacté négativement si à par exemple, des agents de santé ayant beneficier d'une formation par rapport à la prematurité partent ailleurs? [00:20:13-1](http://localhost:2300/file=C:/Users/USER/Desktop/ENTRETIENS%20PREMATURITE/CSPS-COLMA1/MAJOR%20CSPS%20COLMA1.MP3time=1213100)

[00:20:13-1](http://localhost:2300/file=C:/Users/USER/Desktop/ENTRETIENS%20PREMATURITE/CSPS-COLMA1/MAJOR%20CSPS%20COLMA1.MP3time=1213100) Person 1: OK, donc la mobilité en fait en ville, c'est vrai que ça bouge, mais pas trop, en tout cas, à ma connaissance, de mes 3 ans ici parce que j'etais en milieu rural, maintenant de mes 3 ans ici Il y a des gens qui qui sont partis à la retraite, là ça c'est normal. Voilà, ils sont partis à la retraite, et il y a des gens qui ont été affectés, mais ça aussi ce n'est pas beaucoup. Donc vous prenez un effectif de 19 agents, il y a trois seulement qui ont été affectés,On ne peut pas dire que c'est seulement, mais bon, les détroits qui ont qui qui sont allées dans d'autres structures, voilà qui sont dans d'autres structures. Hormis ça, les autres sont là, voilà ceux qui sont plus là sont à la retraite aussi. Voilà, je dirais que en tout cas, la mobilité n'est pas beaucoup ressentie en ville mais c'est frequent en milieu rural. même si ici aussi on sait que souvent ça quitte dans un endroit vers un autre, mais nous recevons beaucoup plus de nouveaux qui arrivent aussi. [00:21:19-0](http://localhost:2300/file=C:/Users/USER/Desktop/ENTRETIENS%20PREMATURITE/CSPS-COLMA1/MAJOR%20CSPS%20COLMA1.MP3time=1279000)

[00:21:19-0](http://localhost:2300/file=C:/Users/USER/Desktop/ENTRETIENS%20PREMATURITE/CSPS-COLMA1/MAJOR%20CSPS%20COLMA1.MP3time=1279000) Interviewer: Peut-être qu'ils ne sont pas à jour par rapport à la formation? [00:21:20-7](http://localhost:2300/file=C:/Users/USER/Desktop/ENTRETIENS%20PREMATURITE/CSPS-COLMA1/MAJOR%20CSPS%20COLMA1.MP3time=1280700)

[00:21:20-7](http://localhost:2300/file=C:/Users/USER/Desktop/ENTRETIENS%20PREMATURITE/CSPS-COLMA1/MAJOR%20CSPS%20COLMA1.MP3time=1280700) Person 1: Voilà la formation , c'est ça. [00:21:23-0](http://localhost:2300/file=C:/Users/USER/Desktop/ENTRETIENS%20PREMATURITE/CSPS-COLMA1/MAJOR%20CSPS%20COLMA1.MP3time=1283000)

[00:21:23-0](http://localhost:2300/file=C:/Users/USER/Desktop/ENTRETIENS%20PREMATURITE/CSPS-COLMA1/MAJOR%20CSPS%20COLMA1.MP3time=1283000) Interviewer: dans ce cas, qu'est ce que vous proposez ?[00:21:24-4](http://localhost:2300/file=C:/Users/USER/Desktop/ENTRETIENS%20PREMATURITE/CSPS-COLMA1/MAJOR%20CSPS%20COLMA1.MP3time=1284400)

[00:21:24-4](http://localhost:2300/file=C:/Users/USER/Desktop/ENTRETIENS%20PREMATURITE/CSPS-COLMA1/MAJOR%20CSPS%20COLMA1.MP3time=1284400)Person 1: bon, ici, c'est en interne d'abord ce sont ceux qui sont déjà briefé par rapport à certaines choses. Voilà donc en interne on se fait donc ce qu'on appelle dans les formations continues en interne. Voilà ce que nous faisons. Mais ce n'est pas toujours suffisant. Voilà, lorsque l'agent lui-même participe bien sûr, donc pleinement à la chose. Je crois que les résultats donc est plus ressenti. [00:21:49-0](http://localhost:2300/file=C:/Users/USER/Desktop/ENTRETIENS%20PREMATURITE/CSPS-COLMA1/MAJOR%20CSPS%20COLMA1.MP3time=1309000)

[00:21:49-0](http://localhost:2300/file=C:/Users/USER/Desktop/ENTRETIENS%20PREMATURITE/CSPS-COLMA1/MAJOR%20CSPS%20COLMA1.MP3time=1309000) Interviewer: Maintenant un autre facteur par rapport à à la prise en charge que vous exercez sur la naissance prématurée au niveau des mères, les enfants prématurés, oui. Est-ce que vous pensez que les pesanteurs socioculturels peuvent être un facteur qui freine un peu la mère dans son milieu naturel ? [00:22:15-2](http://localhost:2300/file=C:/Users/USER/Desktop/ENTRETIENS%20PREMATURITE/CSPS-COLMA1/MAJOR%20CSPS%20COLMA1.MP3time=1335200)

[00:22:15-2](http://localhost:2300/file=C:/Users/USER/Desktop/ENTRETIENS%20PREMATURITE/CSPS-COLMA1/MAJOR%20CSPS%20COLMA1.MP3time=1335200) Person 1: oui, oui, oui, oui. C'est un élément très important, les pesanteurs socioculturels, parce que d'une certaine manière, oui, parce que, unel mère qui est dans un milieu, d'abord l'entretien de la grossesse c'est d'abord psychologique. Voilà une mère qui arrive à tenir une grossesse du début à la fin,et ça témoigne du fait que l'esprit est tranquille et que vraiment, en tout cas elle arrive à tenir bien la grossesse. Voilà. Mais lorsque dans son milieu, là où elle a assise, là elle a installé dans la famille là où il se trouve, si y a beaucoup de perturbations, s'il y a beaucoup de facteurs qui viennent déranger donc sa tranquillité il va de soit que ça peut impacter sur la grossesse négativement et aboutir donc à ce qu'on appelle donc les prématurités également. Voilà, je crois que ça tient compte. [00:23:15-9](http://localhost:2300/file=C:/Users/USER/Desktop/ENTRETIENS%20PREMATURITE/CSPS-COLMA1/MAJOR%20CSPS%20COLMA1.MP3time=1395900)

00:17:07-9 Interviewer: Maintenant, est-ce que par rapport à tout ce que vous avez à dire, comment est-ce que on peut améliorer la prise en charge des naissances prématurées ? 00:17:16-1

00:17:16-1 Person 1: Ah OK bien donc il s'agit donc d'agir sur ces quelques causes que je viens de citer ici. Voilà la prévention du PALU. Voilà, c'est vrai que déjà et au cours de la CPN, si vraiment la CPN est précoce, il y a des dispositions que nous prenons déjà donc au niveau de la CPN, on les donne des moustiquaires, on les donne donc la SP. Voilà pour la prévention du paludisme. Ensuite on les invite à venir au centre de santé devant tout problème de santé, devant tout danger, on les enseigne tout ça, mais nous restons dans un milieu encore, je ne vais pas dire analphabète ou le niveau de connaissance n'est pas encore à un niveau qu'on souhaite. Donc si bien qu'il y a des problèmes qui vont se poser en communauté et les gens ont resté avec et traîner, traîner, traîner jusqu'à la dernière minute jusqu'au moment où on ne peut rien faire ils vont arriver ici. 00:18:03-2

00:18:03-2 Interviewer: Voilà maintenant au sein même de votre centre de santé puisque vous avez évoqué tantôt le manque de plateau technique, 00:18:08-9

00:18:08-9 Person 1: Oui. 00:18:09-7

00:18:09-7 Interviewer: Pour déjà une première offre de soins et de services est-ce que vous pensez que l'apport du plateau technique, des médicaments, une formation du personnel, peut aussi vous aider déjà à ce niveau de vous améliorer ? 00:18:24-8

00:18:24-8 Person 1: Oui, oui, oui, oui, oui, oui, ça peut améliorer le plateau si effectivement la formation du personnel, ça peut renforcer les capacités, ça peut les permettre donc de percevoir certaines choses et qui peuvent exposer donc à la prématurité. Voilà, parce qu’essentiellement dans notre équipe ici le maximum là ce sont des accoucheuses auxiliaires. Je ne dirai pas que le niveau n'est pas très élevé, mais quand même ces gens ont besoin donc d'être mis à niveau. C'est vrai qu'en interne ici nous faisons donc des staffs et des formations en continu, mais si un plus vient s'ajouter, je crois que ça peut renforcer encore les capacités et au-delà de ça, il y a également les moyens. Les consommables peuvent aider également. Parce qu'effectivement, toujours nous, on n'a pas, ce qu'on appelle de la SP ici, la sulfadoxine-pyriméthamine qu'on utilise donc pour la prévention du paludisme, ce n'est pas toujours disponible dans le système normal d'approvisionnement, il y a des moments c'est disponible mais il y a des moments, ce n'est pas disponible donc cela fait que certaines femmes peuvent partir parce que les sage-femmes vont prescrire mais les gens ne vont pas payer, voilà, les gens peuvent repartir à la maison comme ça, sans prendre et ça peut exposer la femme au paludisme et exposer donc à la prématurité. Donc s'il y a un renforcement donc aussi bien au niveau des médicaments et consommables qu'en capacité donc formation du personnel, je crois que ça peut renforcer un tant soit peu ce que nous faisons. 00:19:51-4

00:19:51-4 Interviewer: Par rapport à la mobilité des agents de santé, que ce que vous proposez pour que le centre de santé n'en sort pas impacté négativement si à par exemple, des agents de santé ayant bénéficié d'une formation par rapport à la prématurité partent ailleurs ? 00:20:13-1

00:20:13-1 Person 1: OK, donc la mobilité en fait en ville, c'est vrai que ça bouge, mais pas trop, en tout cas, à ma connaissance, de mes 3 ans ici parce que j'étais en milieu rural, maintenant de mes 3 ans ici. Il y a des gens qui qui sont partis à la retraite, là ça c'est normal. Voilà, ils sont partis à la retraite, et il y a des gens qui ont été affectés, mais ça aussi ce n'est pas beaucoup. Donc vous prenez un effectif de 19 agents, il y a trois seulement qui ont été affectés, On ne peut pas dire que c'est seulement, mais bon, les départs qui ont qui qui sont allées dans d'autres structures, voilà qui sont dans d'autres structures. Hormis ça, les autres sont là, voilà ceux qui sont plus là sont à la retraite aussi. Voilà, je dirais que en tout cas, la mobilité n'est pas beaucoup ressentie en ville mais c'est fréquent en milieu rural. Même si ici aussi on sait que souvent ça quitte dans un endroit vers un autre, mais nous recevons beaucoup plus de nouveaux qui arrivent aussi. 00:21:19-0

00:21:19-0 Interviewer: Peut-être qu'ils ne sont pas à jour par rapport à la formation ? 00:21:20-7

00:21:20-7 Person 1: Voilà la formation, c'est ça. 00:21:23-0

00:21:23-0 Interviewer: Dans ce cas, qu'est-ce que vous proposez ? 00:21:24-4

00:21:24-4 Person 1: Bon, ici, c'est en interne d'abord ce sont ceux qui sont déjà briefés par rapport à certaines choses. Voilà donc en interne on se fait donc ce qu'on appelle des formations continues en interne. Voilà ce que nous faisons. Mais ce n'est pas toujours suffisant. Voilà, lorsque l'agent lui-même participe bien sûr, donc pleinement à la chose. Je crois que les résultats donc sont plus ressentis. 00:21:49-0

00:21:49-0 Interviewer: Maintenant, un autre facteur par rapport à la prise en charge que vous exercez sur la naissance prématurée au niveau des mères, les enfants prématurés, oui. Est-ce que vous pensez que les pesanteurs socioculturelles peuvent être un facteur qui freine un peu la mère dans son milieu naturel ? 00:22:15-2

00:22:15-2 Personne 1: Oui, oui, oui, oui. C'est un élément très important, les pesanteurs socioculturelles, parce que d'une certaine manière, oui. Parce que, une mère qui est dans un milieu, d'abord, l'entretien de la grossesse, c'est d'abord psychologique. Voilà une mère qui arrive à tenir une grossesse du début à la fin, et ça témoigne du fait que l'esprit est tranquille et que vraiment, en tout cas, elle arrive à bien tenir la grossesse. Voilà. Mais lorsque dans son milieu, là où elle est assise, là où elle est installée dans la famille, là où elle se trouve, s'il y a beaucoup de perturbations, s'il y a beaucoup de facteurs qui viennent déranger donc sa tranquillité, il va de soi que ça peut impacter sur la grossesse négativement et aboutir donc à ce qu'on appelle les prématurités également. Voilà, je crois que ça tient compte. 00:23:15-9

**00:17:07-9 Interviewer:** Based on everything you've said, how can we improve the management of premature births?

**00:17:16-1 Person 1:** We need to focus on the causes of prematurity, like malaria prevention during prenatal care (CPN). We distribute mosquito nets and sulfadoxine-pyrimethamine (SP) for malaria prevention. Educating women to seek immediate care for any health issue is crucial. However, our community still faces challenges with literacy and awareness, leading to delayed healthcare seeking until it's often too late.

**00:18:03-2 Interviewer:** What improvements are needed within your health center, especially considering the lack of technical support?

**00:18:08-9 Person 1:** Yes, improvements are necessary. Enhancing staff training can strengthen our capacity to identify and manage prematurity risks. Our team mainly consists of auxiliary midwives who could benefit from advanced training. Also, ensuring a steady supply of essential medicines and consumables, like SP for malaria prevention, is critical. If we can strengthen our staff’s capabilities and ensure the availability of medicines, it would greatly improve our care for premature babies.

**00:19:51-4 Interviewer:** Regarding the mobility of health workers, how can we ensure the health center isn't negatively impacted, especially when trained workers move to other locations?

**00:20:13-1 Person 1:** Mobility is less felt in the city compared to rural areas. In my three years here, only a few staff have been reassigned or retired. We do get new staff, though they might not be up to date with training.

**00:21:19-0 Interviewer:** So, what would you suggest to address this issue?

**00:21:24-4 Person 1:** We focus on internal continuous training. It’s crucial for maintaining the quality of care, but it's not always sufficient. Active participation in training leads to better results. We try to keep our staff updated and well-informed on managing premature births.

**Interviewer (00:21:49-0):** "Regarding your management of premature births, do you think that socio-cultural constraints in a mother's natural environment can be a factor that hinders her during pregnancy?"

**Person 1 (00:22:15-2):** "Absolutely, socio-cultural constraints are a significant element. The psychological state of a mother is crucial for maintaining a pregnancy. A mother who carries her pregnancy to term typically does so in a peaceful and supportive environment, indicating her mental well-being. However, if she is in a disruptive environment with many disturbances that affect her peace of mind, it can negatively impact her pregnancy. Such stressors can lead to complications, including premature birth. I believe these factors are very important to consider.

### Maternity manager of Colma1 health and social promotion center

[00:29:47-6](http://localhost:2300/file=C:/Users/USER/Desktop/ENTRETIENS%20PREMATURITE/CSPS-COLMA1/RESPONSABLE%20MATERNITE-COLMA1.mp3time=1787600) Interviewer: Ok. Maintenant, au vu de tout ça, qu'est- ce que vous avez comme solution pour améliorer la prise en charge des naissances prématurés dans votre centre de santé ? Qu'est- ce que vous proposez ? [00:29:57-1](http://localhost:2300/file=C:/Users/USER/Desktop/ENTRETIENS%20PREMATURITE/CSPS-COLMA1/RESPONSABLE%20MATERNITE-COLMA1.mp3time=1797100)

[00:29:57-1](http://localhost:2300/file=C:/Users/USER/Desktop/ENTRETIENS%20PREMATURITE/CSPS-COLMA1/RESPONSABLE%20MATERNITE-COLMA1.mp3time=1797100) Person 1: Pour améliorer, c'est tout simplement, d'abord chez nous, puisque chaque fois vous demandez chez nous, c'est de mettre l'accent sur vraiment, je vais dire quoi, le counseling individuel. Ça veut dire la CPN ne doit pas être comme la CPN de routine de depuis nos mamans. Ça doit être une CPN recentrée. Connaître les problèmes de la femme, les décélés et les prendre en charge. Réellement, ce qui doit être fait, il faut le faire tôt. Même l'âge de la grossesse, comme je vous dis, quand, par exemple, c'est connu tôt, on réfère à temps pour la prise en charge. Et aussi, on va essayer de décèler les pathologies qui peuvent faire que la femme fasse un accouchement prématuré ou bien un avortement tardif. Par exemple, la femme est venue, la première des choses, l'infection urinaire, il faut vraiment demander et puis traiter. Le paludisme, il y a la SP qu'on donne ? Pour nous, je vous dis, chez nous, la prise est supervisée. Avant, quand j'étais en brousse (milieu rural), les femmes prenaient de manière supervisée. En ville, les femmes pensent qu'elles sont sophistiquées. C'est des femmes de quoi quoi quoi. Moi, j'ai remarqué, on avait beaucoup d'avortements, beaucoup d'accouchements prématurés.En ce temps, je n'étais même pas responsable, mais j'ai dit, il faut que la prise soit supervisée. Une femme qui refuse, qu'elle n'a pas mangé, elle n'a qu'à aller manger et revenir. Mais depuis qu'on a commencé , réellement, ça va. Mais le problème est que, s'il n'y a pas de SP au centre de santé ici, quand tu donnes l'ordonnance aux femmes, elles ne payent pas. Et quand elles ne payent pas, par exemple, je peux ne pas être en CPN, mais en salle seulement, quand je vois une femme avec TDR positif, je sais qu'il n'y a pas de SP en gratuité. Donc, ça veut dire que tout ce qui concerne vraiment la femme enceinte, il faut disponibiliser. Ne pas oublier l' échographie aussi. Voilà, les examens complémentaires. Il faut vraiment que tout soit honnête. Aussi, quand la femme vient, on n'a pas dit de faire un interrogatoire policier, mais il faut essayer de comprendre, être en amie avec cette femme,elle va se confier,elle va vous dire en tout cas, c'est quel problème socio-culturel elle a. Et là, vous allez essayer de prendre en charge. Si vous ne le pouvez pas, actuellement, on a des sociologues qui sont là,on peut vraiment diriger ces femmes-là vers ces gens. Et puis, une fois seulement qu'une femme a déjà fait un accouchement prématuré ou un avortement tardif, cette femme, il faut vraiment référer cette femme-là chez un gynécologue qui verra est-ce que cette femme a besoin d'un cerclage ou pas. Parce que souvent, il y a des femmes qui sont là. L'introgatoire n'est pas correct. Elle avait déjà fait. C'est quand elle est sur la elle est venue pour expulser son enfant prématuré et que vous ne pouvez rien dire. < Non, non, non cela fait la deuxième fois maintenant >. en ce moment,toi, en tant qu'agent de santé, c'est toi qui n'a pas fait ton travail. Parce qu'il y a des gens qui pensent que si je dis ça, peut- être on va me faire comme ça, on va me réprimander, il y a tout ça. Il faut que les l'interrogatoires soient capitales. Il faut que la CPN soit une CPN recentrée. On n'a pas besoin de prendre 50 femmes, si vous êtes deux, si vous prénez 20 femmes, 15 femmes et que le travail est correct, même quelqu'en soit ce que les gens vont dire « C'est Dieu qui fait ce que vous avez fait. Et vous allez voir, même si le problème vient là, ça ne porte pas sur vous. [00:33:43-9](http://localhost:2300/file=C:/Users/USER/Desktop/ENTRETIENS%20PREMATURITE/CSPS-COLMA1/RESPONSABLE%20MATERNITE-COLMA1.mp3time=2023900)

[00:33:43-9](http://localhost:2300/file=C:/Users/USER/Desktop/ENTRETIENS%20PREMATURITE/CSPS-COLMA1/RESPONSABLE%20MATERNITE-COLMA1.mp3time=2023900) Interviewer: Et par rapport à la formation du personnel de santé aussi ? [00:33:49-3](http://localhost:2300/file=C:/Users/USER/Desktop/ENTRETIENS%20PREMATURITE/CSPS-COLMA1/RESPONSABLE%20MATERNITE-COLMA1.mp3time=2029300)

[00:33:49-3](http://localhost:2300/file=C:/Users/USER/Desktop/ENTRETIENS%20PREMATURITE/CSPS-COLMA1/RESPONSABLE%20MATERNITE-COLMA1.mp3time=2029300) Person 1: Oui. Vous voyez, quand, par exemple, le questionnaire est là, vous voyez souvent, je suis là, je cherche. C'est pour dire que quand les gens sont sur le terrain, il y a des gens qui refusent vraiment de s'auto former. Oui, ou bien ils vont s'auto former sur autre chose, mais pas sur la prématurité. Mais si, par exemple, on a une formation par rapport à la prématurité, ça va nous aider. Et puis, avec des recyclages à l'appui, et puis, des supervisions. Par exemple, le revue documentaire, par exemple, cet enfant vous avez fait un accouchement la dernière fois, l'enfant, c'est 2 200, selon vous, c'était un enfant prématuré, c'était un enfant hippotrophe ? C'est comment ? Quelle a été la prise en charge ? Ça peut vraiment aider des gens à s'améliorer. [00:34:50-1](http://localhost:2300/file=C:/Users/USER/Desktop/ENTRETIENS%20PREMATURITE/CSPS-COLMA1/RESPONSABLE%20MATERNITE-COLMA1.mp3time=2090100)

[00:34:50-1](http://localhost:2300/file=C:/Users/USER/Desktop/ENTRETIENS%20PREMATURITE/CSPS-COLMA1/RESPONSABLE%20MATERNITE-COLMA1.mp3time=2090100) Interviewer: Et par rapport à l'équipement même du centre de santé? [00:34:52-8](http://localhost:2300/file=C:/Users/USER/Desktop/ENTRETIENS%20PREMATURITE/CSPS-COLMA1/RESPONSABLE%20MATERNITE-COLMA1.mp3time=2092800)

00:34:52-8 Person 1: Je vous ai dit que l'infection peut être... Souvent, il y a des femmes même qui vont prendre des infections ici. Vous voyez les tables. Si vous avez fait un tour, comme vos gens sont avec nous, il y a des femmes qui vont venir. Elles vont même pas étaler leurs pagnes, elles vont se coucher. Vous voyez tout de suite, il y a une femme qui a déposé une bouillard là, une autre personne a pris le même bouillard. Il y a beaucoup de ces cas. Mais moi, je me demande, est-ce qu'on pourra corriger ? c'est pour dire que l'équipement, c'est forcé. il y a des années de cela, vous allez voir, normalement, quand on est en salle, je n'ai pas porter des bonnets, bien vrai que je suis au service. Je dois tout porter. Je dois pas toucher à un nouveau né . Je dois porter des bavettes. L'équipement doit être au total. Les blouses doivent même être lavé, repassé totalement. Les bottes, tout doit être propre. Ça veut dire nos salles même, de passage, je remercie dembalo Parce que quand on à repris à travailler dans notre salle, c'était une grande fierté pour moi. On a eu une formation et j'ai tout fait pour que les docteurs viennent voir comment c'était propre et quand les gens viennent, il n y avait pas grand-chose, on n'avait même pas amener nos affiches, mais le fait que c'est propre déjà, ça menait,c'était bien. Même d'autres collègues voyaient et disaient, « Je vous là c'est bien, je vous là c'est bien » . On vous remercie (projet dembalo) de passage, vraiment. Et puis, merci aussi pour les tables d'accouchement. On avait besoin de ça. On a besoin d'équipements. On a besoin même, il y a beaucoup de choses, même la salle d'accouchement doit vraiment être adéquate. Parce que vous voyez, quand il y a la réanimation, peut- être si vous voulez, on va faire un tour, j'ai été obligée de prendre la lampe baladeuse de la salle PEF pour envoyer pour la réanimation des nouveaux nés. Pour qu'au moins cette lampe- là chauffe un peu. [00:37:07-4](http://localhost:2300/file=C:/Users/USER/Desktop/ENTRETIENS%20PREMATURITE/CSPS-COLMA1/RESPONSABLE%20MATERNITE-COLMA1.mp3time=2227400)

[00:37:07-4](http://localhost:2300/file=C:/Users/USER/Desktop/ENTRETIENS%20PREMATURITE/CSPS-COLMA1/RESPONSABLE%20MATERNITE-COLMA1.mp3time=2227400) Interviewer: Maintenant, il y a un phenomène qui est beaucoup plus fréquent en milieu médical, le mouvement des agents.Pensez-vous que cela peut être un facteur handicapant pour votre centre de santé au cas ou ceux qui ont eu une formation spécifique sur la prématurité vont ailleurs? Comment est- ce qu'on peut palier à cela ? [00:37:29-3](http://localhost:2300/file=C:/Users/USER/Desktop/ENTRETIENS%20PREMATURITE/CSPS-COLMA1/RESPONSABLE%20MATERNITE-COLMA1.mp3time=2249300)

[00:37:29-3](http://localhost:2300/file=C:/Users/USER/Desktop/ENTRETIENS%20PREMATURITE/CSPS-COLMA1/RESPONSABLE%20MATERNITE-COLMA1.mp3time=2249300) Person 1: Par exemple, il y a des gens, je prends un exemple terre à terre, on a PATFINDER a former tous les agents qui étaient là, même les accoucheuses, je veux dire tout agent à la maternité sur la planification familiale. Et d'autres sont partis, d'autres ont eu des concours, d'autres sont partis. Moi, je me dis si on veut former les agents de santé par rapport à la prématurité, je pense que c'est mieux de former de façon nationale. Oui, de façon nationale et puis essayer de faire des recyclages chaque fois. Les gens qui sont sur place, parce qu'il y a des gens aussi qui quittent l'école pour venir. Quand les gens quittent l'école pour venir et que ceux qui sont sur place là forment vraiment ceux qui viennent de l'école et que ça soit vraiment, je vais dire quoi, documenté. Et puis essayer de faire des formations périodiques. Peut- être, on va dire quoi ? Chaque an, pour les nouveaux qui viennent, voilà. Malgré le recyclage des aînés qui sont sur le terrain. Je pense que ça peut se faire. Parce qu'il y a des gens même qui vont bénéficier de la formation, par exemple, à Ouaga, mais ils sont affectés ici.Lorsqu'ils vont venir et par exemple, on demande « Est-ce que vous avez été formée sur la prématurité ? » « Non, je n'ai pas été formée. » Parce que les gens pensent que la formation, c'est de l'argent seulement. Mais si ça a été fait de façon nationale, vous voyez que personne ne peut vous dire que « Non, je n'ai pas été formée. » Si tu es sortie, ça vaut deux ou trois ans. Parce qu'il y a des gens qui veulent seulement les perdièms. Et quand tu es formée aussi, il faut que réellement on voit ce que ça t'a apporté. Parce que depuis que moi- même, j'ai quitté l'école, à l'école j'ai eu la formation mais le district n'a jamais formé par rapport à ça, à la prématurité. [00:39:52-0](http://localhost:2300/file=C:/Users/USER/Desktop/ENTRETIENS%20PREMATURITE/CSPS-COLMA1/RESPONSABLE%20MATERNITE-COLMA1.mp3time=2392000)

00:29:47-6 Interviewer: Ok. Maintenant, au vu de tout ça, qu'est-ce que vous avez comme solution pour améliorer la prise en charge des naissances prématurées dans votre centre de santé ? Qu'est-ce que vous proposez ? 00:29:57-1

00:29:57-1 Person 1: Pour améliorer, c'est tout simplement, d'abord chez nous, puisque chaque fois vous demandez chez nous, c'est de mettre l'accent sur vraiment, je vais dire quoi, le counseling individuel. Ça veut dire la CPN ne doit pas être comme la CPN de routine de depuis nos mamans. Ça doit être une CPN recentrée. Connaître les problèmes de la femme, les déceler et les prendre en charge. Réellement, ce qui doit être fait, il faut le faire tôt. Même l'âge de la grossesse, comme je vous dis, quand, par exemple, c'est connu tôt, on réfère à temps pour la prise en charge. Et aussi, on va essayer de déceler les pathologies qui peuvent faire que la femme fasse un accouchement prématuré ou bien un avortement tardif. Par exemple, la femme est venue, la première des choses, l'infection urinaire, il faut vraiment demander et puis traiter. Le paludisme, il y a la SP qu'on donne ? Pour nous, je vous dis, chez nous, la prise est supervisée. Avant, quand j'étais en brousse (milieu rural), les femmes prenaient de manière supervisée. En ville, les femmes pensent qu'elles sont sophistiquées. Ce sont des femmes de quoi ! quoi ! quoi ! Moi, j'ai remarqué, on avait beaucoup d'avortements, beaucoup d'accouchements prématurés. En ce temps, je n'étais même pas responsable, mais j'ai dit, il faut que la prise soit supervisée. Une femme qui refuse, qu'elle n'a pas mangé, elle n'a qu'à aller manger et revenir. Mais depuis qu'on a commencé, réellement, ça va. Mais le problème est que, s'il n'y a pas de SP au centre de santé ici, quand tu donnes l'ordonnance aux femmes, elles ne payent pas. Et quand elles ne payent pas, par exemple, je peux ne pas être en CPN, mais en salle seulement, quand je vois une femme avec TDR positif, je sais qu'il n'y a pas de SP en gratuité. Donc, ça veut dire que tout ce qui concerne vraiment la femme enceinte, il faut disponibiliser. Ne pas oublier l'échographie aussi. Voilà, les examens complémentaires. Il faut vraiment que tout soit honnête. Aussi, quand la femme vient, on n'a pas dit de faire un interrogatoire policier, mais il faut essayer de comprendre, être en amie avec cette femme, elle va se confier, elle va vous dire en tout cas, c'est quel problème socio-culturel elle a. Et là, vous allez essayer de prendre en charge. Si vous ne le pouvez pas, actuellement, on a des sociologues qui sont là, on peut vraiment diriger ces femmes-là vers ces gens. Et puis, une fois seulement qu'une femme a déjà fait un accouchement prématuré ou un avortement tardif, cette femme, il faut vraiment référer cette femme-là chez un gynécologue qui verra est-ce que cette femme a besoin d'un cerclage ou pas. Parce que souvent, il y a des femmes qui sont là. L'interrogatoire n'est pas correct. Elle avait déjà fait. C'est quand elle est sur la table, elle est venue pour expulser son enfant prématuré et que vous ne pouvez rien dire. « Non, non, non cela fait la deuxième fois maintenant ». En ce moment, toi, en tant qu'agent de santé, c'est toi qui n'a pas fait ton travail. Parce qu'il y a des gens qui pensent que si je dis ça, peut-être on va me faire comme ça, on va me réprimander, il y a tout ça. Il faut que les interrogatoires soient capitaux. Il faut que la CPN soit une CPN recentrée. On n'a pas besoin de prendre 50 femmes, si vous êtes deux, si vous prenez 20 femmes, 15 femmes et que le travail est correct, même quel qu'en soit ce que les gens vont dire « C'est Dieu qui fait ce que vous avez fait. Et vous allez voir, même si le problème vient là, ça ne porte pas sur vous. 00:33:43-9

00:33:43-9 Interviewer: Et par rapport à la formation du personnel de santé aussi ? 00:33:49-3

00:33:49-3 Person 1: Oui. Vous voyez, quand, par exemple, le questionnaire est là, vous voyez souvent, je suis là, je cherche. C'est pour dire que quand les gens sont sur le terrain, il y a des gens qui refusent vraiment de s'auto former. Oui, ou bien ils vont s'auto former sur autre chose, mais pas sur la prématurité. Mais si, par exemple, on a une formation par rapport à la prématurité, ça va nous aider. Et puis, avec des recyclages à l'appui, et puis, des supervisions. Par exemple, la revue documentaire, par exemple, cet enfant vous avez fait un accouchement la dernière fois, l'enfant, c'est 2 200, selon vous, c'était un enfant prématuré, c'était un enfant hypotrophe ? C'est comment ? Quelle a été la prise en charge ? Ça peut vraiment aider des gens à s'améliorer. 00:34:50-1

00:34:50-1 Interviewer: Et par rapport à l'équipement même du centre de santé ? 00:34:52-8

00:34:52-8 Person 1: Je vous ai dit que l'infection peut être... Souvent, il y a des femmes même qui vont prendre des infections ici. Vous voyez les tables. Si vous avez fait un tour, comme vos gens sont avec nous, il y a des femmes qui vont venir. Elles ne vont même pas étaler leurs pagnes, elles vont se coucher. Vous voyez tout de suite, il y a une femme qui a déposé une bouillotte là, une autre personne a pris la même bouillotte. Il y a beaucoup de ces cas. Mais moi, je me demande, est-ce qu'on pourra corriger ? C'est pour dire que l'équipement, c'est forcé. Il y a des années de cela, vous allez voir, normalement, quand on est en salle, je n'ai pas porté de bonnets, bien vrai que je suis au service. Je dois tout porter. Je ne dois pas toucher à un nouveau-né. Je dois porter des bavettes. L'équipement doit être au total. Les blouses doivent même être lavées, repassées totalement. Les bottes, tout doit être propre. Ça veut dire nos salles même, de passage, je remercie Denbalo. Parce que quand on a repris à travailler dans notre salle, c'était une grande fierté pour moi. On a eu une formation et j'ai tout fait pour que les docteurs viennent voir comment c'était propre et quand les gens viennent, il n'y avait pas grand-chose, on n'avait même pas amené nos affiches, mais le fait que c'est propre déjà, ça menait, c'était bien. Même d'autres collègues voyaient et disaient, « Je vous là c'est bien, je vous là c'est bien ». On vous remercie (projet Denbalo) de passage, vraiment. Et puis, merci aussi pour les tables d'accouchement. On avait besoin de ça. On a besoin d'équipements. On a besoin même, il y a beaucoup de choses, même la salle d'accouchement doit vraiment être adéquate. Parce que vous voyez, quand il y a la réanimation, peut-être si vous voulez, on va faire un tour, j'ai été obligée de prendre la lampe baladeuse de la salle PEF pour envoyer pour la réanimation des nouveau-nés. Pour qu'au moins cette lampe-là chauffe un peu. 00:37:07-4

00:37:07-4 Interviewer: Maintenant, il y a un phénomène qui est beaucoup plus fréquent en milieu médical, le mouvement des agents. Pensez-vous que cela peut être un facteur handicapant pour votre centre de santé au cas où ceux qui ont eu une formation spécifique sur la prématurité vont ailleurs ? Comment est-ce qu'on peut pallier à cela ? 00:37:29-3

00:37:29-3 Personne 1: Par exemple, il y a des gens, je prends un exemple terre à terre, on a PATHFINDER a formé tous les agents qui étaient là, même les accoucheuses, je veux dire tout agent à la maternité sur la planification familiale. Et d'autres sont partis, d'autres ont eu des concours, d'autres sont partis. Moi, je me dis si on veut former les agents de santé par rapport à la prématurité, je pense que c'est mieux de former de façon nationale. Oui, de façon nationale et puis essayer de faire des recyclages chaque fois. Les gens qui sont sur place, parce qu'il y a des gens aussi qui quittent l'école pour venir. Quand les gens quittent l'école pour venir et que ceux qui sont sur place là forment vraiment ceux qui viennent de l'école et que ça soit vraiment, je vais dire quoi, documenté. Et puis essayer de faire des formations périodiques. Peut-être, on va dire quoi ? Chaque an, pour les nouveaux qui viennent, voilà. Malgré le recyclage des aînés qui sont sur le terrain. Je pense que ça peut se faire. Parce qu'il y a des gens même qui vont bénéficier de la formation, par exemple, à Ouaga, mais ils sont affectés ici. Lorsqu'ils vont venir et par exemple, on demande « Est-ce que vous avez été formé sur la prématurité ? » « Non, je n'ai pas été formé. » Parce que les gens pensent que la formation, c'est de l'argent seulement. Mais si ça a été fait de façon nationale, vous voyez que personne ne peut vous dire que « Non, je n'ai pas été formé. » Si tu es sorti, ça vaut deux ou trois ans. Parce qu'il y a des gens qui veulent seulement les perdiems. Et quand tu es formé aussi, il faut que réellement on voit ce que ça t'a apporté. Parce que depuis que moi-même, j'ai quitté l'école, à l'école j'ai eu la formation mais le district n'a jamais formé par rapport à ça, à la prématurité. 00:39:52-0

**00:29:47-6 Interviewer:** Given everything we've discussed, what solutions do you propose to improve the management of premature births at your health center?

**00:29:57-1 Person 1:** The key to improvement is to focus on individual counseling and refocused prenatal care (CPN). We need to understand and address women's problems early on. For instance, we should promptly detect and treat conditions like urinary infections and malaria, which can lead to premature births or late abortions. In our center, we ensure that treatments like sulfadoxine-pyrimethamine (SP) are supervised, which wasn't the case in urban settings initially. Since implementing supervised intake, we've seen a decrease in abortions and premature births. However, we face challenges when SP isn't available at the health center. It's also crucial to have all necessary resources for pregnant women's care, including ultrasounds and other diagnostic tools. A welcoming environment for women to share their socio-cultural concerns is important, and when necessary, referring them to sociologists. Additionally, women with a history of premature birth or late abortion should be referred to a gynecologist for further assessment, such as the need for cerclage. The quality of CPN is more important than the quantity. Even if we can only manage 15 to 20 women but provide thorough care, it's more beneficial. We aim to do a good job, knowing that even if challenges arise, we have done our best.

**00:32:01-5 Interviewer:** What about the training of health personnel?

**00:33:49-3 Person 1:** Training in prematurity management would be beneficial. Some health workers are resistant to self-training in this area. Regular retraining and supervision, including case reviews, would help improve their skills and knowledge.

**00:34:50-1 Interviewer:** And regarding the equipment at the health center?

**00:34:52-8 Person 1:** Infection control is a significant issue here. We often see that women who come in don't take adequate sanitary measures, like laying out their loincloths before lying down. Some patients even share items like porridge, which can spread infections. We need to address these practices to improve hygiene. In the past, when I worked in the dining area, I didn't always wear the necessary protective gear, but now I understand its importance. Every piece of equipment, from blouses to boots, must be thoroughly cleaned and sanitized.After we revamped our room, I took pride in maintaining cleanliness and encouraged others to do the same. We're grateful for the support from the Denbalo project, which has provided us with necessary items like delivery tables. Our equipment needs are extensive, and even the delivery room requires upgrades. For example, for newborn resuscitation, I've had to improvise with a portable lamp from another room to provide some warmth to the babies. We need more suitable equipment to provide proper care in these critical situations.

**00:37:07-4 Interviewer:** The movement of staff is a common phenomenon in the medical field. Do you think this could be a hindrance for your health center, especially if those who have received specific training in prematurity care leave? How can this be addressed?

**00:37:29-3 Person 1:** For instance, we had a training program by Pathfinder for all staff in maternity care, including midwives, on family planning. However, some of them left for various reasons. To tackle the issue of training in prematurity, I believe national-level training is more effective. Regular refresher courses should be organized for existing staff, and new staff coming from schools should be trained by those already on the ground. This process should be well-documented.Periodic training could be done annually for newcomers, in addition to the retraining of senior staff. Even if some trained personnel are assigned elsewhere, like from Ouaga to here, they can bring their knowledge. But the mindset needs to change; training shouldn’t be seen just as a means to earn per diem. It should be valued for the knowledge it imparts. Since I finished school, I haven't received any specific training on prematurity from the district, which indicates a gap in our training approach.

### Focus group of Farakan health and social promotion center

[00:39:03-4](http://localhost:2300/file=C:/Users/USER/Desktop/ENTRETIENS%20PREMATURITE/CSPS_FARAKAN/FOCUS_GROUPE-CSPS_FARAKAN.MP3time=2343400) Interviewer: Ok, s'il n'y a pas d'autres interventions, nous allons terminer maintenant la dernière question, comment peut-on améliorer la prise en charge de naissances prématurées au sein de votre CSPS,selon vous comment peut on améliorer la prise en charge des naissances prématurées? [00:39:38-3](http://localhost:2300/file=C:/Users/USER/Desktop/ENTRETIENS%20PREMATURITE/CSPS_FARAKAN/FOCUS_GROUPE-CSPS_FARAKAN.MP3time=2378300)

[00:39:38-3](http://localhost:2300/file=C:/Users/USER/Desktop/ENTRETIENS%20PREMATURITE/CSPS_FARAKAN/FOCUS_GROUPE-CSPS_FARAKAN.MP3time=2378300) Person 3: Peut-être, si on forme les le personnel sur une prise en charge, les gestes importants pendant l'accouchement, qu'est-ce qu'il faut faire ? Bon, en tout cas, une petite formation avec des éléments capitaux résumés et affichés cela va permettre d'améliorer la prise en charge en attendant le niveau superieur. [00:40:11-4](http://localhost:2300/file=C:/Users/USER/Desktop/ENTRETIENS%20PREMATURITE/CSPS_FARAKAN/FOCUS_GROUPE-CSPS_FARAKAN.MP3time=2411400)

[00:40:11-4](http://localhost:2300/file=C:/Users/USER/Desktop/ENTRETIENS%20PREMATURITE/CSPS_FARAKAN/FOCUS_GROUPE-CSPS_FARAKAN.MP3time=2411400) Person 4: Vraiment, on veut éviter ça, mais avec vraiment une sensibilisation et que les agents soient dynamiques pour les sensibiliser dès leur première arrivée dans les centres de santé. Iil faut que les agents soient bien formés pour une sensibilisation meilleure. Là vraiment, on peut éviter beaucoup de cas de prématurités. [00:40:43-4](http://localhost:2300/file=C:/Users/USER/Desktop/ENTRETIENS%20PREMATURITE/CSPS_FARAKAN/FOCUS_GROUPE-CSPS_FARAKAN.MP3time=2443400)

[00:40:43-4](http://localhost:2300/file=C:/Users/USER/Desktop/ENTRETIENS%20PREMATURITE/CSPS_FARAKAN/FOCUS_GROUPE-CSPS_FARAKAN.MP3time=2443400) Person 8: La formation, vraiment former les agents de santé. [00:40:46-4](http://localhost:2300/file=C:/Users/USER/Desktop/ENTRETIENS%20PREMATURITE/CSPS_FARAKAN/FOCUS_GROUPE-CSPS_FARAKAN.MP3time=2446400)

[00:40:46-4](http://localhost:2300/file=C:/Users/USER/Desktop/ENTRETIENS%20PREMATURITE/CSPS_FARAKAN/FOCUS_GROUPE-CSPS_FARAKAN.MP3time=2446400) Person 7: Pour moi, il faut une prise de conscience des 2 côtés, chez les agents de santé et la population aussi.C'est ce que je voulais ajouter. [00:41:04-3](http://localhost:2300/file=C:/Users/USER/Desktop/ENTRETIENS%20PREMATURITE/CSPS_FARAKAN/FOCUS_GROUPE-CSPS_FARAKAN.MP3time=2464300)

[00:41:04-3](http://localhost:2300/file=C:/Users/USER/Desktop/ENTRETIENS%20PREMATURITE/CSPS_FARAKAN/FOCUS_GROUPE-CSPS_FARAKAN.MP3time=2464300) Person 3: J'ai oublié, il faut un plateau technique également, parce que, même si les gens sont formés, que les plateaux n'est pas là, cela ne va pas concourir à l'atteinte de l'objectif. [00:41:21-3](http://localhost:2300/file=C:/Users/USER/Desktop/ENTRETIENS%20PREMATURITE/CSPS_FARAKAN/FOCUS_GROUPE-CSPS_FARAKAN.MP3time=2481300)

[00:41:21-3](http://localhost:2300/file=C:/Users/USER/Desktop/ENTRETIENS%20PREMATURITE/CSPS_FARAKAN/FOCUS_GROUPE-CSPS_FARAKAN.MP3time=2481300) Person 5: C'est comme les autres l'ont dit, il faut une formation des agents de santé et puis nous doter aussi en plateau technique pourqu'on puisse bien prendre en charge les prématurées. [00:41:35-4](http://localhost:2300/file=C:/Users/USER/Desktop/ENTRETIENS%20PREMATURITE/CSPS_FARAKAN/FOCUS_GROUPE-CSPS_FARAKAN.MP3time=2495400)

[00:41:52-7](http://localhost:2300/file=C:/Users/USER/Desktop/ENTRETIENS%20PREMATURITE/CSPS_FARAKAN/FOCUS_GROUPE-CSPS_FARAKAN.MP3time=2512700) Interviewer: Ok, donc je vous remercie bien. Qu'est ce qu'il faut faire pour améliorer la prise en charge des naissances prématurées au sein de votre CSPS ? [00:42:13-0](http://localhost:2300/file=C:/Users/USER/Desktop/ENTRETIENS%20PREMATURITE/CSPS_FARAKAN/FOCUS_GROUPE-CSPS_FARAKAN.MP3time=2533000)

[00:42:13-0](http://localhost:2300/file=C:/Users/USER/Desktop/ENTRETIENS%20PREMATURITE/CSPS_FARAKAN/FOCUS_GROUPE-CSPS_FARAKAN.MP3time=2533000) Person 2: Bon, il faut renforcer la surveillance au niveau de la CPN et puis demander les examens complémentaires pour detecter les cas de menaces afin de prevenir. [00:42:54-1](http://localhost:2300/file=C:/Users/USER/Desktop/ENTRETIENS%20PREMATURITE/CSPS_FARAKAN/FOCUS_GROUPE-CSPS_FARAKAN.MP3time=2574100)

[00:42:54-1](http://localhost:2300/file=C:/Users/USER/Desktop/ENTRETIENS%20PREMATURITE/CSPS_FARAKAN/FOCUS_GROUPE-CSPS_FARAKAN.MP3time=2574100) Interviewer: Les cas de menaces,vous faites allusion à quels types de menaces? [00:42:56-9](http://localhost:2300/file=C:/Users/USER/Desktop/ENTRETIENS%20PREMATURITE/CSPS_FARAKAN/FOCUS_GROUPE-CSPS_FARAKAN.MP3time=2576900)

[00:42:56-9](http://localhost:2300/file=C:/Users/USER/Desktop/ENTRETIENS%20PREMATURITE/CSPS_FARAKAN/FOCUS_GROUPE-CSPS_FARAKAN.MP3time=2576900) Person 2: Bon, si on fait les examens, si la prise en charge est correct.Par exemple, si la tension est élevée, on peut prévenir, Puisque la HTA (Hypertension arteriel) peut provoquer l'accouchement prématuré.C'est un exemple. [00:43:28-8](http://localhost:2300/file=C:/Users/USER/Desktop/ENTRETIENS%20PREMATURITE/CSPS_FARAKAN/FOCUS_GROUPE-CSPS_FARAKAN.MP3time=2608800)

[00:43:28-8](http://localhost:2300/file=C:/Users/USER/Desktop/ENTRETIENS%20PREMATURITE/CSPS_FARAKAN/FOCUS_GROUPE-CSPS_FARAKAN.MP3time=2608800) Person 3: Pour ajouter à ce que ma collègue vient de dire, effectivement, nous allons prévenir depuis la grossesse et en détectant les pathologies associées et en les traitant correctement, et pendant l'accouchement, il faut franchement améliorer nos salles d'accouchement parce que une salle d'accouchement où il y a trop d'air, souvent même une température qui n'est vraiment pas adaptée même aux nouveau-nés. Parce que les nouveaux-nés sont franchement exposés à l'hypothermie. Parce que les salles ne sont pas du tout adaptées, au moins si on avait des salles adaptées, même quand l'accouchement se produit, au moins l'enfant a une prise en charge correcte ici avant même d'être évacué, ça lui donne plus de survie. La chance de survivre. [00:44:13-2](http://localhost:2300/file=C:/Users/USER/Desktop/ENTRETIENS%20PREMATURITE/CSPS_FARAKAN/FOCUS_GROUPE-CSPS_FARAKAN.MP3time=2653200)

00:39:03-4 Interviewer: Ok, s'il n'y a pas d'autres interventions, nous allons terminer maintenant la dernière question, comment peut-on améliorer la prise en charge de naissances prématurées au sein de votre CSPS, selon vous comment peut-on améliorer la prise en charge des naissances prématurées ? 00:39:38-3

00:39:38-3 Person 3: Peut-être, si on forme le personnel sur une prise en charge, les gestes importants pendant l'accouchement, qu'est-ce qu'il faut faire ? Bon, en tout cas, une petite formation avec des éléments capitaux résumés et affichés cela va permettre d'améliorer la prise en charge en attendant le niveau supérieur. 00:40:11-4

00:40:11-4 Person 4: Vraiment, on veut éviter ça, mais avec vraiment une sensibilisation et que les agents soient dynamiques pour les sensibiliser dès leur première arrivée dans les centres de santé. Il faut que les agents soient bien formés pour une sensibilisation meilleure. Là vraiment, on peut éviter beaucoup de cas de prématurités. 00:40:43-4

00:40:43-4 Person 8: La formation, vraiment former les agents de santé. 00:40:46-4

00:40:46-4 Person 7: Pour moi, il faut une prise de conscience des deux côtés, chez les agents de santé et la population aussi. C'est ce que je voulais ajouter. 00:41:04-3

00:41:04-3 Person 3: J'ai oublié, il faut un plateau technique également, parce que, même si les gens sont formés, que les plateaux ne sont pas là, cela ne va pas concourir à l'atteinte de l'objectif. 00:41:21-3

00:41:21-3 Person 5: C'est comme les autres l'ont dit, il faut une formation des agents de santé et puis nous doter aussi en plateau technique pour qu'on puisse bien prendre en charge les prématurées. 00:41:35-4

00:41:35-4 Interviewer: Je pense que nous sommes au terme donc de l'entretien. Je vous remercie beaucoup pour votre patience et la qualité des réponses que vous avez apportées. S'il y a quelque chose d'autre à ajouter avant de conclure, je pense que nous sommes toutes oreilles. Nous sommes à votre disposition. 00:41:52-7

00:41:52-7 Person 3: Hors antenne. 00:41:52-7

00:41:52-7 Interviewer: Ok, donc je vous remercie bien. Qu'est-ce qu'il faut faire pour améliorer la prise en charge des naissances prématurées au sein de votre CSPS ? 00:42:13-0

00:42:13-0 Person 2: Bon, il faut renforcer la surveillance au niveau de la CPN et puis demander les examens complémentaires pour détecter les cas de menaces afin de prévenir. 00:42:54-1

00:42:54-1 Interviewer: Les cas de menaces, vous faites allusion à quels types de menaces ? 00:42:56-9

00:42:56-9 Person 2: Bon, si on fait les examens, si la prise en charge est correcte. Par exemple, si la tension est élevée, on peut prévenir, puisque la HTA (Hypertension artérielle) peut provoquer l'accouchement prématuré. C'est un exemple. 00:43:28-8

00:43:28-8 Person 3: Pour ajouter à ce que ma collègue vient de dire, effectivement, nous allons prévenir depuis la grossesse et en détectant les pathologies associées et en les traitant correctement, et pendant l'accouchement, il faut franchement améliorer nos salles d'accouchement parce que une salle d'accouchement où il y a trop d'air, souvent même une température qui n'est vraiment pas adaptée même aux nouveau-nés. Parce que les nouveau-nés sont franchement exposés à l'hypothermie. Parce que les salles ne sont pas du tout adaptées, au moins si on avait des salles adaptées, même quand l'accouchement se produit, au moins l'enfant a une prise en charge correcte ici avant même d'être évacué, ça lui donne plus de survie. La chance de survivre. 00:44:13-2

**00:39:03-4 Interviewer:** Okay, if there are no further comments, we'll move to the last question. How can we improve the management of premature births within your CSPS? In your opinion, what improvements can be made?

**00:39:38-3 Person 3:** Training staff on essential actions during childbirth and care of premature babies is key. Having a brief, summarized training that can be quickly referenced will help improve care while waiting for higher-level intervention.

**00:40:11-4 Person 4:** Real awareness and dynamic engagement from health workers from the moment they arrive at health centers is crucial. Well-trained agents can significantly reduce the incidence of prematurity through effective awareness.

**00:40:43-4 Person 8:** The focus should be on training health workers comprehensively.

**00:40:46-4 Person 7:** Awareness is needed on both sides, among health workers and the community. That's an important aspect.

**00:41:04-3 Person 3:** Additionally, we need an adequate technical platform. Training is important, but without the proper equipment and facilities, we can't achieve our goals.

**00:41:21-3 Person 5:** As others have said, we need both training for health workers and the right technical platform to properly care for premature babies.

**00:41:52-7 Interviewer:** Thank you for your responses. Is there anything else to add before we conclude?

**00:42:13-0 Person 2:** Strengthening surveillance during prenatal care (CPN) and requesting additional exams to detect and prevent potential complications is essential.

**00:42:54-1 Interviewer:** What kind of threats are you referring to?

**00:42:56-9 Person 2:** By conducting thorough exams and providing appropriate care, we can prevent issues like hypertension, which can lead to premature birth.

**00:43:28-8 Person 3:** To add, it's about preventive measures during pregnancy by detecting and treating associated pathologies. We also need to improve our delivery rooms. Proper temperature control is crucial, as newborns are highly susceptible to hypothermia. Adapted rooms would offer better immediate care and increase survival chances for premature babies

### Major of Farakan health and social promotion center

[00:13:32-0](http://localhost:2300/file=C:/Users/USER/Desktop/ENTRETIENS%20PREMATURITE/CSPS_FARAKAN/MAJOR%20FARAKAN.MP3time=812000) Interviewer: Selon vous, comment est ce qu'on peut améliorer concrètement la prise en charge des naissances prématurées au sein de votre centre de santé? [00:13:45-7](http://localhost:2300/file=C:/Users/USER/Desktop/ENTRETIENS%20PREMATURITE/CSPS_FARAKAN/MAJOR%20FARAKAN.MP3time=825700)

[00:13:45-7](http://localhost:2300/file=C:/Users/USER/Desktop/ENTRETIENS%20PREMATURITE/CSPS_FARAKAN/MAJOR%20FARAKAN.MP3time=825700) Interviewer: C'est l'équipement, renforcer les compétences du personnel en matière de prise en charge,si on a le plateau technique, le personnel bien formé,je pense qu'on pourra gerer. [00:13:58-9](http://localhost:2300/file=C:/Users/USER/Desktop/ENTRETIENS%20PREMATURITE/CSPS_FARAKAN/MAJOR%20FARAKAN.MP3time=838900)

[00:13:58-9](http://localhost:2300/file=C:/Users/USER/Desktop/ENTRETIENS%20PREMATURITE/CSPS_FARAKAN/MAJOR%20FARAKAN.MP3time=838900) Interviewer: Il y a également un défi auquel les centres de santés sont confrontés à cause des mouvements des agents. Quelles solutions est-ce que vous préconisez pour ne pas perdre la qualité des prestataires ? [00:14:15-5](http://localhost:2300/file=C:/Users/USER/Desktop/ENTRETIENS%20PREMATURITE/CSPS_FARAKAN/MAJOR%20FARAKAN.MP3time=855500)

[00:14:15-5](http://localhost:2300/file=C:/Users/USER/Desktop/ENTRETIENS%20PREMATURITE/CSPS_FARAKAN/MAJOR%20FARAKAN.MP3time=855500) Person 1: Ça sest très difficile d'empêcher les mouvements là même moi voilà, je suis partie moi ça fait 11 ans que je suis làv et voilà, je m'en vais,pour moi, c'est la retraite. Est-ce qu'on peut empêcher quelqu'un d'aller à la retraite ? Voilà. Donc voilà, c'est compliqué. Peut-être qu'il faut former de façon régulière comme ça si certains partent, d'autres restent, il y a toujours la compétence qui est là. il faut faire des recyclages aussi. [00:14:46-2](http://localhost:2300/file=C:/Users/USER/Desktop/ENTRETIENS%20PREMATURITE/CSPS_FARAKAN/MAJOR%20FARAKAN.MP3time=886200)

[00:14:46-2](http://localhost:2300/file=C:/Users/USER/Desktop/ENTRETIENS%20PREMATURITE/CSPS_FARAKAN/MAJOR%20FARAKAN.MP3time=886200) Interviewer: Est-ce que vous pensez que les présentateurs sociaux-culturels , peuvent affecter les mères des enfants prématurés? [00:14:54-1](http://localhost:2300/file=C:/Users/USER/Desktop/ENTRETIENS%20PREMATURITE/CSPS_FARAKAN/MAJOR%20FARAKAN.MP3time=894100)

[00:14:54-1](http://localhost:2300/file=C:/Users/USER/Desktop/ENTRETIENS%20PREMATURITE/CSPS_FARAKAN/MAJOR%20FARAKAN.MP3time=894100) Person 1: C'est possible, c'est possible.Le milieu aussi, ça peut jouer beaucoup. [00:15:05-7](http://localhost:2300/file=C:/Users/USER/Desktop/ENTRETIENS%20PREMATURITE/CSPS_FARAKAN/MAJOR%20FARAKAN.MP3time=905700)

[00:15:05-7](http://localhost:2300/file=C:/Users/USER/Desktop/ENTRETIENS%20PREMATURITE/CSPS_FARAKAN/MAJOR%20FARAKAN.MP3time=905700) Interviewer: Comment? [00:15:07-2](http://localhost:2300/file=C:/Users/USER/Desktop/ENTRETIENS%20PREMATURITE/CSPS_FARAKAN/MAJOR%20FARAKAN.MP3time=907200)

[00:15:07-2](http://localhost:2300/file=C:/Users/USER/Desktop/ENTRETIENS%20PREMATURITE/CSPS_FARAKAN/MAJOR%20FARAKAN.MP3time=907200) Person 1: Parce que, quand on est dans un milieu où les gens ne comprennent pas, surtout les enfants qui sortent petit comme ça. Generalement ont dit qu' il ne va pas survivre.Souvent les parents même par rapport aux références, ils sont reticents, ils disent c'est un petit bébé et c'est pas sûr qu'il survive. Quand nous les referons il y a beaucoup qui ne veulent pas aller mais on les encourage quand même.Nous leurs disons que le bébé peut survivre.Si on prend soin de lui correctement, là en tout cas il peut survivre et être plus solide que l'enfant qui est né à terme à la longue.Moi j'ai l'enfant de ma grande soeur qui est né prématuré,mais il est là, les sapeurs-pompiers aujourd'hui. [00:15:47-4](http://localhost:2300/file=C:/Users/USER/Desktop/ENTRETIENS%20PREMATURITE/CSPS_FARAKAN/MAJOR%20FARAKAN.MP3time=947400)

[00:15:47-4](http://localhost:2300/file=C:/Users/USER/Desktop/ENTRETIENS%20PREMATURITE/CSPS_FARAKAN/MAJOR%20FARAKAN.MP3time=947400) Interviewer: Félicitation [00:15:48-2](http://localhost:2300/file=C:/Users/USER/Desktop/ENTRETIENS%20PREMATURITE/CSPS_FARAKAN/MAJOR%20FARAKAN.MP3time=948200)

[00:15:48-2](http://localhost:2300/file=C:/Users/USER/Desktop/ENTRETIENS%20PREMATURITE/CSPS_FARAKAN/MAJOR%20FARAKAN.MP3time=948200) Et il est fort (rire) [00:15:53-1](http://localhost:2300/file=C:/Users/USER/Desktop/ENTRETIENS%20PREMATURITE/CSPS_FARAKAN/MAJOR%20FARAKAN.MP3time=953100)

[00:15:53-1](http://localhost:2300/file=C:/Users/USER/Desktop/ENTRETIENS%20PREMATURITE/CSPS_FARAKAN/MAJOR%20FARAKAN.MP3time=953100) Interviewer: Il ya vraiment un travail de sensibilisation [00:15:55-7](http://localhost:2300/file=C:/Users/USER/Desktop/ENTRETIENS%20PREMATURITE/CSPS_FARAKAN/MAJOR%20FARAKAN.MP3time=955700)

[00:15:55-7](http://localhost:2300/file=C:/Users/USER/Desktop/ENTRETIENS%20PREMATURITE/CSPS_FARAKAN/MAJOR%20FARAKAN.MP3time=955700) Person 1: Voilà, il faut sensibiliser dans le milieu pour qu'il accepte les bébés nés prématurés. [00:16:02-8](http://localhost:2300/file=C:/Users/USER/Desktop/ENTRETIENS%20PREMATURITE/CSPS_FARAKAN/MAJOR%20FARAKAN.MP3time=962800)

[00:16:02-8](http://localhost:2300/file=C:/Users/USER/Desktop/ENTRETIENS%20PREMATURITE/CSPS_FARAKAN/MAJOR%20FARAKAN.MP3time=962800) Interviewer: Est ce que les préjugés peuvent amener un delaissement? [00:16:04-6](http://localhost:2300/file=C:/Users/USER/Desktop/ENTRETIENS%20PREMATURITE/CSPS_FARAKAN/MAJOR%20FARAKAN.MP3time=964600)

[00:16:04-6](http://localhost:2300/file=C:/Users/USER/Desktop/ENTRETIENS%20PREMATURITE/CSPS_FARAKAN/MAJOR%20FARAKAN.MP3time=964600) Person 1: C'est vraiment ça aussi. [00:16:08-6](http://localhost:2300/file=C:/Users/USER/Desktop/ENTRETIENS%20PREMATURITE/CSPS_FARAKAN/MAJOR%20FARAKAN.MP3time=968600)

[00:16:08-6](http://localhost:2300/file=C:/Users/USER/Desktop/ENTRETIENS%20PREMATURITE/CSPS_FARAKAN/MAJOR%20FARAKAN.MP3time=968600) Interviewer: Cela alourdi la tâche des prestateurs de soins? [00:16:10-8](http://localhost:2300/file=C:/Users/USER/Desktop/ENTRETIENS%20PREMATURITE/CSPS_FARAKAN/MAJOR%20FARAKAN.MP3time=970800)

[00:16:10-8](http://localhost:2300/file=C:/Users/USER/Desktop/ENTRETIENS%20PREMATURITE/CSPS_FARAKAN/MAJOR%20FARAKAN.MP3time=970800) Person 1: On le voit ici, il y a beaucoup qui refusent, ils ne veulent pas aller (les references) mais nous insistons [00:16:18-2](http://localhost:2300/file=C:/Users/USER/Desktop/ENTRETIENS%20PREMATURITE/CSPS_FARAKAN/MAJOR%20FARAKAN.MP3time=978200)

00:13:32-0 Interviewer: Selon vous, comment est-ce qu'on peut améliorer concrètement la prise en charge des naissances prématurées au sein de votre centre de santé ? 00:13:45-7

00:13:45-7 Interviewer: C'est l'équipement, renforcer les compétences du personnel en matière de prise en charge, si on a le plateau technique, le personnel bien formé, je pense qu'on pourra gérer. 00:13:58-9

00:13:58-9 Interviewer: Il y a également un défi auquel les centres de santé sont confrontés à cause des mouvements des agents. Quelles solutions est-ce que vous préconisez pour ne pas perdre la qualité des prestataires ? 00:14:15-5

00:14:15-5 Person 1: Ça c'est très difficile d'empêcher les mouvements là, même moi voilà, je suis partie moi ça fait 11 ans que je suis là et voilà, je m'en vais, pour moi, c'est la retraite. Est-ce qu'on peut empêcher quelqu'un d'aller à la retraite ? Voilà. Donc voilà, c'est compliqué. Peut-être qu'il faut former de façon régulière comme ça si certains partent, d'autres restent, il y a toujours la compétence qui est là. Il faut faire des recyclages aussi. 00:14:46-2

00:14:46-2 Interviewer: Est-ce que vous pensez que les pesanteurs socio-culturelles peuvent affecter les mères des enfants prématurés ? 00:14:54-1

00:14:54-1 Personne 1: C'est possible, c'est possible. Le milieu aussi, ça peut jouer beaucoup. 00:15:05-7

00:15:05-7 Interviewer: Comment ? 00:15:07-2

00:15:07-2 Personne 1: Parce que, quand on est dans un milieu où les gens ne comprennent pas, surtout les enfants qui sortent petits comme ça. Généralement, on dit qu'il ne va pas survivre. Souvent les parents, même par rapport aux références, ils sont réticents, ils disent c'est un petit bébé et c'est pas sûr qu'il survive. Quand nous les référons, il y a beaucoup qui ne veulent pas aller, mais on les encourage quand même. Nous leur disons que le bébé peut survivre. Si on prend soin de lui correctement, là, en tout cas, il peut survivre et être plus solide que l'enfant qui est né à terme à la longue. Moi, j'ai l'enfant de ma grande sœur qui est né prématuré, mais il est là, les sapeurs-pompiers aujourd'hui. 00:15:47-4

00:15:47-4 Interviewer: Félicitation. 00:15:48-2

00:15:48-2 Personne 1: Et il est fort (rire). 00:15:53-1

00:15:53-1 Interviewer: Il y a vraiment un travail de sensibilisation. 00:15:55-7

00:15:55-7 Personne 1: Voilà, il faut sensibiliser dans le milieu pour qu'ils acceptent les bébés nés prématurés. 00:16:02-8

00:16:02-8 Interviewer: Est-ce que les préjugés peuvent amener à un délaissement ? 00:16:04-6

00:16:04-6 Personne 1: C'est vraiment ça aussi. 00:16:08-6

00:16:08-6 Interviewer: Cela alourdit la tâche des prestataires de soins ? 00:16:10-8

00:16:10-8 Personne 1: On le voit ici, il y a beaucoup qui refusent, ils ne veulent pas aller (les références), mais nous insistons. 00:16:18-2

**Interviewer (00:13:32-0):** "In your view, how can the management of premature births be concretely improved in your health center?"

**Person 1 (00:13:45-7):** "Improvement hinges on equipment and enhancing staff skills. With the right technical platform and well-trained staff, I believe we can manage better."

**Interviewer (00:13:58-9):** "Considering the challenges faced by health centers due to staff turnover, what solutions can be implemented to maintain service quality?"

**Person 1 (00:14:15-5):** "It's difficult to prevent staff turnover. For example, I've been here 11 years and am now retiring. We can't stop someone from retiring. The solution might be regular training, so skills remain even as some staff leave. Recycling knowledge is also necessary."

**Interviewer (00:14:46-2):** "Do you think socio-cultural constraints can impact mothers of premature infants?"

**Person 1 (00:14:54-1):** "Yes, it's possible. The environment plays a significant role."

**Interviewer (00:15:05-7):** "In what way?"

**Person 1 (00:15:07-2):** "In environments where people don't understand the situation of small babies, there's often a belief they won't survive. This leads to reluctance in following medical referrals. We encourage them, explaining that with proper care, these babies can survive and even thrive. I have a nephew who was premature and is now a strong firefighter."

**Interviewer (00:15:47-4):** "Congratulations."

**Person 1 (00:15:48-2):** "[Laughs] Yes, he is strong."

**Interviewer (00:15:53-1):** "There's definitely a need for awareness-raising."

**Person 1 (00:15:55-7):** "Exactly. We need to educate the community to accept premature babies."

**Interviewer (00:16:02-8):** "Can prejudice lead to neglect?"

**Person 1 (00:16:04-6):** "That's a big issue, yes."

**Interviewer (00:16:08-6):** "Does this increase the burden on healthcare providers?"

**Person 1 (00:16:10-8):** "We see it here. Many refuse to follow referrals, but we insist on them."

### Maternity manager of Farakan health and social promotion center

[00:23:16-2](http://localhost:2300/file=C:/Users/USER/Desktop/ENTRETIENS%20PREMATURITE/CSPS_FARAKAN/RESPONSABLE%20MATERNITE%20FARAKAN.MP3time=1396200) Interviewer: Au regard de tout ce que vous avez dit sur la naissance prématurée, comment est ce qu'on ameliorer la prise en charge des naissances prematurées dans votre centre de santé? [00:23:44-0](http://localhost:2300/file=C:/Users/USER/Desktop/ENTRETIENS%20PREMATURITE/CSPS_FARAKAN/RESPONSABLE%20MATERNITE%20FARAKAN.MP3time=1424000)

[00:23:44-0](http://localhost:2300/file=C:/Users/USER/Desktop/ENTRETIENS%20PREMATURITE/CSPS_FARAKAN/RESPONSABLE%20MATERNITE%20FARAKAN.MP3time=1424000) Person 1: La prise en charge, bon ce qui est à l'origine de la prematuritée, si nous arrivons à prévenir ses causes par exemple le paludisme, les infections, et si on arrive à sensibiliser les femmes, je pense qu'on peut ameliorer la prise en charge. Aussi depister les pathologies tôt [00:24:37-5](http://localhost:2300/file=C:/Users/USER/Desktop/ENTRETIENS%20PREMATURITE/CSPS_FARAKAN/RESPONSABLE%20MATERNITE%20FARAKAN.MP3time=1477500)

[00:24:37-5](http://localhost:2300/file=C:/Users/USER/Desktop/ENTRETIENS%20PREMATURITE/CSPS_FARAKAN/RESPONSABLE%20MATERNITE%20FARAKAN.MP3time=1477500) Interviewer: Les pathologies qui sont susceptibles d'amener la prématurité? [00:24:37-9](http://localhost:2300/file=C:/Users/USER/Desktop/ENTRETIENS%20PREMATURITE/CSPS_FARAKAN/RESPONSABLE%20MATERNITE%20FARAKAN.MP3time=1477900)

[00:24:37-9](http://localhost:2300/file=C:/Users/USER/Desktop/ENTRETIENS%20PREMATURITE/CSPS_FARAKAN/RESPONSABLE%20MATERNITE%20FARAKAN.MP3time=1477900) Person 1: Oui, les pathologies qui peuvent entrainer les cas de naissances prmaturées. il y a le paludisme, la malnutrition, l'hypertension, l'anemie, les infections et surtout les infecions urinaires etc. [00:25:09-3](http://localhost:2300/file=C:/Users/USER/Desktop/ENTRETIENS%20PREMATURITE/CSPS_FARAKAN/RESPONSABLE%20MATERNITE%20FARAKAN.MP3time=1509300)

[00:25:09-3](http://localhost:2300/file=C:/Users/USER/Desktop/ENTRETIENS%20PREMATURITE/CSPS_FARAKAN/RESPONSABLE%20MATERNITE%20FARAKAN.MP3time=1509300) Interviewer: Et en terme de platau technique, est ce que votre structure, s'elle est équiper peut offrir un meilleure prise en charge ou bien le personnel fait defaut? [00:25:20-1](http://localhost:2300/file=C:/Users/USER/Desktop/ENTRETIENS%20PREMATURITE/CSPS_FARAKAN/RESPONSABLE%20MATERNITE%20FARAKAN.MP3time=1520100)

[00:25:20-1](http://localhost:2300/file=C:/Users/USER/Desktop/ENTRETIENS%20PREMATURITE/CSPS_FARAKAN/RESPONSABLE%20MATERNITE%20FARAKAN.MP3time=1520100) Person 1: Si le personnel est qualifié je me dis que cela peut ameliorer la prise en charge. [00:25:26-2](http://localhost:2300/file=C:/Users/USER/Desktop/ENTRETIENS%20PREMATURITE/CSPS_FARAKAN/RESPONSABLE%20MATERNITE%20FARAKAN.MP3time=1526200)

[00:25:26-2](http://localhost:2300/file=C:/Users/USER/Desktop/ENTRETIENS%20PREMATURITE/CSPS_FARAKAN/RESPONSABLE%20MATERNITE%20FARAKAN.MP3time=1526200) Interviewer: Y a t il un besoin de formation du personnel par rapport àla prise en charge des naissances prematurées? [00:25:32-9](http://localhost:2300/file=C:/Users/USER/Desktop/ENTRETIENS%20PREMATURITE/CSPS_FARAKAN/RESPONSABLE%20MATERNITE%20FARAKAN.MP3time=1532900)

[00:25:32-9](http://localhost:2300/file=C:/Users/USER/Desktop/ENTRETIENS%20PREMATURITE/CSPS_FARAKAN/RESPONSABLE%20MATERNITE%20FARAKAN.MP3time=1532900) Person 1: Souvent aussi même,il faut que les gens sachent comment prendre en charge le prématuré. Parceque il faut des bonnets, il faut bien protèger l'enfant, mais si on arrive pas à le protèger bien, cela peut l'exposer. Il faut que le personnel ait les noions sur tous ses aspects. [00:26:09-0](http://localhost:2300/file=C:/Users/USER/Desktop/ENTRETIENS%20PREMATURITE/CSPS_FARAKAN/RESPONSABLE%20MATERNITE%20FARAKAN.MP3time=1569000)

[00:26:09-0](http://localhost:2300/file=C:/Users/USER/Desktop/ENTRETIENS%20PREMATURITE/CSPS_FARAKAN/RESPONSABLE%20MATERNITE%20FARAKAN.MP3time=1569000) Interviewer: Sensibilisations sur les gestes pour ameliorer la prise en charge? [00:26:13-5](http://localhost:2300/file=C:/Users/USER/Desktop/ENTRETIENS%20PREMATURITE/CSPS_FARAKAN/RESPONSABLE%20MATERNITE%20FARAKAN.MP3time=1573500)

[00:26:13-5](http://localhost:2300/file=C:/Users/USER/Desktop/ENTRETIENS%20PREMATURITE/CSPS_FARAKAN/RESPONSABLE%20MATERNITE%20FARAKAN.MP3time=1573500) Person 1: Oui sensibilisation du personnel sur les gestes pour ameliorer la prise en charge. Par exemple si la femme rentre au niveau du CMA, on sait que c'est une menace, dès qu'ils cinstatent, ils peuvent faire la maturation pulmonaire.On evacue les prématurés làbas, mais quand c'est tard souvent aussi c'est un peu compliqué. il y en a, on envoie au CMA et ça va mais il y en a aussi c'est compliqué.Maintenant si les gens sont formés,il y a des gestes aussi qu'il faut faire.Désobstruer correctement puisque géneralement quand ils sortent (les enfants prématurés) ils n'arrivent pas à respirer. la plupart du temps c'est la poche qui se rompt pourtant la grossesse n'est pas à terme sûrement l'enfant en sortant, il va prendre le liquide. quand il y a une rupture de la poche, et que ça dure cela peut entrainer une infection puisqu'il y a maintenant un passage qui est crée et l'enfant peut donc sortir avec une infection.Un prématuré avec une infection associée. [00:27:29-7](http://localhost:2300/file=C:/Users/USER/Desktop/ENTRETIENS%20PREMATURITE/CSPS_FARAKAN/RESPONSABLE%20MATERNITE%20FARAKAN.MP3time=1649700)

[00:27:29-7](http://localhost:2300/file=C:/Users/USER/Desktop/ENTRETIENS%20PREMATURITE/CSPS_FARAKAN/RESPONSABLE%20MATERNITE%20FARAKAN.MP3time=1649700) Interviewer: Quel genre de formation destiné aux agents de santé? [00:27:32-9](http://localhost:2300/file=C:/Users/USER/Desktop/ENTRETIENS%20PREMATURITE/CSPS_FARAKAN/RESPONSABLE%20MATERNITE%20FARAKAN.MP3time=1652900)

[00:27:32-9](http://localhost:2300/file=C:/Users/USER/Desktop/ENTRETIENS%20PREMATURITE/CSPS_FARAKAN/RESPONSABLE%20MATERNITE%20FARAKAN.MP3time=1652900) Person 1: Même si c'est un recyclage [00:27:40-9](http://localhost:2300/file=C:/Users/USER/Desktop/ENTRETIENS%20PREMATURITE/CSPS_FARAKAN/RESPONSABLE%20MATERNITE%20FARAKAN.MP3time=1660900)

[00:27:40-9](http://localhost:2300/file=C:/Users/USER/Desktop/ENTRETIENS%20PREMATURITE/CSPS_FARAKAN/RESPONSABLE%20MATERNITE%20FARAKAN.MP3time=1660900) Interviewer: Est ce que vous avez quelque chose d'autre à ajouter? [00:27:44-7](http://localhost:2300/file=C:/Users/USER/Desktop/ENTRETIENS%20PREMATURITE/CSPS_FARAKAN/RESPONSABLE%20MATERNITE%20FARAKAN.MP3time=1664700)

[00:27:44-7](http://localhost:2300/file=C:/Users/USER/Desktop/ENTRETIENS%20PREMATURITE/CSPS_FARAKAN/RESPONSABLE%20MATERNITE%20FARAKAN.MP3time=1664700) Person 1: Quelque chose à ajouter,Bon c'est vous dire merci (rire) je ne sais pas si j'ai pu repondre à vos questions? [00:27:52-5](http://localhost:2300/file=C:/Users/USER/Desktop/ENTRETIENS%20PREMATURITE/CSPS_FARAKAN/RESPONSABLE%20MATERNITE%20FARAKAN.MP3time=1672500)

[00:27:52-5](http://localhost:2300/file=C:/Users/USER/Desktop/ENTRETIENS%20PREMATURITE/CSPS_FARAKAN/RESPONSABLE%20MATERNITE%20FARAKAN.MP3time=1672500) Interviewer: Vous avez été parfaite madame [00:27:53-5](http://localhost:2300/file=C:/Users/USER/Desktop/ENTRETIENS%20PREMATURITE/CSPS_FARAKAN/RESPONSABLE%20MATERNITE%20FARAKAN.MP3time=1673500)

[00:27:53-5](http://localhost:2300/file=C:/Users/USER/Desktop/ENTRETIENS%20PREMATURITE/CSPS_FARAKAN/RESPONSABLE%20MATERNITE%20FARAKAN.MP3time=1673500) Person 1: rire. [00:28:04-3](http://localhost:2300/file=C:/Users/USER/Desktop/ENTRETIENS%20PREMATURITE/CSPS_FARAKAN/RESPONSABLE%20MATERNITE%20FARAKAN.MP3time=1684300)

[00:28:04-3](http://localhost:2300/file=C:/Users/USER/Desktop/ENTRETIENS%20PREMATURITE/CSPS_FARAKAN/RESPONSABLE%20MATERNITE%20FARAKAN.MP3time=1684300) Interviewer: Vos avis sont importants pour permettre d'ameliorer la prise en charge des naissances prématurées au niveau du Burkina Faso. [00:28:15-2](http://localhost:2300/file=C:/Users/USER/Desktop/ENTRETIENS%20PREMATURITE/CSPS_FARAKAN/RESPONSABLE%20MATERNITE%20FARAKAN.MP3time=1695200)

[00:28:15-2](http://localhost:2300/file=C:/Users/USER/Desktop/ENTRETIENS%20PREMATURITE/CSPS_FARAKAN/RESPONSABLE%20MATERNITE%20FARAKAN.MP3time=1695200) Person 1: Parce que la naissance prématurée, souvent quand cela arrive, tout le monde ne sait pas, ils peuvent faire d'autres types de traitements qui ne sont pas adaptés.Par exemple nous avons un cas ou une femme va accouché par contre la grosesse n'est pas à terme donc nous savons que c'est un cas de naissance prématurée. [00:28:41-0](http://localhost:2300/file=C:/Users/USER/Desktop/ENTRETIENS%20PREMATURITE/CSPS_FARAKAN/RESPONSABLE%20MATERNITE%20FARAKAN.MP3time=1721000)

[00:28:41-0](http://localhost:2300/file=C:/Users/USER/Desktop/ENTRETIENS%20PREMATURITE/CSPS_FARAKAN/RESPONSABLE%20MATERNITE%20FARAKAN.MP3time=1721000) Interviewer: Donc la gestion des cas depend du niveau de complication de la grossesse? S'il y a lieu de la referer? [00:28:42-8](http://localhost:2300/file=C:/Users/USER/Desktop/ENTRETIENS%20PREMATURITE/CSPS_FARAKAN/RESPONSABLE%20MATERNITE%20FARAKAN.MP3time=1722800)

[00:28:42-8](http://localhost:2300/file=C:/Users/USER/Desktop/ENTRETIENS%20PREMATURITE/CSPS_FARAKAN/RESPONSABLE%20MATERNITE%20FARAKAN.MP3time=1722800) Person 1: Oui oui c'est cela même. [00:28:59-4](http://localhost:2300/file=C:/Users/USER/Desktop/ENTRETIENS%20PREMATURITE/CSPS_FARAKAN/RESPONSABLE%20MATERNITE%20FARAKAN.MP3time=1739400)

[00:28:59-4](http://localhost:2300/file=C:/Users/USER/Desktop/ENTRETIENS%20PREMATURITE/CSPS_FARAKAN/RESPONSABLE%20MATERNITE%20FARAKAN.MP3time=1739400) Interviewer: S-il y avait un plateau technique vous arriverez à mieux gerer ses situations de naissances prématurées? [00:28:59-3](http://localhost:2300/file=C:/Users/USER/Desktop/ENTRETIENS%20PREMATURITE/CSPS_FARAKAN/RESPONSABLE%20MATERNITE%20FARAKAN.MP3time=1739300)

[00:28:59-3](http://localhost:2300/file=C:/Users/USER/Desktop/ENTRETIENS%20PREMATURITE/CSPS_FARAKAN/RESPONSABLE%20MATERNITE%20FARAKAN.MP3time=1739300) Person 1: Surtout diagnostiquer tôt pour pouvoir traiter. [00:29:02-1](http://localhost:2300/file=C:/Users/USER/Desktop/ENTRETIENS%20PREMATURITE/CSPS_FARAKAN/RESPONSABLE%20MATERNITE%20FARAKAN.MP3time=1742100)

[00:29:02-1](http://localhost:2300/file=C:/Users/USER/Desktop/ENTRETIENS%20PREMATURITE/CSPS_FARAKAN/RESPONSABLE%20MATERNITE%20FARAKAN.MP3time=1742100) Interviewer: Comment est ce qu'on peut diagnostiquer? [00:29:04-8](http://localhost:2300/file=C:/Users/USER/Desktop/ENTRETIENS%20PREMATURITE/CSPS_FARAKAN/RESPONSABLE%20MATERNITE%20FARAKAN.MP3time=1744800)

[00:29:04-8](http://localhost:2300/file=C:/Users/USER/Desktop/ENTRETIENS%20PREMATURITE/CSPS_FARAKAN/RESPONSABLE%20MATERNITE%20FARAKAN.MP3time=1744800) Person 1: Diagnostiquer, par exemple si la femme vient et elle a des symptômes, la nuit je me lève plusieurs fois uriner et quand j'urine cela me fait mal, ou bien est ce qu'elle prend la SP regulièrement, est ce qu'elle dort sous moustiquaire et même son alimentation. Puisque dans nos documents on parle de la nutrition de la maman, c'est à dire on demande si nous avons fait un counseling sur cela du fait qu'elle doit manger.Donc si on arrive à la prendre correctement en charge cela va diminuer le risque de prématurité.Pendant l'interrogatoire on demande s'elle a des douleurs pelviennes. [00:30:05-6](http://localhost:2300/file=C:/Users/USER/Desktop/ENTRETIENS%20PREMATURITE/CSPS_FARAKAN/RESPONSABLE%20MATERNITE%20FARAKAN.MP3time=1805600)

00:23:16-2 Interviewer: Au regard de tout ce que vous avez dit sur la naissance prématurée, comment est-ce qu'on peut améliorer la prise en charge des naissances prématurées dans votre centre de santé ? 00:23:44-0

00:23:44-0 Person 1: La prise en charge, bon ce qui est à l'origine de la prématurité, si nous arrivons à prévenir ses causes par exemple le paludisme, les infections, et si on arrive à sensibiliser les femmes, je pense qu'on peut améliorer la prise en charge. Aussi dépister les pathologies tôt. 00:24:37-5

00:24:37-5 Interviewer: Les pathologies qui sont susceptibles d'amener la prématurité ? 00:24:37-9

00:24:37-9 Person 1: Oui, les pathologies qui peuvent entraîner les cas de naissances prématurées. Il y a le paludisme, la malnutrition, l'hypertension, l'anémie, les infections et surtout les infections urinaires, etc. 00:25:09-3

00:25:09-3 Interviewer: Et en termes de plateau technique, est-ce que votre structure, si elle est équipée, peut offrir une meilleure prise en charge ou bien le personnel fait défaut ? 00:25:20-1

00:25:20-1 Person 1: Si le personnel est qualifié, je me dis que cela peut améliorer la prise en charge. 00:25:26-2

00:25:26-2 Interviewer: Y a-t-il un besoin de formation du personnel par rapport à la prise en charge des naissances prématurées ? 00:25:32-9

00:25:32-9 Person 1: Souvent aussi, il faut que les gens sachent comment prendre en charge le prématuré. Parce qu'il faut des bonnets, il faut bien protéger l'enfant, mais si on n'arrive pas à le protéger correctement, cela peut l'exposer. Il faut que le personnel ait les notions sur tous ces aspects. 00:26:09-0

00:26:09-0 Interviewer: Sensibilisations sur les gestes pour améliorer la prise en charge ? 00:26:13-5

00:26:13-5 Person 1: Oui, sensibilisation du personnel sur les gestes pour améliorer la prise en charge. Par exemple, si la femme rentre au niveau du CMA, on sait que c'est une menace, dès qu'ils constatent, ils peuvent faire la maturation pulmonaire. On évacue les prématurés là-bas, mais quand c'est tard, souvent aussi, c'est un peu compliqué. Il y en a, on envoie au CMA et ça va, mais il y en a aussi, c'est compliqué. Maintenant, si les gens sont formés, il y a des gestes aussi qu'il faut faire. Désobstruer correctement, puisque généralement quand ils sortent (les enfants prématurés), ils n'arrivent pas à respirer. La plupart du temps, c'est la poche qui se rompt pourtant la grossesse n'est pas à terme, sûrement l'enfant en sortant, il va prendre le liquide. Quand il y a une rupture de la poche, et que ça dure, cela peut entraîner une infection, puisqu'il y a maintenant un passage qui est créé et l'enfant peut donc sortir avec une infection. Un prématuré avec une infection associée. 00:27:29-7

00:27:29-7 Interviewer: Quel genre de formation destiné aux agents de santé ? 00:27:32-9

00:27:32-9 Person 1: Même si c'est un recyclage. 00:27:40-9

00:27:40-9 Interviewer: Est-ce que vous avez quelque chose d'autre à ajouter ? 00:27:44-7

00:27:44-7 Person 1: Quelque chose à ajouter. Bon c'est vous dire merci (rire), je ne sais pas si j'ai pu répondre à vos questions ? 00:27:52-5

00:27:52-5 Interviewer: Vous avez été parfaite madame. 00:27:53-5

00:27:53-5 Person 1: Rire. 00:28:04-3

00:28:04-3 Interviewer: Vos avis sont importants pour permettre d'améliorer la prise en charge des naissances prématurées au niveau du Burkina Faso. 00:28:15-2

00:28:15-2 Person 1: Parce que la naissance prématurée, souvent quand cela arrive, tout le monde ne sait pas, ils peuvent faire d'autres types de traitements qui ne sont pas adaptés. Par exemple, nous avons un cas où une femme va accoucher, par contre, la grossesse n'est pas à terme, donc nous savons que c'est un cas de naissance prématurée. 00:28:41-0

00:28:41-0 Interviewer: Donc la gestion des cas dépend du niveau de complication de la grossesse ? S'il y a lieu de la référer ? 00:28:42-8

00:28:42-8 Person 1: Oui, oui c'est cela même. 00:28:59-4

00:28:59-4 Interviewer: S'il y avait un plateau technique, vous arriveriez à mieux gérer ces situations de naissances prématurées ? 00:28:59-3

00:28:59-3 Person 1: Surtout diagnostiquer tôt pour pouvoir traiter. 00:29:02-1

00:29:02-1 Interviewer: Comment est-ce qu'on peut diagnostiquer ? 00:29:04-8

00:29:04-8 Person 1: Diagnostiquer, par exemple, si la femme vient et elle a des symptômes, la nuit je me lève plusieurs fois uriner et quand j'urine cela me fait mal, ou bien est-ce qu'elle prend la SP régulièrement, est-ce qu'elle dort sous moustiquaire, et même son alimentation. Puisque dans nos documents on parle de la nutrition de la maman, c'est-à-dire on demande si nous avons fait un counseling sur cela du fait qu'elle doit manger. Donc si on arrive à la prendre correctement en charge, cela va diminuer le risque de prématurité. Pendant l'interrogatoire, on demande si elle a des douleurs pelviennes. 00:30:05-6

**00:23:16-2 Interviewer:** Considering everything you've said about premature birth, how can we improve the care of premature births in your health center?

**00:23:44-0 Person 1:** Effective care begins with preventing the causes of prematurity, such as malaria and infections. Raising awareness among women and early detection of pathologies are crucial steps.

**00:24:37-5 Interviewer:** Are you referring to pathologies that can lead to premature births?

**00:24:37-9 Person 1:** Yes, these include malaria, malnutrition, hypertension, anemia, infections, especially urinary infections, etc.

**00:25:09-3 Interviewer:** And in terms of technical support, could better equipment and staff training enhance care?

**00:25:20-1 Person 1:** Qualified staff are essential for improving care. Training is crucial for effective management of premature births.

**00:26:09-0 Interviewer:** What about raising awareness among staff to improve care?

**00:26:13-5 Person 1:** Yes, staff need to be aware of actions that can improve care. For example, if there's a threat, like premature labor, staff should know immediate actions, like making the lungs mature or clearing airways for babies who can't breathe. A premature baby with an associated infection requires special care.

**00:27:29-7 Interviewer:** What kind of training is needed for health workers?

**00:27:32-9 Person 1:** Recurring training, even if it's refresher courses, is necessary.

**00:28:04-3 Interviewer:** Your opinions are important for improving premature birth care in Burkina Faso.

**00:28:15-2 Person 1:** Often, not everyone knows how to handle premature births. For instance, in a premature delivery case, knowing the appropriate treatment is crucial.

**00:28:42-8 Interviewer:** So, case management depends on the complication level of the pregnancy?

**00:28:59-4 Person 1:** Yes, exactly. Early diagnosis and appropriate treatment are key.

**00:29:02-1 Interviewer:** How can we improve diagnosis?

**00:29:04-8 Person 1:** Diagnosing involves assessing symptoms, ensuring regular preventive measures like SP intake, mosquito net usage, and proper nutrition. During questioning, pelvic pain and other symptoms should be probed.

# 12. Others

### Focus group of Accart-ville urban medical center

[00:38:20-6](http://localhost:2300/file=C:/Users/USER/Desktop/ENTRET)Interviewer: y a t il quelque chose d'autre à ajouter? [00:38:26-9](http://localhost:2300/file=C:/Users/USER/Desktop/ENTRETIENS)

[00:38:26-9](http://localhost:2300/file=C:/Users/USER/Desktop/ENTRETIENS%20PREMATURITE/CSPS-ACCART-VILLE/FOCUS%20GROUPE%20ACCART-VILLE-BON.mp3time=2306900)Interviewer: d'accord nous sommes arrivés au terme de l'entretien,nous vous remercions beaucoup pour avoir participer à cet entretien, merçi à vous et excellente suite de programme à tous. [00:38:41-9](http://localhost:2300/file=C:/Users/USER/Desktop/ENTRETIENS)

00:38:20-6 Interviewer: Y a-t-il quelque chose d'autre à ajouter ? 00:38:26-9

00:38:26-9 Interviewer: D'accord, nous sommes arrivés au terme de l'entretien, nous vous remercions beaucoup pour avoir participé à cet entretien, merci à vous et excellente suite de programme à tous. 00:38:41-9

**Interviewer (00:38:20-6):** "Is there anything else you would like to add?"

**Interviewer (00:38:26-9):** "Okay, we have reached the end of our interview. Thank you very much for your participation. Wishing you all an excellent continuation of your program."

### Manager of Accart-ville urban medical center

[00:26:15-6](http://localhost:2300/file=C:/Users/USER/Desktop/ENTRETIENS%20PREMATURITE/CSPS-ACCART-VILLE/RESPONSABLE%20CMU%20ACCART-VILLE.MP3time=1575600) Interviewer: d'accord. Je pense que nous sommes au terme de l'entretien. Mais avant de finir, est ce qu'il y a un point sur lequel vous souhaiteriez partager et qu'on n'a pas abordé à travers les questions sur le volet naissance prématurée? [00:26:31-4](http://localhost:2300/file=C:/Users/USER/Desktop/ENTRETIENS%20PREMATURITE/CSPS-ACCART-VILLE/RESPONSABLE%20CMU%20ACCART-VILLE.MP3time=1591400)

[00:26:31-4](http://localhost:2300/file=C:/Users/USER/Desktop/ENTRETIENS%20PREMATURITE/CSPS-ACCART-VILLE/RESPONSABLE%20CMU%20ACCART-VILLE.MP3time=1591400)Person 1: moi je pense qu'on a tout dit (rire) [00:26:34-8](http://localhost:2300/file=C:/Users/USER/Desktop/ENTRETIENS%20PREMATURITE/CSPS-ACCART-VILLE/RESPONSABLE%20CMU%20ACCART-VILLE.MP3time=1594800)

[00:26:34-8](http://localhost:2300/file=C:/Users/USER/Desktop/ENTRETIENS%20PREMATURITE/CSPS-ACCART-VILLE/RESPONSABLE%20CMU%20ACCART-VILLE.MP3time=1594800) Interviewer: Maintenant, moi je vais revenir sur une question par rapport à la définition. Est ce que la définition par rapport aux types La grossesses, Quand est ce qu'on peut parler de grossesse? Quand est ce qu'on peut parler de prématuré? Parce que.parce que je pense que l'avortement aussi se situe dans un grille.est ce qu'il y a des exceptions qui peuvent aussi impacter le travail des agents de santé? [00:27:01-2](http://localhost:2300/file=C:/Users/USER/Desktop/ENTRETIENS%20PREMATURITE/CSPS-ACCART-VILLE/RESPONSABLE%20CMU%20ACCART-VILLE.MP3time=1621200)

[00:27:01-2](http://localhost:2300/file=C:/Users/USER/Desktop/ENTRETIENS%20PREMATURITE/CSPS-ACCART-VILLE/RESPONSABLE%20CMU%20ACCART-VILLE.MP3time=1621200)Person 1: oui, en terme de prematuré,c'est entre 28 semaines et inferieur à 37 semaines ce sont des enfants dont l'âge de la grossesse est compris entre cette fourchette. si nous sommes en bas de 28 semaines on se trouve à un avortement tardive. dans mon experience ce que j'ai constater tous les enfants qui sont né avant 28 semaines, au depart ils etaient viables mais la continuité a été un peu difficile nous n'avons pas les moyens pour pouvoir prendre en charge en fait, la pluspart succombe après. le plateau technique n'est pas adequat en fait pour leurs prises en charges. au depart tu vois que l'enfant est viable mais après la prise en charge est un peu difficile, peu de temps après quelques jours ou quelques semaines l'enfant va succomber. [00:28:12-5](http://localhost:2300/file=C:/Users/USER/Desktop/ENTRETIENS%20PREMATURITE/CSPS-ACCART-VILLE/RESPONSABLE%20CMU%20ACCART-VILLE.MP3time=1692500)

[00:28:12-5](http://localhost:2300/file=C:/Users/USER/Desktop/ENTRETIENS%20PREMATURITE/CSPS-ACCART-VILLE/RESPONSABLE%20CMU%20ACCART-VILLE.MP3time=1692500) Interviewer: Merci pour votre participation. [00:28:14-6](http://localhost:2300/file=C:/Users/USER/Desktop/ENTRETIENS%20PREMATURITE/CSPS-ACCART-VILLE/RESPONSABLE%20CMU%20ACCART-VILLE.MP3time=1694600)

[00:28:14-6](http://localhost:2300/file=C:/Users/USER/Desktop/ENTRETIENS%20PREMATURITE/CSPS-ACCART-VILLE/RESPONSABLE%20CMU%20ACCART-VILLE.MP3time=1694600) Person 1: Merci à vous pareillement. [00:28:17-7](http://localhost:2300/file=C:/Users/USER/Desktop/ENTRETIENS%20PREMATURITE/CSPS-ACCART-VILLE/RESPONSABLE%20CMU%20ACCART-VILLE.MP3time=1697700)

[00:28:17-7](http://localhost:2300/file=C:/Users/USER/Desktop/ENTRETIENS%20PREMATURITE/CSPS-ACCART-VILLE/RESPONSABLE%20CMU%20ACCART-VILLE.MP3time=1697700)Interviewer: J'espère que les contributions vontt surtout aider à améliorer un peu tous les aspects liés à la naissance prématurée. [00:28:24-8](http://localhost:2300/file=C:/Users/USER/Desktop/ENTRETIENS%20PREMATURITE/CSPS-ACCART-VILLE/RESPONSABLE%20CMU%20ACCART-VILLE.MP3time=1704800)

00:26:15-6 Interviewer: D'accord. Je pense que nous sommes au terme de l'entretien. Mais avant de finir, est-ce qu'il y a un point sur lequel vous souhaiteriez partager et qu'on n'a pas abordé à travers les questions sur le volet naissance prématurée ?

00:26:31-4 Person 1: Moi, je pense qu'on a tout dit (rire).

00:26:34-8 Interviewer: Maintenant, je vais revenir sur une question par rapport à la définition. Est-ce que la définition par rapport aux types de grossesses, quand est-ce qu'on peut parler de grossesse ? Quand est-ce qu'on peut parler de prématuré ? Parce que je pense que l'avortement aussi se situe dans une grille. Est-ce qu'il y a des exceptions qui peuvent aussi impacter le travail des agents de santé ?

00:27:01-2 Person 1: Oui, en termes de prématuré, c'est entre 28 semaines et inférieur à 37 semaines, ce sont des enfants dont l'âge de la grossesse est compris entre cette fourchette. Si nous sommes en bas de 28 semaines, on se trouve à un avortement tardif. Dans mon expérience, ce que j'ai constaté, tous les enfants qui sont nés avant 28 semaines, au départ, ils étaient viables, mais la continuité a été un peu difficile. Nous n'avons pas les moyens pour pouvoir prendre en charge, en fait, la plupart succombent après. Le plateau technique n'est pas adéquat en fait pour leurs prises en charges. Au départ, tu vois que l'enfant est viable, mais après, la prise en charge est un peu difficile, peu de temps après quelques jours ou quelques semaines, l'enfant va succomber.

00:28:12-5 Interviewer: Merci pour votre participation.

00:28:14-6 Person 1: Merci à vous pareillement.

00:28:17-7 Interviewer: J'espère que les contributions vont surtout aider à améliorer un peu tous les aspects liés à la naissance prématurée. [00:28:24-8](http://localhost:2300/file=C:/Users/USER/Desktop/ENTRETIENS%20PREMATURITE/CSPS-ACCART-VILLE/RESPONSABLE%20CMU%20ACCART-VILLE.MP3time=1704800)

**Interviewer (00:26:15-6):** "Okay. As we conclude the interview, is there anything else you'd like to share about premature birth that we haven't covered through the questions?"

**Person 1 (00:26:31-4):** "I think we've covered everything [laughs]."

**Interviewer (00:26:34-8):** "Now, I'd like to ask about the definition. Could you clarify when we can speak of pregnancy and premature birth? Also, does abortion fit into this categorization, and are there exceptions that impact healthcare workers' duties?"

**Person 1 (00:27:01-2):** "In terms of prematurity, it's defined as a gestational age between 28 weeks and less than 37 weeks. Births before 28 weeks are considered late abortions. From my experience, babies born before 28 weeks may initially seem viable, but continuing care is difficult due to inadequate resources and technical support. Although these children may initially survive, they often succumb after a few days or weeks due to lack of adequate care."

**Interviewer (00:28:12-5):** "Thank you for your participation in this interview."

**Person 1 (00:28:14-6):** "Thank you as well."

**Interviewer (00:28:17-7):** "I hope the information shared today will help improve various aspects related to premature birth."

### Maternity manager of Accart-ville urban medical center

[00:31:07-4](http://localhost:2300/file=C:/Users/USER/Desktop/ENTRETIENS%20PREMATURITE/CSPS-ACCART-VILLE/RESPONSABLE%20MATERNITE%20CMU%20ACCART-VILLE.MP3time=1867400)Interviewer: maintenant audélà de tout ce qu'on a parcouru comme echange autour de la naissance prématurée en milieu urbain au Burkina faso, est ce qu'il y a des aspects que nous n'avons pas touchés et qui vous tiennent à coeurs et que vous souhaiterez partager avec nous? [00:31:19-9](http://localhost:2300/file=C:/Users/USER/Desktop/ENTRETIENS%20PREMATURITE/CSPS-ACCART-VILLE/RESPONSABLE%20MATERNITE%20CMU%20ACCART-VILLE.MP3time=1879900)

[00:31:19-9](http://localhost:2300/file=C:/Users/USER/Desktop/ENTRETIENS%20PREMATURITE/CSPS-ACCART-VILLE/RESPONSABLE%20MATERNITE%20CMU%20ACCART-VILLE.MP3time=1879900) Person 1: je trouve qu'au niveau du questionnaire l'accent n'a pas été mis sur les causes, les étiologies les facteurs de risque de la prematurité et la prise en charge de la prematurité.l'impact de la prematurité sur la femme est un facteur qui n'a pas été abordé, pourtant la femme sera contrainte de laisser toutes ses activités pour rester à la maison ce qui va jouer sur ses sources de revenues de la mère du prematuré. je pense que tous ses aspects peuvent contribuer à bouster la prise en charge des nouveaux-nées prematurés. [00:32:45-0](http://localhost:2300/file=C:/Users/USER/Desktop/ENTRETIENS%20PREMATURITE/CSPS-ACCART-VILLE/RESPONSABLE%20MATERNITE%20CMU%20ACCART-VILLE.MP3time=1965000)

[00:32:45-0](http://localhost:2300/file=C:/Users/USER/Desktop/ENTRETIENS%20PREMATURITE/CSPS-ACCART-VILLE/RESPONSABLE%20MATERNITE%20CMU%20ACCART-VILLE.MP3time=1965000) Interviewer: je vous remercie beaucoup, nous sommes donc au terme de notre entretien [00:32:48-2](http://localhost:2300/file=C:/Users/USER/Desktop/ENTRETIENS%20PREMATURITE/CSPS-ACCART-VILLE/RESPONSABLE%20MATERNITE%20CMU%20ACCART-VILLE.MP3time=1968200)

00:31:07-4 Interviewer: Maintenant, au-delà de tout ce que nous avons parcouru comme échange autour de la naissance prématurée en milieu urbain au Burkina Faso, est-ce qu'il y a des aspects que nous n'avons pas touchés et qui vous tiennent à cœur et que vous souhaiteriez partager avec nous ?

00:31:19-9 Person 1: Je trouve qu'au niveau du questionnaire, l'accent n'a pas été mis sur les causes, les étiologies, les facteurs de risque de la prématurité et la prise en charge de la prématurité. L'impact de la prématurité sur la femme est un facteur qui n'a pas été abordé, pourtant la femme sera contrainte de laisser toutes ses activités pour rester à la maison, ce qui va jouer sur ses sources de revenus en tant que mère du prématuré. Je pense que tous ces aspects peuvent contribuer à booster la prise en charge des nouveau-nés prématurés.

00:32:45-0 Interviewer: Je vous remercie beaucoup, nous sommes donc au terme de notre entretien.

**Interviewer (00:31:07-4):** "As we conclude our discussion on premature birth in an urban environment in Burkina Faso, are there any aspects that we haven't touched upon but are important to you and you'd like to share?"

**Person 1 (00:31:19-9):** "In the questionnaire, we didn't focus much on the causes, etiologies, and risk factors of prematurity, nor on its management. One overlooked impact is on the mother of the premature child. She often has to abandon her activities to care for her child, which affects her income sources. Addressing these aspects could significantly enhance the care of premature newborns."

**Interviewer (00:32:45-0):** "Thank you very much for your input. We have now reached the end of our interview.

### Focus group of Do medical center with surgical branch

[00:27:53-8](http://localhost:2300/file=C:/Users/USER/Desktop/ENTRETIENS%20PREMATURITE/CMA-DO/FOCUS%20GROUPE%20CMA-DO.mp3time=1673800) Interviewer: Je pense que sommes au terme de l'entretien, est ce q'il y a des aspects qui vous tiennent à coeurs que nous n'avons pas abordés et que vous souhaiterez évoquer? [00:28:05-9](http://localhost:2300/file=C:/Users/USER/Desktop/ENTRETIENS%20PREMATURITE/CMA-DO/FOCUS%20GROUPE%20CMA-DO.mp3time=1685900)

[00:28:05-9](http://localhost:2300/file=C:/Users/USER/Desktop/ENTRETIENS%20PREMATURITE/CMA-DO/FOCUS%20GROUPE%20CMA-DO.mp3time=1685900) Person 9: Je pense que c'est renforcer notre service, si vous avez la capacité l'unité kangourou qui s'occupe déjà de ses jeunes enfants là, peut être par l'octroi des offres, tout ce qui concours à l'entretien de cette unité , en personnel cela va être difficile mais en formation oui , ce serai mieux de renforcer cette unité en formation [00:28:29-9](http://localhost:2300/file=C:/Users/USER/Desktop/ENTRETIENS%20PREMATURITE/CMA-DO/FOCUS%20GROUPE%20CMA-DO.mp3time=1709900)

[00:28:29-9](http://localhost:2300/file=C:/Users/USER/Desktop/ENTRETIENS%20PREMATURITE/CMA-DO/FOCUS%20GROUPE%20CMA-DO.mp3time=1709900) Interviewer: Est ce qu'il y a quelque chose d'autre à ajouter? [00:28:33-6](http://localhost:2300/file=C:/Users/USER/Desktop/ENTRETIENS%20PREMATURITE/CMA-DO/FOCUS%20GROUPE%20CMA-DO.mp3time=1713600)

[00:28:33-6](http://localhost:2300/file=C:/Users/USER/Desktop/ENTRETIENS%20PREMATURITE/CMA-DO/FOCUS%20GROUPE%20CMA-DO.mp3time=1713600) Person 4: Bon. Je pense que dans l'ensemble, beaucoup d'éléments sont ressortis. Mais maintenant, comme elle l'a dit, un personnel bien formé qui travaille avec du matériel dérisoire, avec des ruptures, forcément, il y aura un impact. Un personnel bien formé,

sans motivation, ou sous-motivé. La motivation n'est pas forcément pécunière. Mais elle est importante aussi, l'aspect pecunière. C'est sûr qu'il y aura un impact sur les activités. C'est tout ça, l'un dans l'autre en tenant compte de tous ces aspects. Parce que si on se focalise sur un seul aspect, par exemple, on amène du matériel de façon au top, mais on a du personnel qui ne sait pas peut-être l'utiliser. Ou bien, on amène du matériel, le personnel, qui n'est pas motivé. Voilà. Donc c'est compliqué. Ça va impacter, forcément. Donc c'est de regarder tous ces aspects. Pas à 100%, mais un peu de ceci, un peu de cela (rire). "Pour sauver notre nez" (rire). Je pense que ça pourra faire l'affaire [00:29:56-5](http://localhost:2300/file=C:/Users/USER/Desktop/ENTRETIENS%20PREMATURITE/CMA-DO/FOCUS%20GROUPE%20CMA-DO.mp3time=1796500)

[00:29:56-5](http://localhost:2300/file=C:/Users/USER/Desktop/ENTRETIENS%20PREMATURITE/CMA-DO/FOCUS%20GROUPE%20CMA-DO.mp3time=1796500) Interviewer: C'est une contribution, s'il y a d'autres élements qui vous viennent à l'esprit que vous souhaiterez partager, vous pouvez le faire. [00:30:03-2](http://localhost:2300/file=C:/Users/USER/Desktop/ENTRETIENS%20PREMATURITE/CMA-DO/FOCUS%20GROUPE%20CMA-DO.mp3time=1803200)

[00:30:03-2](http://localhost:2300/file=C:/Users/USER/Desktop/ENTRETIENS%20PREMATURITE/CMA-DO/FOCUS%20GROUPE%20CMA-DO.mp3time=1803200) Person 9: C'est un plaidoyer pour qu'on ai un service de néonatologie vraiment . [00:30:07-3](http://localhost:2300/file=C:/Users/USER/Desktop/ENTRETIENS%20PREMATURITE/CMA-DO/FOCUS%20GROUPE%20CMA-DO.mp3time=1807300)

[00:30:07-3](http://localhost:2300/file=C:/Users/USER/Desktop/ENTRETIENS%20PREMATURITE/CMA-DO/FOCUS%20GROUPE%20CMA-DO.mp3time=1807300) Person 6: En tout cas, doit avoir un service de néonatologie se serait vraiment bien. [00:30:10-2](http://localhost:2300/file=C:/Users/USER/Desktop/ENTRETIENS%20PREMATURITE/CMA-DO/FOCUS%20GROUPE%20CMA-DO.mp3time=1810200)

[00:30:10-2](http://localhost:2300/file=C:/Users/USER/Desktop/ENTRETIENS%20PREMATURITE/CMA-DO/FOCUS%20GROUPE%20CMA-DO.mp3time=1810200) Person 2: Je voulais ajouter quelque chose, en fait ce qu'ils ont dit c'est la réalité que nous vivons, par exemple moi je suis en salle d'accouchement, et je dois réanimer mon bébé, et le materiel que j'ai est defectueux. Je sais que cet enfant, si j'avais un bon ballon pour le ventiler, ou une bonne poire pour l'aspirer, je peux recuperer le bébé. Mais malheureusement, le matériel n'est pas bon et je vois ce bébé-là qui est en train de sombrer et sous mes bras, je fais comment? Donc ça veut dire qu'on a besoin de matériel de réanimation. Le matériel pour bien prendre soin de nos prématurés. Par exemple, un grand prématuré. Quand on le fait sortir, il y a tous les facteurs qui sont réunis pour que ce bébé-là parte dans les 5 minutes ou 10 minutes qui suivent l'accouchement. Mais si on a du matériel, en salle d'accouchement même, parce qu'ici, comme ils ont même à l'unité kangourou, pour prendre en charge le grand prématuré, c'est un peu difficile. Il faut l'envoyer au CHURSS.Mais d'ici même, le trajet, c'est pas evident que ce bébé-là. Le transfert, c'est pas evident que ce bébé arrive au CHURSS vivant. Voilà, donc il y a tous ces éléments qu'il faut voire. Donc vraiment, on a besoin même en salle d'accouchement, si vous rentrez tout de suite, on a des ballons, mais ces ballons pour ventiler ne sont pas bons. Donc, vous êtes là, vous pompez, vous sentez que l'air sort de partout. Est-ce qu'on peut y animer un enfant correctement avec ça? C'est difficile. Donc on a besoin du matériel. C'est vraiment un plaidoyer. On a vraiment besoin du matériel pour prendre en charge nos prématurés et pour réanimer nos bébés. [00:32:12-8](http://localhost:2300/file=C:/Users/USER/Desktop/ENTRETIENS%20PREMATURITE/CMA-DO/FOCUS%20GROUPE%20CMA-DO.mp3time=1932800)

[00:32:12-8](http://localhost:2300/file=C:/Users/USER/Desktop/ENTRETIENS%20PREMATURITE/CMA-DO/FOCUS%20GROUPE%20CMA-DO.mp3time=1932800) Interviewer: Merci beaucoup. Est-ce qu'il y a des choses que vous souhaitez ajouter? Je vous remercie beaucoup d'avoir participé à cet entretien. J'espère que toutes les contributions vont aller dans les sens de l'amélioration de ces aspects liés à la prématurité au niveau du Burkina Faso, en général, je vous remercie. [00:32:43-1](http://localhost:2300/file=C:/Users/USER/Desktop/ENTRETIENS%20PREMATURITE/CMA-DO/FOCUS%20GROUPE%20CMA-DO.mp3time=1963100)

[00:32:43-1](http://localhost:2300/file=C:/Users/USER/Desktop/ENTRETIENS%20PREMATURITE/CMA-DO/FOCUS%20GROUPE%20CMA-DO.mp3time=1963100) Person 6: Merci. [00:32:44-2](http://localhost:2300/file=C:/Users/USER/Desktop/ENTRETIENS%20PREMATURITE/CMA-DO/FOCUS%20GROUPE%20CMA-DO.mp3time=1964200)

[00:32:44-2](http://localhost:2300/file=C:/Users/USER/Desktop/ENTRETIENS%20PREMATURITE/CMA-DO/FOCUS%20GROUPE%20CMA-DO.mp3time=1964200) Person 9: Merci

00:27:53-8 Interviewer: Je pense que nous sommes au terme de l'entretien. Est-ce qu'il y a des aspects qui vous tiennent à cœur que nous n'avons pas abordés et que vous souhaiteriez évoquer ?

00:28:05-9 Person 9: Je pense que c'est renforcer notre service. Si vous avez la capacité, l'unité kangourou qui s'occupe déjà de ces jeunes enfants-là, peut-être par l'octroi des offres, tout ce qui concourt à l'entretien de cette unité, en personnel, cela va être difficile, mais en formation, oui, ce serait mieux de renforcer cette unité en formation.

00:28:29-9 Interviewer: Est-ce qu'il y a quelque chose d'autre à ajouter ?

00:28:33-6 Person 4: Bon. Je pense que dans l'ensemble, beaucoup d'éléments sont ressortis. Mais maintenant, comme elle l'a dit, un personnel bien formé qui travaille avec du matériel dérisoire, avec des ruptures, forcément, il y aura un impact. Un personnel bien formé, sans motivation, ou sous-motivé. La motivation n'est pas forcément pécuniaire, mais elle est importante aussi, l'aspect pécuniaire. C'est sûr qu'il y aura un impact sur les activités. C'est tout ça, l'un dans l'autre, en tenant compte de tous ces aspects. Parce que si on se focalise sur un seul aspect, par exemple, on amène du matériel de façon au top, mais on a du personnel qui ne sait pas peut-être l'utiliser. Ou bien, on amène du matériel, le personnel, qui n'est pas motivé. Voilà. Donc c'est compliqué. Ça va impacter, forcément. Donc c'est de regarder tous ces aspects. Pas à 100%, mais un peu de ceci, un peu de cela (rire). "Pour sauver notre nez" (rire). Je pense que ça pourra faire l'affaire.

00:29:56-5 Interviewer: C'est une contribution, s'il y a d'autres éléments qui vous viennent à l'esprit que vous souhaiteriez partager, vous pouvez le faire.

00:30:03-2 Person 9: C'est un plaidoyer pour qu'on ait un service de néonatologie vraiment.

00:30:07-3 Person 6: En tout cas, il doit y avoir un service de néonatologie. Ce serait vraiment bien.

00:30:10-2 Person 2: Je voulais ajouter quelque chose, en fait ce qu'ils ont dit, c'est la réalité que nous vivons. Par exemple, moi je suis en salle d'accouchement, et je dois réanimer mon bébé, et le matériel que j'ai est défectueux. Je sais que cet enfant, si j'avais un bon ballon pour le ventiler, ou une bonne poire pour l'aspirer, je peux récupérer le bébé. Mais malheureusement, le matériel n'est pas bon et je vois ce bébé-là qui est en train de sombrer sous mes bras, je fais comment ? Donc ça veut dire qu'on a besoin de matériel de réanimation. Le matériel pour bien prendre soin de nos prématurés. Par exemple, un grand prématuré. Quand on le fait sortir, il y a tous les facteurs qui sont réunis pour que ce bébé-là parte dans les 5 minutes ou 10 minutes qui suivent l'accouchement. Mais si on a du matériel, en salle d'accouchement même, parce qu'ici, comme ils ont même à l'unité kangourou, pour prendre en charge le grand prématuré, c'est un peu difficile. Il faut l'envoyer au CHURSS. Mais d'ici même, le trajet, ce n'est pas évident que ce bébé-là. Le transfert, ce n'est pas évident que ce bébé arrive au CHURSS vivant. Voilà, donc il y a tous ces éléments qu'il faut voir. Donc vraiment, on a besoin même en salle d'accouchement, si vous rentrez tout de suite, on a des ballons, mais ces ballons pour ventiler ne sont pas bons. Donc, vous êtes là, vous pompez, vous sentez que l'air sort de partout. Est-ce qu'on peut animer un enfant correctement avec ça ? C'est difficile. Donc on a besoin du matériel. C'est vraiment un plaidoyer. On a vraiment besoin du matériel pour prendre en charge nos prématurés et pour réanimer nos bébés.

00:32:12-8 Interviewer: Merci beaucoup. Est-ce qu'il y a des choses que vous souhaitez ajouter ? Je vous remercie beaucoup d'avoir participé à cet entretien. J'espère que toutes les contributions vont aller dans le sens de l'amélioration de ces aspects liés à la prématurité au Burkina Faso. En général, je vous remercie.

00:32:43-1 Person 6: Merci.

00:32:44-2 Person 9: Merci.

**00:27:53-8 Interviewer:** As we're coming to the end of the interview, are there any important aspects that we haven't covered which you would like to discuss?

**00:28:05-9 Person 9:** Strengthening our service, especially the kangaroo unit that cares for young children, is crucial. While it might be challenging in terms of personnel, focusing on training to enhance this unit would be beneficial.

**00:28:33-6 Person 4:** It's essential to consider all aspects. Having well-trained staff without adequate equipment, or having motivated staff with subpar facilities, will impact the quality of care. The motivation of staff isn’t only about money; the financial aspect, however, is also important. We need a balanced approach, not focusing solely on one aspect but addressing equipment, training, and motivation collectively.

**00:30:07-3 Person 6:** Establishing a neonatology department would be extremely beneficial for us.

**00:30:10-2 Person 2:** In my experience, especially in the delivery room, having defective equipment is a significant challenge. If we had better resuscitation equipment, many babies could be saved. Currently, the lack of quality equipment means we are unable to effectively resuscitate and care for premature babies. This is particularly true for very premature infants, who often don't survive the transfer to better-equipped facilities. We desperately need better equipment in the delivery room for immediate care and resuscitation of newborns.

**00:32:43-1 Interviewer:** Thank you for your contributions. I hope they will help improve the management of prematurity in Burkina Faso. Thank you for participating in this interview.

**00:32:44-2 Person 6 & 9:** Thank you.

### Gynecologist 1

[00:17:39-2](http://localhost:2300/file=C:/Users/USER/Desktop/ENTRETIENS%20PREMATURITE/CMA-DO/GENYCOLOGUE%2001.mp3time=1059200) Interviewer: Je pense que nous sommes au terme de l'entretien. Je ne sais pas s'il y a des sujets auxquels on n'a pas abordé qui vous tiennent à coeur par rapport à la situation de la prématurité et auxquels vous souhaiteriez aborder. [00:17:53-3](http://localhost:2300/file=C:/Users/USER/Desktop/ENTRETIENS%20PREMATURITE/CMA-DO/GENYCOLOGUE%2001.mp3time=1073300)

[00:17:53-3](http://localhost:2300/file=C:/Users/USER/Desktop/ENTRETIENS%20PREMATURITE/CMA-DO/GENYCOLOGUE%2001.mp3time=1073300) Person 1: En fait, surtout ce qui me tient à coeur, on en a déjà un peu parlé, c'est de pouvoir vraiment décentraliser la prise en charge. Comme je l'ai dit, on a une seule unité de neonatologie dans toute la région des Hauts-Bassins.

Ça veut dire que tous les districts, Hounde, Ourodara, Dô,Dafra, Léna, Karangasso Sambla, tout le monde envoit.Ndorola, tout le monde envoit. Si vraiment on peut déjà commencer à décentraliser,parce que la seule unité de Souro SANOU ne peut pas. Si nous ne pouvons pas mettre dans les milieux rurals, déjà peut-être à DO,Dafra, songez à faire des unités de neonatologie. Ça va désengorger le Souro SANOU et ça va donner plus de chance à ces enfants. [00:18:42-3](http://localhost:2300/file=C:/Users/USER/Desktop/ENTRETIENS%20PREMATURITE/CMA-DO/GENYCOLOGUE%2001.mp3time=1122300)

[00:18:42-3](http://localhost:2300/file=C:/Users/USER/Desktop/ENTRETIENS%20PREMATURITE/CMA-DO/GENYCOLOGUE%2001.mp3time=1122300) Interviewer: Je vous remercie infiniment pour ses éléments de reponses. [00:18:44-5](http://localhost:2300/file=C:/Users/USER/Desktop/ENTRETIENS%20PREMATURITE/CMA-DO/GENYCOLOGUE%2001.mp3time=1124500)

[00:18:44-5](http://localhost:2300/file=C:/Users/USER/Desktop/ENTRETIENS%20PREMATURITE/CMA-DO/GENYCOLOGUE%2001.mp3time=1124500) Person 1: Pas de quoi. [00:18:48-4](http://localhost:2300/file=C:/Users/USER/Desktop/ENTRETIENS%20PREMATURITE/CMA-DO/GENYCOLOGUE%2001.mp3time=1128400)

00:17:39-2 Interviewer: Je pense que nous sommes au terme de l'entretien. Je ne sais pas s'il y a des sujets auxquels on n'a pas abordé qui vous tiennent à cœur par rapport à la situation de la prématurité et auxquels vous souhaiteriez aborder.

00:17:53-3 Person 1: En fait, surtout ce qui me tient à cœur, on en a déjà un peu parlé, c'est de pouvoir vraiment décentraliser la prise en charge. Comme je l'ai dit, on a une seule unité de néonatologie dans toute la région des Hauts-Bassins. Ça veut dire que tous les districts, Houndé, Ourodara, Dô, Dafra, Léna, Karangasso Sambla, tout le monde envoie. Ndorola, tout le monde envoie. Si vraiment on peut déjà commencer à décentraliser, parce que la seule unité de Souro SANOU ne peut pas. Si nous ne pouvons pas mettre dans les milieux ruraux, déjà peut-être à Dô, Dafra, songez à faire des unités de néonatologie. Ça va désengorger le Souro SANOU et ça va donner plus de chance à ces enfants.

00:18:42-3 Interviewer: Je vous remercie infiniment pour ces éléments de réponses.

00:18:44-5 Person 1: Pas de quoi.

**Interviewer (00:17:39-2):** "I think we're nearing the end of our interview. Are there any important topics related to prematurity that we haven't discussed yet, which you'd like to address?"

**Person 1 (00:17:53-3):** "What's important to me, and we've touched on it a bit, is the need for decentralizing support for premature births. As I mentioned, in the entire Hauts-Bassins region, we only have one neonatology unit. This unit serves all districts, including Houndé, Ourodara, Dô, Dafra, Léna, Karangasso Sambla, Ndorola, and others. If we could start decentralizing these services, perhaps even creating neonatology units in places like Dô and Dafra, it would greatly alleviate the burden on Souro SANOU and provide more opportunities for these children."

**Interviewer (00:18:42-3):** "Thank you very much for your insightful answers."

**Person 1 (00:18:44-5):** "No problem.

### Gynecologist 2

[00:31:02-6](http://localhost:2300/file=C:/Users/USER/Desktop/ENTRETIENS%20PREMATURITE/CMA-DO/GENYCOLOGUE%2002.mp3time=1862600) Interviewer: Je pense que nous sommes au terme de l'entrtien, mais est ce qu'il y a certains aspects qui vous tient à coeurs et que nous n'avons pas abordés? [00:31:11-9](http://localhost:2300/file=C:/Users/USER/Desktop/ENTRETIENS%20PREMATURITE/CMA-DO/GENYCOLOGUE%2002.mp3time=1871900)

[00:31:11-9](http://localhost:2300/file=C:/Users/USER/Desktop/ENTRETIENS%20PREMATURITE/CMA-DO/GENYCOLOGUE%2002.mp3time=1871900) Person 1: Je pense qu'on a abordé l'essentiel, mais ce sur quoi je vais revenir c'est nos équipements pour la prise en charge des enfants de façon générale et particulièrement les prématurés. Notre unité kangourou fonctionne quelques heures dans la journée et dans la soirée, ils n'ont pas assez de personnel et on n'a pas assez d'équipement. En fait c'est ce qu'on fait ici, c'est ce que j'ai expliqué, c'est juste, ce n'est pas négligeable mais on peut faire mieux. On va donner des conseils par rapport à comment mettre l'enfant au chaud, comment alimenter l'enfant de façon convenable jusqu'à ce qu'il prenne du poids. Donc on ne l'hospitalise pas. Donc les nouveaux-nés prématurés malades, on ne peut pas les recevoir ici. Donc déjà c'est un déficit. Il faut envoyer les enfants à l'hôpital et souvent l'hôpital est débordé parce que les enfants prématurés arrivent de toutes les structures. Ils arrivent de Banfora, les naissances de Bobo, Gaoua, souvent, donc les enfants prématurés vont arriver ici, déjà la ville de Bobo même seule, suffit à remplir l'unité de neonatologique à l'hôpital. Donc si on prend les autres structures qui avoisines, s'elles vont envoyer des enfants prématurés ici ou bien les grossesses avec des problèmes, l'enfant va naître prématuré, on va l'envoyer, donc ça devient un autre gros souci. Mais si déjà c'est décentralisé, si au CMA de DÔ on peut prendre en charge correctement ou bien si on a une unité de pédiatrie, ça ne peut qu'améliorer la prise en charge. [00:32:39-8](http://localhost:2300/file=C:/Users/USER/Desktop/ENTRETIENS%20PREMATURITE/CMA-DO/GENYCOLOGUE%2002.mp3time=1959800)

[00:32:39-8](http://localhost:2300/file=C:/Users/USER/Desktop/ENTRETIENS%20PREMATURITE/CMA-DO/GENYCOLOGUE%2002.mp3time=1959800) Interviewer: Je vous remercie bien docteur. [00:32:41-2](http://localhost:2300/file=C:/Users/USER/Desktop/ENTRETIENS%20PREMATURITE/CMA-DO/GENYCOLOGUE%2002.mp3time=1961200)

[00:32:41-2](http://localhost:2300/file=C:/Users/USER/Desktop/ENTRETIENS%20PREMATURITE/CMA-DO/GENYCOLOGUE%2002.mp3time=1961200) Person 1: Je vous remercie également. Merci. [00:32:45-8](http://localhost:2300/file=C:/Users/USER/Desktop/ENTRETIENS%20PREMATURITE/CMA-DO/GENYCOLOGUE%2002.mp3time=1965800)

00:31:02-6 Interviewer: Je pense que nous sommes au terme de l'entretien, mais est-ce qu'il y a certains aspects qui vous tiennent à cœur et que nous n'avons pas abordés ?

00:31:11-9 Person 1: Je pense qu'on a abordé l'essentiel, mais ce sur quoi je vais revenir c'est nos équipements pour la prise en charge des enfants de façon générale et particulièrement les prématurés. Notre unité kangourou fonctionne quelques heures dans la journée et dans la soirée, ils n'ont pas assez de personnel et on n'a pas assez d'équipement. En fait c'est ce qu'on fait ici, c'est ce que j'ai expliqué, c'est juste, ce n'est pas négligeable mais on peut faire mieux. On va donner des conseils par rapport à comment mettre l'enfant au chaud, comment alimenter l'enfant de façon convenable jusqu'à ce qu'il prenne du poids. Donc on ne l'hospitalise pas. Donc les nouveau-nés prématurés malades, on ne peut pas les recevoir ici. Donc déjà c'est un déficit. Il faut envoyer les enfants à l'hôpital et souvent l'hôpital est débordé parce que les enfants prématurés arrivent de toutes les structures. Ils arrivent de Banfora, les naissances de Bobo, Gaoua, souvent, donc les enfants prématurés vont arriver ici, déjà la ville de Bobo même seule, suffit à remplir l'unité de néonatologie à l'hôpital. Donc si on prend les autres structures qui avoisinent, s'elles vont envoyer des enfants prématurés ici ou bien les grossesses avec des problèmes, l'enfant va naître prématuré, on va l'envoyer, donc ça devient un autre gros souci. Mais si déjà c'est décentralisé, si au CMA de DÔ on peut prendre en charge correctement ou bien si on a une unité de pédiatrie, ça ne peut qu'améliorer la prise en charge.

00:32:39-8 Interviewer: Je vous remercie bien, docteur.

00:32:41-2 Person 1: Je vous remercie également. Merci

**Interviewer (00:31:02-6):** "As we conclude our interview, are there any important aspects we haven't yet discussed that you'd like to mention?"

**Person 1 (00:31:11-9):** "I believe we've covered the essentials. However, I'd like to revisit our equipment for caring for children, especially premature babies. Our kangaroo unit only operates for a few hours during the day. In the evening, we're short-staffed and lack adequate equipment. While our current approach, including advice on keeping the child warm and properly fed until they gain weight, is fair, there's room for improvement. We're not equipped to hospitalize sick premature newborns, which is a significant shortcoming. We often have to send these children to the hospital, but the hospital is frequently overwhelmed with premature babies from various regions. Bobo alone can fill the neonatology unit. If other areas also send their cases here, it exacerbates the problem. Decentralizing care, perhaps by establishing a pediatric unit at the CMA de Dô, could greatly improve our ability to provide proper care."

**Interviewer (00:32:39-8):** "Thank you very much for your insights, doctor."

**Person 1 (00:32:41-2):** "Thank you as well. You're welcome."

### Chief physician of Do health district

[00:31:03-2](http://localhost:2300/file=C:/Users/USER/Desktop/ENTRETIENS%20PREMATURITE/CMA-DO/MCD-DO.mp3time=1863200) Interviewer: D'accord. Au-delà de tout ce que nous venons de parcourir, est-ce qu'il y a des aspects particuliers qu'on n'a pas abordés, qui vous tiennent à cœur par rapport à la naissance prématurée et aux défis auxquels vous faites face ici, que vous souhaiteriez partager? [00:31:16-7](http://localhost:2300/file=C:/Users/USER/Desktop/ENTRETIENS%20PREMATURITE/CMA-DO/MCD-DO.mp3time=1876700)

[00:31:16-7](http://localhost:2300/file=C:/Users/USER/Desktop/ENTRETIENS%20PREMATURITE/CMA-DO/MCD-DO.mp3time=1876700) Person 1: Je crois qu'on a parcouru. Ce qui nous importe, nous, c'est si on a des équipements pour répondre, on trouvera le personnel, on formera le personnel ensemble. Ce sont les équipements qui manquent. C'est un peu ça aussi. Je crois qu'on a des gynécologues. on a un attaché en pédiatrie. J'attends un pédiatrie qui va arriver le prochain mois. Donc, on peut dire que ça doit pouvoir répondre. Ce sont les équipements. Bon, le personnel qui est là. Les capacités. Parce que la routine, finit par faire perdre certaines bonnes pratiques. C'est surtout ça. [00:32:01-5](http://localhost:2300/file=C:/Users/USER/Desktop/ENTRETIENS%20PREMATURITE/CMA-DO/MCD-DO.mp3time=1921500)

[00:32:01-5](http://localhost:2300/file=C:/Users/USER/Desktop/ENTRETIENS%20PREMATURITE/CMA-DO/MCD-DO.mp3time=1921500) Interviewer: Merci beaucoup. Je pense que nous sommes au terme. [00:32:04-2](http://localhost:2300/file=C:/Users/USER/Desktop/ENTRETIENS%20PREMATURITE/CMA-DO/MCD-DO.mp3time=1924200)

[00:32:04-2](http://localhost:2300/file=C:/Users/USER/Desktop/ENTRETIENS%20PREMATURITE/CMA-DO/MCD-DO.mp3time=1924200) Person 1: Merci à vous [00:32:05-0](http://localhost:2300/file=C:/Users/USER/Desktop/ENTRETIENS%20PREMATURITE/CMA-DO/MCD-DO.mp3time=1925000)

00:31:03-2 Interviewer: D'accord. Au-delà de tout ce que nous venons de parcourir, est-ce qu'il y a des aspects particuliers qu'on n'a pas abordés, qui vous tiennent à cœur par rapport à la naissance prématurée et aux défis auxquels vous faites face ici, que vous souhaiteriez partager ? 00:31:16-7

00:31:16-7 Personne 1: Je crois qu'on a parcouru. Ce qui nous importe, nous, c'est si on a des équipements pour répondre, on trouvera le personnel, on formera le personnel ensemble. Ce sont les équipements qui manquent. C'est un peu ça aussi. Je crois qu'on a des gynécologues. On a un attaché en pédiatrie. J'attends un pédiatre qui va arriver le prochain mois. Donc, on peut dire que ça doit pouvoir répondre. Ce sont les équipements. Bon, le personnel qui est là. Les capacités. Parce que la routine finit par faire perdre certaines bonnes pratiques. C'est surtout ça. 00:32:01-5

00:32:01-5 Interviewer: Merci beaucoup. Je pense que nous sommes au terme. 00:32:04-2

00:32:04-2 Personne 1: Merci à vous. 00:32:05-0

**00:31:03-2 Interviewer:** Okay. Beyond everything we've discussed, are there any other important aspects related to premature birth and the challenges you face in your health center that we haven't covered? Anything else you'd like to share?

**00:31:16-7 Person 1:** I think we've covered the main points. The primary issue for us is the lack of equipment. If we have the necessary equipment, we can find and train the staff. That's our biggest limitation right now. We do have gynecologists and are expecting a pediatrician next month, so we have the professional capabilities. It’s really about having the right equipment and maintaining our skills since routine work can sometimes lead to the loss of certain best practices. That's the main area of focus for us.

**00:32:01-5 Interviewer:** Thank you very much for your insights. I believe we've covered all the essential points.

**00:32:04-2 Person 1:** Thank you.

### Manager for maternity care unit of Do medical center with surgical branch

[00:28:32-0](http://localhost:2300/file=C:/Users/USER/Desktop/ENTRETIENS%20PREMATURITE/CMA-DO/RESPONSABLE%20UNITE%20DE%20SOIN%20CMA-DO.mp3time=1712000) Interviewer: Je pense que nous sommes au terme de l'entretien. Maintenant, je ne sais pas s'il y a des sujets ou des aspects auxquels vous tenez, mais qu'on n'a pas abordé, donc tout au long de cet entretien. [00:28:41-3](http://localhost:2300/file=C:/Users/USER/Desktop/ENTRETIENS%20PREMATURITE/CMA-DO/RESPONSABLE%20UNITE%20DE%20SOIN%20CMA-DO.mp3time=1721300)

[00:28:41-3](http://localhost:2300/file=C:/Users/USER/Desktop/ENTRETIENS%20PREMATURITE/CMA-DO/RESPONSABLE%20UNITE%20DE%20SOIN%20CMA-DO.mp3time=1721300) Person 1: Je pense que tout a été abordé, tout a été dit. Voilà. Mais seulement peut-être que l'enregistrement là, bon, la question m'a amené peut-être à dire, je vais revoir le registre là pour voir. Parce que normalement, la hauteur utérine notifiée là-bas est un élément, mais pas un élément tout fait. Parce que tu peux avoir, comme j'ai dit là, faible poids de naissance. Donc, tu peux rentrer dans une hauteur utérine que tu vas prendre, tu vas trouver que c'est petit par rapport à l'âge de la grossesse. Mais également à la naissance, qu'on puisse avoir vraiment tous les éléments nécessaires. C'est-à-dire pouvoir examiner. Mais ça me renvoie encore à cette question de formation, des agents, sur la prise en charge peut-être de nouveau des plus maturés. Et même la réalisation de l'accouchement, si ça doit se faire. Voilà. Comment on doit vraiment prendre en charge ces nouveaux-nés-là. Et également, les conseils à donner à la mère. [00:29:37-7](http://localhost:2300/file=C:/Users/USER/Desktop/ENTRETIENS%20PREMATURITE/CMA-DO/RESPONSABLE%20UNITE%20DE%20SOIN%20CMA-DO.mp3time=1777700)

[00:29:37-7](http://localhost:2300/file=C:/Users/USER/Desktop/ENTRETIENS%20PREMATURITE/CMA-DO/RESPONSABLE%20UNITE%20DE%20SOIN%20CMA-DO.mp3time=1777700) Interviewer: Une formation assez spécifique sur la prématurité? [00:29:38-6](http://localhost:2300/file=C:/Users/USER/Desktop/ENTRETIENS%20PREMATURITE/CMA-DO/RESPONSABLE%20UNITE%20DE%20SOIN%20CMA-DO.mp3time=1778600)

[00:29:38-6](http://localhost:2300/file=C:/Users/USER/Desktop/ENTRETIENS%20PREMATURITE/CMA-DO/RESPONSABLE%20UNITE%20DE%20SOIN%20CMA-DO.mp3time=1778600) Person 1: Voilà. C'est vrai qu'à l'école de base, oui, on a eu ça. Mais je pense qu'on doit encore toujours revenir sur ça, sur le terrain. Voilà. Pour amener les gens. Sinon, c'est pas noté. Le registre, en tout cas, ne nous permet pas de collecter. Voilà. C'est donné là. [00:29:56-7](http://localhost:2300/file=C:/Users/USER/Desktop/ENTRETIENS%20PREMATURITE/CMA-DO/RESPONSABLE%20UNITE%20DE%20SOIN%20CMA-DO.mp3time=1796700)

[00:29:56-7](http://localhost:2300/file=C:/Users/USER/Desktop/ENTRETIENS%20PREMATURITE/CMA-DO/RESPONSABLE%20UNITE%20DE%20SOIN%20CMA-DO.mp3time=1796700)Interviewer: Merci beaucoup. [00:29:58-0](http://localhost:2300/file=C:/Users/USER/Desktop/ENTRETIENS%20PREMATURITE/CMA-DO/RESPONSABLE%20UNITE%20DE%20SOIN%20CMA-DO.mp3time=1798000)

[00:29:58-0](http://localhost:2300/file=C:/Users/USER/Desktop/ENTRETIENS%20PREMATURITE/CMA-DO/RESPONSABLE%20UNITE%20DE%20SOIN%20CMA-DO.mp3time=1798000) Person 1: Merci également à vous. [00:30:00-4](http://localhost:2300/file=C:/Users/USER/Desktop/ENTRETIENS%20PREMATURITE/CMA-DO/RESPONSABLE%20UNITE%20DE%20SOIN%20CMA-DO.mp3time=1800400)

00:28:32-0 Interviewer: Je pense que nous sommes au terme de l'entretien. Maintenant, je ne sais pas s'il y a des sujets ou des aspects auxquels vous tenez, mais qu'on n'a pas abordés, donc tout au long de cet entretien.

00:28:41-3 Person 1: Je pense que tout a été abordé, tout a été dit. Voilà. Mais seulement peut-être que l'enregistrement là, bon, la question m'a amené peut-être à dire, je vais revoir le registre là pour voir. Parce que normalement, la hauteur utérine notifiée là-bas est un élément, mais pas un élément tout fait. Parce que tu peux avoir, comme j'ai dit là, faible poids de naissance. Donc, tu peux rentrer dans une hauteur utérine que tu vas prendre, tu vas trouver que c'est petit par rapport à l'âge de la grossesse. Mais également à la naissance, qu'on puisse avoir vraiment tous les éléments nécessaires. C'est-à-dire pouvoir examiner. Mais ça me renvoie encore à cette question de formation, des agents, sur la prise en charge peut-être de nouveau des plus maturés. Et même la réalisation de l'accouchement, si ça doit se faire. Voilà. Comment on doit vraiment prendre en charge ces nouveau-nés-là. Et également, les conseils à donner à la mère.

00:29:37-7 Interviewer: Une formation assez spécifique sur la prématurité ?

00:29:38-6 Person 1: Voilà. C'est vrai qu'à l'école de base, oui, on a eu ça. Mais je pense qu'on doit encore toujours revenir sur ça, sur le terrain. Voilà. Pour amener les gens. Sinon, c'est pas noté. Le registre, en tout cas, ne nous permet pas de collecter. Voilà. C'est donné là.

00:29:56-7

Interviewer: Merci beaucoup.

00:29:58-0 Person 1: Merci également à vous.

**Interviewer (00:28:32-0):** "As we're concluding our interview, are there any topics or aspects important to you that we haven't discussed?"

**Person 1 (00:28:41-3):** "I believe we've covered everything. However, I'd like to revisit the topic of fundal height, which is important but not definitive for assessing birth weight. Sometimes, the uterine height may seem small relative to the pregnancy's age. Accurate evaluation really requires examining the newborn at birth. This brings me to the importance of training healthcare workers in managing more mature births and providing the right care and advice to mothers. While we did cover this in basic training, it's something that needs to be revisited and reinforced in practice."

**Interviewer (00:29:37-7):** "So, specific training on prematurity is what you're suggesting?"

**Person 1 (00:29:38-6):** "Exactly. Although it was part of our basic education, it's crucial to continually update this knowledge on the field. Otherwise, the register doesn't capture this information adequately."

**Interviewer (00:29:56-7):** "Thank you very much for your insights."

**Person 1 (00:29:58-0):** "Thank you as well."

### Focus group of Colma1 health and social promotion center

[00:35:13-7](http://localhost:2300/file=C:/Users/USER/Desktop/ENTRETIENS%20PREMATURITE/CSPS-COLMA1/FOCUS%20GROUPE%20COLMA1.mp3time=2113700) Interviewer: Je vous remercie pour votre disponibilité cela a été très enrichissant j'espère que toutes les recommandations et les avis donnés vont contribuer à ameliorer la prise en charge des naissances prématurées au burkina faso. Merci bien [00:35:33-2](http://localhost:2300/file=C:/Users/USER/Desktop/ENTRETIENS%20PREMATURITE/CSPS-COLMA1/FOCUS%20GROUPE%20COLMA1.mp3time=2133200)

00:35:13-7 Interviewer: Je vous remercie pour votre disponibilité, cela a été très enrichissant. J'espère que toutes les recommandations et les avis donnés vont contribuer à améliorer la prise en charge des naissances prématurées au Burkina Faso. Merci bien. 00:35:33-2

**Interviewer (00:35:13-7):** "Thank you for your time and valuable contributions. It has been a very enlightening discussion. I hope that all the recommendations and insights shared will help enhance the care of premature births in Burkina Faso. Thank you very much."

### Major of Colma1 health and social promotion center

[00:23:15-9](http://localhost:2300/file=C:/Users/USER/Desktop/ENTRETIENS%20PREMATURITE/CSPS-COLMA1/MAJOR%20CSPS%20COLMA1.MP3time=1395900) Interviewer: Je pense que nous avons un peu parcouru toutes les questions maintenant, au-delà des questions que nous avons posée, est-ce qu'il y a un point sur lequel vous aimeriez échanger que nous n'avons pas abordé par rapport à la question sur la naissance prématurée ? [00:23:34-9](http://localhost:2300/file=C:/Users/USER/Desktop/ENTRETIENS%20PREMATURITE/CSPS-COLMA1/MAJOR%20CSPS%20COLMA1.MP3time=1414900)

[00:23:34-9](http://localhost:2300/file=C:/Users/USER/Desktop/ENTRETIENS%20PREMATURITE/CSPS-COLMA1/MAJOR%20CSPS%20COLMA1.MP3time=1414900) Person 1: par rapport à la naissance prématurée, non, bien non, je ne vois pas, je ne vois pas. [00:23:38-6](http://localhost:2300/file=C:/Users/USER/Desktop/ENTRETIENS%20PREMATURITE/CSPS-COLMA1/MAJOR%20CSPS%20COLMA1.MP3time=1418600)

[00:23:38-6](http://localhost:2300/file=C:/Users/USER/Desktop/ENTRETIENS%20PREMATURITE/CSPS-COLMA1/MAJOR%20CSPS%20COLMA1.MP3time=1418600) Interviewer: je vous remercie beaucoup pour votre disponibilité, merci. [00:23:42-2](http://localhost:2300/file=C:/Users/USER/Desktop/ENTRETIENS%20PREMATURITE/CSPS-COLMA1/MAJOR%20CSPS%20COLMA1.MP3time=1422200)

[00:23:42-2](http://localhost:2300/file=C:/Users/USER/Desktop/ENTRETIENS%20PREMATURITE/CSPS-COLMA1/MAJOR%20CSPS%20COLMA1.MP3time=1422200) Person 1: Merci, merci.

00:23:15-9 Interviewer: Je pense que nous avons un peu parcouru toutes les questions maintenant, au-delà des questions que nous avons posées, est-ce qu'il y a un point sur lequel vous aimeriez échanger que nous n'avons pas abordé par rapport à la question sur la naissance prématurée ?

00:23:34-9 Person 1: Par rapport à la naissance prématurée, non, bien non, je ne vois pas, je ne vois pas.

00:23:38-6 Interviewer: Je vous remercie beaucoup pour votre disponibilité, merci.

00:23:42-2 Person 1: Merci, merci.

**Interviewer (00:23:15-9):** "I think we've covered all the questions now. Is there anything else you'd like to discuss related to premature birth that we haven't addressed?"

**Person 1 (00:23:34-9):** "Regarding premature birth, no, there's nothing else I can think of right now."

**Interviewer (00:23:38-6):** "Thank you very much for your time and input."

**Person 1 (00:23:42-2):** "Thank you, thank you.

### Maternity manager of Colma1 health and social promotion center

[00:39:52-0](http://localhost:2300/file=C:/Users/USER/Desktop/ENTRETIENS%20PREMATURITE/CSPS-COLMA1/RESPONSABLE%20MATERNITE-COLMA1.mp3time=2392000) Interviewer: Je vous remercie beaucoup. [00:39:54-4](http://localhost:2300/file=C:/Users/USER/Desktop/ENTRETIENS%20PREMATURITE/CSPS-COLMA1/RESPONSABLE%20MATERNITE-COLMA1.mp3time=2394400)

[00:39:54-4](http://localhost:2300/file=C:/Users/USER/Desktop/ENTRETIENS%20PREMATURITE/CSPS-COLMA1/RESPONSABLE%20MATERNITE-COLMA1.mp3time=2394400) Person 1: Merci à vous également. [00:39:57-6](http://localhost:2300/file=C:/Users/USER/Desktop/ENTRETIENS%20PREMATURITE/CSPS-COLMA1/RESPONSABLE%20MATERNITE-COLMA1.mp3time=2397600)

[00:39:57-6](http://localhost:2300/file=C:/Users/USER/Desktop/ENTRETIENS%20PREMATURITE/CSPS-COLMA1/RESPONSABLE%20MATERNITE-COLMA1.mp3time=2397600) Interviewer: Si vous n''avez pas d'autres questions, je pense que l'entretien est arrivé à terme. [00:40:02-1](http://localhost:2300/file=C:/Users/USER/Desktop/ENTRETIENS%20PREMATURITE/CSPS-COLMA1/RESPONSABLE%20MATERNITE-COLMA1.mp3time=2402100)

[00:40:02-1](http://localhost:2300/file=C:/Users/USER/Desktop/ENTRETIENS%20PREMATURITE/CSPS-COLMA1/RESPONSABLE%20MATERNITE-COLMA1.mp3time=2402100) Person 1: D'accord [00:40:02-6](http://localhost:2300/file=C:/Users/USER/Desktop/ENTRETIENS%20PREMATURITE/CSPS-COLMA1/RESPONSABLE%20MATERNITE-COLMA1.mp3time=2402600)

[00:40:02-6](http://localhost:2300/file=C:/Users/USER/Desktop/ENTRETIENS%20PREMATURITE/CSPS-COLMA1/RESPONSABLE%20MATERNITE-COLMA1.mp3time=2402600) Interviewer: Merci pour votre disponibilité. [00:40:04-3](http://localhost:2300/file=C:/Users/USER/Desktop/ENTRETIENS%20PREMATURITE/CSPS-COLMA1/RESPONSABLE%20MATERNITE-COLMA1.mp3time=2404300)

[00:40:04-3](http://localhost:2300/file=C:/Users/USER/Desktop/ENTRETIENS%20PREMATURITE/CSPS-COLMA1/RESPONSABLE%20MATERNITE-COLMA1.mp3time=2404300) Person 1: C'est normal.

00:39:52-0 Interviewer: Je vous remercie beaucoup.

00:39:54-4 Person 1: Merci à vous également.

00:39:57-6 Interviewer: Si vous n'avez pas d'autres questions, je pense que l'entretien est arrivé à terme.

00:40:02-1 Person 1: D'accord.

00:40:02-6 Interviewer: Merci pour votre disponibilité.

00:40:04-3 Person 1: C'est normal.

**00:39:54-4 Interviewer:** Thank you very much for your insights.

**00:39:57-6 Person 1:** Thank you too.

**00:40:02-1 Interviewer:** If there are no further questions, I believe we can conclude the interview.

**00:40:02-6 Person 1:** Okay.

**00:40:04-3 Interviewer:** Thank you for your time and availability.

**00:40:04-3 Person 1:** It's normal."

### Focus group of Farakan health and social promotion center

[00:41:35-4](http://localhost:2300/file=C:/Users/USER/Desktop/ENTRETIENS%20PREMATURITE/CSPS_FARAKAN/FOCUS_GROUPE-CSPS_FARAKAN.MP3time=2495400) Interviewer: Je pense que nous sommes au terme donc de l'entretien. Je vous remercie beaucoup pour votre patience et la qualité des réponses que vous avez apportées. S'il y a quelque chose d'autre à ajouter avant de conclure, je pense que nous sommes toutes oreilles. Nous sommes à votre disposition. [00:41:52-7](http://localhost:2300/file=C:/Users/USER/Desktop/ENTRETIENS%20PREMATURITE/CSPS_FARAKAN/FOCUS_GROUPE-CSPS_FARAKAN.MP3time=2512700)

[00:41:52-7](http://localhost:2300/file=C:/Users/USER/Desktop/ENTRETIENS%20PREMATURITE/CSPS_FARAKAN/FOCUS_GROUPE-CSPS_FARAKAN.MP3time=2512700) Person 3: Hors antenne. [00:41:52-7](http://localhost:2300/file=C:/Users/USER/Desktop/ENTRETIENS%20PREMATURITE/CSPS_FARAKAN/FOCUS_GROUPE-CSPS_FARAKAN.MP3time=2512700)

[00:44:13-2](http://localhost:2300/file=C:/Users/USER/Desktop/ENTRETIENS%20PREMATURITE/CSPS_FARAKAN/FOCUS_GROUPE-CSPS_FARAKAN.MP3time=2653200) Interviewer: Merci beaucoup je pense que nous sommes au terme de l'entretien. Merci à vous pour votre participation. [00:44:22-6](http://localhost:2300/file=C:/Users/USER/Desktop/ENTRETIENS%20PREMATURITE/CSPS_FARAKAN/FOCUS_GROUPE-CSPS_FARAKAN.MP3time=2662600)

00:41:35-4 Interviewer: Je pense que nous sommes au terme de l'entretien. Je vous remercie beaucoup pour votre patience et la qualité des réponses que vous avez apportées. S'il y a quelque chose d'autre à ajouter avant de conclure, je pense que nous sommes toutes oreilles. Nous sommes à votre disposition. 00:41:52-7

00:41:52-7 Personne 3: Hors antenne. 00:41:52-7

00:44:13-2 Interviewer: Merci beaucoup, je pense que nous sommes au terme de l'entretien. Merci à vous pour votre participation. 00:44:22-6

**Interviewer (00:41:35-4):** "I believe we've come to the end of our interview. Thank you very much for your patience and the insightful answers you've provided. If there's anything else you'd like to add before we conclude, we're ready to listen. We are at your service."

**Person 3 (00:41:52-7):** "Off the air."

**Interviewer (00:44:13-2):** "Thank you very much for your participation in this interview. I think that concludes our session."

### Major of Farakan health and social promotion center

[00:16:18-2](http://localhost:2300/file=C:/Users/USER/Desktop/ENTRETIENS%20PREMATURITE/CSPS_FARAKAN/MAJOR%20FARAKAN.MP3time=978200) Interviewer: Ok.Je pense que nous avons abordé toutes les questions, mais je ne sais pas si vous avez d'autres. [00:16:22-5](http://localhost:2300/file=C:/Users/USER/Desktop/ENTRETIENS%20PREMATURITE/CSPS_FARAKAN/MAJOR%20FARAKAN.MP3time=982500)

[00:16:22-5](http://localhost:2300/file=C:/Users/USER/Desktop/ENTRETIENS%20PREMATURITE/CSPS_FARAKAN/MAJOR%20FARAKAN.MP3time=982500) Person 1: Non, c'est bon (rire), merci beaucoup. [00:16:26-4](http://localhost:2300/file=C:/Users/USER/Desktop/ENTRETIENS%20PREMATURITE/CSPS_FARAKAN/MAJOR%20FARAKAN.MP3time=986400)

[00:16:26-4](http://localhost:2300/file=C:/Users/USER/Desktop/ENTRETIENS%20PREMATURITE/CSPS_FARAKAN/MAJOR%20FARAKAN.MP3time=986400) Interviewer: Merci infiniment pour les éléments de réponse que vous nous avez apportés. Je pense que cela va contribuer énormément à améliorer la différents services pour la prise en charge de la prématurité. [00:16:38-4](http://localhost:2300/file=C:/Users/USER/Desktop/ENTRETIENS%20PREMATURITE/CSPS_FARAKAN/MAJOR%20FARAKAN.MP3time=998400)

[00:16:38-4](http://localhost:2300/file=C:/Users/USER/Desktop/ENTRETIENS%20PREMATURITE/CSPS_FARAKAN/MAJOR%20FARAKAN.MP3time=998400) Person 1: C'est à vous les remerciements aussi.

00:16:18-2 Interviewer: Ok. Je pense que nous avons abordé toutes les questions, mais je ne sais pas si vous avez d'autres. 00:16:22-5

00:16:22-5 Personne 1: Non, c'est bon (rire), merci beaucoup. 00:16:26-4

00:16:26-4 Interviewer: Merci infiniment pour les éléments de réponse que vous nous avez apportés. Je pense que cela va contribuer énormément à améliorer les différents services pour la prise en charge de la prématurité. 00:16:38-4

00:16:38-4 Personne 1: C'est à vous les remerciements aussi.

**Interviewer (00:16:18-2):** "Okay. We've gone through all the questions, but I want to check if there's anything else you'd like to add or discuss."

**Person 1 (00:16:22-5):** "No, everything's fine [laughs], thank you very much."

**Interviewer (00:16:26-4):** "Thank you very much for your responses. I believe your insights will greatly contribute to improving care services for premature infants."

**Person 1 (00:16:38-4):** "Thank you as well."

### Maternity manager of Farakan health and social promotion center

[00:30:05-6](http://localhost:2300/file=C:/Users/USER/Desktop/ENTRETIENS%20PREMATURITE/CSPS_FARAKAN/RESPONSABLE%20MATERNITE%20FARAKAN.MP3time=1805600) Interviewer: Merci beaucoup madame pour votre disponibilité.je pense que nous sommes au terme de l'entretien [00:30:12-3](http://localhost:2300/file=C:/Users/USER/Desktop/ENTRETIENS%20PREMATURITE/CSPS_FARAKAN/RESPONSABLE%20MATERNITE%20FARAKAN.MP3time=1812300)

[00:30:12-3](http://localhost:2300/file=C:/Users/USER/Desktop/ENTRETIENS%20PREMATURITE/CSPS_FARAKAN/RESPONSABLE%20MATERNITE%20FARAKAN.MP3time=1812300) Person 1: Ok. Merci à vous aussi (rire) [00:30:18-7](http://localhost:2300/file=C:/Users/USER/Desktop/ENTRETIENS%20PREMATURITE/CSPS_FARAKAN/RESPONSABLE%20MATERNITE%20FARAKAN.MP3time=1818700)

[00:30:18-7](http://localhost:2300/file=C:/Users/USER/Desktop/ENTRETIENS%20PREMATURITE/CSPS_FARAKAN/RESPONSABLE%20MATERNITE%20FARAKAN.MP3time=1818700) Interviewer: Je repasserai pour les informations sur les naissances prematurées des sept derniers mois. [00:30:32-6](http://localhost:2300/file=C:/Users/USER/Desktop/ENTRETIENS%20PREMATURITE/CSPS_FARAKAN/RESPONSABLE%20MATERNITE%20FARAKAN.MP3time=1832600)

[00:30:32-6](http://localhost:2300/file=C:/Users/USER/Desktop/ENTRETIENS%20PREMATURITE/CSPS_FARAKAN/RESPONSABLE%20MATERNITE%20FARAKAN.MP3time=1832600) Person 1: D'accord nous avons les rapports c'est plus facile à les trouver.il ya aussi les registres d'accouchement par rapport au poids [00:31:05-2](http://localhost:2300/file=C:/Users/USER/Desktop/ENTRETIENS%20PREMATURITE/CSPS_FARAKAN/RESPONSABLE%20MATERNITE%20FARAKAN.MP3time=1865200)

[00:31:11-5](http://localhost:2300/file=C:/Users/USER/Desktop/ENTRETIENS%20PREMATURITE/CSPS_FARAKAN/RESPONSABLE%20MATERNITE%20FARAKAN.MP3time=1871500) Interviewer: Merci.

00:30:05-6 Interviewer: Merci beaucoup madame pour votre disponibilité. Je pense que nous sommes au terme de l'entretien.

00:30:12-3 Person 1: Ok. Merci à vous aussi (rire).

00:30:18-7 Interviewer: Je repasserai pour les informations sur les naissances prématurées des sept derniers mois.

00:30:32-6 Person 1: D'accord, nous avons les rapports c'est plus facile à les trouver. Il y a aussi les registres d'accouchement par rapport au poids. 00:31:05-2

00:31:05-2 Interviewer: Merci

**00:30:12-3 Interviewer:** Thank you for your insights. I think we're at the end of the interview.

**00:30:18-7 Interviewer:** I'll return for information on premature births in the last seven months.

**00:30:32-6 Person 1:** Okay, we have the reports. It's easy to find them. We also have birth records related to weight.

**00:31:11-5 Interviewer:** Thank you.
