# Supplementary material for: Perceptions and practices in urban Burkina Faso: a qualitative study on gestational age estimation among health workers
Source: Int J Qual Stud Health Well-being. 2025 Jul 4;20(1):2508421. doi: 10.1080/17482631.2025.2508421 (PMC12231294; doi:10.1080/17482631.2025.2508421)
Supplement: Supplementary_file_4_interview_guide.docx [file ZQHW_A_2508421_SM4866.docx]

**GROUP INTERVIEW AND DISCUSSION GUIDE**

***Interviews:*** *MCD, Gynecologists, Head Doctors/Nurses, Maternity Ward Managers*

***Focus group:*** bring together at least 7 to 8 midwives/midwives from the 4 maternity hospitals

## **INTRODUCTIONS**

- Introduce yourself and let the health care staff in front know that you are here to better understand some aspects related to preterm birth. All answers are welcome: there are no right or wrong answers.
- The interview will be recorded if it is convenient for her. Only the research team will listen to the recording. We will not associate her name with her opinions and experiences about prematurity.
  - 1. The brief consent form should also be recorded and their agreement to participate should also be recorded.

***Start your voice recorder and say out loud:***

- The name of the CSPS
- Today's date and time (interview date)
  - 1. The function, title and role of your contact: ICP of (NAME of CSPS), Maternity manager of (name of CSPS) ***(see question 1)***
- Do not say the name of the person you are talking to.

**INFORMED CONSENT:** Hello, thank you very much in advance for your availability and the time you give us. We are a research team from Ghent University, IRSS and AFRICSanté. We are conducting qualitative research on preterm birth in urban areas of Burkina Faso. Our goal is to better understand this phenomenon in order to improve services and health care for mothers and preterm infants. Your participation is essential to help us understand the specific challenges you face in your health center and to identify opportunities for improvement.

Today’s interview will be confidential and the information collected will be used for research purposes only. You are free not to answer any questions that make you uncomfortable and you can stop the interview at any time. Do you have any questions before we begin? With your permission, may we begin the interview?

1. Could you start by introducing yourself (no name), your title, your position and your role, please? How long have you been in this position?
2. How do you define premature birth in your health center?
3. Now that we have defined what premature birth is, how is it measured in your health center?
   1. Out of 10 women who come to their first CPN, how many know the date of their last period? In which trimester of pregnancy or what gestational age do women most often come to their first CPN? Do you think that women know precisely (plus or minus a week, plus or minus a month) the date of their last period during their first CPN?
   2. Do pregnant women who present for their first ANC later (after the 3rd month of pregnancy) in pregnancy always remember the date of their last period?
      1. If the pregnant woman does not remember at all, how does the midwife determine the gestational age (fundal height)?
      2. How do you calculate gestational age from fundal height? By gestogram or by table)?
   3. Do you have the equipment to perform the gestational age calculation? If not, what approach do you use in this case?
   4. When calculating gestational age, which estimate takes priority: that of the uterine height or that of the date of the last menstrual period?
   5. Do women sometimes come with an ultrasound estimate? In that case, which estimate takes priority: the ultrasound estimate, the uterine height estimate, or the date of the last period?
   6. In case the gestational age estimates by last menstrual period, fundal height and ultrasound are different, which estimate do you decide to use and why?
4. Is information on premature births recorded in the delivery register? Is this reflected in your monthly activity report?
5. How many premature babies are born each month in your health center?
   1. To get a rough idea, he can possibly use his birth register.
   2. If he has no idea and wants to consult his register, continue the interview and offer to come back to get the information on prematurity for the last 7 months (January to July).
6. Is preterm birth a major public health challenge you face in your health center? What types of challenges do you face in your department regarding pregnancy, childbirth and newborn care?
7. Do you have the necessary technical platform, that is to say the trained personnel, the medications and the equipment to manage cases of premature birth?
   1. Probe for very premature babies?
   2. What do you do when you find that you cannot handle a case of premature birth in your department?
8. What types of services are provided to premature infants and their mothers, if any, in your department?
9. What are the specific health risks that premature infants face in urban Burkina Faso?
   1. Mortality survey?
   2. Also probe morbidities - diseases?
10. Have you noticed any specific characteristics regarding mothers of premature babies?
    1. Do they tend to be younger or older?
    2. Do they come from a specific socio-economic background?
    3. Do they have any special health conditions?
11. How can we improve the management of premature births in your health center?
    1. Probe about skill building?
    2. Probe on human and material resources?
