# Supplementary material for: Perceptions and practices in urban Burkina Faso: a qualitative study on gestational age estimation among health workers
Source: Int J Qual Stud Health Well-being. 2025 Jul 4;20(1):2508421. doi: 10.1080/17482631.2025.2508421 (PMC12231294; doi:10.1080/17482631.2025.2508421)
Supplement: supplementary_file_3_codebook.docx [file ZQHW_A_2508421_SM4864.docx]

| Interview | Topic | Subtopic | Interviewee | Answers | Interviewee | Number and Responses |
| --- | --- | --- | --- | --- | --- | --- |
| Accart-ville focus group | Informed consent and presentation of participants | n/a | person 1 | No, we don't have any questions. | person 1 | 1 No, we don't have any questions. |
| Accart-ville focus group | Informed consent and presentation of participants | n/a | person 1 | Yes. | person 1 | 2 Yes. |
| Accart-ville focus group | Informed consent and presentation of participants | n/a | person 5 | Yes, we agree. | person 5 | 3 Yes, we agree. |
| Accart-ville focus group | Informed consent and presentation of participants | n/a | person 1 | I'm a state midwife and have been assigned to Accart-Ville for a year, newly appointed. | person 1 | 4 I'm a state midwife and have been assigned to Accart-Ville for a year, newly appointed. |
| Accart-ville CMU manager | Informed consent and presentation of participants | n/a | person 1 | No, I’m fine with proceeding. | person 1 | 5 No, I’m fine with proceeding. |
| Accart-ville CMU manager | Informed consent and presentation of participants | n/a | person 1 | I am a general practitioner and the manager of the Accart-Ville urban medical center. I've been with the center since December 2021, making this my second year. My responsibilities include administrative duties and providing medical consultations. | person 1 | 6 I am a general practitioner and the manager of the Accart-Ville urban medical center. I've been with the center since December 2021, making this my second year. My responsibilities include administrative duties and providing medical consultations. |
| Accart-ville maternity manager | Informed consent and presentation of participants | n/a | person 1 | No. | person 1 | 7 No. |
| Accart-ville maternity manager | Informed consent and presentation of participants | n/a | person 1 | Yes, we can begin. | person 1 | 8 Yes, we can begin. |
| Accart-ville maternity manager | Informed consent and presentation of participants | n/a | person 1 | I am a health officer specializing in obstetric and gynecological care. I've been at CMU Accart-Ville for one year, serving as the head of maternity. | person 1 | 9 I am a health officer specializing in obstetric and gynecological care. I've been at CMU Accart-Ville for one year, serving as the head of maternity. |
| CMA Do focus group | Informed consent and presentation of participants | n/a | person 3 | How exactly should we introduce ourselves? | person 3 | 10 How exactly should we introduce ourselves? |
| CMA Do focus group | Informed consent and presentation of participants | n/a | person 1 | I am a midwife at the CMA de Dô, with six years of service here. | person 1 | 11 I am a midwife at the CMA de Dô, with six years of service here. |
| CMA Do focus group | Informed consent and presentation of participants | n/a | person 2 | I've been a midwife at CMA in Dô for twelve years. | person 2 | 12 I've been a midwife at CMA in Dô for twelve years. |
| CMA Do focus group | Informed consent and presentation of participants | n/a | person 3 | As a midwife at CMA de Dô, I've been in charge of the delivery room for four years. | person 3 | 13 As a midwife at CMA de Dô, I've been in charge of the delivery room for four years. |
| CMA Do focus group | Informed consent and presentation of participants | n/a | person 4 | I am a state midwife at CMA in Dô, working in the delivery room. | person 4 | 14 I am a state midwife at CMA in Dô, working in the delivery room. |
| CMA Do focus group | Informed consent and presentation of participants | n/a | person 5 | State midwife with six years of tenure, also in the delivery room. | person 5 | 15 State midwife with six years of tenure, also in the delivery room. |
| CMA Do focus group | Informed consent and presentation of participants | n/a | person 6 | State midwife for 13 years, currently responsible for the maternity ward. | person 6 | 16 State midwife for 13 years, currently responsible for the maternity ward. |
| CMA Do focus group | Informed consent and presentation of participants | n/a | person 7 | Midwife for five years at CMA de Dô, working in post-natal care. | person 7 | 17 Midwife for five years at CMA de Dô, working in post-natal care. |
| CMA Do focus group | Informed consent and presentation of participants | n/a | person 8 | I've been a midwife for ten years at CMA de Dô. | person 8 | 18 I've been a midwife for ten years at CMA de Dô. |
| CMA Do focus group | Informed consent and presentation of participants | n/a | person 9 | I am a midwife with eleven years of service at CMA de Dô. | person 9 | 19 I am a midwife with eleven years of service at CMA de Dô. |
| Gynecologist 1 | Informed consent and presentation of participants | n/a | person 1 | Okay, I agree to participate in this research. There's no problem. I am the gynecologist in charge of the maternity department at the CMA de Dô. | person 1 | 20 Okay, I agree to participate in this research. There's no problem. I am the gynecologist in charge of the maternity department at the CMA de Dô. |
| Gynecologist 1 | Informed consent and presentation of participants | n/a | person 1 | I've been in this position for 13 years. | person 1 | 21 I've been in this position for 13 years. |
| Gynecologist 2 | Informed consent and presentation of participants | n/a | person 1 | I consent to participate in this study. I am a gynecologist and have been working here for 4 years. | person 1 | 22 I consent to participate in this study. I am a gynecologist and have been working here for 4 years. |
| Gynecologist 2 | Informed consent and presentation of participants | n/a | person 1 | My role isn't strictly defined as there's a department manager and care unit supervision already. As the second gynecologist here, my duties involve participating in patient care, consultations, and managing emergencies. | person 1 | 23 My role isn't strictly defined as there's a department manager and care unit supervision already. As the second gynecologist here, my duties involve participating in patient care, consultations, and managing emergencies. |
| MCD | Informed consent and presentation of participants | n/a | person 1 | Let's start, as I need to leave by 10:30 AM. | person 1 | 24 Let's start, as I need to leave by 10:30 AM. |
| MCD | Informed consent and presentation of participants | n/a | person 1 | Without my first and last name, right? | person 1 | 25 Without my first and last name, right? |
| MCD | Informed consent and presentation of participants | n/a | person 1 | I am the chief doctor of the Dô Health District. I started my service here on May 29, 2021, so it's been 2 years and 2 months. | person 1 | 26 I am the chief doctor of the Dô Health District. I started my service here on May 29, 2021, so it's been 2 years and 2 months. |
| MCD | Informed consent and presentation of participants | n/a | person 1 | As the head doctor, my primary responsibility is to lead the district management team, which governs the health district in Burkina Faso. I ensure that all planned activities at the district level are executed effectively, and I work to guarantee that our responsible population has access to healthcare to improve their overall health status. | person 1 | 27 As the head doctor, my primary responsibility is to lead the district management team, which governs the health district in Burkina Faso. I ensure that all planned activities at the district level are executed effectively, and I work to guarantee that our responsible population has access to healthcare to improve their overall health status. |
| CMA Do maternity care unit manager | Informed consent and presentation of participants | n/a | person 1 | Yes. | person 1 | 28 Yes. |
| CMA Do maternity care unit manager | Informed consent and presentation of participants | n/a | person 1 | Yes, no problem. | person 1 | 29 Yes, no problem. |
| CMA Do maternity care unit manager | Informed consent and presentation of participants | n/a | person 1 | Okay. I am the health officer in obstetric and gynecological care, and I also oversee the maternity care unit of the CMA de Dô. | person 1 | 30 Okay. I am the health officer in obstetric and gynecological care, and I also oversee the maternity care unit of the CMA de Dô. |
| CMA Do maternity care unit manager | Informed consent and presentation of participants | n/a | person 1 | Eh... since 2019. So, how many years is that? | person 1 | 31 Eh... since 2019. So, how many years is that? |
| CMA Do maternity care unit manager | Informed consent and presentation of participants | n/a | person 1 | No, I've been here since 2015. But I've been in the position of care unit supervisor since 2019. Ah, yes, since 2019. | person 1 | 32 No, I've been here since 2015. But I've been in the position of care unit supervisor since 2019. Ah, yes, since 2019. |
| Colma1 focus group | Informed consent and presentation of participants | n/a | person 1 | Yes, yes. | person 1 | 33 Yes, yes. |
| Colma1 major | Informed consent and presentation of participants | n/a | person 1 | No, I don't have any questions. | person 1 | 34 No, I don't have any questions. |
| Colma1 major | Informed consent and presentation of participants | n/a | person 1 | Yes, please go ahead. | person 1 | 35 Yes, please go ahead. |
| Colma1 major | Informed consent and presentation of participants | n/a | person 1 | I am the head nurse, a state-certified nurse. I've been working in Colma1 for 3 years, fulfilling responsibilities as a nurse manager. | person 1 | 36 I am the head nurse, a state-certified nurse. I've been working in Colma1 for 3 years, fulfilling responsibilities as a nurse manager. |
| Colma1 major | Informed consent and presentation of participants | n/a | person 1 | As I mentioned earlier, my primary role as a nurse manager involves coordinating activities, supervising, and planning. I integrate various activities within the CSPS to ensure that everyone works towards our common goal, which is to provide services to the population. | person 1 | 37 As I mentioned earlier, my primary role as a nurse manager involves coordinating activities, supervising, and planning. I integrate various activities within the CSPS to ensure that everyone works towards our common goal, which is to provide services to the population. |
| Colma1 maternity manager | Informed consent and presentation of participants | n/a | person 1 | Regarding our CSPS, Madam Sawadogo previously informed us about this interview, and you were here last time for the same reason. I believe the appointment was set for today at 11:30. We've made an effort to meet the proposed schedule. I see no issues with proceeding with the interview. If questions are asked, they will aid in refreshing our memories and benefit us professionally, as well as the women we serve. Successful outcomes in our work not only benefit the families but also enhance our reputation as practitioners. So, there's no problem; we are always eager to learn | person 1 | 38 Regarding our CSPS, Madam Sawadogo previously informed us about this interview, and you were here last time for the same reason. I believe the appointment was set for today at 11:30. We've made an effort to meet the proposed schedule. I see no issues with proceeding with the interview. If questions are asked, they will aid in refreshing our memories and benefit us professionally, as well as the women we serve. Successful outcomes in our work not only benefit the families but also enhance our reputation as practitioners. So, there's no problem; we are always eager to learn |
| Colma1 maternity manager | Informed consent and presentation of participants | n/a | person 1 | No, not at all. | person 1 | 39 No, not at all. |
| Colma1 maternity manager | Informed consent and presentation of participants | n/a | person 1 | Okay. | person 1 | 40 Okay. |
| Colma1 maternity manager | Informed consent and presentation of participants | n/a | person 1 | I'm a midwife at the CSPS in Colma 1. I've been the maternity manager for about two years now. Overall, I've been in public service for 16 years, but specifically as a midwife, it's been five years. | person 1 | 41 I'm a midwife at the CSPS in Colma 1. I've been the maternity manager for about two years now. Overall, I've been in public service for 16 years, but specifically as a midwife, it's been five years. |
| Farakan focus group | Informed consent and presentation of participants | n/a | person 1 | No. | person 1 | 42 No. |
| Farakan focus group | Informed consent and presentation of participants | n/a | person 2 | Yes, I agree. | person 2 | 43 Yes, I agree. |
| Farakan focus group | Informed consent and presentation of participants | n/a | person 3 | Yes, I am willing. | person 3 | 44 Yes, I am willing. |
| Farakan focus group | Informed consent and presentation of participants | n/a | person 4 | Okay, yes, I agree. | person 4 | 45 Okay, yes, I agree. |
| Farakan focus group | Informed consent and presentation of participants | n/a | person 6 | Yes. | person 6 | 46 Yes. |
| Farakan focus group | Informed consent and presentation of participants | n/a | person 7 | Yes. | person 7 | 47 Yes. |
| Farakan focus group | Informed consent and presentation of participants | n/a | person 1 | To introduce myself? I am an auxiliary midwife, and I have been at the Farakan CSPS for 3 years. | person 1 | 48 To introduce myself? I am an auxiliary midwife, and I have been at the Farakan CSPS for 3 years. |
| Farakan focus group | Informed consent and presentation of participants | n/a | person 2 | I am an auxiliary birth attendant and have been here for 3 years. | person 2 | 49 I am an auxiliary birth attendant and have been here for 3 years. |
| Farakan focus group | Informed consent and presentation of participants | n/a | person 3 | I'm also an auxiliary midwife, with 3 years at the CSPS. | person 3 | 50 I'm also an auxiliary midwife, with 3 years at the CSPS. |
| Farakan focus group | Informed consent and presentation of participants | n/a | person 4 | I am a midwife and have been working at the Farakan CSPS for 8 years. | person 4 | 51 I am a midwife and have been working at the Farakan CSPS for 8 years. |
| Farakan focus group | Informed consent and presentation of participants | n/a | person 5 | I am an auxiliary midwife, at Farakan CSPS for 7 years. | person 5 | 52 I am an auxiliary midwife, at Farakan CSPS for 7 years. |
| Farakan focus group | Informed consent and presentation of participants | n/a | person 6 | I am an auxiliary midwife at Farakan CSPS, with a tenure of 4 years. | person 6 | 53 I am an auxiliary midwife at Farakan CSPS, with a tenure of 4 years. |
| Farakan focus group | Informed consent and presentation of participants | n/a | person 7 | I've been an auxiliary midwife for 10 years at the CSPS. | person 7 | 54 I've been an auxiliary midwife for 10 years at the CSPS. |
| Farakan focus group | Informed consent and presentation of participants | n/a | person 8 | Similarly, I have been an auxiliary midwife for 10 years at the CSPS. | person 8 | 55 Similarly, I have been an auxiliary midwife for 10 years at the CSPS. |
| Farakan major | Informed consent and presentation of participants | n/a | person 1 | Okay. | person 1 | 56 Okay. |
| Farakan major | Informed consent and presentation of participants | n/a | person 1 | It's okay. | person 1 | 57 It's okay. |
| Farakan major | Informed consent and presentation of participants | n/a | person 1 | Without the name? (laughter) I am the outgoing coordinator, as I have been reassigned. I'm an IDE (Infirmier Diplômé d'État, or State Registered Nurse). | person 1 | 58 Without the name? (laughter) I am the outgoing coordinator, as I have been reassigned. I'm an IDE (Infirmier Diplômé d'État, or State Registered Nurse). |
| Farakan maternity manager | Informed consent and presentation of participants | n/a | person 1 | Actually, it's a CMU, an urban medical center. | person 1 | 59 Actually, it's a CMU, an urban medical center. |
| Farakan maternity manager | Informed consent and presentation of participants | n/a | person 1 | Regarding prematurity, no, I don't have any questions. | person 1 | 60 Regarding prematurity, no, I don't have any questions. |
| Farakan maternity manager | Informed consent and presentation of participants | n/a | person 1 | Yes, you can proceed. | person 1 | 61 Yes, you can proceed. |
| Farakan maternity manager | Informed consent and presentation of participants | n/a | person 1 | I am responsible for the maternity ward. I'm a midwife and have been working at the Farakan CSPS since 2012 | person 1 | 62 I am responsible for the maternity ward. I'm a midwife and have been working at the Farakan CSPS since 2012 |
| Accart-ville focus group | Definition of preterm birth | n/a | person 1 | Premature birth is defined as any birth that occurs before the thirty-seventh week, or before 37 weeks of amenorrhea. | person 1 | 63 Premature birth is defined as any birth that occurs before the thirty-seventh week, or before 37 weeks of amenorrhea. |
| Accart-ville CMU manager | Definition of preterm birth | n/a | person 1 | Premature birth in our health center is defined as any preterm delivery, specifically occurring before 36 weeks of amenorrhea | person 1 | 64 Premature birth in our health center is defined as any preterm delivery, specifically occurring before 36 weeks of amenorrhea |
| Accart-ville maternity manager | Definition of preterm birth | n/a | person 1 | Premature birth is defined as any birth that occurs between the twenty-eighth week and thirty-six weeks and six days of pregn/acy. | person 1 | 65 Premature birth is defined as any birth that occurs between the twenty-eighth week and thirty-six weeks and six days of pregn/acy. |
| CMA Do focus group | Definition of preterm birth | n/a | person 1 | It's any birth that occurs before 37 weeks. | person 1 | 66 It's any birth that occurs before 37 weeks. |
| CMA Do focus group | Definition of preterm birth | n/a | person 3 | No. | person 3 | 67 No. |
| Gynecologist 1 | Definition of preterm birth | n/a | person 1 | In our health center, we define premature birth in two ways. Firstly, in terms of gestational age: if the age of the pregn/acy is less than 36 weeks, we classify it as premature. Secondly, we consider fetal weight; often, if it is less than 2 kilograms, we tend to classify the birth as premature. Of course, there are other morphological criteria, but the primary criterion is a gestational age of less than 36 weeks. | person 1 | 68 In our health center, we define premature birth in two ways. Firstly, in terms of gestational age: if the age of the pregn/acy is less than 36 weeks, we classify it as premature. Secondly, we consider fetal weight; often, if it is less than 2 kilograms, we tend to classify the birth as premature. Of course, there are other morphological criteria, but the primary criterion is a gestational age of less than 36 weeks. |
| Gynecologist 2 | Definition of preterm birth | n/a | person 1 | Yes, in our setting, premature birth is classically defined as births that occur between 28 weeks and 37 weeks of amenorrhea. Therefore, a child born within this range, from 28 to 37 weeks, is considered premature. | person 1 | 69 Yes, in our setting, premature birth is classically defined as births that occur between 28 weeks and 37 weeks of amenorrhea. Therefore, a child born within this range, from 28 to 37 weeks, is considered premature. |
| MCD | Definition of preterm birth | n/a | person 1 | Within CMA-Dô? I don't think there's a specific definition for our center. The definition of premature birth is somewhat universal in health practice. Generally, it's any birth that occurs before 9 months, or more specifically, before 37 weeks of amenorrhea is considered premature. However, we also need to consider the aspect of abortion. When the products of conception are expelled before 22 weeks of amenorrhea, it's classified as an abortion. So, there's this distinction: before 22 weeks, it's an abortion, but any birth from 22 weeks up until before 37 weeks is considered premature. | person 1 | 70 Within CMA-Dô? I don't think there's a specific definition for our center. The definition of premature birth is somewhat universal in health practice. Generally, it's any birth that occurs before 9 months, or more specifically, before 37 weeks of amenorrhea is considered premature. However, we also need to consider the aspect of abortion. When the products of conception are expelled before 22 weeks of amenorrhea, it's classified as an abortion. So, there's this distinction: before 22 weeks, it's an abortion, but any birth from 22 weeks up until before 37 weeks is considered premature. |
| CMA Do maternity care unit manager | Definition of preterm birth | n/a | person 1 | Premature birth is a birth that occurs before 36 weeks of pregn/acy. So, any birth below 36 weeks is considered premature. | person 1 | 71 Premature birth is a birth that occurs before 36 weeks of pregn/acy. So, any birth below 36 weeks is considered premature. |
| Colma1 focus group | Definition of preterm birth | n/a | person 1 | Premature birth is when a child has not reached its full term of 9 months. | person 1 | 72 Premature birth is when a child has not reached its full term of 9 months. |
| Colma1 focus group | Definition of preterm birth | n/a | person 2 | It's a child who is born before completing the full term of 9 months. | person 2 | 73 It's a child who is born before completing the full term of 9 months. |
| Colma1 focus group | Definition of preterm birth | n/a | person 3 | Generally, it refers to a child who hasn't reached the end of 9 months. However, in our region, it starts from the 28th week, but in developed countries, it can be considered from as early as the 22nd week, according to what we learned in our courses. | person 3 | 74 Generally, it refers to a child who hasn't reached the end of 9 months. However, in our region, it starts from the 28th week, but in developed countries, it can be considered from as early as the 22nd week, according to what we learned in our courses. |
| Colma1 major | Definition of preterm birth | n/a | person 1 | In our center, and I believe this applies globally within the health system, premature birth is defined in a standard way. A child is considered premature when the birth occurs from 7 months of pregn/acy, so that's generally around 7 months. We also consider the weight of the child; typically, a premature baby will weigh less than 2.5 kilograms. | person 1 | 75 In our center, and I believe this applies globally within the health system, premature birth is defined in a standard way. A child is considered premature when the birth occurs from 7 months of pregn/acy, so that's generally around 7 months. We also consider the weight of the child; typically, a premature baby will weigh less than 2.5 kilograms. |
| Colma1 maternity manager | Definition of preterm birth | n/a | person 1 | Yes, in our health center, premature birth is defined as any birth that occurs before the 37th week of amenorrhea. Specifically, this includes children born between the 28th week and the 36th week, plus six days. | person 1 | 76 Yes, in our health center, premature birth is defined as any birth that occurs before the 37th week of amenorrhea. Specifically, this includes children born between the 28th week and the 36th week, plus six days. |
| Farakan focus group | Definition of preterm birth | n/a | person 1 | A premature birth is a birth where the woman gave birth before reaching 9 months. | person 1 | 77 A premature birth is a birth where the woman gave birth before reaching 9 months. |
| Farakan focus group | Definition of preterm birth | n/a | person 2 | They will complete. It's before 32 weeks. | person 2 | 78 They will complete. It's before 32 weeks. |
| Farakan focus group | Definition of preterm birth | n/a | person 5 | So, before 32 weeks. | person 5 | 79 So, before 32 weeks. |
| Farakan focus group | Definition of preterm birth | n/a | person 4 | It's before the end. | person 4 | 80 It's before the end. |
| Farakan focus group | Definition of preterm birth | n/a | person 7 | And it's typically a low-weight baby. | person 7 | 81 And it's typically a low-weight baby. |
| Farakan major | Definition of preterm birth | n/a | person 1 | It's a child born prematurely, which means before 9 months. There's also the aspect of weight. We often say less than 2.5 kilograms, but there are also children who weigh less than 2.5 kilograms and are born at full term. | person 1 | 82 It's a child born prematurely, which means before 9 months. There's also the aspect of weight. We often say less than 2.5 kilograms, but there are also children who weigh less than 2.5 kilograms and are born at full term. |
| Farakan major | Definition of preterm birth | n/a | person 1 | No, not necessarily. They may weigh less than 2.5 kilograms, but if they were born at full term, within the required time frame, they are not considered premature. | person 1 | 83 No, not necessarily. They may weigh less than 2.5 kilograms, but if they were born at full term, within the required time frame, they are not considered premature. |
| Farakan maternity manager | Definition of preterm birth | n/a | person 1 | Premature birth, in our understanding, refers to any preterm birth. We define prematurity as a birth that occurs between 28 and 37 weeks of pregn/acy. So, any birth that happens before 37 weeks is classified as premature. | person 1 | 84 Premature birth, in our understanding, refers to any preterm birth. We define prematurity as a birth that occurs between 28 and 37 weeks of pregn/acy. So, any birth that happens before 37 weeks is classified as premature. |
| Accart-ville focus group | Measurement of preterm birth | n/a | person 6 | Measure? | person 6 | 85 Measure? |
| Accart-ville focus group | Measurement of preterm birth | n/a | person 1 | Are you asking about the parameters to determine prematurity during labor or after the child's birth? | person 1 | 86 Are you asking about the parameters to determine prematurity during labor or after the child's birth? |
| Accart-ville focus group | Measurement of preterm birth | n/a | person 1 | During labor, we measure the fundal height, which should match certain standards. We also consider the date of the patient's last period and review ultrasound results if available | person 1 | 87 During labor, we measure the fundal height, which should match certain standards. We also consider the date of the patient's last period and review ultrasound results if available |
| Accart-ville focus group | Measurement of preterm birth | n/a | person 1 | In a full-term pregn/acy, the fundal height typically starts at around 32 cm. In the third trimester, we usually add 3 cm to the measurement to approximate the gestational age | person 1 | 88 In a full-term pregn/acy, the fundal height typically starts at around 32 cm. In the third trimester, we usually add 3 cm to the measurement to approximate the gestational age |
| Accart-ville CMU manager | Measurement of preterm birth | n/a | person 1 | Generally, we rely on dating ultrasounds or the chronological age of the pregn/acy. Sometimes, we identify premature children based on signs of prematurity, especially if mothers are unable to provide accurate age information. We assess whether a birth is premature by considering the baby's weight and other physical signs of prematurity | person 1 | 89 Generally, we rely on dating ultrasounds or the chronological age of the pregn/acy. Sometimes, we identify premature children based on signs of prematurity, especially if mothers are unable to provide accurate age information. We assess whether a birth is premature by considering the baby's weight and other physical signs of prematurity |
| Accart-ville maternity manager | Measurement of preterm birth | n/a | person 1 | We primarily use gestational age as a starting point. Additionally, we assess various physiological criteria indicative of prematurity. These include the absence of cartilage in the pinnae of the ears, thin abdominal skin with visible small veins, and specific genital characteristics. For male newborns, this involves the non-descent of the testicles, and for female newborns, we look for clitoral hypertrophy. Besides these, we also consider the weight and height, which are typically lower than those of a full-term newborn. | person 1 | 90 We primarily use gestational age as a starting point. Additionally, we assess various physiological criteria indicative of prematurity. These include the absence of cartilage in the pinnae of the ears, thin abdominal skin with visible small veins, and specific genital characteristics. For male newborns, this involves the non-descent of the testicles, and for female newborns, we look for clitoral hypertrophy. Besides these, we also consider the weight and height, which are typically lower than those of a full-term newborn. |
| CMA Do focus group | Measurement of preterm birth | n/a | person 2 | One indicator is low birth weight. | person 2 | 91 One indicator is low birth weight. |
| CMA Do focus group | Measurement of preterm birth | n/a | person 2 | There are several indicators. Low birth weight, typically less than 2,500 grams, is a key factor. The baby's coloration can also be a clue; a premature baby often has a slightly red appearance. Additionally, the development of the genitalia, such as the labia minora in girls, can be indicative. | person 2 | 92 There are several indicators. Low birth weight, typically less than 2,500 grams, is a key factor. The baby's coloration can also be a clue; a premature baby often has a slightly red appearance. Additionally, the development of the genitalia, such as the labia minora in girls, can be indicative. |
| CMA Do focus group | Measurement of preterm birth | n/a | person 6 | Gestational age is another important factor. At birth, the absence of vernix caseosa can be a sign. The baby's weight alone doesn't always give a complete picture; for instance, a diabetic mother might have a premature baby that weighs 3,000 grams. We look at the overall gestational age along with the absence of vernix caseosa. For boys, undescended scrotal sacs can be a sign, and for girls, the prominence of the labia minora over the labia majora. | person 6 | 93 Gestational age is another important factor. At birth, the absence of vernix caseosa can be a sign. The baby's weight alone doesn't always give a complete picture; for instance, a diabetic mother might have a premature baby that weighs 3,000 grams. We look at the overall gestational age along with the absence of vernix caseosa. For boys, undescended scrotal sacs can be a sign, and for girls, the prominence of the labia minora over the labia majora. |
| CMA Do focus group | Measurement of preterm birth | n/a | person 8 | We can also consider the baby's head circumference, thoracic circumference, and length. The overall appearance of the baby often gives us a clear indication of prematurity. | person 8 | 94 We can also consider the baby's head circumference, thoracic circumference, and length. The overall appearance of the baby often gives us a clear indication of prematurity. |
| Gynecologist 1 | Measurement of preterm birth | n/a | person 1 | As I mentioned, at childbirth, we first consider the information available from the mother. Often, they have ultrasounds that give the gestational age. In some cases, they know the date of their last menstrual period, so we can estimate the pregn/acy age from that and determine if it's premature. Another criterion at birth is the baby's weight. Generally, when the weight is around 2 kg, we also look at morphological criteria, like the nipples and ear lobules. If we find that a child with below-normal weight exhibits these premature characteristics, we classify them as premature. | person 1 | 95 As I mentioned, at childbirth, we first consider the information available from the mother. Often, they have ultrasounds that give the gestational age. In some cases, they know the date of their last menstrual period, so we can estimate the pregn/acy age from that and determine if it's premature. Another criterion at birth is the baby's weight. Generally, when the weight is around 2 kg, we also look at morphological criteria, like the nipples and ear lobules. If we find that a child with below-normal weight exhibits these premature characteristics, we classify them as premature. |
| Gynecologist 2 | Measurement of preterm birth | n/a | person 1 | If we know the gestational age, either from the date of the last menstrual period or from a dating ultrasound, we can determine if the pregn/acy is of a certain number of weeks and hence consider the likelihood of premature birth. In cases where we don’t have this information, we measure the fundal height and examine the child at birth. It's the combination of these elements that helps us conclude whether the child is premature. | person 1 | 96 If we know the gestational age, either from the date of the last menstrual period or from a dating ultrasound, we can determine if the pregn/acy is of a certain number of weeks and hence consider the likelihood of premature birth. In cases where we don’t have this information, we measure the fundal height and examine the child at birth. It's the combination of these elements that helps us conclude whether the child is premature. |
| MCD | Measurement of preterm birth | n/a | person 1 | As I mentioned in its definition, any pregn/acy that ends before full term is considered premature. Specifically, if a birth occurs before 37 weeks of amenorrhea, we conclude that it is a premature birth. | person 1 | 97 As I mentioned in its definition, any pregn/acy that ends before full term is considered premature. Specifically, if a birth occurs before 37 weeks of amenorrhea, we conclude that it is a premature birth. |
| CMA Do maternity care unit manager | Measurement of preterm birth | n/a | person 1 | We measure it by conducting an examination. Measuring the fundal height can give us some guidance. We also consider the date of the woman's last menstrual period, which helps us calculate the probable date of delivery. | person 1 | 98 We measure it by conducting an examination. Measuring the fundal height can give us some guidance. We also consider the date of the woman's last menstrual period, which helps us calculate the probable date of delivery. |
| Colma1 focus group | Measurement of preterm birth | n/a | person 3 | For women who have had ultrasounds, we can determine premature birth based on the ultrasound results. Clinically, at delivery, there are certain signs that indicate the child is premature. | person 3 | 99 For women who have had ultrasounds, we can determine premature birth based on the ultrasound results. Clinically, at delivery, there are certain signs that indicate the child is premature. |
| Colma1 focus group | Measurement of preterm birth | n/a | person 3 | We look for low birth weight and the absence of vernix caseosa on the premature baby. Premature babies often don't respond to certain reflexes, like sucking reflexes. There are multiple reflexes a premature baby may not exhibit. | person 3 | 100 We look for low birth weight and the absence of vernix caseosa on the premature baby. Premature babies often don't respond to certain reflexes, like sucking reflexes. There are multiple reflexes a premature baby may not exhibit. |
| Colma1 focus group | Measurement of preterm birth | n/a | person 4 | During labor, the fundal height can be a warning sign. A normal fundal height should reach 32 cm when the pregn/acy is full term. If a woman's fundal height is only 27 or 28 cm, it indicates that the pregn/acy is not full term. Additionally, as mentioned, certain reflexes are lacking in premature babies, and generally, a low birth weight, sometimes as little as 1 kg, can be a sign. | person 4 | 101 During labor, the fundal height can be a warning sign. A normal fundal height should reach 32 cm when the pregn/acy is full term. If a woman's fundal height is only 27 or 28 cm, it indicates that the pregn/acy is not full term. Additionally, as mentioned, certain reflexes are lacking in premature babies, and generally, a low birth weight, sometimes as little as 1 kg, can be a sign. |
| Colma1 focus group | Measurement of preterm birth | n/a | person 4 | Normally, a healthy pregn/acy's fundal height reaches 32 cm at full term. Excessive or insufficient fundal height, like 27 or 28 cm, can indicate that the pregn/acy is not full term. | person 4 | 102 Normally, a healthy pregn/acy's fundal height reaches 32 cm at full term. Excessive or insufficient fundal height, like 27 or 28 cm, can indicate that the pregn/acy is not full term. |
| Colma1 major | Measurement of preterm birth | n/a | person 1 | The key parameter for measuring premature birth is the age of the pregn/acy, which is very important. I believe that is the primary determin/at. | person 1 | 103 The key parameter for measuring premature birth is the age of the pregn/acy, which is very important. I believe that is the primary determin/at. |
| Colma1 maternity manager | Measurement of preterm birth | n/a | person 1 | First, it's through interrogation, specifically regarding the date of the last menstrual period. That's usually the starting point. If the date of the last period is unknown, we rely on early pregn/acy indicators, like a positive pregn/acy test after a missed period, to estimate the gestational age. Measuring fundal height is also helpful. Ultrasound, especially in the first trimester (between five to twelve weeks of amenorrhea), is very reliable for determining pregn/acy age. The abdominal perimeter measurement is another method, but it's not as widely known. Clinically, at birth, we can also recognize a premature child based on various signs. | person 1 | 104 First, it's through interrogation, specifically regarding the date of the last menstrual period. That's usually the starting point. If the date of the last period is unknown, we rely on early pregn/acy indicators, like a positive pregn/acy test after a missed period, to estimate the gestational age. Measuring fundal height is also helpful. Ultrasound, especially in the first trimester (between five to twelve weeks of amenorrhea), is very reliable for determining pregn/acy age. The abdominal perimeter measurement is another method, but it's not as widely known. Clinically, at birth, we can also recognize a premature child based on various signs. |
| Colma1 maternity manager | Measurement of preterm birth | n/a | person 1 | A hypotrophic child may be covered in vernix caseosa, which is easy to spot. In contrast, a premature child may have little to no vernix caseosa. Their skin coloring is often red instead of pink. Their hair might be less developed compared to a full-term child. In terms of genital development, in girls, the labia may not fully cover the clitoris, and in boys, the testicles may not be descended. Hypotrophic children usually display archaic reflexes and appear more awake. | person 1 | 105 A hypotrophic child may be covered in vernix caseosa, which is easy to spot. In contrast, a premature child may have little to no vernix caseosa. Their skin coloring is often red instead of pink. Their hair might be less developed compared to a full-term child. In terms of genital development, in girls, the labia may not fully cover the clitoris, and in boys, the testicles may not be descended. Hypotrophic children usually display archaic reflexes and appear more awake. |
| Farakan focus group | Measurement of preterm birth | n/a | person 1 | In our facility, we mainly rely on the weeks of amenorrhea. For instance, if a woman has less than 37 weeks of amenorrhea, we classify the birth as premature. When a child is born weighing less than 2,500 grams, we also look for accompanying signs and examine the newborn for specific indications of prematurity. | person 1 | 106 In our facility, we mainly rely on the weeks of amenorrhea. For instance, if a woman has less than 37 weeks of amenorrhea, we classify the birth as premature. When a child is born weighing less than 2,500 grams, we also look for accompanying signs and examine the newborn for specific indications of prematurity. |
| Farakan focus group | Measurement of preterm birth | n/a | person 3 | A key factor is very low birth weight, less than 2,500 kilograms. | person 3 | 107 A key factor is very low birth weight, less than 2,500 kilograms. |
| Farakan focus group | Measurement of preterm birth | n/a | person 4 | Our approach depends on the child's weight. If it's a very low weight that warrants referral, we do so. Otherwise, we offer advice on proper care. The climate also influences our care methods. For example, in colder months like December, we emphasize keeping the child warm and advising skin-to-skin contact with the mother. | person 4 | 108 Our approach depends on the child's weight. If it's a very low weight that warrants referral, we do so. Otherwise, we offer advice on proper care. The climate also influences our care methods. For example, in colder months like December, we emphasize keeping the child warm and advising skin-to-skin contact with the mother. |
| Farakan focus group | Measurement of preterm birth | n/a | person 2 | We wrap the child in warm, dry cloth and facilitate skin-to-skin contact with the mother. In cases of very premature babies, we refer them to higher-level facilities like the kangaroo unit at the CMA of Dô or, for critical cases, directly to the neonatology department at Souro Sanou Hospital by ambulance. | person 2 | 109 We wrap the child in warm, dry cloth and facilitate skin-to-skin contact with the mother. In cases of very premature babies, we refer them to higher-level facilities like the kangaroo unit at the CMA of Dô or, for critical cases, directly to the neonatology department at Souro Sanou Hospital by ambulance. |
| Farakan focus group | Measurement of preterm birth | n/a | person 6 | Our facility rarely manages cases of premature babies. We usually refer them to higher-level facilities for better care after delivery. | person 6 | 110 Our facility rarely manages cases of premature babies. We usually refer them to higher-level facilities for better care after delivery. |
| Farakan focus group | Measurement of preterm birth | n/a | person 4 | Due to a lack of equipment, we prefer immediate referral. We don't have the necessary equipment to adequately care for premature babies here. | person 4 | 111 Due to a lack of equipment, we prefer immediate referral. We don't have the necessary equipment to adequately care for premature babies here. |
| Farakan focus group | Measurement of preterm birth | n/a | person 2 | Yes. We administer vitamin K1 to all newborns here, regardless of whether they are premature or of normal weight. | person 2 | 112 Yes. We administer vitamin K1 to all newborns here, regardless of whether they are premature or of normal weight. |
| Farakan major | Measurement of preterm birth | n/a | person 1 | The main parameters we use are the birth rate and the number of months of pregn/acy. We consider a birth to be premature if it occurs before 37 weeks. | person 1 | 113 The main parameters we use are the birth rate and the number of months of pregn/acy. We consider a birth to be premature if it occurs before 37 weeks. |
| Farakan maternity manager | Measurement of preterm birth | n/a | person 1 | We encounter a variety of cases. If a woman arrives early enough for a referral, we refer her. However, if she arrives late and is already in the active phase of labor, with birth imminent, there's not much we can do. When there's a threat of premature birth, we send her to CHURSS. After delivery, for specialized care, especially for very premature babies, we send them to the pediatric hospital, as we can't manage them here. But there are also babies born prematurely who are relatively healthy in terms of weight and show no signs of distress. In such cases, we can keep them here and provide advice to the mother. | person 1 | 114 We encounter a variety of cases. If a woman arrives early enough for a referral, we refer her. However, if she arrives late and is already in the active phase of labor, with birth imminent, there's not much we can do. When there's a threat of premature birth, we send her to CHURSS. After delivery, for specialized care, especially for very premature babies, we send them to the pediatric hospital, as we can't manage them here. But there are also babies born prematurely who are relatively healthy in terms of weight and show no signs of distress. In such cases, we can keep them here and provide advice to the mother. |
| Farakan maternity manager | Measurement of preterm birth | n/a | person 1 | How do we measure it? It's based on the level of care needed. Premature babies require special care, different from what other children need. | person 1 | 115 How do we measure it? It's based on the level of care needed. Premature babies require special care, different from what other children need. |
| Accart-ville focus group | Measurement of preterm birth | Control of the date of the last period | person 4 | In Africa, the number is quite low. Out of 10 patients, maybe around 4 know the date. Due to illiteracy, many women relate it to events or seasons. For example, they might refer to the time of corn harvesting or the month of Ramadan. However, such references don't provide an exact day. | person 4 | 116 In Africa, the number is quite low. Out of 10 patients, maybe around 4 know the date. Due to illiteracy, many women relate it to events or seasons. For example, they might refer to the time of corn harvesting or the month of Ramadan. However, such references don't provide an exact day. |
| Accart-ville focus group | Measurement of preterm birth | Control of the date of the last period | person 1 | Not all women do. | person 1 | 117 Not all women do. |
| Accart-ville focus group | Measurement of preterm birth | Control of the date of the last period | person 1 | I would say about 4 out of 10. There aren't many. | person 1 | 118 I would say about 4 out of 10. There aren't many. |
| Accart-ville focus group | Measurement of preterm birth | Control of the date of the last period | person 1 | Often, they come around three months into their pregn/acy, as that's the traditional recommendation. Though the WHO advises coming as soon as pregn/acy is realized, they typically adhere to the three-month mark. Some come after the first three months. | person 1 | 119 Often, they come around three months into their pregn/acy, as that's the traditional recommendation. Though the WHO advises coming as soon as pregn/acy is realized, they typically adhere to the three-month mark. Some come after the first three months. |
| Accart-ville CMU manager | Measurement of preterm birth | Control of the date of the last period | person 1 | It's quite rare to define that precisely. I always encourage midwives in prenatal consultations to try to correlate the date of the last period with major events to estimate the pregn/acy age. It's sometimes difficult, but we manage. | person 1 | 120 It's quite rare to define that precisely. I always encourage midwives in prenatal consultations to try to correlate the date of the last period with major events to estimate the pregn/acy age. It's sometimes difficult, but we manage. |
| Accart-ville CMU manager | Measurement of preterm birth | Control of the date of the last period | person 1 | It won't exceed three out of ten. | person 1 | 121 It won't exceed three out of ten. |
| Accart-ville CMU manager | Measurement of preterm birth | Control of the date of the last period | person 1 | Usually, it's after 3 to 4 months. Some women, especially at the start of pregn/acy, may delay ANC until after 20 weeks of amenorrhea, around four or five months. | person 1 | 122 Usually, it's after 3 to 4 months. Some women, especially at the start of pregn/acy, may delay ANC until after 20 weeks of amenorrhea, around four or five months. |
| Accart-ville CMU manager | Measurement of preterm birth | Control of the date of the last period | person 1 | It's very rare. Maybe only one out of ten will remember. Even educated women find it difficult to recall if they don't start ANC early. If they've been given a notebook with the date noted down by a health worker, they might remember. But generally, for the first ANC around 16 weeks or more, it's difficult to recall. | person 1 | 123 It's very rare. Maybe only one out of ten will remember. Even educated women find it difficult to recall if they don't start ANC early. If they've been given a notebook with the date noted down by a health worker, they might remember. But generally, for the first ANC around 16 weeks or more, it's difficult to recall. |
| Accart-ville maternity manager | Measurement of preterm birth | Control of the date of the last period | person 1 | Very few actually know their last period date, very few. To give an estimate, I would say about three out of ten. Three is a generous estimate, so on average, it's around three women who know the date of their last period. | person 1 | 124 Very few actually know their last period date, very few. To give an estimate, I would say about three out of ten. Three is a generous estimate, so on average, it's around three women who know the date of their last period. |
| Accart-ville maternity manager | Measurement of preterm birth | Control of the date of the last period | person 1 | Generally speaking, women come late for their first visit. Most of them arrive after the sixteenth week, so they're in the second trimester. | person 1 | 125 Generally speaking, women come late for their first visit. Most of them arrive after the sixteenth week, so they're in the second trimester. |
| Accart-ville maternity manager | Measurement of preterm birth | Control of the date of the last period | person 1 | Many don't know the exact date. They often reference it according to social events within the community or their family, like funerals, baptisms, weddings, or customary celebrations. We then do some calculations to estimate the date. Some may recall the month, like February, but not the specific day. Only a few people know the exact date of their last period. | person 1 | 126 Many don't know the exact date. They often reference it according to social events within the community or their family, like funerals, baptisms, weddings, or customary celebrations. We then do some calculations to estimate the date. Some may recall the month, like February, but not the specific day. Only a few people know the exact date of their last period. |
| CMA Do focus group | Measurement of preterm birth | Control of the date of the last period | person 6 | In our context, it's no more than 2 out of 10. It's quite complicated here. | person 6 | 127 In our context, it's no more than 2 out of 10. It's quite complicated here. |
| CMA Do focus group | Measurement of preterm birth | Control of the date of the last period | person 6 | Many women come at 13 weeks, especially those in urban areas. Some come just for the maternity bag (laughing). But the majority do come in the first trimester. | person 6 | 128 Many women come at 13 weeks, especially those in urban areas. Some come just for the maternity bag (laughing). But the majority do come in the first trimester. |
| CMA Do focus group | Measurement of preterm birth | Control of the date of the last period | person 6 | No. | person 6 | 129 No. |
| CMA Do focus group | Measurement of preterm birth | Control of the date of the last period | person 2 | Yes, some do know. | person 2 | 130 Yes, some do know. |
| CMA Do focus group | Measurement of preterm birth | Control of the date of the last period | person 6 | Those who typically know are women who are actively trying to conceive. They monitor their ovulation, so they're more likely to know. But for multiparous women, it's less common. We can't really exceed 2 out of 10. | person 6 | 131 Those who typically know are women who are actively trying to conceive. They monitor their ovulation, so they're more likely to know. But for multiparous women, it's less common. We can't really exceed 2 out of 10. |
| Gynecologist 1 | Measurement of preterm birth | Control of the date of the last period | person 1 | It's quite rare. Out of 10 women who come for ANC, maybe only one or two know the date of their last period. | person 1 | 132 It's quite rare. Out of 10 women who come for ANC, maybe only one or two know the date of their last period. |
| Gynecologist 1 | Measurement of preterm birth | Control of the date of the last period | person 1 | Although we don’t have a large number of ANC visits, the women who do come usually arrive around 5 months, in the second trimester. It's very rare to see them in the first trimester unless they are experiencing problems | person 1 | 133 Although we don’t have a large number of ANC visits, the women who do come usually arrive around 5 months, in the second trimester. It's very rare to see them in the first trimester unless they are experiencing problems |
| Gynecologist 1 | Measurement of preterm birth | Control of the date of the last period | person 1 | Yes, some do, especially the younger girls. Nowadays, with cell phones and apps, they regularly track their periods. So, when they're late, they can precisely provide the date of their last period using the app. | person 1 | 134 Yes, some do, especially the younger girls. Nowadays, with cell phones and apps, they regularly track their periods. So, when they're late, they can precisely provide the date of their last period using the app. |
| Gynecologist 2 | Measurement of preterm birth | Control of the date of the last period | person 1 | It's hard to give an exact number, but very few are aware of the exact date of their last period. The women often have different systems of calculation compared to ours. They might reference it to a recent week, which is still relatively vague. They often link it to specific events, saying it was around the month of Ramadan or the day after a particular celebration. This makes it challenging to calculate accurately without all the necessary calendars. | person 1 | 135 It's hard to give an exact number, but very few are aware of the exact date of their last period. The women often have different systems of calculation compared to ours. They might reference it to a recent week, which is still relatively vague. They often link it to specific events, saying it was around the month of Ramadan or the day after a particular celebration. This makes it challenging to calculate accurately without all the necessary calendars. |
| Gynecologist 2 | Measurement of preterm birth | Control of the date of the last period | person 1 | I would estimate about 3 or 4 out of 10. It's not a large number. | person 1 | 136 I would estimate about 3 or 4 out of 10. It's not a large number. |
| Gynecologist 2 | Measurement of preterm birth | Control of the date of the last period | person 1 | It varies, but on average, women tend to come within the first 3 months of their pregn/acy. | person 1 | 137 It varies, but on average, women tend to come within the first 3 months of their pregn/acy. |
| MCD | Measurement of preterm birth | Control of the date of the last period | person 1 | It's hard to give a precise number. If we assume a large portion of the population is literate, they may not have the tools to record the date of their last periods. It's not easy to remember for a long time. By extrapolation, maybe half may not know, but I don't have solid data to confirm that. We could refer to the registers for more accurate information. | person 1 | 138 It's hard to give a precise number. If we assume a large portion of the population is literate, they may not have the tools to record the date of their last periods. It's not easy to remember for a long time. By extrapolation, maybe half may not know, but I don't have solid data to confirm that. We could refer to the registers for more accurate information. |
| MCD | Measurement of preterm birth | Control of the date of the last period | person 1 | According to this year's data for our district, about 48.8% of women are seen in the first trimester for their first ANC. Ideally, we prefer to see them in the first trimester for early monitoring. | person 1 | 139 According to this year's data for our district, about 48.8% of women are seen in the first trimester for their first ANC. Ideally, we prefer to see them in the first trimester for early monitoring. |
| MCD | Measurement of preterm birth | Control of the date of the last period | person 1 | Yes, those who are precise do know. They keep track, sometimes in notebooks or with mobile applications. These apps help them track their menstrual cycles and predict fertility periods. However, literacy is key, as educated women are more likely to remember because they can read and write. | person 1 | 140 Yes, those who are precise do know. They keep track, sometimes in notebooks or with mobile applications. These apps help them track their menstrual cycles and predict fertility periods. However, literacy is key, as educated women are more likely to remember because they can read and write. |
| MCD | Measurement of preterm birth | Control of the date of the last period | person 1 | There are practical mobile applications available now that assist in tracking menstrual cycles. They can predict the next period based on stable cycles, helping women prepare for their menstruation. These tools are becoming quite common. | person 1 | 141 There are practical mobile applications available now that assist in tracking menstrual cycles. They can predict the next period based on stable cycles, helping women prepare for their menstruation. These tools are becoming quite common. |
| MCD | Measurement of preterm birth | Control of the date of the last period | person 1 | I see many online, but as I'm not a woman, I don’t use them. I am aware of them through interactions with women | person 1 | 142 I see many online, but as I'm not a woman, I don’t use them. I am aware of them through interactions with women |
| CMA Do maternity care unit manager | Measurement of preterm birth | Control of the date of the last period | person 1 | It's hard to say exactly, as most women don't know the precise date. They often relate it to events like the month of Lent or other feasts. It's challenging to calculate accurately based on such references. Our questioning doesn't always enable them to provide an exact date, which results in a very low number. On a scale of 1 to 10, I would estimate about 4. | person 1 | 143 It's hard to say exactly, as most women don't know the precise date. They often relate it to events like the month of Lent or other feasts. It's challenging to calculate accurately based on such references. Our questioning doesn't always enable them to provide an exact date, which results in a very low number. On a scale of 1 to 10, I would estimate about 4. |
| CMA Do maternity care unit manager | Measurement of preterm birth | Control of the date of the last period | person 1 | It varies. Some women come early, as soon as they notice a missed period. Others wait until around 12 or 14 weeks, or even longer. We start the ANC as soon as we make a pregn/acy diagnosis, which is often around 12 weeks | person 1 | 144 It varies. Some women come early, as soon as they notice a missed period. Others wait until around 12 or 14 weeks, or even longer. We start the ANC as soon as we make a pregn/acy diagnosis, which is often around 12 weeks |
| CMA Do maternity care unit manager | Measurement of preterm birth | Control of the date of the last period | person 1 | I'd say about 5. | person 1 | 145 I'd say about 5. |
| CMA Do maternity care unit manager | Measurement of preterm birth | Control of the date of the last period | person 1 | Most of the time, they don’t know precisely. Those who do manage to provide a date, as I mentioned earlier, are about 4 out of 10. | person 1 | 146 Most of the time, they don’t know precisely. Those who do manage to provide a date, as I mentioned earlier, are about 4 out of 10. |
| Colma1 focus group | Measurement of preterm birth | Control of the date of the last period | person 5 | In my opinion, it won't exceed 2 out of 10 who know their last period date | person 5 | 147 In my opinion, it won't exceed 2 out of 10 who know their last period date |
| Colma1 focus group | Measurement of preterm birth | Control of the date of the last period | person 3 | They usually can't give a precise date. They might talk about being a month or two late. That's a common way women express it. | person 3 | 148 They usually can't give a precise date. They might talk about being a month or two late. That's a common way women express it. |
| Colma1 focus group | Measurement of preterm birth | Control of the date of the last period | person 3 | Let's let the ANC staff answer. | person 3 | 149 Let's let the ANC staff answer. |
| Colma1 focus group | Measurement of preterm birth | Control of the date of the last period | peerson 6 | Most start in the 2nd and 3rd trimester. They don't come early. | peerson 6 | 150 Most start in the 2nd and 3rd trimester. They don't come early. |
| Colma1 focus group | Measurement of preterm birth | Control of the date of the last period | person 6 | No, they usually remember the month. We try to calculate roughly from that | person 6 | 151 No, they usually remember the month. We try to calculate roughly from that |
| Colma1 focus group | Measurement of preterm birth | Control of the date of the last period | person 6 | About 3 out of 10, no more (laughing). | person 6 | 152 About 3 out of 10, no more (laughing). |
| Colma1 focus group | Measurement of preterm birth | Control of the date of the last period | person 2 | The exact date? | person 2 | 153 The exact date? |
| Colma1 focus group | Measurement of preterm birth | Control of the date of the last period | person 1 | We're talking about the exact last period date | person 1 | 154 We're talking about the exact last period date |
| Colma1 focus group | Measurement of preterm birth | Control of the date of the last period | person 3 | In my opinion, out of 10, it doesn't even exceed 2. Educated women, like workers, may provide the exact date, but others will just mention it was in the past month or two. | person 3 | 155 In my opinion, out of 10, it doesn't even exceed 2. Educated women, like workers, may provide the exact date, but others will just mention it was in the past month or two. |
| Colma1 focus group | Measurement of preterm birth | Control of the date of the last period | person 3 | Yes, exactly. | person 3 | 156 Yes, exactly. |
| Colma1 major | Measurement of preterm birth | Control of the date of the last period | person 1 | Out of 10 women... | person 1 | 157 Out of 10 women... |
| Colma1 major | Measurement of preterm birth | Control of the date of the last period | person 1 | As an estimate, I would say about 3 to 4 out of 10. | person 1 | 158 As an estimate, I would say about 3 to 4 out of 10. |
| Colma1 major | Measurement of preterm birth | Control of the date of the last period | person 1 | Usually, it's in the first trimester. | person 1 | 159 Usually, it's in the first trimester. |
| Colma1 major | Measurement of preterm birth | Control of the date of the last period | person 1 | Not precisely, which is why I said earlier that they are rare. Out of 10 women, maybe 3 can know the date of their last period. The other 7 generally don't have an idea. | person 1 | 160 Not precisely, which is why I said earlier that they are rare. Out of 10 women, maybe 3 can know the date of their last period. The other 7 generally don't have an idea. |
| Colma1 maternity manager | Measurement of preterm birth | Control of the date of the last period | person 1 | Considering the community at the CSPS of Colma 1, which is similar to a rural area, I would say about four out of ten women know the date of their last period. With more in-depth questioning, we might be able to add another three to four women to that number. It's not just about asking the question directly. For example, even among civil servants, including teachers and healthcare workers, many initially say they don't remember the date of their last period. But if we link the date to specific events, like something they saw on Facebook or during the month of Ramadan, more women can recall approximately when it was. With this approach, we might get six to seven out of ten women providing an estimate. | person 1 | 161 Considering the community at the CSPS of Colma 1, which is similar to a rural area, I would say about four out of ten women know the date of their last period. With more in-depth questioning, we might be able to add another three to four women to that number. It's not just about asking the question directly. For example, even among civil servants, including teachers and healthcare workers, many initially say they don't remember the date of their last period. But if we link the date to specific events, like something they saw on Facebook or during the month of Ramadan, more women can recall approximately when it was. With this approach, we might get six to seven out of ten women providing an estimate. |
| Colma1 maternity manager | Measurement of preterm birth | Control of the date of the last period | person 1 | Yes, it's more about the month. A few, about four out of ten, can give the exact date. Most women tend to provide the ending date of their period. Then, with further investigation, such as asking about the duration of their period, we can deduce the starting date. For instance, if today is the 14th and they say their period ended on the 14th and lasted four days, we know it started on the 10th. | person 1 | 162 Yes, it's more about the month. A few, about four out of ten, can give the exact date. Most women tend to provide the ending date of their period. Then, with further investigation, such as asking about the duration of their period, we can deduce the starting date. For instance, if today is the 14th and they say their period ended on the 14th and lasted four days, we know it started on the 10th. |
| Colma1 maternity manager | Measurement of preterm birth | Control of the date of the last period | person 1 | With the current awareness campaigns, we're seeing women come a bit earlier than before. However, there's a common belief that prenatal weighing should start from five months. Few women come at three months; most arrive at the beginning of the second trimester. This is despite our advice. Sometimes, women who initially visit for pregn/acy-related symptoms delay their ANC even after being advised to start early. They may return with the same consultation slip, indicating pregn/acy, for other issues. It's a challenge, and changing this pattern isn't easy | person 1 | 163 With the current awareness campaigns, we're seeing women come a bit earlier than before. However, there's a common belief that prenatal weighing should start from five months. Few women come at three months; most arrive at the beginning of the second trimester. This is despite our advice. Sometimes, women who initially visit for pregn/acy-related symptoms delay their ANC even after being advised to start early. They may return with the same consultation slip, indicating pregn/acy, for other issues. It's a challenge, and changing this pattern isn't easy |
| Farakan focus group | Measurement of preterm birth | Control of the date of the last period | person 3 | If we had to give a percentage, it would be about 1 in 10. | person 3 | 164 If we had to give a percentage, it would be about 1 in 10. |
| Farakan focus group | Measurement of preterm birth | Control of the date of the last period | person 5 | Only 1 out of 10, and even that's rare. | person 5 | 165 Only 1 out of 10, and even that's rare. |
| Farakan focus group | Measurement of preterm birth | Control of the date of the last period | person 6 | I would say practically 0 out of 10, but realistically, it's about 1 out of 10. | person 6 | 166 I would say practically 0 out of 10, but realistically, it's about 1 out of 10. |
| Farakan focus group | Measurement of preterm birth | Control of the date of the last period | person 7 | I also estimate it at about 1 out of 10. | person 7 | 167 I also estimate it at about 1 out of 10. |
| Farakan focus group | Measurement of preterm birth | Control of the date of the last period | person 8 | Considering we're dealing with a population that often lacks formal education, determining the exact date of the last menstrual period is challenging. So, the percentage is really close to 1, if not zero. | person 8 | 168 Considering we're dealing with a population that often lacks formal education, determining the exact date of the last menstrual period is challenging. So, the percentage is really close to 1, if not zero. |
| Farakan focus group | Measurement of preterm birth | Control of the date of the last period | person 4 | In CPN, we do ask them, but they usually say they don't know. The percentage is around 1 in 10. | person 4 | 169 In CPN, we do ask them, but they usually say they don't know. The percentage is around 1 in 10. |
| Farakan focus group | Measurement of preterm birth | Control of the date of the last period | person 7 | Some come as early as the 6th week, while others come earlier | person 7 | 170 Some come as early as the 6th week, while others come earlier |
| Farakan focus group | Measurement of preterm birth | Control of the date of the last period | person 4 | I think it's around 4 weeks, just a month, we see more women coming in. | person 4 | 171 I think it's around 4 weeks, just a month, we see more women coming in. |
| Farakan focus group | Measurement of preterm birth | Control of the date of the last period | person 5 | There are women who come in the first trimester at 3 months, but for others, it extends beyond the first trimester. It really varies. | person 5 | 172 There are women who come in the first trimester at 3 months, but for others, it extends beyond the first trimester. It really varies. |
| Farakan focus group | Measurement of preterm birth | Control of the date of the last period | person 3 | Some come at 12 weeks, others from 4 weeks, and there are those who come for their first ANC as late as 6 months. | person 3 | 173 Some come at 12 weeks, others from 4 weeks, and there are those who come for their first ANC as late as 6 months. |
| Farakan focus group | Measurement of preterm birth | Control of the date of the last period | person 4 | With increased awareness, most women now understand the importance and come in the first trimester, within the first 3 months. | person 4 | 174 With increased awareness, most women now understand the importance and come in the first trimester, within the first 3 months. |
| Farakan focus group | Measurement of preterm birth | Control of the date of the last period | person 6 | The majority are in the first trimester, and others come around the 4th month. | person 6 | 175 The majority are in the first trimester, and others come around the 4th month. |
| Farakan major | Measurement of preterm birth | Control of the date of the last period | person 1 | It's often difficult... | person 1 | 176 It's often difficult... |
| Farakan major | Measurement of preterm birth | Control of the date of the last period | person 1 | As an estimate, I would say about half of them do not know the date of their last period. | person 1 | 177 As an estimate, I would say about half of them do not know the date of their last period. |
| Farakan major | Measurement of preterm birth | Control of the date of the last period | person 1 | Generally, many come even after the first trimester. Awareness campaigns have improved this a bit. According to our reports, the majority still come after the first trimester. | person 1 | 178 Generally, many come even after the first trimester. Awareness campaigns have improved this a bit. According to our reports, the majority still come after the first trimester. |
| Farakan major | Measurement of preterm birth | Control of the date of the last period | person 1 | They often know the month, give or take a few weeks. But recalling the exact day is usually difficult, especially for those who are not very literate. | person 1 | 179 They often know the month, give or take a few weeks. But recalling the exact day is usually difficult, especially for those who are not very literate. |
| Farakan maternity manager | Measurement of preterm birth | Control of the date of the last period | person 1 | Can I really give a number? (Laughs) It's usually difficult for women to recall the exact date of their last period. They generally provide the month, like it's been 2, 3, 4, or 5 months, but knowing the specific date is hard. Maybe out of 10, about 3 can recall the exact date of their last period. | person 1 | 180 Can I really give a number? (Laughs) It's usually difficult for women to recall the exact date of their last period. They generally provide the month, like it's been 2, 3, 4, or 5 months, but knowing the specific date is hard. Maybe out of 10, about 3 can recall the exact date of their last period. |
| Farakan maternity manager | Measurement of preterm birth | Control of the date of the last period | person 1 | Ideally, it should be in the first trimester. As soon as a woman realizes she's pregn/at, she should start ANC. However, we've noticed that they often don't come early. Many arrive at 4 or 5 months. In the past, with only 4 scheduled ANC visits, women tended not to come early. But now, with up to 8 contacts and monthly appointments, they should start ANC earlier. While some do come early, many still don't. | person 1 | 181 Ideally, it should be in the first trimester. As soon as a woman realizes she's pregn/at, she should start ANC. However, we've noticed that they often don't come early. Many arrive at 4 or 5 months. In the past, with only 4 scheduled ANC visits, women tended not to come early. But now, with up to 8 contacts and monthly appointments, they should start ANC earlier. While some do come early, many still don't. |
| Accart-ville focus group | Measurement of preterm birth | Control of the date of the last period by women who present late to their ANC | person 1 | Those who were able to provide the date of their last period in the first trimester usually can still recall it later on. Yes. | person 1 | 182 Those who were able to provide the date of their last period in the first trimester usually can still recall it later on. Yes. |
| Accart-ville CMU manager | Measurement of preterm birth | Control of the date of the last period by women who present late to their ANC | person 1 | It's rare to find someone who can recall this date; it's very unusual. That's why we often rely on the first ultrasound for dating, but I always recommend getting an ultrasound at the first contact to try to accurately date the pregn/acy | person 1 | 183 It's rare to find someone who can recall this date; it's very unusual. That's why we often rely on the first ultrasound for dating, but I always recommend getting an ultrasound at the first contact to try to accurately date the pregn/acy |
| Accart-ville maternity manager | Measurement of preterm birth | Control of the date of the last period by women who present late to their ANC | person 1 | No, most of them don't remember the date of their last period. | person 1 | 184 No, most of them don't remember the date of their last period. |
| CMA Do focus group | Measurement of preterm birth | Control of the date of the last period by women who present late to their ANC | person 6 | No. If she couldn’t recall the date at the first ANC, it's unlikely she'll remember it later. Since she couldn't find it during the first visit. | person 6 | 185 No. If she couldn’t recall the date at the first ANC, it's unlikely she'll remember it later. Since she couldn't find it during the first visit. |
| Gynecologist 1 | Measurement of preterm birth | Control of the date of the last period by women who present late to their ANC | person 1 | No, generally, if they haven't noted it down, especially outside of the app, by the time they are 5 or 6 months pregn/at, they often don't remember | person 1 | 186 No, generally, if they haven't noted it down, especially outside of the app, by the time they are 5 or 6 months pregn/at, they often don't remember |
| Gynecologist 2 | Measurement of preterm birth | Control of the date of the last period by women who present late to their ANC | person 1 | No, they often don't remember. This is why having the first consultation early is important. It helps us to approximately determine the start of the pregn/acy and the date of the last menstrual period. As the pregn/acy progresses, they tend to forget. However, when we diagnose pregn/acy early on, we document it in their health notebook for future reference. | person 1 | 187 No, they often don't remember. This is why having the first consultation early is important. It helps us to approximately determine the start of the pregn/acy and the date of the last menstrual period. As the pregn/acy progresses, they tend to forget. However, when we diagnose pregn/acy early on, we document it in their health notebook for future reference. |
| MCD | Measurement of preterm birth | Control of the date of the last period by women who present late to their ANC | person 1 | It's not always obvious, but we document it during ANC as soon as she recalls. The ANC record includes various details like the date of the last period, previous pregn/acies, and miscarriage history to facilitate pregn/acy monitoring | person 1 | 188 It's not always obvious, but we document it during ANC as soon as she recalls. The ANC record includes various details like the date of the last period, previous pregn/acies, and miscarriage history to facilitate pregn/acy monitoring |
| CMA Do maternity care unit manager | Measurement of preterm birth | Control of the date of the last period by women who present late to their ANC | person 1 | Once we ask and record the date, we don’t usually revisit the question. We continue the ANC referring to the initially given date. It’s possible they might remember, especially if they can read and have noted it in their notebook, but we don’t typically ask them to recall it again since it's already documented. | person 1 | 189 Once we ask and record the date, we don’t usually revisit the question. We continue the ANC referring to the initially given date. It’s possible they might remember, especially if they can read and have noted it in their notebook, but we don’t typically ask them to recall it again since it's already documented. |
| Colma1 focus group | Measurement of preterm birth | Control of the date of the last period by women who present late to their ANC | person 3 | Remember? They usually never remember. From the start, they might say it's been a month, and it continues like that. They often even ask us how many months it has been. We usually refer to the notebook, but it's more a formality. | person 3 | 190 Remember? They usually never remember. From the start, they might say it's been a month, and it continues like that. They often even ask us how many months it has been. We usually refer to the notebook, but it's more a formality. |
| Colma1 major | Measurement of preterm birth | Control of the date of the last period by women who present late to their ANC | person 1 | Not always. As time passes, they start to forget. At the beginning, in the first trimester, some might recall it was about a month ago when they last saw their period. But as the pregn/acy progresses and they start prenatal care, they tend to focus more on how many months pregn/at they are, rather than remembering the exact date of their last period. | person 1 | 191 Not always. As time passes, they start to forget. At the beginning, in the first trimester, some might recall it was about a month ago when they last saw their period. But as the pregn/acy progresses and they start prenatal care, they tend to focus more on how many months pregn/at they are, rather than remembering the exact date of their last period. |
| Colma1 maternity manager | Measurement of preterm birth | Control of the date of the last period by women who present late to their ANC | person 1 | In the first trimester, if questioned properly, most women can recall the month of their last period. The exact date is often not remembered, but they can usually provide a close approximation. | person 1 | 192 In the first trimester, if questioned properly, most women can recall the month of their last period. The exact date is often not remembered, but they can usually provide a close approximation. |
| Farakan focus group | Measurement of preterm birth | Control of the date of the last period by women who present late to their ANC | person 3 | I don't think so. Often, when they give dates, we predict excesses, but after investigation, we find there is no excess. | person 3 | 193 I don't think so. Often, when they give dates, we predict excesses, but after investigation, we find there is no excess. |
| Farakan focus group | Measurement of preterm birth | Control of the date of the last period by women who present late to their ANC | person 5 | Many women do not know the exact date of their last period | person 5 | 194 Many women do not know the exact date of their last period |
| Farakan focus group | Measurement of preterm birth | Control of the date of the last period by women who present late to their ANC | person 7 | It's challenging because some are on contraceptive methods which affect their menstruation. They might be surprised by a pregn/acy and struggle to know the exact date of their last period. Some are aware, but they are few. | person 7 | 195 It's challenging because some are on contraceptive methods which affect their menstruation. They might be surprised by a pregn/acy and struggle to know the exact date of their last period. Some are aware, but they are few. |
| Farakan focus group | Measurement of preterm birth | Control of the date of the last period by women who present late to their ANC | person 4 | They generally don't know. | person 4 | 196 They generally don't know. |
| Farakan focus group | Measurement of preterm birth | Control of the date of the last period by women who present late to their ANC | person 8 | Sometimes they relate it to events, like not seeing their period since Tabaski or Ramadan, so we help them count from there. They don't determine it precisely. | person 8 | 197 Sometimes they relate it to events, like not seeing their period since Tabaski or Ramadan, so we help them count from there. They don't determine it precisely. |
| Farakan focus group | Measurement of preterm birth | Control of the date of the last period by women who present late to their ANC | person 2 | It's in terms of months. | person 2 | 198 It's in terms of months. |
| Farakan major | Measurement of preterm birth | Control of the date of the last period by women who present late to their ANC | person 1 | It's mainly the literate people, like civil servants, who know how to read and write, that remember. For illiterate ones, we often have to assist them with calendars and such. | person 1 | 199 It's mainly the literate people, like civil servants, who know how to read and write, that remember. For illiterate ones, we often have to assist them with calendars and such. |
| Farakan maternity manager | Measurement of preterm birth | Control of the date of the last period by women who present late to their ANC | person 1 | Yes, some do know precisely. They often relate it to holidays, like during Ramadan or Lent, to estimate the month, but the exact date is more complicated. We can usually calculate based on the month they provide | person 1 | 200 Yes, some do know precisely. They often relate it to holidays, like during Ramadan or Lent, to estimate the month, but the exact date is more complicated. We can usually calculate based on the month they provide |
| Farakan maternity manager | Measurement of preterm birth | Control of the date of the last period by women who present late to their ANC | person 1 | Yes, it's usually in terms of months, not the number of weeks of amenorrhea. | person 1 | 201 Yes, it's usually in terms of months, not the number of weeks of amenorrhea. |
| Accart-ville focus group | Measurement of preterm birth | Ways health workers help women remember the date of their last period | person 1 | If they can't recall, we use alternative methods. One approach is to measure the fundal height, which can give us an approximation of gestational age. We also frequently use ultrasound as it can provide a more precise measurement. | person 1 | 202 If they can't recall, we use alternative methods. One approach is to measure the fundal height, which can give us an approximation of gestational age. We also frequently use ultrasound as it can provide a more precise measurement. |
| Accart-ville CMU manager | Measurement of preterm birth | Ways health workers help women remember the date of their last period | person 1 | When determining gestational age and the woman is unable to recall the date of her last period, we primarily rely on ultrasound measurements. However, there are cases where women haven't undergone an ultrasound. In such situations, we start by considering the number of months they think they might be pregn/at. We also estimate based on a physical examination, particularly by assessing the uterine height | person 1 | 203 When determining gestational age and the woman is unable to recall the date of her last period, we primarily rely on ultrasound measurements. However, there are cases where women haven't undergone an ultrasound. In such situations, we start by considering the number of months they think they might be pregn/at. We also estimate based on a physical examination, particularly by assessing the uterine height |
| Accart-ville maternity manager | Measurement of preterm birth | Ways health workers help women remember the date of their last period | person 1 | In such cases, we rely on clinical assessments. When the chronological age, which is usually determined based on the date of the last period, is unknown, we start with a clinical approach. If we face difficulties on the clinical level, and if the woman's fin/acial situation allows, we might request an ultrasound. However, as our center doesn't have an ultrasound machine, this would need to be done externally. Therefore, it's a last resort. We generally determine the clinical age based on the measurement of the uterine height. | person 1 | 204 In such cases, we rely on clinical assessments. When the chronological age, which is usually determined based on the date of the last period, is unknown, we start with a clinical approach. If we face difficulties on the clinical level, and if the woman's fin/acial situation allows, we might request an ultrasound. However, as our center doesn't have an ultrasound machine, this would need to be done externally. Therefore, it's a last resort. We generally determine the clinical age based on the measurement of the uterine height. |
| CMA Do focus group | Measurement of preterm birth | Ways health workers help women remember the date of their last period | person 6 | In such cases, we refer to significant events that the woman can recall. She might mention that she didn't have her period during a specific time like 'Sounkalo', the month of Lent, or Tabaski. For instance, if she recalls not having her period during the month of Tabaski and if Tabaski was in June, we calculate from that point. Based on this, if her last period was in June, we would estimate that her due date is around March. | person 6 | 205 In such cases, we refer to significant events that the woman can recall. She might mention that she didn't have her period during a specific time like 'Sounkalo', the month of Lent, or Tabaski. For instance, if she recalls not having her period during the month of Tabaski and if Tabaski was in June, we calculate from that point. Based on this, if her last period was in June, we would estimate that her due date is around March. |
| Gynecologist 1 | Measurement of preterm birth | Ways health workers help women remember the date of their last period | person 1 | If she can't recall at all, we use two main methods. One option is to base it on the fundal height. There's a correlation between the fundal height and the gestational age, which gives us an approximate age in months. It's not exact but offers a general idea. The other method is using ultrasound, which can provide us with a gestational age. However, it's important to note that the further along in the pregn/acy, the larger the margin of error with ultrasound measurements. So, we rely on both fundal height and ultrasound to determine the gestational age. | person 1 | 206 If she can't recall at all, we use two main methods. One option is to base it on the fundal height. There's a correlation between the fundal height and the gestational age, which gives us an approximate age in months. It's not exact but offers a general idea. The other method is using ultrasound, which can provide us with a gestational age. However, it's important to note that the further along in the pregn/acy, the larger the margin of error with ultrasound measurements. So, we rely on both fundal height and ultrasound to determine the gestational age. |
| Gynecologist 2 | Measurement of preterm birth | Ways health workers help women remember the date of their last period | person 1 | When a woman comes in and doesn't know the date of her last period, it complicates the calculation of gestational age. In such situations, we base our estimate on the measurement of the fundal height, which varies depending on the stage of pregn/acy. If we have access to an ultrasound machine, we use it to estimate and calculate the term of the pregn/acy. However, if an ultrasound isn't available, we rely on assessing the fundal height to determine the gestational age. | person 1 | 207 When a woman comes in and doesn't know the date of her last period, it complicates the calculation of gestational age. In such situations, we base our estimate on the measurement of the fundal height, which varies depending on the stage of pregn/acy. If we have access to an ultrasound machine, we use it to estimate and calculate the term of the pregn/acy. However, if an ultrasound isn't available, we rely on assessing the fundal height to determine the gestational age. |
| MCD | Measurement of preterm birth | Ways health workers help women remember the date of their last period | person 1 | If a woman can't recall the date of her last period, we generally use two methods for approximation. One method is ultrasound, where we estimate the age of the pregn/acy based on the size of the fetus or embryo. The other method involves measuring the fundal height, which allows us to approximate the pregn/acy age through certain calculation techniques. However, this approach can vary depending on the operator and technical factors. For example, in individuals with a larger build, skin and fat might be included in the measurement, potentially leading to an overestimation of the gestational age. Despite these limitations, fundal height measurement is useful for monitoring. While ultrasound is more sensitive and widely used in urban areas where literacy levels are higher, in rural areas, we primarily rely on fundal height as our estimation tool, as ultrasounds are not always readily available. | person 1 | 208 If a woman can't recall the date of her last period, we generally use two methods for approximation. One method is ultrasound, where we estimate the age of the pregn/acy based on the size of the fetus or embryo. The other method involves measuring the fundal height, which allows us to approximate the pregn/acy age through certain calculation techniques. However, this approach can vary depending on the operator and technical factors. For example, in individuals with a larger build, skin and fat might be included in the measurement, potentially leading to an overestimation of the gestational age. Despite these limitations, fundal height measurement is useful for monitoring. While ultrasound is more sensitive and widely used in urban areas where literacy levels are higher, in rural areas, we primarily rely on fundal height as our estimation tool, as ultrasounds are not always readily available. |
| CMA Do maternity care unit manager | Measurement of preterm birth | Ways health workers help women remember the date of their last period | person 1 | If she can't recall the date of her last period, we use a couple of methods. First, we perform a physical examination, including measuring the fundal height. This measurement can give us some guidance regarding the gestational age. Additionally, we also rely on ultrasounds. If necessary, we request an ultrasound to help establish a more accurate gestational age. | person 1 | 209 If she can't recall the date of her last period, we use a couple of methods. First, we perform a physical examination, including measuring the fundal height. This measurement can give us some guidance regarding the gestational age. Additionally, we also rely on ultrasounds. If necessary, we request an ultrasound to help establish a more accurate gestational age. |
| Colma1 focus group | Measurement of preterm birth | Ways health workers help women remember the date of their last period | person 3 | In such cases, we primarily use the measurement of the fundal height (HU). By measuring the HU, we can estimate the gestational age. The height of the uterus provides an approximate date of how far along the pregn/acy is. | person 3 | 210 In such cases, we primarily use the measurement of the fundal height (HU). By measuring the HU, we can estimate the gestational age. The height of the uterus provides an approximate date of how far along the pregn/acy is. |
| Colma1 focus group | Measurement of preterm birth | Ways health workers help women remember the date of their last period | person 1 | Yes. | person 1 | 211 Yes. |
| Colma1 focus group | Measurement of preterm birth | Ways health workers help women remember the date of their last period | person 3 | So, our estimation largely depends on that measurement. | person 3 | 212 So, our estimation largely depends on that measurement. |
| Colma1 major | Measurement of preterm birth | Ways health workers help women remember the date of their last period | person 1 | Generally, midwives use the measurement of the fundal height as a basis for estimating gestational age. Additionally, ultrasounds are often requested, as they not only help determine the pregn/acy age but also provide insight into many other aspects. In our urban environment, ultrasounds are geographically accessible. However, the cost can be a barrier for some people. Consequently, they might not undergo the ultrasound and return for the next prenatal consultation (CPN) without having had it, due to the high expense. | person 1 | 213 Generally, midwives use the measurement of the fundal height as a basis for estimating gestational age. Additionally, ultrasounds are often requested, as they not only help determine the pregn/acy age but also provide insight into many other aspects. In our urban environment, ultrasounds are geographically accessible. However, the cost can be a barrier for some people. Consequently, they might not undergo the ultrasound and return for the next prenatal consultation (CPN) without having had it, due to the high expense. |
| Colma1 maternity manager | Measurement of preterm birth | Ways health workers help women remember the date of their last period | person 1 | When she can't recall, we often use the fundal height (HU) measurement to estimate. However, this method isn't always reliable for every woman. For instance, you might measure a fundal height of 16 centimeters, but there are variables like the abdominal wall thickness that can affect the accuracy. In cases like these, it becomes a bit challenging. Ultrasound is the tool that can truly determine the exact age of the pregn/acy. While fundal height can give us a rough idea of gestational age, it lacks precise accuracy. | person 1 | 214 When she can't recall, we often use the fundal height (HU) measurement to estimate. However, this method isn't always reliable for every woman. For instance, you might measure a fundal height of 16 centimeters, but there are variables like the abdominal wall thickness that can affect the accuracy. In cases like these, it becomes a bit challenging. Ultrasound is the tool that can truly determine the exact age of the pregn/acy. While fundal height can give us a rough idea of gestational age, it lacks precise accuracy. |
| Farakan focus group | Measurement of preterm birth | Ways health workers help women remember the date of their last period | person 7 | We often request an ultrasound. | person 7 | 215 We often request an ultrasound. |
| Farakan focus group | Measurement of preterm birth | Ways health workers help women remember the date of their last period | person 3 | I agree. Requesting an ultrasound is our usual approach to determine gestational age in such cases | person 3 | 216 I agree. Requesting an ultrasound is our usual approach to determine gestational age in such cases |
| Farakan focus group | Measurement of preterm birth | Ways health workers help women remember the date of their last period | person 8 | We ask them to undergo an ultrasound, which then helps us in determining the age of the pregn/acy | person 8 | 217 We ask them to undergo an ultrasound, which then helps us in determining the age of the pregn/acy |
| Farakan focus group | Measurement of preterm birth | Ways health workers help women remember the date of their last period | person 5 | While we often ask for an ultrasound, we also rely on measuring the fundal height as a way to estimate the pregn/acy age. | person 5 | 218 While we often ask for an ultrasound, we also rely on measuring the fundal height as a way to estimate the pregn/acy age. |
| Farakan focus group | Measurement of preterm birth | Ways health workers help women remember the date of their last period | person 3 | Our main tool for estimation is the fundal height (HU). | person 3 | 219 Our main tool for estimation is the fundal height (HU). |
| Farakan focus group | Measurement of preterm birth | Ways health workers help women remember the date of their last period | person 4 | The majority of the time, we base our estimates on the fundal height measurement. | person 4 | 220 The majority of the time, we base our estimates on the fundal height measurement. |
| Farakan focus group | Measurement of preterm birth | Ways health workers help women remember the date of their last period | person 2 | HU stands for fundal height. | person 2 | 221 HU stands for fundal height. |
| Farakan focus group | Measurement of preterm birth | Ways health workers help women remember the date of their last period | person 2 | This is the measurement of the height of the uterus. | person 2 | 222 This is the measurement of the height of the uterus. |
| Farakan major | Measurement of preterm birth | Ways health workers help women remember the date of their last period | person 1 | In general, if we're able to request an ultrasound, it can help us determine the gestational age. Alternatively, we start by measuring the fundal height. From this measurement, we can estimate the pregn/acy's duration in months, although it's a rough estimate. | person 1 | 223 In general, if we're able to request an ultrasound, it can help us determine the gestational age. Alternatively, we start by measuring the fundal height. From this measurement, we can estimate the pregn/acy's duration in months, although it's a rough estimate. |
| Farakan maternity manager | Measurement of preterm birth | Ways health workers help women remember the date of their last period | person 1 | The determination is based on the fundal height (HU). We measure the fundal height as an initial assessment. Additionally, we also request an ultrasound, which provides further clarification and helps us ascertain the gestational age more accurately. | person 1 | 224 The determination is based on the fundal height (HU). We measure the fundal height as an initial assessment. Additionally, we also request an ultrasound, which provides further clarification and helps us ascertain the gestational age more accurately. |
| Accart-ville focus group | Measurement of preterm birth | Method of calculating gestational age from fundal height | person 1 | To estimate gestational age from fundal height, we first determine if the pregn/acy is in the second trimester. This is done by asking the woman if she has started feeling fetal movements, as such movements are not typically present in the first trimester. Fetal movements usually begin in the second trimester, which is after the first four months. We might also use an ultrasound for more accuracy. For a rough estimate, when measuring fundal height, we add three (+3) to the measurement to approximate the week of pregn/acy in terms of amenorrhea. | person 1 | 225 To estimate gestational age from fundal height, we first determine if the pregn/acy is in the second trimester. This is done by asking the woman if she has started feeling fetal movements, as such movements are not typically present in the first trimester. Fetal movements usually begin in the second trimester, which is after the first four months. We might also use an ultrasound for more accuracy. For a rough estimate, when measuring fundal height, we add three (+3) to the measurement to approximate the week of pregn/acy in terms of amenorrhea. |
| Accart-ville focus group | Measurement of preterm birth | Method of calculating gestational age from fundal height | person 1 | We don’t have a gestogram here. Since I’ve been here, I haven’t seen one. My colleagues mentioned that it used to be provided by the delegates, but now its availability is uncertain. The effectiveness of a gestogram largely depends on the woman knowing the date of her last menstrual period. Without that information, it’s challenging to use the gestogram effectively for measurements. | person 1 | 226 We don’t have a gestogram here. Since I’ve been here, I haven’t seen one. My colleagues mentioned that it used to be provided by the delegates, but now its availability is uncertain. The effectiveness of a gestogram largely depends on the woman knowing the date of her last menstrual period. Without that information, it’s challenging to use the gestogram effectively for measurements. |
| Accart-ville CMU manager | Measurement of preterm birth | Method of calculating gestational age from fundal height | person 1 | Honestly, I don’t have the specifics memorized, so it’s challenging to answer without having the fundal height chart on hand. It’s something I usually refer to. | person 1 | 227 Honestly, I don’t have the specifics memorized, so it’s challenging to answer without having the fundal height chart on hand. It’s something I usually refer to. |
| Accart-ville CMU manager | Measurement of preterm birth | Method of calculating gestational age from fundal height | person 1 | Yes, that's correct. I use a chart for guidance. | person 1 | 228 Yes, that's correct. I use a chart for guidance. |
| Accart-ville maternity manager | Measurement of preterm birth | Method of calculating gestational age from fundal height | person 1 | We base our calculations on fundal height measurements. Specifically, we add 4 cm to the measurement until the 7th month of pregn/acy. After the 7th month, we add 3 cm, and after the 8th month, we add 2 cm. For example, a fundal height (HU) of 20 cm, plus 4 cm, gives an estimated 24 weeks of amenorrhea. | person 1 | 229 We base our calculations on fundal height measurements. Specifically, we add 4 cm to the measurement until the 7th month of pregn/acy. After the 7th month, we add 3 cm, and after the 8th month, we add 2 cm. For example, a fundal height (HU) of 20 cm, plus 4 cm, gives an estimated 24 weeks of amenorrhea. |
| Accart-ville maternity manager | Measurement of preterm birth | Method of calculating gestational age from fundal height | person 1 | Yes, we do mental calculations, but we also sometimes use gestograms. A gestogram is particularly useful when the woman knows the date of her last period. If she doesn’t, the gestogram we have can't be used effectively. | person 1 | 230 Yes, we do mental calculations, but we also sometimes use gestograms. A gestogram is particularly useful when the woman knows the date of her last period. If she doesn’t, the gestogram we have can't be used effectively. |
| Accart-ville maternity manager | Measurement of preterm birth | Method of calculating gestational age from fundal height | person 1 | We often rely on the date of the last known period for calculations. If this date is unknown, we start from a clinical perspective. | person 1 | 231 We often rely on the date of the last known period for calculations. If this date is unknown, we start from a clinical perspective. |
| CMA Do focus group | Measurement of preterm birth | Method of calculating gestational age from fundal height | person 2 | From the 4th or 7th month onwards, we add four centimeters to the fundal height measurement each time | person 2 | 232 From the 4th or 7th month onwards, we add four centimeters to the fundal height measurement each time |
| CMA Do focus group | Measurement of preterm birth | Method of calculating gestational age from fundal height | person 2 | We utilize a gestogram for these calculations. | person 2 | 233 We utilize a gestogram for these calculations. |
| Gynecologist 1 | Measurement of preterm birth | Method of calculating gestational age from fundal height | person 1 | To determine the pregn/acy age, we measure the fundal height and then divide that number by 4. | person 1 | 234 To determine the pregn/acy age, we measure the fundal height and then divide that number by 4. |
| Gynecologist 1 | Measurement of preterm birth | Method of calculating gestational age from fundal height | person 1 | We rely on tables specifically designed for this purpose. The gestogram is used only when the woman knows the date of her last menstrual period. However, when using fundal height, we refer to tables that correlate this measurement with the gestational age. | person 1 | 235 We rely on tables specifically designed for this purpose. The gestogram is used only when the woman knows the date of her last menstrual period. However, when using fundal height, we refer to tables that correlate this measurement with the gestational age. |
| Gynecologist 2 | Measurement of preterm birth | Method of calculating gestational age from fundal height | person 1 | We measure the fundal height in centimeters. In the first trimester, it's typically between 12 to 15 centimeters, indicating about 3 to 4 months of pregn/acy. Around 20 centimeters correlates with approximately 5 months, and 24 centimeters with around 6 months. Towards the end of pregn/acy, the fundal height reaches between 34 and 36 centimeters, indicating the 9th month. The height may decrease slightly in the last month | person 1 | 236 We measure the fundal height in centimeters. In the first trimester, it's typically between 12 to 15 centimeters, indicating about 3 to 4 months of pregn/acy. Around 20 centimeters correlates with approximately 5 months, and 24 centimeters with around 6 months. Towards the end of pregn/acy, the fundal height reaches between 34 and 36 centimeters, indicating the 9th month. The height may decrease slightly in the last month |
| Gynecologist 2 | Measurement of preterm birth | Method of calculating gestational age from fundal height | person 1 | Gestograms are useful when we know the date of the last menstrual period. It helps us calculate the current gestational age and predict the due date. If the date of the last period is unknown, the gestogram isn't as useful | person 1 | 237 Gestograms are useful when we know the date of the last menstrual period. It helps us calculate the current gestational age and predict the due date. If the date of the last period is unknown, the gestogram isn't as useful |
| MCD | Measurement of preterm birth | Method of calculating gestational age from fundal height | person 1 | After the first trimester, we use a tape measure to determine fundal height. We measure from the symphysis pubis to the fundus of the uterus vertically. The tape measure is flexible, which helps in following the curvature of the abdomen. This method is typically used from the fourth month of pregn/acy until the end. The height measured in centimeters, when divided by 4, gives an approximate number of months of pregn/acy. | person 1 | 238 After the first trimester, we use a tape measure to determine fundal height. We measure from the symphysis pubis to the fundus of the uterus vertically. The tape measure is flexible, which helps in following the curvature of the abdomen. This method is typically used from the fourth month of pregn/acy until the end. The height measured in centimeters, when divided by 4, gives an approximate number of months of pregn/acy. |
| MCD | Measurement of preterm birth | Method of calculating gestational age from fundal height | person 1 | We use a standard tape measure for this purpose. Measuring fundal height is straightforward. After measuring, I divide the height by 4 to estimate the gestational age in months | person 1 | 239 We use a standard tape measure for this purpose. Measuring fundal height is straightforward. After measuring, I divide the height by 4 to estimate the gestational age in months |
| CMA Do maternity care unit manager | Measurement of preterm birth | Method of calculating gestational age from fundal height | person 1 | Well, regarding fundal height... When we measure the fundal height, we use a specific scale. For instance, if I observe a globular uterus, I start there. We don't measure it in terms of size like an orange, lemon, or grapefruit. When I determine a certain size, like that of an orange, it suggests to me that I might be dealing with a pregn/acy around 12 weeks. Similarly, if I measure a uterine height of 16 or 20 centimeters, I correlate it to a pregn/acy of 16 or 20 weeks, respectively. However, we always confirm these assessments with an ultrasound, since factors like twin pregn/acies or the fetus's size can affect the uterine height. The ultrasound helps to confirm the pregn/acy's progression and gestational age | person 1 | 240 Well, regarding fundal height... When we measure the fundal height, we use a specific scale. For instance, if I observe a globular uterus, I start there. We don't measure it in terms of size like an orange, lemon, or grapefruit. When I determine a certain size, like that of an orange, it suggests to me that I might be dealing with a pregn/acy around 12 weeks. Similarly, if I measure a uterine height of 16 or 20 centimeters, I correlate it to a pregn/acy of 16 or 20 weeks, respectively. However, we always confirm these assessments with an ultrasound, since factors like twin pregn/acies or the fetus's size can affect the uterine height. The ultrasound helps to confirm the pregn/acy's progression and gestational age |
| CMA Do maternity care unit manager | Measurement of preterm birth | Method of calculating gestational age from fundal height | person 1 | Yes, there's a gestogram. If we know the date of the last menstrual period, we use the gestogram to determine the probable delivery date. It even helps us estimate the conception period | person 1 | 241 Yes, there's a gestogram. If we know the date of the last menstrual period, we use the gestogram to determine the probable delivery date. It even helps us estimate the conception period |
| Colma1 focus group | Measurement of preterm birth | Method of calculating gestational age from fundal height | person 2 | The fundal height (HU) measurements are estimated as follows: 4th month: 16 cm ; 5th month: 20 cm ; 6th month: 24 cm ; 7th month: 28 cm ; 8th month: Around 30 cm ; 9th month: 30 to 32 cm or more. These measurements are used to estimate gestational age in months based on the fundal height | person 2 | 242 The fundal height (HU) measurements are estimated as follows: 4th month: 16 cm ; 5th month: 20 cm ; 6th month: 24 cm ; 7th month: 28 cm ; 8th month: Around 30 cm ; 9th month: 30 to 32 cm or more. These measurements are used to estimate gestational age in months based on the fundal height |
| Colma1 major | Measurement of preterm birth | Method of calculating gestational age from fundal height | person 1 | Fundal height measurements vary with gestational age. For instance: 12 cm indicates the first trimester (up to 3 months); 16 cm suggests the fourth month; 20 cm corresponds to the fifth month; 24 cm is typical of the sixth month ; 27 or 28 cm indicates the seventh month. | person 1 | 243 Fundal height measurements vary with gestational age. For instance: 12 cm indicates the first trimester (up to 3 months); 16 cm suggests the fourth month; 20 cm corresponds to the fifth month; 24 cm is typical of the sixth month ; 27 or 28 cm indicates the seventh month. |
| Colma1 major | Measurement of preterm birth | Method of calculating gestational age from fundal height | person 1 | Yes, there's a chart in the CPN (Prenatal Care) room that midwives and birth attendants use as a guide. It's displayed for reference. | person 1 | 244 Yes, there's a chart in the CPN (Prenatal Care) room that midwives and birth attendants use as a guide. It's displayed for reference. |
| Colma1 maternity manager | Measurement of preterm birth | Method of calculating gestational age from fundal height | person 1 | The calculation is based on fundal height (HU). For example, if a woman has a fundal height of 16 centimeters, we refer to a pre-established table to equate this to the number of months of pregn/acy. Generally, the age is multiplied by four, but after seven months of pregn/acy, the calculation changes: we subtract two from the result. If a woman says her last period was in January, we can estimate her due month accordingly. Currently, under the DenBalo project, many women benefit from ultrasound services, which significantly aid in determining pregn/acy age, especially compared to when only targeted women had access to this facility | person 1 | 245 The calculation is based on fundal height (HU). For example, if a woman has a fundal height of 16 centimeters, we refer to a pre-established table to equate this to the number of months of pregn/acy. Generally, the age is multiplied by four, but after seven months of pregn/acy, the calculation changes: we subtract two from the result. If a woman says her last period was in January, we can estimate her due month accordingly. Currently, under the DenBalo project, many women benefit from ultrasound services, which significantly aid in determining pregn/acy age, especially compared to when only targeted women had access to this facility |
| Farakan focus group | Measurement of preterm birth | Method of calculating gestational age from fundal height | person 4 | The calculation is based on fundal height (HU). For example, if the fundal height is 12 cm, it suggests that the pregn/acy is not more than 3 months. Similarly, a fundal height of 16 cm usually indicates that the pregn/acy is no more than 4 months. | person 4 | 246 The calculation is based on fundal height (HU). For example, if the fundal height is 12 cm, it suggests that the pregn/acy is not more than 3 months. Similarly, a fundal height of 16 cm usually indicates that the pregn/acy is no more than 4 months. |
| Farakan focus group | Measurement of preterm birth | Method of calculating gestational age from fundal height | person 3 | In the first trimester, we measure the fundal height and calculate the pregn/acy age by adding 4 cm for each month. From the second trimester, we add 5 cm per month, and in the third trimester, the increment changes to 4 cm and then 3 cm. | person 3 | 247 In the first trimester, we measure the fundal height and calculate the pregn/acy age by adding 4 cm for each month. From the second trimester, we add 5 cm per month, and in the third trimester, the increment changes to 4 cm and then 3 cm. |
| Farakan major | Measurement of preterm birth | Method of calculating gestational age from fundal height | person 1 | Gestational age is estimated based on the number of centimeters of fundal height, with a specific age assigned to each measurement. However, these parameters can be biased. For instance, larger women with more abdominal wall can have distorted measurements, making the estimation approximate | person 1 | 248 Gestational age is estimated based on the number of centimeters of fundal height, with a specific age assigned to each measurement. However, these parameters can be biased. For instance, larger women with more abdominal wall can have distorted measurements, making the estimation approximate |
| Farakan major | Measurement of preterm birth | Method of calculating gestational age from fundal height | person 1 | Yes, gestograms are also available. They are used to assist in calculating gestational age. | person 1 | 249 Yes, gestograms are also available. They are used to assist in calculating gestational age. |
| Farakan maternity manager | Measurement of preterm birth | Method of calculating gestational age from fundal height | person 1 | The gestational age is approximated based on fundal height measurements. For instance, a measurement of 12 cm is typically associated with a pregn/acy of 3 months, indicating the first trimester | person 1 | 250 The gestational age is approximated based on fundal height measurements. For instance, a measurement of 12 cm is typically associated with a pregn/acy of 3 months, indicating the first trimester |
| Farakan maternity manager | Measurement of preterm birth | Method of calculating gestational age from fundal height | person 1 | The gestational age calculation using fundal height is generally based on the date of the last period. If the date is known, a gestogram can be used for more accurate calculations. Without this date, the calculation is more approximate, with specific centimeter measurements (like 12 cm, 16 cm, etc.) being equated to certain months of pregn/acy. | person 1 | 251 The gestational age calculation using fundal height is generally based on the date of the last period. If the date is known, a gestogram can be used for more accurate calculations. Without this date, the calculation is more approximate, with specific centimeter measurements (like 12 cm, 16 cm, etc.) being equated to certain months of pregn/acy. |
| Accart-ville focus group | Measurement of preterm birth | Materials needed for calculating gestational age | person 1 | Necessary equipment? Yes, we do have a tape measure, which we use for fundal height measurement. Apart from that, we rely on ultrasound for more detailed information. | person 1 | 252 Necessary equipment? Yes, we do have a tape measure, which we use for fundal height measurement. Apart from that, we rely on ultrasound for more detailed information. |
| Accart-ville CMU manager | Measurement of preterm birth | Materials needed for calculating gestational age | person 1 | Yes, we use applications on our phones for this purpose. I personally rely on an app that I have on my phone to calculate gestational age | person 1 | 253 Yes, we use applications on our phones for this purpose. I personally rely on an app that I have on my phone to calculate gestational age |
| Accart-ville CMU manager | Measurement of preterm birth | Materials needed for calculating gestational age | person 1 | I mostly use an app called Medicalcul | person 1 | 254 I mostly use an app called Medicalcul |
| Accart-ville maternity manager | Measurement of preterm birth | Materials needed for calculating gestational age | person 1 | As I mentioned earlier, our primary tools for calculating gestational age are the gestogram and the tape measure. These are the only means we have at our disposal. We rely on both the gestogram and the tape measure for this purpose | person 1 | 255 As I mentioned earlier, our primary tools for calculating gestational age are the gestogram and the tape measure. These are the only means we have at our disposal. We rely on both the gestogram and the tape measure for this purpose |
| CMA Do focus group | Measurement of preterm birth | Materials needed for calculating gestational age | person 6 | Yes, we have an ultrasound machine here, which is the necessary equipment for that purpose. | person 6 | 256 Yes, we have an ultrasound machine here, which is the necessary equipment for that purpose. |
| CMA Do focus group | Measurement of preterm birth | Materials needed for calculating gestational age | person 4 | However, it's not commonly used by us, the midwives. We don't use ultrasound systematically. It's typically employed in specific situations where we need to consult with a gynecologist to determine the gestational age. In our regular encounters with women, we use different methods. If they know the date of their last period, we use that. If not, we try to link it to an event, like Tabaski, for an approximate date. But even then, we're not always certain of the accuracy. | person 4 | 257 However, it's not commonly used by us, the midwives. We don't use ultrasound systematically. It's typically employed in specific situations where we need to consult with a gynecologist to determine the gestational age. In our regular encounters with women, we use different methods. If they know the date of their last period, we use that. If not, we try to link it to an event, like Tabaski, for an approximate date. But even then, we're not always certain of the accuracy. |
| Gynecologist 1 | Measurement of preterm birth | Materials needed for calculating gestational age | person 1 | Yes, we have the necessary materials. | person 1 | 258 Yes, we have the necessary materials. |
| Gynecologist 1 | Measurement of preterm birth | Materials needed for calculating gestational age | person 1 | We don't need much for this purpose. We have an ultrasound machine that can provide us with the gestational age. Additionally, as I mentioned earlier, we use a table based on uterine height for estimation. However, there's a higher risk of error with this method. Factors like insufficient amniotic fluid or developmental issues in the fetus that result in smaller size can lead to inaccuracies. But generally, we manage with the tools we have. | person 1 | 259 We don't need much for this purpose. We have an ultrasound machine that can provide us with the gestational age. Additionally, as I mentioned earlier, we use a table based on uterine height for estimation. However, there's a higher risk of error with this method. Factors like insufficient amniotic fluid or developmental issues in the fetus that result in smaller size can lead to inaccuracies. But generally, we manage with the tools we have. |
| Gynecologist 2 | Measurement of preterm birth | Materials needed for calculating gestational age | person 1 | Yes, the primary tool we need is a tape measure for calculating the fundal height. We measure the fundal height and then use it to estimate roughly how many months the pregn/acy has progressed. Additionally, we have access to tables that correlate fundal height with gestational age. These tables allow us to convert the fundal height measurement directly into an estimated gestational age, which is quite helpful as we don’t need to remember all the conversion details. | person 1 | 260 Yes, the primary tool we need is a tape measure for calculating the fundal height. We measure the fundal height and then use it to estimate roughly how many months the pregn/acy has progressed. Additionally, we have access to tables that correlate fundal height with gestational age. These tables allow us to convert the fundal height measurement directly into an estimated gestational age, which is quite helpful as we don’t need to remember all the conversion details. |
| MCD | Measurement of preterm birth | Materials needed for calculating gestational age | person 1 | The tool we use here is a tailor’s tape measure. For fundal height, it's simple: once I measure, I know how to divide it and then I can estimate the pregn/acy duration. | person 1 | 261 The tool we use here is a tailor’s tape measure. For fundal height, it's simple: once I measure, I know how to divide it and then I can estimate the pregn/acy duration. |
| CMA Do maternity care unit manager | Measurement of preterm birth | Materials needed for calculating gestational age | person 1 | Yes, we have a gestogram available. That's one tool we use. Additionally, we utilize a tape measure to take uterine height measurements. We also have access to ultrasound, although it's not located within the same room. It's typically used in gynecological consultations for confirmation purposes. | person 1 | 262 Yes, we have a gestogram available. That's one tool we use. Additionally, we utilize a tape measure to take uterine height measurements. We also have access to ultrasound, although it's not located within the same room. It's typically used in gynecological consultations for confirmation purposes. |
| Colma1 focus group | Measurement of preterm birth | Materials needed for calculating gestational age | person 2 | We don't have a gestogram. We use a tape measure, but the effectiveness of this method depends on the woman being able to provide at least a month of her last period. Most of the time, however, she doesn’t even know the month. | person 2 | 263 We don't have a gestogram. We use a tape measure, but the effectiveness of this method depends on the woman being able to provide at least a month of her last period. Most of the time, however, she doesn’t even know the month. |
| Colma1 focus group | Measurement of preterm birth | Materials needed for calculating gestational age | person 1 | As he mentioned, we rely on measuring the fundal height (HU). | person 1 | 264 As he mentioned, we rely on measuring the fundal height (HU). |
| Colma1 focus group | Measurement of preterm birth | Materials needed for calculating gestational age | person 3 | Yes, generally it depends on the HU measurement. | person 3 | 265 Yes, generally it depends on the HU measurement. |
| Colma1 major | Measurement of preterm birth | Materials needed for calculating gestational age | person 1 | Regarding the necessary materials, aside from the poster we've created that outlines the procedure I just described, we don't have a gestogram. That's the extent of our resources for calculating gestational age. | person 1 | 266 Regarding the necessary materials, aside from the poster we've created that outlines the procedure I just described, we don't have a gestogram. That's the extent of our resources for calculating gestational age. |
| Colma1 maternity manager | Measurement of preterm birth | Materials needed for calculating gestational age | person 1 | When the date of the last menstrual period is known, we use calculation methods we all learned in school. For instance, if a woman says she had her period on August 14th, we add 10 days to the 14th and then add three months to August to estimate the year of delivery. This calculation gives us the probable date of delivery, plus or minus two weeks, considering the 10-day variation. However, when the woman doesn't know the exact date and can only specify the month, like February, we estimate the delivery around November. We try our best to determine the date, but the precision depends on the information provided. If we can, we recommend an ultrasound for more accurate confirmation of pregn/acy or to assess the fetus's development. Unfortunately, some women are unable to get an ultrasound until very late in their pregn/acy, leading to unexpected cases at times. | person 1 | 267 When the date of the last menstrual period is known, we use calculation methods we all learned in school. For instance, if a woman says she had her period on August 14th, we add 10 days to the 14th and then add three months to August to estimate the year of delivery. This calculation gives us the probable date of delivery, plus or minus two weeks, considering the 10-day variation. However, when the woman doesn't know the exact date and can only specify the month, like February, we estimate the delivery around November. We try our best to determine the date, but the precision depends on the information provided. If we can, we recommend an ultrasound for more accurate confirmation of pregn/acy or to assess the fetus's development. Unfortunately, some women are unable to get an ultrasound until very late in their pregn/acy, leading to unexpected cases at times. |
| Farakan focus group | Measurement of preterm birth | Materials needed for calculating gestational age | person 4 | Yes, we have a gestogram, though it's often underused. Many of us carry one in our bags. Additionally, we rely heavily on measuring fundal height (HU), especially in urban settings where ultrasounds are frequently used. We tend to overuse them, requesting them in the first, second, and third trimesters. | person 4 | 268 Yes, we have a gestogram, though it's often underused. Many of us carry one in our bags. Additionally, we rely heavily on measuring fundal height (HU), especially in urban settings where ultrasounds are frequently used. We tend to overuse them, requesting them in the first, second, and third trimesters. |
| Farakan major | Measurement of preterm birth | Materials needed for calculating gestational age | person 1 | Yes, we use the gestogram for this purpose. | person 1 | 269 Yes, we use the gestogram for this purpose. |
| Farakan maternity manager | Measurement of preterm birth | Materials needed for calculating gestational age | person 1 | Yes, I have the essential tools. Specifically, I have a gestogram in my office. However, the calculations depend on the information provided by the patient. For instance, if a woman informs me that she last had her period on December 12th, my approach would be to subtract three months from this date and then add ten days to estimate the gestational age. | person 1 | 270 Yes, I have the essential tools. Specifically, I have a gestogram in my office. However, the calculations depend on the information provided by the patient. For instance, if a woman informs me that she last had her period on December 12th, my approach would be to subtract three months from this date and then add ten days to estimate the gestational age. |
| Accart-ville focus group | Measurement of preterm birth | Priority between different methods of measuring gestational age | person 1 | In terms of priority, we focus on physical measurements. We measure the relevant parameters and add any necessary adjustments to approximate the gestational age. | person 1 | 271 In terms of priority, we focus on physical measurements. We measure the relevant parameters and add any necessary adjustments to approximate the gestational age. |
| Accart-ville CMU manager | Measurement of preterm birth | Priority between different methods of measuring gestational age | person 1 | Generally, we prioritize the age determined by ultrasound, as it's often challenging to ascertain the exact chronological age. So, we rely more on the ultrasound age when a woman has one. | person 1 | 272 Generally, we prioritize the age determined by ultrasound, as it's often challenging to ascertain the exact chronological age. So, we rely more on the ultrasound age when a woman has one. |
| Accart-ville maternity manager | Measurement of preterm birth | Priority between different methods of measuring gestational age | person 1 | We prioritize the date of the last menstrual period (DDR). | person 1 | 273 We prioritize the date of the last menstrual period (DDR). |
| Accart-ville maternity manager | Measurement of preterm birth | Priority between different methods of measuring gestational age | person 1 | Yes. | person 1 | 274 Yes. |
| Accart-ville maternity manager | Measurement of preterm birth | Priority between different methods of measuring gestational age | person 1 | We rely on the chronological age, considering the margins of error in other dating methods. For instance, fundal height (HU) measurements can be influenced by the woman's body size. In obese women, even locating the uterus can be challenging. Additionally, if an ultrasound isn't performed early, before the fifteenth week, it becomes difficult to accurately determine the last period date. Therefore, we prioritize the chronological age based on the last menstrual period in our assessments. | person 1 | 275 We rely on the chronological age, considering the margins of error in other dating methods. For instance, fundal height (HU) measurements can be influenced by the woman's body size. In obese women, even locating the uterus can be challenging. Additionally, if an ultrasound isn't performed early, before the fifteenth week, it becomes difficult to accurately determine the last period date. Therefore, we prioritize the chronological age based on the last menstrual period in our assessments. |
| CMA Do focus group | Measurement of preterm birth | Priority between different methods of measuring gestational age | person 7 | The gestogram. | person 7 | 276 The gestogram. |
| CMA Do focus group | Measurement of preterm birth | Priority between different methods of measuring gestational age | person 7 | Because it's readily accessible to us. We have it at our fingertips. | person 7 | 277 Because it's readily accessible to us. We have it at our fingertips. |
| Gynecologist 1 | Measurement of preterm birth | Priority between different methods of measuring gestational age | person 1 | It's the ultrasound. Definitely, the ultrasound. | person 1 | 278 It's the ultrasound. Definitely, the ultrasound. |
| Gynecologist 1 | Measurement of preterm birth | Priority between different methods of measuring gestational age | person 1 | The ultrasound offers a more precise gestational age due to its various measurements. Fundal height can be influenced by many factors, like the amount of amniotic fluid or potential issues with the fetus. In contrast, an ultrasound provides a gestational age with a relatively small margin of error, maybe one week to ten days. | person 1 | 279 The ultrasound offers a more precise gestational age due to its various measurements. Fundal height can be influenced by many factors, like the amount of amniotic fluid or potential issues with the fetus. In contrast, an ultrasound provides a gestational age with a relatively small margin of error, maybe one week to ten days. |
| Gynecologist 2 | Measurement of preterm birth | Priority between different methods of measuring gestational age | person 1 | You mean, which method provides the most accurate information? | person 1 | 280 You mean, which method provides the most accurate information? |
| Gynecologist 2 | Measurement of preterm birth | Priority between different methods of measuring gestational age | person 1 | In our practice, we typically use a combination of methods. We measure fundal height, but often, especially in urban areas, we recommend an ultrasound. If a woman in her first trimester doesn’t remember the date of her last period, we specifically request a dating ultrasound. That’s our most commonly used approach. | person 1 | 281 In our practice, we typically use a combination of methods. We measure fundal height, but often, especially in urban areas, we recommend an ultrasound. If a woman in her first trimester doesn’t remember the date of her last period, we specifically request a dating ultrasound. That’s our most commonly used approach. |
| MCD | Measurement of preterm birth | Priority between different methods of measuring gestational age | person 1 | At the CMA, we first ask if the woman remembers her last menstrual period. Knowing this date simplifies things. We use the gestogram, which calculates the weeks of amenorrhea, adding two weeks for the maturation phase. If the last period date is unknown, we then measure fundal height. The number of centimeters helps us estimate the gestational age. Ultrasounds are also used, but the most common methods here are the gestogram and fundal height measurement. In rural areas, we mainly rely on fundal height due to the lack of ultrasound facilities. | person 1 | 282 At the CMA, we first ask if the woman remembers her last menstrual period. Knowing this date simplifies things. We use the gestogram, which calculates the weeks of amenorrhea, adding two weeks for the maturation phase. If the last period date is unknown, we then measure fundal height. The number of centimeters helps us estimate the gestational age. Ultrasounds are also used, but the most common methods here are the gestogram and fundal height measurement. In rural areas, we mainly rely on fundal height due to the lack of ultrasound facilities. |
| CMA Do maternity care unit manager | Measurement of preterm birth | Priority between different methods of measuring gestational age | person 1 | For me, the primary method is using the gestogram. I make every effort to have the woman provide the date of her last menstrual period. With that information, I can effectively use the gestogram to estimate gestational age. | person 1 | 283 For me, the primary method is using the gestogram. I make every effort to have the woman provide the date of her last menstrual period. With that information, I can effectively use the gestogram to estimate gestational age. |
| CMA Do maternity care unit manager | Measurement of preterm birth | Priority between different methods of measuring gestational age | person 1 | It's because I already have the date of the last period, which is crucial. The challenge is in gathering this date. Often, people don't take the time to encourage women to recall and provide this period. If a woman says she doesn't know, many quickly move on to labeling it as unknown. But if you persist a bit, she can usually provide the date, which is very helpful. However, generally, people here rely more on ultrasound for estimation. | person 1 | 284 It's because I already have the date of the last period, which is crucial. The challenge is in gathering this date. Often, people don't take the time to encourage women to recall and provide this period. If a woman says she doesn't know, many quickly move on to labeling it as unknown. But if you persist a bit, she can usually provide the date, which is very helpful. However, generally, people here rely more on ultrasound for estimation. |
| Colma1 focus group | Measurement of preterm birth | Priority between different methods of measuring gestational age | person 1 | Our priority is to base the estimate on the date of the last menstrual period. If the woman cannot provide the date of her last period, we then turn to using the fundal height (HU) measurement. | person 1 | 285 Our priority is to base the estimate on the date of the last menstrual period. If the woman cannot provide the date of her last period, we then turn to using the fundal height (HU) measurement. |
| Colma1 major | Measurement of preterm birth | Priority between different methods of measuring gestational age | person 1 | For the most accurate determination of pregn/acy age, we rely on ultrasound. It's the recommended method. While some women get it done, others don't. Relying solely on standard methods or charts displayed in the rooms can sometimes lead to errors. | person 1 | 286 For the most accurate determination of pregn/acy age, we rely on ultrasound. It's the recommended method. While some women get it done, others don't. Relying solely on standard methods or charts displayed in the rooms can sometimes lead to errors. |
| Colma1 maternity manager | Measurement of preterm birth | Priority between different methods of measuring gestational age | person 1 | The preferred method? Ideally, it's when the woman knows the date of her last menstrual period. That's the best scenario for us. Once we have that date, our calculation method, whether we add 14 days, 10 days, or 7 days, becomes very effective. Knowing the date of the last menstrual period is crucial for accurately determining the gestational age of the pregn/acy. | person 1 | 287 The preferred method? Ideally, it's when the woman knows the date of her last menstrual period. That's the best scenario for us. Once we have that date, our calculation method, whether we add 14 days, 10 days, or 7 days, becomes very effective. Knowing the date of the last menstrual period is crucial for accurately determining the gestational age of the pregn/acy. |
| Farakan focus group | Measurement of preterm birth | Priority between different methods of measuring gestational age | person 7 | Usually, we rely more on the date of the last menstrual period (LMP), if the woman remembers it. However, ultrasound estimates can vary by about two weeks. | person 7 | 288 Usually, we rely more on the date of the last menstrual period (LMP), if the woman remembers it. However, ultrasound estimates can vary by about two weeks. |
| Farakan focus group | Measurement of preterm birth | Priority between different methods of measuring gestational age | person 4 | I prefer using the gestogram, even if the woman doesn't know her LMP date. I find it helpful | person 4 | 289 I prefer using the gestogram, even if the woman doesn't know her LMP date. I find it helpful |
| Farakan focus group | Measurement of preterm birth | Priority between different methods of measuring gestational age | person 3 | The LMP date is key, but since it's often unknown, we mainly use fundal height (HU) measurements. | person 3 | 290 The LMP date is key, but since it's often unknown, we mainly use fundal height (HU) measurements. |
| Farakan focus group | Measurement of preterm birth | Priority between different methods of measuring gestational age | person 5 | (laughing) As mentioned, the LMP date is important, but when unknown, we rely on fundal height to estimate pregn/acy age | person 5 | 291 (laughing) As mentioned, the LMP date is important, but when unknown, we rely on fundal height to estimate pregn/acy age |
| Farakan focus group | Measurement of preterm birth | Priority between different methods of measuring gestational age | person 6 | Agreeing with others, besides the LMP date, we depend on fundal height measurements. | person 6 | 292 Agreeing with others, besides the LMP date, we depend on fundal height measurements. |
| Farakan major | Measurement of preterm birth | Priority between different methods of measuring gestational age | person 1 | Here, we frequently use fundal height measurements. | person 1 | 293 Here, we frequently use fundal height measurements. |
| Farakan major | Measurement of preterm birth | Priority between different methods of measuring gestational age | person 1 | We also use gestograms, but being in an urban area, we often recommend ultrasounds for those who can afford them. Ultrasounds are preferred because they are more accurate. | person 1 | 294 We also use gestograms, but being in an urban area, we often recommend ultrasounds for those who can afford them. Ultrasounds are preferred because they are more accurate. |
| Farakan maternity manager | Measurement of preterm birth | Priority between different methods of measuring gestational age | person 1 | The primary estimate we rely on is the ultrasound, but the date of the last menstrual period is also important. If the date of the last period is unknown, we have to depend on the ultrasound results. However, if she knows the date of her last period, we can calculate the probable date of delivery using both the last period date and ultrasound results. Both methods are frequently used. | person 1 | 295 The primary estimate we rely on is the ultrasound, but the date of the last menstrual period is also important. If the date of the last period is unknown, we have to depend on the ultrasound results. However, if she knows the date of her last period, we can calculate the probable date of delivery using both the last period date and ultrasound results. Both methods are frequently used. |
| Accart-ville focus group | Measurement of preterm birth | Appointment for prenatal consultations with ultrasound measurement carried out | person 1 | Women who experience issues like stomach pains before starting their antenatal care (ANC) often consult us not for ANC but due to these ailments. We request an ultrasound for them. Then, when they attend their first prenatal visit (CPN), they typically bring this ultrasound with them | person 1 | 296 Women who experience issues like stomach pains before starting their antenatal care (ANC) often consult us not for ANC but due to these ailments. We request an ultrasound for them. Then, when they attend their first prenatal visit (CPN), they typically bring this ultrasound with them |
| Accart-ville focus group | Measurement of preterm birth | Appointment for prenatal consultations with ultrasound measurement carried out | person 1 | If the ultrasound is recent, we prioritize it and compare its findings with our measurements. If the ultrasound was done a while ago, we use our measurement methods and, if there are doubts, we may send her for another ultrasound | person 1 | 297 If the ultrasound is recent, we prioritize it and compare its findings with our measurements. If the ultrasound was done a while ago, we use our measurement methods and, if there are doubts, we may send her for another ultrasound |
| Accart-ville focus group | Measurement of preterm birth | Appointment for prenatal consultations with ultrasound measurement carried out | person 2 | Initially, we start with clinical assessments. If these are inconclusive, we then prioritize ultrasound. | person 2 | 298 Initially, we start with clinical assessments. If these are inconclusive, we then prioritize ultrasound. |
| Accart-ville focus group | Measurement of preterm birth | Appointment for prenatal consultations with ultrasound measurement carried out | person 5 | Some women come with an ultrasound, but they are a minority. | person 5 | 299 Some women come with an ultrasound, but they are a minority. |
| Accart-ville focus group | Measurement of preterm birth | Appointment for prenatal consultations with ultrasound measurement carried out | person 6 | Women who first visited us for health issues like persistent pain or bleeding, and were asked to get an ultrasound, often bring it to their prenatal visit. | person 6 | 300 Women who first visited us for health issues like persistent pain or bleeding, and were asked to get an ultrasound, often bring it to their prenatal visit. |
| Accart-ville focus group | Measurement of preterm birth | Appointment for prenatal consultations with ultrasound measurement carried out | person 1 | It depends on the age of the ultrasound at the time of her first prenatal visit. | person 1 | 301 It depends on the age of the ultrasound at the time of her first prenatal visit. |
| Accart-ville focus group | Measurement of preterm birth | Appointment for prenatal consultations with ultrasound measurement carried out | person 2 | We also make comparisons. If necessary, we request the date of the last menstrual period for additional comparison. Ultrasounds have a margin of error, so the chronological age from the last period can be more reliable. If there's a discrepancy between the clinical observation and the ultrasound, we may ask for the LMP date. | person 2 | 302 We also make comparisons. If necessary, we request the date of the last menstrual period for additional comparison. Ultrasounds have a margin of error, so the chronological age from the last period can be more reliable. If there's a discrepancy between the clinical observation and the ultrasound, we may ask for the LMP date. |
| Accart-ville CMU manager | Measurement of preterm birth | Appointment for prenatal consultations with ultrasound measurement carried out | person 1 | Yes, they do. | person 1 | 303 Yes, they do. |
| Accart-ville CMU manager | Measurement of preterm birth | Appointment for prenatal consultations with ultrasound measurement carried out | person 1 | Yes, that's our standard practice. | person 1 | 304 Yes, that's our standard practice. |
| Accart-ville maternity manager | Measurement of preterm birth | Appointment for prenatal consultations with ultrasound measurement carried out | person 1 | It's actually rare for women to come in with an ultrasound already done. Usually, it's only those who have encountered health issues early in pregn/acy, like pelvic pain or bleeding. These women might consult for these issues, and we ask them to get an ultrasound. When they begin their antenatal care (ANC) or prenatal visits, they bring this ultrasound with them. However, for those who haven’t had early pregn/acy problems, we typically request an ultrasound here at our center. | person 1 | 305 It's actually rare for women to come in with an ultrasound already done. Usually, it's only those who have encountered health issues early in pregn/acy, like pelvic pain or bleeding. These women might consult for these issues, and we ask them to get an ultrasound. When they begin their antenatal care (ANC) or prenatal visits, they bring this ultrasound with them. However, for those who haven’t had early pregn/acy problems, we typically request an ultrasound here at our center. |
| Accart-ville maternity manager | Measurement of preterm birth | Appointment for prenatal consultations with ultrasound measurement carried out | person 1 | We start with the date of the last menstrual period, if she knows it. We usually believe that clinical evaluation, including patient interviews and physical examinations, are more reliable than just relying on ultrasound. If we are convinced of the accuracy based on these clinical methods, we prioritize them over ultrasound. Ultrasound is used primarily when other means don’t provide a clear dating of the pregn/acy. | person 1 | 306 We start with the date of the last menstrual period, if she knows it. We usually believe that clinical evaluation, including patient interviews and physical examinations, are more reliable than just relying on ultrasound. If we are convinced of the accuracy based on these clinical methods, we prioritize them over ultrasound. Ultrasound is used primarily when other means don’t provide a clear dating of the pregn/acy. |
| CMA Do focus group | Measurement of preterm birth | Appointment for prenatal consultations with ultrasound measurement carried out | person 8 | Yes. | person 8 | 307 Yes. |
| CMA Do focus group | Measurement of preterm birth | Appointment for prenatal consultations with ultrasound measurement carried out | person 3 | It depends on the timing of the ultrasound. An early ultrasound is more reliable and provides the best dating. However, if it's a late ultrasound, it's less precise | person 3 | 308 It depends on the timing of the ultrasound. An early ultrasound is more reliable and provides the best dating. However, if it's a late ultrasound, it's less precise |
| CMA Do focus group | Measurement of preterm birth | Appointment for prenatal consultations with ultrasound measurement carried out | person 3 | An ultrasound done in the third trimester is less accurate. But one done in the first trimester generally offers the best dating. | person 3 | 309 An ultrasound done in the third trimester is less accurate. But one done in the first trimester generally offers the best dating. |
| Gynecologist 1 | Measurement of preterm birth | Appointment for prenatal consultations with ultrasound measurement carried out | person 1 | Yes, they do. | person 1 | 310 Yes, they do. |
| Gynecologist 1 | Measurement of preterm birth | Appointment for prenatal consultations with ultrasound measurement carried out | person 1 | We always prioritize the ultrasound. | person 1 | 311 We always prioritize the ultrasound. |
| Gynecologist 2 | Measurement of preterm birth | Appointment for prenatal consultations with ultrasound measurement carried out | person 1 | Yes, many women have an ultrasound before consulting with us. They might visit a local health center or even get an ultrasound independently, then bring the results to us. We then base our assessment on the ultrasound findings | person 1 | 312 Yes, many women have an ultrasound before consulting with us. They might visit a local health center or even get an ultrasound independently, then bring the results to us. We then base our assessment on the ultrasound findings |
| Gynecologist 2 | Measurement of preterm birth | Appointment for prenatal consultations with ultrasound measurement carried out | person 1 | Yes, it becomes the priority, especially if the woman is uncertain about her last menstrual period. The ultrasound provides a tangible reference for us to use | person 1 | 313 Yes, it becomes the priority, especially if the woman is uncertain about her last menstrual period. The ultrasound provides a tangible reference for us to use |
| MCD | Measurement of preterm birth | Appointment for prenatal consultations with ultrasound measurement carried out | person 1 | Occasionally. Midwives in peripheral health facilities, especially in areas like Bobo and rural districts, sometimes request ultrasounds. Women may bring these ultrasounds to the CMA. We also have an ultrasound machine here. The CMA CPNs are reference centers, complementing peripheral health facilities. If a woman brings an ultrasound, we review it without issue. | person 1 | 314 Occasionally. Midwives in peripheral health facilities, especially in areas like Bobo and rural districts, sometimes request ultrasounds. Women may bring these ultrasounds to the CMA. We also have an ultrasound machine here. The CMA CPNs are reference centers, complementing peripheral health facilities. If a woman brings an ultrasound, we review it without issue. |
| MCD | Measurement of preterm birth | Appointment for prenatal consultations with ultrasound measurement carried out | person 1 | Regardless of whether a woman has had an ultrasound or knows her last period date, we measure fundal height (HU). This measurement helps us assess the pregn/acy's progress. Sometimes, there are discrepancies between the last period date and fundal height measurements, indicating potential fetal growth issues. In such cases, we might request an additional ultrasound for a clearer understanding. Fundal height is always measured as part of a standard consultation. | person 1 | 315 Regardless of whether a woman has had an ultrasound or knows her last period date, we measure fundal height (HU). This measurement helps us assess the pregn/acy's progress. Sometimes, there are discrepancies between the last period date and fundal height measurements, indicating potential fetal growth issues. In such cases, we might request an additional ultrasound for a clearer understanding. Fundal height is always measured as part of a standard consultation. |
| CMA Do maternity care unit manager | Measurement of preterm birth | Appointment for prenatal consultations with ultrasound measurement carried out | person 1 | Yes, they do. | person 1 | 316 Yes, they do. |
| CMA Do maternity care unit manager | Measurement of preterm birth | Appointment for prenatal consultations with ultrasound measurement carried out | person 1 | Prioritize in what sense? | person 1 | 317 Prioritize in what sense? |
| CMA Do maternity care unit manager | Measurement of preterm birth | Appointment for prenatal consultations with ultrasound measurement carried out | person 1 | Ultrasound is not always used as the primary method due to its cost. While it is recommended that every pregn/at woman should have at least one ultrasound, especially early in pregn/acy, it's not always feasible. An early ultrasound is helpful for confirming intrauterine pregn/acy. However, if the woman can't afford it, we can't rely solely on this method. In such cases, I personally use the date of the last menstrual period and calculate with the gestogram. | person 1 | 318 Ultrasound is not always used as the primary method due to its cost. While it is recommended that every pregn/at woman should have at least one ultrasound, especially early in pregn/acy, it's not always feasible. An early ultrasound is helpful for confirming intrauterine pregn/acy. However, if the woman can't afford it, we can't rely solely on this method. In such cases, I personally use the date of the last menstrual period and calculate with the gestogram. |
| Colma1 focus group | Measurement of preterm birth | Appointment for prenatal consultations with ultrasound measurement carried out | person 1 | Usually, the ultrasounds are ones we've requested. It's rare for women to get them on their own initiative | person 1 | 319 Usually, the ultrasounds are ones we've requested. It's rare for women to get them on their own initiative |
| Colma1 focus group | Measurement of preterm birth | Appointment for prenatal consultations with ultrasound measurement carried out | person 3 | We generally prioritize the ultrasound. We rely more on it for accuracy. | person 3 | 320 We generally prioritize the ultrasound. We rely more on it for accuracy. |
| Colma1 focus group | Measurement of preterm birth | Appointment for prenatal consultations with ultrasound measurement carried out | person 3 | (laughs) With ultrasounds, we use various parameters to calculate gestational age, including measurements of the fetus and the femur. We find it more reliable than fundal height measurement (HU), especially because HU can be misleading in cases like twin pregn/acies. | person 3 | 321 (laughs) With ultrasounds, we use various parameters to calculate gestational age, including measurements of the fetus and the femur. We find it more reliable than fundal height measurement (HU), especially because HU can be misleading in cases like twin pregn/acies. |
| Colma1 major | Measurement of preterm birth | Appointment for prenatal consultations with ultrasound measurement carried out | person 1 | Yes, those who manage to get an ultrasound bring it along. It provides details like the pregn/acy age, sex of the baby, any malformations, and the amount of amniotic fluid. | person 1 | 322 Yes, those who manage to get an ultrasound bring it along. It provides details like the pregn/acy age, sex of the baby, any malformations, and the amount of amniotic fluid. |
| Colma1 major | Measurement of preterm birth | Appointment for prenatal consultations with ultrasound measurement carried out | person 1 | Yes, exactly. | person 1 | 323 Yes, exactly. |
| Colma1 maternity manager | Measurement of preterm birth | Appointment for prenatal consultations with ultrasound measurement carried out | person 1 | Yes, quite a lot. | person 1 | 324 Yes, quite a lot. |
| Colma1 maternity manager | Measurement of preterm birth | Appointment for prenatal consultations with ultrasound measurement carried out | peerson 1 | We prioritize the ultrasound, especially if it's done in the first trimester. After the first trimester, it becomes a bit more complex. From what I learned, ultrasound measurements are based on the cranial and femoral perimeters, which can vary. For example, a younger person may have different measurements compared to someone older. So, a strong focus is placed on the first trimester ultrasound, as it's crucial for accurate dating. | peerson 1 | 325 We prioritize the ultrasound, especially if it's done in the first trimester. After the first trimester, it becomes a bit more complex. From what I learned, ultrasound measurements are based on the cranial and femoral perimeters, which can vary. For example, a younger person may have different measurements compared to someone older. So, a strong focus is placed on the first trimester ultrasound, as it's crucial for accurate dating. |
| Farakan focus group | Measurement of preterm birth | Appointment for prenatal consultations with ultrasound measurement carried out | person 2 | It's rare. They usually come to us first when their periods stop, and then we might request an ultrasound | person 2 | 326 It's rare. They usually come to us first when their periods stop, and then we might request an ultrasound |
| Farakan focus group | Measurement of preterm birth | Appointment for prenatal consultations with ultrasound measurement carried out | person 4 | Some women come from other places with their ultrasound, which we then interpret and use. | person 4 | 327 Some women come from other places with their ultrasound, which we then interpret and use. |
| Farakan focus group | Measurement of preterm birth | Appointment for prenatal consultations with ultrasound measurement carried out | person 8 | We usually provide most of the ultrasounds. It's uncommon for women to come with their own ultrasound unless they don't know their LMP date. | person 8 | 328 We usually provide most of the ultrasounds. It's uncommon for women to come with their own ultrasound unless they don't know their LMP date. |
| Farakan focus group | Measurement of preterm birth | Appointment for prenatal consultations with ultrasound measurement carried out | person 3 | Most often, we request ultrasounds. However, in urban areas, many women request them for personal convenience | person 3 | 329 Most often, we request ultrasounds. However, in urban areas, many women request them for personal convenience |
| Farakan focus group | Measurement of preterm birth | Appointment for prenatal consultations with ultrasound measurement carried out | person 7 | Educated women often get ultrasounds after clinic consultations and bring them to us. We accept and use these results | person 7 | 330 Educated women often get ultrasounds after clinic consultations and bring them to us. We accept and use these results |
| Farakan major | Measurement of preterm birth | Appointment for prenatal consultations with ultrasound measurement carried out | person 1 | Usually, it's the health workers who suggest an ultrasound, not the women themselves. During prenatal visits (CPNs), we propose an ultrasound to the woman, and if she agrees, we arrange it for her. | person 1 | 331 Usually, it's the health workers who suggest an ultrasound, not the women themselves. During prenatal visits (CPNs), we propose an ultrasound to the woman, and if she agrees, we arrange it for her. |
| Farakan major | Measurement of preterm birth | Appointment for prenatal consultations with ultrasound measurement carried out | person 1 | We prioritize the ultrasound because it's more reliable | person 1 | 332 We prioritize the ultrasound because it's more reliable |
| Farakan maternity manager | Measurement of preterm birth | Appointment for prenatal consultations with ultrasound measurement carried out | person 1 | Yes, they do come with ultrasound results. However, the timing of the ultrasound is crucial. Gynecologists generally recommend an early, first trimester ultrasound for a more accurate probable date of delivery. Ultrasounds performed later, like in the 5th or 6th month of pregn/acy, provide less exact birth dates | person 1 | 333 Yes, they do come with ultrasound results. However, the timing of the ultrasound is crucial. Gynecologists generally recommend an early, first trimester ultrasound for a more accurate probable date of delivery. Ultrasounds performed later, like in the 5th or 6th month of pregn/acy, provide less exact birth dates |
| Farakan maternity manager | Measurement of preterm birth | Appointment for prenatal consultations with ultrasound measurement carried out | person 1 | If the ultrasound is from the very beginning of the pregn/acy, we prioritize it. Gynecologists say that an ultrasound in the first trimester gives a more reliable probable date of delivery compared to one done for the first time at 5 or 6 months of pregn/acy. | person 1 | 334 If the ultrasound is from the very beginning of the pregn/acy, we prioritize it. Gynecologists say that an ultrasound in the first trimester gives a more reliable probable date of delivery compared to one done for the first time at 5 or 6 months of pregn/acy. |
| Accart-ville focus group | Measurement of preterm birth | Priority method if the date of the last period, fundal height and ultrasound are discordant | person 2 | We need to discuss this further. | person 2 | 335 We need to discuss this further. |
| Accart-ville focus group | Measurement of preterm birth | Priority method if the date of the last period, fundal height and ultrasound are discordant | person 1 | If the two examinations don't match, we escalate the case to a higher level (laughs). We refer it to the ASOG for further investigation. | person 1 | 336 If the two examinations don't match, we escalate the case to a higher level (laughs). We refer it to the ASOG for further investigation. |
| Accart-ville focus group | Measurement of preterm birth | Priority method if the date of the last period, fundal height and ultrasound are discordant | person 1 | We mean consulting someone more senior than us. For example, a midwife would refer the case to ASOG. | person 1 | 337 We mean consulting someone more senior than us. For example, a midwife would refer the case to ASOG. |
| Accart-ville focus group | Measurement of preterm birth | Priority method if the date of the last period, fundal height and ultrasound are discordant | person 6 | When the ultrasound and clinical examination don't align, often it's because the ultrasound is outdated. In such cases, we request a new ultrasound to check for any issues. | person 6 | 338 When the ultrasound and clinical examination don't align, often it's because the ultrasound is outdated. In such cases, we request a new ultrasound to check for any issues. |
| Accart-ville focus group | Measurement of preterm birth | Priority method if the date of the last period, fundal height and ultrasound are discordant | person 2 | Usually, when there's a discrepancy, it's due to the ultrasound being old. Therefore, we order a new ultrasound to determine the next steps. | person 2 | 339 Usually, when there's a discrepancy, it's due to the ultrasound being old. Therefore, we order a new ultrasound to determine the next steps. |
| Accart-ville CMU manager | Measurement of preterm birth | Priority method if the date of the last period, fundal height and ultrasound are discordant | person 1 | We consider an early ultrasound, performed before twelve weeks, to be the most accurate. That's the guideline I usually give to our staff. If we have an ultrasound from within the first twelve weeks, we prioritize it. If that's not available, we then consider the chronological age. Lacking both, we resort to clinical examination. | person 1 | 340 We consider an early ultrasound, performed before twelve weeks, to be the most accurate. That's the guideline I usually give to our staff. If we have an ultrasound from within the first twelve weeks, we prioritize it. If that's not available, we then consider the chronological age. Lacking both, we resort to clinical examination. |
| Accart-ville maternity manager | Measurement of preterm birth | Priority method if the date of the last period, fundal height and ultrasound are discordant | person 1 | Discrepancies can occur and can be helpful in diagnosing conditions like twin pregn/acies or polyhydramnios. In such cases, we start with the last menstrual period if it's known | person 1 | 341 Discrepancies can occur and can be helpful in diagnosing conditions like twin pregn/acies or polyhydramnios. In such cases, we start with the last menstrual period if it's known |
| Accart-ville maternity manager | Measurement of preterm birth | Priority method if the date of the last period, fundal height and ultrasound are discordant | person 1 | The last menstrual period is the most effective way because other methods involve measurements, which can vary. For example, measurements may differ based on an individual’s physique. In obese individuals or those with a thick abdominal wall, it's difficult to measure accurately. Even ultrasounds can vary depending on the machine used. That's why we prioritize the date of the last menstrual period | person 1 | 342 The last menstrual period is the most effective way because other methods involve measurements, which can vary. For example, measurements may differ based on an individual’s physique. In obese individuals or those with a thick abdominal wall, it's difficult to measure accurately. Even ultrasounds can vary depending on the machine used. That's why we prioritize the date of the last menstrual period |
| CMA Do focus group | Measurement of preterm birth | Priority method if the date of the last period, fundal height and ultrasound are discordant | person 4 | In cases of discrepancy between what the woman reports, the ultrasound, the gestogram, and the clinical examination, we prioritize the woman's account. We believe it's important to trust the woman carrying the pregn/acy. Even if there's a chance she might be mistaken, we value her input along with the results of the clinical examination. These are the two elements we focus on the most. | person 4 | 343 In cases of discrepancy between what the woman reports, the ultrasound, the gestogram, and the clinical examination, we prioritize the woman's account. We believe it's important to trust the woman carrying the pregn/acy. Even if there's a chance she might be mistaken, we value her input along with the results of the clinical examination. These are the two elements we focus on the most. |
| Gynecologist 1 | Measurement of preterm birth | Priority method if the date of the last period, fundal height and ultrasound are discordant | person 1 | We stick with the ultrasound. It’s more reliable. | person 1 | 344 We stick with the ultrasound. It’s more reliable. |
| Gynecologist 2 | Measurement of preterm birth | Priority method if the date of the last period, fundal height and ultrasound are discordant | person 1 | If there’s a mismatch, say, between fundal height and ultrasound, we have to consider several factors. Fundal height can be biased, influenced by a woman’s body size. It might be overestimated in larger-bodied women and underestimated in slimmer women, so it’s not always reliable. An ultrasound in the first trimester is more accurate. However, if an ultrasound is done later, like in the sixth month, it’s less precise for dating. In such cases, neither method alone gives a clear answer, so we combine both to get a more reliable estimate. | person 1 | 345 If there’s a mismatch, say, between fundal height and ultrasound, we have to consider several factors. Fundal height can be biased, influenced by a woman’s body size. It might be overestimated in larger-bodied women and underestimated in slimmer women, so it’s not always reliable. An ultrasound in the first trimester is more accurate. However, if an ultrasound is done later, like in the sixth month, it’s less precise for dating. In such cases, neither method alone gives a clear answer, so we combine both to get a more reliable estimate. |
| Gynecologist 2 | Measurement of preterm birth | Priority method if the date of the last period, fundal height and ultrasound are discordant | person 1 | Exactly, we use both to synthesize a more reliable estimate. | person 1 | 346 Exactly, we use both to synthesize a more reliable estimate. |
| MCD | Measurement of preterm birth | Priority method if the date of the last period, fundal height and ultrasound are discordant | person 1 | Yes, we typically rely on fundal height as the primary measure. It's not always straightforward, though. When there are discrepancies, we try to understand their causes. For instance, at the CMA, if we suspect a discrepancy, we can use ultrasound to investigate further. Sometimes the discrepancy might be due to a twin pregn/acy, which can lead to measurement errors. It's possible that during a physical examination, only one fetus is detected when there are actually two. This is why we not only measure fundal height but also palpate the abdomen to assess the position and number of fetuses | person 1 | 347 Yes, we typically rely on fundal height as the primary measure. It's not always straightforward, though. When there are discrepancies, we try to understand their causes. For instance, at the CMA, if we suspect a discrepancy, we can use ultrasound to investigate further. Sometimes the discrepancy might be due to a twin pregn/acy, which can lead to measurement errors. It's possible that during a physical examination, only one fetus is detected when there are actually two. This is why we not only measure fundal height but also palpate the abdomen to assess the position and number of fetuses |
| CMA Do maternity care unit manager | Measurement of preterm birth | Priority method if the date of the last period, fundal height and ultrasound are discordant | person 1 | I tend to rely more on the last menstrual period date, as it's generally reliable. You'll find that if you have a clear idea of this date and track it, the difference isn’t too significant compared to an early ultrasound. An early ultrasound, especially before 24 weeks, gives a very accurate estimate. | person 1 | 348 I tend to rely more on the last menstrual period date, as it's generally reliable. You'll find that if you have a clear idea of this date and track it, the difference isn’t too significant compared to an early ultrasound. An early ultrasound, especially before 24 weeks, gives a very accurate estimate. |
| Colma1 focus group | Measurement of preterm birth | Priority method if the date of the last period, fundal height and ultrasound are discordant | person 1 | We place more trust in the ultrasound. The accuracy of the information provided by the woman, such as her last menstrual period, can be uncertain. Similarly, fundal height can be affected by factors like twin pregn/acies or amniotic fluid variations, leading to inaccurate measurements. Ultrasounds give us a closer look at the fetus, providing more detailed information than external measurements. | person 1 | 349 We place more trust in the ultrasound. The accuracy of the information provided by the woman, such as her last menstrual period, can be uncertain. Similarly, fundal height can be affected by factors like twin pregn/acies or amniotic fluid variations, leading to inaccurate measurements. Ultrasounds give us a closer look at the fetus, providing more detailed information than external measurements. |
| Colma1 major | Measurement of preterm birth | Priority method if the date of the last period, fundal height and ultrasound are discordant | person 1 | When there's a discrepancy, we prioritize the ultrasound | person 1 | 350 When there's a discrepancy, we prioritize the ultrasound |
| Colma1 major | Measurement of preterm birth | Priority method if the date of the last period, fundal height and ultrasound are discordant | person 1 | Because it's generally more accurate. Although there can be errors, since it involves human interpretation, we have more confidence in it as a scientific tool. Standard methods, like estimating based on uterine height using posters, can be influenced by various factors, such as the thickness of the uterine wall. Ultrasound is considered much more reliable. | person 1 | 351 Because it's generally more accurate. Although there can be errors, since it involves human interpretation, we have more confidence in it as a scientific tool. Standard methods, like estimating based on uterine height using posters, can be influenced by various factors, such as the thickness of the uterine wall. Ultrasound is considered much more reliable. |
| Colma1 maternity manager | Measurement of preterm birth | Priority method if the date of the last period, fundal height and ultrasound are discordant | person 1 | Let's say a woman comes in the first trimester, and we initially think the pregn/acy is four months along, but the ultrasound indicates two months. In such cases, I would rely on the ultrasound. For instance, if a woman arrives early in her pregn/acy and the physical examination suggests a month of pregn/acy, but the ultrasound shows an advanced gestational age, like in twin pregn/acies, the ultrasound provides a more comprehensive view. In these scenarios, I prefer to base our assessment on the ultrasound findings. | person 1 | 352 Let's say a woman comes in the first trimester, and we initially think the pregn/acy is four months along, but the ultrasound indicates two months. In such cases, I would rely on the ultrasound. For instance, if a woman arrives early in her pregn/acy and the physical examination suggests a month of pregn/acy, but the ultrasound shows an advanced gestational age, like in twin pregn/acies, the ultrasound provides a more comprehensive view. In these scenarios, I prefer to base our assessment on the ultrasound findings. |
| Farakan focus group | Measurement of preterm birth | Priority method if the date of the last period, fundal height and ultrasound are discordant | person 7 | In such cases, we await the birth and examine the newborn for signs of prematurity, like weight and physical appearance | person 7 | 353 In such cases, we await the birth and examine the newborn for signs of prematurity, like weight and physical appearance |
| Farakan focus group | Measurement of preterm birth | Priority method if the date of the last period, fundal height and ultrasound are discordant | person 3 | We trust the woman's words in such cases. | person 3 | 354 We trust the woman's words in such cases. |
| Farakan focus group | Measurement of preterm birth | Priority method if the date of the last period, fundal height and ultrasound are discordant | person 5 | Even if they don't know the exact LMP date, most know the month, which guides us. | person 5 | 355 Even if they don't know the exact LMP date, most know the month, which guides us. |
| Farakan focus group | Measurement of preterm birth | Priority method if the date of the last period, fundal height and ultrasound are discordant | person 7 | We wait for the birth to evaluate based on the baby's weight | person 7 | 356 We wait for the birth to evaluate based on the baby's weight |
| Farakan focus group | Measurement of preterm birth | Priority method if the date of the last period, fundal height and ultrasound are discordant | person 8 | After these exams, it's rare not to determine the pregn/acy age | person 8 | 357 After these exams, it's rare not to determine the pregn/acy age |
| Farakan focus group | Measurement of preterm birth | Priority method if the date of the last period, fundal height and ultrasound are discordant | person 3 | If the woman doesn't know her LMP date, we base it on fundal height. For example, a measurement of 12, 16, or 20 cm corresponds to 3, 4, or 5 months of pregn/acy, respectively. | person 3 | 358 If the woman doesn't know her LMP date, we base it on fundal height. For example, a measurement of 12, 16, or 20 cm corresponds to 3, 4, or 5 months of pregn/acy, respectively. |
| Farakan major | Measurement of preterm birth | Priority method if the date of the last period, fundal height and ultrasound are discordant | person 1 | I find the ultrasound to be more reliable. If we know the exact date of the last menstrual period, we can use our own calculation methods. However, if that date is unknown, it becomes difficult. That's why we rely on the ultrasound. | person 1 | 359 I find the ultrasound to be more reliable. If we know the exact date of the last menstrual period, we can use our own calculation methods. However, if that date is unknown, it becomes difficult. That's why we rely on the ultrasound. |
| Farakan maternity manager | Measurement of preterm birth | Priority method if the date of the last period, fundal height and ultrasound are discordant | person 1 | For the probable date of delivery, an ultrasound can vary by about two weeks from the actual date, and we can adjust for this. If the ultrasound suggests a prolonged pregn/acy, we generally request another ultrasound. After the third trimester, biometrics, especially the child's weight, and placental factors become important. Sometimes, fundal height doesn't align with the pregn/acy age either, which could indicate twin pregn/acy or be affected by maternal obesity. If a woman says she's been pregn/at for 3 months but physical examination suggests 5 or 6 months, there's a discrepancy, and we request an ultrasound for clarity | person 1 | 360 For the probable date of delivery, an ultrasound can vary by about two weeks from the actual date, and we can adjust for this. If the ultrasound suggests a prolonged pregn/acy, we generally request another ultrasound. After the third trimester, biometrics, especially the child's weight, and placental factors become important. Sometimes, fundal height doesn't align with the pregn/acy age either, which could indicate twin pregn/acy or be affected by maternal obesity. If a woman says she's been pregn/at for 3 months but physical examination suggests 5 or 6 months, there's a discrepancy, and we request an ultrasound for clarity |
| Accart-ville focus group | Registration of preterm births | n/a | person 7 | Yes (laughs), it's recorded | person 7 | 361 Yes (laughs), it's recorded |
| Accart-ville focus group | Registration of preterm births | n/a | person 7 | Yes, all deliveries, including premature ones, are recorded. In our monthly reports, we document all relevant parameters, which includes data on prematurity | person 7 | 362 Yes, all deliveries, including premature ones, are recorded. In our monthly reports, we document all relevant parameters, which includes data on prematurity |
| Accart-ville CMU manager | Registration of preterm births | n/a | person 1 | Yes, but I believe there's an issue with under-reporting. We've recently been focusing on quality control and had a meeting to discuss how we can improve and catch up on this. | person 1 | 363 Yes, but I believe there's an issue with under-reporting. We've recently been focusing on quality control and had a meeting to discuss how we can improve and catch up on this. |
| Accart-ville CMU manager | Registration of preterm births | n/a | person 1 | In our monthly reports, we've noticed that premature births aren’t always detailed. We usually just count the number of deliveries without specifying if they were premature. We're now considering how to modify our reporting procedures to better estimate and document this information. | person 1 | 364 In our monthly reports, we've noticed that premature births aren’t always detailed. We usually just count the number of deliveries without specifying if they were premature. We're now considering how to modify our reporting procedures to better estimate and document this information. |
| Accart-ville maternity manager | Registration of preterm births | n/a | person 1 | Currently, we only have the birth register, which also includes parameters for prematurity. Ideally, we should have a separate register specifically for premature births. I've recently completed supervision on newborn care, including the care of premature newborns. The recommendation is to maintain a distinct registry for premature newborns. In our birth register, we record whether a birth is premature or not, along with the gestational age. We also note the number of low birth weight children and specify if they are premature. All these details are included in our delivery register. | person 1 | 365 Currently, we only have the birth register, which also includes parameters for prematurity. Ideally, we should have a separate register specifically for premature births. I've recently completed supervision on newborn care, including the care of premature newborns. The recommendation is to maintain a distinct registry for premature newborns. In our birth register, we record whether a birth is premature or not, along with the gestational age. We also note the number of low birth weight children and specify if they are premature. All these details are included in our delivery register. |
| Accart-ville maternity manager | Registration of preterm births | n/a | person 1 | Yes, this information is included in our monthly activity report. We have specific sections dedicated to recording data on prematurity. | person 1 | 366 Yes, this information is included in our monthly activity report. We have specific sections dedicated to recording data on prematurity. |
| CMA Do focus group | Registration of preterm births | n/a | person 9 | Yes, it is. We record it, as there is a specific section in the register for noting the gestational age of each birth | person 9 | 367 Yes, it is. We record it, as there is a specific section in the register for noting the gestational age of each birth |
| CMA Do focus group | Registration of preterm births | n/a | person 6 | Yes, it is. Prematurity details are included in the monthly activity reports. | person 6 | 368 Yes, it is. Prematurity details are included in the monthly activity reports. |
| Gynecologist 1 | Registration of preterm births | n/a | person 1 | Yes, it is. | person 1 | 369 Yes, it is. |
| Gynecologist 1 | Registration of preterm births | n/a | person 1 | Yes, details on premature births are included in the monthly reports. | person 1 | 370 Yes, details on premature births are included in the monthly reports. |
| Gynecologist 2 | Registration of preterm births | n/a | person 1 | Yes, premature births are recorded in the birth register, just like other births. We have a register where we note various birth parameters when a child is born. These parameters, along with the gestational age determined at the start of the pregn/acy, help us identify if the birth is premature. So, the evidence of prematurity is found in the patient files. In the delivery register, we record the newborn's parameters that indicate prematurity. However, we do not have a separate register specifically for premature births. | person 1 | 371 Yes, premature births are recorded in the birth register, just like other births. We have a register where we note various birth parameters when a child is born. These parameters, along with the gestational age determined at the start of the pregn/acy, help us identify if the birth is premature. So, the evidence of prematurity is found in the patient files. In the delivery register, we record the newborn's parameters that indicate prematurity. However, we do not have a separate register specifically for premature births. |
| Gynecologist 2 | Registration of preterm births | n/a | person 1 | I'm not entirely sure. I don't think so, as it should be consolidated with other childbirth data. I believe the head of the maternity care unit might have more detailed information. I'mMCD not certain if there's a distinction between premature and full-term births in the report. All I know is that all births, regardless of their nature, are recorded in the birth register. | person 1 | 372 I'm not entirely sure. I don't think so, as it should be consolidated with other childbirth data. I believe the head of the maternity care unit might have more detailed information. I'mMCD not certain if there's a distinction between premature and full-term births in the report. All I know is that all births, regardless of their nature, are recorded in the birth register. |
| Gynecologist 2 | Registration of preterm births | n/a | person 1 | Yes, she can clarify whether there's a differentiation between premature and full-term births in our reporting. But as far as I know, all births are listed in the birth register. | person 1 | 373 Yes, she can clarify whether there's a differentiation between premature and full-term births in our reporting. But as far as I know, all births are listed in the birth register. |
| MCD | Registration of preterm births | n/a | person 1 | Yes, we do note it in the registers, but it's not systematically collected. For example, if we record that a child was born at 7 months, we implicitly indicate it's a premature birth. However, we don't explicitly collect 'prematurity' as a separate data point. | person 1 | 374 Yes, we do note it in the registers, but it's not systematically collected. For example, if we record that a child was born at 7 months, we implicitly indicate it's a premature birth. However, we don't explicitly collect 'prematurity' as a separate data point. |
| MCD | Registration of preterm births | n/a | person 1 | No, we don't include it. Our primary data collection process doesn't specifically account for prematurity details. | person 1 | 375 No, we don't include it. Our primary data collection process doesn't specifically account for prematurity details. |
| CMA Do maternity care unit manager | Registration of preterm births | n/a | person 1 | Not really. We do record the birth weight, but that alone isn't enough. There are other factors that should be considered, but most of the time, they aren't noted. We can't determine prematurity based solely on weight, as there are also cases of low birth weight due to other reasons, like hypotrophy. So, we often lack sufficient information to classify a birth as premature. | person 1 | 376 Not really. We do record the birth weight, but that alone isn't enough. There are other factors that should be considered, but most of the time, they aren't noted. We can't determine prematurity based solely on weight, as there are also cases of low birth weight due to other reasons, like hypotrophy. So, we often lack sufficient information to classify a birth as premature. |
| CMA Do maternity care unit manager | Registration of preterm births | n/a | person 1 | When preparing the monthly report, it's hard to trace prematurity because it's not specifically noted in the register. We use the register for data collection. We can identify low birth weight, like weights less than 2500 grams, but we don’t have enough information to distinguish between premature births and hypotrophic cases | person 1 | 377 When preparing the monthly report, it's hard to trace prematurity because it's not specifically noted in the register. We use the register for data collection. We can identify low birth weight, like weights less than 2500 grams, but we don’t have enough information to distinguish between premature births and hypotrophic cases |
| CMA Do maternity care unit manager | Registration of preterm births | n/a | person 1 | No, there isn’t a specific register for that. Ideally, such details should be included in the birth register. | person 1 | 378 No, there isn’t a specific register for that. Ideally, such details should be included in the birth register. |
| Colma1 focus group | Registration of preterm births | n/a | person 5 | In a register? | person 5 | 379 In a register? |
| Colma1 focus group | Registration of preterm births | n/a | person 6 | Of course, premature births are recorded in the register. We even include this information in our monthly reports. | person 6 | 380 Of course, premature births are recorded in the register. We even include this information in our monthly reports. |
| Colma1 focus group | Registration of preterm births | n/a | person 2 | Yes, in the birth register, we specifically note whether a child is full-term or premature. It's clearly indicated | person 2 | 381 Yes, in the birth register, we specifically note whether a child is full-term or premature. It's clearly indicated |
| Colma1 focus group | Registration of preterm births | n/a | person 2 | Do you mean starting from today, going back seven months? | person 2 | 382 Do you mean starting from today, going back seven months? |
| Colma1 focus group | Registration of preterm births | n/a | person 2 | Okay, I understand. However, our senior staff member who usually handles this is currently away. | person 2 | 383 Okay, I understand. However, our senior staff member who usually handles this is currently away. |
| Colma1 major | Registration of preterm births | n/a | person 1 | Yes, it's recorded. | person 1 | 384 Yes, it's recorded. |
| Colma1 major | Registration of preterm births | n/a | person 1 | Yes, information about premature births is included in the monthly report. | person 1 | 385 Yes, information about premature births is included in the monthly report. |
| Colma1 maternity manager | Registration of preterm births | n/a | person 1 | Yes, it's included in the birth register. There's even a specific section that indicates whether a child is full-term or premature | person 1 | 386 Yes, it's included in the birth register. There's even a specific section that indicates whether a child is full-term or premature |
| Colma1 maternity manager | Registration of preterm births | n/a | person 1 | Yes, of course. We are required to include it. Premature births are clearly marked in the report. | person 1 | 387 Yes, of course. We are required to include it. Premature births are clearly marked in the report. |
| Colma1 maternity manager | Registration of preterm births | n/a | person 1 | There is no problem with that. We can provide those estimates | person 1 | 388 There is no problem with that. We can provide those estimates |
| Farakan focus group | Registration of preterm births | n/a | person 2 | Yes, premature births are recorded in the birth registers | person 2 | 389 Yes, premature births are recorded in the birth registers |
| Farakan focus group | Registration of preterm births | n/a | person 2 | Yes, information on premature births is included in the monthly reports as well. | person 2 | 390 Yes, information on premature births is included in the monthly reports as well. |
| Farakan focus group | Registration of preterm births | n/a | person 3 | Indeed, our registers have a specific section for premature babies, and this is also highlighted in our reports | person 3 | 391 Indeed, our registers have a specific section for premature babies, and this is also highlighted in our reports |
| Farakan focus group | Registration of preterm births | n/a | person 7 | In the registers, premature births are clearly noted. We document it in the birth register, and if there is a need for referral, that information is also included. | person 7 | 392 In the registers, premature births are clearly noted. We document it in the birth register, and if there is a need for referral, that information is also included. |
| Farakan major | Registration of preterm births | n/a | person 1 | Yes, it is. | person 1 | 393 Yes, it is. |
| Farakan major | Registration of preterm births | n/a | person 1 | In our current reporting template, details about prematurity are not highlighted. However, in the birth registers, we do record information on premature births. The monthly report primarily mentions the 'number of full-term newborns,' so prematurity data isn't explicitly noted there | person 1 | 394 In our current reporting template, details about prematurity are not highlighted. However, in the birth registers, we do record information on premature births. The monthly report primarily mentions the 'number of full-term newborns,' so prematurity data isn't explicitly noted there |
| Farakan maternity manager | Registration of preterm births | n/a | person 1 | Yes, it is. We specifically mention if a birth is premature in the birth register, including the gestational age | person 1 | 395 Yes, it is. We specifically mention if a birth is premature in the birth register, including the gestational age |
| Farakan maternity manager | Registration of preterm births | n/a | person 1 | In the monthly reports? Yes, it is. The data in our monthly reports come from what is recorded in the register. | person 1 | 396 In the monthly reports? Yes, it is. The data in our monthly reports come from what is recorded in the register. |
| Accart-ville focus group | Number of preterm births per month | n/a | person 2 | We would need to refer to the monthly activity report (RMA) for exact numbers. It's hard to say without checking. | person 2 | 397 We would need to refer to the monthly activity report (RMA) for exact numbers. It's hard to say without checking. |
| Accart-ville focus group | Number of preterm births per month | n/a | person 1 | It's not a high number. On average, there are about one or two premature births per month. It doesn't usually exceed that. | person 1 | 398 It's not a high number. On average, there are about one or two premature births per month. It doesn't usually exceed that. |
| Accart-ville focus group | Number of preterm births per month | n/a | person 3 | I believe it's higher than that. Based on the reports I've reviewed, prematurity rates are about 10 to 15% per month. I have the reports for the last nine months. | person 3 | 399 I believe it's higher than that. Based on the reports I've reviewed, prematurity rates are about 10 to 15% per month. I have the reports for the last nine months. |
| Accart-ville focus group | Number of preterm births per month | n/a | person 3 | Okay. | person 3 | 400 Okay. |
| Accart-ville CMU manager | Number of preterm births per month | n/a | person 1 | It's difficult to give an exact number. Our current system doesn't specifically track the number of premature births in the final report. We would need to individually review the gestational age recorded in the birth register for each birth to determine this. If our monthly activity reports specifically asked for the number of premature births, it would encourage more careful tracking. As it stands, however, this level of detail is not routinely monitored | person 1 | 401 It's difficult to give an exact number. Our current system doesn't specifically track the number of premature births in the final report. We would need to individually review the gestational age recorded in the birth register for each birth to determine this. If our monthly activity reports specifically asked for the number of premature births, it would encourage more careful tracking. As it stands, however, this level of detail is not routinely monitored |
| Accart-ville CMU manager | Number of preterm births per month | n/a | person 1 | Even for the maternity manager, it might be challenging. As I mentioned, our monthly reports indicate the total number of births, but not the number of premature births. To determine that, we'd need to analyze the birth data for each individual, checking gestational age, birth weight, and other parameters from the physical examination. This analysis would have to be done case by case to gather the information. | person 1 | 402 Even for the maternity manager, it might be challenging. As I mentioned, our monthly reports indicate the total number of births, but not the number of premature births. To determine that, we'd need to analyze the birth data for each individual, checking gestational age, birth weight, and other parameters from the physical examination. This analysis would have to be done case by case to gather the information. |
| Accart-ville CMU manager | Number of preterm births per month | n/a | person 1 | In summary, our final reports currently don't include specific data on premature births. | person 1 | 403 In summary, our final reports currently don't include specific data on premature births. |
| Accart-ville maternity manager | Number of preterm births per month | n/a | person 1 | It's challenging to give an exact figure because there's often confusion between low birth weight, hypotrophy, and prematurity, leading to underestimation. However, we do see a significant number of premature births. I would estimate it to be around 5 to 10% of our monthly deliveries. | person 1 | 404 It's challenging to give an exact figure because there's often confusion between low birth weight, hypotrophy, and prematurity, leading to underestimation. However, we do see a significant number of premature births. I would estimate it to be around 5 to 10% of our monthly deliveries. |
| Accart-ville maternity manager | Number of preterm births per month | n/a | person 1 | Sure, there's no problem with that. We have both the logs and the monthly activity reports (RMA) for the last nine months. I recently used them to prepare my slides for an activity | person 1 | 405 Sure, there's no problem with that. We have both the logs and the monthly activity reports (RMA) for the last nine months. I recently used them to prepare my slides for an activity |
| CMA Do focus group | Number of preterm births per month | n/a | person 6 | The number of premature births usually doesn't exceed 30 to 40 per month. | person 6 | 406 The number of premature births usually doesn't exceed 30 to 40 per month. |
| CMA Do focus group | Number of preterm births per month | n/a | person 1 | It varies from month to month. Some months we might exceed that number, while in others we don't. That's just an average. | person 1 | 407 It varies from month to month. Some months we might exceed that number, while in others we don't. That's just an average. |
| CMA Do focus group | Number of preterm births per month | n/a | person 6 | Yes, that information can be found in the monthly activity reports, which are managed by the SUS (Care Unit Monitoring) manager | person 6 | 408 Yes, that information can be found in the monthly activity reports, which are managed by the SUS (Care Unit Monitoring) manager |
| Gynecologist 1 | Number of preterm births per month | n/a | person 1 | To give you an exact figure would be difficult without consulting the statistics. But from what I recall, last month, we had about two or three premature babies. | person 1 | 409 To give you an exact figure would be difficult without consulting the statistics. But from what I recall, last month, we had about two or three premature babies. |
| Gynecologist 1 | Number of preterm births per month | n/a | person 1 | Yes, we can collect that information on premature births | person 1 | 410 Yes, we can collect that information on premature births |
| Gynecologist 1 | Number of preterm births per month | n/a | person 1 | Yes, there is. | person 1 | 411 Yes, there is. |
| Gynecologist 2 | Number of preterm births per month | n/a | person 1 | We average around a hundred births per week. | person 1 | 412 We average around a hundred births per week. |
| Gynecologist 2 | Number of preterm births per month | n/a | person 1 | No, not a hundred premature births, I got confused. That's the overall number of births. As for premature births, we haven't conducted statistical studies to determine their proportion. It's difficult to provide an exact figure because we haven't done the calculations yet. However, if we were to examine the birth registers retrospectively, we might be able to find the information we need. | person 1 | 413 No, not a hundred premature births, I got confused. That's the overall number of births. As for premature births, we haven't conducted statistical studies to determine their proportion. It's difficult to provide an exact figure because we haven't done the calculations yet. However, if we were to examine the birth registers retrospectively, we might be able to find the information we need. |
| MCD | Number of preterm births per month | n/a | person 1 | We don’t specifically collect that data. It's not part of our traditional data collection. I've had similar inquiries before, and I've mentioned that we don't track this information routinely. However, if someone needed this information for a study, I could potentially help them gather it, allowing for an extrapolation. But as it stands, we don’t have this data readily available | person 1 | 414 We don’t specifically collect that data. It's not part of our traditional data collection. I've had similar inquiries before, and I've mentioned that we don't track this information routinely. However, if someone needed this information for a study, I could potentially help them gather it, allowing for an extrapolation. But as it stands, we don’t have this data readily available |
| CMA Do maternity care unit manager | Number of preterm births per month | n/a | person 1 | As an estimate, I would say we have about 10 premature births per month. I'll go with 10 as the approximate number. | person 1 | 415 As an estimate, I would say we have about 10 premature births per month. I'll go with 10 as the approximate number. |
| Colma1 focus group | Number of preterm births per month | n/a | person 2 | As an estimate, the number of premature births varies. Some months, we might have two cases, and other times, there might not be any | person 2 | 416 As an estimate, the number of premature births varies. Some months, we might have two cases, and other times, there might not be any |
| Colma1 focus group | Number of preterm births per month | n/a | person 3 | During our last shift, we had one case of a premature birth. | person 3 | 417 During our last shift, we had one case of a premature birth. |
| Colma1 major | Number of preterm births per month | n/a | person 1 | How many? | person 1 | 418 How many? |
| Colma1 major | Number of preterm births per month | n/a | person 1 | Well, the monthly report doesn’t clearly specify premature births. For instance, we note the number of full-term newborns weighing less than 2.5kg, implying that these are full-term but low-weight births. However, there isn't a specific line in the report that lists premature births separately. We don't see a separate count for premature births in the report, but we can find this information in the birth register | person 1 | 419 Well, the monthly report doesn’t clearly specify premature births. For instance, we note the number of full-term newborns weighing less than 2.5kg, implying that these are full-term but low-weight births. However, there isn't a specific line in the report that lists premature births separately. We don't see a separate count for premature births in the report, but we can find this information in the birth register |
| Colma1 major | Number of preterm births per month | n/a | person 1 | Yes, the registers with this information are available. | person 1 | 420 Yes, the registers with this information are available. |
| Colma1 maternity manager | Number of preterm births per month | n/a | person 1 | Every month? | person 1 | 421 Every month? |
| Colma1 maternity manager | Number of preterm births per month | n/a | person 1 | As an estimate, out of 100 women who give birth, around five to ten are premature births. That’s a general figure. In terms of monthly numbers, it’s often around two, but it varies. In our register, I don't think the number of premature births exceeds two or three a month. However, during the winter, the number of premature births tends to increase due to complications from conditions like malaria and other infections. These can lead to an uptick in premature births as well as abortions. | person 1 | 422 As an estimate, out of 100 women who give birth, around five to ten are premature births. That’s a general figure. In terms of monthly numbers, it’s often around two, but it varies. In our register, I don't think the number of premature births exceeds two or three a month. However, during the winter, the number of premature births tends to increase due to complications from conditions like malaria and other infections. These can lead to an uptick in premature births as well as abortions. |
| Farakan focus group | Number of preterm births per month | n/a | person 6 | At our health center, there are some months when we don't have any cases of prematurity. Looking back over the last six months, the highest number we've seen in a single month would be around two or three premature births | person 6 | 423 At our health center, there are some months when we don't have any cases of prematurity. Looking back over the last six months, the highest number we've seen in a single month would be around two or three premature births |
| Farakan focus group | Number of preterm births per month | n/a | person 8 | Premature births are actually quite rare here. They do occur, but it’s not a common situation. | person 8 | 424 Premature births are actually quite rare here. They do occur, but it’s not a common situation. |
| Farakan focus group | Number of preterm births per month | n/a | person 4 | Since we provide antenatal care (ANC), we advise women on proper nutrition to help prevent premature births. We emphasize good dietary habits, which has led to a decrease in the number of premature babies at our center | person 4 | 425 Since we provide antenatal care (ANC), we advise women on proper nutrition to help prevent premature births. We emphasize good dietary habits, which has led to a decrease in the number of premature babies at our center |
| Farakan major | Number of preterm births per month | n/a | person 1 | Premature births are quite rare here. We usually don't have more than one or two per month. | person 1 | 426 Premature births are quite rare here. We usually don't have more than one or two per month. |
| Farakan major | Number of preterm births per month | n/a | person 1 | In the current monthly activity report (RMA), premature births aren’t specifically highlighted. In the old RMA format, they were more apparent. Now, the RMA mentions 'Full term babies' weighing less than 2.5kg, but that's not the same as tracking premature births. | person 1 | 427 In the current monthly activity report (RMA), premature births aren’t specifically highlighted. In the old RMA format, they were more apparent. Now, the RMA mentions 'Full term babies' weighing less than 2.5kg, but that's not the same as tracking premature births. |
| Farakan maternity manager | Number of preterm births per month | n/a | person 1 | I don't have an exact figure offhand. | person 1 | 428 I don't have an exact figure offhand. |
| Farakan maternity manager | Number of preterm births per month | n/a | person 1 | Well, it could be around 4 to 5 premature births per month. We also frequently have twin deliveries | person 1 | 429 Well, it could be around 4 to 5 premature births per month. We also frequently have twin deliveries |
| Farakan maternity manager | Number of preterm births per month | n/a | person 1 | Yes, I would need to check the reports to provide accurate information. I had the report earlier, but I need to retrieve it again | person 1 | 430 Yes, I would need to check the reports to provide accurate information. I had the report earlier, but I need to retrieve it again |
| Accart-ville focus group | Types of challenges faced during preterm births | n/a | person 2 | Premature birth is indeed a priority and a health problem due to its consequences. We deal with prematurity cases daily and often need to transfer them to higher-level facilities for better care | person 2 | 431 Premature birth is indeed a priority and a health problem due to its consequences. We deal with prematurity cases daily and often need to transfer them to higher-level facilities for better care |
| Accart-ville focus group | Types of challenges faced during preterm births | n/a | person 2 | Our main challenge is the lack of adequate care resources. We don't have a kangaroo care unit or incubators, which are essential for managing hypothermia and other issues in premature babies. When a baby's reflexes are not fully developed, we have to evacuate them to a facility with more resources | person 2 | 432 Our main challenge is the lack of adequate care resources. We don't have a kangaroo care unit or incubators, which are essential for managing hypothermia and other issues in premature babies. When a baby's reflexes are not fully developed, we have to evacuate them to a facility with more resources |
| Accart-ville focus group | Types of challenges faced during preterm births | n/a | person 1 | A kangaroo unit is crucial for combating hypothermia, a leading cause of neonatal death. | person 1 | 433 A kangaroo unit is crucial for combating hypothermia, a leading cause of neonatal death. |
| Accart-ville focus group | Types of challenges faced during preterm births | n/a | person 1 | As previously mentioned, hypothermia is a major concern. We lack the facilities to adequately care for premature babies, so they are usually transferred to the CHUSS for specialized care under lamps and to prevent infections. We don't have the capacity to treat premature babies on-site and must rely on higher-level facilities. | person 1 | 434 As previously mentioned, hypothermia is a major concern. We lack the facilities to adequately care for premature babies, so they are usually transferred to the CHUSS for specialized care under lamps and to prevent infections. We don't have the capacity to treat premature babies on-site and must rely on higher-level facilities. |
| Accart-ville CMU manager | Types of challenges faced during preterm births | n/a | person 1 | Yes, we face challenges with premature births. Our facility lacks the resources to adequately care for these cases, leading us to transfer almost all cases to Souro Sanou hospital. We're planning to establish a link with neonatology to better track statistics on prematurity. We'll have a reference book to monitor and evaluate our performance, which should be ready within the week. | person 1 | 435 Yes, we face challenges with premature births. Our facility lacks the resources to adequately care for these cases, leading us to transfer almost all cases to Souro Sanou hospital. We're planning to establish a link with neonatology to better track statistics on prematurity. We'll have a reference book to monitor and evaluate our performance, which should be ready within the week. |
| Accart-ville CMU manager | Types of challenges faced during preterm births | n/a | person 1 | We face several challenges. Despite being upgraded to a higher-level health center, our infrastructure hasn't kept up. We often experience high patient volume, with up to ten deliveries in one night, but with only two or three staff members available, it's difficult to provide adequate care to everyone. We also have a shortage of beds, leading to patients lying on the floor. Additionally, handling pathologies linked to pregn/acy is challenging due to staff limitations. Previously, we would refer any pregn/acy-related illness to higher-level facilities, but now, being a higher-level center ourselves, we're expected to manage these cases with limited resources and staff. | person 1 | 436 We face several challenges. Despite being upgraded to a higher-level health center, our infrastructure hasn't kept up. We often experience high patient volume, with up to ten deliveries in one night, but with only two or three staff members available, it's difficult to provide adequate care to everyone. We also have a shortage of beds, leading to patients lying on the floor. Additionally, handling pathologies linked to pregn/acy is challenging due to staff limitations. Previously, we would refer any pregn/acy-related illness to higher-level facilities, but now, being a higher-level center ourselves, we're expected to manage these cases with limited resources and staff. |
| Accart-ville maternity manager | Types of challenges faced during preterm births | n/a | person 1 | Yes, premature birth poses a major health challenge. We often face difficulties in providing care, and parents frequently refuse to transfer their premature infants to higher-level facilities for further treatment. Even when we do evacuate, hospitals like Souro Sanou, which are equipped with incubators, often lack space, leading to infants being sent back to us with only oral prescriptions. This situation is problematic due to the limited resources and space for premature infants in the entire region | person 1 | 437 Yes, premature birth poses a major health challenge. We often face difficulties in providing care, and parents frequently refuse to transfer their premature infants to higher-level facilities for further treatment. Even when we do evacuate, hospitals like Souro Sanou, which are equipped with incubators, often lack space, leading to infants being sent back to us with only oral prescriptions. This situation is problematic due to the limited resources and space for premature infants in the entire region |
| Accart-ville maternity manager | Types of challenges faced during preterm births | n/a | person 1 | Parents' refusal is often rooted in their perceptions about the viability of premature newborns. Many believe that the child may not survive, so they feel it's not worth the time and effort. They worry about the time commitment and the impact on their daily activities. | person 1 | 438 Parents' refusal is often rooted in their perceptions about the viability of premature newborns. Many believe that the child may not survive, so they feel it's not worth the time and effort. They worry about the time commitment and the impact on their daily activities. |
| Accart-ville maternity manager | Types of challenges faced during preterm births | n/a | person 1 | Infections are a common problem that can lead to prematurity. We often lack the necessary medications to manage the threat of premature births effectively. When prescriptions are given for medication from private pharmacies, many families cannot afford them. Additionally, once a premature child is born, we lack the essential equipment and resources to provide adequate care | person 1 | 439 Infections are a common problem that can lead to prematurity. We often lack the necessary medications to manage the threat of premature births effectively. When prescriptions are given for medication from private pharmacies, many families cannot afford them. Additionally, once a premature child is born, we lack the essential equipment and resources to provide adequate care |
| Accart-ville maternity manager | Types of challenges faced during preterm births | n/a | person 1 | Primarily, we need heat lamps to prevent hypothermia in newborns, especially premature ones. Infection prevention is another concern, as we don't have individual rooms or dedicated spaces for premature babies or even newborns. Our facility only has one large room that serves multiple purposes. It accommodates sick individuals, women in labor, and postpartum women, all sharing 10 beds. Frequently, these beds are fully occupied, leading some women to spread loincloths on the floor for themselves and their premature newborns, which is far from ideal. This shared room is designed for 10 beds. | person 1 | 440 Primarily, we need heat lamps to prevent hypothermia in newborns, especially premature ones. Infection prevention is another concern, as we don't have individual rooms or dedicated spaces for premature babies or even newborns. Our facility only has one large room that serves multiple purposes. It accommodates sick individuals, women in labor, and postpartum women, all sharing 10 beds. Frequently, these beds are fully occupied, leading some women to spread loincloths on the floor for themselves and their premature newborns, which is far from ideal. This shared room is designed for 10 beds. |
| CMA Do focus group | Types of challenges faced during preterm births | n/a | person 6 | Yes, premature birth is definitely an obstetric emergency and a major challenge, especially in our context where we lack specialists like pediatricians and intensivists. | person 6 | 441 Yes, premature birth is definitely an obstetric emergency and a major challenge, especially in our context where we lack specialists like pediatricians and intensivists. |
| CMA Do focus group | Types of challenges faced during preterm births | n/a | person 7 | The main challenge is the lack of equipment needed to care for premature births. | person 7 | 442 The main challenge is the lack of equipment needed to care for premature births. |
| CMA Do focus group | Types of challenges faced during preterm births | n/a | person 6 | This leads to a high rate of neonatal mortality. | person 6 | 443 This leads to a high rate of neonatal mortality. |
| CMA Do focus group | Types of challenges faced during preterm births | n/a | person 6 | Yes, and these cases are usually sent to the SUS or CHURSS, as our facility primarily provides kangaroo mother care and manages only low birth weights. | person 6 | 444 Yes, and these cases are usually sent to the SUS or CHURSS, as our facility primarily provides kangaroo mother care and manages only low birth weights. |
| CMA Do focus group | Types of challenges faced during preterm births | n/a | person 6 | Delivering extremely premature babies is a big challenge. We have to be careful with maneuvers, sometimes requiring episiotomies or cesarean sections, especially in breech presentations. | person 6 | 445 Delivering extremely premature babies is a big challenge. We have to be careful with maneuvers, sometimes requiring episiotomies or cesarean sections, especially in breech presentations. |
| CMA Do focus group | Types of challenges faced during preterm births | n/a | person 5 | Resuscitation is also a critical aspect, including heating, clearing obstructions, and ensuring oxygenation. | person 5 | 446 Resuscitation is also a critical aspect, including heating, clearing obstructions, and ensuring oxygenation. |
| CMA Do focus group | Types of challenges faced during preterm births | n/a | person 9 | Yes, especially since it's hard to predict premature births unless there are obvious threats or a patient's history indicating a risk. | person 9 | 447 Yes, especially since it's hard to predict premature births unless there are obvious threats or a patient's history indicating a risk. |
| Gynecologist 1 | Types of challenges faced during preterm births | n/a | person 1 | Yes, absolutely. Premature babies present numerous challenges for us, especially in terms of their care and rearing. These aspects pose significant difficulties | person 1 | 448 Yes, absolutely. Premature babies present numerous challenges for us, especially in terms of their care and rearing. These aspects pose significant difficulties |
| Gynecologist 1 | Types of challenges faced during preterm births | n/a | person 1 | Premature babies are very fragile and often experience respiratory distress, difficulties in regulating temperature, and are prone to infections. They require close monitoring and careful treatment. | person 1 | 449 Premature babies are very fragile and often experience respiratory distress, difficulties in regulating temperature, and are prone to infections. They require close monitoring and careful treatment. |
| Gynecologist 1 | Types of challenges faced during preterm births | n/a | person 1 | In terms of pregn/acy, the challenge is often related to the causes leading to prematurity. Many premature births here occur due to reasons like the breaking of water or severe preeclampsia, necessitating early delivery of the babies. This situation itself poses a significant challenge during pregn/acy | person 1 | 450 In terms of pregn/acy, the challenge is often related to the causes leading to prematurity. Many premature births here occur due to reasons like the breaking of water or severe preeclampsia, necessitating early delivery of the babies. This situation itself poses a significant challenge during pregn/acy |
| Gynecologist 2 | Types of challenges faced during preterm births | n/a | person 1 | Yes, it's definitely a challenge, not just for us but for other centers too. A majority of our hospitalizations involve pregn/at women who are not full term. The challenge is to manage these pregn/acies, either to carry them to term or until the child is mature enough for delivery or cesarean section. We frequently encounter pregn/at women hospitalized with pregn/acies that aren't full term, often leading to premature births. | person 1 | 451 Yes, it's definitely a challenge, not just for us but for other centers too. A majority of our hospitalizations involve pregn/at women who are not full term. The challenge is to manage these pregn/acies, either to carry them to term or until the child is mature enough for delivery or cesarean section. We frequently encounter pregn/at women hospitalized with pregn/acies that aren't full term, often leading to premature births. |
| Gynecologist 2 | Types of challenges faced during preterm births | n/a | person 1 | Yes, premature birth presents numerous problems. We take measures, though we lack some resources, to ensure that the premature baby is in a viable condition for survival. | person 1 | 452 Yes, premature birth presents numerous problems. We take measures, though we lack some resources, to ensure that the premature baby is in a viable condition for survival. |
| Gynecologist 2 | Types of challenges faced during preterm births | n/a | person 1 | From birth, we utilize our kangaroo unit. It's not a pediatric unit but is staffed with trained personnel for caring for non-sick premature babies. We focus on keeping the child warm, avoiding contact with external air, and if the child shows good reflexes and adapts well, we entrust them to the kangaroo unit. This unit then educates the mother and family on how to feed and keep the child warm until they reach an acceptable weight to behave like other babies. | person 1 | 453 From birth, we utilize our kangaroo unit. It's not a pediatric unit but is staffed with trained personnel for caring for non-sick premature babies. We focus on keeping the child warm, avoiding contact with external air, and if the child shows good reflexes and adapts well, we entrust them to the kangaroo unit. This unit then educates the mother and family on how to feed and keep the child warm until they reach an acceptable weight to behave like other babies. |
| Gynecologist 2 | Types of challenges faced during preterm births | n/a | person 1 | In our facility, the minimum acceptable weight is 2.5 kg. Premature babies usually weigh less than this. If the child has good reflexes and can feed properly, we provide care until they reach this weight. However, if they can't feed or suck properly and are below 2.5 kg, they are usually sent to pediatrics in Souro Sanou. | person 1 | 454 In our facility, the minimum acceptable weight is 2.5 kg. Premature babies usually weigh less than this. If the child has good reflexes and can feed properly, we provide care until they reach this weight. However, if they can't feed or suck properly and are below 2.5 kg, they are usually sent to pediatrics in Souro Sanou. |
| Gynecologist 2 | Types of challenges faced during preterm births | n/a | person 1 | Yes, we see a range of prematurity. Babies born between 28 and 37 weeks are considered premature. Those around 36 weeks can behave like full-term babies, but very premature babies, around 28 weeks and often weighing between 1.2 to 1.5 kg, are the biggest challenge. They can't be fed properly here and are usually evacuated to the hospital. | person 1 | 455 Yes, we see a range of prematurity. Babies born between 28 and 37 weeks are considered premature. Those around 36 weeks can behave like full-term babies, but very premature babies, around 28 weeks and often weighing between 1.2 to 1.5 kg, are the biggest challenge. They can't be fed properly here and are usually evacuated to the hospital. |
| MCD | Types of challenges faced during preterm births | n/a | person 1 | Yes, prematurity presents significant challenges. The difficulty lies in the degree of prematurity; extreme cases are particularly hard to manage due to limited resources. In such instances, we are compelled to send the babies to the hospital. However, for those with reasonable birth weights, we have a kangaroo baby care service that supports the mother and her baby until the baby reaches a weight conducive to growth. Our current limitations include a lack of equipment and training necessary to effectively handle these situations. They are challenging for health workers to manage effectively at any time. Hence, prematurity is indeed a significant challenge for us, and we are hopeful for improvements in our operational capacity to better address these challenges in the future | person 1 | 456 Yes, prematurity presents significant challenges. The difficulty lies in the degree of prematurity; extreme cases are particularly hard to manage due to limited resources. In such instances, we are compelled to send the babies to the hospital. However, for those with reasonable birth weights, we have a kangaroo baby care service that supports the mother and her baby until the baby reaches a weight conducive to growth. Our current limitations include a lack of equipment and training necessary to effectively handle these situations. They are challenging for health workers to manage effectively at any time. Hence, prematurity is indeed a significant challenge for us, and we are hopeful for improvements in our operational capacity to better address these challenges in the future |
| CMA Do maternity care unit manager | Types of challenges faced during preterm births | n/a | person 1 | It's both a challenge and not. Firstly, the diagnosis of prematurity is sometimes inaccurately made, impacting the management of childbirth. If a premature birth, especially in a breech position, is not correctly diagnosed, the chosen method of delivery, like vaginal birth, could lead to complications, including stillbirth. Therefore, a cesarean section might be preferred. If the diagnosis is inaccurate, it affects the entire delivery process and the outcome for the premature baby. After birth, these babies are usually referred to the CHU (Central Hospital Unit) due to the specific and complex care they require, which is challenging at the CMA level. Additionally, family involvement and understanding are crucial in caring for premature babies. Our context often leans towards immediate newborn care practices like bathing, which might not be suitable for premature babies. They need careful monitoring and specific care to avoid any negative impacts. Thus, effectively supporting premature births is a challenge not just at our level but throughout the entire healthcare chain. | person 1 | 457 It's both a challenge and not. Firstly, the diagnosis of prematurity is sometimes inaccurately made, impacting the management of childbirth. If a premature birth, especially in a breech position, is not correctly diagnosed, the chosen method of delivery, like vaginal birth, could lead to complications, including stillbirth. Therefore, a cesarean section might be preferred. If the diagnosis is inaccurate, it affects the entire delivery process and the outcome for the premature baby. After birth, these babies are usually referred to the CHU (Central Hospital Unit) due to the specific and complex care they require, which is challenging at the CMA level. Additionally, family involvement and understanding are crucial in caring for premature babies. Our context often leans towards immediate newborn care practices like bathing, which might not be suitable for premature babies. They need careful monitoring and specific care to avoid any negative impacts. Thus, effectively supporting premature births is a challenge not just at our level but throughout the entire healthcare chain. |
| Colma1 focus group | Types of challenges faced during preterm births | n/a | person 3 | Yes, it certainly is. | person 3 | 458 Yes, it certainly is. |
| Colma1 focus group | Types of challenges faced during preterm births | n/a | person 2 | Premature birth presents significant challenges, especially in terms of the specialized care required. A primary issue is the lack of necessary equipment, like incubators, at our CSPS and even at the CMA level. We often have to transfer premature infants to CHURSS for adequate care. This lack of equipment makes providing appropriate support for these infants challenging. | person 2 | 459 Premature birth presents significant challenges, especially in terms of the specialized care required. A primary issue is the lack of necessary equipment, like incubators, at our CSPS and even at the CMA level. We often have to transfer premature infants to CHURSS for adequate care. This lack of equipment makes providing appropriate support for these infants challenging. |
| Colma1 focus group | Types of challenges faced during preterm births | n/a | person 2 | Yes, it's largely a matter of having the right equipment. | person 2 | 460 Yes, it's largely a matter of having the right equipment. |
| Colma1 focus group | Types of challenges faced during preterm births | n/a | person 2 | Definitely, it's a challenge. | person 2 | 461 Definitely, it's a challenge. |
| Colma1 focus group | Types of challenges faced during preterm births | n/a | person 5 | The challenge is also about the vitality of the fetus. Premature babies may lack certain reflexes at birth, which could endanger their lives. | person 5 | 462 The challenge is also about the vitality of the fetus. Premature babies may lack certain reflexes at birth, which could endanger their lives. |
| Colma1 focus group | Types of challenges faced during preterm births | n/a | person 3 | These infants are extremely fragile and must be protected from hypothermia, as it can quickly lead to life-threatening conditions | person 3 | 463 These infants are extremely fragile and must be protected from hypothermia, as it can quickly lead to life-threatening conditions |
| Colma1 focus group | Types of challenges faced during preterm births | n/a | person 5 | Respiratory distress is another common issue in premature births. When this occurs, we have to send them to CHURSS for specialized assistance. We also offer guidance to mothers of premature infants to help manage these challenges. | person 5 | 464 Respiratory distress is another common issue in premature births. When this occurs, we have to send them to CHURSS for specialized assistance. We also offer guidance to mothers of premature infants to help manage these challenges. |
| Colma1 focus group | Types of challenges faced during preterm births | n/a | person 7 | We emphasize several key points: breastfeeding, proper child care, and minimizing washing the baby too frequently. We also advise against allowing visitors to handle the baby indiscriminately. Overall, these tips focus on protecting the child, so it's crucial for the mother to take good care of her baby | person 7 | 465 We emphasize several key points: breastfeeding, proper child care, and minimizing washing the baby too frequently. We also advise against allowing visitors to handle the baby indiscriminately. Overall, these tips focus on protecting the child, so it's crucial for the mother to take good care of her baby |
| Colma1 major | Types of challenges faced during preterm births | n/a | person 1 | Premature birth, yes. Although the numbers might not be very high, it's still a critical issue. Each case of prematurity is significant as it involves a life at risk. Even if the statistics don't show a high frequency of such births, the impact and risks associated with each case make it a substantial challenge. It's important to recognize the gravity of each instance, as premature births have serious consequences for the infants. | person 1 | 466 Premature birth, yes. Although the numbers might not be very high, it's still a critical issue. Each case of prematurity is significant as it involves a life at risk. Even if the statistics don't show a high frequency of such births, the impact and risks associated with each case make it a substantial challenge. It's important to recognize the gravity of each instance, as premature births have serious consequences for the infants. |
| Colma1 major | Types of challenges faced during preterm births | n/a | person 1 | Yes, it does. In terms of pregn/acy, we cannot predict which woman will have a premature birth. When premature births occur, our facility lacks the necessary resources to provide adequate care for these infants. We have to refer them to larger healthcare facilities like the CMA of Dô or even CHURSS for specialized care. Our limitations in handling these cases mean that the challenges mostly arise due to our inability to provide necessary care within our facility. | person 1 | 467 Yes, it does. In terms of pregn/acy, we cannot predict which woman will have a premature birth. When premature births occur, our facility lacks the necessary resources to provide adequate care for these infants. We have to refer them to larger healthcare facilities like the CMA of Dô or even CHURSS for specialized care. Our limitations in handling these cases mean that the challenges mostly arise due to our inability to provide necessary care within our facility. |
| Colma1 maternity manager | Types of challenges faced during preterm births | n/a | person 1 | Yes, premature birth is indeed a major public health issue. It's a leading cause of perinatal morbidity and mortality. The technical resources required for the proper care of premature babies are not widely accessible, particularly in Burkina or Bobo-Dioulasso. The CHURSS is one of the few facilities that can somewhat manage these cases, but even there, the conditions and resources are not fully adequate | person 1 | 468 Yes, premature birth is indeed a major public health issue. It's a leading cause of perinatal morbidity and mortality. The technical resources required for the proper care of premature babies are not widely accessible, particularly in Burkina or Bobo-Dioulasso. The CHURSS is one of the few facilities that can somewhat manage these cases, but even there, the conditions and resources are not fully adequate |
| Colma1 maternity manager | Types of challenges faced during preterm births | n/a | person 1 | The main challenge is the inadequacy of support for premature births. Often, when a woman is in premature labor, she is referred to another facility, especially if the dilation reaches about 7 cm. For instance, a recent case involved a premature baby weighing 1,500 grams. In such situations, we face dilemmas in ensuring the baby's safe transport to a better-equipped facility. There's a risk of suffocating the baby while trying to keep it warm, the possibility of hypoglycemia developing during transport, and the concern of infection due to the baby's immature skin. Additionally, premature babies often have underdeveloped lungs, which complicates their care even further. | person 1 | 469 The main challenge is the inadequacy of support for premature births. Often, when a woman is in premature labor, she is referred to another facility, especially if the dilation reaches about 7 cm. For instance, a recent case involved a premature baby weighing 1,500 grams. In such situations, we face dilemmas in ensuring the baby's safe transport to a better-equipped facility. There's a risk of suffocating the baby while trying to keep it warm, the possibility of hypoglycemia developing during transport, and the concern of infection due to the baby's immature skin. Additionally, premature babies often have underdeveloped lungs, which complicates their care even further. |
| Farakan focus group | Types of challenges faced during preterm births | n/a | person 8 | Premature birth is not seen as a challenge per se because it's not necessarily the fault of the health worker or the healthcare system. It's a situation that can occur naturally. When it does happen, we make sure to act swiftly and ensure the child is in good condition for recovery. | person 8 | 470 Premature birth is not seen as a challenge per se because it's not necessarily the fault of the health worker or the healthcare system. It's a situation that can occur naturally. When it does happen, we make sure to act swiftly and ensure the child is in good condition for recovery. |
| Farakan focus group | Types of challenges faced during preterm births | n/a | person 5 | It's not a challenge in the conventional sense. Some women naturally have premature births, and while it's not desired, it's inevitable in some cases. Our role as health workers is to ensure the well-being of the baby when such situations arise, ensuring proper care and monitoring for optimal recovery and development. | person 5 | 471 It's not a challenge in the conventional sense. Some women naturally have premature births, and while it's not desired, it's inevitable in some cases. Our role as health workers is to ensure the well-being of the baby when such situations arise, ensuring proper care and monitoring for optimal recovery and development. |
| Farakan focus group | Types of challenges faced during preterm births | n/a | person 3 | It is a challenge for us. We've even established a kangaroo unit in our district, which is performing well and is one of the best in the city. We have protocols in place for premature deliveries, including lung maturation and possible evacuation of the mother for delivery in better-equipped facilities. In severe cases, we ensure immediate care of the newborn. We are also proactive in preventing premature births, despite some resource limitations. The kangaroo unit specifically caters to premature babies, providing specialized care. | person 3 | 472 It is a challenge for us. We've even established a kangaroo unit in our district, which is performing well and is one of the best in the city. We have protocols in place for premature deliveries, including lung maturation and possible evacuation of the mother for delivery in better-equipped facilities. In severe cases, we ensure immediate care of the newborn. We are also proactive in preventing premature births, despite some resource limitations. The kangaroo unit specifically caters to premature babies, providing specialized care. |
| Farakan focus group | Types of challenges faced during preterm births | n/a | person 3 | The kangaroo unit is a specialized facility for the care of premature babies. It's not located here but is part of our district healthcare services. | person 3 | 473 The kangaroo unit is a specialized facility for the care of premature babies. It's not located here but is part of our district healthcare services. |
| Farakan major | Types of challenges faced during preterm births | n/a | person 1 | Definitely, it's a problem. Premature babies require extensive care and attention to survive. Every human life is a priority, so we focus on caring for these babies to ensure their survival. | person 1 | 474 Definitely, it's a problem. Premature babies require extensive care and attention to survive. Every human life is a priority, so we focus on caring for these babies to ensure their survival. |
| Farakan major | Types of challenges faced during preterm births | n/a | person 1 | The challenge is present at all stages. During pregn/acy, it's crucial to monitor women closely to prevent premature births. Childbirth needs to be well-managed to ensure the health of the baby. And after birth, the newborn requires proper mainten/ace and care to survive. Each phase is critical in its own right. | person 1 | 475 The challenge is present at all stages. During pregn/acy, it's crucial to monitor women closely to prevent premature births. Childbirth needs to be well-managed to ensure the health of the baby. And after birth, the newborn requires proper mainten/ace and care to survive. Each phase is critical in its own right. |
| Farakan maternity manager | Types of challenges faced during preterm births | n/a | person 1 | Yes, childbirth is an important challenge. There was a project focused on caring for babies with low birth weight, which indicates the significance of this issue. | person 1 | 476 Yes, childbirth is an important challenge. There was a project focused on caring for babies with low birth weight, which indicates the significance of this issue. |
| Farakan maternity manager | Types of challenges faced during preterm births | n/a | person 1 | The primary challenge is prevention. To prevent premature births, interrogation, or thorough questioning, is vital. Many premature births are associated with infections and twin deliveries. Recently, infections have been the major cause | person 1 | 477 The primary challenge is prevention. To prevent premature births, interrogation, or thorough questioning, is vital. Many premature births are associated with infections and twin deliveries. Recently, infections have been the major cause |
| Farakan maternity manager | Types of challenges faced during preterm births | n/a | person 1 | Urinary infections are particularly common. | person 1 | 478 Urinary infections are particularly common. |
| Farakan maternity manager | Types of challenges faced during preterm births | n/a | person 1 | It's challenging to explain. Nobody wants a premature birth because it's tough for the parents and managing a premature baby, especially extremely premature ones, is difficult. Babies weighing around 2.1kg to 2.2kg can be managed more easily, but those around 1.5kg to 1.8kg are more challenging. Prevention is key to avoid such situations. Diseases like malaria can also lead to premature births. | person 1 | 479 It's challenging to explain. Nobody wants a premature birth because it's tough for the parents and managing a premature baby, especially extremely premature ones, is difficult. Babies weighing around 2.1kg to 2.2kg can be managed more easily, but those around 1.5kg to 1.8kg are more challenging. Prevention is key to avoid such situations. Diseases like malaria can also lead to premature births. |
| Accart-ville focus group | Staff skills and existence of equipment for better care | n/a | person 1 | At our facility, we usually manage full-term births. When a premature birth is imminent, we prefer transferring the patient to the CMA (Central Medical Association) due to our lack of equipment for such cases. | person 1 | 480 At our facility, we usually manage full-term births. When a premature birth is imminent, we prefer transferring the patient to the CMA (Central Medical Association) due to our lack of equipment for such cases. |
| Accart-ville focus group | Staff skills and existence of equipment for better care | n/a | person 3 | Regarding different types of prematurity, we evacuate cases of extreme prematurity to the CMA before labor begins for better care. For average prematurity, we try to manage it here before transferring to a neonatal unit. We face shortages in medications, such as vitamin K1 and aqueous eosin for cord care. Our facility also lacks incubators and separate rooms for premature babies, which is essential as they should be isolated from adults. Overall, we are lacking in both equipment and resources | person 3 | 481 Regarding different types of prematurity, we evacuate cases of extreme prematurity to the CMA before labor begins for better care. For average prematurity, we try to manage it here before transferring to a neonatal unit. We face shortages in medications, such as vitamin K1 and aqueous eosin for cord care. Our facility also lacks incubators and separate rooms for premature babies, which is essential as they should be isolated from adults. Overall, we are lacking in both equipment and resources |
| Accart-ville focus group | Staff skills and existence of equipment for better care | n/a | person 2 | Extreme prematurity refers to births occurring between the 28th and 32nd weeks of pregn/acy, while medium prematurity is classified as births occurring between the 33rd and 36th weeks and 6 days. | person 2 | 482 Extreme prematurity refers to births occurring between the 28th and 32nd weeks of pregn/acy, while medium prematurity is classified as births occurring between the 33rd and 36th weeks and 6 days. |
| Accart-ville CMU manager | Staff skills and existence of equipment for better care | n/a | person 1 | No, we don't have any of those resources. | person 1 | 483 No, we don't have any of those resources. |
| Accart-ville CMU manager | Staff skills and existence of equipment for better care | n/a | person 1 | As I mentioned earlier, we lack even the basic resuscitation equipment. We're extremely limited in our capabilities. Although we've been designated as a medical center, the necessary upgrades and resources haven't followed. We're doing the best we can with what we have. | person 1 | 484 As I mentioned earlier, we lack even the basic resuscitation equipment. We're extremely limited in our capabilities. Although we've been designated as a medical center, the necessary upgrades and resources haven't followed. We're doing the best we can with what we have. |
| Accart-ville maternity manager | Staff skills and existence of equipment for better care | n/a | person 1 | We do have trained staff, including eight midwives, five auxiliary birth attendants, an obstetric and gynecological health officer, and two doctors. When difficulties arise, we can call on the doctors for assistance. Our midwives are also trained in newborn care. However, our support capabilities are limited due to a lack of specific facilities and rooms for premature infants. Daily practice in managing premature births can enhance the practitioners' skills, and individual experiences in handling such cases contribute to the quality of care. The level of expertise varies; some have worked in well-equipped hospitals and are knowledgeable in premature newborn care, while others, mainly those who have worked at the peripheral level, may lack this experience | person 1 | 485 We do have trained staff, including eight midwives, five auxiliary birth attendants, an obstetric and gynecological health officer, and two doctors. When difficulties arise, we can call on the doctors for assistance. Our midwives are also trained in newborn care. However, our support capabilities are limited due to a lack of specific facilities and rooms for premature infants. Daily practice in managing premature births can enhance the practitioners' skills, and individual experiences in handling such cases contribute to the quality of care. The level of expertise varies; some have worked in well-equipped hospitals and are knowledgeable in premature newborn care, while others, mainly those who have worked at the peripheral level, may lack this experience |
| Accart-ville maternity manager | Staff skills and existence of equipment for better care | n/a | person 1 | Others have established health centers like hospitals, where there is equipment, which means that they have knowledge of the care of premature newborns, others have not done so, they have evolved in the CSPS, which means that at the peripheral level many have no idea about support. | person 1 | 486 Others have established health centers like hospitals, where there is equipment, which means that they have knowledge of the care of premature newborns, others have not done so, they have evolved in the CSPS, which means that at the peripheral level many have no idea about support. |
| Accart-ville maternity manager | Staff skills and existence of equipment for better care | n/a | person 1 | No, we typically evacuate cases of prematurity | person 1 | 487 No, we typically evacuate cases of prematurity |
| CMA Do focus group | Staff skills and existence of equipment for better care | n/a | person 6 | Unfortunately, we lack a technical platform for managing premature births. We make do with what we have | person 6 | 488 Unfortunately, we lack a technical platform for managing premature births. We make do with what we have |
| CMA Do focus group | Staff skills and existence of equipment for better care | n/a | person 9 | For healthy prematurity cases, we provide kangaroo mother care. However, pathological cases require hospitalization. | person 9 | 489 For healthy prematurity cases, we provide kangaroo mother care. However, pathological cases require hospitalization. |
| CMA Do focus group | Staff skills and existence of equipment for better care | n/a | person 5 | Our unit ensures the availability of qualified personnel, including pediatricians and midwives | person 5 | 490 Our unit ensures the availability of qualified personnel, including pediatricians and midwives |
| CMA Do focus group | Staff skills and existence of equipment for better care | n/a | person 7 | In such scenarios, we evacuate the cases to CHURSS's neonatology department for specialized care | person 7 | 491 In such scenarios, we evacuate the cases to CHURSS's neonatology department for specialized care |
| Gynecologist 1 | Staff skills and existence of equipment for better care | n/a | person 1 | For premature babies, unfortunately, no. We have a kangaroo mother care unit that does its best, but it's limited to healthy premature babies. Any baby with additional pathologies must be sent to the hospital. | person 1 | 492 For premature babies, unfortunately, no. We have a kangaroo mother care unit that does its best, but it's limited to healthy premature babies. Any baby with additional pathologies must be sent to the hospital. |
| Gynecologist 1 | Staff skills and existence of equipment for better care | n/a | person 1 | A healthy premature baby typically only has a birth weight issue, not yet reaching 2.5 kg. However, if they cry well, show good reactions, appear pink without infections or respiratory distress, we can care for them here. | person 1 | 493 A healthy premature baby typically only has a birth weight issue, not yet reaching 2.5 kg. However, if they cry well, show good reactions, appear pink without infections or respiratory distress, we can care for them here. |
| Gynecologist 1 | Staff skills and existence of equipment for better care | n/a | person 1 | Yes, we do sometimes have very premature babies. | person 1 | 494 Yes, we do sometimes have very premature babies. |
| Gynecologist 1 | Staff skills and existence of equipment for better care | n/a | person 1 | If the very premature babies, like those weighing around 1.2 or 1.3 kg, don't have associated problems, we can manage them here with the kangaroo mother care unit. | person 1 | 495 If the very premature babies, like those weighing around 1.2 or 1.3 kg, don't have associated problems, we can manage them here with the kangaroo mother care unit. |
| Gynecologist 1 | Staff skills and existence of equipment for better care | n/a | person 1 | We evacuate such cases to the hospital, specifically to the neonatology unit at the university hospital. | person 1 | 496 We evacuate such cases to the hospital, specifically to the neonatology unit at the university hospital. |
| Gynecologist 2 | Staff skills and existence of equipment for better care | n/a | person 1 | n/a | person 1 | 497 NA |
| MCD | Staff skills and existence of equipment for better care | n/a | person 1 | Our technical platforms are limited. We lack essential medical equipment, such as incubators, and we don't have a specialized neonatology department. Our Kangaroo Mother Care (SMK) service can manage babies who are near their due date and have low birth weights, but not the very premature ones. When babies reach a certain stage of prematurity, beyond our capacity, we must send them to the hospital. We're not equipped to handle very premature babies here at the CMA. | person 1 | 498 Our technical platforms are limited. We lack essential medical equipment, such as incubators, and we don't have a specialized neonatology department. Our Kangaroo Mother Care (SMK) service can manage babies who are near their due date and have low birth weights, but not the very premature ones. When babies reach a certain stage of prematurity, beyond our capacity, we must send them to the hospital. We're not equipped to handle very premature babies here at the CMA. |
| MCD | Staff skills and existence of equipment for better care | n/a | person 1 | I'm referring to those premature babies who, when they reach a certain weight, we can manage. If they are too premature or under a specific weight threshold, we cannot handle them and must refer them to the hospital for specialized care | person 1 | 499 I'm referring to those premature babies who, when they reach a certain weight, we can manage. If they are too premature or under a specific weight threshold, we cannot handle them and must refer them to the hospital for specialized care |
| CMA Do maternity care unit manager | Staff skills and existence of equipment for better care | n/a | person 1 | No, I would say no. The technical platform is not, in any case, sufficiently provided to be able to do this. Because even when we want to refer them there, there aren't too many conditions, we'll say, well, it's to wrap the child well, the new one, this premature baby, and that's it. And send it directly. So, that means that it can affect the transfer time and all that. So. So, we don't really have the necessary equipment to do it properly. | person 1 | 500 No, I would say no. The technical platform is not, in any case, sufficiently provided to be able to do this. Because even when we want to refer them there, there aren't too many conditions, we'll say, well, it's to wrap the child well, the new one, this premature baby, and that's it. And send it directly. So, that means that it can affect the transfer time and all that. So. So, we don't really have the necessary equipment to do it properly. |
| Colma1 focus group | Staff skills and existence of equipment for better care | n/a | person 6 | (laughs) We don't have any. Frankly, we don't have any; we make do with the means at hand. When he goes out we really try to protect him, keep him warm, and like, she said, advise his mom. In any case, if the reflexes are good, it is only the advice; otherwise, we have nothing else. Now if there is another problem, we refer it, that's all. | person 6 | 501 (laughs) We don't have any. Frankly, we don't have any; we make do with the means at hand. When he goes out we really try to protect him, keep him warm, and like, she said, advise his mom. In any case, if the reflexes are good, it is only the advice; otherwise, we have nothing else. Now if there is another problem, we refer it, that's all. |
| Colma1 focus group | Staff skills and existence of equipment for better care | n/a | person 5 | Yes, we had very premature babies here. In this case, we refer them. | person 5 | 502 Yes, we had very premature babies here. In this case, we refer them. |
| Colma1 focus group | Staff skills and existence of equipment for better care | n/a | person 1 | (laughs) As we told you, resources are limited here. It's to protect him, keep him warm, and call the ambulance for an evacuation. Usually, an hour after giving birth, he has already gone to the next level; we do not keep him here | person 1 | 503 (laughs) As we told you, resources are limited here. It's to protect him, keep him warm, and call the ambulance for an evacuation. Usually, an hour after giving birth, he has already gone to the next level; we do not keep him here |
| Colma1 major | Staff skills and existence of equipment for better care | n/a | person 1 | No, we don't have the technical platform needed. Our facility operates as a CSPS (Community Health Service Provider), and CSPS facilities are not equipped to handle premature babies. | person 1 | 504 No, we don't have the technical platform needed. Our facility operates as a CSPS (Community Health Service Provider), and CSPS facilities are not equipped to handle premature babies. |
| Colma1 major | Staff skills and existence of equipment for better care | n/a | person 1 | Yes, that's correct. When we encounter cases of premature births that we can't manage due to our limited resources, we refer or transfer them to facilities that have the necessary capabilities and equipment for their care. | person 1 | 505 Yes, that's correct. When we encounter cases of premature births that we can't manage due to our limited resources, we refer or transfer them to facilities that have the necessary capabilities and equipment for their care. |
| Colma1 maternity manager | Staff skills and existence of equipment for better care | n/a | person 1 | I told you, we don't have the technical platform. There are no technical platforms. | person 1 | 506 I told you, we don't have the technical platform. There are no technical platforms. |
| Colma1 maternity manager | Staff skills and existence of equipment for better care | n/a | person 1 | Extreme prematurity? Great prematurity? Yes, between 28 to 34 weeks of amenorrhea. Yes. | person 1 | 507 Extreme prematurity? Great prematurity? Yes, between 28 to 34 weeks of amenorrhea. Yes. |
| Farakan focus group | Staff skills and existence of equipment for better care | n/a | person 3 | No, we don't have any of the necessary equipment | person 3 | 508 No, we don't have any of the necessary equipment |
| Farakan focus group | Staff skills and existence of equipment for better care | n/a | person 1 | No, we lack the needed equipment. That's why we evacuate cases of premature births as soon as possible. However, in cases of force majeure, when immediate evacuation is required, we transport them right away | person 1 | 509 No, we lack the needed equipment. That's why we evacuate cases of premature births as soon as possible. However, in cases of force majeure, when immediate evacuation is required, we transport them right away |
| Farakan focus group | Staff skills and existence of equipment for better care | n/a | person 7 | Yes, we have had such cases. Often due to security issues, some women arrive late. Some might even give birth at home before being sent here. At times, a woman might reach full dilation before she can be evacuated, so we have to conduct the delivery here. We've indeed encountered many such instances. | person 7 | 510 Yes, we have had such cases. Often due to security issues, some women arrive late. Some might even give birth at home before being sent here. At times, a woman might reach full dilation before she can be evacuated, so we have to conduct the delivery here. We've indeed encountered many such instances. |
| Farakan major | Staff skills and existence of equipment for better care | n/a | person 1 | When we encounter cases of premature births, we refer them to a higher-level facility because we do not have the necessary technical support here | person 1 | 511 When we encounter cases of premature births, we refer them to a higher-level facility because we do not have the necessary technical support here |
| Farakan maternity manager | Staff skills and existence of equipment for better care | n/a | person 1 | To take care of a premature newborn, no. Premature newborns, if there are no problems, are taken care of at the CMA level which has a kangaroo unit | person 1 | 512 To take care of a premature newborn, no. Premature newborns, if there are no problems, are taken care of at the CMA level which has a kangaroo unit |
| Farakan maternity manager | Staff skills and existence of equipment for better care | n/a | person 1 | We refer. | person 1 | 513 We refer. |
| Farakan maternity manager | Staff skills and existence of equipment for better care | n/a | person 1 | We refer them to SOURO SANOU hospital. Extremely premature babies are evacuated to the hospital; the kangaroo unit at the CMA cannot care for premature babies with respiratory distress. Even cases of infections are evacuated to the CHURSS. Generally, the kangaroo unit takes care of healthy babies, there is no infection, and also weight matters, I think it's from 2 kg. | person 1 | 514 We refer them to SOURO SANOU hospital. Extremely premature babies are evacuated to the hospital; the kangaroo unit at the CMA cannot care for premature babies with respiratory distress. Even cases of infections are evacuated to the CHURSS. Generally, the kangaroo unit takes care of healthy babies, there is no infection, and also weight matters, I think it's from 2 kg. |
| Farakan maternity manager | Staff skills and existence of equipment for better care | n/a | person 1 | Yes. | person 1 | 515 Yes. |
| Farakan maternity manager | Staff skills and existence of equipment for better care | n/a | person 1 | Yes, there is the mother who is included since we will show her how to do it. | person 1 | 516 Yes, there is the mother who is included since we will show her how to do it. |
| Accart-ville focus group | Services and care provided to premature babies and their mothers | n/a | person 6 | When we have a premature birth, the mother receives the same care as other women. However, we take extra precautions to protect the baby from hypothermia. As we've mentioned earlier, due to our limited resources, we often need to transfer these cases to a higher-level facility for more specialized care. | person 6 | 517 When we have a premature birth, the mother receives the same care as other women. However, we take extra precautions to protect the baby from hypothermia. As we've mentioned earlier, due to our limited resources, we often need to transfer these cases to a higher-level facility for more specialized care. |
| Accart-ville CMU manager | Services and care provided to premature babies and their mothers | n/a | person 1 | Our initial response includes basic resuscitation efforts, like clearing airways and checking for any signs of distress. We use a saturometer to monitor oxygen levels, but our capabilities are quite restricted. For instance, setting up an umbilical route is critical for premature babies, but no one here, including myself, is trained for that procedure. | person 1 | 518 Our initial response includes basic resuscitation efforts, like clearing airways and checking for any signs of distress. We use a saturometer to monitor oxygen levels, but our capabilities are quite restricted. For instance, setting up an umbilical route is critical for premature babies, but no one here, including myself, is trained for that procedure. |
| Accart-ville maternity manager | Services and care provided to premature babies and their mothers | n/a | person 1 | If necessary, and if there's no space available for transfer, we keep the baby for at least 72 hours for monitoring. During this time, we focus on combating hypothermia and maintaining food hygiene and cleanliness. We also educate mothers about the fragility of these children and do our best under the circumstances. | person 1 | 519 If necessary, and if there's no space available for transfer, we keep the baby for at least 72 hours for monitoring. During this time, we focus on combating hypothermia and maintaining food hygiene and cleanliness. We also educate mothers about the fragility of these children and do our best under the circumstances. |
| CMA Do focus group | Services and care provided to premature babies and their mothers | n/a | person 6 | For mothers, we offer the same care as for normal deliveries. The care for premature infants, however, includes specific newborn care. This involves heating and other measures to prevent the baby from getting cold. In cases where the child experiences respiratory distress, intubation and oxygenation are necessary | person 6 | 520 For mothers, we offer the same care as for normal deliveries. The care for premature infants, however, includes specific newborn care. This involves heating and other measures to prevent the baby from getting cold. In cases where the child experiences respiratory distress, intubation and oxygenation are necessary |
| CMA Do focus group | Services and care provided to premature babies and their mothers | n/a | person 9 | We have some equipment, but it's not sufficient. There are heaters in the room and resuscitation equipment in the kangaroo unit, but it's inadequate for handling more than two or three babies | person 9 | 521 We have some equipment, but it's not sufficient. There are heaters in the room and resuscitation equipment in the kangaroo unit, but it's inadequate for handling more than two or three babies |
| CMA Do focus group | Services and care provided to premature babies and their mothers | n/a | person 6 | It seems that we have some equipment | person 6 | 522 It seems that we have some equipment |
| CMA Do focus group | Services and care provided to premature babies and their mothers | n/a | person 9 | Yes, we have the essential items like heaters and resuscitation equipment, but it's not enough for multiple cases simultaneously | person 9 | 523 Yes, we have the essential items like heaters and resuscitation equipment, but it's not enough for multiple cases simultaneously |
| Gynecologist 1 | Services and care provided to premature babies and their mothers | n/a | person 1 | For mothers, the care is standard postpartum care, no different from what we offer to all mothers. However, for premature infants, the focus is on thermal protection to prevent them from getting cold. Nutrition is also crucial. Some premature babies have a developed sucking reflex and can feed on their own, while others don't. In such cases, we use feeding tubes for force-feeding. We closely monitor these infants for weight gain and temperature regulation. | person 1 | 524 For mothers, the care is standard postpartum care, no different from what we offer to all mothers. However, for premature infants, the focus is on thermal protection to prevent them from getting cold. Nutrition is also crucial. Some premature babies have a developed sucking reflex and can feed on their own, while others don't. In such cases, we use feeding tubes for force-feeding. We closely monitor these infants for weight gain and temperature regulation. |
| Gynecologist 2 | Services and care provided to premature babies and their mothers | n/a | person 1 | The primary service we offer is guidance. Initially, we ensure the child is in a comfortable and warm environment, using warm clothes consistently. We teach these practices to the mother, and if present, the father, as well as other family members like grandparents or aunts accompanying the child. It's important that the child is kept warm at all times. Our kangaroo care unit is well-equipped for this. The method of carrying the baby, usually skin-to-skin on the chest and covered, is crucial. In cases like cesarean sections, where the mother can't carry the baby, another family member steps in. The baby, with a covered head and feet, is held skin-to-skin and wrapped in cloth. During feeding, if someone other than the mother is carrying the baby, we unwrap it for feeding by the mother. If the mother is carrying, the baby remains in position for continuous feeding. This approach ensures constant body warmth, continuous feeding, and warmth for the baby, significantly improving survival | person 1 | 525 The primary service we offer is guidance. Initially, we ensure the child is in a comfortable and warm environment, using warm clothes consistently. We teach these practices to the mother, and if present, the father, as well as other family members like grandparents or aunts accompanying the child. It's important that the child is kept warm at all times. Our kangaroo care unit is well-equipped for this. The method of carrying the baby, usually skin-to-skin on the chest and covered, is crucial. In cases like cesarean sections, where the mother can't carry the baby, another family member steps in. The baby, with a covered head and feet, is held skin-to-skin and wrapped in cloth. During feeding, if someone other than the mother is carrying the baby, we unwrap it for feeding by the mother. If the mother is carrying, the baby remains in position for continuous feeding. This approach ensures constant body warmth, continuous feeding, and warmth for the baby, significantly improving survival |
| Gynecologist 2 | Services and care provided to premature babies and their mothers | n/a | person 1 | For mothers, we focus on advice related to these practices, especially keeping the child warm and maintaining personal and clothing hygiene. The mother must be clean, with clean linen for covering the child, and she should eat well to ensure sufficient breast milk production. | person 1 | 526 For mothers, we focus on advice related to these practices, especially keeping the child warm and maintaining personal and clothing hygiene. The mother must be clean, with clean linen for covering the child, and she should eat well to ensure sufficient breast milk production. |
| MCD | Services and care provided to premature babies and their mothers | n/a | person 1 | Yes, the services primarily involve dietary advice for the child. We also offer guidance to the mother regarding her baby's nutrition. Additionally, we emphasize the importance of hygiene for both the mother and child, given the baby's fragility. We contribute by ensuring these aspects are taken care of. Our team includes experienced staff, including a pediatrician and midwives, who are dedicated to offering this specialized care | person 1 | 527 Yes, the services primarily involve dietary advice for the child. We also offer guidance to the mother regarding her baby's nutrition. Additionally, we emphasize the importance of hygiene for both the mother and child, given the baby's fragility. We contribute by ensuring these aspects are taken care of. Our team includes experienced staff, including a pediatrician and midwives, who are dedicated to offering this specialized care |
| CMA Do maternity care unit manager | Services and care provided to premature babies and their mothers | n/a | person 1 | To mothers, well, it's the same care that we offer to other women. Now, for premature newborns, it's true that we have the SMK unit which is there, sometimes this baby passes through the kangaroo unit, which perhaps offers first aid quickly, and we transferred. So. So, I don't want to say, there is no specific care that we offer in the delivery room. Maybe it's a good cover for this child. Give advice to the mother or caregivers on how to hold this newborn. So, above all, avoid him getting cold and all that. | person 1 | 528 To mothers, well, it's the same care that we offer to other women. Now, for premature newborns, it's true that we have the SMK unit which is there, sometimes this baby passes through the kangaroo unit, which perhaps offers first aid quickly, and we transferred. So. So, I don't want to say, there is no specific care that we offer in the delivery room. Maybe it's a good cover for this child. Give advice to the mother or caregivers on how to hold this newborn. So, above all, avoid him getting cold and all that. |
| Colma1 focus group | Services and care provided to premature babies and their mothers | n/a | person 5 | Hesitantly] Well, in terms of maternity care, we have quite a few... [laughs] | person 5 | 529 Hesitantly] Well, in terms of maternity care, we have quite a few... [laughs] |
| Colma1 focus group | Services and care provided to premature babies and their mothers | n/a | person 3 | Actually, we don't have specialized care for premature infants. After delivery, our priority is to protect the child. We don't delay because their life is critical. We start by weighing the baby to estimate its weight and then quickly evacuate it. We lack suitable medications, training, and technical equipment, so we don't keep the child here for long | person 3 | 530 Actually, we don't have specialized care for premature infants. After delivery, our priority is to protect the child. We don't delay because their life is critical. We start by weighing the baby to estimate its weight and then quickly evacuate it. We lack suitable medications, training, and technical equipment, so we don't keep the child here for long |
| Colma1 focus group | Services and care provided to premature babies and their mothers | n/a | person 3 | We evacuate them to CHURSS | person 3 | 531 We evacuate them to CHURSS |
| Colma1 major | Services and care provided to premature babies and their mothers | n/a | person 1 | The premature one? | person 1 | 532 The premature one? |
| Colma1 major | Services and care provided to premature babies and their mothers | n/a | person 1 | Right. Since we're in an urban environment, our primary approach is to refer them to a higher level of care. However, before that, we focus on essential immediate care. This includes thermal protection – we ensure the child is wrapped warmly and placed skin-to-skin with the mother to prevent exposure to cold. That's our general practice. Often, some premature infants struggle with proper sucking reflex, so they can't breastfeed immediately. In such cases, we give them a little glucose orally to stimulate sucking and prevent hypoglycemia. These are the primary services we provide here, but ultimately, we refer the child to more specialized facilities. | person 1 | 533 Right. Since we're in an urban environment, our primary approach is to refer them to a higher level of care. However, before that, we focus on essential immediate care. This includes thermal protection – we ensure the child is wrapped warmly and placed skin-to-skin with the mother to prevent exposure to cold. That's our general practice. Often, some premature infants struggle with proper sucking reflex, so they can't breastfeed immediately. In such cases, we give them a little glucose orally to stimulate sucking and prevent hypoglycemia. These are the primary services we provide here, but ultimately, we refer the child to more specialized facilities. |
| Colma1 maternity manager | Services and care provided to premature babies and their mothers | n/a | person 1 | For management. First, generally even at small dilation, we refer to it. But when that happens and we can't do it anymore, normally, it's Célestène that we should do even to the mother. I think it's 12 milligrams, right? And 12 hours later, we have to really do the lungs, but often, women who come in these cases, come, for example, to full dilation. Once it is extremely prematurity, we know that the care even at the CHURSS will be quite difficult, but we will put this child in the conditions with his mother to really evacuate urgently. But when it is a little prematurity, perhaps at 36 weeks, a few days and the reflexes are really good, especially the sucking reflex, perhaps we will really observe this child. We will perhaps try to help the mother with feeding, because the child needs to take something, especially take milk and then avoid hypoglycemia, really wrap him up. Give the necessary advice, really. But if we see that there is only a small problem, this child will be evacuated. Not referred, but evacuated. And we are going to ask not to bathe this child. The very bathing of children, in general, whether it is a full-term child, is up to 24 hours after delivery. This child, we will do everything not to wash him. Maybe it's cleaning the stools, in any case, wrapping it, especially the slippers and hats, it's at the first moment. As soon as we have a woman in the room, that's what we ask for. As we are next to the market, if it is not night, they come out to pay. | person 1 | 534 For management. First, generally even at small dilation, we refer to it. But when that happens and we can't do it anymore, normally, it's Célestène that we should do even to the mother. I think it's 12 milligrams, right? And 12 hours later, we have to really do the lungs, but often, women who come in these cases, come, for example, to full dilation. Once it is extremely prematurity, we know that the care even at the CHURSS will be quite difficult, but we will put this child in the conditions with his mother to really evacuate urgently. But when it is a little prematurity, perhaps at 36 weeks, a few days and the reflexes are really good, especially the sucking reflex, perhaps we will really observe this child. We will perhaps try to help the mother with feeding, because the child needs to take something, especially take milk and then avoid hypoglycemia, really wrap him up. Give the necessary advice, really. But if we see that there is only a small problem, this child will be evacuated. Not referred, but evacuated. And we are going to ask not to bathe this child. The very bathing of children, in general, whether it is a full-term child, is up to 24 hours after delivery. This child, we will do everything not to wash him. Maybe it's cleaning the stools, in any case, wrapping it, especially the slippers and hats, it's at the first moment. As soon as we have a woman in the room, that's what we ask for. As we are next to the market, if it is not night, they come out to pay. |
| Colma1 maternity manager | Services and care provided to premature babies and their mothers | n/a | person 1 | For example, reflexes mean that the child cannot speak. If the archaic reflexes are not there, especially if the suction is not there, can we do anything? And the child didn't cry. As I'm talking about the Apgar scores which are good, the Apgar scores are good, the reflexes are there, the child is able to breastfeed, for a small premature baby, that means the child himself, in any case , it's a few days, maybe two days that were missing for the child to really be full term, 37 weeks there. This child, surely, perhaps, if it goes well, we can keep it. The rest we send. | person 1 | 535 For example, reflexes mean that the child cannot speak. If the archaic reflexes are not there, especially if the suction is not there, can we do anything? And the child didn't cry. As I'm talking about the Apgar scores which are good, the Apgar scores are good, the reflexes are there, the child is able to breastfeed, for a small premature baby, that means the child himself, in any case , it's a few days, maybe two days that were missing for the child to really be full term, 37 weeks there. This child, surely, perhaps, if it goes well, we can keep it. The rest we send. |
| Colma1 maternity manager | Services and care provided to premature babies and their mothers | n/a | person 1 | To the mother, yes. Because when there is premature birth, there is a problem. The problem must be identified and taken care of. Many things can cause a woman to have a premature birth. First, the social and cultural problems are there. I start there. Perhaps a pregn/acy before, 18 years old or well over 35 years old. A single woman. So. And illegitimate pregn/acy. So. And unwanted pregn/acy. You see everything that is there. | person 1 | 536 To the mother, yes. Because when there is premature birth, there is a problem. The problem must be identified and taken care of. Many things can cause a woman to have a premature birth. First, the social and cultural problems are there. I start there. Perhaps a pregn/acy before, 18 years old or well over 35 years old. A single woman. So. And illegitimate pregn/acy. So. And unwanted pregn/acy. You see everything that is there. |
| Colma1 maternity manager | Services and care provided to premature babies and their mothers | n/a | person 1 | Okay, what am I going to say? Maybe it's someone who is at her aunt's house and the aunt's husband is going out with her. Nap ? It may not be direct. Maybe you took a girl from the village to help you. Ok. In the big distant family. And the girl comes to your house, that means, it's not normal for her to get pregn/at by your husband. Ok. That's it, she's here, that means, what am I going to say? And voluntary termination of pregn/acy according to the law is not applied. And there you have it, this child. All of this causes someone to give birth prematurely. That's how it is. We always take the maternal side. And local causes can be many things. There you go, it could be a uterine malformation. There you go, what else could it be? Fibroma. A fibroid can cause a woman to give birth prematurely. There's a lot in there. And again, infections, when we take infections in general, especially urinary infections, malaria, hysteriosis, there are many diseases that can lead to, even HIV, diabetes. What do I mean ? Many pathologies can lead a woman to give birth. So, you really have to do the questioning, really focus on this woman, know the problem and really help her to correct it. It can even lead to gaping. | person 1 | 537 Okay, what am I going to say? Maybe it's someone who is at her aunt's house and the aunt's husband is going out with her. Nap ? It may not be direct. Maybe you took a girl from the village to help you. Ok. In the big distant family. And the girl comes to your house, that means, it's not normal for her to get pregn/at by your husband. Ok. That's it, she's here, that means, what am I going to say? And voluntary termination of pregn/acy according to the law is not applied. And there you have it, this child. All of this causes someone to give birth prematurely. That's how it is. We always take the maternal side. And local causes can be many things. There you go, it could be a uterine malformation. There you go, what else could it be? Fibroma. A fibroid can cause a woman to give birth prematurely. There's a lot in there. And again, infections, when we take infections in general, especially urinary infections, malaria, hysteriosis, there are many diseases that can lead to, even HIV, diabetes. What do I mean ? Many pathologies can lead a woman to give birth. So, you really have to do the questioning, really focus on this woman, know the problem and really help her to correct it. It can even lead to gaping. |
| Farakan focus group | Services and care provided to premature babies and their mothers | n/a | person 3 | For the infants, our primary actions include immediate assessment and a warm, dry wrap. We also administer Vitamin K1. That's essentially all we do for the newborn. As for the mothers, they receive the standard care given to all women in our facility. We place a strong emphasis on skin-to-skin contact, which is especially important during transport for evacuation | person 3 | 538 For the infants, our primary actions include immediate assessment and a warm, dry wrap. We also administer Vitamin K1. That's essentially all we do for the newborn. As for the mothers, they receive the standard care given to all women in our facility. We place a strong emphasis on skin-to-skin contact, which is especially important during transport for evacuation |
| Farakan major | Services and care provided to premature babies and their mothers | n/a | person 1 | In our facility, after providing first aid, we generally refer the premature baby to a higher level of care. The mother stays here under our surveillance until it's appropriate for her to leave | person 1 | 539 In our facility, after providing first aid, we generally refer the premature baby to a higher level of care. The mother stays here under our surveillance until it's appropriate for her to leave |
| Farakan major | Services and care provided to premature babies and their mothers | n/a | person 1 | For the newborn, we start by cleaning and warming them up. If the baby is capable of breastfeeding, we initiate that process. Meanwhile, we prepare for the baby's transfer to a facility with more specialized care | person 1 | 540 For the newborn, we start by cleaning and warming them up. If the baby is capable of breastfeeding, we initiate that process. Meanwhile, we prepare for the baby's transfer to a facility with more specialized care |
| Farakan maternity manager | Services and care provided to premature babies and their mothers | n/a | person 1 | After birth, generally speaking, we don't take care of it. If it is a case of extreme prematurity, we evacuate because at home here we cannot provide care. On the other hand, there are premature babies weighing less than 2 kg 500 (2 kg 200 or 2 kg 100) when we see, they are fine. These are children with reflexes, and these children we can keep them and advise the mother on their care, not expose them to the wind, this child must be well protected. Now below 2 kg, with respiratory distress, we cannot manage these cases here. | person 1 | 541 After birth, generally speaking, we don't take care of it. If it is a case of extreme prematurity, we evacuate because at home here we cannot provide care. On the other hand, there are premature babies weighing less than 2 kg 500 (2 kg 200 or 2 kg 100) when we see, they are fine. These are children with reflexes, and these children we can keep them and advise the mother on their care, not expose them to the wind, this child must be well protected. Now below 2 kg, with respiratory distress, we cannot manage these cases here. |
| Farakan maternity manager | Services and care provided to premature babies and their mothers | n/a | person 1 | In any case, those under 1 kg 800, even 1 kg 800 are already very premature. There are even premature babies weighing 1 kg. We happened to have twins weighing 800g and 900g and we referred them to the hospital, and it wasn't easy. I think there was one who died afterwards, the one who stayed, until she was 2 years old, she couldn't sit up, so it was really a burden for the parents | person 1 | 542 In any case, those under 1 kg 800, even 1 kg 800 are already very premature. There are even premature babies weighing 1 kg. We happened to have twins weighing 800g and 900g and we referred them to the hospital, and it wasn't easy. I think there was one who died afterwards, the one who stayed, until she was 2 years old, she couldn't sit up, so it was really a burden for the parents |
| Accart-ville focus group | Health risks in premature infants | n/a | person 7 | I believe that infections are a major risk for many premature babies. Without proper care, they are highly vulnerable to diseases, and unfortunately, many do not survive. | person 7 | 543 I believe that infections are a major risk for many premature babies. Without proper care, they are highly vulnerable to diseases, and unfortunately, many do not survive. |
| Accart-ville focus group | Health risks in premature infants | n/a | person 4 | Premature babies can be considered immunocompromised as their bodies aren't as developed as those of full-term children. Additionally, some are unable to effectively breastfeed, missing out on the antibodies in breast milk. This further weakens their immune system, making them susceptible to opportunistic diseases. | person 4 | 544 Premature babies can be considered immunocompromised as their bodies aren't as developed as those of full-term children. Additionally, some are unable to effectively breastfeed, missing out on the antibodies in breast milk. This further weakens their immune system, making them susceptible to opportunistic diseases. |
| Accart-ville focus group | Health risks in premature infants | n/a | person 3 | Another aspect to consider are the deficits associated with prematurity, like weight and possibly deficits affecting the sense organs. There's also the issue of stigmatization. Premature children are often given specific names in different communities, signifying their premature birth. This stigmatization, along with a general perception of their lack of viability, can negatively impact their care. | person 3 | 545 Another aspect to consider are the deficits associated with prematurity, like weight and possibly deficits affecting the sense organs. There's also the issue of stigmatization. Premature children are often given specific names in different communities, signifying their premature birth. This stigmatization, along with a general perception of their lack of viability, can negatively impact their care. |
| Accart-ville focus group | Health risks in premature infants | n/a | person 2 | The names vary by community. In Bobo, the name might be different, but on the Mossi plateau, they have a specific name, which I can't recall right now. These names are based on their term of birth. | person 2 | 546 The names vary by community. In Bobo, the name might be different, but on the Mossi plateau, they have a specific name, which I can't recall right now. These names are based on their term of birth. |
| Accart-ville focus group | Health risks in premature infants | n/a | person 1 | Returning to the topic of infections, respiratory infections are also common in premature babies. Their bronchi are underdeveloped, making them prone to respiratory problems. | person 1 | 547 Returning to the topic of infections, respiratory infections are also common in premature babies. Their bronchi are underdeveloped, making them prone to respiratory problems. |
| Accart-ville focus group | Health risks in premature infants | n/a | person 7 | I tell myself that it's the infections because that's what affects many premature babies. If premature babies are not well cared for, it opens the doors to diseases and usually the children do not survive. | person 7 | 548 I tell myself that it's the infections because that's what affects many premature babies. If premature babies are not well cared for, it opens the doors to diseases and usually the children do not survive. |
| Accart-ville focus group | Health risks in premature infants | n/a | person 4 | Premature babies can be considered immunocompromised since their bodies are not like those of children who are born full term. In addition, there are others who cannot even express breast milk well, so they do not benefit from the antibodies in breast milk, this weakens them and they are not immune. So this opens the doors to opportunistic diseases. | person 4 | 549 Premature babies can be considered immunocompromised since their bodies are not like those of children who are born full term. In addition, there are others who cannot even express breast milk well, so they do not benefit from the antibodies in breast milk, this weakens them and they are not immune. So this opens the doors to opportunistic diseases. |
| Accart-ville focus group | Health risks in premature infants | n/a | person 3 | What we could also add are the deficits which are linked to prematurity, he can have a height and weight deficit, he can also have a deficit which can affect the sense organs, we can also have stigmatization because these children can be stigmatized and they (premature children) have a name even depending on the community to show that they have not come to term. There are also perceptions regarding their viability, we generally say in our communities that these are not children who are viable and that the child born in the 8th month will not be able to live. All of these aspects can be negative factors in the care of premature infants. | person 3 | 550 What we could also add are the deficits which are linked to prematurity, he can have a height and weight deficit, he can also have a deficit which can affect the sense organs, we can also have stigmatization because these children can be stigmatized and they (premature children) have a name even depending on the community to show that they have not come to term. There are also perceptions regarding their viability, we generally say in our communities that these are not children who are viable and that the child born in the 8th month will not be able to live. All of these aspects can be negative factors in the care of premature infants. |
| Accart-ville focus group | Health risks in premature infants | n/a | person 2 | The names vary depending on the community, in Bobo here I don't know, but in the Mossi plateau they have a name that I don't remember, but I know that there are names that we attribute to them based on their term of birth, perhaps we could come back to that later. | person 2 | 551 The names vary depending on the community, in Bobo here I don't know, but in the Mossi plateau they have a name that I don't remember, but I know that there are names that we attribute to them based on their term of birth, perhaps we could come back to that later. |
| Accart-ville focus group | Health risks in premature infants | n/a | person 1 | This is to come back to infections, we can also add respiratory infections, which are also noticed in premature babies since the bronchi are not well developed, so premature babies tend to have breathing problems | person 1 | 552 This is to come back to infections, we can also add respiratory infections, which are also noticed in premature babies since the bronchi are not well developed, so premature babies tend to have breathing problems |
| Accart-ville focus group | Health risks in premature infants | n/a | person 2 | Of course, these burdens affect the health of the child because when they are told to take the child to the hospital at the higher level, they refuse since they think that the child is not going to live and they are going to waste the money. Also those who are nearby will tell the story elsewhere, and when the mother returns to the family with the child, people out of curiosity want to come and see the child. So, we tell the parents to keep the child in the room and not to leave the child within reach of everyone, that is to say people who come to see since it is just to come get news and go tell. So for this reason, we advise parents to leave the child in the room and not take them out. Then, we make an appointment with the mother so that she can bring the child back to us every two weeks for weighing and administering the vaccine, because the weight is not normal when it is not a question of 'a very premature baby. So, we make appointments with the mother frequently to see how the child is progressing | person 2 | 553 Of course, these burdens affect the health of the child because when they are told to take the child to the hospital at the higher level, they refuse since they think that the child is not going to live and they are going to waste the money. Also those who are nearby will tell the story elsewhere, and when the mother returns to the family with the child, people out of curiosity want to come and see the child. So, we tell the parents to keep the child in the room and not to leave the child within reach of everyone, that is to say people who come to see since it is just to come get news and go tell. So for this reason, we advise parents to leave the child in the room and not take them out. Then, we make an appointment with the mother so that she can bring the child back to us every two weeks for weighing and administering the vaccine, because the weight is not normal when it is not a question of 'a very premature baby. So, we make appointments with the mother frequently to see how the child is progressing |
| Accart-ville CMU manager | Health risks in premature infants | n/a | person 1 | Health issues related to prematurity are significant. Premature infants are inherently fragile and are therefore highly susceptible to various infections, particularly neonatal infections. They also face nutritional challenges due to their underdeveloped state. Respiratory issues are common as well, given that their lungs are not fully developed. Moreover, caring for a premature infant presents an economic burden. If a child needs to be treated at Souro SANOU hospital, for example, parents often have to leave work to be with their newborn, which adds to the challenges. | person 1 | 554 Health issues related to prematurity are significant. Premature infants are inherently fragile and are therefore highly susceptible to various infections, particularly neonatal infections. They also face nutritional challenges due to their underdeveloped state. Respiratory issues are common as well, given that their lungs are not fully developed. Moreover, caring for a premature infant presents an economic burden. If a child needs to be treated at Souro SANOU hospital, for example, parents often have to leave work to be with their newborn, which adds to the challenges. |
| Accart-ville CMU manager | Health risks in premature infants | n/a | person 1 | Primarily, we're talking about neonatal infections. This includes lung infections, among others. Sometimes, the prematurity itself might be caused by an infection in the mother, like a genital infection that disrupts the pregn/acy. These infections can lead to bacteremia, where bacteria enter the baby through the birth canal, causing premature birth. Being fragile, the child is also at risk of acquiring circulating infections in the community if not adequately protected. | person 1 | 555 Primarily, we're talking about neonatal infections. This includes lung infections, among others. Sometimes, the prematurity itself might be caused by an infection in the mother, like a genital infection that disrupts the pregn/acy. These infections can lead to bacteremia, where bacteria enter the baby through the birth canal, causing premature birth. Being fragile, the child is also at risk of acquiring circulating infections in the community if not adequately protected. |
| Accart-ville CMU manager | Health risks in premature infants | n/a | person 1 | Yes, it is actually a mortality factor. So yes, in newborns first. It is also a psychological factor for the mother. Being able to endure a pregn/acy for a long time and then losing the child during childbirth is actually something that is not easy. It can affect the mother's mind. | person 1 | 556 Yes, it is actually a mortality factor. So yes, in newborns first. It is also a psychological factor for the mother. Being able to endure a pregn/acy for a long time and then losing the child during childbirth is actually something that is not easy. It can affect the mother's mind. |
| Accart-ville CMU manager | Health risks in premature infants | n/a | person 1 | There are cases where childbirth leads to psychological disorders, what was previously known as puerperal psychoses. This occurs after giving birth. Some women experience a significant change in their mental state. It's like they enter a state of delirium. These changes can significantly affect their emotional well-being and mental health. | person 1 | 557 There are cases where childbirth leads to psychological disorders, what was previously known as puerperal psychoses. This occurs after giving birth. Some women experience a significant change in their mental state. It's like they enter a state of delirium. These changes can significantly affect their emotional well-being and mental health. |
| Accart-ville maternity manager | Health risks in premature infants | n/a | person 1 | The primary health risks for these infants are infections, often linked to the living conditions of their mothers. In crowded living spaces, the spread of germs is common. Additionally, food hygiene is a concern. Many people have less than three meals a day. If a mother is malnourished, it's likely that her child will also face nutritional issues due to insufficient breast milk. These are the common problems we encounter: nutrition and hygiene, both of which are significant sources of infections. Another issue is the unsuitability of housing when the mother returns to the community. She often shares her living space with others and can't impose necessary measures to combat hypothermia, such as closing doors and windows. We often hear about conflicts arising when a mother tries to take these precautions against hypothermia and infection, but faces objections from others in the house. In such conditions, it becomes challenging to effectively protect against these risks. | person 1 | 558 The primary health risks for these infants are infections, often linked to the living conditions of their mothers. In crowded living spaces, the spread of germs is common. Additionally, food hygiene is a concern. Many people have less than three meals a day. If a mother is malnourished, it's likely that her child will also face nutritional issues due to insufficient breast milk. These are the common problems we encounter: nutrition and hygiene, both of which are significant sources of infections. Another issue is the unsuitability of housing when the mother returns to the community. She often shares her living space with others and can't impose necessary measures to combat hypothermia, such as closing doors and windows. We often hear about conflicts arising when a mother tries to take these precautions against hypothermia and infection, but faces objections from others in the house. In such conditions, it becomes challenging to effectively protect against these risks. |
| Accart-ville maternity manager | Health risks in premature infants | n/a | person 1 | Indeed, it has an influence on mortality. Hypothermia is a cause of mortality, and infection is also one of the top five causes of neonatal mortality. Given the fragility of the newborn, infection is an important factor in neonatal death in general. | person 1 | 559 Indeed, it has an influence on mortality. Hypothermia is a cause of mortality, and infection is also one of the top five causes of neonatal mortality. Given the fragility of the newborn, infection is an important factor in neonatal death in general. |
| CMA Do focus group | Health risks in premature infants | n/a | person 6 | The primary risk is neonatal infections | person 6 | 560 The primary risk is neonatal infections |
| CMA Do focus group | Health risks in premature infants | n/a | person 9 | We previously discussed neonatal mortality, which is a significant concern. | person 9 | 561 We previously discussed neonatal mortality, which is a significant concern. |
| CMA Do focus group | Health risks in premature infants | n/a | person 5 | Thermal protection is also crucial. The clothing for these infants, including hats and socks, is essential but not always affordable. Since these items aren't free, the cost can be a barrier, making it challenging to adequately protect these babies from the cold. | person 5 | 562 Thermal protection is also crucial. The clothing for these infants, including hats and socks, is essential but not always affordable. Since these items aren't free, the cost can be a barrier, making it challenging to adequately protect these babies from the cold. |
| Gynecologist 1 | Health risks in premature infants | n/a | person 1 | The main risk for these infants is infection. | person 1 | 563 The main risk for these infants is infection. |
| Gynecologist 1 | Health risks in premature infants | n/a | person 1 | Yes, it does. The risk of infection has a considerable impact on mortality. | person 1 | 564 Yes, it does. The risk of infection has a considerable impact on mortality. |
| Gynecologist 2 | Health risks in premature infants | n/a | person 1 | The primary risk for these children is hypothermia. They are exposed to cold environments which can often be fatal. After hypothermia, the next major risk is infection. In our hospital system, where hygiene standards may not always be optimal, children can easily become infected, either from the external environment or within the hospital setting itself. This is why we emphasize hygiene-related advice. For example, if a premature child is infected by a foodborne germ and develops diarrhea, this further deteriorates their health. We give similar advice to mothers, focusing on maintaining sufficient hygiene for the child, ensuring proper feeding, and keeping them warm. Concerning the mothers, prematurity is often linked to a health or social issue. If an illness caused premature birth, the mother will need care based on the specific cause. In cases where no cause is identified, we advise the mother on how to care for her child effectively. | person 1 | 565 The primary risk for these children is hypothermia. They are exposed to cold environments which can often be fatal. After hypothermia, the next major risk is infection. In our hospital system, where hygiene standards may not always be optimal, children can easily become infected, either from the external environment or within the hospital setting itself. This is why we emphasize hygiene-related advice. For example, if a premature child is infected by a foodborne germ and develops diarrhea, this further deteriorates their health. We give similar advice to mothers, focusing on maintaining sufficient hygiene for the child, ensuring proper feeding, and keeping them warm. Concerning the mothers, prematurity is often linked to a health or social issue. If an illness caused premature birth, the mother will need care based on the specific cause. In cases where no cause is identified, we advise the mother on how to care for her child effectively. |
| Gynecologist 2 | Health risks in premature infants | n/a | person 1 | We're mostly dealing with bacterial infections. Often in the premature babies we see, the condition is linked to premature rupture of membranes. This means the pregn/acy was progressing, but then the amniotic sac broke prematurely, which is frequently associated with bacterial infections in the mother's genital area. After the sac ruptures, and the mother is hospitalized, we conduct tests to identify the infection. We try to maintain the pregn/acy as long as possible until delivery is unavoidable, which might result in either a vaginal birth or a cesarean section. Typically, these are bacterial infections. | person 1 | 566 We're mostly dealing with bacterial infections. Often in the premature babies we see, the condition is linked to premature rupture of membranes. This means the pregn/acy was progressing, but then the amniotic sac broke prematurely, which is frequently associated with bacterial infections in the mother's genital area. After the sac ruptures, and the mother is hospitalized, we conduct tests to identify the infection. We try to maintain the pregn/acy as long as possible until delivery is unavoidable, which might result in either a vaginal birth or a cesarean section. Typically, these are bacterial infections. |
| Gynecologist 2 | Health risks in premature infants | n/a | person 1 | Of course. If, for example, it was a bacterial infection that caused premature rupture of the membranes, the same infection can spread in the amniotic fluids and then infect the child even before birth. So the child will be born with an infectious problem that will have to be taken care of while he is there. And that can worsen his state of survival. | person 1 | 567 Of course. If, for example, it was a bacterial infection that caused premature rupture of the membranes, the same infection can spread in the amniotic fluids and then infect the child even before birth. So the child will be born with an infectious problem that will have to be taken care of while he is there. And that can worsen his state of survival. |
| MCD | Health risks in premature infants | n/a | person 1 | A child born even at full term is fragile, even more so than a child who is not born at full term. So, the risk is mortality, linked to infections. They can have these infections, and as they are not sufficiently well prepared, this can happen if the follow-up is not really optimal. | person 1 | 568 A child born even at full term is fragile, even more so than a child who is not born at full term. So, the risk is mortality, linked to infections. They can have these infections, and as they are not sufficiently well prepared, this can happen if the follow-up is not really optimal. |
| CMA Do maternity care unit manager | Health risks in premature infants | n/a | person 1 | Health risks... Could you clarify? | person 1 | 569 Health risks... Could you clarify? |
| CMA Do maternity care unit manager | Health risks in premature infants | n/a | person 1 | Yes, the primary risk is infections. Premature infants have a weaker immune system, making them more susceptible to infectious diseases. It's primarily infections that are the concern. | person 1 | 570 Yes, the primary risk is infections. Premature infants have a weaker immune system, making them more susceptible to infectious diseases. It's primarily infections that are the concern. |
| CMA Do maternity care unit manager | Health risks in premature infants | n/a | person 1 | When we talk about infections, I mean things like neonatal infections. As for specific examples, although it's not exactly my area of expertise, we can include conditions like malaria and bronchopulmonary infections. | person 1 | 571 When we talk about infections, I mean things like neonatal infections. As for specific examples, although it's not exactly my area of expertise, we can include conditions like malaria and bronchopulmonary infections. |
| CMA Do maternity care unit manager | Health risks in premature infants | n/a | person 1 | Yes, they can indeed affect mortality. In discussing pathology, it's important to note that premature newborns can also experience hypoglycemia. This condition, hypoglycemia, can be life-threatening for these newborns. So, that's another critical aspect to consider. | person 1 | 572 Yes, they can indeed affect mortality. In discussing pathology, it's important to note that premature newborns can also experience hypoglycemia. This condition, hypoglycemia, can be life-threatening for these newborns. So, that's another critical aspect to consider. |
| Colma1 focus group | Health risks in premature infants | n/a | person 2 | The main health risks for premature infants generally include hypothermia; it's crucial to keep them warm. A premature child, being more vulnerable than a full-term infant, is significantly more exposed to infections. It's important to prevent infections in these infants before considering their evacuation for further care. Are there other risks to consider? | person 2 | 573 The main health risks for premature infants generally include hypothermia; it's crucial to keep them warm. A premature child, being more vulnerable than a full-term infant, is significantly more exposed to infections. It's important to prevent infections in these infants before considering their evacuation for further care. Are there other risks to consider? |
| Colma1 focus group | Health risks in premature infants | n/a | person 3 | Malnutrition is another major concern. Premature infants often lack the sucking reflex necessary for feeding and require special attention in this regard. Additionally, they are susceptible to respiratory problems. | person 3 | 574 Malnutrition is another major concern. Premature infants often lack the sucking reflex necessary for feeding and require special attention in this regard. Additionally, they are susceptible to respiratory problems. |
| Colma1 focus group | Health risks in premature infants | n/a | person 3 | Absolutely, they do. A premature baby who is malnourished or has a respiratory issue that isn't adequately addressed is at a significant risk of mortality. | person 3 | 575 Absolutely, they do. A premature baby who is malnourished or has a respiratory issue that isn't adequately addressed is at a significant risk of mortality. |
| Colma1 major | Health risks in premature infants | n/a | person 1 | Are you referring to preemies? | person 1 | 576 Are you referring to preemies? |
| Colma1 major | Health risks in premature infants | n/a | person 1 | Right, as I mentioned earlier, one major concern is their underdeveloped thermoregulation, leading to a risk of hypothermia. Premature babies are particularly vulnerable to both hypothermia and hypoglycemia. These conditions can arise quickly, especially if they are unable to feed properly. Without immediate care, these risks can indeed be life-threatening | person 1 | 577 Right, as I mentioned earlier, one major concern is their underdeveloped thermoregulation, leading to a risk of hypothermia. Premature babies are particularly vulnerable to both hypothermia and hypoglycemia. These conditions can arise quickly, especially if they are unable to feed properly. Without immediate care, these risks can indeed be life-threatening |
| Colma1 major | Health risks in premature infants | n/a | person 1 | Exactly, that's precisely it. | person 1 | 578 Exactly, that's precisely it. |
| Colma1 maternity manager | Health risks in premature infants | n/a | person 1 | The main risks are morbidity and mortality. | person 1 | 579 The main risks are morbidity and mortality. |
| Colma1 maternity manager | Health risks in premature infants | n/a | person 1 | Certainly. Consider a premature baby, for instance. Even a healthy child is at risk if certain crucial steps aren't taken immediately after birth. One significant risk is infection due to their immature skin. Then there's hypoglycemia. These factors are critical in the survival of a premature infant. I should also mention hypothermia, particularly in newborns and preemies. In these babies, every system, starting from the head, is fragile and underdeveloped, making their care complex and delicate. | person 1 | 580 Certainly. Consider a premature baby, for instance. Even a healthy child is at risk if certain crucial steps aren't taken immediately after birth. One significant risk is infection due to their immature skin. Then there's hypoglycemia. These factors are critical in the survival of a premature infant. I should also mention hypothermia, particularly in newborns and preemies. In these babies, every system, starting from the head, is fragile and underdeveloped, making their care complex and delicate. |
| Colma1 maternity manager | Health risks in premature infants | n/a | person 1 | That's correct | person 1 | 581 That's correct |
| Farakan focus group | Health risks in premature infants | n/a | person 3 | A major issue is the lack of adequate support and transportation. Often, premature infants need to be referred to specialized facilities, but due to the absence of ambulances, their parents must use personal means to transport them, which is a significant problem. Additionally, there's the issue of inadequate clothing for the newborns, like using nylon fabric, which isn't suitable for wrapping them properly | person 3 | 582 A major issue is the lack of adequate support and transportation. Often, premature infants need to be referred to specialized facilities, but due to the absence of ambulances, their parents must use personal means to transport them, which is a significant problem. Additionally, there's the issue of inadequate clothing for the newborns, like using nylon fabric, which isn't suitable for wrapping them properly |
| Farakan focus group | Health risks in premature infants | n/a | person 7 | Premature babies are particularly prone to respiratory diseases, especially pulmonary issues, and neonatal infections | person 7 | 583 Premature babies are particularly prone to respiratory diseases, especially pulmonary issues, and neonatal infections |
| Farakan focus group | Health risks in premature infants | n/a | person 8 | There's also a risk of anemia, both in premature children and their mothers, which can be quite serious. | person 8 | 584 There's also a risk of anemia, both in premature children and their mothers, which can be quite serious. |
| Farakan focus group | Health risks in premature infants | n/a | person 4 | Malnutrition is another concern. If a child is unable to feed properly, it can severely impact their health. | person 4 | 585 Malnutrition is another concern. If a child is unable to feed properly, it can severely impact their health. |
| Farakan focus group | Health risks in premature infants | n/a | person 3 | I don't have extensive knowledge about specific illnesses, as we're not a treatment center. However, from what I've seen in medical records, symptoms like febrile jaundice are quite common among surviving premature infants | person 3 | 586 I don't have extensive knowledge about specific illnesses, as we're not a treatment center. However, from what I've seen in medical records, symptoms like febrile jaundice are quite common among surviving premature infants |
| Farakan focus group | Health risks in premature infants | n/a | person 6 | Additionally, malaria can be a contributing factor to prematurity. If a mother has malaria, the child can be born with it, further complicating their health. | person 6 | 587 Additionally, malaria can be a contributing factor to prematurity. If a mother has malaria, the child can be born with it, further complicating their health. |
| Farakan major | Health risks in premature infants | n/a | person 1 | Premature infants are extremely vulnerable. They require careful attention to prevent infections and protect them from catching colds. Proper nutrition is also vital for their growth | person 1 | 588 Premature infants are extremely vulnerable. They require careful attention to prevent infections and protect them from catching colds. Proper nutrition is also vital for their growth |
| Farakan major | Health risks in premature infants | n/a | person 1 | Yes, the risk is significant. Without proper care, their chances of survival decrease. Infections and susceptibility to colds can be particularly harmful for them. | person 1 | 589 Yes, the risk is significant. Without proper care, their chances of survival decrease. Infections and susceptibility to colds can be particularly harmful for them. |
| Farakan maternity manager | Health risks in premature infants | n/a | person 1 | The mortality rate for premature infants is significantly higher. | person 1 | 590 The mortality rate for premature infants is significantly higher. |
| Farakan maternity manager | Health risks in premature infants | n/a | person 1 | Yes, premature children are more fragile compared to full-term infants, making them more prone to infections | person 1 | 591 Yes, premature children are more fragile compared to full-term infants, making them more prone to infections |
| Farakan maternity manager | Health risks in premature infants | n/a | person 1 | Respiratory infections are a common risk for these infants. | person 1 | 592 Respiratory infections are a common risk for these infants. |
| Accart-ville focus group | Specificities of mothers of premature children | n/a | person 3 | What we often see is a link to poverty. Prematurity is more common in households with lower incomes or fin/acial instability. While there are exceptions, the majority of premature births occur in areas where poverty is more prevalent | person 3 | 593 What we often see is a link to poverty. Prematurity is more common in households with lower incomes or fin/acial instability. While there are exceptions, the majority of premature births occur in areas where poverty is more prevalent |
| Accart-ville focus group | Specificities of mothers of premature children | n/a | person 3 | Another factor is the living environment, including issues like poor hygiene, crowded living conditions, and the mother's health status, especially malnutrition. So, we observe that women giving birth to premature babies often come from very low socio-economic backgrounds. | person 3 | 594 Another factor is the living environment, including issues like poor hygiene, crowded living conditions, and the mother's health status, especially malnutrition. So, we observe that women giving birth to premature babies often come from very low socio-economic backgrounds. |
| Accart-ville focus group | Specificities of mothers of premature children | n/a | person 1 | Regarding age, we notice that primiparas, or first-time mothers, tend to have more premature births compared to multiparas, or mothers who have had multiple births. Also, women under 18 years old have a higher incidence of premature births compared to those over 18. | person 1 | 595 Regarding age, we notice that primiparas, or first-time mothers, tend to have more premature births compared to multiparas, or mothers who have had multiple births. Also, women under 18 years old have a higher incidence of premature births compared to those over 18. |
| Accart-ville CMU manager | Specificities of mothers of premature children | n/a | person 1 | In my experience, I have noticed certain patterns. During my internships, I observed that premature births often involved mothers who had malaria, were anemic, or had genital infections that led to rising infections and ultimately prematurity. Others had conditions like preeclampsia, which necessitated early delivery. These are some of the factors I've observed. | person 1 | 596 In my experience, I have noticed certain patterns. During my internships, I observed that premature births often involved mothers who had malaria, were anemic, or had genital infections that led to rising infections and ultimately prematurity. Others had conditions like preeclampsia, which necessitated early delivery. These are some of the factors I've observed. |
| Accart-ville CMU manager | Specificities of mothers of premature children | n/a | person 1 | Often, there is a lack of understanding about how to care for a premature baby. For example, if a premature baby is in relatively good health and doesn't require hospitalization, we advise protective measures like not washing them too rigorously, only wiping them. However, due to traditional practices, parents may not follow this advice. They might bathe the baby extensively or force-feed them. Such practices can become risk factors, leading to associated infections. We advise against shaving them, as their skin is very fragile, but often this advice is ignored | person 1 | 597 Often, there is a lack of understanding about how to care for a premature baby. For example, if a premature baby is in relatively good health and doesn't require hospitalization, we advise protective measures like not washing them too rigorously, only wiping them. However, due to traditional practices, parents may not follow this advice. They might bathe the baby extensively or force-feed them. Such practices can become risk factors, leading to associated infections. We advise against shaving them, as their skin is very fragile, but often this advice is ignored |
| Accart-ville maternity manager | Specificities of mothers of premature children | n/a | person 1 | These mothers often experience anxiety and guilt, questioning why they gave birth prematurely. They worry about the viability and survival of their child. We need to reassure them and encourage them to follow our advice. | person 1 | 598 These mothers often experience anxiety and guilt, questioning why they gave birth prematurely. They worry about the viability and survival of their child. We need to reassure them and encourage them to follow our advice. |
| Accart-ville maternity manager | Specificities of mothers of premature children | n/a | person 1 | We see mothers from all socio-professional strata, but prematurity is most common in environments where poverty is prevalent. Many are housewives who engage in heavy physical work during pregn/acy. Regarding age, prematurity is mostly seen in first-time mothers and women under 18 | person 1 | 599 We see mothers from all socio-professional strata, but prematurity is most common in environments where poverty is prevalent. Many are housewives who engage in heavy physical work during pregn/acy. Regarding age, prematurity is mostly seen in first-time mothers and women under 18 |
| Accart-ville maternity manager | Specificities of mothers of premature children | n/a | person 1 | Yes, women experiencing their first pregn/acy | person 1 | 600 Yes, women experiencing their first pregn/acy |
| Accart-ville maternity manager | Specificities of mothers of premature children | n/a | person 1 | Often, maternal infections are a factor in prematurity. Due to the high number of prenatal consultations we handle daily, more than forty, it's challenging for a midwife to effectively screen for pathologies that can affect pregn/acy, such as urinary infections or bacterial vaginosis. We also try to understand their living conditions and stress levels, and discuss with their partners to prevent premature delivery. Urinary infections are a common cause of premature births. | person 1 | 601 Often, maternal infections are a factor in prematurity. Due to the high number of prenatal consultations we handle daily, more than forty, it's challenging for a midwife to effectively screen for pathologies that can affect pregn/acy, such as urinary infections or bacterial vaginosis. We also try to understand their living conditions and stress levels, and discuss with their partners to prevent premature delivery. Urinary infections are a common cause of premature births. |
| Accart-ville maternity manager | Specificities of mothers of premature children | n/a | person 1 | Yes, there's the time constraint, but also the lack of necessary laboratory facilities. In some countries, testing for urinary infections is routine, but in Burkina, it's not. This means that midwives may not detect the infection. Additionally, these tests are not free, and many women can't afford them, limiting the midwife's ability to make an effective diagnosis and prevent premature births. | person 1 | 602 Yes, there's the time constraint, but also the lack of necessary laboratory facilities. In some countries, testing for urinary infections is routine, but in Burkina, it's not. This means that midwives may not detect the infection. Additionally, these tests are not free, and many women can't afford them, limiting the midwife's ability to make an effective diagnosis and prevent premature births. |
| CMA Do focus group | Specificities of mothers of premature children | n/a | person 9 | Yes, certain health conditions stand out. Some obstetric pathologies almost invariably lead to premature delivery. As for age, it seems that all women, regardless of their age, are susceptible to these risks | person 9 | 603 Yes, certain health conditions stand out. Some obstetric pathologies almost invariably lead to premature delivery. As for age, it seems that all women, regardless of their age, are susceptible to these risks |
| Gynecologist 1 | Specificities of mothers of premature children | n/a | person 1 | From what we've noticed, prematurity affects mothers from all socio-economic backgrounds and ages equally. There doesn't seem to be a particular trend or specific demographic that is more affected than others in our observations | person 1 | 604 From what we've noticed, prematurity affects mothers from all socio-economic backgrounds and ages equally. There doesn't seem to be a particular trend or specific demographic that is more affected than others in our observations |
| Gynecologist 2 | Specificities of mothers of premature children | n/a | person 1 | Prematurity occurs across all social strata. We see premature births in both wealthy and disadvantaged social classes. However, a common factor we often observe is linked to certain illnesses. For example, in our system, prematurity is frequently associated with preeclampsia, a condition characterized by high blood pressure developing during pregn/acy. This condition leads to a lot of premature births and is found across all social classes.In terms of infectious problems, these are more commonly seen in the disadvantaged social classes. These women often miss antenatal care (ANC), lack preventive treatments against malaria, and do not receive consultations or necessary nutrient supplements like iron and other vitamins. Sometimes, they are women who have physically exerted themselves during pregn/acy, leading to premature labor due to physical weakness or premature rupture of membranes.Prematurity is also common in multiple pregn/acies, such as twins or more, which can lead to increased abdominal tension and cause the sac to mechanically rupture, resulting in premature birth. This can happen in any social class. To summarize, while infectious problems leading to prematurity are more prevalent in disadvantaged classes, conditions like preeclampsia and complications from multiple pregn/acies are found across all social backgrounds. | person 1 | 605 Prematurity occurs across all social strata. We see premature births in both wealthy and disadvantaged social classes. However, a common factor we often observe is linked to certain illnesses. For example, in our system, prematurity is frequently associated with preeclampsia, a condition characterized by high blood pressure developing during pregn/acy. This condition leads to a lot of premature births and is found across all social classes.In terms of infectious problems, these are more commonly seen in the disadvantaged social classes. These women often miss antenatal care (ANC), lack preventive treatments against malaria, and do not receive consultations or necessary nutrient supplements like iron and other vitamins. Sometimes, they are women who have physically exerted themselves during pregn/acy, leading to premature labor due to physical weakness or premature rupture of membranes.Prematurity is also common in multiple pregn/acies, such as twins or more, which can lead to increased abdominal tension and cause the sac to mechanically rupture, resulting in premature birth. This can happen in any social class. To summarize, while infectious problems leading to prematurity are more prevalent in disadvantaged classes, conditions like preeclampsia and complications from multiple pregn/acies are found across all social backgrounds. |
| MCD | Specificities of mothers of premature children | n/a | person 1 | I haven't observed any specific trends or particularities in this regard. I haven't conducted a detailed analysis to identify such patterns | person 1 | 606 I haven't observed any specific trends or particularities in this regard. I haven't conducted a detailed analysis to identify such patterns |
| MCD | Specificities of mothers of premature children | n/a | person 1 | It's not so much about predisposition as it is about certain conditions and illnesses during pregn/acy that can lead to prematurity. For instance, a simple case of malaria can result in a premature birth. This is just one example among many other illnesses. In our country, aside from malaria, there are other pathologies that can cause prematurity. It's more about exposure to certain conditions rather than a predisposition. | person 1 | 607 It's not so much about predisposition as it is about certain conditions and illnesses during pregn/acy that can lead to prematurity. For instance, a simple case of malaria can result in a premature birth. This is just one example among many other illnesses. In our country, aside from malaria, there are other pathologies that can cause prematurity. It's more about exposure to certain conditions rather than a predisposition. |
| MCD | Specificities of mothers of premature children | n/a | person 1 | Exactly. Exposure to conditions like malaria can certainly contribute to prematurity. Additionally, socio-economic challenges and physically demanding work can be contributing factors. Lifestyle choices, such as smoking or drug use, can also play a role, potentially leading to chronic conditions like diabetes that can trigger prematurity. There are also certain uterine malformations, including issues with the cervix, which can impact pregn/acy. In cases of multiple pregn/acies, the presence of chromosomal abnormalities in the children can also be a factor. So, there are several factors, rather than specific causes, that can lead to premature birth | person 1 | 608 Exactly. Exposure to conditions like malaria can certainly contribute to prematurity. Additionally, socio-economic challenges and physically demanding work can be contributing factors. Lifestyle choices, such as smoking or drug use, can also play a role, potentially leading to chronic conditions like diabetes that can trigger prematurity. There are also certain uterine malformations, including issues with the cervix, which can impact pregn/acy. In cases of multiple pregn/acies, the presence of chromosomal abnormalities in the children can also be a factor. So, there are several factors, rather than specific causes, that can lead to premature birth |
| CMA Do maternity care unit manager | Specificities of mothers of premature children | n/a | person 1 | Not really. We haven’t specifically looked for correlations between premature births and factors like socio-economic background or age. So, we don't have any particular observations in that regard. We haven’t made a connection between prematurity and specific age groups, such as those between 19 to 30 years old. Overall, it seems to be a general occurrence without distinct patterns based on our observations and approach | person 1 | 609 Not really. We haven’t specifically looked for correlations between premature births and factors like socio-economic background or age. So, we don't have any particular observations in that regard. We haven’t made a connection between prematurity and specific age groups, such as those between 19 to 30 years old. Overall, it seems to be a general occurrence without distinct patterns based on our observations and approach |
| Colma1 focus group | Specificities of mothers of premature children | n/a | person 2 | One example we had was a woman who experienced premature labor. She came for consultation when her cervix was already open, and she was already in labor, so it was too late to intervene. This suggests that late consultations are a common factor leading to premature births. If she had received earlier care, her premature labor might have been avoided. It’s often the case of delayed medical consultations and missed prenatal check-ups that contribute to these situations. | person 2 | 610 One example we had was a woman who experienced premature labor. She came for consultation when her cervix was already open, and she was already in labor, so it was too late to intervene. This suggests that late consultations are a common factor leading to premature births. If she had received earlier care, her premature labor might have been avoided. It’s often the case of delayed medical consultations and missed prenatal check-ups that contribute to these situations. |
| Colma1 focus group | Specificities of mothers of premature children | n/a | person 2 | No, I mean general medical consultations. When they are sick, they often come late for consultation. In this case, the delay led to premature labor, which could have been avoided. | person 2 | 611 No, I mean general medical consultations. When they are sick, they often come late for consultation. In this case, the delay led to premature labor, which could have been avoided. |
| Colma1 focus group | Specificities of mothers of premature children | n/a | person 2 | Often, these women face social challenges. They might be students or experiencing relationship problems in their couple. It's usually women in these situations who give birth prematurely. Others may be struggling socially, and some delay seeking medical consultations. | person 2 | 612 Often, these women face social challenges. They might be students or experiencing relationship problems in their couple. It's usually women in these situations who give birth prematurely. Others may be struggling socially, and some delay seeking medical consultations. |
| Colma1 focus group | Specificities of mothers of premature children | n/a | person 5 | To add, these women often have other health issues as well, such as malaria or hypertension, contributing to the risk of premature birth. | person 5 | 613 To add, these women often have other health issues as well, such as malaria or hypertension, contributing to the risk of premature birth. |
| Colma1 major | Specificities of mothers of premature children | n/a | person 1 | Honestly, I've never conducted a detailed analysis on that. However, from what I know, many cases of prematurity are due to malaria in the mother. The fever associated with malaria can trigger premature labor before the pregn/acy has reached full term. In my experience, infections and malaria are the two main health issues that I associate with prematurity. | person 1 | 614 Honestly, I've never conducted a detailed analysis on that. However, from what I know, many cases of prematurity are due to malaria in the mother. The fever associated with malaria can trigger premature labor before the pregn/acy has reached full term. In my experience, infections and malaria are the two main health issues that I associate with prematurity. |
| Colma1 major | Specificities of mothers of premature children | n/a | person 1 | Yes, it's linked to the mother's health. That's what I can recall from my experiences. | person 1 | 615 Yes, it's linked to the mother's health. That's what I can recall from my experiences. |
| Colma1 maternity manager | Specificities of mothers of premature children | n/a | person 1 | n/a | person 1 | 616 NA |
| Farakan focus group | Specificities of mothers of premature children | n/a | person 3 | What I've particularly noticed is that these women are often from deprived backgrounds. The majority are underprivileged, and many of them experience complications during pregn/acy, such as preeclampsia and malaria | person 3 | 617 What I've particularly noticed is that these women are often from deprived backgrounds. The majority are underprivileged, and many of them experience complications during pregn/acy, such as preeclampsia and malaria |
| Farakan focus group | Specificities of mothers of premature children | n/a | person 7 | I think a key issue is neglect. Often, these women neglect the advice given to them. For example, they don't adhere to their treatments or preventive measures against malaria, and malnutrition is common. They don’t follow our dietary advice. | person 7 | 618 I think a key issue is neglect. Often, these women neglect the advice given to them. For example, they don't adhere to their treatments or preventive measures against malaria, and malnutrition is common. They don’t follow our dietary advice. |
| Farakan focus group | Specificities of mothers of premature children | n/a | person 6 | Malnutrition is a common issue, both in rural and urban settings, where heavy work can also lead to prematurity. | person 6 | 619 Malnutrition is a common issue, both in rural and urban settings, where heavy work can also lead to prematurity. |
| Farakan focus group | Specificities of mothers of premature children | n/a | person 3 | Recently, the misconception about free medical services has worsened the situation. When treatments are prescribed, compliance is poor, particularly for malaria prevention. Many women don’t pay for essential medications like sulfadoxine, leading to untreated conditions | person 3 | 620 Recently, the misconception about free medical services has worsened the situation. When treatments are prescribed, compliance is poor, particularly for malaria prevention. Many women don’t pay for essential medications like sulfadoxine, leading to untreated conditions |
| Farakan focus group | Specificities of mothers of premature children | n/a | person 2 | I agree about the issue of neglect and poverty. For instance, women might receive mosquito nets but don't use them, and then suffer from malaria. These factors, along with poor health practices, contribute to premature births. | person 2 | 621 I agree about the issue of neglect and poverty. For instance, women might receive mosquito nets but don't use them, and then suffer from malaria. These factors, along with poor health practices, contribute to premature births. |
| Farakan focus group | Specificities of mothers of premature children | n/a | person 6 | Many women also neglect taking supplements like iron, which is crucial. This neglect leads to conditions like anemia, further complicating their pregn/acies. | person 6 | 622 Many women also neglect taking supplements like iron, which is crucial. This neglect leads to conditions like anemia, further complicating their pregn/acies. |
| Farakan major | Specificities of mothers of premature children | n/a | person 1 | Typically, we see premature births more frequently in young girls. It's often the case that first pregn/acies result in premature birth, so it predomin/atly concerns younger women | person 1 | 623 Typically, we see premature births more frequently in young girls. It's often the case that first pregn/acies result in premature birth, so it predomin/atly concerns younger women |
| Farakan maternity manager | Specificities of mothers of premature children | n/a | person 1 | Yes, several factors can lead to prematurity. Malnutrition is a significant one. Additionally, anemia, high blood pressure, and preeclampsia are common factors that favor premature births | person 1 | 624 Yes, several factors can lead to prematurity. Malnutrition is a significant one. Additionally, anemia, high blood pressure, and preeclampsia are common factors that favor premature births |
| Farakan maternity manager | Specificities of mothers of premature children | n/a | person 1 | It varies; we see prematurity across all age groups. | person 1 | 625 It varies; we see prematurity across all age groups. |
| Farakan maternity manager | Specificities of mothers of premature children | n/a | person 1 | Malnutrition, which is linked to socio-economic status, plays a role. If a mother can't eat well, it can lead to prematurity. We also frequently see young girls with unwanted pregn/acies experiencing premature births. | person 1 | 626 Malnutrition, which is linked to socio-economic status, plays a role. If a mother can't eat well, it can lead to prematurity. We also frequently see young girls with unwanted pregn/acies experiencing premature births. |
| Accart-ville focus group | Improvement in care of preterm birth | n/a | person 3 | Raising awareness in the community is crucial. Women should start antenatal care (ANC) early and focus on taking iron supplements and maintaining a good diet. Proper follow-up care is also key. With increased awareness, we can potentially reduce the incidence of prematurity. | person 3 | 627 Raising awareness in the community is crucial. Women should start antenatal care (ANC) early and focus on taking iron supplements and maintaining a good diet. Proper follow-up care is also key. With increased awareness, we can potentially reduce the incidence of prematurity. |
| Accart-ville focus group | Improvement in care of preterm birth | n/a | person 3 | We can approach this in two stages. Firstly, we need to focus on preventing prematurity by closely monitoring pregn/acies. Once prematurity occurs, managing issues like infections and hypothermia is vital. However, we currently lack the necessary medical-technical equipment, including a dedicated room for premature babies, incubators, and ultrasound machines. While we have two doctors and adequate staff, the challenge lies in having the right equipment. | person 3 | 628 We can approach this in two stages. Firstly, we need to focus on preventing prematurity by closely monitoring pregn/acies. Once prematurity occurs, managing issues like infections and hypothermia is vital. However, we currently lack the necessary medical-technical equipment, including a dedicated room for premature babies, incubators, and ultrasound machines. While we have two doctors and adequate staff, the challenge lies in having the right equipment. |
| Accart-ville focus group | Improvement in care of preterm birth | n/a | person 3 | Yes, we have qualified staff, but continuous training is essential since the skills needed for this care are not used daily. The idea is to train a few staff members who can then share their knowledge with others, ensuring skills are passed on and care quality is maintained even if trained personnel leave. | person 3 | 629 Yes, we have qualified staff, but continuous training is essential since the skills needed for this care are not used daily. The idea is to train a few staff members who can then share their knowledge with others, ensuring skills are passed on and care quality is maintained even if trained personnel leave. |
| Accart-ville focus group | Improvement in care of preterm birth | n/a | person 1 | When we train health agents, they should share their skills with those who didn’t receive the training. This way, the knowledge is disseminated, ensuring continuous and effective care. Even if trained staff move on, the remaining staff who have learned from them can maintain the quality of care. | person 1 | 630 When we train health agents, they should share their skills with those who didn’t receive the training. This way, the knowledge is disseminated, ensuring continuous and effective care. Even if trained staff move on, the remaining staff who have learned from them can maintain the quality of care. |
| Accart-ville focus group | Improvement in care of preterm birth | n/a | person 3 | To add, staff movement is indeed a challenge. To address this, we could have a policy where staff sign a commitment to stay for a certain number of years after receiving training or focus on training local staff who are more likely to remain in the center. | person 3 | 631 To add, staff movement is indeed a challenge. To address this, we could have a policy where staff sign a commitment to stay for a certain number of years after receiving training or focus on training local staff who are more likely to remain in the center. |
| Accart-ville CMU manager | Improvement in care of preterm birth | n/a | person 1 | The first step is enhancing our technical platform and staffing. More staff would allow us to improve care for women, aiming to reduce premature births. If we do have premature babies, we need to be able to manage them effectively to ease the burden on higher-level facilities like the CMA or CHUSS. Currently, we lack incubators, which are only available at the CHUSS level, causing overcrowding there. The lack of essential equipment like oxygen is a significant problem. If we had the right equipment and infrastructure, I believe the necessary skills among our staff would follow. We have the basic knowledge, but we need an improved technical platform to enhance our capabilities. | person 1 | 632 The first step is enhancing our technical platform and staffing. More staff would allow us to improve care for women, aiming to reduce premature births. If we do have premature babies, we need to be able to manage them effectively to ease the burden on higher-level facilities like the CMA or CHUSS. Currently, we lack incubators, which are only available at the CHUSS level, causing overcrowding there. The lack of essential equipment like oxygen is a significant problem. If we had the right equipment and infrastructure, I believe the necessary skills among our staff would follow. We have the basic knowledge, but we need an improved technical platform to enhance our capabilities. |
| Accart-ville CMU manager | Improvement in care of preterm birth | n/a | person 1 | Staff movement can affect the service, but it shouldn't disrupt continuity. Losing skilled personnel who are familiar with prematurity care and onboarding new staff does take time. However, typically not everyone leaves at once; it's usually one or two people at a time. So, I believe it's a manageable situation. We can maintain service quality despite these movements. | person 1 | 633 Staff movement can affect the service, but it shouldn't disrupt continuity. Losing skilled personnel who are familiar with prematurity care and onboarding new staff does take time. However, typically not everyone leaves at once; it's usually one or two people at a time. So, I believe it's a manageable situation. We can maintain service quality despite these movements. |
| Accart-ville CMU manager | Improvement in care of preterm birth | n/a | person 1 | In my opinion, the community's perception doesn't directly affect the support provided. However, there's a common belief that premature children eventually catch up in development. For example, a child born two months early is often expected to reach developmental milestones within two months. Despite this, there's still a stigma attached to being premature, which can be hurtful. Even when a child has caught up developmentally, they may still be labeled as 'premature' in the community. This kind of stigma can lead to feelings of rejection, which can be psychologically damaging | person 1 | 634 In my opinion, the community's perception doesn't directly affect the support provided. However, there's a common belief that premature children eventually catch up in development. For example, a child born two months early is often expected to reach developmental milestones within two months. Despite this, there's still a stigma attached to being premature, which can be hurtful. Even when a child has caught up developmentally, they may still be labeled as 'premature' in the community. This kind of stigma can lead to feelings of rejection, which can be psychologically damaging |
| Accart-ville CMU manager | Improvement in care of preterm birth | n/a | person 1 | Yes, but I believe it doesn't pose a physical health issue, rather a mental or psychological one. For instance, if a baby born at 7 months catches up developmentally in 2 months, physically, they're no different from a full-term baby. However, societal views and stigmatization can have a profound impact. It's not the physical aspect but the social stigma that's the real problem. | person 1 | 635 Yes, but I believe it doesn't pose a physical health issue, rather a mental or psychological one. For instance, if a baby born at 7 months catches up developmentally in 2 months, physically, they're no different from a full-term baby. However, societal views and stigmatization can have a profound impact. It's not the physical aspect but the social stigma that's the real problem. |
| Accart-ville maternity manager | Improvement in care of preterm birth | n/a | person 1 | One solution is to have a specific room dedicated to premature newborns. Even though we don't currently manage them, having incubators would be beneficial. When we evacuate babies to the hospital, they often return to us, and waiting for an available incubator impacts their survival. If we had emergency equipment like incubators and heat lamps, we could stabilize the child before evacuation. It would also be helpful if our center had a pediatrician or a pediatric health representative to improve overall care for children, especially newborns. So, our needs include incubators, a dedicated room for newborns, and heating lamps. | person 1 | 636 One solution is to have a specific room dedicated to premature newborns. Even though we don't currently manage them, having incubators would be beneficial. When we evacuate babies to the hospital, they often return to us, and waiting for an available incubator impacts their survival. If we had emergency equipment like incubators and heat lamps, we could stabilize the child before evacuation. It would also be helpful if our center had a pediatrician or a pediatric health representative to improve overall care for children, especially newborns. So, our needs include incubators, a dedicated room for newborns, and heating lamps. |
| Accart-ville maternity manager | Improvement in care of preterm birth | n/a | person 1 | Retraining is also crucial. While having a pediatrician or pediatric health officer is ideal, retraining in newborn care, especially for premature infants, can help reduce neonatal mortality. | person 1 | 637 Retraining is also crucial. While having a pediatrician or pediatric health officer is ideal, retraining in newborn care, especially for premature infants, can help reduce neonatal mortality. |
| Accart-ville maternity manager | Improvement in care of preterm birth | n/a | person 1 | It is a challenge, as trained staff often move to other locations. A potential solution is to have trained staff sign a commitment to stay for a certain number of years. Another approach could be to train mentors not necessarily from our health center. These mentors could work for a week per quarter in real-time at the center, training our staff on newborn care. This way, even if trained staff leave, there is continued mentorship and knowledge transfer. These solutions could help maintain a consistent level of care despite the movement of trained health workers. | person 1 | 638 It is a challenge, as trained staff often move to other locations. A potential solution is to have trained staff sign a commitment to stay for a certain number of years. Another approach could be to train mentors not necessarily from our health center. These mentors could work for a week per quarter in real-time at the center, training our staff on newborn care. This way, even if trained staff leave, there is continued mentorship and knowledge transfer. These solutions could help maintain a consistent level of care despite the movement of trained health workers. |
| CMA Do focus group | Improvement in care of preterm birth | n/a | person 3 | We urgently need incubators and a dedicated department for prematurity, essentially a neonatology unit equipped with the necessary tools and staff. | person 3 | 639 We urgently need incubators and a dedicated department for prematurity, essentially a neonatology unit equipped with the necessary tools and staff. |
| CMA Do focus group | Improvement in care of preterm birth | n/a | person 9 | It's crucial to strengthen our resuscitation equipment. We need heating lamps, oxygenation facilities, and improved suction capabilities. There's also a shortage of nasogastric tubes, which are essential but rare and expensive. Having these available at our center would significantly improve our ability to manage premature births. | person 9 | 640 It's crucial to strengthen our resuscitation equipment. We need heating lamps, oxygenation facilities, and improved suction capabilities. There's also a shortage of nasogastric tubes, which are essential but rare and expensive. Having these available at our center would significantly improve our ability to manage premature births. |
| CMA Do focus group | Improvement in care of preterm birth | n/a | person 1 | Staff assignments will inevitably affect the quality of care services. | person 1 | 641 Staff assignments will inevitably affect the quality of care services. |
| CMA Do focus group | Improvement in care of preterm birth | n/a | person 1 | One approach is to ensure that competent staff members are trained so that they can pass on their knowledge. This way, there are always trained responders available, even if some have to leave. | person 1 | 642 One approach is to ensure that competent staff members are trained so that they can pass on their knowledge. This way, there are always trained responders available, even if some have to leave. |
| CMA Do focus group | Improvement in care of preterm birth | n/a | person 6 | According to our standards, all health workers should be trained in neonatal care. So, a person moving from one location to another, say from Bobo to Ouaga, should already be qualified to care for premature babies. If there are untrained individuals in a team, those who are trained should educate their untrained colleagues. The goal is for every health worker, regardless of their location, to be qualified in caring for premature newborns. | person 6 | 643 According to our standards, all health workers should be trained in neonatal care. So, a person moving from one location to another, say from Bobo to Ouaga, should already be qualified to care for premature babies. If there are untrained individuals in a team, those who are trained should educate their untrained colleagues. The goal is for every health worker, regardless of their location, to be qualified in caring for premature newborns. |
| Gynecologist 1 | Improvement in care of preterm birth | n/a | person 1 | To enhance the care of premature babies, we need to strengthen our capabilities. Specifically, we should create a neonatology unit staffed with pediatricians to provide dedicated care. This is crucial because the only neonatology unit in Bobo, at the University Hospital, is constantly overloaded. The overcrowding increases the risk of infection and contributes to higher mortality rates. Creating neonatology units at the two CMAs would not only relieve congestion at the hospital but also provide these children with better care opportunities. We need to bolster our infrastructure, personnel, and skills at the CMAs. | person 1 | 644 To enhance the care of premature babies, we need to strengthen our capabilities. Specifically, we should create a neonatology unit staffed with pediatricians to provide dedicated care. This is crucial because the only neonatology unit in Bobo, at the University Hospital, is constantly overloaded. The overcrowding increases the risk of infection and contributes to higher mortality rates. Creating neonatology units at the two CMAs would not only relieve congestion at the hospital but also provide these children with better care opportunities. We need to bolster our infrastructure, personnel, and skills at the CMAs. |
| Gynecologist 1 | Improvement in care of preterm birth | n/a | person 1 | Yes, it can have an impact. | person 1 | 645 Yes, it can have an impact. |
| Gynecologist 1 | Improvement in care of preterm birth | n/a | person 1 | If we have a neonatology unit, specific training for newborn care isn't necessarily lengthy. A staff member can become operational in about one or two months. Challenges like venous line placement in premature babies, which is more difficult than in older children or adults, can be managed. Even with staff movements, the institutional memory and expertise remain. This knowledge can be passed on to train new staff, ensuring continuity and progress in care | person 1 | 646 If we have a neonatology unit, specific training for newborn care isn't necessarily lengthy. A staff member can become operational in about one or two months. Challenges like venous line placement in premature babies, which is more difficult than in older children or adults, can be managed. Even with staff movements, the institutional memory and expertise remain. This knowledge can be passed on to train new staff, ensuring continuity and progress in care |
| Gynecologist 1 | Improvement in care of preterm birth | n/a | person 1 | Yes, they can significantly affect the health of the mother of a premature child. | person 1 | 647 Yes, they can significantly affect the health of the mother of a premature child. |
| Gynecologist 1 | Improvement in care of preterm birth | n/a | person 1 | For instance, there's a cultural notion that if a baby doesn't yet have a name or hasn't been baptized, there's less inclination to invest in their health. The attitude is like, 'If the child is meant to pass away, let it be.' We encountered a case like this not long ago. This leads to less enthusiasm for caring for premature babies compared to others. Moreover, caring for a premature baby demands more time and patience, which can be challenging for a mother also responsible for other children and household duties | person 1 | 648 For instance, there's a cultural notion that if a baby doesn't yet have a name or hasn't been baptized, there's less inclination to invest in their health. The attitude is like, 'If the child is meant to pass away, let it be.' We encountered a case like this not long ago. This leads to less enthusiasm for caring for premature babies compared to others. Moreover, caring for a premature baby demands more time and patience, which can be challenging for a mother also responsible for other children and household duties |
| Gynecologist 1 | Improvement in care of preterm birth | n/a | person 1 | We engage in discussions with the parents, explaining the benefits of the care we provide and the process involved. We acknowledge that it takes time but emphasize that it's outpatient care. With the kangaroo mother care system, premature babies can stay close to their mothers, allowing the mothers to continue with minor activities. We strive to raise awareness and encourage acceptance of this approach. Some parents are receptive, but others, unfortunately, are lost to follow-up, indicating their non-acceptance | person 1 | 649 We engage in discussions with the parents, explaining the benefits of the care we provide and the process involved. We acknowledge that it takes time but emphasize that it's outpatient care. With the kangaroo mother care system, premature babies can stay close to their mothers, allowing the mothers to continue with minor activities. We strive to raise awareness and encourage acceptance of this approach. Some parents are receptive, but others, unfortunately, are lost to follow-up, indicating their non-acceptance |
| Gynecologist 2 | Improvement in care of preterm birth | n/a | person 1 | As I mentioned earlier, many of our midwives have been trained, although not formally. Some have spent time in the Kangaroo Unit and have acquired skills to care for premature babies. However, this unit isn't operating at full capacity; we don't have enough staff to run it 24/7. It's mostly operational during the day or afternoon, but not at night. Additionally, we're facing equipment shortages. We need trained personnel operating the Kangaroo unit around the clock. Ideally, we should establish a full pediatric unit with a pediatrician and dedicated staff. We have around 6,000 births per year, and a significant number of these are premature. Having a neonatology unit within the pediatric facility would greatly improve care, including the provision of incubators for very premature babies. Instead of sending them to the hospital, they could be cared for here. This requires additional equipment, dedicated buildings, and trained staff. | person 1 | 650 As I mentioned earlier, many of our midwives have been trained, although not formally. Some have spent time in the Kangaroo Unit and have acquired skills to care for premature babies. However, this unit isn't operating at full capacity; we don't have enough staff to run it 24/7. It's mostly operational during the day or afternoon, but not at night. Additionally, we're facing equipment shortages. We need trained personnel operating the Kangaroo unit around the clock. Ideally, we should establish a full pediatric unit with a pediatrician and dedicated staff. We have around 6,000 births per year, and a significant number of these are premature. Having a neonatology unit within the pediatric facility would greatly improve care, including the provision of incubators for very premature babies. Instead of sending them to the hospital, they could be cared for here. This requires additional equipment, dedicated buildings, and trained staff. |
| Gynecologist 2 | Improvement in care of preterm birth | n/a | person 1 | With a pediatric unit, the dynamic changes. Currently, midwives handle deliveries and provide initial care to premature babies, but for in-depth care, they need to be transferred to the Kangaroo unit. The midwives are limited in how much they can do while also delivering babies. Having a unit that operates full-time with a pediatrician would mean comprehensive training for staff in caring for premature babies and other children, leading to more skilled personnel in this area. | person 1 | 651 With a pediatric unit, the dynamic changes. Currently, midwives handle deliveries and provide initial care to premature babies, but for in-depth care, they need to be transferred to the Kangaroo unit. The midwives are limited in how much they can do while also delivering babies. Having a unit that operates full-time with a pediatrician would mean comprehensive training for staff in caring for premature babies and other children, leading to more skilled personnel in this area. |
| Gynecologist 2 | Improvement in care of preterm birth | n/a | person 1 | Yes, definitely. For instance, a pregn/at woman who is undernourished or lacks the means for proper food, clothing, and hygiene can be at risk of giving birth prematurely. Furthermore, if the child is born into an unfavorable environment, the chances of successfully nurturing the child are reduced | person 1 | 652 Yes, definitely. For instance, a pregn/at woman who is undernourished or lacks the means for proper food, clothing, and hygiene can be at risk of giving birth prematurely. Furthermore, if the child is born into an unfavorable environment, the chances of successfully nurturing the child are reduced |
| Gynecologist 2 | Improvement in care of preterm birth | n/a | person 1 | Certainly, but I think there's a shift happening. Community practices and prohibitions can contribute to premature births. Early and child marriages are prime examples. A very young bride, lacking experience in managing her pregn/acy or even trying to conceal it, often results in premature birth. If there's no support system to assist with the baby, care becomes exceedingly difficult. Early marriages and pregn/acies out of wedlock, like those occurring in schools, frequently involve adolescents who may attempt to hide their pregn/acies. This lack of support often leads to premature births. Moreover, if the young mother is not supported by her family, viewed as bringing dishonor or shame, both she and her child are left in a vulnerable position. This lack of support makes care extremely challenging, a scenario that's also common in early marriages. Without the support of the husband's family, such as the mother-in-law or aunts, care for the young mother becomes difficult. | person 1 | 653 Certainly, but I think there's a shift happening. Community practices and prohibitions can contribute to premature births. Early and child marriages are prime examples. A very young bride, lacking experience in managing her pregn/acy or even trying to conceal it, often results in premature birth. If there's no support system to assist with the baby, care becomes exceedingly difficult. Early marriages and pregn/acies out of wedlock, like those occurring in schools, frequently involve adolescents who may attempt to hide their pregn/acies. This lack of support often leads to premature births. Moreover, if the young mother is not supported by her family, viewed as bringing dishonor or shame, both she and her child are left in a vulnerable position. This lack of support makes care extremely challenging, a scenario that's also common in early marriages. Without the support of the husband's family, such as the mother-in-law or aunts, care for the young mother becomes difficult. |
| MCD | Improvement in care of preterm birth | n/a | person 1 | Firstly, it begins with improved pregn/acy monitoring. We encourage women to visit us early in the first trimester to receive optimal advice for their pregn/acy. Pregn/acy isn't an illness; it's a state that requires guidance on managing it effectively. During visits, we not only offer routine care but also educate mothers on danger signs that might indicate a risk of premature birth. Signs like severe abdominal pain, bleeding, or excruciating headaches in an incomplete term of pregn/acy are red flags we teach them to recognize.We also emphasize proper nutrition throughout the pregn/acy to ensure the mother's and baby's health. This preparation can lead to easier delivery as the mother is better informed and prepared.Regarding the care of the premature child, once the baby is born, we need a responsive platform. Currently, we lack facilities like an incubator, a pediatric unit, or a neonatology unit. We manage with a Kangaroo Mother Care (SMK) unit. Hypothermia is a major risk for premature babies, often more dangerous than infection. The SMK method involves skin-to-skin contact where the baby is held close to the mother's belly, similar to a kangaroo carrying its young, to provide warmth and prevent hypothermia. But if we're unable to manage a case effectively, we have no choice but to evacuate the child to a better-equipped facility. | person 1 | 654 Firstly, it begins with improved pregn/acy monitoring. We encourage women to visit us early in the first trimester to receive optimal advice for their pregn/acy. Pregn/acy isn't an illness; it's a state that requires guidance on managing it effectively. During visits, we not only offer routine care but also educate mothers on danger signs that might indicate a risk of premature birth. Signs like severe abdominal pain, bleeding, or excruciating headaches in an incomplete term of pregn/acy are red flags we teach them to recognize.We also emphasize proper nutrition throughout the pregn/acy to ensure the mother's and baby's health. This preparation can lead to easier delivery as the mother is better informed and prepared.Regarding the care of the premature child, once the baby is born, we need a responsive platform. Currently, we lack facilities like an incubator, a pediatric unit, or a neonatology unit. We manage with a Kangaroo Mother Care (SMK) unit. Hypothermia is a major risk for premature babies, often more dangerous than infection. The SMK method involves skin-to-skin contact where the baby is held close to the mother's belly, similar to a kangaroo carrying its young, to provide warmth and prevent hypothermia. But if we're unable to manage a case effectively, we have no choice but to evacuate the child to a better-equipped facility. |
| MCD | Improvement in care of preterm birth | n/a | person 1 | Regarding the impact of socio-cultural factors on prematurity, I'm not entirely sure. I'm not well-informed about any specific consequences related to prematurity in our society. As for pregn/acy, miscarriages are a known issue, and it's understood that this means the pregn/acy hasn't reached full term. Society is also aware of abortions. But when it comes to the specifics of how these issues might relate to premature births, I can't say much. I'm not fully aware of any particular apprehensions or beliefs surrounding prematurity. | person 1 | 655 Regarding the impact of socio-cultural factors on prematurity, I'm not entirely sure. I'm not well-informed about any specific consequences related to prematurity in our society. As for pregn/acy, miscarriages are a known issue, and it's understood that this means the pregn/acy hasn't reached full term. Society is also aware of abortions. But when it comes to the specifics of how these issues might relate to premature births, I can't say much. I'm not fully aware of any particular apprehensions or beliefs surrounding prematurity. |
| CMA Do maternity care unit manager | Improvement in care of preterm birth | n/a | person 1 | First, accurate diagnosis is crucial. We need to reliably identify premature births, whether through the date of the last menstrual period or an ultrasound. Once diagnosed, we can take appropriate measures to manage the birth, preparing the mother for potential complications, like hypoglycemia, and how to address them. Awareness and preparation for potential infections post-delivery are also important.Another key aspect is arranging for the safe transfer of premature babies from facilities like the CMA to the CHU, especially in cases where there is no ambulance available. We also need training for health workers, not just in care but in how to safely transfer newborns to specialized care centers like neonatology units. It's essential to have a clear protocol and the necessary resources for these transfers. | person 1 | 656 First, accurate diagnosis is crucial. We need to reliably identify premature births, whether through the date of the last menstrual period or an ultrasound. Once diagnosed, we can take appropriate measures to manage the birth, preparing the mother for potential complications, like hypoglycemia, and how to address them. Awareness and preparation for potential infections post-delivery are also important.Another key aspect is arranging for the safe transfer of premature babies from facilities like the CMA to the CHU, especially in cases where there is no ambulance available. We also need training for health workers, not just in care but in how to safely transfer newborns to specialized care centers like neonatology units. It's essential to have a clear protocol and the necessary resources for these transfers. |
| CMA Do maternity care unit manager | Improvement in care of preterm birth | n/a | person 1 | It can have an impact, but not significantly. Even if staff are trained and then assigned elsewhere, the knowledge they've acquired can be shared with others in the group. This way, the expertise remains within the center. Additionally, those who move to new locations can impart their skills there, benefiting a wider population. So, while staff movement is a factor, it should not drastically affect the overall quality of care. Trained individuals can play a key role in disseminating knowledge and guiding others in managing premature births effectively. | person 1 | 657 It can have an impact, but not significantly. Even if staff are trained and then assigned elsewhere, the knowledge they've acquired can be shared with others in the group. This way, the expertise remains within the center. Additionally, those who move to new locations can impart their skills there, benefiting a wider population. So, while staff movement is a factor, it should not drastically affect the overall quality of care. Trained individuals can play a key role in disseminating knowledge and guiding others in managing premature births effectively. |
| CMA Do maternity care unit manager | Improvement in care of preterm birth | n/a | person 1 | Yes, I believe so. There's a tendency to feel discouraged when dealing with a premature baby. People often prematurely assume that the child won't survive. There's also a perception, even among healthcare workers, that premature girls have better survival rates than boys, though there's no scientific evidence for this. This belief can lead to hesitancy in providing care, especially for premature boys, as people might doubt the effectiveness of treatment. Moreover, there are many misconceptions and harmful practices in the community regarding the care of premature babies. For instance, I recall a case from my time as a midwifery student. A relative had a baby with low birth weight, but the community assumed it was a case of prematurity. They shaved the baby's head and applied a product to it, and a traditional practitioner put something in the baby's mouth, which caused an injury and infection. The baby couldn't breastfeed anymore, and the injury went unnoticed in medical examinations. Only when I examined the baby did I discover a mass in its mouth, a result of the harmful practice.This incident highlights the impact of socio-cultural beliefs and practices on the care of premature babies. However, I think proper guidance and clear information about how to care for these fragile babies, including advice against force-feeding and purging, can make a significant difference | person 1 | 658 Yes, I believe so. There's a tendency to feel discouraged when dealing with a premature baby. People often prematurely assume that the child won't survive. There's also a perception, even among healthcare workers, that premature girls have better survival rates than boys, though there's no scientific evidence for this. This belief can lead to hesitancy in providing care, especially for premature boys, as people might doubt the effectiveness of treatment. Moreover, there are many misconceptions and harmful practices in the community regarding the care of premature babies. For instance, I recall a case from my time as a midwifery student. A relative had a baby with low birth weight, but the community assumed it was a case of prematurity. They shaved the baby's head and applied a product to it, and a traditional practitioner put something in the baby's mouth, which caused an injury and infection. The baby couldn't breastfeed anymore, and the injury went unnoticed in medical examinations. Only when I examined the baby did I discover a mass in its mouth, a result of the harmful practice.This incident highlights the impact of socio-cultural beliefs and practices on the care of premature babies. However, I think proper guidance and clear information about how to care for these fragile babies, including advice against force-feeding and purging, can make a significant difference |
| Colma1 focus group | Improvement in care of preterm birth | n/a | person 2 | For prenatal care, we emphasize early consultation and awareness of danger signs during antenatal care (ANC). Postnatally, providing incubators in larger health facilities could significantly reduce neonatal death risks | person 2 | 659 For prenatal care, we emphasize early consultation and awareness of danger signs during antenatal care (ANC). Postnatally, providing incubators in larger health facilities could significantly reduce neonatal death risks |
| Colma1 focus group | Improvement in care of preterm birth | n/a | person 2 | Regarding postnatal care, if we had incubators, even basic ones, at our health center, it would help immensely. Currently, we have to transfer cases to CHURSS for lack of facilities | person 2 | 660 Regarding postnatal care, if we had incubators, even basic ones, at our health center, it would help immensely. Currently, we have to transfer cases to CHURSS for lack of facilities |
| Colma1 focus group | Improvement in care of preterm birth | n/a | person 5 | Training staff on immediate postnatal care is crucial. Knowing what to do for the newborn while waiting for evacuation can save lives. Training in prematurity care is essential for our staff | person 5 | 661 Training staff on immediate postnatal care is crucial. Knowing what to do for the newborn while waiting for evacuation can save lives. Training in prematurity care is essential for our staff |
| Colma1 focus group | Improvement in care of preterm birth | n/a | person 2 | Adding heat lamps at our health center could be beneficial, especially when we don't have access to incubators | person 2 | 662 Adding heat lamps at our health center could be beneficial, especially when we don't have access to incubators |
| Colma1 focus group | Improvement in care of preterm birth | n/a | person 2 | The danger signs include pelvic pain, bleeding, fever, severe headaches, and dizziness. | person 2 | 663 The danger signs include pelvic pain, bleeding, fever, severe headaches, and dizziness. |
| Colma1 focus group | Improvement in care of preterm birth | n/a | person 5 | There's also the psychological and social aspect. Addressing social issues and mediating conflicts within families can have a positive impact on preventing prematurity. | person 5 | 664 There's also the psychological and social aspect. Addressing social issues and mediating conflicts within families can have a positive impact on preventing prematurity. |
| Colma1 focus group | Improvement in care of preterm birth | n/a | person 5 | Continuity is key. If trained staff leave, the habits and protocols they've established can be passed on to new staff. It's like on-site training | person 5 | 665 Continuity is key. If trained staff leave, the habits and protocols they've established can be passed on to new staff. It's like on-site training |
| Colma1 focus group | Improvement in care of preterm birth | n/a | person 6 | Socio-cultural issues, like domestic disputes or denial of paternity, can stress the mother and lead to premature births. We use invitation notes to involve husbands in consultations, aiming to resolve conflicts and promote unity, as discord negatively impacts the pregn/acy | person 6 | 666 Socio-cultural issues, like domestic disputes or denial of paternity, can stress the mother and lead to premature births. We use invitation notes to involve husbands in consultations, aiming to resolve conflicts and promote unity, as discord negatively impacts the pregn/acy |
| Colma1 focus group | Improvement in care of preterm birth | n/a | person 7 | We also encounter cases of physical violence and aggression. Counseling the couple, especially the husband, to be gentle and supportive is crucial as such stress can trigger premature births | person 7 | 667 We also encounter cases of physical violence and aggression. Counseling the couple, especially the husband, to be gentle and supportive is crucial as such stress can trigger premature births |
| Colma1 focus group | Improvement in care of preterm birth | n/a | person 5 | Regarding societal perceptions, I can't comment much. However, I've noticed an increasing awareness about the care of premature babies. People now understand that such babies shouldn’t be exposed outdoors and need to be protected. There’s growing knowledge about the special needs of premature babies, like avoiding baths or keeping them in a bedroom. When we give advice, especially to a mother-in-law, they tend to follow it meticulously | person 5 | 668 Regarding societal perceptions, I can't comment much. However, I've noticed an increasing awareness about the care of premature babies. People now understand that such babies shouldn’t be exposed outdoors and need to be protected. There’s growing knowledge about the special needs of premature babies, like avoiding baths or keeping them in a bedroom. When we give advice, especially to a mother-in-law, they tend to follow it meticulously |
| Colma1 focus group | Improvement in care of preterm birth | n/a | person 3 | Adding to that, for the mother, issues like hypertension can seriously affect pregn/acy. If hypertension isn’t detected early, it can lead to premature birth | person 3 | 669 Adding to that, for the mother, issues like hypertension can seriously affect pregn/acy. If hypertension isn’t detected early, it can lead to premature birth |
| Colma1 focus group | Improvement in care of preterm birth | n/a | person 6 | We had a case where a woman’s premature birth was linked to her husband's death. She faced neglect and lack of care from her in-laws, leading to unmanaged hypertension. Despite starting antenatal care, her condition worsened due to the stress and lack of support. She couldn’t afford her medication and eventually delivered a stillborn, before the end of her second trimester | person 6 | 670 We had a case where a woman’s premature birth was linked to her husband's death. She faced neglect and lack of care from her in-laws, leading to unmanaged hypertension. Despite starting antenatal care, her condition worsened due to the stress and lack of support. She couldn’t afford her medication and eventually delivered a stillborn, before the end of her second trimester |
| Colma1 focus group | Improvement in care of preterm birth | n/a | person 6 | If I recall correctly, it was around 5 months. She had only taken one dose of her medication before going into labor | person 6 | 671 If I recall correctly, it was around 5 months. She had only taken one dose of her medication before going into labor |
| Colma1 focus group | Improvement in care of preterm birth | n/a | person 6 | We also see cases involving internally displaced women. Often, their husbands are dead or absent, and they lack family support. In these situations, I make calls to their families to raise awareness. Without support, these women's pregn/acies become extremely challenging. I focus a lot on raising awareness about love and support in the family. | person 6 | 672 We also see cases involving internally displaced women. Often, their husbands are dead or absent, and they lack family support. In these situations, I make calls to their families to raise awareness. Without support, these women's pregn/acies become extremely challenging. I focus a lot on raising awareness about love and support in the family. |
| Colma1 major | Improvement in care of preterm birth | n/a | person 1 | We need to focus on the causes of prematurity, like malaria prevention during prenatal care (CPN). We distribute mosquito nets and sulfadoxine-pyrimethamine (SP) for malaria prevention. Educating women to seek immediate care for any health issue is crucial. However, our community still faces challenges with literacy and awareness, leading to delayed healthcare seeking until it's often too late. | person 1 | 673 We need to focus on the causes of prematurity, like malaria prevention during prenatal care (CPN). We distribute mosquito nets and sulfadoxine-pyrimethamine (SP) for malaria prevention. Educating women to seek immediate care for any health issue is crucial. However, our community still faces challenges with literacy and awareness, leading to delayed healthcare seeking until it's often too late. |
| Colma1 major | Improvement in care of preterm birth | n/a | person 1 | Yes, improvements are necessary. Enhancing staff training can strengthen our capacity to identify and manage prematurity risks. Our team mainly consists of auxiliary midwives who could benefit from advanced training. Also, ensuring a steady supply of essential medicines and consumables, like SP for malaria prevention, is critical. If we can strengthen our staff’s capabilities and ensure the availability of medicines, it would greatly improve our care for premature babies | person 1 | 674 Yes, improvements are necessary. Enhancing staff training can strengthen our capacity to identify and manage prematurity risks. Our team mainly consists of auxiliary midwives who could benefit from advanced training. Also, ensuring a steady supply of essential medicines and consumables, like SP for malaria prevention, is critical. If we can strengthen our staff’s capabilities and ensure the availability of medicines, it would greatly improve our care for premature babies |
| Colma1 major | Improvement in care of preterm birth | n/a | person 1 | Mobility is less felt in the city compared to rural areas. In my three years here, only a few staff have been reassigned or retired. We do get new staff, though they might not be up to date with training. | person 1 | 675 Mobility is less felt in the city compared to rural areas. In my three years here, only a few staff have been reassigned or retired. We do get new staff, though they might not be up to date with training. |
| Colma1 major | Improvement in care of preterm birth | n/a | person 1 | We focus on internal continuous training. It’s crucial for maintaining the quality of care, but it's not always sufficient. Active participation in training leads to better results. We try to keep our staff updated and well-informed on managing premature births. | person 1 | 676 We focus on internal continuous training. It’s crucial for maintaining the quality of care, but it's not always sufficient. Active participation in training leads to better results. We try to keep our staff updated and well-informed on managing premature births. |
| Colma1 major | Improvement in care of preterm birth | n/a | person 1 | Absolutely, socio-cultural constraints are a significant element. The psychological state of a mother is crucial for maintaining a pregn/acy. A mother who carries her pregn/acy to term typically does so in a peaceful and supportive environment, indicating her mental well-being. However, if she is in a disruptive environment with many disturbances that affect her peace of mind, it can negatively impact her pregn/acy. Such stressors can lead to complications, including premature birth. I believe these factors are very important to consider. | person 1 | 677 Absolutely, socio-cultural constraints are a significant element. The psychological state of a mother is crucial for maintaining a pregn/acy. A mother who carries her pregn/acy to term typically does so in a peaceful and supportive environment, indicating her mental well-being. However, if she is in a disruptive environment with many disturbances that affect her peace of mind, it can negatively impact her pregn/acy. Such stressors can lead to complications, including premature birth. I believe these factors are very important to consider. |
| Colma1 maternity manager | Improvement in care of preterm birth | n/a | person 1 | The key to improvement is to focus on individual counseling and refocused prenatal care (CPN). We need to understand and address women's problems early on. For instance, we should promptly detect and treat conditions like urinary infections and malaria, which can lead to premature births or late abortions. In our center, we ensure that treatments like sulfadoxine-pyrimethamine (SP) are supervised, which wasn't the case in urban settings initially. Since implementing supervised intake, we've seen a decrease in abortions and premature births. However, we face challenges when SP isn't available at the health center. It's also crucial to have all necessary resources for pregn/at women's care, including ultrasounds and other diagnostic tools. A welcoming environment for women to share their socio-cultural concerns is important, and when necessary, referring them to sociologists. Additionally, women with a history of premature birth or late abortion should be referred to a gynecologist for further assessment, such as the need for cerclage. The quality of CPN is more important than the quantity. Even if we can only manage 15 to 20 women but provide thorough care, it's more beneficial. We aim to do a good job, knowing that even if challenges arise, we have done our best. | person 1 | 678 The key to improvement is to focus on individual counseling and refocused prenatal care (CPN). We need to understand and address women's problems early on. For instance, we should promptly detect and treat conditions like urinary infections and malaria, which can lead to premature births or late abortions. In our center, we ensure that treatments like sulfadoxine-pyrimethamine (SP) are supervised, which wasn't the case in urban settings initially. Since implementing supervised intake, we've seen a decrease in abortions and premature births. However, we face challenges when SP isn't available at the health center. It's also crucial to have all necessary resources for pregn/at women's care, including ultrasounds and other diagnostic tools. A welcoming environment for women to share their socio-cultural concerns is important, and when necessary, referring them to sociologists. Additionally, women with a history of premature birth or late abortion should be referred to a gynecologist for further assessment, such as the need for cerclage. The quality of CPN is more important than the quantity. Even if we can only manage 15 to 20 women but provide thorough care, it's more beneficial. We aim to do a good job, knowing that even if challenges arise, we have done our best. |
| Colma1 maternity manager | Improvement in care of preterm birth | n/a | person 1 | Training in prematurity management would be beneficial. Some health workers are resistant to self-training in this area. Regular retraining and supervision, including case reviews, would help improve their skills and knowledge | person 1 | 679 Training in prematurity management would be beneficial. Some health workers are resistant to self-training in this area. Regular retraining and supervision, including case reviews, would help improve their skills and knowledge |
| Colma1 maternity manager | Improvement in care of preterm birth | n/a | person 1 | Infection control is a significant issue here. We often see that women who come in don't take adequate sanitary measures, like laying out their loincloths before lying down. Some patients even share items like porridge, which can spread infections. We need to address these practices to improve hygiene. In the past, when I worked in the dining area, I didn't always wear the necessary protective gear, but now I understand its importance. Every piece of equipment, from blouses to boots, must be thoroughly cleaned and sanitized.After we revamped our room, I took pride in maintaining cleanliness and encouraged others to do the same. We're grateful for the support from the Denbalo project, which has provided us with necessary items like delivery tables. Our equipment needs are extensive, and even the delivery room requires upgrades. For example, for newborn resuscitation, I've had to improvise with a portable lamp from another room to provide some warmth to the babies. We need more suitable equipment to provide proper care in these critical situations. | person 1 | 680 Infection control is a significant issue here. We often see that women who come in don't take adequate sanitary measures, like laying out their loincloths before lying down. Some patients even share items like porridge, which can spread infections. We need to address these practices to improve hygiene. In the past, when I worked in the dining area, I didn't always wear the necessary protective gear, but now I understand its importance. Every piece of equipment, from blouses to boots, must be thoroughly cleaned and sanitized.After we revamped our room, I took pride in maintaining cleanliness and encouraged others to do the same. We're grateful for the support from the Denbalo project, which has provided us with necessary items like delivery tables. Our equipment needs are extensive, and even the delivery room requires upgrades. For example, for newborn resuscitation, I've had to improvise with a portable lamp from another room to provide some warmth to the babies. We need more suitable equipment to provide proper care in these critical situations. |
| Colma1 maternity manager | Improvement in care of preterm birth | n/a | person 1 | For instance, we had a training program by Pathfinder for all staff in maternity care, including midwives, on family planning. However, some of them left for various reasons. To tackle the issue of training in prematurity, I believe national-level training is more effective. Regular refresher courses should be organized for existing staff, and new staff coming from schools should be trained by those already on the ground. This process should be well-documented.Periodic training could be done annually for newcomers, in addition to the retraining of senior staff. Even if some trained personnel are assigned elsewhere, like from Ouaga to here, they can bring their knowledge. But the mindset needs to change; training shouldn’t be seen just as a means to earn per diem. It should be valued for the knowledge it imparts. Since I finished school, I haven't received any specific training on prematurity from the district, which indicates a gap in our training approach. | person 1 | 681 For instance, we had a training program by Pathfinder for all staff in maternity care, including midwives, on family planning. However, some of them left for various reasons. To tackle the issue of training in prematurity, I believe national-level training is more effective. Regular refresher courses should be organized for existing staff, and new staff coming from schools should be trained by those already on the ground. This process should be well-documented.Periodic training could be done annually for newcomers, in addition to the retraining of senior staff. Even if some trained personnel are assigned elsewhere, like from Ouaga to here, they can bring their knowledge. But the mindset needs to change; training shouldn’t be seen just as a means to earn per diem. It should be valued for the knowledge it imparts. Since I finished school, I haven't received any specific training on prematurity from the district, which indicates a gap in our training approach. |
| Farakan focus group | Improvement in care of preterm birth | n/a | person 3 | Training staff on essential actions during childbirth and care of premature babies is key. Having a brief, summarized training that can be quickly referenced will help improve care while waiting for higher-level intervention. | person 3 | 682 Training staff on essential actions during childbirth and care of premature babies is key. Having a brief, summarized training that can be quickly referenced will help improve care while waiting for higher-level intervention. |
| Farakan focus group | Improvement in care of preterm birth | n/a | person 4 | Real awareness and dynamic engagement from health workers from the moment they arrive at health centers is crucial. Well-trained agents can significantly reduce the incidence of prematurity through effective awareness. | person 4 | 683 Real awareness and dynamic engagement from health workers from the moment they arrive at health centers is crucial. Well-trained agents can significantly reduce the incidence of prematurity through effective awareness. |
| Farakan focus group | Improvement in care of preterm birth | n/a | person 8 | The focus should be on training health workers comprehensively. | person 8 | 684 The focus should be on training health workers comprehensively. |
| Farakan focus group | Improvement in care of preterm birth | n/a | person 7 | Awareness is needed on both sides, among health workers and the community. That's an important aspect. | person 7 | 685 Awareness is needed on both sides, among health workers and the community. That's an important aspect. |
| Farakan focus group | Improvement in care of preterm birth | n/a | person 3 | Additionally, we need an adequate technical platform. Training is important, but without the proper equipment and facilities, we can't achieve our goals. | person 3 | 686 Additionally, we need an adequate technical platform. Training is important, but without the proper equipment and facilities, we can't achieve our goals. |
| Farakan focus group | Improvement in care of preterm birth | n/a | person 5 | As others have said, we need both training for health workers and the right technical platform to properly care for premature babies. | person 5 | 687 As others have said, we need both training for health workers and the right technical platform to properly care for premature babies. |
| Farakan focus group | Improvement in care of preterm birth | n/a | person 2 | Strengthening surveillance during prenatal care (CPN) and requesting additional exams to detect and prevent potential complications is essential | person 2 | 688 Strengthening surveillance during prenatal care (CPN) and requesting additional exams to detect and prevent potential complications is essential |
| Farakan focus group | Improvement in care of preterm birth | n/a | person 2 | By conducting thorough exams and providing appropriate care, we can prevent issues like hypertension, which can lead to premature birth. | person 2 | 689 By conducting thorough exams and providing appropriate care, we can prevent issues like hypertension, which can lead to premature birth. |
| Farakan focus group | Improvement in care of preterm birth | n/a | person 3 | To add, it's about preventive measures during pregn/acy by detecting and treating associated pathologies. We also need to improve our delivery rooms. Proper temperature control is crucial, as newborns are highly susceptible to hypothermia. Adapted rooms would offer better immediate care and increase survival chances for premature babies | person 3 | 690 To add, it's about preventive measures during pregn/acy by detecting and treating associated pathologies. We also need to improve our delivery rooms. Proper temperature control is crucial, as newborns are highly susceptible to hypothermia. Adapted rooms would offer better immediate care and increase survival chances for premature babies |
| Farakan major | Improvement in care of preterm birth | n/a | person 1 | Improvement hinges on equipment and enhancing staff skills. With the right technical platform and well-trained staff, I believe we can manage better | person 1 | 691 Improvement hinges on equipment and enhancing staff skills. With the right technical platform and well-trained staff, I believe we can manage better |
| Farakan major | Improvement in care of preterm birth | n/a | person 1 | It's difficult to prevent staff turnover. For example, I've been here 11 years and am now retiring. We can't stop someone from retiring. The solution might be regular training, so skills remain even as some staff leave. Recycling knowledge is also necessary. | person 1 | 692 It's difficult to prevent staff turnover. For example, I've been here 11 years and am now retiring. We can't stop someone from retiring. The solution might be regular training, so skills remain even as some staff leave. Recycling knowledge is also necessary. |
| Farakan major | Improvement in care of preterm birth | n/a | person 1 | Yes, it's possible. The environment plays a significant role | person 1 | 693 Yes, it's possible. The environment plays a significant role |
| Farakan major | Improvement in care of preterm birth | n/a | person 1 | In environments where people don't understand the situation of small babies, there's often a belief they won't survive. This leads to reluctance in following medical referrals. We encourage them, explaining that with proper care, these babies can survive and even thrive. I have a nephew who was premature and is now a strong firefighter | person 1 | 694 In environments where people don't understand the situation of small babies, there's often a belief they won't survive. This leads to reluctance in following medical referrals. We encourage them, explaining that with proper care, these babies can survive and even thrive. I have a nephew who was premature and is now a strong firefighter |
| Farakan major | Improvement in care of preterm birth | n/a | person 1 | "[Laughs] Yes, he is strong | person 1 | 695 "[Laughs] Yes, he is strong |
| Farakan major | Improvement in care of preterm birth | n/a | person 1 | Exactly. We need to educate the community to accept premature babies. | person 1 | 696 Exactly. We need to educate the community to accept premature babies. |
| Farakan major | Improvement in care of preterm birth | n/a | person 1 | That's a big issue, yes | person 1 | 697 That's a big issue, yes |
| Farakan major | Improvement in care of preterm birth | n/a | person 1 | We see it here. Many refuse to follow referrals, but we insist on them | person 1 | 698 We see it here. Many refuse to follow referrals, but we insist on them |
| Farakan maternity manager | Improvement in care of preterm birth | n/a | person 1 | Effective care begins with preventing the causes of prematurity, such as malaria and infections. Raising awareness among women and early detection of pathologies are crucial steps | person 1 | 699 Effective care begins with preventing the causes of prematurity, such as malaria and infections. Raising awareness among women and early detection of pathologies are crucial steps |
| Farakan maternity manager | Improvement in care of preterm birth | n/a | person 1 | Yes, these include malaria, malnutrition, hypertension, anemia, infections, especially urinary infections, etc. | person 1 | 700 Yes, these include malaria, malnutrition, hypertension, anemia, infections, especially urinary infections, etc. |
| Farakan maternity manager | Improvement in care of preterm birth | n/a | person 1 | Qualified staff are essential for improving care. Training is crucial for effective management of premature births | person 1 | 701 Qualified staff are essential for improving care. Training is crucial for effective management of premature births |
| Farakan maternity manager | Improvement in care of preterm birth | n/a | person 1 | Yes, staff need to be aware of actions that can improve care. For example, if there's a threat, like premature labor, staff should know immediate actions, like making the lungs mature or clearing airways for babies who can't breathe. A premature baby with an associated infection requires special care. | person 1 | 702 Yes, staff need to be aware of actions that can improve care. For example, if there's a threat, like premature labor, staff should know immediate actions, like making the lungs mature or clearing airways for babies who can't breathe. A premature baby with an associated infection requires special care. |
| Farakan maternity manager | Improvement in care of preterm birth | n/a | person 1 | Recurring training, even if it's refresher courses, is necessary | person 1 | 703 Recurring training, even if it's refresher courses, is necessary |
| Farakan maternity manager | Improvement in care of preterm birth | n/a | person 1 | Often, not everyone knows how to handle premature births. For instance, in a premature delivery case, knowing the appropriate treatment is crucial. | person 1 | 704 Often, not everyone knows how to handle premature births. For instance, in a premature delivery case, knowing the appropriate treatment is crucial. |
| Farakan maternity manager | Improvement in care of preterm birth | n/a | person 1 | Yes, exactly. Early diagnosis and appropriate treatment are key | person 1 | 705 Yes, exactly. Early diagnosis and appropriate treatment are key |
| Farakan maternity manager | Improvement in care of preterm birth | n/a | person 1 | Diagnosing involves assessing symptoms, ensuring regular preventive measures like SP intake, mosquito net usage, and proper nutrition. During questioning, pelvic pain and other symptoms should be probed. | person 1 | 706 Diagnosing involves assessing symptoms, ensuring regular preventive measures like SP intake, mosquito net usage, and proper nutrition. During questioning, pelvic pain and other symptoms should be probed. |
| Accart-ville focus group | Others | n/a | n/a | n/a | n/a | 707 NA |
| Accart-ville CMU manager | Others | n/a | person 1 | I think we've covered everything [laughs] | person 1 | 708 I think we've covered everything [laughs] |
| Accart-ville CMU manager | Others | n/a | person 1 | In terms of prematurity, it's defined as a gestational age between 28 weeks and less than 37 weeks. Births before 28 weeks are considered late abortions. From my experience, babies born before 28 weeks may initially seem viable, but continuing care is difficult due to inadequate resources and technical support. Although these children may initially survive, they often succumb after a few days or weeks due to lack of adequate care | person 1 | 709 In terms of prematurity, it's defined as a gestational age between 28 weeks and less than 37 weeks. Births before 28 weeks are considered late abortions. From my experience, babies born before 28 weeks may initially seem viable, but continuing care is difficult due to inadequate resources and technical support. Although these children may initially survive, they often succumb after a few days or weeks due to lack of adequate care |
| Accart-ville CMU manager | Others | n/a | person 1 | Thank you as well | person 1 | 710 Thank you as well |
| Accart-ville maternity manager | Others | n/a | person 1 | In the questionnaire, we didn't focus much on the causes, etiologies, and risk factors of prematurity, nor on its management. One overlooked impact is on the mother of the premature child. She often has to abandon her activities to care for her child, which affects her income sources. Addressing these aspects could significantly enhance the care of premature newborns | person 1 | 711 In the questionnaire, we didn't focus much on the causes, etiologies, and risk factors of prematurity, nor on its management. One overlooked impact is on the mother of the premature child. She often has to abandon her activities to care for her child, which affects her income sources. Addressing these aspects could significantly enhance the care of premature newborns |
| CMA Do focus group | Others | n/a | person 9 | Strengthening our service, especially the kangaroo unit that cares for young children, is crucial. While it might be challenging in terms of personnel, focusing on training to enhance this unit would be beneficial. | person 9 | 712 Strengthening our service, especially the kangaroo unit that cares for young children, is crucial. While it might be challenging in terms of personnel, focusing on training to enhance this unit would be beneficial. |
| CMA Do focus group | Others | n/a | person 4 | It's essential to consider all aspects. Having well-trained staff without adequate equipment, or having motivated staff with subpar facilities, will impact the quality of care. The motivation of staff isn’t only about money; the fin/acial aspect, however, is also important. We need a balanced approach, not focusing solely on one aspect but addressing equipment, training, and motivation collectively. | person 4 | 713 It's essential to consider all aspects. Having well-trained staff without adequate equipment, or having motivated staff with subpar facilities, will impact the quality of care. The motivation of staff isn’t only about money; the fin/acial aspect, however, is also important. We need a balanced approach, not focusing solely on one aspect but addressing equipment, training, and motivation collectively. |
| CMA Do focus group | Others | n/a | person 6 | Establishing a neonatology department would be extremely beneficial for us. | person 6 | 714 Establishing a neonatology department would be extremely beneficial for us. |
| CMA Do focus group | Others | n/a | person 2 | In my experience, especially in the delivery room, having defective equipment is a significant challenge. If we had better resuscitation equipment, many babies could be saved. Currently, the lack of quality equipment means we are unable to effectively resuscitate and care for premature babies. This is particularly true for very premature infants, who often don't survive the transfer to better-equipped facilities. We desperately need better equipment in the delivery room for immediate care and resuscitation of newborns. | person 2 | 715 In my experience, especially in the delivery room, having defective equipment is a significant challenge. If we had better resuscitation equipment, many babies could be saved. Currently, the lack of quality equipment means we are unable to effectively resuscitate and care for premature babies. This is particularly true for very premature infants, who often don't survive the transfer to better-equipped facilities. We desperately need better equipment in the delivery room for immediate care and resuscitation of newborns. |
| CMA Do focus group | Others | n/a | person 6 | Thank you. | person 6 | 716 Thank you. |
| CMA Do focus group | Others | n/a | person 9 | Thank you. | person 9 | 717 Thank you. |
| Gynecologist 1 | Others | n/a | person 1 | What's important to me, and we've touched on it a bit, is the need for decentralizing support for premature births. As I mentioned, in the entire Hauts-Bassins region, we only have one neonatology unit. This unit serves all districts, including Houndé, Ourodara, Dô, Dafra, Léna, Karangasso Sambla, Ndorola, and others. If we could start decentralizing these services, perhaps even creating neonatology units in places like Dô and Dafra, it would greatly alleviate the burden on Souro SANOU and provide more opportunities for these children | person 1 | 718 What's important to me, and we've touched on it a bit, is the need for decentralizing support for premature births. As I mentioned, in the entire Hauts-Bassins region, we only have one neonatology unit. This unit serves all districts, including Houndé, Ourodara, Dô, Dafra, Léna, Karangasso Sambla, Ndorola, and others. If we could start decentralizing these services, perhaps even creating neonatology units in places like Dô and Dafra, it would greatly alleviate the burden on Souro SANOU and provide more opportunities for these children |
| Gynecologist 1 | Others | n/a | person 1 | No problem. | person 1 | 719 No problem. |
| Gynecologist 2 | Others | n/a | person 1 | I believe we've covered the essentials. However, I'd like to revisit our equipment for caring for children, especially premature babies. Our kangaroo unit only operates for a few hours during the day. In the evening, we're short-staffed and lack adequate equipment. While our current approach, including advice on keeping the child warm and properly fed until they gain weight, is fair, there's room for improvement. We're not equipped to hospitalize sick premature newborns, which is a significant shortcoming. We often have to send these children to the hospital, but the hospital is frequently overwhelmed with premature babies from various regions. Bobo alone can fill the neonatology unit. If other areas also send their cases here, it exacerbates the problem. Decentralizing care, perhaps by establishing a pediatric unit at the CMA de Dô, could greatly improve our ability to provide proper care | person 1 | 720 I believe we've covered the essentials. However, I'd like to revisit our equipment for caring for children, especially premature babies. Our kangaroo unit only operates for a few hours during the day. In the evening, we're short-staffed and lack adequate equipment. While our current approach, including advice on keeping the child warm and properly fed until they gain weight, is fair, there's room for improvement. We're not equipped to hospitalize sick premature newborns, which is a significant shortcoming. We often have to send these children to the hospital, but the hospital is frequently overwhelmed with premature babies from various regions. Bobo alone can fill the neonatology unit. If other areas also send their cases here, it exacerbates the problem. Decentralizing care, perhaps by establishing a pediatric unit at the CMA de Dô, could greatly improve our ability to provide proper care |
| Gynecologist 2 | Others | n/a | person 1 | Thank you as well. You're welcome | person 1 | 721 Thank you as well. You're welcome |
| MCD | Others | n/a | person 1 | I think we've covered the main points. The primary issue for us is the lack of equipment. If we have the necessary equipment, we can find and train the staff. That's our biggest limitation right now. We do have gynecologists and are expecting a pediatrician next month, so we have the professional capabilities. It’s really about having the right equipment and maintaining our skills since routine work can sometimes lead to the loss of certain best practices. That's the main area of focus for us. | person 1 | 722 I think we've covered the main points. The primary issue for us is the lack of equipment. If we have the necessary equipment, we can find and train the staff. That's our biggest limitation right now. We do have gynecologists and are expecting a pediatrician next month, so we have the professional capabilities. It’s really about having the right equipment and maintaining our skills since routine work can sometimes lead to the loss of certain best practices. That's the main area of focus for us. |
| MCD | Others | n/a | person 1 | Thank you. | person 1 | 723 Thank you. |
| CMA Do maternity care unit manager | Others | n/a | person 1 | I believe we've covered everything. However, I'd like to revisit the topic of fundal height, which is important but not definitive for assessing birth weight. Sometimes, the uterine height may seem small relative to the pregn/acy's age. Accurate evaluation really requires examining the newborn at birth. This brings me to the importance of training healthcare workers in managing more mature births and providing the right care and advice to mothers. While we did cover this in basic training, it's something that needs to be revisited and reinforced in practice | person 1 | 724 I believe we've covered everything. However, I'd like to revisit the topic of fundal height, which is important but not definitive for assessing birth weight. Sometimes, the uterine height may seem small relative to the pregn/acy's age. Accurate evaluation really requires examining the newborn at birth. This brings me to the importance of training healthcare workers in managing more mature births and providing the right care and advice to mothers. While we did cover this in basic training, it's something that needs to be revisited and reinforced in practice |
| CMA Do maternity care unit manager | Others | n/a | person 1 | Exactly. Although it was part of our basic education, it's crucial to continually update this knowledge on the field. Otherwise, the register doesn't capture this information adequately. | person 1 | 725 Exactly. Although it was part of our basic education, it's crucial to continually update this knowledge on the field. Otherwise, the register doesn't capture this information adequately. |
| CMA Do maternity care unit manager | Others | n/a | person 1 | Thank you as well | person 1 | 726 Thank you as well |
| Colma1 focus group | Others | n/a | person 1 | n/a | person 1 | 727 NA |
| Colma1 major | Others | n/a | person 1 | Regarding premature birth, no, there's nothing else I can think of right now. | person 1 | 728 Regarding premature birth, no, there's nothing else I can think of right now. |
| Colma1 major | Others | n/a | person 1 | Thank you, thank you. | person 1 | 729 Thank you, thank you. |
| Colma1 maternity manager | Others | n/a | person 1 | Thank you too. | person 1 | 730 Thank you too. |
| Colma1 maternity manager | Others | n/a | person 1 | Okay. | person 1 | 731 Okay. |
| Colma1 maternity manager | Others | n/a | person 1 | It's normal. | person 1 | 732 It's normal. |
| Farakan focus group | Others | n/a | person 3 | Off the air. | person 3 | 733 Off the air. |
| Farakan major | Others | n/a | person 1 | No, everything's fine [laughs], thank you very much | person 1 | 734 No, everything's fine [laughs], thank you very much |
| Farakan major | Others | n/a | person 1 | Thank you as well. | person 1 | 735 Thank you as well. |
| Farakan maternity manager | Others | n/a | person 1 | Okay, we have the reports. It's easy to find them. We also have birth records related to weight. | person 1 | 736 Okay, we have the reports. It's easy to find them. We also have birth records related to weight. |
