# Supplementary material for: Perceptions and practices in urban Burkina Faso: a qualitative study on gestational age estimation among health workers
Source: Int J Qual Stud Health Well-being. 2025 Jul 4;20(1):2508421. doi: 10.1080/17482631.2025.2508421 (PMC12231294; doi:10.1080/17482631.2025.2508421)
Supplement: A_qualitative_study_with_autors_revision_1_ (1).docx [file ZQHW_A_2508421_SM4863.docx]

Perceptions and Practices in Urban Burkina Faso: A Qualitative Study on Gestational Age Estimation Among Health Workers

Anderson Compaore^1†^, Moctar Ouedraogo^1†^, Cheick Ahmed Ouattara^1†^, Lionel Olivier Ouedraogo^2,3^, Lishi Deng^2^, Nicolas Sawadogo^2^ , Carl Lachat^2^ , Laéticia Celine Toe ^2,4^, and Trenton Dailey-Chwalibóg^1,2†*^

1 Agence de Formation de Recherche et d’Expertise en Santé pour l’Afrique (AFRICSanté), Bobo-Dioulasso 01 BP 298, Burkina Faso; discompa4523@gmail.com (A.C.); ouattaracheickahmed@gmail.com (C.A.O.); obmoctar @gmail.com (M.O.)

2 Department of Food Technology, Safety and Health, Faculty of Bioscience Engineering, Ghent University, 9000 Ghent, Belgium; lionel.olivier.ouedraogo@gmail.com (L.O.O.); pegdwende.sawadogo@ugent.be (N.S.); lishi.deng@ugent.be (L.D.); laeticiaceline.toe@ugent.be (L.C.T.); carl.lachat@ugent.be (C.L.)

3 Centre Muraz, Bobo-Dioulasso 01 BP 390, Burkina Faso

4 Unité Nutrition et Maladies Métaboliques, Institut de Recherche en Sciences de la Santé (IRSS), Bobo-Dioulasso 01 BP 545, Burkina Faso

* Correspondence: trenton.daileychwalibog@ugent.be (T.D.-C.)

† These authors contributed equally to the work.

[Number of words with abstract: 7926]

[Number of words without abstract: 7626]

**Abstract**

**Purpose**: Accurate gestational age estimation is essential for effective prenatal care and the management of preterm births, which remain a major contributor to infant mortality in sub-Saharan Africa. In 2023, the DenBalo study in Burkina Faso aimed to examine biological vulnerability in preterm compared to full-term newborns. However, the study recorded fewer preterm births than anticipated based on routine health center statistics. To investigate this discrepancy, a qualitative study was conducted to understand how healthcare workers assess gestational age in urban Burkina Faso.

**Methods**: Data were collected through ten in-depth interviews and four focus group discussions with health workers across four health centers in Bobo-Dioulasso. Participants were selected based on their experience, specialization, responsibilities, and involvement in maternal and child health. Thematic analysis identified five central themes: definitions of preterm birth, methods used for gestational age estimation, registration and incidence of preterm births, challenges in care provision, and proposed improvements.

**Results**: While some health workers define it as birth before 37 weeks, others rely on a birth weight threshold of <2.5 kg. According to respondents, gestational age is often estimated using the last menstrual period although the method is frequently considered unreliable. Respondents expressed a preference for early ultrasound due to its accuracy; however, its high cost and limited accessibility often necessitate reliance on fundal height measurements, despite their known limitations. For some health professionals, documentation practices vary, with inconsistent recording of preterm births. Key challenges include difficulties in data collection, shortages of resources and equipment, and parental reluctance to seek specialized care. So, respondents stated that improving care for preterm newborns requires investment in community awareness, facility infrastructure, and ongoing staff training.

**Conclusion**: Standardizing gestational age estimation methods and enhancing data recording could improve the accuracy of preterm birth surveillance and contribute to reducing neonatal mortality in resource-constrained settings.

**Keywords**: gestational age, preterm birth, prenatal care, DenBalo, SpacY

# Introduction

Accurate gestational age estimation is crucial for prenatal care, as it plays a pivotal role in tracking fetal development, scheduling prenatal tests, and predicting the due date of the baby (Kramer, 2013). Additionally, it holds significance in the management of early labor and protracted gestation, assessment of complication risks, informed decision-making in high-risk pregnancies, and guiding medical interventions (National Institute for Health Care and Excellence, 2015; Yuce et al., 2015). It allows for the precise categorization of low-birth-weight infants as either preterm or small for gestational age (Wylie et al., 2013). In cases of preterm birth, precise gestational age assessment facilitates personalized neonatal care, particularly for extreme prematurity, and supports informed decision-making. Furthermore, it contributes to developmental monitoring of preterm infants, advances in neonatal research, and the development of public health strategies (A. S. Morgan et al., 2022).

Globally, preterm birth is a leading cause of child mortality under the age of five, accounting for one-third of neonatal deaths (Liu et al., 2015; Perin et al., 2022) ). Alarmingly, 65% of preterm births occurs in South Asia and sub-Saharan Africa (Ohuma et al., 2023) and 80% of deaths due to preterm birth complications occur in low- and middle-income countries (World Health Organization, 2023a) where preterm births are especially concerning. Efforts must focus on both prevention and improving neonatal care to increase infant survival rates.

With this goal in mind, the DenBalo (Describing and Comparing Biological Vulnerability in Small Vulnerable Neonates versus Healthy Community Controls in Urban Burkina Faso: Gut Microbiota, Immune System, and Breastmilk Assembly and Development in the First Days and Weeks of Life) project was initiated in early 2023 (Ouédraogo et al., 2024).

The DenBalo study initially aimed to include 50 preterm and 50 full-term infants. Based on data acquired from the health information systems at the study site, the project expected to reach that sample size within six months, at a rate of 8-12 preterm births per month. However, from April 7th, 2023 to October 6th, 2023, we observed an unexpected trend: only four preterm births occurred, compared to the 48 expected based on prematurity prevalence data in urban Bobo-Dioulasso (District sanitaire de Do, 2023).

This unexpected deviation led us to investigate the potential causes behind the low incidence of preterm births observed in our study. We considered whether the use of the Alliance for Maternal and Newborn Health Improvement (AMANHI) late-pregnancy gestational age dating technique—which estimates gestational age based on Transcerebellar Diameter (TCD) and Femur Length (FL) measurements between 24 weeks and 29 weeks 6 days of gestation—could explain the difference in the identification of preterm births. This prompted a thorough examination of the factors influencing both the reporting and identification of preterm birth.

Consequently, we conducted a qualitative study to assess the knowledge and practices of healthcare personnel in urban Burkina Faso regarding gestational age assessment and preterm birth identification; this was complemented with in depth quantitative study published separately (Ouattara et al., 2025). Indeed, inaccurate identification of preterm births can lead to two main consequences. First, the misidentification of preterm labor or preterm birth prevents the provision of adequate care for both the mother and the newborn, potentially contributing to an increase in perinatal mortality. Second, it results in the consolidation of erroneous statistics, which guide health service development and resource allocation, such as neonatal care units, incubators, and essential medicines, thus compromising the care of vulnerable newborns. The study focused on investigating the key factors contributing to the discrepancy between the preterm birth rates reported by the district health statistics office and the lower occurrence observed by DenBalo study sonographers. To achieve this, we assessed the understanding and practical approaches used by urban health providers to define and measure preterm births and identified the challenges and resource needs, both human and material, faced by different health centers in managing preterm births.

# Materials and methods

## Study design and setting

The health system in Burkina Faso health is organized as a three-tiered pyramid. The first tier includes the Centre de Santé et de Promotion Sociale (CSPS) which provides basic healthcare services, and the Centre Médical avec Antenne Chirurgicale (CMA), which offers more advanced services, including surgery. First tier health centers are organised geographically and administratively into a Health District. The second tier consists of the Centre Hospitalier Regional (CHR), which acts as referral center for the CMA. The third tier includes the Centre Hospitalier Universitaire (CHU), like that of Souro Sanou in Bobo-Dioulasso, which delivers the highest level of specialized care. However, in Bobo-Dioulasso, the second tier is absent, and patients from the CSPS are referred directly to the CHU.

This study employed a qualitative descriptive design and was conducted in four health centers, including three CSPS and one CMA in the Health District of Dô. Through semi-structured interviews with key informants and open-ended questions, the study explored the perspectives and experiences of healthcare workers regarding preterm birth diagnosis and management. The interviews were conducted from August 9th to August 24th, 2024.

## Participants and enrolment

We conducted ten in-depth interviews and four focus group discussions. The interview involved, in each healthcare facility, the head of the facility, the head of the maternity ward, and consultant gynecologists from the CMA. Generally, participants included two gynecologists, two general practitioners, two nurses, and four midwives.

For the focus groups, all other maternity staff participated, including midwives, nurses, and auxiliary birth attendants. Eligibility was based on their involvement in maternal and child health within the context of the Denbalo study. To ensure a diverse range of profiles, participants were selected to represent varying levels of professional experience, specialization, and responsibilities within the health district. A detailed description of the study was provided to all participants, and informed consent was obtained prior to participation. Confidentiality and anonymity were strictly maintained throughout the research process.

## Data collection

Data collection was guided by a structured interview tool (see Appendix A) and involved both individual interviews and focus group discussions with general practitioners, gynecologists, midwives, nurses, and maternity unit managers. These activities aimed to explore clinical practices and perceptions related to the definition, measurement, and management of preterm births in Burkina Faso. A trained sociologist—independent from the research team and unaffiliated with the participating health centers—conducted all interviews and focus groups. Prior to each session, participants were informed about the purpose of the study, assured of confidentiality, and asked to provide informed consent. Discussions addressed the classification and documentation of preterm births, challenges faced by healthcare providers, available resources, and perceived opportunities for improving care. Individual, semi-structured, audio-recorded interviews were conducted in French at the four participating health centers, in private meeting rooms to ensure confidentiality and minimize interruptions.

## Data analysis

To ensure a systematic and rigorous approach to identifying, organizing, and interpreting patterns of meaning within the qualitative data (Braun & Clarke, 2006; Pope, 2000), a thematic analysis was conducted to explore key issues related to gestational age estimation and preterm birth management in urban health centers in Burkina Faso. Data from individual interviews and focus group discussions were transcribed, coded, and subsequently organized into recurring themes. In addition to traditional manual thematic coding, a novel combination of AI-assisted qualitative analysis tools was employed to deepen analytical insight and enhance methodological rigor. This integrative approach improved the consistency of theme identification and facilitated the emergence of unexpected patterns across interviews, which were subsequently validated and interpreted through iterative review. The detailed steps of this process are outlined below.

After the interviews were completed, the audio recordings were transcribed and anonymized using NVivo software (Version 14). The transcriptions, initially in Rich Text Format (RTF), were then converted to .docx format and organized according to the questions in the interview guide. To improve punctuation and capitalization, ChatGPT 4.0 (prompt: “Please correct spelling and capitalization”) was used, followed by translation from French to English using Google Translate. The readability of the translations was further enhanced with ChatGPT 4.0 (prompt: “Please improve for readability”). This process resulted in a structured file containing the question, the original French response, the improved French response, and the translated English response. The finalized English responses were compiled into an Excel file, and imported into Python for algorithmic analysis.

We employed a combination of Python libraries and network analysis to examine and visualize the relationships between various textual responses. This is a novel and enhanced method, previously validated and applied in earlier studies (Hu et al., 2022; Prakash et al., 2022). The methodology can be summarized in the following steps:

1. Natural Language Processing (NLP) with spaCy: The spaCy library, specifically the ‘en_core_web_lg’ model, was used for parsing the textual responses. This involved processing each response in the dataset to extract tokens, lemmas, and parts of speech. The parsed documents were then added to the dataset, along with the extracted tokens, lemmas, and parts of speech.
2. Stopword removal: To refine the analysis, stopwords (commonly used words that generally do not contain important meaning) were identified and removed using spaCy’s predefined list of English stopwords.
3. Network analysis with NetworkX: The responses were then analyzed using NetworkX, a library for the creation, manipulation, and study of complex networks. A graph (raw_G) was constructed where each node represented a parsed document, and edges between nodes were weighted based on the similarity of the responses. The similarity between responses was calculated using spaCy’s similarity function.
4. Edge filtering and graph refinement: We applied a threshold to filter out edges with a weight below a certain cutoff value, allowing us to focus on stronger relationships between responses. The graph (strong_G) was then reconstructed with these filtered edges.
5. Visualization: The refined graph was visualized using NetworkX and Matplotlib. The Fruchterman-Reingold force-directed algorithm (nx.fruchterman_reingold_layout) was used to calculate node positions, aiming for a clear and informative layout.
6. Additional visualization techniques: To enhance the visual representation, further visualizations were conducted, adjusting aspects such as node size, edge color, and label positioning to make the graph more interpretable.

This methodology allowed for a comprehensive and nuanced analysis of the dataset, leveraging the strengths of NLP, network analysis, and visualization techniques to uncover patterns and relationships within the responses related to each topic (see Figure 1 and 2). Note: the topic “assessment of preterm birth” includes several subtopics, each represented by its own graph (see Figure 2). Themes identified through NLP-assisted analysis were subsequently validated using traditional thematic analysis to ensure analytical rigor and contextual robustness. To further ground the findings, we returned to the original list of exhaustive responses and extracted illustrative quotes that exemplify each validated theme, reinforcing the interpretive depth of the analysis. The entire process is illustrated in Figure 3.

Based on the interview guide, the ten themes were initially identified, which were further consolidated into five key themes based on the similarity in responses and objectives of study. These themes include the definition of preterm birth (1), the assessment of preterm birth (2), the registration and incidence of preterm births (3), the challenges encountered in preterm birth management (4) and improvement in preterm birth care (5) (see Figure 4). The full thematic analysis of all ten themes can be found in the supplementary material.

## Ethical Considerations

The study was approved by Comité d’Ethique Institutionnel de Recherhche en Sciences de la Santé (CEIRES) of the Institut de Recherche en Sciences de la Santé (IRSS) Direction Régionale de l’Ouest (protocol code R021-2024/CEIRES and the date of 06/10/2024). The confidentiality and anonymity of participants were strictly maintained throughout the research process.

- 1. **Informed consent**

The study was explained to participants, and informed consent was obtained verbally. Given the qualitative nature of the research—which involved interviews and focus group discussions in a less formal and more conversational setting—verbal consent was deemed appropriate. This approach allowed participants to engage comfortably while ensuring they fully understood the study’s purpose and procedures. All verbal consents were audio-recorded for documentation and ethical compliance.

# Results

Ten initial themes were identified based on the interview guide. These were later consolidated into five key themes, reflecting similarities in participant responses and alignment with the study’s objectives.

## Definition of preterm birth

Respondents reported that gestational age and birth weight are the two primary criteria used to define prematurity. Most participants defined preterm birth as any birth occurring between 28 and 37 weeks of gestation. As one gynecologist with 13 years of experience managing maternity ward noted, “*If the age of the pregnancy is less than 36 weeks, we classify it as preterm birth.”* Another gynecologist with 4 years of experience explained, *“In our setting, preterm birth is classically defined as births that occur between 28 weeks and 37 weeks of amenorrhea.”*

In the absence of gestational age information, some healthcare professionals reported using birth weight as an alternative criterion. However, weight cutoffs varied across health centers. One gynecologist described their approach, stating, *“We consider fetal weight; often, if it is less than 2 kilograms, we tend to classify the birth as preterm, but the primary criterion is the gestational age.”* Another participant, anurse with 1 year of experience of managing the CSPS, added, “*We also consider the weight of the child; typically, a preterm baby will weigh less than 2.5 kilograms.*” The definition based on birth weight is informal and serves as a complementary clinical indicator, especially in settings where precise pregnancy dating is challenging.

## Assessment of preterm birth

Within this theme, two subthemes were identified.

### **Methods of gestational age estimation**

Healthcare workers primarily use gestational age as a key indicator to assess prematurity, employing various methods for its estimation.

Last Menstrual Period (LMP): Participants identified significant challenges in utilizing the LMP to estimate gestational age, primarily because many women struggle to recall the precise date of their last menstrual cycle. They reported that this difficulty was frequently linked to low literacy levels, limiting women’s ability to accurately track menstrual cycles. One midwife, with four years of experience, explained: “*In Africa, recall accuracy is quite low. Out of ten patients, maybe around four know the exact date. Many women relate their periods to events or seasons, such as the corn harvest or the month of Ramadan, due to limited literacy.*” Participants emphasized that precise recall of the LMP date is uncommon, noting that women typically provide only approximate months, thus reducing the accuracy of gestational age estimation. One nurse, a manager at a primary health center (CSPS), remarked: “*They often know only the approximate month, give or take a few weeks. Recalling the exact day tends to be challenging, particularly for those with lower literacy levels.*”

Respondents indicated that although the Gestogram tool (see Figure 5) can facilitate gestational age estimation when the LMP date is known, its effectiveness largely depends on the accuracy of the provided LMP. As a midwife stated: “*The effectiveness of a gestogram largely depends on the woman accurately recalling the date of her last menstrual period*.”

Participants also noted that late initiation of antenatal care, commonly occurring in the second trimester, further complicates accurate LMP-based estimations. This practice contrasts with the World Health Organization’s recommendations advocating early antenatal consultations. One midwife reported: “*Women typically come for antenatal visits around three months into pregnancy, aligning with local traditional practices, though WHO guidelines recommend attending as soon as pregnancy is suspected*.” Additionally, a gynecologist managing a maternity ward observed: *“Women usually present around five months, in the second trimester. First-trimester visits are rare unless there are complications.”*

Ultrasound: Ultrasound, particularly in the first trimester, were highly valued by participants for their accuracy in gestational dating. However, participants noted that many women do not have an ultrasound test unless they have experienced early pregnancy complications. As explained by a maternity unit manager: “*It's actually rare for women to come in with an ultrasound already done. Usually, it's only those who have encountered health issues early in pregnancy.”*

The cost associated with ultrasounds was frequently mentioned by respondents as a significant barrier, significantly limiting its routine utilization in maternity care. Many highlighted that its consistent use remains restricted due to financial challenges. One maternity care unit manager explicitly addressed this issue by explaining: *“Ultrasound isn't always our primary method due to financial constraints faced by patients.”*

This remark underscores the practical dilemma health providers encounter, balancing clinical accuracy with the financial realities of patients, thus necessitating reliance on alternative methods such as fundal height measurement

Fundal Height Measurement: Participants described fundal height measurement as a common alternative method when the LMP date is unavailable. The procedure for estimating gestational age using fundal height was explained by a health officer specialized in obstetric and gynecological care as follows: *“We add 4 cm to fundal height measurements until the seventh month of pregnancy. After the seventh month, we add 3 cm, and after the eighth month, we add 2 cm. For example, a 20 cm fundal height measurement plus 4 cm corresponds to approximately 24 weeks of amenorrhea.”*

Some participants reported utilizing pre-established correlation tables (Appendix B), which systematically standardize fundal height measurements in cases where the LMP is unknown. A gynecologist stated: *“When using fundal height, we refer to standardized tables correlating measurements with gestational age.”*

Some respondents also emphasized the importance of supplementing fundal height measurements with thorough clinical evaluations to enhance the accuracy of gestational age estimation. They described that relying solely on fundal height could lead to imprecise results due to variations in maternal anatomy or fetal positioning. One midwife elaborated specifically on their procedure, stating: *“To estimate gestational age from fundal height, we first confirm whether the pregnancy is in the second trimester by asking if fetal movements have been perceived, as they typically begin during this period.”* Such clinical assessments, were therefore considered essential for improving estimation reliability by some participants.

### **Preferences for gestational age estimation methods**

Opinions among participants were divided regarding the priority of methods used for estimating gestational age.

Preference for early ultrasound: A recurring theme among respondents was the preference for early ultrasounds, particularly those conducted before 12 weeks of gestation, as the most precise method for dating pregnancies. As one medical doctor with 2 years experience managing the CSPS explained, *“…if we have an ultrasound from within the first twelve weeks, we prioritize it…”.* This preference is based on the well-established accuracy of early ultrasounds in estimating gestational age, as precision diminishes with advancing pregnancy. However, respondents recognized that accessibility and cost often limit its widespread use.

Preference for the LMP: Several respondents indicated a preference for using the LMP date when available, emphasizing its role in clinical practice. *“We first ask if the woman remembers her last menstrual period. We use the gestogram, which calculates the weeks of amenorrhea, adding two weeks for the maturation phase,”* explained a medical doctor with three years of experience managing the CMA. Another midwife stressed the importance of obtaining the LMP date, calling it *“the best scenario for us”* when determining gestational age. However, they acknowledged the challenge of obtaining precise recall from patients and noted that some practitioners quickly shift to alternative methods when the LMP is not immediately known.

The use of fundal height: Fundal height measurement remains a commonly used method, particularly in settings where ultrasound is unavailable. However, respondents frequently highlighted its limitations, noting that factors such as maternal body size and amniotic fluid levels can influence accuracy. A gynecologist with 13 years of experience noted, *“Fundal height can be influenced by many factors, like the amount of amniotic fluid.”* Another health officer specializing in obstetric and gynecological care added, *“…fundal height measurements can be influenced by the woman's body size. In obese women, even locating the uterus can be challenging…”.* Despite these challenges, fundal height measurement continues to serve as a practical tool in resource-limited contexts. As one medical doctor with 3 years experience of managing CMA explained, *“…in rural areas, we mainly rely on fundal height due to the lack of ultrasound facilities”.*

The findings highlight the diversity of approaches used in gestational age estimation, each with its advantages and limitations. While early ultrasound is the preferred and most accurate method, its limited accessibility leads healthcare providers to rely on alternative techniques such as LMP and fundal height measurements.

## Registration and incidence of preterm birth

Participants reported that preterm births are typically recorded in birth registers maintained at health centers. Many respondents, especially maternity managers at primary health centers (CSPS) and nurses involved directly in obstetric care, confirmed the existence of dedicated sections for noting whether a birth is preterm, alongside parameters such as gestational age and birth weight. For example, a health officer responsible for obstetric and gynecological care and maternity management at a primary health center (CSPS) highlighted their practices clearly: *“…in our birth register, we record whether a birth is preterm or not, along with the gestational age. We also note the number of low birth weight children and specify if they are preterm.”*

Conversely, other participants, particularly those working at higher-level health facilities like the medical center with surgical annex (CMA), mentioned the absence of specialized registers dedicated exclusively to preterm births. A maternity care unit manager at the CMA with nine years of professional experience noted this gap: *“No, there isn’t a specific register for that. Ideally, such details should be included in the birth register.”*

The integration of preterm birth data into monthly activity reports also differed significantly across health centers. For instance, a medical doctor with two years of experience managing a CSPS explained that their center’s reports do not systematically detail preterm birth data: *“In our monthly reports, we've noticed that preterm births aren’t always detailed. We usually just count the number of deliveries without specifying if they were preterm.”*

In contrast, another facility consistently included preterm bith details in its monthly reports, as described by an experienced midwife with eleven years of practice: *“Yes, it is. Prematurity details are included in the monthly activity reports.”*

Furthermore, participants provided varying estimates regarding the incidence of preterm births in their respective centers. A health officer managing obstetric and gynecological care at a primary health center (CSPS) provided an approximate monthly frequency: *“I would estimate it to be around 5 to 10% of our monthly deliveries.”*

Meanwhile, another midwife with five years of experience emphasized the variability in monthly incidence: *“As an estimate, the number of premature births varies. Some months, we might have two cases, and other times, there might not be any.”*

These variations and inconsistencies highlight significant challenges in accurately tracking and reporting preterm births within the health system, inevitably leading to ambiguity and reduced confidence in the reliability of reported preterm birth rates.

## Types of challenges faced during preterm birth

Challenges in prematurity data collection: Respondents identified several significant obstacles that complicate the accurate recording and classification of preterm births within their healthcare facilities. A health officer specializing in obstetric and gynecological care, with nine years of experience managing a maternity care unit at the CMA, described the challenge clearly: “*When preparing the monthly report, it's hard to trace prematurity because it's not specifically noted in the register. We can identify low birth weight, like weights less than 2,500 grams, but we don’t have enough information to distinguish between preterm births and hypotrophic cases.”* This highlights a fundamental gap in existing data collection processes, wherein critical indicators such as gestational age at birth are not routinely documented

Furthermore, limitations in current data-tracking systems compound these issues, making it challenging to reliably quantify preterm births. A medical doctor managing a CSPS for two years, echoed similar concerns, noting that existing reporting structures often only capture aggregate data: *“…our monthly reports indicate the total number of births, but not the number of preterm births. To determine that, we'd need to analyze the birth data for each individual, checking gestational age, birth weight, and other parameters from the physical examination.”*

Collectively, these accounts underline critical shortcomings within the health district's statistical systems, suggesting that currently reported statistics on preterm births are likely incomplete or insufficiently accurate for robust analysis and effective decision-making.

Lack of resources and equipment: Preterm neonates require specialized medical attention to manage critical complications such as hypothermia, respiratory distress syndrome, and infections, all common risks associated with premature births. One gynecologist, with 13 years of experience managing a maternity ward at the CMA, emphasized the vulnerability of these infants: *“Preterm babies are very fragile and often experience respiratory distress, difficulties in regulating temperature, and are prone to infections. They require close monitoring and careful treatment.”*

However, respondents frequently reported severe shortages in essential neonatal care resources, notably incubators, kangaroo mother care units, and necessary medications. A health officer managing a maternity unit at a CSPS elaborated on the implications of these shortages: *“Our main challenge is the lack of adequate care resources. We don't have a kangaroo care unit or incubators, which are essential for managing hypothermia and other issues in preterm babies.”*

Additionally, interviewees also highlighted that their facilities were insufficiently equipped to provide the necessary care for preterm infants. These resource constraints frequently forced healthcare professionals to refer infants to higher-level health facilities, as stated by midwife: *“We lack the facilities to adequately care for preterm babies, so they are usually transferred to the CHU for specialized care under lamps and to prevent infections. We don't have the capacity to treat preterm babies on-site and must rely on higher-level facilities.”*

Frequent transfers to specialized hospitals introduce further complications, as these higher-level facilities often face overcrowding. One health officer specializing in obstetric and gynecological care from a CSPS maternity unit explained the difficulties arising from these transfers: *“Even when we do evacuate, hospitals like Souro Sanou, which are equipped with incubators, often lack space, leading to infants being sent back to us with only oral prescriptions.”*

These findings clearly illustrate significant gaps in neonatal care resources, directly compromising preterm infants' survival.

Sociocultural and socioeconomic barriers to preterm birth management: Parental perceptions about preterm births pose additional challenges. Respondents reported frequent encounters with parental resistance or refusal to transfer preterm babies for advanced care at higher-level facilities. A health officer managing a maternity unit at a CSPS described the nature of these refusals: *“Parents' refusal is often rooted in their perceptions about the viability of preterm newborns. Many believe that the child may not survive, so they feel it's not worth the time and effort. They worry about the time commitment and the impact on their daily activities.”*

In addition to this, malnutrition and limited access to prenatal care, especially among socioeconomically disadvantaged women, were also reported as significant contributors to prematurity. As a midwife with eleven years of practice explained: *“Malnutrition, which is linked to socio-economic status, plays a role. If a mother can’t eat well, it can lead to prematurity.”*

A parallel concern was the stigma associated with prematurity, which was described as both isolating and psychologically harmful to mothers. This suggests that any intervention aimed at improving care must also confront prevailing social narratives and cultural beliefs about preterm birth: According to a medical doctor with two years of experience in CSPS management: “*There’s still a stigma attached to being premature, which can be hurtful… This kind of stigma can lead to feelings of rejection, which can be psychologically damaging.”*

These insights underscore the need for culturally sensitive interventions that not only address medical needs but also engage families and communities in reshaping beliefs, reducing stigma, and fostering trust in the continuum of care for preterm infants.

- 1. **Improvement in care of preterm birth**

Three subthemes emerged within this theme.

### **Raising community awareness and promoting preventive practices**

Respondents widely emphasized the importance of community awareness as a foundational step in reducing the incidence of premature births. This reflects a shared understanding that health outcomes are not solely determined by clinical care but are deeply embedded within socio-behavioral practices. Early antenatal care, nutritional support, and consistent pregnancy monitoring were commonly cited. A midwife stated, *“Raising awareness in the community is crucial. Women should start antenatal care early and focus on taking iron supplements and maintaining a good diet. Proper follow-up care is also key. With increased awareness, we can potentially reduce the incidence of prematurity.”*

Beyond general health promotion, respondents described the need for proactive identification and management of maternal conditions such as urinary tract infections and malaria—recognized contributors to preterm labor in the local context. This indicates a shift toward more targeted, risk-based approaches within antenatal care, tailored to local epidemiological realities. As one midwife with two years of experience noted, *“We need to understand and address women’s problems early on. For instance, we should promptly detect and treat conditions like urinary infections and malaria, which can lead to premature births or late miscarriage.”*

Participants also emphasized the importance of empowering women through health education, particularly regarding danger signs during pregnancy. This reflects an underlying strategy of shifting care-seeking behavior from reactive to anticipatory. A medical doctor with three years of experience managing the CMA shared, *“We not only offer routine care but also educate mothers on danger signs that might indicate a risk of premature birth. Signs like severe abdominal pain, bleeding, or excruciating headaches during pregnancy are red flags we teach them to recognize.”*

Importantly, respondents broadened the focus beyond medical risks to include psychosocial stressors. Domestic conflict, denial of paternity, and economic hardship were commonly cited as latent drivers of stress-induced prematurity. This highlights the need to address gendered and socio-cultural dimensions of maternal well-being. A midwife explained, *“Socio-cultural issues like domestic disputes or denial of paternity can stress the mother and lead to premature births… Counseling the couple, especially the husband, to be gentle and supportive is crucial, as such stress can trigger premature births.”*

The stigma associated with prematurity also emerged as a barrier to appropriate care. To counter this, respondents recommended community education and awareness-building efforts. As one midwife observed, *“There’s growing knowledge about the special needs of premature babies… When we give advice, especially to a mother-in-law, they tend to follow it meticulously.”*

- - 1. ***Enhancing Technical Capabilities and Resources***

The second major axis of improvement concerned the limitations of health facility infrastructure and equipment. Respondents repeatedly described the urgent need for dedicated neonatology units, especially at the district and referral hospital levels. This reflects a broader recognition of the mismatch between the clinical complexity of preterm newborn care and the limited resources currently available. As one gynecologist with extensive experience in a maternity ward put it, *“To enhance the care of premature babies, we need to strengthen our capabilities. Specifically, we should create a neonatology unit staffed with pediatricians to provide dedicated care… We need to bolster our infrastructure, personnel, and skills at the CMA.”*

Participants painted a vivid picture of systemic under-resourcing, where essential equipment such as radiant warmers, oxygen supplies, and nasogastric tubes were either scarce or entirely absent, especially in primary-level facilities. The consequences were not abstract but immediate, affecting survival and long-term outcomes. As a midwife with over ten years of experience noted, *“It’s crucial to strengthen our resuscitation equipment. We need heating lamps, oxygenation facilities, and improved suction capabilities. There’s also a shortage of nasogastric tubes, which are essential but rare and expensive.”*

- - 1. ***Continuous training and knowledge dissemination***

The final axis of improvement related to capacity-building through ongoing professional training. Participants emphasized that effective care for preterm newborns requires up-to-date skills that are not always acquired during basic professional education, especially for midwives working in rural or underserved areas. As one midwife noted, *“…continuous training is essential since the skills needed for this care are not used daily…”*

Knowledge dissemination was described as both a necessity and a challenge. The transient nature of healthcare staffing in many regions, marked by high turnover and mobility, was seen as a threat to institutional memory. In this context, peer training and knowledge-sharing mechanisms were viewed as critical stopgaps. A midwife with two years of experience explained, *“When we train health agents, they should share their skills with those who didn’t receive the training. This way, the knowledge is disseminated, ensuring continuous and effective care.”*

Respondents also highlighted the value of nationally coordinated training strategies, suggesting that fragmented or ad-hoc initiatives may be insufficient to create long-term improvements. A midwife with five years of experience stated, *“To tackle the issue of training in prematurity, I believe national-level training is more effective. Regular refresher courses should be organized for existing staff.”*

Some providers called for retention incentives, such as post-training service commitments or prioritizing the training of local staff more likely to remain in the area. These suggestions reflect a deeper awareness of the interplay between human resource planning and sustainable quality improvement. As one midwife put it, *“We could have a policy where staff sign a commitment to stay for a certain number of years after receiving training or focus on training local staff who are more likely to remain in the center.”*

These interconnected areas of improvement (community engagement, infrastructural reinforcement, and ongoing capacity-building) not only reflect the realities of care provision in resource-limited settings but also offer a strategic foundation for shaping more resilient, context-responsive neonatal care systems.

# Discussion

This qualitative study explored the multifaceted aspects of diagnosis and managing preterm births in urban health centers in Burkina Faso, focusing on four critical themes identified from the interviews: definition of preterm birth, assessment of preterm birth, registration and incidence of preterm births, and the challenges faced during preterm births. These themes provide a nuanced understanding of the diagnosis and management of preterm births in this context.

The findings underscore the importance of gestational age as the primary criterion for defining preterm birth, with health centers generally adopting a range between 28 and 37 weeks of gestation. This approach aligns with the Burkina Faso midwifery training curricula (Ministère de la santé du Burkina Faso, 2018) and previous study (Chawanpaiboon et al., 2019). It should be noted that this definition partly follows WHO guidelines which define prematurity as babies born alive before 37 weeks of gestation are completed (World Health Organization, 2023b). Notably, birth weight is also used as a secondary criterion, providing heterogeneity in the definition of prematurity. This variability highlights the critical need for standardized definitions and training to ensure uniform identification, documentation, and management of preterm births within Burkina Faso’s healthcare system.

The consensus among respondents favors early ultrasounds, particularly those conducted before 12 weeks of gestation, as the most accurate method for dating pregnancies. This preference is supported by evidence that early ultrasounds provide a high degree of accuracy, with diminishes as the pregnancy progresses (J. A. Morgan & Cooper, 2025; Murphy et al., 2020; World Health Organization, 2016b). However, due to geographical and financial barriers, as well as limited access to ultrasound technology, many women in resource-limited countries either do not have access to ultrasound or only receive it later in their pregnancies, as this study highlights (Kim et al., 2018). Another study from Cameroon found that only 23% of women received an obstetric ultrasound in the first trimester (Ngowa et al., 2014). The limited availability of early ultrasounds emphasizes the need for targeted interventions to enhance access to accurate gestational dating methods, which could substantially improve preterm birth management and neonatal health outcomes in similar resource-limited settings.

Despite the strong preference for early ultrasound, the LMP remains valuable for gestational dating when available (Macaulay et al., 2019). The LMP simplifies the estimation process and enables the use of tools like gestograms for assessing gestational age (Macaulay et al., 2019; Rosenberg et al., 2009). However, the difficulty in obtaining accurate LMP data, often due to recall issues or delayed prenatal visits; additionally, the LMP is subject to memory bias, may be misleading in cases of implantation bleeding, and is inherently unreliable in women with irregular cycles. Furthermore, in women with longer-than-average cycles, estimating gestational age based on LMP is inaccurate, as the calculation assumes a 28-day cycle, forces practitioners to rely on alternative methods (Ogbe et al., 2015). In settings with limited resources, fundal height measurements remain a critical tool, despite their limitations. Studies show that accuracy improves when multiple measurements are available (Unger et al., 2019; White et al., 2012). These measurements are influenced by various factors such as maternal body type, multiple gestations, and amniotic fluid levels. The reliance on fundal height in resource-constrained environments underlines the need for flexible and adaptable methods in prenatal care, particularly in regions with limited access to advanced diagnostic tools like ultrasound. The data also highlight the significance of incorporating clinical evaluations and patient reports, particularly when early ultrasounds are unavailable or when discrepancies arise. The challenges in accurately determining gestational age complicate the estimation of preterm birth rates, further underscoring the need for integrated approaches to prenatal care in resource-limited settings.

The variability in how preterm births are recorded across health center complicates data aggregation and analysis. Although preterm births are typically recorded in birth registers, detailed information about preterm births is not consistently included in monthly activity reports. The inconsistency makes it challenging to analyze preterm birth rates at a broader level. The difficulties in distinguishing between prematurity and other conditions like hypotrophy further complicate accurate data collection. Many health centers lack mechanisms to specifically track preterm births, and manual record reviews are often necessary to extract this data. Some centers report that 5 to 10% of births are preterm, but these figures are often imprecise and fluctuate monthly. The suggestion to implement separate or distinct registers for documenting preterm births reflects a recognized need for more targeted data collection, which could enhance tracking, management, and research. Similar challenges with data inconsistency are reported in studies from both developed and developing countries. In lower-resource settings often struggle with a lack of comprehensive data systems, impacting healthcare planning and interventions (Beck et al., 2010; Ohuma et al., 2023; Vogel et al., 2016). Therefore, establishing standardized documentation and reporting protocols emerges as a critical step toward addressing data inconsistencies, enabling more effective management of preterm births, and strengthening maternal and neonatal health outcomes in low-income settings.

Preterm births represent a significant public health issue due to their frequency and the associated severe consequences (Ohuma et al., 2023; Perin et al., 2022). A major barrier to improving outcomes for preterm infants is the shortage of essential medical resources, including incubators, kangaroo care units, and necessary medications. This inadequacy often necessitates transfers to higher-level facilities, complicating care and highlighting the dependency on external facilities. Additionally, the lack of essential medications complicates the management of preterm births, underscoring systemic issue in supply chains and resource allocation. These challenges with medication availability are well-documented in neonatal care literature, with calls for improved logistical support to ensure access to life-saving drugs and treatments for preterm infants (Harrison & Goldenberg, 2016). Addressing these resource gaps through targeted investments in neonatal infrastructure, health workers training, and pharmaceutical supply chains is crucial to reducing neonatal morbidity and mortality associated with prematurity in resource-limited settings like Burkina Faso.

Parental perceptions and refusal to transfer preterm babies for further care also present challenges, driven by doubts about the viability of these infants and concerns over the time commitment and impact on daily activities. This study also identified infections as a significant cause of prematurity, complicating the care process, particularly in the absence of necessary medications to manage these threats effectively (Barros et al., 2011). Preterm infants require specialized care for conditions like hypothermia and respiratory distress, necessitating specialized training and equipment, such as Kangaroo Mother Care (Lawn et al., 2013). However, current capacity is limited, and many facilities are only equipped to manage healthier preterm infants.

In our study, the link between malnutrition, socio-economic hardship, and preterm birth was consistently raised, echoing findings from similar contexts in sub-Saharan Africa where food insecurity and poverty are recognized as indirect drivers of poor pregnancy outcomes (Christian et al., 2013). Moreover, the stigma associated with prematurity emerged as a unique barrier to optimal care-seeking and acceptance of newborns within the family and community. Similar findings have been reported in Ethiopia and Malawi, where community-held misconceptions about prematurity can lead to maternal isolation or reduced family support (Moro, 2019). These results call for integrated community education programs that not only promote preventive behaviors but also aim to shift harmful social narratives about prematurity. Addressing to these challenges requires not only strengthening clinical capacity but also tackling community-level barriers, particularly parental perceptions, stigma, and socio-economic constraints that compromise care-seeking and adherence to medical recommendations

Community awareness plays role in both the prevention and early detection of conditions leading to preterm birth. Respondents emphasized early antenatal care (ANC), maternal nutrition, and education about pregnancy danger signs. These priorities are consistent with previous studies showing that timely ANC initiation and maternal empowerment can significantly reduce the risk of preterm birth and improve neonatal outcomes (Kuhnt & Vollmer, 2017; Lincetto, 2006). Secondly, in response to the critical shortage of neonatal equipment, participants proposed a range of practical and context-adapted solutions aimed at mitigating the impact of infrastructural deficits on preterm newborn care. A recurrent suggestion was the establishment of dedicated neonatology units within district and referral hospitals, staffed with trained pediatricians and equipped with essential tools such as radiant warmers, oxygen concentrators, and nasogastric tubes. This aligns with global recommendations emphasizing the decentralization of specialized newborn services to improve timely access to care (Moxon, 2015; World Health Organization, 2016a).

Finally, continuous professional development was widely viewed by respondents as a cornerstone of quality improvement. In contexts where neonatal care is not part of daily practice, frequent refresher trainings are essential to maintain provider competence. This aligns with the literature showing that targeted in-service training programs, especially those that combine theory with hands-on practice, can significantly improve provider performance and neonatal outcomes (Opiyo, 2008; Spector, 2013). Together, these findings emphasize that improving care for preterm newborns in Burkina Faso, and similar settings, requires a multi-level strategy. At the community level, public health campaigns must address both biomedical prevention and social stigma. At the facility level, health systems require better infrastructure, standardized equipment provision, and improved infection control. And at the workforce level, sustainable investments in training, supervision, and retention mechanisms are essential.

The discrepancy between the higher prevalence of prematurity reported by health centers and the lower prevalence detected using the AMANHI algorithm likely stems from an overestimation of preterm births due to reliance on non-ultrasound methods of gestational dating. In settings where ultrasound technology is limited, only a small proportion of pregnant women undergo ultrasound-based gestational age assessment. As a result, health workers often depend on less accurate methods such as the last menstrual period (LMP) recall and fundal height measurements. Consequently, the prevalence estimates derived from late-pregnancy ultrasounds (such as those obtained through the AMANHI algorithm) offer improved accuracy. This underscores the necessity of integrating ultrasound-based methods, like the AMANHI algorithm, into routine prenatal care to minimize misclassification of preterm births, enhance data reliability, and ultimately strengthen public health strategies in settings like urban Burkina Faso.

1. **Limitations of the study**

The study has some limitations. Any in-depth interview is susceptible to desirability bias, where the interviewee may adjust their responses to align with what they perceive the interviewer wants to hear. However, this bias was minimized by employing an independent interviewer with no affiliations to the healthcare setting, reducing the likelihood of influenced responses.

Second, the qualitative data analysis employed a novel NLP approach that is not yet widely adopted in qualitative health research, requiring a rigorous and iterative informatic coding, and integration with traditional methods (triangulation, saturation and manual theme extraction) to ensure the validity of results.

A notable limitation lies in the variability of participants’ responses, shaped by their diverse yet complementary professional backgrounds, which made direct comparisons across interviews more complex. Additionally, potential selection bias in identifying and prioritizing relevant themes may have influenced the interpretation of findings, potentially amplifying certain perspectives while underrepresenting others. This subjectivity may have constrained the breadth of viewpoints reflected in the final analysis.

# Conclusion

This study provides valuable insights into the complexity of gestational age estimation and the challenges involved in managing preterm births within urban health centers in Burkina Faso. The findings emphasize gestational age as the primary criterion for identifying preterm births but also highlight significant limitations, especially the limited availability of early ultrasound—the gold standard for precise pregnancy dating. Consequently, healthcare workers frequently rely on alternative methods such as the last menstrual period and fundal height measurements, which are susceptible to inaccuracies. Such inaccuracies can lead to misclassification of preterm births, affecting the reliability of public health data and compromising neonatal care.

In addition, the study identifies considerable inconsistencies in the recording and monitoring of preterm births across health facilities. These inconsistencies hinder effective data aggregation and limit the potential for informed decision-making at both clinical and public health levels. To overcome these challenges, it is essential to standardize gestational age assessment methods through targeted training programs, improve access to affordable ultrasounds, potentially via government subsidies or external funding, and introduce consistent documentation protocols specifically designed for tracking prematurity. Clinically, implementing these measures will allow healthcare providers to accurately classify preterm births, tailor interventions effectively, and improve the quality of care provided to preterm infants.

Nevertheless, the results of this qualitative study should be interpreted with caution due to certain inherent limitations. The subjective nature of qualitative research, potential biases in thematic selection and interpretation, and the variability in participant responses across different professional profiles, may affect the generalizability of our conclusions.

Future research should explore additional postnatal gestational age estimation methods, such as the Ballard score, to complement antenatal assessments.

Despite these limitations, recommendations based on the results of the study are important. Indeed, data accuracy enhance will support health policymakers in allocating resources more appropriately, leading to better-equipped neonatal units, optimized medication supplies, and targeted prevention strategies. Ultimately, this comprehensive approach could significantly reduce neonatal mortality and morbidity associated with preterm births in Burkina Faso and other similar resource-constrained settings.

**Availability of data and materials**

# Given the personal nature of the data, data will be made available

# through a data-sharing agreement. Please contact trenton@dailey-chwalibog.com for any queries.

**Author Contributions**

**Anderson Compaoré**: conceptualization (equal); methodology (equal); formal analysis (lead); investigation (equal); data curation (lead); writing – original draft (lead); writing—review and editing (lead). **Moctar Ouédraogo**: conceptualization (equal); methodology (equal); investigation (equal); resources (equal); data curation (equal); writing—review and editing (equal). **Cheick Ahmed Ouattara**: conceptualization (equal); methodology (equal); formal analysis (equal); investigation (equal); data curation (equal); writing – original draft (equal); writing—review and editing (equal). **Lionel Olivier Ouédraogo**: writing—review and editing (equal). **Lishi Deng**: writing—review and editing (equal). **Nicolas Sawadogo**: software (lead); formal analysis (equal); writing—review and editing (equal). **Carl Lachat**: writing—review and editing (equal). **Laéticia Celine Toé**: conceptualization (equal); validation (lead); resources(lead); writing—review and editing (equal); supervision (lead); project administration (lead); funding acquisition (lead). **Trenton Dailey-Chwalibóg**: conceptualization (lead); methodology (equal); software (equal); validation (lead); formal analysis (equal); investigation (equal); resources (lead); data curation (equal); writing – original draft (equal); writing—review and editing (equal); supervision (lead); project administration (lead); funding acquisition (lead). All authors have read and agreed to the published version of the manuscript.

# Ethical approval and consent to participate

# This study was approved by the Comité d’Ethique Institutionel de Recherche en Sciences de la Santé (CEIRES) of the Institut de Recherche en Sciences de la santé (IRSS) (protocol code R021-2024/CEIRES and the date of 06/10/2024) and strictly adhered to the principles of the Declaration of Helsinki. Informed consent was obtained from all participants.

**Consent for publication**

The manuscript is approved by all authors for publication.

**Disclosure statement**

No potential conflict of interest was reported by the authors.

# Additional information

# Funding

# This research was funded by the Bill & Melinda Gates Foundation [INV-035474, INV-036154]. The funders had no role in the design and conduct of the study; in the collection, management, analysis, and interpretation of the data; or in the preparation, review, or approval of the manuscript.

Note : There’s no affiliation between authors and Bill & Melinda Gates Foundation. This is because the initial project (DenBalo) was funded by the Foundation.

# References

Barros, F. C., Barros, A. J. D., Villar, J., Matijasevich, A., Domingues, M. R., & Victora, C. G. (2011). How many low birthweight babies in low- and middle-income countries are preterm? *Revista De Saude Publica*, *45*(3), 607–616. https://doi.org/10.1590/s0034-89102011005000019

Beck, S., Wojdyla, D., Say, L., Pilar Bertran, A., Meraldi, M., Harris Requejo, J., Rubens, C., Menon, R., & Van Look, P. (2010). The worldwide incidence of preterm birth: A systematic review of maternal mortality and morbidity. *Bulletin of the World Health Organization*, *88*(1), 31–38. https://doi.org/10.2471/BLT.08.062554

Braun, V., & Clarke, V. (2006). Using thematic analysis in psychology. *Qualitative Research in Psychology*, *3*(2), 77–101. https://doi.org/10.1191/1478088706qp063oa

Chawanpaiboon, S., Vogel, J. P., Moller, A.-B., Lumbiganon, P., Petzold, M., Hogan, D., Landoulsi, S., Jampathong, N., Kongwattanakul, K., Laopaiboon, M., Lewis, C., Rattanakanokchai, S., Teng, D. N., Thinkhamrop, J., Watananirun, K., Zhang, J., Zhou, W., & Gülmezoglu, A. M. (2019). Global, regional, and national estimates of levels of preterm birth in 2014: A systematic review and modelling analysis. *The Lancet Global Health*, *7*(1), e37–e46. https://doi.org/10.1016/S2214-109X(18)30451-0

Christian, P., Lee, S. E., Donahue Angel, M., & Adair, L. S. (2013). Risk of childhood undernutrition related to small-for-gestational age and preterm birth in low- and middle-income countries. *International Journal of Epidemiology*, *42*(5), 1340–1355. https://doi.org/10.1093/ije/dyt109

District sanitaire de Do. (2023). *Rapports mensuels d’activité Avril à Octobre 2023.Bobo-Dioulasso(Burkina Faso)*.

Harrison, M. S., & Goldenberg, R. L. (2016). Global burden of prematurity. *Seminars in Fetal and Neonatal Medicine*, *21*(2), 74–79. https://doi.org/10.1016/j.siny.2015.12.007

Hu, C., Gong, H., & He, Y. (2022). Data driven identification of international cutting edge science and technologies using SpaCy. *PLOS ONE*, *17*, e0275872. https://doi.org/10.1371/journal.pone.0275872

Kim, E. T., Singh, K., Moran, A., Armbruster, D., & Kozuki, N. (2018). Obstetric ultrasound use in low and middle income countries: A narrative review. *Reproductive Health*, *15*(1), 129. https://doi.org/10.1186/s12978-018-0571-y

Kramer, M. S. (2013). Born too small or too soon. *The Lancet Global Health*, *1*(1), e7–e8. https://doi.org/10.1016/S2214-109X(13)70014-7

Kuhnt, J., & Vollmer, S. (2017). Antenatal care services and its implications for vital and health outcomes of children: Evidence from 193 surveys in 69 low-income and middle-income countries. *BMJ Open*, *7*(11), e017122.

Lawn, J. E., Kinney, M. V., Belizan, J. M., Mason, E. M., McDougall, L., Larson, J., Lackritz, E., Friberg, I. K., & Howson, C. P. (2013). Born Too Soon: Accelerating actions for prevention and care of 15 million newborns born too soon. *Reproductive Health*, *10*(S1), S6. https://doi.org/10.1186/1742-4755-10-S1-S6

Lincetto, O. (2006). Antenatal care. *Opportunities for Africa’s Newborns: Practical Data, Policy and Programmatic Support for Newborn Care in Africa*.

Liu, L., Oza, S., Hogan, D., Perin, J., Rudan, I., Lawn, J. E., Cousens, S., Mathers, C., & Black, R. E. (2015). Global, regional, and national causes of child mortality in 2000–13, with projections to inform post-2015 priorities: An updated systematic analysis. *The Lancet*, *385*(9966), 430–440. https://doi.org/10.1016/S0140-6736(14)61698-6

Macaulay, S., Buchmann, E. J., Dunger, D. B., & Norris, S. A. (2019). Reliability and validity of last menstrual period for gestational age estimation in a low‐to‐middle‐income setting. *Journal of Obstetrics and Gynaecology Research*, *45*(1), 217–225. https://doi.org/10.1111/jog.13801

Ministère de la santé du Burkina Faso. (2018). *Protocole de santé de la reproduction*.

Morgan, A. S., Mendonça, M., Thiele, N., & David, A. L. (2022). Management and outcomes of extreme preterm birth. *BMJ*, e055924. https://doi.org/10.1136/bmj-2021-055924

Morgan, J. A., & Cooper, D. B. (2025). Pregnancy Dating. In *StatPearls*. StatPearls Publishing. http://www.ncbi.nlm.nih.gov/books/NBK442018/

Moro, M. (2019). Family perceptions of preterm newborns in Ethiopia and Malawi: A qualitative study. *BMJ Global Health*, *4*(Suppl 5), e001902.

Moxon, S. G. (2015). Quality of care for newborns: A health systems perspective. *The Lancet*, *384*(9941), 1225–1235.

Murphy, D., Strachan, B., Bahl, R., & the Royal College of Obstetricians and Gynaecologists. (2020). Assisted Vaginal Birth: Green‐top Guideline No. 26. *BJOG: An International Journal of Obstetrics & Gynaecology*, *127*(9). https://doi.org/10.1111/1471-0528.16092

National Institute for Health Care and Excellence. (2015). *Preterm labour and birth*. https://www.nice.org.uk/guidance/ng25

Ngowa, J. D., Mando, E., Guegang, E., Ngassam, A., & Kasia, J. M. (2014). Datation de la grossesse en pratique courante au Cameroun: Fiabilité de la date de dernières règles. *Pan African Medical Journal*, *17*. https://doi.org/10.11604/pamj.2014.17.264.3692

Ogbe, A., Ekwempu, C., Musa, J., & Anzaku, A. (2015). A Comparison of the Accuracy of the Use of Last Menstrual Period and Symphysio-Fundal Height for Gestational Age Determination among Nigerian Women. *International Journal of TROPICAL DISEASE & Health*, *7*(1), 32–39. https://doi.org/10.9734/IJTDH/2015/15826

Ohuma, E. O., Moller, A.-B., Bradley, E., Chakwera, S., Hussain-Alkhateeb, L., Lewin, A., Okwaraji, Y. B., Mahanani, W. R., Johansson, E. W., Lavin, T., Fernandez, D. E., Domínguez, G. G., De Costa, A., Cresswell, J. A., Krasevec, J., Lawn, J. E., Blencowe, H., Requejo, J., & Moran, A. C. (2023). National, regional, and global estimates of preterm birth in 2020, with trends from 2010: A systematic analysis. *The Lancet*, *402*(10409), 1261–1271. https://doi.org/10.1016/S0140-6736(23)00878-4

Opiyo, N. (2008). The effect of training health care providers on the quality of perinatal care in low-resource settings. *Cochrane Database of Systematic Reviews*, *3*.

Ouattara, C. A., Compaoré, A., Ouédraogo, L. O., Ouédraogo, M., Ouattara, H., Coulibaly, M., Deng, L., Nikiéma, Z., Hanley-Cook, G. T., Argaw, A., Huybregts, L., Hadush, K. T., Lachat, C., Toe, L. C., & Dailey-Chwalibóg, T. (2025). Comparison of Gestational Age Assessment Methods in the Second and Third Trimesters: Evaluating Alternative Approaches Against Ultrasound in Urban Burkina Faso. *Journal of Clinical Medicine*, *14*(5). https://doi.org/10.3390/jcm14051421

Ouédraogo, L. O., Deng, L., Ouattara, C. A., Compaoré, A., Ouédraogo, M., Argaw, A., Lachat, C., Houpt, E. R., Saidi, Q., Haerynck, F., Sonnenburg, J., Azad, M. B., Tavernier, S. J., Bastos-Moreira, Y., Toe, L. C., & Dailey-Chwalibóg, T. (2024). Describing Biological Vulnerability in Small, Vulnerable Newborns in Urban Burkina Faso (DenBalo): Gut Microbiota, Immune System, and Breastmilk Assembly. *Nutrients*, *16*(23). https://doi.org/10.3390/nu16234242

Perin, J., Mulick, A., Yeung, D., Villavicencio, F., Lopez, G., Strong, K. L., Prieto-Merino, D., Cousens, S., Black, R. E., & Liu, L. (2022). Global, regional, and national causes of under-5 mortality in 2000–19: An updated systematic analysis with implications for the Sustainable Development Goals. *The Lancet Child & Adolescent Health*, *6*(2), 106–115. https://doi.org/10.1016/S2352-4642(21)00311-4

Pope, C. (2000). Qualitative research in health care: Analysing qualitative data. *BMJ*, *320*(7227), 114–116. https://doi.org/10.1136/bmj.320.7227.114

Prakash, N., Prasad N, A., Nagaraj, J., Pareek, P., Maruthikumar, N., & Manjunath, R. (2022). Implementation of NLP based automatic text summarization using spacy. *International Journal of Health Sciences*, 7508–7521. https://doi.org/10.53730/ijhs.v6nS5.10574

Rosenberg, R. E., Ahmed, A. N. U., Ahmed, S., Saha, S. K., Chowdhury, M. A., Black, R. E., Santosham, M., & Darmstadt, G. L. (2009). Determining Gestational Age in a Low-resource Setting: Validity of Last Menstrual Period. *Journal of Health, Population and Nutrition*, *27*(3), 332–338. https://doi.org/10.3329/jhpn.v27i3.3375

Spector, J. M. (2013). Scaling up quality improvement: Lessons from maternal and newborn health programs in five countries. *International Journal for Quality in Health Care*, *25*(3), 278–286.

Unger, H., Thriemer, K., Ley, B., Tinto, H., Traoré, M., Valea, I., Tagbor, H., Antwi, G., Gbekor, P., Nambozi, M., Kabuya, J.-B. B., Mulenga, M., Mwapasa, V., Chapotera, G., Madanitsa, M., Rulisa, S., De Crop, M., Claeys, Y., Ravinetto, R., & D’Alessandro, U. (2019). The assessment of gestational age: A comparison of different methods from a malaria pregnancy cohort in sub-Saharan Africa. *BMC Pregnancy and Childbirth*, *19*(1), 12. https://doi.org/10.1186/s12884-018-2128-z

Vogel, J. P., Chawanpaiboon, S., Watananirun, K., Lumbiganon, P., Petzold, M., Moller, A.-B., Thinkhamrop, J., Laopaiboon, M., Seuc, A. H., Hogan, D., Tunçalp, O., Allanson, E., Betrán, A. P., Bonet, M., Oladapo, O. T., & Gülmezoglu, A. M. (2016). Global, regional and national levels and trends of preterm birth rates for 1990 to 2014: Protocol for development of World Health Organization estimates. *Reproductive Health*, *13*(1), 76. https://doi.org/10.1186/s12978-016-0193-1

White, L. J., Lee, S. J., Stepniewska, K., Simpson, J. A., Dwell, S. L. M., Arunjerdja, R., Singhasivanon, P., White, N. J., Nosten, F., & McGready, R. (2012). Estimation of gestational age from fundal height: A solution for resource-poor settings. *Journal of The Royal Society Interface*, *9*(68), 503–510. https://doi.org/10.1098/rsif.2011.0376

World Health Organization. (2016a). *Standards for improving quality of maternal and newborn care in health facilities*. World Health Organization; WHO IRIS. https://iris.who.int/handle/10665/249155

World Health Organization. (2016b). *WHO recommendations on antenatal care for a positive pregnancy experience*. World Health Organization. https://iris.who.int/handle/10665/250796

World Health Organization. (2023a). *Born Too Soon: Decade of action on preterm birth.* World Health Organization.

World Health Organization. (2023b). *Preterm birth*. https://www.who.int/news-room/fact-sheets/detail/preterm-birth

Wylie, B. J., Kalilani-Phiri, L., Madanitsa, M., Membe, G., Nyirenda, O., Mawindo, P., Kuyenda, R., Malenga, A., Masonbrink, A., Makanani, B., Thesing, P., & Laufer, M. K. (2013). Gestational age assessment in malaria pregnancy cohorts: A prospective ultrasound demonstration project in Malawi. *Malaria Journal*, *12*(1), 183. https://doi.org/10.1186/1475-2875-12-183

You, D., Hug, L., Ejdemyr, S., Idele, P., Hogan, D., Mathers, C., Gerland, P., New, J. R., & Alkema, L. (2015). Global, regional, and national levels and trends in under-5 mortality between 1990 and 2015, with scenario-based projections to 2030: A systematic analysis by the UN Inter-agency Group for Child Mortality Estimation. *The Lancet*, *386*(10010), 2275–2286. https://doi.org/10.1016/S0140-6736(15)00120-8

Yuce, T., Keskin, M., Seval, M. M., & Söylemez, F. (2015). Effect of the timing of delivery on perinatal outcomes at gestational hypertension. *Interventional Medicine and Applied Science*, *7*(2), 59–62. https://doi.org/10.1556/1646.7.2015.2.3

| **Appendix A: Interview guide**  ***Interviews:*** *MCD, Gynecologists, Head Doctors/Nurses, Maternity Ward Managers*  ***Focus group:*** bring together at least 7 to 8 midwives/midwives from the 4 maternity hospitals INTRODUCTIONS  - Introduce yourself and let the health care staff in front know that you are here to better understand some aspects related to preterm birth. All answers are welcome: there are no right or wrong answers. - The interview will be recorded if it is convenient for her. Only the research team will listen to the recording. We will not associate her name with her opinions and experiences about prematurity.   The brief consent form should also be recorded and their agreement to participate should also be recorded.  ***Start your voice recorder and say out loud:***   - The name of the CSPS - Today's date and time (interview date)   The function, title and role of your contact: ICP of (NAME of CSPS), Maternity manager of (name of CSPS) ***(see question 1)***   - Do not say the name of the person you are talking to.   **INFORMED CONSENT:** Hello, thank you very much in advance for your availability and the time you give us. We are a research team from Ghent University, IRSS and AFRICSanté. We are conducting qualitative research on preterm birth in urban areas of Burkina Faso. Our goal is to better understand this phenomenon in order to improve services and health care for mothers and preterm infants. Your participation is essential to help us understand the specific challenges you face in your health center and to identify opportunities for improvement.  Today’s interview will be confidential and the information collected will be used for research purposes only. You are free not to answer any questions that make you uncomfortable and you can stop the interview at any time. Do you have any questions before we begin? With your permission, may we begin the interview?   1. Could you start by introducing yourself (no name), your title, your position and your role, please? How long have you been in this position? 2. How do you define premature birth in your health center? 3. Now that we have defined what premature birth is, how is it measured in your health center?    1. Out of 10 women who come to their first CPN, how many know the date of their last period? In which trimester of pregnancy or what gestational age do women most often come to their first CPN? Do you think that women know precisely (plus or minus a week, plus or minus a month) the date of their last period during their first CPN?    2. Do pregnant women who present for their first ANC later (after the 3rd month of pregnancy) in pregnancy always remember the date of their last period?   If the pregnant woman does not remember at all, how does the midwife determine the gestational age (fundal height)?  How do you calculate gestational age from fundal height? By gestogram or by table)?   - 1. Do you have the equipment to perform the gestational age calculation? If not, what approach do you use in this case?   2. When calculating gestational age, which estimate takes priority: that of the uterine height or that of the date of the last menstrual period?   3. Do women sometimes come with an ultrasound estimate? In that case, which estimate takes priority: the ultrasound estimate, the uterine height estimate, or the date of the last period?   4. In case the gestational age estimates by last menstrual period, fundal height and ultrasound are different, which estimate do you decide to use and why?  1. Is information on premature births recorded in the delivery register? Is this reflected in your monthly activity report? 2. How many premature babies are born each month in your health center?    1. To get a rough idea, he can possibly use his birth register.    2. If he has no idea and wants to consult his register, continue the interview and offer to come back to get the information on prematurity for the last 7 months (January to July). 3. Is preterm birth a major public health challenge you face in your health center? What types of challenges do you face in your department regarding pregnancy, childbirth and newborn care? 4. Do you have the necessary technical platform, that is to say the trained personnel, the medications and the equipment to manage cases of premature birth?    1. Probe for very premature babies?    2. What do you do when you find that you cannot handle a case of premature birth in your department? 5. What types of services are provided to premature infants and their mothers, if any, in your department? 6. What are the specific health risks that premature infants face in urban Burkina Faso?    1. Mortality survey?    2. Also probe morbidities - diseases? 7. Have you noticed any specific characteristics regarding mothers of premature babies?    1. Do they tend to be younger or older?    2. Do they come from a specific socio-economic background?    3. Do they have any special health conditions? 8. How can we improve the management of premature births in your health center?    1. Probe about skill building?    2. Probe on human and material resources? |
| --- |

| **Appendix B : Uterine height/gestational age correspondence table**   \| **Fundal height in centimeters** \| **Week of amenorrhea** \| \| --- \| --- \| \| 12 cm \| 16 weeks \| \| 14 cm \| 18 Weeks \| \| 16 cm \| 20 Weeks \| \| 19 cm \| 22 Weeks \| \| 21 cm \| 24 Weeks \| \| 23 cm \| 26 Weeks \| \| 25 cm \| 28 Weeks \| \| 27 cm \| 30 Weeks \| \| 28 cm \| 32 Weeks \| \| 30 cm \| 34 Weeks \| \| 34 cm \| 38 Weeks \| \| 36 cm \| 40 weeks \| |
| --- | --- | --- | --- | --- | --- | --- | --- | --- | --- | --- | --- | --- | --- | --- | --- | --- | --- | --- | --- | --- | --- | --- | --- | --- | --- | --- |

**Figure 1 : Force directed graphs for 9 of 10 topics based on interview guide**

**A**: definition of preterm birth;

**B**: health risks in premature infants;

**C** improvement in care of preterm birth;

**D**: number of preterm birth per month;

**E**: registration of preterm births;

**F**: specificities of mothers of preterm children;

**G**: services and care provided to preterm babies and their mothers;

**H**: staff skills and existence of equipment for better care; **I**: types of challenges faced during preterm births

**Figure 2: Force directed graphs for subtopics from assessment of preterm birth topic**

**A**: method of calculating gestational age from fundal height;

**B**: appointment for prenatal consultations with ultrasound measurement carried out;

**C**: control of the date of the last period by women who present late to their ANC;

**D**: control of the last menstrual period;

**E**: materials needed for calculating gestational age;

**F**: priority between different methods of measuring gestational age;

**G**: priority method if the date of the last period, fundal height and ultrasound are discordant;

**H**: ways health workers help women remember the date of their last period.

**Figure 3: Workflow for qualitative data analysis: from data collection to thematic synthesis**

**Figure 4: Selection of themes**

**Figure 5: Gestogram**
