# Supplementary material for: Perceptions and practices in urban Burkina Faso: a qualitative study on gestational age estimation among health workers
Source: Int J Qual Stud Health Well-being. 2025 Jul 4;20(1):2508421. doi: 10.1080/17482631.2025.2508421 (PMC12231294; doi:10.1080/17482631.2025.2508421)
Supplement: Supplementary_file_2_preliminary_results.docx [file ZQHW_A_2508421_SM4862.docx]

# Supplementary file 2: preliminary results

## 1.Definition of preterm birth

Table 1: Results emulation for scientific article for “Definition of preterm birth”

| **Gestational Age as the Primary Criterion:** a consistent theme across the responses is the use of gestational age as the primary criterion for defining premature birth. Many health centers adhere to a specific range of weeks to classify a birth as premature. For instance, one response indicates that any birth occurring before 36 weeks of gestation is considered premature (Response 68). Similarly, another health center defines premature birth as any birth occurring between 28 and 37 weeks of amenorrhea (Response 69). This range of 28 to 37 weeks is commonly cited, with slight variations in the exact weeks considered. For example, some centers specify the period between the 28th and 36th week plus six days (Response 76), while others extend it to before 37 weeks (Response 84). This reliance on gestational age underscores its importance as the primary criterion for classifying premature births.  **Weight as a Secondary Criterion:** in addition to gestational age, some health centers also consider the weight of the newborn as a secondary criterion for defining premature birth. One response mentions that if the fetal weight is less than 2 kilograms, the birth is classified as premature (Response 68). Another center considers a birth premature if the baby weighs less than 2.5 kilograms (Response 75). These weight-based criteria supplement the gestational age guidelines, providing an additional parameter for identifying premature births.  **Universal and Local Definitions:** there is an acknowledgment of both universal and local definitions of premature birth. One response highlights that the definition of premature birth is somewhat universal in health practice, generally encompassing any birth occurring before 37 weeks of amenorrhea (Response 70). This response also makes a distinction between abortion (before 22 weeks) and premature birth (22 to 37 weeks). Another response compares local definitions with those in developed countries, indicating that in some regions, premature birth can be considered from as early as 22 weeks (Response 74). This comparison underscores the influence of local and global medical practices on defining prematurity.  **Standardized Definitions and Educational Influence:** some health centers adhere to standardized definitions within the global health system, emphasizing the importance of consistency in medical practice. One response states that premature birth is defined in a standard way, with births occurring from 7 months of pregnancy considered premature (Response 75). This standardization is reinforced by medical education and training, as noted in another response that references what was learned in medical courses (Response 74). These responses highlight the role of standardized definitions and educational influence in shaping the understanding and classification of premature births.  **Morphological and Additional Criteria:** while gestational age and weight are the primary criteria, some responses mention other morphological and additional criteria for defining premature birth. For instance, one health center notes the existence of other morphological criteria but emphasizes gestational age as the primary criterion (Response 68). This indicates that while additional factors may be considered, they are secondary to the primary criteria of gestational age and weight. |
| --- |

## 2. Measurement of preterm birth

### 2.1 Control of the date of the last period

Table 2: Results emulation for scientific article for “control of the date of last period”

| **Knowledge of Last Menstrual Period:** a common theme among the responses is that a significant number of women do not know the exact date of their last menstrual period (LMP). This lack of precise knowledge is often linked to illiteracy and the use of non-specific references to events or seasons to recall the date. One respondent noted that out of 10 women, approximately 4 might know their LMP date, often relating it to events like the harvest season or religious observances (Response 116). Another respondent indicated that about 3 out of 10 women could recall the date of their last period, highlighting the difficulty even among educated women if they delay starting antenatal care (Response 124). These responses suggest that precise recall of the LMP date is relatively uncommon, and in many cases, women provide an approximate month rather than an exact day (Response 179).  **Timing of First Antenatal Care Visit:** the timing of the first antenatal care (ANC) visit varies, with many women attending their first visit late in the first trimester or in the second trimester. Several respondents noted that women often come for their first ANC visit around three months into their pregnancy, which aligns with traditional recommendations rather than the WHO's advice to start as soon as pregnancy is realized (Responses 119, 137). In many cases, women arrive for their first ANC visit after the sixteenth week, placing them firmly in the second trimester (Response 125). Some respondents highlighted that a significant portion of women do not come for their first ANC visit until five months into the pregnancy (Response 133). Despite awareness campaigns encouraging earlier visits, there remains a tendency for women to delay their first ANC visit until the second trimester (Response 178).  **Awareness and Recall Precision:** the responses indicate that precise knowledge of the LMP date during the first ANC visit is relatively rare. One respondent mentioned that even among educated women, recalling the exact date is challenging unless they have been given a notebook by a health worker to record the date (Response 123). Another highlighted that while some women, particularly younger ones who use mobile apps to track their menstrual cycles, can recall the exact date, this is not the norm for most women (Response 134). Generally, most women can recall the month or provide an estimate rather than the exact day (Responses 143, 179, 180). |
| --- |

### 2.2 Appointment for prenatal consultations with ultrasound measurement carried out

Table 3: Results emulation for scientific article for “control of the date of last period”

| **Frequency of Ultrasound Usage:** the responses reveal that it is rare for women to come to their first antenatal care (ANC) visit with an ultrasound estimate, particularly if they have experienced early pregnancy complications. For instance, women who initially visit for issues like persistent pain or bleeding are often advised to get an ultrasound, which they then bring to their first ANC visit (Responses 296, 300, 305). While it is not the norm for all women to have an ultrasound prior to their first ANC visit, those who do often prioritize the ultrasound findings in their prenatal assessments (Responses 312, 313, 316).  **Comparison with Clinical Evaluations:** healthcare providers often use ultrasounds as a supplementary tool alongside clinical evaluation. Several respondents emphasized the importance of correlating ultrasound findings with clinical observations and the last menstrual period (LMP) date. For instance, one respondent noted that if there is a discrepancy between the ultrasound and clinical observations, they might prioritize the LMP date or request another ultrasound for confirmation (Responses 297, 302, 306). This approach highlights the perceived reliability of early ultrasounds, particularly those conducted in the first trimester, which are considered more accurate for dating pregnancies (Responses 308, 325, 333, 334).  **Accessibility and Timing of Ultrasounds:** the availability and timing of ultrasounds significantly impact their utilization. Early ultrasounds, typically done in the first trimester, are highly valued for their accuracy in estimating gestational age (Response 325). However, the cost of ultrasounds can be a barrier, making it less feasible for all women to obtain one early in their pregnancy (Response 318). As a result, many women rely on the LMP date and clinical evaluations, with ultrasounds being used when clinically indicated or when there is uncertainty about the pregnancy timeline (Responses 319, 328, 331).  **Practical Implementation:** in practice, health centers often request ultrasounds for pregnant women, especially if there are any clinical indications or uncertainties about gestational age. For example, fundal height measurements are routinely taken during consultations to assess pregnancy progress, and any discrepancies with the LMP date might prompt a request for an additional ultrasound (Response 315). This integrated approach ensures that healthcare providers have multiple sources of information to accurately monitor and manage pregnancies. |
| --- |

### 2.3 Method of calculating gestational age from fundal height

Table 4: Results emulation for scientific article for “ Method of calculating gestational age from fundal height”

| **Methods and Approximations:** in the absence of precise knowledge of the last menstrual period (LMP), midwives rely on various clinical methods to estimate gestational age. One common approach involves measuring the fundal height, which is the distance from the symphysis pubis to the top of the uterus (fundus). This measurement, typically in centimeters, provides an approximation of the gestational age. For example, one respondent noted that adding three centimeters to the fundal height measurement can help estimate the week of pregnancy (Response 225). Another method involves adding 4 cm to the fundal height measurement up to the seventh month of pregnancy, then adjusting to 3 cm for the eighth month, and 2 cm thereafter (Response 229).  **Clinical Correlations and Tools:** several respondents emphasized the use of clinical observations alongside fundal height measurements. For instance, one midwife mentioned the importance of asking whether the woman has felt fetal movements, which usually start in the second trimester, to help confirm the stage of pregnancy (Response 225). Additionally, tools such as gestograms can be used if the LMP is known, but they are less effective without this information (Responses 226, 237). Some respondents rely on pre-established tables that correlate fundal height with gestational age to provide a more systematic approach (Responses 235, 245).  **Practical Implementation and Challenges:**the practical implementation of these methods varies, with some midwives preferring to start with the LMP date if known, then confirming or adjusting based on fundal height measurements and other clinical signs (Response 231). For example, if a discrepancy exists between the fundal height and the expected gestational age based on LMP, further investigation, such as an ultrasound, might be warranted (Response 302). In some cases, the fundal height measurement is divided by four to estimate the number of months of pregnancy (Responses 238, 239). This method is particularly useful from the second trimester onwards when the uterus becomes more palpable above the pelvis.  **Importance of Ultrasound:** while fundal height measurements provide a useful clinical tool, several respondents highlighted the supplementary role of ultrasounds in accurately dating pregnancies, particularly when there are discrepancies or uncertainties (Responses 302, 315). Ultrasounds performed in the first trimester are considered more accurate for estimating gestational age (Response 325). However, due to cost and accessibility issues, not all women have early ultrasounds, necessitating reliance on clinical methods like fundal height measurements. |
| --- |

### 2.4 Priority method if the date of the last period, fundal height and ultrasound are discordant

Table 5: Results emulation for scientific article for “Priority method if the date of the last period, fundal height and ultrasound are discordant”

| **Preference for Early Ultrasound:** a common theme among respondents is the preference for early ultrasounds, particularly those conducted before twelve weeks of gestation, as the most accurate method for dating pregnancies. One respondent emphasized that early ultrasounds are generally considered the gold standard and are prioritized over other methods when available (Response 340). This preference is due to the high accuracy of early ultrasounds in estimating gestational age, which diminishes as pregnancy progresses (Response 345). Consequently, if there is a discrepancy between the ultrasound and other methods such as fundal height or the reported date of the last menstrual period (LMP), the early ultrasound is often given precedence.  **Clinical and Patient Reports:** in the absence of an early ultrasound or when there is a significant discrepancy, respondents noted that clinical evaluations and patient reports are also important. For example, one midwife mentioned that they trust the woman's account of her pregnancy timeline, valuing her input along with clinical examination results (Response 343). Another respondent highlighted the use of clinical signs, such as the onset of fetal movements, to help confirm the stage of pregnancy (Response 225). This approach underscores the importance of a holistic assessment, combining clinical observations with the patient’s history and reports.  **Fundal Height Measurements:** fundal height measurements are commonly used as a practical tool for estimating gestational age, especially in resource-limited settings where ultrasounds might not be readily available. One method involves adding centimeters to the fundal height measurement to approximate the week of pregnancy (Responses 225, 229). However, respondents acknowledged the limitations of fundal height measurements, which can be influenced by factors such as maternal obesity, twin pregnancies, and variations in amniotic fluid (Responses 345, 347). Therefore, while fundal height is a useful clinical tool, it is often supplemented with ultrasounds for a more accurate assessment.  **Integrating Multiple Methods:** in practice, healthcare providers often integrate multiple methods to resolve discrepancies in gestational age estimation. For instance, one respondent mentioned using both fundal height measurements and ultrasounds to cross-check and confirm the gestational age, especially in cases where physical examination and reported LMP do not align (Response 347). Another emphasized the importance of obtaining a new ultrasound when the initial one is outdated or when there is significant disagreement between methods (Responses 338, 339). This integrative approach ensures a more reliable estimation by leveraging the strengths of different methods.  **Reliability and Accuracy:** overall, the reliability and accuracy of ultrasounds, particularly early ultrasounds, make them a preferred method for dating pregnancies when discrepancies arise. However, the integration of clinical evaluations, patient history, and fundal height measurements provides a comprehensive approach to managing and resolving differences in gestational age estimation. This multi-faceted strategy ensures that healthcare providers can offer the most accurate and effective prenatal care to their patients. |
| --- |

### 2.5 Priority between different methods of measuring gestational age

Table 6: Results emulation for scientific article for “Priority between different methods of measuring gestational age”

| Preference for Ultrasound Estimates: a recurring theme among the responses is the preference for using ultrasound estimates when determining gestational age. Several respondents highlighted the accuracy of early ultrasounds, particularly those conducted in the first trimester, as the primary reason for their reliance on this method. For instance, one respondent emphasized that ultrasounds provide a precise gestational age with a small margin of error, typically between one week to ten days (Response 279). Another mentioned that due to the challenges in ascertaining the exact chronological age, they rely more on the ultrasound age when available (Response 272).  Use of Combined Methods: some respondents noted the importance of using a combination of methods to estimate gestational age. This integrated approach includes using fundal height measurements, the date of the last menstrual period (LMP), and ultrasounds. One respondent mentioned that they measure fundal height but recommend an ultrasound, especially if the woman does not remember her LMP (Response 281). Another respondent highlighted that in urban areas, they combine all available methods, including gestogram calculations, to arrive at a more accurate estimate (Response 282).  Priority of the Last Menstrual Period (LMP): several responses indicated a preference for using the date of the last menstrual period (LMP) when available. One respondent stated that knowing the LMP date simplifies the estimation process and allows for effective use of a gestogram to estimate gestational age (Response 282). Another emphasized the importance of obtaining the LMP date, noting that it is the best scenario for accurately determining gestational age (Response 287). However, they also acknowledged the challenge of gathering this date and the tendency of some practitioners to quickly move on to other methods if the LMP is not immediately known (Response 284).  Challenges with Fundal Height Measurements: the limitations and challenges associated with fundal height measurements were frequently mentioned. One respondent noted that fundal height can be influenced by factors such as the woman's body size and the amount of amniotic fluid, making it less reliable (Response 279). Another respondent emphasized that fundal height is biased, particularly in obese women where locating the uterus can be challenging (Response 275). Despite these challenges, fundal height measurements are still used, particularly in resource-limited settings where ultrasounds may not be readily available (Responses 282, 285).  Contextual Factors and Method Integration: in practice, healthcare providers often integrate multiple methods to ensure the most accurate estimation of gestational age. One respondent mentioned that when there are discrepancies between methods, they try to understand the causes and use additional tools like ultrasound to investigate further (Response 347). Another emphasized the importance of a new ultrasound when there is a significant disagreement between the initial ultrasound and clinical findings (Responses 338, 339). This integrative approach helps to cross-check and confirm gestational age estimates, leveraging the strengths of each method. |
| --- |

### 2.6 Ways health workers help women remember the date of their last period

Table 7: Results emulation for scientific article for “Priority between different methods of measuring gestational age”

| Reliance on Fundal Height Measurements: a common method for estimating gestational age when the date of the last menstrual period (LMP) is unknown involves measuring the fundal height. This measurement, from the symphysis pubis to the top of the uterus, provides an approximate gestational age. Many respondents indicated that fundal height is frequently used in such situations. For example, one respondent noted that this measurement can offer a general idea of gestational age, despite not being exact (Response 206). Another highlighted that fundal height measurement is useful for monitoring and is often relied upon in rural areas where ultrasounds are less accessible (Response 208). However, several respondents acknowledged the limitations and potential inaccuracies of this method, particularly in women with larger builds or thick abdominal walls (Responses 208, 214).  Use of Ultrasound for Accurate Dating: ultrasound is preferred for its precision in estimating gestational age, especially when the LMP is unknown. Many respondents emphasized the importance of ultrasound in providing a more accurate gestational age. For instance, one respondent mentioned that ultrasounds offer a precise measurement with a smaller margin of error compared to fundal height (Response 202). Another respondent highlighted that while clinical assessments can be useful, ultrasound remains the most accurate method for determining gestational age, especially in urban areas where this technology is more accessible (Response 208). However, the cost and availability of ultrasound can be barriers, particularly in rural areas (Response 213).  Clinical Assessments and Physical Examination: in addition to fundal height and ultrasound, clinical assessments and physical examinations are crucial in estimating gestational age. One approach involves asking women to recall significant events or seasons to approximate the timing of their pregnancy (Response 205). Another respondent noted that physical examinations, including assessing the uterine height, are primary methods used when the LMP is unknown (Response 203). These assessments can help provide a rough estimate of gestational age, although they may not be as precise as ultrasound measurements.  Integrating Multiple Methods: a comprehensive approach often involves integrating multiple methods to ensure a more accurate estimation of gestational age. One respondent described using a combination of fundal height measurement and ultrasound to cross-check and confirm gestational age estimates (Response 206). Another respondent emphasized starting with the LMP if known, then using clinical assessments and fundal height measurements, and resorting to ultrasound if necessary (Response 204). This integrative approach helps mitigate the limitations of relying on a single method and provides a more reliable estimate of gestational age.  Challenges and Practical Implementation: despite the preferred methods, practical challenges remain in accurately determining gestational age. Factors such as cost, accessibility of ultrasound, and variability in fundal height measurements due to individual differences can complicate the estimation process. Respondents noted that while ultrasound is the most accurate, its high cost can prevent some women from obtaining it, necessitating reliance on fundal height and clinical assessments (Response 213). Additionally, the margin of error in later ultrasounds increases, making early ultrasounds more valuable for precise dating (Response 206). |
| --- |

### 2.7 Control of the date of the last period by women who present late to their ANC

Table 8: Results emulation for scientific article for “Priority between different methods of measuring gestational age”

| Consistency of Recall of Last Menstrual Period: a recurring theme among the responses is the diminishing ability of pregnant women to recall the date of their last menstrual period (LMP) as pregnancy progresses, particularly if their first antenatal care (ANC) visit occurs after the third month. One respondent noted that women who could recall the LMP date in the first trimester usually retain this memory later on (Response 182). However, several respondents highlighted that the reliability of recall decreases over time, emphasizing the importance of early ANC visits for accurate dating (Responses 187, 191).  Documentation and Reference: healthcare providers stress the importance of documenting the LMP date during the initial ANC visit to avoid future recall issues. One respondent indicated that once the LMP date is recorded in the health notebook, it serves as a reference throughout the pregnancy, eliminating the need for the woman to remember the date repeatedly (Response 189). Another respondent mentioned that they document various details, including the LMP date, as soon as the woman recalls it during the ANC visit (Response 188). This practice ensures a consistent reference point for pregnancy monitoring.  Challenges in Recall: respondents pointed out several challenges women face in remembering the exact date of their last period as pregnancy advances. For example, one respondent noted that women often forget the exact date and might rely on the health notebook or healthcare providers for reminders (Response 190). Another respondent highlighted that as pregnancy progresses, women tend to focus more on their current stage of pregnancy rather than remembering the exact LMP date (Response 191).  Estimations Based on Significant Events: in cases where women cannot recall the exact date of their last period, respondents noted that they often relate it to significant events or holidays, such as Tabaski or Ramadan, to provide an approximate month (Responses 197, 200). This method aids healthcare providers in estimating the LMP date, though it may not be precise.  Practical Implications and Strategies: the responses underscore the critical importance of early ANC visits to accurately document the LMP date and ensure reliable pregnancy dating. Healthcare providers are advised to encourage women to attend ANC as soon as they suspect pregnancy to facilitate accurate and consistent documentation. Additionally, recording the LMP date and other relevant details in the health notebook during the first visit helps maintain a reliable reference throughout the pregnancy, reducing the dependence on the woman’s recall as time progresses. Utilizing significant events or holidays to estimate the LMP date can also be helpful when exact recall is challenging. |
| --- |

### 2.8 Materials needed for calculating gestational age

Table 9: Results emulation for scientific article for “Materials needed for calculating gestational age”

| Reliance on Basic Tools for Gestational Age Calculation: a prominent theme in the responses is the reliance on basic tools such as tape measures and gestograms for calculating gestational age. Several respondents highlighted the use of tape measures to determine fundal height, which provides an approximate gestational age. For instance, one respondent stated that they use a tape measure for fundal height measurement and tables to correlate these measurements with gestational age (Response 260). Another noted that they have a gestogram available, which they use alongside a tape measure to estimate gestational age (Response 262). These basic tools are fundamental in settings where more advanced equipment may not be readily available, underscoring their critical role in prenatal care.  Limited Use and Access to Ultrasound: the use of ultrasound is another recurring theme, albeit with limitations in frequency and accessibility. While some respondents mentioned having access to ultrasound machines, they also noted that these are not always used systematically for every patient. For example, one respondent explained that ultrasounds are typically employed in specific situations that require consultation with a gynecologist to determine the gestational age (Response 257). Another highlighted that although they have access to an ultrasound machine, it is primarily used for confirmation purposes rather than routine checks (Response 262). This suggests that while ultrasounds are available, their use is often reserved for particular cases rather than being a standard practice, reflecting both logistical constraints and clinical priorities.  Challenges and Constraints: several respondents pointed out challenges and constraints related to the available equipment and methods. One respondent mentioned the potential inaccuracies of using fundal height due to factors such as insufficient amniotic fluid or fetal developmental issues, which can lead to errors (Response 259). Another noted the difficulty in using a tape measure effectively when the woman cannot provide even an approximate month of her last period (Response 263). These challenges highlight the limitations of relying solely on basic tools without more advanced technology or accurate patient history, emphasizing the need for a comprehensive approach to gestational age estimation.  Practical Implementation and Usage: practical implementation of these tools varies among respondents. Some noted that they carry gestograms in their bags and use them alongside fundal height measurements, especially in urban settings where ultrasound use is more common (Response 268). Another respondent emphasized the importance of documenting the LMP during the first ANC visit to facilitate accurate calculations later on (Response 189). This practice helps ensure that there is a reliable reference point for gestational age estimation throughout the pregnancy, illustrating a practical strategy to enhance the accuracy of prenatal care.  Integration of Multiple Methods: integrating multiple methods to estimate gestational age is a common approach among healthcare providers. One respondent described using a combination of the LMP date, clinical assessments, and fundal height measurements, resorting to ultrasound for confirmation if necessary (Response 267). Another mentioned using the LMP date as the primary reference and supplementing it with fundal height measurements and gestograms to estimate the probable delivery date (Response 270). This integrative approach helps mitigate the limitations of any single method and provides a more comprehensive assessment of gestational age, ensuring more accurate and effective prenatal care. |
| --- |

## 3. Registration of preterm birth

Table 10: Results emulation for scientific article for “Registration of preterm birth”

| **Inclusion in Birth Registers:** a predominant theme among the responses is that premature births are generally recorded in the birth registers of health centers. Many respondents confirm that their birth registers include specific sections for noting whether a birth is premature, along with parameters such as gestational age and birth weight. For instance, one response mentions that the birth register includes whether a birth is premature and notes the gestational age and the number of low-birth-weight children (Response 365). Another response states that there is a specific section in the register for noting the gestational age of each birth (Response 367). Other responses similarly confirm that premature births are recorded in the birth register (Responses 371, 381, 386, 391, 392, 395).  **Inclusion in Monthly Reports:** the inclusion of premature birth data in monthly activity reports is more varied. While some health centers include details of premature births in their monthly reports, others do not. One response notes that premature births are not always detailed in the monthly reports, as the reports usually count the number of deliveries without specifying if they were premature (Response 364). Another response confirms that prematurity details are included in the monthly activity reports (Response 368). However, there are responses indicating that while the birth registers record premature births, the monthly reports primarily mention the 'number of full-term newborns,' thus not explicitly noting prematurity data (Response 394).  **Need for Separate Registers:** a few responses suggest the need for separate or distinct registers specifically for premature births to improve documentation and tracking. One respondent recommends maintaining a distinct registry for premature newborns (Response 365). Another response highlights that there is no specific register for premature births and suggests that such details should ideally be included in the birth register (Response 378). These suggestions underscore the need for more targeted documentation to enhance the tracking and management of premature births.  **Challenges in Data Collection and Classification:** some responses highlight challenges in accurately recording and classifying premature births. One response points out that the birth weight alone is not sufficient to determine prematurity, as there are also cases of low birth weight due to other reasons like hypotrophy (Response 376). Another respondent mentions that it is hard to trace prematurity in the monthly report because it is not specifically noted in the register, making it difficult to distinguish between premature births and hypotrophic cases (Response 377). These challenges indicate the need for more precise data collection methods and clearer classification criteria.  **Consistency and Clarity:** several responses emphasize the importance of consistency and clarity in recording and reporting premature births. One response confirms that premature births are clearly marked in the report, highlighting the requirement to include this information (Response 387). Another response mentions that their registers have a specific section for premature babies, which is also highlighted in their reports (Response 391). These responses underscore the need for clear and consistent documentation practices to ensure accurate and comprehensive reporting. |
| --- |

## 4.Number of preterm births per month

Table 11: Results emulation for scientific article for “Number of preterm birth per month”

| **Difficulty in Providing Exact Numbers:** a prominent theme is the difficulty health centers face in providing exact numbers of premature births due to limitations in their current tracking systems. Many responses indicate that their systems do not specifically track the number of premature births, and obtaining this information would require a detailed review of individual birth records. For instance, one respondent noted that their current system does not track premature births in the final report, and a detailed review of the birth register would be needed to determine the number (Response 401). Similarly, another response highlighted that monthly reports indicate the total number of births but not specifically premature births, necessitating case-by-case analysis (Response 402).  **Estimates and Variability:** despite the challenges in tracking, some health centers provide estimates for the number of premature births. These estimates indicate variability in the number of premature births from month to month. One response estimated that 5 to 10% of monthly deliveries are premature (Response 404). Another noted that the number varies, with some months having as few as two cases and other months having none (Response 416). One respondent recalled that their center had about two or three premature babies last month (Response 409). These estimates suggest that while precise numbers are hard to come by, health centers still have a general sense of the prevalence of premature births.  **Challenges in Data Collection and Classification:** several responses highlight the challenges in accurately collecting and classifying data on premature births. One response pointed out that confusion between low birth weight, hypotrophy, and prematurity often leads to underestimation of the number of premature births (Response 404). Another noted that the monthly report does not clearly specify premature births, making it difficult to distinguish them from full-term but low-weight births (Response 419). These challenges underscore the need for improved data collection practices to accurately classify and report premature births.  **Lack of Routine Data Collection:** a significant number of responses indicate that the data on premature births is not routinely collected. One respondent mentioned that their center does not specifically collect data on premature births as part of traditional data collection, and similar inquiries have highlighted this gap (Response 414). Another response noted that the center does not have a specific line in the report that lists premature births separately (Response 419). This lack of routine data collection highlights the need for systemic changes to ensure that premature birth data is consistently recorded and reported.  **Seasonal Variations and External Factors:** some responses mention seasonal variations and external factors that influence the number of premature births. One response estimated that out of 100 women who give birth, around five to ten are premature, with monthly numbers often around two but varying. The number of premature births tends to increase during the winter due to complications from conditions like malaria and other infections (Response 422). This indicates that external factors such as seasonal changes and health conditions can affect the prevalence of premature births. |
| --- |

## 5.Types of challenges face during preterm birth

Table 12: Results emulation for scientific article for “Types of challenges face during preterm birth”

| **Premature Birth as a Priority Health Challenge:** premature birth is widely recognized as a significant public health challenge by many health centers, primarily due to its frequency and severe consequences. Respondents frequently identify premature births as a top priority, requiring focused attention and resources. One respondent highlighted the daily management of prematurity cases, often necessitating transfers to higher-level facilities for better care (Response 431). Another emphasized the major health challenge posed by premature births, noting the difficulty in providing adequate care (Response 437). The recognition of premature births as a critical issue underscores the need for targeted strategies and enhanced resources to effectively manage this challenge.  **Lack of Resources and Equipment:** a recurring issue in managing premature births is the lack of adequate resources and equipment necessary for effective care. Respondents commonly reported shortages of essential items such as incubators, kangaroo care units, and medications. One respondent pointed out the absence of kangaroo care units and incubators, which are vital for managing hypothermia and other complications in premature babies (Response 432). Another highlighted the insufficiency of facilities to care for premature babies adequately, resulting in frequent transfers to higher-level facilities (Response 434). This shortage of resources and equipment is a significant barrier to providing effective care for premature infants.  **Transfer and Referral Issues:** due to the lack of on-site resources, many health centers rely heavily on transferring premature infants to better-equipped facilities, which often complicates care. One respondent mentioned that their facility transfers almost all premature cases to Souro Sanou hospital (Response 435). Another noted that even when evacuated, hospitals like Souro Sanou, equipped with incubators, often lack space, resulting in infants being returned with only oral prescriptions (Response 437). These responses highlight the systemic challenges in managing premature births locally and the dependency on external facilities for adequate care.  **Parental Perceptions and Refusals:** parental perceptions and refusals to transfer premature babies for further care present additional challenges. One respondent noted that parents often refuse transfers because they doubt the viability of their premature newborns, viewing the effort and time commitment as not worthwhile (Response 438). This refusal complicates the ability of health centers to provide optimal care, as parents frequently reject necessary transfers to better-equipped facilities (Response 437).  **Infections and Associated Risks:** infections are a significant problem contributing to prematurity and complicating care. One respondent mentioned the common problem of infections leading to prematurity and the lack of necessary medications to manage these threats effectively (Response 439). Another response identified infections as a major cause of premature births, emphasizing the need for improved infection control measures (Response 477). The prevalence of infections underscores the need for comprehensive healthcare strategies to mitigate these risks.  **Specific Care Needs of Premature Babies:** premature babies require specialized care to address issues such as hypothermia, respiratory distress, and other complications. One respondent highlighted the fragility of premature babies, noting their susceptibility to respiratory distress and the need for close monitoring and careful treatment (Response 449). Another respondent emphasized the specific challenges during pregnancy that lead to prematurity, such as the breaking of water or severe preeclampsia (Response 450). These specific care needs necessitate specialized training and equipment, which many health centers currently lack.  **Challenges in Managing Pregnancies and Childbirth:** general challenges in managing pregnancies and childbirth also contribute to the occurrence of premature births. One respondent noted that it is crucial to monitor women closely during pregnancy to prevent premature births (Response 475). Another emphasized the importance of projects focused on caring for babies with low birth weight, highlighting the significance of this issue (Response 476). These responses indicate that effective management of pregnancies and childbirth is essential to preventing premature births.  **Importance of Specialized Care Units:** the use of specialized care units, such as Kangaroo Mother Care (KMC) units, is highlighted as an important aspect of managing premature births. One respondent mentioned their reliance on a kangaroo unit to care for non-sick premature babies, focusing on keeping the child warm and educating the mother and family (Response 453). Another response highlighted the performance of their district's kangaroo unit and its protocols for premature deliveries (Response 472). The establishment and utilization of specialized care units are crucial for improving the outcomes for premature infants. |
| --- |

## 6.Staff skills and existence of equipment for better care

Table 13: Results emulation for scientific article for “Staff skills and existence of equipment for better care”

| **Lack of Necessary Resources and Equipment:** a prominent challenge in managing premature births in health centers is the significant lack of essential resources and equipment. Respondents frequently highlighted the absence of basic resuscitation equipment, incubators, and specialized neonatal units. For example, one respondent emphasized the need for isolation from adults, noting the absence of incubators and separate rooms for premature babies (Response 481). Another respondent stated bluntly, "we don't have any of those resources" (Response 483). This inadequacy extends to technical platforms, resulting in frequent referrals of premature babies to higher-level facilities for better care (Responses 488, 500, 508, 509). The consistent theme across these responses is the critical shortage of resources necessary to manage premature births effectively.  **Reliance on Referrals and Transfers:** given the inadequacy of on-site resources and equipment, many health centers heavily rely on transferring premature infants to better-equipped facilities. Respondents noted that extreme cases of prematurity are often evacuated to hospitals such as CMA or Souro Sanou hospital for specialized care (Responses 481, 505, 509). This dependency on external facilities underscores a significant gap in local capacity to manage premature births. One respondent succinctly pointed out that they make do with the resources they have and refer cases they cannot handle (Response 501). This reliance highlights the need for strengthening local capabilities to reduce the dependency on higher-level facilities.  **Trained Personnel and Expertise:** while some health centers have trained personnel, including midwives and doctors, the lack of specific facilities and equipment limits their ability to provide comprehensive care for premature infants. One respondent mentioned having a well-trained staff but noted the absence of necessary facilities for premature infants (Response 485). Another response emphasized that although there is some knowledge of premature newborn care, practical implementation is hindered by the lack of necessary equipment and resources (Response 486). This indicates that while personnel may be adequately trained, the absence of appropriate infrastructure severely limits their effectiveness.  **Use of Kangaroo Mother Care Units**: several responses highlighted the utilization of Kangaroo Mother Care (KMC) units for managing premature babies. These units, which focus on skin-to-skin contact to maintain the babies' warmth and stability, are primarily limited to healthier premature infants without severe complications. For instance, one response noted that the KMC unit could manage babies near their due date and with low birth weights but not those that are very premature (Response 498). Another respondent mentioned that the KMC unit is limited to healthy premature babies and that any baby with additional pathologies must be sent to the hospital (Response 492). While KMC units provide crucial support, their limited capacity underscores the need for more comprehensive care options for all premature infants.  **Challenges in Medication Availability**: in addition to equipment shortages, the availability of necessary medications poses a significant challenge in managing premature births. One respondent highlighted shortages of essential medications such as vitamin K1 and aqueous eosin for cord care (Response 481). Another response pointed out the general lack of medications needed to effectively manage the threat of premature births (Response 439). These medication shortages further complicate the efforts to provide adequate care for premature infants.  **Parental Involvement and Education:** A few responses emphasized the importance of involving and educating mothers in the care of premature infants. One respondent noted that mothers are included in the care process, being shown how to handle and care for their premature babies (Response 516). This involvement is crucial for the success of interventions like Kangaroo Mother Care, ensuring that parents are well-prepared to support their infants' needs. Engaging and educating parents can significantly enhance the overall care and outcomes for premature infants. |
| --- |

## 7.Services and care provided to premature babies and their mothers

Tableau 14: Results emulation for scientific article for “Services and care provided to preterm babies and their mothers”

| **Basic Care and Transfer for Specialized Treatment:** many health centers provide basic care for premature infants immediately after birth, but due to limited resources, they often transfer these cases to higher-level facilities for specialized care. One respondent indicated that while mothers receive standard postpartum care, extra precautions are taken to protect premature babies from hypothermia, often necessitating transfers to better-equipped facilities (Response 517). Another center's initial response includes basic resuscitation efforts such as clearing airways and monitoring oxygen levels with a saturometer, though the capabilities are restricted (Response 518).  **Monitoring and Education:** some centers keep premature infants for short-term monitoring if immediate transfer is not possible. One respondent mentioned keeping the baby for at least 72 hours to combat hypothermia and ensure food hygiene and cleanliness while educating mothers about the fragility of their children (Response 519). Another highlighted that while the same care is given to mothers as for normal deliveries, premature infants receive specific newborn care including heating and measures to prevent cold exposure. In cases of respiratory distress, intubation and oxygenation are necessary (Response 520).  **Limited Equipment and Resources:** the recurring challenge of insufficient equipment and resources is evident. Some centers have basic equipment such as heaters and resuscitation tools but find them inadequate for handling multiple premature cases simultaneously (Responses 521, 523). One center emphasized that while mothers receive standard care, the primary focus for premature infants is on thermal protection and nutrition, using feeding tubes when necessary (Response 524).  **Emphasis on Hygiene and Nutrition:** many centers stress the importance of hygiene and nutrition for both mothers and infants. One respondent highlighted the advice given to mothers on keeping the child warm and maintaining personal and clothing hygiene, which is crucial for ensuring sufficient breast milk production (Response 526). Another noted that dietary advice for the child and guidance on hygiene are critical aspects of the care provided, supported by experienced staff including a pediatrician and midwives (Response 527).  **Kangaroo Mother Care (KMC) Units:** several centers utilize Kangaroo Mother Care (KMC) units for managing premature babies, focusing on skin-to-skin contact to maintain warmth and stability. One respondent mentioned the use of the KMC unit for initial care before transferring the baby to a specialized facility (Response 528). Another emphasized the protocols in place for premature deliveries in their district's KMC unit, highlighting its effectiveness in providing specialized care (Response 533).  **Challenges and Immediate Actions:** many centers face significant challenges due to the lack of specialized care capabilities. One respondent noted that after delivery, the priority is to protect the child by weighing and warming them before quickly evacuating them due to the lack of suitable medications, training, and technical equipment (Response 530). Another respondent indicated that they generally refer premature babies to higher levels of care after providing immediate essential care like thermal protection and glucose administration to stimulate sucking (Response 533).  **Specialized Care Needs and Transfer Criteria:** some centers differentiate between premature infants based on their weight and reflexes, keeping those who are slightly premature but transferring those with more severe conditions. One respondent mentioned keeping babies weighing more than 2 kg and showing good reflexes but evacuating those below this weight with respiratory distress (Response 541). Another emphasized the importance of not bathing the baby immediately and ensuring thermal protection during transport for evacuation (Response 534). |
| --- |

## 8.Health risks in premature infants

Tableau 15: Results emulation for scientific article for “health risks in premature infants”

| **Infections as a Major Risk**: a dominant theme among the responses is the high susceptibility of premature infants to infections. Multiple respondents highlighted the vulnerability of these infants to various infections due to their underdeveloped immune systems. One respondent pointed out that infections are a significant risk for premature babies, many of whom do not survive without proper care (Response 543). Another noted that premature infants are immunocompromised and often miss out on the antibodies provided by breast milk, making them more susceptible to opportunistic diseases (Response 544). Respiratory infections, in particular, were mentioned as common due to underdeveloped bronchi (Response 547). This recurrent mention of infections underscores the critical need for effective infection control and prevention measures.  **Immunocompromised State and Nutritional Challenges**: premature infants are often considered immunocompromised, which exacerbates their vulnerability to infections. Respondents noted that these infants' bodies are not as developed as those of full-term children, and many cannot effectively breastfeed, missing out on essential antibodies (Responses 544, 549). Nutritional challenges are significant, as premature infants may lack the sucking reflex necessary for feeding, requiring special attention and sometimes force-feeding through tubes (Response 574). These nutritional deficiencies further weaken their immune systems, increasing the risk of infections and other health complications.  **Stigmatization and Perceptions of Viability**: stigmatization and negative perceptions regarding the viability of premature infants also emerged as a notable theme. One respondent discussed how premature children are often given specific names in different communities, signifying their premature birth and contributing to stigmatization (Response 545). This social stigma, coupled with a general belief in some communities that premature infants are not viable, can negatively impact their care and the willingness of parents to seek necessary medical interventions (Response 550).  **Respiratory Issues and Hypothermia**: respiratory issues are a common problem for premature infants, as their lungs are often underdeveloped. One respondent mentioned the frequent occurrence of respiratory infections, which are a significant health risk (Response 547). Additionally, hypothermia is a critical concern due to the infants' inability to regulate their body temperature effectively. Respondents emphasized the importance of thermal protection, noting that inadequate clothing and environmental conditions can exacerbate this risk (Responses 562, 565, 577). Maintaining proper warmth is essential to prevent hypothermia, which can be life-threatening for these vulnerable infants.  **Economic and Social Factors**: economic and social factors significantly impact the health outcomes of premature infants. One respondent noted that caring for a premature infant presents an economic burden, as parents often have to leave work to be with their newborns (Response 554). Another highlighted the crowded living conditions and poor food hygiene that many mothers face, which contribute to the spread of infections and nutritional deficiencies (Response 558). These factors complicate the care of premature infants, making it difficult to provide the necessary protection against infections and ensure proper nutrition.  **Transportation and Accessibility of Care**: the lack of adequate transportation and accessibility to specialized care facilities is another critical issue. Respondents mentioned that premature infants often need to be referred to specialized facilities, but the absence of ambulances means parents must use personal means to transport their infants, which poses significant risks (Response 582). This lack of proper transportation further complicates the ability to provide timely and appropriate care for premature infants.  **Other Health Risks**: additional health risks for premature infants include hypoglycemia, anemia, and malaria. One respondent noted that hypoglycemia can be life-threatening for premature newborns, emphasizing the need for immediate and proper feeding to prevent this condition (Response 572). Another mentioned anemia as a significant risk for both premature children and their mothers (Response 584). Malaria was also highlighted as a contributing factor to prematurity and a complicating health issue for newborns (Response 587). |
| --- |

## 9.Specificities of mothers of preterm children

Tableau 16: Results emulation for scientific article for “specificities of mothers of preterm children”

| **Socio-Economic and Environmental Factors Influencing Premature Births in Urban Burkina Faso:** a prominent theme among the responses is the significant influence of socio-economic and environmental factors on the incidence of premature births. Several respondents highlighted that poverty is a common characteristic among mothers of premature infants. One respondent noted that prematurity is more prevalent in households with lower incomes or financial instability (Response 593). Another pointed out that poor living conditions, such as inadequate hygiene and crowded environments, are frequently associated with mothers of premature babies (Response 594). These socio-economic challenges contribute to a higher risk of prematurity due to factors like malnutrition and poor access to healthcare.  **Health Conditions and Infections as Contributors to Premature Births:** many respondents emphasized the role of maternal health conditions and infections in causing premature births. Commonly mentioned conditions include malaria, anemia, genital infections, and preeclampsia. One respondent observed that premature births often involve mothers who had malaria, were anemic, or had genital infections that led to rising infections and ultimately prematurity (Response 596). Another noted that maternal infections, such as urinary infections or bacterial vaginosis, are significant risk factors for prematurity (Response 601). These health issues highlight the need for effective prenatal care and infection control measures.  **Lack of Awareness and Traditional Practices Impacting Premature Infant Care:** a recurring theme is the lack of awareness and adherence to medical advice among mothers of premature infants. Respondents noted that traditional practices often conflict with medical recommendations, leading to adverse outcomes. One respondent mentioned that parents might bathe premature babies extensively or force-feed them, despite advice to the contrary, which can lead to infections (Response 597). Another emphasized the neglect of treatments and preventive measures against malaria, as well as poor compliance with dietary advice (Response 618). These behaviors underscore the importance of education and awareness programs to improve health outcomes for premature infants.  **Delayed Medical Consultations and Social Challenges:** delayed medical consultations and social challenges also emerged as significant factors contributing to premature births. Respondents noted that many women seek medical care late, often when premature labor is already underway, making it difficult to intervene effectively (Response 610). Social challenges, such as relationship problems, being students, or experiencing other forms of social instability, were also linked to higher rates of prematurity (Response 612). These findings suggest the need for improved access to and utilization of prenatal care services.  **Malnutrition and Physical Strain as Contributing Factors:** malnutrition and physical strain were frequently mentioned as contributing factors to prematurity. One respondent highlighted that malnutrition is common among mothers of premature infants, both in rural and urban settings, where heavy physical work can also lead to premature labor (Response 619). Another noted that socio-economic status plays a role, as mothers who cannot afford adequate nutrition are more likely to give birth prematurely (Response 626). Addressing these nutritional deficiencies is crucial for reducing the incidence of premature births.  **Preeclampsia and Multiple Pregnancies:** several respondents identified preeclampsia and multiple pregnancies as significant medical conditions associated with prematurity. Preeclampsia, characterized by high blood pressure during pregnancy, is a common cause of premature births and affects women across all social classes (Response 605). Multiple pregnancies, such as twins or triplets, also increase the risk of prematurity due to increased abdominal tension and the potential for premature rupture of membranes (Response 605). These conditions highlight the need for specialized medical care and monitoring during pregnancy. |
| --- |

## 10.Improvement in care of preterm birth

Tableau 17: Results emulation for scientific article for “improvement in care of preterm birth”

| **Raising Community Awareness and Preventive Measures**: raising awareness within the community is deemed essential for reducing the incidence of premature births. Respondents emphasized the importance of early antenatal care (ANC), including taking iron supplements and maintaining a nutritious diet, as well as ensuring proper follow-up care (Response 627). Preventive measures also include closely monitoring pregnancies to manage and mitigate risks such as infections and hypothermia (Response 628).  **Enhancing Technical Capabilities and Resources:** a significant challenge highlighted by respondents is the lack of essential medical equipment and resources necessary for the effective management of premature births. The absence of incubators, dedicated rooms for premature infants, and basic resuscitation equipment were noted as critical gaps (Responses 628, 632, 639, 640). Upgrading the technical platform, including the availability of heating lamps, oxygenation facilities, and nasogastric tubes, was suggested to improve care capabilities (Responses 632, 640).  **Continuous Training and Knowledge Dissemination:** continuous training of health staff is crucial for maintaining high-quality care for premature infants. Respondents suggested regular training sessions to keep staff updated on the latest care protocols (Response 629). Additionally, it was recommended that trained personnel share their knowledge with colleagues to ensure continuity of care even if some staff members leave (Responses 630, 638). Establishing a mentorship program with external experts could also provide ongoing training and support (Response 638).  **Addressing Staff Movement and Retention:** staff movement and retention pose challenges to maintaining consistent care for premature infants. One approach to mitigate this issue is to have trained staff sign a commitment to stay for a certain number of years post-training (Response 631). Another solution is to focus on training local staff who are more likely to remain in the center (Response 631). Ensuring that trained staff pass on their knowledge to new team members can also help maintain service quality (Response 642).  **Community Perception and Stigmatization:** community perceptions and stigmatization of premature infants can impact their care. Respondents noted that premature infants are often stigmatized, which can lead to psychological issues and reluctance from parents to seek medical care (Responses 634, 635). Addressing these socio-cultural beliefs through education and awareness programs can help improve community support and reduce stigmatization (Response 668).  **Dedicated Neonatology Units and Specialized Care:** the establishment of dedicated neonatology units with specialized staff was frequently mentioned as a necessary step to improve care for premature infants. Respondents emphasized the need for units equipped with incubators and managed by pediatricians to handle the specific needs of premature babies (Responses 639, 644). The creation of such units at the CMA level would help alleviate the burden on higher-level facilities and improve overall care quality (Response 644).  **Improving Prenatal and Postnatal Care Practices:** enhancing prenatal care practices by encouraging early and regular consultations was highlighted as critical for preventing premature births. Proper nutritional advice, early detection, and management of conditions like hypertension and infections were identified as key factors (Responses 654, 656, 678). For postnatal care, having the necessary equipment and trained personnel to provide immediate and effective care is essential (Response 660).  **Infection Control and Hygiene Practices:** infection control remains a significant concern, with respondents noting the need for improved hygiene practices among patients and staff (Response 680). Providing thorough training on hygiene standards and ensuring the availability of protective gear and clean equipment can help mitigate the risk of infections.  **Addressing Socio-Cultural and Economic Factors:** socio-cultural and economic factors, such as domestic disputes, physical violence, and economic hardship, can stress mothers and lead to premature births. Engaging with families to resolve conflicts and provide support during pregnancy is crucial (Responses 666, 667). Additionally, addressing malnutrition and ensuring proper prenatal care can reduce the incidence of premature births related to socio-economic challenges (Responses 619, 626).  **Regular Refresher Training and Continuous Improvement:** regular refresher training for all staff members, coupled with periodic supervision and case reviews, can help maintain and enhance the skills required for managing premature births (Response 679). Implementing structured training programs at the national level and ensuring that all health workers are equipped with the necessary knowledge and skills can lead to better outcomes (Response 681). |
| --- |
